# The bony labyrinth of toothed whales reflects both phylogeny and habitat preferences

**DOI:** 10.1038/s41598-018-26094-0

**Published:** 2018-05-18

**Authors:** Loïc Costeur, Camille Grohé, Gabriel Aguirre-Fernández, Eric Ekdale, Georg Schulz, Bert Müller, Bastien Mennecart

**Affiliations:** 1 Naturhistorisches Museum Basel, Augustinergasse 2, 4001 Basel, Switzerland; 2 Division of Paleontology, American Museum of Natural History, Central Park West at 79th Street, New York, NY 10024 USA; 3 Paleontological Institute and Museum, University of Zurich, Karl-Schmid-Strasse 4, Zürich, 8006 Switzerland; 4 Department of Biology, San Diego State University, San Diego, California USA; 5 Department of Paleontology, San Diego Natural History Museum, San Diego, California USA; 6 Biomaterials Science Center, University of Basel, c/o University Hospital Basel, Basel, Switzerland; 7 Naturhistorisches Museum Wien, Burgring 7, 1010 Vienna, Austria

## Abstract

The inner ear of toothed whales (odontocetes) is known to have evolved particular shapes related to their abilities to echolocate and move under water. While the origin of these capacities is now more and more examined, thanks to new imaging techniques, little is still known about how informative inner ear shape could be to tackle phylogenetic issues or questions pertaining to the habitat preferences of extinct species. Here we show that the shape of the bony labyrinth of toothed whales provides key information both about phylogeny and habitat preferences (freshwater versus coastal and fully marine habitats). Our investigation of more than 20 species of extinct and modern odontocetes shows that the semi-circular canals are not very informative, in contrast to baleen whales, while the cochlea alone bears a strong signal. Inner ear shape thus provides a novel source of information to distinguish between morphologically convergent lineages (e.g. river dolphins).

## Introduction

Toothed whales (odontocetes) are a very diverse group of aquatic mammals containing more than 70 living species of small to very large animals^1^. They inhabit a wide range of aquatic habitats from open marine environments to the shallow freshwaters of the Asian and South American river systems. Navigation and orientation in dolphins are dependent on echolocation, the production and reception of high frequency sounds of up to 200 kHz^2,3^ (the highest ranges known for mammals). This differentiates them from their mysticete relatives, the baleen whales, which are low frequency specialists^2^. All odontocetes are, to various degrees, high frequency specialists, whether they live in open marine waters or shallow freshwaters. Sound is processed by the cochlea, at the interface between the ear and the brain. The cochlea of odontocetes has been shown to be morphologically different from that of mysticetes, being generally shorter and showing a much stronger bony lamina supporting a stiffer basilar membrane in the basal cochlear turn, a crucial characteristic for high frequency hearing^2^. This clear morphology has fostered research in fossil cetaceans to understand the origin of echolocation in this clade^4–7^. On the other side of the inner ear, posteriorly, the vestibular system is the organ of balance responsible for acceleration and rotational movements. The adaptation to underwater locomotion in the cetacean lineage is linked to dramatical morphological changes of the vestibular system, since the semi-circular canals in whales are reduced in comparison to terrestrial mammals because of the need of a lower sensitivity to cope with fast moving rotation behaviours under water^8^. Building on that hypothesis, pioneering works identified a positive correlation between the radii of the semi-circular canals (i.e., a measure of their size) and locomotor agility in mammals^9^. This result at the level of Mammalia fostered the use of the semi-circular canals to investigate environmental preferences of extinct taxa (e.g.^10^). Recent investigations identified that deviation from orthogonality in the angles between the semi-circular canals could be correlated with rotational head speed^11^. The first investigation of this parameter in the cetacean clade indicated that a low deviation from orthogonality in the semi-circular canal angles was more likely to be found in open marine odontocetes than in nearshore species^12^, which would constitute a proxy for habitat preference reconstructions in extinct taxa. Since another study, based on a limited sample of freshwater odontocetes, did not yield the same result^13^, this observation required more investigation, especially because identifying extinct freshwater odontocetes in the fossil record is critical to understand the origin of this adaptation in the clade. All the above-mentioned studies show that the bony labyrinth has mostly been used to infer ecological preferences and their origin^4–8,12^. However recent works have indicated how phylogenetically informative the inner ear in mammals could be^14^. The shape of the inner ear of baleen whales in both its vestibular and cochlear parts is partly explained by their phylogenetic relationships^15^. Early development of the inner ear largely before birth may partly explain its strong link to phylogeny^16–18^. In addition, intraspecific variability of this structure is lower than the inter-genera disparity giving solid grounds to its use in systematics and phylogeny^19–21^. While molecular data help us understand the relationships of living taxa, the search for pertinent morphological characters is critical to our understanding of the origin and divergence of clades. As an example, the polyphyly of “river dolphins” has been understood thanks to molecular data when morphological data consistently indicated monophyly because of strong morphological convergences related to their skeletal adaptations to freshwater shallow environments (see^22^ for a review). The prospects of finding new morphological characters in the inner ear that may have the potential to differentiate extinct taxa is thus very promising and constitute one of the aims of this contribution. We show that the bony labyrinth contains both a strong phylogenetic and ecological signal in odontocetes. Freshwater, coastal, and fully marine species are statistically different based on the shape of the bony labyrinth as a whole, and of the cochlea when analysed separately. Likewise, the main clades examined are distinguished, including the polyphyletic “river dolphins”. The semi-circular canals investigated separately do not have the same discriminative power as the cochlea, partly because of their much reduced morphology in odontocetes.

## Results

**Figure 1:**
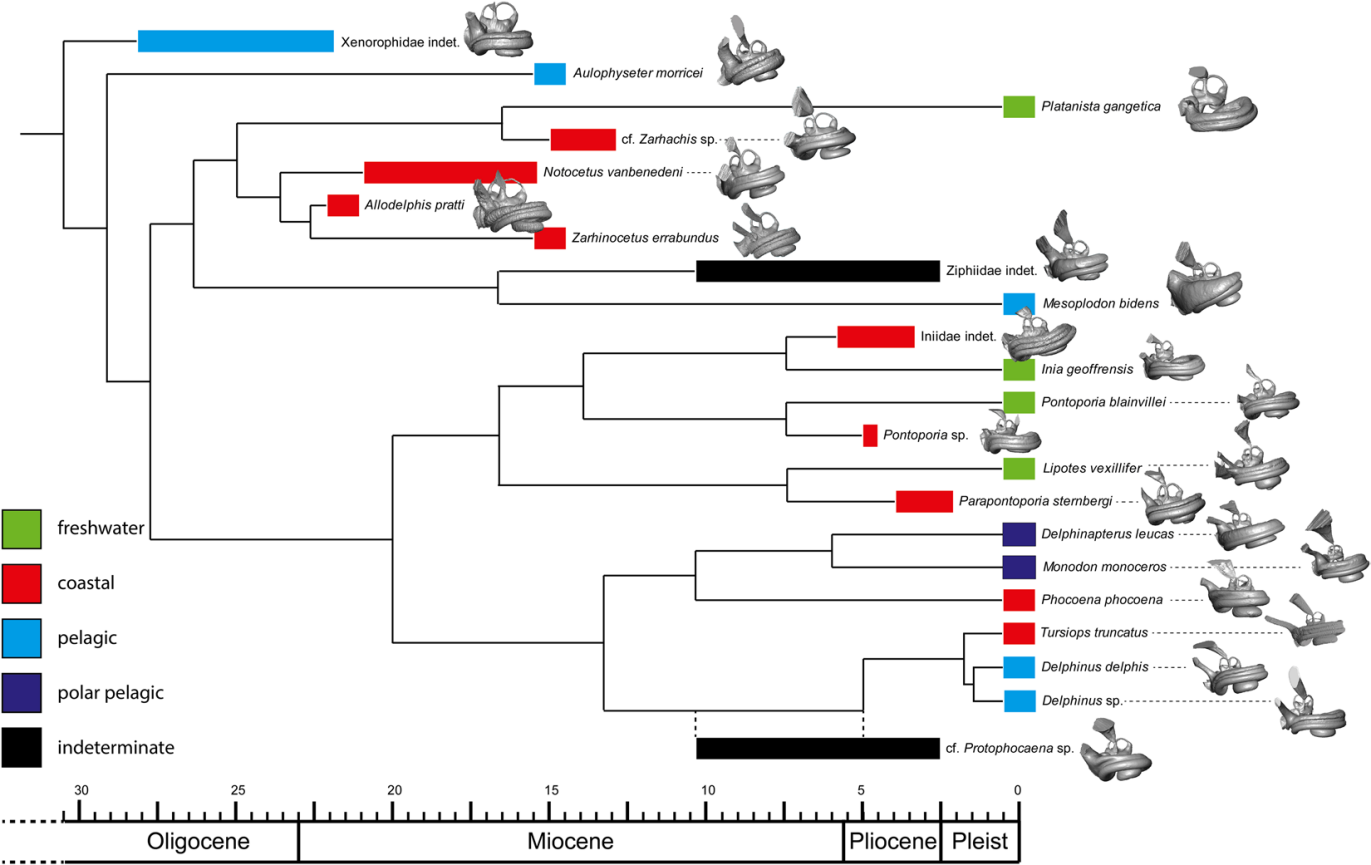
Composite phylogeny of the species examined in this study. See Supplementary data 8 for phylogenetic hypotheses, age of the nodes and ecological inferences. Bony labyrinths are shown in anteromedial view and are not to scale.

**Figure 2:**
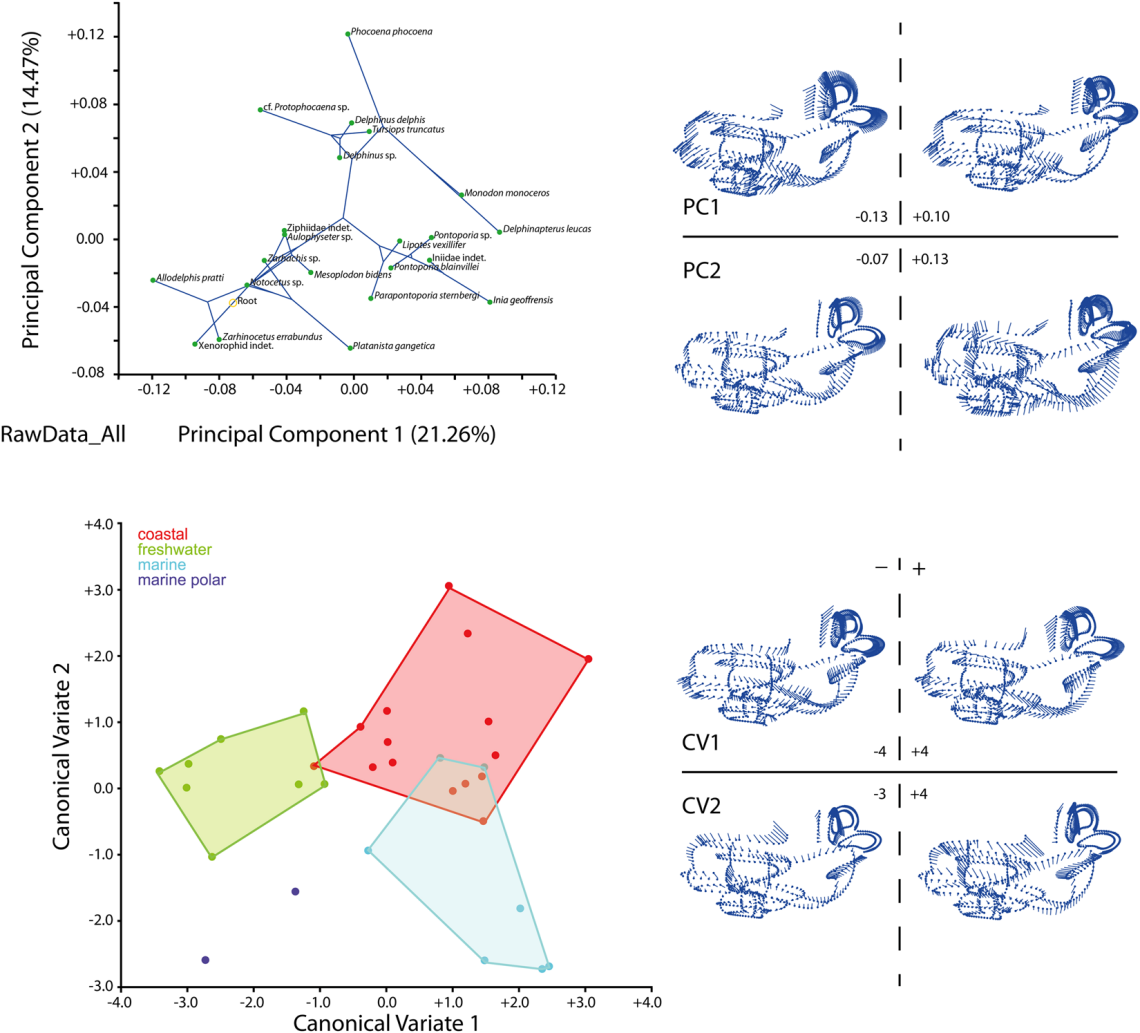
Top left, Principal Component Analysis of the Raw Data dataset (see text) with superimposed phylogeny. Top right, shape variation of the bony labyrinth on the PCA morphospace. Bottom left, Canonical Variate Analysis of the Raw Data dataset, colors are environmental categories as indicated on the legend. Bottom right, shape variation of the bony labyrinth on the CVA morphospace. Electronic supplementary material 6 completes the results with the sliding protocols and for the subsets analysed (semi-circular canals and cochlea) and Supplementary data 6 gives the results of the geometric morphometric analyses.

We sampled bony labyrinths across the odontocete phylogeny (Fig. 1). Our multivariate shape analysis of the odontocete bony labyrinths indicates a large degree of morphological variation (Fig. 2). While cochleae show a limited and relatively constant number of turns (between 1.75 and 2.25; see Supplementary data 1), their shapes are variable; the cochlear aqueduct is highly different between clades (i.e., straight, relatively thin and elongated in delphinids, thick, shorter and dorsally oriented in *Aulophyseter*, *Allodelphys*, or in the ziphiids, Fig. 1 and Supplementary data 2). Conversely, our statistical tests revealed that the shape of the semi-circular canals cannot be used to separate clades. The results of the permutation test (resampling method) reject the null hypothesis of no phylogenetic signal when considering the entire bony labyrinth (p-values < 0.0001) as well as the cochlea itself (p-values = 0.0001). The null hypothesis is not rejected by the semi-circular canals dataset in all the analyses (p-values > 0.1). The phylogenetic signal observed in the bony labyrinth of our dataset is thus concentrated in the cochlea and not in the semi-circular canals (Supplementary data 3). As a consequence, discrete characters of phylogenetic interest can be proposed. Ziphiids have a wedge-like dorsal part of the basal turn (scala tympani) with a large tympanal recess (*sensu*^16^) overlapping the secondary bony lamina. The condition is extreme in *Mesoplodon bidens*, less so in the indeterminate fossil ziphiid under consideration here, but still much more than in any other odontocete of our dataset. Delphinids have a long, funnel-shaped endolymphatic sac (*Tursiops* and *Delphinus*, Supplementary data 2), and an elongated and straight cochlear aqueduct; the latter is medioposteriorly bent in phocoenids and their sister taxa the monodontids, the two of them sharing a similar conical to triangular endolymphatic sac. The cochlear aqueduct is comparatively thicker and more dorsally oriented in early diverging lineages (e.g., the xenorophiid specimen, *Aulophyseter*, or *Allodelphis*, see Supplementary data 2).

The allometric signal was tested through permutation tests. They reject the null hypothesis of independence between shape and centroid size (not body size) for the entire bony labyrinth (p-values < 0.05) and the cochlea only (p-values < 0.001). However, the results for the semi-circular canals are insignificant (p-values > 0.1), indicating that the allometric signal observed is again located in the cochlea and not in the semi-circular canals. This is likely due to the fact that the inner ear is most comprised of the cochlea, whereas the semi-circular canals are greatly reduced. In our sample, larger dolphins with larger cochleae and bony labyrinths are mostly fully marine while smaller dolphins live in river systems, so a habitat-size relationship would be tempting to draw, but small dolphins are also known in fully marine habitats, like *Phocoena*^1^.

The statistical results of the CVA to separate habitat preferences based on bony labyrinth morphology are not significant for marine polar species (see Supplementary data 3) since the number of individuals (N = 2) is too low. Nevertheless, the other environmental categories separate well and are statistically supported (Fig. 2 and Supplementary data 4).

The results of the 81 statistical analyses are very similar using RawData, data slid by Bending Energy, or data slid by Procrutes Distances (see Supplementary data 3) with the exception of the marine vs. coastal comparison based on Mahalanobis Distances among groups, and of the freshwater vs. coastal comparison based on Procrustes Distances among groups, both being based on the semi-circular canals dataset slid using Procrustes Distances. Thus, an approach with multiple landmarks makes it possible to independently use any of the datasets. The statistical results show that the tested signal is different when the bony labyrinth is considered as a whole or when the cochlea and the semi-circular canals are taken separately.

The distinction of the fully marine and coastal habitats is not recovered using the entire bony labyrinth, while all the habitats can be separated based on the shape of the cochlea only (all p-values are significant to highly significant; see Supplementary data 3). In contrast, almost half of the tests on the semi-circular canals are not significant. In particular, the distinction of coastal from fully marine habitats is not supported based on the semi-circular canals (see Supplementary data 3). The environmental signal on the bony labyrinth is here mainly supported by the shape of the cochlea. It is noteworthy that along CV1, the entire bony labyrinth and cochlea of coastal dwelling species overlap the morphospace of freshwater and mostly marine species (Fig. 2 and Supplementary data 4). In addition, freshwater and fully marine species show no overlap. CV2 allows a better discrimination of coastal species from the other habitats.

Marine species are distinguished from freshwater ones by a more massive cochlea (i.e., first turn laterally wider and higher dorso-ventrally). The total cochlear number of turns is generally a little longer in marine specimens than in freshwater ones, even if the variation is extremely small (1/2 turn observed in our odontocete dataset, Supplementary data 1). The fenestra vestibuli is situated a little more ventrally and is broader and more circular in marine species than in freshwater ones.

## Discussion

Attempts at reconstructing biological or ecological traits of extinct cetaceans based on sensory organs have been focussed mostly on the cochlea in order to infer either the origin of echolocation^5^, the ancestral hearing capabilities (high vs. low frequency hearing, e.g.^16,23,24^), or to investigate more precisely the ultrasonic vocalisation and hearing in odontocetes^2,25,26^. Other studies on the vestibular system of odontocetes, and in particular on the deviation to orthogonality of the semi-circular canals (i.e.^12^) indicated that the latter could also be used to infer habitat preferences. Our results based on a larger dataset of extant and extinct odontocetes are in contradiction to^12^ as the shape of the semi-circular canals is mostly not significantly different between marine, coastal, and freshwater species in our sample, confirming a preliminary investigation^13^. Difficulties in measuring canal angles on 3D models may explain results obtained before 3D geometric morphometrics started being systematically applied. As exemplified here, the semi-circular canals of toothed whales are much reduced (Supplementary data 2) probably to limit sensitivity to fast rotational movements^2^ and they seem to lose their power to discriminate habitat preferences.

In contrast, the shape of the cochlea is a good proxy to distinguish odontocete species living in different habitats. Our statistical tests show a significant shape distinction between the cochlea of freshwater taxa like the “river dolphins” and that of coastal taxa or fully marine ones like delphinids. While all investigated taxa use echolocation and are specialised on high-frequency and ultrasonic sounds, they face different challenges. River dwelling species indeed have to locate obstacles and their prey in shallow environments and communicate over short distances while marine dwelling species live in a fully open space without obstacles and where communication may occur over several kilometres^3^. In contrast to more solitary habits in “river dolphins”, fully open marine taxa are often highly social and gregarious species with complex communication achieved through a wide range of sound production, both in high and lower frequencies^3^. This may explain why the cochlea shows a different shape in different habitats^2,3,26^. Empirical data show that the Amazon river dolphin *Inia geoffrensis* commonly uses ultra-high frequencies of 200 kHz to navigate and scan its immediate environment^2^ while fully marine taxa rarely reach such high frequencies (but exceptions exist: i.e., the white beaked dolphin *Lagenorhynchus albirostris*^3^). This ability is related to morphological characters of the cochlea. The large and long secondary bony lamina evidenced here on all “river dolphins”, running over more than half of the cochlea, is a functional adaptation to ultra-high frequency hearing (*sensu*^2^), it supports the stiff and thick (especially in the basal turn) basilar membrane (not preserved inside bony labyrinths) adapted to cope with high resonant frequencies such as demonstrated by^2^. In addition, all “river dolphins” except *Lipotes* have a very loosely coiled cochlea in comparison to other dolphins of our sample, which is interpreted again as an adaptation to very high frequency hearing^2,11^. Specificities of riverine habitats with shallow water and obstacles thus may have participated in driving the evolution of ultra-high frequency adapted cochleae to scan the immediate environment. It is unclear why *Lipotes* has a more tightly coiled cochlea. Either this taxon was less adapted to ultra-high frequencies as the other dolphins living in river systems or the length of its cochlea, among the longest of our sample, makes it more tightly coiled to fit within the periotic. In any case, its secondary bony lamina is long and thick, as in the other “river dolphins”, attesting to the stiffness of its basilar membrane and thus to its good abilities to hear high frequency sounds. More data on cochleae of other dolphins, like *Lagenorhynchus* which is known for ultra-high frequency production, may help clarify this relationship.

Our results also show that the shape of the bony labyrinth of odontocetes bears a highly significant phylogenetic signal, especially when the cochlea alone is analysed (Supplementary data 3). This result is similar to recent observations made on the bony labyrinth of mysticetes^15,16^ and altogether indicate the strong phylogenetic relevance of this sensory structure as a whole in Cetacea, as already shown in other mammal clades (e.g.^14,27,28^). Conversely, the semi-circular canals do not provide much phylogenetic information in odontocetes, in contrast to mysticetes^15^. Despite the lack of precise quantification, the semi-circular canals are apparently less reduced in size in most mysticetes compared to odontocetes, which could result in more shape variability and thus more phylogenetic information. While the polyphyly of the “river dolphins” *Platanista*, *Inia*, *Pontoporia*, and *Lipotes* has become a consensus^22,29^, finding non-convergent morphological characters allowing the distinction of the clades Inioidea, Lipotidae and Platanistidae remains challenging, especially in fossil specimens. It is however crucial to the understanding of their origin and divergence in the fossil record, where molecular data is not available. The same is true for the other odontocete clades for which our results add potentially relevant morphological characters.

*Platanista* occupies a morphospace far from that occupied by the inioids and their sister taxon *Lipotes* on our PCA (Fig. 1). Discrete characters of the bony labyrinth that allow separation of the two clades are as follows: a more elongated endolymphatic sac in the inioids (see Supplementary data 2), a much more dorsally tilted cochlea visible on the anterior view in Fig. 1 (and see Supplementary data 2), and a more posteriorly projecting cochlear aqueduct in *Platanista*. The platanistid *Zarhachis* sp. and *Platanista* share a similar bowl-shaped endolymphatic sac, the divergence pattern of the vestibular aqueduct from the common crus as well as an anteromedially elongated sacculus (Supplementary data 2). As seen above, *Lipotes*, closely related to the inioids, shows a longer and more tightly coiled cochlea. Our limited sample of *Pontoporia blainvillei* (N = 4) gives us some control on the variability of the structures discussed here and indicates that the bony labyrinth in odontocetes does not show much intraspecific variability as already indicated before (i.e.^12^).

Developing echolocation abilities has fostered the diversification of odontocetes over the last 28 Ma^6,30^ and helped them colonize complex turbid freshwater environments. The adaptation to riverine habitats has led to strong morphological convergence in different odotoncete clades. Our study shows that the shape of the cochlea in odontocetes reflects not only their phylogenetic relationships but also their habitat preferences, emphasizing the essential use of the cochlea as a proxy when investigating the palaeoecology of fossil toothed whales. The complex interplay between shape, function and phylogeny of the cochlea would benefit from more exhaustive studies across the odontocete clade, as well as from more empirical data on living species to improve our functional interpretations.

## Methods

Digital endocast of 32 bony labyrinths were reconstructed for 21 species of extant (N = 11, about 15% of odontocete present diversity) and extinct (N = 11) odontocetes (Fig. 1 and see Supplementary data 5 for information on the phylogenetic hypothesis used and habitat preferences of each investigated species as well as information on specimens and scanning parameters). The dataset includes Oligo-Miocene (N = 1), Miocene (N = 5), Mio-Pliocene (N = 2), Pliocene (N = 3), and extant (N = 10) odontocete species from the Atlantic, Pacific, and Arctic Oceans, as well as freshwater river systems of South America, India, and China mainland. The ecology and palaeoecology of the species examined encompass freshwater (N = 4), coastal (N = 9), fully marine (N = 4), and marine polar habitats (N = 2). All specimens were micro-computed tomography scanned (see Supplementary data 5) and segmentation was done using Avizo Standard Edition 7.0®, 8.0® (Visualization Sciences Group, an FEI Company, 2013), and AVIZO Lite® (2016). Measurements of the bony labyrinths are given in Supplementary data 1.

Digitalization of the specimens for 3D geometric morphometrics was performed using Landmark Editor 3.6 (Wiley 2006). 78 curves of 10 equally-distant semilandmarks and 1 landmark were digitised on the surface of the specimens following^13^. We test the hypothesis that the original data (hereafter RawData) present homologous curves and thus homologous points (without sliding the semi-landmarks) by comparing with two additional datasets generated by sliding the semi-landmarks during the superimposition process using the method of minimum Bending Energy (BE) and Procrustes Distances (PD) following^31^. Shape analyses were performed on the whole bony labyrinth as well as on subsets of it: the cochlea alone and the semi-circular canals alone. Principal Component Analysis (PCA) and Canonical Variate Analysis (CVA) were applied to the nine datasets (raw data, BE, PD on whole bony labyrinth, cochlea, and semi-circular canals) to investigate shape variation, to test the phylogenetic signal, and to characterize shape similarities related to the above-mentioned environmental categories (see Supplementary data 6 for methods). Permutations tests (resampling method) applied to the bony labyrinth dataset and subsets of it (cochlea and semi-circular canals) allow us to test for a phylogenetic and for an allometric signal.

Statistical packages and software that were used here are described in Supplementary data 7. Bony labyrinth nomenclature follows^27^ and is given in Supplemetary data 8.

The results of the geometric morphometric analyses are given in Supplementary data 9.

## Electronic supplementary material

Supplementary Dataset 1

Supplementary Dataset 3

Supplementary Data
