# Supplementary material for: The bony labyrinth of toothed whales reflects both phylogeny and habitat preferences
Source: Sci Rep. 2018 May 18;8:7841. doi: 10.1038/s41598-018-26094-0 (PMC5959912; doi:10.1038/s41598-018-26094-0)
Supplement: Supplementary file 3 — Supplementary Data [file 41598_2018_26094_MOESM3_ESM.pdf]

The bony labyrinth of toothed whales reflects both phylogeny and habitat preferences.

Loïc Costeur, Camille Grohé, Gabriel Aguirre-Fernandez, Eric Ekdale, Georg Schulz, Bert Müller, Bastien Mennecart

Supplementary Data 2: Bony labyrinths of all studied species in 7 views, arranged by clades following the composite phylogeny of Figure 1. “Left” and “right mirrored” on top refer to the side of the bony labyrinth.

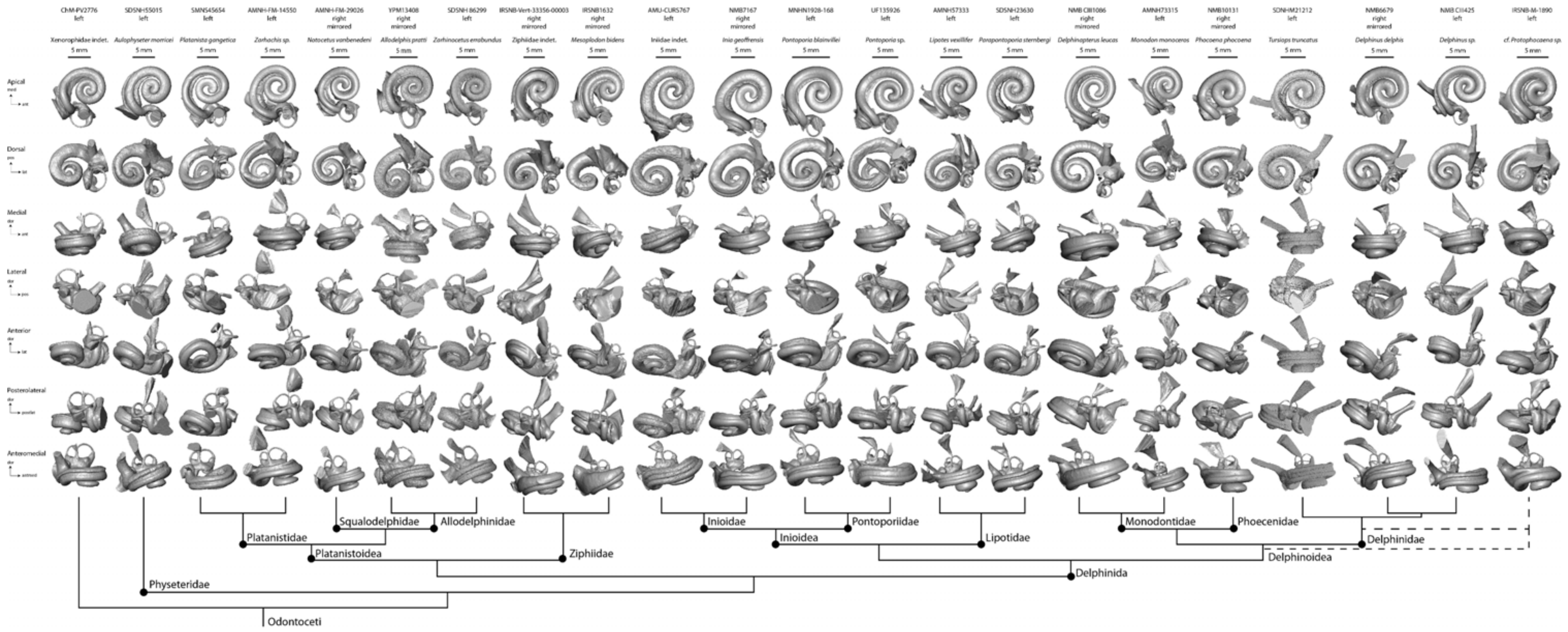

ChM-PV2776 left Xenorophidae indet. 5 mm
SDSNH55015 left Aulophyseter morricei 5 mm
SMNS45654 left Platanista gangetica 5 mm
AMNH-FM-14550 left Zarhachis sp. 5 mm
AMNH-FM-29026 right mirrored Notocetus vanbenedeni 5 mm
YPM13408 right mirrored Allodelphis pratti 5 mm
SDSNH 86299 left Zarhinocetus errabundus 5 mm
IRSNB-Vert-33356-00003 right mirrored Ziphiidae indet. 5 mm
IRSNB1632 right mirrored Mesoplodon bidens 5 mm
AMU-CURS767 left Iniidae indet. 5 mm
NMB7167 right mirrored Inia geoffrensis 5 mm
MNHN1928-168 left Pontoporia blainvillei 5 mm
UF135926 left Pontoporia sp. 5 mm
AMNH57333 left Lipotes vexillifer 5 mm
SDSNH23630 left Parapontoporia sternbergi 5 mm
NMB CIII1086 right mirrored Delphinapterus leucas 5 mm
AMNH73315 left Monodon monoceros 5 mm
NMB10131 right mirrored Phocoena phocoena 5 mm
SDNHM21212 left Tursiops truncatus 5 mm
NMB6679 right mirrored Delphinus delphis 5 mm
NMB CII425 left Delphinus sp. 5 mm
IRSNB-M-1890 left cf. Protophocaena sp. 5 mm
Apical
Dorsal
Medial
Lateral
Anterior
Posterolateral
Anteromedial
Squalodelphidae
Allodelphinidae
Platanistidae
Platanistoidea
Ziphiidae
Inioidae
Pontoporiidae
Inioidea
Lipotidae
Monodontidae
Phoecenidae
Delphinidae
Delphinoidea
Delphinida
Physeteridae
Odontoceti

The bony labyrinth of toothed whales reflects both phylogeny and habitat preferences.

Loïc Costeur, Camille Grohé, Gabriel Aguirre-Fernandez, Eric Ekdale, Georg Schulz, Bert Müller, Bastien Mennecart

Supplementary Data 4: PCAs on the dataset with original data and slid landmarks by Bending Energy and Procrustres Distances. CVAs on the dataset with original data and slid landmarks by Bending Energy and Procrustres Distances. The first two figures illustrate PCA and CVA for the whole bony labyrinth, the two next figures illustrate PCA and CVA for the semi-circular canals only and the last two figures illustrate PCA and CVA for the cochlea only.

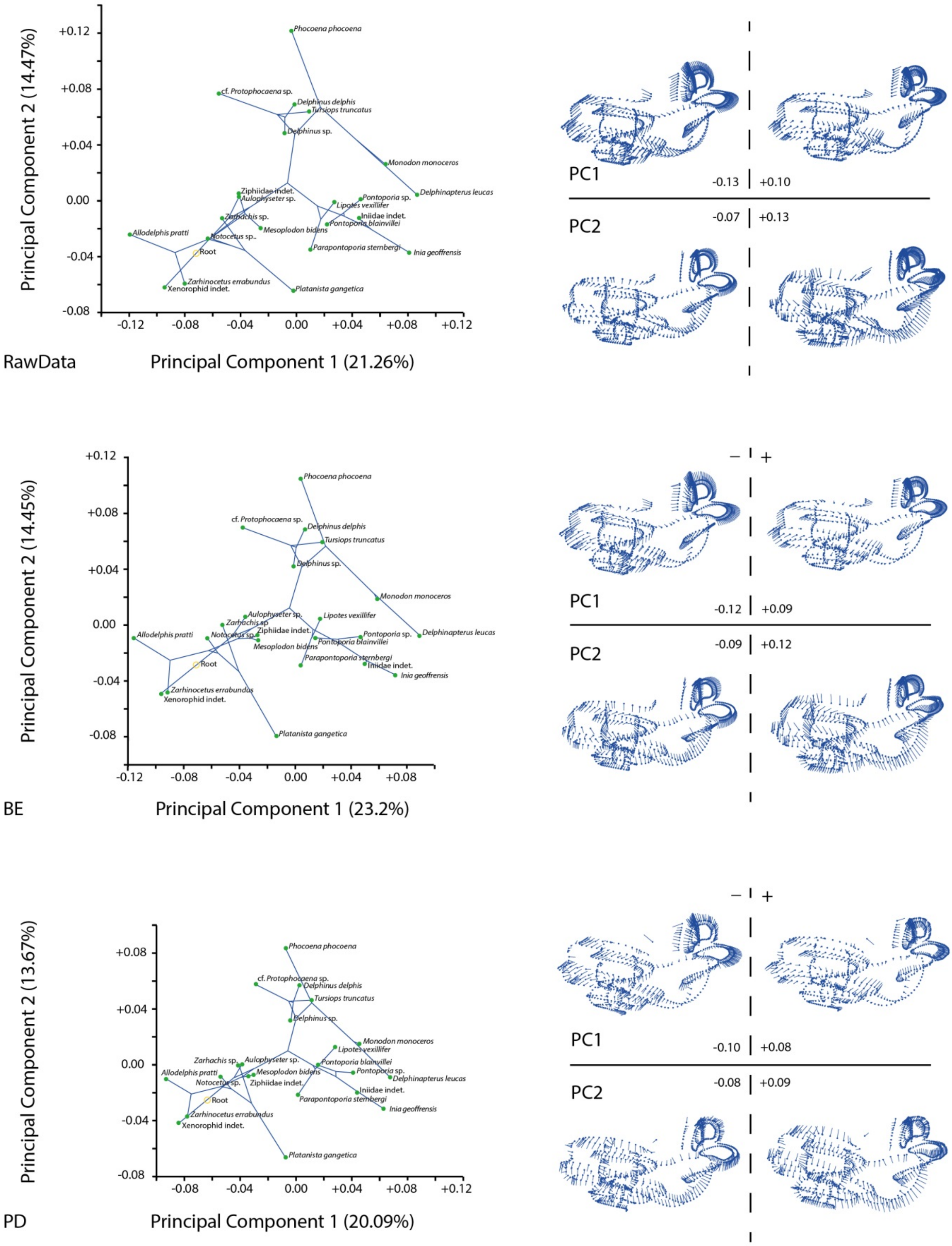

Principal Component 2 (14.47%)
Principal Component 1 (21.26%)
RawData
Phocoena phocoena
cf. Protophocaena sp.
Delphinus delphis
Tursiops truncatus
Delphinus sp.
Monodon monoceros
Delphinapterus leucas
Ziphiidae indet.
Aulophyseter sp.
Zarhachis sp.
Mesoplodon bidens
Notocetus sp.
Allodelphis pratti
Root
Zarhinocetus errabundus
Xenorophid indet.
Platanista gangetica
Pontoporia sp.
Lipotes vexillifer
Iniidae indet.
Pontoporia blainvillei
Parapontoporia sternbergi
Inia geoffrensis
PC1
-0.13
+0.10
PC2
-0.07
+0.13
Principal Component 2 (14.45%)
Principal Component 1 (23.2%)
BE
PC1
-0.12
+0.09
PC2
-0.09
+0.12
Principal Component 2 (13.67%)
Principal Component 1 (20.09%)
PD
PC1
-0.10
+0.08
PC2
-0.08
+0.09

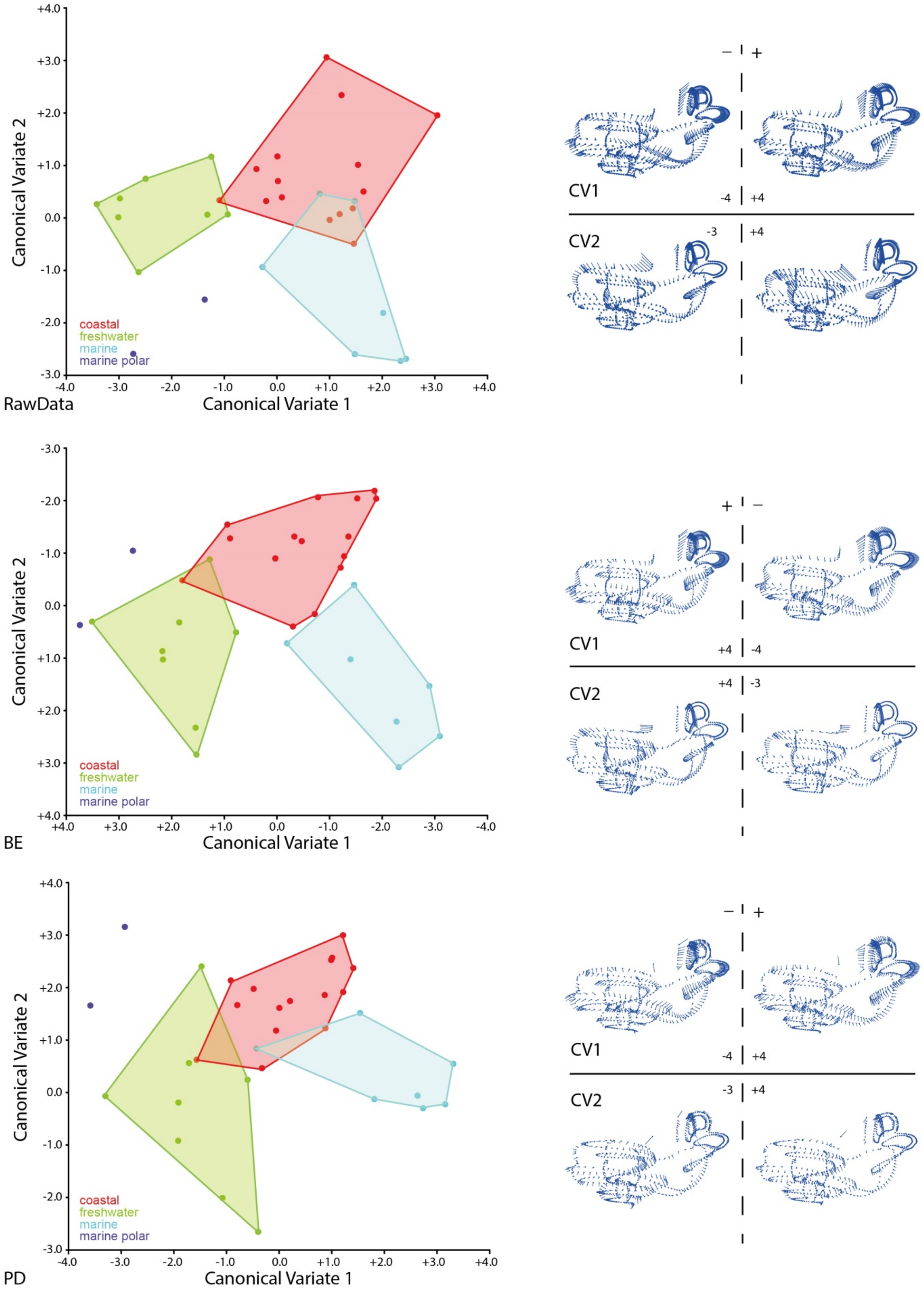

Canonical Variate 2
Canonical Variate 1
coastal
freshwater
marine
marine polar
RawData
BE
PD
CV1
CV2
−
+
-4
+4
-3
+4
+4
-4
+4
-3
+4.0
+3.0
+2.0
+1.0
0.0
-1.0
-2.0
-3.0
-4.0

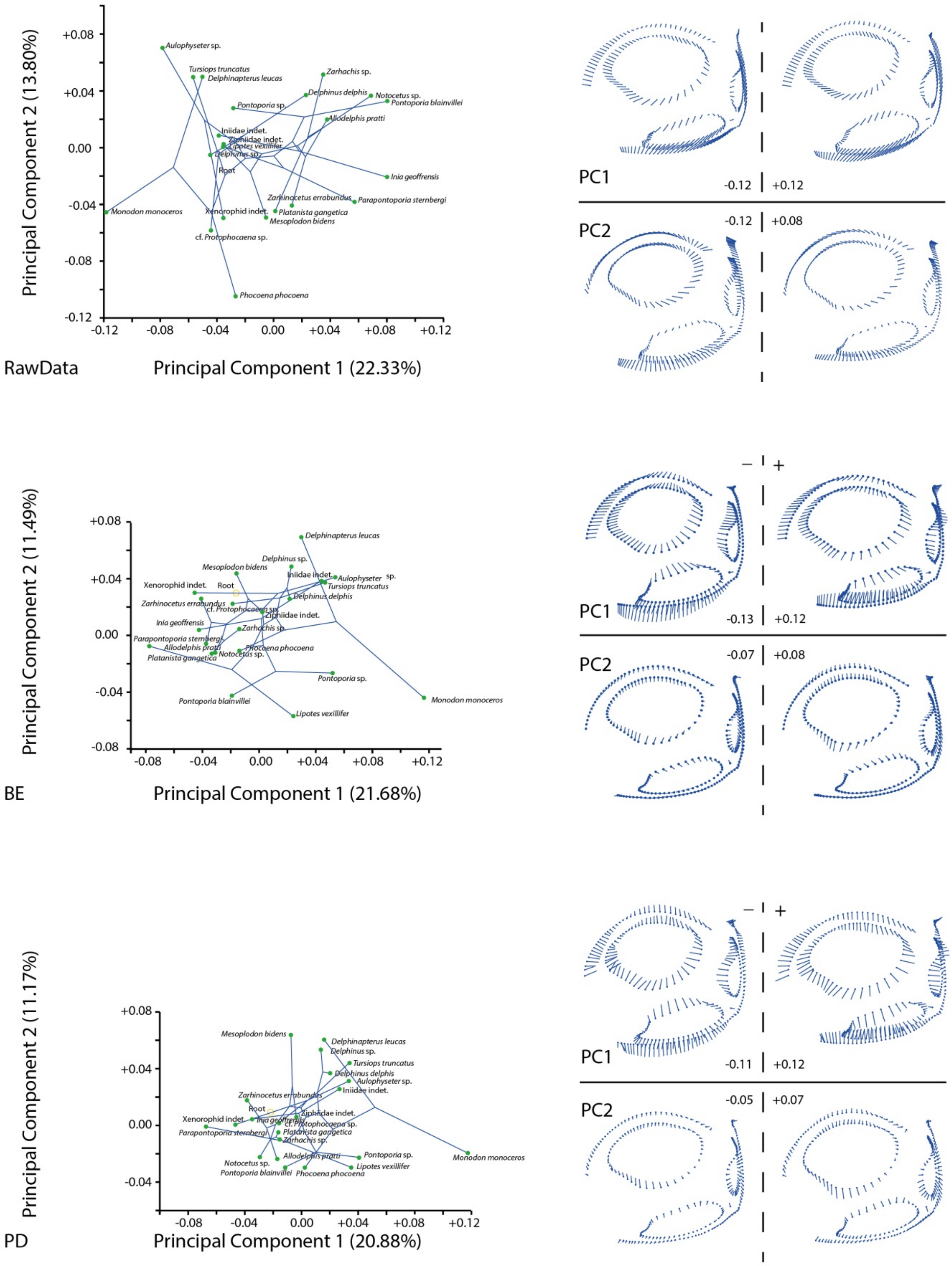

Principal Component 2 (13.80%)
Principal Component 1 (22.33%)
RawData
PC1
-0.12
+0.12
PC2
-0.12
+0.08
Principal Component 2 (11.49%)
Principal Component 1 (21.68%)
BE
PC1
-0.13
+0.12
PC2
-0.07
+0.08
Principal Component 2 (11.17%)
Principal Component 1 (20.88%)
PD
PC1
-0.11
+0.12
PC2
-0.05
+0.07

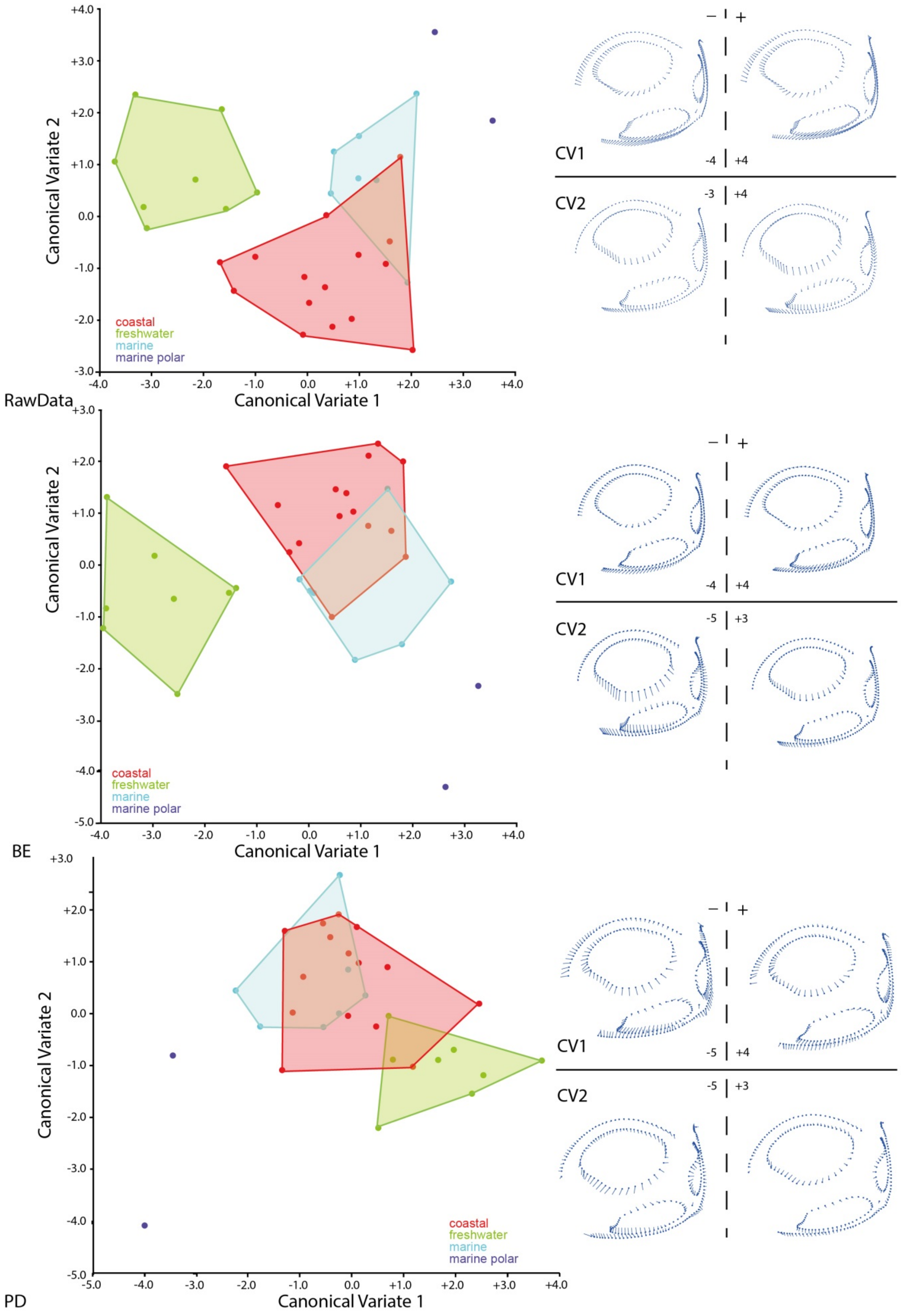

RawData
Canonical Variate 1
Canonical Variate 2
coastal
freshwater
marine
marine polar
+4.0
+3.0
+2.0
+1.0
0.0
-1.0
-2.0
-3.0
-4.0
CV1
CV2
−
+
-4
+4
-3
BE
Canonical Variate 1
Canonical Variate 2
coastal
freshwater
marine
marine polar
-5.0
-5
+3
PD
Canonical Variate 1
Canonical Variate 2
coastal
freshwater
marine
marine polar

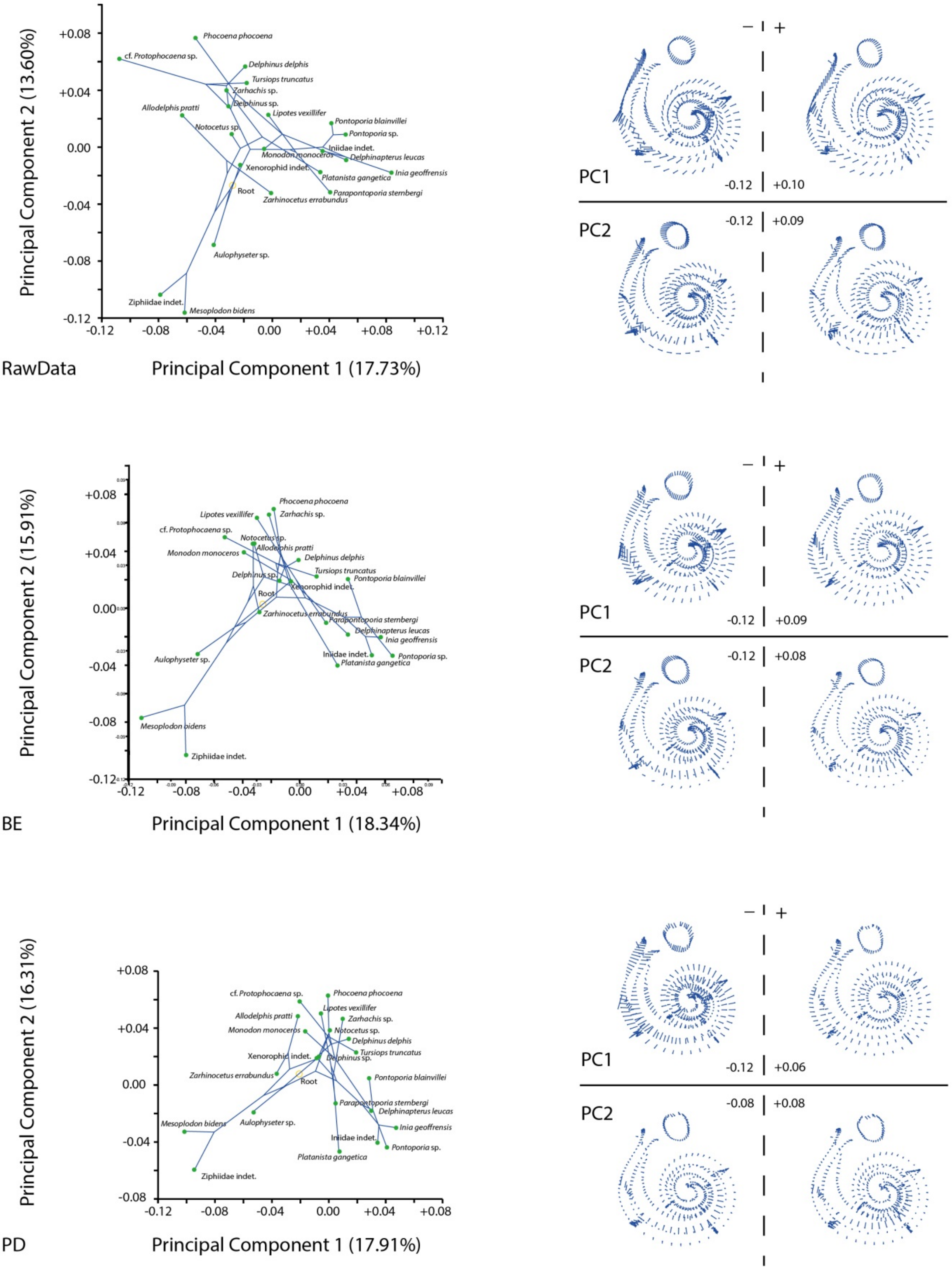

Principal Component 2 (13.60%)
Principal Component 1 (17.73%)
RawData
Phocoena phocoena
cf. Protophocaena sp.
Delphinus delphis
Tursiops truncatus
Zarhachis sp.
Allodelphis pratti
Delphinus sp.
Lipotes vexillifer
Pontoporia blainvillei
Pontoporia sp.
Notocetus sp.
Iniidae indet.
Monodon monoceros
Delphinapterus leucas
Xenorophid indet.
Inia geoffrensis
Platanista gangetica
Root
Zarhinocetus errabundus
Parapontoporia sternbergi
Aulophyseter sp.
Ziphiidae indet.
Mesoplodon bidens
PC1
-0.12
+0.10
PC2
-0.12
+0.09
Principal Component 2 (15.91%)
Principal Component 1 (18.34%)
BE
PC1
-0.12
+0.09
PC2
-0.12
+0.08
Principal Component 2 (16.31%)
Principal Component 1 (17.91%)
PD
PC1
-0.12
+0.06
PC2
-0.08
+0.08

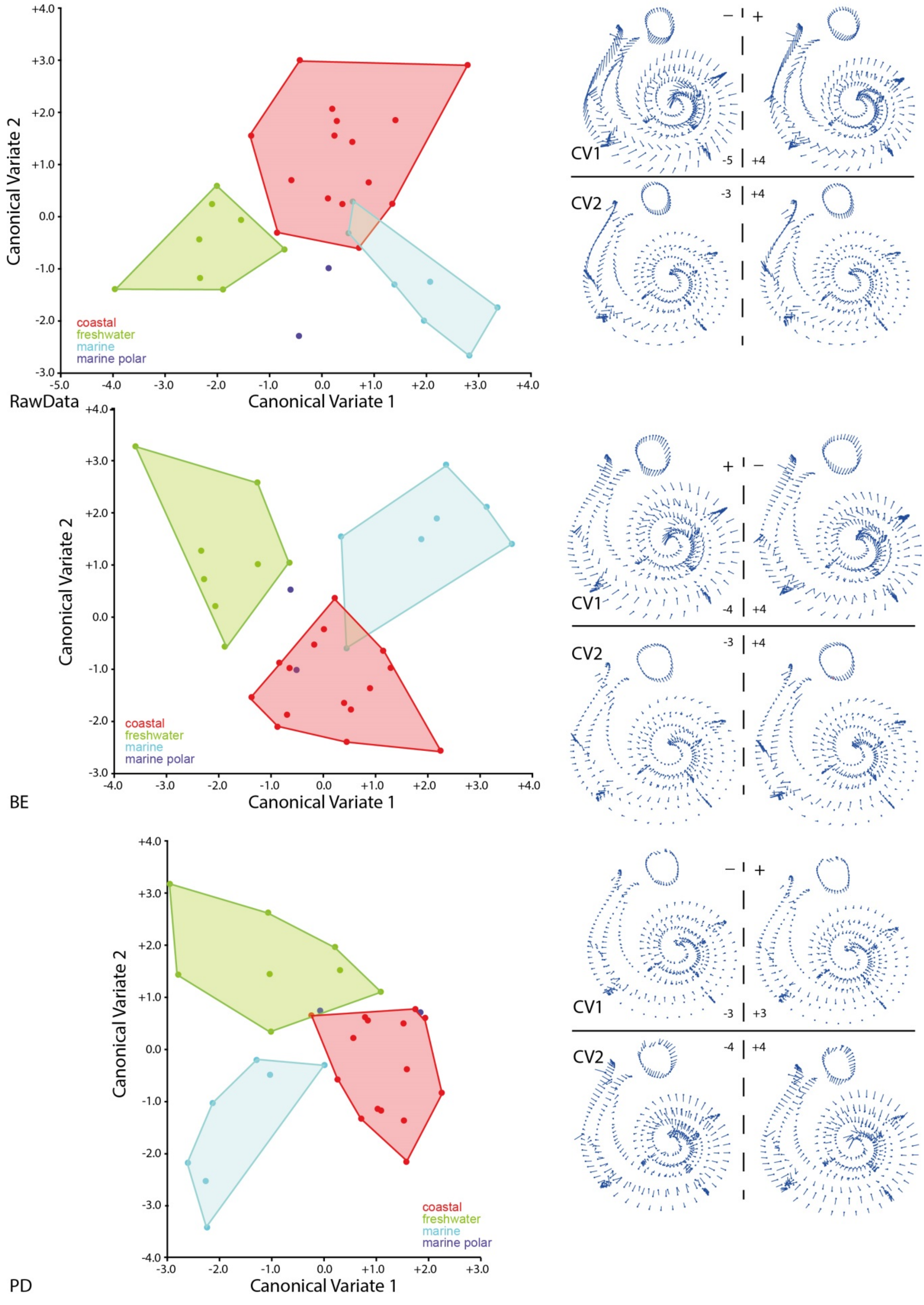

Canonical Variate 2
Canonical Variate 1
coastal
freshwater
marine
marine polar
RawData
BE
PD
CV1
CV2
−
+
-5
+4
-3
+4
-4
+3

The bony labyrinth of toothed whales reflects both phylogeny and habitat preferences.

Loïc Costeur, Camille Grohé, Gabriel Aguirre-Fernandez, Eric Ekdale, Georg Schulz, Bert Müller, Bastien Mennecart

Supplementary Information 5: Specimens information and scanning parameters.

Genus	Species	Collection Number	Institution	source	Scanning resolution (µm)	Scanner location
Allodelphis	pratti	YPM13408	Yale Peabody Museum	Boersma and Pyenson, 2016	21	Yale Peabody Museum of Natural History, CT
Aulophyseter	morricei	SDSNH55015	San Diego Natural History Museum	Ekdale, 2016	22.44	University of Texas CT-facility in Austin, TX (UTCT)
cf. Protophocaena	sp.	IRSNB-M-1890	Belgian Royal Institute of Natural Sciences	this study	40	Biomaterials Science Center, University of Basel
cf. Protophocaena	sp.	IRSNB-Vert-33356-00001	Belgian Royal Institute of Natural Sciences	this study	40	Biomaterials Science Center, University of Basel
Xenorophid	indet.	ChM-PV2776	Charleston Museum	Ekdale, 2016	25	University of Texas CT-facility in Austin, TX (UTCT)
Delphinapterus	leucas	NMB CIII1086	Naturhistorisches Museum Basel	this study	40	Biomaterials Science Center, University of Basel
Delphinus	delphis	NMB6679	Naturhistorisches Museum Basel	this study	40	Biomaterials Science Center, University of Basel
Delphinus	sp.	NMB CIII425	Naturhistorisches Museum Basel	this study	40	Biomaterials Science Center, University of Basel
Iniidae	indet.	AMU-CURS767	Coleccion Paleontologica de la Alcaldia Bolivariana de Urumaco, Venezuela	Aguirre-Fernandez et al. 2017	35	Biomaterials Science Center, University of Basel
Inia	geoffrensis	NMB7167	Naturhistorisches Museum Basel	Aguirre-Fernandez et al. 2017	30	Biomaterials Science Center, University of Basel
Inia	geoffrensis	NMB7168	Naturhistorisches Museum Basel	this study	30	Biomaterials Science Center, University of Basel
Lipotes	vexillifer	AMNH57333	American Museum of Natural History	this study	30.7	American Museum of Natural History, NY
Mesoplodon	bidens	IRSNB16232	Belgian Royal Institute of Natural Sciences	this study	35	Biomaterials Science Center, University of Basel
Monodon	monoceros	AMNH73315	American Museum of Natural History	this study	40.69	American Museum of Natural History, NY
Notocetus	vanbenedeni	AMNH-FM-29026	American Museum of Natural History	this study	38.21	American Museum of Natural History, NY
Parapontoporia	sternbergi	SDSNH20941	San Diego Natural History Museum	this study	35	Biomaterials Science Center, University of Basel
Parapontoporia	sternbergi	SDSNH23630	San Diego Natural History Museum	this study	35	Biomaterials Science Center, University of Basel
Phocaena	phocaena	NMB10131	Naturhistorisches Museum Basel	this study	40	Biomaterials Science Center, University of Basel
Platanista	gangetica	SMNS45654	Staatliches Museum für Naturkunde Stuttgart	this study	40	Biomaterials Science Center, University of Basel
Pontoporia	blainvillei	MNHN-1928-168	Muséum National df'Histoire Naturelle	this study	38	Biomaterials Science Center, University of Basel
Pontoporia	blainvillei	MNHN-1934-375	Muséum National df'Histoire Naturelle	this study	38	Biomaterials Science Center, University of Basel
Pontoporia	blainvillei	MNHN-1983-110	Muséum National df'Histoire Naturelle	this study	38	Biomaterials Science Center, University of Basel
Pontoporia	blainvillei	MNHN-1983-111	Muséum National df'Histoire Naturelle	Aguirre-Fernandez et al. 2017	38	Biomaterials Science Center, University of Basel
Pontoporia	sp.	UF135926	University of Florida Museum	this study	45	Biomaterials Science Center, University of Basel
Pontoporia	sp.	UF135935	University of Florida Museum	this study	45	Biomaterials Science Center, University of Basel
Pontoporia	sp.	UF-TRO26383	University of Florida Museum	this study	45	Biomaterials Science Center, University of Basel
Pontoporia	sp.	UF-TRO26394	University of Florida Museum	this study	45	Biomaterials Science Center, University of Basel
Tursiops	truncatus	SDNHM21212	San Diego Natural History Museum	Ekdale, 2016	40	University of Texas CT-facility in Austin, TX (UTCT)
Zarhachis	sp.	AMNH-FM-145500	American Museum of Natural History	this study	43.46	American Museum of Natural History, NY
Zarhinocetus	errabundus	SDSNH 86299	San Diego Natural History Museum	Ekdale, 2016	16.46	University of Texas CT-facility in Austin, TX (UTCT)
Ziphiidae	indet.	IRSNB-Vert-33356-00002	Belgian Royal Institute of Natural Sciences	this study	40	Biomaterials Science Center, University of Basel
Ziphiidae	indet.	IRSNB-Vert-33356-00003	Belgian Royal Institute of Natural Sciences	this study	40	Biomaterials Science Center, University of Basel

The bony labyrinth of toothed whales reflects both phylogeny and habitat preferences.

Loïc Costeur, Camille Grohé, Gabriel Aguirre-Fernandez, Eric Ekdale, Georg Schulz, Bert Müller, Bastien Mennecart

Supplementary Information 6: Methods and statistics used in this study.

Methods

Geometric morphometrics analyses

Bony labyrinths were first segmented using Avizo Standard Edition 7.0 ®, 8.0 ® (Visualization Sciences Group, an FEI Company, 2013), and AVIZO Lite ® (2016). Segmentation of structures that open outside the periotic (cochlear aqueduct and endolymphatic sac) were carried out up to the aperture and stopped when they opened.
Left bony labyrinths were preferably selected when available. When not, right ones were selected and reflected using an application of Landmark Editor 3.6 software (Wiley, 2006).
Aguirre-Fernandez et al. (2017) detail the 3D geometric morphometrics analyses applied here. We used Landmark Editor to digitize the specimens with a large number of landmarks (i.e., 717). This brings strong congruence with the actual shape of the bony labyrinth. After collecting the landmarks, two other approaches were tested where we slid the semi-landmarks along the curves during the superimposition process of the Generalized Procrustes Analysis minimizing Bending Energy (BE) and the Procrutes Distances (PD) (see Tseng et al. (2015) and Grohé et al. (2016)). Both methods were carried out with the Geomorph package implemented in R, version 3.1.1. Finally, shape variation in bony labyrinth morphology (disparity and similarity using PCA and CVA) was investigated using the geometric morphometrics and statistical methods of MorphoJ 1.06d (Klingenberg, 2011).
All methods were applied to several datasets consisting of either the entire bony labyrinth or subsets as defined by the semi-circular canals only (i.e., 256; Landmarks 1 to 256) and the cochlea only (i.e., 451 landmarks; Landmarks 257 to 707). The landmarks 708 to 717 represent the vestibular aqueduct. The three datasets were then analysed without sliding (Raw Data) or using both criteria (BE and PD): nine datasets were then created. We run the multivariate analyses on the three datasets for comparative purposes.
Principal Component Analysis (PCA) is used to visualise the overall shape variation among specimens. Distances in this space are real measures of differences among specimens. In the space defined by PC axes, groups may appear as clusters if there is similarity of shape within them. It is noteworthy that the axes describe variation within the overall sample and not morphological differences between the groups.

The phylogenetic signal was tested by first mapping the tree to the shape data (polymorphospace), via squared-change parsimony, and calculating the length of the tree. A permutation procedure was then used to shuffle the shape data among the tips of the tree. Finally, the permuted tree lengths were compared to the original tree (randomised rounds: 10000) (Klingenberg and Gidaszewski, 2010).
The phylogenetic hypothesis (presented on Figure 1) is based on the combined data of Barnes (2006), Hassanin et al. (2012), and Geisler et al. (2014). In contrast to Lambert & Post (2005), we suggest that the specimens attributed to cf. *Protophocaena* from Belgium are not pontoporiids, but basal delphinids based on the shape of the bony labyrinth. The phylogenetic tree was created using Mesquite 3.04 (Maddison and Maddison 2010).

The impact of allometry on the shape has been tested using a MorphoJ routine. A regression using the PC scores and the centroid size was performed. A permutation test (number of rounds 10,000) gave the p-values.
Canonical Variate Analysis (CVA) was applied to all three above-mentioned datasets (RawData, BE, PD). It is an ordination method that maximises between group variation, relative to within group variation, according to the specified grouping variable (randomised rounds: 10000). Distances in this space are distorted, and do not represent the actual

difference between specimens or groups, but are instead scaled relative to within group variation. This analysis allows us to investigate shape differences that may be related to habitat preference (freshwater, coastal, fully marine, and marine polar animals) by performing permutation tests of pairwise distances (using Mahalanobis and Procrustes distances) between the grouping variable (here the type of habitat). It is important to note that CVA has some important assumptions regarding the number of specimens and groups relative to the number of variables in the analysis (see Mitteroecker and Bookstein, 2011, particularly Figures 3-5). The group that has few taxa (in our dataset: " marine polar") is problematic, because the method uses within-group versus between group differences to calculate the axes. And thus in small groups, the difference between them and the others will be incorrectly estimated. These morphological modifications are highlighted in the results obtained from the different datasets. Nevertheless, more thorough conclusions can be provided on the relationship between shape and ecology based on the three other habitats.

References

Aguirre-Fernández, G., Mennecart, B., Sánchez-Villagra, M., Sánchez, R. & Costeur, L. A dolphin fossil ear bone from the northern Neotropics–insights into habitat transitions in iniid evolution. *J. Vert. Paleontol.* **37(3)**, e1315817 (2017).

Barnes, L. G. A phylogenetic analysis of the superfamily Platanistoidea (Mammalia, Cetacea, Odontoceti). *Beitr. Paläont.* **30**, 24–42 (2006).

Geisler, J. H., Colbert, M. W. & Carew, J. L. A new fossil species supports an early origin for toothed whale echolocation. *Nature* **508**, 383–386 (2014).

Grohé, C., Tseng, J., Lebrun, R., Boistel, R. & Flynn, J. J. Bony labyrinth shape variation in extant Carnivora: a case study of Musteloidea. *J. Anat.* **228(3)**, 366–383 (2016).

Hassanin, A. *et al*. Pattern and timing of diversification of Cetartiodactyla (Mammalia, Laurasiatheria), as revealed by a comprehensive analysis of mitochondrial genomes. *C. R. Biol.* **335**, 32–50 (2012).

Klingenberg, C. P. MorphoJ: an integrated software package for geometric morphometrics. *Mol. Ecol. Resour.* **11(2)**, 353–357 (2001).

Klingenberg, C. P.& Gidaszewski, N. A. Testing and quantifying phylogenetic signals and homoplasy in morphometric data. *Systematic Biology*, **59(3),** 245–261 (2010).

Lambert, O. & Post K. First European pontoporiid dolphins (Mammalia: Cetacea, Odontoceti) from the Miocene of Belgium and the Netherlands. *Deinsea* **11**, 7–20 (2005).

Mitteroecker, P. & Bookstein, F. L. Linear Discrimination, Ordination, and the Visualization of Selection Gradients in Modern Morphometrics. *Evol. Biol.* **38(1)**, 100–114 (2011).

Tseng Z. J. & Flynn, J. J.. Are cranial biomechanical simulation data linked to known diets in extant taxa? A method for applying diet-biomechanics linkage models to infer feeding capability of extinct species. *PLoS ONE* **10(4),** e0124020 (2015).

Wiley, D. F. *Landmark Editor* 3.0. Institute for Data Analysis and Visualization, University of California, Davis (http://graphics. idav.ucdavis.edu/research/EvoMorph) (2006).

The bony labyrinth of toothed whales reflects both phylogeny and habitat preferences.

Loïc Costeur, Camille Grohé, Gabriel Aguirre-Fernandez, Eric Ekdale, Georg Schulz, Bert Müller, Bastien Mennecart

Supplementary Information 7: References for data used in Figure 1.

Age of the nodes
Aguirre-Fernández, G., Mennecart, B., Sánchez-Villagra, M., Sánchez, R. & Costeur, L. A dolphin fossil ear bone from the northern Neotropics–insights into habitat transitions in iniid evolution. *J. Vert. Paleontol.* **37(3)**, e1315817 (2017).

Barnes, L. G. Fossil pontoporiid dolphins (Mammalia: Cetacea) from the Pacific coast of North America. *Contrib. sci. (Los Angel. Calif.)* **363**, 1–3 (1985).

Barnes, L. G. A phylogenetic analysis of the superfamily Platanistoidea (Mammalia, Cetacea, Odontoceti). *Beitr. Paläont.* **30**, 24–42 (2006).

de Muizon, C. The affinities of *Notocetus vanbenedeni*, an Early Miocene Platanistoid (Cetacea, Mammalia) from Patagonia, Southern Argentina. *Am. Mus. Novit.* **2904**, 1–27 (1987).

Ekdale, E. G. Morphological variation among the inner ears of extinct and extant baleen whales (Cetacea: Mysticeti). *J. Morphol.* **277(12)**, 1599–1615 (2016).

Hassanin, A. *et al*. Pattern and timing of diversification of Cetartiodactyla (Mammalia, Laurasiatheria), as revealed by a comprehensive analysis of mitochondrial genomes. *C. R. Biol.* **335**, 32–50 (2012).

Kimura, T. & Barnes, L. G. New Miocene fossil Allodelphinidae (Cetacea, Odontoceti, Platanistoidea) from the North Pacific Ocean. *Bull. Gunma Mus. Natu. Hist.* **20**, 1–58 (2016).

Lambert, O. & Post K. First European pontoporiid dolphins (Mammalia: Cetacea, Odontoceti) from the Miocene of Belgium and the Netherlands. *Deinsea* **11**, 7–20 (2005).

Murray, B. W. & White, B. N. Sequence variation at the major histocompatibility complex DRB loci in beluga (Delphinapterus leucas) and narwhal (Monodon monoceros). *Immunogenetics* **48**, 242–252 (1998).

Webb, S. D., Hulbert, R. C., Morgan, G. S. & Evans, H. F. Terrestrial mammals of the Palmetto Fauna (early Pliocene, latest Hemphillian) from the Central Florida Phosphate District. *Science Series* **41**, 293–312 (2008).

Tree topography
Barnes, L. G. A phylogenetic analysis of the superfamily Platanistoidea (Mammalia, Cetacea, Odontoceti). *Beitr. Paläont.* **30**, 24–42 (2006).

Geisler, J. H., Colbert, M. W. & Carew, J. L. A new fossil species supports an early origin for toothed whale echolocation. *Nature* **508**, 383–386 (2014).

Hassanin, A. *et al.* Pattern and timing of diversification of Cetartiodactyla (Mammalia, Laurasiatheria), as revealed by a comprehensive analysis of mitochondrial genomes. *C. R. Biol.* **335**, 32–50 (2012).

Ecological and palaeoecological inferences
Aguirre-Fernández, G., Mennecart, B., Sánchez-Villagra, M., Sánchez, R. & Costeur, L. A dolphin fossil ear bone from the northern Neotropics–insights into habitat transitions in iniid evolution. *J. Vert. Paleontol.* **37(3)**, e1315817 (2017).

Barnes, L. G. A phylogenetic analysis of the superfamily Platanistoidea (Mammalia, Cetacea, Odontoceti). B*eitr. Paläont.* **30**, 24–42 (2006).

Bianucci, G., Urbina, M. & Lambert, O. A new record of *Notocetus vanbenedeni* (Squalodelphinidae, Odontoceti, Cetacea) from the Early Miocene of Peru. *C. R. Palevol* **14**, 5–13 (2015).

Boersma, A. T. & Pyenson, N. D. *Albicetus oxymycterus*, a new generic name and redescription of a basal physeteroid (Mammalia, Cetacea) from the Miocene of California, and the evolution of body size in sperm whales. *PLoS ONE* **10(12)**, e0135551 (2015).

Boessenaker, R. W. & Poust, A. W. Freshwater occurrence of the extinct dolphin Parapontoporia (Cetacea: Lipotidae) from the Upper Pliocene nonmarine Tulare Formation of California. *Palaeontology* **58(3)**, 489–496 (2015).

de Muizon, C. The affinities of *Notocetus vanbenedeni*, an Early Miocene Platanistoid (Cetacea, Mammalia) from Patagonia, Southern Argentina. *Am. Mus. Novit.* **2904**, 1–27 (1987).

Fordyce, R. E. Whale evolution and Oligocene southern ocean environments. *Palaeogeogr. Palaeoclimatol., Palaeoecol.* **31**, 319–336 (1990).

Kimura, T. & Barnes, L. G. New Miocene fossil Allodelphinidae (Cetacea, Odontoceti, Platanistoidea) from the North Pacific Ocean. *Bull. Gunma Mus. Natu. Hist.* **20**, 1–58 (2016).

Lambert, O., de Muizon, C. & Bianucci, G. The most basal beaked whale *Ninoziphius platyrostris* Muizon, 1983: clues on the evolutionary history of the family Ziphiidae (Cetacea: Odontoceti). *Zool. J Linn. Soc.* **167**, 569–598 (2013).

Nowak, R. M. *Walker's Mammals of the World.* Baltimore: The John Hopkins University Press. 2015 p (1999).

The bony labyrinth of toothed whales reflects both phylogeny and habitat preferences.

Loïc Costeur, Camille Grohé, Gabriel Aguirre-Fernandez, Eric Ekdale, Georg Schulz, Bert Müller, Bastien Mennecart

Supplementary Information 8: Nomenclature of the bony labyrinth; specimen NMB 7167, *Inia geoffrensis.*

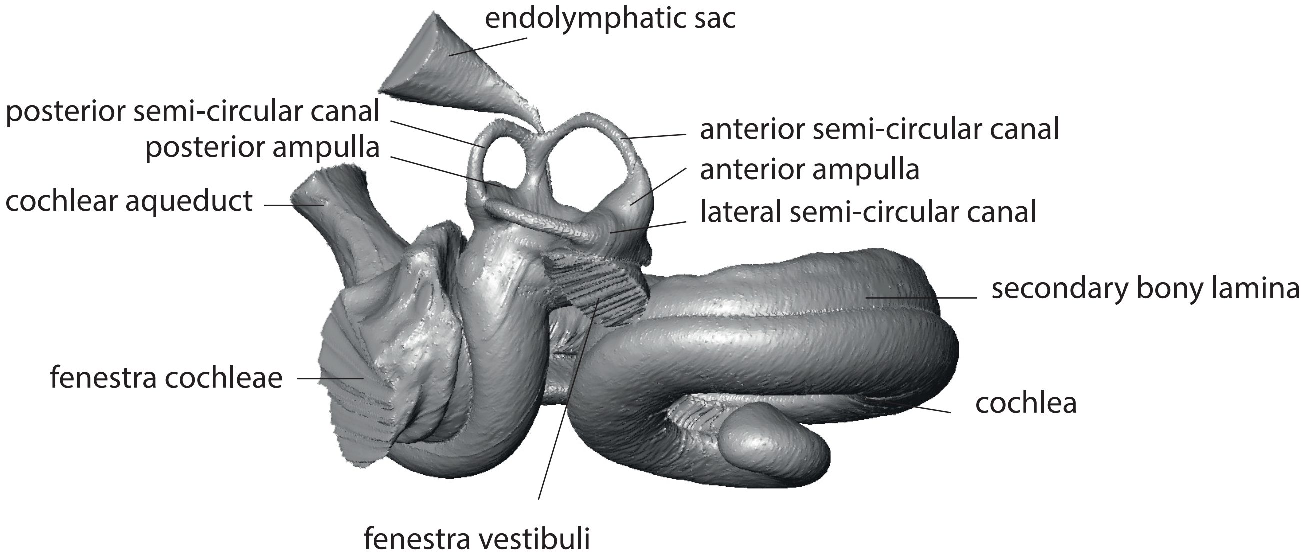
endolymphatic sac
posterior semi-circular canal
posterior ampulla
cochlear aqueduct
anterior semi-circular canal
anterior ampulla
lateral semi-circular canal
secondary bony lamina
fenestra cochleae
cochlea
fenestra vestibuli

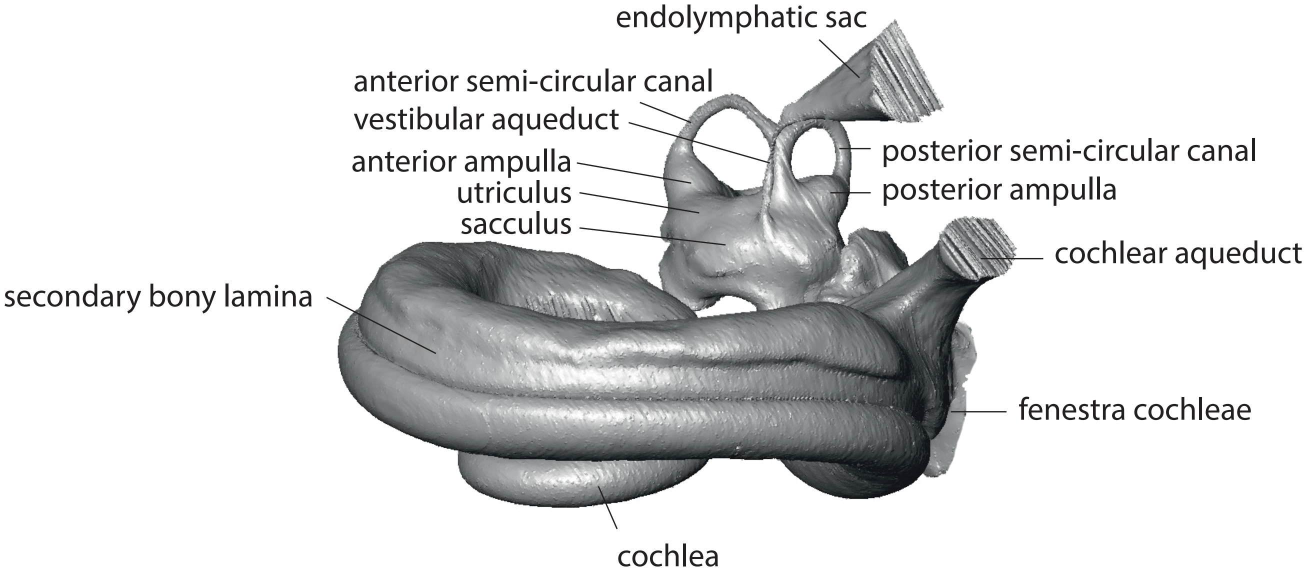
endolymphatic sac
anterior semi-circular canal
vestibular aqueduct
anterior ampulla
utriculus
sacculus
posterior semi-circular canal
posterior ampulla
cochlear aqueduct
secondary bony lamina
fenestra cochleae
cochlea

The bony labyrinth of toothed whales reflects both phylogeny and habitat preferences.

Loïc Costeur, Camille Grohé, Gabriel Aguirre-Fernandez, Eric Ekdale, Georg Schulz, Bert Müller, Bastien Mennecart

Supplementary Data 9: Results of the geometric morphometric analyses for the three datasets using the original data (RawData) and the two sliding protocols (Bending Energy and Procrustres Distances).

New Procrustes fit:

Dataset 'BE_All'

717 landmarks in 3 dimensions.

The dataset contains 32 observations, of which 32 are included for analyses.

Average shape:

Lmk.	Axis 1 (x)	Axis 2 (y)	Axis 3 (z)
1	-0.02961287	0.00007956	-0.01542495
2	-0.02688184	-0.01715574	-0.00513417
3	-0.02726320	-0.01727447	-0.00565098
4	-0.02761520	-0.01723263	-0.00634578
5	-0.02787930	-0.01703551	-0.00709598
6	-0.02811674	-0.01673799	-0.00785903
7	-0.02833863	-0.01635572	-0.00862419
8	-0.02857050	-0.01584521	-0.00940742
9	-0.02877505	-0.01525608	-0.01017585
10	-0.02898250	-0.01461619	-0.01093455
11	-0.02916764	-0.01388747	-0.01174577
12	-0.02929433	-0.01310197	-0.01247140
13	-0.02939805	-0.01228808	-0.01310077
14	-0.02945195	-0.01143297	-0.01367248
15	-0.02946972	-0.01053827	-0.01414700
16	-0.02941046	-0.00960975	-0.01453705
17	-0.02934366	-0.00863582	-0.01485643
18	-0.02920144	-0.00765484	-0.01506528
19	-0.02898743	-0.00668120	-0.01513642

20	-0.02874612	-0.00566804	-0.01516863
21	-0.02829323	-0.00431345	-0.01505532
22	-0.02785277	-0.00313155	-0.01477510
23	-0.02736512	-0.00203913	-0.01433250
24	-0.02672310	-0.00112912	-0.01362951
25	-0.02600015	-0.00051806	-0.01272510
26	-0.02517343	0.00003406	-0.01158476
27	-0.02427858	0.00049156	-0.01018811
28	-0.02325083	0.00074414	-0.00855101
29	-0.02212164	0.00067072	-0.00678330
30	-0.02154042	0.00024057	-0.00544540
31	-0.02113444	-0.00070394	-0.00430513
32	-0.02098579	-0.00186264	-0.00356623
33	-0.02096887	-0.00318391	-0.00294485
34	-0.02108271	-0.00469324	-0.00238386
35	-0.02133778	-0.00633864	-0.00188889
36	-0.02173256	-0.00809185	-0.00146348
37	-0.02227817	-0.00990887	-0.00112538
38	-0.02289490	-0.01153259	-0.00134231
39	-0.02319160	-0.01211671	-0.00176420
40	-0.02360232	-0.01272447	-0.00232029
41	-0.02404658	-0.01335561	-0.00288818
42	-0.02447239	-0.01400222	-0.00337117
43	-0.02491398	-0.01467447	-0.00375340
44	-0.02541278	-0.01537931	-0.00402765
45	-0.02594728	-0.01607269	-0.00425744
46	-0.02649058	-0.01670432	-0.00461637
47	-0.02739189	-0.02131576	-0.00331920

48	-0.02762199	-0.02120635	-0.00376542
49	-0.02784330	-0.02105954	-0.00424012
50	-0.02808388	-0.02085317	-0.00483128
51	-0.02832169	-0.02058738	-0.00547844
52	-0.02856412	-0.02024060	-0.00616852
53	-0.02881270	-0.01985963	-0.00690788
54	-0.02904427	-0.01943267	-0.00766381
55	-0.02929223	-0.01896058	-0.00842875
56	-0.02953362	-0.01847111	-0.00931756
57	-0.02974282	-0.01789935	-0.01013716
58	-0.02994083	-0.01730244	-0.01090713
59	-0.03013274	-0.01668564	-0.01164350
60	-0.03030021	-0.01605320	-0.01235490
61	-0.03041069	-0.01536534	-0.01295610
62	-0.03053785	-0.01471604	-0.01360805
63	-0.03063087	-0.01403706	-0.01416304
64	-0.03070848	-0.01331736	-0.01468989
65	-0.03072993	-0.01260763	-0.01519698
66	-0.03071036	-0.01189696	-0.01561249
67	-0.03067375	-0.01116113	-0.01601025
68	-0.03060896	-0.01042235	-0.01634418
69	-0.03050566	-0.00967427	-0.01663010
70	-0.03036984	-0.00892578	-0.01685881
71	-0.03022189	-0.00816635	-0.01700094
72	-0.03003824	-0.00733692	-0.01708447
73	-0.02987233	-0.00653742	-0.01718934
74	-0.02964881	-0.00575210	-0.01717885
75	-0.02944187	-0.00505604	-0.01711339

76	-0.02923186	-0.00436047	-0.01707613
77	-0.02897648	-0.00361326	-0.01697326
78	-0.02868680	-0.00286445	-0.01678003
79	-0.02836075	-0.00206065	-0.01653012
80	-0.02800098	-0.00124777	-0.01627846
81	-0.02758535	-0.00039414	-0.01604932
82	-0.02711088	0.00052260	-0.01581910
83	-0.02660062	0.00144529	-0.01551448
84	-0.03370399	0.01055076	-0.00519351
85	-0.03423478	0.01046795	-0.00527142
86	-0.03479850	0.01033496	-0.00548411
87	-0.03526775	0.01017186	-0.00581784
88	-0.03569485	0.00998337	-0.00623081
89	-0.03603485	0.00977635	-0.00667510
90	-0.03632333	0.00956153	-0.00715773
91	-0.03652723	0.00935738	-0.00764406
92	-0.03673201	0.00912097	-0.00817761
93	-0.03687399	0.00884952	-0.00873755
94	-0.03698385	0.00851927	-0.00931452
95	-0.03700982	0.00818631	-0.00989772
96	-0.03694444	0.00784582	-0.01048961
97	-0.03682216	0.00747347	-0.01106694
98	-0.03663121	0.00707753	-0.01164251
99	-0.03637392	0.00667805	-0.01220265
100	-0.03602070	0.00625090	-0.01273214
101	-0.03558115	0.00582786	-0.01320688
102	-0.03506976	0.00546295	-0.01363739
103	-0.03439488	0.00501406	-0.01398466

104	-0.03360900	0.00460070	-0.01419126
105	-0.03284100	0.00424215	-0.01431475
106	-0.03206296	0.00395038	-0.01435639
107	-0.03126935	0.00367907	-0.01429864
108	-0.03056336	0.00348121	-0.01414620
109	-0.02975833	0.00328496	-0.01391283
110	-0.02897592	0.00322402	-0.01356743
111	-0.02820134	0.00326181	-0.01310731
112	-0.02679511	0.00347265	-0.01166095
113	-0.02575236	0.00396272	-0.01026455
114	-0.02499029	0.00441572	-0.00903402
115	-0.02456950	0.00504613	-0.00790649
116	-0.02442030	0.00559269	-0.00704476
117	-0.02445594	0.00609845	-0.00620998
118	-0.02481072	0.00676610	-0.00548775
119	-0.02559303	0.00763196	-0.00500053
120	-0.02659039	0.00845338	-0.00491318
121	-0.02720599	0.00901420	-0.00513043
122	-0.02793478	0.00949700	-0.00543984
123	-0.02870989	0.00988815	-0.00571470
124	-0.02952246	0.01021178	-0.00585584
125	-0.03036780	0.01045651	-0.00586040
126	-0.03121398	0.01061490	-0.00569915
127	-0.03207727	0.01068921	-0.00548225
128	-0.03281907	0.01067149	-0.00530317
129	-0.03522247	0.01348301	-0.00274899
130	-0.03566538	0.01321856	-0.00296691
131	-0.03606731	0.01296748	-0.00319753

132	-0.03652008	0.01262572	-0.00353562
133	-0.03698027	0.01231427	-0.00390405
134	-0.03740770	0.01207504	-0.00426865
135	-0.03778600	0.01181143	-0.00464837
136	-0.03812073	0.01151785	-0.00508583
137	-0.03843249	0.01124520	-0.00554624
138	-0.03868341	0.01098971	-0.00606781
139	-0.03896539	0.01070592	-0.00679466
140	-0.03920901	0.01037214	-0.00749449
141	-0.03936874	0.01000108	-0.00818368
142	-0.03950443	0.00966471	-0.00883804
143	-0.03952244	0.00928987	-0.00946444
144	-0.03949690	0.00889262	-0.01010061
145	-0.03949746	0.00849871	-0.01075889
146	-0.03943780	0.00813839	-0.01136594
147	-0.03928790	0.00773950	-0.01197694
148	-0.03904430	0.00734980	-0.01252591
149	-0.03877991	0.00697174	-0.01311562
150	-0.03843138	0.00658604	-0.01362178
151	-0.03802534	0.00616284	-0.01413524
152	-0.03758061	0.00576744	-0.01461147
153	-0.03707709	0.00538446	-0.01503228
154	-0.03651047	0.00500053	-0.01544309
155	-0.03590778	0.00464218	-0.01577309
156	-0.03531226	0.00427466	-0.01606339
157	-0.03445526	0.00388701	-0.01631146
158	-0.03349783	0.00353956	-0.01646398
159	-0.03256729	0.00322715	-0.01654123

160	-0.03161213	0.00290525	-0.01649008
161	-0.03067548	0.00256920	-0.01636964
162	-0.02976746	0.00226678	-0.01623153
163	-0.02875676	0.00196918	-0.01601596
164	-0.02768511	0.00169958	-0.01578716
165	-0.02660062	0.00144529	-0.01551448
166	-0.03504173	-0.01271315	0.00819804
167	-0.03568491	-0.01268801	0.00851005
168	-0.03640602	-0.01250622	0.00872035
169	-0.03710697	-0.01223304	0.00886615
170	-0.03780282	-0.01184930	0.00890302
171	-0.03850091	-0.01137873	0.00889719
172	-0.03919870	-0.01081092	0.00885082
173	-0.03986695	-0.01014890	0.00873099
174	-0.04053998	-0.00940796	0.00855712
175	-0.04119649	-0.00854854	0.00831410
176	-0.04165337	-0.00788913	0.00808366
177	-0.04206452	-0.00720309	0.00783678
178	-0.04240404	-0.00650693	0.00756766
179	-0.04264916	-0.00581436	0.00724597
180	-0.04284504	-0.00514014	0.00693075
181	-0.04299798	-0.00443790	0.00659952
182	-0.04307620	-0.00372134	0.00622382
183	-0.04308110	-0.00299547	0.00581510
184	-0.04301728	-0.00223126	0.00535587
185	-0.04287295	-0.00156433	0.00491292
186	-0.04264401	-0.00086461	0.00446056
187	-0.04234234	-0.00020192	0.00401337

188	-0.04195557	0.00052564	0.00354093
189	-0.04151779	0.00113203	0.00306240
190	-0.04105076	0.00167816	0.00262708
191	-0.04050230	0.00222521	0.00216933
192	-0.03986990	0.00280471	0.00172957
193	-0.03923045	0.00334995	0.00139757
194	-0.03818764	0.00422462	0.00088592
195	-0.03705966	0.00486549	0.00043662
196	-0.03583831	0.00534181	0.00008386
197	-0.03457400	0.00566068	-0.00016251
198	-0.03322842	0.00577310	-0.00027826
199	-0.03182264	0.00564142	-0.00029500
200	-0.03036488	0.00539864	-0.00023035
201	-0.02891513	0.00495813	-0.00012057
202	-0.02736309	0.00427893	0.00004555
203	-0.02598863	0.00302616	0.00020501
204	-0.02512585	0.00148168	0.00045965
205	-0.02463905	-0.00006928	0.00069813
206	-0.02440279	-0.00166216	0.00105470
207	-0.02437186	-0.00326696	0.00156217
208	-0.02471362	-0.00492516	0.00212733
209	-0.02548901	-0.00657576	0.00269785
210	-0.02664011	-0.00811174	0.00323109
211	-0.02816935	-0.00946709	0.00369457
212	-0.02909076	-0.00998299	0.00400988
213	-0.03005181	-0.01041578	0.00438579
214	-0.03098065	-0.01083647	0.00483391
215	-0.03183311	-0.01128245	0.00537222

216	-0.03253553	-0.01170361	0.00593937
217	-0.03320076	-0.01209291	0.00655490
218	-0.03377822	-0.01243506	0.00717057
219	-0.03437975	-0.01262330	0.00771527
220	-0.03481423	-0.01748033	0.01003175
221	-0.03567265	-0.01700530	0.01026749
222	-0.03648573	-0.01646786	0.01044655
223	-0.03725552	-0.01590912	0.01057335
224	-0.03796835	-0.01532103	0.01066042
225	-0.03864748	-0.01471681	0.01072333
226	-0.03927765	-0.01409820	0.01076050
227	-0.03986418	-0.01348764	0.01075146
228	-0.04043636	-0.01287652	0.01070604
229	-0.04095920	-0.01233994	0.01061814
230	-0.04155058	-0.01174578	0.01048897
231	-0.04209223	-0.01112903	0.01031520
232	-0.04263721	-0.01049364	0.01010499
233	-0.04313644	-0.00979544	0.00989667
234	-0.04360569	-0.00908271	0.00965202
235	-0.04404134	-0.00835802	0.00939217
236	-0.04445268	-0.00763865	0.00910995
237	-0.04480893	-0.00689281	0.00881700
238	-0.04514884	-0.00614534	0.00850833
239	-0.04538253	-0.00524872	0.00810150
240	-0.04561149	-0.00435888	0.00765839
241	-0.04569922	-0.00350147	0.00720420
242	-0.04568637	-0.00259547	0.00667752
243	-0.04564395	-0.00174088	0.00616163

244	-0.04550164	-0.00089122	0.00562919
245	-0.04525052	-0.00000794	0.00502896
246	-0.04490514	0.00085943	0.00438836
247	-0.04444391	0.00166743	0.00376719
248	-0.04383529	0.00254630	0.00309380
249	-0.04311532	0.00332642	0.00247694
250	-0.04237714	0.00407140	0.00190874
251	-0.04157067	0.00482416	0.00132118
252	-0.04066996	0.00548609	0.00078981
253	-0.03967441	0.00614619	0.00026565
254	-0.03856772	0.00678540	-0.00026976
255	-0.03746026	0.00745031	-0.00080336
256	-0.03635985	0.00807653	-0.00136057
257	-0.03283597	-0.00755995	0.01129782
258	-0.03284175	-0.00625567	0.01120888
259	-0.03246985	-0.00495578	0.01119424
260	-0.03181461	-0.00368946	0.01124957
261	-0.03087023	-0.00250337	0.01139923
262	-0.02982475	-0.00148826	0.01165896
263	-0.02848960	-0.00069932	0.01201036
264	-0.02692161	-0.00014250	0.01244223
265	-0.02515645	0.00011575	0.01295102
266	-0.02314940	0.00005959	0.01361793
267	-0.02066859	-0.00024108	0.01429625
268	-0.01847602	-0.00061812	0.01478537
269	-0.01676892	-0.00103096	0.01510747
270	-0.01512343	-0.00168075	0.01535654
271	-0.01393933	-0.00254473	0.01550485

272	-0.01301775	-0.00356454	0.01551009
273	-0.01234659	-0.00463953	0.01537344
274	-0.01173939	-0.00606445	0.01503172
275	-0.01147459	-0.00760952	0.01446206
276	-0.01154836	-0.00934541	0.01387550
277	-0.01192904	-0.01100245	0.01338127
278	-0.01265938	-0.01244800	0.01292938
279	-0.01361598	-0.01368064	0.01258738
280	-0.01490202	-0.01471859	0.01226820
281	-0.01648917	-0.01560876	0.01199874
282	-0.01833678	-0.01630417	0.01182986
283	-0.02042147	-0.01685780	0.01170816
284	-0.02279611	-0.01719172	0.01182102
285	-0.02520757	-0.01720999	0.01208192
286	-0.02718980	-0.01703972	0.01208847
287	-0.02884691	-0.01646427	0.01212053
288	-0.02992599	-0.01577794	0.01214439
289	-0.03098763	-0.01476188	0.01206681
290	-0.03194663	-0.01338661	0.01190881
291	-0.03264389	-0.01164839	0.01169577
292	-0.03281080	-0.00951835	0.01146905
293	0.02138341	0.03090165	-0.02228122
294	0.02303554	0.03371662	-0.02299116
295	0.02546538	0.03656466	-0.02227769
296	0.02724977	0.03804234	-0.02077421
297	0.02822899	0.03845561	-0.01933985
298	0.02887672	0.03830046	-0.01779852
299	0.02930295	0.03772791	-0.01614329

300	0.02972122	0.03685285	-0.01437480
301	0.03026195	0.03644156	-0.01317737
302	0.03064050	0.03616237	-0.01229847
303	0.03166900	0.03778328	-0.01200486
304	0.03277505	0.03902930	-0.01135144
305	0.03358141	0.03962450	-0.01046901
306	0.03427569	0.03983353	-0.00945414
307	0.03492844	0.03977135	-0.00834763
308	0.03553146	0.03935160	-0.00702397
309	0.03598772	0.03822485	-0.00519957
310	0.03582295	0.03607500	-0.00331271
311	0.03510974	0.03368507	-0.00228859
312	0.03844813	-0.01305685	-0.02617517
313	0.04074304	-0.01421714	-0.02724783
314	0.04370962	-0.01543359	-0.02735507
315	0.04574523	-0.01609787	-0.02650201
316	0.04686400	-0.01629297	-0.02532057
317	0.04739622	-0.01623209	-0.02396186
318	0.04769242	-0.01599310	-0.02236595
319	0.04807907	-0.01580565	-0.02085427
320	0.04855887	-0.01561317	-0.01935649
321	0.04881799	-0.01555318	-0.01802526
322	0.05053817	-0.01618435	-0.01767859
323	0.05201483	-0.01657116	-0.01701956
324	0.05296970	-0.01668294	-0.01611194
325	0.05362784	-0.01663776	-0.01503785
326	0.05406904	-0.01645841	-0.01377919
327	0.05431533	-0.01612646	-0.01221949

328	0.05406043	-0.01546835	-0.01015611
329	0.05289639	-0.01438581	-0.00784527
330	0.05105817	-0.01319332	-0.00623933
331	0.01193084	-0.02396644	-0.00982877
332	0.01124557	-0.02609452	-0.00932141
333	0.01099019	-0.02822373	-0.00815299
334	0.01119507	-0.02972252	-0.00672630
335	0.01168717	-0.03054466	-0.00533698
336	0.01231956	-0.03082806	-0.00416218
337	0.01285148	-0.03086647	-0.00331106
338	0.01343589	-0.03094366	-0.00225727
339	0.01413583	-0.03110500	-0.00082685
340	0.01474670	-0.03130876	0.00053315
341	0.01501216	-0.03243591	0.00157267
342	0.01538615	-0.03328116	0.00260941
343	0.01588534	-0.03378410	0.00365566
344	0.01650757	-0.03392314	0.00471561
345	0.01724085	-0.03380220	0.00583910
346	0.01819828	-0.03334225	0.00712311
347	0.01955369	-0.03216158	0.00854230
348	0.02110104	-0.02998885	0.00955205
349	0.02228858	-0.02745162	0.00979012
350	0.02736178	0.01071418	-0.00049084
351	0.02926678	0.01309232	0.00092045
352	0.03096006	0.01470643	0.00296506
353	0.03218289	0.01542574	0.00487799
354	0.03309986	0.01571986	0.00651676
355	0.03375703	0.01574526	0.00787341

356	0.03422557	0.01541442	0.00926504
357	0.03444337	0.01441573	0.01078786
358	0.03421712	0.01257007	0.01223565
359	0.03347118	0.01025768	0.01304261
360	0.03626888	-0.00772611	-0.00001093
361	0.03884792	-0.00888330	0.00044536
362	0.04107107	-0.00971609	0.00139649
363	0.04264667	-0.01016488	0.00262707
364	0.04362654	-0.01031209	0.00389898
365	0.04415123	-0.01025041	0.00511918
366	0.04437265	-0.00999053	0.00643539
367	0.04416844	-0.00947365	0.00791930
368	0.04333403	-0.00861847	0.00959320
369	0.04180901	-0.00751754	0.01102375
370	0.02586576	-0.01326425	0.00650355
371	0.02611933	-0.01543266	0.00833004
372	0.02641186	-0.01710019	0.00993513
373	0.02687584	-0.01825597	0.01148034
374	0.02746722	-0.01877839	0.01283610
375	0.02810941	-0.01883866	0.01400607
376	0.02885181	-0.01849825	0.01511509
377	0.02980779	-0.01751306	0.01617493
378	0.03088795	-0.01574896	0.01695802
379	0.03182390	-0.01350047	0.01711653
380	-0.02756687	0.02035947	0.00384800
381	-0.02467375	0.01992597	0.00389049
382	-0.02105461	0.01855647	0.00316008
383	-0.01725925	0.01703863	0.00211942

384	-0.01328657	0.01681975	0.00164519
385	-0.01020744	0.01808088	0.00189688
386	-0.00834824	0.01981250	0.00222068
387	-0.00553428	0.02158343	0.00217849
388	-0.00227626	0.02285544	0.00156516
389	0.00071873	0.02386865	0.00026242
390	0.00264900	0.02477514	-0.00085465
391	0.00471207	0.02529769	-0.00208377
392	0.00686739	0.02569169	-0.00341858
393	0.00904027	0.02585233	-0.00478197
394	0.01112998	0.02580830	-0.00610574
395	0.01330980	0.02543220	-0.00763605
396	0.01558210	0.02473358	-0.00922040
397	0.01781344	0.02374549	-0.01079821
398	0.01998150	0.02264080	-0.01233339
399	0.02375162	0.01987225	-0.01432236
400	0.02700024	0.01686373	-0.01583134
401	0.02951603	0.01370361	-0.01695816
402	0.03165853	0.01019498	-0.01787214
403	0.03340547	0.00659405	-0.01835679
404	0.03457541	0.00277970	-0.01855304
405	0.03514936	-0.00109866	-0.01840256
406	0.03534727	-0.00512363	-0.01785351
407	0.03447914	-0.00936979	-0.01708699
408	0.03310446	-0.01199038	-0.01612184
409	0.03152551	-0.01423700	-0.01506491
410	0.02964564	-0.01603176	-0.01391467
411	0.02768977	-0.01733541	-0.01285649

412	0.02563635	-0.01822762	-0.01172908
413	0.02346179	-0.01870346	-0.01051278
414	0.02113698	-0.01887345	-0.00919805
415	0.01868482	-0.01850125	-0.00791758
416	0.01616353	-0.01763361	-0.00667322
417	0.01428685	-0.01657235	-0.00538290
418	0.01271713	-0.01519844	-0.00424170
419	0.01140805	-0.01368369	-0.00319222
420	0.01043308	-0.01196872	-0.00224876
421	0.00975934	-0.01021634	-0.00148171
422	0.00929954	-0.00829116	-0.00050612
423	0.00917964	-0.00623202	0.00035304
424	0.00934392	-0.00404202	0.00102469
425	0.00989240	-0.00166111	0.00147647
426	0.01096025	0.00035586	0.00200404
427	0.01222054	0.00206531	0.00229623
428	0.01356444	0.00332928	0.00225168
429	0.01516280	0.00443119	0.00224274
430	0.01686200	0.00523930	0.00217201
431	0.01868614	0.00573160	0.00201049
432	0.02069000	0.00592850	0.00169099
433	0.02301692	0.00575839	0.00121632
434	0.02513988	0.00519573	0.00074867
435	0.02685754	0.00445082	0.00096335
436	0.02886275	0.00293804	0.00110803
437	0.03016203	0.00137687	0.00123122
438	0.03094592	-0.00036959	0.00131133
439	0.03136429	-0.00231435	0.00149919

440	0.03147135	-0.00392483	0.00196694
441	0.03111536	-0.00552515	0.00230064
442	0.03021748	-0.00693645	0.00236689
443	0.02893944	-0.00828217	0.00283380
444	0.02839016	-0.00821587	0.00415361
445	0.02716203	-0.00803540	0.00530420
446	0.02547242	-0.00767695	0.00567793
447	0.02478955	-0.00738748	0.00602289
448	0.02405203	-0.00659152	0.00694877
449	0.02386545	-0.00577764	0.00776342
450	0.02405281	-0.00474654	0.00924590
451	0.02482960	-0.00351171	0.01221473
452	0.02590000	-0.00319789	0.01470447
453	-0.00218827	0.04138855	-0.01458078
454	-0.00183789	0.04132139	-0.01357428
455	-0.00123899	0.04106393	-0.01261206
456	0.00020246	0.04062225	-0.01181492
457	0.00214902	0.03989525	-0.01176794
458	0.00476742	0.03875691	-0.01262043
459	0.00798200	0.03728228	-0.01423555
460	0.01176139	0.03541406	-0.01666035
461	0.01631099	0.03321573	-0.01939427
462	0.02139512	0.03092354	-0.02228143
463	0.02603420	0.02679101	-0.02432804
464	0.03013644	0.02242182	-0.02577003
465	0.03341655	0.01784621	-0.02695767
466	0.03587800	0.01312113	-0.02774642
467	0.03783972	0.00819159	-0.02804405

468	0.03900548	0.00306108	-0.02809192
469	0.03951204	-0.00216797	-0.02779397
470	0.03935623	-0.00757965	-0.02711839
471	0.03845718	-0.01309236	-0.02617735
472	0.03628176	-0.01663618	-0.02468231
473	0.03379776	-0.01969264	-0.02312739
474	0.03115235	-0.02210501	-0.02148570
475	0.02824030	-0.02394459	-0.01980758
476	0.02524436	-0.02516715	-0.01806279
477	0.02210659	-0.02575915	-0.01609787
478	0.01873170	-0.02574315	-0.01408603
479	0.01530575	-0.02511654	-0.01204778
480	0.01191572	-0.02397590	-0.00982256
481	0.00931153	-0.02201944	-0.00774909
482	0.00710232	-0.01988213	-0.00585868
483	0.00524643	-0.01748019	-0.00424036
484	0.00384003	-0.01492786	-0.00284175
485	0.00289067	-0.01218561	-0.00155273
486	0.00241124	-0.00928502	-0.00049599
487	0.00241421	-0.00615683	0.00046821
488	0.00289411	-0.00290827	0.00129257
489	0.00398098	0.00031475	0.00210484
490	0.00602023	0.00353889	0.00265927
491	0.00821560	0.00621467	0.00300307
492	0.01049367	0.00838921	0.00308400
493	0.01288691	0.01011606	0.00284998
494	0.01546035	0.01135058	0.00243622
495	0.01826216	0.01205324	0.00182497

496	0.02125660	0.01221412	0.00101399
497	0.02437234	0.01173114	0.00021247
498	0.02755663	0.01065725	-0.00054112
499	0.02970458	0.00910809	-0.00108471
500	0.03164921	0.00739745	-0.00147791
501	0.03329500	0.00553180	-0.00172157
502	0.03460918	0.00354288	-0.00183917
503	0.03558503	0.00142971	-0.00175040
504	0.03621892	-0.00079295	-0.00154286
505	0.03654076	-0.00310860	-0.00117862
506	0.03655131	-0.00544134	-0.00063586
507	0.03627003	-0.00795069	0.00004578
508	0.03544952	-0.00952743	0.00073825
509	0.03452697	-0.01094889	0.00143220
510	0.03351389	-0.01211382	0.00214481
511	0.03240901	-0.01299040	0.00288622
512	0.03124770	-0.01356949	0.00363310
513	0.03001624	-0.01389889	0.00433532
514	0.02870285	-0.01394422	0.00506268
515	0.02729935	-0.01376945	0.00576559
516	0.02558108	-0.01321502	0.00661221
517	0.02451900	-0.01237661	0.00710184
518	0.02362855	-0.01159831	0.00765776
519	0.02284637	-0.01064540	0.00834677
520	0.02217421	-0.00949627	0.00911329
521	0.02181332	-0.00825343	0.00990242
522	0.02190509	-0.00695155	0.01070822
523	0.02263951	-0.00529899	0.01184546

524	0.02435573	-0.00368747	0.01343003
525	0.02590000	-0.00319789	0.01470447
526	-0.02939037	0.01606814	0.00664550
527	-0.02929998	0.01574916	0.00865161
528	-0.02898915	0.01550822	0.01061128
529	-0.02835585	0.01531377	0.01254164
530	-0.02754984	0.01522225	0.01430676
531	-0.02654927	0.01523578	0.01604624
532	-0.02538966	0.01538393	0.01771309
533	-0.02404888	0.01564738	0.01926950
534	-0.02256289	0.01606578	0.02075470
535	-0.02088364	0.01669807	0.02214739
536	-0.01892957	0.01775340	0.02343260
537	-0.01699078	0.01893894	0.02443446
538	-0.01509107	0.02030039	0.02521835
539	-0.01320872	0.02179364	0.02577437
540	-0.01134123	0.02340776	0.02608788
541	-0.00954435	0.02515365	0.02620688
542	-0.00779069	0.02700817	0.02605370
543	-0.00612559	0.02896094	0.02568740
544	-0.00446810	0.03092978	0.02508733
545	-0.00290492	0.03293923	0.02411435
546	-0.00144515	0.03485987	0.02298131
547	0.00009537	0.03664622	0.02165560
548	0.00167448	0.03837184	0.02020558
549	0.00325572	0.04001847	0.01863491
550	0.00488750	0.04151254	0.01693643
551	0.00662872	0.04287273	0.01507994

552	0.00840996	0.04410668	0.01314093
553	0.01025573	0.04500448	0.01098897
554	0.01254751	0.04578334	0.00835307
555	0.01500994	0.04619680	0.00579287
556	0.01758802	0.04630802	0.00334835
557	0.02022759	0.04604853	0.00100029
558	0.02298457	0.04552461	-0.00125421
559	0.02585660	0.04461244	-0.00340318
560	0.02873604	0.04335223	-0.00538308
561	0.03167700	0.04174031	-0.00722259
562	0.03461114	0.03983715	-0.00889052
563	0.04054703	0.03484018	-0.01210760
564	0.04532761	0.02929569	-0.01458122
565	0.04912039	0.02341358	-0.01636377
566	0.05203114	0.01735144	-0.01751530
567	0.05419011	0.01108692	-0.01811964
568	0.05557889	0.00456542	-0.01815855
569	0.05616861	-0.00226469	-0.01761986
570	0.05566084	-0.00935537	-0.01637125
571	0.05387141	-0.01655969	-0.01442045
572	0.05117408	-0.02194279	-0.01302857
573	0.04787037	-0.02636107	-0.01137563
574	0.04422514	-0.02989002	-0.00952475
575	0.04035012	-0.03262411	-0.00750284
576	0.03624044	-0.03465487	-0.00529378
577	0.03182727	-0.03589223	-0.00289986
578	0.02708840	-0.03629463	-0.00030085
579	0.02206029	-0.03567219	0.00245721

580	0.01686719	-0.03388850	0.00527955
581	0.01254664	-0.03127585	0.00764181
582	0.00899296	-0.02808589	0.00972083
583	0.00621032	-0.02456751	0.01155533
584	0.00407656	-0.02080420	0.01318579
585	0.00255731	-0.01685797	0.01471222
586	0.00175771	-0.01256297	0.01599772
587	0.00164137	-0.00799367	0.01714857
588	0.00231902	-0.00321071	0.01800297
589	0.00392243	0.00157257	0.01833128
590	0.00627232	0.00603728	0.01813954
591	0.00901106	0.00967246	0.01757863
592	0.01199972	0.01250738	0.01674130
593	0.01513888	0.01463218	0.01569729
594	0.01843101	0.01616328	0.01447169
595	0.02188840	0.01717052	0.01302656
596	0.02557882	0.01754577	0.01131185
597	0.02948414	0.01718711	0.00933563
598	0.03345280	0.01574437	0.00721919
599	0.03685074	0.01375048	0.00598011
600	0.03967143	0.01126356	0.00499843
601	0.04185658	0.00852626	0.00428893
602	0.04345183	0.00571028	0.00381478
603	0.04457421	0.00279886	0.00352807
604	0.04523958	-0.00025626	0.00344011
605	0.04539248	-0.00350104	0.00356564
606	0.04501691	-0.00687908	0.00390182
607	0.04391906	-0.01030208	0.00451827

608	0.04275894	-0.01277601	0.00527297
609	0.04132793	-0.01489338	0.00613841
610	0.03970782	-0.01662915	0.00709371
611	0.03794450	-0.01794949	0.00809116
612	0.03610519	-0.01887293	0.00911216
613	0.03418909	-0.01943228	0.01015137
614	0.03217551	-0.01966472	0.01121419
615	0.03003545	-0.01951919	0.01231328
616	0.02779840	-0.01883864	0.01344329
617	0.02566603	-0.01755671	0.01503114
618	0.02394095	-0.01577890	0.01627811
619	0.02272149	-0.01379297	0.01719513
620	0.02205836	-0.01189957	0.01776168
621	0.02175741	-0.00979351	0.01801572
622	0.02196844	-0.00767367	0.01800256
623	0.02285337	-0.00546051	0.01744792
624	0.02454340	-0.00364659	0.01606672
625	0.02589855	-0.00318834	0.01474129
626	-0.01714814	0.00766848	0.01280826
627	-0.01602847	0.00856348	0.01632405
628	-0.01466947	0.00993715	0.01966138
629	-0.01307452	0.01184707	0.02234661
630	-0.01123767	0.01402434	0.02427975
631	-0.00914233	0.01630777	0.02566048
632	-0.00680270	0.01874038	0.02649355
633	-0.00423310	0.02128547	0.02685480
634	-0.00149965	0.02401718	0.02666621
635	0.00132739	0.02698466	0.02584807

636	0.00305160	0.02904092	0.02503044
637	0.00487384	0.03091961	0.02394385
638	0.00674858	0.03265930	0.02260657
639	0.00866477	0.03424095	0.02095775
640	0.01066217	0.03565456	0.01904493
641	0.01284287	0.03689225	0.01698158
642	0.01506275	0.03792800	0.01465699
643	0.01752215	0.03880487	0.01216451
644	0.02014270	0.03944882	0.00954716
645	0.02340023	0.03943492	0.00647093
646	0.02673268	0.03871490	0.00364816
647	0.03008663	0.03726287	0.00105856
648	0.03344046	0.03507928	-0.00123823
649	0.03667868	0.03221603	-0.00324791
650	0.03981107	0.02872670	-0.00494051
651	0.04291168	0.02450239	-0.00629910
652	0.04590004	0.01959179	-0.00741564
653	0.04879830	0.01409664	-0.00822162
654	0.05068151	0.00689406	-0.00834764
655	0.05181140	0.00037476	-0.00803719
656	0.05213294	-0.00552906	-0.00746948
657	0.05163719	-0.01078857	-0.00668413
658	0.05030713	-0.01547305	-0.00574009
659	0.04818115	-0.01953440	-0.00452684
660	0.04528586	-0.02298292	-0.00301353
661	0.04168932	-0.02582057	-0.00111313
662	0.03732637	-0.02807688	0.00115063
663	0.03359518	-0.02911164	0.00316084

664	0.03005810	-0.02946220	0.00507341
665	0.02680777	-0.02917642	0.00701736
666	0.02373827	-0.02820005	0.00888963
667	0.02090544	-0.02658400	0.01067561
668	0.01827106	-0.02430519	0.01243513
669	0.01584971	-0.02129268	0.01412034
670	0.01362293	-0.01750124	0.01570763
671	0.01179273	-0.01300780	0.01724954
672	0.01200996	-0.00734099	0.01817775
673	0.01294675	-0.00252484	0.01895725
674	0.01446004	0.00143259	0.01935239
675	0.01641046	0.00458804	0.01925005
676	0.01876308	0.00701671	0.01871820
677	0.02156132	0.00881190	0.01777147
678	0.02493034	0.00997127	0.01650205
679	0.02890828	0.01047790	0.01487471
680	0.03358545	0.01029535	0.01299573
681	0.03577511	0.00890359	0.01212176
682	0.03769097	0.00733229	0.01139926
683	0.03930814	0.00559615	0.01087341
684	0.04055220	0.00370790	0.01047069
685	0.04146589	0.00168853	0.01028547
686	0.04202705	-0.00045995	0.01022936
687	0.04227211	-0.00272680	0.01037014
688	0.04219857	-0.00510634	0.01062953
689	0.04179406	-0.00762861	0.01104035
690	0.04095892	-0.00930572	0.01160926
691	0.04003147	-0.01077235	0.01222707

692	0.03900715	-0.01198729	0.01285521
693	0.03791750	-0.01292111	0.01353237
694	0.03675270	-0.01357024	0.01421624
695	0.03556127	-0.01394403	0.01492729
696	0.03431049	-0.01404469	0.01567399
697	0.03304742	-0.01389941	0.01641429
698	0.03156475	-0.01339451	0.01727044
699	0.03006722	-0.01192353	0.01818341
700	0.02871193	-0.01034120	0.01892260
701	0.02781598	-0.00881156	0.01936846
702	0.02733167	-0.00746988	0.01946722
703	0.02712914	-0.00643493	0.01931227
704	0.02696520	-0.00552820	0.01897578
705	0.02670683	-0.00450978	0.01832306
706	0.02630382	-0.00354606	0.01695839
707	0.02589855	-0.00318834	0.01474129
708	-0.01520552	0.00590155	-0.00466121
709	-0.01587966	0.00515075	-0.00634812
710	-0.01665369	0.00450525	-0.00807283
711	-0.01759561	0.00393161	-0.00991982
712	-0.01852073	0.00353691	-0.01164814
713	-0.01948182	0.00326484	-0.01321439
714	-0.02033413	0.00309327	-0.01452637
715	-0.02113927	0.00304111	-0.01628112
716	-0.02203928	0.00325219	-0.01803815
717	-0.02230605	0.00335715	-0.01886560

Procrustes sums of squares: 0.34826545124891567

Tangent sums of squares: 0.34382815329249755

Data matrices in this dataset:

- BE_All, raw data
- BE_All, centroid size
- BE_All, Procrustes coordinates

New Procrustes fit:

Dataset 'BE_All'

717 landmarks in 3 dimensions.

The dataset contains 32 observations, of which 32 are included for analyses.

Average shape:

Lmk.	Axis 1 (x)	Axis 2 (y)	Axis 3 (z)
1	-0.02961287	0.00007956	-0.01542495
2	-0.02688184	-0.01715574	-0.00513417
3	-0.02726320	-0.01727447	-0.00565098
4	-0.02761520	-0.01723263	-0.00634578
5	-0.02787930	-0.01703551	-0.00709598
6	-0.02811674	-0.01673799	-0.00785903
7	-0.02833863	-0.01635572	-0.00862419
8	-0.02857050	-0.01584521	-0.00940742
9	-0.02877505	-0.01525608	-0.01017585
10	-0.02898250	-0.01461619	-0.01093455

11	-0.02916764	-0.01388747	-0.01174577
12	-0.02929433	-0.01310197	-0.01247140
13	-0.02939805	-0.01228808	-0.01310077
14	-0.02945195	-0.01143297	-0.01367248
15	-0.02946972	-0.01053827	-0.01414700
16	-0.02941046	-0.00960975	-0.01453705
17	-0.02934366	-0.00863582	-0.01485643
18	-0.02920144	-0.00765484	-0.01506528
19	-0.02898743	-0.00668120	-0.01513642
20	-0.02874612	-0.00566804	-0.01516863
21	-0.02829323	-0.00431345	-0.01505532
22	-0.02785277	-0.00313155	-0.01477510
23	-0.02736512	-0.00203913	-0.01433250
24	-0.02672310	-0.00112912	-0.01362951
25	-0.02600015	-0.00051806	-0.01272510
26	-0.02517343	0.00003406	-0.01158476
27	-0.02427858	0.00049156	-0.01018811
28	-0.02325083	0.00074414	-0.00855101
29	-0.02212164	0.00067072	-0.00678330
30	-0.02154042	0.00024057	-0.00544540
31	-0.02113444	-0.00070394	-0.00430513
32	-0.02098579	-0.00186264	-0.00356623
33	-0.02096887	-0.00318391	-0.00294485
34	-0.02108271	-0.00469324	-0.00238386
35	-0.02133778	-0.00633864	-0.00188889
36	-0.02173256	-0.00809185	-0.00146348
37	-0.02227817	-0.00990887	-0.00112538
38	-0.02289490	-0.01153259	-0.00134231

39	-0.02319160	-0.01211671	-0.00176420
40	-0.02360232	-0.01272447	-0.00232029
41	-0.02404658	-0.01335561	-0.00288818
42	-0.02447239	-0.01400222	-0.00337117
43	-0.02491398	-0.01467447	-0.00375340
44	-0.02541278	-0.01537931	-0.00402765
45	-0.02594728	-0.01607269	-0.00425744
46	-0.02649058	-0.01670432	-0.00461637
47	-0.02739189	-0.02131576	-0.00331920
48	-0.02762199	-0.02120635	-0.00376542
49	-0.02784330	-0.02105954	-0.00424012
50	-0.02808388	-0.02085317	-0.00483128
51	-0.02832169	-0.02058738	-0.00547844
52	-0.02856412	-0.02024060	-0.00616852
53	-0.02881270	-0.01985963	-0.00690788
54	-0.02904427	-0.01943267	-0.00766381
55	-0.02929223	-0.01896058	-0.00842875
56	-0.02953362	-0.01847111	-0.00931756
57	-0.02974282	-0.01789935	-0.01013716
58	-0.02994083	-0.01730244	-0.01090713
59	-0.03013274	-0.01668564	-0.01164350
60	-0.03030021	-0.01605320	-0.01235490
61	-0.03041069	-0.01536534	-0.01295610
62	-0.03053785	-0.01471604	-0.01360805
63	-0.03063087	-0.01403706	-0.01416304
64	-0.03070848	-0.01331736	-0.01468989
65	-0.03072993	-0.01260763	-0.01519698
66	-0.03071036	-0.01189696	-0.01561249

67	-0.03067375	-0.01116113	-0.01601025
68	-0.03060896	-0.01042235	-0.01634418
69	-0.03050566	-0.00967427	-0.01663010
70	-0.03036984	-0.00892578	-0.01685881
71	-0.03022189	-0.00816635	-0.01700094
72	-0.03003824	-0.00733692	-0.01708447
73	-0.02987233	-0.00653742	-0.01718934
74	-0.02964881	-0.00575210	-0.01717885
75	-0.02944187	-0.00505604	-0.01711339
76	-0.02923186	-0.00436047	-0.01707613
77	-0.02897648	-0.00361326	-0.01697326
78	-0.02868680	-0.00286445	-0.01678003
79	-0.02836075	-0.00206065	-0.01653012
80	-0.02800098	-0.00124777	-0.01627846
81	-0.02758535	-0.00039414	-0.01604932
82	-0.02711088	0.00052260	-0.01581910
83	-0.02660062	0.00144529	-0.01551448
84	-0.03370399	0.01055076	-0.00519351
85	-0.03423478	0.01046795	-0.00527142
86	-0.03479850	0.01033496	-0.00548411
87	-0.03526775	0.01017186	-0.00581784
88	-0.03569485	0.00998337	-0.00623081
89	-0.03603485	0.00977635	-0.00667510
90	-0.03632333	0.00956153	-0.00715773
91	-0.03652723	0.00935738	-0.00764406
92	-0.03673201	0.00912097	-0.00817761
93	-0.03687399	0.00884952	-0.00873755
94	-0.03698385	0.00851927	-0.00931452

95	-0.03700982	0.00818631	-0.00989772
96	-0.03694444	0.00784582	-0.01048961
97	-0.03682216	0.00747347	-0.01106694
98	-0.03663121	0.00707753	-0.01164251
99	-0.03637392	0.00667805	-0.01220265
100	-0.03602070	0.00625090	-0.01273214
101	-0.03558115	0.00582786	-0.01320688
102	-0.03506976	0.00546295	-0.01363739
103	-0.03439488	0.00501406	-0.01398466
104	-0.03360900	0.00460070	-0.01419126
105	-0.03284100	0.00424215	-0.01431475
106	-0.03206296	0.00395038	-0.01435639
107	-0.03126935	0.00367907	-0.01429864
108	-0.03056336	0.00348121	-0.01414620
109	-0.02975833	0.00328496	-0.01391283
110	-0.02897592	0.00322402	-0.01356743
111	-0.02820134	0.00326181	-0.01310731
112	-0.02679511	0.00347265	-0.01166095
113	-0.02575236	0.00396272	-0.01026455
114	-0.02499029	0.00441572	-0.00903402
115	-0.02456950	0.00504613	-0.00790649
116	-0.02442030	0.00559269	-0.00704476
117	-0.02445594	0.00609845	-0.00620998
118	-0.02481072	0.00676610	-0.00548775
119	-0.02559303	0.00763196	-0.00500053
120	-0.02659039	0.00845338	-0.00491318
121	-0.02720599	0.00901420	-0.00513043
122	-0.02793478	0.00949700	-0.00543984

123	-0.02870989	0.00988815	-0.00571470
124	-0.02952246	0.01021178	-0.00585584
125	-0.03036780	0.01045651	-0.00586040
126	-0.03121398	0.01061490	-0.00569915
127	-0.03207727	0.01068921	-0.00548225
128	-0.03281907	0.01067149	-0.00530317
129	-0.03522247	0.01348301	-0.00274899
130	-0.03566538	0.01321856	-0.00296691
131	-0.03606731	0.01296748	-0.00319753
132	-0.03652008	0.01262572	-0.00353562
133	-0.03698027	0.01231427	-0.00390405
134	-0.03740770	0.01207504	-0.00426865
135	-0.03778600	0.01181143	-0.00464837
136	-0.03812073	0.01151785	-0.00508583
137	-0.03843249	0.01124520	-0.00554624
138	-0.03868341	0.01098971	-0.00606781
139	-0.03896539	0.01070592	-0.00679466
140	-0.03920901	0.01037214	-0.00749449
141	-0.03936874	0.01000108	-0.00818368
142	-0.03950443	0.00966471	-0.00883804
143	-0.03952244	0.00928987	-0.00946444
144	-0.03949690	0.00889262	-0.01010061
145	-0.03949746	0.00849871	-0.01075889
146	-0.03943780	0.00813839	-0.01136594
147	-0.03928790	0.00773950	-0.01197694
148	-0.03904430	0.00734980	-0.01252591
149	-0.03877991	0.00697174	-0.01311562
150	-0.03843138	0.00658604	-0.01362178

151	-0.03802534	0.00616284	-0.01413524
152	-0.03758061	0.00576744	-0.01461147
153	-0.03707709	0.00538446	-0.01503228
154	-0.03651047	0.00500053	-0.01544309
155	-0.03590778	0.00464218	-0.01577309
156	-0.03531226	0.00427466	-0.01606339
157	-0.03445526	0.00388701	-0.01631146
158	-0.03349783	0.00353956	-0.01646398
159	-0.03256729	0.00322715	-0.01654123
160	-0.03161213	0.00290525	-0.01649008
161	-0.03067548	0.00256920	-0.01636964
162	-0.02976746	0.00226678	-0.01623153
163	-0.02875676	0.00196918	-0.01601596
164	-0.02768511	0.00169958	-0.01578716
165	-0.02660062	0.00144529	-0.01551448
166	-0.03504173	-0.01271315	0.00819804
167	-0.03568491	-0.01268801	0.00851005
168	-0.03640602	-0.01250622	0.00872035
169	-0.03710697	-0.01223304	0.00886615
170	-0.03780282	-0.01184930	0.00890302
171	-0.03850091	-0.01137873	0.00889719
172	-0.03919870	-0.01081092	0.00885082
173	-0.03986695	-0.01014890	0.00873099
174	-0.04053998	-0.00940796	0.00855712
175	-0.04119649	-0.00854854	0.00831410
176	-0.04165337	-0.00788913	0.00808366
177	-0.04206452	-0.00720309	0.00783678
178	-0.04240404	-0.00650693	0.00756766

179	-0.04264916	-0.00581436	0.00724597
180	-0.04284504	-0.00514014	0.00693075
181	-0.04299798	-0.00443790	0.00659952
182	-0.04307620	-0.00372134	0.00622382
183	-0.04308110	-0.00299547	0.00581510
184	-0.04301728	-0.00223126	0.00535587
185	-0.04287295	-0.00156433	0.00491292
186	-0.04264401	-0.00086461	0.00446056
187	-0.04234234	-0.00020192	0.00401337
188	-0.04195557	0.00052564	0.00354093
189	-0.04151779	0.00113203	0.00306240
190	-0.04105076	0.00167816	0.00262708
191	-0.04050230	0.00222521	0.00216933
192	-0.03986990	0.00280471	0.00172957
193	-0.03923045	0.00334995	0.00139757
194	-0.03818764	0.00422462	0.00088592
195	-0.03705966	0.00486549	0.00043662
196	-0.03583831	0.00534181	0.00008386
197	-0.03457400	0.00566068	-0.00016251
198	-0.03322842	0.00577310	-0.00027826
199	-0.03182264	0.00564142	-0.00029500
200	-0.03036488	0.00539864	-0.00023035
201	-0.02891513	0.00495813	-0.00012057
202	-0.02736309	0.00427893	0.00004555
203	-0.02598863	0.00302616	0.00020501
204	-0.02512585	0.00148168	0.00045965
205	-0.02463905	-0.00006928	0.00069813
206	-0.02440279	-0.00166216	0.00105470

207	-0.02437186	-0.00326696	0.00156217
208	-0.02471362	-0.00492516	0.00212733
209	-0.02548901	-0.00657576	0.00269785
210	-0.02664011	-0.00811174	0.00323109
211	-0.02816935	-0.00946709	0.00369457
212	-0.02909076	-0.00998299	0.00400988
213	-0.03005181	-0.01041578	0.00438579
214	-0.03098065	-0.01083647	0.00483391
215	-0.03183311	-0.01128245	0.00537222
216	-0.03253553	-0.01170361	0.00593937
217	-0.03320076	-0.01209291	0.00655490
218	-0.03377822	-0.01243506	0.00717057
219	-0.03437975	-0.01262330	0.00771527
220	-0.03481423	-0.01748033	0.01003175
221	-0.03567265	-0.01700530	0.01026749
222	-0.03648573	-0.01646786	0.01044655
223	-0.03725552	-0.01590912	0.01057335
224	-0.03796835	-0.01532103	0.01066042
225	-0.03864748	-0.01471681	0.01072333
226	-0.03927765	-0.01409820	0.01076050
227	-0.03986418	-0.01348764	0.01075146
228	-0.04043636	-0.01287652	0.01070604
229	-0.04095920	-0.01233994	0.01061814
230	-0.04155058	-0.01174578	0.01048897
231	-0.04209223	-0.01112903	0.01031520
232	-0.04263721	-0.01049364	0.01010499
233	-0.04313644	-0.00979544	0.00989667
234	-0.04360569	-0.00908271	0.00965202

235	-0.04404134	-0.00835802	0.00939217
236	-0.04445268	-0.00763865	0.00910995
237	-0.04480893	-0.00689281	0.00881700
238	-0.04514884	-0.00614534	0.00850833
239	-0.04538253	-0.00524872	0.00810150
240	-0.04561149	-0.00435888	0.00765839
241	-0.04569922	-0.00350147	0.00720420
242	-0.04568637	-0.00259547	0.00667752
243	-0.04564395	-0.00174088	0.00616163
244	-0.04550164	-0.00089122	0.00562919
245	-0.04525052	-0.00000794	0.00502896
246	-0.04490514	0.00085943	0.00438836
247	-0.04444391	0.00166743	0.00376719
248	-0.04383529	0.00254630	0.00309380
249	-0.04311532	0.00332642	0.00247694
250	-0.04237714	0.00407140	0.00190874
251	-0.04157067	0.00482416	0.00132118
252	-0.04066996	0.00548609	0.00078981
253	-0.03967441	0.00614619	0.00026565
254	-0.03856772	0.00678540	-0.00026976
255	-0.03746026	0.00745031	-0.00080336
256	-0.03635985	0.00807653	-0.00136057
257	-0.03283597	-0.00755995	0.01129782
258	-0.03284175	-0.00625567	0.01120888
259	-0.03246985	-0.00495578	0.01119424
260	-0.03181461	-0.00368946	0.01124957
261	-0.03087023	-0.00250337	0.01139923
262	-0.02982475	-0.00148826	0.01165896

263	-0.02848960	-0.00069932	0.01201036
264	-0.02692161	-0.00014250	0.01244223
265	-0.02515645	0.00011575	0.01295102
266	-0.02314940	0.00005959	0.01361793
267	-0.02066859	-0.00024108	0.01429625
268	-0.01847602	-0.00061812	0.01478537
269	-0.01676892	-0.00103096	0.01510747
270	-0.01512343	-0.00168075	0.01535654
271	-0.01393933	-0.00254473	0.01550485
272	-0.01301775	-0.00356454	0.01551009
273	-0.01234659	-0.00463953	0.01537344
274	-0.01173939	-0.00606445	0.01503172
275	-0.01147459	-0.00760952	0.01446206
276	-0.01154836	-0.00934541	0.01387550
277	-0.01192904	-0.01100245	0.01338127
278	-0.01265938	-0.01244800	0.01292938
279	-0.01361598	-0.01368064	0.01258738
280	-0.01490202	-0.01471859	0.01226820
281	-0.01648917	-0.01560876	0.01199874
282	-0.01833678	-0.01630417	0.01182986
283	-0.02042147	-0.01685780	0.01170816
284	-0.02279611	-0.01719172	0.01182102
285	-0.02520757	-0.01720999	0.01208192
286	-0.02718980	-0.01703972	0.01208847
287	-0.02884691	-0.01646427	0.01212053
288	-0.02992599	-0.01577794	0.01214439
289	-0.03098763	-0.01476188	0.01206681
290	-0.03194663	-0.01338661	0.01190881

291	-0.03264389	-0.01164839	0.01169577
292	-0.03281080	-0.00951835	0.01146905
293	0.02138341	0.03090165	-0.02228122
294	0.02303554	0.03371662	-0.02299116
295	0.02546538	0.03656466	-0.02227769
296	0.02724977	0.03804234	-0.02077421
297	0.02822899	0.03845561	-0.01933985
298	0.02887672	0.03830046	-0.01779852
299	0.02930295	0.03772791	-0.01614329
300	0.02972122	0.03685285	-0.01437480
301	0.03026195	0.03644156	-0.01317737
302	0.03064050	0.03616237	-0.01229847
303	0.03166900	0.03778328	-0.01200486
304	0.03277505	0.03902930	-0.01135144
305	0.03358141	0.03962450	-0.01046901
306	0.03427569	0.03983353	-0.00945414
307	0.03492844	0.03977135	-0.00834763
308	0.03553146	0.03935160	-0.00702397
309	0.03598772	0.03822485	-0.00519957
310	0.03582295	0.03607500	-0.00331271
311	0.03510974	0.03368507	-0.00228859
312	0.03844813	-0.01305685	-0.02617517
313	0.04074304	-0.01421714	-0.02724783
314	0.04370962	-0.01543359	-0.02735507
315	0.04574523	-0.01609787	-0.02650201
316	0.04686400	-0.01629297	-0.02532057
317	0.04739622	-0.01623209	-0.02396186
318	0.04769242	-0.01599310	-0.02236595

319	0.04807907	-0.01580565	-0.02085427
320	0.04855887	-0.01561317	-0.01935649
321	0.04881799	-0.01555318	-0.01802526
322	0.05053817	-0.01618435	-0.01767859
323	0.05201483	-0.01657116	-0.01701956
324	0.05296970	-0.01668294	-0.01611194
325	0.05362784	-0.01663776	-0.01503785
326	0.05406904	-0.01645841	-0.01377919
327	0.05431533	-0.01612646	-0.01221949
328	0.05406043	-0.01546835	-0.01015611
329	0.05289639	-0.01438581	-0.00784527
330	0.05105817	-0.01319332	-0.00623933
331	0.01193084	-0.02396644	-0.00982877
332	0.01124557	-0.02609452	-0.00932141
333	0.01099019	-0.02822373	-0.00815299
334	0.01119507	-0.02972252	-0.00672630
335	0.01168717	-0.03054466	-0.00533698
336	0.01231956	-0.03082806	-0.00416218
337	0.01285148	-0.03086647	-0.00331106
338	0.01343589	-0.03094366	-0.00225727
339	0.01413583	-0.03110500	-0.00082685
340	0.01474670	-0.03130876	0.00053315
341	0.01501216	-0.03243591	0.00157267
342	0.01538615	-0.03328116	0.00260941
343	0.01588534	-0.03378410	0.00365566
344	0.01650757	-0.03392314	0.00471561
345	0.01724085	-0.03380220	0.00583910
346	0.01819828	-0.03334225	0.00712311

347	0.01955369	-0.03216158	0.00854230
348	0.02110104	-0.02998885	0.00955205
349	0.02228858	-0.02745162	0.00979012
350	0.02736178	0.01071418	-0.00049084
351	0.02926678	0.01309232	0.00092045
352	0.03096006	0.01470643	0.00296506
353	0.03218289	0.01542574	0.00487799
354	0.03309986	0.01571986	0.00651676
355	0.03375703	0.01574526	0.00787341
356	0.03422557	0.01541442	0.00926504
357	0.03444337	0.01441573	0.01078786
358	0.03421712	0.01257007	0.01223565
359	0.03347118	0.01025768	0.01304261
360	0.03626888	-0.00772611	-0.00001093
361	0.03884792	-0.00888330	0.00044536
362	0.04107107	-0.00971609	0.00139649
363	0.04264667	-0.01016488	0.00262707
364	0.04362654	-0.01031209	0.00389898
365	0.04415123	-0.01025041	0.00511918
366	0.04437265	-0.00999053	0.00643539
367	0.04416844	-0.00947365	0.00791930
368	0.04333403	-0.00861847	0.00959320
369	0.04180901	-0.00751754	0.01102375
370	0.02586576	-0.01326425	0.00650355
371	0.02611933	-0.01543266	0.00833004
372	0.02641186	-0.01710019	0.00993513
373	0.02687584	-0.01825597	0.01148034
374	0.02746722	-0.01877839	0.01283610

375	0.02810941	-0.01883866	0.01400607
376	0.02885181	-0.01849825	0.01511509
377	0.02980779	-0.01751306	0.01617493
378	0.03088795	-0.01574896	0.01695802
379	0.03182390	-0.01350047	0.01711653
380	-0.02756687	0.02035947	0.00384800
381	-0.02467375	0.01992597	0.00389049
382	-0.02105461	0.01855647	0.00316008
383	-0.01725925	0.01703863	0.00211942
384	-0.01328657	0.01681975	0.00164519
385	-0.01020744	0.01808088	0.00189688
386	-0.00834824	0.01981250	0.00222068
387	-0.00553428	0.02158343	0.00217849
388	-0.00227626	0.02285544	0.00156516
389	0.00071873	0.02386865	0.00026242
390	0.00264900	0.02477514	-0.00085465
391	0.00471207	0.02529769	-0.00208377
392	0.00686739	0.02569169	-0.00341858
393	0.00904027	0.02585233	-0.00478197
394	0.01112998	0.02580830	-0.00610574
395	0.01330980	0.02543220	-0.00763605
396	0.01558210	0.02473358	-0.00922040
397	0.01781344	0.02374549	-0.01079821
398	0.01998150	0.02264080	-0.01233339
399	0.02375162	0.01987225	-0.01432236
400	0.02700024	0.01686373	-0.01583134
401	0.02951603	0.01370361	-0.01695816
402	0.03165853	0.01019498	-0.01787214

403	0.03340547	0.00659405	-0.01835679
404	0.03457541	0.00277970	-0.01855304
405	0.03514936	-0.00109866	-0.01840256
406	0.03534727	-0.00512363	-0.01785351
407	0.03447914	-0.00936979	-0.01708699
408	0.03310446	-0.01199038	-0.01612184
409	0.03152551	-0.01423700	-0.01506491
410	0.02964564	-0.01603176	-0.01391467
411	0.02768977	-0.01733541	-0.01285649
412	0.02563635	-0.01822762	-0.01172908
413	0.02346179	-0.01870346	-0.01051278
414	0.02113698	-0.01887345	-0.00919805
415	0.01868482	-0.01850125	-0.00791758
416	0.01616353	-0.01763361	-0.00667322
417	0.01428685	-0.01657235	-0.00538290
418	0.01271713	-0.01519844	-0.00424170
419	0.01140805	-0.01368369	-0.00319222
420	0.01043308	-0.01196872	-0.00224876
421	0.00975934	-0.01021634	-0.00148171
422	0.00929954	-0.00829116	-0.00050612
423	0.00917964	-0.00623202	0.00035304
424	0.00934392	-0.00404202	0.00102469
425	0.00989240	-0.00166111	0.00147647
426	0.01096025	0.00035586	0.00200404
427	0.01222054	0.00206531	0.00229623
428	0.01356444	0.00332928	0.00225168
429	0.01516280	0.00443119	0.00224274
430	0.01686200	0.00523930	0.00217201

431	0.01868614	0.00573160	0.00201049
432	0.02069000	0.00592850	0.00169099
433	0.02301692	0.00575839	0.00121632
434	0.02513988	0.00519573	0.00074867
435	0.02685754	0.00445082	0.00096335
436	0.02886275	0.00293804	0.00110803
437	0.03016203	0.00137687	0.00123122
438	0.03094592	-0.00036959	0.00131133
439	0.03136429	-0.00231435	0.00149919
440	0.03147135	-0.00392483	0.00196694
441	0.03111536	-0.00552515	0.00230064
442	0.03021748	-0.00693645	0.00236689
443	0.02893944	-0.00828217	0.00283380
444	0.02839016	-0.00821587	0.00415361
445	0.02716203	-0.00803540	0.00530420
446	0.02547242	-0.00767695	0.00567793
447	0.02478955	-0.00738748	0.00602289
448	0.02405203	-0.00659152	0.00694877
449	0.02386545	-0.00577764	0.00776342
450	0.02405281	-0.00474654	0.00924590
451	0.02482960	-0.00351171	0.01221473
452	0.02590000	-0.00319789	0.01470447
453	-0.00218827	0.04138855	-0.01458078
454	-0.00183789	0.04132139	-0.01357428
455	-0.00123899	0.04106393	-0.01261206
456	0.00020246	0.04062225	-0.01181492
457	0.00214902	0.03989525	-0.01176794
458	0.00476742	0.03875691	-0.01262043

459	0.00798200	0.03728228	-0.01423555
460	0.01176139	0.03541406	-0.01666035
461	0.01631099	0.03321573	-0.01939427
462	0.02139512	0.03092354	-0.02228143
463	0.02603420	0.02679101	-0.02432804
464	0.03013644	0.02242182	-0.02577003
465	0.03341655	0.01784621	-0.02695767
466	0.03587800	0.01312113	-0.02774642
467	0.03783972	0.00819159	-0.02804405
468	0.03900548	0.00306108	-0.02809192
469	0.03951204	-0.00216797	-0.02779397
470	0.03935623	-0.00757965	-0.02711839
471	0.03845718	-0.01309236	-0.02617735
472	0.03628176	-0.01663618	-0.02468231
473	0.03379776	-0.01969264	-0.02312739
474	0.03115235	-0.02210501	-0.02148570
475	0.02824030	-0.02394459	-0.01980758
476	0.02524436	-0.02516715	-0.01806279
477	0.02210659	-0.02575915	-0.01609787
478	0.01873170	-0.02574315	-0.01408603
479	0.01530575	-0.02511654	-0.01204778
480	0.01191572	-0.02397590	-0.00982256
481	0.00931153	-0.02201944	-0.00774909
482	0.00710232	-0.01988213	-0.00585868
483	0.00524643	-0.01748019	-0.00424036
484	0.00384003	-0.01492786	-0.00284175
485	0.00289067	-0.01218561	-0.00155273
486	0.00241124	-0.00928502	-0.00049599

487	0.00241421	-0.00615683	0.00046821
488	0.00289411	-0.00290827	0.00129257
489	0.00398098	0.00031475	0.00210484
490	0.00602023	0.00353889	0.00265927
491	0.00821560	0.00621467	0.00300307
492	0.01049367	0.00838921	0.00308400
493	0.01288691	0.01011606	0.00284998
494	0.01546035	0.01135058	0.00243622
495	0.01826216	0.01205324	0.00182497
496	0.02125660	0.01221412	0.00101399
497	0.02437234	0.01173114	0.00021247
498	0.02755663	0.01065725	-0.00054112
499	0.02970458	0.00910809	-0.00108471
500	0.03164921	0.00739745	-0.00147791
501	0.03329500	0.00553180	-0.00172157
502	0.03460918	0.00354288	-0.00183917
503	0.03558503	0.00142971	-0.00175040
504	0.03621892	-0.00079295	-0.00154286
505	0.03654076	-0.00310860	-0.00117862
506	0.03655131	-0.00544134	-0.00063586
507	0.03627003	-0.00795069	0.00004578
508	0.03544952	-0.00952743	0.00073825
509	0.03452697	-0.01094889	0.00143220
510	0.03351389	-0.01211382	0.00214481
511	0.03240901	-0.01299040	0.00288622
512	0.03124770	-0.01356949	0.00363310
513	0.03001624	-0.01389889	0.00433532
514	0.02870285	-0.01394422	0.00506268

515	0.02729935	-0.01376945	0.00576559
516	0.02558108	-0.01321502	0.00661221
517	0.02451900	-0.01237661	0.00710184
518	0.02362855	-0.01159831	0.00765776
519	0.02284637	-0.01064540	0.00834677
520	0.02217421	-0.00949627	0.00911329
521	0.02181332	-0.00825343	0.00990242
522	0.02190509	-0.00695155	0.01070822
523	0.02263951	-0.00529899	0.01184546
524	0.02435573	-0.00368747	0.01343003
525	0.02590000	-0.00319789	0.01470447
526	-0.02939037	0.01606814	0.00664550
527	-0.02929998	0.01574916	0.00865161
528	-0.02898915	0.01550822	0.01061128
529	-0.02835585	0.01531377	0.01254164
530	-0.02754984	0.01522225	0.01430676
531	-0.02654927	0.01523578	0.01604624
532	-0.02538966	0.01538393	0.01771309
533	-0.02404888	0.01564738	0.01926950
534	-0.02256289	0.01606578	0.02075470
535	-0.02088364	0.01669807	0.02214739
536	-0.01892957	0.01775340	0.02343260
537	-0.01699078	0.01893894	0.02443446
538	-0.01509107	0.02030039	0.02521835
539	-0.01320872	0.02179364	0.02577437
540	-0.01134123	0.02340776	0.02608788
541	-0.00954435	0.02515365	0.02620688
542	-0.00779069	0.02700817	0.02605370

543	-0.00612559	0.02896094	0.02568740
544	-0.00446810	0.03092978	0.02508733
545	-0.00290492	0.03293923	0.02411435
546	-0.00144515	0.03485987	0.02298131
547	0.00009537	0.03664622	0.02165560
548	0.00167448	0.03837184	0.02020558
549	0.00325572	0.04001847	0.01863491
550	0.00488750	0.04151254	0.01693643
551	0.00662872	0.04287273	0.01507994
552	0.00840996	0.04410668	0.01314093
553	0.01025573	0.04500448	0.01098897
554	0.01254751	0.04578334	0.00835307
555	0.01500994	0.04619680	0.00579287
556	0.01758802	0.04630802	0.00334835
557	0.02022759	0.04604853	0.00100029
558	0.02298457	0.04552461	-0.00125421
559	0.02585660	0.04461244	-0.00340318
560	0.02873604	0.04335223	-0.00538308
561	0.03167700	0.04174031	-0.00722259
562	0.03461114	0.03983715	-0.00889052
563	0.04054703	0.03484018	-0.01210760
564	0.04532761	0.02929569	-0.01458122
565	0.04912039	0.02341358	-0.01636377
566	0.05203114	0.01735144	-0.01751530
567	0.05419011	0.01108692	-0.01811964
568	0.05557889	0.00456542	-0.01815855
569	0.05616861	-0.00226469	-0.01761986
570	0.05566084	-0.00935537	-0.01637125

571	0.05387141	-0.01655969	-0.01442045
572	0.05117408	-0.02194279	-0.01302857
573	0.04787037	-0.02636107	-0.01137563
574	0.04422514	-0.02989002	-0.00952475
575	0.04035012	-0.03262411	-0.00750284
576	0.03624044	-0.03465487	-0.00529378
577	0.03182727	-0.03589223	-0.00289986
578	0.02708840	-0.03629463	-0.00030085
579	0.02206029	-0.03567219	0.00245721
580	0.01686719	-0.03388850	0.00527955
581	0.01254664	-0.03127585	0.00764181
582	0.00899296	-0.02808589	0.00972083
583	0.00621032	-0.02456751	0.01155533
584	0.00407656	-0.02080420	0.01318579
585	0.00255731	-0.01685797	0.01471222
586	0.00175771	-0.01256297	0.01599772
587	0.00164137	-0.00799367	0.01714857
588	0.00231902	-0.00321071	0.01800297
589	0.00392243	0.00157257	0.01833128
590	0.00627232	0.00603728	0.01813954
591	0.00901106	0.00967246	0.01757863
592	0.01199972	0.01250738	0.01674130
593	0.01513888	0.01463218	0.01569729
594	0.01843101	0.01616328	0.01447169
595	0.02188840	0.01717052	0.01302656
596	0.02557882	0.01754577	0.01131185
597	0.02948414	0.01718711	0.00933563
598	0.03345280	0.01574437	0.00721919

599	0.03685074	0.01375048	0.00598011
600	0.03967143	0.01126356	0.00499843
601	0.04185658	0.00852626	0.00428893
602	0.04345183	0.00571028	0.00381478
603	0.04457421	0.00279886	0.00352807
604	0.04523958	-0.00025626	0.00344011
605	0.04539248	-0.00350104	0.00356564
606	0.04501691	-0.00687908	0.00390182
607	0.04391906	-0.01030208	0.00451827
608	0.04275894	-0.01277601	0.00527297
609	0.04132793	-0.01489338	0.00613841
610	0.03970782	-0.01662915	0.00709371
611	0.03794450	-0.01794949	0.00809116
612	0.03610519	-0.01887293	0.00911216
613	0.03418909	-0.01943228	0.01015137
614	0.03217551	-0.01966472	0.01121419
615	0.03003545	-0.01951919	0.01231328
616	0.02779840	-0.01883864	0.01344329
617	0.02566603	-0.01755671	0.01503114
618	0.02394095	-0.01577890	0.01627811
619	0.02272149	-0.01379297	0.01719513
620	0.02205836	-0.01189957	0.01776168
621	0.02175741	-0.00979351	0.01801572
622	0.02196844	-0.00767367	0.01800256
623	0.02285337	-0.00546051	0.01744792
624	0.02454340	-0.00364659	0.01606672
625	0.02589855	-0.00318834	0.01474129
626	-0.01714814	0.00766848	0.01280826

627	-0.01602847	0.00856348	0.01632405
628	-0.01466947	0.00993715	0.01966138
629	-0.01307452	0.01184707	0.02234661
630	-0.01123767	0.01402434	0.02427975
631	-0.00914233	0.01630777	0.02566048
632	-0.00680270	0.01874038	0.02649355
633	-0.00423310	0.02128547	0.02685480
634	-0.00149965	0.02401718	0.02666621
635	0.00132739	0.02698466	0.02584807
636	0.00305160	0.02904092	0.02503044
637	0.00487384	0.03091961	0.02394385
638	0.00674858	0.03265930	0.02260657
639	0.00866477	0.03424095	0.02095775
640	0.01066217	0.03565456	0.01904493
641	0.01284287	0.03689225	0.01698158
642	0.01506275	0.03792800	0.01465699
643	0.01752215	0.03880487	0.01216451
644	0.02014270	0.03944882	0.00954716
645	0.02340023	0.03943492	0.00647093
646	0.02673268	0.03871490	0.00364816
647	0.03008663	0.03726287	0.00105856
648	0.03344046	0.03507928	-0.00123823
649	0.03667868	0.03221603	-0.00324791
650	0.03981107	0.02872670	-0.00494051
651	0.04291168	0.02450239	-0.00629910
652	0.04590004	0.01959179	-0.00741564
653	0.04879830	0.01409664	-0.00822162
654	0.05068151	0.00689406	-0.00834764

655	0.05181140	0.00037476	-0.00803719
656	0.05213294	-0.00552906	-0.00746948
657	0.05163719	-0.01078857	-0.00668413
658	0.05030713	-0.01547305	-0.00574009
659	0.04818115	-0.01953440	-0.00452684
660	0.04528586	-0.02298292	-0.00301353
661	0.04168932	-0.02582057	-0.00111313
662	0.03732637	-0.02807688	0.00115063
663	0.03359518	-0.02911164	0.00316084
664	0.03005810	-0.02946220	0.00507341
665	0.02680777	-0.02917642	0.00701736
666	0.02373827	-0.02820005	0.00888963
667	0.02090544	-0.02658400	0.01067561
668	0.01827106	-0.02430519	0.01243513
669	0.01584971	-0.02129268	0.01412034
670	0.01362293	-0.01750124	0.01570763
671	0.01179273	-0.01300780	0.01724954
672	0.01200996	-0.00734099	0.01817775
673	0.01294675	-0.00252484	0.01895725
674	0.01446004	0.00143259	0.01935239
675	0.01641046	0.00458804	0.01925005
676	0.01876308	0.00701671	0.01871820
677	0.02156132	0.00881190	0.01777147
678	0.02493034	0.00997127	0.01650205
679	0.02890828	0.01047790	0.01487471
680	0.03358545	0.01029535	0.01299573
681	0.03577511	0.00890359	0.01212176
682	0.03769097	0.00733229	0.01139926

683	0.03930814	0.00559615	0.01087341
684	0.04055220	0.00370790	0.01047069
685	0.04146589	0.00168853	0.01028547
686	0.04202705	-0.00045995	0.01022936
687	0.04227211	-0.00272680	0.01037014
688	0.04219857	-0.00510634	0.01062953
689	0.04179406	-0.00762861	0.01104035
690	0.04095892	-0.00930572	0.01160926
691	0.04003147	-0.01077235	0.01222707
692	0.03900715	-0.01198729	0.01285521
693	0.03791750	-0.01292111	0.01353237
694	0.03675270	-0.01357024	0.01421624
695	0.03556127	-0.01394403	0.01492729
696	0.03431049	-0.01404469	0.01567399
697	0.03304742	-0.01389941	0.01641429
698	0.03156475	-0.01339451	0.01727044
699	0.03006722	-0.01192353	0.01818341
700	0.02871193	-0.01034120	0.01892260
701	0.02781598	-0.00881156	0.01936846
702	0.02733167	-0.00746988	0.01946722
703	0.02712914	-0.00643493	0.01931227
704	0.02696520	-0.00552820	0.01897578
705	0.02670683	-0.00450978	0.01832306
706	0.02630382	-0.00354606	0.01695839
707	0.02589855	-0.00318834	0.01474129
708	-0.01520552	0.00590155	-0.00466121
709	-0.01587966	0.00515075	-0.00634812
710	-0.01665369	0.00450525	-0.00807283

711	-0.01759561	0.00393161	-0.00991982
712	-0.01852073	0.00353691	-0.01164814
713	-0.01948182	0.00326484	-0.01321439
714	-0.02033413	0.00309327	-0.01452637
715	-0.02113927	0.00304111	-0.01628112
716	-0.02203928	0.00325219	-0.01803815
717	-0.02230605	0.00335715	-0.01886560

Procrustes sums of squares: 0.34826545124891567

Tangent sums of squares: 0.34382815329249755

Data matrices in this dataset:

- BE_All, raw data
- BE_All, centroid size
- BE_All, Procrustes coordinates

Principal Component Analysis: PCA: CovMatrix, BE_All, Procrustes coordinates

	Eigenvalues	% Variance	Cumulative %
1.	0.00257477	23.214	23.214
2.	0.00160293	14.452	37.667
3.	0.00133771	12.061	49.728
4.	0.00093675	8.446	58.173
5.	0.00065046	5.865	64.038
6.	0.00061976	5.588	69.626

7.	0.00051956	4.684	74.310
8.	0.00036711	3.310	77.620
9.	0.00033446	3.015	80.636
10.	0.00024465	2.206	82.842
11.	0.00023014	2.075	84.916
12.	0.00018305	1.650	86.567
13.	0.00016516	1.489	88.056
14.	0.00014966	1.349	89.405
15.	0.00013769	1.241	90.647
16.	0.00011962	1.078	91.725
17.	0.00010429	0.940	92.665
18.	0.00009963	0.898	93.564
19.	0.00009075	0.818	94.382
20.	0.00008418	0.759	95.141
21.	0.00007843	0.707	95.848
22.	0.00007193	0.649	96.497
23.	0.00006882	0.620	97.117
24.	0.00005992	0.540	97.657
25.	0.00005137	0.463	98.121
26.	0.00004562	0.411	98.532
27.	0.00004426	0.399	98.931
28.	0.00003553	0.320	99.251
29.	0.00003152	0.284	99.535
30.	0.00002931	0.264	99.800
31.	0.00002221	0.200	100.000

Total variance: 0.01109123

Variance of the eigenvalues: 0.0000000062628

Eigenvalue variance scaled by total variance: 0.00005

Eigenvalue variance scaled by total variance and number of variables: 0.10920

Note: throughout all calculations of eigenvalue variances, the dimensionality used was 2144.

Please double-check because this dimensionality may not be appropriate for all situations.

Warning: There appear to be fewer PCs than dimensions in shape space. This can have the following reasons, among others:

-- the degrees of freedom are less than the shape dimensionality (sample size, etc.).

-- there is complete integration in the data.

Interpret the results cautiously and check for reasons why this is the case.

Principal Component Coefficients

PC1 PC2 PC3 PC4 PC5 PC6 PC7 PC8 PC9 PC10 PC11 PC12
PC13 PC14 PC15 PC16 PC17 PC18 PC19 PC20 PC21 PC22 PC23 PC24
PC25 PC26 PC27 PC28 PC29 PC30 PC31

x1	0.002820	0.031757	0.027090	-0.001847	0.012634	0.031140
	0.036100	-0.016403	-0.018428	0.023260	0.030205	-0.030654
	-0.009872	0.003615	0.007616	0.006617	0.015050	-0.005242
	-0.006196	0.023375	-0.021947	0.002355	-0.025966	0.013446
	-0.003973	-0.009093	0.003502	0.020367	-0.000479	-0.004317
	-0.008694					
y1	-0.008692	0.002924	-0.014565	-0.000665	0.001961	0.017528
	-0.009914	0.013999	0.004578	0.001741	0.028657	-0.002136
	-0.006323	-0.005686	-0.008932	0.011776	-0.013399	-0.012892
	0.009055	-0.005538	0.020428	-0.010014	0.007107	0.008024
	0.001486	-0.045736	-0.028222	0.020641	-0.001985	-0.007806
	0.002169					
z1	0.045174	0.010382	0.009367	-0.012406	-0.023659	0.009472
	0.000337	-0.001231	0.001702	0.028817	0.022570	0.016817
	0.009209	0.016511	-0.000125	-0.007698	-0.002631	0.010725
	0.014146	-0.013606	-0.025390	-0.032253	-0.014856	0.001750
	0.015009	0.003969	0.007587	-0.013702	-0.007640	-0.001916
	-0.013895					
x2	-0.012043	0.004469	-0.005026	0.011890	0.016239	-0.024798
	0.014021	0.026748	-0.024449	0.004194	0.007293	-0.012403

	-0.023803	0.007209	-0.002484	-0.020008	-0.021959	-0.019950
	-0.001887	-0.030706	0.023335	-0.014215	-0.002562	-0.004544
	-0.018827	0.003468	-0.001526	0.003346	-0.029431	0.012196
	-0.021156					
y2	0.054579	0.012741	0.003586	-0.007049	0.008484	-0.016289
	-0.021801	0.026692	0.003766	0.016809	-0.032419	-0.018683
	0.003186	0.002647	-0.004410	-0.031190	-0.000563	0.004882
	-0.033311	0.003799	0.030944	0.016911	0.013238	-0.016000
	0.023992	0.007866	0.012916	0.022834	0.002651	-0.011744
	-0.019396					
z2	0.014560	-0.017946	0.031911	-0.008069	-0.000311	-0.013189
	0.000504	-0.007095	0.000486	0.010592	-0.028264	-0.010509
	-0.013223	-0.016328	0.001217	-0.002714	0.025058	-0.019570
	0.001745	0.004883	0.020224	0.010650	-0.003653	0.004291
	-0.019563	-0.004572	-0.004921	0.005317	0.013407	0.006868
	-0.005530					
x3	-0.011547	0.004961	-0.002906	0.011933	0.015606	-0.023177
	0.013466	0.027010	-0.022566	0.005486	0.004687	-0.010918
	-0.024833	0.009104	0.000377	-0.024209	-0.028555	-0.019473
	-0.003076	-0.032305	0.016705	-0.011614	0.003644	-0.007318
	-0.022229	0.004257	-0.004035	-0.001664	-0.027682	0.013157
	-0.018513					
y3	0.056303	0.014741	0.005539	-0.007579	0.005997	-0.014886
	-0.021144	0.026245	0.002651	0.016427	-0.031143	-0.015875
	0.003540	0.001594	-0.004877	-0.031589	0.002251	0.013733
	-0.031777	0.011962	0.026781	0.020453	0.009665	-0.015300
	0.024898	0.004471	0.015540	0.017110	0.001122	-0.015060
	-0.023481					
z3	0.016818	-0.017638	0.031166	-0.008668	-0.000130	-0.014341
	0.000106	-0.007733	-0.001040	0.013752	-0.029193	-0.011463
	-0.012484	-0.016034	0.003161	-0.001604	0.023979	-0.016153
	0.001652	0.007077	0.020257	0.012618	-0.004965	0.004079
	-0.018958	-0.002331	-0.005026	0.002270	0.013669	0.006588
	-0.000211					
x4	-0.010687	0.006035	-0.000147	0.012328	0.015473	-0.021151
	0.014099	0.027063	-0.021370	0.006730	0.002864	-0.009786
	-0.027736	0.011055	0.001728	-0.026362	-0.030524	-0.016592
	-0.005081	-0.030564	0.010505	-0.009980	0.007303	-0.007700
	-0.022135	0.004077	-0.004102	-0.005874	-0.025931	0.010180
	-0.015025					

y4	0.056876	0.016832	0.007153	-0.007910	0.004682	-0.013378
	-0.020658	0.026404	0.001863	0.017157	-0.030276	-0.012849
	0.002944	0.002125	-0.005994	-0.030799	0.003669	0.021005
	-0.030003	0.017689	0.024075	0.020781	0.006882	-0.013677
	0.023516	0.001802	0.015984	0.010396	0.003002	-0.018373
	-0.022573					
z4	0.019896	-0.016579	0.030776	-0.009270	-0.000958	-0.015038
	-0.000650	-0.008335	-0.001698	0.016563	-0.029887	-0.011712
	-0.011482	-0.016096	0.004251	-0.000662	0.021777	-0.012657
	0.001258	0.008470	0.019116	0.014795	-0.003580	0.002167
	-0.018161	-0.000102	-0.004006	-0.001258	0.012992	0.004894
	-0.000117					
x5	-0.009307	0.007471	0.001970	0.013244	0.015963	-0.019012
	0.014871	0.027783	-0.021699	0.008700	0.001354	-0.008136
	-0.029387	0.011730	0.002212	-0.027921	-0.028175	-0.012878
	-0.004234	-0.029162	0.004871	-0.005410	0.009243	-0.006747
	-0.015398	0.004309	-0.007344	-0.005669	-0.033950	0.007014
	-0.007368					
y5	0.055940	0.018081	0.007533	-0.007862	0.004177	-0.011579
	-0.020987	0.027637	0.000846	0.018765	-0.029935	-0.009090
	0.003118	0.000784	-0.006598	-0.027547	0.002996	0.024569
	-0.026507	0.020030	0.021982	0.023961	0.007299	-0.013193
	0.021854	0.001028	0.012306	0.007671	0.005291	-0.018953
	-0.020123					
z5	0.022831	-0.015417	0.030623	-0.009689	-0.002059	-0.015070
	-0.001414	-0.008227	-0.002098	0.018640	-0.031074	-0.011201
	-0.009301	-0.015586	0.003891	-0.000148	0.018192	-0.009793
	0.001885	0.009087	0.018277	0.015287	-0.001404	-0.000028
	-0.019590	0.000988	-0.002652	-0.004389	0.014749	0.001432
	0.000392					
x6	-0.007881	0.008872	0.004109	0.013585	0.015538	-0.016517
	0.016192	0.026599	-0.022004	0.009217	0.000235	-0.008371
	-0.029819	0.011145	0.003354	-0.026688	-0.026109	-0.009131
	-0.005400	-0.025707	0.000082	-0.000886	0.010473	-0.007138
	-0.009915	0.000799	-0.007722	-0.004348	-0.034019	0.003944
	-0.001465					
y6	0.054694	0.018539	0.007471	-0.007716	0.002984	-0.009721
	-0.020735	0.027553	0.000050	0.018639	-0.028778	-0.006835
	0.003079	-0.001660	-0.007815	-0.022861	0.003668	0.027232
	-0.023856	0.022339	0.020081	0.026402	0.008018	-0.012837
	0.023193	-0.001326	0.010077	0.006188	0.005942	-0.022633
	-0.015547					

z6	0.025564	-0.014239	0.030308	-0.010015	-0.003175	-0.014795
	-0.002010	-0.008070	-0.002271	0.020394	-0.031650	-0.010423
	-0.007371	-0.015661	0.003274	0.001072	0.014390	-0.007755
	0.002246	0.009484	0.017166	0.016206	0.001972	-0.003361
	-0.020515	0.002666	-0.000838	-0.006914	0.017534	-0.002551
	-0.000852					
x7	-0.005991	0.011539	0.005520	0.014082	0.015711	-0.014044
	0.017734	0.026644	-0.021344	0.009313	0.000561	-0.009194
	-0.030360	0.011720	0.004757	-0.025547	-0.023995	-0.008007
	-0.007139	-0.022303	-0.001249	0.003480	0.008799	-0.007793
	-0.008019	-0.003432	-0.007734	-0.002653	-0.026896	0.006258
	-0.002580					
y7	0.053467	0.019102	0.007073	-0.007109	0.001901	-0.008018
	-0.020281	0.028232	0.000356	0.018473	-0.027144	-0.006241
	0.002302	-0.002381	-0.009423	-0.020904	0.004213	0.026301
	-0.020487	0.023021	0.020889	0.026660	0.009230	-0.012847
	0.023467	-0.004060	0.007473	0.004530	0.005554	-0.023575
	-0.016025					
z7	0.028432	-0.012831	0.030178	-0.010049	-0.004499	-0.014395
	-0.002618	-0.007363	-0.001919	0.022308	-0.031894	-0.010322
	-0.005392	-0.014817	0.002724	0.001095	0.010868	-0.005839
	0.003254	0.008977	0.016875	0.015756	0.005329	-0.006881
	-0.020521	0.004385	0.000672	-0.010130	0.019044	-0.006358
	-0.002915					
x8	-0.004442	0.013042	0.007702	0.013678	0.015387	-0.012825
	0.017552	0.025261	-0.019589	0.009758	-0.001319	-0.008372
	-0.030153	0.011163	0.001791	-0.026946	-0.024672	-0.007031
	-0.008660	-0.019204	-0.004651	0.002404	0.005030	-0.007937
	-0.007007	-0.003898	-0.009657	0.000605	-0.019623	0.009018
	0.003236					
y8	0.051300	0.019270	0.006821	-0.006159	0.001272	-0.006999
	-0.020855	0.029726	0.002361	0.018847	-0.025970	-0.005158
	0.003104	-0.002226	-0.012792	-0.021365	0.002312	0.024421
	-0.016454	0.022162	0.020683	0.022434	0.009982	-0.012160
	0.023348	-0.003766	0.002601	0.004989	0.007199	-0.020632
	-0.013567					
z8	0.030993	-0.011213	0.030311	-0.009652	-0.005540	-0.014229
	-0.004130	-0.006274	-0.000834	0.024775	-0.032437	-0.009456
	-0.002240	-0.013746	0.000817	0.000045	0.005002	-0.004713
	0.003991	0.007648	0.016889	0.013723	0.010027	-0.010285
	-0.021015	0.007661	0.000901	-0.011826	0.020657	-0.009547
	-0.005945					

x9	-0.002754	0.015521	0.010057	0.013103	0.015540	-0.011857
	0.017048	0.023607	-0.018786	0.011742	-0.003383	-0.006930
	-0.029460	0.010832	0.000396	-0.026983	-0.024731	-0.005614
	-0.012219	-0.014385	-0.008493	0.000665	0.002353	-0.009276
	-0.005680	-0.003986	-0.008567	0.001446	-0.013838	0.008096
	0.007110					
y9	0.048772	0.019612	0.006497	-0.004805	0.001251	-0.005659
	-0.021247	0.031621	0.003872	0.021008	-0.025272	-0.003984
	0.004487	-0.002460	-0.014441	-0.021564	0.001316	0.023020
	-0.013967	0.022029	0.019963	0.018281	0.012033	-0.011341
	0.024086	-0.002211	-0.001343	0.001693	0.008474	-0.020197
	-0.013796					
z9	0.033387	-0.009732	0.030651	-0.008840	-0.006142	-0.013862
	-0.005419	-0.004426	0.000263	0.028613	-0.033468	-0.007220
	0.001333	-0.012467	0.000439	-0.000761	-0.001556	-0.002967
	0.004117	0.006310	0.015666	0.010171	0.015533	-0.013556
	-0.021444	0.012535	0.000510	-0.016052	0.023012	-0.014131
	-0.009242					
x10	-0.000878	0.018359	0.011784	0.012761	0.015757	-0.009729
	0.017925	0.020996	-0.019448	0.012462	-0.002891	-0.005870
	-0.027099	0.010818	0.002611	-0.023677	-0.021675	-0.001100
	-0.014696	-0.012601	-0.012108	0.003820	-0.005433	-0.012311
	-0.004633	-0.007808	-0.009759	0.006889	-0.009003	0.012717
	0.015844					
y10	0.046432	0.019567	0.005878	-0.003145	0.001174	-0.003249
	-0.020231	0.033159	0.004163	0.022432	-0.022465	-0.001916
	0.006813	-0.003098	-0.013760	-0.018145	0.002794	0.023894
	-0.011631	0.020821	0.017333	0.016446	0.012584	-0.011144
	0.026789	-0.004620	-0.008457	0.000702	0.007848	-0.017261
	-0.009996					
z10	0.036179	-0.008093	0.030628	-0.007790	-0.006715	-0.012812
	-0.005586	-0.002072	0.000721	0.031830	-0.032439	-0.006286
	0.006054	-0.010511	0.002683	-0.000596	-0.004497	0.002235
	0.005478	0.005376	0.014175	0.009216	0.017198	-0.016168
	-0.017382	0.014005	-0.002114	-0.018594	0.026359	-0.013574
	-0.011959					
x11	0.000355	0.020624	0.013156	0.010359	0.015897	-0.007941
	0.018710	0.016099	-0.020972	0.011689	-0.002077	-0.006081
	-0.027850	0.007807	0.003046	-0.021765	-0.018297	0.002226
	-0.015656	-0.010996	-0.013962	0.007726	-0.007191	-0.014074
	-0.005836	-0.008158	-0.008949	0.006743	-0.004684	0.009780
	0.019174					

y11	0.044124	0.019133	0.005413	-0.001688	0.001018	-0.001228
	-0.019087	0.033578	0.005437	0.021229	-0.019529	-0.000576
	0.007452	-0.005547	-0.015054	-0.017468	0.005034	0.024950
	-0.009693	0.022151	0.017738	0.013434	0.011668	-0.007741
	0.029439	-0.010947	-0.014522	-0.001100	0.002819	-0.018504
	-0.011116					
z11	0.039862	-0.006705	0.031207	-0.006520	-0.007658	-0.012132
	-0.005625	-0.001102	0.001889	0.033130	-0.032067	-0.004400
	0.009835	-0.009906	0.002719	-0.003193	-0.007625	0.007159
	0.005459	0.005528	0.013009	0.006257	0.019269	-0.016477
	-0.014422	0.012519	-0.004771	-0.020422	0.025061	-0.019055
	-0.014704					
x12	0.002369	0.021644	0.016010	0.008820	0.016324	-0.006597
	0.020049	0.010621	-0.019505	0.012349	-0.003117	-0.009389
	-0.031210	0.001351	0.000157	-0.019423	-0.013090	0.002527
	-0.020638	-0.003972	-0.013972	0.009387	-0.003907	-0.015301
	-0.003248	-0.006112	-0.001602	0.005757	0.004871	0.008374
	0.016440					
y12	0.041016	0.018371	0.004548	-0.001144	0.000278	-0.000472
	-0.018694	0.033201	0.005922	0.018281	-0.017262	0.001301
	0.008310	-0.008097	-0.016354	-0.014997	0.003248	0.023475
	-0.008622	0.020953	0.017765	0.011421	0.013076	-0.004876
	0.026194	-0.012461	-0.018219	-0.002005	-0.003317	-0.020097
	-0.004398					
z12	0.042338	-0.005426	0.030939	-0.005968	-0.008971	-0.012028
	-0.006680	0.000337	0.002136	0.032219	-0.032997	-0.000712
	0.014518	-0.008174	0.003856	-0.004037	-0.015702	0.008851
	0.007957	0.001325	0.011012	0.004818	0.024843	-0.018377
	-0.020439	0.016148	-0.008639	-0.023737	0.022361	-0.024691
	-0.009026					
x13	0.004140	0.023578	0.018033	0.008204	0.016910	-0.005000
	0.021195	0.008079	-0.018572	0.013457	-0.004062	-0.008509
	-0.034231	-0.002440	-0.001786	-0.016990	-0.009412	0.005066
	-0.028923	0.002660	-0.014336	0.012252	-0.006869	-0.014855
	-0.005794	-0.010014	0.001941	0.012222	0.021737	0.009775
	0.003156					
y13	0.037340	0.017883	0.003195	-0.000904	-0.000489	0.000762
	-0.018019	0.033288	0.006610	0.016615	-0.013197	0.002563
	0.007892	-0.009888	-0.015577	-0.010496	0.002330	0.021239
	-0.010333	0.020139	0.017380	0.010807	0.013009	-0.004612
	0.022719	-0.014039	-0.018825	0.001083	0.003199	-0.014969
	-0.009316					

z13	0.044469	-0.004159	0.030480	-0.005317	-0.010576	-0.011841
	-0.007507	0.001440	0.001504	0.032445	-0.031877	0.002250
	0.017516	-0.006324	0.005231	-0.004279	-0.019011	0.012476
	0.008513	-0.001891	0.006923	0.003817	0.028445	-0.020165
	-0.021194	0.019350	-0.010451	-0.023494	0.023494	-0.026965
	-0.009141					
x14	0.005814	0.024775	0.019495	0.006893	0.017237	-0.003176
	0.023256	0.005431	-0.016612	0.014786	-0.001197	-0.006556
	-0.032229	-0.004569	-0.003462	-0.012884	-0.004233	0.002711
	-0.026637	0.009230	-0.015611	0.013695	-0.006164	-0.014459
	-0.001999	-0.010760	0.002961	0.015277	0.028918	-0.003269
	0.000383					
y14	0.034044	0.017280	0.001589	-0.000610	-0.001145	0.002287
	-0.016996	0.032586	0.007991	0.014775	-0.009182	0.004288
	0.008270	-0.010727	-0.016406	-0.006925	0.003404	0.020771
	-0.007766	0.019667	0.015874	0.008260	0.011380	-0.003025
	0.022294	-0.016063	-0.020277	0.001777	0.001220	-0.015162
	-0.004372					
z14	0.046593	-0.002442	0.029882	-0.005466	-0.012101	-0.011372
	-0.007800	0.001356	0.002212	0.031883	-0.029532	0.005493
	0.019933	-0.005232	0.005247	-0.003769	-0.021358	0.016869
	0.009774	-0.002635	0.001819	0.002226	0.029315	-0.022042
	-0.020260	0.021248	-0.009400	-0.023854	0.021972	-0.033673
	-0.003684					
x15	0.006982	0.026119	0.021534	0.005767	0.017373	-0.001349
	0.024745	0.002554	-0.017030	0.017291	0.000409	-0.006052
	-0.031046	-0.005632	-0.001925	-0.011832	-0.000293	0.003774
	-0.020822	0.013986	-0.016764	0.013400	-0.006625	-0.010590
	0.004338	-0.012046	0.000899	0.015224	0.023902	-0.011235
	-0.003724					
y15	0.030292	0.016571	0.000344	-0.000937	-0.001562	0.003563
	-0.016444	0.032272	0.007935	0.012831	-0.005290	0.004709
	0.007608	-0.011588	-0.015385	-0.003373	0.004064	0.021031
	-0.004858	0.017458	0.015302	0.005548	0.010468	0.001124
	0.022922	-0.017900	-0.021149	0.002599	-0.002433	-0.013084
	-0.001615					
z15	0.048316	-0.000782	0.029985	-0.005469	-0.013561	-0.010605
	-0.007945	0.001886	0.000883	0.031871	-0.026888	0.007438
	0.021872	-0.002995	0.007126	-0.004474	-0.022259	0.021346
	0.012569	-0.005718	-0.000954	0.000231	0.029171	-0.021883
	-0.016590	0.022161	-0.007708	-0.025674	0.016257	-0.036807
	0.002779					

x16	0.007134	0.027462	0.023362	0.005040	0.018602	0.000992
	0.024533	-0.001869	-0.016283	0.018954	0.002102	-0.006283
	-0.031941	-0.008388	-0.000864	-0.009857	0.000564	0.005423
	-0.021756	0.019199	-0.018966	0.016604	-0.010798	-0.009727
	0.007107	-0.012895	-0.000944	0.018480	0.023809	-0.012403
	-0.011879					
y16	0.026453	0.015616	-0.001184	-0.000941	-0.001380	0.004840
	-0.015871	0.031504	0.008499	0.011481	-0.001166	0.004557
	0.006457	-0.012645	-0.015156	-0.000992	0.005355	0.019161
	-0.002172	0.015623	0.015085	0.003980	0.009685	0.003946
	0.023614	-0.020163	-0.021601	0.003463	-0.006250	-0.009250
	0.000099					
z16	0.049568	0.000805	0.029578	-0.005300	-0.014218	-0.009411
	-0.008078	0.001964	0.001794	0.032607	-0.022391	0.008776
	0.022681	-0.002409	0.007548	-0.005737	-0.022931	0.021593
	0.013675	-0.007698	-0.002696	0.000012	0.027609	-0.023635
	-0.012902	0.021899	-0.004503	-0.025192	0.016184	-0.033423
	0.004631					
x17	0.007395	0.028407	0.024753	0.004702	0.019376	0.003737
	0.025524	-0.005802	-0.016756	0.019891	0.002792	-0.007179
	-0.030037	-0.007880	0.001423	-0.010396	0.001774	0.000732
	-0.021825	0.023677	-0.019250	0.020242	-0.014153	-0.008824
	0.006579	-0.015951	-0.005084	0.021656	0.021417	-0.017743
	-0.021286					
y17	0.022352	0.014512	-0.002596	-0.000598	-0.000862	0.006219
	-0.015171	0.031129	0.008606	0.009859	0.002384	0.004482
	0.005373	-0.013562	-0.015156	0.001399	0.005979	0.017420
	-0.000316	0.013679	0.014770	0.001739	0.008838	0.006956
	0.023047	-0.021670	-0.023177	0.004864	-0.007954	-0.004783
	0.001797					
z17	0.050535	0.002180	0.029370	-0.004932	-0.015103	-0.007751
	-0.007303	0.002146	0.001471	0.032564	-0.019021	0.011165
	0.023679	-0.002082	0.007392	-0.009435	-0.024751	0.020241
	0.013045	-0.008423	-0.003992	-0.001532	0.025963	-0.023579
	-0.012470	0.022213	-0.003017	-0.023555	0.019896	-0.030137
	0.007937					
x18	0.007102	0.028694	0.025158	0.004198	0.018323	0.007822
	0.026431	-0.006004	-0.017803	0.020028	0.000988	-0.006665
	-0.027120	-0.003713	0.003802	-0.010126	-0.000174	0.001122
	-0.016824	0.025578	-0.020720	0.024693	-0.014895	-0.010728
	0.008351	-0.019971	-0.003897	0.025094	0.015766	-0.028047
	-0.023875					

y18	0.018019	0.013437	-0.003990	-0.000481	-0.000765	0.007736
	-0.014213	0.031112	0.008746	0.007810	0.005187	0.004592
	0.005228	-0.012975	-0.014512	0.004049	0.004970	0.015654
	0.002005	0.011009	0.014147	0.000299	0.007288	0.007977
	0.022242	-0.024795	-0.022645	0.006233	-0.008698	-0.003526
	0.005931					
z18	0.050844	0.003568	0.028843	-0.004147	-0.016800	-0.004657
	-0.006272	0.005587	0.000194	0.032406	-0.018016	0.012808
	0.026209	0.002034	0.008513	-0.012251	-0.027682	0.021558
	0.017680	-0.011835	-0.005218	-0.002210	0.024520	-0.026649
	-0.007280	0.020256	0.001316	-0.022331	0.018351	-0.030480
	0.022987					
x19	0.005685	0.028335	0.026199	0.004280	0.018495	0.010772
	0.025422	-0.006050	-0.017582	0.021276	0.001096	-0.006573
	-0.024114	0.001490	0.003121	-0.006717	0.000319	-0.004219
	-0.008768	0.026195	-0.022617	0.025052	-0.014805	-0.010603
	0.000860	-0.023031	-0.009926	0.021074	0.014110	-0.033677
	-0.030272					
y19	0.014472	0.012139	-0.005497	-0.000094	-0.000680	0.008980
	-0.012634	0.030195	0.008698	0.005773	0.009307	0.004135
	0.005027	-0.012950	-0.014394	0.005761	0.005800	0.013185
	0.003724	0.009235	0.014402	-0.001233	0.006308	0.010492
	0.023136	-0.027303	-0.021445	0.007427	-0.010104	-0.003445
	0.008430					
z19	0.049907	0.004207	0.028484	-0.002870	-0.017037	-0.001578
	-0.006093	0.009233	0.000621	0.033696	-0.014770	0.014641
	0.026371	0.005114	0.006429	-0.012929	-0.027202	0.016879
	0.024505	-0.014002	-0.004135	-0.003321	0.026619	-0.025593
	-0.013381	0.020014	-0.005463	-0.024755	0.024295	-0.019256
	0.023535					
x20	0.005072	0.028432	0.026408	0.003866	0.017509	0.013003
	0.027151	-0.008229	-0.018804	0.021070	0.004273	-0.007092
	-0.020442	0.005697	0.006026	-0.005333	0.000412	-0.006539
	-0.009137	0.026606	-0.022685	0.022224	-0.017213	-0.010820
	0.002275	-0.024003	-0.003667	0.021507	0.008284	-0.042839
	-0.022462					
y20	0.010349	0.010726	-0.007215	0.000318	0.000096	0.010104
	-0.012086	0.029410	0.008928	0.003767	0.012819	0.003617
	0.003066	-0.013404	-0.013296	0.008557	0.004596	0.010520
	0.005355	0.006246	0.014872	-0.001510	0.005141	0.011934
	0.020877	-0.030290	-0.022607	0.007985	-0.008668	-0.000269
	0.008375					

z20	0.050037	0.005001	0.027409	-0.001665	-0.019373	0.001670
	-0.003210	0.008509	-0.000406	0.031315	-0.008198	0.013047
	0.022750	0.004358	0.003943	-0.013556	-0.023675	0.013645
	0.019454	-0.012689	-0.001912	-0.005478	0.021126	-0.023832
	-0.008479	0.023618	-0.004272	-0.015621	0.024482	-0.016293
	0.002123					
x21	0.003357	0.028354	0.027622	0.003382	0.015871	0.016329
	0.027511	-0.010323	-0.015816	0.019271	0.004463	-0.009819
	-0.019031	0.004467	0.010539	-0.002003	-0.000522	-0.007631
	-0.007515	0.024266	-0.021276	0.019596	-0.015973	-0.004639
	-0.000623	-0.023284	-0.004171	0.021879	0.013302	-0.044802
	-0.009333					
y21	0.005210	0.009165	-0.010132	0.001073	0.000983	0.011684
	-0.010993	0.028444	0.007593	0.002338	0.017022	0.003553
	0.002035	-0.011704	-0.011258	0.011927	0.003697	0.007519
	0.006564	0.001845	0.015178	-0.002057	0.003684	0.013520
	0.019682	-0.033448	-0.023053	0.007146	-0.008784	0.002690
	0.004812					
z21	0.049348	0.006127	0.027895	-0.003592	-0.021007	0.004406
	-0.002107	0.006720	-0.003150	0.026123	-0.001554	0.011222
	0.017555	0.003825	-0.000766	-0.016703	-0.019565	0.011237
	0.022401	-0.004613	-0.007168	-0.002580	0.018834	-0.021339
	-0.012103	0.014132	-0.002315	-0.005319	0.015367	-0.014975
	-0.015611					
x22	0.001778	0.028355	0.028038	0.001896	0.014979	0.018317
	0.028626	-0.015000	-0.015005	0.017748	0.009435	-0.013233
	-0.021577	0.004228	0.008864	0.000981	0.005625	-0.007205
	-0.011246	0.023836	-0.019821	0.010210	-0.015923	0.001949
	-0.000109	-0.018083	-0.003741	0.016861	0.006002	-0.046798
	0.000656					
y22	0.000984	0.007291	-0.012388	0.002086	0.001676	0.012076
	-0.010789	0.027611	0.007484	0.000841	0.018214	0.004321
	0.001636	-0.011205	-0.008520	0.013747	-0.001796	0.004014
	0.007997	-0.002237	0.014780	-0.000627	0.004522	0.013073
	0.016800	-0.036354	-0.022876	0.006679	-0.006046	0.002596
	0.002382					
z22	0.048242	0.007609	0.027050	-0.005864	-0.020800	0.007370
	0.000565	0.005248	-0.005349	0.024021	0.008547	0.009503
	0.016010	0.007846	-0.001353	-0.015161	-0.012719	0.008980
	0.023741	0.001197	-0.010422	-0.004044	0.013224	-0.015831
	-0.010453	0.006392	0.001213	-0.002291	0.014597	-0.007309
	-0.018674					

x23	0.000664	0.028560	0.027715	0.001421	0.014263	0.019725
	0.029093	-0.020561	-0.012204	0.015952	0.014549	-0.014307
	-0.022042	0.002089	0.006394	0.006220	0.010668	-0.006475
	-0.013505	0.022277	-0.016984	-0.000130	-0.020159	0.005384
	0.001524	-0.021111	0.001223	0.008066	0.004074	-0.051476
	0.000332					
y23	-0.002510	0.005442	-0.013808	0.003583	0.002290	0.011499
	-0.010938	0.027954	0.007381	-0.000211	0.018601	0.003844
	0.002516	-0.009368	-0.005183	0.013869	-0.004976	0.004190
	0.009186	-0.004053	0.012266	0.001145	0.006165	0.012989
	0.015380	-0.036461	-0.026159	0.008510	-0.003161	0.007609
	0.005929					
z23	0.045758	0.008504	0.025411	-0.008439	-0.019060	0.010354
	0.002461	0.002956	-0.006233	0.023379	0.014660	0.010574
	0.015793	0.012315	-0.002749	-0.013457	-0.012830	0.002306
	0.024180	0.001392	-0.013531	-0.005500	0.011866	-0.012574
	-0.013491	0.001208	0.004931	-0.007071	0.012045	-0.006903
	-0.024833					
x24	-0.002462	0.027759	0.026078	0.002622	0.013813	0.019757
	0.028727	-0.022757	-0.009000	0.014836	0.013073	-0.012086
	-0.018310	0.000703	0.006690	0.011095	0.008420	-0.007075
	-0.012357	0.014195	-0.015368	-0.007276	-0.014689	0.003717
	0.000194	-0.020910	0.001695	-0.000387	0.001810	-0.049270
	0.000689					
y24	-0.003742	0.004245	-0.013076	0.005020	0.002894	0.009398
	-0.010522	0.027011	0.007497	-0.001605	0.019354	0.004171
	0.001796	-0.008880	-0.005497	0.011130	-0.004686	0.006555
	0.009100	0.001670	0.011385	0.003048	0.004445	0.013859
	0.015694	-0.035791	-0.025914	0.009085	0.000402	0.006851
	0.006978					
z24	0.042316	0.009667	0.023952	-0.010069	-0.016799	0.012421
	0.003822	0.001726	-0.006409	0.022860	0.016695	0.011494
	0.016502	0.016106	-0.001693	-0.007998	-0.016199	-0.002757
	0.024503	-0.001058	-0.015633	-0.004800	0.011651	-0.012874
	-0.012911	-0.004018	0.005926	-0.010650	0.010265	-0.003360
	-0.022158					
x25	-0.006366	0.027157	0.024613	0.004604	0.014256	0.018520
	0.029128	-0.022568	-0.006380	0.010998	0.009063	-0.009342
	-0.015889	-0.000402	0.005612	0.013595	0.005241	-0.006526
	-0.010931	0.008703	-0.012052	-0.008228	-0.008904	-0.000077
	-0.003325	-0.017068	-0.004556	-0.007101	-0.004359	-0.048077
	-0.001998					

y25	-0.003488	0.001886	-0.010643	0.005773	0.001833	0.007113
	-0.009076	0.025679	0.008846	-0.002014	0.021694	0.003553
	0.005146	-0.007713	-0.006404	0.006272	0.002491	0.002346
	0.010983	0.011195	0.012404	-0.000246	0.003940	0.013790
	0.023007	-0.039079	-0.016809	0.006632	0.008905	-0.000026
	0.002467					
z25	0.038273	0.011370	0.023704	-0.009615	-0.013707	0.013487
	0.004996	0.002526	-0.006575	0.020824	0.015534	0.011577
	0.015923	0.016938	-0.000312	-0.000291	-0.020905	-0.004535
	0.023254	-0.000866	-0.015526	-0.000278	0.010787	-0.012263
	-0.013529	-0.005399	0.004157	-0.011363	0.010364	0.001484
	-0.013419					
x26	-0.009595	0.025561	0.022916	0.006055	0.015371	0.015251
	0.031791	-0.022386	-0.004166	0.004821	0.007522	-0.007834
	-0.014272	-0.001592	0.003526	0.012390	0.010243	-0.003333
	-0.014471	0.009392	-0.008078	-0.006899	-0.009546	-0.001805
	0.002052	-0.019415	-0.002098	-0.013321	-0.008376	-0.047163
	-0.004401					
y26	-0.004726	0.002001	-0.008638	0.008393	-0.001459	0.006701
	-0.011169	0.026145	0.012846	-0.002464	0.015341	0.001643
	0.007551	-0.007381	-0.005570	0.001995	-0.004135	0.002394
	0.018701	0.014331	0.013338	-0.004003	0.009059	0.014039
	0.021791	-0.030170	-0.022835	0.010418	0.019290	0.001828
	-0.000827					
z26	0.033008	0.012599	0.024288	-0.008990	-0.009180	0.014352
	0.006545	0.005044	-0.008118	0.019009	0.014348	0.011703
	0.016750	0.018424	0.004727	0.007473	-0.023174	-0.006329
	0.023037	0.002765	-0.014600	0.004772	0.010521	-0.011195
	-0.016978	-0.004632	0.001477	-0.017028	0.013304	0.006727
	-0.002162					
x27	-0.013448	0.024398	0.019889	0.007184	0.015465	0.012712
	0.033933	-0.021136	-0.001540	-0.001746	0.006580	-0.007476
	-0.012295	-0.001801	0.000658	0.011555	0.012706	-0.000358
	-0.015318	0.010321	-0.004428	-0.004167	-0.008658	-0.004758
	0.009872	-0.018842	-0.005030	-0.015253	-0.007185	-0.043672
	-0.019259					
y27	-0.004670	-0.000143	-0.007067	0.010717	-0.002174	0.004080
	-0.011602	0.030387	0.013916	-0.001505	0.008854	0.005189
	0.005529	-0.007601	-0.002478	-0.002991	-0.001186	0.003850
	0.019484	0.018018	0.008610	-0.001196	0.012784	0.018022
	0.015771	-0.025367	-0.021860	0.000643	0.018045	0.000281
	0.014561					

z27	0.027117	0.013914	0.024959	-0.008013	-0.005049	0.014687
	0.007951	0.007851	-0.008000	0.015442	0.012548	0.009223
	0.016343	0.018572	0.010056	0.012317	-0.025428	-0.009116
	0.023338	0.006813	-0.011144	0.008919	0.011959	-0.007119
	-0.016072	-0.003647	-0.000131	-0.022698	0.021113	0.010657
	0.007258					
x28	-0.017104	0.022590	0.016051	0.008471	0.014338	0.008940
	0.034625	-0.019010	0.001389	-0.007046	0.007070	-0.007389
	-0.011114	-0.000326	-0.003587	0.007527	0.016791	0.002594
	-0.012702	0.012960	0.000230	0.000735	-0.010279	-0.004125
	0.022412	-0.016130	-0.009221	-0.017654	-0.009489	-0.036045
	-0.030771					
y28	-0.004376	-0.002128	-0.005969	0.011975	-0.001169	0.003057
	-0.007469	0.036396	0.012847	-0.000747	-0.000451	0.011221
	0.003693	-0.006708	0.003016	-0.008226	0.003180	0.008080
	0.017305	0.018173	-0.000889	0.001636	0.018693	0.017448
	0.009940	-0.021349	-0.022301	-0.011130	0.016629	-0.012440
	0.020642					
z28	0.021121	0.014347	0.025586	-0.006955	-0.001079	0.014888
	0.008559	0.011626	-0.007090	0.011039	0.010328	0.005523
	0.012404	0.016623	0.016170	0.013785	-0.026640	-0.012160
	0.019791	0.010296	-0.009254	0.012731	0.015197	-0.003104
	-0.016163	-0.005630	-0.000464	-0.030020	0.033126	0.013280
	0.014144					
x29	-0.019930	0.020725	0.011456	0.011157	0.011114	0.005815
	0.034263	-0.016656	0.006874	-0.017093	0.005664	-0.003512
	-0.008952	0.003084	-0.010494	-0.000261	0.017973	0.009647
	-0.011672	0.012567	0.005505	0.008408	-0.017376	-0.000251
	0.029684	-0.029834	-0.014459	-0.022261	-0.010156	-0.032068
	-0.041224					
y29	-0.004744	-0.002776	-0.003531	0.014425	0.000253	0.001566
	-0.004029	0.040879	0.019461	-0.005026	-0.003916	0.010582
	0.005798	-0.009543	0.005177	-0.015104	0.009923	0.003970
	0.007100	0.019633	-0.005717	0.003511	0.018324	0.012774
	0.009604	-0.017048	-0.013907	-0.013660	0.033683	-0.021579
	-0.008216					
z29	0.014955	0.014559	0.025882	-0.006250	0.002767	0.015370
	0.008431	0.016015	-0.006853	0.009416	0.008244	-0.001618
	0.004874	0.012703	0.021390	0.013042	-0.027004	-0.017390
	0.014693	0.013572	-0.002065	0.014763	0.020793	-0.002967
	-0.013582	0.002474	-0.000757	-0.033203	0.042342	0.016458
	0.015697					

x30	-0.020426	0.016957	0.009378	0.014469	0.006679	0.006416
	0.034847	-0.011828	0.005384	-0.023434	0.003523	0.003999
	0.000743	0.000701	-0.015529	-0.009163	0.020678	0.015141
	-0.017779	0.011481	0.017304	0.018085	-0.029330	0.007980
	0.024745	-0.038143	-0.018222	-0.027168	0.000483	-0.039935
	-0.037278					
y30	-0.003457	-0.003391	-0.001576	0.016086	0.000775	-0.000579
	-0.003958	0.040884	0.022565	-0.010551	-0.005023	0.010801
	0.005057	-0.006537	0.005605	-0.021228	0.012989	-0.003053
	-0.000362	0.020805	-0.012984	0.009240	0.021039	0.007173
	0.009852	-0.024007	-0.012896	-0.021921	0.030755	-0.029933
	-0.031802					
z30	0.010403	0.014814	0.025928	-0.007390	0.004925	0.013684
	0.006256	0.017115	-0.003206	0.007712	0.007352	-0.007665
	-0.003567	0.011914	0.022484	0.010427	-0.029484	-0.019488
	0.014987	0.012939	0.001799	0.013769	0.024428	-0.002151
	-0.008718	0.001665	-0.000251	-0.029906	0.034978	0.019166
	0.007853					
x31	-0.021434	0.014021	0.007080	0.015158	0.003565	0.004831
	0.035595	-0.007948	0.002999	-0.023179	0.005429	0.007394
	0.005233	0.000117	-0.014334	-0.010279	0.020768	0.018158
	-0.019530	0.004253	0.023972	0.022422	-0.031363	0.008264
	0.020894	-0.038210	-0.013132	-0.030268	0.008901	-0.040646
	-0.031370					
y31	-0.001194	-0.004029	-0.001375	0.012667	0.004079	-0.005267
	-0.006675	0.040071	0.022860	-0.008410	-0.002638	0.005600
	-0.000619	-0.004796	0.009029	-0.016955	0.006668	-0.001970
	-0.003973	0.017644	-0.013963	0.008306	0.019452	0.004620
	0.006310	-0.023225	-0.011675	-0.021150	0.019089	-0.022048
	-0.033878					
z31	0.006966	0.014278	0.025825	-0.009638	0.007036	0.011210
	0.002860	0.016202	0.001653	0.008452	0.006615	-0.014598
	-0.013713	0.010781	0.026234	0.008941	-0.034803	-0.019465
	0.014207	0.011261	0.008568	0.010123	0.022053	-0.004082
	-0.008392	0.001805	0.003304	-0.027138	0.025569	0.026274
	0.008143					
x32	-0.022902	0.011356	0.004886	0.014133	0.001711	0.002622
	0.036641	-0.005327	0.002140	-0.021293	0.009473	0.010242
	0.008618	-0.002676	-0.014871	-0.008506	0.017628	0.020128
	-0.024770	-0.003752	0.031817	0.024560	-0.031886	0.005309
	0.010880	-0.027028	-0.012600	-0.027322	0.023147	-0.035626
	-0.028079					

y32	0.000646	-0.005045	-0.002529	0.008509	0.007297	-0.009468
	-0.011219	0.037332	0.023991	-0.005623	-0.000991	0.001416
	-0.005926	-0.005018	0.008580	-0.013201	0.003140	-0.001939
	-0.007195	0.012912	-0.011416	0.004191	0.015744	0.003199
	0.002961	-0.018469	-0.011396	-0.015530	0.009799	-0.013046
	-0.032880					
z32	0.003022	0.013018	0.025479	-0.014298	0.008770	0.009156
	-0.000498	0.013027	0.008085	0.008893	0.006451	-0.019239
	-0.022019	0.006749	0.026080	0.008988	-0.038134	-0.020881
	0.015751	0.010379	0.017323	0.003760	0.016118	-0.006305
	-0.007404	0.008637	0.004143	-0.018144	0.018302	0.032847
	0.006625					
x33	-0.023604	0.009090	0.003180	0.013166	0.000359	-0.000495
	0.037185	-0.003332	0.000473	-0.017794	0.012941	0.011167
	0.011475	-0.005642	-0.012872	-0.007822	0.015263	0.021386
	-0.028033	-0.013312	0.037579	0.022464	-0.031018	0.004731
	0.002771	-0.012636	-0.013787	-0.028282	0.034277	-0.030798
	-0.021096					
y33	0.003457	-0.005610	-0.002936	0.003952	0.009826	-0.013526
	-0.015910	0.033984	0.023733	-0.003462	-0.000048	-0.003189
	-0.012162	-0.004152	0.009067	-0.010504	-0.001318	-0.004362
	-0.010571	0.009256	-0.008829	-0.001614	0.009746	0.002957
	-0.000074	-0.014687	-0.011715	-0.008476	0.005374	-0.006293
	-0.023576					
z33	0.000928	0.012342	0.026670	-0.018013	0.008828	0.006396
	-0.003274	0.008814	0.012007	0.010101	0.004704	-0.023277
	-0.031098	0.006898	0.027671	0.007344	-0.040207	-0.024267
	0.018823	0.009275	0.024186	-0.001511	0.007929	-0.011771
	-0.005746	0.012449	0.004797	-0.014049	0.007780	0.037570
	0.014218					
x34	-0.023589	0.007363	0.001482	0.012126	-0.001579	-0.003629
	0.037137	-0.000715	-0.001462	-0.012795	0.016339	0.010853
	0.012713	-0.007696	-0.006517	-0.006609	0.011992	0.021952
	-0.025892	-0.019340	0.040229	0.016720	-0.026621	0.010351
	-0.002297	0.001144	-0.014087	-0.030452	0.040249	-0.030243
	-0.012240					
y34	0.007135	-0.006068	-0.003239	-0.000436	0.010930	-0.016899
	-0.020007	0.031123	0.022795	-0.002016	0.000332	-0.006276
	-0.018073	-0.000964	0.009121	-0.008174	-0.006896	-0.006181
	-0.012388	0.009073	-0.006044	-0.005672	0.003368	0.005286
	-0.002840	-0.012719	-0.012147	0.002227	0.000900	-0.004754
	-0.011117					

z34	0.000405	0.011530	0.027935	-0.020061	0.006728	0.004008
	-0.002678	0.005251	0.013542	0.011117	0.004539	-0.024793
	-0.038020	0.007740	0.030840	0.004068	-0.039329	-0.026575
	0.024105	0.018079	0.028781	-0.006886	0.002868	-0.008991
	-0.004834	0.017103	0.001203	-0.005422	-0.002596	0.035056
	0.026693					
x35	-0.023355	0.005891	0.000534	0.012083	-0.002266	-0.006646
	0.035542	0.003268	-0.003161	-0.006281	0.017570	0.009443
	0.014287	-0.008164	-0.001164	-0.006445	0.009535	0.023312
	-0.022097	-0.029336	0.035298	0.008450	-0.018890	0.016802
	-0.006380	0.003440	-0.009083	-0.038035	0.038246	-0.026887
	-0.008718					
y35	0.011177	-0.006546	-0.003007	-0.003602	0.011942	-0.019589
	-0.023488	0.029116	0.021619	-0.000897	-0.000632	-0.008670
	-0.023058	0.003552	0.007196	-0.007645	-0.011639	-0.006220
	-0.014623	0.009768	-0.002909	-0.007968	-0.003774	0.007525
	-0.005109	-0.009153	-0.013011	0.010464	-0.007692	-0.006224
	-0.001859					
z35	0.000425	0.009706	0.030386	-0.018401	0.004456	0.002012
	-0.000415	0.003656	0.014060	0.010968	0.001935	-0.023073
	-0.038763	0.008192	0.027682	-0.002956	-0.033065	-0.026872
	0.025578	0.024708	0.023152	-0.011059	-0.005174	-0.001955
	-0.009276	0.008114	0.003356	-0.008772	-0.014867	0.031564
	0.028532					
x36	-0.022691	0.004624	-0.000290	0.012102	-0.000938	-0.008974
	0.032706	0.008297	-0.004479	0.000772	0.017237	0.006666
	0.014271	-0.005703	0.004198	-0.006425	0.005012	0.024540
	-0.018634	-0.042079	0.030057	-0.000161	-0.007476	0.017555
	-0.007279	0.002273	-0.001998	-0.043968	0.033674	-0.019127
	-0.009722					
y36	0.016186	-0.006536	-0.002489	-0.005182	0.012487	-0.021964
	-0.025812	0.026345	0.019069	0.000478	-0.002633	-0.010723
	-0.026516	0.008091	0.005747	-0.009580	-0.015352	-0.006158
	-0.018402	0.009941	0.000912	-0.008692	-0.008945	0.010096
	-0.005759	-0.002351	-0.017041	0.017245	-0.016487	-0.007934
	0.007151					
z36	0.001769	0.007987	0.033741	-0.014328	0.001929	0.002943
	0.004481	0.002244	0.011555	0.008533	-0.001827	-0.018628
	-0.033224	0.011609	0.022572	-0.010257	-0.026585	-0.024924
	0.025249	0.025867	0.018543	-0.011753	-0.010062	0.004817
	-0.013707	-0.002611	-0.001343	-0.013710	-0.020046	0.031234
	0.025817					

x37	-0.022293	0.002708	-0.002164	0.010842	0.002655	-0.010197
	0.028552	0.013390	-0.004379	0.005740	0.018264	0.002595
	0.010421	-0.003179	0.008768	-0.001781	-0.002727	0.023478
	-0.018453	-0.052408	0.023583	-0.007794	0.007439	0.010746
	-0.006605	0.004946	0.008103	-0.043510	0.035053	-0.008588
	-0.019958					
y37	0.021987	-0.006240	-0.002127	-0.005879	0.013458	-0.023977
	-0.027694	0.023089	0.016118	0.001574	-0.005452	-0.011944
	-0.028663	0.010817	0.004753	-0.013378	-0.016388	-0.004882
	-0.023275	0.010369	0.004685	-0.009283	-0.011760	0.014740
	-0.004819	0.006619	-0.019942	0.023007	-0.020656	-0.009494
	0.013169					
z37	0.002160	0.002934	0.034499	-0.010293	0.000608	0.006764
	0.008952	0.000680	0.007573	0.001902	-0.007383	-0.009021
	-0.022099	0.013596	0.012640	-0.011696	-0.015324	-0.024972
	0.024514	0.027221	0.008332	-0.006103	-0.007731	0.009405
	-0.013078	-0.012102	-0.004524	-0.016481	-0.012197	0.028437
	0.008111					
x38	-0.021563	0.002161	-0.003031	0.011516	0.007510	-0.011906
	0.022306	0.019552	-0.005143	0.011092	0.016464	-0.002671
	0.008548	0.001008	0.018904	0.003522	-0.012512	0.019559
	-0.019600	-0.061949	0.011636	-0.018099	0.019942	-0.002902
	-0.009091	-0.001768	0.024495	-0.042637	0.030758	0.009109
	-0.036628					
y38	0.027988	-0.004561	-0.001058	-0.005805	0.013758	-0.025206
	-0.027703	0.021601	0.014319	0.002317	-0.007317	-0.011400
	-0.030019	0.012970	0.002626	-0.019102	-0.017390	-0.001878
	-0.026211	0.011804	0.013178	-0.006728	-0.011606	0.020627
	-0.001766	0.017239	-0.024437	0.027586	-0.020780	-0.015659
	0.019369					
z38	0.005110	-0.003180	0.033209	-0.006141	0.000650	0.008004
	0.011126	-0.002198	0.004799	-0.005728	-0.013934	-0.002913
	-0.014504	0.009209	0.006590	-0.013477	-0.002282	-0.026762
	0.023061	0.034426	0.000626	0.004472	-0.007889	0.002250
	-0.009472	-0.017791	-0.002412	-0.020581	-0.011238	0.019177
	-0.004461					
x39	-0.022283	0.002601	-0.004022	0.010333	0.008011	-0.013688
	0.020574	0.020829	-0.012560	0.008299	0.012044	-0.007217
	0.007362	0.003887	0.018679	-0.001517	-0.014313	0.016768
	-0.016336	-0.057871	0.011192	-0.020076	0.012486	-0.007170
	-0.013502	-0.002336	0.025711	-0.040656	0.021367	0.020225
	-0.045392					

y39	0.031215	-0.003654	-0.000191	-0.005349	0.014242	-0.024861
	-0.026246	0.023549	0.017080	0.000743	-0.004937	-0.010968
	-0.032297	0.015698	0.005405	-0.018828	-0.021389	0.000713
	-0.026831	0.012995	0.016220	-0.009043	-0.007477	0.020259
	0.002537	0.015650	-0.023275	0.031141	-0.020397	-0.021683
	0.019921					
z39	0.006113	-0.005029	0.032890	-0.005681	0.000606	0.007405
	0.010065	-0.004380	0.003740	-0.002231	-0.018612	-0.001721
	-0.008038	0.002603	0.001318	-0.008903	0.006688	-0.029879
	0.018184	0.032004	-0.002357	0.012870	-0.004261	-0.002057
	-0.010534	-0.017163	-0.000778	-0.017570	-0.007949	0.018171
	-0.004731					
x40	-0.021893	0.003395	-0.004511	0.008693	0.008663	-0.016691
	0.020431	0.022440	-0.018709	0.004687	0.010679	-0.012065
	0.005200	0.002877	0.016858	-0.007001	-0.017170	0.016813
	-0.010196	-0.051233	0.015148	-0.016486	0.009464	-0.011930
	-0.015018	-0.000332	0.026017	-0.039922	0.017505	0.022863
	-0.047911					
y40	0.033875	-0.002467	0.000402	-0.004604	0.014309	-0.023275
	-0.026409	0.025119	0.018532	0.003705	-0.005703	-0.010010
	-0.029754	0.016537	0.006561	-0.018958	-0.018632	0.001920
	-0.031347	0.008892	0.016709	-0.008872	-0.005405	0.021857
	0.008151	0.012235	-0.022567	0.032246	-0.021156	-0.024961
	0.019364					
z40	0.008371	-0.006288	0.033474	-0.005185	-0.000128	0.006199
	0.008691	-0.006616	0.003209	0.001227	-0.023018	-0.000775
	-0.006261	0.001308	-0.003338	-0.006669	0.010850	-0.033649
	0.014775	0.031237	-0.000918	0.015288	-0.004550	-0.006733
	-0.015282	-0.016917	0.003388	-0.011981	-0.006058	0.017419
	-0.009954					
x41	-0.021087	0.004162	-0.004953	0.007550	0.009425	-0.019566
	0.019713	0.023711	-0.023766	0.001958	0.009247	-0.015157
	0.001659	0.001038	0.014658	-0.011673	-0.019038	0.015759
	-0.005399	-0.044182	0.019219	-0.012042	0.007635	-0.016136
	-0.015852	0.001391	0.026912	-0.040672	0.014168	0.021431
	-0.048423					
y41	0.036528	-0.001089	0.000745	-0.004208	0.014280	-0.021340
	-0.025747	0.026009	0.019041	0.005480	-0.008407	-0.009869
	-0.025725	0.018103	0.005642	-0.018700	-0.014841	0.000586
	-0.035107	0.006063	0.017646	-0.008375	-0.003219	0.020355
	0.011082	0.006780	-0.017440	0.032743	-0.020239	-0.027747
	0.012910					

z41	0.010705	-0.007425	0.034259	-0.005029	-0.000847	0.004705
	0.006522	-0.008273	0.002881	0.005900	-0.025876	-0.000598
	-0.006086	-0.001732	-0.005185	-0.005710	0.014243	-0.034745
	0.012548	0.027032	0.001041	0.016993	-0.004049	-0.006802
	-0.017337	-0.013602	0.001856	-0.004648	-0.005156	0.018015
	-0.009408					
x42	-0.019730	0.004816	-0.005629	0.007333	0.010561	-0.021595
	0.019205	0.025288	-0.026759	0.000427	0.008944	-0.017946
	-0.001061	0.000734	0.012643	-0.015299	-0.019925	0.013785
	-0.001227	-0.037358	0.022012	-0.009300	0.002390	-0.017964
	-0.015780	0.002659	0.023821	-0.036324	0.011149	0.024126
	-0.049751					
y42	0.039727	0.000756	0.001447	-0.003883	0.013683	-0.019555
	-0.024853	0.026413	0.019056	0.007720	-0.011393	-0.010142
	-0.020206	0.018362	0.003488	-0.019325	-0.012620	-0.000796
	-0.038284	0.003545	0.019285	-0.006056	0.000356	0.017604
	0.013481	0.003478	-0.013401	0.033928	-0.017374	-0.028687
	0.006729					
z42	0.011851	-0.009149	0.034749	-0.005582	-0.001017	0.002522
	0.003872	-0.009647	0.001271	0.008106	-0.028547	-0.000976
	-0.007734	-0.005328	-0.004821	-0.004261	0.018045	-0.035125
	0.011698	0.021876	0.004370	0.017429	-0.002718	-0.007651
	-0.019899	-0.012505	0.002257	-0.002536	-0.006316	0.013927
	-0.006511					
x43	-0.018194	0.004978	-0.006126	0.007578	0.011667	-0.023251
	0.018513	0.026325	-0.028108	-0.000737	0.008742	-0.019355
	-0.004051	0.002499	0.010369	-0.017822	-0.020597	0.009043
	0.001303	-0.033046	0.023416	-0.009229	-0.001568	-0.018887
	-0.016558	0.003418	0.019661	-0.030878	0.004964	0.025466
	-0.049412					
y43	0.043157	0.002810	0.002179	-0.003936	0.012938	-0.018120
	-0.024066	0.026284	0.017316	0.010703	-0.015387	-0.011434
	-0.014324	0.017489	0.002374	-0.020596	-0.009019	-0.002166
	-0.039756	0.001230	0.021907	-0.002383	0.004939	0.013628
	0.015389	0.000939	-0.008779	0.033222	-0.015516	-0.028515
	0.002035					
z43	0.012154	-0.011327	0.034987	-0.006123	-0.000982	0.000489
	0.001973	-0.009956	0.001022	0.007369	-0.029655	-0.000103
	-0.009645	-0.009476	-0.004947	-0.002206	0.018942	-0.032574
	0.010837	0.018343	0.007800	0.017490	-0.003249	-0.007344
	-0.021713	-0.010934	0.001674	-0.000135	-0.003977	0.010118
	-0.004403					

x44	-0.016739	0.004451	-0.006423	0.007811	0.013028	-0.024599
	0.017484	0.026320	-0.028048	-0.001054	0.008565	-0.019046
	-0.007853	0.004975	0.007141	-0.019701	-0.021848	0.001063
	0.002337	-0.032074	0.024627	-0.011477	-0.003718	-0.017652
	-0.017980	0.005021	0.014745	-0.024445	-0.004894	0.023005
	-0.044235					
y44	0.046524	0.005118	0.002883	-0.004505	0.012094	-0.016736
	-0.023356	0.026079	0.014615	0.012987	-0.019150	-0.013720
	-0.008459	0.015312	0.001880	-0.022199	-0.005098	-0.002118
	-0.038639	-0.000433	0.026130	0.002176	0.007582	0.009078
	0.018583	-0.000488	-0.004544	0.031745	-0.013082	-0.026521
	-0.004525					
z44	0.012338	-0.013466	0.034819	-0.006097	-0.000863	-0.002025
	0.000923	-0.008690	0.001344	0.007182	-0.030967	0.000113
	-0.010028	-0.011422	-0.003746	-0.000276	0.019539	-0.029582
	0.008131	0.015936	0.011286	0.016705	-0.004031	-0.005365
	-0.022184	-0.010667	0.001887	0.001933	0.002329	0.010194
	-0.001486					
x45	-0.015079	0.003620	-0.006573	0.008443	0.014257	-0.025377
	0.016571	0.026059	-0.026836	-0.000575	0.008096	-0.017704
	-0.012160	0.007351	0.004094	-0.019912	-0.022683	-0.008569
	0.001996	-0.033528	0.026793	-0.014528	-0.003965	-0.015712
	-0.018654	0.005832	0.008656	-0.016031	-0.015351	0.017601
	-0.037363					
y45	0.049587	0.007532	0.003428	-0.005226	0.011029	-0.015410
	-0.022837	0.026572	0.011520	0.015093	-0.023443	-0.016350
	-0.002215	0.013288	0.001556	-0.023543	-0.001437	-0.000976
	-0.036547	-0.000799	0.031774	0.007440	0.008903	0.003338
	0.021780	-0.000182	0.000344	0.029709	-0.008662	-0.022536
	-0.010847					
z45	0.012402	-0.015336	0.034490	-0.006727	-0.000190	-0.005461
	0.000047	-0.007987	0.001848	0.006824	-0.029222	-0.002260
	-0.011230	-0.012912	-0.001481	-0.001242	0.021364	-0.025485
	0.005370	0.013167	0.014196	0.015344	-0.007746	0.000147
	-0.020860	-0.011879	-0.000457	0.001933	0.006995	0.011539
	-0.003049					
x46	-0.013384	0.003301	-0.006087	0.009886	0.015067	-0.024893
	0.015944	0.026527	-0.025161	0.000858	0.007544	-0.015694
	-0.016045	0.008528	0.001650	-0.018816	-0.022435	-0.016663
	0.000764	-0.036017	0.030405	-0.015973	-0.003837	-0.010890
	-0.018758	0.004676	0.003151	-0.005267	-0.023556	0.011824
	-0.029863					

y46	0.052381	0.009777	0.003708	-0.006197	0.009840	-0.014848
	-0.022402	0.027107	0.007931	0.016983	-0.028095	-0.018360
	0.002856	0.011147	-0.000421	-0.026002	0.000518	-0.000626
	-0.035157	-0.000426	0.036573	0.012889	0.009934	-0.003679
	0.023398	0.001108	0.005253	0.027410	-0.004766	-0.019142
	-0.016609					
z46	0.012905	-0.017121	0.034147	-0.007652	-0.000228	-0.008607
	-0.000918	-0.007593	0.003078	0.007549	-0.026895	-0.004921
	-0.011707	-0.013869	0.000902	-0.002252	0.024626	-0.022559
	0.005621	0.009689	0.019459	0.012098	-0.008511	0.005916
	-0.018399	-0.011106	-0.001806	0.001141	0.009818	0.007079
	-0.004970					
x47	-0.018671	-0.002700	-0.014305	0.002638	0.022223	-0.031316
	0.004275	0.035707	-0.007163	-0.009789	0.008107	-0.007612
	-0.040287	0.014931	0.018327	-0.031018	-0.065478	-0.030085
	0.005004	-0.046412	0.011313	-0.040708	-0.009908	-0.001362
	-0.013439	0.021150	0.008694	0.008240	-0.011389	0.029171
	-0.030250					
y47	0.054794	0.015356	0.001966	-0.015619	0.016197	-0.019404
	-0.027456	0.029784	-0.007754	0.010481	-0.027398	-0.027988
	0.000013	-0.019495	0.033182	-0.022210	-0.002889	0.009265
	-0.015981	0.001568	0.017372	0.012177	0.011843	0.009968
	0.013119	-0.011179	0.009895	0.013142	-0.001061	0.038135
	-0.021171					
z47	0.015385	-0.028760	0.035561	-0.010034	0.001108	-0.025370
	0.004165	-0.018032	-0.006491	0.013748	-0.035794	-0.018343
	-0.027055	-0.018909	0.024878	-0.006963	0.014959	-0.007168
	-0.035604	0.023028	0.001716	0.053358	-0.007923	-0.005235
	-0.017250	0.029575	-0.036073	-0.015714	-0.034870	-0.003583
	0.001671					
x48	-0.018387	-0.001689	-0.012512	0.004695	0.021494	-0.030143
	0.004814	0.035225	-0.009038	-0.008001	0.009563	-0.010779
	-0.042968	0.015103	0.015125	-0.031759	-0.061883	-0.027310
	0.004308	-0.043155	0.006034	-0.037714	-0.008454	0.000444
	-0.016386	0.018514	0.007448	0.006990	-0.012939	0.032501
	-0.024377					
y48	0.054728	0.015909	0.002296	-0.013575	0.014247	-0.017998
	-0.027055	0.030417	-0.006604	0.012322	-0.028264	-0.026675
	0.001406	-0.018030	0.030062	-0.022360	-0.002822	0.011153
	-0.015194	0.002287	0.016649	0.013506	0.011293	0.005174
	0.013849	-0.013165	0.009392	0.011613	-0.001255	0.036800
	-0.013601					

z48	0.016306	-0.028100	0.034497	-0.010133	0.000627	-0.024324
	0.003142	-0.016375	-0.005600	0.015046	-0.036536	-0.016301
	-0.023670	-0.019497	0.023686	-0.005197	0.017229	-0.007967
	-0.032213	0.021269	0.006644	0.049744	-0.006407	-0.007433
	-0.016294	0.025176	-0.035732	-0.016174	-0.031297	-0.002663
	0.001740					
x49	-0.017943	-0.000657	-0.010569	0.006540	0.020729	-0.028946
	0.005224	0.034374	-0.010536	-0.006563	0.010616	-0.013161
	-0.045171	0.015058	0.012713	-0.031916	-0.058794	-0.023904
	0.003290	-0.039333	0.001624	-0.035250	-0.008038	0.002258
	-0.018788	0.016203	0.006303	0.006259	-0.013592	0.035269
	-0.019983					
y49	0.054732	0.016530	0.002509	-0.012379	0.012411	-0.015590
	-0.025568	0.031383	-0.006279	0.015252	-0.027712	-0.025015
	0.005440	-0.016397	0.026054	-0.021856	-0.000292	0.008434
	-0.010068	0.003271	0.018842	0.014695	0.012235	0.001207
	0.016464	-0.015379	0.007117	0.010758	0.003160	0.032651
	-0.016346					
z49	0.016936	-0.026707	0.033179	-0.010231	0.000584	-0.022795
	0.002477	-0.014416	-0.005057	0.016703	-0.036500	-0.015431
	-0.019844	-0.019811	0.023543	-0.003442	0.018988	-0.007553
	-0.027246	0.018451	0.011303	0.046621	-0.006409	-0.006629
	-0.015164	0.021189	-0.035927	-0.015247	-0.024561	0.001378
	0.000682					
x50	-0.017495	0.000486	-0.008739	0.008501	0.020038	-0.027206
	0.005171	0.034882	-0.012131	-0.004913	0.010138	-0.014327
	-0.046602	0.015760	0.010880	-0.031877	-0.057097	-0.021373
	0.003439	-0.036617	-0.001831	-0.033270	-0.005392	0.003828
	-0.022135	0.015088	0.003565	0.005968	-0.013349	0.038864
	-0.013361					
y50	0.054788	0.018453	0.003114	-0.011501	0.011309	-0.014041
	-0.026922	0.032195	-0.005729	0.016470	-0.029136	-0.022636
	0.006094	-0.015846	0.020508	-0.023484	0.003397	0.013521
	-0.012671	0.008349	0.018548	0.013264	0.011254	0.001998
	0.015785	-0.016822	0.009877	0.006478	0.004274	0.026944
	-0.011529					
z50	0.018057	-0.025199	0.032038	-0.010294	0.000556	-0.021850
	0.001226	-0.013642	-0.004562	0.018095	-0.035846	-0.014902
	-0.017802	-0.021032	0.021695	-0.002795	0.021795	-0.006294
	-0.024755	0.016691	0.013472	0.041843	-0.005285	-0.003780
	-0.014550	0.015722	-0.031512	-0.014883	-0.020654	0.000340
	0.000181					

x51	-0.016776	0.001903	-0.006829	0.009976	0.019511	-0.025356
	0.005203	0.034771	-0.013738	-0.003674	0.009677	-0.014473
	-0.047414	0.016178	0.009123	-0.031091	-0.054649	-0.018845
	0.003362	-0.033728	-0.004006	-0.031121	-0.003475	0.005110
	-0.025079	0.013716	0.000813	0.004722	-0.014941	0.039563
	-0.008527					
y51	0.054801	0.019031	0.003893	-0.010060	0.009172	-0.012228
	-0.027608	0.032152	-0.005179	0.019034	-0.029818	-0.019061
	0.007992	-0.014654	0.015443	-0.024530	0.004949	0.017713
	-0.011733	0.012931	0.020518	0.014712	0.009869	0.003818
	0.017493	-0.022367	0.008663	0.009060	0.005369	0.017892
	-0.011265					
z51	0.019340	-0.024138	0.030989	-0.009879	0.000074	-0.020905
	-0.000034	-0.012948	-0.004239	0.020453	-0.034785	-0.014880
	-0.015277	-0.021461	0.020076	-0.002591	0.023297	-0.005566
	-0.020701	0.014940	0.015171	0.037873	-0.004029	-0.000154
	-0.012442	0.009632	-0.027809	-0.011424	-0.015836	-0.000697
	0.000090					
x52	-0.015967	0.003276	-0.004579	0.011243	0.018891	-0.023058
	0.005107	0.035185	-0.014269	-0.002437	0.008902	-0.014166
	-0.047086	0.016470	0.008185	-0.030038	-0.051591	-0.016003
	0.002576	-0.030251	-0.005220	-0.028462	-0.002542	0.006809
	-0.025597	0.013384	-0.000508	0.003575	-0.014427	0.040733
	-0.002877					
y52	0.055003	0.019820	0.004215	-0.008590	0.006977	-0.010305
	-0.026623	0.032246	-0.003495	0.017870	-0.028400	-0.016419
	0.007922	-0.013440	0.013609	-0.024581	0.004984	0.020730
	-0.010721	0.013148	0.020504	0.015968	0.009380	0.007387
	0.021437	-0.022606	0.009905	0.007644	0.008115	0.014393
	-0.007192					
z52	0.020992	-0.022501	0.029702	-0.009402	-0.000484	-0.019722
	-0.000384	-0.012044	-0.003821	0.021552	-0.033015	-0.015308
	-0.013706	-0.021712	0.019746	-0.001226	0.023858	-0.004740
	-0.015517	0.011665	0.016820	0.034353	-0.003362	0.003911
	-0.010478	0.004480	-0.023507	-0.009844	-0.008430	-0.000865
	0.000325					
x53	-0.014903	0.004744	-0.002111	0.012041	0.018501	-0.020987
	0.005354	0.034970	-0.015041	-0.001362	0.007322	-0.013461
	-0.046710	0.016242	0.006962	-0.028875	-0.049155	-0.013785
	0.001918	-0.026857	-0.006297	-0.025791	-0.001201	0.007043
	-0.026440	0.012398	-0.002658	0.003754	-0.013221	0.038594
	0.002447					

y53	0.055154	0.021200	0.005024	-0.007655	0.005279	-0.007743
	-0.025703	0.030994	-0.003332	0.020570	-0.029184	-0.014163
	0.006524	-0.013066	0.008540	-0.025876	0.005693	0.020833
	-0.010652	0.016127	0.020827	0.017349	0.008093	0.009821
	0.026505	-0.024409	0.010919	0.008726	0.013338	0.006970
	-0.007846					
z53	0.022894	-0.020755	0.028777	-0.008973	-0.001297	-0.018092
	-0.000468	-0.011696	-0.003905	0.024168	-0.032544	-0.015239
	-0.012858	-0.022302	0.017699	-0.001117	0.023294	-0.005142
	-0.012024	0.009297	0.017672	0.030598	-0.001625	0.007353
	-0.008486	0.000224	-0.018303	-0.008025	-0.001233	-0.002285
	-0.001046					
x54	-0.013510	0.006604	0.000606	0.012755	0.018060	-0.018558
	0.005838	0.034832	-0.015201	-0.000601	0.006018	-0.013184
	-0.046028	0.016153	0.006039	-0.027285	-0.045541	-0.009641
	0.001861	-0.023280	-0.006678	-0.022966	-0.001588	0.008105
	-0.024079	0.011332	-0.004334	0.004690	-0.013384	0.037263
	0.007186					
y54	0.055229	0.021780	0.005774	-0.006735	0.004204	-0.004900
	-0.024008	0.031520	-0.003232	0.023400	-0.028602	-0.012309
	0.008693	-0.011974	0.006760	-0.025139	0.007688	0.020821
	-0.006530	0.016521	0.022310	0.020316	0.008067	0.012608
	0.034486	-0.024982	0.009714	0.008034	0.017576	0.000867
	-0.008039					
z54	0.025237	-0.019075	0.027991	-0.008542	-0.001510	-0.016345
	-0.000187	-0.010240	-0.004365	0.027163	-0.031745	-0.015638
	-0.009584	-0.021413	0.018345	-0.000291	0.023150	-0.004066
	-0.006089	0.006635	0.018558	0.028721	-0.000592	0.010300
	-0.004813	-0.002711	-0.015450	-0.007606	0.005864	-0.001103
	-0.001048					
x55	-0.012121	0.008301	0.002942	0.012941	0.017755	-0.016416
	0.006113	0.033969	-0.015636	-0.000009	0.003312	-0.012131
	-0.045575	0.014978	0.005402	-0.025552	-0.042732	-0.006036
	-0.000863	-0.020197	-0.006528	-0.019695	0.000405	0.009976
	-0.024109	0.012020	-0.004378	0.005336	-0.013254	0.033217
	0.013724					
y55	0.053950	0.022337	0.006612	-0.005858	0.003515	-0.003374
	-0.023282	0.032462	-0.000450	0.024470	-0.028110	-0.008333
	0.009899	-0.010326	-0.000866	-0.024957	0.003733	0.017102
	-0.004265	0.014084	0.023660	0.015093	0.012509	0.014483
	0.039122	-0.025858	0.011529	0.006184	0.019049	-0.007427
	-0.006586					

z55	0.026789	-0.016461	0.027684	-0.007559	-0.002529	-0.015576
	-0.001871	-0.009510	-0.001622	0.028218	-0.031181	-0.014895
	-0.006303	-0.021119	0.011612	-0.001307	0.016714	-0.006645
	-0.002454	0.002329	0.019664	0.021286	0.003831	0.010319
	0.000591	-0.003530	-0.010165	-0.006840	0.010513	-0.006705
	-0.003706					
x56	-0.010317	0.010352	0.006008	0.013019	0.017679	-0.014569
	0.006359	0.032469	-0.015711	0.002112	0.002325	-0.010893
	-0.044765	0.012372	0.004726	-0.024837	-0.040026	-0.004611
	-0.004509	-0.015976	-0.007748	-0.018600	0.000553	0.009263
	-0.020282	0.015181	-0.005412	0.007168	-0.011019	0.026930
	0.016925					
y56	0.053796	0.023038	0.006291	-0.005417	0.002134	-0.002120
	-0.020609	0.032877	-0.000289	0.023252	-0.028472	-0.008696
	0.006601	-0.010994	-0.003134	-0.024190	0.007505	0.018394
	-0.003003	0.015028	0.024881	0.015605	0.014746	0.011262
	0.036339	-0.028709	0.013679	0.011141	0.020196	-0.009322
	-0.002696					
z56	0.030018	-0.014499	0.026879	-0.007150	-0.003621	-0.014514
	-0.001087	-0.007828	-0.001354	0.029435	-0.031884	-0.015360
	-0.005785	-0.019698	0.011287	-0.000160	0.014999	-0.005257
	0.001117	0.001119	0.019961	0.018985	0.009072	0.007493
	-0.002024	-0.006343	-0.003807	-0.004588	0.016070	-0.007934
	-0.002313					
x57	-0.009107	0.012361	0.008416	0.012756	0.017598	-0.012719
	0.006903	0.031501	-0.016467	0.004911	0.003361	-0.010810
	-0.043593	0.010048	0.005371	-0.025041	-0.036834	-0.005167
	-0.001520	-0.012731	-0.008255	-0.017174	0.000639	0.004557
	-0.015811	0.015391	-0.002872	0.008098	-0.010114	0.025607
	0.018679					
y57	0.052230	0.023714	0.005627	-0.004892	0.001467	-0.000477
	-0.020593	0.033085	0.000766	0.024040	-0.025624	-0.005991
	0.006455	-0.013598	-0.001894	-0.023948	0.007151	0.023779
	-0.002743	0.012845	0.021800	0.018168	0.016451	0.008391
	0.036137	-0.027125	0.008390	0.009927	0.017325	-0.002716
	0.000823					
z57	0.032456	-0.012336	0.026177	-0.007001	-0.004423	-0.013185
	-0.001833	-0.006619	-0.000520	0.030892	-0.030775	-0.013990
	-0.002953	-0.019426	0.012634	-0.000728	0.011185	0.001450
	0.001337	-0.001317	0.018155	0.018462	0.011204	0.006137
	-0.003565	-0.006262	-0.003650	-0.007530	0.019108	-0.004846
	-0.000578					

x58	-0.008124	0.014605	0.010717	0.012397	0.017355	-0.010476
	0.007861	0.031041	-0.017025	0.006537	0.003251	-0.010235
	-0.043789	0.008800	0.006116	-0.023200	-0.034612	-0.003578
	0.000945	-0.009875	-0.008012	-0.014289	0.000761	0.001524
	-0.014725	0.013663	-0.003170	0.010175	-0.005178	0.024991
	0.022401					
y58	0.049774	0.024351	0.004853	-0.004477	0.001565	0.001644
	-0.020844	0.033978	0.002032	0.024338	-0.024842	-0.003805
	0.009987	-0.012769	-0.003856	-0.022127	0.003921	0.024068
	-0.001767	0.013032	0.022673	0.017235	0.015668	0.008337
	0.037757	-0.021783	0.002087	0.008014	0.020372	-0.005008
	0.001223					
z58	0.034365	-0.010135	0.025638	-0.006510	-0.004755	-0.011848
	-0.003335	-0.005220	0.000848	0.032467	-0.031260	-0.012558
	0.002496	-0.017425	0.011374	-0.000360	0.005083	0.002533
	0.002970	-0.003027	0.018924	0.015343	0.013258	0.005510
	-0.002471	-0.001627	-0.004494	-0.009720	0.025287	-0.007439
	-0.001945					
x59	-0.006253	0.016922	0.012272	0.011876	0.016674	-0.008641
	0.009606	0.028970	-0.017701	0.007501	0.003488	-0.010938
	-0.042989	0.006492	0.006359	-0.021633	-0.031141	-0.001330
	0.000433	-0.007440	-0.007555	-0.011721	0.000167	-0.000147
	-0.012593	0.008422	0.001408	0.011706	-0.001735	0.024955
	0.021082					
y59	0.048581	0.024702	0.005199	-0.003705	0.001502	0.003063
	-0.020320	0.034056	0.004932	0.023987	-0.022783	-0.000561
	0.009724	-0.013641	-0.007085	-0.019903	0.005293	0.026721
	-0.002386	0.013997	0.021640	0.013209	0.013645	0.010793
	0.038254	-0.026742	0.000445	0.007258	0.020560	-0.002282
	-0.000087					
z59	0.037227	-0.008211	0.026447	-0.005920	-0.004987	-0.011169
	-0.004450	-0.004132	0.003324	0.034119	-0.030695	-0.009401
	0.004927	-0.016426	0.010419	0.000442	0.002302	0.006165
	0.002652	-0.003739	0.016862	0.009789	0.013820	0.005969
	-0.001308	-0.002827	-0.002132	-0.011309	0.029272	-0.007276
	-0.003977					
x60	-0.004615	0.018959	0.014505	0.011423	0.016255	-0.007363
	0.011043	0.026777	-0.018253	0.009362	0.003641	-0.010608
	-0.042124	0.003442	0.005699	-0.018537	-0.029445	-0.000132
	-0.000735	-0.004364	-0.007781	-0.010169	0.000323	-0.001190
	-0.012461	0.006531	0.005103	0.012992	-0.000425	0.022000
	0.020194					

y60	0.046351	0.024612	0.005218	-0.003629	0.000568	0.003970
	-0.020886	0.034338	0.005480	0.022270	-0.021002	0.001291
	0.009689	-0.016170	-0.010220	-0.017338	0.005275	0.025943
	-0.003833	0.016257	0.021216	0.008453	0.012729	0.011139
	0.031201	-0.026234	-0.000551	0.003132	0.016413	-0.003914
	0.001302					
z60	0.039482	-0.006534	0.026660	-0.006162	-0.006315	-0.010566
	-0.005929	-0.003000	0.004070	0.033988	-0.030609	-0.007909
	0.006472	-0.016981	0.009362	0.000756	-0.001579	0.007399
	0.001871	-0.003053	0.015963	0.004462	0.015561	0.004791
	-0.005933	-0.000328	-0.000390	-0.015312	0.029233	-0.010295
	-0.002366					
x61	-0.003416	0.020908	0.016373	0.010889	0.016991	-0.005977
	0.012528	0.024350	-0.018078	0.011246	0.003399	-0.011191
	-0.041325	0.001069	0.005386	-0.015021	-0.027848	0.002184
	-0.003272	-0.001702	-0.008587	-0.006214	-0.000281	-0.000993
	-0.010887	0.005892	0.003079	0.014188	0.003250	0.020848
	0.018759					
y61	0.043818	0.023716	0.003921	-0.003123	0.001188	0.005223
	-0.020062	0.034063	0.006401	0.021606	-0.018997	0.000546
	0.012058	-0.017033	-0.011939	-0.015620	0.002728	0.021753
	-0.001481	0.017259	0.023232	0.007722	0.014122	0.008591
	0.034533	-0.024064	-0.007230	0.002375	0.016421	-0.007293
	0.000573					
z61	0.041094	-0.005322	0.025839	-0.005674	-0.006567	-0.009520
	-0.006712	-0.002194	0.005266	0.033822	-0.030532	-0.008547
	0.011142	-0.016454	0.008204	-0.000044	-0.007802	0.006039
	0.005633	-0.003737	0.017362	0.002207	0.018471	0.001094
	-0.002812	0.002797	-0.003734	-0.015864	0.033102	-0.014787
	-0.001910					
x62	-0.002269	0.022870	0.018172	0.009890	0.017268	-0.004606
	0.014571	0.021562	-0.017749	0.010972	0.002925	-0.011921
	-0.041370	-0.000756	0.004440	-0.012591	-0.025003	0.004452
	-0.005500	0.001571	-0.009228	-0.001644	-0.000514	-0.001955
	-0.010505	0.003323	0.002065	0.015650	0.006078	0.020166
	0.019662					
y62	0.041163	0.022739	0.002843	-0.002894	0.000897	0.007136
	-0.019257	0.034464	0.006213	0.021481	-0.017730	0.002571
	0.013051	-0.016034	-0.013027	-0.011247	-0.000394	0.021435
	-0.000292	0.018089	0.023787	0.006168	0.014149	0.006369
	0.039897	-0.022985	-0.009552	0.002147	0.011944	-0.012124
	0.004499					

z62	0.042887	-0.004517	0.025601	-0.005368	-0.007196	-0.007926
	-0.007491	-0.000397	0.004918	0.036140	-0.030656	-0.005093
	0.013874	-0.013732	0.008590	0.001670	-0.014122	0.008721
	0.007881	-0.004030	0.016303	-0.001213	0.020295	-0.002292
	0.003981	0.005700	-0.003585	-0.017814	0.031608	-0.021150
	0.003459					
x63	-0.000773	0.024744	0.020412	0.009626	0.017390	-0.003865
	0.016431	0.019136	-0.017129	0.012437	0.002369	-0.011905
	-0.040423	-0.002606	0.003035	-0.010637	-0.022867	0.005742
	-0.008213	0.004742	-0.011016	-0.000019	-0.001606	-0.001108
	-0.009868	0.000518	0.003323	0.017168	0.011014	0.019589
	0.015208					
y63	0.039360	0.022451	0.002246	-0.001774	-0.000017	0.007648
	-0.019204	0.035351	0.006582	0.019690	-0.017695	0.003431
	0.013532	-0.015348	-0.013628	-0.010836	-0.001770	0.021928
	0.001708	0.017899	0.025674	0.002429	0.015068	0.007603
	0.035511	-0.024739	-0.011235	0.005012	0.009862	-0.012031
	0.008994					
z63	0.045353	-0.002946	0.025492	-0.004205	-0.008480	-0.007687
	-0.008743	0.001615	0.005043	0.035793	-0.032284	-0.003684
	0.016551	-0.011051	0.010291	-0.001355	-0.018125	0.012110
	0.011220	-0.005753	0.017360	-0.005631	0.023965	-0.002339
	0.001278	0.006798	-0.003652	-0.014845	0.029534	-0.023676
	0.011086					
x64	0.000724	0.026516	0.022342	0.008968	0.018028	-0.003331
	0.017949	0.015816	-0.016184	0.013533	0.002100	-0.012836
	-0.040288	-0.004961	0.002155	-0.010418	-0.019340	0.006463
	-0.011954	0.007796	-0.013166	0.003378	-0.001680	-0.000444
	-0.007754	0.001565	0.001958	0.018047	0.012935	0.020331
	0.010719					
y64	0.036963	0.022428	0.001887	-0.001630	-0.000508	0.007959
	-0.018664	0.034637	0.008286	0.017983	-0.015216	0.003780
	0.013075	-0.016251	-0.014790	-0.011318	-0.003621	0.019140
	0.000897	0.018500	0.026341	-0.000977	0.014952	0.008184
	0.032759	-0.019964	-0.012401	0.007713	0.008362	-0.009626
	0.004332					
z64	0.047084	-0.000457	0.025846	-0.003769	-0.009929	-0.007572
	-0.009393	0.001783	0.007317	0.034209	-0.031407	-0.002846
	0.018698	-0.010960	0.008486	-0.005168	-0.024045	0.011223
	0.011172	-0.006488	0.018024	-0.011488	0.024964	-0.002720
	0.000252	0.014181	-0.002577	-0.011821	0.030184	-0.024717
	0.006125					

x65	0.002147	0.027636	0.024330	0.008017	0.018643	-0.002432
	0.018919	0.011439	-0.014985	0.013018	0.003370	-0.013705
	-0.039724	-0.007732	-0.000326	-0.008486	-0.015295	0.006966
	-0.015743	0.010279	-0.016091	0.007995	-0.004837	0.000516
	-0.001973	0.003142	-0.001470	0.019090	0.018744	0.019939
	0.007561					
y65	0.034424	0.021863	0.000678	-0.001648	-0.000987	0.008722
	-0.017283	0.034174	0.007527	0.016046	-0.011968	0.004856
	0.010619	-0.018539	-0.013561	-0.007429	-0.002565	0.019460
	0.000434	0.018643	0.025280	-0.002250	0.014360	0.008397
	0.032198	-0.018662	-0.016096	0.008336	0.007430	-0.007620
	0.004250					
z65	0.048203	0.001029	0.024881	-0.003840	-0.010723	-0.006370
	-0.008432	0.003393	0.005431	0.033557	-0.029376	-0.000447
	0.017728	-0.012643	0.011890	-0.002849	-0.026276	0.015700
	0.011593	-0.007402	0.015720	-0.013048	0.024926	-0.004120
	0.000695	0.016591	-0.005712	-0.014645	0.032180	-0.023677
	0.008316					
x66	0.003791	0.028082	0.026528	0.006881	0.018086	-0.001292
	0.020002	0.006756	-0.016162	0.013520	0.005756	-0.013132
	-0.036800	-0.010298	-0.000779	-0.007500	-0.008773	0.009554
	-0.017737	0.013088	-0.018542	0.010176	-0.009152	0.002027
	0.002128	0.003315	-0.002347	0.018356	0.021639	0.014855
	0.008369					
y66	0.031996	0.020891	-0.000462	-0.001273	-0.001297	0.009038
	-0.016572	0.032525	0.007753	0.014336	-0.009108	0.004424
	0.009646	-0.020274	-0.013127	-0.005554	-0.000545	0.020452
	0.000682	0.019528	0.023572	-0.003517	0.012065	0.009428
	0.032921	-0.018977	-0.018085	0.005959	0.002299	-0.008083
	0.001903					
z66	0.049116	0.001641	0.024280	-0.003278	-0.011291	-0.005611
	-0.008458	0.002728	0.004661	0.033455	-0.028826	0.000248
	0.019689	-0.013107	0.014994	-0.003593	-0.027798	0.019600
	0.015009	-0.007691	0.013346	-0.015009	0.023690	-0.005348
	0.001577	0.018094	-0.008389	-0.020343	0.028410	-0.024890
	0.008367					
x67	0.004876	0.028704	0.028561	0.005928	0.017396	-0.000330
	0.021146	0.003453	-0.017221	0.015860	0.006892	-0.011522
	-0.034899	-0.010977	-0.000299	-0.006003	-0.004783	0.011308
	-0.018486	0.016329	-0.020741	0.010942	-0.010743	0.001318
	0.002913	0.000199	-0.001473	0.019703	0.021623	0.010758
	0.007113					

y67	0.029296	0.019685	-0.001317	-0.001755	-0.001195	0.009411
	-0.016527	0.032278	0.007280	0.013653	-0.007447	0.004752
	0.007579	-0.019811	-0.012855	-0.005155	0.000445	0.020641
	0.002108	0.017208	0.023487	-0.002466	0.010133	0.009227
	0.029784	-0.022672	-0.018419	0.006010	-0.004462	-0.005794
	0.002511					
z67	0.050021	0.002336	0.024332	-0.004014	-0.011204	-0.005154
	-0.009458	0.004062	0.003151	0.034904	-0.029819	0.002519
	0.019449	-0.011026	0.016518	-0.007214	-0.029696	0.021326
	0.017414	-0.012809	0.013574	-0.014696	0.022875	-0.007555
	-0.003994	0.014749	-0.007724	-0.021066	0.023348	-0.022160
	0.010407					
x68	0.005839	0.029323	0.030178	0.004965	0.016760	0.000959
	0.022187	0.000669	-0.018349	0.017693	0.008157	-0.010408
	-0.033249	-0.010757	0.000303	-0.005714	-0.001525	0.011930
	-0.018698	0.018766	-0.022450	0.011607	-0.012796	0.001279
	0.004655	-0.002602	-0.001773	0.021034	0.021984	0.009790
	0.010094					
y68	0.026619	0.018822	-0.002141	-0.001827	-0.000927	0.010164
	-0.015827	0.031817	0.007645	0.012556	-0.005050	0.004949
	0.006730	-0.018951	-0.013633	-0.003093	0.000193	0.020106
	0.001892	0.015581	0.022792	-0.002678	0.008232	0.009555
	0.026897	-0.025226	-0.019397	0.006003	-0.007435	-0.003339
	0.004809					
z68	0.050890	0.003886	0.024249	-0.004318	-0.010846	-0.004075
	-0.008863	0.005595	0.003304	0.034860	-0.028635	0.004503
	0.020289	-0.008190	0.016491	-0.008084	-0.032881	0.021095
	0.016453	-0.016815	0.013297	-0.015367	0.020602	-0.009351
	-0.008019	0.012617	-0.007206	-0.021278	0.023988	-0.017279
	0.015301					
x69	0.006423	0.030001	0.031660	0.004025	0.016159	0.002375
	0.022583	-0.001931	-0.019850	0.019051	0.008491	-0.009425
	-0.032768	-0.010730	0.001567	-0.006650	0.000881	0.014055
	-0.018074	0.021149	-0.025746	0.012353	-0.015906	-0.000148
	0.005873	-0.003774	-0.004657	0.023690	0.021931	0.007008
	0.010895					
y69	0.023718	0.017709	-0.003325	-0.002017	-0.000505	0.010678
	-0.015186	0.031385	0.008122	0.011414	-0.001298	0.004618
	0.005873	-0.019261	-0.013910	-0.001124	0.000729	0.017509
	0.002971	0.015280	0.021557	-0.001753	0.007297	0.008497
	0.025325	-0.024089	-0.020748	0.007874	-0.008326	-0.002106
	0.001057					

z69	0.051437	0.004743	0.023346	-0.004987	-0.010264	-0.003420
	-0.009155	0.006572	0.003326	0.034847	-0.024359	0.005063
	0.020119	-0.008621	0.017426	-0.009090	-0.033920	0.018392
	0.018621	-0.018438	0.010708	-0.013885	0.020634	-0.015225
	-0.008252	0.017749	-0.009660	-0.017038	0.027575	-0.016594
	0.008413					
x70	0.006560	0.030342	0.032630	0.003455	0.015539	0.004158
	0.023013	-0.004114	-0.021278	0.020441	0.008166	-0.008509
	-0.031554	-0.010291	0.002286	-0.007778	0.001880	0.014894
	-0.017110	0.022612	-0.026401	0.013610	-0.016944	0.000281
	0.003607	-0.004968	-0.008446	0.023115	0.020517	0.006098
	0.010112					
y70	0.020937	0.016680	-0.004430	-0.001825	-0.000168	0.011365
	-0.014734	0.031068	0.008626	0.010652	0.001042	0.005002
	0.005054	-0.018962	-0.015018	0.001525	0.000237	0.015247
	0.004069	0.015085	0.020420	-0.001217	0.008024	0.009111
	0.023602	-0.025849	-0.020614	0.009069	-0.008354	-0.003244
	-0.003138					
z70	0.051850	0.006152	0.022718	-0.004548	-0.010630	-0.001858
	-0.009647	0.008197	0.003941	0.035628	-0.024263	0.007764
	0.019709	-0.007393	0.015471	-0.007980	-0.038160	0.015216
	0.021515	-0.018678	0.009192	-0.013626	0.027250	-0.014466
	-0.011904	0.016817	-0.007165	-0.018302	0.036637	-0.022098
	0.002621					
x71	0.006538	0.030681	0.033441	0.002943	0.015196	0.006366
	0.023675	-0.005407	-0.022689	0.021137	0.008178	-0.007792
	-0.030305	-0.009575	0.002257	-0.009398	0.003261	0.012997
	-0.015821	0.024955	-0.027300	0.014142	-0.017919	0.001506
	0.002956	-0.006211	-0.011285	0.023394	0.022989	0.005764
	0.010715					
y71	0.018049	0.015518	-0.005349	-0.001516	-0.000160	0.012120
	-0.014255	0.030571	0.009017	0.009269	0.003139	0.005166
	0.005321	-0.017804	-0.015761	0.003601	-0.000064	0.014217
	0.004781	0.014404	0.019429	-0.001285	0.007330	0.009581
	0.021868	-0.027209	-0.020499	0.010039	-0.009374	-0.003183
	-0.001877					
z71	0.052283	0.007641	0.022378	-0.003704	-0.011493	-0.000170
	-0.008900	0.010555	0.004025	0.033386	-0.021876	0.011206
	0.023238	-0.005662	0.013866	-0.009049	-0.038325	0.012881
	0.021092	-0.019990	0.009718	-0.014113	0.025378	-0.008925
	-0.014035	0.020184	-0.007730	-0.016372	0.047156	-0.019982
	0.002241					

x72	0.006032	0.030399	0.033849	0.002482	0.015102	0.008160
	0.023844	-0.006380	-0.022691	0.022428	0.008885	-0.007610
	-0.028985	-0.008731	0.002944	-0.008803	0.002365	0.010209
	-0.014567	0.026801	-0.025325	0.014587	-0.018408	0.001689
	-0.001661	-0.009722	-0.013734	0.023439	0.025621	0.007093
	0.007901					
y72	0.015044	0.014485	-0.006493	-0.001113	0.000070	0.012783
	-0.013407	0.029731	0.008834	0.008279	0.005561	0.004755
	0.005465	-0.016640	-0.016101	0.005220	0.000393	0.012292
	0.006412	0.013176	0.018317	-0.000949	0.006763	0.010177
	0.021911	-0.027277	-0.019770	0.010610	-0.013451	-0.003162
	-0.001861					
z72	0.052256	0.008665	0.021531	-0.003264	-0.012637	0.000924
	-0.009881	0.010879	0.003239	0.035382	-0.019712	0.009998
	0.023480	-0.003300	0.015372	-0.009509	-0.041087	0.008350
	0.023945	-0.020920	0.008840	-0.013440	0.028540	-0.009284
	-0.012505	0.023903	-0.006750	-0.020072	0.041229	-0.014953
	-0.000709					
x73	0.005858	0.030181	0.034306	0.002054	0.014489	0.009832
	0.025084	-0.007245	-0.022396	0.021144	0.009513	-0.007801
	-0.026884	-0.007363	0.004396	-0.011240	0.002817	0.007245
	-0.014050	0.029626	-0.026498	0.014271	-0.021395	-0.001609
	0.000481	-0.011724	-0.012479	0.024993	0.030887	-0.000613
	0.007763					
y73	0.011899	0.013499	-0.007447	-0.000762	0.000138	0.013769
	-0.012677	0.029082	0.008815	0.006904	0.007424	0.004807
	0.005364	-0.015478	-0.016425	0.006811	0.001305	0.011163
	0.008008	0.011732	0.016827	-0.001488	0.005678	0.010426
	0.021317	-0.028424	-0.019590	0.012172	-0.014968	-0.003720
	0.000441					
z73	0.052209	0.010062	0.022552	-0.003582	-0.014564	0.003235
	-0.010683	0.012034	0.003349	0.032922	-0.015732	0.012820
	0.020808	-0.003705	0.014093	-0.011853	-0.036856	0.008399
	0.023552	-0.020735	0.006780	-0.014055	0.023416	-0.008198
	-0.011689	0.017392	-0.005951	-0.018301	0.048937	-0.016095
	0.007995					
x74	0.005381	0.029017	0.034500	0.002408	0.014602	0.010958
	0.026828	-0.006471	-0.022718	0.020770	0.010333	-0.005033
	-0.025989	-0.005916	0.007575	-0.014245	0.002047	0.007089
	-0.011058	0.031317	-0.024370	0.014706	-0.020789	-0.002812
	-0.000171	-0.013840	-0.013009	0.021304	0.026989	-0.007717
	0.008567					

y74	0.008891	0.012444	-0.008552	-0.000687	0.000100	0.014899
	-0.012294	0.027679	0.009325	0.006391	0.010487	0.003538
	0.005444	-0.014770	-0.017157	0.010650	0.000742	0.008034
	0.008571	0.010277	0.015946	-0.001781	0.005399	0.010327
	0.018446	-0.030512	-0.019833	0.014140	-0.012395	-0.000713
	-0.002107					
z74	0.052123	0.012072	0.022861	-0.004236	-0.014843	0.004864
	-0.011176	0.012399	0.001961	0.028896	-0.012280	0.014435
	0.019463	-0.005079	0.015020	-0.006788	-0.040002	0.009746
	0.021354	-0.024418	0.004048	-0.012517	0.021812	-0.003703
	-0.019999	0.015166	-0.006160	-0.014531	0.050915	-0.010775
	0.003414					
x75	0.004856	0.028499	0.035026	0.002749	0.014359	0.013149
	0.027677	-0.006473	-0.024045	0.021888	0.011212	-0.006947
	-0.026721	-0.005619	0.009137	-0.016481	0.006275	0.004386
	-0.006975	0.033909	-0.022558	0.015492	-0.021229	-0.003448
	-0.003885	-0.013531	-0.009121	0.020125	0.019734	-0.008313
	0.007942					
y75	0.006221	0.011475	-0.009685	-0.000267	0.000401	0.015243
	-0.011918	0.026411	0.009465	0.005109	0.012846	0.003658
	0.005427	-0.013881	-0.017237	0.013093	-0.000576	0.006922
	0.008723	0.007549	0.015732	-0.001920	0.005480	0.010943
	0.017147	-0.032114	-0.021237	0.015089	-0.011770	-0.000491
	-0.002114					
z75	0.052188	0.012623	0.021935	-0.004432	-0.015803	0.006209
	-0.010233	0.011373	0.000643	0.027695	-0.007253	0.014498
	0.018293	-0.001152	0.015851	-0.007303	-0.038303	0.007206
	0.021225	-0.027240	0.002134	-0.014447	0.019406	-0.000281
	-0.019782	0.012344	-0.006175	-0.013960	0.049465	-0.010265
	-0.005982					
x76	0.004259	0.028702	0.034291	0.002830	0.013299	0.016241
	0.028611	-0.008627	-0.024039	0.022325	0.011908	-0.012163
	-0.023619	-0.005120	0.009578	-0.016367	0.007435	0.002470
	-0.006636	0.033611	-0.022596	0.013826	-0.023422	-0.004083
	-0.004828	-0.011624	-0.005032	0.023898	0.015311	-0.010670
	0.004124					
y76	0.003981	0.010439	-0.010761	-0.000071	0.000888	0.014832
	-0.011245	0.025182	0.008582	0.003152	0.015245	0.004929
	0.005225	-0.012768	-0.016561	0.014573	-0.001821	0.006124
	0.008376	0.006440	0.016154	-0.002223	0.005441	0.011730
	0.017394	-0.034169	-0.020620	0.014456	-0.013120	-0.002458
	-0.002403					

z76	0.051766	0.012649	0.022585	-0.005201	-0.016987	0.007684
	-0.009170	0.010411	0.001840	0.024689	-0.001152	0.013877
	0.014264	-0.001278	0.014350	-0.007538	-0.030163	0.005846
	0.018459	-0.022153	-0.001055	-0.013979	0.013751	-0.001539
	-0.017391	0.010074	-0.000883	-0.018026	0.047493	-0.005625
	-0.015976					
x77	0.003132	0.028825	0.032930	0.002929	0.012874	0.018854
	0.029457	-0.010803	-0.023251	0.022038	0.011976	-0.016671
	-0.019897	-0.004671	0.010183	-0.014698	0.005606	0.000698
	-0.005622	0.032740	-0.020318	0.013288	-0.024862	-0.006266
	-0.007427	-0.010773	-0.000012	0.024856	0.014005	-0.010400
	-0.002303					
y77	0.001533	0.009290	-0.011782	0.000164	0.001143	0.014952
	-0.010485	0.024195	0.007710	0.001526	0.017352	0.005525
	0.004478	-0.010655	-0.015194	0.015897	-0.002419	0.005254
	0.008877	0.005048	0.015344	-0.002836	0.004823	0.012558
	0.017245	-0.036086	-0.021062	0.014658	-0.014972	-0.003998
	-0.003159					
z77	0.051035	0.012597	0.022991	-0.006608	-0.017716	0.008340
	-0.008671	0.009419	0.001650	0.025178	0.005372	0.013508
	0.009771	-0.001988	0.012836	-0.006192	-0.025556	0.003984
	0.016592	-0.020818	-0.003129	-0.014227	0.011715	0.003668
	-0.013944	0.005925	0.003527	-0.015155	0.039522	-0.004101
	-0.006667					
x78	0.001304	0.028724	0.032546	0.002775	0.012924	0.021116
	0.029855	-0.012490	-0.021083	0.020917	0.012142	-0.019355
	-0.019323	-0.005450	0.008335	-0.012446	0.006092	-0.001995
	-0.004091	0.032177	-0.018454	0.014761	-0.024824	-0.006578
	-0.012080	-0.011311	0.001269	0.021787	0.014148	-0.006462
	-0.008857					
y78	-0.000698	0.007862	-0.012771	0.000250	0.001295	0.014736
	-0.009736	0.023070	0.006670	0.000700	0.019106	0.005962
	0.003166	-0.008765	-0.013744	0.017063	-0.004974	0.003368
	0.008677	0.002846	0.015428	-0.004391	0.003978	0.013126
	0.015789	-0.037289	-0.020071	0.012569	-0.014673	-0.003508
	-0.003836					
z78	0.050630	0.012374	0.021641	-0.007218	-0.017509	0.010269
	-0.006904	0.009082	-0.001258	0.027247	0.012390	0.014108
	0.012192	0.001848	0.015635	-0.004277	-0.019535	0.004432
	0.015749	-0.020247	-0.010235	-0.016031	0.009940	0.005885
	-0.012387	0.003803	0.001518	-0.009360	0.034288	-0.007284
	0.001096					

x79	-0.000242	0.028735	0.031897	0.002489	0.013249	0.022846
	0.030619	-0.014626	-0.018803	0.020649	0.012633	-0.021277
	-0.017553	-0.005973	0.007829	-0.008695	0.006566	-0.003350
	-0.004500	0.031102	-0.016899	0.015122	-0.024000	-0.006511
	-0.015069	-0.012401	0.001940	0.019656	0.014718	-0.006678
	-0.012026					
y79	-0.002907	0.006499	-0.013600	0.000832	0.001403	0.014088
	-0.009592	0.021593	0.006386	0.000108	0.022308	0.004869
	0.002751	-0.007068	-0.011735	0.017359	-0.007372	0.002639
	0.009250	0.002228	0.016127	-0.006172	0.003847	0.012913
	0.014920	-0.039738	-0.019681	0.012717	-0.013120	-0.003438
	-0.002716					
z79	0.048846	0.012294	0.019876	-0.007516	-0.017425	0.013489
	-0.005337	0.009034	-0.003135	0.025832	0.015311	0.014923
	0.012896	0.007250	0.012781	-0.004051	-0.016003	0.000580
	0.019114	-0.022216	-0.015336	-0.016722	0.009619	0.010004
	-0.013006	0.002543	0.003208	-0.010063	0.026897	-0.006608
	0.000143					
x80	-0.001903	0.028685	0.031246	0.002112	0.013789	0.024206
	0.030915	-0.016682	-0.016486	0.020198	0.011528	-0.021410
	-0.015189	-0.006516	0.006753	-0.003891	0.006693	-0.004903
	-0.005231	0.028076	-0.014790	0.015449	-0.019022	-0.005691
	-0.020348	-0.013455	0.003927	0.015630	0.012609	-0.006674
	-0.012698					
y80	-0.004897	0.005264	-0.014274	0.001653	0.001555	0.013242
	-0.009568	0.019834	0.006375	-0.000567	0.023840	0.005529
	0.002220	-0.004837	-0.010853	0.015382	-0.010869	0.000840
	0.009024	0.000772	0.015646	-0.008804	0.006165	0.012958
	0.012167	-0.040387	-0.017782	0.011945	-0.013652	-0.007079
	0.000009					
z80	0.047251	0.011992	0.018077	-0.008677	-0.017262	0.015527
	-0.003338	0.008249	-0.004484	0.026974	0.020965	0.014540
	0.011906	0.011902	0.010789	0.000770	-0.013199	-0.000689
	0.024424	-0.022532	-0.019588	-0.016086	0.001044	0.007969
	-0.006868	-0.003398	0.002439	-0.006468	0.021677	-0.002136
	-0.003709					
x81	-0.003532	0.028778	0.030831	0.001863	0.015065	0.025414
	0.030569	-0.017449	-0.013599	0.019932	0.009760	-0.019313
	-0.012669	-0.006312	0.005096	0.002112	0.006869	-0.005410
	-0.004562	0.024263	-0.014386	0.013699	-0.012420	-0.006144
	-0.027427	-0.016264	-0.000290	0.008816	0.010683	-0.003943
	-0.015699					

y81	-0.006294	0.004297	-0.015139	0.003396	0.001826	0.012188
	-0.009037	0.017246	0.006635	-0.001375	0.025953	0.006842
	0.003152	-0.002491	-0.010553	0.015677	-0.014513	0.002260
	0.008583	-0.000046	0.014026	-0.012375	0.006367	0.011980
	0.011689	-0.041522	-0.015609	0.012670	-0.015675	-0.010572
	-0.000274					
z81	0.046703	0.011945	0.016499	-0.010397	-0.018780	0.015271
	-0.000693	0.003975	-0.004347	0.026321	0.027146	0.011993
	0.009733	0.013682	0.006791	0.000674	-0.009560	-0.002530
	0.023790	-0.020348	-0.021900	-0.014877	-0.007573	0.009270
	0.004125	-0.005077	0.012378	-0.005197	0.016957	-0.003894
	-0.001433					
x82	-0.004749	0.029066	0.030259	0.001722	0.016776	0.026108
	0.030177	-0.019232	-0.010450	0.020687	0.006768	-0.014176
	-0.008188	-0.005493	0.003844	0.010014	0.006650	-0.005149
	-0.003519	0.018044	-0.014468	0.009157	-0.003518	-0.007634
	-0.033040	-0.018434	-0.006438	-0.000245	0.008545	0.000022
	-0.015834					
y82	-0.007512	0.003384	-0.016158	0.005980	0.002584	0.010778
	-0.009033	0.014070	0.007687	-0.001751	0.027390	0.009508
	0.005099	0.000089	-0.010645	0.017185	-0.019780	0.004047
	0.008740	-0.003092	0.010990	-0.015065	0.008344	0.009252
	0.010057	-0.041734	-0.017048	0.012118	-0.015795	-0.011908
	0.003374					
z82	0.045833	0.011642	0.015301	-0.012502	-0.020821	0.013893
	0.000638	0.001872	-0.004000	0.026047	0.029426	0.010767
	0.008022	0.013718	0.000563	-0.002540	-0.009501	-0.004570
	0.023352	-0.016212	-0.023044	-0.014501	-0.008627	0.009740
	0.004055	-0.009795	0.021878	-0.007731	0.007923	-0.005412
	-0.003213					
x83	-0.006175	0.028837	0.028898	0.000697	0.017905	0.026371
	0.030431	-0.020720	-0.008311	0.020870	0.001794	-0.009009
	-0.003752	-0.004930	0.003325	0.017643	0.007524	-0.004966
	-0.002371	0.012415	-0.013543	0.003695	0.006810	-0.011553
	-0.038732	-0.024427	-0.009439	-0.012532	0.004384	0.006826
	-0.020191					
y83	-0.008542	0.002552	-0.017524	0.008583	0.002901	0.009464
	-0.009631	0.011354	0.008347	-0.001123	0.027990	0.010926
	0.006257	0.003225	-0.011015	0.016223	-0.026777	0.005960
	0.010263	-0.007705	0.007900	-0.020422	0.011239	0.005456
	0.005837	-0.042999	-0.016816	0.009633	-0.017762	-0.011216
	0.004826					

z83	0.044293	0.011390	0.014450	-0.014079	-0.019431	0.013379
	0.000773	0.000851	-0.001128	0.026155	0.031465	0.014853
	0.010523	0.011788	-0.001886	0.001331	-0.012780	-0.004352
	0.027008	-0.018528	-0.024916	-0.013566	-0.002292	0.015120
	0.002533	-0.012758	0.017852	-0.010926	0.004664	-0.010812
	0.000992					
x84	0.019484	0.020219	-0.006445	-0.003846	0.014677	0.010646
	0.021221	-0.009581	-0.012652	-0.018166	-0.015585	-0.008089
	-0.006680	0.018235	0.008317	0.006920	0.009046	-0.016773
	-0.056125	-0.011890	0.019308	-0.008953	0.012770	-0.021356
	-0.005262	0.003942	-0.009437	-0.013080	-0.009538	0.000679
	0.000583					
y84	-0.028905	-0.006902	-0.001890	0.010949	0.007283	0.020411
	0.027424	-0.010187	-0.007289	0.000959	0.029176	-0.008381
	0.007194	0.005878	-0.000722	0.003862	-0.007687	-0.005712
	-0.008599	0.003622	0.001461	-0.014027	0.008001	-0.006406
	0.025267	0.026288	-0.010971	-0.007203	-0.004580	-0.000488
	0.031508					
z84	0.006575	0.001619	-0.016896	-0.007372	-0.013323	0.002955
	-0.005652	0.021591	0.004062	-0.013847	0.018851	-0.015842
	-0.004565	0.018977	-0.017331	0.040150	0.011337	-0.001659
	-0.022336	0.007860	-0.002409	0.016944	-0.000821	-0.014034
	-0.000833	-0.025913	0.010874	-0.008025	0.005289	0.019137
	-0.021967					
x85	0.019441	0.020194	-0.005487	-0.006268	0.015133	0.012790
	0.018613	-0.012018	-0.014610	-0.013512	-0.016449	-0.005851
	-0.007525	0.016660	0.007176	0.003446	0.007062	-0.020039
	-0.047430	-0.012678	0.019749	-0.008393	0.012323	-0.020176
	-0.004662	0.003626	-0.004714	-0.016942	-0.011398	0.002034
	-0.004656					
y85	-0.029446	-0.007681	-0.002174	0.010128	0.006763	0.021805
	0.028476	-0.009018	-0.007978	-0.000832	0.029901	-0.009879
	0.004618	0.005070	-0.001725	0.003604	-0.009011	-0.003914
	-0.005748	0.002309	0.002347	-0.011797	0.006693	-0.007312
	0.023476	0.019979	-0.008393	-0.012871	-0.000674	-0.002736
	0.028677					
z85	0.007556	0.001385	-0.017047	-0.006696	-0.013879	0.002431
	-0.005002	0.020723	0.005141	-0.015551	0.023977	-0.015492
	-0.001332	0.021912	-0.013122	0.044568	0.008852	-0.000328
	-0.026494	0.005867	-0.005819	0.010194	0.000794	-0.014327
	-0.001614	-0.024140	0.008749	0.000173	0.008379	0.019742
	-0.013710					

x86	0.019639	0.020821	-0.004236	-0.008363	0.015211	0.015399
	0.016087	-0.014603	-0.015093	-0.008025	-0.017413	-0.001681
	-0.008396	0.015586	0.004527	0.001539	0.004785	-0.024690
	-0.040646	-0.015493	0.018560	-0.006202	0.012077	-0.018416
	-0.005088	0.004153	-0.004796	-0.020302	-0.010224	0.003744
	-0.007449					
y86	-0.029893	-0.008103	-0.002953	0.009169	0.005592	0.023810
	0.029098	-0.008025	-0.008954	-0.001896	0.030892	-0.011086
	0.002616	0.003457	-0.002402	0.003248	-0.010180	-0.001752
	-0.002094	0.000523	0.002898	-0.009664	0.004820	-0.009767
	0.020854	0.014536	-0.006124	-0.016348	0.001572	-0.002448
	0.025849					
z86	0.008441	0.000276	-0.017774	-0.006793	-0.014295	0.000694
	-0.004557	0.019232	0.005021	-0.017095	0.026143	-0.014659
	-0.000034	0.021077	-0.010562	0.046975	0.006122	0.002926
	-0.026708	0.003826	-0.008258	0.002216	0.002194	-0.013059
	-0.002565	-0.019940	0.010661	0.005114	0.004925	0.019130
	-0.008149					
x87	0.019812	0.022282	-0.003176	-0.009675	0.014217	0.017240
	0.013995	-0.017634	-0.013966	-0.003468	-0.020443	0.001874
	-0.008689	0.013134	-0.000448	0.001130	0.001850	-0.027139
	-0.035944	-0.018660	0.016174	-0.005213	0.013934	-0.015871
	-0.003672	0.007780	-0.006263	-0.020303	-0.008083	0.001830
	-0.008255					
y87	-0.030115	-0.008255	-0.004161	0.008450	0.004439	0.025669
	0.029114	-0.007447	-0.009058	-0.002055	0.032198	-0.011694
	0.001704	0.002588	-0.004146	0.001972	-0.010733	-0.001252
	0.001361	-0.001222	0.003553	-0.007868	0.003118	-0.012923
	0.017745	0.011191	-0.003384	-0.019690	0.003328	-0.002447
	0.021471					
z87	0.009604	-0.001294	-0.018664	-0.007271	-0.014002	-0.000755
	-0.004426	0.019034	0.005172	-0.017281	0.029194	-0.012531
	0.000702	0.021474	-0.006212	0.046799	0.005004	0.003450
	-0.026187	0.001983	-0.006897	-0.002289	-0.001036	-0.014581
	-0.005612	-0.020314	0.013797	0.006781	-0.000308	0.021037
	-0.006418					
x88	0.020294	0.023674	-0.002290	-0.010804	0.013474	0.018219
	0.012265	-0.019846	-0.012387	0.002534	-0.022586	0.005382
	-0.009283	0.010696	-0.002434	0.002257	-0.001859	-0.027767
	-0.033036	-0.019490	0.012356	-0.005549	0.013617	-0.014026
	-0.001345	0.010486	-0.006910	-0.019156	-0.003349	0.000804
	-0.011876					

y88	-0.029989	-0.008055	-0.005276	0.007723	0.003664	0.027645
	0.028754	-0.006576	-0.009650	-0.001354	0.033763	-0.011885
	0.001714	0.002818	-0.005425	0.001420	-0.010851	-0.000750
	0.004452	-0.002654	0.004615	-0.005058	0.001044	-0.016303
	0.016114	0.008836	0.000745	-0.023255	0.006562	-0.001277
	0.018309					
z88	0.010891	-0.002496	-0.019135	-0.007768	-0.013798	-0.001478
	-0.004644	0.018357	0.004666	-0.017431	0.029993	-0.008988
	0.001204	0.021192	-0.005542	0.044837	0.005745	0.003012
	-0.024309	-0.001961	-0.005128	-0.004320	-0.002123	-0.015157
	-0.008917	-0.019031	0.014414	0.006611	-0.007057	0.019716
	-0.000416					
x89	0.021099	0.024651	-0.001460	-0.011836	0.012834	0.018823
	0.011474	-0.020902	-0.012802	0.007814	-0.024181	0.009977
	-0.009538	0.008302	-0.005607	-0.000162	-0.000417	-0.028487
	-0.029171	-0.022283	0.010872	-0.003030	0.013262	-0.013488
	-0.000735	0.012183	-0.010856	-0.020555	-0.005872	0.001287
	-0.011614					
y89	-0.029705	-0.007751	-0.006518	0.007025	0.002896	0.028974
	0.028189	-0.005972	-0.010447	-0.000612	0.034719	-0.012443
	0.001322	0.002974	-0.006565	0.000307	-0.010089	-0.001241
	0.007108	-0.003072	0.005552	-0.001313	0.000290	-0.019082
	0.012667	0.004882	0.002248	-0.025843	0.007555	0.001386
	0.019814					
z89	0.011896	-0.003006	-0.019394	-0.008309	-0.013578	-0.002131
	-0.005192	0.017063	0.006151	-0.016897	0.030118	-0.006056
	0.001637	0.020484	-0.004274	0.045690	0.002789	0.001922
	-0.023596	-0.003654	-0.005084	-0.008541	-0.003736	-0.014279
	-0.010252	-0.017515	0.016782	0.008140	-0.006255	0.017262
	0.000159					
x90	0.021353	0.026621	-0.000493	-0.012786	0.012218	0.019422
	0.010774	-0.022737	-0.011870	0.011584	-0.025529	0.012698
	-0.010611	0.004838	-0.008313	-0.000810	0.001137	-0.031601
	-0.025698	-0.026345	0.012246	0.002521	0.012500	-0.014416
	-0.003054	0.012831	-0.009012	-0.022577	-0.002123	0.002254
	-0.011143					
y90	-0.029275	-0.007267	-0.007318	0.006337	0.002539	0.029699
	0.027078	-0.005328	-0.010608	0.000275	0.036182	-0.012062
	0.000364	0.003470	-0.007645	0.000236	-0.010064	-0.002945
	0.008278	-0.002683	0.005682	0.001027	0.000356	-0.023263
	0.008895	0.001106	0.003503	-0.026805	0.008606	0.003771
	0.020556					

z90	0.013444	-0.003834	-0.020063	-0.008835	-0.013505	-0.002737
	-0.004858	0.015780	0.006499	-0.016691	0.029386	-0.002566
	0.002732	0.020208	-0.004026	0.043969	0.000720	0.002017
	-0.021196	-0.005229	-0.006023	-0.013883	-0.005023	-0.011422
	-0.011633	-0.015439	0.013726	0.009584	-0.009841	0.015982
	-0.000538					
x91	0.021277	0.028466	0.002140	-0.013251	0.012023	0.020011
	0.008844	-0.022929	-0.007920	0.016243	-0.024130	0.017086
	-0.010406	0.003275	-0.013620	0.000244	0.000338	-0.033475
	-0.023177	-0.026359	0.010164	0.003150	0.010503	-0.013154
	-0.003906	0.011064	-0.012945	-0.018557	0.000840	0.005121
	-0.009605					
y91	-0.028745	-0.006826	-0.006965	0.005909	0.002776	0.029535
	0.024902	-0.004654	-0.010123	0.002047	0.038589	-0.010551
	-0.001876	0.002898	-0.009098	0.000398	-0.008687	-0.005741
	0.008211	-0.001973	0.005352	0.003234	0.000555	-0.026774
	0.005299	-0.001739	0.003183	-0.026074	0.010292	0.007325
	0.021356					
z91	0.014889	-0.004590	-0.020491	-0.008942	-0.013362	-0.003805
	-0.004956	0.014444	0.007011	-0.015111	0.027381	0.000663
	0.002462	0.018692	-0.003686	0.042113	0.000514	0.002217
	-0.018445	-0.007083	-0.005218	-0.017276	-0.006498	-0.009020
	-0.011684	-0.012177	0.012924	0.007209	-0.011921	0.012794
	-0.002281					
x92	0.021404	0.029001	0.004634	-0.013687	0.012166	0.020309
	0.007213	-0.024646	-0.005297	0.022492	-0.024556	0.021481
	-0.008385	0.001776	-0.016220	0.001732	-0.001202	-0.030161
	-0.018434	-0.026988	0.007846	0.001070	0.007128	-0.009330
	0.001095	0.010693	-0.016984	-0.020756	-0.000835	0.005946
	-0.003537					
y92	-0.027679	-0.006378	-0.006852	0.005453	0.003230	0.029231
	0.022638	-0.004422	-0.010775	0.004230	0.040323	-0.008461
	-0.003656	0.002455	-0.010122	-0.000268	-0.006628	-0.008509
	0.008675	-0.001227	0.004243	0.004513	0.002537	-0.029487
	0.002316	-0.004517	0.002842	-0.024907	0.005731	0.007833
	0.025571					
z92	0.016322	-0.004521	-0.020946	-0.009013	-0.013493	-0.004779
	-0.005007	0.013524	0.007922	-0.013133	0.026128	0.004644
	0.002841	0.017955	-0.003733	0.040642	-0.001637	0.000999
	-0.017319	-0.008695	-0.004777	-0.019989	-0.009040	-0.008677
	-0.011927	-0.008385	0.014024	0.008642	-0.010453	0.009562
	-0.007432					

x93	0.021089	0.030484	0.006475	-0.014730	0.012919	0.020553
	0.006249	-0.024886	-0.003750	0.027061	-0.025336	0.025066
	-0.007843	-0.001917	-0.018203	0.001130	-0.001151	-0.029604
	-0.014941	-0.027033	0.006326	-0.000849	0.004072	-0.005903
	0.002598	0.013034	-0.013431	-0.022070	-0.003301	-0.000760
	0.000523					
y93	-0.026290	-0.005470	-0.006925	0.004801	0.004314	0.028446
	0.020956	-0.003644	-0.010680	0.006951	0.042627	-0.005691
	-0.003510	0.001957	-0.008594	0.002664	-0.007164	-0.008583
	0.006845	0.000575	0.002335	0.002647	-0.000001	-0.030990
	0.006720	-0.006851	0.007430	-0.022548	0.006831	0.006494
	0.024292					
z93	0.017925	-0.004573	-0.021349	-0.008730	-0.014251	-0.005426
	-0.005481	0.012004	0.008935	-0.010885	0.024384	0.008569
	0.003182	0.018058	-0.005618	0.038665	-0.004106	-0.001114
	-0.014944	-0.012008	-0.004107	-0.021068	-0.010253	-0.009278
	-0.013986	-0.005394	0.010447	0.009564	-0.010249	0.009903
	-0.010310					
x94	0.020603	0.031267	0.007208	-0.016651	0.013548	0.019843
	0.005441	-0.026278	-0.003268	0.030525	-0.026806	0.028930
	-0.008328	-0.005133	-0.021012	0.000997	-0.004207	-0.030539
	-0.013308	-0.024977	0.002706	-0.000380	0.004416	-0.004699
	-0.002802	0.015076	-0.011353	-0.021853	-0.004602	-0.007805
	0.000233					
y94	-0.025144	-0.004134	-0.007328	0.004301	0.005112	0.028790
	0.020503	-0.003508	-0.008374	0.011346	0.044995	-0.002228
	-0.002979	0.001847	-0.008203	0.006828	-0.008793	-0.007276
	0.007125	0.000053	-0.001784	-0.000232	-0.003945	-0.029532
	0.011782	-0.007149	0.014397	-0.021029	0.008099	0.003933
	0.019215					
z94	0.019903	-0.004285	-0.021168	-0.008362	-0.015039	-0.006391
	-0.006908	0.011563	0.009281	-0.008654	0.023086	0.011457
	0.003574	0.017673	-0.006251	0.036726	-0.004419	-0.002194
	-0.012394	-0.015070	-0.001274	-0.021179	-0.012660	-0.010154
	-0.013651	-0.003703	0.005716	0.009745	-0.008242	0.011126
	-0.010578					
x95	0.020470	0.032405	0.008155	-0.017417	0.013020	0.019432
	0.004982	-0.027527	-0.001864	0.033520	-0.025844	0.031080
	-0.004956	-0.005590	-0.024209	0.000586	-0.007797	-0.028058
	-0.010158	-0.028564	0.002069	0.001648	-0.003497	-0.003147
	0.000965	0.016327	-0.017616	-0.018481	-0.004974	-0.003707
	-0.000449					

y95	-0.024022	-0.002902	-0.007689	0.004109	0.004976	0.029321
	0.019387	-0.004205	-0.006448	0.014719	0.046143	-0.000233
	-0.002887	0.002019	-0.010160	0.008459	-0.009852	-0.008391
	0.007987	-0.000836	-0.005379	-0.001924	-0.005438	-0.028935
	0.014320	-0.006888	0.018199	-0.017697	0.005972	0.001475
	0.016613					
z95	0.021769	-0.003835	-0.021144	-0.008540	-0.015470	-0.007508
	-0.007978	0.010794	0.009828	-0.006531	0.021108	0.015021
	0.003160	0.016017	-0.006107	0.035284	-0.005327	-0.002868
	-0.010810	-0.016425	0.001110	-0.022816	-0.012924	-0.010547
	-0.014073	-0.002159	0.005587	0.007774	-0.004921	0.008571
	-0.012594					
x96	0.019841	0.033790	0.009780	-0.017625	0.012602	0.018499
	0.004739	-0.027136	0.000168	0.035018	-0.026462	0.032718
	-0.002390	-0.007024	-0.024954	-0.001065	-0.007080	-0.025734
	-0.006577	-0.027895	0.004658	0.004250	-0.008394	-0.000501
	0.003126	0.016859	-0.016753	-0.014262	-0.004478	-0.003897
	-0.003336					
y96	-0.023130	-0.001619	-0.007964	0.003660	0.005033	0.029521
	0.018038	-0.004419	-0.004234	0.016221	0.046638	0.000278
	-0.003749	0.001770	-0.012490	0.009297	-0.009390	-0.010404
	0.010046	-0.000860	-0.006423	-0.002661	-0.007294	-0.028118
	0.015537	-0.008581	0.021293	-0.014714	0.005829	0.001094
	0.011197					
z96	0.023909	-0.003293	-0.021154	-0.008528	-0.016061	-0.008615
	-0.009019	0.009730	0.010182	-0.003969	0.019406	0.019768
	0.003352	0.014649	-0.006770	0.033833	-0.007233	-0.003305
	-0.009973	-0.019011	0.002286	-0.024611	-0.013726	-0.011345
	-0.013362	0.000506	0.004418	0.006769	-0.003455	0.006571
	-0.012818					
x97	0.018897	0.034873	0.011459	-0.018728	0.012751	0.017744
	0.006054	-0.027103	0.000247	0.034745	-0.024373	0.034620
	-0.003297	-0.008244	-0.024631	-0.000009	-0.010126	-0.026419
	-0.004136	-0.028685	0.001953	0.003578	-0.009999	0.000882
	-0.003001	0.017396	-0.014640	-0.012354	-0.003624	-0.001793
	-0.003186					
y97	-0.022120	-0.000827	-0.007869	0.002631	0.005485	0.029383
	0.017025	-0.004877	-0.003279	0.017740	0.047018	0.002478
	-0.006843	0.000137	-0.015053	0.010439	-0.009819	-0.013674
	0.010348	-0.001318	-0.008134	-0.002683	-0.006485	-0.026559
	0.012124	-0.009555	0.023663	-0.013615	0.005982	-0.001352
	0.008298					

z97	0.026102	-0.002390	-0.021099	-0.008384	-0.016606	-0.009490
	-0.009954	0.008929	0.010549	-0.001292	0.017988	0.024738
	0.004088	0.013784	-0.007366	0.031678	-0.008606	-0.003156
	-0.008317	-0.022457	0.003214	-0.025821	-0.015766	-0.012196
	-0.011594	0.002644	0.003211	0.006042	-0.003726	0.005222
	-0.012888					
x98	0.018225	0.035628	0.012905	-0.019222	0.012587	0.017297
	0.006278	-0.027946	0.000938	0.035282	-0.022048	0.034154
	-0.004318	-0.008320	-0.024182	-0.000376	-0.010860	-0.025556
	0.001687	-0.027172	-0.001291	0.001720	-0.011727	0.001218
	-0.005044	0.015936	-0.015863	-0.008095	-0.009633	0.003083
	-0.003374					
y98	-0.020942	-0.000138	-0.007854	0.002002	0.005835	0.029047
	0.015407	-0.005675	-0.001949	0.019311	0.046803	0.003924
	-0.009662	-0.001016	-0.018033	0.010835	-0.010149	-0.016297
	0.011991	-0.000016	-0.009154	-0.002720	-0.006172	-0.025058
	0.009941	-0.010982	0.022863	-0.010947	0.004790	-0.001286
	0.004408					
z98	0.028327	-0.001781	-0.020908	-0.008335	-0.017411	-0.010353
	-0.010986	0.007959	0.011085	0.001462	0.016943	0.029143
	0.004579	0.012870	-0.007999	0.029507	-0.010005	-0.002524
	-0.007562	-0.026174	0.003541	-0.027596	-0.017257	-0.013740
	-0.010446	0.003741	0.002047	0.005476	-0.003546	0.003783
	-0.012699					
x99	0.017598	0.035806	0.014542	-0.019084	0.011719	0.017242
	0.006520	-0.026867	0.002661	0.036307	-0.019275	0.031108
	-0.001637	-0.009446	-0.024228	-0.000333	-0.007593	-0.023278
	0.003687	-0.025637	0.000287	0.003215	-0.014735	0.002485
	-0.001677	0.010563	-0.018107	-0.001023	-0.007875	0.009251
	-0.010585					
y99	-0.019591	0.000908	-0.008355	0.001842	0.006030	0.028601
	0.013901	-0.005094	-0.000396	0.020108	0.047013	0.003101
	-0.009161	-0.000892	-0.019196	0.012246	-0.010372	-0.016728
	0.013541	0.000607	-0.007636	-0.001853	-0.009864	-0.024595
	0.012558	-0.014336	0.021234	-0.005062	0.007809	0.003481
	-0.004782					
z99	0.030615	-0.001025	-0.020443	-0.008145	-0.018485	-0.010804
	-0.012147	0.007326	0.012164	0.004857	0.016379	0.033351
	0.005072	0.011312	-0.009123	0.027382	-0.009814	-0.001782
	-0.006821	-0.029820	0.004480	-0.028971	-0.018194	-0.014362
	-0.008416	0.004424	0.001132	0.004588	-0.002437	0.002448
	-0.011696					

x100	0.016633	0.035826	0.015863	-0.019740	0.011626	0.016844
	0.008622	-0.027104	0.001800	0.036677	-0.018182	0.030118
	0.000328	-0.010927	-0.024732	-0.002464	-0.005920	-0.020725
	0.003843	-0.022245	0.000512	0.003208	-0.016524	0.005903
	-0.001845	0.007719	-0.017255	0.001592	-0.009310	0.010152
	-0.006745					
y100	-0.018165	0.001180	-0.009272	0.000858	0.006391	0.027127
	0.013023	-0.005690	-0.001010	0.020724	0.045120	0.004804
	-0.009236	-0.001618	-0.021208	0.011128	-0.013087	-0.018246
	0.012889	0.001861	-0.005997	-0.001561	-0.011042	-0.023282
	0.012595	-0.014756	0.022251	0.000028	0.007390	0.000946
	-0.006931					
z100	0.032706	-0.000118	-0.019462	-0.008195	-0.019555	-0.011004
	-0.013034	0.006095	0.013085	0.008309	0.015797	0.038058
	0.004975	0.009482	-0.010920	0.024364	-0.010008	-0.000709
	-0.006422	-0.033017	0.004033	-0.030323	-0.018684	-0.014539
	-0.008919	0.005070	0.000182	0.002695	-0.002561	0.001391
	-0.009817					
x101	0.015260	0.036128	0.017527	-0.019294	0.010937	0.016726
	0.011163	-0.027772	0.000782	0.037089	-0.014689	0.028437
	-0.001167	-0.011323	-0.024267	-0.003042	-0.004267	-0.016900
	0.004797	-0.021041	-0.001674	0.004764	-0.016971	0.008355
	-0.005450	0.008328	-0.015019	0.006250	-0.013213	0.004863
	-0.007226					
y101	-0.016844	0.001715	-0.009757	0.000237	0.006014	0.025111
	0.011689	-0.005921	-0.001599	0.021784	0.044465	0.007339
	-0.009999	-0.001844	-0.022606	0.010639	-0.015373	-0.018588
	0.012688	0.001223	-0.004291	0.000350	-0.012557	-0.021503
	0.011701	-0.013303	0.022120	0.009067	0.005712	-0.004706
	-0.011780					
z101	0.034693	0.001119	-0.018315	-0.008127	-0.020590	-0.010836
	-0.013647	0.004640	0.013924	0.011637	0.015790	0.042418
	0.003031	0.008029	-0.012829	0.021895	-0.010695	0.000086
	-0.006307	-0.036561	0.001765	-0.031939	-0.017573	-0.015544
	-0.011366	0.005589	0.000733	0.000364	-0.003630	-0.002104
	-0.007711					
x102	0.014536	0.036272	0.019756	-0.018258	0.010770	0.016626
	0.012834	-0.028730	0.001876	0.036530	-0.010862	0.026455
	-0.001132	-0.013937	-0.021607	-0.000817	-0.000146	-0.012951
	0.002871	-0.018063	-0.001815	0.006877	-0.016662	0.010513
	-0.004876	0.007447	-0.015326	0.007267	-0.012055	0.004142
	-0.002518					

y102	-0.015625	0.002038	-0.009677	0.000530	0.006657	0.022350
	0.009797	-0.005895	0.000351	0.022009	0.042479	0.009459
	-0.009649	-0.003506	-0.023915	0.009394	-0.015692	-0.018175
	0.010617	0.003207	0.002375	0.003168	-0.013707	-0.017016
	0.013351	-0.013324	0.018666	0.013270	0.010073	-0.009014
	-0.014142					
z102	0.036840	0.002343	-0.016540	-0.007747	-0.021391	-0.009904
	-0.014970	0.003460	0.015189	0.014747	0.017237	0.046782
	0.000427	0.006317	-0.013674	0.022327	-0.011904	0.000212
	-0.007645	-0.039391	-0.003872	-0.034943	-0.015831	-0.017584
	-0.012849	0.006259	0.002396	-0.001196	-0.006265	-0.002249
	-0.004570					
x103	0.013725	0.036757	0.021842	-0.016705	0.009945	0.017886
	0.016372	-0.029471	0.001246	0.034614	-0.004984	0.020700
	-0.001717	-0.015587	-0.018538	0.000380	0.005308	-0.006577
	-0.001175	-0.010874	-0.004255	0.005918	-0.021175	0.013164
	0.003354	0.004500	-0.009571	0.009058	-0.011470	-0.001401
	-0.003425					
y103	-0.014094	0.003494	-0.010644	0.002028	0.006438	0.021309
	0.008389	-0.004557	0.002150	0.023298	0.042317	0.010700
	-0.008078	-0.003012	-0.023015	0.009843	-0.017665	-0.014831
	0.007919	0.003826	0.006829	0.002607	-0.016967	-0.015679
	0.019403	-0.016515	0.018884	0.016111	0.014145	-0.015845
	-0.020950					
z103	0.038662	0.003596	-0.013428	-0.008204	-0.022485	-0.008272
	-0.015473	0.001571	0.013659	0.016655	0.017902	0.047520
	-0.000699	0.004350	-0.014515	0.019086	-0.011741	0.001363
	-0.007075	-0.039938	-0.007034	-0.036305	-0.017573	-0.014877
	-0.011596	0.008645	0.000853	0.001296	-0.008850	-0.000359
	0.000538					
x104	0.011218	0.036237	0.022939	-0.015101	0.010657	0.019116
	0.019605	-0.028043	-0.000019	0.032286	-0.002267	0.016178
	-0.000146	-0.014626	-0.014254	0.002563	0.004748	-0.004741
	-0.002008	-0.005746	-0.005961	0.005747	-0.019853	0.012013
	0.003330	0.004572	-0.008449	0.010541	-0.006866	-0.001509
	-0.005968					
y104	-0.013388	0.003446	-0.011851	0.002820	0.006727	0.019775
	0.007019	-0.003525	0.002673	0.022723	0.040153	0.013280
	-0.006313	-0.002411	-0.022790	0.011969	-0.022960	-0.015847
	0.005060	0.003063	0.009661	0.001339	-0.014625	-0.016346
	0.018592	-0.016665	0.014998	0.020318	0.018049	-0.019788
	-0.027634					

z104	0.039267	0.004470	-0.010823	-0.008133	-0.022583	-0.006703
	-0.014880	0.001147	0.012068	0.018064	0.016575	0.047495
	-0.000573	0.004062	-0.015857	0.014200	-0.013520	0.000702
	-0.003875	-0.038672	-0.007549	-0.036153	-0.014889	-0.013208
	-0.015138	0.012108	-0.002365	0.000814	-0.008949	0.000900
	0.003370					
x105	0.009194	0.035439	0.024646	-0.012953	0.011509	0.020246
	0.022682	-0.026939	-0.000924	0.029446	0.001136	0.010469
	-0.001119	-0.012053	-0.010559	0.003809	0.007733	-0.003540
	-0.003089	-0.001188	-0.008191	0.005669	-0.020869	0.011797
	0.002730	0.002355	-0.005710	0.011136	-0.008275	-0.001856
	-0.009018					
y105	-0.012591	0.002801	-0.011542	0.004443	0.006951	0.018155
	0.005367	-0.002198	0.003364	0.021455	0.038346	0.013659
	-0.007734	0.001037	-0.023209	0.012354	-0.023282	-0.017392
	0.002705	0.001773	0.011607	0.000125	-0.014530	-0.016710
	0.016090	-0.019070	0.011691	0.021893	0.013907	-0.023233
	-0.032037					
z105	0.040489	0.005150	-0.007602	-0.007880	-0.022681	-0.005836
	-0.013085	0.000311	0.010781	0.017746	0.017711	0.045204
	-0.002100	0.005644	-0.017390	0.010248	-0.012608	-0.001010
	-0.003181	-0.034501	-0.009085	-0.036406	-0.016473	-0.013179
	-0.016845	0.012560	-0.000279	0.000255	-0.012042	0.001943
	-0.000036					
x106	0.007168	0.034904	0.026236	-0.011441	0.012184	0.021472
	0.025466	-0.026063	-0.001916	0.026523	0.004632	0.006016
	-0.001155	-0.010839	-0.007292	0.005313	0.009642	-0.002196
	-0.003942	0.003314	-0.010587	0.005357	-0.021291	0.009599
	0.002868	-0.000062	-0.007048	0.009594	-0.005816	-0.001124
	-0.009960					
y106	-0.011236	0.002490	-0.010429	0.006089	0.006864	0.016839
	0.003731	-0.001763	0.003236	0.020789	0.036787	0.015226
	-0.007216	0.002998	-0.024986	0.010549	-0.021587	-0.015537
	0.004849	0.000929	0.012261	-0.003355	-0.014344	-0.017357
	0.020443	-0.020236	-0.000939	0.018126	0.003776	-0.029328
	-0.026629					
z106	0.041476	0.006259	-0.004584	-0.007551	-0.022405	-0.004138
	-0.011421	-0.000613	0.009429	0.018737	0.019670	0.043355
	-0.002147	0.006402	-0.018450	0.007691	-0.013133	-0.002276
	-0.001155	-0.031524	-0.011287	-0.035895	-0.019598	-0.013090
	-0.016403	0.013010	-0.001687	-0.001789	-0.010517	0.003887
	-0.002813					

x107	0.005049	0.034219	0.027392	-0.009797	0.012777	0.022545
	0.027337	-0.024975	-0.003187	0.024689	0.007286	0.001107
	-0.001400	-0.008608	-0.005134	0.006451	0.012006	-0.001342
	-0.003742	0.007161	-0.013167	0.005499	-0.020218	0.009726
	0.003090	-0.001854	-0.006152	0.006833	-0.003692	0.000045
	-0.008917					
y107	-0.011123	0.001971	-0.010017	0.007754	0.006343	0.016102
	0.000832	0.000926	0.002968	0.021640	0.034968	0.014517
	-0.006377	0.005387	-0.025795	0.010548	-0.023894	-0.017658
	0.005275	-0.001471	0.014995	-0.005079	-0.011621	-0.016123
	0.018932	-0.019653	-0.006069	0.014816	0.008080	-0.029368
	-0.026615					
z107	0.041950	0.007449	-0.001740	-0.007045	-0.021823	-0.001960
	-0.010334	-0.000500	0.007388	0.022080	0.020805	0.040380
	-0.001351	0.008421	-0.018519	0.005727	-0.014358	-0.004088
	0.002656	-0.028448	-0.011663	-0.033812	-0.021217	-0.011253
	-0.014793	0.013269	-0.000363	-0.005632	-0.006662	0.006757
	-0.004294					
x108	0.003407	0.033348	0.027540	-0.008255	0.012694	0.023259
	0.029570	-0.024920	-0.004717	0.022714	0.009462	-0.003590
	0.000216	-0.007343	-0.002064	0.007826	0.013045	-0.001113
	-0.004819	0.008824	-0.016387	0.003644	-0.020057	0.008519
	0.006727	-0.001951	-0.000110	0.004672	-0.003106	-0.003588
	-0.012085					
y108	-0.011948	0.002673	-0.011760	0.009765	0.006228	0.015337
	-0.002232	0.002502	0.001634	0.022042	0.029655	0.013875
	-0.002363	0.005739	-0.018899	0.009322	-0.033761	-0.011898
	0.001060	-0.004905	0.014366	-0.008725	-0.009434	-0.015385
	0.012715	-0.014388	-0.005075	0.017443	0.011535	-0.027626
	-0.031332					
z108	0.041949	0.009160	0.000186	-0.006593	-0.020394	-0.000253
	-0.009032	-0.001164	0.005798	0.024750	0.021749	0.037373
	0.000183	0.010083	-0.014360	0.003298	-0.017101	-0.001563
	0.002565	-0.023275	-0.013215	-0.032933	-0.020737	-0.008955
	-0.015075	0.013245	0.002260	-0.004365	-0.007308	0.007312
	-0.006557					
x109	0.001207	0.032632	0.027880	-0.006988	0.012747	0.023732
	0.030869	-0.024692	-0.005592	0.019887	0.009347	-0.006927
	0.001145	-0.006679	0.000654	0.008738	0.014011	0.001277
	-0.005503	0.010488	-0.019455	0.002385	-0.017508	0.007699
	0.006803	-0.002838	0.001897	0.000536	-0.003010	-0.004364
	-0.012376					

y109	-0.011970	0.002176	-0.012575	0.010752	0.004377	0.015196
	-0.003632	0.004921	-0.000600	0.020200	0.023157	0.012406
	0.002141	0.008553	-0.016787	0.007148	-0.040887	-0.012144
	0.000273	-0.008693	0.016108	-0.013895	-0.002463	-0.016403
	0.006968	-0.013040	-0.007076	0.018186	0.008592	-0.031903
	-0.032918					
z109	0.041967	0.010100	0.002548	-0.006506	-0.019127	0.002024
	-0.006584	-0.001907	0.003335	0.027152	0.021772	0.034116
	0.001527	0.014073	-0.012755	0.001606	-0.020602	-0.002645
	0.004296	-0.018853	-0.014981	-0.031419	-0.017972	-0.007367
	-0.014779	0.010575	0.003940	-0.002949	-0.009784	0.002500
	-0.008344					
x110	-0.001351	0.031921	0.027509	-0.005677	0.012629	0.023579
	0.031122	-0.023859	-0.006563	0.016981	0.007185	-0.008802
	0.004629	-0.006868	0.004313	0.010278	0.012633	0.003772
	-0.006079	0.010458	-0.021687	0.000049	-0.013343	0.006188
	0.007134	-0.001561	0.004496	-0.002448	-0.002159	-0.005772
	-0.012386					
y110	-0.012485	0.001582	-0.010665	0.010753	0.003816	0.013961
	-0.006097	0.006925	0.001738	0.015490	0.022476	0.010733
	-0.002987	0.007978	-0.019989	0.005575	-0.037120	-0.010855
	0.001649	-0.008585	0.017593	-0.015412	0.000908	-0.015303
	-0.001057	-0.017778	-0.018236	0.014876	0.007765	-0.019298
	-0.030960					
z110	0.041277	0.010863	0.004205	-0.006975	-0.017131	0.004216
	-0.004167	-0.003246	0.001018	0.028206	0.022025	0.030234
	0.001978	0.017476	-0.011246	0.001972	-0.022353	-0.001679
	0.006782	-0.015101	-0.016581	-0.027775	-0.016811	-0.005402
	-0.011705	0.007281	0.004068	-0.003578	-0.011137	0.000028
	-0.007017					
x111	-0.004259	0.030754	0.027802	-0.003860	0.012710	0.022158
	0.030218	-0.022667	-0.006153	0.014944	0.005091	-0.008155
	0.008398	-0.006915	0.005565	0.009444	0.012529	0.003829
	-0.003489	0.009877	-0.021957	-0.003830	-0.010803	0.005555
	0.007635	0.000124	0.004773	-0.003704	-0.001719	-0.001822
	-0.012242					
y111	-0.013015	0.002221	-0.009709	0.010836	0.003481	0.012881
	-0.007657	0.008065	0.002972	0.010110	0.017146	0.010596
	-0.005243	0.004658	-0.019308	0.006734	-0.039438	-0.005310
	-0.004094	-0.010104	0.018739	-0.016365	0.002770	-0.017296
	-0.006747	-0.017607	-0.023573	0.012578	0.006125	-0.010941
	-0.027001					

z111	0.039911	0.010619	0.006089	-0.007429	-0.014551	0.007488
	-0.000009	-0.002750	-0.001972	0.026333	0.024556	0.024796
	0.002017	0.019607	-0.009412	0.006254	-0.018351	-0.008927
	0.013625	-0.011581	-0.015210	-0.019113	-0.009397	-0.006612
	-0.013049	-0.002658	0.001777	-0.010292	-0.009578	-0.007517
	-0.006669					
x112	-0.009746	0.029743	0.024949	0.000159	0.013922	0.021201
	0.030084	-0.021591	-0.005130	0.008709	0.000148	-0.006409
	0.012053	-0.006664	0.006690	0.005238	0.013072	0.003911
	0.006283	0.010868	-0.020716	-0.004087	-0.000860	0.003962
	0.008531	-0.000669	-0.003006	-0.008727	0.005976	0.003546
	-0.011756					
y112	-0.013259	0.000895	-0.008437	0.011371	0.003817	0.012024
	-0.006403	0.010482	0.003830	0.013266	0.006447	0.014760
	-0.002289	0.006569	-0.018783	0.004116	-0.033250	0.000512
	-0.002874	-0.004720	0.014795	-0.022550	0.000156	-0.012516
	-0.000662	-0.009464	-0.022023	0.008657	0.001589	-0.007095
	-0.022712					
z112	0.034098	0.011159	0.011455	-0.008757	-0.009439	0.007983
	0.005980	-0.000555	-0.003377	0.018070	0.025747	0.016076
	0.002009	0.019515	-0.000818	0.018107	-0.017962	-0.011831
	0.012607	-0.003877	-0.012069	-0.006795	-0.005801	-0.007649
	-0.013983	-0.009080	0.001481	-0.018010	-0.007949	-0.008239
	-0.000169					
x113	-0.013421	0.028708	0.022211	0.001315	0.013340	0.019474
	0.030922	-0.022156	-0.004308	0.003704	-0.002450	-0.007138
	0.012949	-0.007843	0.008282	0.002293	0.012271	0.002709
	0.012495	0.011471	-0.020787	-0.006708	0.007126	0.003051
	0.013261	0.001523	-0.002666	-0.016594	0.018006	0.010279
	-0.011995					
y113	-0.015033	-0.000910	-0.006991	0.013155	0.003286	0.014093
	-0.003128	0.012976	0.002585	0.012608	-0.000970	0.015365
	0.006302	0.010921	-0.019728	-0.003890	-0.019947	-0.002004
	0.004841	-0.001114	0.014773	-0.021264	0.004312	-0.009008
	0.000960	-0.006421	-0.026185	0.007613	-0.009435	-0.006539
	-0.024135					
z113	0.027932	0.012151	0.014315	-0.008040	-0.004275	0.007718
	0.009252	0.004205	-0.003336	0.012061	0.025184	0.010326
	0.002391	0.020645	0.005341	0.026588	-0.017925	-0.011973
	0.011756	0.004480	-0.011504	0.003464	-0.004239	-0.007480
	-0.010681	-0.009558	-0.002953	-0.021648	-0.004367	-0.003593
	0.003405					

x114	-0.014883	0.027123	0.020365	0.002669	0.013831	0.018169
	0.032373	-0.022719	-0.005466	0.000448	0.000361	-0.006659
	0.011019	-0.007931	0.008417	-0.000718	0.022331	0.003832
	0.019944	0.015401	-0.027858	-0.006941	0.012600	0.006495
	0.028730	0.001373	-0.004094	-0.024158	0.018955	0.014964
	-0.009767					
y114	-0.016646	-0.002999	-0.006036	0.012423	0.000077	0.014316
	-0.002002	0.010048	0.003446	0.011722	-0.013111	0.015849
	0.008189	0.011966	-0.024921	-0.012853	-0.020840	-0.008360
	0.001798	-0.000573	0.017958	-0.026442	0.015315	-0.007452
	-0.012351	0.001574	-0.020783	0.004559	-0.018026	-0.014171
	-0.024815					
z114	0.022836	0.012880	0.015400	-0.007237	-0.000604	0.006864
	0.010888	0.008902	-0.003075	0.008922	0.023513	0.005152
	0.001192	0.022359	0.010015	0.029843	-0.020101	-0.011453
	0.011701	0.009326	-0.011604	0.009467	-0.001305	-0.008376
	-0.008603	-0.008553	-0.007212	-0.026744	-0.001449	0.000774
	0.005861					
x115	-0.014669	0.024923	0.017960	0.002814	0.013311	0.016846
	0.033931	-0.025063	-0.006522	-0.001976	0.002246	-0.007318
	0.005312	-0.002844	0.007955	-0.005176	0.030355	0.002317
	0.020063	0.018814	-0.031997	-0.010313	0.017240	0.005803
	0.038469	0.006977	-0.001082	-0.029417	0.011971	0.014609
	-0.014912					
y115	-0.017962	-0.002572	-0.004661	0.012361	-0.002762	0.015834
	-0.000021	0.008098	0.005052	0.011074	-0.015884	0.012602
	0.015998	0.011230	-0.025326	-0.012293	-0.013072	-0.005340
	0.005239	0.000392	0.020407	-0.025929	0.013077	0.000082
	-0.006261	0.002672	-0.019115	0.003806	-0.015577	-0.001816
	-0.022501					
z115	0.018259	0.013355	0.015152	-0.006410	0.002199	0.006411
	0.011669	0.012400	-0.002673	0.007959	0.021823	0.000401
	-0.000723	0.023789	0.011756	0.030749	-0.020005	-0.010220
	0.012501	0.012352	-0.013365	0.012213	0.001058	-0.009791
	-0.004386	-0.007884	-0.011972	-0.030541	-0.002178	0.005778
	0.004965					
x116	-0.014109	0.022981	0.015275	0.002739	0.012513	0.016469
	0.034185	-0.027878	-0.006581	-0.003916	0.003326	-0.007647
	0.001578	0.001424	0.005134	-0.004995	0.032303	-0.001768
	0.015219	0.019201	-0.032257	-0.014296	0.023302	0.004691
	0.040262	0.013290	0.000134	-0.030496	0.005721	0.016116
	-0.021137					

y116	-0.017935	-0.002006	-0.002587	0.011800	-0.004193	0.015854
	0.002348	0.006618	0.006473	0.009934	-0.014891	0.008924
	0.020986	0.010955	-0.021788	-0.011483	-0.001068	0.000473
	0.008398	0.004038	0.019194	-0.022867	0.005186	0.007657
	0.008177	0.003359	-0.015392	0.005060	-0.013794	0.009189
	-0.017295					
z116	0.014507	0.013020	0.014418	-0.006281	0.003438	0.005733
	0.011391	0.012635	-0.003320	0.007924	0.018008	-0.001555
	-0.005131	0.022891	0.007993	0.027945	-0.021781	-0.011811
	0.010881	0.013185	-0.016953	0.010390	0.009025	-0.011773
	-0.005460	-0.005129	-0.013722	-0.033848	-0.010151	0.001343
	0.002166					
x117	-0.013023	0.021511	0.013422	0.002293	0.012990	0.017165
	0.033551	-0.027486	-0.005433	-0.004427	0.004364	-0.010410
	0.001462	0.005538	0.005705	-0.003885	0.036862	-0.003605
	0.014209	0.020276	-0.030883	-0.015387	0.022651	0.009110
	0.046616	0.018296	-0.002238	-0.025655	-0.002330	0.025385
	-0.023127					
y117	-0.017849	-0.003220	0.000071	0.009954	-0.004956	0.012719
	0.004067	0.000226	0.006756	0.006257	-0.014706	0.010819
	0.018072	0.005518	-0.024883	-0.009676	-0.000980	-0.001919
	0.000756	0.008340	0.014670	-0.021370	0.012649	0.010112
	0.009187	0.005782	-0.006054	0.001073	-0.012609	0.002159
	-0.010354					
z117	0.011456	0.013828	0.012856	-0.005438	0.004457	0.006541
	0.011294	0.015387	-0.003328	0.009106	0.016622	-0.007571
	-0.005599	0.025590	0.006904	0.025004	-0.016370	-0.007355
	0.014895	0.013170	-0.019096	0.008759	0.005430	-0.012158
	0.002826	-0.003717	-0.020065	-0.032100	-0.018675	0.009672
	-0.004648					
x118	-0.011300	0.019722	0.011971	0.000449	0.013121	0.017745
	0.031394	-0.026969	-0.003375	-0.004998	0.004296	-0.011304
	0.001074	0.008352	0.007466	-0.001175	0.038770	-0.006501
	0.009496	0.018337	-0.027468	-0.018562	0.021725	0.014037
	0.047653	0.018916	-0.004304	-0.022319	-0.009065	0.028244
	-0.023106					
y118	-0.018215	-0.002851	0.002663	0.009527	-0.003882	0.010408
	0.006834	-0.004589	0.008790	0.002357	-0.009042	0.007365
	0.019977	0.003153	-0.022376	-0.001791	0.001180	-0.000491
	-0.002097	0.012997	0.013454	-0.016475	0.011367	0.013473
	0.020500	0.008043	-0.000878	-0.001652	-0.002080	0.014048
	-0.008200					

z118	0.008590	0.013594	0.010942	-0.005889	0.004363	0.006682
	0.010661	0.016450	-0.004205	0.010060	0.013042	-0.010041
	-0.006423	0.026685	0.002925	0.019283	-0.012330	-0.005429
	0.016340	0.010107	-0.022669	0.004310	0.002844	-0.013460
	0.005915	-0.002797	-0.025305	-0.028827	-0.032189	0.010127
	-0.011254					
x119	-0.008409	0.018603	0.010211	-0.002115	0.011315	0.018153
	0.029320	-0.025948	0.000258	-0.006320	0.006057	-0.012660
	0.001674	0.010745	0.012750	0.006296	0.036071	-0.009547
	-0.000238	0.014190	-0.020694	-0.022033	0.020849	0.018702
	0.044013	0.016081	-0.003118	-0.025190	-0.006465	0.030996
	-0.019597					
y119	-0.020107	-0.002611	0.004822	0.009469	-0.001916	0.009820
	0.009663	-0.010300	0.010045	-0.000562	-0.005491	0.002933
	0.023637	0.003644	-0.018988	0.009017	0.003830	-0.001136
	-0.002617	0.015144	0.011963	-0.013359	0.010425	0.017856
	0.030784	0.014624	0.001852	-0.005518	0.000574	0.026793
	-0.003269					
z119	0.005672	0.013267	0.008683	-0.007917	0.001511	0.006075
	0.012325	0.017173	-0.002328	0.010349	0.014234	-0.010684
	-0.003114	0.024964	0.000970	0.015872	-0.012242	-0.007126
	0.010628	0.005644	-0.024054	0.001496	-0.004589	-0.017439
	0.003368	-0.005329	-0.027082	-0.023533	-0.024100	0.016969
	-0.025392					
x120	-0.004559	0.018871	0.008180	-0.003304	0.010653	0.018051
	0.026214	-0.024405	-0.000446	-0.008977	0.006760	-0.015670
	0.003698	0.012693	0.015391	0.017002	0.030130	-0.010194
	-0.009075	0.014464	-0.011283	-0.019951	0.019689	0.019312
	0.041987	0.012128	0.000038	-0.031053	0.000586	0.035143
	-0.016922					
y120	-0.021437	-0.002078	0.005867	0.009897	-0.000694	0.010685
	0.013273	-0.015387	0.007788	-0.003847	-0.000969	-0.000267
	0.031893	0.005598	-0.015967	0.018708	0.001488	-0.002807
	-0.003741	0.017980	0.011588	-0.008596	0.010676	0.018356
	0.042214	0.023377	0.003519	-0.014673	0.003156	0.043140
	0.004994					
z120	0.003238	0.011256	0.003860	-0.009077	-0.000118	0.005100
	0.012456	0.017877	-0.002674	0.007274	0.012760	-0.011808
	0.004539	0.022616	-0.002805	0.013437	-0.010704	-0.001124
	0.013013	0.001661	-0.021569	0.002065	-0.013308	-0.020091
	0.007844	-0.008631	-0.025179	-0.022188	-0.024538	0.011414
	-0.035933					

x121	-0.001485	0.018378	0.006461	-0.002623	0.010568	0.017339
	0.026105	-0.023485	-0.003146	-0.007878	0.004486	-0.013801
	0.003616	0.011627	0.010070	0.014247	0.030470	-0.011563
	-0.019276	0.015273	-0.007964	-0.014490	0.024167	0.017209
	0.041432	0.015350	-0.001465	-0.033135	0.000488	0.034412
	-0.013198					
y121	-0.021930	-0.003562	0.005343	0.010197	-0.000509	0.011042
	0.018213	-0.017674	0.004439	-0.004284	0.000843	0.001444
	0.036024	0.004497	-0.017783	0.013536	0.005034	-0.003833
	-0.005680	0.020320	0.011728	-0.000847	0.016924	0.018359
	0.051550	0.029334	0.007864	-0.023814	-0.006030	0.048994
	0.013381					
z121	0.002412	0.010344	0.001572	-0.009991	-0.002199	0.004784
	0.012790	0.013987	-0.004200	0.001199	0.009944	-0.011850
	0.010272	0.024926	-0.004597	0.016222	-0.013367	-0.000813
	0.018006	-0.001681	-0.020466	0.005458	-0.019097	-0.027071
	0.004724	-0.008944	-0.022583	-0.021734	-0.033839	0.009728
	-0.033925					
x122	0.001183	0.018267	0.004930	-0.002781	0.010991	0.016364
	0.025603	-0.022261	-0.002371	-0.007355	0.001980	-0.012973
	0.004601	0.011906	0.009903	0.013599	0.031105	-0.010809
	-0.025549	0.013351	-0.003098	-0.011454	0.023220	0.016495
	0.041316	0.015985	-0.001285	-0.032344	0.000687	0.033247
	-0.010207					
y122	-0.022725	-0.005251	0.003501	0.009515	0.000534	0.010987
	0.022166	-0.019871	0.004295	-0.004659	0.002849	0.001578
	0.037934	0.002564	-0.015470	0.008946	0.009307	-0.005245
	-0.005115	0.021932	0.012825	0.003590	0.020336	0.017948
	0.052965	0.031679	0.007474	-0.025697	-0.008053	0.050919
	0.018576					
z122	0.002527	0.009431	-0.002010	-0.010211	-0.004229	0.004349
	0.009849	0.013018	-0.006523	-0.002560	0.005739	-0.011507
	0.009649	0.024859	-0.010792	0.017915	-0.018844	-0.006013
	0.015583	-0.004882	-0.019911	0.006516	-0.017750	-0.034680
	-0.007690	-0.009782	-0.023809	-0.016684	-0.030610	0.007130
	-0.035396					
x123	0.003832	0.017975	0.003530	-0.003179	0.011186	0.015165
	0.025213	-0.021994	-0.002725	-0.007244	-0.001661	-0.011007
	0.004263	0.011369	0.008342	0.010886	0.029834	-0.011720
	-0.031727	0.010581	0.000595	-0.010077	0.023061	0.013517
	0.036500	0.016554	-0.000856	-0.031320	-0.002462	0.028969
	-0.005850					

y123	-0.023513	-0.006894	0.001205	0.009712	0.001859	0.010667
	0.024560	-0.020001	0.003833	-0.004687	0.005457	0.000532
	0.037929	0.001067	-0.012710	0.004379	0.012896	-0.005718
	-0.005225	0.022355	0.012611	0.004843	0.023047	0.018700
	0.052453	0.033822	0.008163	-0.025508	-0.010148	0.047122
	0.024989					
z123	0.002917	0.008791	-0.005759	-0.009143	-0.005905	0.004312
	0.005698	0.014591	-0.007147	-0.004278	0.002928	-0.013128
	0.008037	0.024295	-0.014385	0.021249	-0.019107	-0.008176
	0.011125	-0.007535	-0.016768	0.009705	-0.017768	-0.037140
	-0.014392	-0.011218	-0.022758	-0.010651	-0.021875	0.000454
	-0.037674					
x124	0.006551	0.017991	0.001880	-0.003582	0.011561	0.014258
	0.024629	-0.021337	-0.003496	-0.007333	-0.004466	-0.009923
	0.003242	0.011177	0.007329	0.009402	0.027493	-0.012780
	-0.037799	0.007205	0.004508	-0.009139	0.021064	0.009254
	0.030613	0.015680	-0.000774	-0.028611	-0.003385	0.025174
	-0.004138					
y124	-0.024283	-0.007971	-0.000833	0.010139	0.003276	0.010658
	0.025843	-0.018791	0.002801	-0.004211	0.009095	-0.001351
	0.036685	-0.000119	-0.009338	0.000418	0.016086	-0.005219
	-0.004706	0.020848	0.012235	0.003762	0.024256	0.018963
	0.051071	0.035086	0.007690	-0.024566	-0.013218	0.042284
	0.032983					
z124	0.003469	0.007895	-0.009097	-0.008596	-0.007612	0.004147
	0.001499	0.016057	-0.008290	-0.004502	0.000878	-0.013395
	0.005533	0.022427	-0.018522	0.023237	-0.018042	-0.009767
	0.006438	-0.010450	-0.011239	0.009441	-0.013334	-0.036678
	-0.019078	-0.012751	-0.021786	-0.009548	-0.022762	-0.005693
	-0.034617					
x125	0.009399	0.018175	-0.000026	-0.003907	0.011959	0.013555
	0.023655	-0.019934	-0.005049	-0.008187	-0.007400	-0.008998
	0.001096	0.011161	0.006166	0.008734	0.024558	-0.014339
	-0.044266	0.003706	0.008145	-0.008289	0.019050	0.003475
	0.024250	0.014254	-0.001917	-0.025711	-0.003570	0.019459
	-0.003629					
y125	-0.025105	-0.008621	-0.002151	0.010708	0.004816	0.011191
	0.026638	-0.017143	0.001618	-0.003628	0.012791	-0.003495
	0.033869	-0.000563	-0.005902	-0.002470	0.016966	-0.005204
	-0.004433	0.018625	0.011205	0.001783	0.023342	0.017317
	0.048180	0.035734	0.006996	-0.023126	-0.015023	0.036950
	0.038903					

z125	0.004000	0.006400	-0.011603	-0.007495	-0.008332	0.003665
	-0.001655	0.015893	-0.008590	-0.006932	-0.000777	-0.013600
	0.003311	0.018616	-0.021920	0.025326	-0.013531	-0.012344
	0.002033	-0.010585	-0.007872	0.010962	-0.009994	-0.034901
	-0.017827	-0.011730	-0.019659	-0.010880	-0.023903	-0.009701
	-0.031245					
x126	0.012211	0.018555	-0.002120	-0.004022	0.012100	0.012975
	0.022471	-0.018228	-0.007004	-0.010140	-0.010028	-0.008372
	-0.001689	0.011281	0.004692	0.008377	0.020324	-0.016484
	-0.050416	-0.000118	0.011851	-0.008318	0.017745	-0.002956
	0.017719	0.011288	-0.003398	-0.023121	-0.004309	0.011915
	-0.004970					
y126	-0.026034	-0.008731	-0.002695	0.011196	0.005940	0.012581
	0.026888	-0.015421	0.000365	-0.002658	0.016430	-0.004998
	0.029473	-0.000094	-0.003457	-0.004013	0.014409	-0.005571
	-0.005220	0.015945	0.009022	-0.001006	0.022380	0.013879
	0.043514	0.034731	0.005983	-0.021066	-0.015403	0.030247
	0.042316					
z126	0.004067	0.005543	-0.013968	-0.005997	-0.010242	0.003641
	-0.005046	0.015868	-0.005676	-0.008975	0.000933	-0.015195
	0.002431	0.015903	-0.024702	0.025648	-0.007793	-0.013048
	-0.001632	-0.009035	-0.004732	0.012252	-0.007118	-0.031181
	-0.010691	-0.015156	-0.013284	-0.011624	-0.025962	-0.008562
	-0.029510					
x127	0.015155	0.018766	-0.004117	-0.004249	0.012658	0.012236
	0.021916	-0.015525	-0.009660	-0.012181	-0.012296	-0.007352
	-0.005786	0.011917	0.004555	0.008842	0.016340	-0.017286
	-0.056265	-0.003668	0.015324	-0.008524	0.016510	-0.009674
	0.009800	0.007891	-0.004988	-0.020953	-0.004044	0.003432
	-0.006169					
y127	-0.026950	-0.008407	-0.002397	0.011397	0.006879	0.014839
	0.027127	-0.013701	-0.001410	-0.001482	0.020368	-0.005941
	0.023436	0.001255	-0.001587	-0.003409	0.008955	-0.005509
	-0.006016	0.012394	0.006056	-0.003932	0.020458	0.008560
	0.037969	0.032285	0.002632	-0.018891	-0.012952	0.024072
	0.042905					
z127	0.004279	0.003928	-0.015421	-0.006029	-0.011381	0.003082
	-0.006920	0.016968	-0.002023	-0.009166	0.004444	-0.015467
	-0.000651	0.014968	-0.023026	0.027593	-0.001122	-0.011290
	-0.007230	-0.005199	-0.003379	0.015280	-0.004092	-0.026488
	-0.008190	-0.020567	-0.002361	-0.012692	-0.023349	-0.002515
	-0.027868					

x128	0.017546	0.019923	-0.005631	-0.003663	0.013044	0.011987
	0.021815	-0.012082	-0.011169	-0.014609	-0.013009	-0.007777
	-0.008221	0.013952	0.004499	0.010161	0.012887	-0.016363
	-0.058426	-0.006991	0.019378	-0.009205	0.013577	-0.016034
	0.006642	0.005089	-0.010414	-0.016842	-0.001896	-0.002452
	-0.006361					
y128	-0.027833	-0.007964	-0.001378	0.011287	0.007325	0.017146
	0.027515	-0.012174	-0.003496	-0.000794	0.024539	-0.006170
	0.016046	0.003255	-0.001393	-0.000652	0.001556	-0.006362
	-0.007060	0.009353	0.003506	-0.007552	0.018140	0.002624
	0.032905	0.030244	-0.003728	-0.016023	-0.009621	0.017624
	0.040518					
z128	0.005232	0.002552	-0.015785	-0.007267	-0.012015	0.003082
	-0.006088	0.019731	0.000534	-0.008075	0.013424	-0.015465
	-0.004373	0.017849	-0.017079	0.034891	0.007348	-0.006697
	-0.013964	0.001423	-0.004384	0.016792	-0.005307	-0.020841
	-0.004661	-0.026542	0.004006	-0.012081	-0.011736	0.012095
	-0.027660					
x129	0.029753	0.008979	-0.013267	-0.016692	0.019208	0.010402
	-0.007225	-0.022429	-0.045267	-0.009747	-0.003351	-0.045755
	-0.013462	0.022594	0.022464	0.017384	0.029177	-0.024307
	-0.029914	-0.026320	0.031994	-0.051896	0.008278	-0.006603
	0.004830	-0.038104	0.027501	-0.025519	0.006095	0.035740
	-0.013559					
y129	-0.033134	-0.017513	0.004270	0.007891	0.012629	0.012213
	0.022958	-0.017008	-0.014646	0.010098	0.019735	-0.003517
	0.017349	0.013412	0.003623	0.003537	0.006662	0.012301
	-0.003993	-0.014941	0.008034	-0.018586	0.018927	0.015081
	0.010227	-0.017627	-0.005569	-0.027487	0.030178	0.004716
	0.002334					
z129	0.002723	0.002360	-0.028156	-0.003224	-0.020800	0.008782
	-0.000881	0.018214	0.001852	-0.022647	0.018210	-0.013425
	-0.022823	0.020259	-0.015714	0.036581	-0.001688	0.020021
	-0.041513	0.000132	-0.004613	0.032429	0.006424	-0.019524
	-0.016380	0.004975	0.019057	-0.008348	-0.002817	0.031265
	-0.014781					
x130	0.028452	0.011272	-0.011560	-0.014533	0.018902	0.012136
	-0.005460	-0.020943	-0.040091	-0.011066	-0.002499	-0.041182
	-0.012061	0.021426	0.019618	0.017703	0.025220	-0.024433
	-0.025170	-0.021723	0.030977	-0.045236	0.007459	-0.010995
	0.007291	-0.031840	0.023267	-0.026569	0.004073	0.031740
	-0.009337					

y130	-0.033132	-0.017280	0.004310	0.008737	0.012844	0.014064
	0.024863	-0.016346	-0.015474	0.009850	0.020464	-0.000699
	0.014801	0.012097	0.001093	0.001712	0.001134	0.011351
	-0.008239	-0.012252	0.005794	-0.015910	0.022226	0.008735
	0.002220	-0.015049	-0.007340	-0.031784	0.028157	-0.000554
	0.002631					
z130	0.003852	0.000112	-0.029005	-0.005753	-0.020890	0.009284
	-0.002429	0.016719	0.000329	-0.019681	0.020320	-0.014199
	-0.022562	0.021740	-0.010498	0.038195	0.001442	0.017522
	-0.040530	-0.004419	-0.003972	0.029157	0.005319	-0.014986
	-0.015476	0.006840	0.022739	-0.001906	-0.007303	0.034908
	-0.010632					
x131	0.027072	0.013515	-0.009936	-0.011891	0.017677	0.013585
	-0.005323	-0.021465	-0.033493	-0.013304	-0.004091	-0.037490
	-0.010174	0.017872	0.011605	0.014370	0.018116	-0.027120
	-0.021947	-0.019237	0.030664	-0.040166	0.011576	-0.014971
	0.009775	-0.026189	0.021349	-0.023696	0.001060	0.022797
	-0.009706					
y131	-0.033307	-0.016100	0.004068	0.008892	0.013201	0.015449
	0.027035	-0.014588	-0.016576	0.008493	0.022836	0.000109
	0.012920	0.012771	0.001320	0.003957	-0.002018	0.010863
	-0.007956	-0.010206	0.005952	-0.014924	0.019017	0.002390
	-0.001497	-0.012864	-0.011039	-0.035829	0.025930	-0.001782
	0.007123					
z131	0.004669	-0.001206	-0.029605	-0.007258	-0.021243	0.009070
	-0.004077	0.014855	0.000011	-0.019261	0.021991	-0.015504
	-0.021141	0.022662	-0.007706	0.038851	0.002565	0.013735
	-0.039250	-0.008569	-0.003978	0.024908	0.004886	-0.011362
	-0.013491	0.007466	0.025208	0.003923	-0.010402	0.036775
	-0.008739					
x132	0.025509	0.015933	-0.008690	-0.010091	0.018159	0.014659
	-0.003278	-0.019606	-0.028978	-0.013372	-0.005063	-0.032565
	-0.009898	0.015661	0.008853	0.012508	0.014833	-0.027625
	-0.018045	-0.013200	0.029175	-0.031576	0.011842	-0.016627
	0.012655	-0.019092	0.020271	-0.022903	-0.001778	0.016446
	-0.013314					
y132	-0.032813	-0.016041	0.004030	0.008531	0.012794	0.018433
	0.028093	-0.013971	-0.016924	0.007277	0.024381	0.002476
	0.011179	0.012152	-0.000988	0.004548	-0.008188	0.010106
	-0.008780	-0.011663	0.005305	-0.016402	0.020502	-0.005380
	-0.009159	-0.011727	-0.012504	-0.041481	0.023083	-0.003683
	0.017126					

z132	0.005944	-0.003378	-0.029798	-0.009235	-0.021044	0.008744
	-0.005017	0.014173	-0.001607	-0.018191	0.023957	-0.014926
	-0.019737	0.023310	-0.003232	0.039525	0.004043	0.011603
	-0.037707	-0.010988	-0.004495	0.020590	0.003402	-0.007646
	-0.014256	0.006511	0.027503	0.007430	-0.010666	0.035042
	-0.002162					
x133	0.024871	0.017478	-0.007535	-0.009691	0.018773	0.016325
	-0.000701	-0.018084	-0.026662	-0.010648	-0.005350	-0.024535
	-0.011365	0.013273	0.009045	0.013545	0.011596	-0.026837
	-0.016831	-0.011197	0.021736	-0.024008	0.013538	-0.015256
	0.010066	-0.011726	0.015776	-0.025735	-0.006193	0.013417
	-0.011129					
y133	-0.032662	-0.015712	0.003419	0.008272	0.012923	0.021325
	0.027676	-0.013720	-0.016434	0.005541	0.024896	0.001711
	0.010360	0.012136	-0.004491	0.002258	-0.012016	0.009631
	-0.006957	-0.011413	0.009974	-0.017114	0.021310	-0.014286
	-0.011572	-0.009732	-0.008547	-0.041876	0.022179	-0.006329
	0.017710					
z133	0.007005	-0.004646	-0.029343	-0.010080	-0.021102	0.008093
	-0.006219	0.013716	-0.001965	-0.018356	0.025231	-0.013580
	-0.017393	0.023244	-0.002174	0.039901	0.004153	0.009732
	-0.035417	-0.012527	-0.003532	0.016161	0.001483	-0.005253
	-0.013616	0.004993	0.029471	0.011323	-0.007937	0.029609
	0.005861					
x134	0.024681	0.018305	-0.006508	-0.009810	0.018878	0.018018
	0.000710	-0.016714	-0.025299	-0.006043	-0.007904	-0.017167
	-0.013693	0.011378	0.008543	0.012611	0.009676	-0.023477
	-0.017982	-0.010171	0.016598	-0.017421	0.016406	-0.014087
	0.005225	-0.005244	0.012928	-0.029591	-0.011274	0.010713
	-0.008505					
y134	-0.032999	-0.013969	0.002818	0.008776	0.013578	0.022908
	0.028002	-0.013685	-0.014355	0.004209	0.028672	0.000736
	0.011486	0.012717	-0.005506	0.005062	-0.016973	0.005366
	-0.003343	-0.011340	0.009823	-0.020629	0.016258	-0.019297
	-0.006073	-0.007760	-0.007395	-0.037983	0.020956	-0.004686
	0.014025					
z134	0.007974	-0.005008	-0.029568	-0.010248	-0.021776	0.006983
	-0.006622	0.013228	-0.001828	-0.021934	0.025998	-0.015317
	-0.014266	0.023805	-0.002478	0.039571	0.004475	0.007158
	-0.030761	-0.013327	-0.002790	0.011513	-0.001237	-0.003982
	-0.013423	0.000246	0.027389	0.015260	-0.006593	0.030707
	0.013214					

x135	0.024737	0.019086	-0.005361	-0.009648	0.018860	0.020052
	0.002110	-0.017159	-0.024227	-0.000938	-0.010150	-0.010588
	-0.014998	0.010256	0.007023	0.012815	0.009084	-0.025432
	-0.016512	-0.011549	0.010828	-0.012995	0.020863	-0.014306
	0.001276	-0.003871	0.011279	-0.033157	-0.012465	0.008609
	-0.006445					
y135	-0.033395	-0.012911	0.001605	0.008203	0.013701	0.024579
	0.028055	-0.011878	-0.012606	0.002533	0.031596	-0.000457
	0.011271	0.011662	-0.005642	0.004773	-0.021583	0.005268
	-0.002179	-0.010696	0.013628	-0.020611	0.011864	-0.021832
	-0.000403	-0.001000	-0.004074	-0.033908	0.014411	-0.003680
	0.008071					
z135	0.008907	-0.005358	-0.029361	-0.010039	-0.021961	0.005816
	-0.006999	0.013424	-0.001314	-0.023543	0.026437	-0.015650
	-0.011113	0.023061	-0.001762	0.038350	0.007129	0.006773
	-0.027733	-0.014445	-0.003783	0.009615	-0.003979	-0.002746
	-0.015049	-0.007982	0.025712	0.015114	-0.002635	0.031545
	0.016900					
x136	0.024394	0.020598	-0.004611	-0.009868	0.017773	0.021731
	0.002519	-0.018083	-0.020654	0.002030	-0.010901	-0.006715
	-0.014026	0.007249	0.002568	0.013251	0.004922	-0.029251
	-0.012099	-0.013374	0.009110	-0.014695	0.021636	-0.015377
	0.003998	0.000042	0.009531	-0.030024	-0.012574	0.007216
	-0.004004					
y136	-0.033163	-0.012386	0.000316	0.007540	0.014398	0.026743
	0.028241	-0.010778	-0.013592	0.001937	0.032019	-0.000154
	0.008379	0.011978	-0.004877	0.003009	-0.022708	0.004587
	-0.003376	-0.012996	0.015405	-0.015603	0.013172	-0.022847
	-0.000492	0.003326	0.000017	-0.035563	0.004539	-0.004703
	0.002353					
z136	0.009769	-0.005632	-0.028441	-0.009602	-0.021719	0.004074
	-0.007339	0.013983	-0.000056	-0.022567	0.027004	-0.013504
	-0.008501	0.022218	-0.001521	0.038501	0.008362	0.007005
	-0.027024	-0.012755	-0.004619	0.007974	-0.007159	-0.002174
	-0.015406	-0.010696	0.024296	0.016022	0.001262	0.027549
	0.018647					
x137	0.024340	0.022453	-0.003304	-0.009576	0.016289	0.023667
	0.003381	-0.017748	-0.017788	0.006271	-0.011154	-0.001888
	-0.012579	0.006387	0.001206	0.013475	0.000572	-0.028197
	-0.010892	-0.014634	0.007953	-0.012517	0.023068	-0.014432
	0.005799	0.007279	0.009752	-0.027536	-0.015270	0.003449
	-0.005084					

y137	-0.032758	-0.012682	-0.001247	0.006289	0.015182	0.028148
	0.026592	-0.011510	-0.014813	0.001665	0.031838	0.000227
	0.004714	0.009340	-0.006349	0.001524	-0.023429	0.000048
	-0.003623	-0.017372	0.014230	-0.016401	0.013225	-0.025031
	-0.000378	0.001812	0.004164	-0.035863	0.000029	-0.003441
	0.000483					
z137	0.010751	-0.005917	-0.027935	-0.009323	-0.020859	0.002347
	-0.006850	0.014463	0.001246	-0.021189	0.028090	-0.011825
	-0.006011	0.022301	-0.000980	0.039150	0.009856	0.006905
	-0.024620	-0.009679	-0.005541	0.004996	-0.011627	-0.002107
	-0.015365	-0.012211	0.021355	0.017281	0.004992	0.026603
	0.021215					
x138	0.024619	0.023748	-0.002752	-0.010620	0.015594	0.025703
	0.005193	-0.017428	-0.016701	0.011029	-0.013148	0.000325
	-0.011683	0.004724	0.000650	0.013410	-0.000584	-0.030463
	-0.008117	-0.019971	0.008936	-0.009962	0.022574	-0.009115
	0.007667	0.007844	0.011305	-0.030116	-0.007065	0.001039
	0.001058					
y138	-0.032316	-0.013070	-0.002140	0.006358	0.014670	0.029362
	0.025176	-0.010703	-0.014999	0.001883	0.034543	0.000460
	0.003743	0.011660	-0.007007	0.002419	-0.025810	-0.002245
	-0.002991	-0.019482	0.014522	-0.016916	0.010942	-0.027746
	0.002651	0.003203	0.004095	-0.034701	-0.004256	0.003297
	-0.002091					
z138	0.011922	-0.005817	-0.027946	-0.009323	-0.020052	0.000729
	-0.006222	0.014109	0.002044	-0.021250	0.027953	-0.009245
	-0.005273	0.020698	-0.001089	0.038399	0.010798	0.007020
	-0.023078	-0.005288	-0.007478	0.002640	-0.012290	-0.004885
	-0.019689	-0.012940	0.018088	0.019727	0.000522	0.024532
	0.018907					
x139	0.024475	0.026250	-0.001871	-0.010863	0.014059	0.027166
	0.005149	-0.017531	-0.012607	0.015233	-0.013360	0.002692
	-0.010528	0.001677	-0.004516	0.013613	0.000656	-0.029372
	-0.003936	-0.021726	0.008739	-0.006555	0.023054	-0.006141
	0.004669	0.007481	0.003119	-0.022944	-0.004840	-0.004312
	0.004218					
y139	-0.031393	-0.012531	-0.003245	0.004947	0.014325	0.029859
	0.024246	-0.010863	-0.014582	0.003526	0.039334	0.000808
	0.001186	0.014017	-0.008518	0.002957	-0.024706	-0.006031
	0.001463	-0.018460	0.010364	-0.014294	0.006266	-0.026583
	-0.000858	0.000830	0.008909	-0.034080	0.000840	0.009741
	-0.001048					

z139	0.013815	-0.006325	-0.028091	-0.009297	-0.019683	-0.001143
	-0.006254	0.014752	0.002755	-0.020366	0.025318	-0.005571
	-0.003560	0.018362	-0.000115	0.037531	0.011619	0.006285
	-0.022507	-0.004579	-0.006058	-0.002319	-0.012490	-0.006705
	-0.018089	-0.011454	0.016015	0.017414	-0.005544	0.022680
	0.017920					
x140	0.024305	0.027977	0.000290	-0.012159	0.014369	0.027420
	0.006331	-0.017951	-0.009111	0.019578	-0.014012	0.007137
	-0.010156	-0.001490	-0.007885	0.014883	0.002415	-0.028515
	0.000592	-0.020918	0.003809	-0.007778	0.017516	-0.003939
	0.001819	0.007866	0.000293	-0.020201	-0.005272	-0.007008
	0.004661					
y140	-0.030681	-0.011455	-0.004778	0.004183	0.012705	0.031189
	0.022159	-0.010844	-0.014344	0.005005	0.043221	-0.000542
	-0.000882	0.013668	-0.011164	0.002311	-0.025793	-0.007893
	0.003610	-0.018138	0.008842	-0.012193	0.007520	-0.027315
	-0.002132	0.002417	0.010443	-0.028031	0.000083	0.009398
	-0.000708					
z140	0.015689	-0.006829	-0.027705	-0.008992	-0.019150	-0.002509
	-0.006371	0.014981	0.003869	-0.017109	0.023952	-0.000577
	-0.001889	0.016587	0.001101	0.037000	0.011880	0.005044
	-0.022617	-0.005071	-0.004678	-0.005126	-0.013405	-0.006834
	-0.016574	-0.011453	0.014538	0.014217	-0.006821	0.020520
	0.017229					
x141	0.024032	0.029847	0.001601	-0.013362	0.013356	0.028140
	0.006772	-0.019677	-0.007469	0.024123	-0.014498	0.011975
	-0.009500	-0.004460	-0.011949	0.013557	0.001270	-0.028192
	0.005185	-0.022000	0.002461	-0.009762	0.017296	0.000253
	-0.000208	0.007291	-0.001896	-0.019506	-0.011897	-0.009430
	0.018937					
y141	-0.029558	-0.010634	-0.005767	0.003519	0.012058	0.031696
	0.020535	-0.010464	-0.012930	0.005810	0.045549	-0.002206
	-0.003443	0.012357	-0.012713	0.003544	-0.023726	-0.009057
	0.004991	-0.015762	0.005703	-0.010958	0.005683	-0.028550
	-0.003806	0.004274	0.014304	-0.021043	0.004370	0.010036
	-0.004628					
z141	0.017440	-0.007132	-0.027622	-0.008695	-0.018577	-0.003523
	-0.006173	0.015123	0.004126	-0.013983	0.022447	0.003381
	0.000340	0.015957	0.002162	0.035109	0.012236	0.004618
	-0.020359	-0.007371	-0.002824	-0.006655	-0.014170	-0.006748
	-0.014563	-0.011249	0.011600	0.010346	-0.010625	0.018334
	0.015779					

x142	0.023785	0.030977	0.002527	-0.013763	0.011794	0.026903
	0.005128	-0.022310	-0.003816	0.026327	-0.016020	0.014660
	-0.007262	-0.010615	-0.019908	0.012665	0.000993	-0.029622
	0.008591	-0.025602	0.006300	-0.012745	0.015029	0.004791
	-0.000523	0.009005	-0.002091	-0.013774	-0.017843	-0.014106
	0.023381					
y142	-0.028795	-0.009348	-0.006771	0.002435	0.011907	0.033198
	0.020110	-0.009401	-0.012735	0.008183	0.047722	-0.001750
	-0.005390	0.012443	-0.013065	0.004145	-0.023695	-0.009977
	0.006228	-0.013876	0.002454	-0.009104	0.004420	-0.029110
	-0.004836	0.003719	0.015228	-0.017650	0.007283	0.012570
	-0.005142					
z142	0.019043	-0.006413	-0.027837	-0.007464	-0.019174	-0.004747
	-0.007226	0.014451	0.006729	-0.013433	0.021458	0.004376
	0.002554	0.014647	0.000012	0.034302	0.011598	0.002928
	-0.017753	-0.009864	0.000591	-0.010425	-0.014143	-0.007276
	-0.010983	-0.010847	0.010168	0.010223	-0.012182	0.014675
	0.011847					
x143	0.023003	0.033640	0.003409	-0.014291	0.011468	0.026270
	0.003747	-0.023203	-0.000369	0.026278	-0.015480	0.011947
	-0.005376	-0.013637	-0.025453	0.011784	0.002016	-0.029495
	0.012823	-0.029023	0.008877	-0.013951	0.013615	0.006368
	-0.000621	0.007089	-0.004551	-0.010031	-0.017793	-0.006177
	0.017850					
y143	-0.027487	-0.008498	-0.007912	0.001804	0.011162	0.034097
	0.019544	-0.009530	-0.012147	0.010604	0.048102	0.000579
	-0.006842	0.010728	-0.012991	0.005356	-0.023521	-0.010696
	0.007631	-0.011055	-0.000223	-0.008332	0.003053	-0.028990
	-0.004088	0.004811	0.017527	-0.014507	0.005862	0.009350
	-0.003480					
z143	0.020394	-0.005533	-0.027654	-0.006394	-0.019893	-0.005612
	-0.008767	0.014278	0.009276	-0.013205	0.020946	0.005001
	0.004035	0.013350	-0.002753	0.032951	0.010946	0.000353
	-0.015234	-0.012840	0.004698	-0.012215	-0.014643	-0.007101
	-0.008718	-0.011606	0.006944	0.010837	-0.010216	0.013101
	0.008367					
x144	0.022271	0.035564	0.005194	-0.015610	0.012947	0.025874
	0.003640	-0.022431	0.001197	0.029007	-0.014027	0.012860
	-0.004950	-0.014616	-0.023506	0.009578	0.005752	-0.026699
	0.015239	-0.029149	0.007264	-0.013169	0.006536	0.011623
	0.004577	0.006724	-0.008633	-0.004479	-0.014978	0.006979
	0.019558					

y144	-0.026103	-0.007415	-0.009247	0.001450	0.009981	0.034424
	0.018529	-0.009167	-0.011161	0.012240	0.047924	0.001148
	-0.006597	0.007604	-0.014705	0.006990	-0.024220	-0.011078
	0.009352	-0.009938	-0.000313	-0.008426	0.002332	-0.030316
	-0.003985	0.004912	0.019700	-0.010500	0.006110	0.008270
	-0.003254					
z144	0.022017	-0.004738	-0.026997	-0.005748	-0.019476	-0.006225
	-0.009258	0.014406	0.011038	-0.010646	0.021586	0.007525
	0.004350	0.013634	-0.002436	0.032703	0.011746	-0.000143
	-0.013137	-0.014449	0.006150	-0.013593	-0.017074	-0.005965
	-0.006231	-0.011927	0.003923	0.009162	-0.008237	0.013894
	0.005761					
x145	0.021049	0.036536	0.006884	-0.018020	0.012722	0.025115
	0.004032	-0.023768	0.001642	0.029599	-0.015686	0.018492
	-0.003325	-0.017371	-0.028003	0.007108	0.003316	-0.025214
	0.012625	-0.031215	0.006402	-0.010274	0.009876	0.016120
	-0.000400	0.005217	-0.012604	0.003317	-0.019769	0.006811
	0.008354					
y145	-0.024449	-0.006350	-0.010295	0.001454	0.009190	0.034684
	0.016957	-0.008708	-0.009969	0.014547	0.046481	0.000479
	-0.006758	0.004805	-0.014518	0.007980	-0.023799	-0.010234
	0.012838	-0.007259	-0.000055	-0.010930	-0.000416	-0.030322
	-0.000218	0.004415	0.021357	-0.009646	0.005033	0.008816
	0.002700					
z145	0.023635	-0.003579	-0.026916	-0.005950	-0.019422	-0.006662
	-0.009022	0.013720	0.012146	-0.008745	0.021704	0.011292
	0.005246	0.013025	-0.002784	0.031751	0.011759	-0.002376
	-0.011881	-0.017555	0.007784	-0.014626	-0.017431	-0.004802
	-0.005930	-0.011365	0.003391	0.008302	-0.005480	0.012480
	0.003996					
x146	0.020711	0.036407	0.007537	-0.019490	0.011842	0.024852
	0.005525	-0.023373	0.001959	0.030572	-0.015449	0.019083
	0.002306	-0.017489	-0.028719	0.003567	0.003773	-0.022407
	0.014563	-0.031013	0.010161	-0.010315	0.001064	0.019914
	0.001667	0.003168	-0.018620	0.013517	-0.022713	0.012872
	0.011818					
y146	-0.023309	-0.003958	-0.011477	0.000906	0.009444	0.034860
	0.016598	-0.007846	-0.009095	0.015479	0.045323	0.001759
	-0.008844	0.001418	-0.013726	0.011360	-0.023424	-0.013135
	0.014770	-0.007884	-0.000163	-0.011162	0.001577	-0.029659
	0.002039	0.004105	0.026140	-0.012738	0.007132	0.005880
	0.000436					

z146	0.025499	-0.002867	-0.026753	-0.005592	-0.019929	-0.006550
	-0.008670	0.012475	0.013328	-0.006466	0.021176	0.012604
	0.005665	0.013558	-0.002266	0.030867	0.012852	-0.001826
	-0.007965	-0.018726	0.008162	-0.018098	-0.016497	-0.005742
	-0.004737	-0.010567	0.002954	0.003288	-0.005667	0.009338
	0.003665					
x147	0.020004	0.038002	0.008865	-0.019967	0.011626	0.024301
	0.004890	-0.024057	0.003475	0.031605	-0.015536	0.020022
	0.000333	-0.019398	-0.028634	0.004050	0.001280	-0.022555
	0.018290	-0.028884	0.009409	-0.013674	-0.002171	0.025889
	0.004227	0.003806	-0.016951	0.014697	-0.022454	0.009781
	0.015677					
y147	-0.022580	-0.001774	-0.012278	0.000028	0.009301	0.034206
	0.016796	-0.007895	-0.008539	0.016997	0.044008	0.004981
	-0.009805	-0.000473	-0.011593	0.012330	-0.024598	-0.012677
	0.014738	-0.008009	-0.000740	-0.008725	0.000930	-0.026142
	0.001823	0.004363	0.029282	-0.011079	0.007400	0.005018
	-0.003545					
z147	0.027491	-0.002419	-0.026265	-0.004746	-0.020021	-0.007019
	-0.010651	0.012362	0.014618	-0.004363	0.021178	0.015464
	0.006131	0.012187	-0.006179	0.029746	0.012145	-0.003144
	-0.006067	-0.020422	0.008530	-0.022849	-0.017169	-0.005681
	-0.001611	-0.009247	0.001334	0.004596	-0.005452	0.005529
	0.004434					
x148	0.019268	0.040262	0.010903	-0.020424	0.011700	0.023246
	0.004186	-0.024153	0.004904	0.032803	-0.013362	0.019980
	-0.000454	-0.019876	-0.027699	0.005278	0.001332	-0.020379
	0.019574	-0.024864	0.007132	-0.015098	-0.004458	0.026905
	0.004823	0.006211	-0.017947	0.016622	-0.021927	0.012470
	0.008247					
y148	-0.021876	-0.000618	-0.012186	-0.000463	0.009206	0.032711
	0.016069	-0.008087	-0.006984	0.019268	0.042975	0.008036
	-0.011271	-0.002141	-0.010840	0.012069	-0.025849	-0.008310
	0.015166	-0.004637	0.000066	-0.004250	-0.002091	-0.019240
	-0.000698	0.003932	0.025127	-0.007778	0.009554	0.008410
	-0.003580					
z148	0.029260	-0.001122	-0.025504	-0.004509	-0.019930	-0.007412
	-0.012510	0.012488	0.015782	-0.002476	0.021613	0.018111
	0.007151	0.011180	-0.008320	0.030481	0.011415	-0.004222
	-0.005627	-0.023127	0.006421	-0.029429	-0.016494	-0.007434
	0.003091	-0.007675	0.002892	0.005618	-0.004840	0.002343
	-0.000717					

x149	0.018065	0.040878	0.013132	-0.019971	0.010769	0.022012
	0.005163	-0.024399	0.004576	0.032792	-0.014160	0.021531
	0.000679	-0.020485	-0.030105	0.004282	-0.000456	-0.016867
	0.017357	-0.021209	0.006178	-0.011941	-0.007446	0.026397
	-0.000496	0.008772	-0.015847	0.016823	-0.022844	0.009910
	0.007486					
y149	-0.020780	0.000018	-0.012497	-0.000603	0.009348	0.031652
	0.014910	-0.007974	-0.005801	0.021023	0.042399	0.008852
	-0.013123	-0.004306	-0.011669	0.011322	-0.025699	-0.007305
	0.014184	-0.001866	0.001193	-0.001754	-0.002955	-0.016634
	-0.001014	0.002907	0.020044	-0.004208	0.009641	0.011482
	-0.005031					
z149	0.030705	-0.000130	-0.024499	-0.004400	-0.020611	-0.008044
	-0.012917	0.011753	0.015790	-0.000563	0.020512	0.022558
	0.009563	0.009905	-0.010305	0.029348	0.009311	-0.003695
	-0.005078	-0.025819	0.005226	-0.031698	-0.017364	-0.007164
	0.003223	-0.005118	0.006268	0.005589	-0.004075	-0.002000
	-0.002733					
x150	0.016798	0.041211	0.014766	-0.019457	0.010997	0.021603
	0.006942	-0.023973	0.004498	0.033802	-0.013764	0.021581
	0.001045	-0.021267	-0.027161	0.004204	0.000999	-0.012302
	0.016829	-0.016921	0.005723	-0.006683	-0.013262	0.029284
	0.002050	0.008509	-0.019345	0.018785	-0.019898	0.013206
	0.003866					
y150	-0.019554	0.000911	-0.012920	-0.000630	0.008950	0.030746
	0.013895	-0.007174	-0.004435	0.022082	0.042797	0.007776
	-0.013430	-0.004984	-0.013161	0.011727	-0.024686	-0.007691
	0.015871	0.000117	0.002960	-0.000900	-0.002848	-0.015676
	-0.001192	0.000646	0.017429	-0.003433	0.010835	0.011941
	-0.007625					
z150	0.031919	0.000871	-0.023555	-0.004304	-0.020276	-0.008101
	-0.013012	0.010942	0.016118	0.001851	0.019305	0.027048
	0.010798	0.007721	-0.009244	0.028251	0.008056	0.000019
	-0.004952	-0.026891	0.004082	-0.032559	-0.021417	-0.003520
	0.007150	-0.003455	0.004689	0.008927	-0.001232	0.000392
	-0.005249					
x151	0.015513	0.040769	0.016113	-0.020080	0.011062	0.020983
	0.007873	-0.024715	0.002795	0.033963	-0.014617	0.021654
	-0.000670	-0.022335	-0.027871	0.001861	0.000057	-0.010458
	0.014912	-0.012450	0.002972	-0.005048	-0.011887	0.031107
	-0.000297	0.007625	-0.021532	0.017637	-0.019093	0.012867
	0.007661					

y151	-0.018485	0.001475	-0.013347	-0.000289	0.008481	0.029483
	0.013226	-0.006617	-0.002929	0.023436	0.042610	0.007778
	-0.012877	-0.006791	-0.014189	0.012001	-0.023546	-0.010041
	0.016962	0.001473	0.004902	0.000607	-0.003572	-0.014153
	-0.000149	-0.001403	0.015955	-0.000685	0.010460	0.009852
	-0.011476					
z151	0.033365	0.001879	-0.023164	-0.005604	-0.020428	-0.008046
	-0.014631	0.009601	0.015036	0.002881	0.016566	0.030851
	0.008993	0.005367	-0.011267	0.024622	0.004652	0.005656
	-0.006566	-0.027877	0.001633	-0.035251	-0.019725	-0.000532
	0.005231	-0.002394	0.001348	0.008017	0.000554	0.003032
	-0.000909					
x152	0.014605	0.040798	0.017731	-0.019423	0.010496	0.020582
	0.009790	-0.025737	0.001958	0.034421	-0.013911	0.020768
	-0.001349	-0.022602	-0.027887	0.001351	0.002362	-0.006482
	0.012568	-0.008456	0.000952	-0.002412	-0.013230	0.030814
	-0.003315	0.006645	-0.020749	0.016694	-0.018679	0.010714
	0.008597					
y152	-0.017507	0.001871	-0.014186	0.000492	0.007910	0.028264
	0.012055	-0.005505	-0.001890	0.024326	0.041864	0.008100
	-0.012590	-0.007844	-0.014435	0.012115	-0.024002	-0.012220
	0.017353	0.002742	0.007040	0.002979	-0.004574	-0.014486
	0.001344	-0.003208	0.012611	-0.000037	0.011263	0.009580
	-0.014861					
z152	0.035053	0.003041	-0.021900	-0.006216	-0.021073	-0.007822
	-0.014712	0.007191	0.014695	0.005057	0.015470	0.033904
	0.009475	0.004102	-0.013375	0.022045	0.004923	0.009307
	-0.006452	-0.029073	-0.000733	-0.037259	-0.018941	0.001190
	0.002131	-0.001052	0.002336	0.008866	0.001933	0.001572
	0.000176					
x153	0.013851	0.040842	0.019267	-0.018264	0.009874	0.020397
	0.011921	-0.026164	0.000950	0.035461	-0.012057	0.019052
	-0.001670	-0.023761	-0.026152	0.003184	0.004893	-0.002084
	0.010914	-0.004599	-0.001078	0.000725	-0.019254	0.029420
	-0.002169	0.007211	-0.021543	0.015316	-0.015264	0.014601
	0.008510					
y153	-0.016453	0.002360	-0.015094	0.001644	0.007324	0.026815
	0.011510	-0.004152	-0.000622	0.025133	0.041430	0.008103
	-0.011442	-0.006929	-0.014763	0.011667	-0.023692	-0.013740
	0.017839	0.003432	0.009685	0.005380	-0.006166	-0.015640
	0.003637	-0.004877	0.009432	0.001214	0.011112	0.008818
	-0.017426					

z153	0.036782	0.004384	-0.020206	-0.006151	-0.021507	-0.007393
	-0.015239	0.006186	0.014486	0.008491	0.015858	0.036548
	0.010206	0.001479	-0.014120	0.023305	0.003917	0.013768
	-0.006647	-0.029010	-0.003772	-0.039823	-0.024253	0.002215
	0.004477	0.002393	0.001120	0.009989	0.008892	0.007223
	-0.001677					
x154	0.012859	0.040059	0.020315	-0.016806	0.009118	0.019941
	0.014348	-0.026323	0.000907	0.034617	-0.009170	0.016390
	-0.000897	-0.022415	-0.024637	0.003944	0.004901	-0.002125
	0.012534	-0.002579	-0.002074	-0.000282	-0.021725	0.028074
	0.001284	0.005589	-0.023519	0.017212	-0.014517	0.017731
	0.000429					
y154	-0.015642	0.003612	-0.015579	0.002251	0.007315	0.024876
	0.010714	-0.003544	0.000142	0.024519	0.040061	0.010112
	-0.012543	-0.007933	-0.015261	0.011051	-0.023856	-0.011515
	0.016226	0.003569	0.010906	0.008447	-0.009234	-0.014304
	0.003837	-0.006467	0.005315	0.003886	0.009096	0.011174
	-0.019031					
z154	0.038568	0.004535	-0.019896	-0.005793	-0.022888	-0.007260
	-0.015624	0.005572	0.015180	0.009196	0.016620	0.036801
	0.012104	0.001781	-0.016291	0.022543	-0.000800	0.010825
	-0.001935	-0.031534	-0.004920	-0.048073	-0.022865	0.002699
	0.009515	0.003061	-0.000704	0.015327	0.012510	0.009966
	-0.011403					
x155	0.011871	0.039577	0.021919	-0.015183	0.009580	0.019578
	0.016486	-0.025229	-0.000379	0.036004	-0.006414	0.013732
	0.000238	-0.020583	-0.022038	0.002078	0.004904	-0.001520
	0.011251	0.000696	-0.001311	0.001896	-0.023765	0.027016
	0.001381	0.005399	-0.023554	0.018478	-0.012356	0.017145
	0.001398					
y155	-0.014757	0.004494	-0.016337	0.003234	0.006732	0.023123
	0.009821	-0.002761	0.001211	0.023839	0.039090	0.012782
	-0.012154	-0.008310	-0.016499	0.010831	-0.024501	-0.011075
	0.012824	0.002890	0.013082	0.011279	-0.012510	-0.014418
	0.006205	-0.006928	0.001650	0.007399	0.006781	0.009796
	-0.018573					
z155	0.039970	0.006898	-0.018078	-0.005960	-0.020831	-0.007195
	-0.014805	0.008406	0.012755	0.013606	0.018746	0.042854
	0.013894	0.001607	-0.017343	0.018557	-0.003926	0.004498
	-0.000842	-0.035515	-0.004218	-0.047743	-0.022097	0.005585
	0.007141	0.007357	0.003489	0.018484	0.013889	0.003001
	-0.010298					

x156	0.010588	0.038940	0.022985	-0.014141	0.008931	0.019685
	0.017767	-0.024664	-0.001855	0.036052	-0.003474	0.012022
	-0.000250	-0.020558	-0.019222	0.003421	0.003371	-0.002066
	0.008868	0.001894	-0.000833	0.004341	-0.022897	0.023501
	-0.002748	0.005860	-0.020421	0.020545	-0.008011	0.016652
	0.002216					
y156	-0.013881	0.004961	-0.017013	0.003393	0.005677	0.021247
	0.008165	-0.000322	0.000796	0.023308	0.040425	0.014141
	-0.011900	-0.007889	-0.014359	0.011961	-0.024485	-0.010206
	0.010710	0.002846	0.015904	0.014584	-0.014815	-0.017234
	0.002659	-0.008926	0.001724	0.012726	0.010886	0.014357
	-0.017355					
z156	0.040999	0.007097	-0.016934	-0.005606	-0.022778	-0.006061
	-0.015670	0.007241	0.012423	0.017306	0.015887	0.041490
	0.014941	-0.000157	-0.019592	0.017403	-0.011143	0.000985
	0.000978	-0.038715	-0.004770	-0.047810	-0.015834	0.004148
	0.004872	0.010730	0.002763	0.017690	0.014527	-0.002926
	-0.007421					
x157	0.008620	0.036890	0.024961	-0.012559	0.008043	0.020110
	0.019230	-0.024156	-0.002469	0.035451	-0.000938	0.006051
	0.000984	-0.017150	-0.015459	0.004278	0.002821	0.000289
	0.007474	0.008390	-0.001992	0.004915	-0.024956	0.018639
	-0.004866	0.003896	-0.017808	0.021512	-0.002473	0.019242
	0.001944					
y157	-0.013307	0.004423	-0.017042	0.004126	0.004230	0.018576
	0.005448	-0.000002	0.000876	0.022063	0.039022	0.013549
	-0.008988	-0.006450	-0.016220	0.012799	-0.025334	-0.006759
	0.005597	0.007060	0.015848	0.015535	-0.017468	-0.016644
	0.001632	-0.009222	0.000507	0.018319	0.016989	0.015393
	-0.015490					
z157	0.042122	0.007496	-0.014781	-0.006457	-0.024452	-0.004290
	-0.015258	0.008509	0.012610	0.019974	0.016966	0.038910
	0.014515	0.002225	-0.017746	0.015018	-0.014218	-0.001218
	0.008974	-0.038570	-0.005744	-0.047855	-0.018100	0.000086
	0.004710	0.007330	0.003583	0.013385	0.015864	0.003756
	-0.009570					
x158	0.006531	0.035645	0.026693	-0.010840	0.008782	0.020955
	0.021809	-0.023363	-0.003954	0.033767	0.001808	0.002930
	0.001570	-0.013956	-0.012580	0.004402	0.003229	0.000907
	0.005677	0.012230	-0.004153	0.005938	-0.025992	0.016952
	-0.007874	0.003665	-0.015578	0.021195	-0.000809	0.017761
	0.002765					

y158	-0.012406	0.004273	-0.016692	0.005002	0.004593	0.016583
	0.003346	0.000031	0.002391	0.020454	0.036353	0.013998
	-0.006167	-0.003850	-0.020436	0.011459	-0.025250	-0.004693
	0.001361	0.009792	0.015723	0.013712	-0.018309	-0.012368
	0.007740	-0.011283	-0.005349	0.020457	0.016790	0.011271
	-0.015085					
z158	0.043098	0.007732	-0.012224	-0.007654	-0.024189	-0.002238
	-0.014987	0.008258	0.011776	0.022017	0.018850	0.041565
	0.014130	0.003563	-0.017719	0.010000	-0.017359	-0.005812
	0.014917	-0.040858	-0.010104	-0.047354	-0.015056	0.002417
	-0.004213	0.009762	0.003811	0.012648	0.011892	0.001574
	-0.005259					
x159	0.004644	0.034345	0.028147	-0.009193	0.009541	0.022021
	0.024710	-0.022596	-0.005620	0.031861	0.004583	-0.001134
	0.001212	-0.011214	-0.008945	0.005615	0.004364	0.002035
	0.002716	0.017524	-0.006654	0.006516	-0.026687	0.014880
	-0.009783	0.002288	-0.012170	0.019089	0.001642	0.016385
	0.002199					
y159	-0.011343	0.004202	-0.015807	0.006384	0.004037	0.014523
	0.000967	0.000028	0.004170	0.019438	0.034052	0.013463
	-0.003922	-0.000291	-0.024619	0.009282	-0.024895	-0.002537
	0.000959	0.010480	0.014828	0.008910	-0.017411	-0.006788
	0.016981	-0.013230	-0.010128	0.018965	0.011179	0.002426
	-0.012379					
z159	0.044166	0.008184	-0.008687	-0.008611	-0.024379	0.000444
	-0.012939	0.007095	0.010234	0.021623	0.022195	0.037247
	0.013454	0.003224	-0.013662	0.009347	-0.015344	-0.005371
	0.014852	-0.037555	-0.011655	-0.041873	-0.018394	0.003674
	-0.005811	0.005755	0.002159	0.010321	0.019548	0.011952
	-0.006090					
x160	0.002899	0.033406	0.029433	-0.007323	0.010406	0.023175
	0.027692	-0.022017	-0.006986	0.028912	0.007883	-0.005785
	0.000787	-0.008770	-0.005890	0.006783	0.006911	0.002848
	0.001381	0.021602	-0.009222	0.007149	-0.027468	0.012821
	-0.010502	0.000305	-0.008699	0.017053	0.002278	0.014670
	0.000948					
y160	-0.010545	0.003913	-0.015237	0.007170	0.003974	0.012970
	-0.001027	0.000430	0.005371	0.017348	0.032870	0.012680
	-0.003474	0.002097	-0.025577	0.009074	-0.023951	-0.000881
	0.000857	0.010810	0.012983	0.004596	-0.014064	-0.002683
	0.019587	-0.017154	-0.013364	0.017947	0.006397	-0.003527
	-0.009330					

z160	0.045051	0.009305	-0.004337	-0.009352	-0.025031	0.002988
	-0.009782	0.005204	0.009942	0.021837	0.024946	0.033205
	0.011911	0.006640	-0.013702	0.007094	-0.012809	-0.005065
	0.012331	-0.034427	-0.016508	-0.041897	-0.016990	0.005883
	-0.002811	0.003518	0.003812	0.009812	0.018505	0.008394
	-0.006527					
x161	0.001174	0.032499	0.029504	-0.005391	0.011627	0.024575
	0.030696	-0.021806	-0.008714	0.026117	0.009334	-0.010332
	0.002092	-0.005862	-0.001475	0.008854	0.007726	0.002667
	-0.000241	0.023295	-0.011311	0.008182	-0.026670	0.010317
	-0.011697	0.000018	-0.003339	0.016621	0.003201	0.009578
	-0.001435					
y161	-0.009783	0.003590	-0.015801	0.007793	0.003994	0.011823
	-0.002675	0.000360	0.005788	0.014088	0.030868	0.011412
	-0.000980	0.003282	-0.023749	0.011350	-0.024872	0.001250
	-0.000325	0.008598	0.011459	0.001770	-0.011278	0.000386
	0.021859	-0.019090	-0.011995	0.019547	0.003350	-0.012392
	-0.007312					
z161	0.045567	0.010408	0.000671	-0.009838	-0.025964	0.005845
	-0.007686	0.005252	0.009827	0.024393	0.029496	0.029721
	0.010745	0.008442	-0.012879	0.002724	-0.008959	-0.003465
	0.013489	-0.031571	-0.019228	-0.040483	-0.017275	0.007488
	0.002698	0.000787	0.003402	0.002848	0.019295	0.004580
	-0.005311					
x162	-0.000235	0.031922	0.029125	-0.003767	0.012466	0.025728
	0.033353	-0.022239	-0.010388	0.024117	0.010578	-0.013880
	0.003025	-0.003818	0.001767	0.010938	0.007500	0.003092
	-0.002665	0.024866	-0.013098	0.008033	-0.026240	0.007039
	-0.010542	-0.001498	0.003219	0.014468	0.004300	0.004657
	-0.006697					
y162	-0.009385	0.003370	-0.016873	0.008109	0.003812	0.010997
	-0.004441	0.002058	0.005940	0.011091	0.028408	0.010750
	0.002363	0.005451	-0.020608	0.012545	-0.029172	0.002428
	0.000257	0.004303	0.010038	-0.003123	-0.006319	0.001705
	0.020603	-0.020401	-0.010900	0.020849	0.001316	-0.018476
	-0.006294					
z162	0.046552	0.010893	0.005773	-0.011190	-0.025630	0.007970
	-0.005067	0.003099	0.006769	0.023562	0.034894	0.023739
	0.006390	0.008941	-0.015650	-0.000018	-0.001766	-0.003187
	0.012359	-0.022599	-0.025154	-0.038783	-0.016961	0.010757
	0.007473	-0.005505	0.009365	0.003857	0.010324	0.000113
	-0.004637					

x163	-0.002245	0.030885	0.029721	-0.002032	0.014158	0.026311
	0.033164	-0.021211	-0.010649	0.023924	0.008302	-0.013960
	0.000156	-0.002895	0.002541	0.009786	0.004877	-0.000111
	-0.003889	0.022641	-0.013402	0.006601	-0.017366	0.003319
	-0.020016	-0.005964	0.001610	0.007584	0.003866	0.002989
	-0.009911					
y163	-0.008836	0.003400	-0.016348	0.008587	0.003614	0.010002
	-0.006529	0.005167	0.007732	0.008105	0.027388	0.010341
	0.001887	0.006686	-0.018005	0.011038	-0.030546	0.002624
	0.002506	-0.000365	0.010617	-0.010466	-0.001810	0.003921
	0.015169	-0.026802	-0.014171	0.016954	-0.002701	-0.016392
	-0.004697					
z163	0.046128	0.011711	0.009061	-0.012244	-0.024201	0.010482
	-0.001770	0.001335	0.002993	0.022372	0.039651	0.016289
	0.007441	0.011629	-0.012652	0.002869	0.004219	-0.001567
	0.018994	-0.014690	-0.030266	-0.031049	-0.018315	0.012491
	0.013610	-0.009934	0.016030	0.000889	0.007071	0.001171
	-0.005477					
x164	-0.004414	0.029778	0.029625	-0.000490	0.016252	0.026530
	0.031887	-0.020502	-0.008993	0.022444	0.005475	-0.012283
	-0.002248	-0.004350	0.002867	0.013483	0.005143	-0.003134
	-0.003503	0.018496	-0.013875	0.006672	-0.007438	-0.003437
	-0.029302	-0.014081	-0.004270	-0.001025	0.007520	0.006902
	-0.015882					
y164	-0.008602	0.002837	-0.016062	0.008829	0.003487	0.009475
	-0.008239	0.007766	0.008919	0.003243	0.028358	0.010550
	0.002184	0.004928	-0.015802	0.013300	-0.027140	0.003633
	0.006074	-0.003867	0.008963	-0.014739	0.003735	0.005940
	0.009911	-0.035127	-0.017319	0.012836	-0.008891	-0.013906
	0.000649					
z164	0.045116	0.011868	0.011780	-0.012986	-0.022581	0.012317
	-0.000320	0.001147	-0.001215	0.024460	0.036450	0.014649
	0.009944	0.012864	-0.006937	-0.000775	-0.003258	-0.000277
	0.024411	-0.014425	-0.028205	-0.023577	-0.008372	0.013928
	0.011164	-0.010367	0.021034	-0.006860	0.000292	-0.012445
	0.001833					
x165	-0.006175	0.028837	0.028898	0.000697	0.017905	0.026371
	0.030431	-0.020720	-0.008311	0.020870	0.001794	-0.009009
	-0.003752	-0.004930	0.003325	0.017643	0.007524	-0.004966
	-0.002371	0.012415	-0.013543	0.003695	0.006810	-0.011553
	-0.038732	-0.024427	-0.009439	-0.012532	0.004384	0.006826
	-0.020191					

y165	-0.008542	0.002552	-0.017524	0.008583	0.002901	0.009464
	-0.009631	0.011354	0.008347	-0.001123	0.027990	0.010926
	0.006257	0.003225	-0.011015	0.016223	-0.026777	0.005960
	0.010263	-0.007705	0.007900	-0.020422	0.011239	0.005456
	0.005837	-0.042999	-0.016816	0.009633	-0.017762	-0.011216
	0.004826					
z165	0.044293	0.011390	0.014450	-0.014079	-0.019431	0.013379
	0.000773	0.000851	-0.001128	0.026155	0.031465	0.014853
	0.010523	0.011788	-0.001886	0.001331	-0.012780	-0.004352
	0.027008	-0.018528	-0.024916	-0.013566	-0.002292	0.015120
	0.002533	-0.012758	0.017852	-0.010926	0.004664	-0.010812
	0.000992					
x166	0.008860	-0.024566	-0.017636	0.004270	-0.004823	-0.030694
	0.014555	-0.005310	-0.002496	-0.011942	0.020635	-0.035081
	0.002619	-0.004184	-0.007963	-0.005790	0.006377	0.001808
	-0.002611	-0.011878	-0.005323	0.004354	-0.005674	-0.025354
	-0.005856	-0.019746	-0.019476	-0.015478	0.007944	-0.007953
	0.000357					
y166	0.031118	-0.012966	0.009394	-0.011392	0.024603	-0.005138
	-0.021892	-0.000611	0.012530	-0.036057	-0.023797	0.005473
	-0.018094	0.003469	-0.007202	-0.013075	0.002343	-0.024293
	-0.020969	-0.023410	-0.021234	-0.010071	-0.023340	0.003043
	-0.019354	0.006816	-0.019699	0.015856	-0.008536	-0.015249
	0.013623					
z166	-0.023824	-0.028104	0.017279	0.012117	0.004403	0.004194
	0.015955	-0.023351	-0.015109	0.004398	-0.001885	0.019200
	-0.004310	-0.036383	-0.013791	0.011697	0.000404	0.009175
	0.001807	0.006029	-0.006859	0.027728	0.012716	0.008720
	0.016638	-0.027799	-0.001300	-0.005878	0.002199	0.006038
	0.019718					
x167	0.009994	-0.023755	-0.018494	0.005578	-0.003184	-0.031166
	0.014496	-0.003174	0.000173	-0.013436	0.022469	-0.032869
	0.002824	-0.001368	-0.008876	-0.007926	0.007994	0.003854
	-0.002794	-0.011408	-0.006317	0.006076	-0.006929	-0.023206
	-0.002122	-0.017158	-0.018650	-0.013617	0.000881	-0.012903
	0.005404					
y167	0.030147	-0.012848	0.009307	-0.011875	0.025710	-0.002893
	-0.021046	-0.001802	0.014718	-0.035618	-0.025996	0.008217
	-0.018226	0.003014	-0.006874	-0.010109	0.003582	-0.023414
	-0.015771	-0.020435	-0.019637	-0.002131	-0.020696	0.003669
	-0.020606	0.014215	-0.017171	0.009435	-0.007774	-0.008456
	0.007152					

z167	-0.024585	-0.030479	0.016015	0.012866	0.004518	0.006667
	0.020098	-0.023899	-0.014774	0.003252	-0.004473	0.017455
	-0.001785	-0.033358	-0.013599	0.006132	0.002380	0.013836
	0.001585	0.008714	-0.002144	0.027061	0.020050	0.017267
	0.015115	-0.024797	-0.004508	-0.005340	-0.002875	-0.001607
	0.014914					
x168	0.011597	-0.022657	-0.019015	0.006590	-0.002107	-0.031340
	0.014291	-0.001427	0.003709	-0.015783	0.023711	-0.029766
	0.002564	-0.000497	-0.010072	-0.007924	0.009695	0.003964
	-0.002439	-0.010782	-0.006927	0.008554	-0.007466	-0.021158
	-0.001033	-0.013479	-0.017000	-0.011623	-0.000249	-0.013358
	0.007475					
y168	0.028894	-0.012994	0.009551	-0.012858	0.025961	-0.000477
	-0.019611	-0.002872	0.015346	-0.035483	-0.026835	0.010690
	-0.019139	0.002929	-0.007176	-0.010586	0.003168	-0.021605
	-0.009860	-0.017314	-0.018552	0.004252	-0.015423	0.004493
	-0.022200	0.016271	-0.014978	0.004036	-0.008617	-0.001620
	0.004512					
z168	-0.025587	-0.032583	0.014264	0.013568	0.004483	0.008993
	0.022686	-0.022903	-0.013347	0.002962	-0.006675	0.016068
	0.001099	-0.031534	-0.012304	0.005065	0.003996	0.016994
	-0.000726	0.010296	0.002146	0.026289	0.023148	0.023917
	0.014901	-0.017479	-0.005110	-0.002784	-0.000982	-0.009766
	0.006051					
x169	0.013492	-0.021348	-0.019476	0.007639	-0.001244	-0.031137
	0.014181	0.000415	0.006763	-0.018521	0.024575	-0.027258
	0.003030	-0.000076	-0.010713	-0.006791	0.011546	0.004401
	-0.000884	-0.008783	-0.007228	0.010649	-0.007616	-0.018235
	0.001468	-0.009782	-0.014860	-0.009517	0.001343	-0.011386
	0.008210					
y169	0.027463	-0.013527	0.009716	-0.013750	0.025416	0.001929
	-0.019281	-0.003479	0.015568	-0.034574	-0.027736	0.013211
	-0.020687	0.002206	-0.006514	-0.008912	0.003425	-0.018967
	-0.005797	-0.017506	-0.017724	0.009864	-0.010516	0.004752
	-0.022203	0.017576	-0.010562	0.000295	-0.009073	-0.001600
	0.007066					
z169	-0.026634	-0.033917	0.012954	0.014015	0.005004	0.010975
	0.026142	-0.021391	-0.011936	0.001986	-0.007286	0.013000
	0.004433	-0.029913	-0.009935	0.002880	0.004417	0.018916
	-0.001729	0.014090	0.007266	0.024975	0.021934	0.029111
	0.015430	-0.013236	-0.006845	0.001444	0.003649	-0.009729
	-0.007963					

x170	0.015546	-0.020097	-0.019079	0.008218	-0.000833	-0.031031
	0.013804	0.001510	0.009436	-0.020922	0.025153	-0.023771
	0.003222	-0.000162	-0.011703	-0.005081	0.011574	0.003791
	-0.000029	-0.006708	-0.008335	0.011658	-0.004818	-0.015600
	0.003421	-0.006042	-0.011637	-0.005810	0.002378	-0.012331
	0.010382					
y170	0.025825	-0.013872	0.010018	-0.014301	0.024647	0.004251
	-0.018395	-0.003712	0.014614	-0.034389	-0.028204	0.014183
	-0.021114	0.001709	-0.004521	-0.008080	0.003893	-0.016118
	-0.002210	-0.016700	-0.016460	0.013116	-0.005376	0.006112
	-0.023930	0.018657	-0.006062	-0.000134	-0.008578	-0.003533
	0.009529					
z170	-0.027042	-0.035650	0.010568	0.013658	0.004831	0.012884
	0.028235	-0.021525	-0.011515	0.000103	-0.008183	0.009968
	0.003391	-0.031136	-0.009758	-0.000927	0.004680	0.018516
	-0.005015	0.013814	0.012011	0.026035	0.024169	0.030850
	0.013412	-0.012199	-0.008684	0.003943	0.001661	-0.012796
	-0.015515					
x171	0.017893	-0.019145	-0.018748	0.008730	-0.001194	-0.030739
	0.013233	0.002146	0.011144	-0.023455	0.025325	-0.021077
	0.003973	-0.001484	-0.013289	-0.004551	0.011025	0.003887
	0.000671	-0.003802	-0.009235	0.011761	-0.000408	-0.012243
	0.004821	-0.002274	-0.009474	-0.001345	0.005154	-0.013502
	0.011594					
y171	0.024237	-0.014636	0.010016	-0.014534	0.023246	0.006564
	-0.017941	-0.004456	0.012613	-0.033241	-0.028402	0.012786
	-0.021159	0.000572	-0.003033	-0.008527	0.003369	-0.011536
	-0.000088	-0.014376	-0.015504	0.015123	-0.000093	0.007685
	-0.024110	0.019020	-0.003561	0.000613	-0.005466	-0.006990
	0.010522					
z171	-0.027720	-0.036756	0.008574	0.012798	0.004492	0.013954
	0.029385	-0.020523	-0.009800	-0.001695	-0.008783	0.008503
	0.001877	-0.030833	-0.008126	-0.002191	0.005830	0.016618
	-0.006018	0.011533	0.015970	0.027592	0.023568	0.027845
	0.009617	-0.014996	-0.009280	0.006204	-0.005037	-0.014132
	-0.014455					
x172	0.020413	-0.017876	-0.018703	0.008517	-0.001507	-0.030964
	0.012672	0.002572	0.011788	-0.027015	0.024753	-0.017027
	0.003869	-0.003686	-0.015868	-0.003587	0.010550	0.002428
	0.002589	-0.003435	-0.010133	0.013633	0.001643	-0.010209
	0.005786	0.000010	-0.006332	0.003315	0.006294	-0.014899
	0.013863					

y172 0.022603 -0.015160 0.009616 -0.014739 0.022381 0.007977
-0.017267 -0.005020 0.009852 -0.032650 -0.028387 0.012249
-0.021879 -0.001268 -0.001729 -0.008037 0.002590 -0.008053
0.002630 -0.014307 -0.014920 0.017219 0.000608 0.007819
-0.023571 0.018682 -0.000508 0.002544 -0.003354 -0.010868
0.011251

z172 -0.027864 -0.037498 0.007448 0.012543 0.003630 0.015871
0.030164 -0.018936 -0.007741 -0.001073 -0.008119 0.006538
0.001560 -0.029938 -0.006506 -0.003191 0.007156 0.015399
-0.008922 0.009991 0.018518 0.026031 0.024982 0.025274
0.007818 -0.015599 -0.013713 0.008961 -0.009159 -0.014041
-0.013025

x173 0.023002 -0.016505 -0.017996 0.007861 -0.001497 -0.031218
0.012237 0.002979 0.011657 -0.030403 0.024781 -0.011703
0.002748 -0.006279 -0.018848 -0.002541 0.010550 0.000353
0.004414 -0.002977 -0.012674 0.014767 0.004081 -0.009111
0.005459 0.001265 -0.004316 0.007147 0.005375 -0.015127
0.017291

y173 0.020875 -0.015552 0.009079 -0.015166 0.021491 0.009355
-0.016114 -0.005122 0.006764 -0.031604 -0.027211 0.011929
-0.023438 -0.003032 0.000065 -0.007266 0.002450 -0.004644
0.004082 -0.014694 -0.015546 0.019113 0.000875 0.006889
-0.024227 0.017787 0.002374 0.005108 -0.002082 -0.013114
0.012777

z173 -0.027343 -0.037939 0.005253 0.011856 0.001552 0.016501
0.030521 -0.017326 -0.005726 -0.000918 -0.007641 0.002285
0.002221 -0.029264 -0.004406 -0.003516 0.007987 0.013986
-0.010577 0.009362 0.022417 0.027115 0.022981 0.022910
0.007992 -0.016690 -0.013659 0.013953 -0.006863 -0.015390
-0.016122

x174 0.025829 -0.015329 -0.017046 0.006785 -0.001801 -0.031781
0.011594 0.003688 0.011114 -0.033371 0.024461 -0.006329
0.001282 -0.008671 -0.022460 -0.001004 0.010457 -0.001470
0.004547 -0.001842 -0.016273 0.017481 0.007039 -0.006705
0.004650 0.002747 -0.002605 0.009406 0.004842 -0.013129
0.023056

y174 0.019199 -0.016192 0.008672 -0.015491 0.020661 0.010866
-0.014591 -0.004418 0.003377 -0.029254 -0.025535 0.012016
-0.025469 -0.004263 0.002837 -0.005603 0.002430 -0.000485
0.002967 -0.014608 -0.017086 0.022630 0.000385 0.006669
-0.024455 0.018006 0.006016 0.006189 -0.002080 -0.011729
0.017226

z174	-0.026838	-0.038281	0.003064	0.011972	-0.000677	0.016825
	0.029653	-0.017216	-0.003824	-0.001911	-0.007055	-0.003003
	0.002362	-0.031432	-0.002535	-0.005241	0.007476	0.012211
	-0.009713	0.008522	0.025874	0.023951	0.019676	0.016448
	0.009329	-0.018602	-0.011374	0.022554	-0.007210	-0.020353
	-0.023290					
x175	0.028824	-0.013833	-0.015982	0.005525	-0.003186	-0.032846
	0.010422	0.003723	0.011764	-0.036517	0.022936	-0.001777
	0.002272	-0.012551	-0.026371	0.001234	0.009170	-0.004435
	0.005442	-0.000391	-0.019246	0.016967	0.010884	-0.008823
	0.008113	0.006182	-0.000422	0.009537	0.003763	-0.010149
	0.023402					
y175	0.017158	-0.016459	0.008526	-0.015110	0.019268	0.011876
	-0.013293	-0.003749	0.001587	-0.026286	-0.023445	0.011676
	-0.026520	-0.006403	0.004652	-0.002970	0.001716	0.002515
	0.002199	-0.015499	-0.017967	0.024939	-0.000541	0.003031
	-0.020895	0.019918	0.009797	0.006601	-0.002535	-0.009408
	0.019118					
z175	-0.026056	-0.039042	0.001877	0.011278	-0.002195	0.016765
	0.029071	-0.015718	-0.003748	-0.000568	-0.004973	-0.006137
	0.001743	-0.032409	-0.001446	-0.005166	0.007447	0.010806
	-0.011246	0.008950	0.026686	0.022929	0.020526	0.010913
	0.011445	-0.021498	-0.009405	0.027935	-0.007599	-0.020985
	-0.023510					
x176	0.030129	-0.012750	-0.015271	0.003838	-0.004745	-0.032634
	0.009234	0.002758	0.011471	-0.039489	0.019362	-0.000795
	0.004344	-0.016081	-0.030353	0.001732	0.005384	-0.005544
	0.007143	0.000599	-0.019372	0.014085	0.016970	-0.010209
	0.012263	0.005693	0.000397	0.011813	0.005035	-0.010694
	0.020239					
y176	0.014918	-0.016711	0.008316	-0.015366	0.018357	0.013413
	-0.012140	-0.003591	0.000089	-0.023773	-0.023395	0.010119
	-0.026185	-0.007620	0.005815	-0.001394	-0.000381	0.005565
	0.001057	-0.016422	-0.017890	0.025776	0.000812	0.002075
	-0.017394	0.019733	0.011577	0.004937	-0.001081	-0.009629
	0.019740					
z176	-0.025254	-0.038990	0.001365	0.010472	-0.003424	0.016657
	0.028823	-0.013866	-0.003148	0.000228	-0.002055	-0.008145
	0.003497	-0.031436	0.000240	-0.003101	0.009664	0.010886
	-0.013597	0.008570	0.026565	0.023225	0.019041	0.008733
	0.015747	-0.022108	-0.008767	0.019823	-0.004552	-0.015107
	-0.017754					

x177	0.031263	-0.011248	-0.014895	0.002230	-0.005462	-0.032462
	0.007925	0.000713	0.011252	-0.042902	0.016202	0.000182
	0.005828	-0.018950	-0.034217	0.002435	0.003448	-0.005947
	0.008799	0.002036	-0.019692	0.013444	0.019784	-0.009669
	0.012521	0.006105	0.001282	0.014010	0.004998	-0.011476
	0.019785					
y177	0.012758	-0.016617	0.007919	-0.015629	0.017718	0.015058
	-0.011128	-0.004027	-0.000911	-0.022115	-0.023233	0.007928
	-0.026477	-0.008187	0.006841	-0.000115	-0.001706	0.008575
	0.000223	-0.016785	-0.017298	0.026283	-0.000108	0.001565
	-0.015568	0.018802	0.012541	0.006079	-0.000277	-0.010850
	0.019959					
z177	-0.024226	-0.038807	0.001012	0.010055	-0.004879	0.016970
	0.028471	-0.012074	-0.001575	-0.000829	0.000992	-0.010434
	0.003965	-0.029496	0.002705	-0.002848	0.009759	0.011760
	-0.015844	0.006723	0.025835	0.021411	0.016121	0.007528
	0.018375	-0.023561	-0.008853	0.018324	-0.003579	-0.015015
	-0.018394					
x178	0.032228	-0.009740	-0.014152	0.001298	-0.006201	-0.032490
	0.006524	-0.000714	0.011268	-0.045333	0.014315	0.000734
	0.007028	-0.020718	-0.037016	0.003906	0.002349	-0.004540
	0.010561	0.003026	-0.020042	0.012846	0.018919	-0.008635
	0.014315	0.007342	0.000855	0.014102	0.005336	-0.009849
	0.019287					
y178	0.010474	-0.016277	0.007384	-0.015771	0.017182	0.016861
	-0.009824	-0.004177	-0.001589	-0.020445	-0.022646	0.005428
	-0.026422	-0.008028	0.008664	0.001020	-0.003213	0.011592
	0.000126	-0.018132	-0.015907	0.026544	-0.002464	0.001451
	-0.013435	0.018135	0.013676	0.006934	0.000644	-0.010361
	0.019083					
z178	-0.023448	-0.037963	0.000079	0.009019	-0.005707	0.017967
	0.028557	-0.010623	-0.000597	-0.003227	0.003167	-0.012079
	0.004209	-0.026809	0.004924	-0.004208	0.008008	0.010366
	-0.017430	0.004329	0.025273	0.019454	0.016196	0.007737
	0.017114	-0.024999	-0.006063	0.019817	-0.004824	-0.015920
	-0.019762					
x179	0.033203	-0.007348	-0.013898	0.000489	-0.006508	-0.031579
	0.006478	-0.001154	0.011330	-0.047546	0.013996	0.001593
	0.007901	-0.020916	-0.037605	0.005213	0.001766	-0.003617
	0.013209	0.003256	-0.019486	0.012924	0.018058	-0.007947
	0.016504	0.006561	0.000302	0.015747	0.006185	-0.005256
	0.020697					

y179	0.008219	-0.016001	0.006924	-0.015814	0.016768	0.018555
	-0.008546	-0.004740	-0.002022	-0.018441	-0.021854	0.002889
	-0.026237	-0.007716	0.010375	0.002056	-0.005012	0.014867
	-0.000273	-0.018253	-0.014930	0.026005	-0.004300	0.001761
	-0.010576	0.017149	0.014979	0.009254	0.000625	-0.010655
	0.017808					
z179	-0.022671	-0.037895	-0.000140	0.007906	-0.006436	0.017823
	0.027499	-0.010719	-0.000136	-0.005657	0.004109	-0.014144
	0.004455	-0.024351	0.004996	-0.005085	0.005433	0.009589
	-0.019342	0.004372	0.023308	0.016986	0.014524	0.008715
	0.017811	-0.025053	-0.002072	0.022139	-0.007868	-0.018880
	-0.025016					
x180	0.033957	-0.005674	-0.013139	-0.000731	-0.006689	-0.031113
	0.006381	-0.002628	0.010382	-0.050509	0.012101	0.005229
	0.006664	-0.022470	-0.040103	0.005080	-0.000946	-0.004931
	0.013558	0.002675	-0.020819	0.011729	0.020539	-0.006980
	0.014646	0.007134	0.002637	0.021079	0.001286	-0.006740
	0.020735					
y180	0.005895	-0.016128	0.006388	-0.015759	0.016294	0.020083
	-0.007335	-0.005539	-0.002597	-0.016195	-0.021346	0.000469
	-0.025797	-0.007355	0.012067	0.003242	-0.007378	0.017232
	-0.000472	-0.018027	-0.014246	0.025130	-0.004507	0.001142
	-0.008382	0.016641	0.016190	0.010735	-0.000352	-0.012585
	0.017023					
z180	-0.021907	-0.037322	-0.000945	0.007443	-0.006986	0.018102
	0.026845	-0.009796	0.000622	-0.007208	0.006627	-0.018195
	0.007111	-0.019933	0.007533	-0.003973	0.004296	0.009822
	-0.017945	0.005140	0.023140	0.016509	0.010643	0.008331
	0.022446	-0.026271	-0.001217	0.020942	-0.005965	-0.016601
	-0.028767					
x181	0.034757	-0.004020	-0.012284	-0.001975	-0.005491	-0.030388
	0.006324	-0.004512	0.009912	-0.053943	0.009880	0.007842
	0.006823	-0.022369	-0.040021	0.006535	-0.001705	-0.004224
	0.013454	0.000963	-0.020545	0.013291	0.019047	-0.005209
	0.014334	0.007867	0.002441	0.024620	-0.001986	-0.005083
	0.019998					
y181	0.003545	-0.015898	0.005936	-0.015556	0.016051	0.021681
	-0.005690	-0.005873	-0.002723	-0.013655	-0.020019	-0.002270
	-0.024897	-0.006012	0.014048	0.004727	-0.008759	0.018407
	0.000085	-0.018123	-0.012542	0.024324	-0.005926	0.000633
	-0.004886	0.016087	0.017314	0.012719	-0.001239	-0.013739
	0.014920					

z181	-0.021185	-0.036447	-0.001768	0.006749	-0.008231	0.017931
	0.026631	-0.008616	0.000899	-0.009210	0.010177	-0.021261
	0.008594	-0.014657	0.007087	-0.004664	0.001209	0.006605
	-0.016126	0.007172	0.020744	0.012267	0.009200	0.006705
	0.025185	-0.027309	0.001696	0.021552	-0.007925	-0.015355
	-0.033965					
x182	0.034985	-0.002254	-0.011596	-0.003346	-0.004576	-0.030025
	0.006708	-0.005676	0.010268	-0.056612	0.009875	0.008140
	0.009279	-0.020476	-0.037775	0.007749	0.000172	-0.001986
	0.014186	0.003621	-0.020954	0.015462	0.016875	-0.002890
	0.015816	0.007655	0.004508	0.025744	-0.004601	-0.001757
	0.016249					
y182	0.001187	-0.015261	0.005716	-0.015551	0.016190	0.023469
	-0.004486	-0.006479	-0.003089	-0.011488	-0.018777	-0.003395
	-0.025161	-0.005417	0.014956	0.005989	-0.010561	0.020368
	-0.000677	-0.019020	-0.010706	0.024223	-0.007263	0.000302
	-0.002847	0.016720	0.016389	0.014149	-0.003203	-0.013320
	0.015000					
z182	-0.020168	-0.035256	-0.002144	0.005889	-0.008623	0.017950
	0.025578	-0.008540	0.000075	-0.012642	0.012134	-0.021001
	0.007995	-0.010765	0.004243	-0.005290	-0.004229	0.002404
	-0.016372	0.005726	0.018518	0.009252	0.008242	0.004574
	0.024663	-0.026373	0.000953	0.023448	-0.012608	-0.013323
	-0.034751					
x183	0.035133	-0.000515	-0.010907	-0.004133	-0.004698	-0.028313
	0.007364	-0.005252	0.009300	-0.058305	0.012844	0.008721
	0.009794	-0.019598	-0.035790	0.007319	0.000095	0.001695
	0.012089	0.005888	-0.019957	0.020509	0.018612	-0.002063
	0.019543	0.003725	0.005084	0.029851	-0.007964	-0.002844
	0.009894					
y183	-0.001291	-0.014729	0.005647	-0.015542	0.016259	0.024900
	-0.002943	-0.007947	-0.002734	-0.009789	-0.018111	-0.004954
	-0.026109	-0.005553	0.016471	0.008027	-0.011164	0.023794
	-0.001364	-0.019132	-0.009697	0.023213	-0.009495	0.000680
	-0.000035	0.017655	0.016003	0.013846	-0.004950	-0.012243
	0.015571					
z183	-0.019313	-0.034051	-0.002157	0.004947	-0.008826	0.017263
	0.024055	-0.010524	0.001076	-0.015702	0.012456	-0.022076
	0.006891	-0.008857	0.003942	-0.004388	-0.005956	0.001324
	-0.017429	0.005247	0.016104	0.005903	0.007610	0.005607
	0.024069	-0.024127	0.003888	0.023072	-0.014304	-0.013901
	-0.026609					

x184	0.035467	0.002562	-0.010101	-0.005672	-0.003311	-0.027231
	0.007775	-0.006521	0.009196	-0.060048	0.012070	0.011351
	0.007258	-0.023715	-0.032781	0.009992	0.004192	0.003399
	0.010030	0.005914	-0.017669	0.022147	0.019558	0.000932
	0.023276	0.001150	0.009667	0.028082	-0.008578	-0.006551
	0.012137					
y184	-0.003840	-0.014411	0.005519	-0.015306	0.016158	0.027098
	-0.002245	-0.008274	-0.001526	-0.005618	-0.014538	-0.007452
	-0.025423	-0.006958	0.017810	0.008694	-0.008620	0.025561
	-0.001151	-0.017991	-0.006722	0.022008	-0.011154	-0.000146
	0.005438	0.013928	0.015517	0.014956	-0.001218	-0.008567
	0.015432					
z184	-0.018201	-0.031657	-0.002278	0.003776	-0.008735	0.017701
	0.021862	-0.008514	0.002716	-0.016641	0.017729	-0.020798
	0.006932	-0.009078	0.002609	-0.004896	-0.001603	-0.002607
	-0.015275	0.003848	0.017423	0.005445	0.005071	0.004769
	0.028465	-0.028814	0.004935	0.021820	-0.009575	-0.007226
	-0.024532					
x185	0.035228	0.005183	-0.009206	-0.007047	-0.002116	-0.025636
	0.007154	-0.006960	0.007729	-0.060209	0.011113	0.014053
	0.005476	-0.027782	-0.031244	0.010689	0.004382	0.003810
	0.012145	0.000494	-0.014533	0.020127	0.015376	0.001432
	0.023412	-0.001728	0.012804	0.024765	-0.008161	0.001622
	0.017057					
y185	-0.006336	-0.013834	0.005138	-0.014621	0.015963	0.028980
	-0.002750	-0.007965	-0.001659	-0.001247	-0.012855	-0.010470
	-0.024522	-0.009134	0.021038	0.009696	-0.005207	0.027131
	0.001194	-0.015173	-0.004951	0.020026	-0.009066	-0.002759
	0.007591	0.010761	0.018902	0.013303	-0.000540	-0.007738
	0.011429					
z185	-0.017448	-0.028759	-0.002651	0.002696	-0.007837	0.017631
	0.019369	-0.006293	0.001623	-0.017692	0.020000	-0.018062
	0.004636	-0.010690	0.003146	-0.005606	0.003340	-0.008865
	-0.011943	0.001712	0.017226	0.005590	0.010212	0.003568
	0.026102	-0.032489	0.015926	0.015117	-0.010978	-0.012167
	-0.028352					
x186	0.034457	0.006966	-0.007352	-0.007150	-0.001506	-0.023953
	0.006317	-0.008261	0.005953	-0.061375	0.011928	0.014124
	0.008181	-0.026170	-0.027468	0.010275	0.004248	0.008221
	0.016134	-0.000882	-0.015739	0.019485	0.014452	0.002520
	0.019415	-0.001372	0.011935	0.023658	-0.018819	0.002715
	0.014039					

y186	-0.009028	-0.013057	0.004013	-0.014112	0.016238	0.030457
	-0.002288	-0.007680	-0.002781	0.001563	-0.012509	-0.011070
	-0.025311	-0.009289	0.021953	0.012080	-0.006407	0.026361
	0.000310	-0.014360	-0.004126	0.019472	-0.009197	-0.007963
	0.006199	0.010552	0.020725	0.012092	0.002724	-0.002986
	0.009600					
z186	-0.017378	-0.027750	-0.003144	0.002382	-0.007955	0.017168
	0.016097	-0.005054	-0.001082	-0.018913	0.018687	-0.015860
	0.004120	-0.007797	0.002197	-0.005548	-0.001255	-0.014128
	-0.010132	-0.001045	0.015273	0.007220	0.013946	-0.001910
	0.017449	-0.029400	0.017493	0.013125	-0.013036	-0.009965
	-0.026478					
x187	0.033769	0.007511	-0.005796	-0.006629	-0.001358	-0.022054
	0.006814	-0.010510	0.004147	-0.062710	0.011435	0.012862
	0.008672	-0.023472	-0.024738	0.010309	0.003153	0.008659
	0.018394	-0.000133	-0.018806	0.017568	0.017298	0.000727
	0.015876	-0.001126	0.012871	0.020354	-0.025190	0.000172
	0.010599					
y187	-0.011597	-0.011800	0.003333	-0.013125	0.016108	0.031978
	-0.003104	-0.006717	-0.003449	0.005686	-0.010720	-0.012377
	-0.024506	-0.010153	0.023967	0.013935	-0.005745	0.030658
	0.000102	-0.014513	-0.001279	0.020713	-0.013414	-0.009605
	0.009278	0.008150	0.019975	0.014878	0.005685	0.004658
	0.010606					
z187	-0.016718	-0.025298	-0.003036	0.002563	-0.008904	0.016980
	0.012206	-0.003977	-0.001992	-0.019972	0.020850	-0.017751
	0.004629	-0.005747	0.004535	-0.003849	-0.002162	-0.007195
	-0.010643	-0.002025	0.015273	0.010962	0.005958	-0.002071
	0.018748	-0.030132	0.016389	0.017077	-0.009984	0.002736
	-0.025329					
x188	0.032529	0.008035	-0.004582	-0.005958	-0.001195	-0.019675
	0.007028	-0.011801	0.002513	-0.061306	0.011934	0.012210
	0.009136	-0.021808	-0.021880	0.010329	0.001938	0.008532
	0.019667	-0.000757	-0.018209	0.018064	0.017374	0.001073
	0.015648	-0.000224	0.014363	0.018017	-0.027972	-0.001849
	0.009554					
y188	-0.013954	-0.010437	0.002934	-0.012240	0.016314	0.033553
	-0.002426	-0.006668	-0.003355	0.006737	-0.009265	-0.014604
	-0.023467	-0.010325	0.027551	0.016582	-0.004874	0.035694
	0.001834	-0.013684	-0.000741	0.017394	-0.014632	-0.011002
	0.011167	0.006163	0.018228	0.014485	0.007707	0.011915
	0.008126					

z188	-0.016006	-0.022726	-0.002514	0.002073	-0.009595	0.016501
	0.008775	-0.004422	-0.002033	-0.023553	0.020987	-0.018272
	0.003231	-0.005250	0.007159	-0.001383	-0.004377	0.000339
	-0.012305	-0.002903	0.013807	0.012286	0.002407	-0.000090
	0.016854	-0.028151	0.015724	0.018613	-0.007973	0.009498
	-0.025422					
x189	0.031782	0.008974	-0.003144	-0.005570	-0.000829	-0.017306
	0.007771	-0.012599	0.001475	-0.060226	0.013596	0.011294
	0.009357	-0.019905	-0.017854	0.010689	0.004896	0.009451
	0.018436	-0.000356	-0.015267	0.020175	0.017794	0.002081
	0.018294	-0.000459	0.014074	0.016898	-0.028740	-0.006943
	0.009879					
y189	-0.016831	-0.010612	0.002440	-0.012225	0.016334	0.034103
	-0.002374	-0.009177	-0.004834	0.008273	-0.012578	-0.012921
	-0.026092	-0.014389	0.025129	0.016480	-0.012910	0.033963
	0.000047	-0.013186	-0.005202	0.012174	-0.008210	-0.011739
	0.003363	0.008061	0.022099	0.011482	0.004021	0.006913
	0.006616					
z189	-0.015960	-0.021309	-0.002117	0.000862	-0.010118	0.015714
	0.005459	-0.006383	-0.003211	-0.026351	0.018315	-0.015929
	0.000006	-0.006725	0.005008	-0.000095	-0.013066	0.000204
	-0.015648	-0.004388	0.009659	0.011723	0.005423	0.002329
	0.009523	-0.023701	0.018867	0.018882	-0.008583	0.004309
	-0.026613					
x190	0.030676	0.008905	-0.001734	-0.005771	-0.000301	-0.015900
	0.008577	-0.016201	-0.001151	-0.060132	0.011972	0.012757
	0.007223	-0.018178	-0.016826	0.011450	0.001509	0.007460
	0.014128	-0.000810	-0.013631	0.019984	0.024120	0.000402
	0.013953	-0.000183	0.013445	0.013621	-0.035895	-0.018438
	0.009871					
y190	-0.018857	-0.009708	0.001823	-0.011646	0.016735	0.036123
	-0.001873	-0.006558	-0.003737	0.011019	-0.008954	-0.016020
	-0.023886	-0.016582	0.026053	0.018456	-0.011606	0.031320
	0.005397	-0.011151	-0.006305	0.008533	-0.010412	-0.008859
	0.007235	0.005537	0.024066	0.012896	0.013013	0.013243
	-0.002145					
z190	-0.015078	-0.019230	-0.001680	0.000111	-0.010134	0.016197
	0.003609	-0.004508	-0.002416	-0.027629	0.022123	-0.018020
	0.001759	-0.005442	0.004549	0.003947	-0.014105	-0.001882
	-0.013386	-0.005415	0.010002	0.013363	-0.000878	0.006717
	0.013382	-0.024598	0.018656	0.022703	0.000896	0.007822
	-0.036317					

x191	0.029120	0.009927	-0.000980	-0.005847	0.001378	-0.012568
	0.009745	-0.017152	-0.002286	-0.059751	0.013867	0.010168
	0.008755	-0.014245	-0.011389	0.013619	-0.001225	0.008600
	0.013473	-0.001564	-0.013058	0.019839	0.024258	0.003176
	0.014523	-0.000965	0.010358	0.008474	-0.034738	-0.018531
	0.001289					
y191	-0.020009	-0.010029	0.002221	-0.010205	0.015591	0.036376
	-0.000375	-0.006182	-0.003938	0.013419	-0.007300	-0.015202
	-0.023532	-0.017815	0.022920	0.018462	-0.007115	0.026020
	0.007805	-0.008902	-0.003632	0.006398	-0.008361	-0.013145
	0.011811	0.003265	0.026426	0.011535	0.011110	0.007473
	-0.000775					
z191	-0.014022	-0.017984	-0.000601	-0.000552	-0.010628	0.015401
	0.001854	-0.004792	-0.002104	-0.030004	0.023653	-0.016195
	0.001005	-0.004293	0.000291	0.006407	-0.015310	-0.006604
	-0.015154	-0.006659	0.011379	0.015707	-0.002174	0.008576
	0.015413	-0.024345	0.019373	0.023876	0.004014	0.001725
	-0.040822					
x192	0.027079	0.010805	-0.000198	-0.007571	0.002506	-0.009199
	0.010752	-0.017975	-0.004265	-0.060050	0.013635	0.008851
	0.007545	-0.013405	-0.007200	0.014365	-0.005184	0.007753
	0.010699	-0.002133	-0.011310	0.016008	0.027980	0.003702
	0.014233	-0.003407	0.009583	0.002455	-0.032406	-0.022766
	0.001667					
y192	-0.021441	-0.009926	0.002268	-0.007251	0.015781	0.036846
	0.000917	-0.005756	-0.004082	0.016937	-0.005699	-0.015642
	-0.020527	-0.016771	0.021693	0.019217	-0.004703	0.024017
	0.012415	-0.006200	-0.001812	0.006335	-0.010890	-0.011742
	0.015985	0.003703	0.023885	0.010281	0.010409	0.008360
	-0.003933					
z192	-0.013595	-0.016078	0.000358	-0.001639	-0.010298	0.014481
	-0.001099	-0.005409	-0.002943	-0.032102	0.021368	-0.013511
	-0.000679	-0.004656	-0.003089	0.008403	-0.021486	-0.006068
	-0.019222	-0.007756	0.011769	0.016866	-0.004752	0.014603
	0.013512	-0.020959	0.016238	0.023877	0.007846	-0.002569
	-0.041493					
x193	0.024903	0.011353	-0.000630	-0.007412	0.002778	-0.004309
	0.013786	-0.016218	-0.005454	-0.057797	0.018881	0.004107
	0.008200	-0.009643	-0.001118	0.015522	-0.005534	0.001902
	0.012131	-0.000647	-0.002554	0.016461	0.028505	-0.000495
	0.014388	-0.005937	0.013028	-0.001391	-0.025305	-0.025241
	-0.004559					

y193	-0.022845	-0.010259	0.002957	-0.005727	0.016427	0.035584
	0.000459	-0.006848	-0.006476	0.017622	-0.008880	-0.012917
	-0.019341	-0.018097	0.017366	0.017898	-0.005673	0.023284
	0.011827	-0.003092	-0.005103	0.001627	-0.012734	-0.009981
	0.015797	0.006055	0.016923	0.009373	0.008104	0.010703
	0.001692					
z193	-0.013649	-0.013543	-0.000182	-0.001349	-0.009598	0.014791
	-0.002788	-0.002775	-0.005103	-0.032403	0.021985	-0.011163
	-0.000433	-0.002952	-0.004418	0.008149	-0.024777	-0.009255
	-0.018750	-0.006897	0.014488	0.017962	-0.011972	0.015299
	0.008149	-0.017847	0.012300	0.026400	0.015520	0.003199
	-0.038995					
x194	0.021133	0.011134	-0.000805	-0.007088	0.002514	0.000947
	0.016730	-0.017221	-0.008017	-0.055868	0.022213	-0.001774
	0.007198	-0.005476	0.007525	0.015539	-0.008129	-0.000229
	0.013433	-0.004032	0.004033	0.016792	0.027838	-0.002556
	0.011335	-0.004640	0.021107	-0.011416	-0.028820	-0.026113
	-0.013505					
y194	-0.023812	-0.008690	0.003201	-0.002983	0.017652	0.034270
	0.003384	-0.004802	-0.009613	0.020260	-0.009117	-0.012537
	-0.014335	-0.018819	0.015058	0.017984	-0.000888	0.020742
	0.015526	0.003940	-0.004737	-0.006482	-0.015622	-0.011811
	0.021223	0.006007	0.007488	0.007489	0.009123	0.016223
	0.009403					
z194	-0.012618	-0.010365	-0.001135	-0.002391	-0.009973	0.013239
	-0.003574	-0.001437	-0.009170	-0.031781	0.020603	-0.013088
	0.001731	-0.002179	-0.004100	0.010332	-0.024276	-0.009422
	-0.014423	-0.010822	0.018068	0.016426	-0.025616	0.012841
	0.003869	-0.011455	0.012564	0.026247	0.012205	0.018541
	-0.038610					
x195	0.017545	0.011093	0.000142	-0.007869	0.003020	0.005200
	0.019693	-0.016702	-0.008717	-0.053498	0.022503	-0.004705
	0.007181	-0.004839	0.014632	0.016732	-0.009551	-0.002399
	0.014936	-0.006135	0.009291	0.011517	0.027945	-0.002832
	0.016382	-0.003560	0.023433	-0.024521	-0.026221	-0.028846
	-0.021388					
y195	-0.024149	-0.008059	0.002879	0.000053	0.017832	0.033081
	0.005693	-0.003518	-0.012207	0.022875	-0.004471	-0.014727
	-0.009914	-0.012696	0.013190	0.019098	0.005890	0.013914
	0.018134	0.008446	-0.001539	-0.009878	-0.015463	-0.013993
	0.025100	0.003603	0.005448	0.011353	0.005395	0.019799
	0.012092					

z195	-0.012090	-0.006935	-0.001224	-0.004242	-0.010264	0.011386
	-0.005474	-0.001262	-0.011432	-0.031149	0.016968	-0.013848
	-0.000163	-0.000222	-0.004341	0.014746	-0.028110	-0.004721
	-0.013993	-0.014682	0.019702	0.015499	-0.035742	0.009389
	0.003060	-0.006963	0.012884	0.025454	0.008928	0.030396
	-0.036431					
x196	0.014330	0.010954	0.000924	-0.008747	0.003745	0.008047
	0.022536	-0.015558	-0.009106	-0.049972	0.024784	-0.007252
	0.008609	-0.002785	0.021218	0.019150	-0.008746	-0.004793
	0.016501	-0.005663	0.014566	0.004314	0.027172	-0.003676
	0.022190	-0.001424	0.022377	-0.033619	-0.028018	-0.028462
	-0.022643					
y196	-0.025111	-0.007779	0.002836	0.003486	0.017242	0.032096
	0.007590	-0.002761	-0.012076	0.024852	-0.003936	-0.015522
	-0.008004	-0.008891	0.011661	0.014990	0.011747	0.011560
	0.016021	0.007460	0.002065	-0.007719	-0.008099	-0.012564
	0.024824	-0.000472	0.006251	0.011506	0.010802	0.013770
	0.010322					
z196	-0.011768	-0.003457	-0.001006	-0.005808	-0.010327	0.009705
	-0.007339	-0.001026	-0.012325	-0.029874	0.013808	-0.013107
	-0.003735	0.000925	-0.003789	0.016607	-0.033489	0.002256
	-0.015967	-0.017404	0.023179	0.014798	-0.038748	0.005325
	0.000362	-0.005802	0.011702	0.023646	0.005906	0.035492
	-0.030960					
x197	0.011251	0.010687	0.001070	-0.008667	0.004418	0.010388
	0.025080	-0.014593	-0.009153	-0.043875	0.025482	-0.009636
	0.011071	0.000640	0.025645	0.020036	-0.006711	-0.005587
	0.015967	-0.004904	0.015696	-0.000781	0.027487	-0.001078
	0.028441	0.000156	0.021850	-0.036630	-0.028945	-0.031916
	-0.020671					
y197	-0.025688	-0.007497	0.003905	0.004896	0.015999	0.027173
	0.006532	-0.003247	-0.009394	0.024757	-0.007834	-0.011211
	-0.006801	-0.011353	0.009092	0.012539	0.018523	0.008295
	0.008805	0.004652	0.007508	-0.005930	-0.004353	-0.012902
	0.019577	0.000373	-0.002456	0.001092	0.018777	0.021202
	0.008675					
z197	-0.011032	-0.000924	-0.000043	-0.006043	-0.010057	0.008334
	-0.008470	-0.000603	-0.012271	-0.027578	0.011881	-0.011837
	-0.008153	0.003550	-0.005206	0.015510	-0.037070	0.005843
	-0.017601	-0.017749	0.025206	0.013892	-0.036701	0.000714
	-0.002070	-0.005711	0.009329	0.019880	0.001329	0.034841
	-0.019320					

x198	0.008200	0.010148	0.001736	-0.008750	0.005187	0.011596
	0.026399	-0.012751	-0.007631	-0.034660	0.024313	-0.009744
	0.013521	0.002514	0.027842	0.020857	0.000427	-0.006297
	0.014161	-0.002769	0.014089	-0.003342	0.027687	0.000652
	0.032858	0.001532	0.015641	-0.039476	-0.027097	-0.029247
	-0.018159					
y198	-0.025732	-0.006627	0.004224	0.007976	0.015498	0.023326
	0.006623	-0.006345	-0.008392	0.019220	-0.008236	-0.010450
	-0.001224	-0.009113	0.002536	0.014084	0.013129	0.007968
	0.007275	-0.001099	0.004987	-0.001427	-0.005432	-0.006297
	0.018274	0.004428	-0.007089	-0.005308	0.029065	0.026925
	0.015634					
z198	-0.009807	0.001522	0.000847	-0.006212	-0.009002	0.007618
	-0.008899	0.000037	-0.012458	-0.024027	0.010309	-0.012787
	-0.011434	0.007649	-0.008207	0.013776	-0.039213	0.007064
	-0.019493	-0.013464	0.024598	0.011130	-0.033351	-0.004066
	-0.002661	-0.007722	0.005337	0.018324	-0.001130	0.031080
	-0.008675					
x199	0.004637	0.009820	0.002900	-0.008478	0.005438	0.012510
	0.027338	-0.010711	-0.004963	-0.024187	0.023005	-0.009135
	0.017281	0.003807	0.028428	0.021283	0.008664	-0.006925
	0.013669	0.000023	0.011630	-0.004264	0.027617	0.001785
	0.037740	0.003082	0.008069	-0.041608	-0.024061	-0.026043
	-0.015762					
y199	-0.024030	-0.004967	0.004820	0.009176	0.014362	0.019435
	0.006488	-0.004913	-0.006287	0.015637	-0.009224	-0.008212
	0.005528	-0.004123	-0.002213	0.015851	0.008910	0.003660
	0.013228	-0.004106	0.003428	-0.002805	-0.005940	0.001119
	0.023227	0.010215	-0.007495	-0.011282	0.032769	0.023884
	0.017471					
z199	-0.008478	0.004280	0.002555	-0.006725	-0.007311	0.007113
	-0.009940	0.000613	-0.012327	-0.019548	0.009386	-0.014433
	-0.016109	0.011548	-0.010705	0.011455	-0.043274	0.009640
	-0.022280	-0.007524	0.022591	0.009635	-0.027482	-0.006401
	-0.004158	-0.012579	0.001232	0.017668	-0.002021	0.029126
	0.001974					
x200	0.000164	0.010035	0.004495	-0.008593	0.005477	0.013520
	0.028359	-0.008798	-0.001634	-0.014136	0.024187	-0.007909
	0.021493	0.002964	0.028881	0.021551	0.016180	-0.005292
	0.015325	0.004533	0.009079	-0.003977	0.027318	0.004889
	0.042899	0.004457	0.001573	-0.042537	-0.018848	-0.024255
	-0.015460					

y200	-0.022737	-0.002989	0.005253	0.007306	0.013114	0.017085
	0.006349	-0.003703	-0.005485	0.010354	-0.008332	-0.003133
	0.014477	-0.007682	-0.007338	0.021358	0.012825	0.009782
	0.024113	0.002469	0.000795	0.000595	-0.000025	0.006665
	0.039447	0.011352	-0.013269	-0.017752	0.024107	0.011315
	0.025285					
z200	-0.007120	0.006851	0.005086	-0.006615	-0.005002	0.007913
	-0.010930	0.001217	-0.011801	-0.013380	0.009176	-0.016014
	-0.020338	0.015955	-0.013572	0.007693	-0.045070	0.010711
	-0.020419	-0.002901	0.019318	0.011477	-0.018123	-0.006075
	-0.000730	-0.019371	-0.003841	0.014942	-0.005518	0.024349
	0.016815					
x201	-0.004811	0.010413	0.006380	-0.009708	0.005228	0.013929
	0.029190	-0.005733	0.003194	-0.003573	0.027025	-0.006719
	0.025537	0.002033	0.030528	0.020677	0.021506	-0.005670
	0.015410	0.008566	0.009200	-0.004725	0.025667	0.007863
	0.044103	0.004125	-0.003432	-0.040156	-0.008895	-0.019187
	-0.021934					
y201	-0.021037	-0.000709	0.005541	0.002488	0.011793	0.013657
	0.004678	0.002004	0.001397	0.012148	-0.002784	-0.002587
	0.019315	-0.006552	-0.007105	0.023364	0.007248	0.010728
	0.022038	0.010320	0.004566	0.000691	0.004648	0.013807
	0.038933	0.003834	-0.010217	-0.011510	0.036908	0.010538
	0.006606					
z201	-0.005792	0.009837	0.008236	-0.006557	-0.002069	0.009182
	-0.011423	0.001856	-0.010292	-0.007423	0.009728	-0.016791
	-0.025874	0.021028	-0.016284	0.003338	-0.048978	0.008090
	-0.018022	-0.001085	0.016815	0.018036	-0.012025	-0.008428
	0.005798	-0.029201	-0.004112	0.010649	-0.001965	0.022041
	0.032713					
x202	-0.009550	0.010006	0.008827	-0.009723	0.005849	0.012251
	0.030363	-0.000386	0.005426	0.007328	0.029585	-0.002467
	0.031178	0.003611	0.029289	0.017565	0.029414	-0.005387
	0.016095	0.010768	0.009931	-0.001331	0.020395	0.007859
	0.043965	0.003348	-0.012957	-0.037450	-0.005402	-0.014377
	-0.020121					
y202	-0.018146	0.000107	0.006656	-0.000262	0.012923	0.007221
	0.001607	0.009571	0.002900	0.015373	0.000536	0.001672
	0.022821	-0.000563	-0.008825	0.020351	0.005099	0.012622
	0.017562	0.015674	0.002984	0.006610	0.010806	0.014557
	0.030424	-0.006291	-0.008512	-0.001599	0.035390	0.004253
	0.002576					

z202	-0.004099	0.010928	0.010541	-0.006559	0.000574	0.011149
	-0.011434	0.000513	-0.009596	0.001255	0.009723	-0.016325
	-0.025331	0.025042	-0.017630	-0.003512	-0.051339	0.005053
	-0.010538	0.011547	0.013537	0.022379	0.003393	-0.010116
	0.014180	-0.036634	-0.004158	0.008515	-0.003401	0.015094
	0.050700					
x203	-0.012869	0.008274	0.011623	-0.008298	0.005423	0.008940
	0.031478	0.006176	0.009454	0.015367	0.030600	0.002949
	0.032651	0.001391	0.030986	0.018012	0.037356	-0.006853
	0.011835	0.005314	0.010569	-0.003016	0.012494	0.005347
	0.040179	0.006221	-0.020609	-0.034599	-0.006152	-0.017159
	-0.008188					
y203	-0.012931	-0.002733	0.006203	0.001681	0.014651	0.001814
	-0.002799	0.015312	0.002703	0.015595	-0.003778	0.006528
	0.020404	-0.000847	-0.008434	0.015055	0.006412	0.014656
	0.009313	0.006493	-0.005415	0.000621	0.007042	0.009322
	0.023296	-0.011386	-0.011071	-0.005209	0.019912	-0.004088
	0.008371					
z203	0.000151	0.010825	0.014385	-0.004103	0.002800	0.007948
	-0.011077	-0.004919	-0.015650	0.003346	0.002678	-0.012746
	-0.025843	0.021833	-0.014718	-0.010799	-0.039197	0.007751
	-0.009227	0.015069	0.015127	0.021984	-0.001472	-0.008228
	0.011453	-0.027114	0.004463	-0.002416	-0.010987	0.004227
	0.052349					
x204	-0.014222	0.006295	0.012514	-0.006527	0.002037	0.004548
	0.033411	0.011407	0.012316	0.017542	0.033973	0.003033
	0.032730	-0.000847	0.034282	0.015018	0.043695	-0.007668
	0.007386	-0.000878	0.014547	-0.005729	0.002618	0.006168
	0.032708	0.012976	-0.025162	-0.037816	0.000139	-0.016958
	-0.009176					
y204	-0.007851	-0.005895	0.003994	0.003291	0.015669	-0.003389
	-0.006074	0.018223	0.001393	0.010866	-0.005502	0.006741
	0.015825	-0.001721	-0.006395	0.007888	0.007316	0.015974
	-0.002919	-0.002162	-0.013163	-0.001945	0.001864	0.005599
	0.010613	-0.014889	-0.012134	-0.010859	0.017379	-0.003955
	0.000549					
z204	0.003250	0.010151	0.018899	-0.003153	0.004942	0.005026
	-0.009392	-0.009134	-0.019633	0.004797	-0.001832	-0.008241
	-0.029051	0.018057	-0.013206	-0.013265	-0.028199	0.009230
	-0.015844	0.012678	0.017461	0.020971	-0.012112	-0.009520
	0.006062	-0.022195	0.009041	-0.012387	-0.007410	0.003886
	0.046623					

x205	-0.016080	0.004177	0.011507	-0.006171	-0.003265	0.001746
	0.032439	0.014665	0.020447	0.015845	0.038185	-0.001209
	0.027784	-0.000745	0.036983	0.017333	0.045553	-0.012459
	0.006935	-0.003944	0.019112	-0.004700	0.003930	0.004369
	0.019831	0.017115	-0.026895	-0.036976	0.017715	-0.017703
	-0.015734					
y205	-0.003818	-0.008913	0.001774	0.003273	0.016344	-0.007462
	-0.010896	0.018741	0.002142	0.005866	-0.006813	0.005415
	0.009196	0.000448	-0.005361	0.003848	0.006750	0.015454
	-0.010298	-0.007982	-0.020591	-0.001409	-0.000878	0.003553
	-0.000215	-0.020055	-0.012646	-0.014981	0.014439	-0.001815
	-0.005446					
z205	0.005147	0.008677	0.022680	-0.003354	0.005347	0.002414
	-0.008651	-0.012662	-0.019900	0.005036	-0.002851	-0.006981
	-0.032232	0.015324	-0.010023	-0.010363	-0.018372	0.008661
	-0.022582	0.012032	0.019674	0.019704	-0.020804	-0.015199
	0.002137	-0.022255	0.015243	-0.021330	0.002879	0.008879
	0.034976					
x206	-0.017034	0.003012	0.008207	-0.004821	-0.007454	-0.000537
	0.032144	0.015869	0.023317	0.013791	0.037937	-0.001840
	0.026038	-0.003750	0.037636	0.015798	0.042287	-0.017451
	0.014616	-0.010073	0.023678	-0.001788	0.012434	0.006531
	0.010545	0.026208	-0.027635	-0.033509	0.024658	-0.028712
	-0.010038					
y206	0.000195	-0.011333	-0.000444	0.002997	0.017740	-0.010818
	-0.015410	0.016963	0.000189	0.002266	-0.009965	0.003540
	0.003748	0.002771	-0.007257	0.001611	0.003326	0.014538
	-0.016074	-0.011378	-0.026761	-0.000930	-0.003282	0.004759
	-0.006070	-0.023139	-0.013294	-0.013589	0.011862	0.001620
	-0.008486					
z206	0.006570	0.007546	0.025740	-0.001991	0.005114	0.000828
	-0.006752	-0.015543	-0.021191	0.005827	-0.007048	-0.004190
	-0.034151	0.010996	-0.010716	-0.010754	-0.007156	0.005190
	-0.019906	0.009942	0.022848	0.018347	-0.024106	-0.013920
	0.005331	-0.019668	0.016975	-0.025887	0.002519	0.011385
	0.030003					
x207	-0.017700	0.002202	0.003785	-0.002087	-0.010170	-0.000958
	0.031477	0.017996	0.021791	0.013933	0.033470	-0.001927
	0.025006	-0.005528	0.036053	0.010983	0.040778	-0.019621
	0.018900	-0.015845	0.027554	-0.001126	0.012639	0.008282
	0.006974	0.031644	-0.022476	-0.029518	0.024603	-0.037707
	-0.004149					

y207	0.003874	-0.013598	-0.002236	0.002561	0.019524	-0.013196
	-0.019518	0.014161	-0.003581	0.000201	-0.014507	0.001330
	-0.001639	0.006110	-0.011567	0.000273	0.000697	0.013945
	-0.022384	-0.013803	-0.032028	-0.001012	-0.007741	0.005309
	-0.009419	-0.026389	-0.013821	-0.008686	0.006680	0.008911
	-0.008351					
z207	0.006818	0.006907	0.027184	0.000416	0.003907	0.001150
	-0.003986	-0.016350	-0.022508	0.008405	-0.012688	-0.000126
	-0.034787	0.006910	-0.013815	-0.013001	0.004223	0.001790
	-0.015371	0.006278	0.024508	0.016306	-0.027102	-0.011324
	0.012205	-0.018853	0.016155	-0.026162	-0.000333	0.020074
	0.028136					
x208	-0.016955	0.000015	-0.000397	0.001390	-0.011268	-0.001320
	0.028466	0.018925	0.016917	0.015097	0.028747	-0.003660
	0.025561	-0.008501	0.031433	0.008614	0.034538	-0.017740
	0.018922	-0.018328	0.027025	-0.001324	0.016469	0.005820
	0.004270	0.033721	-0.017510	-0.024314	0.027505	-0.047808
	-0.000261					
y208	0.007130	-0.016063	-0.003692	0.001395	0.021275	-0.015032
	-0.023520	0.011139	-0.007502	-0.000818	-0.018663	-0.000965
	-0.006042	0.010790	-0.016051	-0.000044	-0.000250	0.013826
	-0.029703	-0.015571	-0.038247	-0.000943	-0.012557	0.002793
	-0.014502	-0.028524	-0.014952	-0.004260	0.003181	0.020988
	-0.009165					
z208	0.006328	0.004687	0.027686	0.003109	0.002686	0.000981
	-0.000896	-0.015960	-0.023563	0.011721	-0.017476	0.005838
	-0.032712	0.002200	-0.018256	-0.015313	0.015674	0.000505
	-0.014832	0.005013	0.020995	0.012530	-0.023839	-0.013473
	0.012916	-0.018995	0.016195	-0.026388	-0.005071	0.027704
	0.025352					
x209	-0.016624	-0.004149	-0.003482	0.003605	-0.009166	-0.002461
	0.025793	0.017300	0.013757	0.012650	0.028040	-0.008393
	0.025111	-0.013826	0.028688	0.007856	0.023163	-0.011752
	0.019641	-0.020691	0.027786	0.000114	0.012650	0.001272
	0.005391	0.031489	-0.012061	-0.030210	0.035060	-0.049769
	-0.003792					
y209	0.010061	-0.018094	-0.004849	-0.001020	0.022399	-0.016356
	-0.026764	0.009086	-0.010588	-0.001878	-0.021738	-0.001273
	-0.007893	0.017627	-0.019252	-0.000138	0.002298	0.012236
	-0.038200	-0.015609	-0.046246	-0.002337	-0.014309	-0.003111
	-0.022364	-0.028079	-0.015103	0.002859	-0.004065	0.032639
	-0.010187					

z209	0.004555	0.001334	0.028432	0.004609	0.003393	-0.001138
	0.004461	-0.017034	-0.022755	0.012342	-0.016266	0.013995
	-0.025826	-0.001990	-0.020615	-0.017592	0.025902	0.002768
	-0.018138	0.005219	0.015327	0.011957	-0.021700	-0.021099
	0.007422	-0.019988	0.016985	-0.037994	-0.008517	0.038845
	0.019646					
x210	-0.017474	-0.009208	-0.007327	0.002818	-0.007084	-0.003934
	0.021090	0.015827	0.011909	0.011376	0.028066	-0.016622
	0.022681	-0.015583	0.027159	0.009131	0.010804	-0.006016
	0.021807	-0.021572	0.027459	0.002927	0.007468	-0.002048
	0.011172	0.023389	-0.013925	-0.034030	0.040680	-0.041064
	-0.006497					
y210	0.013435	-0.018779	-0.005069	-0.002436	0.024172	-0.016922
	-0.027924	0.008835	-0.013588	-0.004949	-0.023553	0.001813
	-0.007337	0.024542	-0.022714	-0.001286	0.007188	0.007457
	-0.046995	-0.013415	-0.054142	-0.003584	-0.013149	-0.013377
	-0.032513	-0.024355	-0.012965	0.011010	-0.013331	0.039987
	-0.011354					
z210	0.000574	-0.003312	0.027728	0.004776	0.003436	-0.003855
	0.008098	-0.017294	-0.020767	0.014584	-0.013786	0.020880
	-0.016839	-0.002559	-0.021509	-0.016383	0.034155	0.005079
	-0.024203	0.008666	0.009067	0.015030	-0.018663	-0.028315
	0.001677	-0.023102	0.011213	-0.043498	-0.013182	0.055887
	0.017419					
x211	-0.016048	-0.013873	-0.011235	0.000664	-0.003941	-0.007233
	0.016093	0.011714	0.008066	0.011378	0.024829	-0.023673
	0.017566	-0.013339	0.021998	0.009553	0.000727	-0.002615
	0.023042	-0.022575	0.024220	0.009494	0.009747	-0.002697
	0.009576	0.015459	-0.020295	-0.030057	0.040367	-0.031867
	-0.002953					
y211	0.016434	-0.017754	-0.004161	-0.002306	0.025246	-0.015918
	-0.028165	0.010873	-0.012738	-0.012147	-0.021450	0.004111
	-0.003659	0.030362	-0.024324	-0.001455	0.014366	-0.000810
	-0.054518	-0.008716	-0.061455	-0.006986	-0.018625	-0.025499
	-0.037680	-0.021782	-0.006769	0.018419	-0.020023	0.048234
	-0.019057					
z211	-0.004312	-0.008861	0.024407	0.006289	0.003569	-0.006974
	0.010776	-0.017399	-0.021290	0.018571	-0.013416	0.028189
	-0.007920	-0.000175	-0.025064	-0.013840	0.042444	0.002178
	-0.029387	0.013426	-0.001537	0.024060	-0.011267	-0.028555
	-0.007693	-0.026405	0.003060	-0.034322	-0.025045	0.069560
	0.019172					

x212	-0.014965	-0.016510	-0.013910	-0.000127	-0.002943	-0.009523
	0.015176	0.010438	0.005164	0.011198	0.020939	-0.025769
	0.015171	-0.013872	0.017799	0.010066	-0.002496	0.000749
	0.021620	-0.021665	0.019558	0.014100	0.007859	-0.007384
	0.006137	0.016739	-0.021889	-0.025060	0.040076	-0.024507
	-0.001866					
y212	0.018375	-0.016147	-0.002097	-0.002178	0.024570	-0.014300
	-0.030779	0.011072	-0.007483	-0.020301	-0.017476	0.001927
	-0.000577	0.033193	-0.021211	-0.003651	0.015479	-0.009414
	-0.053273	-0.006024	-0.062998	-0.010519	-0.022889	-0.024509
	-0.036132	-0.030461	-0.005498	0.021825	-0.022697	0.048808
	-0.023556					
z212	-0.006348	-0.011555	0.021969	0.009583	0.002984	-0.005895
	0.012626	-0.015485	-0.018705	0.017102	-0.013362	0.025693
	-0.003305	0.000526	-0.026892	-0.012800	0.041625	0.000969
	-0.026412	0.010064	-0.010140	0.031077	-0.015698	-0.027340
	-0.009180	-0.025064	0.008320	-0.024207	-0.025490	0.073530
	0.014490					
x213	-0.013314	-0.019241	-0.015941	-0.000815	-0.001346	-0.011792
	0.014007	0.007806	0.002550	0.008510	0.019744	-0.027245
	0.012858	-0.015435	0.013931	0.010701	-0.004359	0.000047
	0.018950	-0.021192	0.011224	0.017518	0.005687	-0.011086
	0.001659	0.016587	-0.024309	-0.020842	0.038538	-0.014965
	-0.003459					
y213	0.020273	-0.014473	0.000546	-0.003618	0.023889	-0.013181
	-0.029933	0.010406	-0.003573	-0.025066	-0.015724	0.000087
	0.001735	0.034215	-0.017816	-0.008046	0.013929	-0.008428
	-0.050065	-0.003792	-0.059653	-0.013496	-0.023004	-0.026942
	-0.033157	-0.035821	-0.000666	0.021900	-0.022594	0.045571
	-0.021396					
z213	-0.008172	-0.014220	0.020573	0.010463	0.003172	-0.005287
	0.013813	-0.015334	-0.017017	0.013997	-0.012685	0.026913
	-0.001272	-0.003926	-0.027119	-0.013052	0.036417	0.002081
	-0.024504	0.008151	-0.016993	0.036885	-0.017513	-0.026239
	-0.012501	-0.026101	0.011065	-0.019524	-0.022327	0.081316
	0.008716					
x214	-0.011147	-0.021178	-0.017367	-0.001145	-0.000113	-0.014271
	0.013258	0.004090	-0.000371	0.004676	0.020340	-0.027502
	0.010490	-0.016961	0.009275	0.011433	-0.005677	-0.001462
	0.014444	-0.018982	0.003669	0.021918	0.002408	-0.017431
	-0.001748	0.015930	-0.025439	-0.016841	0.036321	-0.006254
	-0.001836					

y214	0.022311	-0.013700	0.002970	-0.005085	0.022769	-0.012207
	-0.029263	0.010149	0.000725	-0.029051	-0.015338	-0.002761
	0.001789	0.034558	-0.014069	-0.012268	0.012330	-0.007590
	-0.045247	-0.002952	-0.054290	-0.016728	-0.025115	-0.025147
	-0.030639	-0.038699	0.001841	0.020701	-0.020875	0.040996
	-0.020350					
z214	-0.010157	-0.015985	0.019677	0.011084	0.003775	-0.004567
	0.015278	-0.016329	-0.016355	0.010786	-0.010046	0.028523
	-0.000369	-0.008890	-0.026531	-0.012033	0.029971	0.002171
	-0.022665	0.007526	-0.021119	0.042319	-0.018822	-0.025814
	-0.014235	-0.026436	0.014345	-0.015053	-0.017846	0.084039
	0.005690					
x215	-0.008028	-0.022330	-0.017992	-0.001236	0.000473	-0.017313
	0.011934	0.000201	-0.002458	0.000220	0.022201	-0.028484
	0.007652	-0.016637	0.004725	0.010237	-0.007246	-0.002266
	0.008566	-0.015296	0.001358	0.023758	-0.000829	-0.023445
	-0.006255	0.011724	-0.023441	-0.014256	0.034124	-0.000842
	0.000140					
y215	0.024066	-0.013669	0.004884	-0.006304	0.021693	-0.010969
	-0.027829	0.009317	0.004616	-0.031112	-0.016673	-0.004517
	-0.000585	0.032717	-0.011323	-0.015123	0.011073	-0.008995
	-0.040468	-0.004346	-0.051343	-0.018894	-0.027790	-0.021196
	-0.029051	-0.037336	0.001041	0.019844	-0.018827	0.035466
	-0.018790					
z215	-0.011986	-0.017335	0.019212	0.011481	0.004560	-0.003824
	0.015947	-0.018161	-0.015923	0.007140	-0.006741	0.029049
	-0.000513	-0.013654	-0.025455	-0.009659	0.023553	0.003280
	-0.020607	0.008029	-0.021815	0.044762	-0.017330	-0.024511
	-0.013670	-0.028227	0.017246	-0.013002	-0.013621	0.078804
	0.005070					
x216	-0.004503	-0.022942	-0.017822	-0.000575	0.000180	-0.020679
	0.010982	-0.003244	-0.003706	-0.003888	0.024045	-0.032250
	0.005533	-0.013409	0.003283	0.010214	-0.007479	-0.003238
	0.006288	-0.013463	0.001759	0.021425	-0.008427	-0.027836
	-0.008284	0.004442	-0.024679	-0.014752	0.030320	0.009239
	-0.001390					
y216	0.025622	-0.014161	0.006057	-0.007702	0.021291	-0.009931
	-0.026471	0.007637	0.007089	-0.032674	-0.018117	-0.003474
	-0.005212	0.027884	-0.012162	-0.018768	0.008453	-0.011633
	-0.038947	-0.003602	-0.049870	-0.019536	-0.023644	-0.017151
	-0.030212	-0.033286	0.001542	0.021443	-0.018522	0.020843
	-0.013054					

z216	-0.013813	-0.019102	0.019183	0.012030	0.004949	-0.003148
	0.016091	-0.020765	-0.015979	0.004861	-0.005453	0.028165
	-0.001217	-0.017472	-0.023471	-0.006205	0.018316	0.004734
	-0.015583	0.007096	-0.021524	0.043114	-0.014517	-0.021640
	-0.010690	-0.029629	0.015681	-0.014526	-0.014187	0.070555
	0.006762					
x217	-0.001410	-0.023671	-0.017033	0.000486	-0.001621	-0.025006
	0.010537	-0.006236	-0.004021	-0.004840	0.024545	-0.034111
	0.001437	-0.012278	0.000019	0.008119	-0.008110	-0.005006
	-0.000084	-0.010192	0.000750	0.017201	-0.009440	-0.031561
	-0.012205	-0.003071	-0.019317	-0.017096	0.027449	0.007722
	-0.003543					
y217	0.028010	-0.014047	0.006969	-0.008490	0.021970	-0.007872
	-0.025215	0.005760	0.009350	-0.035403	-0.018168	-0.004116
	-0.007811	0.023525	-0.010041	-0.018446	0.008771	-0.012551
	-0.034680	-0.005100	-0.043488	-0.016170	-0.025356	-0.013088
	-0.026193	-0.028296	-0.004014	0.023491	-0.013257	0.014217
	-0.006839					
z217	-0.016484	-0.020900	0.019002	0.012757	0.004877	-0.002242
	0.016734	-0.022491	-0.015669	0.005195	-0.004764	0.026808
	-0.001826	-0.021918	-0.020804	-0.001271	0.012940	0.006622
	-0.011240	0.006243	-0.020805	0.040390	-0.008762	-0.017584
	-0.006371	-0.029462	0.015233	-0.016138	-0.012484	0.058178
	0.007794					
x218	0.002535	-0.023822	-0.016249	0.002560	-0.002986	-0.027345
	0.010699	-0.007706	-0.003869	-0.006341	0.025103	-0.038603
	0.000811	-0.007852	-0.001134	0.004028	-0.002048	-0.001208
	0.000857	-0.007602	0.000572	0.012710	-0.012486	-0.031169
	-0.007312	-0.013680	-0.020364	-0.018871	0.020023	0.008193
	-0.000508					
y218	0.029814	-0.014544	0.007858	-0.009271	0.022404	-0.007558
	-0.024083	0.002831	0.009846	-0.037151	-0.020221	-0.000781
	-0.012470	0.016507	-0.010424	-0.018736	0.006250	-0.017327
	-0.033820	-0.008247	-0.037518	-0.013093	-0.022426	-0.009082
	-0.026985	-0.018844	-0.007909	0.022840	-0.014405	-0.000259
	0.002555					
z218	-0.019262	-0.023296	0.018513	0.013206	0.005138	-0.000035
	0.017028	-0.023486	-0.015688	0.005859	-0.005541	0.024652
	-0.001244	-0.025331	-0.018622	0.002700	0.009356	0.010870
	-0.003578	0.004324	-0.019188	0.036596	-0.001079	-0.011233
	0.000333	-0.028003	0.011815	-0.017582	-0.013148	0.043663
	0.012017					

x219	0.006612	-0.024854	-0.015508	0.003964	-0.004727	-0.030347
	0.011014	-0.009207	-0.003384	-0.009385	0.024979	-0.039392
	-0.001109	-0.006266	-0.004073	-0.000950	0.001179	-0.002496
	-0.004070	-0.003580	-0.001051	0.008320	-0.008295	-0.029674
	-0.009543	-0.023423	-0.021183	-0.016226	0.017730	0.000398
	0.000959					
y219	0.031221	-0.013138	0.008558	-0.009437	0.023565	-0.006744
	-0.022174	0.001768	0.010890	-0.038047	-0.020485	0.001982
	-0.014674	0.011981	-0.008457	-0.016727	0.006114	-0.020749
	-0.030783	-0.015488	-0.028693	-0.008545	-0.025754	-0.006354
	-0.025271	-0.009419	-0.012008	0.021019	-0.011590	-0.003872
	0.008171					
z219	-0.022510	-0.026295	0.017175	0.013122	0.005401	0.002765
	0.017076	-0.022643	-0.015814	0.006500	-0.006250	0.023732
	0.000258	-0.029280	-0.017188	0.006276	0.004969	0.013510
	0.003431	0.001274	-0.016565	0.033323	0.007041	-0.004470
	0.007370	-0.023636	0.006500	-0.016609	-0.011385	0.027154
	0.016339					
x220	0.000868	-0.031604	-0.024881	0.007803	0.000668	-0.047447
	0.011547	-0.004380	-0.001864	0.013774	0.020205	-0.057594
	0.001814	0.013543	-0.001679	-0.032766	0.006606	0.013898
	-0.015993	-0.018741	-0.010758	-0.001088	-0.065585	-0.024059
	0.052752	-0.042308	-0.015990	-0.008777	-0.000621	-0.031991
	-0.012051					
y220	0.044459	-0.014515	0.010531	-0.019799	0.030944	-0.007174
	-0.022446	-0.002537	0.011546	-0.023659	-0.010812	-0.001777
	-0.021142	-0.009250	0.003577	-0.006086	0.007121	-0.007851
	-0.003468	0.000825	0.003382	0.000355	-0.009747	0.010548
	-0.024102	0.032223	-0.028263	-0.021128	-0.016850	-0.019234
	0.010795					
z220	-0.028578	-0.046142	0.016912	0.011972	0.019380	0.016140
	0.021776	-0.024759	-0.026027	0.019059	-0.033774	0.038770
	-0.000609	-0.039447	-0.002685	0.005346	-0.006857	0.004055
	0.002506	0.024931	0.002017	0.031915	0.045869	0.026651
	-0.010950	0.012191	-0.021037	-0.033458	0.005364	-0.003319
	0.020674					
x221	0.004237	-0.030507	-0.024488	0.008512	0.000881	-0.044502
	0.012253	-0.003546	-0.000461	0.007925	0.022597	-0.052224
	0.000177	0.011957	-0.002705	-0.028048	0.009352	0.016510
	-0.012715	-0.012780	-0.012889	0.003278	-0.055658	-0.019974
	0.046566	-0.035267	-0.013969	-0.009514	-0.002928	-0.030067
	-0.006350					

y221	0.043211	-0.014197	0.011217	-0.018680	0.029870	-0.005887
	-0.021425	-0.004625	0.010782	-0.025562	-0.011844	0.000426
	-0.021878	-0.008970	0.004294	-0.004244	0.009099	-0.006958
	0.001748	0.002505	-0.001201	0.006058	-0.010221	0.011569
	-0.022818	0.033161	-0.024440	-0.015398	-0.019476	-0.016641
	0.010429					
z221	-0.028859	-0.046669	0.014512	0.012791	0.017574	0.015524
	0.024795	-0.023794	-0.021735	0.018410	-0.035042	0.031732
	-0.000295	-0.033884	-0.003358	0.003596	-0.004437	0.006735
	0.000298	0.017534	0.006798	0.032617	0.041651	0.033011
	-0.003878	0.014855	-0.026751	-0.026450	0.003614	-0.009223
	0.016335					
x222	0.007340	-0.029151	-0.024189	0.009456	0.001620	-0.042231
	0.011670	-0.001925	0.000808	0.002399	0.023570	-0.046497
	-0.001205	0.011584	-0.004293	-0.022894	0.009476	0.016741
	-0.010422	-0.008670	-0.016698	0.003946	-0.045635	-0.016253
	0.038595	-0.029032	-0.011287	-0.010183	-0.005905	-0.026986
	-0.003743					
y222	0.041425	-0.014048	0.011616	-0.017310	0.029693	-0.004572
	-0.022139	-0.004900	0.009722	-0.026778	-0.014994	0.003997
	-0.022861	-0.006751	0.003290	-0.001770	0.007027	-0.008226
	0.004849	0.002577	-0.006875	0.007780	-0.007479	0.013339
	-0.024472	0.033307	-0.020374	-0.012655	-0.023677	-0.012383
	0.007869					
z222	-0.028893	-0.046976	0.012276	0.013224	0.015425	0.015265
	0.027889	-0.023735	-0.018271	0.016031	-0.035376	0.025316
	-0.000289	-0.030836	-0.004517	0.001176	-0.001467	0.008953
	-0.002690	0.011049	0.010195	0.033656	0.037501	0.037695
	0.001581	0.016181	-0.031027	-0.018932	0.002136	-0.013258
	0.012346					
x223	0.010007	-0.027930	-0.024342	0.010184	0.002115	-0.040000
	0.010774	-0.000715	0.001606	-0.004292	0.025021	-0.042495
	-0.001042	0.010169	-0.006409	-0.018559	0.010633	0.016446
	-0.009455	-0.003817	-0.020175	0.004989	-0.036868	-0.012969
	0.032723	-0.023912	-0.007246	-0.009771	-0.009078	-0.027231
	-0.002553					
y223	0.039126	-0.014699	0.011533	-0.016729	0.029454	-0.002926
	-0.022684	-0.005377	0.008308	-0.029589	-0.018028	0.006456
	-0.023685	-0.005142	0.000358	-0.001932	0.005132	-0.009956
	0.003430	0.003747	-0.011318	0.009819	-0.003237	0.015775
	-0.025641	0.032829	-0.014860	-0.013399	-0.029004	-0.016342
	0.005104					

z223	-0.028675	-0.046782	0.010365	0.014059	0.013085	0.015075
	0.030289	-0.023406	-0.015479	0.014163	-0.035051	0.020081
	-0.000292	-0.029644	-0.004565	-0.000694	0.000057	0.010125
	-0.003493	0.005102	0.012787	0.031949	0.033710	0.039926
	0.004200	0.017841	-0.032529	-0.010248	0.000377	-0.012268
	0.008225					
x224	0.012562	-0.025805	-0.024603	0.011618	0.002594	-0.037450
	0.009544	0.001764	0.003559	-0.009414	0.026879	-0.041025
	-0.000107	0.008157	-0.006593	-0.014127	0.015580	0.017747
	-0.005742	0.000524	-0.019726	0.009133	-0.031638	-0.009249
	0.031151	-0.020423	-0.004291	-0.009739	-0.007285	-0.025914
	-0.001099					
y224	0.037023	-0.014383	0.011123	-0.015236	0.029177	-0.000555
	-0.023664	-0.003804	0.008215	-0.030366	-0.020004	0.006086
	-0.023928	-0.005104	0.000445	-0.001599	0.009274	-0.008352
	0.005789	0.004490	-0.010802	0.015496	-0.002954	0.018350
	-0.023100	0.030857	-0.011417	-0.013912	-0.027513	-0.018637
	0.003427					
z224	-0.028545	-0.046374	0.008940	0.014880	0.011212	0.015242
	0.031991	-0.022761	-0.012368	0.013177	-0.033617	0.015678
	0.000418	-0.029057	-0.004427	-0.002099	0.001620	0.011497
	-0.006118	0.000460	0.016557	0.031426	0.028971	0.041857
	0.007869	0.018253	-0.032956	-0.003358	0.001392	-0.010857
	0.004209					
x225	0.014986	-0.023625	-0.025211	0.012018	0.002163	-0.034484
	0.009651	0.003505	0.004747	-0.013939	0.030075	-0.038977
	0.001924	0.005865	-0.007049	-0.010214	0.017556	0.019999
	-0.003397	0.003460	-0.020856	0.010514	-0.027550	-0.006607
	0.030644	-0.015943	-0.004944	-0.010542	-0.003696	-0.019488
	0.001935					
y225	0.035117	-0.014027	0.010437	-0.015067	0.027674	0.002796
	-0.022566	-0.003645	0.007487	-0.031339	-0.019764	0.006635
	-0.022937	-0.004989	0.000389	-0.001669	0.009642	-0.005781
	0.006690	0.003298	-0.012582	0.017297	-0.001809	0.019280
	-0.021191	0.030812	-0.012668	-0.015975	-0.024655	-0.013744
	0.006363					
z225	-0.028453	-0.045926	0.007719	0.015586	0.009808	0.015060
	0.033168	-0.021472	-0.009751	0.011925	-0.032304	0.012179
	0.001263	-0.028609	-0.003541	-0.003227	0.002499	0.013020
	-0.007540	-0.001570	0.019605	0.029743	0.024149	0.041520
	0.011409	0.018042	-0.031280	0.002889	-0.000502	-0.009063
	-0.002074					

x226	0.017479	-0.021633	-0.025514	0.011597	0.001689	-0.033390
	0.009533	0.005840	0.006411	-0.018580	0.030010	-0.037206
	0.004365	0.004861	-0.004892	-0.006798	0.018402	0.021640
	-0.000373	0.006843	-0.019861	0.010315	-0.022272	-0.005432
	0.028817	-0.013911	-0.001992	-0.005842	-0.001786	-0.011603
	0.004536					
y226	0.033279	-0.013988	0.009866	-0.015401	0.026318	0.004288
	-0.021632	-0.002769	0.006789	-0.032822	-0.022008	0.006484
	-0.021814	-0.004579	0.003169	-0.002122	0.009883	-0.003090
	0.009320	0.003162	-0.012278	0.017703	-0.000201	0.019141
	-0.021982	0.029468	-0.007818	-0.013045	-0.023805	-0.007923
	0.007795					
z226	-0.028253	-0.045381	0.006599	0.015621	0.008977	0.015118
	0.034147	-0.020171	-0.007006	0.009863	-0.030447	0.009347
	0.003510	-0.027327	-0.003178	-0.003773	0.003711	0.014776
	-0.010384	-0.002109	0.021465	0.027806	0.019891	0.040953
	0.015914	0.016946	-0.032261	0.005524	-0.001862	-0.008111
	-0.008953					
x227	0.019094	-0.019583	-0.024858	0.011637	0.001898	-0.031986
	0.008492	0.008205	0.007894	-0.022894	0.032216	-0.036236
	0.005079	0.002511	-0.004172	-0.002317	0.020886	0.023639
	0.003868	0.007824	-0.018818	0.012674	-0.020461	-0.003411
	0.027008	-0.013287	-0.002782	-0.004986	0.002808	0.000811
	0.008589					
y227	0.031056	-0.013756	0.010068	-0.015441	0.025548	0.006160
	-0.021401	-0.001542	0.006068	-0.033992	-0.021919	0.005931
	-0.021940	-0.005523	0.005068	-0.001186	0.011293	0.000358
	0.012026	0.001476	-0.011394	0.019599	-0.000394	0.019187
	-0.022543	0.027229	-0.006158	-0.013019	-0.021072	0.001548
	0.010849					
z227	-0.028045	-0.045116	0.005726	0.015629	0.008362	0.015327
	0.034559	-0.018940	-0.004668	0.007754	-0.029128	0.007262
	0.003880	-0.027807	-0.003291	-0.005801	0.004755	0.015351
	-0.013085	-0.002195	0.023968	0.025466	0.018013	0.040396
	0.017671	0.014902	-0.032740	0.007100	-0.005587	-0.009037
	-0.013130					
x228	0.020756	-0.018667	-0.023346	0.011764	0.001661	-0.031864
	0.007297	0.008650	0.009879	-0.026793	0.033822	-0.033021
	0.002980	-0.001687	-0.006788	0.000220	0.022397	0.022035
	0.007112	0.008433	-0.021399	0.014540	-0.013574	-0.000659
	0.022173	-0.013951	-0.000946	-0.003072	0.005722	0.006430
	0.013755					

y228	0.029134	-0.013988	0.010669	-0.015170	0.024627	0.007203
	-0.021027	-0.001436	0.005982	-0.034621	-0.021100	0.006364
	-0.022806	-0.007312	0.005899	-0.000984	0.012237	0.002523
	0.014154	-0.000843	-0.013122	0.020956	0.001166	0.019956
	-0.023628	0.024951	-0.002454	-0.011747	-0.018421	0.007072
	0.013575					
z228	-0.027895	-0.044758	0.004601	0.015093	0.007944	0.015687
	0.034475	-0.017451	-0.002641	0.005425	-0.027979	0.006001
	0.004890	-0.028844	-0.003387	-0.008361	0.006317	0.015377
	-0.014930	-0.002598	0.027137	0.023202	0.017355	0.039860
	0.017571	0.011369	-0.032968	0.006530	-0.009788	-0.010527
	-0.015996					
x229	0.022541	-0.017631	-0.023076	0.009572	0.000152	-0.031747
	0.006455	0.009203	0.010800	-0.030401	0.031816	-0.028187
	0.004729	-0.005415	-0.010361	0.000083	0.023687	0.020049
	0.006610	0.009046	-0.022266	0.013163	-0.006263	0.000102
	0.022575	-0.011072	0.002063	-0.003894	0.000995	0.004004
	0.019275					
y229	0.027275	-0.014536	0.010730	-0.016865	0.023285	0.008225
	-0.020728	-0.002100	0.005487	-0.035121	-0.022676	0.008197
	-0.023248	-0.009563	0.004884	-0.003992	0.011578	0.003610
	0.012667	-0.003302	-0.014782	0.020341	0.005151	0.022794
	-0.022655	0.025539	0.003855	-0.012686	-0.023125	0.002325
	0.018603					
z229	-0.028248	-0.044791	0.004361	0.013463	0.007155	0.015535
	0.033990	-0.017891	0.000196	0.003365	-0.026131	0.004872
	0.004346	-0.032535	-0.002371	-0.010184	0.005569	0.016800
	-0.016235	-0.002699	0.029731	0.019438	0.016887	0.038734
	0.014776	0.008104	-0.030654	0.009028	-0.013105	-0.014259
	-0.019988					
x230	0.024307	-0.016112	-0.022664	0.007945	-0.001036	-0.030312
	0.007016	0.008667	0.011444	-0.034598	0.030888	-0.024745
	0.003621	-0.010061	-0.014218	0.002313	0.024128	0.014705
	0.006583	0.007000	-0.021468	0.013941	-0.001734	0.001874
	0.023025	-0.007251	0.006394	0.000914	-0.001247	0.002064
	0.022078					
y230	0.025394	-0.014668	0.010730	-0.017923	0.022128	0.010420
	-0.018900	-0.003185	0.005180	-0.035332	-0.022171	0.007439
	-0.025057	-0.011559	0.005220	-0.003622	0.010835	0.003780
	0.010926	-0.007012	-0.013082	0.021062	0.004322	0.023465
	-0.020378	0.027764	0.007644	-0.009614	-0.024435	0.000436
	0.020441					

z230	-0.028816	-0.045070	0.004488	0.012099	0.006448	0.016350
	0.034484	-0.019176	0.001898	0.002596	-0.022422	0.001192
	0.004922	-0.033598	0.000745	-0.011265	0.005124	0.018049
	-0.016543	-0.004696	0.031255	0.015571	0.013785	0.037439
	0.012307	0.006356	-0.028554	0.013684	-0.016909	-0.017142
	-0.025424					
x231	0.025296	-0.014301	-0.022014	0.007095	-0.001921	-0.029045
	0.005823	0.008335	0.012882	-0.036701	0.031128	-0.022316
	0.005619	-0.013399	-0.016618	0.004568	0.018749	0.011592
	0.009300	0.006746	-0.021001	0.013721	0.004483	0.004013
	0.021476	-0.002435	0.006846	0.003262	0.001152	0.001536
	0.022591					
y231	0.023043	-0.014479	0.010450	-0.018700	0.021120	0.012680
	-0.018089	-0.003395	0.005302	-0.033734	-0.020638	0.006211
	-0.024581	-0.012302	0.007368	-0.002211	0.005413	0.005794
	0.012274	-0.009263	-0.010767	0.020752	0.005550	0.025480
	-0.019625	0.033325	0.006433	-0.007559	-0.022106	-0.000616
	0.022169					
z231	-0.028871	-0.045315	0.003179	0.010801	0.005307	0.017588
	0.034234	-0.018413	0.003301	0.001454	-0.019239	-0.004638
	0.005545	-0.032377	0.003739	-0.011480	0.006140	0.020236
	-0.014968	-0.004006	0.032620	0.012032	0.011691	0.037690
	0.014975	0.005257	-0.028723	0.016694	-0.018832	-0.015379
	-0.026236					
x232	0.026617	-0.013547	-0.020690	0.005394	-0.002592	-0.028280
	0.005883	0.007176	0.014700	-0.037783	0.029055	-0.018962
	0.006622	-0.015998	-0.018128	0.007484	0.017747	0.008950
	0.009913	0.008116	-0.020662	0.015485	0.009988	0.004180
	0.024591	-0.002434	0.012277	0.006204	-0.000143	-0.001485
	0.023981					
y232	0.020931	-0.014980	0.010433	-0.019479	0.020364	0.014480
	-0.016849	-0.004103	0.006031	-0.031521	-0.021278	0.005469
	-0.024169	-0.012667	0.009574	-0.001669	0.004427	0.008104
	0.010874	-0.011308	-0.009013	0.023150	0.006227	0.025940
	-0.016022	0.033293	0.011042	-0.006865	-0.022476	-0.004449
	0.023695					
z232	-0.028559	-0.045481	0.001774	0.009202	0.003922	0.018751
	0.033510	-0.018398	0.004498	-0.000539	-0.016684	-0.010024
	0.007014	-0.032347	0.005981	-0.012072	0.006855	0.020915
	-0.014355	-0.003276	0.031938	0.006830	0.011661	0.037750
	0.015252	0.004114	-0.029524	0.018108	-0.018843	-0.013758
	-0.028515					

x233	0.027904	-0.012143	-0.020039	0.003744	-0.002952	-0.027254
	0.005522	0.007307	0.015658	-0.041096	0.026836	-0.015578
	0.008029	-0.019964	-0.021899	0.008564	0.020018	0.005085
	0.007972	0.010419	-0.021010	0.014196	0.013475	0.002676
	0.023592	-0.003337	0.012630	0.005696	-0.000378	0.000909
	0.023443					
y233	0.018781	-0.014995	0.010138	-0.019475	0.019928	0.016450
	-0.015147	-0.003958	0.005477	-0.030782	-0.021515	0.005413
	-0.024521	-0.013035	0.009745	-0.002289	0.004832	0.008881
	0.007329	-0.012696	-0.008607	0.024482	0.004153	0.023126
	-0.016178	0.032306	0.012379	-0.007034	-0.022211	-0.003349
	0.023953					
z233	-0.027937	-0.045236	0.000786	0.008412	0.002120	0.019717
	0.033292	-0.017744	0.005332	-0.001758	-0.014527	-0.012022
	0.007159	-0.031319	0.006868	-0.011794	0.006544	0.020562
	-0.015627	-0.003092	0.032205	0.005559	0.011791	0.034064
	0.016535	0.002721	-0.028821	0.019735	-0.021518	-0.011831
	-0.030084					
x234	0.029400	-0.010740	-0.020392	0.003494	-0.003853	-0.027318
	0.005056	0.007456	0.016384	-0.045573	0.026224	-0.015354
	0.007345	-0.022718	-0.024857	0.011228	0.018424	0.002404
	0.011501	0.013017	-0.020372	0.013810	0.016159	0.002154
	0.025392	-0.002538	0.011293	0.008197	0.003850	0.003440
	0.026738					
y234	0.016800	-0.014981	0.009307	-0.019125	0.019180	0.017406
	-0.013795	-0.003636	0.005284	-0.030390	-0.020566	0.003812
	-0.025378	-0.012260	0.010508	-0.001417	0.003028	0.010990
	0.006891	-0.012515	-0.007288	0.025892	0.001388	0.020042
	-0.014819	0.034531	0.010734	-0.007394	-0.020044	-0.002365
	0.026362					
z234	-0.027507	-0.045276	0.000186	0.007921	0.000068	0.020206
	0.032433	-0.017720	0.006545	-0.002534	-0.011596	-0.014120
	0.007951	-0.028502	0.006589	-0.011821	0.004588	0.021126
	-0.018143	-0.000600	0.031150	0.005829	0.011600	0.028558
	0.015498	0.005073	-0.028118	0.017018	-0.024028	-0.012747
	-0.031526					
x235	0.030356	-0.010312	-0.018647	0.002088	-0.006082	-0.026299
	0.005423	0.005112	0.018073	-0.047683	0.026834	-0.014518
	0.008105	-0.025410	-0.030211	0.011302	0.019957	-0.000578
	0.013537	0.015263	-0.020282	0.015963	0.019127	0.000059
	0.024773	-0.007576	0.011648	0.009818	0.002267	0.007062
	0.020540					

y235 0.014694 -0.015908 0.009611 -0.018988 0.017359 0.018786
-0.012578 -0.004530 0.006106 -0.028636 -0.019190 0.001595
-0.025736 -0.011367 0.009483 -0.001449 0.003250 0.013136
0.005036 -0.012082 -0.007442 0.027514 -0.000309 0.016300
-0.013049 0.031508 0.010468 -0.007104 -0.020307 -0.000867
0.023448

z235 -0.026898 -0.045697 -0.000603 0.007844 -0.002235 0.020397
0.030793 -0.017348 0.007745 -0.003070 -0.009088 -0.016891
0.008040 -0.025812 0.005821 -0.012045 0.003340 0.020992
-0.020641 0.002768 0.028693 0.004123 0.011845 0.023102
0.015084 0.005852 -0.027548 0.014338 -0.022583 -0.010993
-0.026486

x236 0.031483 -0.009276 -0.018822 0.000919 -0.006494 -0.026254
0.004834 0.003779 0.017341 -0.050734 0.025648 -0.012833
0.009752 -0.027231 -0.030163 0.013632 0.016022 -0.001974
0.018007 0.015404 -0.018521 0.016415 0.025782 0.000079
0.022671 -0.006606 0.012652 0.012374 -0.002504 0.004144
0.026545

y236 0.012523 -0.015823 0.008863 -0.019294 0.016972 0.019620
-0.011394 -0.005012 0.005521 -0.027870 -0.018795 0.001365
-0.025650 -0.010806 0.011218 -0.000820 -0.001010 0.014536
0.005174 -0.014112 -0.005619 0.028885 -0.000306 0.014139
-0.012129 0.033099 0.011021 -0.006109 -0.022764 -0.004539
0.027303

z236 -0.026463 -0.044794 -0.001367 0.006683 -0.003161 0.020547
0.030215 -0.016184 0.008602 -0.005261 -0.006063 -0.016556
0.007334 -0.024128 0.005412 -0.011785 0.000663 0.018950
-0.021884 0.003715 0.026564 0.004846 0.011235 0.021872
0.015961 0.006828 -0.025018 0.011316 -0.021550 -0.013714
-0.027753

x237 0.032628 -0.007784 -0.018455 -0.000441 -0.005993 -0.025141
0.005728 0.002789 0.016034 -0.052556 0.024732 -0.009466
0.009566 -0.028861 -0.033544 0.015781 0.019266 -0.001626
0.020233 0.013547 -0.018936 0.015271 0.027066 0.003170
0.021471 -0.001341 0.015284 0.013286 -0.003348 0.000789
0.029191

y237 0.010240 -0.015654 0.008474 -0.019278 0.016692 0.020740
-0.009692 -0.005754 0.004970 -0.026310 -0.018599 0.000595
-0.026570 -0.010231 0.010964 -0.000142 -0.001905 0.017735
0.003767 -0.016125 -0.005077 0.029810 -0.002539 0.012997
-0.011630 0.034984 0.011147 -0.005850 -0.022399 -0.006270
0.030091

z237	-0.026037	-0.042999	-0.002501	0.005998	-0.004011	0.020371
	0.030092	-0.014957	0.008654	-0.008520	-0.003111	-0.015499
	0.005556	-0.022616	0.004867	-0.011582	-0.002283	0.016736
	-0.023941	0.003722	0.024659	0.008847	0.009658	0.020310
	0.013828	0.007318	-0.021954	0.008472	-0.021821	-0.014482
	-0.027419					
x238	0.033361	-0.006875	-0.017972	-0.001389	-0.005569	-0.024728
	0.006680	0.001630	0.015249	-0.054080	0.027174	-0.006633
	0.011623	-0.030815	-0.033118	0.017907	0.015414	-0.003968
	0.021692	0.013911	-0.021812	0.016659	0.031280	0.003982
	0.016006	-0.002544	0.021376	0.015608	-0.001700	0.002529
	0.020560					
y238	0.008054	-0.016002	0.008189	-0.018709	0.016689	0.021561
	-0.008429	-0.006186	0.004610	-0.024531	-0.017002	0.000051
	-0.025740	-0.009338	0.012668	0.000337	-0.004445	0.018710
	0.002023	-0.016889	-0.005392	0.030490	-0.002804	0.010231
	-0.011117	0.033495	0.012088	-0.005992	-0.019715	-0.006411
	0.027810					
z238	-0.024994	-0.042323	-0.003064	0.006641	-0.004171	0.020515
	0.029353	-0.013685	0.008998	-0.010391	-0.000181	-0.017045
	0.007406	-0.017853	0.006441	-0.010901	-0.000890	0.015667
	-0.024448	0.006434	0.023678	0.009595	0.008418	0.015856
	0.017385	0.004477	-0.022445	0.004531	-0.019183	-0.013594
	-0.022532					
x239	0.034278	-0.003626	-0.017049	-0.002397	-0.004303	-0.023656
	0.007490	0.001694	0.013931	-0.054538	0.026809	-0.001259
	0.012791	-0.031083	-0.030250	0.021219	0.011303	0.004574
	0.022616	0.014845	-0.022147	0.018855	0.027010	0.004782
	0.016428	0.001517	0.021222	0.017455	-0.002956	0.007642
	0.017094					
y239	0.005489	-0.016038	0.007987	-0.018229	0.016605	0.023002
	-0.007226	-0.006181	0.004028	-0.021130	-0.015811	-0.000516
	-0.024682	-0.006802	0.014326	0.001284	-0.006509	0.020492
	0.001756	-0.017724	-0.004811	0.029915	-0.004197	0.006029
	-0.008167	0.032010	0.011290	-0.005553	-0.020556	-0.007259
	0.026407					
z239	-0.024145	-0.040999	-0.003464	0.006197	-0.004655	0.020554
	0.027403	-0.012340	0.009654	-0.012682	0.002632	-0.017981
	0.007800	-0.013741	0.007380	-0.010220	-0.000894	0.012551
	-0.025816	0.008734	0.021341	0.006729	0.009608	0.012318
	0.017801	0.000725	-0.020414	0.003528	-0.020039	-0.012431
	-0.019218					

x240	0.035055	-0.001565	-0.015780	-0.004177	-0.003727	-0.023579
	0.008440	-0.000438	0.012763	-0.056717	0.022946	0.004808
	0.009496	-0.035764	-0.031095	0.022925	0.009634	0.007445
	0.017369	0.017676	-0.022262	0.018383	0.032023	0.009137
	0.008420	0.005420	0.023158	0.020636	-0.007187	0.001975
	0.021959					
y240	0.002837	-0.016310	0.007890	-0.017912	0.016196	0.024239
	-0.006120	-0.006942	0.003621	-0.018415	-0.015118	-0.001224
	-0.025413	-0.005625	0.013808	0.000746	-0.008032	0.020666
	0.000908	-0.019119	-0.003476	0.030398	-0.003866	0.003354
	-0.007804	0.031439	0.011606	-0.004888	-0.023381	-0.010730
	0.029009					
z240	-0.023365	-0.039494	-0.004108	0.005668	-0.005824	0.021031
	0.025381	-0.010698	0.009774	-0.015018	0.005829	-0.019766
	0.007777	-0.009804	0.006723	-0.010454	-0.001001	0.011357
	-0.024378	0.009588	0.019762	0.007844	0.009494	0.009108
	0.018132	-0.001014	-0.019340	0.001431	-0.021217	-0.008973
	-0.017100					
x241	0.035871	0.000037	-0.014757	-0.004678	-0.003848	-0.022928
	0.009578	-0.001338	0.009847	-0.056935	0.019803	0.009745
	0.008280	-0.036330	-0.032572	0.026132	0.009387	0.008520
	0.012448	0.019722	-0.019511	0.020625	0.033776	0.003313
	0.000748	0.011518	0.024080	0.019302	-0.004954	0.004755
	0.030313					
y241	0.000271	-0.016156	0.007720	-0.017444	0.015965	0.025549
	-0.005137	-0.007455	0.003084	-0.015551	-0.014153	-0.002490
	-0.025661	-0.004217	0.013872	0.000924	-0.008864	0.021782
	0.000240	-0.020692	-0.002039	0.031452	-0.005393	0.001133
	-0.006326	0.030637	0.011643	-0.003887	-0.024555	-0.010719
	0.031438					
z241	-0.022424	-0.037847	-0.004556	0.005523	-0.007250	0.021062
	0.023641	-0.009387	0.010027	-0.017035	0.008767	-0.021241
	0.007781	-0.006237	0.005410	-0.010082	-0.000022	0.009765
	-0.024328	0.010551	0.018527	0.010547	0.008236	0.004754
	0.018216	-0.002008	-0.017056	0.000135	-0.022746	-0.006253
	-0.015209					
x242	0.036796	0.003035	-0.014182	-0.004343	-0.005370	-0.020827
	0.011358	-0.001607	0.010028	-0.058007	0.022926	0.008776
	0.008694	-0.034075	-0.030711	0.027902	0.011983	0.015754
	0.013562	0.025574	-0.018338	0.022685	0.023661	-0.002129
	0.007501	0.011627	0.020439	0.020571	-0.000230	0.012340
	0.022490					

y242	-0.002499	-0.016260	0.007599	-0.017077	0.015403	0.026566
	-0.004360	-0.008611	0.002280	-0.012766	-0.013984	-0.002958
	-0.026963	-0.003494	0.012752	0.000244	-0.010574	0.021967
	-0.001758	-0.021719	-0.001771	0.031841	-0.005765	-0.002258
	-0.005959	0.030910	0.012076	-0.002472	-0.027250	-0.014974
	0.032854					
z242	-0.021665	-0.036527	-0.004888	0.005094	-0.008790	0.020841
	0.021015	-0.008608	0.009967	-0.018594	0.010930	-0.022759
	0.007462	-0.002838	0.003867	-0.009612	-0.000979	0.007118
	-0.023712	0.010939	0.015753	0.012096	0.008163	0.001152
	0.016244	-0.002139	-0.014120	-0.000291	-0.022800	-0.006354
	-0.011796					
x243	0.037978	0.005916	-0.012507	-0.004743	-0.003710	-0.019446
	0.011510	-0.004011	0.010514	-0.058411	0.023775	0.008006
	0.009983	-0.033473	-0.029571	0.029821	0.016991	0.018092
	0.014895	0.026709	-0.015872	0.021998	0.017914	0.003840
	0.009095	0.015836	0.022970	0.022817	0.004261	0.007643
	0.015726					
y243	-0.004958	-0.015961	0.007463	-0.016291	0.014963	0.027748
	-0.003268	-0.009121	0.001706	-0.009517	-0.012698	-0.004935
	-0.026674	-0.002669	0.012595	0.000497	-0.010483	0.022693
	-0.002369	-0.022056	-0.000227	0.031900	-0.008375	-0.003546
	-0.002581	0.030821	0.011407	-0.000717	-0.026177	-0.014485
	0.031635					
z243	-0.020945	-0.035050	-0.005126	0.004676	-0.010023	0.020730
	0.019114	-0.007552	0.009311	-0.019730	0.013494	-0.022870
	0.006980	-0.001185	0.001027	-0.009260	-0.001823	0.003624
	-0.022245	0.010592	0.013600	0.012304	0.008509	0.000864
	0.015182	-0.000619	-0.011122	-0.000291	-0.020869	-0.007879
	-0.012211					
x244	0.037286	0.008030	-0.011081	-0.006374	-0.003288	-0.017946
	0.011193	-0.007050	0.008126	-0.059178	0.018996	0.014135
	0.008767	-0.035323	-0.031967	0.030857	0.013892	0.012604
	0.013810	0.024922	-0.020036	0.020053	0.026348	0.006913
	-0.000390	0.017981	0.023679	0.013557	-0.005199	-0.000969
	0.017309					
y244	-0.007430	-0.015838	0.007389	-0.015636	0.014397	0.029033
	-0.002488	-0.009464	0.001474	-0.006433	-0.010778	-0.007521
	-0.025976	-0.001566	0.012115	0.000963	-0.010669	0.024274
	-0.002982	-0.021667	0.001350	0.031346	-0.011806	-0.004717
	0.002390	0.030644	0.010885	0.002696	-0.025029	-0.010479
	0.029985					

z244	-0.020027	-0.032244	-0.005104	0.003667	-0.011014	0.020702
	0.017755	-0.005533	0.009301	-0.022222	0.016938	-0.022361
	0.006975	0.001078	-0.000678	-0.007114	-0.000641	0.001031
	-0.020565	0.009688	0.012763	0.014265	0.004286	0.001809
	0.018264	0.000106	-0.006359	0.001071	-0.020637	-0.002461
	-0.013220					
x245	0.036763	0.008913	-0.009345	-0.007655	-0.003517	-0.017039
	0.010260	-0.007823	0.004949	-0.060600	0.017866	0.017487
	0.006641	-0.036398	-0.030278	0.029510	0.009193	0.014327
	0.015150	0.028688	-0.020240	0.021438	0.029077	0.007791
	-0.001103	0.012999	0.024790	0.018575	-0.009637	0.002299
	0.014340					
y245	-0.010192	-0.014849	0.007228	-0.015042	0.014352	0.030671
	-0.001520	-0.009727	0.001866	-0.003064	-0.009143	-0.010410
	-0.024848	-0.000191	0.012206	0.002941	-0.010141	0.025173
	-0.002321	-0.022415	0.002392	0.029787	-0.015460	-0.006486
	0.007412	0.031421	0.011267	0.001003	-0.023475	-0.006824
	0.028542					
z245	-0.019250	-0.029998	-0.004622	0.002377	-0.012498	0.020240
	0.014824	-0.004586	0.009793	-0.024780	0.019093	-0.022855
	0.006118	0.002821	-0.002278	-0.004878	-0.002674	0.000882
	-0.019502	0.010661	0.010389	0.015324	0.002011	0.002345
	0.019680	0.001296	-0.002021	0.002458	-0.019853	0.002712
	-0.011563					
x246	0.036620	0.011467	-0.008026	-0.008275	-0.002847	-0.014783
	0.009997	-0.008801	0.005092	-0.061513	0.017228	0.014894
	0.005271	-0.032878	-0.023712	0.029003	0.009872	0.017808
	0.017399	0.027742	-0.020339	0.021994	0.022744	0.006137
	0.003708	0.010131	0.024032	0.021923	-0.006844	0.005846
	0.010053					
y246	-0.013322	-0.014643	0.007110	-0.014770	0.014200	0.031790
	-0.000843	-0.010176	0.000824	0.000815	-0.008153	-0.010945
	-0.024665	-0.001099	0.010272	0.003799	-0.012042	0.022863
	-0.001151	-0.023656	0.003275	0.026459	-0.015197	-0.007423
	0.008401	0.031177	0.012629	0.001882	-0.025313	-0.007865
	0.027992					
z246	-0.018761	-0.027769	-0.004471	0.000943	-0.013640	0.019884
	0.011571	-0.004074	0.009814	-0.025976	0.020919	-0.022956
	0.004814	0.003547	-0.004555	-0.002573	-0.005651	-0.000564
	-0.017446	0.009307	0.008670	0.015274	-0.000811	0.003254
	0.019012	0.002929	0.002324	0.005669	-0.019256	0.004047
	-0.011034					

x247	0.035432	0.013308	-0.006408	-0.009088	-0.000507	-0.012753
	0.008012	-0.011336	0.002694	-0.059738	0.018165	0.017153
	0.005731	-0.032183	-0.022214	0.024721	0.008465	0.016107
	0.016301	0.023136	-0.019220	0.026307	0.022333	0.006018
	0.002684	0.009132	0.025383	0.015160	-0.015425	0.004417
	0.014170					
y247	-0.016205	-0.014265	0.007172	-0.014100	0.013822	0.033483
	0.000004	-0.009265	0.000535	0.003764	-0.007772	-0.014445
	-0.022254	-0.001403	0.011834	0.006889	-0.012178	0.020688
	0.002732	-0.023439	0.004140	0.018706	-0.016623	-0.009526
	0.011563	0.029226	0.011972	0.005235	-0.020126	-0.004246
	0.023112					
z247	-0.018534	-0.025054	-0.003465	-0.000396	-0.013474	0.019808
	0.008574	-0.002057	0.008315	-0.027649	0.022337	-0.021120
	0.005957	0.003230	-0.005622	-0.000930	-0.007882	-0.006641
	-0.014283	0.004129	0.008938	0.014942	-0.003079	0.004698
	0.017331	0.004939	0.005761	0.008916	-0.018079	0.006292
	-0.010462					
x248	0.033855	0.013861	-0.005409	-0.009310	0.000630	-0.011271
	0.008139	-0.013412	-0.000638	-0.059797	0.018347	0.014091
	0.005941	-0.030500	-0.018193	0.024407	0.003221	0.015145
	0.015824	0.023370	-0.015278	0.024227	0.023297	0.006971
	0.001715	0.004646	0.022444	0.010942	-0.017987	0.007095
	0.010870					
y248	-0.018694	-0.013291	0.006782	-0.012814	0.013681	0.035412
	0.000249	-0.009077	-0.001138	0.007604	-0.009169	-0.014768
	-0.019449	-0.003383	0.011200	0.007183	-0.010304	0.019076
	0.002818	-0.022685	0.005246	0.014706	-0.017430	-0.014248
	0.012502	0.028828	0.010893	0.006891	-0.016456	-0.002881
	0.020947					
z248	-0.017620	-0.022764	-0.003410	-0.001496	-0.014822	0.018771
	0.004645	-0.001181	0.005611	-0.029815	0.020370	-0.021468
	0.007872	0.001810	-0.006746	0.001319	-0.011458	-0.007210
	-0.017218	0.004937	0.010408	0.014276	-0.004627	0.002307
	0.012304	0.005035	0.003095	0.011085	-0.013437	0.011385
	-0.010170					
x249	0.032543	0.014782	-0.004400	-0.011145	0.001198	-0.007731
	0.008192	-0.013773	-0.000651	-0.059036	0.019289	0.009268
	0.005889	-0.025579	-0.012250	0.025996	0.001069	0.014467
	0.017074	0.023004	-0.009579	0.020238	0.026001	0.001634
	0.008784	-0.001287	0.025790	0.008053	-0.019814	0.008391
	0.007009					

y249	-0.021302	-0.012620	0.006149	-0.011102	0.013804	0.035740
	0.000761	-0.009909	-0.003379	0.011380	-0.009830	-0.012174
	-0.018827	-0.004695	0.008792	0.007315	-0.011700	0.017231
	0.003248	-0.021911	0.004231	0.014457	-0.017811	-0.016993
	0.010039	0.027133	0.009599	0.007795	-0.018777	-0.003275
	0.022940					
z249	-0.016408	-0.019790	-0.003699	-0.003395	-0.015668	0.018427
	0.001453	-0.000064	0.005632	-0.030818	0.020456	-0.019236
	0.007286	0.003678	-0.008250	0.004887	-0.014713	-0.005917
	-0.018046	0.006766	0.011457	0.016544	-0.004582	-0.003334
	0.011394	0.004368	0.005266	0.013165	-0.011077	0.015718
	-0.008379					
x250	0.030737	0.015692	-0.003721	-0.011743	0.003314	-0.005279
	0.007701	-0.015511	-0.002046	-0.059901	0.019086	0.007392
	0.004837	-0.023277	-0.007986	0.021771	-0.004032	0.012267
	0.018746	0.015962	-0.005619	0.015835	0.030518	0.006347
	0.008641	-0.005482	0.025369	0.003173	-0.023590	0.001085
	0.006125					
y250	-0.023281	-0.012345	0.005297	-0.008802	0.013710	0.037160
	0.002849	-0.009859	-0.004813	0.016152	-0.008316	-0.013471
	-0.016827	-0.003273	0.008788	0.010596	-0.008653	0.016517
	0.005510	-0.016920	0.003950	0.013714	-0.019858	-0.021924
	0.011112	0.025059	0.009648	0.008113	-0.016229	0.002289
	0.020217					
z250	-0.015589	-0.015974	-0.004739	-0.003687	-0.014496	0.017781
	-0.001063	0.000467	0.004296	-0.033087	0.020463	-0.015828
	0.005239	0.005213	-0.008828	0.005246	-0.018311	-0.002319
	-0.019143	0.003129	0.010762	0.022391	-0.009147	-0.000156
	0.005283	0.004588	0.005294	0.016053	-0.009833	0.019318
	-0.004695					
x251	0.028425	0.015930	-0.002236	-0.011196	0.003899	-0.001465
	0.009553	-0.016406	-0.003183	-0.060480	0.022034	0.004646
	0.003269	-0.020595	-0.003534	0.017089	-0.005104	0.009640
	0.016725	0.012361	-0.003795	0.013005	0.032668	0.009166
	0.005835	-0.003946	0.025005	-0.002886	-0.026948	-0.008922
	0.004796					
y251	-0.025147	-0.011604	0.003917	-0.006109	0.015178	0.037583
	0.004266	-0.010109	-0.006580	0.019891	-0.007868	-0.014034
	-0.014446	-0.001159	0.008273	0.012948	-0.008958	0.015715
	0.009580	-0.013248	0.004621	0.012103	-0.021895	-0.025746
	0.014179	0.024419	0.008411	0.007875	-0.011774	0.007516
	0.018690					

z251	-0.015012	-0.012904	-0.004927	-0.003464	-0.013927	0.017281
	-0.002395	0.001702	0.003942	-0.035135	0.022746	-0.013737
	0.004337	0.007232	-0.008953	0.004717	-0.020086	-0.000336
	-0.019418	0.001974	0.008407	0.025727	-0.013327	0.003385
	0.000685	0.009472	0.003883	0.017339	-0.006514	0.019438
	-0.001108					
x252	0.026666	0.015869	-0.000586	-0.010736	0.005655	0.002404
	0.011602	-0.016001	-0.006083	-0.060748	0.025291	0.001261
	0.002562	-0.014242	0.003808	0.016203	-0.005266	0.008441
	0.016132	0.009726	-0.001401	0.010628	0.030953	0.004268
	0.008380	-0.008777	0.021713	-0.011004	-0.021205	-0.008264
	0.000223					
y252	-0.027474	-0.011170	0.002950	-0.003599	0.015207	0.037339
	0.005203	-0.011286	-0.007108	0.023754	-0.009680	-0.011518
	-0.013307	-0.003145	0.004987	0.012003	-0.010187	0.011416
	0.009458	-0.010451	0.003525	0.009654	-0.016477	-0.024564
	0.013105	0.026282	0.010722	0.009574	-0.016267	-0.001949
	0.020510					
z252	-0.014340	-0.010444	-0.003736	-0.003353	-0.012692	0.016628
	-0.005099	0.002832	0.003631	-0.035067	0.023808	-0.010420
	0.004174	0.011119	-0.009641	0.006240	-0.022889	0.001327
	-0.020292	0.000221	0.003640	0.027224	-0.016122	0.003047
	0.002321	0.009057	0.003337	0.018335	0.001610	0.017737
	-0.000777					
x253	0.024434	0.016836	0.000207	-0.010057	0.006305	0.006592
	0.013409	-0.016679	-0.007516	-0.060356	0.027141	-0.002391
	0.001462	-0.011026	0.008194	0.017849	-0.009656	0.005702
	0.017864	0.008026	0.003771	0.005193	0.033790	0.002466
	0.011226	-0.010594	0.025139	-0.020212	-0.025627	-0.012067
	-0.009118					
y253	-0.029116	-0.011809	0.002565	-0.001170	0.016543	0.036835
	0.007015	-0.010341	-0.009073	0.026920	-0.007610	-0.010521
	-0.009685	-0.000678	0.005647	0.010844	-0.007052	0.010460
	0.009636	-0.007729	0.004254	0.010395	-0.018035	-0.024101
	0.015558	0.024217	0.004093	0.014585	-0.012347	-0.000413
	0.024330					
z253	-0.013522	-0.007376	-0.003576	-0.003237	-0.011933	0.016103
	-0.007349	0.003057	0.004307	-0.032850	0.024743	-0.007666
	0.003287	0.013225	-0.010746	0.011675	-0.028163	0.003839
	-0.017486	0.000651	0.001242	0.026778	-0.015296	0.004302
	0.003593	0.009059	0.005419	0.015827	0.000526	0.017195
	-0.001476					

x254	0.022088	0.017844	0.000153	-0.008560	0.006510	0.010990
	0.016437	-0.017256	-0.008795	-0.060549	0.028996	-0.007426
	0.004129	-0.008691	0.015652	0.016134	-0.012715	0.002462
	0.018912	0.002600	0.014821	-0.003354	0.032548	0.001243
	0.018942	-0.009360	0.030299	-0.026800	-0.023861	-0.019258
	-0.018775					
y254	-0.030385	-0.013016	0.002576	0.000643	0.018278	0.036326
	0.010060	-0.009388	-0.012858	0.029178	-0.005774	-0.008635
	-0.009998	0.003235	0.007041	0.012777	-0.004117	0.009789
	0.009160	-0.000808	-0.001789	0.011758	-0.011978	-0.022990
	0.011768	0.021691	-0.003498	0.012099	-0.013700	-0.000283
	0.027884					
z254	-0.012644	-0.004601	-0.004151	-0.002607	-0.011840	0.016060
	-0.008456	0.002470	0.004548	-0.033280	0.024814	-0.006226
	0.005194	0.013669	-0.012333	0.011532	-0.030758	0.004318
	-0.012902	-0.003045	0.001642	0.025564	-0.013300	0.007740
	0.005172	0.011195	0.002669	0.016670	0.004597	0.012774
	-0.004076					
x255	0.019484	0.016398	0.000519	-0.008369	0.006814	0.014973
	0.020525	-0.017142	-0.013129	-0.061037	0.030607	-0.009798
	0.003589	-0.007587	0.023514	0.016654	-0.013680	-0.001399
	0.016765	0.000588	0.020165	-0.009875	0.033649	-0.001531
	0.020200	-0.007673	0.035504	-0.040785	-0.027190	-0.028548
	-0.023038					
y255	-0.031256	-0.010805	0.002027	0.003939	0.019448	0.035302
	0.011768	-0.008507	-0.012172	0.028212	-0.002479	-0.011315
	-0.004720	0.008963	0.006513	0.012027	-0.002353	0.008680
	0.012685	0.002251	0.001761	0.010923	-0.009365	-0.022111
	0.018768	0.019900	-0.012048	0.018406	-0.009307	0.002790
	0.028091					
z255	-0.012035	-0.002781	-0.003979	-0.002986	-0.011984	0.016741
	-0.009254	0.004617	0.003524	-0.032872	0.026541	-0.005685
	0.007264	0.015246	-0.011790	0.012729	-0.030052	0.004111
	-0.008351	-0.006133	-0.000715	0.024943	-0.012109	0.008606
	0.007802	0.012078	-0.001803	0.011091	0.007693	0.007310
	-0.003790					
x256	0.017077	0.014543	0.000025	-0.009311	0.009119	0.018385
	0.026721	-0.016978	-0.019220	-0.060717	0.033149	-0.014595
	0.004980	-0.004236	0.036397	0.018049	-0.012876	-0.001013
	0.016287	0.003957	0.024468	-0.016449	0.036620	0.001024
	0.022720	-0.010041	0.040901	-0.050491	-0.033971	-0.026964
	-0.024272					

y256	-0.031739	-0.009397	0.001868	0.007536	0.019077	0.034006
	0.013253	-0.007522	-0.010989	0.026098	0.000854	-0.011247
	-0.002368	0.013048	0.005029	0.011249	-0.001461	0.005919
	0.014325	0.001729	0.004988	0.009242	-0.009003	-0.021980
	0.023528	0.022180	-0.017045	0.019759	-0.003239	-0.004470
	0.028888					
z256	-0.011664	-0.002732	-0.004153	-0.003352	-0.009700	0.017071
	-0.008686	0.005282	0.002430	-0.028472	0.027515	-0.007149
	0.010803	0.019539	-0.010370	0.012384	-0.026817	0.003823
	0.001789	-0.003904	-0.005525	0.022618	-0.007534	0.014083
	0.013131	0.009322	-0.006210	0.006919	0.004941	0.005576
	-0.001037					
x257	-0.025823	-0.046815	-0.033728	0.003632	-0.000011	0.000664
	0.005831	0.015091	-0.003776	0.001697	0.027957	-0.024151
	0.025264	-0.000334	0.001428	0.021929	-0.028899	0.040357
	0.008930	-0.035545	-0.015594	0.018165	0.000995	-0.037383
	-0.029026	0.050848	-0.057972	0.011959	0.009471	-0.010483
	0.010436					
y257	-0.015376	-0.062013	0.010740	-0.042193	0.021794	0.013230
	-0.042997	-0.018930	-0.099719	0.000490	-0.048734	-0.057864
	0.000114	0.031122	0.025039	0.024106	-0.004502	0.061954
	0.107394	0.009063	-0.133247	-0.035796	-0.015177	0.003321
	-0.019925	-0.066538	-0.028816	-0.000478	0.096728	0.043599
	-0.079526					
z257	-0.002817	-0.006176	0.054291	0.021229	-0.016415	-0.017146
	0.025778	-0.024236	-0.002597	-0.004838	0.032996	0.011200
	-0.049682	-0.049665	-0.021246	-0.072193	0.018818	0.010698
	-0.032847	0.031404	0.027008	-0.055999	-0.018236	0.008328
	-0.013058	-0.017441	-0.022681	-0.039854	-0.067386	-0.020128
	0.008535					
x258	-0.025728	-0.051682	-0.030641	-0.000740	-0.001225	0.000818
	0.000962	0.006644	-0.007177	0.011225	0.020969	-0.034731
	0.022569	0.002595	0.006160	0.027797	-0.032850	0.047377
	0.014178	-0.015648	-0.031566	0.007633	0.005048	-0.022042
	-0.022074	0.026167	-0.059098	0.007876	0.014231	0.002588
	0.010000					
y258	-0.012263	-0.053491	0.010667	-0.035575	0.021858	0.008864
	-0.037220	-0.015417	-0.082075	-0.005870	-0.045641	-0.045939
	0.000327	0.024466	0.022503	0.017440	-0.008967	0.053103
	0.093953	0.003348	-0.111530	-0.033694	-0.009209	-0.000590
	-0.021675	-0.046642	-0.025508	-0.005048	0.085550	0.045265
	-0.064429					

z258	-0.003210	-0.004782	0.053252	0.020249	-0.015120	-0.017813
	0.024611	-0.022752	-0.009175	-0.010297	0.035504	0.007152
	-0.044448	-0.044110	-0.019686	-0.083078	0.023269	0.017501
	-0.025699	0.030909	0.023198	-0.054828	-0.019009	-0.005068
	-0.009819	-0.015327	-0.015463	-0.018723	-0.060582	-0.010765
	0.008377					
x259	-0.027370	-0.052927	-0.029048	-0.002965	0.000271	0.005709
	-0.003837	0.001943	-0.009699	0.019240	0.013180	-0.038403
	0.023092	0.003775	0.014818	0.032804	-0.037151	0.049407
	0.026499	-0.007282	-0.036768	0.004606	0.013807	-0.012352
	-0.015859	0.013622	-0.062727	0.001712	0.022938	0.011788
	0.015661					
y259	-0.009276	-0.045728	0.010571	-0.031017	0.021704	0.003872
	-0.030095	-0.013630	-0.068756	-0.014564	-0.039580	-0.034307
	-0.001726	0.017661	0.014831	0.012133	-0.008490	0.044455
	0.078050	-0.002374	-0.094083	-0.034221	-0.010070	-0.001883
	-0.019643	-0.030993	-0.015459	-0.006354	0.069311	0.040906
	-0.058107					
z259	-0.003348	-0.001455	0.052682	0.020227	-0.014575	-0.020728
	0.024883	-0.019840	-0.013763	-0.016737	0.038213	0.001700
	-0.041630	-0.039911	-0.014175	-0.092353	0.027790	0.021147
	-0.019176	0.030485	0.019794	-0.054676	-0.019477	-0.016753
	-0.003590	-0.014558	-0.009024	-0.005236	-0.057918	-0.006654
	0.006242					
x260	-0.029322	-0.051286	-0.027901	-0.005054	0.001277	0.010879
	-0.007732	-0.000230	-0.010868	0.025221	0.006141	-0.034756
	0.022833	0.007396	0.021347	0.034256	-0.042302	0.044660
	0.039540	-0.004794	-0.034809	0.002052	0.025072	-0.002833
	-0.014649	0.003752	-0.064857	-0.002836	0.031969	0.016330
	0.013486					
y260	-0.007590	-0.039221	0.009941	-0.026412	0.022557	-0.000865
	-0.022871	-0.012144	-0.058115	-0.022713	-0.033926	-0.023851
	-0.001836	0.009793	0.006210	0.009451	-0.007339	0.036729
	0.062985	-0.008919	-0.079134	-0.033847	-0.012298	-0.003060
	-0.016208	-0.014820	-0.008324	-0.007503	0.050346	0.035615
	-0.049181					
z260	-0.002847	0.002943	0.052773	0.020079	-0.014518	-0.024814
	0.025707	-0.016701	-0.017773	-0.023194	0.041124	-0.004129
	-0.040476	-0.035396	-0.009923	-0.099301	0.033121	0.021708
	-0.013501	0.032138	0.016047	-0.055932	-0.021279	-0.025811
	0.005118	-0.014874	-0.001040	0.005796	-0.056116	-0.005357
	0.003116					

x261	-0.030878	-0.049574	-0.027335	-0.005603	0.001106	0.015106
	-0.008310	-0.001226	-0.010499	0.026726	-0.000744	-0.027117
	0.023279	0.010068	0.024893	0.032742	-0.046646	0.040264
	0.048624	-0.003108	-0.032151	-0.001078	0.037967	0.004469
	-0.016382	0.000519	-0.066604	-0.006474	0.035234	0.012641
	0.009593					
y261	-0.007686	-0.033517	0.008719	-0.021815	0.024529	-0.004499
	-0.018081	-0.009263	-0.049022	-0.027048	-0.029949	-0.014667
	-0.000305	0.004846	-0.000403	0.007770	-0.009789	0.026649
	0.051911	-0.019343	-0.063158	-0.032718	-0.015541	-0.005260
	-0.018065	0.000651	-0.007346	-0.009959	0.034499	0.036235
	-0.040447					
z261	-0.002673	0.007678	0.053001	0.019999	-0.013547	-0.028570
	0.025919	-0.012493	-0.022727	-0.026923	0.041075	-0.009927
	-0.038816	-0.028805	-0.007687	-0.102727	0.035037	0.018836
	-0.007555	0.034837	0.012002	-0.057369	-0.021095	-0.035923
	0.010767	-0.013132	0.006521	0.014123	-0.048986	-0.001680
	0.004511					
x262	-0.029947	-0.047024	-0.026156	-0.006514	-0.000753	0.017170
	-0.006752	-0.006348	-0.008694	0.025241	-0.001121	-0.022802
	0.017608	0.007808	0.022192	0.027826	-0.041084	0.039723
	0.046273	0.001736	-0.029509	-0.001858	0.040322	0.010866
	-0.005631	0.000581	-0.059455	-0.007716	0.029113	0.006935
	0.002743					
y262	-0.008985	-0.028960	0.007203	-0.016900	0.026633	-0.007823
	-0.013876	-0.004174	-0.042053	-0.031748	-0.029496	-0.004619
	0.004333	0.003732	-0.005400	0.009735	-0.016681	0.014942
	0.046349	-0.028330	-0.049049	-0.034179	-0.016802	-0.005689
	-0.027606	0.008257	-0.009988	-0.011652	0.021603	0.034002
	-0.030464					
z262	-0.003492	0.011512	0.052697	0.019875	-0.011460	-0.031458
	0.024960	-0.007294	-0.026941	-0.027054	0.038822	-0.015281
	-0.037087	-0.020883	-0.004085	-0.103842	0.033427	0.012456
	-0.001266	0.034003	0.007824	-0.057891	-0.015162	-0.045279
	0.012682	-0.005973	0.009697	0.021248	-0.038718	0.006883
	0.006295					
x263	-0.029520	-0.044248	-0.026552	-0.006708	-0.001379	0.019223
	-0.004772	-0.009724	-0.008646	0.022508	0.000494	-0.016281
	0.015288	0.006037	0.018692	0.023803	-0.033502	0.036165
	0.045120	0.010291	-0.024877	-0.002197	0.039017	0.017787
	-0.000547	0.003950	-0.046733	-0.010231	0.018986	0.010537
	0.000600					

y263	-0.009537	-0.024867	0.007216	-0.012663	0.027090	-0.013941
	-0.009336	-0.001484	-0.035058	-0.035615	-0.030994	0.003127
	0.001590	0.001475	-0.013552	0.006045	-0.027974	0.006142
	0.035887	-0.042013	-0.038445	-0.037253	-0.014452	-0.007628
	-0.032190	0.012018	-0.018581	-0.011914	0.005706	0.022835
	-0.027013					
z263	-0.004968	0.015001	0.052574	0.019530	-0.009964	-0.032601
	0.023600	-0.001716	-0.030683	-0.024610	0.034284	-0.018896
	-0.035732	-0.011538	-0.000825	-0.100093	0.031471	0.003215
	0.005737	0.031735	0.002022	-0.056716	-0.007161	-0.052545
	0.012140	0.001702	0.009807	0.027532	-0.026995	0.013187
	0.002037					
x264	-0.030048	-0.041299	-0.027917	-0.006655	-0.001008	0.020849
	-0.003693	-0.012154	-0.009944	0.018456	-0.000825	-0.009861
	0.013688	0.006919	0.014754	0.016672	-0.031969	0.033804
	0.043611	0.020609	-0.023243	-0.002863	0.037169	0.020148
	0.001517	0.011601	-0.037657	-0.013062	0.008493	0.016360
	0.002351					
y264	-0.008360	-0.021762	0.008006	-0.007345	0.026561	-0.020674
	-0.004763	0.000314	-0.027949	-0.040352	-0.026798	0.011099
	-0.001546	-0.004121	-0.024903	0.004862	-0.030893	-0.004378
	0.028505	-0.058470	-0.022487	-0.037388	-0.013767	-0.004516
	-0.033128	0.009647	-0.022880	-0.009543	-0.008546	0.012112
	-0.034240					
z264	-0.006788	0.018494	0.052600	0.018866	-0.009328	-0.033273
	0.022537	0.002746	-0.033228	-0.020320	0.028757	-0.022285
	-0.035586	-0.003347	0.001072	-0.091615	0.031441	-0.006858
	0.008042	0.029297	-0.005362	-0.054178	0.000361	-0.055152
	0.014988	0.009482	0.008764	0.032938	-0.014195	0.014670
	-0.000450					
x265	-0.031141	-0.037532	-0.029289	-0.005355	-0.002100	0.020619
	-0.003308	-0.013909	-0.010843	0.011211	-0.003963	-0.005091
	0.011774	0.006937	0.008309	0.003953	-0.032069	0.030776
	0.042250	0.028102	-0.021929	-0.004006	0.033085	0.020538
	0.003810	0.021264	-0.033014	-0.012611	0.000103	0.016977
	0.000901					
y265	-0.007721	-0.020137	0.008377	-0.001914	0.026567	-0.026802
	0.001694	0.000152	-0.022768	-0.040768	-0.019610	0.020502
	-0.003195	-0.017849	-0.031600	0.016106	-0.023312	-0.012598
	0.017045	-0.068252	-0.011032	-0.026230	-0.012411	0.003060
	-0.032141	0.002417	-0.018446	-0.015909	-0.027976	-0.003946
	-0.025382					

z265	-0.008358	0.021486	0.052402	0.018193	-0.008665	-0.033809
	0.022034	0.007005	-0.033005	-0.015592	0.023524	-0.023666
	-0.037227	0.006396	0.001131	-0.080755	0.029808	-0.018540
	0.006343	0.025334	-0.010362	-0.052918	0.009477	-0.051431
	0.017608	0.018234	0.011841	0.035501	-0.007313	0.024084
	0.001757					
x266	-0.031007	-0.033532	-0.030903	-0.002916	-0.001855	0.019059
	-0.002268	-0.015759	-0.010301	0.001604	-0.004850	0.001686
	0.006032	0.007037	0.000171	-0.012522	-0.034879	0.029866
	0.036108	0.034477	-0.015543	-0.005728	0.027724	0.024206
	0.008681	0.033937	-0.020710	-0.015619	-0.017787	0.023880
	-0.005362					
y266	-0.004848	-0.019981	0.011108	0.000729	0.024708	-0.030653
	0.007201	0.002778	-0.021103	-0.032906	-0.013040	0.022434
	-0.009629	-0.025047	-0.029568	0.018799	-0.008485	-0.019709
	0.003826	-0.072384	-0.004935	-0.019726	-0.014312	0.007563
	-0.029593	0.006460	-0.009721	-0.021997	-0.036152	-0.015099
	-0.017399					
z266	-0.011882	0.023270	0.051953	0.017212	-0.011642	-0.033964
	0.022526	0.009265	-0.034477	-0.006956	0.018880	-0.022918
	-0.030821	0.007979	0.001467	-0.060791	0.038345	-0.034125
	0.012539	0.009266	-0.013623	-0.044238	0.015301	-0.049239
	0.009285	0.013044	0.007974	0.039049	0.012170	0.004173
	0.008158					
x267	-0.031389	-0.029291	-0.032107	0.000783	-0.001860	0.016771
	-0.001356	-0.016786	-0.012396	-0.009541	-0.008300	0.009669
	0.000271	0.013264	-0.008755	-0.027966	-0.039721	0.031041
	0.033643	0.037130	-0.012711	-0.009359	0.020481	0.027462
	0.006367	0.040287	-0.010121	-0.016396	-0.026067	0.026433
	-0.007199					
y267	-0.000685	-0.020846	0.009814	0.004210	0.024889	-0.032219
	0.012141	0.006487	-0.019377	-0.029681	-0.012761	0.020765
	-0.010677	-0.030010	-0.035615	0.022239	0.000396	-0.022453
	0.006489	-0.059491	-0.013142	-0.012374	-0.022614	0.003817
	-0.032934	0.005439	-0.001767	-0.014308	-0.020745	-0.023754
	0.003630					
z267	-0.015047	0.025679	0.047582	0.015580	-0.013227	-0.028980
	0.021814	0.015829	-0.039808	-0.012540	0.013178	-0.017362
	-0.015997	0.003799	-0.008726	-0.038019	0.040927	-0.033389
	0.021860	0.004757	-0.004864	-0.050640	0.005760	-0.042548
	-0.026031	0.006134	0.014326	0.040228	0.033871	-0.027041
	0.014147					

x268	-0.031367	-0.027482	-0.033387	0.004409	-0.002386	0.013728
	-0.002876	-0.017782	-0.017632	-0.016733	-0.010174	0.009568
	-0.001724	0.015557	-0.014347	-0.037179	-0.041707	0.033635
	0.030693	0.035549	-0.012370	-0.010440	0.014752	0.026639
	0.004060	0.041802	-0.006147	-0.014293	-0.025357	0.019967
	-0.010629					
y268	0.000754	-0.023349	0.006698	0.005928	0.023769	-0.035612
	0.009002	0.005242	-0.021311	-0.028254	-0.014693	0.011726
	-0.005123	-0.033423	-0.040887	0.026638	0.006510	-0.018259
	0.007634	-0.046277	-0.017420	-0.011984	-0.028238	0.000928
	-0.035231	-0.001376	0.010126	-0.013865	-0.007122	-0.030255
	0.009417					
z268	-0.017395	0.028912	0.045153	0.014143	-0.012157	-0.025280
	0.020994	0.025487	-0.045139	-0.018748	0.006491	-0.010740
	-0.008919	0.005424	-0.017110	-0.014852	0.036805	-0.028567
	0.023484	0.005656	-0.002112	-0.059609	-0.004722	-0.030414
	-0.053363	0.002280	0.022817	0.038980	0.045304	-0.042041
	0.023098					
x269	-0.031245	-0.026613	-0.033845	0.007802	-0.003907	0.012047
	-0.003334	-0.016148	-0.023326	-0.021713	-0.012035	0.007331
	-0.000216	0.017241	-0.020138	-0.038814	-0.042061	0.040895
	0.028677	0.034714	-0.010830	-0.013010	0.006360	0.028933
	-0.003888	0.031656	-0.005082	-0.012157	-0.015764	0.004024
	-0.004980					
y269	0.000425	-0.024912	0.003321	0.004909	0.021271	-0.040911
	0.003276	0.005491	-0.025080	-0.020508	-0.012485	-0.004577
	-0.008980	-0.039153	-0.031681	0.023163	0.007518	-0.020481
	-0.000958	-0.048493	-0.018536	-0.005190	-0.022672	0.001099
	-0.021648	0.011721	0.006983	-0.014624	-0.013623	-0.021269
	-0.008119					
z269	-0.019343	0.031160	0.044518	0.012892	-0.012398	-0.020903
	0.019766	0.035006	-0.048690	-0.024518	0.000296	-0.003065
	-0.004593	0.010165	-0.020865	0.003301	0.030079	-0.024229
	0.024186	0.007329	0.002862	-0.068247	-0.017239	-0.016035
	-0.075684	-0.006872	0.032004	0.033737	0.053557	-0.053103
	0.031666					
x270	-0.030379	-0.025948	-0.034102	0.010283	-0.003775	0.007427
	-0.005910	-0.015575	-0.030173	-0.020606	-0.010629	0.001082
	-0.001136	0.016610	-0.018378	-0.045599	-0.040810	0.043503
	0.023243	0.024605	-0.013244	-0.008075	0.003306	0.027249
	-0.001820	0.032648	-0.005098	-0.007731	-0.010321	-0.002386
	-0.014136					

y270	0.001560	-0.023402	0.000724	0.004606	0.018776	-0.041934
	0.000340	0.008431	-0.027403	-0.014179	-0.009726	-0.010745
	-0.009141	-0.036907	-0.032095	0.019044	0.005802	-0.017208
	-0.002020	-0.047990	-0.015202	-0.002509	-0.028999	0.001823
	-0.018128	0.008590	0.008611	-0.013756	-0.013335	-0.020725
	-0.017098					
z270	-0.021035	0.033449	0.043948	0.011292	-0.011152	-0.019028
	0.018740	0.043928	-0.051402	-0.025959	-0.002927	0.002328
	-0.005448	0.014100	-0.019581	0.013199	0.029040	-0.023438
	0.020790	0.001609	0.002686	-0.068903	-0.025276	-0.012262
	-0.082047	-0.005194	0.038931	0.030160	0.057400	-0.054125
	0.030304					
x271	-0.030109	-0.026080	-0.033353	0.012796	-0.004782	0.006089
	-0.005357	-0.013967	-0.032829	-0.020159	-0.011218	0.000058
	0.002229	0.017943	-0.022340	-0.045789	-0.040995	0.053298
	0.023699	0.022913	-0.012731	-0.009142	-0.005334	0.028140
	-0.010577	0.020526	-0.005869	-0.002855	0.004446	-0.014035
	-0.008042					
y271	0.002346	-0.022560	-0.001581	0.004109	0.015999	-0.040256
	0.000019	0.010158	-0.022497	-0.010590	-0.008578	-0.011157
	-0.006733	-0.030688	-0.036844	0.019926	0.003658	-0.009749
	0.002593	-0.035602	-0.009699	-0.003365	-0.040557	0.006737
	-0.022085	-0.000144	0.009955	-0.009161	-0.005547	-0.018954
	-0.012628					
z271	-0.022266	0.034297	0.042674	0.009820	-0.009153	-0.019013
	0.015398	0.048316	-0.050773	-0.025170	-0.003667	0.006194
	-0.011482	0.015635	-0.009732	0.014809	0.032951	-0.030775
	0.015647	-0.003370	-0.004684	-0.059418	-0.029204	-0.013616
	-0.067501	0.008170	0.041892	0.028412	0.052522	-0.044563
	0.019499					
x272	-0.030121	-0.025662	-0.032863	0.014698	-0.005619	0.005788
	-0.004372	-0.014846	-0.033514	-0.019173	-0.010702	0.001156
	0.006146	0.019082	-0.023477	-0.047066	-0.039317	0.063720
	0.026569	0.026558	-0.015642	-0.011592	-0.011991	0.030274
	-0.023819	0.008962	-0.009606	-0.005869	0.016993	-0.027782
	-0.004428					
y272	0.002863	-0.021342	-0.003822	0.002189	0.014718	-0.039394
	-0.001101	0.007774	-0.016776	-0.006150	-0.005585	-0.011347
	-0.006811	-0.026653	-0.035830	0.017922	0.004291	-0.004998
	0.004561	-0.022419	-0.010544	-0.003433	-0.041643	0.011038
	-0.027948	0.000312	0.009109	-0.010186	-0.006501	-0.018089
	-0.015118					

z272	-0.023662	0.034589	0.041828	0.008787	-0.005257	-0.019751
	0.012931	0.049272	-0.047643	-0.022917	-0.004146	0.010668
	-0.020618	0.012681	-0.000747	0.012716	0.040401	-0.037754
	0.008456	-0.009947	-0.018418	-0.051693	-0.022401	-0.019405
	-0.044855	0.025352	0.042454	0.029250	0.039139	-0.036346
	0.007139					
x273	-0.030364	-0.024391	-0.031612	0.015916	-0.006323	0.005275
	-0.001179	-0.012619	-0.033887	-0.019399	-0.008596	0.002824
	0.009481	0.015847	-0.024151	-0.043751	-0.042722	0.070176
	0.029972	0.028599	-0.013637	-0.013148	-0.021181	0.033715
	-0.033468	-0.000582	-0.014605	-0.001831	0.033101	-0.036183
	0.002603					
y273	0.003497	-0.020044	-0.005628	-0.000226	0.014707	-0.040089
	-0.002510	0.006822	-0.010452	-0.001117	-0.000085	-0.014249
	-0.008525	-0.026547	-0.030005	0.017452	0.003745	-0.004916
	0.004520	-0.013480	-0.011537	0.001262	-0.040919	0.014264
	-0.024009	0.010855	0.005551	-0.007255	-0.007936	-0.006127
	-0.022743					
z273	-0.025053	0.034036	0.042018	0.008048	-0.000688	-0.019917
	0.010904	0.048640	-0.041558	-0.019449	-0.002058	0.010633
	-0.028970	0.007425	0.010068	0.012849	0.047613	-0.042459
	0.000885	-0.017039	-0.032129	-0.045130	-0.013798	-0.031645
	-0.014467	0.040527	0.037033	0.033192	0.029304	-0.020555
	-0.001468					
x274	-0.030867	-0.024768	-0.031271	0.016889	-0.005739	0.000871
	-0.001239	-0.011505	-0.034450	-0.016542	-0.006827	0.000274
	0.014426	0.013394	-0.023697	-0.048157	-0.047475	0.069667
	0.028685	0.021272	-0.016539	-0.011389	-0.022386	0.035924
	-0.032914	-0.008333	-0.020907	0.001638	0.048286	-0.037518
	0.009041					
y274	0.004269	-0.017994	-0.007218	-0.002843	0.015214	-0.039548
	-0.003006	0.008488	-0.002427	0.002575	0.003868	-0.012432
	-0.007075	-0.021913	-0.030366	0.019446	0.002409	-0.003911
	0.005316	-0.003832	-0.010296	0.003492	-0.042037	0.023956
	-0.026820	0.012419	0.005624	-0.006294	-0.003215	-0.001899
	-0.018874					
z274	-0.026714	0.029655	0.045073	0.007964	0.001537	-0.017739
	0.006733	0.046330	-0.033552	-0.017036	-0.004420	0.008988
	-0.036251	0.004843	0.019581	0.014610	0.050098	-0.046787
	-0.008562	-0.017931	-0.045453	-0.045084	-0.004359	-0.047066
	0.011599	0.037595	0.031934	0.033764	0.028186	-0.015357
	0.008845					

x275	-0.031896	-0.024559	-0.031342	0.017845	-0.004174	-0.001826
	-0.003734	-0.010585	-0.035009	-0.011731	-0.004696	-0.002481
	0.017966	0.009271	-0.019850	-0.052241	-0.048296	0.069304
	0.029435	0.005466	-0.015347	-0.006356	-0.014911	0.034442
	-0.029461	-0.003561	-0.027030	0.001318	0.046715	-0.039254
	0.003676					
y275	0.004461	-0.016419	-0.008113	-0.006118	0.015753	-0.039323
	-0.005071	0.011822	0.003334	0.004810	0.006867	-0.010508
	-0.006226	-0.017183	-0.028189	0.021808	0.001245	-0.004343
	0.005491	0.001176	-0.010785	0.009126	-0.036681	0.033476
	-0.030538	0.021952	0.002750	-0.007770	0.000189	-0.001771
	-0.015586					
z275	-0.027996	0.025055	0.048227	0.009380	0.005000	-0.010286
	0.005037	0.041759	-0.022084	-0.015503	-0.005047	0.008960
	-0.042171	0.001838	0.023269	0.024099	0.055653	-0.047979
	-0.016458	-0.011102	-0.057788	-0.039051	0.000868	-0.062699
	0.046484	0.019371	0.025895	0.043805	0.028533	-0.008171
	0.024654					
x276	-0.033156	-0.025774	-0.031351	0.019707	-0.002265	-0.003792
	-0.005263	-0.006226	-0.036352	-0.012778	-0.005864	-0.002477
	0.019829	0.004926	-0.019198	-0.054825	-0.046767	0.066508
	0.026929	-0.001755	-0.012580	-0.002514	-0.014914	0.027612
	-0.027682	-0.012035	-0.018378	0.005607	0.040385	-0.036772
	-0.005843					
y276	0.004508	-0.016280	-0.006708	-0.010205	0.014497	-0.036381
	-0.008942	0.017298	0.009732	0.006211	0.007367	-0.014090
	-0.005661	-0.009788	-0.022322	0.025914	-0.000178	-0.003705
	0.003947	0.005841	-0.009604	0.012716	-0.028741	0.031632
	-0.030988	0.023780	0.001451	-0.014479	0.007196	-0.005037
	-0.004556					
z276	-0.028448	0.022501	0.049272	0.013900	0.011036	-0.006147
	0.004430	0.033232	-0.016216	-0.015289	-0.001510	0.014143
	-0.042126	-0.009991	0.024165	0.028068	0.064175	-0.047722
	-0.022421	0.000543	-0.069452	-0.034131	0.000361	-0.057353
	0.067744	0.017637	0.021558	0.053896	0.015759	0.005037
	0.024133					
x277	-0.034416	-0.025638	-0.031614	0.022714	0.000811	-0.005064
	-0.003741	-0.005952	-0.035917	-0.011923	-0.009261	-0.006606
	0.017372	-0.001462	-0.014814	-0.051150	-0.040333	0.063109
	0.023145	-0.000596	-0.015293	-0.004231	-0.006159	0.025436
	-0.022473	-0.007082	-0.011763	-0.006781	0.045228	-0.044652
	0.000761					

y277 0.005468 -0.016405 -0.005665 -0.013290 0.013322 -0.031698
-0.013145 0.021765 0.016680 0.008159 0.008263 -0.018011
-0.006275 -0.002892 -0.017206 0.027212 -0.001705 -0.002120
0.002336 0.011845 -0.006431 0.015307 -0.023267 0.027646
-0.027849 0.021090 -0.001019 -0.015684 0.009657 0.000408
0.000510

z277 -0.028327 0.020574 0.051256 0.016550 0.016071 -0.004194
0.005780 0.028030 -0.013038 -0.018415 0.001299 0.021396
-0.043419 -0.018231 0.027580 0.029669 0.069087 -0.048704
-0.025433 0.004192 -0.075599 -0.037664 -0.000244 -0.050572
0.072488 0.028163 0.023319 0.049505 0.007930 0.002451
0.031802

x278 -0.035158 -0.024542 -0.031055 0.023752 0.002598 -0.004618
-0.001210 -0.003591 -0.033076 -0.011830 -0.010452 -0.003486
0.018226 -0.005568 -0.012463 -0.052930 -0.033520 0.061501
0.019914 -0.004428 -0.015895 0.000702 -0.003121 0.024799
-0.019336 0.002371 -0.004751 -0.012071 0.056469 -0.050147
-0.002845

y278 0.006964 -0.017068 -0.005455 -0.014855 0.013500 -0.027919
-0.016731 0.024266 0.020787 0.009018 0.007884 -0.021763
-0.009155 0.001943 -0.012295 0.028751 -0.003175 -0.003890
0.000358 0.016582 -0.004535 0.013506 -0.016510 0.023323
-0.024673 0.016727 -0.004418 -0.017375 0.001050 0.006690
0.010123

z278 -0.028626 0.017853 0.053345 0.017636 0.018686 -0.003749
0.006834 0.024735 -0.009801 -0.019891 0.001907 0.023575
-0.044230 -0.022182 0.030146 0.029750 0.068837 -0.043733
-0.028369 0.012228 -0.075698 -0.039032 -0.005800 -0.043026
0.071309 0.034613 0.026353 0.044023 0.011153 -0.002330
0.025818

x279 -0.035139 -0.024658 -0.030707 0.024361 0.002354 -0.007280
0.000563 -0.004219 -0.029018 -0.007816 -0.008198 -0.004228
0.019835 -0.006195 -0.011966 -0.051465 -0.033756 0.060794
0.020646 -0.006545 -0.010543 0.003512 -0.010801 0.017240
-0.021486 0.000601 -0.002093 -0.011631 0.046291 -0.040897
-0.007045

y279 0.008305 -0.017911 -0.005570 -0.016240 0.016122 -0.021576
-0.019405 0.026828 0.024022 0.007288 0.003877 -0.024290
-0.013625 0.006630 -0.006783 0.029301 -0.000083 -0.005033
-0.005812 0.020084 -0.004610 0.013636 -0.001248 0.021246
-0.018601 0.018269 -0.004603 -0.025848 0.009015 -0.000485
0.018534

z279	-0.028837	0.014695	0.054188	0.018063	0.019460	-0.004878
	0.006813	0.022846	-0.008115	-0.020133	0.003259	0.025829
	-0.044740	-0.025553	0.031372	0.024943	0.068660	-0.033081
	-0.023716	0.022156	-0.072427	-0.039617	-0.015856	-0.036742
	0.071878	0.042681	0.037954	0.040283	0.009554	-0.000561
	0.017870					
x280	-0.034838	-0.025970	-0.030761	0.023162	0.003718	-0.009002
	-0.000078	-0.002995	-0.025122	-0.005923	-0.006095	-0.005667
	0.019073	-0.008196	-0.012400	-0.048079	-0.033950	0.057912
	0.022373	-0.012669	-0.009975	-0.000111	-0.013263	0.015841
	-0.019540	0.005468	-0.009711	-0.005783	0.040663	-0.035158
	-0.015714					
y280	0.009744	-0.017170	-0.006137	-0.014836	0.016679	-0.017173
	-0.018807	0.027645	0.026853	0.007526	0.000028	-0.027289
	-0.017014	0.011947	0.001962	0.029448	0.006252	-0.004203
	-0.009808	0.029213	-0.003424	0.017691	0.006723	0.010862
	-0.016203	0.014994	0.006526	-0.043587	0.012455	-0.002022
	0.031195					
z280	-0.028068	0.011166	0.054050	0.017314	0.017919	-0.006067
	0.007542	0.017985	-0.006970	-0.018173	0.005934	0.029905
	-0.045921	-0.027089	0.028003	0.021158	0.061624	-0.023804
	-0.024442	0.024959	-0.074327	-0.039890	-0.017162	-0.026855
	0.069815	0.049815	0.036326	0.033153	0.008141	-0.001187
	0.018800					
x281	-0.034452	-0.026922	-0.031930	0.022245	0.006263	-0.009980
	-0.001238	-0.000879	-0.019982	-0.002303	-0.003184	-0.006505
	0.015774	-0.008872	-0.009311	-0.042095	-0.030209	0.054493
	0.020776	-0.017495	-0.008036	-0.001287	-0.012867	0.011814
	-0.018379	0.012105	-0.011443	-0.003078	0.032938	-0.029168
	-0.018974					
y281	0.011471	-0.018712	-0.005430	-0.014923	0.016039	-0.014165
	-0.016474	0.025286	0.027393	0.005715	-0.005100	-0.028938
	-0.014631	0.015727	0.002398	0.022445	0.006467	-0.003541
	-0.009602	0.035104	-0.002202	0.021898	0.007047	0.007991
	-0.008693	0.010466	0.003419	-0.052092	0.015057	0.001072
	0.036389					
z281	-0.027009	0.006897	0.052493	0.015818	0.015402	-0.008120
	0.007132	0.014038	-0.007043	-0.014118	0.008444	0.033743
	-0.046527	-0.029863	0.025181	0.016365	0.054675	-0.011944
	-0.023570	0.027754	-0.076812	-0.039596	-0.019851	-0.016244
	0.064648	0.053587	0.038774	0.027218	0.005456	0.000678
	0.016197					

x282	-0.034249	-0.028741	-0.032957	0.020787	0.009113	-0.010151
	-0.001124	0.001176	-0.012386	-0.000186	-0.001104	-0.007230
	0.016032	-0.006999	-0.006022	-0.036154	-0.025907	0.050967
	0.018661	-0.019281	-0.004630	0.000204	-0.013840	0.009087
	-0.012482	0.016353	-0.014034	0.000246	0.032298	-0.025469
	-0.012672					
y282	0.014340	-0.020263	-0.006275	-0.013578	0.015684	-0.013347
	-0.016250	0.022848	0.023041	0.004215	-0.007869	-0.031951
	-0.020846	0.018958	0.000226	0.017034	0.007072	-0.002881
	-0.007704	0.039119	-0.009595	0.016851	0.010116	-0.000023
	-0.009160	0.013001	-0.001559	-0.062266	-0.001454	0.011900
	0.026232					
z282	-0.025875	0.001785	0.049985	0.013371	0.014077	-0.009250
	0.007779	0.009427	-0.006825	-0.008465	0.008695	0.036278
	-0.046206	-0.027574	0.020522	0.009437	0.046091	0.000649
	-0.025565	0.028094	-0.074644	-0.033912	-0.024112	-0.002810
	0.063654	0.048768	0.037818	0.015409	0.007761	-0.006522
	0.013753					
x283	-0.033504	-0.030922	-0.034586	0.018741	0.011000	-0.011353
	-0.001374	0.001779	-0.003511	0.002962	0.000139	-0.008481
	0.015763	-0.003806	-0.003212	-0.029837	-0.021394	0.048989
	0.017148	-0.019809	-0.003004	0.003370	-0.014355	0.006660
	-0.009393	0.023497	-0.015912	0.003938	0.031038	-0.019974
	-0.003499					
y283	0.016959	-0.023119	-0.006626	-0.011214	0.018248	-0.010594
	-0.015073	0.022332	0.016598	-0.000004	-0.007103	-0.035154
	-0.023508	0.018532	-0.002691	0.011627	0.011396	-0.004645
	-0.006023	0.045795	-0.014564	0.007833	0.010773	-0.004043
	-0.000290	0.009420	-0.010555	-0.070703	-0.010435	0.023018
	0.001941					
z283	-0.024654	-0.002781	0.046558	0.010771	0.011965	-0.009040
	0.007529	0.004822	-0.007157	-0.000052	0.007240	0.041042
	-0.045500	-0.025258	0.015532	0.002073	0.031916	0.013306
	-0.026891	0.021773	-0.070214	-0.029691	-0.025320	0.008820
	0.053131	0.039923	0.036565	0.000706	0.009634	-0.011124
	0.016589					
x284	-0.032278	-0.033717	-0.036163	0.017074	0.013882	-0.011374
	-0.000601	0.000885	0.005921	0.006701	0.000320	-0.010537
	0.016773	-0.001064	-0.000869	-0.022872	-0.015443	0.045195
	0.015808	-0.018234	-0.000634	0.010188	-0.017416	0.001633
	-0.006741	0.030708	-0.017015	0.008365	0.023505	-0.016028
	0.002922					

y284	0.019009	-0.027444	-0.006724	-0.008888	0.018706	-0.007998
	-0.017540	0.019333	0.011669	-0.000508	-0.005543	-0.029965
	-0.025170	0.014064	-0.006183	0.008359	0.020786	0.001784
	-0.003960	0.052230	-0.022662	-0.002658	0.013539	0.000381
	0.017583	0.005196	-0.023651	-0.075279	-0.008975	0.023806
	-0.000353					
z284	-0.023614	-0.008747	0.042524	0.008548	0.010922	-0.010630
	0.011385	-0.006744	-0.008966	0.002532	0.012227	0.039826
	-0.039456	-0.026906	0.010496	0.001880	0.022523	0.018420
	-0.022507	0.020995	-0.069711	-0.019615	-0.024048	0.009076
	0.042363	0.030400	0.035025	-0.015478	0.009803	-0.014193
	0.019033					
x285	-0.030211	-0.036675	-0.037451	0.015841	0.015771	-0.010870
	0.000500	0.000498	0.013507	0.010082	0.001899	-0.012962
	0.016853	0.001548	-0.001385	-0.017452	-0.010998	0.041825
	0.011215	-0.015463	0.002123	0.016065	-0.020611	-0.003301
	-0.004423	0.037479	-0.016587	0.011796	0.015134	-0.011806
	0.005478					
y285	0.017340	-0.034317	-0.003070	-0.010059	0.018891	-0.003378
	-0.023351	0.013139	0.005580	-0.000716	-0.010057	-0.032348
	-0.019124	0.016735	-0.000319	0.020304	0.030304	0.005407
	-0.004266	0.042386	-0.007536	-0.001204	0.010481	0.001946
	0.013601	0.006101	-0.022457	-0.072050	-0.003382	0.018315
	0.004404					
z285	-0.021221	-0.013401	0.039077	0.011616	0.010808	-0.013349
	0.017814	-0.019837	-0.001213	0.004140	0.004621	0.035987
	-0.030116	-0.024855	0.005612	0.010396	0.016960	0.008126
	-0.015907	0.020024	-0.063160	-0.010377	-0.014838	0.014290
	0.019111	0.021182	0.017100	-0.014679	0.014903	0.001172
	0.014295					
x286	-0.028790	-0.039738	-0.039346	0.013581	0.012419	-0.006454
	-0.000208	0.004053	0.014347	0.016203	0.010787	-0.013934
	0.013032	-0.000296	-0.009031	-0.022332	-0.012721	0.046150
	0.006644	-0.011765	0.003736	0.013107	-0.020311	-0.012767
	0.006627	0.035253	-0.017129	0.000100	0.014916	-0.005409
	0.002851					
y286	0.019370	-0.038543	-0.004176	-0.008596	0.031613	-0.001018
	-0.028847	-0.000342	0.016621	-0.001380	-0.027336	-0.022562
	-0.012619	0.030419	0.004625	0.033122	0.039573	0.000775
	0.006588	0.045389	-0.008584	0.008743	0.005678	-0.004787
	-0.002491	0.014746	-0.022484	-0.046944	-0.007984	0.021429
	-0.001977					

z286	-0.019667	-0.015384	0.034577	0.014626	0.008225	-0.014980
	0.020496	-0.023362	0.007247	0.013492	-0.007453	0.037643
	-0.027140	-0.022302	0.000917	0.009207	0.012383	0.003263
	-0.012163	0.019051	-0.054516	-0.012870	-0.008649	0.028743
	-0.001627	0.002895	-0.001702	-0.012246	0.014705	0.014206
	0.009796					
x287	-0.026884	-0.041089	-0.039534	0.013384	0.011761	-0.006260
	0.001548	0.003368	0.019345	0.018378	0.011786	-0.016439
	0.014325	-0.000660	-0.010647	-0.018627	-0.009837	0.046190
	0.002372	-0.005403	0.003921	0.015582	-0.021140	-0.014070
	0.006598	0.038577	-0.022523	0.000234	0.008474	-0.000161
	0.007208					
y287	0.018163	-0.038855	-0.001132	-0.010959	0.030101	0.002343
	-0.028804	-0.001572	0.011259	-0.003602	-0.030804	-0.018840
	-0.008747	0.026578	0.000909	0.028564	0.033326	0.003241
	0.007655	0.042354	-0.022406	0.003407	0.003039	0.001012
	-0.009800	0.022011	-0.044364	-0.035629	-0.001617	0.023518
	-0.005023					
z287	-0.016820	-0.016700	0.031417	0.016391	0.008825	-0.014084
	0.017817	-0.025232	0.013403	0.020191	-0.013450	0.038054
	-0.028162	-0.017807	-0.005740	0.007608	0.013332	0.001683
	-0.011874	0.011493	-0.034370	-0.010682	-0.000235	0.034153
	-0.014417	-0.005452	-0.004869	-0.013503	0.016041	0.014311
	0.007178					
x288	-0.027077	-0.042225	-0.037022	0.010062	0.004320	-0.001842
	0.002855	0.008174	0.012479	0.019848	0.023056	-0.022434
	0.013373	-0.006161	-0.015700	-0.026727	-0.016776	0.053439
	-0.003688	-0.005805	0.007008	0.012592	-0.020899	-0.021924
	0.011524	0.035776	-0.015939	-0.016282	0.006070	0.001747
	0.010776					
y288	0.015660	-0.039487	0.002416	-0.016381	0.027878	0.006311
	-0.028917	-0.002139	0.001489	-0.005956	-0.028505	-0.022344
	-0.007652	0.022333	-0.002409	0.019693	0.027798	0.010068
	0.011348	0.033698	-0.028050	0.001463	-0.001770	-0.000523
	-0.013904	0.015617	-0.036849	-0.028980	-0.000420	0.023017
	-0.005767					
z288	-0.014352	-0.017043	0.031403	0.018135	0.005757	-0.013421
	0.015597	-0.024064	0.012375	0.022709	-0.013711	0.034953
	-0.030996	-0.017828	-0.009095	0.002761	0.015931	-0.000099
	-0.016006	0.003759	-0.019556	-0.008771	0.006863	0.039468
	-0.017864	-0.009257	-0.009066	-0.016604	0.015182	0.006114
	0.000110					

x289	-0.025437	-0.042635	-0.035173	0.008181	0.000193	0.000842
	0.004039	0.013581	0.008795	0.017797	0.030641	-0.025085
	0.011437	-0.006739	-0.015946	-0.030557	-0.025551	0.055643
	-0.005142	-0.005632	0.009353	0.005964	-0.016234	-0.028781
	0.005925	0.037934	-0.013720	-0.026845	0.003684	0.006532
	0.015111					
y289	0.014131	-0.040365	0.005594	-0.022199	0.024330	0.008596
	-0.027485	-0.003684	-0.012361	-0.009168	-0.024118	-0.025738
	-0.011387	0.019371	-0.003578	0.010889	0.019022	0.014747
	0.016941	0.028984	-0.037666	-0.010801	-0.007206	-0.000201
	-0.017192	0.000926	-0.028343	-0.028033	0.002829	0.023119
	-0.016606					
z289	-0.010853	-0.016622	0.032823	0.018767	0.002182	-0.014785
	0.016366	-0.024828	0.012363	0.021046	-0.009081	0.029066
	-0.036085	-0.018114	-0.013652	-0.007217	0.024831	-0.003164
	-0.025295	0.000396	-0.011540	-0.010468	0.008319	0.041122
	-0.013361	-0.015117	-0.013157	-0.016207	0.002689	-0.002479
	-0.010478					
x290	-0.021621	-0.042031	-0.034083	0.008195	-0.000614	0.001296
	0.004095	0.016848	0.011038	0.013437	0.033320	-0.022127
	0.010846	-0.003937	-0.010507	-0.030007	-0.028409	0.053773
	-0.001492	-0.009925	0.010210	-0.000690	-0.011303	-0.031933
	-0.011894	0.049698	-0.017918	-0.029170	0.005299	-0.001970
	0.015076					
y290	0.010573	-0.042699	0.007930	-0.027073	0.021076	0.009647
	-0.027435	-0.006689	-0.029450	-0.010574	-0.024879	-0.029836
	-0.011945	0.020592	0.000077	0.003058	0.011962	0.016193
	0.029840	0.020391	-0.054118	-0.024201	-0.011961	-0.000910
	-0.022009	-0.015477	-0.028338	-0.027796	0.015654	0.018364
	-0.035080					
z290	-0.007687	-0.015008	0.035237	0.019709	-0.002233	-0.017334
	0.019657	-0.025604	0.013588	0.019431	-0.003252	0.022376
	-0.042978	-0.022037	-0.016460	-0.019716	0.031220	-0.007176
	-0.033572	0.001296	-0.002681	-0.016092	0.006976	0.040973
	-0.007005	-0.023515	-0.018040	-0.014307	-0.010029	-0.008816
	-0.010479					
x291	-0.019139	-0.041307	-0.032321	0.007821	0.001280	0.001537
	0.003910	0.018648	0.014278	0.002440	0.036566	-0.017383
	0.013177	-0.005456	-0.006221	-0.018347	-0.028750	0.053319
	-0.006912	-0.019501	0.004705	0.011383	-0.007307	-0.032988
	-0.014617	0.064236	-0.020552	-0.015524	0.003043	-0.005744
	0.025253					

y291	0.001425	-0.048956	0.009888	-0.033078	0.019298	0.012518
	-0.030425	-0.011931	-0.054828	-0.011869	-0.030692	-0.038717
	-0.006079	0.022473	0.007296	0.005108	0.005246	0.021161
	0.053050	0.011720	-0.080188	-0.028315	-0.014876	-0.007688
	-0.020579	-0.038778	-0.033085	-0.019077	0.039671	0.024433
	-0.052439					
z291	-0.005764	-0.011460	0.040091	0.021744	-0.008057	-0.018795
	0.020967	-0.024515	0.011218	0.016590	0.003453	0.018886
	-0.045587	-0.031011	-0.016476	-0.034940	0.029276	-0.003406
	-0.033257	0.008764	0.008708	-0.027798	0.000478	0.037409
	-0.013511	-0.024628	-0.021485	-0.021926	-0.026637	-0.011907
	-0.000423					
x292	-0.020980	-0.042504	-0.032331	0.008303	0.003561	0.002907
	0.006006	0.019893	0.010089	-0.007850	0.037180	-0.014119
	0.021766	-0.005127	-0.003892	0.005599	-0.032419	0.043287
	-0.001478	-0.035406	0.000154	0.018756	-0.003030	-0.042581
	-0.024040	0.071110	-0.040232	0.005618	-0.003365	-0.006948
	0.032909					
y292	-0.014357	-0.062286	0.010961	-0.042285	0.018170	0.018994
	-0.035372	-0.021103	-0.097115	-0.008800	-0.040296	-0.056800
	0.005669	0.027429	0.016976	0.017066	0.002904	0.032939
	0.095275	0.006865	-0.114478	-0.030421	-0.014787	-0.020611
	-0.018436	-0.074428	-0.048363	-0.014948	0.078312	0.035576
	-0.080413					
z292	-0.003001	-0.007989	0.048918	0.020406	-0.013769	-0.019816
	0.025417	-0.024520	0.003127	0.006200	0.020511	0.014535
	-0.049338	-0.041305	-0.018398	-0.057676	0.020281	0.002053
	-0.034165	0.020610	0.019879	-0.045241	-0.011416	0.027520
	-0.011034	-0.023709	-0.018672	-0.040327	-0.049364	-0.010861
	0.002583					
x293	0.011456	-0.013359	0.004003	0.040816	-0.074543	-0.012721
	0.014282	0.005732	0.048714	0.032534	0.004647	0.027903
	-0.019063	0.005803	0.013182	0.000498	-0.036599	0.003664
	0.018710	0.094798	0.023388	0.027282	-0.002171	-0.039322
	0.013753	0.031125	-0.064648	0.008816	-0.009434	0.016353
	-0.004732					
y293	0.019816	-0.005949	0.048774	-0.035693	0.037922	-0.004311
	-0.011175	-0.023585	-0.007294	0.002808	0.025733	-0.002415
	0.047306	-0.063346	0.043643	-0.031942	0.009184	0.013441
	-0.044019	0.013875	-0.002225	-0.037901	-0.026322	0.004249
	-0.022550	-0.010197	0.009115	0.000976	-0.018498	-0.062040
	-0.076718					

z293	-0.016401	-0.039250	-0.054316	-0.001476	0.022743	0.004662
	0.034695	-0.039766	0.059969	0.038626	0.005007	0.013181
	-0.000990	0.013555	0.000775	0.005254	-0.009398	-0.029364
	-0.025543	0.007595	-0.033828	0.033075	-0.017167	-0.050391
	-0.032407	0.003706	0.033787	0.025670	-0.011177	0.034535
	0.019878					
x294	0.009699	-0.008851	0.013395	0.039735	-0.078491	-0.020842
	0.012159	0.000283	0.036822	0.018054	0.002169	0.017779
	-0.015587	0.003017	0.024955	0.011029	-0.051922	-0.004354
	0.013731	0.085244	0.019263	0.026111	-0.008402	-0.035556
	0.002987	0.022557	-0.056469	-0.003828	-0.021272	0.010785
	-0.016469					
y294	0.016275	-0.011260	0.060698	-0.043889	0.047501	-0.006525
	-0.011926	-0.022789	-0.012905	-0.010024	0.028116	-0.012997
	0.048807	-0.045078	0.041789	-0.022773	0.015405	0.018912
	-0.025789	0.015453	-0.002424	-0.026778	-0.020471	0.013703
	-0.030283	-0.015550	-0.003925	0.010722	-0.008215	-0.036503
	-0.042091					
z294	-0.015162	-0.043101	-0.040325	-0.010069	0.024034	0.008321
	0.033435	-0.034629	0.063577	0.045386	0.013624	0.018961
	0.008327	-0.001729	0.008650	-0.007458	-0.016903	-0.017864
	-0.025978	-0.004326	-0.044476	0.013598	-0.016318	-0.041236
	-0.018412	0.013350	0.039001	0.018902	-0.013816	0.011542
	0.004718					
x295	0.008778	-0.006483	0.022600	0.041197	-0.078290	-0.029278
	0.009064	-0.003243	0.025676	0.003950	-0.006545	0.009171
	-0.012931	0.010998	0.033785	0.025929	-0.066897	-0.013125
	0.016216	0.075091	0.019760	0.030915	-0.008684	-0.044425
	-0.007624	0.018766	-0.048618	-0.009231	-0.020192	0.007404
	-0.024962					
y295	0.015132	-0.017167	0.068193	-0.047128	0.057768	-0.008191
	-0.008393	-0.026522	-0.016825	-0.026049	0.028089	-0.023602
	0.050464	-0.024259	0.040396	-0.014343	0.019518	0.019632
	-0.005059	0.020973	-0.001701	-0.016349	-0.021271	0.013623
	-0.041578	-0.024938	-0.013496	0.015775	-0.008871	-0.011431
	-0.006356					
z295	-0.014666	-0.049231	-0.010184	-0.019035	0.021666	0.005131
	0.025720	-0.019711	0.059811	0.046105	0.021359	0.016529
	0.009069	-0.008202	0.014706	-0.013928	-0.015325	-0.010449
	-0.025893	-0.018831	-0.038106	0.009251	-0.014722	-0.033426
	-0.013243	0.013870	0.047032	0.008107	0.005603	0.008935
	0.000347					

x296	0.006789	-0.005635	0.029400	0.040269	-0.074611	-0.033951
	0.006181	-0.005819	0.016945	-0.008167	-0.010752	0.001781
	-0.006075	0.012906	0.044065	0.040847	-0.072335	-0.018420
	0.023748	0.065551	0.016319	0.028536	-0.008825	-0.046996
	-0.008828	0.012814	-0.036933	-0.007692	-0.018854	0.000730
	-0.030051					
y296	0.017244	-0.020391	0.072791	-0.047682	0.061072	-0.009271
	-0.006175	-0.028517	-0.022013	-0.036195	0.028672	-0.029684
	0.049810	-0.007788	0.036641	-0.007702	0.014428	0.018679
	0.000451	0.018967	0.004410	-0.010271	-0.021813	0.002417
	-0.046119	-0.023641	-0.017343	0.014375	-0.010783	-0.000341
	0.014294					
z296	-0.016274	-0.053264	0.011730	-0.020729	0.021631	0.001227
	0.021801	-0.005816	0.053904	0.042659	0.019162	0.013547
	0.009439	-0.005726	0.018164	-0.017583	-0.009605	-0.010056
	-0.016829	-0.024333	-0.028860	0.007045	-0.015056	-0.020516
	-0.022977	0.012231	0.042114	-0.002619	0.015588	0.016102
	-0.001431					
x297	0.004816	-0.005580	0.034939	0.035621	-0.068074	-0.034817
	0.000414	-0.008138	0.012102	-0.017924	-0.008185	-0.003063
	0.002975	0.011488	0.054705	0.047721	-0.070694	-0.017418
	0.034891	0.053871	0.012290	0.018549	-0.011524	-0.045443
	-0.010229	0.011590	-0.019527	-0.010789	-0.020631	-0.007052
	-0.035277					
y297	0.017471	-0.023058	0.073901	-0.048736	0.061605	-0.010270
	-0.005037	-0.028620	-0.024774	-0.041689	0.030297	-0.034093
	0.049609	0.003031	0.036304	-0.002272	0.005029	0.014781
	0.000042	0.014140	0.013492	-0.009368	-0.022861	-0.006861
	-0.053414	-0.015939	-0.019862	0.000845	-0.016816	-0.003199
	0.019925					
z297	-0.016675	-0.053707	0.025891	-0.019379	0.019367	-0.003089
	0.020912	0.004081	0.047886	0.039825	0.013407	0.010688
	0.007738	-0.002801	0.016734	-0.018399	-0.005414	-0.013371
	-0.011559	-0.023671	-0.024761	0.008744	-0.013800	-0.008192
	-0.024150	0.005277	0.030783	-0.002947	0.021503	0.026677
	0.002313					
x298	0.002193	-0.005565	0.040318	0.035504	-0.065317	-0.039106
	-0.002860	-0.006543	0.006803	-0.019736	-0.006484	-0.007834
	0.007276	0.010943	0.058147	0.053693	-0.064498	-0.021552
	0.035883	0.045051	0.004703	0.015488	-0.015686	-0.050914
	-0.022031	0.006871	-0.011043	-0.016536	-0.023573	-0.004323
	-0.031915					

y298	0.016425	-0.026109	0.073259	-0.049719	0.059712	-0.011474
	-0.006285	-0.025672	-0.026106	-0.041214	0.028646	-0.038669
	0.039007	0.009174	0.028369	0.003219	-0.001826	0.006388
	-0.003241	0.007646	0.015133	-0.011940	-0.014294	-0.002821
	-0.061052	-0.019282	-0.025413	-0.010343	-0.009703	-0.001338
	0.019820					
z298	-0.016022	-0.054166	0.037975	-0.018832	0.017701	-0.005170
	0.019210	0.010451	0.040359	0.032142	0.010494	0.010028
	0.007639	-0.000289	0.016895	-0.017537	-0.003223	-0.014021
	-0.004984	-0.023248	-0.017725	0.010052	-0.013224	-0.002823
	-0.022627	0.005592	0.024923	-0.001666	0.025013	0.032239
	0.001651					
x299	0.001102	-0.005566	0.045170	0.041457	-0.068422	-0.046925
	-0.000083	-0.002380	0.000850	-0.012652	-0.005651	-0.011411
	0.008186	0.002552	0.056231	0.056626	-0.051615	-0.033327
	0.039298	0.034648	-0.009116	0.018704	-0.025108	-0.047246
	-0.021098	-0.009714	-0.005258	-0.015404	-0.042336	0.003975
	-0.018676					
y299	0.019614	-0.029331	0.067273	-0.042683	0.050384	-0.011358
	-0.004330	-0.020245	-0.029853	-0.034187	0.023219	-0.038419
	0.028149	0.005358	0.014418	0.001388	-0.004039	0.000632
	-0.005549	-0.001291	0.003482	-0.004511	-0.011753	-0.007543
	-0.056099	-0.023457	-0.013974	-0.005038	-0.006004	0.003702
	0.021220					
z299	-0.016790	-0.053324	0.049817	-0.019633	0.016084	-0.008504
	0.015795	0.017128	0.031115	0.021976	0.008844	0.006152
	0.010362	0.000344	0.020951	-0.012005	-0.001556	-0.018678
	0.003994	-0.023695	-0.010455	0.005696	-0.015728	0.010527
	-0.022323	0.011334	0.013336	-0.006357	0.025921	0.036458
	-0.002241					
x300	0.006600	-0.005139	0.041014	0.048431	-0.073781	-0.048718
	-0.000058	0.000334	0.000268	-0.004199	-0.003196	-0.008903
	0.014752	-0.009407	0.051093	0.050556	-0.024916	-0.045406
	0.051893	0.025329	-0.015414	0.020897	-0.027955	-0.034262
	-0.009763	-0.012891	-0.007207	-0.009442	-0.051515	0.008233
	-0.013158					
y300	0.028974	-0.030244	0.048212	-0.031558	0.034433	-0.006003
	-0.001781	-0.015047	-0.029753	-0.025002	0.014221	-0.034330
	0.028843	-0.004917	0.002770	-0.011369	0.005748	-0.002298
	-0.001781	-0.004890	-0.014388	0.005137	-0.012563	0.000295
	-0.040710	-0.020310	-0.008167	0.000867	-0.000295	0.005668
	0.017978					

z300	-0.015725	-0.050636	0.057346	-0.021968	0.012981	-0.009302
	0.009063	0.026036	0.021311	0.008083	0.008530	0.000628
	0.014092	-0.001970	0.031103	-0.001814	0.002296	-0.020718
	0.015538	-0.028335	0.006123	-0.001175	-0.016811	0.015203
	-0.019116	0.021428	0.009520	0.002100	0.024973	0.039099
	-0.003930					
x301	0.008944	-0.001777	0.027111	0.050104	-0.068618	-0.047779
	0.000856	-0.003312	0.008776	0.003828	-0.012116	-0.002010
	0.017316	-0.015672	0.042038	0.042736	-0.012338	-0.038802
	0.048741	0.019781	-0.017199	0.029326	-0.005861	-0.021847
	-0.010080	-0.010707	-0.017097	-0.011461	-0.052162	-0.000979
	0.000725					
y301	0.033496	-0.028661	0.029693	-0.028178	0.032953	0.000624
	0.004328	-0.015121	-0.023611	-0.018714	0.005712	-0.031096
	0.028343	-0.007634	-0.018061	-0.022087	0.014152	0.005363
	-0.015573	-0.012104	-0.026234	0.009470	0.008290	0.004454
	-0.033896	-0.012611	-0.002423	0.000114	0.018065	-0.017199
	0.031276					
z301	-0.012903	-0.048347	0.054883	-0.021712	0.013651	-0.006195
	0.006837	0.031033	0.022954	-0.000584	-0.000209	-0.001676
	0.010586	-0.000254	0.036767	-0.002441	0.006074	-0.013995
	0.019694	-0.026467	0.017445	0.004180	-0.013553	0.014263
	-0.011060	0.016803	-0.001827	0.010650	0.025391	0.036300
	0.009768					
x302	0.008105	0.000362	0.023930	0.051066	-0.063946	-0.045459
	0.000072	-0.001403	0.012562	-0.000743	-0.018592	0.005471
	0.016810	-0.015932	0.035901	0.031751	-0.003635	-0.028536
	0.050379	0.025842	-0.026414	0.029234	-0.008918	-0.023744
	-0.016501	-0.010398	-0.022405	-0.004511	-0.035958	-0.004406
	0.014335					
y302	0.034911	-0.026597	0.018117	-0.024260	0.031871	0.002958
	0.007525	-0.019460	-0.018961	-0.016127	0.007917	-0.030689
	0.028248	-0.005675	-0.021255	-0.024243	0.014981	-0.002421
	-0.018777	-0.009204	-0.025027	0.009299	0.006885	-0.002024
	-0.029058	-0.013691	0.007127	-0.007797	0.016041	-0.019082
	0.025399					
z302	-0.010690	-0.047534	0.053381	-0.021505	0.013556	-0.006322
	0.009324	0.029053	0.024430	-0.000498	-0.002097	-0.003228
	0.014536	0.004699	0.042911	-0.003237	0.002706	-0.021622
	0.025226	-0.025354	0.020576	0.002505	-0.012300	0.019643
	0.003977	0.014596	-0.004013	0.013553	0.022403	0.037355
	0.003861					

x303	0.006596	0.004509	0.020095	0.053760	-0.059398	-0.046547
	0.002054	-0.002030	0.016050	-0.009356	-0.029200	0.008231
	0.024045	-0.014921	0.031603	0.030407	-0.005362	-0.017751
	0.051332	0.032512	-0.027741	0.022741	-0.014997	-0.033853
	-0.011583	-0.011158	-0.010038	0.001038	-0.013152	-0.002816
	0.004785					
y303	0.035699	-0.024007	0.010890	-0.021084	0.034985	0.002686
	0.010313	-0.028471	-0.015842	-0.015105	0.006354	-0.031234
	0.023747	0.003100	-0.017677	-0.028807	0.006481	-0.012059
	-0.023407	-0.011652	-0.012601	0.002348	0.004345	-0.004071
	-0.021873	-0.011511	0.001652	-0.007803	-0.000044	0.002676
	0.026814					
z303	-0.011803	-0.049622	0.062447	-0.022995	0.016490	-0.008311
	0.013864	0.030475	0.025054	-0.001277	-0.000982	-0.003802
	0.017302	0.004261	0.028298	-0.001815	0.005125	-0.010832
	0.023665	-0.023262	0.008064	0.006169	0.002180	0.014457
	-0.012943	0.019131	0.005693	0.012674	0.014212	0.016938
	-0.003642					
x304	0.005907	0.009532	0.013200	0.056839	-0.055012	-0.051298
	0.002231	-0.007869	0.021322	-0.012276	-0.032215	0.004809
	0.027639	-0.017037	0.034215	0.029016	-0.004566	-0.018876
	0.045127	0.027288	-0.025716	0.024958	-0.016536	-0.035233
	0.003923	-0.008300	-0.004267	-0.001698	-0.002417	0.012960
	-0.004574					
y304	0.037008	-0.020965	0.003268	-0.017585	0.035540	0.006662
	0.012784	-0.031080	-0.014624	-0.017101	0.001028	-0.026260
	0.024119	0.009492	-0.022482	-0.030441	-0.000684	-0.012266
	-0.027712	-0.005692	-0.002960	-0.011171	0.000944	-0.003478
	-0.029608	-0.011324	0.006067	-0.006280	-0.000870	-0.000852
	0.031607					
z304	-0.012720	-0.050259	0.065080	-0.021783	0.018479	-0.007834
	0.019870	0.031197	0.025634	-0.008043	-0.005715	-0.005534
	0.020001	0.011131	0.018006	-0.005555	0.006541	-0.003334
	0.023409	-0.018180	0.006029	0.014193	0.001247	0.009092
	-0.018543	0.013756	0.011331	0.011691	0.008966	0.001795
	0.005957					
x305	0.005227	0.013464	0.008217	0.057934	-0.052395	-0.053361
	0.002020	-0.011239	0.024403	-0.014996	-0.035315	0.002326
	0.031680	-0.019251	0.033962	0.028454	-0.002202	-0.019215
	0.041487	0.021972	-0.022233	0.023424	-0.016963	-0.032525
	0.011516	-0.012775	0.000459	-0.005186	0.008619	0.019801
	-0.004376					

y305	0.038339	-0.020114	-0.002545	-0.015529	0.035159	0.009283
	0.012875	-0.031902	-0.015106	-0.016155	0.002362	-0.027291
	0.022107	0.011601	-0.023860	-0.032608	-0.001409	-0.017065
	-0.029226	-0.008154	0.002306	-0.014443	0.008487	-0.001405
	-0.025438	-0.005704	0.005023	-0.006837	-0.004389	-0.002841
	0.024586					
z305	-0.012849	-0.048817	0.065214	-0.018937	0.019416	-0.008867
	0.024254	0.030570	0.024697	-0.014420	-0.011225	-0.006204
	0.019757	0.014715	0.011974	-0.009765	0.008738	0.000886
	0.017648	-0.014694	0.004644	0.019942	-0.003730	0.004519
	-0.024514	0.016566	0.015303	0.013180	0.002120	-0.002906
	0.014007					
x306	0.004308	0.016761	0.004541	0.057738	-0.050105	-0.054365
	0.000440	-0.015053	0.025182	-0.016411	-0.039737	0.000504
	0.034329	-0.021104	0.031429	0.025927	-0.000305	-0.021066
	0.036280	0.019467	-0.020086	0.023298	-0.015548	-0.031411
	0.015172	-0.016947	0.006152	-0.008665	0.017106	0.022852
	-0.005072					
y306	0.039865	-0.019030	-0.006262	-0.013852	0.033800	0.012339
	0.011608	-0.034342	-0.019135	-0.012524	0.002956	-0.026587
	0.021316	0.014169	-0.029147	-0.033071	-0.003564	-0.022273
	-0.029499	-0.005733	0.003367	-0.011888	0.016444	-0.002691
	-0.024259	-0.007117	0.004730	-0.008452	-0.004833	-0.003162
	0.017688					
z306	-0.012635	-0.046511	0.064166	-0.015624	0.020521	-0.010283
	0.028636	0.029870	0.024441	-0.020269	-0.013412	-0.008569
	0.019130	0.017260	0.008604	-0.011760	0.012156	0.003930
	0.013448	-0.014774	0.006105	0.023498	-0.008844	0.003270
	-0.025171	0.022993	0.016755	0.016718	-0.004524	-0.001841
	0.020650					
x307	0.002857	0.019204	0.001987	0.055911	-0.048743	-0.054013
	-0.001692	-0.018670	0.027485	-0.016926	-0.042326	0.000755
	0.035621	-0.022326	0.031587	0.023716	-0.001037	-0.020230
	0.030962	0.014967	-0.017156	0.021051	-0.016400	-0.028075
	0.015627	-0.020518	0.005600	-0.010788	0.018259	0.027073
	-0.009333					
y307	0.040844	-0.018565	-0.009920	-0.013061	0.031609	0.015381
	0.010420	-0.035182	-0.020566	-0.010047	0.006181	-0.023611
	0.019354	0.016991	-0.028651	-0.032022	-0.008592	-0.019599
	-0.028569	-0.005538	0.005460	-0.011476	0.017652	-0.001697
	-0.026097	-0.010095	0.000048	-0.007779	-0.009167	0.000570
	0.014575					

z307	-0.012119	-0.045029	0.063961	-0.013452	0.020772	-0.011769
	0.031185	0.027270	0.024216	-0.023988	-0.014382	-0.008461
	0.018269	0.017525	0.003392	-0.010902	0.011565	0.002535
	0.010845	-0.016135	0.006155	0.023871	-0.008893	0.004514
	-0.022679	0.027410	0.017510	0.019409	-0.007952	-0.001442
	0.020071					
x308	0.000918	0.020685	-0.000160	0.052278	-0.047831	-0.052224
	-0.003883	-0.023512	0.028785	-0.014541	-0.044565	0.002288
	0.035098	-0.024031	0.028422	0.020772	-0.002660	-0.024723
	0.027348	0.011443	-0.014905	0.017991	-0.013827	-0.023192
	0.013831	-0.026404	0.003789	-0.015055	0.011044	0.027980
	-0.016860					
y308	0.041939	-0.018738	-0.015166	-0.012843	0.029029	0.019259
	0.009760	-0.034735	-0.023684	-0.007311	0.010102	-0.021612
	0.016319	0.019172	-0.028538	-0.033963	-0.009639	-0.020429
	-0.023057	-0.001678	0.006430	-0.008451	0.018101	-0.001648
	-0.025009	-0.017706	0.001123	-0.011294	-0.015097	0.003539
	0.011514					
z308	-0.011396	-0.044080	0.064251	-0.013168	0.020378	-0.013177
	0.030948	0.022165	0.024132	-0.025989	-0.014968	-0.005760
	0.016463	0.015502	-0.002362	-0.006407	0.004283	-0.001042
	0.009681	-0.016927	0.006641	0.022012	-0.006436	0.010321
	-0.017374	0.031509	0.014948	0.024789	-0.009542	0.001885
	0.009479					
x309	-0.001513	0.020856	-0.001471	0.048471	-0.045704	-0.051156
	-0.006188	-0.028021	0.030901	-0.008039	-0.044106	0.005529
	0.037362	-0.023880	0.026124	0.017309	-0.005601	-0.030035
	0.019521	0.006667	-0.016425	0.008567	-0.009405	-0.017958
	0.009033	-0.033618	0.003262	-0.024273	0.002089	0.020991
	-0.023139					
y309	0.043465	-0.020306	-0.020190	-0.011077	0.027026	0.023610
	0.007448	-0.030315	-0.026616	-0.003186	0.019023	-0.016889
	0.018108	0.023930	-0.024247	-0.037100	-0.008664	-0.017995
	-0.017198	0.003081	0.002707	-0.009733	0.016273	-0.005410
	-0.023337	-0.026239	0.007268	-0.023070	-0.013091	0.000397
	0.008645					
z309	-0.010041	-0.042505	0.062597	-0.012625	0.018832	-0.014726
	0.028650	0.014342	0.024289	-0.026802	-0.013028	0.000259
	0.014487	0.012359	-0.011015	0.002019	-0.007491	-0.007886
	0.005681	-0.019132	0.007875	0.015394	-0.003222	0.017240
	-0.007076	0.040235	0.013742	0.030958	-0.010149	0.005544
	-0.010195					

x310	-0.002822	0.021581	-0.005169	0.042935	-0.041827	-0.049528
	-0.005818	-0.028178	0.028216	-0.003356	-0.044472	0.004257
	0.033400	-0.021618	0.031078	0.011576	-0.001227	-0.025422
	0.008834	0.000397	-0.012606	0.009250	-0.006048	-0.018847
	0.000828	-0.039350	-0.000914	-0.030480	-0.012212	0.008104
	-0.003811					
y310	0.045420	-0.020656	-0.026091	-0.008693	0.025817	0.028902
	0.006644	-0.020510	-0.033405	-0.003088	0.029138	-0.016096
	0.017295	0.029408	-0.012400	-0.040784	0.001444	-0.005853
	-0.007491	0.007148	0.004902	-0.001411	0.010997	-0.015131
	-0.024494	-0.034059	0.010378	-0.032639	-0.009786	-0.007358
	0.027962					
z310	-0.007043	-0.038824	0.053707	-0.012263	0.015084	-0.014269
	0.025858	0.006147	0.019783	-0.029530	-0.011214	0.001942
	0.006753	0.007988	-0.019192	0.012947	-0.016729	-0.011231
	0.000745	-0.025715	0.018382	0.014831	0.002819	0.022517
	0.004990	0.050266	0.010253	0.041068	-0.010139	0.012061
	-0.019619					
x311	-0.001206	0.018751	-0.006107	0.036891	-0.037020	-0.041097
	-0.007554	-0.029252	0.023087	-0.001565	-0.041899	0.006897
	0.030595	-0.015938	0.041318	-0.003502	0.009263	-0.007737
	0.006037	-0.007877	-0.009978	0.011412	-0.006239	-0.037275
	-0.011091	-0.042145	0.011040	-0.039094	-0.013640	-0.011050
	0.020808					
y311	0.045853	-0.020339	-0.028518	-0.004146	0.023557	0.030259
	0.007699	-0.012332	-0.038104	-0.001987	0.038957	-0.016656
	0.019989	0.031616	0.001854	-0.041678	0.012789	0.004313
	0.002548	0.011972	0.002148	0.006227	0.000187	-0.024619
	-0.027519	-0.044546	0.009291	-0.043795	-0.010667	-0.016034
	0.053913					
z311	-0.005296	-0.034449	0.044522	-0.009812	0.008772	-0.015298
	0.027019	-0.002930	0.012423	-0.025465	-0.007941	0.002489
	0.007638	0.003816	-0.012292	0.018575	-0.018238	-0.013041
	-0.003518	-0.031029	0.016974	0.017445	-0.001181	0.025387
	0.009481	0.040004	0.008032	0.031921	-0.015301	0.011895
	-0.004593					
x312	0.058728	-0.045401	0.028074	0.025114	-0.009631	0.013075
	0.022123	0.002677	0.003445	-0.003739	0.027808	-0.021615
	0.000482	0.053724	0.002207	0.021842	-0.014834	0.010131
	-0.003846	-0.014724	-0.006499	-0.028813	-0.046374	-0.001615
	0.003191	-0.009560	0.005300	0.010675	-0.018118	0.024893
	-0.006478					

y312 -0.007297 -0.019355 -0.001769 -0.024779 -0.039840 0.000503
0.022873 -0.011967 0.015300 -0.007901 -0.041913 0.034905
-0.022207 -0.024382 0.003172 -0.010191 -0.002186 -0.033382
-0.002258 0.016434 -0.013154 -0.005179 0.001891 0.002620
0.056425 0.033335 0.017906 -0.014474 0.020398 -0.002925
0.030717

z312 -0.001522 -0.024347 -0.048602 0.052344 -0.013604 0.025963
-0.001995 0.008260 0.009731 0.001897 -0.014395 -0.006867
-0.009633 -0.028299 -0.015860 -0.029959 -0.021833 -0.018832
-0.016353 -0.046826 -0.007191 0.014637 0.006002 0.004991
-0.002378 -0.022983 0.037028 -0.029666 0.009804 -0.052862
-0.005486

x313 0.056820 -0.049181 0.033194 0.022731 -0.005514 0.011425
0.018744 0.000228 -0.001574 -0.011376 0.033471 -0.025401
-0.003099 0.053939 0.000603 0.039691 -0.015482 0.012487
-0.001669 -0.012285 -0.015678 -0.029024 -0.045134 -0.006557
0.013671 -0.012129 -0.003444 0.006179 -0.011397 0.016123
-0.005700

y313 -0.007372 -0.020420 -0.006478 -0.025948 -0.038943 0.002125
0.026640 -0.007209 0.013484 -0.009125 -0.052455 0.028333
-0.030444 -0.015635 0.003547 -0.019635 -0.001915 -0.033669
0.012707 0.029768 0.004062 -0.007243 0.001708 -0.004329
0.051712 0.021361 0.026156 -0.025001 0.014869 -0.006141
0.000388

z313 0.002542 -0.023898 -0.045428 0.052873 -0.013790 0.026529
-0.003493 0.009046 0.011400 -0.000454 -0.006157 -0.011772
0.007272 -0.025365 -0.021591 -0.023147 -0.026621 -0.032189
-0.011451 -0.057927 -0.002611 0.015690 0.010162 -0.023744
-0.018114 -0.015651 0.015537 -0.026766 0.003587 -0.042435
-0.014159

x314 0.054304 -0.052155 0.037830 0.018904 0.000062 0.007000
0.015101 -0.003898 -0.005277 -0.019324 0.035304 -0.027896
-0.010225 0.047700 0.001308 0.056832 -0.013756 0.015689
-0.003151 -0.008867 -0.025114 -0.029633 -0.043820 -0.004926
0.030276 -0.017989 -0.004255 0.011916 -0.001599 0.002531
0.002056

y314 -0.005380 -0.019804 -0.007384 -0.025417 -0.039277 0.002659
0.027714 -0.007688 0.011817 -0.009405 -0.061327 0.024731
-0.035365 -0.007646 0.003032 -0.023862 0.000379 -0.036116
0.026937 0.032766 0.015460 -0.009816 0.006068 -0.009330
0.037117 0.010656 0.020135 -0.033753 0.002486 0.000771
-0.008008

z314	0.006095	-0.027076	-0.035376	0.053872	-0.014816	0.022738
	-0.008408	0.015092	0.014070	-0.003987	-0.001323	-0.013591
	0.016107	-0.023046	-0.032655	-0.021096	-0.019964	-0.049694
	-0.009307	-0.060332	0.007747	0.015554	0.009074	-0.037213
	-0.038459	-0.019260	-0.002279	-0.025297	-0.010332	-0.037987
	-0.015296					
x315	0.053160	-0.051966	0.040579	0.015922	0.003480	0.004707
	0.011761	-0.007748	-0.006477	-0.023838	0.039771	-0.028409
	-0.014973	0.041962	0.001320	0.070369	-0.011992	0.016682
	-0.006742	-0.013825	-0.033737	-0.030468	-0.037327	-0.003714
	0.038887	-0.020484	-0.015588	0.016168	0.009420	-0.001374
	0.015035					
y315	-0.002703	-0.016995	-0.006953	-0.023515	-0.040610	0.004287
	0.027698	-0.008795	0.010051	-0.008203	-0.062280	0.021945
	-0.029138	0.000465	0.002276	-0.020856	0.000689	-0.040099
	0.035906	0.020436	0.017838	-0.014126	0.017673	-0.008560
	0.028073	0.006387	0.002138	-0.034253	-0.000251	0.012879
	-0.008236					
z315	0.008126	-0.028021	-0.026701	0.053950	-0.016977	0.019456
	-0.008810	0.022647	0.013954	-0.005356	-0.003665	-0.014349
	0.024461	-0.018988	-0.035660	-0.025759	-0.015299	-0.054242
	-0.002678	-0.055411	0.015038	0.008725	0.010238	-0.027531
	-0.037754	-0.023660	0.007854	-0.016798	-0.018720	-0.035153
	-0.032970					
x316	0.050739	-0.052638	0.041542	0.014128	0.006376	0.001419
	0.010556	-0.007701	-0.007337	-0.027060	0.041489	-0.031249
	-0.017542	0.035664	-0.002070	0.074761	-0.007936	0.014075
	-0.010949	-0.013583	-0.035830	-0.029733	-0.036488	-0.004584
	0.046516	-0.026033	-0.013266	0.015972	0.013789	-0.010795
	0.013666					
y316	-0.002477	-0.015887	-0.007411	-0.022195	-0.040363	0.004488
	0.027017	-0.005221	0.007935	-0.004767	-0.060899	0.018896
	-0.024057	0.006764	0.000624	-0.021446	0.001120	-0.044709
	0.043711	0.015782	0.019668	-0.017828	0.030674	-0.010021
	0.021829	0.003228	-0.010106	-0.037035	0.002003	0.019828
	-0.008327					
z316	0.010229	-0.027282	-0.020498	0.052427	-0.018334	0.017913
	-0.011314	0.025771	0.012590	-0.005314	-0.003085	-0.012197
	0.027202	-0.015816	-0.031519	-0.025560	-0.012102	-0.050777
	0.002950	-0.050720	0.013447	0.000239	0.016222	-0.016477
	-0.036714	-0.020456	0.008090	-0.011589	-0.019963	-0.026052
	-0.032812					

x317	0.048536	-0.052000	0.041604	0.013972	0.006591	-0.005086
	0.010852	-0.007317	-0.006159	-0.027233	0.039472	-0.030839
	-0.008276	0.029005	-0.007416	0.074190	-0.005275	-0.005951
	-0.018922	-0.012637	-0.041860	-0.030736	-0.027993	0.003673
	0.041038	-0.033285	-0.022401	0.007261	-0.003303	-0.021996
	0.019804					
y317	-0.002251	-0.015623	-0.007619	-0.022044	-0.039247	0.006468
	0.025339	-0.002032	0.005152	-0.000820	-0.055385	0.016150
	-0.025739	0.012851	0.001694	-0.019858	-0.000444	-0.038204
	0.049849	0.011232	0.019683	-0.020774	0.036679	-0.016578
	0.023251	0.003999	-0.017859	-0.036457	0.013715	0.024974
	-0.007664					
z317	0.010124	-0.027899	-0.015107	0.049927	-0.018477	0.015699
	-0.012792	0.027970	0.008050	-0.004625	-0.001335	-0.011548
	0.022466	-0.014124	-0.022401	-0.020349	-0.006783	-0.039789
	0.005846	-0.040614	0.007990	-0.004768	0.015951	-0.007371
	-0.030109	-0.018444	0.007370	-0.008154	-0.016666	-0.022119
	-0.029858					
x318	0.049334	-0.050357	0.035829	0.014246	0.004580	-0.010066
	0.006610	-0.003080	-0.000517	-0.026057	0.030107	-0.024219
	-0.006499	0.016920	-0.015161	0.060108	0.007819	-0.020475
	-0.018788	-0.003650	-0.046839	-0.021662	-0.023755	0.016560
	0.030685	-0.030846	-0.027672	0.002568	-0.014362	-0.031813
	0.011960					
y318	-0.003034	-0.016113	-0.005524	-0.022158	-0.035809	0.007763
	0.022663	-0.000271	0.000944	0.003352	-0.048328	0.012389
	-0.026221	0.022414	0.008778	-0.014899	-0.010838	-0.027757
	0.050559	0.005550	0.021236	-0.028503	0.039975	-0.025315
	0.025083	0.005647	-0.019787	-0.035622	0.021785	0.029738
	-0.002039					
z318	0.009671	-0.029961	-0.009066	0.046422	-0.016310	0.014831
	-0.014620	0.029019	0.001943	-0.002995	0.003700	-0.010974
	0.015443	-0.009347	-0.010629	-0.011238	-0.002318	-0.022119
	0.008280	-0.032100	0.002966	-0.012354	0.018549	-0.003133
	-0.021923	-0.018329	0.005278	-0.002635	-0.010421	-0.011794
	-0.017691					
x319	0.053348	-0.045433	0.026524	0.014073	0.001477	-0.007218
	0.004419	-0.003080	0.007142	-0.020380	0.012393	-0.016090
	-0.009860	0.008871	-0.009872	0.038759	0.004242	-0.017525
	0.001152	-0.001561	-0.040670	-0.000625	-0.007086	0.020012
	0.019705	-0.013906	-0.016016	0.009477	0.007425	-0.021430
	-0.003373					

y319	-0.004236	-0.017413	-0.001229	-0.021923	-0.032966	0.005743
	0.020767	-0.000263	-0.002082	0.004471	-0.036396	0.007468
	-0.023993	0.027611	0.011728	-0.008350	-0.012389	-0.024825
	0.040816	0.004712	0.018001	-0.038760	0.032749	-0.029456
	0.032600	0.000565	-0.026831	-0.034562	0.012578	0.028028
	0.005213					
z319	0.009721	-0.031030	-0.004715	0.043087	-0.013471	0.014406
	-0.016053	0.028418	0.000003	-0.001211	0.009312	-0.011688
	0.011057	-0.006688	-0.001732	-0.009547	0.010171	-0.006692
	0.011506	-0.019821	0.002437	-0.021131	0.015387	-0.001583
	-0.015030	-0.023318	0.003985	-0.001878	-0.011260	0.000513
	-0.010739					
x320	0.057291	-0.037567	0.014017	0.010528	-0.000297	-0.003539
	0.008070	-0.007258	0.019920	-0.018607	0.003912	-0.016332
	-0.009073	0.010691	0.002755	0.009604	-0.002595	-0.004587
	0.005011	0.014494	-0.029859	0.007362	-0.008564	0.018432
	0.010682	-0.013587	-0.002383	0.013624	0.036623	-0.006651
	-0.016645					
y320	-0.006230	-0.020407	0.004425	-0.019855	-0.028276	0.003441
	0.014873	0.002182	-0.005908	0.007482	-0.031619	0.006482
	-0.019035	0.030559	0.013941	0.002567	-0.013058	-0.031128
	0.042103	-0.006666	0.017282	-0.036925	0.035958	-0.031691
	0.032296	0.007963	-0.038320	-0.029766	0.007055	0.029333
	0.012264					
z320	0.011716	-0.032981	-0.004185	0.040336	-0.009354	0.013377
	-0.015578	0.027595	0.002353	0.003106	0.008480	-0.008525
	0.004678	-0.000315	0.002225	-0.011865	0.016835	-0.001474
	0.024283	-0.011340	0.000351	-0.028386	0.017445	-0.000018
	-0.008423	-0.024754	0.002069	0.008232	-0.008730	0.017505
	-0.013698					
x321	0.056359	-0.034665	0.005735	0.011797	-0.001011	-0.000099
	0.009508	-0.019821	0.022263	-0.017867	-0.001714	-0.007912
	-0.012465	-0.001931	0.002188	0.002919	-0.010468	-0.003647
	-0.007874	0.007700	-0.032164	0.007784	-0.000669	0.013613
	0.004088	-0.004716	0.001204	0.010946	0.024843	-0.015738
	0.001430					
y321	-0.003803	-0.021020	0.007227	-0.019753	-0.027829	-0.000218
	0.010158	0.006172	-0.005195	0.010978	-0.027835	0.002916
	-0.011544	0.031674	0.025771	-0.001434	-0.004844	-0.027583
	0.049533	-0.004627	0.011742	-0.036670	0.031332	-0.037187
	0.034424	0.022207	-0.030707	-0.010509	0.015751	0.033674
	0.010314					

z321	0.012652	-0.036110	-0.000129	0.036725	-0.006857	0.011288
	-0.017502	0.031415	-0.002622	0.007875	0.009198	-0.008155
	0.003850	0.005435	0.013793	-0.007604	0.022069	0.002803
	0.036132	-0.000850	-0.002995	-0.033704	0.022173	0.003224
	-0.003992	-0.023269	0.002464	0.014991	0.001307	0.027438
	-0.018356					
x322	0.055590	-0.031441	0.002696	0.008633	-0.001282	-0.000523
	0.009583	-0.025213	0.021640	-0.019015	-0.004957	-0.011668
	-0.017156	-0.001498	-0.000475	0.007285	-0.018426	-0.002604
	-0.005943	0.008057	-0.018150	-0.004436	-0.008075	0.018045
	0.015240	-0.004153	-0.000219	0.017260	0.033537	-0.010822
	0.005109					
y322	-0.002468	-0.022678	0.008048	-0.018132	-0.028785	-0.000538
	0.011959	0.007509	-0.006169	0.005502	-0.023475	0.001117
	-0.005793	0.025437	0.020628	-0.004470	0.011316	-0.024212
	0.047502	0.003745	0.001618	-0.026813	0.027813	-0.039632
	0.019348	0.015368	-0.037295	-0.005235	0.006684	0.030391
	0.029965					
z322	0.014255	-0.036600	0.005467	0.039882	-0.008927	0.015473
	-0.011539	0.031095	-0.002981	-0.003269	0.011561	0.001361
	0.006930	0.002989	0.003738	0.000125	0.026568	0.021104
	0.033378	0.005961	-0.013232	-0.025861	0.023067	0.006595
	-0.020660	-0.044406	0.009834	0.009970	-0.001001	-0.003835
	-0.003156					
x323	0.054227	-0.028019	-0.001253	0.006649	0.000344	-0.001134
	0.008737	-0.031381	0.021451	-0.017612	-0.007613	-0.014072
	-0.021025	-0.002020	-0.000561	0.007100	-0.026246	-0.002399
	-0.007033	0.005478	-0.010196	-0.007563	-0.006224	0.018525
	0.021973	0.001937	-0.001775	0.018634	0.036159	-0.000911
	0.011419					
y323	-0.003007	-0.024276	0.008636	-0.016696	-0.028610	-0.002017
	0.012520	0.011201	-0.005460	0.004629	-0.021658	-0.002694
	-0.002212	0.024998	0.015795	-0.009354	0.018214	-0.025862
	0.047294	0.007632	-0.002827	-0.020388	0.027612	-0.033902
	0.015796	0.015836	-0.040706	-0.001031	0.001344	0.034624
	0.038467					
z323	0.013687	-0.037450	0.007043	0.042351	-0.012153	0.018485
	-0.007413	0.030142	-0.001438	-0.007277	0.011194	0.004061
	0.004576	-0.002182	-0.002621	0.003520	0.025199	0.026206
	0.026865	0.006866	-0.014396	-0.025987	0.024382	0.015740
	-0.012055	-0.051831	0.014981	0.011581	-0.003696	-0.017136
	-0.001201					

x324	0.053070	-0.025314	-0.004634	0.005774	0.002370	-0.001279
	0.008635	-0.035511	0.022218	-0.017156	-0.007649	-0.016108
	-0.023695	-0.003886	-0.000005	0.004971	-0.032590	-0.001510
	-0.008702	0.001088	-0.003065	-0.008498	-0.004005	0.018333
	0.024333	0.005307	-0.003478	0.020363	0.038421	0.005007
	0.015309					
y324	-0.003856	-0.024795	0.008478	-0.016250	-0.027329	-0.003098
	0.011840	0.012422	-0.006013	0.005824	-0.022451	-0.006320
	-0.000841	0.025067	0.012222	-0.014081	0.022277	-0.026262
	0.047286	0.008617	-0.003870	-0.017730	0.026528	-0.029659
	0.013011	0.015153	-0.045475	0.001163	0.000329	0.032350
	0.044633					
z324	0.012881	-0.037573	0.007109	0.042421	-0.015252	0.019987
	-0.006008	0.026850	-0.001216	-0.008277	0.006310	0.007553
	0.000804	-0.005451	-0.007009	0.005752	0.023316	0.029600
	0.022401	0.009741	-0.015130	-0.028600	0.026451	0.023327
	-0.004451	-0.052730	0.019539	0.009209	-0.004105	-0.026376
	0.003251					
x325	0.051188	-0.023056	-0.007855	0.004801	0.004846	-0.001266
	0.006820	-0.037958	0.021805	-0.017031	-0.008170	-0.017867
	-0.025296	-0.004186	-0.004714	-0.000162	-0.037096	-0.003103
	-0.015976	-0.001294	0.003145	-0.012820	-0.003983	0.019539
	0.023463	0.008913	-0.002579	0.015469	0.036989	0.005345
	0.013479					
y325	-0.004128	-0.024290	0.008592	-0.015989	-0.025324	-0.003166
	0.011753	0.011595	-0.005772	0.006825	-0.022572	-0.009956
	-0.000276	0.025174	0.012274	-0.016547	0.024437	-0.023773
	0.047657	0.006819	-0.002634	-0.014710	0.026174	-0.026704
	0.013659	0.013245	-0.051604	0.003950	0.001281	0.030781
	0.051708					
z325	0.012489	-0.037222	0.006958	0.042463	-0.017993	0.021010
	-0.004590	0.022251	-0.000292	-0.008655	0.003086	0.010172
	-0.002690	-0.008660	-0.006249	0.009620	0.021316	0.034449
	0.021419	0.010882	-0.015258	-0.028776	0.028791	0.029272
	0.003916	-0.051152	0.022099	0.009855	-0.002219	-0.029551
	0.010073					
x326	0.049185	-0.020786	-0.010677	0.003865	0.007196	-0.000974
	0.005484	-0.041110	0.021357	-0.016582	-0.008370	-0.018905
	-0.025776	-0.002429	-0.009102	-0.003626	-0.041239	-0.004135
	-0.024433	-0.004911	0.008403	-0.011196	-0.007113	0.019507
	0.021388	0.011660	-0.008202	0.009808	0.032415	0.005514
	0.010331					

y326	-0.004324	-0.023045	0.008982	-0.015677	-0.022534	-0.003152
	0.011304	0.010715	-0.005722	0.007375	-0.022886	-0.013267
	-0.001568	0.025375	0.012397	-0.019770	0.025591	-0.021190
	0.047041	0.004120	0.001006	-0.014875	0.026730	-0.024411
	0.015320	0.009441	-0.053422	0.005312	0.003343	0.026707
	0.055036					
z326	0.011743	-0.036633	0.007487	0.042835	-0.018977	0.020342
	-0.004190	0.017577	-0.001740	-0.010703	0.000562	0.012947
	-0.005542	-0.008816	-0.006560	0.010764	0.018666	0.036544
	0.018452	0.012467	-0.014410	-0.028718	0.028693	0.030761
	0.005927	-0.047015	0.025049	0.009352	-0.001643	-0.026949
	0.012830					
x327	0.046595	-0.018793	-0.012682	0.002272	0.010513	-0.000837
	0.003878	-0.043973	0.020450	-0.014626	-0.007638	-0.021054
	-0.024795	0.000226	-0.009545	-0.009354	-0.044444	-0.008009
	-0.025369	-0.009916	0.012392	-0.009005	-0.010263	0.016811
	0.020212	0.014003	-0.012365	0.002339	0.026289	0.011243
	0.002113					
y327	-0.004359	-0.020833	0.009369	-0.014556	-0.019633	-0.003469
	0.010769	0.009311	-0.006117	0.007478	-0.023504	-0.015462
	-0.004443	0.026126	0.010459	-0.021645	0.025126	-0.018272
	0.040836	0.000519	0.006868	-0.015282	0.026414	-0.023248
	0.015747	0.003790	-0.055326	0.006277	0.004530	0.017114
	0.056971					
z327	0.010766	-0.035702	0.008905	0.043782	-0.018291	0.018171
	-0.004708	0.011599	-0.005902	-0.015523	-0.001366	0.016280
	-0.007892	-0.003680	-0.009983	0.010480	0.014709	0.036796
	0.009570	0.013961	-0.011896	-0.025801	0.024173	0.027217
	-0.000015	-0.040956	0.024542	0.007919	-0.002609	-0.019892
	0.011704					
x328	0.044151	-0.017555	-0.013891	0.000477	0.013299	-0.001000
	0.001849	-0.044048	0.020342	-0.011540	-0.004634	-0.022419
	-0.024552	-0.002608	-0.008809	-0.014380	-0.043700	-0.019113
	-0.019167	-0.013714	0.014756	-0.014531	-0.005176	0.012519
	0.017321	0.010901	-0.009189	-0.005955	0.020767	0.002095
	-0.005195					
y328	-0.004709	-0.017087	0.009525	-0.012331	-0.016174	-0.003893
	0.010233	0.005846	-0.007242	0.008284	-0.025517	-0.017077
	-0.008136	0.028757	0.007280	-0.024361	0.021122	-0.012798
	0.028043	-0.004462	0.016510	-0.011995	0.021253	-0.023986
	0.015995	-0.001945	-0.057446	0.005378	0.004886	0.008714
	0.056328					

z328	0.009504	-0.034797	0.009698	0.045058	-0.017233	0.015078
	-0.005913	0.003629	-0.011589	-0.022221	-0.003950	0.020438
	-0.011853	0.003234	-0.014723	0.009335	0.007285	0.033790
	-0.001910	0.013942	-0.007403	-0.022021	0.019215	0.022632
	-0.008279	-0.031437	0.024144	0.008638	-0.003132	-0.011006
	0.009946					
x329	0.042277	-0.016278	-0.014670	-0.002560	0.016269	-0.001364
	0.002077	-0.042124	0.024475	-0.005631	0.000197	-0.027634
	-0.023141	-0.010152	-0.004477	-0.018672	-0.034296	-0.019227
	-0.013713	-0.019099	0.019048	-0.016759	-0.000008	0.006925
	0.026627	0.003600	-0.002270	-0.012410	0.016280	-0.009560
	-0.006926					
y329	-0.005162	-0.012134	0.009666	-0.009036	-0.012466	-0.003654
	0.008739	0.000309	-0.009795	0.008741	-0.028841	-0.016420
	-0.013373	0.032637	0.002091	-0.028870	0.012710	-0.008467
	0.010481	-0.010586	0.029213	-0.007691	0.013673	-0.025923
	0.012501	-0.008139	-0.058056	0.001685	0.006631	-0.003010
	0.050155					
z329	0.008377	-0.033353	0.008349	0.045790	-0.015460	0.011299
	-0.006999	-0.005518	-0.016681	-0.029293	-0.007192	0.023024
	-0.016859	0.010174	-0.019121	0.007751	-0.001481	0.034328
	-0.016923	0.010689	0.000882	-0.014788	0.011040	0.016239
	-0.012844	-0.018894	0.024308	0.012396	-0.002049	0.000815
	0.011184					
x330	0.040750	-0.014974	-0.011741	-0.008802	0.017490	0.001054
	0.001729	-0.040926	0.030628	-0.001787	0.007238	-0.026800
	-0.020452	-0.023595	0.000790	-0.022812	-0.024392	-0.010835
	-0.006145	-0.030438	0.018846	-0.028088	0.009812	0.007248
	0.032995	-0.002622	-0.001629	-0.011128	0.005866	-0.022165
	0.005037					
y330	-0.003848	-0.008301	0.009807	-0.008606	-0.006138	0.001170
	0.004232	-0.006394	-0.011881	0.006185	-0.030472	-0.015455
	-0.020479	0.039102	-0.003416	-0.034637	0.002750	0.002301
	-0.001075	-0.022854	0.043799	-0.008007	0.009073	-0.026817
	0.015738	-0.011050	-0.051902	0.001684	0.020146	-0.017742
	0.031575					
z330	0.007043	-0.031424	0.007148	0.042265	-0.015508	0.007378
	-0.008353	-0.013841	-0.018133	-0.031581	-0.007362	0.026735
	-0.019417	0.005044	-0.019338	0.006039	-0.008522	0.033711
	-0.026931	0.002660	0.002222	-0.017091	0.008438	0.017381
	-0.015654	-0.008347	0.017196	0.017692	-0.015894	0.006718
	0.027083					

x331	0.003368	-0.030448	0.004071	0.022255	-0.013154	0.010821
	-0.036871	0.024556	-0.038343	0.041671	0.007072	0.012642
	0.011548	-0.055030	-0.045305	0.017163	0.005777	0.017674
	0.029199	-0.003092	0.024579	0.013180	0.012459	0.016497
	-0.007517	0.006895	0.015594	0.002562	-0.047818	0.012528
	-0.014009					
y331	-0.032238	0.014123	-0.002783	-0.008455	-0.040579	-0.020695
	-0.027742	-0.007612	0.009074	-0.038221	0.001281	-0.010208
	0.005438	-0.062963	0.010395	-0.047564	-0.046334	-0.002316
	-0.031320	0.008612	-0.025404	-0.017478	-0.023624	0.012430
	0.015985	-0.005468	-0.003604	0.013859	0.012069	0.026284
	0.016539					
z331	-0.020031	0.014658	-0.007835	0.016456	0.012872	0.027976
	-0.001381	0.024346	0.048117	-0.006033	-0.016745	-0.025656
	0.029744	0.021089	-0.008198	-0.021950	-0.020710	-0.063462
	0.000267	-0.001198	-0.025624	0.030237	0.004688	-0.018163
	-0.002791	0.026361	0.035231	0.016336	0.024038	-0.047912
	0.009590					
x332	0.002026	-0.026684	0.003123	0.021414	-0.012353	0.011162
	-0.033435	0.024880	-0.040146	0.048054	-0.000065	0.006450
	0.007885	-0.060229	-0.060782	0.016744	-0.004009	0.018609
	0.028481	-0.003812	0.023286	0.016664	0.008806	0.001486
	-0.004247	0.013887	0.035523	-0.001833	-0.038818	-0.001689
	-0.010278					
y332	-0.033094	0.013886	-0.003513	-0.009800	-0.043679	-0.018057
	-0.024584	-0.003770	0.005952	-0.034881	-0.004860	-0.003864
	0.003699	-0.068483	0.011264	-0.055922	-0.051390	-0.006472
	-0.030211	-0.002249	-0.011375	-0.004652	-0.026023	0.012837
	0.006396	-0.004087	-0.004726	0.007206	0.011752	0.032013
	0.016621					
z332	-0.021476	0.018579	-0.004152	0.017334	0.017133	0.026715
	-0.004650	0.021474	0.053859	-0.003688	-0.012928	-0.027622
	0.028115	0.021202	-0.010216	-0.021196	-0.035150	-0.058986
	-0.003199	-0.007376	-0.019263	0.040736	0.003547	-0.019486
	-0.000115	0.018755	0.039162	0.016726	0.024435	-0.046272
	0.010666					
x333	-0.000115	-0.023470	-0.000463	0.019924	-0.011546	0.010757
	-0.030717	0.026983	-0.043751	0.052546	-0.016456	0.002182
	0.004675	-0.064893	-0.067921	0.010511	-0.013165	0.024830
	0.037047	-0.003310	0.026726	0.023193	0.003762	-0.005214
	-0.011401	0.020518	0.047742	-0.007368	-0.040306	-0.002850
	-0.009947					

y333	-0.034579	0.013806	-0.004150	-0.009402	-0.045716	-0.016319
	-0.021442	0.001376	0.001350	-0.028791	-0.012803	0.002460
	0.001526	-0.071750	0.011990	-0.059440	-0.054351	-0.007515
	-0.029059	-0.008921	0.006239	0.011339	-0.028443	0.017213
	-0.003669	-0.000979	-0.000451	-0.000647	0.006348	0.040144
	0.013599					
z333	-0.020684	0.021346	-0.002977	0.017502	0.021194	0.023439
	-0.007731	0.019525	0.054874	-0.002315	-0.014939	-0.030845
	0.027391	0.017721	-0.010127	-0.025783	-0.041245	-0.049295
	0.003302	-0.005904	-0.010529	0.046722	0.002528	-0.005960
	-0.001124	0.012027	0.034464	0.022115	0.019226	-0.030902
	-0.000713					
x334	-0.003014	-0.021370	-0.001846	0.018440	-0.009393	0.011727
	-0.029349	0.030626	-0.042752	0.053203	-0.027086	0.003574
	0.004180	-0.066149	-0.071939	0.007527	-0.021184	0.030715
	0.037821	0.000508	0.026069	0.025013	-0.005441	-0.006552
	-0.014360	0.034990	0.047342	-0.011551	-0.041143	-0.001078
	-0.006300					
y334	-0.036004	0.013527	-0.003685	-0.007697	-0.046731	-0.013844
	-0.019910	0.006459	-0.001770	-0.024903	-0.017407	0.008877
	0.001323	-0.073498	0.012695	-0.060144	-0.054087	-0.007476
	-0.031843	-0.010856	0.019163	0.021622	-0.030309	0.022547
	-0.013231	0.009781	-0.002039	-0.011860	0.003318	0.047786
	0.016139					
z334	-0.019817	0.022844	-0.001181	0.017719	0.025306	0.022175
	-0.011251	0.019651	0.057533	-0.003987	-0.012356	-0.030794
	0.028899	0.013927	-0.009163	-0.027683	-0.042996	-0.040370
	0.005140	0.001504	-0.004783	0.045116	0.000898	0.006663
	-0.001183	0.016592	0.023873	0.021100	0.016461	-0.017361
	-0.002750					
x335	-0.004240	-0.019866	-0.001412	0.016755	-0.007148	0.012553
	-0.029102	0.032751	-0.038902	0.055061	-0.030394	0.004140
	0.003752	-0.064924	-0.070021	0.008737	-0.021586	0.035472
	0.035921	-0.002101	0.021166	0.019017	-0.013527	-0.012302
	-0.008615	0.038131	0.051544	-0.012642	-0.035164	0.006345
	-0.011177					
y335	-0.035390	0.012599	-0.002106	-0.006498	-0.048063	-0.011744
	-0.020299	0.008664	-0.002440	-0.020441	-0.015448	0.012178
	-0.000364	-0.073915	0.017345	-0.057007	-0.046491	-0.007841
	-0.035152	-0.016763	0.027934	0.020951	-0.031052	0.019155
	-0.015065	0.008922	0.002255	-0.018709	0.005739	0.054505
	0.016980					

z335	-0.018063	0.023902	0.000244	0.017446	0.028435	0.021308
	-0.015444	0.019459	0.059551	-0.003878	-0.008911	-0.031356
	0.028709	0.010715	-0.004746	-0.029376	-0.041520	-0.033587
	0.006796	0.003617	-0.000950	0.037428	0.001349	0.011621
	-0.002189	0.012412	0.018068	0.020758	0.018705	-0.005728
	-0.004607					
x336	-0.005441	-0.018704	-0.000443	0.014802	-0.003502	0.012775
	-0.028077	0.032884	-0.035706	0.054263	-0.024089	-0.000455
	0.005939	-0.060203	-0.063504	0.018131	-0.015086	0.035883
	0.033400	-0.002887	0.016998	0.009512	-0.013823	-0.019066
	0.002718	0.035674	0.048903	-0.015508	-0.040913	0.006666
	-0.010339					
y336	-0.035205	0.012435	0.000514	-0.006192	-0.046915	-0.011923
	-0.021270	0.008278	-0.006237	-0.017624	-0.002674	0.003459
	0.002035	-0.070277	0.028213	-0.042082	-0.030388	-0.011848
	-0.037429	-0.022499	0.034580	0.011656	-0.029045	0.011719
	-0.007600	0.002434	-0.002489	-0.027490	-0.004064	0.052086
	0.021234					
z336	-0.016576	0.024348	0.001176	0.016389	0.031795	0.020668
	-0.019479	0.017787	0.060851	-0.003111	-0.004638	-0.031114
	0.029474	0.009129	-0.002836	-0.031095	-0.038416	-0.031434
	0.007464	0.006008	-0.000222	0.026894	0.001070	0.013462
	-0.004146	0.007710	0.011606	0.019734	0.014473	0.001496
	-0.004964					
x337	-0.007012	-0.017910	0.000973	0.012882	0.000687	0.011844
	-0.026957	0.032769	-0.032040	0.055504	-0.018069	-0.004655
	0.007474	-0.054213	-0.060596	0.029318	-0.008722	0.034618
	0.030170	-0.002785	0.015661	-0.000857	-0.008093	-0.025416
	0.009611	0.027497	0.045762	-0.017476	-0.049424	0.004681
	-0.007348					
y337	-0.036965	0.011909	0.004965	-0.006807	-0.043507	-0.014178
	-0.023412	0.006814	-0.011285	-0.011890	0.009247	-0.005477
	0.003382	-0.065705	0.032773	-0.017925	-0.016941	-0.017999
	-0.044111	-0.024680	0.037466	-0.001685	-0.019519	0.004147
	-0.002254	-0.009496	-0.006087	-0.030240	-0.016965	0.037720
	0.026337					
z337	-0.015297	0.025033	-0.000016	0.015277	0.035285	0.020693
	-0.021547	0.016428	0.064366	-0.001697	-0.002145	-0.031056
	0.031074	0.009742	-0.001707	-0.036217	-0.032926	-0.029938
	0.012180	0.007705	-0.001797	0.019659	-0.002472	0.011244
	-0.001631	0.003524	0.005389	0.020286	0.011882	0.016285
	-0.005108					

x338	-0.008083	-0.017073	0.003409	0.010895	0.004675	0.010068
	-0.026950	0.032841	-0.029400	0.055688	-0.013597	-0.008726
	0.007579	-0.045915	-0.059243	0.036121	-0.007019	0.032073
	0.026204	-0.001209	0.016063	-0.011152	-0.002378	-0.027117
	0.008706	0.014666	0.041535	-0.012341	-0.054602	0.004275
	-0.004718					
y338	-0.038879	0.009609	0.011186	-0.005959	-0.037812	-0.018046
	-0.026740	0.004306	-0.018286	-0.009045	0.023136	-0.010286
	0.003390	-0.058142	0.030147	0.005433	-0.011122	-0.027611
	-0.051343	-0.017643	0.032111	-0.014426	-0.007547	0.003198
	-0.004876	-0.028769	-0.008543	-0.015804	-0.025687	0.023314
	0.035330					
z338	-0.013749	0.026274	-0.001959	0.013903	0.037389	0.021625
	-0.023913	0.013534	0.064547	-0.001124	-0.001762	-0.036165
	0.031682	0.007465	0.002492	-0.035074	-0.023936	-0.026446
	0.018385	0.006092	-0.000025	0.015361	-0.005708	0.009424
	0.003894	0.002778	0.002907	0.012791	0.013689	0.027367
	-0.006848					
x339	-0.008697	-0.015334	0.006570	0.009458	0.008134	0.008214
	-0.026689	0.035162	-0.024972	0.052741	-0.012293	-0.010260
	0.007966	-0.038430	-0.056792	0.033605	-0.009286	0.032775
	0.027046	-0.003320	0.009795	-0.014486	-0.002242	-0.023932
	0.006685	0.003095	0.033138	-0.005486	-0.052004	0.011889
	-0.005239					
y339	-0.040104	0.007650	0.017710	-0.001719	-0.031876	-0.021114
	-0.030741	0.005871	-0.020878	-0.004969	0.030794	-0.006448
	0.004637	-0.051087	0.025590	0.016262	-0.012044	-0.035231
	-0.043305	-0.012167	0.012764	-0.012919	0.003396	0.012948
	-0.011840	-0.042924	-0.009982	-0.005318	-0.019385	0.025910
	0.042315					
z339	-0.012425	0.027837	-0.003308	0.012011	0.040411	0.022214
	-0.027115	0.009422	0.061100	-0.001487	0.003626	-0.045642
	0.034035	0.005092	0.009438	-0.027132	-0.017946	-0.020389
	0.020816	0.007780	0.004838	0.006985	-0.006225	0.009355
	0.009194	0.004713	0.002879	0.010145	0.010005	0.027844
	-0.006424					
x340	-0.009734	-0.014420	0.009518	0.006776	0.011203	0.009416
	-0.026986	0.037801	-0.022284	0.048824	-0.011987	-0.013274
	0.012266	-0.036436	-0.049051	0.034920	-0.007338	0.034961
	0.031320	-0.006812	0.011307	-0.014861	-0.008019	-0.026845
	0.014416	0.002437	0.019095	-0.008247	-0.054718	0.019188
	-0.006234					

y340	-0.036958	0.009636	0.022618	-0.001710	-0.027683	-0.019840
	-0.035930	0.009167	-0.019333	0.001432	0.035502	-0.010785
	0.011044	-0.039332	0.035564	0.017077	-0.004600	-0.026164
	-0.027773	-0.007840	0.014606	-0.015959	0.001014	0.013050
	-0.012881	-0.047774	-0.012046	-0.010915	-0.018131	0.039157
	0.031823					
z340	-0.011048	0.029547	-0.002482	0.009722	0.043847	0.019563
	-0.032001	0.004415	0.060173	0.000043	0.007116	-0.049871
	0.031989	0.004058	0.012072	-0.023537	-0.017069	-0.016946
	0.017742	0.012366	0.003430	-0.007188	-0.001965	0.010824
	0.003642	0.003440	0.004923	0.007336	0.003447	0.026280
	-0.010071					
x341	-0.011924	-0.011553	0.010520	0.004693	0.013775	0.011543
	-0.026447	0.035953	-0.013528	0.040568	-0.013407	-0.009193
	0.013769	-0.031116	-0.051260	0.034290	-0.003074	0.041001
	0.029902	0.002219	0.004132	-0.012623	-0.010665	-0.030565
	0.015935	0.011037	0.020464	-0.004541	-0.043204	0.019810
	-0.007222					
y341	-0.038411	0.012231	0.027223	-0.000554	-0.027339	-0.017471
	-0.037419	0.011501	-0.011602	-0.003453	0.038681	-0.006664
	0.010121	-0.035117	0.033975	0.014917	-0.002854	-0.018134
	-0.021116	0.006959	0.016116	-0.004975	0.001678	0.003193
	-0.018012	-0.045339	0.003205	-0.001486	-0.012232	0.026671
	0.025879					
z341	-0.011433	0.032229	-0.001021	0.009555	0.046329	0.018217
	-0.034398	0.003540	0.062442	-0.004968	0.012401	-0.052192
	0.032000	-0.000885	0.014829	-0.017676	-0.017424	-0.009995
	0.016000	0.017113	0.003009	-0.007914	-0.005763	0.000100
	0.005320	0.003898	0.008056	0.011102	0.004786	0.019148
	-0.011684					
x342	-0.013950	-0.009654	0.009837	0.004588	0.015598	0.012055
	-0.025435	0.033871	-0.007203	0.038532	-0.016948	-0.007082
	0.014286	-0.026587	-0.052718	0.025832	-0.004377	0.039268
	0.027216	0.006616	-0.004792	-0.014591	-0.008359	-0.022847
	0.014201	0.015224	0.018952	-0.004152	-0.041275	0.025766
	-0.011642					
y342	-0.040639	0.011897	0.029012	0.002035	-0.027936	-0.015412
	-0.038510	0.013097	-0.009043	-0.004996	0.039026	-0.002732
	0.011397	-0.035698	0.028391	0.012826	-0.005657	-0.020055
	-0.014477	0.011699	0.012082	0.004579	0.002627	0.002831
	-0.020605	-0.042472	0.007766	0.000503	-0.010494	0.023131
	0.022762					

z342	-0.012480	0.032491	-0.001339	0.010837	0.048005	0.017484
	-0.036401	0.001799	0.061086	-0.006600	0.016767	-0.052974
	0.032331	-0.009104	0.013537	-0.012722	-0.020612	-0.009304
	0.015229	0.016593	0.000279	-0.008787	-0.006849	-0.001223
	0.008441	0.005719	0.006675	0.010614	0.002932	0.020632
	-0.010609					
x343	-0.016017	-0.008191	0.009087	0.005115	0.016562	0.012118
	-0.024150	0.032674	-0.002509	0.036850	-0.018745	-0.006894
	0.015817	-0.025112	-0.053346	0.020907	-0.004311	0.036861
	0.023943	0.008529	-0.014369	-0.016535	-0.009982	-0.014395
	0.014464	0.015344	0.014994	-0.002459	-0.038692	0.029447
	-0.015502					
y343	-0.041474	0.011155	0.030749	0.004559	-0.029026	-0.013576
	-0.039058	0.015355	-0.007296	-0.005565	0.040985	-0.001526
	0.012943	-0.036732	0.022932	0.011610	-0.005808	-0.022029
	-0.010450	0.013490	0.008771	0.008592	0.000995	0.005452
	-0.021439	-0.042635	0.009727	0.002108	-0.007632	0.021514
	0.017201					
z343	-0.012932	0.032474	-0.001394	0.011411	0.048856	0.016802
	-0.037782	0.000160	0.059043	-0.007893	0.020972	-0.054303
	0.032710	-0.015408	0.011772	-0.007982	-0.022920	-0.009660
	0.013713	0.015437	-0.000583	-0.011657	-0.008436	-0.001288
	0.011548	0.006612	0.003784	0.010001	0.002190	0.022760
	-0.010614					
x344	-0.017953	-0.006899	0.008843	0.005939	0.017746	0.011758
	-0.023665	0.031436	0.001116	0.036018	-0.021145	-0.008483
	0.017616	-0.023930	-0.052031	0.017909	-0.004071	0.033909
	0.022177	0.007159	-0.021809	-0.017094	-0.014090	-0.008186
	0.017208	0.015094	0.011227	-0.001170	-0.040594	0.031874
	-0.017469					
y344	-0.041315	0.010169	0.032530	0.007483	-0.028099	-0.012198
	-0.039836	0.017368	-0.005666	-0.004872	0.040436	-0.003402
	0.017634	-0.035169	0.021631	0.011511	-0.002398	-0.024127
	-0.004328	0.013940	0.008585	0.010848	-0.000718	0.009213
	-0.017943	-0.043014	0.009082	0.001640	-0.012367	0.021400
	0.013967					
z344	-0.013134	0.032226	-0.001441	0.011357	0.049475	0.016175
	-0.038690	-0.002188	0.056931	-0.009565	0.024640	-0.055062
	0.033200	-0.019517	0.009337	-0.003241	-0.024657	-0.011871
	0.010828	0.016065	-0.000358	-0.015122	-0.008178	-0.000467
	0.013463	0.007666	0.000258	0.009221	0.002930	0.025292
	-0.011769					

x345	-0.019971	-0.005514	0.008983	0.005781	0.018849	0.010828
	-0.022881	0.028710	0.003667	0.035384	-0.022619	-0.010295
	0.016554	-0.022846	-0.050202	0.015474	-0.004024	0.031185
	0.018306	0.005080	-0.027649	-0.016108	-0.017659	-0.007059
	0.016655	0.012018	0.008814	-0.000016	-0.039692	0.028794
	-0.019140					
y345	-0.040548	0.009233	0.034360	0.009206	-0.026189	-0.011389
	-0.039909	0.018838	-0.002907	-0.003867	0.041968	-0.007220
	0.022236	-0.035804	0.020584	0.011796	-0.001466	-0.030914
	-0.003380	0.014326	0.007773	0.010658	-0.001058	0.010604
	-0.021375	-0.045625	0.007596	-0.001179	-0.008585	0.019825
	0.005726					
z345	-0.013156	0.031779	-0.002029	0.010832	0.050073	0.015536
	-0.038988	-0.006654	0.052854	-0.011528	0.026915	-0.053379
	0.031388	-0.018168	0.007055	-0.001275	-0.023213	-0.011736
	0.008967	0.018827	0.002018	-0.018478	-0.003673	-0.001353
	0.015195	0.008166	-0.004983	0.007430	0.005521	0.025531
	-0.009403					
x346	-0.021968	-0.003842	0.009454	0.004787	0.020211	0.009749
	-0.021700	0.023175	0.004554	0.034596	-0.023719	-0.011461
	0.012455	-0.019878	-0.048502	0.013183	-0.002375	0.029824
	0.013596	0.003670	-0.031947	-0.011807	-0.019691	-0.010187
	0.015357	0.007742	0.007183	-0.000422	-0.038096	0.019785
	-0.017542					
y346	-0.039440	0.008633	0.036446	0.011023	-0.022371	-0.011576
	-0.040709	0.018052	-0.001996	-0.003543	0.044129	-0.008472
	0.025927	-0.034796	0.018742	0.011446	0.003014	-0.039198
	-0.001460	0.016860	0.009113	0.009263	0.001271	0.011038
	-0.021107	-0.043666	0.007651	-0.011849	-0.004703	0.018239
	0.001487					
z346	-0.012606	0.030891	-0.003297	0.008956	0.050551	0.014992
	-0.038044	-0.013182	0.047463	-0.013537	0.027857	-0.050975
	0.026910	-0.012872	0.004890	-0.002271	-0.021211	-0.009448
	0.005570	0.023392	0.005016	-0.019840	0.004297	-0.006133
	0.011435	0.005870	-0.012566	0.006547	0.013547	0.021336
	-0.005795					
x347	-0.024034	-0.001795	0.010001	0.003432	0.021451	0.008467
	-0.020512	0.014929	0.004205	0.033585	-0.024047	-0.012690
	0.006130	-0.015119	-0.047029	0.010517	-0.003050	0.027188
	0.007345	0.003293	-0.035675	-0.002874	-0.020973	-0.016413
	0.012805	0.001696	0.004865	0.000164	-0.033772	0.004169
	-0.014272					

y347	-0.037786	0.009364	0.036154	0.011869	-0.017265	-0.012417
	-0.042584	0.014332	-0.003634	-0.004733	0.045256	-0.009226
	0.024088	-0.031839	0.016303	0.009175	0.007688	-0.040181
	-0.006143	0.026196	0.008824	0.005947	0.003086	0.011458
	-0.023239	-0.044751	0.001113	-0.018422	0.005505	0.014606
	-0.002455					
z347	-0.011640	0.028544	-0.003766	0.006344	0.050277	0.014526
	-0.036017	-0.021223	0.040899	-0.014476	0.027677	-0.046817
	0.022826	-0.005065	0.000277	-0.004118	-0.019410	-0.013586
	0.003387	0.025966	0.008988	-0.019277	0.012310	-0.012577
	0.006803	0.008625	-0.018172	-0.000801	0.021462	0.018521
	-0.002440					
x348	-0.025297	0.000446	0.010481	0.002855	0.021731	0.006092
	-0.020629	0.005819	0.002034	0.032682	-0.023021	-0.013201
	-0.001617	-0.011441	-0.044301	0.006513	-0.006634	0.022914
	0.002934	0.002832	-0.037727	0.009482	-0.023378	-0.025741
	0.009021	-0.003550	0.004567	0.000758	-0.027825	-0.012367
	-0.010083					
y348	-0.035508	0.010108	0.033331	0.010318	-0.010397	-0.011549
	-0.042945	0.003526	-0.008080	-0.004181	0.042689	-0.010228
	0.019540	-0.021742	0.013023	0.002291	0.009682	-0.044815
	-0.013065	0.039809	0.010825	0.010361	0.009676	0.009251
	-0.026969	-0.045112	-0.011840	-0.025311	0.026523	0.005129
	-0.005993					
z348	-0.009954	0.025709	-0.003088	0.004096	0.046804	0.013419
	-0.033991	-0.026051	0.035189	-0.016967	0.028845	-0.039988
	0.019304	0.000777	-0.007970	-0.001984	-0.013371	-0.015743
	-0.000428	0.026682	0.010777	-0.024772	0.012460	-0.017294
	0.005045	0.012942	-0.018620	-0.010337	0.022793	0.024518
	-0.000521					
x349	-0.025379	0.001786	0.011282	0.002691	0.020464	0.002455
	-0.022142	0.000871	-0.000199	0.032407	-0.022913	-0.010141
	-0.010722	-0.013048	-0.039115	0.001670	-0.015212	0.021964
	0.002518	-0.001672	-0.036897	0.020672	-0.029295	-0.032394
	0.004826	-0.007591	0.009521	0.005048	-0.028557	-0.024750
	-0.003714					
y349	-0.032265	0.010844	0.028164	0.008856	-0.005110	-0.008374
	-0.043591	-0.008818	-0.011973	-0.003448	0.040431	-0.011895
	0.017262	-0.008096	0.007290	-0.002788	0.014614	-0.051212
	-0.017779	0.051803	0.013079	0.015042	0.019227	0.003851
	-0.028456	-0.043846	-0.024829	-0.034356	0.046891	0.000063
	-0.009491					

z349	-0.009663	0.022812	-0.000186	0.000131	0.040809	0.012751
	-0.030250	-0.023568	0.034479	-0.019385	0.025276	-0.029052
	0.015263	0.004725	-0.010870	0.001531	-0.017004	-0.014063
	-0.003255	0.023137	0.013811	-0.030439	0.007716	-0.011376
	0.004941	0.016427	-0.018055	-0.011159	0.020158	0.035247
	0.004459					
x350	-0.009380	0.010163	-0.008692	-0.006376	-0.003419	0.000080
	-0.006165	0.039337	-0.033195	-0.003451	0.025042	0.008355
	-0.022383	-0.019103	0.035259	-0.016415	-0.019219	-0.006097
	-0.026788	0.005334	-0.010001	-0.009669	0.028622	0.021343
	-0.020857	-0.000207	-0.064818	0.004369	-0.013902	0.022656
	-0.023549					
y350	0.001763	0.017616	-0.024881	0.001447	-0.015052	0.015240
	0.022759	0.012575	0.025364	-0.029648	0.005668	0.005799
	0.000497	-0.028817	0.007001	0.021745	0.035495	0.017806
	0.009987	0.012357	-0.009988	0.027290	-0.013635	-0.024249
	-0.030906	0.027473	0.019078	-0.022367	0.003228	0.008166
	-0.015751					
z350	-0.019377	0.015783	-0.024797	-0.043194	-0.025821	0.013293
	-0.003069	0.012110	-0.029156	-0.012116	-0.034017	-0.032676
	-0.005438	-0.002750	0.007094	0.014636	-0.001927	-0.013367
	-0.015323	0.035964	0.011758	0.016841	-0.023220	0.008474
	0.024720	-0.008568	-0.014722	0.015109	-0.020740	-0.018248
	-0.038351					
x351	-0.010456	0.015770	-0.004839	-0.004461	-0.001182	-0.006379
	-0.000454	0.037386	-0.019397	-0.005926	0.018798	0.009589
	-0.027778	-0.022464	0.040268	-0.014540	-0.025876	0.008924
	-0.029056	-0.011843	-0.014484	-0.023375	0.034672	0.014648
	-0.014986	-0.001240	-0.045933	0.013364	-0.013345	0.016407
	-0.009451					
y351	0.005148	0.022304	-0.020851	-0.001831	-0.015712	0.016634
	0.026639	0.010773	0.033683	-0.023646	0.003159	0.007339
	0.006594	-0.016489	0.004683	0.023040	0.041284	0.019374
	0.021794	0.008180	-0.002562	0.014462	-0.016961	-0.011325
	-0.035580	0.016357	0.026965	-0.038414	0.010161	0.016347
	-0.011983					
z351	-0.014324	0.015626	-0.026271	-0.047074	-0.028431	0.008437
	-0.005708	0.005509	-0.028970	-0.012171	-0.034110	-0.039570
	-0.008110	-0.000719	0.004920	0.017723	0.006034	0.000687
	-0.018948	0.030825	0.017186	0.025047	-0.006122	0.004089
	0.015218	-0.008580	0.007154	0.002028	-0.030587	-0.011062
	-0.056473					

x352	-0.011621	0.022379	-0.003789	-0.004544	0.000289	-0.011229
	0.003705	0.033095	-0.008020	-0.008054	0.012661	0.005526
	-0.032016	-0.024307	0.044192	-0.011410	-0.028247	0.018976
	-0.028712	-0.025505	-0.017889	-0.032243	0.042846	0.004820
	-0.006443	0.001059	-0.030965	0.018240	-0.014082	0.013803
	0.001380					
y352	0.007694	0.026504	-0.020036	-0.002347	-0.013676	0.019600
	0.025777	0.012213	0.035407	-0.018900	0.002799	0.006722
	0.005605	-0.005018	0.002791	0.025279	0.044855	0.020504
	0.026931	0.009294	-0.000772	0.010394	-0.018592	-0.001468
	-0.032216	0.007880	0.024824	-0.040503	0.004651	0.028952
	-0.021607					
z352	-0.011247	0.016110	-0.021814	-0.051648	-0.033272	0.000886
	-0.008226	-0.002662	-0.018070	-0.006627	-0.030604	-0.040799
	-0.002193	0.002900	0.001463	0.024247	0.012234	0.011732
	-0.014065	0.012084	0.018386	0.011605	0.010052	0.001836
	0.000137	-0.011358	0.028113	-0.021241	-0.023210	-0.017941
	-0.045372					
x353	-0.012624	0.027998	-0.006428	-0.005764	0.001621	-0.014126
	0.005996	0.028772	-0.002186	-0.008808	0.005800	-0.000451
	-0.032429	-0.025736	0.047854	-0.008685	-0.030737	0.024360
	-0.028265	-0.036512	-0.017163	-0.037525	0.046625	-0.002986
	0.000134	0.005266	-0.018305	0.022164	-0.013795	0.011934
	0.008061					
y353	0.010714	0.028752	-0.026238	-0.002140	-0.011539	0.021600
	0.022418	0.010356	0.028353	-0.017172	0.002406	-0.000097
	0.004163	-0.004459	-0.000225	0.028628	0.047297	0.019367
	0.025637	0.005346	0.006230	0.007307	-0.023917	0.005039
	-0.023728	0.007296	0.022085	-0.028576	-0.003630	0.036471
	-0.027518					
z353	-0.010559	0.018686	-0.014297	-0.054972	-0.037052	-0.003639
	-0.008606	-0.004498	-0.004957	-0.003820	-0.027802	-0.037307
	0.002582	0.011222	-0.002909	0.029148	0.015118	0.023726
	-0.007632	0.003587	0.013990	0.000801	0.024490	0.001335
	-0.012846	-0.019947	0.043430	-0.042958	-0.013858	-0.022162
	-0.032700					
x354	-0.013502	0.030907	-0.008286	-0.006764	0.002573	-0.016431
	0.008497	0.025670	0.003465	-0.009063	0.000868	-0.003461
	-0.031072	-0.027420	0.051059	-0.008210	-0.032499	0.026475
	-0.029019	-0.046220	-0.015257	-0.040943	0.045202	-0.007668
	0.001341	0.006824	-0.007820	0.023365	-0.011959	0.010301
	0.013376					

y354	0.013133	0.027664	-0.029827	-0.002193	-0.010710	0.023287
	0.022838	0.008216	0.023023	-0.018451	0.001551	-0.003170
	0.008401	-0.009106	-0.004269	0.028514	0.045688	0.014418
	0.021202	0.000080	0.011076	0.008522	-0.032905	0.010540
	-0.025414	0.004697	0.017621	-0.017857	-0.004495	0.038136
	-0.019707					
z354	-0.010584	0.022374	-0.008903	-0.056921	-0.038964	-0.007030
	-0.009843	-0.006649	0.003505	-0.002216	-0.025156	-0.034712
	0.004226	0.017429	-0.006409	0.031775	0.016859	0.034992
	-0.000592	-0.001758	0.011763	-0.005781	0.037148	-0.000574
	-0.015823	-0.023770	0.051804	-0.053645	-0.009450	-0.019740
	-0.029541					
x355	-0.014006	0.032149	-0.008976	-0.005758	0.003570	-0.018147
	0.010956	0.024855	0.008870	-0.010015	-0.002104	-0.002899
	-0.031146	-0.030533	0.051625	-0.011328	-0.031701	0.024921
	-0.026645	-0.048594	-0.009880	-0.041326	0.039245	-0.006742
	0.003858	0.010477	-0.002794	0.031990	-0.010127	0.012336
	0.020977					
y355	0.015417	0.027036	-0.030462	0.000757	-0.010518	0.024965
	0.024383	0.009331	0.021043	-0.022176	0.003321	-0.000432
	0.013358	-0.018295	-0.006255	0.023815	0.048703	0.011107
	0.025936	0.002770	0.017148	0.007966	-0.041785	0.019127
	-0.020881	0.011374	0.007485	0.000171	0.000101	0.040856
	0.000069					
z355	-0.011166	0.024631	-0.004883	-0.056462	-0.038607	-0.009491
	-0.010187	-0.009161	0.007879	-0.005076	-0.023717	-0.029902
	0.004880	0.019928	-0.013381	0.031978	0.013899	0.038303
	0.002440	-0.001274	0.011123	-0.004123	0.041801	-0.001800
	-0.019319	-0.021755	0.050769	-0.049582	-0.008726	-0.014844
	-0.026383					
x356	-0.014248	0.032292	-0.009952	-0.004446	0.005538	-0.019316
	0.013207	0.023974	0.012821	-0.010565	-0.004620	-0.001904
	-0.033475	-0.030945	0.048867	-0.016182	-0.028444	0.019870
	-0.026444	-0.044778	-0.002988	-0.035806	0.030067	-0.003474
	0.004545	0.014172	0.000104	0.034396	-0.007858	0.015935
	0.022399					
y356	0.017029	0.025993	-0.030769	0.004298	-0.009300	0.027093
	0.026211	0.010706	0.018751	-0.024300	0.003806	0.004079
	0.013901	-0.020321	-0.010399	0.019044	0.051211	0.008786
	0.027287	0.007633	0.020824	0.015729	-0.047042	0.023863
	-0.018254	0.017104	-0.005776	0.005492	0.010740	0.040810
	0.011249					

z356	-0.011897	0.026009	-0.001465	-0.052855	-0.036852	-0.011487
	-0.008513	-0.011369	0.010415	-0.013200	-0.023442	-0.022968
	0.004113	0.017447	-0.023754	0.029525	0.007563	0.035399
	0.005028	0.005535	0.013471	0.001006	0.041583	0.003134
	-0.019941	-0.011877	0.042255	-0.029384	-0.006535	-0.006509
	-0.014264					
x357	-0.014363	0.032327	-0.012915	-0.004891	0.007771	-0.019200
	0.014303	0.021709	0.015458	-0.009979	-0.006126	-0.002359
	-0.037923	-0.029547	0.046026	-0.017850	-0.023558	0.014840
	-0.024834	-0.037196	0.003548	-0.030366	0.019347	0.000293
	-0.000593	0.012712	0.004207	0.028731	-0.007411	0.018503
	0.017006					
y357	0.018723	0.025450	-0.032247	0.005300	-0.009433	0.029513
	0.026788	0.012017	0.016505	-0.026926	0.004506	0.007086
	0.008739	-0.020726	-0.011332	0.019086	0.051724	0.012106
	0.029149	0.014021	0.020465	0.020034	-0.052443	0.026765
	-0.019402	0.014402	-0.017923	0.012003	0.017923	0.036134
	0.022533					
z357	-0.012814	0.027981	0.001143	-0.048694	-0.034578	-0.012932
	-0.005617	-0.014285	0.011367	-0.024378	-0.022977	-0.015323
	0.000017	0.012415	-0.037150	0.026240	0.000250	0.030702
	0.007432	0.017543	0.016097	0.007164	0.040311	0.009461
	-0.019592	-0.000567	0.031394	-0.000779	-0.003577	0.004495
	-0.000358					
x358	-0.013497	0.030455	-0.018288	-0.006720	0.010981	-0.018036
	0.013719	0.020597	0.015461	-0.002196	-0.007897	-0.003558
	-0.036035	-0.020704	0.052349	-0.017818	-0.018384	0.012677
	-0.026249	-0.028705	0.009494	-0.024285	0.005743	0.001440
	-0.006690	0.010226	0.013890	0.002851	-0.006873	0.012175
	-0.000207					
y358	0.020658	0.023875	-0.034934	0.005484	-0.009041	0.031586
	0.024686	0.016660	0.014244	-0.021513	0.000721	0.008698
	0.009414	-0.012256	-0.000880	0.019744	0.045693	0.024466
	0.028241	0.015581	0.019324	0.019950	-0.056468	0.021942
	-0.021114	0.011067	-0.028062	0.003279	0.029656	0.018526
	0.027703					
z358	-0.013609	0.029571	0.002269	-0.043598	-0.030571	-0.014449
	-0.003087	-0.015080	0.009078	-0.032703	-0.026008	-0.007299
	0.002534	0.010979	-0.043070	0.021267	-0.012962	0.028073
	0.006583	0.026402	0.018638	0.011761	0.039384	0.012039
	-0.013703	0.013372	0.024098	0.023478	0.002139	0.010148
	0.006951					

x359	-0.010527	0.026635	-0.021555	-0.010190	0.014792	-0.017888
	0.010411	0.020910	0.014701	0.010624	-0.008362	-0.002982
	-0.038785	-0.005619	0.066613	-0.017877	-0.010761	0.013879
	-0.027545	-0.018675	0.016211	-0.019552	-0.009313	0.004425
	-0.016163	0.000328	0.020995	-0.042473	-0.000966	0.002371
	-0.018463					
y359	0.022680	0.022031	-0.035936	0.004605	-0.007932	0.032709
	0.020693	0.019346	0.014321	-0.013013	-0.003462	0.009557
	0.008257	-0.002273	0.011010	0.019995	0.037719	0.039980
	0.022777	0.013415	0.018018	0.014268	-0.058352	0.014790
	-0.022038	0.004359	-0.039550	-0.014405	0.041358	-0.007198
	0.030544					
z359	-0.012904	0.031131	0.005603	-0.039492	-0.024214	-0.017939
	-0.003051	-0.016435	0.007612	-0.040375	-0.024746	0.002151
	0.000995	0.013064	-0.041504	0.019049	-0.021591	0.032051
	0.008151	0.031388	0.019564	0.014446	0.038572	0.014994
	-0.005837	0.017762	0.009119	0.023225	0.014644	0.009823
	0.017754					
x360	0.010031	-0.012676	-0.009948	-0.017843	-0.005243	0.045029
	-0.009351	0.047842	-0.013148	0.007610	-0.005719	0.011644
	0.007454	-0.009098	0.024597	-0.010552	0.013996	-0.043081
	-0.015482	0.021594	-0.021948	-0.011623	0.010993	-0.011259
	-0.027388	0.017515	-0.017369	0.018683	-0.017965	0.014018
	0.015066					
y360	-0.011276	0.023841	0.020805	0.015399	-0.012069	0.009577
	0.002434	-0.016809	0.036827	-0.003649	-0.026741	0.017495
	-0.010355	0.013365	0.020497	0.000792	-0.011274	0.035369
	-0.014150	-0.031768	-0.001055	0.018288	0.004477	0.018951
	-0.024598	-0.018063	0.032209	-0.019745	0.016736	0.015772
	-0.016928					
z360	0.002002	-0.025508	-0.025144	-0.019396	-0.009521	0.021615
	-0.027828	-0.006035	-0.045267	-0.014186	0.019104	0.010479
	-0.028499	-0.013385	0.013157	-0.006958	0.005289	-0.027052
	0.014772	0.005571	0.031778	-0.016076	0.010739	-0.008362
	0.011746	-0.008089	-0.003994	0.019812	0.015551	-0.009486
	0.003589					
x361	0.009673	-0.000946	-0.009641	-0.015304	-0.002280	0.038071
	-0.002346	0.039030	-0.003801	-0.003934	-0.014152	0.012048
	0.010686	-0.008166	0.022720	-0.007723	0.006377	-0.022795
	-0.017747	0.013706	-0.009873	-0.011773	0.000677	-0.003071
	-0.023980	0.003504	-0.001516	0.002877	-0.013867	0.021429
	0.006528					

y361	-0.009653	0.021372	0.021369	0.017233	-0.013839	0.010383
	-0.002511	-0.012957	0.038781	0.004058	-0.028431	0.013064
	-0.012751	0.013382	0.013033	-0.007728	-0.009915	0.032403
	-0.009181	-0.024858	-0.004405	0.016961	0.000673	0.014517
	-0.015930	-0.017715	0.023906	-0.003464	0.003791	0.020310
	-0.015992					
z361	0.006301	-0.026585	-0.019813	-0.014156	-0.009451	0.016543
	-0.032926	-0.008144	-0.045189	-0.008653	0.027163	0.008245
	-0.026621	-0.016365	0.017678	-0.004581	0.006851	-0.013001
	0.008855	-0.002151	0.028784	-0.017267	-0.003429	-0.019640
	0.023547	0.000109	-0.005615	0.017717	0.001508	0.000880
	0.017982					
x362	0.008682	0.009675	-0.011791	-0.014096	0.000295	0.032739
	0.005967	0.033292	0.003377	-0.013160	-0.020998	0.010532
	0.016301	-0.006224	0.020429	-0.002120	0.001702	-0.006261
	-0.018460	0.006201	-0.000195	-0.007009	-0.010642	0.002428
	-0.020810	-0.005230	0.012208	-0.011618	-0.006071	0.025350
	-0.007167					
y362	-0.008818	0.019148	0.021464	0.018789	-0.014841	0.010882
	-0.006770	-0.011261	0.039982	0.009130	-0.031817	0.009904
	-0.013967	0.012892	0.007128	-0.015077	-0.011410	0.029156
	-0.005030	-0.018324	-0.007619	0.011610	0.002028	0.010823
	-0.010184	-0.017824	0.019346	0.007128	-0.007715	0.018936
	-0.018064					
z362	0.007422	-0.025629	-0.016029	-0.012958	-0.013543	0.011534
	-0.033550	-0.013187	-0.043896	-0.007949	0.023498	0.007588
	-0.020014	-0.020700	0.021584	-0.002991	-0.000781	0.002933
	-0.002510	-0.013538	0.025783	-0.025296	-0.005111	-0.030287
	0.022672	0.012551	-0.001242	0.007272	-0.006403	0.000423
	0.017179					
x363	0.007420	0.017485	-0.014308	-0.013786	0.003009	0.028135
	0.010589	0.031851	0.005932	-0.018191	-0.025736	0.010526
	0.018387	-0.007500	0.018807	0.002476	-0.001927	0.007175
	-0.021593	-0.001286	0.006148	-0.002866	-0.019034	0.005352
	-0.017712	-0.008442	0.021607	-0.021121	-0.004332	0.023214
	-0.015700					
y363	-0.008512	0.017780	0.021370	0.020235	-0.014404	0.012287
	-0.008549	-0.010427	0.041739	0.009662	-0.033701	0.007220
	-0.012683	0.015891	0.002748	-0.018494	-0.011065	0.025782
	0.001777	-0.009539	-0.011111	0.008710	0.002845	0.008616
	-0.006224	-0.020504	0.014985	0.013040	-0.011951	0.021624
	-0.021879					

z363	0.006663	-0.022759	-0.012643	-0.013209	-0.017342	0.010295
	-0.030018	-0.017411	-0.039501	-0.013608	0.018677	0.005242
	-0.007728	-0.016677	0.025026	-0.000692	-0.005194	0.013637
	-0.005861	-0.018728	0.023494	-0.026156	-0.006559	-0.038771
	0.023214	0.018542	0.002810	-0.005370	0.001388	0.008524
	0.005365					
x364	0.006511	0.022974	-0.015196	-0.014387	0.005193	0.025433
	0.014042	0.031377	0.005809	-0.018799	-0.028352	0.013808
	0.021238	-0.009159	0.020504	0.001302	-0.005376	0.015770
	-0.020148	-0.005371	0.009176	0.005417	-0.024959	0.005188
	-0.016967	-0.007487	0.027370	-0.024630	0.001576	0.022585
	-0.019235					
y364	-0.008373	0.016686	0.020266	0.021148	-0.012623	0.012865
	-0.009755	-0.011067	0.041991	0.008874	-0.033220	0.003680
	-0.012739	0.017863	-0.001141	-0.016110	-0.009762	0.023401
	0.004932	-0.002645	-0.012112	0.004605	0.003376	0.007467
	-0.003354	-0.024936	0.012671	0.015038	-0.018869	0.021751
	-0.026365					
z364	0.005242	-0.019624	-0.011046	-0.013740	-0.019443	0.008811
	-0.028438	-0.020144	-0.035458	-0.020422	0.013979	0.001134
	-0.000823	-0.014307	0.023947	0.004131	-0.007895	0.022187
	-0.011351	-0.023275	0.025282	-0.029908	-0.005374	-0.043316
	0.023160	0.019620	0.006616	-0.018477	0.003040	0.012365
	-0.006234					
x365	0.006373	0.026614	-0.016177	-0.015347	0.006264	0.023257
	0.014913	0.030277	0.001353	-0.016846	-0.030410	0.019548
	0.022684	-0.009844	0.022541	-0.001418	-0.008823	0.018935
	-0.015877	-0.004407	0.010070	0.010710	-0.022303	0.003635
	-0.016313	-0.004563	0.036536	-0.017605	0.005185	0.012530
	-0.018785					
y365	-0.008273	0.016152	0.019261	0.021699	-0.010194	0.013732
	-0.009763	-0.011990	0.041876	0.007926	-0.030566	0.000597
	-0.012136	0.019654	-0.002303	-0.012762	-0.007446	0.023218
	0.005868	0.001620	-0.012485	0.002884	0.001007	0.006426
	-0.001172	-0.028436	0.008959	0.013333	-0.022769	0.021143
	-0.030227					
z365	0.004089	-0.015330	-0.008402	-0.013951	-0.020513	0.006985
	-0.029007	-0.022558	-0.031909	-0.024247	0.012278	0.000176
	0.001879	-0.010479	0.020652	0.005821	-0.007857	0.027868
	-0.012726	-0.021627	0.027592	-0.030105	-0.004106	-0.043950
	0.021071	0.018877	0.005906	-0.023293	0.009603	0.016217
	-0.013164					

x366	0.006164	0.028345	-0.016008	-0.016542	0.008273	0.022418
	0.015339	0.030596	-0.000825	-0.012102	-0.026659	0.024655
	0.019965	-0.008851	0.025242	-0.003708	-0.007075	0.018870
	-0.008631	-0.000367	0.010692	0.016243	-0.019471	0.005587
	-0.015738	-0.009293	0.036286	-0.008076	0.011050	0.011464
	-0.013736					
y366	-0.008023	0.016334	0.018652	0.021844	-0.007403	0.014346
	-0.010162	-0.014001	0.039212	0.006527	-0.027433	-0.000373
	-0.011477	0.020933	-0.002271	-0.009367	-0.004710	0.023290
	0.005889	0.005182	-0.012688	0.002847	0.000354	0.003538
	-0.002701	-0.028626	0.007407	0.010600	-0.023786	0.014341
	-0.034431					
z366	0.003292	-0.009501	-0.004634	-0.013824	-0.021397	0.003930
	-0.032074	-0.025895	-0.029602	-0.025803	0.011537	0.003638
	0.000962	-0.004626	0.014347	0.004658	-0.007206	0.030539
	-0.009941	-0.014065	0.030067	-0.027444	0.001918	-0.041455
	0.015355	0.018211	0.005036	-0.017966	0.021371	0.015333
	-0.015967					
x367	0.004502	0.028947	-0.016119	-0.017527	0.012029	0.020543
	0.014932	0.030770	-0.001476	-0.005449	-0.024184	0.030285
	0.012926	-0.009828	0.025091	-0.006129	-0.007102	0.014223
	-0.005069	0.000685	0.008813	0.019507	-0.015258	0.007825
	-0.022795	-0.008896	0.034841	-0.006786	0.014159	0.005526
	-0.005701					
y367	-0.007171	0.016920	0.018438	0.021442	-0.004356	0.015441
	-0.010516	-0.016606	0.034093	0.004815	-0.021954	-0.000880
	-0.010128	0.022777	-0.000265	-0.004610	-0.000465	0.023705
	0.006133	0.010350	-0.012545	0.004653	0.000477	0.000191
	-0.003425	-0.029760	0.006740	0.009754	-0.020568	0.007566
	-0.039658					
z367	0.002375	-0.002817	-0.000575	-0.013358	-0.021878	-0.000287
	-0.035942	-0.029445	-0.025326	-0.027430	0.010682	0.008012
	-0.002867	0.000529	0.004977	0.000595	-0.006969	0.032279
	-0.005769	-0.005148	0.032919	-0.024437	0.011288	-0.036009
	0.005924	0.016708	0.003269	-0.009790	0.033301	0.012003
	-0.015370					
x368	0.003638	0.027605	-0.018072	-0.018478	0.014742	0.019352
	0.017341	0.032706	-0.004110	0.003774	-0.020146	0.026775
	0.006991	-0.008603	0.031444	-0.003641	-0.003579	0.015127
	-0.004020	-0.002397	0.002337	0.022028	-0.015224	0.011549
	-0.021062	-0.011172	0.027974	-0.012389	0.011340	0.009536
	0.003668					

y368	-0.006319	0.018085	0.017960	0.020605	-0.000228	0.016528
	-0.011449	-0.020007	0.026875	0.002201	-0.015173	0.001122
	-0.007933	0.024299	0.003045	0.000198	0.003923	0.022528
	0.006672	0.015324	-0.011656	0.007713	0.002467	-0.003382
	-0.007866	-0.029438	0.007367	0.009429	-0.013736	-0.004431
	-0.043474					
z368	0.001311	0.005360	0.003856	-0.011962	-0.021999	-0.006413
	-0.038883	-0.031532	-0.019143	-0.029021	0.010803	0.010036
	-0.008273	0.006642	-0.005854	-0.003191	-0.004670	0.035661
	-0.000236	0.003810	0.034427	-0.020495	0.020601	-0.026006
	-0.004832	0.010286	-0.003729	-0.001398	0.044661	0.014178
	-0.011641					
x369	0.001468	0.023898	-0.021021	-0.022188	0.019689	0.018311
	0.021384	0.036774	-0.009892	0.013475	-0.021752	0.024971
	-0.002801	-0.009265	0.041194	0.000943	-0.006310	0.012986
	-0.005869	-0.011031	0.001349	0.024663	-0.017243	0.018119
	-0.008349	-0.007290	0.030438	-0.022449	-0.000037	0.005522
	0.013163					
y369	-0.005415	0.019525	0.016733	0.018920	0.003310	0.017549
	-0.012023	-0.024108	0.018615	-0.001616	-0.005547	0.003658
	-0.004382	0.025322	0.008649	0.005946	0.008128	0.021747
	0.007867	0.019824	-0.012122	0.011145	0.005407	-0.007670
	-0.014961	-0.027442	0.008498	0.010838	-0.006248	-0.021325
	-0.044808					
z369	-0.001401	0.012777	0.007685	-0.012754	-0.019420	-0.014282
	-0.038444	-0.029925	-0.014339	-0.029787	0.005948	0.014548
	-0.017574	0.008730	-0.014941	-0.005527	-0.006581	0.036781
	0.006460	0.006238	0.037584	-0.015583	0.029941	-0.011350
	-0.008417	0.003448	-0.004555	0.002999	0.047508	0.011120
	-0.001613					
x370	-0.013609	-0.005695	0.009929	-0.004704	-0.007245	0.038294
	-0.060065	0.012174	0.010022	-0.031244	-0.001274	0.009212
	0.006307	0.006369	-0.015344	0.011606	-0.003858	-0.020759
	0.015448	-0.011373	0.006454	-0.008279	0.027629	-0.019927
	0.018528	-0.008458	-0.015006	0.028625	0.014442	0.007833
	0.052772					
y370	-0.015329	0.009187	0.009383	0.011571	-0.002019	-0.018500
	-0.016755	-0.018859	0.008555	0.011390	-0.002726	0.021499
	-0.008731	0.028338	0.016968	0.017700	0.015206	0.002171
	0.000089	-0.046714	-0.007310	0.022678	-0.018050	0.010140
	-0.007828	0.005318	0.026974	0.027856	-0.027755	-0.017192
	-0.029331					

z370	-0.001293	-0.012663	-0.040486	-0.034126	0.010840	0.002421
	0.007632	-0.004195	-0.021918	-0.008003	0.021752	-0.002865
	-0.052395	-0.042388	-0.013889	-0.024255	0.015303	-0.016676
	0.000120	0.023015	0.051688	0.022378	0.008468	-0.012989
	-0.046906	-0.024335	-0.023073	-0.034713	0.021774	0.004971
	0.019441					
x371	-0.015654	-0.004657	0.013766	-0.007367	0.000952	0.033621
	-0.058878	0.010416	0.015404	-0.029351	0.002358	0.021457
	0.000361	0.004288	-0.020003	-0.000599	0.000966	-0.022529
	0.030496	-0.011518	0.010754	0.000289	0.025592	-0.018056
	0.015806	-0.023972	-0.013065	0.013000	0.022305	0.014659
	0.044370					
y371	-0.013850	0.005136	0.007464	0.015005	-0.006182	-0.014015
	-0.020657	-0.026426	0.007309	0.016262	-0.002808	0.012495
	-0.005668	0.030943	0.014800	0.022394	0.008193	0.005959
	-0.005956	-0.035894	-0.001239	0.021549	-0.007500	0.005027
	-0.007939	0.008642	0.024958	0.024611	-0.021601	-0.002459
	-0.029107					
z371	-0.000161	-0.006281	-0.031515	-0.027621	0.016028	-0.004178
	0.003013	-0.004199	-0.020955	-0.000072	0.022291	0.008718
	-0.034393	-0.029529	-0.012783	-0.023714	0.007323	-0.009460
	-0.000205	0.023698	0.036678	0.015078	-0.003976	-0.020662
	-0.023977	-0.008111	-0.019041	-0.025314	-0.004005	0.006545
	0.004796					
x372	-0.017022	-0.002514	0.017422	-0.010027	0.007803	0.032567
	-0.055213	0.008060	0.023053	-0.025787	0.004451	0.036897
	-0.001812	-0.002905	-0.017792	-0.004595	-0.005251	-0.020111
	0.032028	-0.018572	0.009906	-0.000557	0.023599	-0.014488
	0.021034	-0.030251	-0.013464	0.003708	0.029955	0.012690
	0.045218					
y372	-0.012526	0.004032	0.008880	0.017771	-0.009430	-0.005931
	-0.021375	-0.033795	0.012716	0.023089	0.002363	0.012654
	-0.006584	0.029615	0.016774	0.032119	-0.005513	0.012830
	-0.014826	-0.026554	-0.001381	0.010941	0.002346	0.007364
	-0.002569	0.019686	0.018165	0.027519	-0.013273	-0.001081
	-0.018692					
z372	0.000605	-0.001079	-0.022523	-0.025497	0.019389	-0.007932
	0.003419	-0.006792	-0.018363	0.010467	0.020388	0.022511
	-0.013855	-0.026684	-0.005236	-0.017422	-0.005076	-0.003193
	-0.008953	0.018615	0.022766	0.003562	-0.014429	-0.026920
	-0.000072	0.008958	-0.017486	-0.015553	-0.022461	0.008508
	-0.003585					

x373	-0.018872	-0.000700	0.017482	-0.013873	0.013035	0.029759
	-0.050111	0.006380	0.026179	-0.026987	0.003107	0.046409
	-0.004201	-0.009670	-0.013646	-0.010066	-0.003956	-0.018397
	0.038802	-0.024527	0.011337	0.003005	0.019349	-0.017960
	0.020058	-0.035686	-0.013666	-0.004706	0.031721	0.014224
	0.037899					
y373	-0.011372	0.003787	0.006769	0.018719	-0.014016	0.001651
	-0.020837	-0.038829	0.014219	0.020514	0.003824	0.007881
	-0.008038	0.029502	0.019144	0.039505	-0.006200	0.017647
	-0.013753	-0.022251	0.002832	0.006809	0.014400	0.003503
	-0.005950	0.026492	0.004493	0.027158	-0.010237	0.007545
	-0.011801					
z373	0.000547	0.002773	-0.016978	-0.024929	0.021198	-0.012638
	0.003895	-0.007547	-0.019897	0.015360	0.015741	0.030686
	0.004135	-0.025051	0.001326	-0.012623	-0.010113	0.000888
	-0.014112	0.015123	0.013676	-0.003708	-0.023192	-0.037392
	0.015670	0.022641	-0.017132	-0.008016	-0.039250	0.012507
	-0.015502					
x374	-0.020868	0.000561	0.017160	-0.017164	0.018854	0.024995
	-0.046854	0.004947	0.027030	-0.025282	0.002886	0.053130
	-0.008369	-0.015781	-0.011749	-0.015204	-0.001388	-0.020967
	0.040663	-0.028564	0.009363	0.005332	0.013463	-0.021505
	0.020540	-0.037042	-0.010512	-0.007131	0.028442	0.015058
	0.025990					
y374	-0.011152	0.003513	0.006633	0.019178	-0.015276	0.004379
	-0.023215	-0.043267	0.014603	0.020556	0.006981	0.003818
	-0.010593	0.027915	0.015888	0.045143	-0.002402	0.015484
	-0.016868	-0.017115	0.001410	-0.000658	0.021587	-0.000676
	-0.001961	0.032634	-0.002234	0.033389	-0.010110	0.013175
	-0.015779					
z374	0.000471	0.005571	-0.012822	-0.024822	0.022931	-0.017338
	0.003389	-0.007534	-0.022569	0.018427	0.012484	0.035929
	0.017391	-0.025342	0.004366	-0.010930	-0.010287	0.002082
	-0.019755	0.014720	0.008114	-0.009136	-0.029618	-0.045708
	0.026162	0.030700	-0.014423	-0.002148	-0.051222	0.016848
	-0.024078					
x375	-0.022431	0.001875	0.016387	-0.019198	0.023636	0.021138
	-0.044061	0.004093	0.027312	-0.023269	0.001848	0.057753
	-0.012786	-0.017812	-0.009265	-0.018850	-0.002569	-0.020498
	0.041162	-0.033599	0.007295	0.007878	0.008011	-0.020212
	0.017818	-0.038329	-0.008177	-0.009805	0.026663	0.011490
	0.018586					

y375	-0.011925	0.003075	0.007632	0.020292	-0.014805	0.004312
	-0.025088	-0.045527	0.014782	0.020284	0.005710	-0.000246
	-0.011317	0.022973	0.013331	0.044329	0.003298	0.014078
	-0.019232	-0.012794	0.001837	-0.007688	0.023342	0.000404
	0.002524	0.033032	-0.004767	0.036824	-0.017943	0.011784
	-0.027759					
z375	0.001023	0.008148	-0.010358	-0.024612	0.023620	-0.019682
	0.002533	-0.006674	-0.024810	0.019804	0.011818	0.039985
	0.025880	-0.020228	0.008658	-0.008499	-0.011450	0.004617
	-0.021772	0.016012	0.006117	-0.010307	-0.032657	-0.050487
	0.028907	0.032965	-0.012618	0.000282	-0.048564	0.019056
	-0.024464					
x376	-0.023184	0.004097	0.015029	-0.020349	0.027172	0.017935
	-0.041516	0.003269	0.027283	-0.021118	0.001872	0.061480
	-0.016775	-0.013364	-0.005666	-0.018956	-0.006721	-0.017886
	0.040221	-0.038418	0.005231	0.011654	0.003162	-0.015724
	0.013381	-0.040631	-0.007858	-0.013704	0.029953	0.004793
	0.018604					
y376	-0.012409	0.003713	0.009192	0.021326	-0.013645	0.005022
	-0.028074	-0.046673	0.015534	0.020058	0.005206	-0.001649
	-0.008773	0.022190	0.011416	0.043536	0.010813	0.013556
	-0.019129	-0.009372	0.004508	-0.014058	0.023885	0.002584
	0.009616	0.027889	-0.009967	0.034096	-0.023737	0.006541
	-0.030713					
z376	0.002577	0.011021	-0.009197	-0.024267	0.023218	-0.020414
	0.001330	-0.006377	-0.026646	0.019342	0.014131	0.043370
	0.029385	-0.010299	0.014019	-0.005530	-0.013704	0.008970
	-0.022210	0.018337	0.006041	-0.008083	-0.032953	-0.051931
	0.024181	0.032738	-0.010595	0.001921	-0.032824	0.018914
	-0.019577					
x377	-0.023383	0.007479	0.012505	-0.019653	0.030149	0.013732
	-0.038297	0.002457	0.025921	-0.018424	0.001307	0.063820
	-0.020202	-0.003860	-0.000042	-0.017741	-0.013852	-0.014010
	0.034742	-0.042339	0.002246	0.018567	-0.005273	-0.011223
	0.008323	-0.039767	-0.006277	-0.014421	0.030004	-0.004220
	0.021013					
y377	-0.012269	0.005804	0.012532	0.023701	-0.011943	0.007003
	-0.031551	-0.047295	0.017815	0.017858	0.007046	0.004027
	-0.008854	0.027157	0.011054	0.042185	0.014586	0.011341
	-0.019776	-0.006130	0.011998	-0.017253	0.018692	-0.003562
	0.011304	0.027252	-0.015949	0.032672	-0.020791	0.002311
	-0.024207					

z377	0.004468	0.014188	-0.010802	-0.024333	0.022643	-0.020841
	-0.000389	-0.005671	-0.029017	0.016959	0.014776	0.041800
	0.032576	-0.000822	0.019085	-0.005370	-0.010784	0.013621
	-0.017915	0.019386	0.007796	-0.005496	-0.028505	-0.042860
	0.019624	0.025456	-0.008617	0.003274	-0.018846	0.015374
	-0.015371					
x378	-0.022746	0.011981	0.008536	-0.017462	0.031752	0.009222
	-0.034369	0.000643	0.023170	-0.015024	-0.000430	0.062086
	-0.021719	0.010070	0.007294	-0.014590	-0.022425	-0.006701
	0.024133	-0.045912	-0.001858	0.027512	-0.016080	-0.006328
	0.006513	-0.034042	-0.002706	-0.011957	0.026097	-0.015364
	0.024099					
y378	-0.011665	0.010151	0.015493	0.025681	-0.006712	0.009455
	-0.037064	-0.046140	0.021375	0.014565	0.012929	0.015866
	-0.009671	0.038032	0.014097	0.038257	0.017546	0.004926
	-0.017544	-0.002003	0.020298	-0.019937	0.007761	-0.007155
	0.005155	0.021995	-0.024122	0.030283	-0.016647	-0.004474
	-0.014260					
z378	0.006107	0.017185	-0.014089	-0.023454	0.019243	-0.022223
	-0.000356	-0.006574	-0.032275	0.012105	0.010383	0.032793
	0.036303	0.006580	0.019472	-0.007456	-0.004747	0.019575
	-0.015084	0.019285	0.014868	0.000514	-0.016091	-0.034177
	0.017489	0.016624	-0.004068	0.004122	-0.001677	0.010401
	-0.010232					
x379	-0.022080	0.016089	0.006561	-0.015730	0.032501	0.005407
	-0.031286	-0.001179	0.021913	-0.010886	-0.002252	0.057912
	-0.026610	0.020065	0.009445	-0.009476	-0.027417	0.000547
	0.014201	-0.049535	-0.006658	0.037521	-0.020553	-0.002363
	0.000244	-0.025269	-0.005084	-0.011913	0.015112	-0.012796
	0.025496					
y379	-0.010475	0.015411	0.015852	0.027568	0.000796	0.010558
	-0.041581	-0.045653	0.024233	0.011531	0.018290	0.027550
	-0.007585	0.050652	0.019152	0.032101	0.020422	-0.002736
	-0.021073	0.002751	0.026949	-0.022255	-0.003386	-0.010702
	-0.001948	0.011625	-0.030264	0.025331	-0.011410	-0.013650
	-0.007636					
z379	0.005455	0.018307	-0.013050	-0.027227	0.016748	-0.026709
	0.001693	-0.005634	-0.032105	0.008146	0.005796	0.022849
	0.031966	0.002270	0.006818	-0.009428	0.005374	0.022849
	-0.004399	0.016800	0.022151	0.008229	0.012879	-0.023738
	0.008608	0.002138	-0.012189	0.000895	0.003942	0.020371
	0.003354					

x380	0.009080	-0.016068	-0.052395	0.026285	-0.023683	-0.010243
	0.013780	-0.028082	0.050057	0.017105	0.013480	0.020773
	0.018712	0.063068	0.029196	-0.085359	0.029237	-0.001918
	-0.067312	0.072391	0.021932	0.097948	0.004370	-0.054176
	-0.135779	-0.006143	-0.050832	-0.025917	0.044585	0.064774
	0.000265					
y380	-0.014048	0.006231	0.010013	0.084934	-0.011285	-0.025089
	0.031691	-0.000352	-0.068363	0.029262	-0.047853	0.038376
	0.032485	0.021312	-0.028369	0.004542	0.024947	0.026579
	-0.101480	-0.010229	0.021949	0.104663	-0.038773	-0.153963
	-0.015705	0.076933	-0.064842	0.008639	0.065232	0.034254
	0.061934					
z380	0.019317	0.029737	-0.009587	0.014457	-0.030343	0.028447
	-0.016379	0.029416	-0.086492	-0.052251	-0.018127	-0.010625
	-0.070734	0.059204	0.055094	0.009792	0.043231	0.043933
	0.080161	0.027143	0.030460	0.057221	-0.175868	-0.066323
	0.078503	-0.060746	-0.029376	0.072091	0.022649	-0.145288
	0.031640					
x381	0.000602	-0.009898	-0.039364	0.019219	-0.006150	-0.012707
	0.029113	-0.009794	0.033623	0.029835	0.005740	-0.000925
	0.026753	0.052568	-0.027404	-0.070007	0.055425	0.043241
	-0.057296	0.019847	0.030878	0.024520	0.027559	-0.092882
	-0.101359	0.012693	-0.015950	0.014099	0.041832	0.074444
	-0.002756					
y381	-0.005980	0.007596	0.001134	0.079436	0.004577	-0.026011
	0.031393	0.015616	-0.054955	0.019769	-0.038432	0.022614
	0.007667	0.009749	-0.046099	0.002951	0.056723	0.050414
	-0.077124	-0.017789	0.017088	0.055296	-0.001159	-0.140522
	0.003466	0.079768	-0.019826	-0.028400	0.039318	0.026273
	0.014842					
z381	0.021073	0.031349	-0.015752	0.011509	-0.029033	0.017653
	-0.038826	0.019353	-0.068271	-0.030708	0.002067	-0.027779
	-0.051005	0.055220	0.066711	-0.020757	0.027211	0.033123
	0.101156	0.023684	0.043956	0.040354	-0.162657	-0.047115
	0.069944	-0.009375	-0.002361	0.056141	-0.015445	-0.117307
	-0.000826					
x382	-0.001272	-0.004965	-0.035881	0.012898	-0.002794	-0.007166
	-0.002175	0.013298	0.021066	0.013389	0.008400	0.000914
	0.021015	0.062314	-0.038781	-0.039321	0.066723	0.024177
	-0.061640	0.015927	0.037917	-0.038651	-0.006128	-0.055491
	-0.083056	0.019744	-0.006159	-0.024473	0.024369	0.019235
	-0.008793					

y382	-0.003509	0.008412	-0.002375	0.075799	0.002904	-0.037301
	0.023952	0.051651	-0.042465	0.016505	-0.031384	-0.003579
	-0.015606	0.001312	-0.090803	0.026328	0.084918	0.064259
	-0.102214	-0.036366	0.007054	0.006350	0.037561	-0.144764
	0.006915	0.090318	0.014626	-0.024236	0.032506	0.008932
	-0.002727					
z382	0.017665	0.037755	-0.018953	0.000420	-0.020198	0.002734
	-0.056183	0.017668	-0.044670	-0.012450	0.014855	-0.069436
	-0.019576	0.046772	0.058474	-0.063513	0.026968	0.026525
	0.112838	0.004909	0.026899	0.002180	-0.125998	-0.051481
	0.072767	0.037981	0.027791	0.037306	-0.037041	-0.079224
	-0.026962					
x383	-0.004690	-0.007137	-0.030582	0.020421	-0.002967	-0.000600
	-0.011601	0.030321	0.006701	0.010756	0.005567	-0.006349
	0.001727	0.075306	-0.045784	-0.008597	0.067062	0.033434
	-0.061070	0.015747	0.047508	-0.051038	-0.020849	-0.031866
	-0.058936	0.029444	-0.002379	-0.019423	0.046121	-0.011208
	0.014940					
y383	0.001895	0.000002	-0.017284	0.081476	-0.015811	-0.032005
	0.002395	0.087938	-0.032078	0.005338	-0.023337	-0.008752
	-0.063064	0.012618	-0.082633	0.054736	0.089972	0.076037
	-0.107119	0.012635	-0.007956	0.017922	0.036020	-0.092218
	0.024909	0.095891	0.020430	-0.022422	0.055736	-0.012861
	-0.009665					
z383	0.018724	0.030167	-0.023563	0.001570	-0.022848	-0.006248
	-0.066891	0.027546	-0.034259	0.016726	0.025376	-0.104088
	-0.038114	0.041476	0.089113	-0.081854	0.026885	0.032998
	0.116953	0.006573	-0.004283	0.018965	-0.118694	-0.029113
	0.081845	0.068184	0.038156	0.030720	-0.022636	-0.067236
	0.009706					
x384	-0.009983	-0.004869	-0.022692	0.017000	-0.002164	-0.001650
	-0.008720	0.020172	-0.002152	0.012886	0.007787	-0.004174
	0.022749	0.080138	-0.050390	-0.012647	0.061558	0.019630
	-0.054662	0.008240	0.052084	-0.042573	-0.045990	-0.010735
	-0.071758	0.022094	-0.011747	-0.035932	0.040384	-0.025239
	-0.000622					
y384	0.000415	0.003176	-0.017655	0.070712	-0.025057	-0.028691
	0.002393	0.063689	-0.025076	-0.010301	-0.021388	-0.000320
	-0.055206	-0.001392	-0.074324	0.016512	0.066204	0.074250
	-0.073645	0.038336	-0.020448	0.055747	0.017764	-0.062166
	-0.013435	0.066806	-0.007779	0.005301	0.043525	-0.019663
	-0.057496					

z384	0.011656	0.032557	-0.028624	0.011949	-0.021884	-0.025814
	-0.079827	0.020968	-0.009584	0.024304	0.038822	-0.127423
	-0.022920	0.039657	0.087859	-0.103574	0.014469	0.040990
	0.072156	-0.005877	-0.032208	0.027689	-0.118402	-0.025011
	0.052515	0.112380	0.022321	-0.000400	-0.069405	-0.050393
	0.025926					
x385	-0.007436	-0.006089	-0.024641	0.017222	-0.001667	-0.010414
	-0.022597	0.034526	-0.002636	0.011427	0.007257	-0.003040
	0.018643	0.072497	-0.056791	-0.003802	0.058285	0.017066
	-0.068817	-0.011890	0.047598	-0.038253	-0.051400	0.035899
	-0.054008	0.011905	-0.028435	0.007525	0.044476	-0.037787
	-0.004930					
y385	-0.001914	0.005777	-0.009407	0.057565	-0.029670	-0.018079
	0.019616	0.043911	-0.017570	-0.010887	-0.018444	0.009070
	-0.048262	-0.014374	-0.059452	0.007798	0.065776	0.055768
	-0.039001	0.035060	-0.023081	0.033556	0.056337	-0.056344
	-0.027192	0.049266	0.007543	-0.025865	0.019999	0.012420
	-0.050841					
z385	0.009693	0.033387	-0.029646	0.016720	-0.016731	-0.032639
	-0.066962	0.003095	-0.005190	0.026600	0.031592	-0.126033
	-0.001206	0.035784	0.081736	-0.107854	0.006735	0.032843
	0.063737	-0.007324	-0.025244	0.035989	-0.064336	-0.042631
	0.032600	0.119861	0.004352	-0.008930	-0.089457	0.008523
	0.019363					
x386	-0.005317	-0.002085	-0.023155	0.003884	-0.006555	-0.012162
	-0.026275	0.036619	-0.008328	0.014096	0.018011	-0.016358
	0.006639	0.067217	-0.063441	-0.002091	0.051194	0.011397
	-0.057267	0.008931	0.069230	-0.018437	-0.044392	0.047730
	-0.021368	0.038253	-0.041597	0.049787	0.025890	-0.052526
	0.030243					
y386	-0.001368	0.003160	-0.002414	0.052606	-0.026930	-0.010956
	0.031891	0.025250	-0.000641	-0.001648	-0.009819	0.022716
	-0.039959	-0.014161	-0.041817	0.016927	0.074633	0.017900
	-0.013610	-0.000871	-0.037834	0.002766	0.062977	-0.049038
	-0.030265	0.022159	0.007013	-0.054536	0.016524	0.016703
	-0.070760					
z386	0.009920	0.030804	-0.033034	0.022524	-0.004466	-0.027480
	-0.061073	-0.011122	-0.009790	0.019447	0.021219	-0.118651
	0.022199	0.027992	0.066490	-0.107880	0.009857	0.011008
	0.085970	-0.032029	-0.019279	0.032279	-0.016282	-0.014395
	0.018951	0.096531	-0.002758	-0.033006	-0.064971	0.064159
	-0.027391					

x387	-0.007895	0.001555	-0.019621	-0.002796	-0.019261	-0.011571
	-0.031503	0.022998	-0.010182	0.018739	0.015021	-0.021932
	0.009050	0.059981	-0.048022	-0.008610	0.047000	0.011852
	-0.036638	-0.005774	0.049387	0.000355	-0.035193	0.026001
	-0.005804	0.028086	-0.012397	0.050265	0.002446	-0.040306
	0.053148					
y387	0.003168	-0.000786	0.003826	0.040974	-0.019173	-0.008743
	0.038771	0.024910	0.013379	0.004089	0.005153	0.034887
	-0.033786	-0.015092	-0.030496	0.028461	0.068568	-0.016319
	-0.007045	-0.022433	-0.029808	-0.017076	0.059625	-0.017070
	-0.040948	-0.011630	-0.016256	-0.021922	0.031833	0.010108
	-0.081268					
z387	0.013843	0.028275	-0.032237	0.029674	0.005338	-0.027699
	-0.048153	-0.015863	-0.011082	0.023944	0.021061	-0.102237
	0.027902	0.019327	0.047293	-0.094444	0.023209	-0.001681
	0.068353	-0.031949	0.002708	0.029017	0.019666	0.034370
	-0.000418	0.070095	-0.018647	-0.027523	-0.043291	0.090407
	-0.048056					
x388	-0.010264	0.002408	-0.012284	-0.005443	-0.032682	-0.013793
	-0.033698	0.006544	-0.006445	0.026083	0.013738	-0.030486
	0.016900	0.043247	-0.032818	-0.008887	0.050864	0.015870
	-0.032739	-0.014587	0.028067	-0.000371	-0.032020	0.010916
	-0.012440	0.014368	-0.010541	0.034710	-0.022609	-0.033334
	0.056745					
y388	0.008865	-0.000323	0.001391	0.035688	-0.014037	-0.004922
	0.038555	0.030495	0.010159	0.014543	0.008640	0.041752
	-0.037856	-0.014387	-0.025736	0.024180	0.059629	-0.032408
	0.014750	-0.023629	-0.003657	-0.001075	0.056743	0.021358
	-0.033126	-0.014750	-0.024822	-0.024582	0.074407	-0.014955
	-0.060928					
z388	0.013431	0.025809	-0.029374	0.036602	0.008173	-0.029675
	-0.037409	-0.012611	-0.009520	0.042926	0.018108	-0.069752
	0.007702	0.019478	0.032049	-0.061604	0.019823	-0.006071
	0.050451	-0.044340	0.029253	0.047776	0.020837	0.068359
	0.005316	0.033251	-0.018996	-0.032547	-0.033122	0.079859
	-0.002610					
x389	-0.011976	0.002906	-0.005379	-0.007472	-0.038868	-0.016908
	-0.025446	-0.006118	-0.008131	0.037555	0.008458	-0.038029
	0.016515	0.034790	-0.034901	0.000757	0.047628	0.015938
	-0.028624	-0.032721	0.014486	-0.000380	-0.025732	0.004184
	-0.012978	-0.015020	-0.006087	0.019871	-0.030359	-0.030407
	0.057825					

y389	0.012287	0.001595	-0.005428	0.023970	-0.016306	-0.005983
	0.031756	0.029303	0.004833	0.004100	0.009150	0.034186
	-0.023235	-0.022836	-0.009153	0.002742	0.056969	-0.026649
	0.024343	-0.019723	-0.003346	-0.024273	0.039424	0.010204
	-0.038119	0.044038	-0.021302	-0.003619	0.053887	-0.027071
	-0.073498					
z389	0.010315	0.024401	-0.026941	0.031347	0.016030	-0.034500
	-0.027701	-0.014621	-0.018568	0.049488	0.002949	-0.047699
	0.006695	0.015547	0.012389	-0.025801	0.033352	0.002927
	0.030674	-0.050632	0.012844	0.035350	0.023280	0.057394
	0.013961	-0.000049	-0.007301	-0.048731	-0.023667	0.067062
	0.001594					
x390	-0.011128	0.000674	-0.001621	-0.009731	-0.042399	-0.012635
	-0.023543	-0.011405	-0.012063	0.039778	0.006427	-0.035637
	0.015069	0.023549	-0.023568	0.000945	0.051392	0.006582
	-0.023061	-0.035682	0.007589	-0.007842	-0.027269	-0.003594
	-0.017644	-0.028784	0.003609	0.009720	-0.017499	-0.031208
	0.046332					
y390	0.013114	0.000482	-0.008547	0.028810	-0.016705	-0.002936
	0.029165	0.013888	0.013563	0.006748	0.015747	0.009418
	-0.026001	-0.023306	0.001295	-0.014710	0.034261	-0.028393
	0.034872	-0.024520	0.003281	-0.016068	0.020290	0.021425
	-0.046732	0.033408	-0.033092	0.029856	0.015265	-0.018163
	-0.072620					
z390	0.008313	0.021112	-0.027937	0.030599	0.017133	-0.025908
	-0.023750	-0.021807	-0.023724	0.050793	-0.006065	-0.036348
	0.007570	0.003475	0.020229	-0.012535	0.046849	-0.013547
	0.028274	-0.047433	0.006608	0.026939	0.006050	0.054289
	0.007129	-0.029293	0.009867	-0.040768	-0.020914	0.057999
	-0.015484					
x391	-0.010394	-0.002731	0.001224	-0.011350	-0.046681	-0.011011
	-0.019921	-0.015808	-0.014862	0.041560	0.009651	-0.025298
	0.008943	0.020696	-0.014763	-0.000666	0.051807	0.005103
	-0.019138	-0.035092	0.015336	-0.004337	-0.022673	0.004567
	-0.019605	-0.031996	0.004360	0.003424	-0.018420	-0.023911
	0.047168					
y391	0.016329	0.000605	-0.011583	0.026726	-0.012526	-0.007683
	0.019579	0.013037	0.016248	0.005464	0.017556	-0.000007
	-0.021383	-0.023523	0.011915	-0.018211	0.018018	-0.036421
	0.039280	-0.023815	0.001225	-0.022645	0.011259	-0.005803
	-0.066253	0.054304	-0.056107	0.028499	-0.012129	-0.035013
	-0.068642					

z391	0.005991	0.014363	-0.029886	0.028796	0.017672	-0.022465
	-0.020510	-0.020304	-0.027146	0.048423	-0.003690	-0.016532
	-0.001423	0.000900	0.026214	-0.005324	0.046712	-0.018459
	0.027434	-0.046076	0.014721	0.026909	-0.002194	0.055619
	-0.005831	-0.029928	0.001795	-0.036795	-0.022900	0.049392
	-0.007288					
x392	-0.009351	-0.006013	0.003222	-0.009873	-0.051625	-0.010039
	-0.017065	-0.020288	-0.016574	0.041814	0.015153	-0.016715
	0.001625	0.018958	-0.004951	-0.004688	0.049253	0.007338
	-0.016593	-0.032056	0.021872	-0.000664	-0.018533	0.013763
	-0.018033	-0.032470	0.002044	0.004993	-0.016440	-0.013271
	0.046777					
y392	0.020295	-0.001058	-0.014259	0.021909	-0.012152	-0.009833
	0.017303	0.011828	0.017856	0.009548	0.015869	-0.006801
	-0.022345	-0.025533	0.017704	-0.009905	0.008724	-0.035383
	0.036268	-0.018593	-0.010293	-0.041069	0.016281	-0.033503
	-0.074647	0.051734	-0.068170	0.034211	-0.012896	-0.036965
	-0.058705					
z392	0.002946	0.007342	-0.031216	0.025701	0.014451	-0.019297
	-0.014968	-0.018769	-0.029308	0.043498	0.002818	0.002215
	-0.009631	-0.002217	0.029919	-0.000251	0.048123	-0.010751
	0.018940	-0.041324	0.018942	0.019120	-0.005107	0.057290
	-0.008294	-0.030462	-0.003946	-0.029491	-0.014700	0.050570
	0.005444					
x393	-0.007114	-0.008262	0.004889	-0.005606	-0.054842	-0.010184
	-0.013484	-0.021486	-0.015250	0.039097	0.020253	-0.008354
	-0.003325	0.017556	0.000145	-0.008775	0.047927	0.002626
	-0.013455	-0.025704	0.031323	0.005987	-0.014928	0.018783
	-0.011648	-0.031580	0.002108	0.007545	-0.015179	-0.003718
	0.042528					
y393	0.022830	-0.002229	-0.012979	0.019550	-0.008752	-0.006114
	0.017601	0.007557	0.024863	0.010624	0.011632	-0.013532
	-0.016011	-0.025946	0.018204	-0.007279	0.002814	-0.041710
	0.029648	-0.003017	-0.010503	-0.039649	0.019250	-0.044591
	-0.080417	0.041281	-0.052589	0.032321	-0.024988	-0.030076
	-0.064515					
z393	0.000214	0.001144	-0.029825	0.023058	0.011511	-0.015907
	-0.009072	-0.018070	-0.028808	0.037806	0.008745	0.016802
	-0.011016	-0.001921	0.029468	0.001314	0.052583	-0.010552
	0.010513	-0.032212	0.026219	0.025159	-0.008355	0.060733
	-0.007327	-0.029654	0.000117	-0.024353	-0.004865	0.051946
	0.011097					

x394	-0.003482	-0.009186	0.005981	0.000371	-0.055326	-0.011544
	-0.009461	-0.019483	-0.013629	0.034868	0.022884	0.001672
	-0.004625	0.015462	0.004289	-0.015314	0.046917	-0.002367
	-0.007721	-0.022310	0.039472	0.014065	-0.011827	0.025870
	0.000588	-0.026954	0.003934	0.005866	-0.022416	0.004978
	0.040153					
y394	0.026268	-0.002999	-0.012221	0.018352	-0.000092	-0.003682
	0.017254	0.003418	0.029124	0.008241	0.007968	-0.017275
	-0.007099	-0.021413	0.018536	-0.017375	-0.002107	-0.044870
	0.024910	-0.000499	-0.004531	-0.024540	0.019560	-0.034188
	-0.066267	0.034668	-0.036529	0.013024	-0.058115	-0.033965
	-0.072482					
z394	-0.001560	-0.003410	-0.028415	0.019520	0.011767	-0.013467
	-0.002394	-0.016138	-0.029673	0.031486	0.010872	0.030917
	-0.009230	0.002735	0.026506	-0.004750	0.059742	-0.011475
	0.003407	-0.036228	0.033894	0.033810	-0.014985	0.073249
	0.007355	-0.027865	0.002914	-0.027442	-0.005560	0.041838
	0.016630					
x395	0.001249	-0.010662	0.006115	0.006621	-0.055922	-0.013633
	-0.006146	-0.016652	-0.009212	0.032160	0.023385	0.004503
	-0.005259	0.013044	0.008592	-0.019856	0.040121	-0.003988
	-0.002037	-0.013560	0.044050	0.015726	-0.007931	0.027350
	0.013242	-0.019199	-0.000995	0.007724	-0.019577	0.008402
	0.036954					
y395	0.029940	-0.004346	-0.012560	0.014417	0.005237	-0.002891
	0.014940	-0.002947	0.034905	0.008673	0.004771	-0.027940
	0.002147	-0.021432	0.019350	-0.022114	-0.008189	-0.042286
	0.021404	0.006177	-0.003264	-0.026785	0.019614	-0.031548
	-0.054290	0.031640	-0.025135	0.008179	-0.063154	-0.033425
	-0.061014					
z395	-0.003112	-0.008951	-0.026164	0.014290	0.008711	-0.011143
	0.002120	-0.014513	-0.026849	0.025696	0.011511	0.035793
	-0.003981	0.004903	0.025650	-0.008718	0.061357	-0.010030
	-0.005339	-0.036542	0.036934	0.033478	-0.019388	0.078295
	0.020556	-0.018789	0.003877	-0.026897	0.008239	0.037391
	0.019692					
x396	0.005990	-0.010585	0.005620	0.012823	-0.054322	-0.015226
	-0.001412	-0.007791	-0.005018	0.030474	0.025671	0.011815
	-0.008163	0.008278	0.009348	-0.021201	0.036324	-0.014481
	0.004811	-0.007391	0.048014	0.022556	-0.003325	0.019492
	0.015839	-0.011883	-0.005524	0.012650	-0.007334	0.008072
	0.031853					

y396	0.031373	-0.004301	-0.012704	0.006902	0.010175	-0.001616
	0.014053	-0.004363	0.038980	0.012112	0.006234	-0.028940
	0.005324	-0.025273	0.014463	-0.017941	-0.007069	-0.043795
	0.022551	0.006194	0.000572	-0.022557	0.020181	-0.039603
	-0.056949	0.017914	-0.009206	0.017752	-0.052250	-0.035815
	-0.056864					
z396	-0.004223	-0.012376	-0.023511	0.008850	0.005113	-0.008840
	0.006308	-0.009054	-0.025322	0.019971	0.012033	0.043493
	0.002099	0.003795	0.023282	-0.012404	0.066085	-0.016611
	-0.012245	-0.039187	0.038016	0.037532	-0.023804	0.074288
	0.025333	-0.005418	0.005053	-0.028940	0.024186	0.031072
	0.020496					
x397	0.010489	-0.011687	0.006055	0.018476	-0.052111	-0.016566
	0.001957	-0.003604	-0.000508	0.026379	0.026010	0.013514
	-0.012300	0.001892	0.010152	-0.021987	0.031634	-0.012155
	0.003720	0.005229	0.045235	0.020774	-0.000345	0.013981
	0.021504	-0.001851	-0.013837	0.012659	-0.003717	0.009194
	0.020494					
y397	0.029953	-0.006606	-0.010904	0.000031	0.014425	-0.000306
	0.012704	-0.012477	0.040058	0.012089	0.008628	-0.030926
	0.004410	-0.030992	0.010673	-0.011345	-0.006803	-0.032547
	0.009153	0.010863	0.002401	-0.022331	0.017330	-0.039468
	-0.058473	0.009985	-0.000834	0.017936	-0.054521	-0.030905
	-0.071329					
z397	-0.005106	-0.015881	-0.020552	0.003408	0.000627	-0.006867
	0.007760	-0.007380	-0.021804	0.013926	0.008660	0.046503
	0.010181	0.002535	0.021945	-0.015537	0.070015	-0.015991
	-0.017262	-0.035630	0.031633	0.033721	-0.025795	0.068879
	0.036521	0.006763	0.001154	-0.037115	0.034737	0.024315
	0.021863					
x398	0.015069	-0.012525	0.006887	0.026055	-0.049751	-0.020192
	0.004794	0.002352	0.002540	0.027474	0.018939	0.011215
	-0.010462	0.001963	0.010424	-0.027839	0.026116	-0.010661
	0.000316	0.013384	0.042051	0.013352	0.001608	0.014291
	0.020299	0.007311	-0.015432	0.005859	-0.007049	0.002931
	0.012060					
y398	0.028498	-0.009892	-0.006929	-0.003231	0.016726	0.001631
	0.014701	-0.014348	0.036109	0.011618	0.005731	-0.025970
	0.004422	-0.032988	0.009397	-0.020599	-0.008693	-0.017700
	0.004641	0.016384	0.003576	-0.022698	0.014303	-0.037181
	-0.046638	0.013442	0.015435	0.025829	-0.043521	-0.042092
	-0.059732					

z398	-0.005720	-0.017419	-0.017767	0.001457	-0.003516	-0.008210
	0.005536	-0.006217	-0.017089	0.014462	-0.001891	0.040574
	0.022765	0.006789	0.018640	-0.017462	0.075009	-0.022001
	-0.027948	-0.035245	0.020593	0.024597	-0.030167	0.067735
	0.034613	0.011422	0.004242	-0.058015	0.030102	0.014953
	0.007386					
x399	0.024000	-0.015992	0.007815	0.031541	-0.042997	-0.017109
	0.008990	0.008191	0.004038	0.025888	0.022585	0.004295
	-0.004507	0.002714	0.010431	-0.028130	0.022503	-0.011795
	0.000524	0.018480	0.037419	0.003088	0.003712	0.015823
	0.021219	-0.007238	-0.024350	0.001851	0.002880	0.009018
	-0.012411					
y399	0.025755	-0.014270	-0.002292	-0.013279	0.018876	0.005930
	0.014337	-0.017711	0.032287	0.006580	0.004481	-0.024436
	0.012182	-0.031511	0.008135	-0.021250	0.003449	-0.005749
	-0.006823	0.021561	0.011376	-0.017227	0.002323	-0.031973
	-0.029044	0.003406	0.024409	0.013574	-0.012344	-0.032792
	-0.056266					
z399	-0.004209	-0.019370	-0.015569	0.003084	-0.013707	0.000670
	0.003578	-0.003458	-0.013380	0.010294	-0.002887	0.035810
	0.030119	0.021816	0.014099	-0.021476	0.065827	-0.030633
	-0.019288	-0.036690	-0.000460	0.008180	-0.028658	0.057247
	0.039212	-0.000592	0.002023	-0.058464	0.025698	0.005217
	-0.010293					
x400	0.031249	-0.019186	0.009220	0.032817	-0.036902	-0.011995
	0.014785	0.008950	0.007682	0.027181	0.022224	-0.000736
	-0.006640	0.000202	0.007086	-0.031606	0.018675	0.001763
	-0.003386	0.014255	0.035750	-0.006259	0.002609	0.024518
	0.023901	-0.004057	-0.034419	0.010584	-0.003358	-0.005937
	-0.010616					
y400	0.022890	-0.017376	-0.000111	-0.017917	0.017543	0.009520
	0.015926	-0.020805	0.034151	0.007360	-0.000182	-0.019953
	0.009538	-0.028913	0.003280	-0.022594	0.012474	0.001192
	-0.010975	0.018912	0.018410	-0.017330	0.000804	-0.022611
	-0.013876	0.008258	0.023889	0.012561	-0.003512	-0.047173
	-0.036966					
z400	-0.002147	-0.019223	-0.013563	0.009009	-0.023576	0.008536
	0.002019	0.002058	-0.011631	0.005636	-0.006758	0.035581
	0.028008	0.036018	0.013332	-0.026269	0.050168	-0.019998
	-0.013945	-0.032607	-0.014202	0.002334	-0.033138	0.046609
	0.050795	0.009170	-0.011932	-0.053187	0.008998	-0.006665
	-0.000546					

x401	0.033794	-0.021939	0.011945	0.027783	-0.030568	-0.010263
	0.020662	0.009890	0.014916	0.023941	0.025484	-0.003475
	-0.009677	-0.010253	0.000489	-0.025423	0.001764	-0.004059
	-0.019597	0.021516	0.026120	-0.013198	0.001525	0.030701
	0.021080	-0.017464	-0.022329	0.024532	0.025358	-0.000021
	-0.053262					
y401	0.017768	-0.018433	0.003057	-0.020079	0.014217	0.008078
	0.017888	-0.019706	0.036853	0.006010	-0.004625	-0.011738
	0.005548	-0.028135	-0.002753	-0.018633	0.006862	-0.006768
	-0.016860	0.028369	0.012030	-0.018056	0.002455	-0.018133
	0.003780	0.004010	0.030175	0.012419	0.006829	-0.049053
	-0.045920					
z401	-0.002693	-0.018063	-0.008227	0.012506	-0.025526	0.011845
	-0.005142	0.006687	-0.004370	0.005186	-0.004559	0.043562
	0.025625	0.043047	0.014889	-0.015895	0.019849	-0.012650
	-0.026194	-0.021846	-0.039596	-0.017232	-0.045202	0.046362
	0.059766	0.017428	-0.010191	-0.039773	0.026929	-0.011481
	-0.019263					
x402	0.039471	-0.026305	0.012051	0.027730	-0.028427	-0.004869
	0.023493	0.009922	0.018402	0.026064	0.031612	-0.016175
	-0.004226	-0.014309	-0.003245	-0.024579	0.008051	-0.006186
	-0.012994	0.015856	0.030345	-0.024088	-0.001561	0.028138
	0.028798	-0.030675	-0.010908	0.034183	0.025914	0.012241
	-0.054360					
y402	0.014344	-0.018671	0.004341	-0.018708	0.007601	0.008359
	0.016497	-0.017836	0.036854	0.005924	-0.005260	-0.009785
	0.004413	-0.025144	-0.003243	-0.015230	0.010092	-0.012863
	-0.013092	0.027406	0.012484	-0.023183	0.001150	-0.018786
	0.020288	0.002276	0.035380	0.012452	0.011821	-0.038794
	-0.034002					
z402	0.000084	-0.020225	-0.010182	0.019212	-0.034128	0.017818
	-0.005925	0.011524	-0.006322	-0.002904	-0.010566	0.037513
	0.020263	0.050650	0.012423	-0.017799	0.012054	-0.012182
	-0.021382	-0.016228	-0.044402	-0.006886	-0.036291	0.030706
	0.054430	0.010428	-0.019690	-0.031022	0.025310	-0.009268
	-0.015428					
x403	0.042123	-0.026050	0.012349	0.023355	-0.020475	-0.002052
	0.025871	0.010747	0.019126	0.024022	0.034841	-0.005735
	-0.011954	-0.019465	-0.007814	-0.024683	0.004352	-0.009772
	-0.015917	0.004867	0.026938	-0.024635	-0.006008	0.041870
	0.029180	-0.026406	0.008440	0.038482	0.019564	0.010946
	-0.058488					

y403	0.010260	-0.016992	0.005829	-0.018497	0.002785	0.007354
	0.015668	-0.014513	0.033228	0.003039	-0.006828	0.002119
	-0.002251	-0.019881	-0.004299	-0.013669	0.008921	-0.022511
	-0.010250	0.022402	0.007188	-0.021893	0.002026	-0.014097
	0.031952	0.009368	0.039484	0.007222	0.007785	-0.039264
	-0.027969					
z403	0.000336	-0.019036	-0.007952	0.022676	-0.033095	0.020009
	-0.010370	0.016768	-0.004941	-0.007681	-0.010971	0.046121
	0.013613	0.052592	0.008856	-0.010235	-0.003351	-0.010034
	-0.031113	-0.010448	-0.052716	-0.007403	-0.040175	0.034529
	0.046860	0.012758	-0.020378	-0.022795	0.027505	-0.011530
	-0.016676					
x404	0.043487	-0.029230	0.011994	0.020605	-0.018801	0.002237
	0.024747	0.007405	0.019214	0.021412	0.031347	-0.011946
	-0.006436	-0.016871	-0.005992	-0.023811	0.010109	-0.001470
	-0.020098	0.002676	0.031490	-0.024216	-0.007196	0.044141
	0.019681	-0.029941	-0.001467	0.046950	0.023142	0.026845
	-0.046566					
y404	0.005618	-0.017021	0.007946	-0.016814	-0.005288	0.006380
	0.013092	-0.012140	0.028588	0.000710	-0.011028	0.006925
	-0.003009	-0.010107	-0.003288	-0.009381	0.008471	-0.027696
	-0.008392	0.021394	0.002082	-0.021755	0.003847	-0.015580
	0.037754	0.012430	0.029268	0.003314	0.007048	-0.033914
	-0.017050					
z404	0.001667	-0.020904	-0.009641	0.027297	-0.033779	0.021709
	-0.016382	0.017551	-0.006656	-0.013354	-0.014268	0.039623
	0.012372	0.056678	0.004023	-0.011039	-0.005246	-0.002215
	-0.041009	0.000518	-0.051192	0.002102	-0.035246	0.027376
	0.023584	0.017231	-0.029784	-0.032809	0.013866	-0.005325
	-0.001610					
x405	0.043793	-0.030861	0.011282	0.017382	-0.015039	0.009336
	0.021567	0.008699	0.020020	0.025331	0.023886	-0.005881
	-0.005824	-0.019581	-0.000654	-0.020967	-0.011937	0.018546
	-0.035878	-0.000872	0.024579	-0.033338	-0.010948	0.055984
	0.012022	-0.015615	0.001379	0.040444	0.013340	0.036230
	-0.034916					
y405	0.000353	-0.016543	0.010250	-0.015045	-0.012378	0.005045
	0.008336	-0.008589	0.023722	0.000630	-0.016747	0.013216
	-0.003467	-0.001925	-0.001720	-0.003936	-0.001808	-0.026558
	-0.008883	0.019565	-0.006636	-0.025266	0.003440	-0.013952
	0.040448	0.017804	0.018868	-0.002713	0.002351	-0.028585
	-0.007126					

z405	0.001124	-0.022108	-0.008311	0.028428	-0.027934	0.019802
	-0.026111	0.017085	-0.005917	-0.010597	-0.015840	0.041354
	0.012978	0.054211	0.004473	-0.004449	-0.015265	0.009782
	-0.051846	0.010638	-0.053119	-0.010694	-0.033327	0.043489
	0.016241	0.030167	-0.027673	-0.034707	-0.001011	-0.015160
	0.007490					
x406	0.044329	-0.021902	0.011517	0.014460	-0.008902	0.009911
	0.020479	0.006870	0.021820	0.018720	0.015284	0.016139
	-0.027335	-0.018034	0.008182	-0.012658	-0.016986	0.052782
	-0.049399	-0.006025	0.010307	-0.028141	-0.024910	0.079916
	-0.001105	0.009971	0.010609	0.046236	0.009914	0.061514
	-0.017195					
y406	-0.005598	-0.011917	0.011157	-0.014290	-0.017981	0.001515
	0.002554	-0.006624	0.014725	-0.004459	-0.026733	0.021606
	-0.009267	0.002214	0.000791	-0.000935	-0.013974	-0.010107
	-0.016107	0.014108	-0.015292	-0.023822	-0.004312	-0.006108
	0.031309	0.023766	0.007160	-0.007208	-0.011156	-0.015513
	0.009706					
z406	0.001003	-0.019091	-0.009410	0.028722	-0.017738	0.018784
	-0.033682	0.019967	-0.009795	-0.011264	-0.019140	0.049008
	0.008312	0.044266	0.005434	-0.002905	-0.016802	0.021134
	-0.057825	0.011252	-0.042703	-0.013505	-0.031596	0.068793
	-0.003268	0.044911	-0.028909	-0.036604	-0.034583	-0.016321
	0.033246					
x407	0.044583	-0.022376	0.009906	0.014801	-0.012408	0.018294
	0.014216	0.005301	0.022358	0.019036	0.005074	0.009831
	-0.030019	-0.014977	0.021262	-0.005430	-0.012099	0.055391
	-0.058414	-0.000271	0.017303	-0.006755	-0.011346	0.071077
	-0.003217	0.017939	0.018200	0.030309	0.007974	0.063676
	0.008588					
y407	-0.011440	-0.012507	0.011619	-0.012327	-0.023126	-0.003450
	-0.003815	-0.000992	0.005776	-0.001073	-0.024942	0.025773
	-0.004735	0.009730	-0.004098	0.005616	-0.017746	-0.021557
	-0.008368	0.011876	-0.020923	-0.034228	-0.004463	-0.007813
	0.028816	0.019886	0.001895	-0.014705	-0.021013	-0.017495
	0.010327					
z407	0.001123	-0.021321	-0.011353	0.028017	-0.012092	0.017269
	-0.043864	0.018576	-0.012629	-0.006598	-0.021754	0.039254
	0.010324	0.040668	0.009397	-0.000155	-0.013125	0.030950
	-0.055980	0.017880	-0.026425	-0.018571	-0.020751	0.078412
	-0.031868	0.060077	-0.037573	-0.045336	-0.064072	-0.009981
	0.063725					

x408	0.041750	-0.023562	0.011256	0.012850	-0.014520	0.019881
	0.012935	0.009317	0.022448	0.017747	0.010143	0.006883
	-0.018200	-0.009745	0.021306	0.000987	0.001706	0.035142
	-0.040608	0.000849	0.029170	-0.006248	-0.000700	0.063593
	0.003281	0.010628	0.036202	0.013302	0.032295	0.042739
	0.004772					
y408	-0.016811	-0.010955	0.010799	-0.010751	-0.024366	-0.007791
	-0.011229	0.001548	-0.002487	0.003287	-0.027297	0.025756
	-0.007763	0.005908	-0.003978	0.006904	-0.025746	-0.013049
	-0.019937	0.012720	-0.028095	-0.035473	-0.013789	-0.010690
	0.027068	0.016506	-0.002397	-0.017315	-0.033357	-0.016821
	0.006169					
z408	-0.001678	-0.022495	-0.011326	0.021737	-0.005437	0.016677
	-0.038951	0.021311	-0.012070	-0.000501	-0.012790	0.029432
	0.011743	0.032659	0.015689	0.004347	-0.013498	0.007480
	-0.054280	0.013622	-0.016980	-0.026695	-0.019341	0.075761
	-0.024142	0.039784	-0.028915	-0.048205	-0.039320	-0.020580
	0.026044					
x409	0.038401	-0.025130	0.015861	0.013361	-0.011351	0.021717
	0.007922	0.014989	0.017561	0.025459	0.011410	0.006769
	-0.011399	0.000713	0.016807	0.011375	0.001883	0.021977
	-0.027521	0.000483	0.030871	-0.013448	-0.014856	0.060914
	0.019878	0.006060	0.019278	0.006164	0.038241	0.026657
	-0.008588					
y409	-0.022039	-0.008891	0.008897	-0.009063	-0.026550	-0.012983
	-0.018423	0.004368	-0.005823	0.006242	-0.024659	0.028529
	-0.005816	-0.000834	-0.003692	0.006501	-0.028781	-0.007385
	-0.030618	0.016509	-0.032408	-0.035992	-0.008326	-0.011734
	0.025170	0.013160	0.014208	-0.021613	-0.040596	-0.018311
	0.014986					
z409	-0.003299	-0.022309	-0.009411	0.019805	0.000611	0.015642
	-0.035194	0.025349	-0.010355	0.009660	-0.006043	0.026638
	0.016301	0.033771	0.016093	0.006733	-0.012135	-0.011410
	-0.046201	0.013716	-0.015246	-0.035904	-0.017838	0.075622
	-0.002473	0.032084	-0.024966	-0.054275	-0.042791	-0.037660
	0.016920					
x410	0.035283	-0.027312	0.015085	0.014036	-0.008727	0.022074
	0.001461	0.018275	0.011311	0.031500	0.008695	0.007883
	-0.003403	-0.001685	0.014382	0.012529	0.007874	0.020901
	-0.013987	-0.003874	0.023017	-0.016808	-0.018991	0.052346
	0.029952	0.009645	0.016576	0.001396	0.020621	0.011070
	-0.007271					

y410	-0.026657	-0.006351	0.009341	-0.006918	-0.028175	-0.015956
	-0.023948	0.008017	-0.010105	0.007402	-0.020964	0.028317
	-0.004628	0.001565	-0.007828	0.007119	-0.029117	-0.008839
	-0.034211	0.022180	-0.027574	-0.035177	-0.007609	-0.005529
	0.028024	0.001256	0.016234	-0.033940	-0.048402	-0.017668
	0.023717					
z410	-0.004073	-0.021712	-0.008546	0.019204	0.006273	0.015650
	-0.030567	0.028301	-0.008031	0.017711	-0.003344	0.024983
	0.022023	0.038477	0.015527	0.001926	-0.005453	-0.025942
	-0.033107	0.012993	-0.017471	-0.042081	-0.015327	0.077215
	0.020770	0.024330	-0.024645	-0.064524	-0.063085	-0.053647
	0.020462					
x411	0.030926	-0.028707	0.014110	0.013658	-0.007112	0.021625
	-0.002374	0.021892	0.004413	0.032115	0.009133	0.010683
	0.002778	0.001610	0.008006	0.012987	0.009738	0.010039
	-0.014846	-0.000161	0.022739	-0.011739	-0.015170	0.045397
	0.029417	0.011025	0.020730	-0.003833	0.013009	0.008914
	-0.019349					
y411	-0.029947	-0.002610	0.008502	-0.004920	-0.029305	-0.018571
	-0.029636	0.010536	-0.010898	0.010206	-0.018004	0.023064
	-0.004290	-0.006052	-0.006197	0.010956	-0.027896	-0.002269
	-0.031612	0.018768	-0.025862	-0.036540	-0.019375	-0.006256
	0.030559	-0.008461	0.014437	-0.040211	-0.042744	-0.013130
	0.037398					
z411	-0.006330	-0.018826	-0.008935	0.015664	0.009243	0.015613
	-0.024202	0.030835	-0.001047	0.019729	0.003506	0.021024
	0.022367	0.037679	0.014602	0.004451	-0.009190	-0.049710
	-0.043057	0.015441	-0.012749	-0.034905	-0.015060	0.065237
	0.014553	0.017358	-0.015344	-0.061584	-0.029372	-0.045471
	-0.004963					
x412	0.026193	-0.030707	0.016004	0.012083	-0.005395	0.022989
	-0.008270	0.026190	0.000469	0.035708	0.009761	0.010676
	0.004137	0.003947	0.000752	0.016845	0.011445	0.003466
	-0.008399	0.003571	0.020356	-0.007274	-0.011968	0.042293
	0.029962	0.008262	0.018209	-0.014891	0.013348	0.015108
	-0.023254					
y412	-0.032783	0.002881	0.004302	-0.001088	-0.029877	-0.024408
	-0.031761	0.012367	-0.012956	0.007142	-0.015120	0.022922
	0.001783	-0.013137	-0.000937	0.009855	-0.028174	0.000772
	-0.040666	0.016905	-0.020305	-0.035701	-0.027829	-0.007806
	0.028936	-0.010437	0.027193	-0.033878	-0.043275	-0.016546
	0.037358					

z412	-0.007707	-0.016366	-0.007987	0.012854	0.012109	0.016816
	-0.018245	0.033418	0.004360	0.022770	0.004905	0.015883
	0.024181	0.039991	0.015027	0.002999	-0.009880	-0.061667
	-0.038647	0.018159	-0.012520	-0.031782	-0.013280	0.062139
	0.020317	0.013548	-0.010056	-0.061161	-0.014445	-0.038578
	-0.013267					
x413	0.021282	-0.030689	0.015636	0.014775	-0.005989	0.022748
	-0.013042	0.032055	-0.006573	0.040164	0.009659	0.013614
	0.011054	0.008207	-0.006858	0.019611	0.014642	-0.003182
	-0.009245	0.009705	0.018759	0.001118	-0.012983	0.029058
	0.027080	0.009646	0.024804	-0.022896	0.014114	0.021110
	-0.025768					
y413	-0.034524	0.006423	0.004092	-0.000919	-0.028970	-0.028649
	-0.034665	0.011116	-0.005627	0.000684	-0.010897	0.019402
	-0.000860	-0.018291	0.002874	0.011543	-0.032679	0.004784
	-0.044896	0.007788	-0.017106	-0.031880	-0.030864	-0.001544
	0.028373	-0.014473	0.018214	-0.036580	-0.045547	-0.018249
	0.041082					
z413	-0.008615	-0.011853	-0.007508	0.012943	0.013594	0.017892
	-0.011605	0.036471	0.008430	0.025036	0.004739	0.013368
	0.028975	0.046496	0.010279	-0.000239	-0.008578	-0.072562
	-0.040046	0.022146	-0.014431	-0.020378	-0.014789	0.050283
	0.021851	0.010588	-0.004539	-0.059101	-0.000420	-0.026259
	-0.015120					
x414	0.015336	-0.029215	0.015998	0.015888	-0.005172	0.021433
	-0.020546	0.036605	-0.010649	0.042886	0.010324	0.017957
	0.019395	0.008950	-0.011899	0.022631	0.016450	-0.005313
	-0.009732	0.014532	0.012799	0.001969	-0.011514	0.027710
	0.029820	0.007398	0.030142	-0.026510	0.007802	0.016024
	-0.022891					
y414	-0.034420	0.008448	0.004704	0.003820	-0.029430	-0.030624
	-0.032835	0.013464	-0.000668	-0.000952	-0.006426	0.013603
	0.000807	-0.015726	-0.001979	0.014646	-0.033921	-0.005790
	-0.037353	0.005105	-0.009453	-0.019716	-0.033438	-0.002730
	0.033359	-0.006234	0.023353	-0.048156	-0.032179	-0.005427
	0.021421					
z414	-0.010485	-0.006704	-0.005797	0.010755	0.016830	0.018901
	-0.008022	0.038294	0.016027	0.025495	0.003897	0.013900
	0.035981	0.049089	0.007139	-0.006170	-0.008130	-0.075964
	-0.036856	0.023062	-0.024527	-0.020497	-0.014787	0.054840
	0.030888	0.006243	-0.001544	-0.053804	0.000727	-0.027014
	-0.012347					

x415	0.009868	-0.027064	0.015583	0.017716	-0.005368	0.020604
	-0.027040	0.041400	-0.014950	0.042678	0.007744	0.020150
	0.026285	0.011428	-0.016212	0.020649	0.020319	-0.005422
	-0.004602	0.015300	0.010662	0.008983	-0.008412	0.029802
	0.030251	0.006154	0.031806	-0.035870	-0.005054	0.016955
	-0.010982					
y415	-0.034567	0.012792	0.004001	0.004402	-0.029403	-0.031869
	-0.034521	0.012639	0.005274	-0.009278	-0.005806	0.010338
	0.007120	-0.016610	0.002680	0.016672	-0.037674	-0.003073
	-0.051246	0.016593	-0.011907	-0.017079	-0.038892	-0.004607
	0.030515	0.001780	0.031792	-0.040325	-0.004181	-0.005106
	0.014007					
z415	-0.010914	-0.001900	-0.007852	0.008187	0.016669	0.021930
	-0.000797	0.040745	0.020445	0.021743	-0.002524	0.008365
	0.038145	0.050690	0.001636	-0.018135	-0.000212	-0.077357
	-0.023373	0.015533	-0.025626	-0.006603	-0.011329	0.058097
	0.031897	-0.000156	-0.006654	-0.055245	-0.006003	-0.009251
	-0.001885					
x416	0.003388	-0.023446	0.016152	0.019692	-0.005817	0.019437
	-0.034602	0.044243	-0.014815	0.038769	0.005942	0.021200
	0.034276	0.015030	-0.021268	0.018671	0.019781	-0.008179
	-0.004825	0.017860	0.009136	0.018477	-0.006210	0.031129
	0.028595	0.005479	0.035690	-0.038927	-0.003520	0.018903
	-0.004459					
y416	-0.032337	0.013491	0.006198	0.005029	-0.024655	-0.032388
	-0.032890	0.009087	0.011376	-0.011031	0.000431	0.010344
	0.011576	-0.012933	0.007438	0.009916	-0.034792	-0.003535
	-0.050965	0.019163	-0.019316	-0.004166	-0.044298	0.004220
	0.028617	0.005812	0.028119	-0.044718	-0.001060	-0.009344
	0.026922					
z416	-0.013105	0.004883	-0.011118	0.005095	0.014658	0.023568
	0.003409	0.042762	0.025671	0.017287	-0.009213	0.003698
	0.040800	0.047074	-0.007203	-0.020495	0.000673	-0.077584
	-0.021913	0.016017	-0.026671	0.005011	-0.010802	0.055785
	0.029301	-0.006976	-0.006730	-0.039905	0.014214	0.013042
	-0.010694					
x417	-0.001499	-0.022619	0.017437	0.021686	-0.002973	0.017785
	-0.039507	0.045107	-0.014156	0.029851	0.003645	0.018948
	0.034797	0.020602	-0.021730	0.017985	0.015991	-0.005540
	-0.006392	0.015538	0.007027	0.018204	-0.006607	0.020581
	0.022705	0.008526	0.045273	-0.039196	-0.006545	0.000830
	0.002372					

y417	-0.030122	0.013038	0.006332	0.005334	-0.021609	-0.037810
	-0.028645	0.008576	0.015727	-0.016571	0.007471	0.011353
	0.012669	-0.010156	0.011532	0.010135	-0.028606	-0.004719
	-0.054134	0.014993	-0.016323	-0.001221	-0.043532	0.003271
	0.024796	0.011632	0.040082	-0.054714	-0.001245	-0.021530
	0.023449					
z417	-0.013475	0.005369	-0.011814	0.003473	0.019086	0.025740
	0.006644	0.041967	0.026306	0.015529	-0.015496	-0.002758
	0.034715	0.044158	-0.017617	-0.020790	-0.004644	-0.063714
	-0.013850	0.007491	-0.031110	0.002843	-0.014373	0.039040
	0.027125	-0.013201	-0.009460	-0.015829	0.010374	0.005890
	-0.004062					
x418	-0.005473	-0.020497	0.018907	0.023991	-0.000559	0.018159
	-0.042668	0.045532	-0.011619	0.019307	0.002801	0.014050
	0.035767	0.031008	-0.019374	0.014852	0.014390	-0.001388
	-0.011282	0.016878	0.005936	0.021646	-0.007989	0.012313
	0.016976	0.012818	0.051404	-0.042346	0.000849	-0.001271
	0.013797					
y418	-0.029042	0.012595	0.006075	0.004086	-0.019006	-0.035499
	-0.029129	0.005531	0.020554	-0.019333	0.008162	0.008357
	0.011375	-0.005348	0.011383	0.010054	-0.028624	0.002902
	-0.055397	0.013730	-0.019038	-0.005252	-0.037268	0.012483
	0.018631	0.012442	0.036527	-0.063695	0.012326	-0.020422
	0.035135					
z418	-0.012467	0.005687	-0.013884	0.003641	0.020320	0.023669
	0.015652	0.044974	0.025130	0.012741	-0.014640	-0.006923
	0.031810	0.039481	-0.021386	-0.022889	-0.000827	-0.055258
	-0.008732	-0.001008	-0.029380	0.007493	-0.023393	0.018315
	0.026311	-0.012989	-0.005454	0.002074	-0.000557	0.007009
	-0.008818					
x419	-0.008828	-0.018625	0.020054	0.025501	0.001261	0.016523
	-0.044840	0.044710	-0.008957	0.010078	0.002401	0.010193
	0.034185	0.038376	-0.016096	0.010148	0.013360	-0.001579
	-0.014158	0.020127	0.004389	0.021297	-0.005032	0.006265
	0.009733	0.022623	0.058362	-0.034817	0.003860	-0.005562
	0.019768					
y419	-0.025844	0.010778	0.004079	0.004578	-0.019439	-0.034272
	-0.025388	0.006606	0.024747	-0.020966	0.012887	0.003870
	0.006005	-0.002891	0.010673	0.010771	-0.021665	-0.000333
	-0.051375	0.015198	-0.019870	-0.007709	-0.033099	0.012797
	0.011327	0.026924	0.041615	-0.054232	0.012389	-0.015374
	0.037632					

z419	-0.013652	0.006525	-0.014603	0.001681	0.023313	0.020558
	0.020638	0.043414	0.022572	0.012207	-0.019381	-0.005491
	0.029758	0.033454	-0.024469	-0.025202	-0.006162	-0.043295
	-0.006615	-0.010098	-0.028153	0.007110	-0.023837	0.011420
	0.027959	-0.023972	-0.014166	0.004714	-0.000229	-0.002183
	-0.007425					
x420	-0.011269	-0.014920	0.020204	0.026697	0.004399	0.013250
	-0.045551	0.043889	-0.008330	0.003541	0.001478	0.008019
	0.034570	0.044041	-0.012002	0.005167	0.011334	-0.002180
	-0.014236	0.022620	0.005708	0.029218	-0.002549	0.000398
	0.007488	0.026184	0.061507	-0.026968	0.013153	-0.004162
	0.013332					
y420	-0.023254	0.010830	0.000803	0.002854	-0.015664	-0.035442
	-0.022517	0.005607	0.022675	-0.018364	0.015032	0.004955
	0.003851	-0.006383	0.010044	0.010233	-0.020206	-0.004031
	-0.037282	0.012048	-0.018442	-0.007435	-0.027691	0.016621
	0.010778	0.025751	0.038998	-0.048350	0.024304	-0.021381
	0.018149					
z420	-0.013632	0.006026	-0.015338	0.002200	0.018987	0.021463
	0.027968	0.045110	0.024857	0.007523	-0.022533	-0.012628
	0.026836	0.032539	-0.026469	-0.025165	-0.003264	-0.032335
	-0.014025	-0.010149	-0.027280	0.009333	-0.024658	0.001716
	0.020802	-0.024600	-0.019227	0.008298	-0.013245	0.012522
	0.015510					
x421	-0.012949	-0.012929	0.020421	0.025872	0.006569	0.009627
	-0.045614	0.041212	-0.006034	-0.000017	0.000922	0.001836
	0.032963	0.047423	-0.009734	-0.001300	0.005354	-0.001546
	-0.020177	0.027774	0.006809	0.031338	-0.001587	-0.003284
	0.002225	0.031184	0.054371	-0.015485	0.021782	-0.010413
	0.018941					
y421	-0.020605	0.008861	-0.001394	0.001358	-0.015039	-0.034726
	-0.019615	0.005212	0.023493	-0.014221	0.019447	-0.000040
	0.001628	-0.008304	0.005708	0.007921	-0.019317	-0.004130
	-0.035048	0.010716	-0.018771	-0.007878	-0.031469	0.014013
	-0.000041	0.032991	0.021736	-0.042802	0.032292	-0.027500
	0.018888					
z421	-0.014983	0.008317	-0.019358	0.001687	0.015792	0.019275
	0.034579	0.043901	0.019440	0.001713	-0.029690	-0.007836
	0.025540	0.029464	-0.020425	-0.023255	0.000366	-0.027599
	-0.007137	-0.015015	-0.021118	0.002992	-0.011975	0.006105
	0.028516	-0.032529	-0.006907	0.013763	-0.022145	0.021882
	0.008917					

x422	-0.013887	-0.009371	0.019774	0.026452	0.008400	0.004410
	-0.043064	0.038707	-0.006587	-0.003478	0.000220	0.001546
	0.030675	0.048137	-0.005251	-0.004036	0.004755	-0.004500
	-0.021435	0.031585	0.011575	0.033428	0.001845	-0.001384
	0.002053	0.030713	0.060225	0.004493	0.023209	-0.005415
	0.019188					
y422	-0.017695	0.007394	-0.005266	0.001654	-0.013778	-0.035476
	-0.013791	0.003579	0.019468	-0.013244	0.020496	0.001405
	0.000726	-0.010097	0.005201	0.009443	-0.010937	-0.009554
	-0.028390	0.005415	-0.013777	-0.007904	-0.030277	0.018353
	-0.004579	0.034565	0.017295	-0.029124	0.032980	-0.027591
	0.014289					
z422	-0.016005	0.009400	-0.019621	-0.001216	0.010938	0.018773
	0.037412	0.046046	0.022874	0.004937	-0.033293	-0.016227
	0.022419	0.024083	-0.023176	-0.022230	-0.005726	-0.015905
	-0.005910	-0.011474	-0.026415	-0.002270	-0.010447	-0.003084
	0.021572	-0.037896	-0.020655	0.008319	-0.022365	0.021959
	0.010987					
x423	-0.015086	-0.007245	0.018582	0.026589	0.007896	0.000587
	-0.039318	0.035854	-0.006420	-0.003245	-0.000743	-0.003621
	0.027913	0.050325	-0.001238	-0.004707	0.003108	-0.002009
	-0.020827	0.038919	0.014332	0.034490	0.009952	0.001608
	0.009283	0.018945	0.058640	0.020147	0.026719	0.003291
	0.014280					
y423	-0.015897	0.004452	-0.009360	0.000821	-0.014699	-0.033824
	-0.008716	0.002040	0.015843	-0.010916	0.021082	-0.001418
	-0.000947	-0.014276	0.001223	0.008249	-0.006154	-0.013432
	-0.018727	0.003541	-0.015187	-0.011034	-0.022250	0.025212
	-0.008203	0.029014	0.004622	-0.022616	0.032909	-0.029558
	0.007713					
z423	-0.015806	0.014679	-0.019934	-0.002620	0.007613	0.015750
	0.041722	0.050741	0.023606	0.007419	-0.034871	-0.015673
	0.021088	0.018103	-0.021566	-0.016486	-0.004599	-0.009855
	-0.008805	-0.015009	-0.020160	-0.005413	-0.022952	-0.014047
	0.011689	-0.030489	-0.020140	0.006582	-0.027146	0.026987
	0.016264					
x424	-0.015483	-0.004556	0.018165	0.024763	0.006501	-0.004666
	-0.035288	0.034246	-0.006254	-0.001480	-0.004046	-0.005822
	0.022750	0.047030	-0.000197	-0.005726	-0.000678	-0.004057
	-0.025268	0.038521	0.013876	0.028846	0.018058	0.003183
	0.002874	0.013199	0.058937	0.033152	0.019200	-0.000678
	0.018717					

y424	-0.013418	0.002680	-0.013178	0.000970	-0.016385	-0.031448
	-0.002979	0.003020	0.013174	-0.009911	0.021757	-0.002756
	0.000163	-0.017885	-0.001198	0.008934	-0.003668	-0.017180
	-0.013401	-0.004582	-0.018474	-0.013843	-0.022816	0.027922
	-0.013033	0.033131	-0.010635	-0.015904	0.030729	-0.032442
	0.006084					
z424	-0.018524	0.019248	-0.020910	-0.006490	0.004732	0.013909
	0.042597	0.047077	0.018177	0.010024	-0.038382	-0.014819
	0.012689	0.010527	-0.021376	-0.014391	0.005546	-0.004199
	-0.001370	-0.005071	-0.008221	-0.014066	-0.009369	-0.001473
	0.015394	-0.051106	-0.022214	0.003798	-0.021207	0.037941
	0.007179					
x425	-0.015854	-0.002286	0.015252	0.022134	0.004501	-0.009486
	-0.031825	0.029159	-0.006986	0.004341	-0.007576	-0.010861
	0.014324	0.047391	0.004286	-0.001742	0.000834	-0.004136
	-0.027377	0.040483	0.014644	0.017692	0.032159	0.002504
	0.005836	-0.003279	0.060517	0.036268	0.026852	-0.018343
	0.017891					
y425	-0.011270	0.001567	-0.016461	0.001782	-0.018286	-0.026834
	0.002018	0.006078	0.012400	-0.011663	0.024069	-0.000864
	-0.000478	-0.025059	-0.005059	0.006091	-0.002419	-0.018128
	-0.008610	-0.011696	-0.022051	-0.014921	-0.024016	0.035655
	-0.018403	0.038657	-0.028043	-0.010444	0.023332	-0.022537
	0.007448					
z425	-0.019059	0.019957	-0.021173	-0.008381	-0.002353	0.011548
	0.045242	0.042111	0.013864	0.016786	-0.044936	-0.017087
	0.013813	0.002878	-0.022160	-0.005979	0.010733	-0.002371
	-0.003492	-0.001795	0.002802	-0.022029	-0.001320	-0.010616
	0.008873	-0.051733	-0.020089	0.008088	-0.014808	0.018309
	0.001300					
x426	-0.014855	-0.001868	0.009896	0.016421	0.003725	-0.013953
	-0.030365	0.025368	-0.011124	0.005595	-0.007589	-0.015633
	0.003776	0.051108	0.010924	0.002482	0.001835	-0.002155
	-0.027576	0.042363	0.016664	0.019571	0.039870	-0.003488
	0.012140	-0.009353	0.062115	0.038053	0.043647	-0.021876
	0.003546					
y426	-0.009195	0.001627	-0.018131	0.003379	-0.022250	-0.019772
	0.007674	0.007778	0.013082	-0.010377	0.025788	-0.000911
	0.000327	-0.034136	-0.008345	0.006143	-0.000029	-0.021166
	-0.003927	-0.019453	-0.020839	-0.022212	-0.020282	0.039616
	-0.026174	0.037913	-0.041966	-0.009604	0.014249	-0.021317
	0.018292					

z426	-0.018820	0.022206	-0.021948	-0.009752	-0.005557	0.009923
	0.045089	0.037523	0.007692	0.011907	-0.042922	-0.008788
	0.014613	-0.005180	-0.023400	0.004806	0.008145	-0.002227
	-0.007585	-0.011416	-0.006660	-0.032796	0.004949	-0.008637
	-0.004682	-0.048679	-0.047568	0.024883	-0.002149	0.027926
	-0.004115					
x427	-0.013709	-0.000981	0.006353	0.010128	0.001087	-0.015759
	-0.026655	0.024620	-0.013888	0.007878	-0.005347	-0.013437
	-0.003385	0.046663	0.013271	0.007086	0.002407	-0.006884
	-0.025232	0.036448	0.017879	0.022114	0.045173	-0.002078
	0.015243	-0.008299	0.048328	0.041868	0.042405	-0.019321
	0.006665					
y427	-0.007822	0.000357	-0.019936	0.005510	-0.024682	-0.013542
	0.012675	0.009844	0.014116	-0.011212	0.026509	-0.006341
	0.001494	-0.038351	-0.010611	0.006336	0.005024	-0.025245
	0.003557	-0.017251	-0.017405	-0.030979	-0.014922	0.040118
	-0.030953	0.041821	-0.046023	-0.009893	0.013342	-0.024899
	0.017106					
z427	-0.019073	0.025236	-0.023984	-0.010665	-0.006760	0.007604
	0.037683	0.035858	-0.002159	0.009583	-0.047726	-0.010488
	0.003309	-0.014529	-0.024732	0.002255	0.005187	0.004989
	-0.009339	-0.009265	-0.016042	-0.028112	0.003421	-0.015613
	-0.017977	-0.071311	-0.039064	0.029139	-0.006444	0.020762
	-0.015273					
x428	-0.012914	-0.001157	0.002343	0.003995	-0.000855	-0.016515
	-0.024452	0.025013	-0.015937	0.009479	-0.001949	-0.012923
	-0.008738	0.039523	0.016281	0.006227	0.005352	-0.010645
	-0.023763	0.031213	0.019828	0.025440	0.041585	-0.003208
	0.012534	-0.006924	0.033884	0.039272	0.033712	-0.022942
	0.004009					
y428	-0.006714	0.000059	-0.020666	0.007318	-0.027176	-0.006435
	0.018410	0.012554	0.014900	-0.011767	0.027671	-0.009719
	-0.000763	-0.041632	-0.010841	0.009955	0.007256	-0.024854
	0.009079	-0.013237	-0.008273	-0.038606	-0.007802	0.041836
	-0.032553	0.054068	-0.054227	-0.004616	0.017236	-0.025765
	0.027923					
z428	-0.020419	0.028475	-0.026123	-0.013061	-0.012654	0.009996
	0.030865	0.035565	-0.003205	0.008075	-0.048082	-0.017934
	-0.009925	-0.013328	-0.025690	0.011798	0.006660	0.015408
	-0.004895	-0.004364	0.004655	-0.021244	0.007049	-0.011197
	-0.012146	-0.056328	-0.051644	0.037669	-0.004172	-0.007066
	0.015324					

x429	-0.011417	-0.002357	-0.001305	-0.001226	-0.003663	-0.016761
	-0.020774	0.025180	-0.018241	0.011349	0.000463	-0.011026
	-0.015718	0.031847	0.019297	0.004477	0.005855	-0.014755
	-0.020849	0.023414	0.015957	0.026427	0.039620	-0.002666
	0.010675	-0.009366	0.022044	0.044229	0.028109	-0.025889
	0.001683					
y429	-0.006270	0.001255	-0.020833	0.009663	-0.027505	0.000268
	0.021018	0.012986	0.015433	-0.014171	0.027138	-0.012270
	0.003105	-0.046159	-0.010170	0.011043	0.011142	-0.023817
	0.010002	-0.012250	-0.005558	-0.048005	0.000251	0.041740
	-0.033782	0.052839	-0.054197	-0.008715	0.023278	-0.017223
	0.026496					
z429	-0.020856	0.028846	-0.026302	-0.014974	-0.014474	0.010372
	0.024172	0.031687	-0.006664	0.008157	-0.050584	-0.019012
	-0.012242	-0.015550	-0.011176	0.021275	0.009086	0.022784
	0.000201	-0.009099	0.006802	-0.019582	0.001546	-0.010691
	-0.003660	-0.060395	-0.052262	0.032619	-0.005189	-0.010988
	0.022251					
x430	-0.010471	-0.003460	-0.004800	-0.006532	-0.004757	-0.015473
	-0.018146	0.025975	-0.020225	0.012404	0.005711	-0.010881
	-0.016319	0.026176	0.021504	0.001536	0.010271	-0.019776
	-0.018395	0.016609	0.010167	0.023970	0.034415	-0.001605
	0.005584	-0.003889	0.008000	0.034126	0.022454	-0.024616
	-0.002327					
y430	-0.004925	0.002563	-0.020809	0.012320	-0.029302	0.005770
	0.024511	0.013185	0.013725	-0.016748	0.024627	-0.012064
	-0.000435	-0.052099	-0.009214	0.013735	0.010643	-0.021675
	0.010490	-0.011482	-0.001243	-0.052525	0.012434	0.038725
	-0.032056	0.043227	-0.050948	0.001960	0.031788	-0.014277
	0.028414					
z430	-0.019865	0.027468	-0.025957	-0.018728	-0.015060	0.006992
	0.017755	0.025448	-0.012668	0.006065	-0.048850	-0.014351
	-0.018970	-0.018884	-0.002975	0.023283	0.009474	0.027245
	0.000408	-0.016379	0.003777	-0.013128	-0.010291	-0.010243
	-0.011620	-0.074199	-0.061631	0.023631	-0.009446	-0.020153
	0.027519					
x431	-0.009374	-0.004937	-0.007462	-0.010126	-0.008036	-0.012516
	-0.015234	0.028358	-0.021102	0.011220	0.009578	-0.011091
	-0.018366	0.019157	0.023770	0.000223	0.010388	-0.027822
	-0.016791	0.012507	0.005580	0.025143	0.036908	0.005813
	0.006064	0.001765	-0.001433	0.030827	0.021689	-0.010190
	-0.003648					

y431	-0.004120	0.004496	-0.021596	0.012601	-0.027636	0.010525
	0.025238	0.012640	0.011841	-0.018118	0.024234	-0.012209
	0.001128	-0.053976	-0.009329	0.013113	0.016123	-0.017210
	0.013309	-0.010731	0.003806	-0.058807	0.014301	0.025317
	-0.037730	0.036794	-0.050384	0.003971	0.028655	-0.032305
	0.028341					
z431	-0.018190	0.025925	-0.026751	-0.022787	-0.011234	0.002592
	0.011242	0.023487	-0.019036	0.006189	-0.045019	-0.013559
	-0.022952	-0.017287	-0.001806	0.023495	0.017004	0.022273
	0.013618	-0.019717	0.008307	-0.010292	-0.025687	-0.017535
	-0.019246	-0.083533	-0.077708	0.028508	-0.025920	-0.055177
	0.031619					
x432	-0.008007	-0.006494	-0.010195	-0.014472	-0.010853	-0.008298
	-0.013738	0.029364	-0.022552	0.010996	0.013666	-0.012910
	-0.019429	0.009820	0.026250	-0.002117	0.011034	-0.035675
	-0.011058	0.007829	0.000138	0.020777	0.037509	0.014166
	0.005756	0.008084	-0.016922	0.027383	0.022380	-0.003844
	-0.006580					
y432	-0.003874	0.006450	-0.022173	0.011010	-0.024790	0.013793
	0.024785	0.010439	0.011078	-0.016977	0.023504	-0.014439
	0.000011	-0.053887	-0.002934	0.016333	0.025303	-0.007045
	0.021742	-0.013083	0.009536	-0.063878	0.012978	0.010187
	-0.038856	0.025776	-0.055782	0.012284	0.030238	-0.051025
	0.025286					
z432	-0.017734	0.023445	-0.025446	-0.028624	-0.007030	-0.001652
	0.002676	0.016866	-0.021255	0.006256	-0.040819	-0.019839
	-0.022330	-0.013204	0.000970	0.023959	0.022519	0.019894
	0.032208	-0.019721	0.013328	-0.006983	-0.039455	-0.013517
	-0.014821	-0.083576	-0.095001	0.023378	-0.035005	-0.060921
	0.030112					
x433	-0.005520	-0.008607	-0.013571	-0.018759	-0.014623	-0.001701
	-0.011771	0.031949	-0.024715	0.013350	0.022601	-0.014453
	-0.017830	-0.000020	0.027221	-0.005300	0.016055	-0.047926
	-0.006823	0.002223	-0.007393	0.015138	0.038012	0.016723
	0.001069	0.014967	-0.036493	0.024574	0.024599	-0.004614
	-0.013386					
y433	-0.003975	0.008191	-0.020314	0.008996	-0.023558	0.019953
	0.024078	0.009107	0.012446	-0.019870	0.021639	-0.010689
	-0.002222	-0.052895	0.004143	0.023487	0.031105	0.000956
	0.015375	-0.015035	0.014802	-0.055401	0.008936	-0.005591
	-0.040982	0.020260	-0.048597	0.008264	0.029408	-0.051929
	0.021864					

z433	-0.016546	0.020820	-0.024703	-0.033041	-0.007436	0.000042
	-0.006294	0.016958	-0.023155	0.005103	-0.025643	-0.018098
	-0.014786	-0.008319	-0.000162	0.023189	0.016300	0.006009
	0.026770	-0.014278	0.012189	-0.002770	-0.044411	-0.002288
	-0.004772	-0.071608	-0.095920	0.009937	-0.045825	-0.054220
	0.015801					
x434	-0.002691	-0.011705	-0.017426	-0.023368	-0.020702	0.008514
	-0.008236	0.038753	-0.025401	0.011644	0.027471	-0.015626
	-0.007798	-0.001548	0.024718	-0.004769	0.019895	-0.054404
	-0.006272	0.002056	-0.016469	0.015551	0.045667	0.030756
	0.005175	0.033414	-0.027824	0.014434	0.014624	0.009619
	-0.011965					
y434	-0.002095	0.007650	-0.018448	0.007692	-0.025899	0.027076
	0.021297	0.006579	0.013494	-0.021884	0.019710	-0.009097
	-0.000100	-0.041471	0.017401	0.028030	0.030952	-0.006750
	0.010017	-0.017241	0.011667	-0.031171	0.020971	-0.007453
	-0.016189	0.034257	-0.019465	-0.008019	0.008138	-0.030407
	0.013361					
z434	-0.014812	0.017321	-0.027796	-0.038750	-0.007868	-0.000701
	-0.022147	0.016726	-0.024784	0.007070	-0.012312	-0.019635
	-0.010520	0.004059	0.006498	0.024837	0.012928	-0.011429
	0.017924	-0.012762	0.000119	0.007005	-0.050143	0.021579
	0.016975	-0.037742	-0.083690	0.005229	-0.080707	-0.046028
	-0.013649					
x435	-0.000307	-0.014772	-0.021142	-0.030061	-0.022214	0.012673
	-0.000498	0.043390	-0.027253	0.011661	0.027399	-0.019569
	-0.001022	-0.005146	0.016333	-0.001816	0.020918	-0.045982
	0.007797	0.002299	-0.022451	0.014324	0.051880	0.024103
	-0.003367	0.033855	-0.020366	0.011510	0.030657	0.005294
	0.002781					
y435	0.000738	0.006469	-0.022159	0.001430	-0.032482	0.025379
	0.004737	-0.002899	0.016079	-0.017743	0.019751	-0.010786
	-0.007071	-0.028956	0.042751	0.018786	0.032641	-0.033651
	0.012629	-0.020457	-0.016347	0.007923	0.022254	0.005492
	0.002147	0.057457	-0.008880	-0.012207	-0.001660	-0.017630
	-0.015783					
z435	-0.015974	0.013482	-0.031655	-0.042548	-0.017692	0.002628
	-0.030950	0.008165	-0.020088	0.012589	-0.016071	-0.008996
	-0.019927	0.016062	0.024451	0.025274	0.029882	-0.017710
	0.002930	-0.024757	-0.030084	0.019827	-0.055969	0.040104
	0.013956	-0.021801	-0.056759	0.019141	-0.085832	-0.026683
	-0.040975					

x436	0.003424	-0.020982	-0.028354	-0.038713	-0.029756	0.019990
	0.005493	0.044211	-0.026196	0.014222	0.025882	-0.020884
	0.006099	-0.006025	0.010653	0.001587	0.034280	-0.047282
	0.013808	0.003084	-0.034452	0.020962	0.050525	0.032715
	-0.015365	0.046998	-0.009535	0.025527	0.042079	0.012140
	0.003331					
y436	0.001051	0.006057	-0.021200	0.000064	-0.031523	0.020282
	0.002073	-0.009331	0.017292	-0.016406	0.015570	-0.007383
	-0.003192	-0.017546	0.042869	0.018854	0.031809	-0.027227
	0.014253	-0.024087	-0.028953	0.027327	0.030855	0.019768
	0.003526	0.060038	0.005262	-0.004036	0.007802	-0.008010
	-0.026915					
z436	-0.013621	0.006857	-0.039255	-0.043548	-0.024165	-0.003341
	-0.039428	-0.004022	-0.020679	0.011765	-0.011425	0.001174
	-0.028035	0.011870	0.043119	0.013591	0.041558	-0.025417
	-0.000588	-0.035363	-0.042125	0.033950	-0.049674	0.064218
	0.011094	-0.002770	-0.053141	0.032996	-0.060567	0.002571
	-0.063285					
x437	0.005795	-0.024337	-0.030931	-0.046185	-0.032766	0.027634
	0.009245	0.046353	-0.022860	0.015733	0.023019	-0.025093
	0.009285	-0.002672	0.006239	0.008077	0.050317	-0.049530
	0.016812	0.001916	-0.042169	0.026463	0.041114	0.026116
	-0.033537	0.039167	-0.012182	0.030426	0.041904	0.007451
	0.007263					
y437	-0.001196	0.007907	-0.015052	0.000931	-0.022446	0.018960
	0.001040	-0.013023	0.020298	-0.015301	0.009882	-0.007804
	-0.005221	-0.009654	0.038105	0.021158	0.033250	-0.021101
	0.006237	-0.025738	-0.023390	0.021713	0.021487	0.014906
	-0.005664	0.034829	0.003435	-0.000087	0.010168	-0.004360
	0.008145					
z437	-0.012414	0.002509	-0.044255	-0.045916	-0.025582	-0.009811
	-0.041379	-0.010928	-0.020257	0.007117	-0.004538	0.003671
	-0.032800	0.012670	0.044250	0.004769	0.051055	-0.025399
	0.001763	-0.042280	-0.038531	0.041914	-0.044666	0.063289
	0.000723	-0.001807	-0.040779	0.027587	-0.020395	0.022829
	-0.012361					
x438	0.009013	-0.025010	-0.031657	-0.047544	-0.031840	0.041287
	0.012307	0.048957	-0.022056	0.016914	0.019144	-0.028838
	0.015794	-0.010000	0.015199	0.014012	0.058908	-0.055930
	0.009715	0.001043	-0.047959	0.027673	0.032448	0.016517
	-0.028859	0.047346	-0.009277	0.042331	0.016025	0.008168
	-0.019611					

y438	-0.002509	0.012141	-0.010078	0.005412	-0.015194	0.020324
	-0.000001	-0.016455	0.019663	-0.017286	0.003993	-0.008399
	-0.006439	-0.005278	0.041259	0.021476	0.031442	-0.011418
	-0.003010	-0.025349	-0.014921	0.024090	0.014405	0.006515
	-0.009710	0.021833	0.003506	0.003562	0.001487	0.002966
	0.017178					
z438	-0.010643	0.001455	-0.043643	-0.042221	-0.028367	-0.011412
	-0.037296	-0.008895	-0.020498	0.008549	0.001711	0.011965
	-0.029326	0.013018	0.034593	0.007291	0.051101	-0.004552
	0.009095	-0.041790	-0.050173	0.061194	-0.031426	0.046031
	-0.008322	0.011623	-0.036634	0.025155	-0.005332	0.009594
	-0.058638					
x439	0.007779	-0.028285	-0.033395	-0.048618	-0.030195	0.047964
	0.008413	0.046315	-0.025935	0.019058	0.014087	-0.025629
	0.014698	-0.016515	0.012889	0.015215	0.056599	-0.069753
	0.002654	0.000573	-0.047048	0.028992	0.034330	0.011183
	-0.032002	0.049995	-0.009919	0.048997	-0.012270	0.006703
	-0.034895					
y439	-0.006081	0.015844	-0.005562	0.011069	-0.006698	0.019231
	-0.004397	-0.022145	0.016554	-0.020240	-0.001491	-0.006618
	-0.012193	-0.004386	0.045910	0.019326	0.021697	0.000689
	-0.010260	-0.025531	-0.000769	0.024461	0.012643	0.001839
	-0.010553	0.004391	-0.000747	0.002808	-0.015252	0.007271
	0.008351					
z439	-0.012292	-0.002085	-0.044868	-0.040230	-0.028126	-0.013567
	-0.036397	-0.010051	-0.025731	0.006004	0.006939	0.021408
	-0.030622	0.010713	0.021626	0.002306	0.049775	0.003139
	0.012373	-0.037027	-0.041020	0.048771	-0.020808	0.046119
	-0.004986	0.008451	-0.020989	0.019678	0.020183	0.018451
	-0.057094					
x440	0.006210	-0.029246	-0.032667	-0.047459	-0.033397	0.051346
	0.005291	0.049789	-0.024849	0.022155	0.013676	-0.018246
	0.015011	-0.021589	0.002515	0.002647	0.044861	-0.066634
	0.009992	-0.000829	-0.059383	0.032909	0.058555	0.001455
	-0.035794	0.033478	-0.009152	0.029609	-0.006096	0.005831
	-0.030963					
y440	-0.008656	0.019152	-0.001623	0.021057	-0.002991	0.017188
	-0.007525	-0.025014	0.012606	-0.019704	-0.003218	-0.003091
	-0.022843	-0.002465	0.047702	0.008459	0.013028	0.011646
	-0.020700	-0.026753	0.010827	0.032378	0.012452	-0.021553
	-0.014619	-0.013983	-0.014706	-0.004572	-0.018248	0.024997
	0.014396					

z440	-0.011687	-0.006762	-0.042434	-0.031823	-0.029245	-0.010182
	-0.027076	-0.006076	-0.025183	0.004441	0.008389	0.025972
	-0.036182	-0.000745	0.004250	-0.009269	0.042940	0.007274
	0.006069	-0.029711	-0.033770	0.041667	-0.011712	0.024169
	-0.012224	-0.002227	-0.011541	0.004337	0.033581	0.020620
	-0.009814					
x441	0.005543	-0.029142	-0.030024	-0.043018	-0.034085	0.054694
	0.006530	0.049214	-0.018545	0.019221	0.009176	-0.014510
	0.017231	-0.020068	0.000427	-0.009124	0.042816	-0.061664
	0.007206	-0.007205	-0.060073	0.016977	0.069076	-0.005481
	-0.032359	0.027474	0.001957	0.022321	-0.000796	-0.001360
	-0.018684					
y441	-0.011245	0.019833	0.001556	0.026688	0.004940	0.008507
	-0.014579	-0.034514	0.014967	-0.020513	-0.003099	-0.003720
	-0.031282	-0.005896	0.063732	-0.000884	0.006859	0.015131
	-0.028369	-0.040593	0.016760	0.029894	0.021508	-0.022292
	0.003327	-0.004404	-0.021326	-0.004781	-0.032390	0.026465
	0.005628					
z441	-0.012310	-0.011099	-0.038382	-0.026527	-0.022974	-0.004983
	-0.014529	-0.000584	-0.020161	-0.005990	0.002998	0.029472
	-0.028888	-0.015141	-0.003319	-0.020401	0.034698	0.017373
	0.000201	-0.027956	-0.027954	-0.001314	-0.011879	0.026698
	0.000362	-0.012715	0.003349	-0.010339	0.049354	-0.003893
	0.039891					
x442	0.005639	-0.026109	-0.022796	-0.037228	-0.024875	0.061003
	-0.001735	0.048975	-0.011456	0.012570	0.004486	-0.017802
	0.026507	-0.007501	0.011129	-0.009589	0.043770	-0.059088
	0.006121	-0.007247	-0.039985	0.015798	0.048712	-0.015533
	-0.025401	0.013502	-0.001177	0.046188	-0.012636	0.005479
	-0.044583					
y442	-0.016263	0.021448	0.003397	0.025919	-0.000445	0.001972
	-0.021705	-0.032343	0.011999	-0.012391	0.010779	0.003839
	-0.038454	0.000288	0.045608	-0.009036	-0.012808	0.041186
	-0.019265	-0.032784	0.001457	0.020499	0.033259	-0.018034
	0.034509	-0.003789	-0.014033	-0.022864	-0.004101	0.011913
	-0.004342					
z442	-0.011429	-0.009477	-0.030614	-0.025812	-0.004174	0.000012
	-0.013660	0.007427	-0.013574	-0.022436	-0.009253	0.023773
	-0.010009	-0.015434	-0.001755	-0.010583	0.032134	0.020278
	-0.008834	-0.014726	-0.017806	-0.013399	-0.029709	0.017521
	-0.002381	-0.018844	0.004124	-0.002750	0.059960	-0.009979
	0.034625					

x443	0.003812	-0.024476	-0.013021	-0.035680	-0.023041	0.063891
	-0.015447	0.043980	-0.007826	-0.002400	-0.002773	0.003017
	0.032824	-0.018277	0.007717	0.001084	0.035735	-0.043131
	0.013486	0.012717	-0.030405	0.023702	0.052763	-0.049015
	-0.011439	-0.009518	-0.019532	0.038926	-0.011677	-0.025386
	-0.044780					
y443	-0.016604	0.021567	0.003242	0.020005	-0.000072	-0.007198
	-0.030377	-0.034058	0.017668	-0.011540	0.006874	-0.000056
	-0.037596	0.024925	0.034673	-0.001243	0.002827	0.055093
	-0.006981	-0.043827	-0.002909	0.023656	0.049443	-0.002622
	0.024095	0.020201	-0.003144	0.004564	0.011785	0.012440
	-0.017977					
z443	-0.010845	-0.008034	-0.026197	-0.036372	0.008134	0.011893
	-0.017488	0.011684	-0.013821	-0.022409	-0.020400	0.018227
	-0.018800	-0.043518	-0.005001	0.002855	0.021284	0.022095
	-0.017490	0.007305	-0.000802	-0.007746	-0.011218	-0.015808
	-0.002933	-0.035177	-0.002987	0.003830	0.031748	-0.007265
	0.009470					
x444	0.003301	-0.025856	-0.006732	-0.049306	-0.018344	0.056428
	-0.028993	0.037169	-0.000127	0.002222	-0.023721	0.005032
	0.044131	-0.026531	0.019524	-0.015949	0.053305	-0.061506
	-0.007842	0.023433	0.001426	-0.006575	0.052176	-0.103521
	0.027352	0.023017	0.003970	0.050747	-0.001046	0.010947
	-0.013750					
y444	-0.012018	0.020402	0.001607	0.021242	0.009678	-0.010260
	-0.028084	-0.040294	0.016755	-0.012913	0.024522	-0.003387
	-0.031244	0.019392	0.037312	-0.010309	0.000140	0.045253
	0.006566	-0.060952	-0.005963	0.040767	0.070090	-0.014736
	0.025784	0.029888	-0.000604	0.016241	-0.002727	0.003276
	-0.021885					
z444	-0.009756	-0.008733	-0.017033	-0.045025	0.010708	0.014897
	-0.018108	0.013044	-0.001218	-0.019254	-0.015568	0.005658
	-0.012366	-0.060419	0.003450	-0.014842	0.035567	0.002313
	-0.032952	0.009961	0.002691	-0.002978	-0.019371	-0.060227
	-0.008611	-0.000850	0.013425	0.019902	0.043956	-0.007382
	0.004424					
x445	-0.001690	-0.025357	-0.003869	-0.045341	-0.011866	0.052525
	-0.035343	0.027202	-0.001093	0.005307	-0.013824	-0.020087
	0.029035	-0.035798	0.031498	-0.016176	0.042476	-0.061812
	-0.024328	-0.006908	-0.020810	0.026935	0.047854	-0.090813
	0.024878	0.044423	0.001023	0.059341	-0.007035	0.002919
	-0.000658					

y445	-0.012389	0.018157	-0.005054	0.020092	0.006883	-0.015015
	-0.017414	-0.040101	0.015613	-0.003794	0.019983	-0.017558
	-0.030347	0.014489	0.044853	-0.018314	0.020880	0.024366
	-0.004048	-0.074312	-0.008728	0.055428	0.060518	-0.010830
	0.021050	0.030775	0.006685	0.029364	-0.032690	0.012623
	-0.002790					
z445	-0.013864	-0.006093	-0.014146	-0.048527	0.013683	0.012102
	-0.006617	0.011526	0.004356	-0.008539	0.000689	-0.020158
	-0.011606	-0.082192	0.008973	-0.012104	0.032515	-0.014192
	-0.044470	-0.000918	-0.016783	0.027488	-0.022108	-0.045191
	-0.033909	0.003612	0.025075	0.027477	0.029441	-0.005185
	0.012361					
x446	-0.007704	-0.018915	-0.002744	-0.032775	-0.011262	0.047434
	-0.038813	0.015923	0.004174	-0.004561	-0.012989	-0.015135
	0.004711	-0.016830	0.018200	-0.004235	0.029240	-0.030824
	-0.032014	-0.016013	-0.006196	0.035015	0.046563	-0.057654
	0.023875	0.019689	-0.012206	0.052196	0.004132	-0.017042
	-0.000178					
y446	-0.014663	0.018358	-0.011346	0.020014	0.000998	-0.017020
	-0.001825	-0.036698	0.010217	-0.005810	0.012715	-0.015648
	-0.017865	0.015995	0.052375	-0.025073	0.037455	0.021907
	-0.014370	-0.059505	-0.001100	0.056409	0.055453	-0.007813
	0.007108	-0.000655	0.015529	0.044427	-0.054913	0.002659
	0.013198					
z446	-0.015577	-0.001983	-0.013541	-0.035701	0.015704	-0.004476
	0.003544	0.017222	0.005014	-0.009579	0.016270	-0.019505
	-0.029470	-0.062026	-0.002239	-0.016286	0.006508	-0.008578
	-0.015715	-0.005761	-0.008019	0.027826	-0.022735	-0.005022
	-0.027127	-0.018830	0.030727	-0.007325	0.033563	-0.013435
	-0.015045					
x447	-0.009763	-0.016520	-0.000033	-0.026104	-0.013849	0.047372
	-0.041074	0.013127	0.007624	0.001075	-0.013936	-0.020021
	-0.004762	-0.006598	0.010517	0.002304	0.020645	-0.023303
	-0.030457	-0.022998	-0.021166	0.037605	0.036832	-0.033708
	0.024030	0.022068	-0.019860	0.051264	-0.015182	-0.041563
	0.008605					
y447	-0.015511	0.017240	-0.014973	0.017758	-0.000763	-0.023549
	0.006135	-0.036332	0.001977	-0.010280	0.018135	-0.009556
	-0.008247	0.020254	0.051352	-0.021965	0.033633	0.018644
	-0.015175	-0.055590	0.004722	0.057612	0.055918	-0.002000
	-0.002824	-0.019020	0.018698	0.043287	-0.034286	-0.004545
	0.012316					

z447	-0.016024	0.000400	-0.013951	-0.026625	0.016391	-0.012589
	0.002109	0.011545	0.004796	-0.015770	0.023512	0.006406
	-0.034544	-0.044209	0.003743	-0.023372	-0.001026	0.006548
	0.005951	0.017247	0.011181	0.016889	-0.010827	-0.005156
	-0.002601	-0.034566	0.015833	-0.021680	0.048697	-0.016709
	-0.006808					
x448	-0.013566	-0.014190	0.000130	-0.022923	-0.009243	0.028065
	-0.042650	0.007939	0.007639	0.004576	-0.006353	-0.008441
	-0.019587	0.008932	0.001667	0.016610	0.008287	-0.017872
	-0.018120	-0.024448	-0.024704	0.036760	0.034870	-0.011883
	0.026765	0.011109	-0.033672	0.042727	0.005360	-0.058830
	0.007385					
y448	-0.013417	0.010570	-0.022573	0.012258	-0.000222	-0.027723
	0.014065	-0.036429	-0.009869	-0.009143	0.017020	-0.012927
	0.001072	0.017056	0.050484	-0.018282	0.026856	0.007038
	-0.009117	-0.040984	0.002953	0.056801	0.054547	-0.001086
	-0.020784	-0.034396	0.018553	0.040242	-0.029538	0.004026
	0.013994					
z448	-0.014250	0.005626	-0.009610	-0.026100	0.016994	-0.014301
	0.002725	0.018286	0.013873	-0.014137	0.029716	0.028289
	-0.033800	-0.033174	0.005198	-0.014886	0.000193	0.013724
	0.013213	0.020030	0.009601	0.015881	0.002702	-0.003124
	0.013732	-0.026874	-0.003244	-0.020076	0.033816	-0.022538
	0.017539					
x449	-0.016224	-0.014681	-0.005109	-0.029184	-0.005035	0.002812
	-0.044435	0.002462	0.005503	0.007451	-0.006113	-0.013764
	-0.030038	0.013196	-0.004234	0.022654	0.002087	-0.022417
	-0.005965	-0.015642	-0.024889	0.038601	0.034792	0.000128
	0.028496	0.009380	-0.031264	0.047278	0.030141	-0.048527
	-0.003078					
y449	-0.008028	0.005606	-0.032345	0.005489	0.001795	-0.027505
	0.023959	-0.033867	-0.021694	-0.010391	0.007183	-0.013327
	0.010877	0.008939	0.046876	-0.008810	0.018959	-0.004894
	-0.003245	-0.036001	0.003461	0.056946	0.062959	-0.006706
	-0.029589	-0.046167	0.004804	0.029258	-0.035841	0.011221
	0.029656					
z449	-0.013695	0.010786	-0.002666	-0.025829	0.016151	-0.012573
	0.001471	0.018330	0.019143	-0.012491	0.040483	0.044091
	-0.031019	-0.024619	0.011893	-0.010284	-0.000706	0.018614
	0.013868	0.015004	0.005436	0.016836	0.011056	-0.002382
	0.018057	-0.019883	-0.019224	-0.012188	0.020622	-0.020280
	0.005321					

x450	-0.018355	-0.016476	-0.015674	-0.040537	0.002692	-0.034515
	-0.039288	-0.003460	0.002025	0.006119	-0.009833	-0.020034
	-0.036944	0.015118	-0.022612	0.027243	0.002803	-0.035199
	0.013527	-0.002437	-0.014412	0.042554	0.028568	0.012141
	0.023242	0.005969	-0.019723	0.041820	0.052583	-0.029319
	-0.008321					
y450	-0.000630	-0.003096	-0.044766	-0.007965	0.005692	-0.030972
	0.032166	-0.029016	-0.037058	-0.001991	-0.008744	-0.015005
	0.028343	0.000177	0.048069	0.012898	-0.007194	-0.012451
	-0.005966	-0.027111	-0.006382	0.048743	0.077394	-0.017660
	-0.023901	-0.051163	-0.017138	0.039196	-0.047846	0.027504
	0.031550					
z450	-0.012882	0.016397	0.006470	-0.020565	0.017531	-0.013645
	0.002581	0.012830	0.023618	-0.008286	0.062139	0.064919
	-0.024517	-0.008759	0.016183	-0.013581	0.002337	0.029673
	0.017144	0.002689	-0.008640	0.027559	0.008004	0.006667
	0.016791	0.000277	-0.027396	-0.007219	0.005210	-0.038100
	-0.015343					
x451	-0.022034	-0.022849	-0.028344	-0.055537	0.023330	-0.085686
	-0.032865	-0.015648	-0.002979	0.007399	-0.013650	-0.030142
	-0.042450	0.023480	-0.055042	0.035417	0.001910	-0.056603
	0.029357	0.033791	0.001333	0.037207	0.007597	0.030020
	-0.002393	-0.006123	0.013563	0.008589	0.038958	-0.009370
	-0.017779					
y451	0.004510	-0.014089	-0.053741	-0.022262	0.019210	-0.042135
	0.044992	-0.015074	-0.057466	0.019765	-0.020324	0.012642
	0.054915	0.005951	0.047141	0.043556	-0.050111	-0.006871
	-0.024668	-0.012952	-0.035182	0.007509	0.057384	-0.010070
	0.001774	-0.025449	-0.019059	0.058202	-0.037113	0.016867
	0.007718					
z451	-0.006783	0.020591	0.013015	-0.026289	0.016503	-0.022060
	0.006238	0.005484	0.028629	0.000879	0.077799	0.080714
	-0.003635	0.005995	0.018867	-0.017322	0.013129	0.031550
	0.028883	-0.018133	-0.021281	0.055598	0.014920	-0.000235
	0.016395	0.015318	-0.038833	0.008542	-0.032595	-0.034804
	-0.021701					
x452	-0.025898	-0.026340	-0.033214	-0.059208	0.043830	-0.111714
	-0.034396	-0.025189	0.004387	0.010214	-0.010410	-0.037043
	-0.044025	0.017649	-0.072778	0.038180	0.010059	-0.076489
	0.027058	0.041687	0.042530	0.019290	-0.013785	0.041869
	0.006951	-0.008234	0.035791	-0.015274	0.023504	0.018200
	-0.022160					

y452	0.006110	-0.017764	-0.046329	-0.025233	0.027203	-0.043025
	0.047980	0.002275	-0.061841	0.046305	-0.037910	0.047265
	0.070904	0.010548	0.044155	0.047745	-0.062503	0.010971
	-0.051731	0.015002	-0.055060	-0.008865	0.032274	0.003135
	0.023195	-0.009167	0.001470	0.044574	0.000452	-0.027938
	0.016831					
z452	-0.002212	0.018280	0.006730	-0.034654	0.019475	-0.034979
	0.015920	-0.005566	0.015074	0.003514	0.077951	0.071433
	0.017222	0.018579	0.017238	-0.028613	0.018666	0.025398
	0.041692	-0.038044	-0.010783	0.067612	0.009090	-0.008133
	0.006710	0.002035	-0.035991	0.018623	-0.071280	-0.010525
	-0.045429					
x453	-0.034224	-0.028536	0.000550	-0.128541	-0.138289	0.034483
	-0.036233	-0.118325	0.047732	0.137496	0.063746	-0.011612
	-0.011631	0.006891	-0.037809	-0.050655	0.024784	0.016899
	-0.015009	0.016717	-0.107599	-0.032006	-0.012589	0.053364
	-0.058915	-0.010060	0.056777	0.032722	-0.000217	-0.016928
	0.012754					
y453	0.005240	0.090872	0.033907	0.022640	-0.008981	-0.035576
	-0.027009	0.026243	-0.012794	0.011099	0.092836	0.063601
	0.126314	0.071648	-0.095168	-0.114100	0.037501	0.048322
	-0.031772	0.028011	-0.060050	0.060736	0.069413	0.038957
	0.038818	0.016783	-0.047117	0.023386	0.011561	0.057981
	0.034129					
z453	0.034268	-0.015024	-0.109997	0.125949	0.070264	-0.056825
	-0.039556	-0.048875	0.038641	0.104401	0.012377	0.016973
	-0.138331	-0.002762	0.064953	-0.010399	0.062793	0.036777
	0.082824	0.052867	-0.001318	-0.036379	0.017959	0.044025
	-0.028711	-0.047590	0.072108	0.027130	0.071302	0.028762
	0.032890					
x454	-0.033269	-0.024023	0.001670	-0.126071	-0.136207	0.035901
	-0.037034	-0.117488	0.046305	0.133896	0.059114	-0.020743
	-0.007556	0.002099	-0.040108	-0.046957	0.030745	0.016516
	-0.020451	0.020027	-0.098546	-0.014230	-0.013485	0.046410
	-0.061159	-0.009440	0.061261	0.028490	0.001448	-0.020459
	0.021500					
y454	0.007681	0.089860	0.029494	0.021923	-0.005138	-0.036497
	-0.022142	0.023905	-0.011962	-0.000280	0.079073	0.052707
	0.112391	0.056739	-0.085863	-0.107944	0.024380	0.040897
	-0.018984	0.019131	-0.050579	0.051823	0.067393	0.046398
	0.028547	0.025633	-0.041867	0.025764	0.014592	0.043961
	0.023380					

z454	0.028348	-0.015245	-0.103694	0.122924	0.079304	-0.056590
	-0.031961	-0.037762	0.037205	0.091521	0.008331	0.028295
	-0.119243	-0.001356	0.064522	-0.010136	0.055224	0.028098
	0.073657	0.058613	-0.002735	-0.047551	0.017161	0.041009
	-0.042341	-0.037702	0.055365	0.019268	0.032762	0.015418
	0.024879					
x455	-0.032521	-0.020350	0.003338	-0.119057	-0.134468	0.035137
	-0.034675	-0.114705	0.047181	0.125747	0.050316	-0.028384
	-0.005459	-0.006167	-0.035883	-0.047596	0.038998	0.017638
	-0.024397	0.019932	-0.078629	-0.000575	-0.014222	0.044109
	-0.064422	-0.005494	0.068613	0.028441	0.011169	-0.022483
	0.021827					
y455	0.008809	0.086995	0.024786	0.017678	-0.001035	-0.037128
	-0.019772	0.021124	-0.008269	-0.009204	0.066410	0.040117
	0.097722	0.037933	-0.072919	-0.098938	0.007057	0.033935
	-0.012464	0.008727	-0.038897	0.044618	0.059932	0.048705
	0.017211	0.027046	-0.027338	0.023682	0.023928	0.030570
	0.005752					
z455	0.022127	-0.015853	-0.097278	0.117200	0.087938	-0.054648
	-0.022851	-0.025995	0.037143	0.080309	0.005297	0.041643
	-0.105157	-0.002612	0.065157	-0.001395	0.038562	0.013984
	0.057748	0.063259	-0.010033	-0.054233	0.022990	0.029705
	-0.057196	-0.035808	0.047314	0.017198	0.009513	0.008698
	0.013381					
x456	-0.031394	-0.016770	0.005986	-0.106939	-0.127556	0.031117
	-0.028520	-0.107265	0.043484	0.108411	0.039921	-0.031173
	-0.004798	-0.004835	-0.028811	-0.046511	0.029757	0.014138
	-0.025520	0.009560	-0.062177	-0.000824	-0.016053	0.036287
	-0.065840	0.000343	0.060647	0.022173	0.007362	-0.018415
	0.025709					
y456	0.012947	0.079223	0.018726	0.015644	-0.000878	-0.036873
	-0.015728	0.014717	-0.007872	-0.017841	0.054891	0.027960
	0.078553	0.026065	-0.065212	-0.091015	-0.007936	0.026340
	-0.008857	-0.000070	-0.034445	0.040160	0.041830	0.030576
	-0.002269	0.023230	-0.027152	0.018409	0.011526	0.032099
	-0.002203					
z456	0.015927	-0.018356	-0.090798	0.112446	0.091061	-0.050279
	-0.014014	-0.012108	0.042655	0.078715	-0.000188	0.054192
	-0.089472	-0.004491	0.062930	0.002075	0.037957	0.007377
	0.044294	0.087060	-0.021231	-0.036361	0.022885	-0.003886
	-0.062353	-0.031105	0.045642	0.001146	-0.014817	-0.006024
	0.025594					

x457	-0.030965	-0.014567	0.007585	-0.094298	-0.116847	0.025498
	-0.022782	-0.096475	0.042609	0.091333	0.030867	-0.030494
	-0.007261	-0.007167	-0.019674	-0.038293	0.017105	0.011274
	-0.021426	0.010548	-0.045544	-0.004100	-0.017383	0.033489
	-0.060085	0.008816	0.050789	0.018849	0.007607	-0.019925
	0.021056					
y457	0.016288	0.069188	0.016122	0.009455	0.002184	-0.034986
	-0.013596	0.007107	-0.008205	-0.026645	0.044872	0.013484
	0.061569	0.011479	-0.053659	-0.082083	-0.014566	0.018499
	0.000496	-0.006416	-0.030947	0.027059	0.028451	0.031537
	-0.013704	0.010376	-0.025109	0.015543	-0.003591	0.034021
	-0.017921					
z457	0.009490	-0.019900	-0.082371	0.098262	0.090477	-0.042720
	-0.007109	-0.003186	0.055851	0.073781	0.004005	0.054818
	-0.069296	-0.019219	0.065285	0.013250	0.033331	0.001363
	0.033450	0.097333	-0.022151	-0.029747	0.036948	-0.008854
	-0.063879	-0.017447	0.036710	0.007432	-0.014850	-0.028020
	0.029119					
x458	-0.029079	-0.012601	0.008976	-0.076976	-0.105583	0.018158
	-0.014939	-0.082007	0.041317	0.074002	0.021323	-0.024061
	-0.002880	-0.009238	-0.009440	-0.028187	-0.000920	0.014016
	-0.018101	0.018345	-0.033514	-0.003213	-0.019297	0.019956
	-0.054663	0.024388	0.037364	0.026183	-0.000001	-0.024942
	0.021763					
y458	0.018518	0.058030	0.017075	0.001188	0.008067	-0.030728
	-0.012469	-0.001046	-0.005859	-0.029715	0.037200	0.000055
	0.047573	-0.003810	-0.040593	-0.073686	-0.013786	0.014399
	0.003555	-0.008452	-0.027151	0.011973	0.017553	0.030884
	-0.026325	0.005261	-0.020815	0.006858	-0.011192	0.028777
	-0.033253					
z458	0.002925	-0.024082	-0.077838	0.080072	0.088239	-0.032754
	0.002265	0.001780	0.066822	0.067334	-0.004780	0.055932
	-0.042354	-0.021007	0.059872	0.023493	0.016956	0.000167
	0.011824	0.091184	-0.025586	-0.018008	0.035599	-0.020939
	-0.059117	0.006515	0.013055	0.019445	-0.020485	-0.043403
	0.040918					
x459	-0.023745	-0.014991	0.007474	-0.053222	-0.096095	0.008948
	-0.004588	-0.061569	0.037785	0.062073	0.015674	-0.018640
	0.006501	-0.006613	-0.006595	-0.016499	-0.008095	0.008544
	-0.011640	0.024986	-0.013722	0.012898	-0.015878	-0.011536
	-0.042711	0.025519	0.024293	0.027613	-0.003046	-0.004975
	0.012892					

y459	0.020873	0.044948	0.021900	-0.004411	0.014581	-0.026450
	-0.009212	-0.005790	-0.002771	-0.021435	0.033460	-0.007910
	0.040788	-0.014937	-0.028455	-0.061613	-0.012958	0.010087
	-0.006734	-0.004882	-0.021296	0.000783	0.008050	0.014739
	-0.036506	0.006615	-0.009448	0.003599	-0.016916	0.013532
	-0.046080					
z459	-0.002654	-0.033733	-0.076652	0.061400	0.078627	-0.023616
	0.013893	-0.000589	0.068111	0.062567	-0.006576	0.047886
	-0.015022	-0.012578	0.045907	0.029477	0.022379	-0.004885
	0.000781	0.075996	-0.020341	0.010784	0.026303	-0.053473
	-0.048167	0.010491	0.005239	0.027165	-0.014810	-0.003870
	0.034600					
x460	-0.014538	-0.016480	0.004674	-0.026037	-0.089392	0.000416
	0.005533	-0.042300	0.034173	0.047583	0.015544	-0.012069
	0.006035	-0.007056	-0.000641	-0.004574	-0.012422	0.001364
	-0.005921	0.040475	0.000253	0.020004	-0.013612	-0.033011
	-0.018820	0.019553	0.007134	0.028333	-0.005664	0.002386
	0.011260					
y460	0.021613	0.028578	0.030031	-0.014673	0.023611	-0.020192
	-0.008894	-0.009902	-0.000207	-0.011317	0.030495	-0.010093
	0.040826	-0.030171	-0.009043	-0.050773	-0.011641	0.008557
	-0.015910	-0.001371	-0.017234	-0.016127	-0.003426	0.009704
	-0.034937	0.004964	0.000662	0.004804	-0.014951	-0.006742
	-0.060724					
z460	-0.007273	-0.040570	-0.073221	0.038949	0.061932	-0.013181
	0.027447	-0.014206	0.063868	0.052302	0.001706	0.030958
	-0.000310	-0.005672	0.031185	0.026106	0.023508	-0.013702
	-0.014631	0.054041	-0.023447	0.019827	0.012138	-0.069455
	-0.029874	0.002242	0.014569	0.037290	-0.011975	0.020323
	0.035516					
x461	-0.003086	-0.015274	0.004312	0.005820	-0.083270	-0.007463
	0.012008	-0.018999	0.037442	0.035273	0.011133	0.005188
	-0.005225	-0.005524	0.008428	0.005552	-0.025591	-0.000335
	-0.001768	0.071473	0.011158	0.027824	-0.011070	-0.042727
	-0.002352	0.027824	-0.020134	0.019256	-0.007146	0.006318
	-0.003718					
y461	0.021010	0.010613	0.039122	-0.026290	0.030560	-0.012234
	-0.010065	-0.016762	-0.003045	-0.004340	0.028147	-0.008235
	0.042939	-0.047156	0.018543	-0.041208	-0.004525	0.010619
	-0.030263	0.005505	-0.009357	-0.028018	-0.017379	0.008150
	-0.030670	-0.000440	0.006442	0.005002	-0.010470	-0.030287
	-0.074245					

z461	-0.012352	-0.042080	-0.063576	0.014480	0.042061	-0.003431
	0.034416	-0.026574	0.064266	0.044526	0.004247	0.021089
	0.004156	0.000104	0.018082	0.021235	0.007131	-0.022665
	-0.031571	0.039566	-0.031092	0.026327	-0.003845	-0.068783
	-0.028959	0.011729	0.030296	0.032144	-0.007190	0.026914
	0.018007					
x462	0.011365	-0.013397	0.004719	0.040715	-0.074972	-0.012906
	0.014000	0.006177	0.047320	0.031856	0.004844	0.026813
	-0.018795	0.005690	0.013636	0.001077	-0.036531	0.003553
	0.018034	0.093274	0.024341	0.027279	-0.002134	-0.039225
	0.013401	0.031234	-0.064283	0.008152	-0.009423	0.015250
	-0.004593					
y462	0.019728	-0.006080	0.049797	-0.035944	0.037520	-0.004429
	-0.011772	-0.022919	-0.009170	0.001846	0.026155	-0.003804
	0.047520	-0.063415	0.043949	-0.031275	0.009784	0.013714
	-0.044532	0.011552	-0.000994	-0.037832	-0.026262	0.003866
	-0.022956	-0.009904	0.009442	0.000438	-0.018075	-0.063499
	-0.076361					
z462	-0.016484	-0.039285	-0.054010	-0.001587	0.022390	0.004589
	0.034789	-0.039633	0.059509	0.038452	0.005061	0.012649
	-0.000608	0.013610	0.001042	0.005493	-0.009742	-0.029623
	-0.025862	0.007296	-0.033450	0.032981	-0.016920	-0.049778
	-0.032544	0.004132	0.034173	0.025426	-0.011220	0.034232
	0.019894					
x463	0.017473	-0.015443	0.013505	0.044979	-0.064802	-0.017631
	0.012667	0.011319	0.031211	0.029356	0.011524	0.020158
	-0.017048	0.006192	0.024680	0.002817	-0.050871	0.012047
	-0.008896	0.073661	0.017892	0.027665	-0.002713	-0.014722
	0.003508	0.024867	-0.057859	-0.007730	-0.008730	0.019646
	0.008056					
y463	0.016259	-0.017138	0.044935	-0.039019	0.037402	-0.002811
	-0.008306	-0.017496	0.003639	0.007045	0.022360	-0.003400
	0.038508	-0.050709	0.040736	-0.030860	0.003131	0.019401
	-0.052940	0.019872	0.000920	-0.027518	-0.012360	0.004039
	-0.002420	0.008812	0.015105	0.004650	-0.000351	-0.052234
	-0.048475					
z463	-0.011836	-0.032063	-0.053734	0.007572	0.003221	0.012503
	0.039394	-0.040188	0.061123	0.033430	-0.009257	0.019894
	-0.007355	0.025986	-0.012671	-0.016571	-0.022165	-0.036223
	-0.013754	0.004832	-0.037731	0.040794	-0.023474	-0.055178
	-0.040276	-0.016879	0.014005	-0.001882	-0.008264	0.035301
	0.043617					

x464	0.023262	-0.021981	0.023652	0.042312	-0.052610	-0.018558
	0.010601	0.013977	0.021101	0.022857	0.017480	0.012016
	-0.013891	0.006135	0.029629	0.000311	-0.050990	0.023254
	-0.028861	0.058977	0.012928	0.024139	-0.008471	0.003771
	0.001879	0.016733	-0.049926	-0.010005	-0.007476	0.023231
	0.001593					
y464	0.014105	-0.023079	0.038195	-0.038686	0.033766	-0.001226
	-0.004161	-0.015495	0.013014	0.006613	0.015386	-0.000314
	0.030718	-0.040813	0.032237	-0.031768	0.000972	0.017135
	-0.054007	0.027509	0.001562	-0.020586	-0.005783	0.005823
	0.015485	0.013693	0.020508	0.004063	0.008048	-0.046714
	-0.035809					
z464	-0.006126	-0.028726	-0.054318	0.015382	-0.010681	0.017760
	0.038736	-0.035589	0.056608	0.020462	-0.022332	0.023687
	-0.013463	0.030416	-0.015700	-0.031842	-0.025440	-0.040522
	-0.008328	0.002893	-0.029970	0.036787	-0.027193	-0.053506
	-0.025590	-0.031981	0.002662	-0.006020	-0.006726	0.027441
	0.028637					
x465	0.028169	-0.028082	0.031244	0.039874	-0.042213	-0.014542
	0.007167	0.012340	0.015178	0.017387	0.026552	0.006025
	-0.012522	0.008141	0.029028	0.007427	-0.053329	0.034684
	-0.036337	0.037893	-0.000500	0.020934	-0.008015	0.013565
	-0.009385	0.008487	-0.056428	-0.008404	-0.005500	0.027619
	0.015363					
y465	0.012185	-0.025706	0.031494	-0.036618	0.027172	0.001151
	-0.000438	-0.015851	0.019561	0.005506	0.008092	0.004532
	0.023400	-0.032560	0.023631	-0.030020	-0.000901	0.011598
	-0.045777	0.029238	-0.001694	-0.015780	-0.000007	0.003137
	0.027050	0.013613	0.022010	0.002422	0.011981	-0.041222
	-0.022209					
z465	-0.003831	-0.025888	-0.055129	0.023267	-0.021640	0.021611
	0.031168	-0.030583	0.045511	0.006728	-0.024660	0.019688
	-0.018547	0.028982	-0.012028	-0.030364	-0.031062	-0.041610
	-0.009368	-0.011838	-0.029548	0.029340	-0.020421	-0.045278
	-0.023426	-0.042650	-0.012078	-0.013924	-0.010602	0.005701
	0.034218					
x466	0.035980	-0.033574	0.037105	0.035341	-0.034578	-0.007587
	0.007184	0.010818	0.014643	0.011094	0.037878	0.000974
	-0.014300	0.014689	0.018209	0.013372	-0.045674	0.035618
	-0.040078	0.028946	-0.000586	0.008780	-0.009631	0.013244
	-0.013025	0.012472	-0.057412	0.002571	-0.012287	0.028681
	0.021709					

y466	0.011171	-0.026790	0.025452	-0.035418	0.018662	0.004280
	0.003599	-0.016444	0.024009	0.001930	0.001909	0.009192
	0.013003	-0.024985	0.012927	-0.028422	0.000865	0.003697
	-0.034123	0.032369	0.000478	-0.014333	0.004009	-0.002968
	0.035101	0.020336	0.023014	0.004198	0.012902	-0.034967
	-0.013432					
z466	0.002359	-0.027048	-0.056650	0.028103	-0.027060	0.030573
	0.023605	-0.021797	0.036450	-0.003353	-0.024569	0.018327
	-0.023467	0.028995	-0.010494	-0.028369	-0.038477	-0.046334
	-0.011844	-0.024116	-0.011274	0.011390	-0.000570	-0.045694
	-0.005534	-0.035416	0.000912	0.008257	-0.000064	-0.016949
	0.025036					
x467	0.040406	-0.037685	0.039858	0.032481	-0.026998	-0.001190
	0.009344	0.009631	0.013564	0.005153	0.038385	-0.001487
	-0.011113	0.022345	0.006183	0.015151	-0.039010	0.032436
	-0.024777	0.021871	-0.006759	0.004039	-0.014083	0.005034
	-0.027512	0.012161	-0.056874	0.004840	-0.011183	0.045409
	0.017373					
y467	0.008867	-0.026317	0.018636	-0.033177	0.008777	0.005601
	0.008606	-0.015985	0.025182	-0.001430	-0.007589	0.013415
	0.005659	-0.018314	0.005646	-0.026157	0.000760	-0.007685
	-0.020759	0.033697	-0.001472	-0.014400	0.006513	-0.008705
	0.039405	0.024071	0.021743	0.001893	0.012622	-0.024978
	-0.009687					
z467	0.002509	-0.026960	-0.058061	0.032854	-0.028530	0.032679
	0.020486	-0.013393	0.029636	-0.010963	-0.027250	0.012878
	-0.023196	0.019742	-0.008937	-0.028267	-0.041773	-0.049990
	-0.007814	-0.024842	-0.007972	0.006165	0.006129	-0.042414
	-0.002569	-0.034236	0.005840	0.007931	0.006191	-0.015416
	0.011611					
x468	0.047685	-0.039952	0.042066	0.029636	-0.023685	0.005215
	0.013356	0.003852	0.015134	0.000703	0.041640	-0.004905
	-0.011644	0.032571	-0.007203	0.015082	-0.020385	0.032802
	-0.021997	0.013491	-0.009619	0.000643	-0.020665	-0.000526
	-0.026088	0.002201	-0.053560	0.005527	-0.022864	0.048233
	0.019640					
y468	0.006130	-0.024896	0.012345	-0.030992	-0.002733	0.006088
	0.012912	-0.015428	0.023981	-0.003901	-0.018122	0.017348
	-0.001090	-0.013153	0.002672	-0.023889	0.001000	-0.016737
	-0.011898	0.031072	-0.002049	-0.014238	0.008483	-0.010834
	0.045734	0.025428	0.019203	-0.002388	0.009521	-0.018747
	-0.003363					

z468	0.003528	-0.026232	-0.056454	0.037428	-0.029831	0.033255
	0.016395	-0.008309	0.024653	-0.013085	-0.023906	0.008115
	-0.022437	0.013507	-0.008400	-0.029020	-0.031171	-0.041673
	-0.022569	-0.029840	-0.000863	0.005069	0.013353	-0.030030
	0.008291	-0.037684	0.006755	-0.001945	-0.000745	-0.028030
	0.012712					
x469	0.050410	-0.043158	0.039929	0.027134	-0.018156	0.009760
	0.018187	0.002619	0.015501	-0.003031	0.041240	-0.006014
	-0.005537	0.042493	-0.011547	0.011871	-0.014031	0.026363
	-0.013045	0.004028	-0.010102	-0.002651	-0.027632	-0.008065
	-0.033040	0.002966	-0.041131	-0.000109	-0.021740	0.048071
	0.008678					
y469	0.002107	-0.023294	0.006052	-0.029116	-0.014309	0.005150
	0.017033	-0.014050	0.021613	-0.006669	-0.029065	0.022646
	-0.008168	-0.011907	0.003748	-0.020224	-0.002887	-0.023636
	-0.006245	0.028024	-0.002888	-0.014045	0.008289	-0.009684
	0.050086	0.028442	0.018812	-0.005795	0.010708	-0.013290
	0.000964					
z469	0.000007	-0.027538	-0.055906	0.041132	-0.024591	0.031965
	0.012219	-0.000755	0.022937	-0.011720	-0.017284	0.008632
	-0.012845	0.003716	-0.006392	-0.032053	-0.031504	-0.034611
	-0.032592	-0.034965	-0.002242	0.007307	0.015518	-0.021608
	0.001224	-0.025012	0.018539	-0.020813	0.010102	-0.037297
	-0.000805					
x470	0.054787	-0.044889	0.033703	0.026611	-0.015166	0.013827
	0.021187	0.001876	0.008890	-0.002724	0.037763	-0.009894
	-0.003008	0.046125	-0.011919	0.011468	-0.014814	0.017246
	-0.011392	-0.006443	-0.016920	-0.016755	-0.035774	-0.006445
	-0.015350	-0.010801	-0.017340	-0.001560	-0.016098	0.036347
	-0.005757					
y470	-0.002236	-0.021299	0.000860	-0.027061	-0.026493	0.003198
	0.020435	-0.012977	0.018344	-0.008202	-0.038807	0.028903
	-0.016268	-0.014855	0.004637	-0.015263	-0.005527	-0.029450
	-0.003766	0.024173	-0.007350	-0.012583	0.006109	-0.004337
	0.053847	0.028117	0.020078	-0.010462	0.014944	-0.007882
	0.008088					
z470	-0.000631	-0.027236	-0.054480	0.047213	-0.020954	0.030787
	0.006368	0.005042	0.015593	-0.003890	-0.012511	0.004999
	-0.012147	-0.015752	-0.010977	-0.032710	-0.033199	-0.029236
	-0.035927	-0.041676	-0.013084	0.008222	0.013621	-0.004945
	0.009671	-0.029029	0.032258	-0.028318	0.019481	-0.051877
	-0.008311					

x471	0.058372	-0.045638	0.028016	0.025086	-0.009500	0.012677
	0.022053	0.003383	0.003295	-0.004135	0.027178	-0.022343
	-0.000029	0.053299	0.002617	0.020886	-0.014379	0.010173
	-0.003705	-0.013001	-0.005444	-0.029367	-0.047373	-0.001807
	0.003930	-0.009925	0.008773	0.009068	-0.017835	0.023269
	-0.007621					
y471	-0.007197	-0.019229	-0.001804	-0.024764	-0.039867	0.000630
	0.022918	-0.012220	0.015321	-0.007713	-0.041772	0.035179
	-0.022160	-0.024198	0.003029	-0.009855	-0.002289	-0.033350
	-0.002078	0.015882	-0.013523	-0.004877	0.002265	0.002679
	0.056016	0.033355	0.016589	-0.013839	0.020347	-0.002304
	0.031062					
z471	-0.001313	-0.024208	-0.048560	0.052359	-0.013761	0.026262
	-0.001906	0.007926	0.009742	0.002249	-0.013742	-0.006462
	-0.009332	-0.028172	-0.016117	-0.029488	-0.022200	-0.018923
	-0.016339	-0.048002	-0.007879	0.014719	0.006678	0.005055
	-0.002651	-0.022800	0.035146	-0.029235	0.009855	-0.051894
	-0.004704					
x472	0.054984	-0.047221	0.025294	0.025464	-0.006047	0.013489
	0.016070	0.002339	-0.001577	-0.001388	0.024494	-0.020013
	0.005370	0.041058	-0.001035	0.031873	-0.007798	-0.002255
	-0.000449	-0.014338	0.007248	-0.022445	-0.041493	0.006187
	0.015164	-0.004288	0.026676	0.006835	-0.007262	0.032253
	-0.022676					
y472	-0.015224	-0.010743	-0.007870	-0.022654	-0.041707	-0.004687
	0.015910	-0.006039	0.009028	-0.002901	-0.041095	0.028484
	-0.032991	-0.041032	0.000542	-0.019604	-0.007856	-0.013429
	0.002407	0.017690	-0.012642	-0.005549	-0.002816	-0.003872
	0.053401	0.022244	0.024924	-0.008266	0.024412	-0.017956
	0.018055					
z472	-0.001555	-0.021172	-0.044368	0.052254	-0.007531	0.022879
	-0.004584	0.010997	0.009382	0.003340	-0.003657	-0.011421
	0.002013	-0.029151	-0.011857	-0.018323	-0.012150	-0.034539
	-0.005786	-0.048121	-0.004459	0.023052	0.005658	-0.002100
	0.004133	-0.005032	0.058571	-0.014722	0.032990	-0.041846
	-0.022487					
x473	0.049041	-0.045585	0.023859	0.025357	-0.004794	0.014574
	0.008265	0.002425	-0.002782	0.002642	0.016018	-0.014977
	0.002340	0.033286	-0.007370	0.038845	-0.006924	-0.000668
	0.002484	-0.019150	0.009542	-0.010691	-0.039008	0.011352
	0.029030	-0.001342	0.031926	0.007978	-0.017424	0.049560
	-0.007672					

y473	-0.020909	-0.004882	-0.012751	-0.020090	-0.043837	-0.010833
	0.008099	-0.002349	0.001949	0.001732	-0.038697	0.023574
	-0.034030	-0.060792	-0.005199	-0.028481	-0.008542	-0.001939
	0.004564	0.019562	-0.009301	-0.009935	-0.009094	-0.009610
	0.045413	0.013500	0.027608	-0.004591	0.030183	-0.034780
	0.005340					
z473	-0.003630	-0.016955	-0.039633	0.050219	-0.003344	0.019706
	-0.008394	0.012056	0.011401	0.002577	-0.007757	-0.010815
	0.002238	-0.023742	-0.011195	-0.009904	-0.007289	-0.033713
	0.001351	-0.045420	-0.006324	0.031967	0.000443	-0.003948
	0.021297	-0.001314	0.067991	-0.007340	0.017004	-0.021386
	-0.006849					
x474	0.041726	-0.042980	0.022274	0.024766	-0.001747	0.015022
	0.001034	0.007207	-0.004935	0.009475	0.006806	-0.010717
	0.004005	0.019326	-0.014058	0.041247	-0.008528	0.000480
	0.003857	-0.021393	0.014125	-0.002386	-0.036300	0.015537
	0.024102	0.005553	0.023697	0.006535	-0.007054	0.055149
	-0.000561					
y474	-0.025993	-0.001581	-0.016999	-0.018308	-0.045568	-0.014805
	-0.002463	0.001907	-0.005495	0.007103	-0.040645	0.021887
	-0.029603	-0.074319	-0.008237	-0.033407	-0.015708	0.004231
	0.009822	0.013673	-0.004558	-0.011230	-0.010363	-0.017653
	0.031616	0.003696	0.025015	-0.005488	0.028443	-0.037048
	0.006637					
z474	-0.006887	-0.013242	-0.034651	0.045466	0.003613	0.018302
	-0.013177	0.017686	0.012592	0.006351	-0.015618	-0.010668
	0.011125	-0.025904	-0.011450	-0.004001	-0.009177	-0.037372
	0.003175	-0.044519	-0.003386	0.035435	-0.000848	-0.003755
	0.013029	0.002009	0.055392	-0.004117	0.027053	-0.017649
	0.004434					
x475	0.035212	-0.041174	0.018660	0.024692	-0.000247	0.013167
	-0.006941	0.011283	-0.010603	0.016123	-0.001192	-0.007281
	0.001744	0.001398	-0.024209	0.035224	-0.007655	0.005828
	0.012377	-0.020494	0.019930	0.005875	-0.030840	0.010476
	0.019719	0.012229	0.023574	0.002894	-0.010697	0.048995
	0.005945					
y475	-0.028706	0.000074	-0.019129	-0.017334	-0.047650	-0.018138
	-0.012081	0.002183	-0.011396	0.008624	-0.040334	0.019854
	-0.023912	-0.084616	-0.011790	-0.035127	-0.019732	0.005981
	0.006349	0.008980	-0.001362	-0.014400	-0.014025	-0.019258
	0.021228	-0.005527	0.023056	-0.004729	0.023617	-0.031265
	0.005334					

z475	-0.008697	-0.011408	-0.031167	0.041416	0.008735	0.015418
	-0.019541	0.019614	0.010982	0.009439	-0.022922	-0.009016
	0.013313	-0.029754	-0.018094	-0.008366	-0.012082	-0.040469
	0.008214	-0.040850	-0.001022	0.036938	0.006332	-0.013065
	0.003540	0.010283	0.050646	-0.001872	0.017623	-0.025320
	0.018115					
x476	0.028468	-0.039256	0.017332	0.024570	-0.002837	0.011542
	-0.014423	0.015414	-0.019553	0.027258	-0.002511	-0.005105
	0.000626	-0.016187	-0.035738	0.028507	-0.002915	0.011102
	0.017010	-0.022599	0.021637	0.008537	-0.031425	0.013136
	0.022504	0.017385	0.023958	-0.005644	-0.014676	0.032540
	-0.003556					
y476	-0.029288	0.000414	-0.019396	-0.017179	-0.049311	-0.021374
	-0.019723	-0.000262	-0.015700	0.002937	-0.037402	0.015901
	-0.019486	-0.090440	-0.013212	-0.037030	-0.024333	0.004218
	-0.000159	0.006211	0.001432	-0.017074	-0.017276	-0.020791
	0.015229	-0.018077	0.023294	-0.000841	0.014395	-0.018182
	0.009563					
z476	-0.011122	-0.007911	-0.023776	0.037227	0.009400	0.014497
	-0.024177	0.021124	0.010294	0.017208	-0.021950	-0.011733
	0.017934	-0.030175	-0.021518	-0.015236	-0.008304	-0.042358
	0.007470	-0.041848	-0.001166	0.036051	0.001703	-0.005777
	0.010309	0.013991	0.044326	-0.005808	0.014409	-0.043153
	0.010277					
x477	0.021115	-0.037338	0.013524	0.021723	-0.005666	0.011739
	-0.019599	0.019374	-0.024155	0.035568	-0.006966	0.002912
	0.005890	-0.027295	-0.045915	0.026480	-0.001831	0.008842
	0.024283	-0.019538	0.019336	0.017435	-0.022296	0.010401
	0.015759	0.025005	0.025548	-0.001524	-0.016672	0.035658
	-0.005527					
y477	-0.030269	0.000613	-0.018428	-0.014752	-0.048258	-0.022451
	-0.026825	-0.002615	-0.017898	-0.003604	-0.032437	0.011327
	-0.011503	-0.094369	-0.011101	-0.040661	-0.030761	0.002270
	-0.005257	0.003473	0.001595	-0.021131	-0.019943	-0.018700
	0.011074	-0.025937	0.009927	-0.002223	0.008441	-0.010220
	0.013563					
z477	-0.014780	-0.003354	-0.018608	0.029632	0.010947	0.017311
	-0.022469	0.023740	0.018399	0.016042	-0.023132	-0.008772
	0.027951	-0.018961	-0.023083	-0.016648	-0.014319	-0.053754
	0.009270	-0.032292	-0.011265	0.041716	0.005725	-0.008062
	0.009482	0.024313	0.037635	0.008907	0.017698	-0.030586
	0.014345					

x478	0.015226	-0.035515	0.009487	0.021618	-0.007844	0.010933
	-0.026071	0.025181	-0.031418	0.041102	-0.005891	0.005847
	0.008982	-0.037991	-0.050645	0.026194	0.004431	0.009008
	0.029194	-0.009330	0.022530	0.017247	-0.010989	0.009700
	0.007983	0.022265	0.024803	-0.007223	-0.023515	0.026058
	-0.008430					
y478	-0.030844	0.002026	-0.014528	-0.013555	-0.047693	-0.022813
	-0.030241	-0.005746	-0.013517	-0.012955	-0.022885	0.006471
	-0.005860	-0.089381	-0.006028	-0.043665	-0.037108	0.000487
	-0.014211	0.000429	-0.004498	-0.021550	-0.023310	-0.011997
	0.011470	-0.030743	0.003869	0.005181	0.004014	0.004042
	0.017878					
z478	-0.017085	0.002672	-0.014679	0.025918	0.013300	0.019782
	-0.019702	0.028896	0.025600	0.012084	-0.020429	-0.013684
	0.034085	-0.004707	-0.018944	-0.013667	-0.013223	-0.061326
	0.011437	-0.016379	-0.012228	0.036713	0.008110	-0.006683
	0.003706	0.025032	0.027839	0.004148	0.018641	-0.038206
	0.015440					
x479	0.010662	-0.034024	0.006999	0.021193	-0.011653	0.011003
	-0.029827	0.026924	-0.035479	0.042564	0.002104	0.006286
	0.006477	-0.049282	-0.051016	0.023116	0.010065	0.011014
	0.028393	-0.003692	0.023040	0.012476	0.001665	0.010861
	0.004510	0.014373	0.029782	-0.003066	-0.041775	0.011357
	-0.010604					
y479	-0.031275	0.006529	-0.009220	-0.011038	-0.044422	-0.022496
	-0.030855	-0.007812	-0.005318	-0.024450	-0.011929	-0.000405
	0.003367	-0.079500	0.000762	-0.047869	-0.041494	-0.001669
	-0.023589	0.003939	-0.014273	-0.021380	-0.024863	0.003675
	0.010599	-0.021108	-0.006219	0.006810	0.012461	0.018181
	0.012806					
z479	-0.016969	0.007922	-0.009281	0.019856	0.012176	0.023818
	-0.009460	0.028381	0.038682	0.001555	-0.012466	-0.023363
	0.025202	0.006251	-0.012632	-0.015615	-0.012492	-0.064351
	0.005169	-0.009914	-0.017519	0.029799	0.006673	-0.011629
	0.009905	0.021299	0.039794	0.014002	0.003514	-0.053560
	0.017568					
x480	0.003308	-0.030456	0.004139	0.022144	-0.013115	0.010868
	-0.036686	0.024868	-0.038423	0.041586	0.007070	0.012715
	0.011524	-0.054985	-0.045108	0.016737	0.005890	0.017779
	0.028979	-0.003243	0.023825	0.013144	0.012390	0.016036
	-0.007397	0.007346	0.015309	0.003071	-0.046722	0.012425
	-0.013407					

y480	-0.032282	0.014036	-0.002630	-0.008670	-0.040558	-0.020612
	-0.027478	-0.006937	0.008867	-0.038541	0.001238	-0.009860
	0.005381	-0.062972	0.010820	-0.048810	-0.045968	-0.002196
	-0.031807	0.008374	-0.027086	-0.017314	-0.024238	0.011754
	0.016687	-0.004696	-0.004645	0.015290	0.014429	0.026120
	0.017801					
z480	-0.020082	0.014758	-0.007792	0.016451	0.012847	0.028056
	-0.001305	0.024377	0.048287	-0.006195	-0.016848	-0.025785
	0.029578	0.021090	-0.008151	-0.022099	-0.020687	-0.063535
	0.000374	-0.001239	-0.025972	0.030088	0.004984	-0.018616
	-0.002652	0.026643	0.035182	0.016112	0.024823	-0.048272
	0.009807					
x481	-0.003962	-0.025337	0.006545	0.021903	-0.014659	0.015597
	-0.038924	0.025965	-0.032094	0.026848	-0.000241	0.004910
	0.018313	-0.040764	-0.034711	-0.000442	-0.005206	0.016530
	0.022045	-0.009127	0.021460	0.012819	0.014655	0.007035
	-0.021540	0.013541	0.024376	0.015585	-0.039221	0.009330
	0.003128					
y481	-0.029097	0.014049	0.001180	-0.009111	-0.036147	-0.022670
	-0.025825	-0.000945	0.014328	-0.037010	0.005690	-0.011422
	0.004397	-0.052962	0.012792	-0.041582	-0.044261	-0.004116
	-0.034328	0.002211	-0.020539	-0.014099	-0.024086	0.005985
	0.014743	-0.001000	0.002201	0.010773	0.018164	0.027232
	0.020781					
z481	-0.018718	0.013817	-0.010313	0.015372	0.015171	0.028633
	0.002557	0.024283	0.047933	-0.004239	-0.022061	-0.019722
	0.027356	0.028503	-0.002441	-0.030275	-0.025628	-0.057414
	-0.003091	-0.001497	-0.017019	0.032210	-0.001991	-0.000518
	-0.002953	0.019879	0.024844	0.014583	0.016782	-0.036102
	0.010761					
x482	-0.009602	-0.020630	0.008573	0.022220	-0.016205	0.016885
	-0.039763	0.027880	-0.029275	0.011557	-0.003717	-0.004600
	0.022208	-0.028453	-0.028726	-0.015341	-0.004956	0.021293
	0.008881	-0.003732	0.018901	0.015094	0.003398	0.001096
	-0.028757	0.019312	0.028575	0.014141	-0.029850	0.004247
	-0.004887					
y482	-0.025594	0.013526	0.004060	-0.009561	-0.032125	-0.023958
	-0.025257	0.003845	0.018240	-0.032728	0.009146	-0.010625
	0.003075	-0.043926	0.014847	-0.033386	-0.043559	-0.007816
	-0.035725	-0.001113	-0.018674	-0.010975	-0.019934	0.002355
	0.010530	-0.000332	0.009529	0.004753	0.011813	0.022743
	0.033407					

z482	-0.016044	0.012553	-0.014450	0.014108	0.015430	0.025284
	0.009043	0.024043	0.043614	-0.006049	-0.022525	-0.021732
	0.022479	0.028643	-0.009287	-0.036112	-0.011742	-0.039327
	-0.010216	0.009267	-0.012287	0.028173	-0.024353	0.006528
	0.006013	0.020615	0.016552	0.001522	0.021810	-0.032494
	-0.021169					
x483	-0.012923	-0.016909	0.011511	0.024560	-0.018070	0.018048
	-0.039441	0.028189	-0.028902	0.002210	-0.009045	-0.008886
	0.025723	-0.019532	-0.024114	-0.022079	-0.004909	0.020682
	0.000390	-0.002993	0.014395	0.022258	0.007476	-0.000434
	-0.036694	0.022689	0.021135	0.014352	-0.031484	-0.001371
	-0.002507					
y483	-0.021665	0.012731	0.005503	-0.009847	-0.026882	-0.024695
	-0.024492	0.008783	0.020134	-0.028746	0.013327	-0.011213
	0.002283	-0.034974	0.017448	-0.023812	-0.039831	-0.010154
	-0.034735	0.002290	-0.015317	-0.011537	-0.020919	0.004222
	0.006046	0.002574	0.014484	-0.000071	0.016510	0.017161
	0.029591					
z483	-0.014849	0.010352	-0.018065	0.015744	0.013305	0.020834
	0.018984	0.023335	0.039476	-0.002190	-0.031579	-0.018853
	0.016976	0.022188	-0.021328	-0.037368	-0.004210	-0.027250
	-0.011907	0.008587	-0.014641	0.030309	-0.021809	0.007969
	0.009780	0.018561	0.002269	-0.006210	0.004941	-0.028183
	-0.023520					
x484	-0.013665	-0.012582	0.015118	0.025844	-0.018502	0.017626
	-0.040321	0.027379	-0.028223	-0.002669	-0.011045	-0.013426
	0.028177	-0.005692	-0.015736	-0.020072	-0.001379	0.013424
	-0.001809	-0.009868	0.004616	0.029846	0.014688	0.001563
	-0.039650	0.023149	0.017556	0.020118	-0.031130	-0.008067
	0.010603					
y484	-0.017892	0.010984	0.005524	-0.009562	-0.020684	-0.025921
	-0.022794	0.011975	0.020702	-0.024879	0.013890	-0.009083
	0.001184	-0.028318	0.016801	-0.013260	-0.035500	-0.012561
	-0.033828	0.009822	-0.009080	-0.013192	-0.019689	0.006645
	-0.000691	0.007954	0.016546	-0.004826	0.019082	0.009235
	0.022742					
z484	-0.013322	0.011268	-0.019852	0.016879	0.009591	0.016432
	0.027166	0.022559	0.037876	0.002604	-0.032289	-0.021075
	0.013723	0.024581	-0.023826	-0.032920	0.004633	-0.021902
	-0.008073	-0.010858	-0.030663	0.032185	-0.020988	0.007391
	0.019710	0.014484	-0.006919	0.001886	-0.001687	-0.018767
	-0.000698					

x485	-0.014768	-0.009261	0.017803	0.025274	-0.014662	0.016272
	-0.041433	0.027185	-0.029015	-0.006362	-0.011073	-0.017691
	0.033850	0.008161	-0.007731	-0.019626	0.000908	0.004470
	-0.000120	-0.006936	0.003494	0.031042	0.015760	0.003669
	-0.047183	0.015848	0.012246	0.017872	-0.033107	-0.025815
	0.009618					
y485	-0.014707	0.009021	0.004261	-0.008714	-0.015963	-0.028241
	-0.019730	0.013840	0.020224	-0.019982	0.012376	-0.006626
	-0.000864	-0.022935	0.012732	-0.003660	-0.027559	-0.013922
	-0.034030	0.015309	-0.006415	-0.012463	-0.018920	0.010016
	-0.004283	0.015182	0.019696	-0.011579	0.019266	0.003907
	0.016551					
z485	-0.014221	0.011355	-0.022805	0.015605	0.009575	0.013708
	0.032586	0.023876	0.035707	0.003073	-0.029701	-0.022736
	0.016946	0.027508	-0.022035	-0.035520	0.007321	-0.018338
	0.004353	-0.018804	-0.030763	0.023213	-0.026717	0.004232
	0.018701	0.001088	-0.012296	0.008689	-0.003984	-0.024726
	0.004981					
x486	-0.015852	-0.008256	0.019017	0.023484	-0.012655	0.012026
	-0.040829	0.032638	-0.029920	-0.006925	-0.013967	-0.019156
	0.030676	0.018728	-0.005119	-0.022581	0.002585	0.001219
	-0.001375	0.003208	0.009485	0.036943	0.014973	0.011086
	-0.042753	0.006461	0.014290	0.023640	-0.039106	-0.036178
	-0.010842					
y486	-0.011443	0.008125	0.002186	-0.006954	-0.012573	-0.029108
	-0.015991	0.012781	0.017598	-0.016341	0.013709	-0.003893
	-0.000811	-0.018648	0.009468	0.006706	-0.023352	-0.015307
	-0.031866	0.011886	-0.008559	-0.015324	-0.015198	0.011028
	-0.009519	0.022112	0.008332	-0.016112	0.023830	0.000677
	0.021138					
z486	-0.017191	0.010288	-0.026565	0.012670	0.006462	0.007570
	0.040110	0.030636	0.036553	0.007978	-0.032083	-0.021915
	0.014895	0.024468	-0.028611	-0.044039	0.013222	-0.007984
	0.007628	-0.018991	-0.021041	0.016558	-0.035096	0.005446
	0.024459	-0.014775	-0.001978	0.026927	-0.016373	-0.022431
	-0.014717					
x487	-0.015005	-0.006979	0.020622	0.020810	-0.011317	0.007657
	-0.037498	0.033442	-0.029896	-0.009177	-0.016848	-0.017216
	0.024185	0.027856	-0.002339	-0.019933	-0.000687	-0.004181
	-0.001963	0.005773	0.010493	0.038439	0.025978	0.015558
	-0.040818	0.007009	0.011070	0.025848	-0.032586	-0.033844
	-0.011844					

y487	-0.010513	0.006874	-0.002165	-0.003611	-0.009915	-0.030977
	-0.010257	0.012575	0.015561	-0.011620	0.014210	-0.003502
	-0.002133	-0.016842	0.006385	0.010582	-0.015312	-0.014194
	-0.028024	0.013290	-0.005758	-0.017476	-0.018222	0.015212
	-0.011846	0.023611	0.005880	-0.021100	0.024802	-0.007528
	0.016637					
z487	-0.017675	0.009947	-0.027275	0.006855	0.001706	0.006052
	0.044500	0.033338	0.035134	0.005637	-0.034951	-0.015189
	0.014318	0.017787	-0.036873	-0.045908	0.009859	-0.001144
	0.008552	-0.029497	-0.021822	0.003059	-0.026454	-0.002896
	0.017241	-0.013394	0.002318	0.045650	-0.008798	-0.009484
	-0.011249					
x488	-0.014632	-0.005182	0.019412	0.017709	-0.008419	0.002559
	-0.035568	0.033035	-0.027428	-0.012247	-0.016425	-0.017429
	0.014003	0.036967	-0.000167	-0.017654	-0.000131	-0.007518
	-0.004673	0.008046	0.015656	0.036376	0.034830	0.013137
	-0.034706	0.014318	0.008673	0.007679	-0.025724	-0.037541
	-0.015275					
y488	-0.009768	0.005336	-0.007148	0.000358	-0.009819	-0.031039
	-0.002297	0.012221	0.015216	-0.006972	0.015543	-0.000018
	-0.002885	-0.017624	0.001703	0.009553	-0.011153	-0.011391
	-0.021293	0.011694	-0.005946	-0.021772	-0.021111	0.020379
	-0.014622	0.022938	0.001341	-0.011952	0.027258	-0.006778
	0.015330					
z488	-0.018943	0.012536	-0.028752	0.001271	-0.001593	0.004277
	0.041777	0.033571	0.030907	0.002359	-0.037712	-0.014781
	0.013888	0.012086	-0.040515	-0.039609	0.016891	0.003819
	0.003112	-0.028042	-0.013635	-0.004751	-0.021882	-0.008115
	0.012613	-0.007355	0.007204	0.033039	-0.005522	-0.014178
	-0.012207					
x489	-0.013817	-0.006848	0.016651	0.015972	-0.009136	-0.003756
	-0.028466	0.030306	-0.027364	-0.010222	-0.017953	-0.020219
	0.002529	0.039825	0.001471	-0.011397	0.003198	-0.013298
	-0.002859	0.007121	0.019238	0.030653	0.041491	0.011973
	-0.023375	0.012131	0.009496	0.003926	-0.018526	-0.034501
	-0.010296					
y489	-0.008487	0.005155	-0.010704	0.003552	-0.010868	-0.029712
	0.003006	0.012766	0.016780	-0.002780	0.017625	0.002113
	-0.000817	-0.017598	-0.001440	0.007972	-0.006099	-0.011187
	-0.017958	0.007085	-0.002761	-0.027209	-0.022179	0.025401
	-0.018315	0.026848	-0.006257	-0.007596	0.030768	-0.006386
	0.012559					

z489	-0.023231	0.018612	-0.032672	-0.002995	-0.004834	0.001698
	0.044648	0.031447	0.022276	0.002389	-0.045160	-0.012325
	0.008582	-0.000127	-0.047834	-0.031222	0.020266	0.008515
	0.002587	-0.008441	-0.023824	-0.006638	-0.016142	-0.007654
	0.012001	-0.020967	0.008814	0.031492	-0.017664	-0.017310
	-0.010647					
x490	-0.013297	-0.005146	0.011916	0.011965	-0.009206	-0.009954
	-0.021095	0.030482	-0.025769	-0.003739	-0.014488	-0.022247
	0.001201	0.036379	0.000453	-0.004999	0.008461	-0.014771
	-0.007614	0.007455	0.021016	0.018006	0.033812	0.013599
	-0.026620	0.006461	0.012186	0.005262	-0.014129	-0.035684
	-0.001533					
y490	-0.006962	0.004327	-0.014558	0.007229	-0.012987	-0.025841
	0.008370	0.011865	0.020460	-0.002174	0.018364	0.004895
	-0.004598	-0.020020	-0.001782	0.008638	0.001026	-0.011217
	-0.011884	0.005867	-0.003572	-0.025462	-0.014847	0.028174
	-0.010737	0.031743	-0.015144	0.000828	0.031433	-0.005813
	0.004619					
z490	-0.026326	0.023808	-0.033707	-0.009722	-0.009392	0.001064
	0.045035	0.028131	0.013291	0.005376	-0.049996	-0.013971
	0.004824	-0.009217	-0.050488	-0.025258	0.007693	0.007770
	-0.001677	-0.008909	-0.021757	-0.016140	-0.011469	-0.004059
	-0.002103	-0.018946	-0.005225	0.014360	-0.021731	-0.019045
	-0.000731					
x491	-0.013721	-0.003141	0.007072	0.006814	-0.009810	-0.012401
	-0.015385	0.029049	-0.025477	-0.002264	-0.007585	-0.021122
	-0.004694	0.032370	0.003321	-0.001936	0.010622	-0.014695
	-0.011059	0.006413	0.024467	0.012176	0.032891	0.013283
	-0.020036	0.007172	0.009475	0.012735	-0.003861	-0.034918
	0.003225					
y491	-0.004518	0.004458	-0.017171	0.009587	-0.015662	-0.022216
	0.012114	0.013421	0.023736	0.000623	0.016814	0.005331
	-0.005247	-0.025093	-0.002732	0.010628	0.005115	-0.011206
	-0.006322	0.002324	-0.004280	-0.027439	-0.012071	0.029219
	-0.013232	0.034140	-0.022629	0.001777	0.025556	-0.006872
	-0.002770					
z491	-0.027425	0.027465	-0.034175	-0.015400	-0.011773	0.002251
	0.044471	0.018290	0.007054	0.004982	-0.051545	-0.013300
	-0.001356	-0.013303	-0.045057	-0.017402	0.006208	0.009420
	-0.002596	-0.010319	-0.011216	-0.016793	-0.009700	0.000627
	-0.004897	-0.013177	-0.015180	0.015804	-0.014269	-0.008486
	0.004696					

x492 -0.013930 -0.002072 0.002221 0.002222 -0.011446 -0.013539
-0.013305 0.027509 -0.025211 -0.000216 -0.003083 -0.019703
-0.011428 0.028971 0.006611 -0.001676 0.011375 -0.017452
-0.014527 0.004321 0.026000 0.007604 0.033972 0.012362
-0.013004 0.009146 0.009282 0.018296 0.001701 -0.032198
0.004417

y492 -0.002158 0.006736 -0.018430 0.011023 -0.016465 -0.018185
0.018224 0.013068 0.027131 0.001793 0.016208 0.008415
-0.003312 -0.032742 -0.003132 0.012032 0.007718 -0.006934
-0.000305 -0.001075 -0.005690 -0.026085 -0.013631 0.027475
-0.018348 0.033070 -0.030072 0.002409 0.018762 -0.006914
-0.010569

z492 -0.028973 0.029778 -0.034547 -0.020719 -0.016151 0.003208
0.037490 0.013503 -0.001736 0.003560 -0.057825 -0.011609
-0.005169 -0.017138 -0.036550 -0.012101 0.001648 0.009401
-0.006953 -0.013373 -0.000078 -0.011544 -0.007498 0.002880
-0.004933 -0.004437 -0.013054 0.013406 -0.011574 -0.003140
0.003158

x493 -0.012896 -0.001088 -0.001104 -0.001552 -0.013466 -0.013626
-0.012150 0.025612 -0.023647 0.002027 0.001327 -0.018726
-0.015611 0.023666 0.011032 -0.003252 0.010443 -0.019374
-0.016423 0.001123 0.025114 0.005224 0.035975 0.010923
-0.006253 0.008757 0.006321 0.023356 0.002528 -0.033186
0.001881

y493 -0.000943 0.010497 -0.019617 0.012251 -0.016463 -0.013804
0.023627 0.012805 0.028846 0.000404 0.015051 0.013217
-0.002031 -0.040471 -0.003355 0.011671 0.009440 0.000145
0.002469 -0.004158 -0.008164 -0.021440 -0.019473 0.025546
-0.023459 0.033428 -0.030485 -0.000351 0.010448 0.001195
-0.017568

z493 -0.027467 0.032047 -0.033795 -0.026615 -0.021250 0.004037
0.031727 0.008606 -0.007552 -0.000511 -0.061390 -0.011724
-0.005704 -0.022823 -0.028421 -0.007711 0.000312 0.016382
-0.014044 -0.013067 0.008018 -0.003440 -0.007079 0.004744
0.002624 -0.002270 -0.009628 0.016301 -0.018784 -0.013242
-0.005436

x494 -0.012116 0.000523 -0.003967 -0.004157 -0.013646 -0.013014
-0.011718 0.025367 -0.022265 0.001789 0.006105 -0.017340
-0.018403 0.017741 0.015401 -0.005390 0.006473 -0.021515
-0.017366 -0.000005 0.021161 0.000996 0.037609 0.011559
-0.002232 0.008704 -0.002855 0.024301 0.005069 -0.024359
-0.002849

y494	-0.000166	0.015225	-0.019975	0.012091	-0.017297	-0.009397
	0.027199	0.012711	0.029030	-0.002820	0.011793	0.018755
	-0.001924	-0.048474	-0.002252	0.010622	0.012096	0.011231
	0.002564	-0.009497	-0.008351	-0.009000	-0.023974	0.022083
	-0.029557	0.033661	-0.025118	-0.004054	0.000200	0.007197
	-0.023830					
z494	-0.026775	0.033552	-0.035059	-0.032226	-0.020870	0.005169
	0.025884	0.009065	-0.011790	-0.010504	-0.061128	-0.014995
	-0.006314	-0.021383	-0.021533	-0.002168	-0.003505	0.017638
	-0.015522	-0.003966	0.009741	-0.003770	-0.009510	0.007017
	0.002827	-0.005190	-0.020300	0.016331	-0.018307	-0.000586
	-0.011661					
x495	-0.011028	0.002980	-0.005517	-0.006257	-0.013489	-0.011992
	-0.010108	0.025481	-0.022440	0.001449	0.008810	-0.011102
	-0.020886	0.010566	0.021522	-0.009239	0.000796	-0.018938
	-0.020939	-0.002193	0.014793	0.000528	0.035756	0.012855
	-0.002296	0.008437	-0.011017	0.024643	0.001232	-0.010483
	-0.009377					
y495	0.000158	0.019915	-0.021988	0.011918	-0.016088	-0.003981
	0.029252	0.013581	0.028575	-0.010800	0.010765	0.020520
	-0.002545	-0.053808	-0.001743	0.009838	0.014531	0.020891
	0.002652	-0.009639	-0.010265	0.002021	-0.026626	0.016836
	-0.031260	0.035414	-0.020495	-0.008494	-0.005651	0.012190
	-0.027156					
z495	-0.024810	0.032868	-0.034018	-0.038668	-0.022865	0.006086
	0.021202	0.008819	-0.019610	-0.016099	-0.062570	-0.015850
	-0.003192	-0.018564	-0.010872	0.002817	-0.004369	0.019386
	-0.021311	0.001671	0.015764	0.003695	-0.017524	0.007374
	0.002868	-0.009675	-0.016939	0.017476	-0.025310	-0.000667
	-0.020964					
x496	-0.010261	0.006045	-0.006071	-0.007167	-0.011471	-0.009348
	-0.007887	0.028881	-0.023686	0.000641	0.013083	-0.002704
	-0.024149	0.000376	0.028498	-0.012948	-0.005503	-0.014179
	-0.024022	-0.001830	0.008535	0.002315	0.033378	0.017170
	-0.003839	0.010816	-0.023866	0.019562	-0.002167	0.005854
	-0.016103					
y496	0.000630	0.023099	-0.023882	0.009486	-0.015058	0.001415
	0.028338	0.013381	0.026287	-0.020103	0.008086	0.019946
	-0.002969	-0.052433	-0.000750	0.011897	0.019123	0.027900
	0.001748	-0.007623	-0.013823	0.009217	-0.028080	0.005047
	-0.036336	0.031576	-0.014308	-0.009940	-0.010124	0.011810
	-0.025276					

z496	-0.023321	0.028810	-0.031266	-0.043057	-0.023058	0.008961
	0.016311	0.012011	-0.023171	-0.015810	-0.055666	-0.019458
	-0.001947	-0.015075	-0.000411	0.008644	-0.002299	0.013649
	-0.020219	0.016134	0.025695	0.016990	-0.022424	0.011143
	0.012012	-0.006022	-0.012754	0.010089	-0.024663	0.000712
	-0.034274					
x497	-0.009621	0.009031	-0.007118	-0.007064	-0.007540	-0.005585
	-0.006598	0.033907	-0.027580	-0.001760	0.019216	0.004511
	-0.024948	-0.010245	0.033307	-0.016112	-0.011829	-0.008928
	-0.027048	-0.000257	-0.001623	-0.001070	0.029526	0.020823
	-0.009743	0.008116	-0.043229	0.012127	-0.008570	0.017601
	-0.019298					
y497	0.001345	0.022091	-0.024876	0.005745	-0.015017	0.008172
	0.026218	0.013518	0.025256	-0.025731	0.005548	0.015123
	-0.002539	-0.043697	0.001728	0.016561	0.026338	0.026451
	0.005212	0.000685	-0.011034	0.018213	-0.023022	-0.009924
	-0.038125	0.028661	0.000162	-0.014132	-0.003529	0.009008
	-0.024050					
z497	-0.021233	0.023107	-0.028488	-0.044760	-0.023098	0.011306
	0.007653	0.013408	-0.027288	-0.016510	-0.042169	-0.027678
	-0.000837	-0.007872	0.005144	0.012098	0.002663	0.002617
	-0.017704	0.028486	0.021178	0.020160	-0.026359	0.011853
	0.021714	-0.007020	-0.016297	0.003663	-0.032575	-0.002517
	-0.032394					
x498	-0.009159	0.009914	-0.009012	-0.006719	-0.003559	0.000369
	-0.005728	0.039810	-0.032869	-0.003230	0.024762	0.008106
	-0.022419	-0.019257	0.034911	-0.016311	-0.019130	-0.005547
	-0.025993	0.005777	-0.010364	-0.009234	0.028645	0.020625
	-0.021532	0.000463	-0.063033	0.004962	-0.012603	0.022447
	-0.022618					
y498	0.001667	0.017324	-0.024928	0.001004	-0.015121	0.015487
	0.022256	0.012840	0.025326	-0.028855	0.005492	0.005650
	0.000163	-0.027787	0.006843	0.021710	0.035164	0.017829
	0.011594	0.012595	-0.009935	0.027744	-0.013163	-0.024364
	-0.032102	0.028127	0.020306	-0.022625	0.005043	0.008184
	-0.014461					
z498	-0.019254	0.015481	-0.024586	-0.043081	-0.025653	0.013427
	-0.003859	0.011974	-0.029492	-0.011937	-0.033252	-0.032839
	-0.005399	-0.002598	0.007117	0.014440	-0.002043	-0.014035
	-0.015708	0.036352	0.011191	0.016606	-0.023411	0.008751
	0.024361	-0.008696	-0.015216	0.014713	-0.020801	-0.019039
	-0.039713					

x499	-0.004783	0.005851	-0.012058	-0.011919	-0.004519	0.006800
	0.000785	0.042899	-0.029373	-0.002911	0.019420	0.005134
	-0.020466	-0.021708	0.034712	-0.011381	-0.010683	-0.001741
	-0.022856	0.013148	-0.010743	-0.008315	0.027295	0.017208
	-0.022692	0.008119	-0.041529	0.014379	-0.006825	0.018655
	-0.025457					
y499	0.001681	0.015749	-0.020564	-0.002393	-0.014061	0.018960
	0.018570	0.009493	0.025326	-0.025667	0.001588	0.004219
	-0.002657	-0.021569	0.014919	0.025526	0.035163	0.013862
	0.012196	0.013896	-0.007762	0.020867	-0.008617	-0.018421
	-0.026471	0.030983	0.024241	-0.017670	0.006381	0.003917
	-0.022173					
z499	-0.016520	0.009495	-0.023132	-0.037936	-0.025615	0.015681
	-0.009680	0.009184	-0.034919	-0.005380	-0.027341	-0.028906
	-0.011140	-0.000717	0.008912	0.016359	-0.003891	-0.017348
	-0.018740	0.031962	0.019636	0.015234	-0.014229	0.021361
	0.021102	-0.018555	-0.017739	0.019986	-0.029205	-0.010795
	-0.055543					
x500	-0.000888	0.001992	-0.015050	-0.017015	-0.006003	0.013875
	0.004165	0.044937	-0.026525	-0.000658	0.014836	0.002021
	-0.018079	-0.021837	0.034760	-0.005297	-0.001477	-0.000814
	-0.019816	0.019631	-0.009483	-0.009116	0.027676	0.012758
	-0.024351	0.013237	-0.025384	0.019544	-0.004427	0.015115
	-0.028774					
y500	0.000960	0.014153	-0.015996	-0.004280	-0.012627	0.021366
	0.013587	0.005137	0.024700	-0.021808	-0.002430	0.003523
	-0.005663	-0.015025	0.020988	0.027187	0.034288	0.010098
	0.010161	0.014044	-0.007199	0.017987	-0.003984	-0.014054
	-0.019393	0.031692	0.025922	-0.009379	0.004875	0.001750
	-0.027492					
z500	-0.013339	0.004528	-0.021444	-0.032406	-0.026102	0.019104
	-0.014084	0.009163	-0.039147	-0.002190	-0.022682	-0.022281
	-0.015776	0.000962	0.011959	0.010259	-0.007669	-0.020875
	-0.017123	0.025006	0.023136	0.006955	-0.004749	0.032874
	0.021907	-0.015876	-0.021570	0.019206	-0.022202	-0.002077
	-0.046600					
x501	0.002584	-0.001749	-0.017440	-0.021481	-0.008048	0.020846
	0.004765	0.045582	-0.024569	0.002799	0.011277	0.000128
	-0.015907	-0.020484	0.034867	0.000292	0.007158	-0.003418
	-0.019761	0.025428	-0.009409	-0.009351	0.029763	0.008697
	-0.025736	0.019052	-0.015307	0.024026	-0.001009	0.013785
	-0.036545					

y501	0.000255	0.013376	-0.011000	-0.004188	-0.011731	0.023097
	0.008613	0.000268	0.024281	-0.018009	-0.005873	0.004314
	-0.008677	-0.007849	0.023565	0.026684	0.030375	0.007492
	0.005063	0.011045	-0.005900	0.015907	0.000811	-0.010904
	-0.014835	0.031843	0.024550	-0.001575	0.006375	0.004342
	-0.027683					
z501	-0.011101	-0.001262	-0.020145	-0.030107	-0.024926	0.021839
	-0.017036	0.009022	-0.039385	-0.004089	-0.015395	-0.014991
	-0.020526	-0.001941	0.016726	0.009774	-0.010611	-0.024282
	-0.014028	0.022053	0.027066	0.003315	-0.001552	0.037641
	0.020447	-0.015927	-0.013455	0.025862	-0.002847	0.001074
	-0.038807					
x502	0.005748	-0.005435	-0.018709	-0.025196	-0.009476	0.027943
	0.002486	0.044969	-0.023279	0.005728	0.008086	0.000180
	-0.013847	-0.018296	0.034828	0.003507	0.013784	-0.007453
	-0.017821	0.028656	-0.010735	-0.011306	0.033475	0.009545
	-0.022934	0.023386	-0.010574	0.028976	-0.000919	0.012099
	-0.041232					
y502	-0.000695	0.013099	-0.005648	-0.002840	-0.010305	0.024355
	0.004386	-0.003942	0.024243	-0.015608	-0.009693	0.005917
	-0.011102	-0.001682	0.024011	0.024217	0.024927	0.007842
	0.001311	0.007341	-0.003557	0.013475	0.003841	-0.005421
	-0.012049	0.028732	0.022744	0.004194	0.008615	0.006998
	-0.023679					
z502	-0.008366	-0.005922	-0.019528	-0.026903	-0.023959	0.023423
	-0.018232	0.006720	-0.041402	-0.005579	-0.007529	-0.008662
	-0.022873	0.000543	0.018452	0.005182	-0.013277	-0.024906
	-0.009424	0.014233	0.030778	0.001618	0.000140	0.025093
	0.019728	-0.016197	-0.008909	0.028628	0.000773	0.000412
	-0.027420					
x503	0.008356	-0.008834	-0.018839	-0.027450	-0.009848	0.034668
	-0.001296	0.044343	-0.022251	0.008113	0.005133	0.001937
	-0.011280	-0.014020	0.033393	0.004238	0.017321	-0.012547
	-0.015900	0.028454	-0.011730	-0.013264	0.036184	0.008439
	-0.019609	0.026070	-0.009424	0.033445	-0.002114	0.011089
	-0.039021					
y503	-0.001932	0.013719	-0.000023	0.000183	-0.008674	0.024775
	0.001319	-0.007412	0.024631	-0.013774	-0.013431	0.007565
	-0.012793	0.003987	0.023283	0.019998	0.018248	0.009851
	-0.001592	0.001756	-0.000224	0.011578	0.005216	-0.001131
	-0.010708	0.021824	0.020335	0.006251	0.008871	0.009451
	-0.016578					

z503	-0.005662	-0.010163	-0.019187	-0.023801	-0.021389	0.024307
	-0.019420	0.005202	-0.042070	-0.008429	-0.004168	-0.002988
	-0.024699	0.004137	0.020094	-0.002963	-0.013441	-0.025089
	-0.005165	0.008896	0.032401	0.002237	0.000938	0.017573
	0.019351	-0.012262	-0.009975	0.026089	0.007213	0.003275
	-0.016063					
x504	0.010388	-0.011454	-0.017732	-0.027616	-0.009378	0.040173
	-0.005246	0.043900	-0.021161	0.009355	0.002813	0.004190
	-0.008501	-0.010217	0.030968	0.002612	0.018540	-0.019058
	-0.015761	0.027280	-0.013193	-0.014585	0.035356	0.004529
	-0.017300	0.026615	-0.010022	0.036107	-0.004386	0.012018
	-0.031334					
y504	-0.003676	0.015253	0.005626	0.004277	-0.007536	0.023533
	-0.000238	-0.010212	0.026222	-0.012581	-0.016788	0.008996
	-0.014248	0.007614	0.023096	0.015022	0.010249	0.013869
	-0.005436	-0.004617	0.003237	0.010717	0.004512	0.002488
	-0.012272	0.012437	0.019370	0.004509	0.008786	0.013092
	-0.012776					
z504	-0.002598	-0.014583	-0.019572	-0.021886	-0.020173	0.024845
	-0.019942	0.000491	-0.042125	-0.011919	0.002087	0.004471
	-0.027302	0.001019	0.019279	-0.005856	-0.014023	-0.024311
	-0.001606	0.005240	0.034057	-0.003244	0.005508	0.010457
	0.010103	-0.007156	-0.007031	0.023609	0.012351	0.005244
	-0.014063					
x505	0.011550	-0.013156	-0.015550	-0.025117	-0.008010	0.044034
	-0.008279	0.044156	-0.019616	0.009647	0.000211	0.007018
	-0.004898	-0.007187	0.028230	-0.001916	0.018187	-0.026463
	-0.016845	0.026144	-0.013960	-0.013686	0.029816	-0.002851
	-0.017899	0.025824	-0.012504	0.033957	-0.008623	0.013214
	-0.018444					
y505	-0.005991	0.017824	0.011065	0.008986	-0.007405	0.020353
	-0.000475	-0.012264	0.028560	-0.010582	-0.021213	0.010337
	-0.014808	0.010187	0.023144	0.008693	0.002518	0.020209
	-0.008838	-0.011809	0.004369	0.011511	0.003157	0.006883
	-0.014150	0.001445	0.019299	-0.001571	0.005719	0.013969
	-0.009247					
z505	-0.000439	-0.018892	-0.021088	-0.021319	-0.015963	0.025025
	-0.021124	-0.000371	-0.042411	-0.015183	0.005033	0.005915
	-0.027066	-0.002710	0.019408	-0.010344	-0.008251	-0.021979
	0.007217	0.003639	0.034618	-0.011052	0.006655	0.009422
	0.014678	-0.008148	-0.003310	0.021943	0.010722	-0.007046
	-0.008014					

x506	0.011439	-0.013549	-0.012849	-0.021687	-0.006553	0.046143
	-0.009290	0.045180	-0.017297	0.008715	-0.002473	0.010073
	0.000462	-0.007110	0.025944	-0.006711	0.015306	-0.034408
	-0.016387	0.024122	-0.016136	-0.012560	0.020613	-0.008796
	-0.022221	0.021046	-0.015046	0.028063	-0.012823	0.013528
	-0.002177					
y506	-0.008694	0.020961	0.016158	0.012890	-0.008665	0.014962
	0.000066	-0.014849	0.032460	-0.006943	-0.024135	0.012474
	-0.014363	0.011193	0.023669	0.003042	-0.004653	0.026717
	-0.012386	-0.022251	0.001424	0.014648	0.004326	0.012821
	-0.016466	-0.007878	0.022047	-0.010257	0.005980	0.017744
	-0.012928					
z506	0.001038	-0.022944	-0.023132	-0.020790	-0.011306	0.022308
	-0.025136	-0.003474	-0.043214	-0.014543	0.013067	0.007572
	-0.028276	-0.007530	0.018774	-0.011141	0.000065	-0.024716
	0.011710	-0.001046	0.030239	-0.015537	0.010665	0.004084
	0.019657	-0.004599	-0.006107	0.018229	0.008486	-0.006600
	-0.006221					
x507	0.009851	-0.012896	-0.009400	-0.018103	-0.005518	0.045134
	-0.009191	0.046290	-0.012790	0.007889	-0.004600	0.013468
	0.008520	-0.010160	0.025020	-0.012719	0.012916	-0.043302
	-0.013556	0.019298	-0.022939	-0.010719	0.009884	-0.012551
	-0.027631	0.016246	-0.015118	0.016947	-0.017444	0.013636
	0.013522					
y507	-0.011379	0.023651	0.020683	0.015838	-0.012049	0.009463
	0.001819	-0.016674	0.036517	-0.003564	-0.026743	0.017005
	-0.010666	0.013737	0.020329	0.001290	-0.011018	0.035425
	-0.014149	-0.030944	-0.000141	0.017793	0.005226	0.018411
	-0.023861	-0.018255	0.031109	-0.018874	0.016433	0.015683
	-0.017423					
z507	0.002113	-0.025744	-0.025101	-0.018924	-0.009153	0.021668
	-0.027945	-0.005909	-0.045009	-0.014308	0.019082	0.010507
	-0.028199	-0.013458	0.012931	-0.006943	0.005892	-0.027430
	0.014646	0.005853	0.032185	-0.016994	0.010525	-0.009484
	0.011262	-0.008576	-0.004176	0.019433	0.015488	-0.009311
	0.005407					
x508	0.007135	-0.012534	-0.007094	-0.016051	-0.004429	0.044300
	-0.015133	0.045605	-0.008937	0.005577	-0.007576	0.011840
	0.009111	-0.007341	0.017056	-0.012766	0.015579	-0.043922
	-0.005762	0.016485	-0.028237	-0.011733	0.017716	-0.012673
	-0.020049	0.004914	-0.014689	0.016125	-0.026385	0.021582
	0.020803					

y508	-0.012142	0.021236	0.018482	0.017874	-0.012272	0.007936
	-0.004005	-0.019910	0.030551	-0.002279	-0.019001	0.017523
	-0.011765	0.014471	0.019717	0.002307	-0.008491	0.035340
	-0.006595	-0.031102	0.007625	0.016067	0.008653	0.009816
	-0.016491	-0.014517	0.023035	-0.017098	0.014514	0.006305
	-0.025048					
z508	0.002433	-0.024917	-0.026672	-0.016746	-0.005191	0.017868
	-0.027568	-0.004163	-0.045525	-0.011786	0.018368	0.011819
	-0.026086	-0.012183	0.006339	-0.007613	0.013398	-0.027188
	0.018457	0.007061	0.029317	-0.019396	0.007890	-0.011625
	0.011637	-0.008381	-0.008192	0.011735	0.008190	-0.006429
	0.024407					
x509	0.004865	-0.012250	-0.005188	-0.014582	-0.003705	0.043188
	-0.020827	0.044076	-0.005563	0.002329	-0.007866	0.010899
	0.007936	-0.005437	0.008455	-0.012153	0.016643	-0.043590
	0.000171	0.013693	-0.030295	-0.012241	0.023164	-0.013326
	-0.013031	-0.003314	-0.013256	0.017065	-0.030664	0.024765
	0.027334					
y509	-0.013163	0.018561	0.016567	0.018070	-0.012061	0.005598
	-0.010005	-0.023918	0.025903	-0.002809	-0.012380	0.019449
	-0.011087	0.014121	0.020165	0.005956	-0.006645	0.033047
	-0.002847	-0.031723	0.012608	0.014097	0.012704	0.003674
	-0.012659	-0.011729	0.021070	-0.012661	0.019611	-0.001963
	-0.036315					
z509	0.002230	-0.023755	-0.028483	-0.016259	-0.001445	0.014304
	-0.026918	-0.005099	-0.043075	-0.013305	0.017568	0.012872
	-0.027562	-0.016331	0.000717	-0.009500	0.014816	-0.030249
	0.013155	0.008299	0.024127	-0.020125	0.009625	-0.009207
	0.003845	-0.011444	-0.007942	0.007570	0.004026	-0.003610
	0.033171					
x510	0.002703	-0.012033	-0.003736	-0.013865	-0.003365	0.042271
	-0.025927	0.041976	-0.002561	-0.000975	-0.006907	0.011447
	0.006943	-0.003805	0.000623	-0.011342	0.016634	-0.043467
	0.006312	0.009941	-0.028757	-0.010417	0.026770	-0.013649
	-0.007076	-0.010174	-0.010673	0.017896	-0.028868	0.024522
	0.030434					
y510	-0.014200	0.015925	0.015646	0.017722	-0.011059	0.003242
	-0.015619	-0.025490	0.021292	-0.002289	-0.007767	0.021976
	-0.008646	0.015837	0.021130	0.010802	-0.003700	0.030960
	0.000557	-0.033079	0.016036	0.011530	0.014595	-0.000132
	-0.008641	-0.008339	0.021921	-0.009751	0.022363	-0.008661
	-0.046451					

z510	0.001679	-0.022347	-0.029646	-0.017218	0.002792	0.012180
	-0.025813	-0.003204	-0.041125	-0.012771	0.018054	0.012327
	-0.028830	-0.018031	-0.004189	-0.011209	0.016552	-0.031764
	0.011538	0.006634	0.021654	-0.018394	0.009236	-0.003412
	-0.000235	-0.014923	-0.006776	0.000417	0.000437	0.000998
	0.033031					
x511	0.000665	-0.011608	-0.002234	-0.013278	-0.003590	0.041738
	-0.030929	0.038585	0.000209	-0.004990	-0.005552	0.012132
	0.006339	-0.002971	-0.005291	-0.009310	0.015940	-0.041176
	0.010638	0.006765	-0.024508	-0.008916	0.028202	-0.014554
	-0.000409	-0.014389	-0.007871	0.019781	-0.023679	0.022843
	0.035137					
y511	-0.015361	0.014020	0.015184	0.017975	-0.010454	0.000757
	-0.020001	-0.027072	0.018330	-0.001808	-0.005658	0.023561
	-0.005390	0.018582	0.022257	0.014949	-0.000831	0.029270
	0.002550	-0.033516	0.018622	0.009575	0.013605	-0.006080
	-0.005440	-0.001594	0.021717	-0.005374	0.020052	-0.011409
	-0.049273					
z511	0.001103	-0.019920	-0.030772	-0.017975	0.005271	0.010630
	-0.022456	-0.003988	-0.037029	-0.012389	0.017644	0.009807
	-0.031393	-0.019937	-0.008295	-0.013682	0.017776	-0.029246
	0.010045	0.007605	0.023733	-0.013749	0.005804	-0.003436
	-0.002904	-0.012747	-0.008875	-0.003064	-0.005700	0.008735
	0.040049					
x512	-0.001826	-0.011270	-0.000798	-0.012753	-0.003965	0.041522
	-0.036228	0.035096	0.002635	-0.009076	-0.004532	0.013237
	0.007038	-0.001898	-0.009374	-0.006663	0.012985	-0.038728
	0.015079	0.003670	-0.017983	-0.008170	0.029055	-0.017320
	0.003906	-0.017147	-0.006874	0.022391	-0.014244	0.019825
	0.038280					
y512	-0.016559	0.012693	0.015122	0.018077	-0.010007	-0.002049
	-0.023359	-0.028287	0.017030	-0.001809	-0.005141	0.023917
	-0.001958	0.021492	0.021891	0.017798	0.001791	0.026104
	0.003979	-0.034062	0.018025	0.007170	0.009833	-0.010590
	-0.004375	0.000824	0.021538	0.000951	0.014275	-0.013152
	-0.050489					
z512	-0.000101	-0.017522	-0.032358	-0.019788	0.006886	0.009138
	-0.018551	-0.004687	-0.031786	-0.012025	0.016966	0.006844
	-0.033576	-0.022458	-0.012226	-0.016551	0.015487	-0.028179
	0.010335	0.008784	0.027066	-0.008417	0.001922	-0.006338
	-0.010252	-0.016159	-0.013342	-0.004171	-0.006344	0.014102
	0.043051					

x513	-0.004554	-0.010888	0.000725	-0.012077	-0.004502	0.040901
	-0.041996	0.031650	0.004439	-0.014031	-0.004061	0.014402
	0.007153	-0.000883	-0.012863	-0.003468	0.011733	-0.034883
	0.016598	0.002650	-0.010190	-0.007786	0.028673	-0.018668
	0.009173	-0.018138	-0.002678	0.023835	-0.005476	0.014361
	0.039043					
y513	-0.017285	0.011078	0.014521	0.016935	-0.008230	-0.005255
	-0.024348	-0.026662	0.013664	0.000312	-0.005614	0.024914
	-0.001019	0.024496	0.023783	0.020404	0.007052	0.023178
	0.002248	-0.034784	0.013866	0.008483	0.004811	-0.009640
	-0.007636	0.002360	0.023921	0.003826	0.007075	-0.011880
	-0.049707					
z513	-0.001018	-0.015876	-0.034493	-0.023284	0.008637	0.007379
	-0.013207	-0.003561	-0.029454	-0.010846	0.016668	0.005140
	-0.038629	-0.026555	-0.014092	-0.018027	0.017342	-0.025441
	0.005395	0.012183	0.031675	-0.000604	-0.000977	-0.005422
	-0.020037	-0.019484	-0.011519	-0.010179	-0.001584	0.015473
	0.039510					
x514	-0.007582	-0.009753	0.004153	-0.010917	-0.006372	0.041184
	-0.047451	0.022543	0.007160	-0.019256	-0.003563	0.013203
	0.010316	-0.000233	-0.015276	-0.002335	0.006881	-0.030693
	0.019790	-0.000106	-0.002844	-0.006898	0.029027	-0.021466
	0.013809	-0.016148	-0.002278	0.026545	0.004053	0.011053
	0.041089					
y514	-0.017927	0.010824	0.014251	0.016624	-0.008005	-0.008776
	-0.025175	-0.028316	0.014247	0.001870	-0.006644	0.020941
	0.000989	0.025369	0.019788	0.018586	0.008647	0.017365
	0.002819	-0.038258	0.009289	0.011015	-0.000148	-0.006558
	-0.006019	0.005365	0.024856	0.012932	-0.001766	-0.013549
	-0.047864					
z514	-0.001659	-0.013916	-0.035220	-0.026303	0.008521	0.006404
	-0.007318	-0.007877	-0.025382	-0.009068	0.016855	0.000014
	-0.040883	-0.032932	-0.017155	-0.023375	0.016273	-0.021718
	0.004744	0.014759	0.036711	0.008159	-0.000800	-0.004756
	-0.027171	-0.020324	-0.013356	-0.015247	0.003836	0.011531
	0.035432					
x515	-0.010542	-0.007727	0.006471	-0.008161	-0.006949	0.039980
	-0.054276	0.017689	0.008673	-0.026101	-0.001994	0.012448
	0.009065	0.002201	-0.017571	0.003909	0.003888	-0.026061
	0.018060	-0.005892	0.004201	-0.007325	0.027856	-0.020299
	0.019169	-0.012951	-0.005365	0.028827	0.010007	0.007160
	0.046114					

y515	-0.017029	0.010015	0.012411	0.014877	-0.004878	-0.013720
	-0.022029	-0.024036	0.010791	0.006309	-0.004256	0.023080
	-0.003255	0.028362	0.019282	0.019519	0.014871	0.011474
	0.000576	-0.042313	0.002263	0.016714	-0.008781	0.002833
	-0.004436	0.007067	0.027746	0.018124	-0.015124	-0.014538
	-0.039793					
z515	-0.001434	-0.012579	-0.038719	-0.029934	0.009450	0.004657
	-0.000933	-0.005944	-0.023855	-0.009491	0.019096	-0.003561
	-0.048209	-0.038419	-0.018570	-0.023895	0.019865	-0.018175
	0.001091	0.016472	0.045455	0.016280	0.000759	-0.005226
	-0.035199	-0.022522	-0.017166	-0.022708	0.009720	0.006961
	0.031703					
x516	-0.013458	-0.006005	0.008189	-0.005920	-0.008244	0.038731
	-0.058960	0.010964	0.009029	-0.034916	-0.002406	0.005643
	0.003016	0.005408	-0.015703	0.008222	0.001947	-0.021010
	0.018041	-0.013922	0.011853	-0.001279	0.031724	-0.019682
	0.011236	-0.012760	-0.019059	0.023354	0.013378	0.013857
	0.054518					
y516	-0.014908	0.007317	0.009392	0.011226	-0.000292	-0.020519
	-0.014591	-0.017994	0.006121	0.015210	0.000131	0.027143
	-0.008360	0.032183	0.021834	0.019465	0.016855	0.001211
	-0.000814	-0.043125	-0.010824	0.022631	-0.016577	0.012926
	-0.004332	0.005921	0.031973	0.023438	-0.027939	-0.010964
	-0.027467					
z516	-0.001215	-0.012282	-0.041077	-0.034920	0.011067	0.002672
	0.008791	-0.003287	-0.022436	-0.009114	0.021528	-0.004337
	-0.053418	-0.043552	-0.011963	-0.024461	0.015122	-0.013966
	0.000616	0.024275	0.051391	0.023914	0.008830	-0.014859
	-0.052556	-0.026086	-0.026680	-0.036019	0.024522	0.003013
	0.020929					
x517	-0.013925	-0.007446	0.012769	-0.007476	-0.005657	0.036146
	-0.061476	0.010919	0.014278	-0.024148	-0.000665	0.005966
	-0.011924	0.003418	-0.018725	0.015971	0.007763	-0.019492
	0.013558	-0.014684	0.008218	-0.001659	0.020076	-0.018050
	0.006490	-0.015196	-0.029570	0.020408	0.017178	0.002216
	0.041527					
y517	-0.014042	0.004022	0.001588	0.008173	-0.001050	-0.029395
	-0.010212	-0.023219	-0.000084	0.017194	-0.005295	0.011791
	-0.003011	0.028573	0.021033	0.013305	0.011875	0.002609
	-0.004699	-0.047972	-0.008616	0.029693	-0.016059	0.013905
	-0.001437	-0.001272	0.034767	0.029111	-0.032363	-0.001877
	-0.025645					

z517	-0.003783	-0.007630	-0.035060	-0.031254	0.013144	0.002953
	0.009451	0.007175	-0.013864	-0.012472	0.029683	0.011366
	-0.049827	-0.037057	-0.010339	-0.014115	0.008201	-0.002273
	0.003739	0.039206	0.038198	0.008627	0.006658	-0.010179
	-0.039871	-0.018124	-0.019679	-0.032297	0.033326	-0.013645
	0.013313					
x518	-0.016296	-0.006554	0.017492	-0.007036	-0.004294	0.031781
	-0.063803	0.007722	0.021025	-0.019537	-0.001375	0.006706
	-0.025109	0.002464	-0.026238	0.017187	0.007010	-0.019850
	0.008210	-0.015876	0.005717	0.000033	0.010172	-0.012421
	0.011887	-0.008070	-0.033443	0.022626	0.017499	-0.012716
	0.022533					
y518	-0.013056	0.001799	-0.009775	0.003139	-0.003556	-0.035720
	-0.003373	-0.027017	-0.008971	0.010788	-0.012265	-0.004607
	-0.000414	0.022897	0.020563	0.002183	0.012148	0.001736
	-0.006031	-0.052675	-0.002705	0.043338	-0.007668	0.014364
	-0.005210	-0.008627	0.034048	0.032086	-0.034156	0.005612
	-0.028045					
z518	-0.006427	-0.002905	-0.029047	-0.029631	0.013441	0.003135
	0.012239	0.010869	-0.006734	-0.020282	0.033259	0.021484
	-0.043081	-0.031024	-0.009864	-0.014484	0.005847	0.005210
	0.012979	0.049117	0.030315	0.003987	0.007646	-0.008960
	-0.032029	-0.011669	-0.014949	-0.028935	0.032898	-0.032056
	-0.000133					
x519	-0.018130	-0.004581	0.020756	-0.006539	-0.001504	0.025362
	-0.064407	0.004793	0.025661	-0.013430	0.002862	0.010247
	-0.038257	0.004089	-0.026215	0.020912	0.005797	-0.020692
	0.001168	-0.023959	-0.001775	0.005115	0.003641	-0.004116
	0.020186	0.007782	-0.041856	0.021553	0.015783	-0.012540
	0.009091					
y519	-0.011778	-0.001147	-0.019985	-0.001566	-0.003378	-0.043238
	0.004861	-0.028596	-0.017203	0.011401	-0.015384	-0.012676
	0.005276	0.020098	0.023177	-0.001567	0.008410	-0.001502
	-0.008044	-0.051695	-0.004025	0.050296	0.000533	0.014882
	-0.000727	-0.011115	0.035280	0.034635	-0.030387	0.010416
	-0.029886					
z519	-0.008443	0.002155	-0.021894	-0.027834	0.014901	0.000636
	0.014052	0.014947	0.001029	-0.025335	0.038917	0.034744
	-0.035175	-0.021433	-0.005252	-0.011674	0.001398	0.013327
	0.020312	0.053887	0.015228	0.000324	0.009519	-0.007439
	-0.019935	0.000326	-0.012739	-0.023247	0.028396	-0.041677
	-0.007425					

x520	-0.021030	-0.003448	0.023991	-0.005824	0.000607	0.019016
	-0.063238	0.002282	0.029050	-0.007326	0.004772	0.014662
	-0.045097	0.006323	-0.028210	0.025586	0.006498	-0.014839
	-0.001588	-0.023699	0.002474	0.012015	-0.007950	0.008963
	0.019135	0.009351	-0.041698	0.013282	0.025618	-0.024184
	0.005339					
y520	-0.009930	-0.005576	-0.029611	-0.007022	-0.001651	-0.050703
	0.012989	-0.028761	-0.025404	0.016929	-0.016932	-0.015831
	0.013250	0.017761	0.025193	0.000719	0.003203	-0.005473
	-0.008903	-0.044810	-0.008454	0.052101	0.011184	0.014721
	0.005617	-0.014188	0.036388	0.035427	-0.021028	0.010241
	-0.029379					
z520	-0.010529	0.006545	-0.014048	-0.026809	0.016367	-0.002560
	0.014812	0.020036	0.008827	-0.027745	0.042251	0.049132
	-0.025843	-0.011076	-0.000478	-0.009296	0.000428	0.026040
	0.029097	0.058670	0.007278	0.004397	0.006646	-0.002600
	-0.013786	0.004651	-0.001731	-0.019170	0.025130	-0.057673
	-0.010620					
x521	-0.022904	-0.002979	0.021697	-0.008521	0.003193	0.007278
	-0.061790	-0.001713	0.028206	-0.006432	0.010458	0.012255
	-0.049862	0.011479	-0.030023	0.026950	0.009507	-0.020185
	-0.003912	-0.024721	0.002901	0.011484	-0.012333	0.012555
	0.012890	0.011736	-0.045535	0.005931	0.028953	-0.021648
	-0.010187					
y521	-0.007838	-0.010235	-0.040592	-0.014234	0.000709	-0.057961
	0.020480	-0.027546	-0.034336	0.021182	-0.020702	-0.018974
	0.021899	0.015339	0.027017	0.003948	-0.001117	-0.007955
	-0.010248	-0.033716	-0.013019	0.052494	0.023434	0.013351
	0.009172	-0.017596	0.038187	0.034227	-0.012017	0.009309
	-0.029244					
z521	-0.010372	0.011157	-0.007614	-0.027832	0.017132	-0.007559
	0.013799	0.020254	0.015064	-0.026955	0.052096	0.061155
	-0.017134	0.002158	0.004395	-0.004564	-0.002130	0.030955
	0.034371	0.048682	-0.007098	0.005148	0.007110	-0.003478
	-0.013175	0.012726	-0.005272	-0.010073	0.012998	-0.059032
	-0.015274					
x522	-0.024469	-0.005600	0.014916	-0.014634	0.005702	-0.011052
	-0.056932	-0.009323	0.026247	-0.003782	0.008591	0.001561
	-0.050030	0.017268	-0.031198	0.022269	0.012737	-0.030474
	0.002633	-0.019114	0.006783	0.017409	-0.008979	0.010072
	0.011049	0.013210	-0.041356	-0.006198	0.027129	-0.014895
	-0.018877					

y522	-0.004293	-0.013404	-0.051065	-0.022014	0.002144	-0.063623
	0.028608	-0.026396	-0.043565	0.024972	-0.025169	-0.017379
	0.033633	0.013484	0.026036	0.015498	-0.013631	-0.007002
	-0.009776	-0.025491	-0.023124	0.046483	0.037257	0.013630
	0.009794	-0.023166	0.029554	0.039883	-0.000700	0.003265
	-0.015677					
z522	-0.007865	0.015615	-0.001994	-0.028801	0.016378	-0.011801
	0.012809	0.016529	0.020658	-0.021190	0.065846	0.072141
	-0.007486	0.013789	0.009166	0.002885	-0.005800	0.029793
	0.036591	0.023965	-0.022751	0.011840	0.016917	-0.005606
	-0.010671	0.022554	-0.022911	-0.001227	-0.003700	-0.050033
	-0.006340					
x523	-0.025033	-0.011826	-0.000005	-0.029609	0.011637	-0.045323
	-0.050022	-0.014561	0.018472	-0.000306	0.001003	-0.015980
	-0.054380	0.024500	-0.041386	0.022216	0.018365	-0.038272
	0.012168	0.004894	0.009879	0.030696	-0.005990	0.018226
	0.009864	0.003210	-0.015751	-0.009178	0.033584	-0.009504
	-0.026891					
y523	0.000581	-0.017327	-0.060675	-0.031105	0.007259	-0.068295
	0.037810	-0.020898	-0.054346	0.032070	-0.028534	-0.007329
	0.050310	0.011538	0.026478	0.034273	-0.033505	-0.007781
	-0.009804	-0.013578	-0.038142	0.030112	0.049496	0.009898
	0.010308	-0.021820	0.016247	0.049740	0.008743	-0.004614
	-0.001150					
z523	-0.005250	0.019060	0.004561	-0.029863	0.016828	-0.016758
	0.012962	0.009803	0.023537	-0.013262	0.079784	0.082495
	-0.001854	0.020633	0.015991	0.003795	-0.004394	0.032642
	0.036127	-0.003252	-0.034647	0.031378	0.024093	-0.006721
	-0.003819	0.024573	-0.040285	0.009170	-0.028751	-0.035459
	-0.006761					
x524	-0.025902	-0.021964	-0.022177	-0.052254	0.027708	-0.091659
	-0.041438	-0.016974	0.008291	0.007973	-0.009596	-0.030985
	-0.052671	0.027306	-0.062543	0.030796	0.020687	-0.057612
	0.032724	0.036381	0.019132	0.033273	-0.004321	0.035548
	0.003329	-0.006384	0.022452	-0.013225	0.039663	0.001715
	-0.027142					
y524	0.004372	-0.020479	-0.059253	-0.032343	0.021525	-0.061592
	0.045777	-0.011019	-0.062151	0.038712	-0.031566	0.014796
	0.062048	0.007069	0.034731	0.046361	-0.055522	-0.004297
	-0.032202	0.005947	-0.046546	0.007205	0.043893	0.004730
	0.018175	-0.014409	0.009236	0.052944	-0.005696	-0.004898
	0.004955					

z524	-0.003473	0.019712	0.008713	-0.032461	0.018667	-0.025295
	0.014242	0.001836	0.021626	-0.003058	0.085610	0.085345
	0.007105	0.020081	0.019957	-0.008793	0.007314	0.031386
	0.040078	-0.027232	-0.032953	0.054288	0.022347	-0.009579
	0.004522	0.017272	-0.051657	0.017936	-0.053536	-0.024394
	-0.028693					
x525	-0.025898	-0.026340	-0.033214	-0.059208	0.043830	-0.111714
	-0.034396	-0.025189	0.004387	0.010214	-0.010410	-0.037043
	-0.044025	0.017649	-0.072778	0.038180	0.010059	-0.076489
	0.027058	0.041687	0.042530	0.019290	-0.013785	0.041869
	0.006951	-0.008234	0.035791	-0.015274	0.023504	0.018200
	-0.022160					
y525	0.006110	-0.017764	-0.046329	-0.025233	0.027203	-0.043025
	0.047980	0.002275	-0.061841	0.046305	-0.037910	0.047265
	0.070904	0.010548	0.044155	0.047745	-0.062503	0.010971
	-0.051731	0.015002	-0.055060	-0.008865	0.032274	0.003135
	0.023195	-0.009167	0.001470	0.044574	0.000452	-0.027938
	0.016831					
z525	-0.002212	0.018280	0.006730	-0.034654	0.019475	-0.034979
	0.015920	-0.005566	0.015074	0.003514	0.077951	0.071433
	0.017222	0.018579	0.017238	-0.028613	0.018666	0.025398
	0.041692	-0.038044	-0.010783	0.067612	0.009090	-0.008133
	0.006710	0.002035	-0.035991	0.018623	-0.071280	-0.010525
	-0.045429					
x526	0.000886	-0.021251	-0.031978	-0.005744	-0.006044	0.025632
	0.005369	-0.029109	0.007095	0.008746	0.016795	-0.004136
	0.012468	-0.008703	0.022386	-0.000799	-0.004905	-0.004975
	-0.003492	0.006212	0.056853	-0.037357	0.008004	-0.006186
	0.016765	-0.005961	0.062717	0.022000	0.010235	-0.005393
	-0.059767					
y526	-0.008691	0.001933	0.009181	0.034104	0.002849	-0.024912
	0.027266	-0.036368	-0.020827	0.012179	-0.020640	-0.001792
	-0.008836	-0.008514	0.004782	-0.012370	0.005000	-0.004006
	0.014117	-0.069971	0.034964	0.016589	0.012665	0.018429
	0.018997	0.018483	0.031344	0.075589	0.062434	0.004673
	0.024731					
z526	0.002544	0.030437	-0.024478	-0.002905	-0.007998	0.030262
	0.003888	0.003006	-0.053245	-0.035520	0.003582	0.001777
	0.040039	0.036073	0.088452	-0.015151	-0.008339	0.023889
	-0.042606	-0.006182	-0.097452	-0.006110	-0.006238	0.063582
	0.036977	0.025509	0.033734	-0.046797	0.105659	0.017348
	-0.027341					

x527 -0.000007 -0.020609 -0.027241 -0.006717 -0.006944 0.028472
-0.001871 -0.025591 0.011188 -0.004478 0.016414 -0.000799
0.008320 0.000659 0.021336 -0.011728 0.008208 -0.011489
0.008675 0.011903 0.036633 -0.034453 0.013066 -0.014832
0.019199 -0.007154 0.039744 0.018114 0.023864 0.002551
-0.036606

y527 -0.005892 0.000613 0.008534 0.032558 0.002968 -0.025456
0.031907 -0.028285 -0.024269 0.013961 -0.017810 -0.002569
-0.009978 0.002252 0.008323 -0.014324 0.005369 -0.003376
0.015806 -0.070370 0.033603 0.017607 0.011678 0.011957
0.018016 0.013920 0.037247 0.067377 0.056917 -0.002570
0.026142

z527 0.000333 0.033423 -0.020664 0.003910 -0.010554 0.025934
0.003770 0.001252 -0.050655 -0.035624 -0.001252 -0.000890
0.027215 0.033243 0.069235 -0.025776 -0.012783 0.017170
-0.031115 0.001611 -0.084523 -0.005702 -0.011034 0.049441
0.034194 0.026427 0.029166 -0.029633 0.090380 0.009106
-0.020411

x528 -0.000687 -0.023362 -0.022729 -0.011694 -0.003950 0.030003
-0.003801 -0.020098 0.005880 -0.008788 0.015015 0.006616
0.001444 0.009904 0.032460 -0.020574 0.018884 -0.003697
0.015804 0.022580 0.011861 -0.032088 0.011701 -0.011743
0.014465 -0.010070 0.022486 0.009654 0.029341 0.010207
-0.022331

y528 -0.004098 0.000289 0.009793 0.032726 0.001410 -0.027142
0.033537 -0.021452 -0.021986 0.012576 -0.013310 -0.005273
-0.012590 0.011579 0.002824 -0.016098 0.005538 -0.006853
0.021143 -0.069958 0.039307 0.019040 0.014485 0.003495
0.020119 0.007824 0.041326 0.059208 0.045340 -0.000653
0.027972

z528 -0.001629 0.036391 -0.017737 0.010554 -0.011817 0.022008
0.003582 -0.001040 -0.047090 -0.033682 -0.004577 -0.002032
0.016698 0.029363 0.051252 -0.032098 -0.016239 0.008917
-0.021252 0.008756 -0.072613 -0.005201 -0.010378 0.039769
0.027702 0.025960 0.025523 -0.019828 0.075338 0.008016
-0.016374

x529 -0.001904 -0.023952 -0.021862 -0.012131 -0.004077 0.029827
-0.003556 -0.016715 0.000345 -0.012673 0.010971 0.013633
0.001491 0.016613 0.031452 -0.025931 0.022999 0.004493
0.013583 0.028368 0.001402 -0.027011 0.003954 -0.018672
0.014621 -0.001841 0.015761 0.011226 0.027396 0.009608
-0.007745

y529	-0.003035	-0.001640	0.013354	0.029469	0.002099	-0.029347
	0.034617	-0.014470	-0.019350	0.012386	-0.006523	-0.006850
	-0.018541	0.018728	0.002057	-0.016737	0.007786	-0.007752
	0.030959	-0.065259	0.040471	0.016795	0.020084	0.006287
	0.020421	-0.003566	0.041248	0.045045	0.030807	0.008800
	0.022704					
z529	-0.003426	0.039616	-0.015099	0.016365	-0.012038	0.018873
	0.001824	-0.004627	-0.042509	-0.031808	-0.007542	-0.003202
	0.007503	0.025603	0.034317	-0.036352	-0.020694	-0.000329
	-0.010174	0.015367	-0.062452	-0.006730	-0.005428	0.036224
	0.020003	0.023433	0.020973	-0.013458	0.060089	0.010741
	-0.017835					
x530	-0.003735	-0.026237	-0.023696	-0.012816	-0.004107	0.028723
	-0.005561	-0.014276	-0.003081	-0.013856	0.006101	0.013348
	0.010090	0.019992	0.034371	-0.029392	0.023342	0.014153
	0.015780	0.038120	-0.000572	-0.027829	-0.007959	-0.017454
	0.023956	0.001729	0.017401	0.023861	0.022846	0.017325
	-0.011235					
y530	-0.002412	-0.003711	0.017538	0.026014	0.002260	-0.032098
	0.037159	-0.008378	-0.017786	0.012319	-0.001215	-0.005086
	-0.025417	0.024454	-0.002768	-0.018017	0.009402	-0.008745
	0.036088	-0.060124	0.039428	0.014344	0.026131	0.006391
	0.017657	-0.009593	0.041466	0.030586	0.015669	0.012644
	0.020367					
z530	-0.004529	0.043386	-0.012804	0.021896	-0.012278	0.016781
	0.000761	-0.009888	-0.039782	-0.030211	-0.010916	-0.000279
	-0.002188	0.022038	0.015313	-0.038377	-0.026651	-0.009481
	-0.006186	0.016887	-0.057177	-0.006482	0.004238	0.032360
	0.006518	0.024755	0.013125	-0.010868	0.044449	0.008229
	-0.011480					
x531	-0.005340	-0.026848	-0.024715	-0.012061	-0.004378	0.027062
	-0.006872	-0.016181	-0.006662	-0.014583	-0.000240	0.018901
	0.011276	0.021674	0.030435	-0.030276	0.019283	0.016855
	0.016155	0.043979	-0.002476	-0.029439	-0.009266	-0.014081
	0.022098	0.008766	0.013574	0.027473	0.011001	0.017383
	-0.007598					
y531	-0.002088	-0.005541	0.020820	0.021592	0.002069	-0.034311
	0.040482	-0.001968	-0.015738	0.013184	0.004647	-0.006062
	-0.027175	0.030090	-0.004095	-0.018612	0.009822	-0.006321
	0.040775	-0.054901	0.037650	0.007957	0.026863	0.009117
	0.021489	-0.013349	0.044170	0.020612	0.005056	0.018374
	0.013205					

z531	-0.005914	0.047162	-0.011492	0.026810	-0.011019	0.015737
	-0.000663	-0.012818	-0.036229	-0.029483	-0.013525	-0.001165
	-0.008355	0.019110	0.001280	-0.039083	-0.026774	-0.015217
	-0.000799	0.020721	-0.051080	-0.004583	0.007221	0.029091
	-0.001073	0.022790	0.010260	-0.007838	0.036345	0.011217
	-0.011677					
x532	-0.007044	-0.026045	-0.026077	-0.011111	-0.003530	0.025701
	-0.007774	-0.016652	-0.008412	-0.017021	-0.006992	0.025190
	0.011483	0.020483	0.025146	-0.031413	0.016565	0.015047
	0.015485	0.048610	-0.003768	-0.027043	-0.008759	-0.010176
	0.013787	0.015707	0.015470	0.027822	-0.000978	0.015437
	-0.010465					
y532	-0.001711	-0.007563	0.023854	0.016898	0.000949	-0.036239
	0.044304	0.003448	-0.013638	0.014903	0.009668	-0.007218
	-0.028438	0.035005	-0.004508	-0.018411	0.008758	-0.003449
	0.046080	-0.049674	0.035665	-0.000997	0.026521	0.014193
	0.027713	-0.016678	0.045885	0.013194	-0.004189	0.023511
	0.008261					
z532	-0.006919	0.050469	-0.010656	0.031460	-0.009719	0.015319
	-0.002723	-0.017468	-0.033833	-0.028110	-0.015982	-0.002751
	-0.013007	0.017331	-0.010272	-0.037649	-0.026908	-0.018693
	0.003617	0.023752	-0.046709	-0.004466	0.009074	0.027351
	-0.005074	0.020523	0.004247	-0.004851	0.030042	0.013411
	-0.007721					
x533	-0.008612	-0.024338	-0.027984	-0.008389	-0.003236	0.024545
	-0.009993	-0.018084	-0.008801	-0.019814	-0.013679	0.027414
	0.013326	0.020798	0.023213	-0.033148	0.015147	0.013740
	0.015462	0.052273	-0.006332	-0.025578	-0.011589	-0.008271
	0.008715	0.018284	0.009947	0.028628	-0.006819	0.019110
	-0.007746					
y533	-0.001477	-0.009415	0.026702	0.011120	-0.000171	-0.037787
	0.048298	0.007854	-0.011336	0.016294	0.014163	-0.006301
	-0.030102	0.037526	-0.006222	-0.017653	0.006058	-0.001684
	0.049981	-0.045711	0.033681	-0.010857	0.028441	0.021984
	0.030801	-0.018761	0.050543	0.006198	-0.013669	0.023240
	0.002030					
z533	-0.007705	0.053642	-0.010084	0.034623	-0.007094	0.015221
	-0.003769	-0.021566	-0.032526	-0.026811	-0.018006	-0.002349
	-0.017480	0.014067	-0.021972	-0.034174	-0.025848	-0.022641
	0.006665	0.025731	-0.041697	-0.002927	0.011434	0.028417
	-0.009325	0.020494	0.004682	-0.002887	0.021923	0.010029
	-0.009651					

x534	-0.009414	-0.022065	-0.030163	-0.005825	-0.002348	0.024265
	-0.011169	-0.020352	-0.010254	-0.022631	-0.020238	0.032027
	0.014177	0.020253	0.021362	-0.032237	0.014669	0.012318
	0.018019	0.055005	-0.007565	-0.023823	-0.011894	-0.002259
	0.005500	0.023188	0.005766	0.029335	-0.014368	0.023932
	-0.002691					
y534	-0.001479	-0.010603	0.029329	0.004976	-0.001009	-0.038029
	0.051269	0.012644	-0.007238	0.016496	0.019031	-0.006441
	-0.028778	0.038213	-0.006674	-0.017779	0.003044	-0.001832
	0.051845	-0.041870	0.027401	-0.019052	0.028375	0.028310
	0.029355	-0.019582	0.053966	-0.003895	-0.018616	0.018810
	-0.002074					
z534	-0.008839	0.056432	-0.009912	0.038008	-0.004238	0.014408
	-0.005952	-0.025944	-0.031038	-0.025391	-0.019590	-0.004108
	-0.020150	0.011629	-0.030934	-0.031172	-0.023773	-0.026510
	0.005838	0.025780	-0.037406	-0.000928	0.010646	0.026486
	-0.013428	0.016828	0.005366	-0.001985	0.018081	0.005158
	-0.016326					
x535	-0.009551	-0.019118	-0.032093	-0.002246	-0.001070	0.023077
	-0.011585	-0.023305	-0.011315	-0.025624	-0.026302	0.037233
	0.012796	0.019302	0.018002	-0.030288	0.017510	0.009868
	0.016910	0.058074	-0.004860	-0.020684	-0.016637	-0.001410
	-0.001372	0.026723	0.010886	0.024607	-0.019906	0.016617
	-0.002641					
y535	-0.004842	-0.010568	0.031097	0.002181	-0.004321	-0.037029
	0.053103	0.017333	-0.003133	0.012905	0.019416	-0.000410
	-0.027770	0.044557	-0.015537	-0.012608	-0.001107	0.001588
	0.054614	-0.034446	0.033413	-0.029566	0.028307	0.024340
	0.019418	-0.017106	0.047064	0.000900	-0.020714	0.017122
	0.011074					
z535	-0.008781	0.058520	-0.009912	0.039332	-0.001075	0.014835
	-0.008284	-0.029839	-0.030164	-0.022784	-0.019576	-0.009231
	-0.017963	0.007105	-0.034802	-0.027536	-0.025629	-0.028751
	0.008004	0.023015	-0.040054	0.001541	0.010501	0.033412
	-0.012103	0.012658	-0.001917	-0.000726	0.014675	0.005401
	-0.018076					
x536	-0.007720	-0.015481	-0.033566	0.000851	0.001307	0.021281
	-0.013821	-0.024568	-0.011465	-0.029357	-0.028669	0.041429
	0.013274	0.018320	0.017050	-0.028352	0.021571	0.005679
	0.020440	0.054312	-0.001786	-0.020639	-0.018615	0.000592
	-0.007875	0.021031	0.010746	0.018132	-0.022049	0.021050
	-0.000875					

y536	-0.009971	-0.010287	0.032341	-0.000196	-0.007993	-0.036371
	0.055804	0.018176	0.001975	0.009803	0.016715	0.004168
	-0.031734	0.052660	-0.021156	-0.010041	-0.005527	0.006786
	0.052201	-0.026232	0.039369	-0.033952	0.028930	0.022940
	0.012370	-0.010115	0.055984	0.007497	-0.014393	0.011275
	0.004836					
z536	-0.008514	0.060718	-0.010962	0.042094	0.001014	0.015753
	-0.010378	-0.034226	-0.032173	-0.017551	-0.020285	-0.013139
	-0.013194	-0.001562	-0.038370	-0.019568	-0.026832	-0.031336
	0.000447	0.021431	-0.043814	0.008637	0.007482	0.032608
	-0.010441	0.017195	-0.017936	-0.002298	0.006891	-0.003218
	-0.012263					
x537	-0.006248	-0.010987	-0.035363	0.003876	0.003898	0.020251
	-0.015399	-0.024371	-0.010330	-0.032445	-0.032107	0.043929
	0.014891	0.016665	0.017154	-0.024166	0.026084	0.003091
	0.022785	0.048121	0.001785	-0.019843	-0.022127	0.005022
	-0.009103	0.016948	0.010363	0.014379	-0.022761	0.025791
	-0.003582					
y537	-0.012440	-0.010149	0.033477	-0.003505	-0.010854	-0.035390
	0.057077	0.017881	0.007075	0.007997	0.017877	0.001982
	-0.033371	0.055002	-0.022717	-0.013508	-0.010168	0.006423
	0.049131	-0.021554	0.035882	-0.035299	0.028330	0.024568
	0.009442	-0.005584	0.062422	0.007988	-0.010142	0.003158
	-0.003339					
z537	-0.008755	0.059928	-0.011403	0.045356	0.002377	0.015255
	-0.014099	-0.038821	-0.037157	-0.011407	-0.022943	-0.012769
	-0.010985	-0.007251	-0.040599	-0.009915	-0.025114	-0.029827
	-0.007223	0.022375	-0.044702	0.013198	0.008580	0.024788
	-0.015607	0.018964	-0.029699	-0.003014	0.001295	-0.009145
	-0.000881					
x538	-0.004630	-0.007702	-0.037217	0.007290	0.005797	0.017701
	-0.017521	-0.025435	-0.010608	-0.034506	-0.037595	0.048186
	0.016227	0.015472	0.018203	-0.021196	0.030484	0.000201
	0.019092	0.043155	0.004873	-0.018268	-0.022337	0.001518
	-0.012669	0.018294	0.013070	0.010341	-0.024092	0.023117
	-0.004746					
y538	-0.014358	-0.009060	0.033924	-0.005723	-0.013542	-0.033396
	0.058130	0.017973	0.010930	0.006962	0.019130	-0.000987
	-0.034115	0.054292	-0.025178	-0.015473	-0.015581	0.005913
	0.045810	-0.018284	0.029770	-0.032339	0.025285	0.026597
	0.004486	-0.003973	0.060225	0.008868	-0.008402	-0.003492
	-0.004955					

z538	-0.008660	0.059755	-0.010944	0.047192	0.004520	0.016633
	-0.016888	-0.041611	-0.040687	-0.005750	-0.018987	-0.015489
	-0.006823	-0.011160	-0.036914	0.002962	-0.018469	-0.024338
	-0.004008	0.016496	-0.043007	0.012875	0.007293	0.028942
	-0.007195	0.011296	-0.039435	-0.004550	0.004611	-0.001501
	0.002231					
x539	-0.002867	-0.004274	-0.037914	0.010445	0.007206	0.016066
	-0.018318	-0.025919	-0.010706	-0.036366	-0.040666	0.050203
	0.019386	0.016114	0.020725	-0.017279	0.034718	0.001269
	0.015103	0.035111	0.007891	-0.017978	-0.022510	-0.001331
	-0.010893	0.018279	0.014721	0.008294	-0.024185	0.024747
	-0.006048					
y539	-0.015896	-0.007451	0.033104	-0.006969	-0.016171	-0.031462
	0.057946	0.017698	0.012914	0.007276	0.019755	-0.003567
	-0.033962	0.051460	-0.026645	-0.016543	-0.018222	0.004270
	0.042637	-0.014031	0.023632	-0.027524	0.020462	0.028181
	-0.000020	-0.003051	0.054230	0.007911	-0.007057	-0.010807
	-0.002565					
z539	-0.008160	0.057557	-0.010736	0.048971	0.006583	0.016189
	-0.021343	-0.043983	-0.044018	0.000275	-0.017892	-0.015812
	-0.005990	-0.017381	-0.032982	0.013517	-0.012689	-0.023330
	-0.001176	0.014615	-0.041275	0.014858	0.010020	0.028024
	-0.006705	0.005186	-0.040613	-0.012174	0.007061	-0.001204
	0.002506					
x540	-0.001506	-0.001550	-0.037349	0.012461	0.008911	0.014027
	-0.019934	-0.026425	-0.009557	-0.036803	-0.042121	0.050124
	0.021673	0.016151	0.023133	-0.016217	0.039984	0.004131
	0.012099	0.026769	0.012552	-0.019967	-0.022588	-0.001276
	-0.008680	0.014197	0.016657	0.003985	-0.024133	0.028069
	-0.007435					
y540	-0.016512	-0.004727	0.030973	-0.006697	-0.019378	-0.028956
	0.057936	0.017556	0.012318	0.007885	0.019308	-0.004686
	-0.032363	0.048294	-0.026841	-0.014936	-0.020487	0.001328
	0.039516	-0.011428	0.015882	-0.021687	0.016357	0.027838
	-0.003576	-0.001733	0.045228	0.008223	-0.006723	-0.018476
	0.001339					
z540	-0.006606	0.055274	-0.011165	0.051270	0.007376	0.016075
	-0.024288	-0.045991	-0.047960	0.003494	-0.017137	-0.014032
	-0.004105	-0.022734	-0.028358	0.027388	-0.011389	-0.023305
	-0.002230	0.012493	-0.042092	0.019344	0.015141	0.021654
	-0.006507	0.007433	-0.035378	-0.018062	0.007902	-0.004274
	0.003231					

x541	-0.000416	0.000752	-0.036391	0.013621	0.009912	0.011817
	-0.020997	-0.026696	-0.007073	-0.037570	-0.043705	0.047208
	0.022466	0.015473	0.026758	-0.015140	0.040685	0.005449
	0.008652	0.020296	0.012525	-0.023131	-0.020462	0.002042
	-0.004923	0.008744	0.020823	-0.002370	-0.028353	0.025894
	-0.010022					
y541	-0.016323	-0.001561	0.028196	-0.005423	-0.022484	-0.025500
	0.056585	0.016212	0.009287	0.009650	0.019606	-0.002772
	-0.028473	0.043949	-0.027556	-0.012348	-0.018218	-0.000105
	0.037431	-0.011551	0.011863	-0.014378	0.012161	0.024726
	-0.007640	-0.000145	0.034542	0.009821	-0.003394	-0.021102
	0.009196					
z541	-0.004687	0.053675	-0.010078	0.052715	0.008680	0.016083
	-0.027037	-0.047393	-0.050572	0.006048	-0.014637	-0.012458
	-0.001652	-0.028366	-0.022951	0.038371	-0.007321	-0.016851
	-0.000891	0.007423	-0.038744	0.022543	0.016322	0.017451
	-0.005226	0.008032	-0.030984	-0.020431	0.011530	0.001166
	0.002137					
x542	0.000385	0.003123	-0.034557	0.013884	0.009265	0.009130
	-0.020700	-0.027278	-0.003802	-0.037672	-0.044472	0.042838
	0.022417	0.012874	0.030003	-0.012182	0.037918	0.007104
	0.006369	0.013405	0.012593	-0.029178	-0.017383	0.011407
	-0.001053	-0.001091	0.026235	-0.005922	-0.031780	0.023113
	-0.010933					
y542	-0.015658	0.001642	0.024540	-0.003742	-0.024289	-0.021258
	0.053719	0.014330	0.005239	0.010893	0.019502	-0.000554
	-0.023728	0.040532	-0.028056	-0.011949	-0.013140	-0.002020
	0.034822	-0.010950	0.007389	-0.005329	0.008396	0.017988
	-0.010536	0.002820	0.024122	0.008048	-0.002118	-0.023198
	0.014646					
z542	-0.003105	0.050747	-0.008123	0.052614	0.011518	0.015255
	-0.030080	-0.047821	-0.050540	0.006335	-0.012133	-0.012667
	-0.002036	-0.031478	-0.018376	0.040925	-0.004054	-0.011535
	0.002570	0.004819	-0.035183	0.023955	0.018071	0.014573
	-0.001257	0.009264	-0.023089	-0.023127	0.008415	-0.000213
	-0.000858					
x543	0.000041	0.004378	-0.031403	0.013597	0.009841	0.007332
	-0.020577	-0.027999	-0.000374	-0.037464	-0.043311	0.036863
	0.021840	0.010842	0.030079	-0.013214	0.037505	0.012640
	0.006380	0.004155	0.017017	-0.037555	-0.011642	0.016871
	0.004960	-0.008100	0.031367	-0.004754	-0.031860	0.017249
	-0.012305					

y543	-0.013734	0.005858	0.019594	-0.002200	-0.027217	-0.017148
	0.051305	0.011268	0.000558	0.011903	0.018332	0.002365
	-0.018685	0.036057	-0.026875	-0.008121	-0.012355	-0.006462
	0.031404	-0.009884	-0.002484	0.004149	0.004992	0.015097
	-0.014852	0.003153	0.016195	0.004265	-0.003839	-0.022048
	0.020541					
z543	-0.001751	0.047515	-0.006543	0.051856	0.014114	0.014718
	-0.032439	-0.048712	-0.049428	0.003501	-0.009064	-0.011482
	-0.002245	-0.035694	-0.013195	0.044060	-0.007686	-0.010223
	0.004805	0.003141	-0.039056	0.025216	0.020103	0.017398
	0.001955	0.006340	-0.013834	-0.024174	0.003899	-0.004877
	-0.003762					
x544	-0.001966	0.003573	-0.028222	0.014512	0.009365	0.005776
	-0.018735	-0.027371	0.003114	-0.035892	-0.041086	0.028282
	0.025634	0.011605	0.025465	-0.013858	0.037600	0.015060
	0.007173	-0.000849	0.017804	-0.043282	-0.007737	0.011844
	0.003310	-0.010645	0.030961	-0.002546	-0.030383	0.008516
	-0.004713					
y544	-0.010660	0.011706	0.015093	-0.001928	-0.029262	-0.012543
	0.047280	0.006132	-0.003795	0.012087	0.017064	0.004472
	-0.017356	0.029351	-0.022926	-0.004539	-0.013911	-0.008016
	0.028546	-0.013830	-0.009855	0.011069	0.004093	0.021659
	-0.012557	0.000273	0.010897	0.003054	-0.005713	-0.017316
	0.020794					
z544	-0.001549	0.044731	-0.003888	0.049966	0.018619	0.013590
	-0.035644	-0.048746	-0.043845	-0.000347	-0.004899	-0.015557
	-0.006692	-0.039029	-0.005447	0.042958	-0.013827	-0.006901
	0.008087	-0.004081	-0.039622	0.025140	0.021517	0.027534
	0.003850	0.004684	-0.014103	-0.020078	-0.000242	0.000310
	-0.005921					
x545	-0.005476	0.003035	-0.024096	0.012660	0.008281	0.006053
	-0.015265	-0.024632	0.006794	-0.034482	-0.038119	0.022666
	0.029679	0.010079	0.024234	-0.008884	0.036371	0.017464
	0.008211	-0.006355	0.015513	-0.045555	-0.003954	0.016110
	0.000940	-0.016844	0.024448	0.013369	-0.021205	0.013942
	0.003139					
y545	-0.007407	0.016824	0.009527	0.000367	-0.031208	-0.010438
	0.042655	-0.000558	-0.009643	0.013197	0.014770	0.004364
	-0.013063	0.025115	-0.024081	-0.008770	-0.011590	-0.008330
	0.019145	-0.014788	-0.015913	0.017380	-0.002533	0.016638
	-0.015143	0.003944	0.011538	-0.012231	-0.016440	-0.030129
	0.019531					

z545	-0.004178	0.040484	-0.001656	0.049354	0.023780	0.009900
	-0.036830	-0.046789	-0.041677	-0.001144	-0.004980	-0.017590
	-0.005794	-0.039742	-0.001602	0.038281	-0.011940	0.000340
	0.004380	-0.005834	-0.042220	0.026526	0.015524	0.029485
	-0.004000	0.005239	-0.017995	-0.023063	-0.011009	-0.010259
	-0.004654					
x546	-0.009840	0.003621	-0.019496	0.009045	0.008075	0.004058
	-0.012230	-0.023639	0.009244	-0.030710	-0.036116	0.019321
	0.035535	0.003755	0.020377	-0.009771	0.038709	0.023156
	0.008279	-0.015420	0.013514	-0.053591	0.003728	0.027961
	0.003035	-0.023096	0.017178	0.025650	-0.020529	0.010317
	0.012339					
y546	-0.003509	0.021101	0.004392	0.003959	-0.034328	-0.007124
	0.040578	-0.004920	-0.013900	0.012232	0.013152	0.002317
	-0.010687	0.024641	-0.023266	-0.009026	-0.010961	-0.009535
	0.015048	-0.014217	-0.019186	0.026516	-0.013250	0.007349
	-0.017043	0.001373	0.012005	-0.012274	-0.013731	-0.025542
	0.015165					
z546	-0.006334	0.037349	0.001478	0.050705	0.027336	0.006155
	-0.034913	-0.041953	-0.038245	-0.001718	-0.002541	-0.018899
	-0.002512	-0.036245	0.003375	0.036108	-0.006999	0.008251
	0.004967	-0.004997	-0.038517	0.025900	0.001593	0.027247
	-0.007206	-0.001526	-0.021017	-0.012562	-0.005721	-0.004332
	-0.008345					
x547	-0.013698	0.003249	-0.014802	0.007785	0.007104	0.001525
	-0.010047	-0.023085	0.011869	-0.027050	-0.031128	0.012937
	0.046182	0.006725	0.014451	-0.011660	0.046060	0.026170
	0.005393	-0.018262	0.013065	-0.057553	0.004068	0.019738
	-0.000951	-0.023409	0.009989	0.025566	-0.013920	0.008050
	0.015237					
y547	0.000979	0.024896	0.000085	0.005814	-0.038143	-0.003519
	0.038215	-0.010306	-0.019540	0.011899	0.010542	0.005990
	-0.012915	0.016822	-0.025181	-0.009111	-0.014120	-0.015584
	0.012172	-0.022203	-0.023799	0.032756	-0.011720	0.010889
	-0.016449	-0.002679	0.009467	-0.003506	-0.020391	-0.026445
	0.022536					
z547	-0.007738	0.032967	0.005469	0.051039	0.030297	0.001881
	-0.032520	-0.037845	-0.036790	-0.001553	-0.001440	-0.014084
	-0.002486	-0.036906	0.002500	0.033875	-0.003878	0.006275
	0.006856	-0.011692	-0.034453	0.022233	0.001625	0.031191
	-0.009380	-0.008055	-0.024101	0.001475	-0.010193	-0.009331
	-0.002136					

x548	-0.017271	0.002786	-0.009697	0.006644	0.004215	-0.001522
	-0.007867	-0.022709	0.014158	-0.023565	-0.027704	0.007502
	0.054518	0.007919	0.010422	-0.013114	0.051338	0.027412
	0.006525	-0.021524	0.014065	-0.059561	0.004813	0.016180
	-0.000807	-0.024923	0.002732	0.031800	-0.008159	0.008054
	0.015696					
y548	0.005757	0.027853	-0.004572	0.007376	-0.039183	-0.000182
	0.035088	-0.016146	-0.022944	0.011573	0.009153	0.007245
	-0.013099	0.011990	-0.025837	-0.009965	-0.014397	-0.020329
	0.007204	-0.025793	-0.029593	0.038364	-0.013991	0.008962
	-0.016915	-0.008023	0.008819	-0.004983	-0.024508	-0.027854
	0.028800					
z548	-0.008949	0.027860	0.009279	0.051098	0.035092	-0.003130
	-0.030819	-0.034184	-0.033426	-0.001066	-0.000406	-0.011062
	0.000341	-0.036114	0.002452	0.031606	0.000879	0.006004
	0.007095	-0.015624	-0.031881	0.017134	-0.001381	0.031464
	-0.012582	-0.014925	-0.024776	0.006393	-0.013307	-0.014347
	0.002301					
x549	-0.020612	0.001658	-0.004383	0.004145	0.000549	-0.005645
	-0.007017	-0.024118	0.015886	-0.018869	-0.025834	0.004454
	0.061221	0.006526	0.007124	-0.015024	0.053272	0.024166
	0.005325	-0.026476	0.012499	-0.059571	0.006850	0.013439
	-0.003747	-0.028834	-0.003765	0.034760	-0.008219	0.003129
	0.021926					
y549	0.010928	0.029933	-0.009716	0.010731	-0.039853	0.003132
	0.032237	-0.018861	-0.023371	0.009315	0.008996	0.002406
	-0.010019	0.012160	-0.027105	-0.010590	-0.009572	-0.028566
	0.009981	-0.024945	-0.028930	0.041098	-0.017904	0.003911
	-0.008668	-0.011406	0.012779	-0.005816	-0.022126	-0.027616
	0.021354					
z549	-0.010036	0.021845	0.012809	0.051804	0.039974	-0.008562
	-0.028932	-0.028500	-0.028016	-0.001528	-0.000039	-0.011949
	0.005673	-0.032884	0.001736	0.029319	0.007353	0.002217
	0.011690	-0.016496	-0.024452	0.009665	-0.005181	0.028862
	-0.010283	-0.018851	-0.019937	0.008900	-0.014389	-0.020941
	-0.000670					
x550	-0.023242	0.000250	0.000852	0.001348	-0.003281	-0.010085
	-0.008200	-0.026184	0.018266	-0.016071	-0.023943	0.000598
	0.069224	0.006030	0.003532	-0.018414	0.053887	0.018895
	0.003829	-0.030128	0.007988	-0.058464	0.004526	0.007889
	-0.010463	-0.029136	-0.007503	0.030540	-0.008755	-0.004021
	0.025428					

y550	0.015854	0.030394	-0.013888	0.014049	-0.040222	0.006579
	0.031065	-0.021118	-0.024939	0.009988	0.008742	-0.001354
	-0.008199	0.012312	-0.026684	-0.007402	-0.004573	-0.039589
	0.016016	-0.024925	-0.023443	0.043226	-0.016320	-0.000358
	0.005699	-0.016173	0.014698	-0.002146	-0.017784	-0.025278
	0.015424					
z550	-0.010931	0.014957	0.016752	0.052040	0.044675	-0.013985
	-0.026039	-0.022727	-0.023454	-0.000532	-0.000430	-0.012318
	0.010729	-0.029599	0.001701	0.029707	0.012373	-0.002934
	0.017399	-0.017292	-0.015394	0.002570	-0.006920	0.026032
	-0.005539	-0.020252	-0.015376	0.010952	-0.014817	-0.026411
	-0.001333					
x551	-0.025190	-0.000588	0.006112	-0.000774	-0.008385	-0.015141
	-0.007186	-0.027393	0.020979	-0.013129	-0.023013	-0.006023
	0.075486	0.005206	0.004232	-0.018037	0.053516	0.012455
	0.007279	-0.033364	0.003028	-0.057636	-0.000876	0.007208
	-0.006919	-0.032983	-0.014339	0.029247	-0.005475	-0.003034
	0.025951					
y551	0.019740	0.029963	-0.017774	0.016483	-0.038468	0.007753
	0.024818	-0.025295	-0.026699	0.007800	0.002806	-0.001045
	-0.008393	0.009057	-0.027240	-0.008201	-0.006264	-0.040328
	0.011266	-0.022064	-0.018924	0.048435	-0.014348	-0.007171
	0.001376	-0.013996	0.017392	-0.005262	-0.018720	-0.026439
	0.017168					
z551	-0.012250	0.007456	0.020696	0.051330	0.050009	-0.020337
	-0.024762	-0.017043	-0.018784	-0.000804	-0.003816	-0.011386
	0.015710	-0.027416	0.002085	0.028761	0.014202	-0.004599
	0.019435	-0.015846	-0.006917	-0.003115	-0.009818	0.022373
	-0.007273	-0.017860	-0.009577	0.007460	-0.015948	-0.031538
	-0.000569					
x552	-0.025934	-0.003109	0.010986	-0.003255	-0.013377	-0.019673
	-0.008896	-0.030396	0.023204	-0.009958	-0.024936	-0.009989
	0.078991	0.003720	0.001852	-0.017050	0.050549	0.003497
	0.011087	-0.036989	0.001625	-0.056801	-0.001655	0.002120
	-0.006159	-0.031362	-0.011977	0.019221	-0.003481	-0.010509
	0.025076					
y552	0.024002	0.031404	-0.020724	0.018909	-0.036600	0.008344
	0.022244	-0.030391	-0.027689	0.004070	-0.001828	-0.004810
	-0.009371	0.006075	-0.020030	-0.008528	-0.009618	-0.027039
	0.005655	-0.018627	-0.020287	0.054530	-0.022396	-0.008827
	-0.000472	-0.012984	0.009979	-0.001214	-0.016392	-0.005762
	0.019419					

z552	-0.012879	0.001021	0.024809	0.049725	0.054795	-0.026346
	-0.022835	-0.012882	-0.014057	-0.001643	-0.008596	-0.011088
	0.019059	-0.026293	0.005259	0.028541	0.012468	0.002451
	0.018183	-0.014779	-0.002036	-0.007431	-0.015769	0.020923
	-0.010555	-0.011278	-0.006867	0.002358	-0.017607	-0.028391
	0.002786					
x553	-0.026426	-0.005154	0.015882	-0.005186	-0.021432	-0.024001
	-0.009454	-0.029864	0.024238	-0.008228	-0.028720	-0.012047
	0.079877	0.003625	0.003438	-0.021139	0.045177	-0.006706
	0.015325	-0.038304	-0.000341	-0.053609	-0.011844	-0.008837
	-0.001081	-0.031224	-0.016433	0.021512	0.007369	-0.010527
	0.024801					
y553	0.028842	0.029614	-0.022348	0.020801	-0.034103	0.007826
	0.019796	-0.034933	-0.029150	0.001418	-0.001247	-0.006229
	-0.003761	0.002662	-0.020096	-0.006359	-0.011949	-0.033762
	0.001534	-0.019615	-0.022617	0.052555	-0.017982	-0.009753
	-0.002181	-0.017047	-0.002890	-0.009948	-0.018152	-0.003528
	0.020057					
z553	-0.013126	-0.007011	0.029212	0.046944	0.056493	-0.031901
	-0.018950	-0.006203	-0.009992	-0.002636	-0.012514	-0.008277
	0.025573	-0.023536	0.007864	0.024988	0.011339	-0.004002
	0.018291	-0.013358	0.002691	-0.014133	-0.020357	0.014282
	-0.011273	-0.009771	-0.011901	-0.001167	-0.015038	-0.029118
	0.004220					
x554	-0.027234	-0.005442	0.020597	-0.008308	-0.030263	-0.027708
	-0.009165	-0.028913	0.026464	-0.003986	-0.031935	-0.015608
	0.079625	0.001144	0.007551	-0.026038	0.038112	-0.017750
	0.015953	-0.036951	-0.005651	-0.045680	-0.013806	-0.012099
	-0.000005	-0.027892	-0.016039	0.014960	0.017136	-0.010715
	0.025157					
y554	0.033358	0.026877	-0.024907	0.023541	-0.027331	0.008403
	0.017216	-0.033828	-0.030730	-0.000058	-0.001376	-0.005162
	-0.002045	0.004327	-0.019558	-0.005283	-0.008283	-0.041917
	0.003446	-0.018851	-0.017948	0.046548	-0.017262	-0.011097
	-0.001844	-0.019700	-0.003517	-0.012396	-0.015564	-0.006089
	0.016816					
z554	-0.014477	-0.015008	0.034268	0.040370	0.058860	-0.035214
	-0.014068	0.002515	-0.004332	-0.000491	-0.015294	-0.006427
	0.032233	-0.020081	0.010662	0.016780	0.011110	-0.009636
	0.018864	-0.010905	0.005841	-0.016079	-0.019908	0.014045
	-0.014614	-0.002939	-0.010032	-0.008527	-0.008651	-0.030338
	0.007995					

x555	-0.026838	-0.005146	0.023615	-0.009207	-0.036490	-0.029002
	-0.010733	-0.029948	0.028253	-0.001673	-0.032753	-0.017241
	0.077550	-0.002060	0.010566	-0.023054	0.031221	-0.019110
	0.017721	-0.035615	-0.010053	-0.035326	-0.012674	-0.011192
	0.002405	-0.018957	-0.010378	0.003557	0.020314	-0.005802
	0.027573					
y555	0.037933	0.026416	-0.025769	0.026684	-0.021045	0.006540
	0.019326	-0.032756	-0.030991	-0.002195	-0.001582	-0.005796
	-0.001178	0.005294	-0.016313	-0.005720	-0.009084	-0.052169
	0.003652	-0.022710	-0.014132	0.040825	-0.015047	-0.016434
	-0.003382	-0.018827	-0.006929	-0.011806	-0.007414	-0.003996
	0.014512					
z555	-0.015705	-0.021648	0.039285	0.032186	0.060269	-0.035244
	-0.009688	0.008901	0.001631	0.001310	-0.014210	-0.005241
	0.037525	-0.017607	0.011351	0.013272	0.011242	-0.009208
	0.018626	-0.012054	0.004309	-0.014937	-0.016875	0.018171
	-0.016743	0.009423	-0.006684	-0.016507	-0.004705	-0.026237
	0.014135					
x556	-0.025055	-0.003605	0.024612	-0.005734	-0.041811	-0.031175
	-0.011110	-0.028752	0.029394	-0.000624	-0.034420	-0.018019
	0.072947	-0.004210	0.014701	-0.018040	0.025480	-0.018995
	0.020161	-0.032042	-0.011925	-0.024712	-0.013826	-0.013524
	0.005186	-0.011760	-0.004420	-0.005017	0.022886	0.000573
	0.027850					
y556	0.040857	0.021739	-0.027486	0.029353	-0.013829	0.003986
	0.017938	-0.031942	-0.030693	-0.006492	-0.001653	-0.010692
	0.001493	0.007466	-0.015548	-0.003749	-0.008401	-0.054690
	-0.001779	-0.015556	-0.012656	0.040705	-0.011437	-0.024531
	0.000293	-0.015757	-0.012088	-0.019922	-0.007958	-0.008405
	0.018511					
z556	-0.016797	-0.027661	0.043955	0.023791	0.059865	-0.033943
	-0.004605	0.015399	0.007478	0.002602	-0.013331	-0.005660
	0.040577	-0.013659	0.011827	0.009652	0.012830	-0.006497
	0.017663	-0.013434	0.003795	-0.011374	-0.016000	0.020602
	-0.016836	0.020247	-0.001371	-0.020593	-0.001785	-0.020278
	0.018140					
x557	-0.022635	-0.000891	0.023743	0.000273	-0.045664	-0.034190
	-0.011270	-0.026349	0.030597	-0.001505	-0.036590	-0.017915
	0.066902	-0.007520	0.020248	-0.011130	0.020497	-0.019417
	0.024209	-0.026871	-0.014556	-0.016962	-0.014742	-0.011433
	0.009335	-0.009411	-0.001687	-0.011638	0.023146	0.005830
	0.026177					

y557	0.043844	0.017759	-0.027589	0.026276	-0.003761	0.003387
	0.015020	-0.029851	-0.028254	-0.010788	0.001797	-0.016270
	-0.000109	0.005885	-0.013751	-0.002797	-0.004276	-0.050256
	-0.000974	-0.012972	-0.008080	0.027578	-0.008140	-0.009583
	0.008306	-0.020521	-0.013927	-0.018715	-0.002587	-0.003263
	0.016411					
z557	-0.017925	-0.032931	0.048518	0.014586	0.057942	-0.031563
	0.000014	0.020656	0.013348	0.002658	-0.012266	-0.006967
	0.042674	-0.010181	0.012535	0.005779	0.014645	-0.003783
	0.017742	-0.015586	0.002492	-0.008645	-0.014888	0.024892
	-0.016411	0.027644	0.001895	-0.022584	0.001253	-0.013212
	0.021341					
x558	-0.019240	0.002427	0.021501	0.008801	-0.048415	-0.037593
	-0.009967	-0.024947	0.031254	-0.003768	-0.039414	-0.015834
	0.060700	-0.011067	0.026258	-0.004480	0.014659	-0.019443
	0.029198	-0.019371	-0.015715	-0.009338	-0.015872	-0.011515
	0.012382	-0.008044	0.002241	-0.015495	0.022715	0.009779
	0.021722					
y558	0.043709	0.012556	-0.026404	0.020622	0.006176	0.003512
	0.014330	-0.032934	-0.026968	-0.014777	-0.001903	-0.018397
	0.001823	0.002468	-0.010328	-0.004916	-0.008197	-0.037926
	-0.006440	-0.008856	-0.009121	0.018306	-0.003141	0.001130
	0.005843	-0.021445	-0.013036	-0.017862	0.001293	0.000322
	0.018297					
z558	-0.018441	-0.037580	0.053068	0.005284	0.054076	-0.028455
	0.005301	0.024544	0.018533	0.001108	-0.010543	-0.008304
	0.042872	-0.006473	0.012950	0.000648	0.016105	-0.000791
	0.017813	-0.017748	0.002613	-0.005424	-0.015617	0.028194
	-0.014612	0.032864	0.006628	-0.020917	0.002743	-0.008012
	0.021938					
x559	-0.014520	0.006333	0.017343	0.020816	-0.050574	-0.041749
	-0.007867	-0.022556	0.030578	-0.007507	-0.042050	-0.012609
	0.053642	-0.013521	0.029452	0.002676	0.010601	-0.020754
	0.032310	-0.009640	-0.015425	0.000236	-0.016928	-0.017283
	0.012731	-0.007670	0.005638	-0.017097	0.021202	0.012746
	0.016950					
y559	0.043638	0.006206	-0.024356	0.015897	0.013585	0.003498
	0.013772	-0.032837	-0.026386	-0.017066	-0.003191	-0.021362
	0.007627	0.003392	-0.012951	-0.012334	-0.006577	-0.034634
	-0.013585	-0.004868	-0.007722	0.011122	0.001214	0.000209
	-0.003799	-0.023254	-0.006830	-0.018087	0.002719	0.003229
	0.020635					

z559	-0.018308	-0.041704	0.057114	-0.003282	0.048233	-0.024663
	0.011253	0.027143	0.021900	-0.002067	-0.009735	-0.009558
	0.039763	-0.002472	0.012793	-0.003664	0.016871	0.002407
	0.017159	-0.018721	0.003536	-0.000210	-0.015701	0.028429
	-0.013921	0.036041	0.010639	-0.015405	0.002926	-0.003928
	0.022853					
x560	-0.008924	0.010827	0.012474	0.034245	-0.051874	-0.046511
	-0.004903	-0.019456	0.030240	-0.009981	-0.042458	-0.008494
	0.047337	-0.015410	0.032012	0.011486	0.007281	-0.024833
	0.035348	-0.000666	-0.017051	0.007923	-0.016862	-0.022429
	0.015372	-0.011135	0.005020	-0.016345	0.017458	0.015410
	0.010300					
y560	0.044669	-0.002385	-0.019788	0.007654	0.021860	0.007684
	0.010945	-0.031001	-0.023643	-0.014759	0.002958	-0.022351
	0.013075	0.010794	-0.016762	-0.019179	-0.005563	-0.033353
	-0.013365	-0.009386	-0.001707	0.000462	0.009735	-0.005001
	-0.006908	-0.018237	-0.001657	-0.012813	0.005168	0.003786
	0.014102					
z560	-0.017561	-0.044276	0.060540	-0.009684	0.040617	-0.020825
	0.017351	0.027857	0.023917	-0.007748	-0.010696	-0.009638
	0.034311	0.001381	0.011172	-0.006942	0.015725	0.006375
	0.013234	-0.018916	0.002235	0.006424	-0.014916	0.025594
	-0.015713	0.036152	0.013044	-0.007170	0.001068	-0.001710
	0.025874					
x561	-0.002816	0.015453	0.007047	0.047122	-0.051440	-0.051034
	-0.002287	-0.017701	0.028479	-0.013592	-0.042758	-0.003388
	0.040909	-0.018559	0.032053	0.019786	0.003691	-0.024512
	0.035894	0.009017	-0.019294	0.016228	-0.016825	-0.027080
	0.016293	-0.013911	0.005550	-0.012922	0.015199	0.018877
	0.002595					
y561	0.043129	-0.010509	-0.014364	-0.002761	0.027946	0.010548
	0.010390	-0.032130	-0.021982	-0.012736	0.005232	-0.023115
	0.017647	0.013978	-0.023700	-0.023242	-0.006727	-0.028996
	-0.020511	-0.008567	-0.000063	-0.006409	0.014385	-0.005076
	-0.013730	-0.012586	0.000778	-0.009671	-0.001748	0.001210
	0.013080					
z561	-0.015383	-0.045771	0.062984	-0.013350	0.031483	-0.016096
	0.023725	0.028585	0.024654	-0.014519	-0.012201	-0.009733
	0.027216	0.008900	0.009087	-0.010783	0.014244	0.006471
	0.011283	-0.018110	0.003607	0.014554	-0.012285	0.016111
	-0.019141	0.032476	0.016133	0.004363	-0.000880	-0.001639
	0.026050					

x562	0.003613	0.018383	0.002890	0.057191	-0.048998	-0.054447
	-0.000451	-0.016947	0.025883	-0.017380	-0.041705	-0.000046
	0.035070	-0.021799	0.030794	0.024200	0.000277	-0.020630
	0.032748	0.017777	-0.018859	0.022689	-0.016041	-0.030170
	0.015194	-0.018493	0.007122	-0.009298	0.018075	0.024664
	-0.005312					
y562	0.040413	-0.018591	-0.007884	-0.013291	0.033121	0.013687
	0.010738	-0.035158	-0.020492	-0.011083	0.004114	-0.024930
	0.020257	0.015531	-0.030261	-0.032121	-0.006588	-0.021600
	-0.030133	-0.004980	0.003458	-0.011579	0.017731	-0.002368
	-0.025481	-0.008019	0.002168	-0.007476	-0.006412	-0.001641
	0.015968					
z562	-0.012275	-0.045258	0.063348	-0.013563	0.020966	-0.011326
	0.030723	0.029168	0.024296	-0.023031	-0.014078	-0.009553
	0.019035	0.018181	0.006294	-0.012292	0.014091	0.004243
	0.011534	-0.015412	0.006513	0.024957	-0.010071	0.001987
	-0.024239	0.025676	0.018735	0.017514	-0.006209	-0.001363
	0.023568					
x563	0.013923	0.010077	0.005382	0.054384	-0.041901	-0.045222
	0.006444	-0.017854	0.024038	-0.017438	-0.037300	-0.000552
	0.022845	-0.015084	0.019764	0.016226	-0.021473	-0.017783
	0.014714	0.022608	-0.012322	0.016095	-0.014447	-0.024467
	0.011914	-0.025994	0.004637	-0.013345	0.006093	0.012018
	-0.024201					
y563	0.037165	-0.028578	-0.000066	-0.026432	0.041324	0.017157
	0.009075	-0.035128	-0.012501	-0.007171	0.009561	-0.022679
	0.025129	0.019966	-0.031618	-0.034051	-0.001581	-0.008044
	-0.026780	0.003400	0.009293	-0.015553	0.008511	-0.006147
	-0.008598	-0.008668	0.015693	0.003001	0.010169	0.002878
	0.017467					
z563	-0.006224	-0.042818	0.057863	-0.008010	0.000915	-0.005020
	0.042949	0.027722	0.019767	-0.032724	-0.021689	-0.000432
	0.004870	0.024476	0.010949	-0.009325	0.007451	-0.001000
	0.008212	-0.010016	-0.007564	0.036287	-0.014720	0.001181
	-0.016701	0.003536	-0.002296	0.012569	0.005746	0.007846
	0.004741					
x564	0.023001	-0.000655	0.008391	0.045969	-0.031594	-0.035966
	0.009603	-0.019929	0.021854	-0.018992	-0.026550	-0.003528
	0.018347	-0.010026	0.008374	0.009860	-0.034963	-0.011061
	-0.003123	0.017211	-0.010218	0.009056	-0.013618	-0.016233
	0.007438	-0.031127	0.002331	-0.022135	0.006153	0.007338
	-0.036692					

y564	0.032803	-0.031146	0.001954	-0.031088	0.040440	0.018347
	0.008456	-0.034073	-0.003871	-0.007219	0.006228	-0.019401
	0.028018	0.021107	-0.030201	-0.031855	0.006651	-0.002875
	-0.023086	0.009821	0.010752	-0.016492	0.003733	-0.009028
	-0.000281	-0.007867	0.022842	0.002602	0.014566	-0.002453
	0.015086					
z564	-0.001155	-0.038452	0.049500	-0.000504	-0.016581	0.001682
	0.050522	0.026523	0.016529	-0.038833	-0.028329	0.008048
	-0.008055	0.030155	0.014518	-0.008324	-0.000795	-0.003597
	0.007844	-0.003874	-0.014885	0.040504	-0.014355	0.001608
	-0.009792	-0.014306	-0.021537	0.009030	0.009236	0.012765
	-0.012662					
x565	0.030688	-0.008403	0.010354	0.037596	-0.022530	-0.027550
	0.012250	-0.024367	0.018025	-0.020717	-0.018285	-0.005258
	0.012914	-0.008434	-0.000035	0.005751	-0.044982	-0.007038
	-0.016114	0.012605	-0.011523	0.005021	-0.013255	-0.009096
	0.001543	-0.034891	0.001200	-0.027951	0.010298	0.005657
	-0.040594					
y565	0.028372	-0.029985	0.002598	-0.031460	0.033612	0.018603
	0.009893	-0.031903	0.001081	-0.007675	-0.001859	-0.015027
	0.024134	0.020757	-0.026096	-0.030016	0.009482	-0.003887
	-0.018008	0.015316	0.009775	-0.014702	0.003526	-0.011408
	0.005473	-0.006005	0.021313	-0.000467	0.016795	-0.006942
	0.014953					
z565	0.003087	-0.035879	0.041615	0.006744	-0.028829	0.007307
	0.052820	0.026575	0.014249	-0.040877	-0.031214	0.014219
	-0.016901	0.034603	0.015236	-0.007966	-0.006347	-0.003206
	0.008102	0.001974	-0.017850	0.039241	-0.010317	0.003108
	-0.002903	-0.026480	-0.032380	0.008719	0.009839	0.014334
	-0.025539					
x566	0.037050	-0.014167	0.010988	0.029229	-0.013780	-0.019771
	0.014483	-0.028880	0.018663	-0.022264	-0.009554	-0.006804
	0.010375	-0.004662	-0.005604	0.001477	-0.046415	-0.002976
	-0.024817	0.009089	-0.008807	0.005010	-0.017410	-0.003047
	0.003056	-0.032004	0.002159	-0.033378	0.017232	0.015530
	-0.046166					
y566	0.024124	-0.028457	0.003666	-0.030562	0.025320	0.018660
	0.012107	-0.027838	0.006232	-0.007718	-0.008741	-0.011400
	0.018950	0.023672	-0.020917	-0.028924	0.012625	-0.005818
	-0.011235	0.018920	0.010863	-0.010810	0.003605	-0.013341
	0.014130	0.000322	0.015447	-0.005116	0.018474	-0.002258
	0.011045					

z566	0.006091	-0.034513	0.034572	0.013786	-0.036832	0.011481
	0.051019	0.026603	0.010799	-0.040076	-0.031942	0.018568
	-0.022975	0.034137	0.014682	-0.007340	-0.011091	-0.001814
	0.008760	0.006871	-0.021178	0.032510	-0.003421	0.005046
	-0.002306	-0.037809	-0.038953	0.007855	0.008196	0.007354
	-0.027115					
x567	0.041614	-0.017981	0.008298	0.022909	-0.006849	-0.014516
	0.017239	-0.032030	0.018519	-0.022623	-0.005842	-0.007368
	0.004426	-0.005381	-0.009405	0.001331	-0.046335	-0.001693
	-0.032909	0.006766	-0.006091	0.003125	-0.022381	0.003522
	0.008375	-0.034695	-0.000385	-0.027018	0.022842	0.017850
	-0.041004					
y567	0.019405	-0.026826	0.004237	-0.028206	0.015599	0.017049
	0.015080	-0.021850	0.008559	-0.006757	-0.016492	-0.008745
	0.011230	0.025838	-0.015164	-0.025750	0.014010	-0.009586
	-0.003829	0.021153	0.010864	-0.009171	0.005150	-0.014231
	0.021557	0.002368	0.003320	-0.005796	0.017280	0.000883
	0.012643					
z567	0.009255	-0.034410	0.029244	0.019994	-0.041038	0.015540
	0.045468	0.026446	0.008406	-0.037540	-0.029206	0.020578
	-0.026272	0.032873	0.013375	-0.007161	-0.012624	0.002544
	0.011943	0.008183	-0.021883	0.024536	0.005447	0.007394
	-0.001909	-0.042437	-0.037792	0.005790	0.008104	0.004880
	-0.026533					
x568	0.044749	-0.019078	0.005355	0.016200	-0.002517	-0.010040
	0.018832	-0.039299	0.018743	-0.020967	-0.006640	-0.006057
	-0.002097	-0.010993	-0.009125	-0.002066	-0.047254	-0.001757
	-0.039252	0.000575	-0.004319	0.000948	-0.024181	0.006648
	0.009121	-0.030769	-0.002504	-0.018037	0.024548	0.014953
	-0.025048					
y568	0.014512	-0.025010	0.005328	-0.025847	0.004744	0.014474
	0.017412	-0.016228	0.008528	-0.004604	-0.023646	-0.007038
	0.003352	0.028037	-0.007686	-0.023911	0.014177	-0.013346
	0.005841	0.019544	0.010529	-0.009283	0.009082	-0.016924
	0.024348	0.004826	-0.012248	-0.007010	0.014329	0.004839
	0.018842					
z568	0.011561	-0.035881	0.024188	0.026114	-0.040854	0.018553
	0.037412	0.026963	0.006053	-0.033727	-0.023262	0.020290
	-0.025547	0.029691	0.010259	-0.004855	-0.009158	0.009145
	0.016830	0.011405	-0.020187	0.015075	0.012779	0.011858
	0.000153	-0.046754	-0.029563	0.003854	0.008626	0.002682
	-0.025069					

x569	0.048216	-0.020006	0.000852	0.011442	0.002039	-0.006148
	0.016582	-0.038306	0.019783	-0.019005	-0.003007	-0.007401
	-0.009663	-0.009039	-0.008596	-0.001922	-0.041922	-0.007082
	-0.034658	-0.005951	0.003301	-0.002917	-0.022245	0.011891
	0.018977	-0.022770	-0.001732	-0.003355	0.029455	0.015850
	-0.021383					
y569	0.009536	-0.023924	0.006241	-0.023212	-0.005955	0.010643
	0.018148	-0.008660	0.005795	-0.001893	-0.027531	-0.007537
	-0.003526	0.031747	0.000417	-0.021775	0.015146	-0.016902
	0.018993	0.015216	0.010523	-0.011414	0.015399	-0.020756
	0.025752	0.006560	-0.029778	-0.007729	0.012059	0.010759
	0.026714					
z569	0.012575	-0.037110	0.019202	0.031978	-0.037795	0.020561
	0.026933	0.024480	0.003749	-0.028312	-0.017441	0.018379
	-0.021729	0.019215	0.006671	-0.000149	-0.003242	0.019823
	0.017941	0.014245	-0.021224	0.003194	0.019318	0.017361
	0.000704	-0.050926	-0.019435	0.003899	0.007840	-0.003171
	-0.008786					
x570	0.050373	-0.021192	-0.003299	0.006941	0.003148	-0.002499
	0.012778	-0.043027	0.021140	-0.016347	-0.006997	-0.009860
	-0.019720	-0.009787	-0.009274	-0.001855	-0.043326	-0.008565
	-0.030090	-0.006102	0.004871	-0.008148	-0.007568	0.017993
	0.016022	-0.008072	0.001383	0.001876	0.033846	0.003232
	-0.004065					
y570	0.003664	-0.023590	0.007057	-0.020196	-0.015734	0.004637
	0.016529	0.000863	0.000475	0.001764	-0.026850	-0.010249
	-0.006292	0.033176	0.009044	-0.019203	0.018167	-0.019548
	0.034040	0.009566	0.008065	-0.014744	0.021873	-0.025747
	0.024804	0.008549	-0.046449	-0.004931	0.009427	0.018674
	0.039142					
z570	0.012451	-0.037745	0.013505	0.037640	-0.029714	0.020937
	0.012890	0.023809	0.001481	-0.020402	-0.008256	0.015457
	-0.013825	0.006203	0.001843	0.004375	0.008019	0.028874
	0.019954	0.015342	-0.019688	-0.010955	0.023450	0.023922
	0.005960	-0.052236	-0.000574	0.009795	0.004379	-0.011980
	0.003656					
x571	0.050238	-0.022106	-0.009476	0.004390	0.005744	-0.000799
	0.005595	-0.039348	0.021695	-0.016733	-0.007748	-0.018773
	-0.025393	-0.003385	-0.007640	-0.001482	-0.038934	-0.003965
	-0.020617	-0.003031	0.005693	-0.013534	-0.004620	0.019481
	0.022480	0.010368	-0.005496	0.012750	0.035921	0.004169
	0.012514					

y571	-0.004234	-0.023627	0.008734	-0.015930	-0.023944	-0.003154
	0.011817	0.010926	-0.005641	0.007091	-0.022904	-0.011501
	-0.000915	0.024971	0.012560	-0.018135	0.025059	-0.022193
	0.047627	0.005691	-0.000965	-0.014530	0.026143	-0.025265
	0.015056	0.011531	-0.051954	0.004319	0.001748	0.028957
	0.053965					
z571	0.012167	-0.036918	0.006723	0.042457	-0.019147	0.021163
	-0.004014	0.019866	-0.000010	-0.009135	0.001371	0.011580
	-0.004769	-0.010642	-0.005739	0.010766	0.019965	0.036601
	0.021069	0.011665	-0.014917	-0.029437	0.029764	0.032658
	0.008138	-0.049385	0.025149	0.010075	-0.001479	-0.030125
	0.013691					
x572	0.047713	-0.020622	-0.008563	0.002859	0.009382	0.001196
	0.002020	-0.033372	0.019506	-0.008983	-0.010495	-0.018289
	-0.026730	-0.003327	0.001546	0.001541	-0.030162	0.001966
	-0.009182	-0.006715	0.004246	-0.014597	-0.006921	0.011232
	0.030926	0.016107	-0.009080	0.014842	0.035228	0.006803
	0.013823					
y572	-0.013091	-0.020953	0.013568	-0.011849	-0.025403	-0.004353
	0.007325	0.017501	-0.013635	0.011092	-0.021764	-0.004327
	0.000204	0.014170	0.007153	-0.007496	0.027080	-0.019482
	0.041752	0.001944	-0.002050	-0.024274	0.026102	-0.026825
	0.013894	-0.011599	-0.034866	-0.008749	-0.010416	0.014979
	0.036451					
z572	0.008918	-0.029962	0.003500	0.040318	-0.005690	0.018970
	-0.016224	0.016021	-0.004455	0.003135	0.004803	0.009007
	0.007855	-0.025937	-0.001081	0.008543	0.034860	0.033253
	0.018649	0.005574	-0.011791	-0.036538	0.040494	0.020493
	0.004077	-0.040547	0.023838	0.005732	-0.015381	-0.017118
	0.015646					
x573	0.042567	-0.020500	-0.006725	0.003204	0.013172	0.003096
	-0.003317	-0.026046	0.015440	-0.001763	-0.015562	-0.014315
	-0.027919	-0.010072	0.000401	0.003005	-0.021474	0.009295
	-0.000661	-0.010225	0.001814	-0.014165	-0.004206	0.009859
	0.028938	0.012293	-0.005080	0.012985	0.029316	0.001428
	0.016383					
y573	-0.021521	-0.016996	0.017342	-0.008258	-0.025923	-0.005297
	0.002376	0.023342	-0.018944	0.016135	-0.016495	-0.001049
	0.005327	0.003726	0.003328	0.000960	0.029101	-0.016946
	0.035953	-0.000056	-0.003411	-0.032809	0.020172	-0.028829
	0.015434	-0.024666	-0.019010	-0.013952	-0.021697	0.013996
	0.020654					

z573	0.005891	-0.022250	0.000095	0.037104	0.005883	0.016860
	-0.026376	0.011011	-0.006152	0.011442	0.008720	0.004166
	0.019192	-0.035385	0.002648	0.005222	0.044398	0.027387
	0.015509	-0.000016	-0.008941	-0.043451	0.046758	0.007818
	0.001361	-0.028468	0.019900	0.000919	-0.027123	-0.005695
	0.014252					
x574	0.036342	-0.019187	-0.004372	0.004840	0.016753	0.005010
	-0.007276	-0.017600	0.011157	0.007561	-0.016589	-0.011966
	-0.023974	-0.014651	-0.003620	0.004906	-0.014146	0.015836
	0.006382	-0.008673	0.003215	-0.014831	-0.006873	0.005080
	0.027222	0.009327	-0.000581	0.012758	0.021252	-0.000125
	0.017805					
y574	-0.029073	-0.013698	0.018833	-0.005765	-0.027112	-0.007056
	-0.004727	0.026617	-0.023164	0.017395	-0.013476	0.000983
	0.009604	-0.009492	0.000345	0.006675	0.030145	-0.015114
	0.030794	-0.005414	-0.009432	-0.036978	0.017977	-0.027207
	0.013235	-0.031236	-0.005245	-0.017699	-0.031558	0.016907
	0.009173					
z574	0.002816	-0.014566	-0.002664	0.033164	0.015672	0.015198
	-0.034522	0.006020	-0.004398	0.015540	0.012314	-0.001882
	0.028494	-0.040805	0.005631	0.002198	0.048736	0.021579
	0.014039	-0.004757	-0.007404	-0.048863	0.049348	-0.000748
	-0.000902	-0.016927	0.016297	-0.003356	-0.034728	0.001051
	0.012020					
x575	0.029051	-0.018031	-0.002052	0.005568	0.020650	0.005481
	-0.012399	-0.009922	0.006858	0.015653	-0.016786	-0.009300
	-0.023159	-0.022005	-0.011401	0.008390	-0.007199	0.020198
	0.012627	-0.009518	0.000844	-0.014635	-0.008510	-0.000543
	0.019962	0.003748	0.000358	0.013843	0.016034	0.004155
	0.011425					
y575	-0.035372	-0.009732	0.019488	-0.002435	-0.029999	-0.007710
	-0.010404	0.028895	-0.025456	0.017271	-0.010075	-0.000594
	0.019414	-0.017833	-0.000185	0.008569	0.028237	-0.012967
	0.024239	-0.005496	-0.010291	-0.039081	0.014830	-0.024465
	0.016265	-0.029549	0.010191	-0.023111	-0.042966	0.017048
	0.008610					
z575	-0.000227	-0.006708	-0.004350	0.029335	0.023787	0.014234
	-0.040366	0.001617	0.000589	0.016823	0.015626	-0.009224
	0.035876	-0.042646	0.008078	-0.001232	0.048064	0.015641
	0.011658	-0.006357	-0.005338	-0.050843	0.048533	-0.006806
	-0.001618	-0.006698	0.014326	-0.006802	-0.037851	0.006631
	0.010208					

x576	0.020848	-0.016081	-0.000125	0.006314	0.023489	0.005812
	-0.017103	-0.003643	0.002975	0.024012	-0.016938	-0.007931
	-0.017336	-0.027466	-0.019836	0.011022	-0.003119	0.025838
	0.016551	-0.010146	-0.002898	-0.016309	-0.010734	-0.006205
	0.020273	0.005391	0.004078	0.010718	0.003005	0.008482
	0.005629					
y576	-0.040109	-0.006782	0.020292	0.000202	-0.032473	-0.008566
	-0.014987	0.032387	-0.027203	0.014503	-0.005879	-0.002363
	0.023566	-0.024906	0.001191	0.011401	0.027896	-0.013939
	0.019108	-0.002855	-0.010956	-0.034222	0.012269	-0.020131
	0.008441	-0.035074	0.017751	-0.023875	-0.043454	0.018141
	0.005425					
z576	-0.002989	0.001369	-0.004946	0.025642	0.031001	0.013724
	-0.044082	-0.001935	0.007732	0.015834	0.018102	-0.017247
	0.041002	-0.041290	0.011036	-0.004222	0.043510	0.010445
	0.011371	-0.005594	-0.003853	-0.049995	0.044247	-0.009959
	-0.000038	0.001785	0.010365	-0.007507	-0.039468	0.011173
	0.006622					
x577	0.011611	-0.014141	0.001399	0.006898	0.024543	0.006075
	-0.019569	0.004335	-0.000394	0.030804	-0.017784	-0.007858
	-0.010841	-0.031371	-0.028347	0.014474	0.000374	0.030077
	0.020668	-0.008586	-0.007609	-0.017398	-0.013529	-0.010815
	0.021634	0.006317	0.009674	0.008292	-0.008819	0.012368
	-0.001364					
y577	-0.042982	-0.002573	0.022345	0.002415	-0.033883	-0.009202
	-0.022898	0.030647	-0.025801	0.012498	0.001372	-0.004277
	0.028374	-0.030727	0.000192	0.013226	0.021922	-0.013682
	0.009240	-0.002239	-0.009298	-0.026491	0.010426	-0.015030
	-0.001005	-0.035660	0.015159	-0.025819	-0.046861	0.021281
	0.002885					
z577	-0.005453	0.009712	-0.004635	0.022163	0.037189	0.014257
	-0.045945	-0.004847	0.017204	0.012281	0.020818	-0.025860
	0.044011	-0.037229	0.012999	-0.006054	0.034002	0.004745
	0.011260	-0.003359	-0.002084	-0.046524	0.037279	-0.009969
	0.002237	0.007470	0.003537	-0.006518	-0.035612	0.016152
	0.003184					
x578	0.001700	-0.012003	0.002792	0.006913	0.023797	0.006304
	-0.021092	0.013045	-0.002615	0.036722	-0.019643	-0.008940
	-0.002153	-0.033590	-0.037739	0.018526	0.001661	0.033546
	0.022312	-0.006611	-0.013039	-0.018160	-0.016388	-0.014214
	0.021903	0.009256	0.012904	0.004886	-0.022718	0.016600
	-0.008777					

y578	-0.045056	0.001465	0.026954	0.003994	-0.032628	-0.009329
	-0.030061	0.028754	-0.020156	0.008751	0.014323	-0.002987
	0.026883	-0.033751	0.001820	0.010742	0.016440	-0.016732
	0.004836	0.005871	-0.006783	-0.015352	0.006524	-0.006532
	-0.006283	-0.041736	0.015411	-0.021940	-0.045209	0.021406
	-0.004905					
z578	-0.007842	0.017791	-0.004054	0.018870	0.042395	0.015245
	-0.045712	-0.006378	0.028703	0.005395	0.022629	-0.035294
	0.044663	-0.031910	0.014304	-0.007796	0.019399	-0.001146
	0.011202	0.001090	-0.000015	-0.040830	0.027355	-0.006285
	0.005620	0.010084	-0.002765	-0.004214	-0.027243	0.020313
	-0.002144					
x579	-0.008813	-0.009571	0.005139	0.006266	0.021459	0.007392
	-0.022030	0.022280	-0.002034	0.039722	-0.021967	-0.010102
	0.007713	-0.031665	-0.047096	0.020962	0.000340	0.035675
	0.022485	-0.002758	-0.019102	-0.018267	-0.019546	-0.014467
	0.021603	0.012880	0.013394	0.002126	-0.037216	0.022378
	-0.014168					
y579	-0.044301	0.006756	0.031645	0.006441	-0.030335	-0.010624
	-0.036666	0.023724	-0.009399	0.000749	0.028258	-0.004956
	0.025151	-0.037937	0.008730	0.011028	0.009870	-0.019401
	-0.002096	0.011002	-0.001002	-0.003244	-0.003562	0.005338
	-0.014387	-0.043043	0.010652	-0.007765	-0.024160	0.028095
	-0.000991					
z579	-0.010329	0.025162	-0.003057	0.015722	0.046718	0.015962
	-0.043619	-0.006389	0.041665	-0.002976	0.024532	-0.044448
	0.041455	-0.024727	0.014129	-0.007644	-0.000221	-0.007785
	0.011623	0.007021	0.001110	-0.031183	0.014059	-0.001779
	0.009447	0.009583	-0.005675	-0.000102	-0.016775	0.021934
	-0.003216					
x580	-0.019045	-0.006230	0.008896	0.006118	0.018228	0.011364
	-0.023560	0.030654	0.002735	0.035583	-0.021930	-0.009653
	0.017909	-0.024201	-0.051477	0.017102	-0.004930	0.031864
	0.020568	0.005560	-0.025186	-0.016890	-0.016934	-0.006773
	0.017308	0.014361	0.010285	-0.000113	-0.041042	0.031651
	-0.018951					
y580	-0.041111	0.009501	0.033290	0.008397	-0.027148	-0.011800
	-0.040073	0.018494	-0.004314	-0.004519	0.040108	-0.005088
	0.019940	-0.035877	0.020914	0.011298	-0.002745	-0.027967
	-0.003654	0.013956	0.007699	0.010937	-0.000184	0.010588
	-0.021310	-0.043713	0.009233	0.000406	-0.011600	0.020682
	0.010102					

z580	-0.013250	0.032112	-0.001583	0.011437	0.049662	0.015922
	-0.039097	-0.003734	0.055480	-0.010485	0.026440	-0.054954
	0.033662	-0.019891	0.008125	-0.001198	-0.024745	-0.012882
	0.009886	0.016920	0.000244	-0.017238	-0.007999	0.000784
	0.016040	0.008580	-0.002442	0.008483	0.003215	0.027725
	-0.011920					
x581	-0.026666	-0.002779	0.014012	0.011459	0.015559	0.014838
	-0.024367	0.034920	0.007767	0.025837	-0.007556	-0.008811
	0.012456	-0.026091	-0.040749	0.014020	-0.006431	0.037679
	0.018404	0.001408	-0.023112	-0.007375	-0.006384	-0.006848
	0.004361	-0.009274	0.015296	0.005506	-0.029224	0.013942
	-0.008934					
y581	-0.035029	0.009620	0.031877	0.006926	-0.022868	-0.015623
	-0.036581	0.014333	-0.000051	-0.008738	0.045973	-0.005186
	0.015408	-0.034731	0.023737	0.010763	-0.005297	-0.033660
	-0.008742	0.015525	0.009907	0.012594	0.002509	0.024324
	-0.024998	-0.036452	0.006210	-0.002006	0.015008	0.023702
	0.007632					
z581	-0.015175	0.031920	-0.003674	0.005247	0.050313	0.016274
	-0.024211	0.002163	0.067716	-0.010089	0.015551	-0.058467
	0.023170	-0.010542	0.004924	-0.002535	-0.017934	-0.002043
	0.004731	0.020161	-0.004331	-0.018474	-0.004871	0.001547
	0.023687	-0.010208	-0.002930	0.008680	0.006845	0.021006
	-0.003871					
x582	-0.032902	0.001203	0.018524	0.015310	0.014162	0.018094
	-0.026462	0.037160	0.012855	0.014585	0.004193	-0.005895
	0.008868	-0.019210	-0.025819	0.010181	-0.010636	0.039060
	0.013947	0.000050	-0.023152	0.000712	0.005087	-0.003167
	-0.006724	-0.028679	0.018410	0.010079	-0.017406	0.004908
	0.006238					
y582	-0.029497	0.007056	0.027080	0.003095	-0.017056	-0.021388
	-0.032266	0.013882	-0.000672	-0.007814	0.042582	-0.003091
	0.013313	-0.026020	0.022604	0.013773	-0.012755	-0.038860
	-0.009898	0.017189	0.006731	0.018234	-0.000696	0.027437
	-0.024735	-0.025838	0.009850	-0.003880	0.022258	0.026594
	0.011587					
z582	-0.016858	0.031032	-0.006035	-0.000978	0.048056	0.014671
	-0.009334	0.006976	0.076543	-0.008862	0.006188	-0.061187
	0.011898	-0.005949	0.000098	-0.002028	-0.006990	0.010714
	-0.000157	0.021577	-0.007039	-0.023119	-0.003168	0.003765
	0.033375	-0.022192	-0.005911	0.009308	0.008919	0.014238
	-0.001846					

x583	-0.035790	0.003616	0.022603	0.018518	0.010702	0.021422
	-0.025305	0.038065	0.017471	0.005700	0.011369	-0.004384
	0.004882	-0.010555	-0.013198	0.005322	-0.009730	0.044473
	0.004187	0.002658	-0.022515	0.008832	0.015765	-0.000675
	-0.012534	-0.038931	0.020379	0.010855	-0.004419	-0.004228
	0.023319					
y583	-0.022010	0.003298	0.022380	0.000673	-0.014329	-0.026340
	-0.025542	0.012446	-0.002071	-0.005251	0.037671	-0.003283
	0.008748	-0.019371	0.016874	0.015158	-0.012594	-0.035869
	-0.012754	0.020634	0.002886	0.022204	-0.005229	0.027415
	-0.018709	-0.012783	0.013654	-0.005097	0.024300	0.023105
	0.019469					
z583	-0.019821	0.030391	-0.009009	-0.007275	0.046380	0.010934
	0.002833	0.013078	0.081421	-0.005142	-0.002207	-0.062006
	0.003507	-0.000986	-0.002831	0.000681	0.000099	0.017628
	-0.002781	0.021780	-0.009760	-0.026405	-0.002551	0.003068
	0.037391	-0.030803	-0.007913	0.010065	0.009633	0.010306
	-0.004726					
x584	-0.038035	0.003916	0.025662	0.020081	0.009800	0.022737
	-0.022932	0.040989	0.022299	0.001192	0.014740	-0.003848
	0.005537	-0.000923	0.002304	0.004380	-0.004621	0.046638
	-0.004157	0.007636	-0.022470	0.014510	0.026281	0.004349
	-0.010855	-0.043458	0.019862	0.007110	-0.003393	-0.012834
	0.033245					
y584	-0.016605	-0.001667	0.017329	-0.000831	-0.009055	-0.031886
	-0.019619	0.014047	-0.002997	-0.001321	0.031053	-0.003496
	0.007937	-0.012477	0.013638	0.018585	-0.008645	-0.035849
	-0.010623	0.025234	0.000290	0.023506	-0.010827	0.027829
	-0.010364	-0.000724	0.014956	-0.006360	0.017669	0.019600
	0.019016					
z584	-0.021433	0.031116	-0.011867	-0.013484	0.042340	0.006676
	0.013896	0.015948	0.082921	-0.001193	-0.008256	-0.062605
	-0.004519	0.001861	-0.007597	0.003785	0.005202	0.023470
	-0.007412	0.017463	-0.013984	-0.029854	-0.000907	0.001904
	0.036499	-0.035714	-0.011982	0.011503	0.011277	0.007591
	-0.002035					
x585	-0.038043	0.002405	0.026719	0.017257	0.009931	0.022933
	-0.019459	0.042770	0.024237	-0.002112	0.015898	-0.005262
	0.008352	0.006762	0.014863	0.003223	-0.000382	0.047605
	-0.012332	0.009043	-0.022394	0.015520	0.038134	0.007628
	-0.009767	-0.042546	0.025450	0.002432	-0.001214	-0.026383
	0.038315					

y585	-0.011504	-0.006120	0.010696	-0.003442	-0.004734	-0.036599
	-0.013190	0.013519	-0.006361	0.000361	0.025227	-0.004337
	0.009732	-0.008545	0.007401	0.020941	-0.008857	-0.037150
	-0.009009	0.023579	-0.002090	0.019640	-0.016675	0.026241
	-0.006689	0.009700	0.017640	-0.003702	0.016890	0.011717
	0.013442					
z585	-0.023904	0.032332	-0.013258	-0.017333	0.038605	0.000307
	0.023092	0.019987	0.082871	0.005079	-0.014854	-0.062053
	-0.011753	0.005874	-0.010782	0.005387	0.015033	0.030871
	-0.005683	0.017043	-0.016807	-0.032404	-0.002217	0.003873
	0.037298	-0.039392	-0.019737	0.009498	0.001461	0.006813
	0.003825					
x586	-0.037511	0.000521	0.027276	0.013215	0.010996	0.017568
	-0.015037	0.044590	0.023301	0.000115	0.016284	-0.009353
	0.007422	0.012524	0.027698	0.005884	0.006730	0.047205
	-0.017053	0.012219	-0.021840	0.015047	0.044668	0.010891
	-0.008385	-0.041019	0.028031	0.001076	-0.007291	-0.024252
	0.038107					
y586	-0.008006	-0.009431	0.003810	-0.004517	-0.000882	-0.042609
	-0.006137	0.012753	-0.011629	0.001466	0.018695	-0.004996
	0.008915	-0.006611	-0.000314	0.019955	-0.007977	-0.036141
	-0.002199	0.022870	-0.002218	0.014579	-0.028246	0.027163
	-0.006337	0.012945	0.016448	0.002703	0.014534	0.011805
	0.004818					
z586	-0.025300	0.035121	-0.016128	-0.021486	0.031916	-0.004745
	0.031667	0.019478	0.081884	0.009454	-0.018995	-0.059745
	-0.011184	0.009161	-0.015780	0.009328	0.015813	0.034465
	-0.010872	0.006802	-0.020642	-0.037552	0.003188	0.003037
	0.033720	-0.034563	-0.030682	0.006353	-0.001150	-0.003792
	0.010692					
x587	-0.036110	-0.002438	0.024727	0.005960	0.013191	0.009105
	-0.011271	0.041726	0.019959	0.009016	0.016652	-0.013402
	0.003973	0.015847	0.036793	0.011355	0.012668	0.046381
	-0.025702	0.010543	-0.019724	0.012887	0.049802	0.013033
	-0.008316	-0.036551	0.017913	-0.005000	-0.013752	-0.036148
	0.041297					
y587	-0.005645	-0.011106	-0.003636	-0.004552	0.000939	-0.046816
	0.001411	0.010474	-0.016828	0.000694	0.012118	-0.002898
	0.007654	-0.005824	-0.010064	0.015246	-0.009682	-0.034204
	0.005183	0.021014	-0.002044	0.006677	-0.038887	0.030093
	-0.008185	0.013491	0.009044	0.007774	0.011764	0.010461
	-0.000814					

z587	-0.027052	0.040040	-0.018040	-0.023722	0.023277	-0.010657
	0.040588	0.020190	0.081162	0.010254	-0.024827	-0.059991
	-0.007630	0.010770	-0.018772	0.011730	0.016370	0.038355
	-0.010090	0.001704	-0.017887	-0.040634	0.004583	0.001719
	0.031806	-0.029605	-0.029312	0.008763	0.001969	-0.002756
	0.003518					
x588	-0.034006	-0.005720	0.020604	-0.001731	0.013530	-0.001796
	-0.002703	0.037422	0.013911	0.017799	0.012735	-0.017554
	0.004042	0.024928	0.040228	0.021623	0.016595	0.048621
	-0.030551	0.012180	-0.020307	0.008618	0.051286	0.012197
	-0.010028	-0.033989	0.009714	-0.018767	-0.015642	-0.042782
	0.041695					
y588	-0.004388	-0.009752	-0.010297	-0.002955	0.000707	-0.046989
	0.008947	0.008965	-0.017201	-0.002897	0.005806	0.001449
	0.006070	-0.005418	-0.017443	0.007151	-0.011385	-0.033418
	0.014396	0.020790	-0.000286	-0.002689	-0.046529	0.034067
	-0.006060	0.014240	0.000617	0.012026	0.008817	0.014015
	-0.006930					
z588	-0.027669	0.046722	-0.018795	-0.025003	0.012991	-0.015696
	0.046814	0.018000	0.080124	0.011108	-0.028518	-0.060027
	-0.006029	0.006131	-0.018667	0.013903	0.014699	0.041482
	-0.011425	-0.005676	-0.012980	-0.043284	0.008847	-0.001897
	0.028668	-0.019963	-0.032763	0.016420	0.001303	0.006217
	0.001076					
x589	-0.032276	-0.004779	0.014599	-0.010006	0.013166	-0.012806
	0.008251	0.032630	0.007146	0.019490	0.005881	-0.018605
	0.001940	0.033559	0.042012	0.028870	0.017323	0.045281
	-0.035033	0.012034	-0.018329	-0.001356	0.039144	0.006382
	-0.016143	-0.030548	0.015483	-0.030406	-0.020906	-0.046553
	0.025189					
y589	-0.003024	-0.005488	-0.015512	0.000328	-0.001912	-0.042220
	0.013479	0.008561	-0.009450	-0.007310	0.001776	0.006264
	0.003511	-0.007594	-0.021018	-0.000465	-0.011626	-0.033938
	0.022832	0.019677	0.005039	-0.010118	-0.046553	0.041202
	0.003942	0.017279	-0.011902	0.019789	0.009124	0.019495
	-0.009056					
z589	-0.027662	0.052967	-0.018563	-0.026343	0.006438	-0.019472
	0.053737	0.016358	0.073775	0.017156	-0.033200	-0.057727
	-0.000482	0.006317	-0.014813	0.020211	0.014698	0.045968
	-0.014140	-0.011492	-0.011811	-0.042705	0.008681	-0.005966
	0.020080	-0.004205	-0.041081	-0.007542	-0.005554	0.023476
	0.009752					

x590	-0.031084	-0.001424	0.009255	-0.014642	0.014323	-0.019642
	0.010362	0.032784	0.004557	0.017579	-0.002027	-0.012293
	-0.001989	0.040006	0.039159	0.027207	0.016224	0.030633
	-0.031532	0.009254	-0.011247	-0.009095	0.028805	0.011355
	-0.028579	-0.025809	0.022054	-0.035490	-0.025623	-0.044476
	0.021853					
y590	-0.000090	0.000103	-0.019444	0.004009	-0.005703	-0.034790
	0.017228	0.006431	0.001145	-0.010359	0.001459	0.007158
	0.000072	-0.012354	-0.019872	-0.002648	-0.008834	-0.029942
	0.027253	0.016637	0.011323	-0.016131	-0.043888	0.040703
	0.014689	0.021050	-0.026514	0.025922	0.014958	0.023094
	-0.016374					
z590	-0.026234	0.053817	-0.015228	-0.028604	0.003046	-0.018075
	0.051468	0.015114	0.069320	0.017970	-0.037304	-0.058482
	-0.004946	0.008054	-0.011111	0.026153	0.011397	0.045436
	-0.016463	-0.008175	-0.008214	-0.041511	0.010221	-0.002160
	-0.000230	-0.002061	-0.042294	-0.015225	0.006699	0.039007
	0.025267					
x591	-0.028942	0.001837	0.004094	-0.017379	0.012998	-0.024635
	0.007480	0.028308	0.006155	0.011835	-0.005250	-0.010758
	-0.003622	0.042256	0.034053	0.022554	0.013980	0.015882
	-0.031449	0.006051	-0.003101	-0.016090	0.028599	0.013509
	-0.030784	-0.014660	0.019439	-0.034285	-0.031435	-0.046064
	0.020968					
y591	0.002799	0.006357	-0.022017	0.008724	-0.007657	-0.026215
	0.020503	0.007729	0.008924	-0.009759	0.002355	0.009075
	-0.004436	-0.021406	-0.015733	-0.001630	-0.003319	-0.023812
	0.031535	0.009470	0.015031	-0.018962	-0.044955	0.038248
	0.015853	0.020108	-0.032326	0.030013	0.022231	0.026767
	-0.020729					
z591	-0.024436	0.055061	-0.012605	-0.030014	-0.001891	-0.016859
	0.042450	0.012530	0.066146	0.015177	-0.037530	-0.056620
	-0.006650	0.004104	-0.014516	0.028947	0.011258	0.045274
	-0.015893	-0.006647	-0.003779	-0.041835	0.011199	0.000002
	-0.018697	0.000877	-0.034641	-0.015207	0.009512	0.034336
	0.032858					
x592	-0.026279	0.004384	-0.000478	-0.018674	0.010771	-0.027890
	0.003880	0.022170	0.006332	0.009141	-0.008094	-0.009521
	-0.007309	0.038213	0.029439	0.015531	0.010180	0.006659
	-0.032193	0.001033	0.001647	-0.023802	0.029020	0.011779
	-0.033437	-0.008461	0.015373	-0.031447	-0.029084	-0.050001
	0.020916					

y592	0.005154	0.012942	-0.023590	0.013216	-0.008313	-0.017968
	0.023302	0.011290	0.016866	-0.011517	0.005524	0.008865
	-0.006626	-0.028186	-0.010178	0.001933	0.002940	-0.019449
	0.033925	-0.000140	0.016777	-0.016199	-0.042638	0.035603
	0.018441	0.022172	-0.034083	0.032323	0.019718	0.037386
	-0.023603					
z592	-0.022892	0.055776	-0.010665	-0.031787	-0.006167	-0.015762
	0.033391	0.010454	0.063538	0.008808	-0.036714	-0.055858
	-0.004894	0.004224	-0.018002	0.031115	0.012249	0.042555
	-0.015005	-0.007844	0.001991	-0.039243	0.012175	0.001236
	-0.028273	0.005746	-0.025949	-0.017570	0.003414	0.028964
	0.031207					
x593	-0.024112	0.007522	-0.003150	-0.018319	0.008963	-0.029768
	0.001447	0.017895	0.008856	0.006475	-0.009519	-0.009750
	-0.012868	0.030409	0.028190	0.010150	0.007094	0.000895
	-0.031461	-0.005303	0.004418	-0.028546	0.028360	0.011002
	-0.032853	-0.007718	0.009051	-0.024746	-0.022593	-0.043775
	0.020310					
y593	0.007622	0.018651	-0.026706	0.016172	-0.009989	-0.010263
	0.025455	0.013649	0.019803	-0.012543	0.008094	0.008637
	-0.004954	-0.033135	-0.005680	0.003785	0.005161	-0.014051
	0.034256	-0.011351	0.015650	-0.013325	-0.034897	0.028106
	0.018554	0.028778	-0.028493	0.031277	0.012875	0.039331
	-0.023516					
z593	-0.021171	0.054905	-0.010382	-0.034630	-0.011637	-0.015015
	0.025473	0.007554	0.056988	0.003621	-0.036630	-0.053298
	-0.002135	0.004744	-0.021072	0.031832	0.009736	0.039204
	-0.013185	-0.010326	0.006490	-0.035882	0.019099	0.001708
	-0.036372	0.010850	-0.013648	-0.021137	-0.002695	0.017923
	0.026793					
x594	-0.021956	0.011678	-0.005441	-0.016319	0.006868	-0.030343
	-0.000475	0.016107	0.011471	0.004350	-0.008941	-0.010575
	-0.017433	0.021727	0.030487	0.005057	0.003231	-0.000145
	-0.029128	-0.014144	0.004578	-0.031716	0.029639	0.008256
	-0.027464	-0.007482	0.003553	-0.018516	-0.020259	-0.033574
	0.019809					
y594	0.009140	0.022734	-0.028573	0.016925	-0.010344	-0.003114
	0.028974	0.013989	0.021675	-0.015273	0.007146	0.008718
	-0.005108	-0.038027	-0.003259	0.004710	0.005286	-0.008466
	0.029335	-0.017833	0.014022	-0.011265	-0.031333	0.022801
	0.014911	0.032376	-0.017806	0.032140	0.012962	0.036634
	-0.021363					

z594	-0.019542	0.052332	-0.009705	-0.038187	-0.016556	-0.014094
	0.018698	0.004267	0.049157	-0.001629	-0.037238	-0.049817
	-0.000763	0.005557	-0.023040	0.031283	0.007401	0.037828
	-0.012821	-0.011201	0.009630	-0.032231	0.023002	0.004067
	-0.039630	0.012292	-0.000925	-0.024514	-0.008853	0.006622
	0.021845					
x595	-0.020281	0.016483	-0.007153	-0.013759	0.005154	-0.029744
	-0.000833	0.015325	0.013183	0.002028	-0.008061	-0.008978
	-0.022993	0.010712	0.034686	-0.000254	-0.004014	0.001474
	-0.028544	-0.026043	0.001572	-0.035007	0.034050	0.003490
	-0.022679	-0.006758	0.003142	-0.011338	-0.019090	-0.025352
	0.018167					
y595	0.010845	0.027322	-0.029204	0.016857	-0.011389	0.005701
	0.030645	0.016052	0.024072	-0.020026	0.006445	0.001079
	-0.004428	-0.038457	-0.001290	0.006111	0.011069	-0.000542
	0.028738	-0.014886	0.014836	-0.005280	-0.032936	0.020170
	0.014563	0.029802	-0.018172	0.029949	0.015519	0.046798
	-0.012913					
z595	-0.017969	0.048761	-0.008839	-0.042759	-0.021450	-0.012282
	0.011635	0.001872	0.040333	-0.006061	-0.035985	-0.048226
	0.000873	0.008236	-0.023167	0.031259	0.006603	0.037765
	-0.011625	-0.009762	0.011389	-0.026381	0.026217	0.004141
	-0.038953	0.008567	0.010657	-0.030279	-0.011529	-0.000297
	0.013623					
x596	-0.018771	0.022500	-0.007856	-0.010173	0.003782	-0.027487
	0.000531	0.017410	0.014503	-0.000403	-0.005418	-0.008128
	-0.028026	-0.000957	0.041777	-0.004080	-0.013029	0.007845
	-0.028144	-0.037255	-0.003204	-0.038371	0.036624	-0.001916
	-0.014033	-0.005435	0.001692	-0.002424	-0.017408	-0.013336
	0.016021					
y596	0.012329	0.028323	-0.028197	0.012807	-0.011104	0.012436
	0.031460	0.014599	0.023400	-0.023007	0.003947	0.005105
	-0.005854	-0.038388	-0.002841	0.007421	0.015658	0.000735
	0.024327	-0.015028	0.011578	-0.003751	-0.026599	0.021335
	0.001243	0.025404	-0.010302	0.024515	0.006477	0.041949
	-0.002546					
z596	-0.016259	0.042629	-0.007975	-0.048451	-0.026600	-0.010863
	0.004106	-0.000794	0.029951	-0.007808	-0.032543	-0.044180
	0.003507	0.012799	-0.022013	0.032389	0.007495	0.037080
	-0.010476	-0.008948	0.011358	-0.020204	0.030490	0.001903
	-0.036671	0.000065	0.024842	-0.039280	-0.015502	-0.008115
	0.000597					

x597	-0.016923	0.028551	-0.007366	-0.007132	0.003180	-0.023548
	0.004205	0.021096	0.012921	-0.004669	-0.002794	-0.006131
	-0.032838	-0.014385	0.048589	-0.007517	-0.023496	0.017512
	-0.029158	-0.046088	-0.009196	-0.041791	0.039328	-0.006096
	-0.004075	-0.001351	-0.000235	0.011513	-0.016531	-0.001913
	0.015258					
y597	0.013929	0.029734	-0.028333	0.007504	-0.009766	0.018274
	0.030094	0.014366	0.023539	-0.026324	0.005967	-0.000049
	0.001460	-0.028117	-0.003601	0.018444	0.032617	0.010540
	0.021078	-0.008243	0.012079	0.000683	-0.040388	0.022066
	-0.003439	0.015369	-0.001906	0.017789	-0.000038	0.044247
	-0.000021					
z597	-0.013956	0.033996	-0.007363	-0.054492	-0.032663	-0.009639
	-0.003722	-0.004366	0.017675	-0.006513	-0.028797	-0.039425
	0.005390	0.018062	-0.017974	0.033343	0.010843	0.036933
	-0.007038	-0.007000	0.010889	-0.012101	0.035776	-0.001489
	-0.030471	-0.013075	0.040415	-0.049141	-0.016195	-0.014604
	-0.016761					
x598	-0.013633	0.031544	-0.008623	-0.006886	0.002745	-0.017533
	0.009267	0.024943	0.006464	-0.008721	-0.000489	-0.004204
	-0.031182	-0.028364	0.052086	-0.008766	-0.031911	0.027212
	-0.028867	-0.048247	-0.013265	-0.042483	0.042807	-0.008121
	0.002902	0.006489	-0.003895	0.025545	-0.011807	0.009739
	0.016866					
y598	0.014550	0.026737	-0.030072	-0.001126	-0.010755	0.023588
	0.022788	0.008780	0.022143	-0.020499	0.002516	-0.003403
	0.010514	-0.013355	-0.006634	0.027173	0.046958	0.014230
	0.020240	0.001483	0.014080	0.006837	-0.037563	0.014489
	-0.022543	0.005837	0.012475	-0.007769	-0.005320	0.037338
	-0.007833					
z598	-0.010933	0.024173	-0.006793	-0.057831	-0.039045	-0.008317
	-0.010484	-0.007945	0.006121	-0.001946	-0.023855	-0.032811
	0.005142	0.019631	-0.008705	0.032679	0.016111	0.038797
	0.001566	-0.004524	0.010480	-0.006619	0.041855	-0.002576
	-0.017471	-0.025542	0.053799	-0.056597	-0.007965	-0.017695
	-0.030847					
x599	-0.007175	0.031151	-0.015985	-0.010370	-0.002047	-0.011471
	0.012846	0.024016	0.003191	-0.009169	-0.000909	-0.001821
	-0.021361	-0.025635	0.049186	-0.001335	-0.018332	0.025736
	-0.024078	-0.035402	-0.005987	-0.032138	0.031226	0.000421
	-0.005457	0.008713	0.006596	0.015459	-0.013066	0.013543
	0.001656					

y599	0.014433	0.023726	-0.026575	-0.005402	-0.010745	0.028063
	0.022498	0.003417	0.020115	-0.021884	0.000927	-0.000460
	0.017384	-0.003801	-0.008989	0.028296	0.051176	0.012359
	0.021582	0.007076	0.016577	0.017319	-0.034248	0.014429
	-0.022441	0.005298	0.014888	-0.003112	-0.004170	0.036127
	-0.010302					
z599	-0.008618	0.018504	-0.004548	-0.054372	-0.043531	-0.008573
	-0.014556	-0.004763	0.001161	-0.007103	-0.022445	-0.022094
	-0.002800	0.027821	0.001462	0.030917	0.011142	0.043154
	0.002874	0.000557	0.008368	-0.008826	0.043763	0.004235
	-0.017131	-0.016311	0.046992	-0.048463	-0.009064	-0.027217
	-0.024298					
x600	-0.001499	0.029540	-0.022361	-0.014794	-0.005145	-0.005386
	0.015094	0.022385	0.000593	-0.010662	-0.001783	0.001877
	-0.009842	-0.022353	0.042410	0.005132	-0.005304	0.025479
	-0.017982	-0.022611	0.001996	-0.023853	0.021660	0.008645
	-0.012846	0.011844	0.014299	0.008965	-0.012926	0.014974
	-0.003013					
y600	0.013025	0.021295	-0.020999	-0.005829	-0.008952	0.030654
	0.019924	-0.001255	0.018924	-0.022762	-0.003004	0.001666
	0.018030	0.002701	-0.008803	0.022906	0.048567	0.014320
	0.018987	0.010153	0.019076	0.018778	-0.029866	0.012839
	-0.018995	0.003777	0.013088	0.004647	-0.005662	0.031242
	-0.004121					
z600	-0.006202	0.013214	-0.002827	-0.049168	-0.046040	-0.007938
	-0.017776	-0.002129	-0.003405	-0.011575	-0.020144	-0.013250
	-0.008834	0.030800	0.011539	0.027374	0.005507	0.045523
	0.003566	0.004506	0.006698	-0.009727	0.042768	0.009740
	-0.015932	-0.006793	0.038231	-0.039939	-0.008243	-0.032038
	-0.021940					
x601	0.002979	0.027699	-0.026846	-0.018056	-0.004798	0.000054
	0.015844	0.023763	-0.002485	-0.013101	-0.001720	0.005761
	-0.003053	-0.019412	0.036862	0.012217	0.002945	0.023540
	-0.014862	-0.015306	0.008427	-0.019346	0.015758	0.014419
	-0.017713	0.016512	0.015559	0.005014	-0.012118	0.012855
	-0.004861					
y601	0.011119	0.019541	-0.015262	-0.003446	-0.005681	0.031177
	0.015531	-0.001882	0.018032	-0.021720	-0.006647	0.002791
	0.012474	0.007811	-0.005948	0.019093	0.042232	0.015054
	0.013519	0.009774	0.019875	0.016405	-0.024032	0.011418
	-0.016172	0.002014	0.006016	0.008658	-0.008555	0.022580
	-0.003400					

z601	-0.004118	0.008363	-0.001352	-0.043483	-0.047070	-0.005992
	-0.019411	-0.003379	-0.007654	-0.015080	-0.017830	-0.007729
	-0.011303	0.029298	0.019595	0.021453	0.000906	0.045898
	0.003306	0.007318	0.007895	-0.011686	0.038097	0.010113
	-0.013300	-0.000488	0.034224	-0.034570	-0.006809	-0.030313
	-0.016493					
x602	0.005667	0.025587	-0.028102	-0.021169	-0.003162	0.005864
	0.015987	0.022872	-0.004578	-0.016755	-0.002950	0.009828
	0.003458	-0.016791	0.031951	0.015400	0.008310	0.022810
	-0.011721	-0.010040	0.012985	-0.014640	0.008550	0.013460
	-0.021064	0.017435	0.020495	0.002926	-0.005706	0.015899
	-0.004387					
y602	0.008504	0.017783	-0.008062	-0.000907	-0.003283	0.031075
	0.011452	-0.005010	0.018816	-0.019594	-0.010890	0.003316
	0.007132	0.011497	-0.003133	0.012349	0.034838	0.017300
	0.010651	0.009394	0.019244	0.014957	-0.019703	0.006055
	-0.014293	-0.002280	0.004667	0.011133	-0.007137	0.019398
	-0.005104					
z602	-0.001817	0.003919	-0.001623	-0.037304	-0.046409	-0.003510
	-0.021209	-0.003330	-0.012106	-0.017592	-0.015544	-0.004087
	-0.012934	0.025658	0.024943	0.017524	-0.002315	0.045049
	0.000056	0.006938	0.010037	-0.015875	0.033186	0.008626
	-0.009100	0.005942	0.028975	-0.030581	-0.007309	-0.028201
	-0.016444					
x603	0.006762	0.024770	-0.028578	-0.022356	-0.000760	0.009811
	0.016087	0.025477	-0.006110	-0.018831	-0.005565	0.012129
	0.010062	-0.014330	0.030226	0.016858	0.010708	0.018614
	-0.009983	-0.009165	0.016400	-0.007711	0.001561	0.015490
	-0.025672	0.016628	0.016690	0.000863	-0.001289	0.021983
	-0.001103					
y603	0.005229	0.016929	-0.001373	0.002917	-0.001965	0.028910
	0.007665	-0.005644	0.020789	-0.014915	-0.015956	0.002172
	0.001981	0.014323	0.000497	0.005512	0.025953	0.017676
	0.008476	0.007070	0.016960	0.014470	-0.014759	0.004052
	-0.014477	-0.008190	-0.000984	0.011311	-0.007898	0.018854
	-0.006817					
z603	0.000408	-0.000626	-0.002133	-0.032007	-0.045065	-0.000777
	-0.022622	-0.006116	-0.016254	-0.019622	-0.012055	-0.001745
	-0.012856	0.020800	0.027655	0.014483	-0.004237	0.044891
	-0.004209	0.006204	0.011909	-0.020536	0.026554	0.002543
	-0.001760	0.010001	0.027319	-0.027296	-0.009122	-0.025491
	-0.019792					

x604	0.008437	0.025973	-0.028176	-0.022653	0.002133	0.014277
	0.018837	0.027373	-0.007499	-0.023181	-0.006628	0.016319
	0.011857	-0.013745	0.027787	0.018463	0.014390	0.014359
	-0.012469	-0.007435	0.019960	-0.001009	-0.008024	0.017670
	-0.022037	0.011669	0.024984	-0.001935	-0.001612	0.023352
	-0.004154					
y604	0.002336	0.017026	0.004962	0.007019	-0.001974	0.025767
	0.004969	-0.006548	0.023736	-0.009910	-0.020107	0.001559
	-0.004351	0.015796	0.002712	-0.001424	0.017760	0.018722
	0.005614	0.004602	0.013141	0.013827	-0.010917	0.003541
	-0.011612	-0.015089	-0.000621	0.011319	-0.011742	0.016643
	-0.013152					
z604	0.001518	-0.006097	-0.002929	-0.026824	-0.042017	0.002128
	-0.026133	-0.009488	-0.019001	-0.019715	-0.008894	-0.002453
	-0.009315	0.016251	0.029598	0.011945	-0.007397	0.044834
	-0.005790	0.003207	0.013549	-0.026021	0.021910	-0.008234
	0.001006	0.014571	0.019046	-0.028162	-0.007419	-0.017311
	-0.020840					
x605	0.009395	0.024977	-0.026170	-0.022207	0.004168	0.017898
	0.017819	0.025718	-0.006204	-0.022461	-0.014096	0.015996
	0.015458	-0.012166	0.026240	0.012875	0.006971	0.011694
	-0.018625	-0.006253	0.016787	0.000392	-0.007682	0.013576
	-0.025293	0.011402	0.028846	-0.012940	-0.001695	0.016734
	0.000870					
y605	-0.000904	0.016881	0.011251	0.011637	-0.003913	0.021912
	0.001285	-0.008040	0.028903	-0.003170	-0.025276	-0.000352
	-0.009149	0.017005	0.003571	-0.008445	0.007420	0.021668
	0.003502	0.002878	0.005796	0.012122	-0.005324	0.003466
	-0.009502	-0.021586	0.000155	0.010159	-0.016051	0.015354
	-0.020300					
z605	0.002520	-0.010322	-0.004434	-0.021883	-0.036826	0.004838
	-0.027340	-0.011531	-0.022768	-0.022077	-0.002598	-0.000822
	-0.007382	0.008939	0.028426	0.011478	-0.007214	0.042566
	-0.006081	-0.003334	0.017600	-0.027185	0.013316	-0.018323
	0.008740	0.015603	0.013815	-0.024616	-0.005112	-0.007831
	-0.026498					
x606	0.009094	0.025075	-0.022240	-0.019118	0.005813	0.022670
	0.017307	0.028371	-0.002264	-0.021197	-0.019172	0.014136
	0.021085	-0.008250	0.025871	0.010712	0.002810	0.017205
	-0.017764	-0.004740	0.016438	0.004912	-0.021247	0.009345
	-0.016958	0.001454	0.029923	-0.018330	0.006340	0.019950
	-0.003861					

y606	-0.004586	0.017021	0.016608	0.016645	-0.007440	0.017199
	-0.003322	-0.009189	0.035816	0.003766	-0.030328	-0.000669
	-0.012345	0.017599	0.001939	-0.014669	-0.002563	0.023395
	0.002690	0.001075	-0.003961	0.009196	0.000289	0.005427
	-0.007026	-0.025271	0.003855	0.010701	-0.021488	0.016914
	-0.028134					
z606	0.003254	-0.014492	-0.007030	-0.017876	-0.029439	0.006631
	-0.028158	-0.015982	-0.027247	-0.023544	0.004156	-0.000616
	-0.003246	-0.001407	0.025875	0.008380	-0.008921	0.035628
	-0.010201	-0.013419	0.020990	-0.029337	0.005643	-0.030492
	0.014199	0.019293	0.010195	-0.024214	-0.001823	0.002568
	-0.023194					
x607	0.006146	0.024852	-0.015805	-0.014904	0.005734	0.024544
	0.014424	0.030980	0.003747	-0.018326	-0.029745	0.016197
	0.022486	-0.010282	0.021380	0.000672	-0.008633	0.017088
	-0.019155	-0.006531	0.009722	0.008513	-0.023211	0.003820
	-0.017612	-0.005396	0.032749	-0.023587	0.005229	0.019399
	-0.020273					
y607	-0.008223	0.016309	0.019543	0.021264	-0.011352	0.013214
	-0.009633	-0.011607	0.041746	0.008327	-0.031896	0.001736
	-0.012386	0.019011	-0.001650	-0.014723	-0.007999	0.023483
	0.005978	-0.000447	-0.012287	0.003678	0.002057	0.007162
	-0.001580	-0.027090	0.010376	0.014632	-0.021668	0.021115
	-0.027918					
z607	0.004574	-0.017843	-0.010196	-0.014037	-0.019882	0.008044
	-0.027749	-0.020844	-0.033048	-0.023552	0.012191	-0.000887
	0.001576	-0.013511	0.022969	0.005170	-0.008076	0.026376
	-0.013389	-0.025065	0.026523	-0.031145	-0.006468	-0.044242
	0.023173	0.019592	0.006682	-0.024800	0.002772	0.015010
	-0.010753					
x608	0.003767	0.024965	-0.010947	-0.013695	0.007368	0.025035
	0.012084	0.030530	0.004820	-0.013926	-0.030001	0.019793
	0.024361	-0.007617	0.022620	-0.002231	-0.008899	0.017971
	-0.011420	-0.004500	0.004725	0.010002	-0.031164	0.003449
	-0.007224	-0.012275	0.032652	-0.022368	0.009956	0.019290
	-0.020965					
y608	-0.009942	0.011007	0.018544	0.020012	-0.010600	0.011555
	-0.015173	-0.019852	0.034802	0.009447	-0.021083	0.004244
	-0.012621	0.021493	0.002598	-0.009830	-0.007245	0.016560
	0.008042	-0.008673	-0.010589	0.001794	0.006737	-0.003574
	0.004352	-0.022055	0.009609	0.018085	-0.011113	0.012822
	-0.024237					

z608	0.004121	-0.013554	-0.010548	-0.012587	-0.016131	0.007922
	-0.025367	-0.023233	-0.038608	-0.017363	0.019405	0.006557
	0.008022	-0.006790	0.023165	0.009705	-0.003740	0.017723
	-0.014286	-0.015539	0.029963	-0.024778	-0.015998	-0.048735
	0.028047	0.013822	0.005789	-0.015624	0.016835	0.022585
	-0.013415					
x609	0.000820	0.023302	-0.005922	-0.013543	0.009958	0.024624
	0.007013	0.027061	0.003718	-0.010398	-0.026787	0.026943
	0.022757	-0.005503	0.020019	-0.003654	-0.010674	0.011218
	-0.002641	-0.006083	0.004231	0.012072	-0.032354	0.000811
	-0.001320	-0.019000	0.029833	-0.017160	0.016305	0.016704
	-0.018600					
y609	-0.011188	0.005486	0.016432	0.019090	-0.010328	0.010232
	-0.020017	-0.026575	0.029162	0.009373	-0.011267	0.005544
	-0.011842	0.022891	0.008292	-0.004861	-0.004086	0.013030
	0.009699	-0.015294	-0.011659	-0.002785	0.009677	-0.012621
	0.012183	-0.015281	0.009299	0.019345	-0.002776	0.005386
	-0.016117					
z609	0.003896	-0.009077	-0.010763	-0.011214	-0.012417	0.007074
	-0.022203	-0.024173	-0.042376	-0.011044	0.023129	0.012525
	0.014543	-0.001141	0.023402	0.010673	0.000486	0.014028
	-0.014212	-0.005498	0.031407	-0.019846	-0.024804	-0.050819
	0.034267	0.009737	0.005444	-0.009772	0.021388	0.028078
	-0.014680					
x610	-0.002041	0.021179	-0.002362	-0.012468	0.011786	0.023116
	0.001303	0.023139	0.002746	-0.006624	-0.022157	0.032726
	0.021120	-0.002568	0.016534	-0.001564	-0.011934	0.006508
	0.003524	-0.006232	0.005298	0.013703	-0.032893	-0.005991
	0.005802	-0.024367	0.028559	-0.013262	0.018936	0.012241
	-0.014699					
y610	-0.012383	0.000772	0.014707	0.017739	-0.009825	0.010025
	-0.024247	-0.031472	0.025247	0.007443	-0.004974	0.008176
	-0.011544	0.021892	0.012436	-0.002759	-0.001754	0.008926
	0.011973	-0.022359	-0.014664	-0.008322	0.013301	-0.015421
	0.017254	-0.008714	0.009276	0.022671	0.004793	0.000268
	-0.008461					
z610	0.003750	-0.005042	-0.010544	-0.010805	-0.008083	0.005914
	-0.018252	-0.023523	-0.043862	-0.005519	0.025139	0.018289
	0.019844	0.001246	0.023184	0.009097	0.003547	0.010745
	-0.013609	0.001437	0.031786	-0.014437	-0.032547	-0.052210
	0.037114	0.007821	0.003760	-0.005810	0.021607	0.032634
	-0.015034					

x611	-0.005167	0.017838	0.000818	-0.012951	0.014437	0.022119
	-0.004922	0.020231	0.002177	-0.005106	-0.018457	0.040214
	0.018622	-0.001580	0.013260	-0.003817	-0.011895	0.000698
	0.009702	-0.007409	0.002257	0.013110	-0.032058	-0.008147
	0.012352	-0.027228	0.028249	-0.009393	0.018748	0.003560
	-0.008451					
y611	-0.013300	-0.001982	0.013167	0.018134	-0.010698	0.009230
	-0.027690	-0.036564	0.023500	0.007161	0.000142	0.007297
	-0.011887	0.021215	0.014256	0.005297	-0.000529	0.008288
	0.012783	-0.026283	-0.012036	-0.011689	0.018672	-0.019711
	0.018735	-0.006899	0.006922	0.024243	0.011320	0.004861
	-0.004923					
z611	0.003470	-0.001676	-0.009946	-0.011318	-0.003698	0.003862
	-0.014446	-0.022415	-0.043316	-0.000167	0.025956	0.023068
	0.022663	0.001333	0.022535	0.007961	0.005476	0.008432
	-0.013611	0.007074	0.031685	-0.010388	-0.037186	-0.053194
	0.036862	0.006181	-0.000727	-0.003505	0.019334	0.038282
	-0.015991					
x612	-0.008568	0.013963	0.003803	-0.013712	0.016943	0.021036
	-0.012091	0.017188	0.003891	-0.004810	-0.015172	0.046392
	0.014274	-0.001387	0.009117	-0.005121	-0.011815	-0.004286
	0.016789	-0.009829	-0.000063	0.011725	-0.028052	-0.012103
	0.017161	-0.031361	0.025626	-0.007693	0.019681	-0.000329
	0.000046					
y612	-0.013548	-0.002934	0.011844	0.018932	-0.011473	0.008970
	-0.029053	-0.039951	0.021151	0.006473	0.004899	0.006118
	-0.010657	0.019087	0.015124	0.010318	0.002509	0.007846
	0.010565	-0.027404	-0.008854	-0.013140	0.022573	-0.015973
	0.017477	-0.002149	0.004437	0.027307	0.012126	0.007129
	-0.007423					
z612	0.003186	0.001369	-0.009357	-0.012474	0.001116	0.001164
	-0.010578	-0.020323	-0.041584	0.004585	0.025963	0.027226
	0.024520	-0.000523	0.020936	0.005733	0.006286	0.006401
	-0.014517	0.011215	0.029797	-0.007308	-0.040552	-0.053289
	0.035510	0.007274	-0.005025	-0.002117	0.011236	0.040202
	-0.017747					
x613	-0.011915	0.010259	0.006644	-0.014636	0.018946	0.020336
	-0.019461	0.013799	0.006935	-0.006716	-0.011586	0.051073
	0.009736	-0.003054	0.004502	-0.007494	-0.010674	-0.009165
	0.023496	-0.014311	-0.001405	0.010315	-0.021486	-0.015146
	0.020129	-0.035231	0.021022	-0.007120	0.022047	-0.000422
	0.007222					

y613	-0.013832	-0.003710	0.010379	0.019509	-0.011795	0.007977
	-0.030201	-0.042341	0.020248	0.007994	0.008836	0.005312
	-0.011080	0.018256	0.015099	0.017739	0.004594	0.009023
	0.008644	-0.024605	-0.000447	-0.014280	0.026488	-0.010752
	0.014199	-0.000067	0.003425	0.030331	0.003273	0.009147
	-0.008911					
z613	0.002642	0.003802	-0.008903	-0.014052	0.006041	-0.002364
	-0.006962	-0.017651	-0.038339	0.008811	0.024630	0.031228
	0.025068	-0.003671	0.018569	0.002768	0.005812	0.004921
	-0.016032	0.014385	0.026827	-0.006706	-0.042126	-0.053513
	0.034357	0.011173	-0.009364	-0.000634	0.000151	0.039167
	-0.019689					
x614	-0.015410	0.006461	0.009510	-0.015987	0.020696	0.019906
	-0.027384	0.010491	0.011729	-0.010878	-0.007470	0.054177
	0.004019	-0.006902	-0.000629	-0.010942	-0.008451	-0.014221
	0.030817	-0.018708	-0.000537	0.008641	-0.012885	-0.017373
	0.021914	-0.038797	0.014487	-0.006158	0.023878	0.002255
	0.012883					
y614	-0.013412	-0.002538	0.008902	0.020492	-0.012263	0.008094
	-0.030250	-0.043174	0.019060	0.010813	0.008896	0.004159
	-0.010088	0.019812	0.014549	0.024927	0.005371	0.009547
	0.004211	-0.024051	0.004529	-0.012317	0.029251	-0.007621
	0.009424	0.007062	-0.000703	0.026346	0.000885	0.011320
	-0.007219					
z614	0.001981	0.005746	-0.008909	-0.016453	0.011450	-0.006589
	-0.003798	-0.014777	-0.034315	0.013139	0.021494	0.034496
	0.025178	-0.008876	0.014749	-0.000025	0.002406	0.003474
	-0.018480	0.016064	0.021574	-0.007871	-0.041752	-0.052754
	0.033156	0.017477	-0.012156	0.000936	-0.014780	0.035137
	-0.022939					
x615	-0.018807	0.003037	0.012488	-0.017550	0.021847	0.020514
	-0.036193	0.007453	0.018504	-0.017405	-0.002940	0.055642
	-0.002475	-0.012632	-0.005797	-0.015543	-0.005023	-0.019074
	0.038106	-0.024745	0.003562	0.007467	-0.001427	-0.019029
	0.021870	-0.040845	0.004801	-0.006684	0.025918	0.007467
	0.017870					
y615	-0.012812	-0.000505	0.008396	0.020091	-0.013801	0.006794
	-0.027896	-0.044910	0.017512	0.014067	0.008280	0.004015
	-0.010050	0.021818	0.014814	0.033288	0.003247	0.010968
	-0.005006	-0.021284	0.004418	-0.008780	0.028474	-0.002954
	0.002975	0.018068	-0.000868	0.028940	-0.006709	0.011548
	-0.012132					

z615	0.001235	0.006935	-0.009643	-0.019726	0.017212	-0.012127
	-0.000549	-0.011325	-0.029266	0.017042	0.016707	0.036748
	0.024529	-0.016390	0.010495	-0.004026	-0.003218	0.002815
	-0.021278	0.015946	0.014577	-0.009709	-0.038644	-0.050996
	0.031610	0.025934	-0.013746	0.001630	-0.033702	0.027386
	-0.026347					
x616	-0.021978	0.001142	0.016715	-0.018028	0.021636	0.022775
	-0.045480	0.005087	0.027250	-0.023770	0.001454	0.055286
	-0.009887	-0.018103	-0.009928	-0.017753	-0.001482	-0.021678
	0.041232	-0.031352	0.008446	0.006815	0.009165	-0.021986
	0.020497	-0.036400	-0.009361	-0.007521	0.025107	0.015493
	0.019634					
y616	-0.012019	0.003247	0.006821	0.019980	-0.014756	0.004608
	-0.024651	-0.043421	0.014928	0.021276	0.005743	0.001024
	-0.010462	0.026783	0.015269	0.044914	-0.000595	0.014650
	-0.016305	-0.014911	0.002248	-0.003652	0.021763	-0.000609
	0.000580	0.034101	-0.003744	0.035909	-0.010473	0.014197
	-0.023371					
z616	0.000509	0.006719	-0.011436	-0.024727	0.023686	-0.019207
	0.003148	-0.007028	-0.023920	0.019141	0.011024	0.038047
	0.022865	-0.025361	0.005763	-0.011054	-0.009538	0.002092
	-0.021160	0.014657	0.006932	-0.010478	-0.031591	-0.048579
	0.029088	0.032954	-0.013351	-0.000024	-0.055809	0.019173
	-0.026354					
x617	-0.024971	0.002356	0.024189	-0.017510	0.015604	0.022897
	-0.049345	0.001168	0.034872	-0.015507	0.004502	0.057984
	-0.019815	-0.013391	-0.012266	0.000916	-0.006162	-0.011310
	0.026242	-0.034930	0.000387	0.004570	0.013967	-0.024557
	0.018862	-0.021516	-0.031362	-0.016399	0.019353	0.004206
	0.011366					
y617	-0.010630	0.003446	0.002569	0.017213	-0.012283	-0.000120
	-0.019620	-0.041211	0.009714	0.024033	0.002071	-0.002875
	-0.004480	0.025847	0.018835	0.047553	-0.002602	0.009333
	-0.023082	-0.006508	0.008184	-0.001214	0.017361	0.003988
	-0.001795	0.036462	-0.001516	0.035532	-0.025318	0.004217
	-0.025835					
z617	-0.001916	0.003213	-0.010790	-0.038823	0.030289	-0.030630
	0.007840	0.001354	-0.021878	0.020929	0.014483	0.047480
	0.021274	-0.025104	0.001456	-0.013712	-0.013448	0.004833
	-0.010785	0.018923	-0.003327	-0.016938	-0.013984	-0.047855
	0.025003	0.017626	-0.009322	-0.019173	-0.029630	0.000843
	-0.023979					

x618	-0.026339	0.004136	0.030003	-0.017236	0.010139	0.022099
	-0.050729	-0.002145	0.041409	-0.009290	0.007721	0.056214
	-0.028555	-0.005639	-0.013206	0.018521	-0.007941	-0.001305
	0.012110	-0.036929	-0.007153	0.004360	0.018704	-0.021700
	0.020385	-0.007988	-0.048871	-0.018869	0.020347	-0.006944
	0.004785					
y618	-0.008663	0.001177	-0.003113	0.012205	-0.007617	-0.008922
	-0.012656	-0.035838	0.001287	0.025920	-0.000312	-0.006201
	0.003018	0.024037	0.018394	0.044894	-0.004631	0.003686
	-0.026772	0.004711	0.013472	-0.003543	0.010546	0.004797
	0.008403	0.029273	0.007010	0.041539	-0.024957	-0.000729
	-0.026911					
z618	-0.004908	0.000086	-0.011724	-0.052593	0.036754	-0.042721
	0.011736	0.007605	-0.017254	0.021205	0.017820	0.055148
	0.022333	-0.024533	-0.002452	-0.017170	-0.017209	0.006080
	0.007962	0.018782	-0.013254	-0.022622	-0.001262	-0.036553
	0.023366	0.000851	-0.005279	-0.028265	-0.010405	-0.013561
	-0.019782					
x619	-0.028093	0.005274	0.032118	-0.018794	0.008432	0.018994
	-0.052411	-0.004440	0.046434	-0.006721	0.009019	0.052760
	-0.033755	0.002900	-0.011353	0.030583	-0.009671	0.002084
	0.006852	-0.037416	-0.010739	0.002711	0.023137	-0.013679
	0.018716	0.002111	-0.058162	-0.022945	0.023635	-0.013709
	-0.001140					
y619	-0.008226	-0.002329	-0.010631	0.005652	0.000346	-0.019667
	-0.006476	-0.029264	-0.006739	0.027732	-0.005505	-0.006328
	0.013236	0.021932	0.019917	0.040906	-0.012096	-0.003985
	-0.022114	0.013899	0.016460	-0.012325	0.004875	0.009460
	0.016307	0.020510	0.017760	0.046284	-0.020347	-0.000340
	-0.029231					
z619	-0.005516	-0.001783	-0.013697	-0.057894	0.037153	-0.054785
	0.017524	0.011319	-0.015031	0.023169	0.021997	0.060877
	0.023670	-0.015960	-0.013264	-0.021901	-0.015418	0.007138
	0.013948	0.022107	-0.019469	-0.010570	-0.000048	-0.048752
	0.016711	-0.003562	-0.007494	-0.033090	-0.001765	-0.022538
	-0.005209					
x620	-0.028154	0.005168	0.031282	-0.018453	0.005954	0.012111
	-0.051901	-0.005634	0.046716	-0.004153	0.009069	0.048729
	-0.038876	0.013904	-0.015278	0.035283	-0.005508	0.002858
	-0.003844	-0.029249	-0.007188	0.014341	0.020628	-0.021436
	0.017276	0.008847	-0.063397	-0.026866	0.020194	-0.020416
	-0.008773					

y620	-0.007499	-0.006025	-0.018271	-0.001637	0.007668	-0.030313
	-0.000149	-0.023489	-0.014881	0.030238	-0.010814	-0.005699
	0.021675	0.018272	0.019400	0.036024	-0.018298	-0.008373
	-0.016066	0.022922	0.016973	-0.020818	0.000300	0.018943
	0.028026	0.005703	0.025269	0.051911	-0.018918	-0.002207
	-0.032962					
z620	-0.005100	-0.002319	-0.015818	-0.058952	0.035262	-0.064723
	0.022140	0.012737	-0.012895	0.024905	0.026583	0.064614
	0.026060	-0.005840	-0.022470	-0.025072	-0.011989	0.006760
	0.016014	0.023700	-0.023323	0.003790	-0.001570	-0.068406
	0.008246	0.001886	-0.011240	-0.035596	0.002379	-0.022859
	0.010145					
x621	-0.027822	0.003906	0.027411	-0.022444	0.006130	0.000658
	-0.052694	-0.007433	0.045975	-0.001114	0.009628	0.040009
	-0.041946	0.023085	-0.018279	0.037950	0.000844	0.001543
	-0.007232	-0.020920	0.000343	0.019946	0.017190	-0.020884
	0.022230	0.011415	-0.066813	-0.032361	0.014575	-0.022426
	-0.014618					
y621	-0.005475	-0.010389	-0.027651	-0.007579	0.013865	-0.042245
	0.008257	-0.017563	-0.025707	0.034707	-0.016292	-0.003283
	0.031252	0.017914	0.016289	0.032110	-0.024871	-0.011660
	-0.015017	0.032451	0.015659	-0.025217	-0.006722	0.017253
	0.033098	-0.006231	0.028016	0.055348	-0.015928	0.000540
	-0.026531					
z621	-0.006503	-0.002207	-0.017495	-0.066663	0.037009	-0.072724
	0.023588	0.012930	-0.006791	0.022989	0.029611	0.063687
	0.027538	-0.008200	-0.021488	-0.028741	-0.006700	0.008290
	0.032397	0.022718	-0.025856	0.004453	0.003535	-0.051664
	0.021124	-0.006386	-0.004155	-0.030856	0.001766	-0.030160
	-0.004621					
x622	-0.028794	-0.001324	0.018648	-0.030789	0.010724	-0.019919
	-0.053318	-0.010292	0.042387	0.001204	0.005507	0.028906
	-0.044797	0.028297	-0.025996	0.033922	0.009576	-0.006473
	-0.003650	-0.001669	0.014503	0.024022	0.009086	-0.016652
	0.020518	0.013208	-0.046679	-0.040474	0.007235	-0.029264
	-0.035513					
y622	-0.002086	-0.014322	-0.037301	-0.013805	0.018973	-0.052521
	0.017726	-0.014242	-0.037100	0.039564	-0.020931	0.002604
	0.042231	0.017024	0.012251	0.031409	-0.033740	-0.015678
	-0.018689	0.035524	0.007997	-0.029653	-0.007543	0.011711
	0.033622	-0.014651	0.020249	0.057413	-0.010782	0.003591
	-0.011142					

z622 -0.006578 -0.001129 -0.017859 -0.065697 0.035862 -0.079377
0.025467 0.012870 -0.002750 0.021557 0.032814 0.061140
0.025941 -0.000929 -0.020271 -0.030779 0.004144 0.009319
0.035829 0.026426 -0.021828 0.025188 -0.001152 -0.053593
0.026872 -0.006181 0.005531 -0.021591 -0.003798 -0.034481
-0.014982

x623 -0.029694 -0.009624 0.003266 -0.042015 0.020044 -0.053311
-0.049689 -0.012457 0.032385 0.004053 -0.001804 0.009659
-0.047633 0.031921 -0.038365 0.029849 0.020076 -0.023440
0.008727 0.022464 0.030900 0.025919 -0.006121 0.001426
0.017443 0.012032 -0.009191 -0.036354 0.012735 -0.027963
-0.049009

y623 0.002449 -0.017809 -0.047380 -0.020832 0.023943 -0.059990
0.028967 -0.011970 -0.049560 0.045501 -0.026277 0.011253
0.055285 0.015145 0.012141 0.036726 -0.046734 -0.017530
-0.027884 0.031261 -0.007729 -0.030776 0.001868 0.005741
0.034502 -0.022266 0.003264 0.057986 -0.007429 0.006187
0.003272

z623 -0.006324 0.002199 -0.014642 -0.062358 0.032993 -0.078174
0.024166 0.008313 0.003875 0.016884 0.042738 0.060178
0.023582 0.007912 -0.013458 -0.028968 0.014273 0.013261
0.042965 0.019397 -0.019640 0.045744 -0.000252 -0.040119
0.029062 -0.004637 0.012150 -0.009673 -0.017271 -0.032869
-0.024067

x624 -0.028996 -0.020665 -0.019292 -0.054322 0.032702 -0.096030
-0.042014 -0.020140 0.016225 0.007559 -0.007853 -0.016803
-0.047729 0.032988 -0.061490 0.030751 0.023017 -0.051951
0.026683 0.040476 0.043102 0.024890 -0.017531 0.030001
0.011398 0.005296 0.033418 -0.019718 0.025501 -0.010028
-0.043907

y624 0.005682 -0.020190 -0.053265 -0.027362 0.028242 -0.058968
0.041033 -0.006528 -0.060108 0.049202 -0.034098 0.023402
0.067516 0.012353 0.022252 0.044042 -0.062890 -0.009969
-0.039406 0.023124 -0.038299 -0.024232 0.020223 0.008158
0.031383 -0.022028 -0.002657 0.054465 0.002265 -0.005486
0.019599

z624 -0.004449 0.010610 -0.004143 -0.050750 0.026139 -0.059803
0.019298 -0.000658 0.012118 0.009121 0.063596 0.063845
0.019848 0.016652 0.001160 -0.025174 0.018473 0.019496
0.049332 -0.010257 -0.021704 0.058878 0.005843 -0.010891
0.017721 0.002024 -0.000905 0.007668 -0.034774 -0.018837
-0.013760

x625	-0.025902	-0.026327	-0.033180	-0.059193	0.043794	-0.111670
	-0.034382	-0.025149	0.004396	0.010170	-0.010424	-0.036976
	-0.044045	0.017725	-0.072767	0.038229	0.010004	-0.076314
	0.027150	0.041685	0.042425	0.019569	-0.013660	0.041808
	0.006920	-0.008286	0.035486	-0.015285	0.023153	0.017908
	-0.023416					
y625	0.006140	-0.017847	-0.046548	-0.025328	0.027444	-0.043313
	0.047887	0.002006	-0.061902	0.046592	-0.037816	0.046826
	0.071037	0.010048	0.044083	0.047418	-0.062138	0.009813
	-0.052335	0.015017	-0.054368	-0.010703	0.031450	0.003533
	0.023398	-0.008823	0.003480	0.044646	0.002765	-0.026013
	0.025111					
z625	-0.002096	0.017961	0.005884	-0.035020	0.020403	-0.036088
	0.015561	-0.006603	0.014839	0.004620	0.078313	0.069742
	0.017734	0.016649	0.016961	-0.029872	0.020072	0.020937
	0.039364	-0.037987	-0.008113	0.060526	0.005914	-0.006600
	0.007491	0.003358	-0.028243	0.018898	-0.062365	-0.003105
	-0.013510					
x626	-0.032731	-0.010680	-0.018626	-0.006575	-0.011624	0.032134
	0.007845	-0.009600	0.005664	-0.043155	-0.028127	0.038584
	-0.004928	0.059485	-0.048327	-0.047015	-0.048335	0.004838
	0.050495	0.106368	0.025781	-0.051579	0.027458	0.010694
	0.002688	0.027528	0.013361	-0.038106	-0.092458	0.057637
	0.026111					
y626	-0.010421	-0.002774	0.023074	0.004456	-0.004786	-0.021106
	0.029108	0.015694	-0.010542	0.024218	-0.004660	0.011631
	-0.009335	0.001369	-0.029360	-0.001909	0.015537	0.006091
	0.037682	0.002668	-0.003644	-0.017287	0.038852	0.039025
	0.061295	-0.013096	-0.004048	-0.009797	-0.003651	0.019850
	0.009650					
z626	-0.006018	0.053307	0.001158	0.020203	-0.030972	-0.006921
	0.002743	0.008334	-0.012679	0.006343	-0.029052	0.010694
	-0.043423	0.026626	-0.094077	-0.027989	-0.067526	-0.024080
	0.017395	0.030529	-0.005790	-0.018680	0.059815	0.031486
	-0.027580	0.037692	0.023072	0.058367	0.008498	0.091907
	0.003060					
x627	-0.028460	-0.008860	-0.022705	-0.005492	-0.001783	0.031018
	0.004392	-0.020413	0.007933	-0.051025	-0.032065	0.046909
	-0.008247	0.056914	-0.038461	-0.050552	-0.034996	-0.002972
	0.051153	0.109363	0.023113	-0.053794	0.022354	0.028988
	-0.025124	0.026314	0.024523	-0.002375	-0.082114	0.017330
	0.016894					

y627	-0.013534	-0.009316	0.029605	0.000924	-0.010614	-0.026629
	0.038428	0.023644	0.000392	0.010956	-0.008231	0.012186
	-0.004114	0.010719	-0.025199	-0.012239	0.016850	0.008693
	0.042869	-0.004123	-0.013227	-0.014855	0.016311	0.026056
	0.037017	-0.006994	0.009184	-0.017670	0.002051	0.025365
	-0.010668					
z627	-0.005247	0.052946	-0.002734	0.027849	-0.020770	-0.008423
	-0.000986	0.004709	-0.026510	0.005871	-0.017607	-0.015213
	-0.031826	0.015978	-0.066686	-0.014033	-0.044197	-0.017833
	0.006081	0.011403	-0.012499	-0.024710	0.046999	0.018243
	-0.015364	0.025032	0.011631	0.045918	0.017933	0.054923
	0.011780					
x628	-0.025143	-0.008560	-0.025811	-0.003560	0.005626	0.028072
	0.001930	-0.029146	0.010785	-0.054596	-0.039603	0.056920
	-0.010826	0.051156	-0.032642	-0.051884	-0.024717	-0.012475
	0.051143	0.100720	0.019103	-0.047600	0.005582	0.033397
	-0.043692	0.028120	0.030325	0.014039	-0.060730	-0.001714
	0.000639					
y628	-0.014168	-0.012779	0.033484	-0.005292	-0.013117	-0.032328
	0.044190	0.027759	0.002188	0.006728	-0.006704	0.005179
	-0.006494	0.015927	-0.021577	-0.007950	0.012068	-0.000130
	0.043940	-0.010600	-0.016910	-0.019187	0.010412	0.023006
	0.027731	0.001414	0.010021	-0.012666	-0.005800	0.018323
	-0.014050					
z628	-0.005089	0.054383	-0.008184	0.034776	-0.013253	-0.006183
	-0.003043	-0.004507	-0.030993	0.008366	-0.006761	-0.031085
	-0.023410	0.008629	-0.047251	-0.005800	-0.028459	-0.007874
	-0.003980	0.003712	-0.011320	-0.024771	0.044091	0.014219
	-0.002085	0.013251	0.007140	0.033760	0.016251	0.032586
	0.017245					
x629	-0.021776	-0.008460	-0.028073	-0.002359	0.011376	0.023579
	-0.000195	-0.033716	0.011644	-0.055391	-0.043027	0.062994
	-0.009883	0.046216	-0.025371	-0.050067	-0.017976	-0.022689
	0.048962	0.085365	0.012906	-0.043797	-0.010125	0.030929
	-0.048491	0.027495	0.029492	0.019379	-0.040329	-0.003379
	-0.016516					
y629	-0.015424	-0.010959	0.033855	-0.007627	-0.014583	-0.034705
	0.045567	0.032105	0.002072	0.008360	-0.002564	-0.004904
	-0.004582	0.018415	-0.014035	-0.000454	0.014872	0.007634
	0.036013	-0.010243	-0.010865	-0.011588	0.002525	0.022294
	0.025024	0.004009	0.012473	-0.018004	-0.010945	0.019296
	-0.010966					

z629	-0.003988	0.054978	-0.012367	0.039495	-0.009381	-0.002187
	-0.003558	-0.016507	-0.033234	0.008764	0.000671	-0.038059
	-0.021043	0.005365	-0.039430	0.000782	-0.022655	-0.006925
	-0.006519	0.000888	-0.010813	-0.028357	0.049378	0.015917
	0.008532	0.006568	0.004167	0.032062	0.008723	0.013467
	0.022098					
x630	-0.018831	-0.007678	-0.029399	-0.000530	0.015341	0.019101
	-0.002918	-0.033982	0.010707	-0.051620	-0.042617	0.065401
	-0.006949	0.040004	-0.016658	-0.046740	-0.010228	-0.028265
	0.043379	0.069617	0.012056	-0.037710	-0.018950	0.025629
	-0.043916	0.022862	0.025662	0.017336	-0.023819	-0.002363
	-0.025015					
y630	-0.014890	-0.007708	0.032584	-0.006946	-0.018545	-0.034527
	0.048144	0.034102	0.001034	0.008642	-0.000414	-0.012407
	-0.005715	0.024623	-0.008867	0.004075	0.010041	0.017010
	0.033184	-0.009592	-0.006281	-0.013629	0.004150	0.020312
	0.023166	-0.002253	0.017478	-0.012010	-0.019182	0.020792
	0.001140					
z630	-0.003677	0.054944	-0.015081	0.041245	-0.005145	0.001400
	-0.006780	-0.028024	-0.035073	0.007968	0.006529	-0.041216
	-0.016791	0.000944	-0.036187	0.007187	-0.013004	-0.009922
	-0.009922	0.001266	-0.008652	-0.025741	0.046181	0.022009
	0.011466	0.012024	0.003505	0.022292	0.004147	-0.006196
	0.016371					
x631	-0.016048	-0.006187	-0.030136	0.002282	0.017597	0.014818
	-0.005588	-0.032598	0.009006	-0.045859	-0.039742	0.064683
	-0.002280	0.033638	-0.007509	-0.042841	-0.004168	-0.029629
	0.034657	0.052987	0.013899	-0.033633	-0.021127	0.019263
	-0.035710	0.015548	0.020129	0.012493	-0.011501	0.000006
	-0.028625					
y631	-0.014027	-0.004073	0.029430	-0.006547	-0.021300	-0.032310
	0.048337	0.034221	-0.000708	0.006171	0.002174	-0.017050
	-0.007675	0.028872	-0.008151	0.009594	0.008840	0.020233
	0.031892	-0.006705	-0.002712	-0.016362	0.003761	0.020536
	0.019576	0.001768	0.021000	-0.011005	-0.026044	0.012876
	0.006099					
z631	-0.002902	0.053978	-0.016069	0.043266	-0.003029	0.004010
	-0.008364	-0.038222	-0.037155	0.008365	0.008265	-0.041970
	-0.013942	-0.003226	-0.030150	0.011674	-0.007285	-0.009091
	-0.010563	0.002601	-0.005892	-0.019038	0.043889	0.027622
	0.013887	0.009862	0.005752	0.025370	0.002786	-0.017815
	0.019985					

x632	-0.013446	-0.004272	-0.030599	0.005555	0.018242	0.010909
	-0.007758	-0.029919	0.006613	-0.040139	-0.035309	0.061157
	0.002610	0.027748	-0.000288	-0.038620	0.001038	-0.029687
	0.025752	0.036855	0.016661	-0.032891	-0.019701	0.011804
	-0.023831	0.008531	0.013013	0.007043	-0.003940	0.003687
	-0.030226					
y632	-0.012787	0.000153	0.025446	-0.005541	-0.023588	-0.028747
	0.047366	0.030398	-0.002133	0.003907	0.002687	-0.018307
	-0.008975	0.030510	-0.006349	0.013363	0.008378	0.023176
	0.030677	-0.004086	-0.000719	-0.015595	0.001597	0.025345
	0.011572	0.004106	0.026283	-0.008035	-0.032619	-0.002075
	0.015844					
z632	-0.001557	0.051819	-0.016627	0.044634	-0.002118	0.005781
	-0.009461	-0.043693	-0.041046	0.007624	0.010195	-0.042282
	-0.011341	-0.005166	-0.027163	0.015756	-0.003120	-0.012742
	-0.008718	0.005856	-0.004067	-0.015379	0.042596	0.024880
	0.019630	0.011016	0.005389	0.025528	0.006928	-0.020708
	0.015068					
x633	-0.011605	-0.002109	-0.030276	0.008769	0.018535	0.007188
	-0.009371	-0.028263	0.004738	-0.034510	-0.031003	0.055362
	0.007325	0.022092	0.005807	-0.034551	0.006487	-0.027081
	0.014911	0.020454	0.020172	-0.031141	-0.017686	0.005874
	-0.011269	0.002847	0.002858	0.001582	-0.000324	0.004364
	-0.027254					
y633	-0.010391	0.004516	0.020136	-0.003917	-0.026597	-0.023799
	0.045616	0.026715	-0.004782	0.001363	0.002653	-0.017183
	-0.009217	0.030592	-0.005322	0.014980	0.007609	0.021380
	0.033200	0.000141	-0.000318	-0.017952	0.000802	0.030280
	0.004473	0.005721	0.034653	-0.003312	-0.035644	-0.014764
	0.020517					
z633	-0.001064	0.050658	-0.016625	0.045361	-0.000317	0.006749
	-0.011966	-0.050997	-0.045466	0.007694	0.011151	-0.042103
	-0.010103	-0.006739	-0.023030	0.018248	-0.001913	-0.008791
	-0.013591	0.007189	-0.005910	-0.008479	0.038201	0.019743
	0.018308	0.014649	-0.001739	0.019184	0.007041	-0.029944
	0.022321					
x634	-0.010060	-0.000598	-0.028569	0.012175	0.019511	0.002102
	-0.011124	-0.026543	0.004775	-0.030235	-0.030027	0.046602
	0.013308	0.016778	0.012328	-0.028934	0.016641	-0.024993
	0.007142	0.005155	0.025376	-0.030529	-0.014764	0.001686
	-0.000741	-0.000167	-0.003614	0.003311	0.001636	0.001451
	-0.025889					

y634	-0.007417	0.009916	0.012976	-0.001896	-0.030709	-0.016333
	0.043833	0.020408	-0.009863	-0.000024	0.004356	-0.013943
	-0.009301	0.030472	-0.006606	0.013688	0.002508	0.017912
	0.033072	0.003875	-0.003142	-0.018631	0.000395	0.033051
	-0.001611	0.007193	0.036793	-0.003654	-0.037379	-0.027268
	0.029639					
z634	-0.000179	0.048345	-0.015664	0.046809	0.003138	0.006011
	-0.016690	-0.054441	-0.047446	0.007578	0.009617	-0.042793
	-0.007267	-0.008278	-0.017578	0.024627	0.005093	-0.005809
	-0.014778	0.010212	-0.004976	-0.004847	0.034551	0.013654
	0.016052	0.018868	-0.008117	0.013595	0.016299	-0.041010
	0.019654					
x635	-0.008979	-0.000109	-0.025410	0.014507	0.019221	-0.001721
	-0.010718	-0.025887	0.004372	-0.030903	-0.033279	0.041785
	0.021303	0.013796	0.010722	-0.024352	0.021211	-0.023755
	0.002019	-0.009263	0.029463	-0.035295	-0.003603	-0.000722
	0.004664	0.000147	-0.000736	0.006424	-0.007014	-0.008981
	-0.014103					
y635	-0.003944	0.016236	0.004134	0.001278	-0.034393	-0.008828
	0.039737	0.013711	-0.015310	0.003378	0.010585	-0.014138
	-0.009691	0.027826	-0.003064	0.011123	0.002875	0.013481
	0.030543	0.007449	-0.008143	-0.012039	-0.008596	0.030639
	-0.004493	0.008935	0.027307	-0.002321	-0.027503	-0.026917
	0.027240					
z635	-0.000404	0.043524	-0.013723	0.047028	0.006533	0.005570
	-0.023060	-0.052703	-0.046540	0.007151	0.011440	-0.039953
	-0.002165	-0.007260	-0.010931	0.028100	0.008947	-0.000466
	-0.017056	0.011019	-0.006722	0.007750	0.027080	0.003739
	0.016650	0.025302	-0.017301	0.013394	0.026259	-0.025464
	0.012306					
x636	-0.009181	-0.001017	-0.022318	0.014311	0.018393	-0.001954
	-0.009597	-0.027618	0.003779	-0.031755	-0.035249	0.041530
	0.028182	0.012643	0.005816	-0.025158	0.023844	-0.025210
	0.003644	-0.017459	0.029385	-0.042733	0.004953	0.000668
	0.003882	-0.001240	0.006956	0.005828	-0.008377	-0.020496
	-0.006185					
y636	-0.000709	0.021805	-0.002292	0.004119	-0.037195	-0.005285
	0.037110	0.010216	-0.019564	0.004422	0.013612	-0.015260
	-0.008869	0.023815	0.000195	0.012383	0.002402	0.009271
	0.027456	0.008384	-0.009635	-0.008262	-0.014204	0.027116
	-0.005105	0.015406	0.019052	0.001793	-0.026439	-0.024524
	0.025006					

z636	-0.000785	0.041087	-0.010936	0.045258	0.009204	0.004193
	-0.023820	-0.051822	-0.046202	0.005009	0.010236	-0.033656
	0.005314	-0.010396	-0.009417	0.028674	0.007489	-0.005089
	-0.011418	0.009768	-0.005974	0.003391	0.024167	0.004535
	0.010158	0.035985	-0.016802	0.016469	0.028140	-0.022173
	0.004742					
x637	-0.009363	-0.003080	-0.019763	0.015196	0.015760	-0.005384
	-0.010969	-0.029740	0.003561	-0.028085	-0.033509	0.034417
	0.035084	0.013258	0.006144	-0.024554	0.031468	-0.020009
	0.002742	-0.024319	0.025614	-0.040842	0.002580	-0.007293
	0.008023	0.001181	-0.001023	0.014319	-0.007434	-0.024276
	-0.002481					
y637	0.002959	0.027484	-0.008129	0.007011	-0.038466	0.000761
	0.036303	0.005781	-0.024639	0.000177	0.009787	-0.012728
	-0.007539	0.019743	-0.002457	0.013745	-0.002561	0.000938
	0.025301	0.010454	-0.008240	-0.014279	-0.010883	0.029165
	-0.006624	0.019430	0.021530	-0.005174	-0.034083	-0.035834
	0.029875					
z637	-0.000412	0.038146	-0.008794	0.045545	0.012104	0.001058
	-0.023862	-0.050526	-0.046710	0.000735	0.004520	-0.029356
	0.011481	-0.010729	-0.012792	0.031349	0.010190	-0.006879
	-0.005297	0.006950	-0.006673	-0.005869	0.022891	0.005139
	0.013947	0.047437	-0.020382	0.013590	0.007467	-0.040020
	0.008754					
x638	-0.011067	-0.004458	-0.016230	0.013533	0.012174	-0.008887
	-0.011537	-0.032922	0.005575	-0.025467	-0.030574	0.028806
	0.037800	0.011216	0.006306	-0.022204	0.031956	-0.016569
	-0.005203	-0.029408	0.024544	-0.031605	0.001236	-0.018279
	0.003422	-0.000943	-0.003267	0.028224	0.004964	-0.011476
	-0.002657					
y638	0.007115	0.032286	-0.013534	0.010730	-0.038644	0.007043
	0.035185	0.002075	-0.029649	-0.003273	0.005447	-0.010860
	-0.006650	0.017295	-0.003065	0.014573	-0.005739	-0.006440
	0.024994	0.012456	-0.007320	-0.020420	-0.007716	0.028322
	-0.004571	0.022548	0.023568	-0.016571	-0.043624	-0.046435
	0.034347					
z638	-0.001888	0.034898	-0.004495	0.042762	0.014863	-0.001713
	-0.021835	-0.050409	-0.043447	-0.003039	0.000177	-0.024594
	0.011617	-0.012437	-0.013058	0.035061	0.006200	-0.008622
	-0.006385	0.005634	-0.002529	-0.003537	0.022673	0.000093
	0.008420	0.048401	-0.016212	0.015994	0.004811	-0.031741
	0.007814					

x639	-0.013127	-0.006241	-0.012994	0.012047	0.009264	-0.013095
	-0.013394	-0.034300	0.007263	-0.022708	-0.028269	0.022732
	0.043739	0.009064	0.004793	-0.020629	0.033634	-0.014299
	-0.012709	-0.033713	0.021648	-0.030114	-0.001182	-0.023895
	-0.000981	0.002528	-0.008816	0.035168	0.012101	-0.002649
	-0.003201					
y639	0.011570	0.035416	-0.017751	0.013915	-0.037571	0.013026
	0.033742	-0.002352	-0.034534	-0.006235	0.002141	-0.009135
	-0.004793	0.015793	-0.003657	0.012029	-0.005422	-0.014047
	0.025651	0.014888	-0.007504	-0.022655	-0.005625	0.026240
	-0.005362	0.024653	0.022503	-0.033942	-0.052706	-0.052819
	0.037953					
z639	-0.003855	0.029169	-0.000216	0.039804	0.019873	-0.005760
	-0.021580	-0.046591	-0.039849	-0.005547	-0.003448	-0.020491
	0.016980	-0.012762	-0.014592	0.035974	0.005961	-0.010298
	-0.009286	0.004093	-0.000671	-0.008721	0.018291	0.001673
	0.002748	0.053488	-0.016204	0.011018	-0.000696	-0.026856
	0.005042					
x640	-0.015305	-0.006930	-0.009677	0.011035	0.004830	-0.017295
	-0.014799	-0.034939	0.010409	-0.020721	-0.027502	0.015014
	0.049345	0.006352	0.000236	-0.017114	0.035185	-0.012854
	-0.016930	-0.036325	0.017851	-0.034045	0.000947	-0.027262
	-0.005513	0.002523	-0.015470	0.037232	0.014141	-0.005198
	0.000459					
y640	0.016174	0.036863	-0.021245	0.016683	-0.035112	0.018204
	0.031869	-0.006789	-0.038824	-0.008161	-0.000028	-0.008158
	-0.001760	0.016346	-0.002513	0.008106	-0.002711	-0.020625
	0.026134	0.018270	-0.009533	-0.022019	-0.003294	0.021776
	-0.008387	0.026132	0.020692	-0.055326	-0.060628	-0.057659
	0.039861					
z640	-0.005997	0.022781	0.004369	0.036742	0.024020	-0.010001
	-0.021074	-0.041181	-0.033861	-0.008083	-0.006963	-0.018647
	0.023880	-0.011936	-0.019205	0.037487	0.007985	-0.011241
	-0.011128	0.003185	0.001014	-0.016700	0.016946	0.003868
	-0.004049	0.053811	-0.018767	0.003883	-0.008585	-0.031639
	0.005526					
x641	-0.016967	-0.007359	-0.005361	0.012271	-0.001081	-0.021271
	-0.013822	-0.038661	0.012085	-0.017194	-0.029813	0.009622
	0.055169	0.005206	-0.002480	-0.016798	0.035761	-0.010587
	-0.015062	-0.037872	0.015631	-0.031079	0.002038	-0.036647
	-0.007966	0.003847	-0.019072	0.034737	0.007799	-0.003230
	0.013720					

y641	0.021353	0.036138	-0.024670	0.019454	-0.031776	0.022285
	0.029177	-0.009209	-0.042043	-0.009718	-0.001322	-0.008030
	0.000356	0.018614	-0.000085	0.005302	0.000738	-0.024258
	0.025831	0.021943	-0.011492	-0.017177	-0.001949	0.016788
	-0.010439	0.024962	0.017334	-0.072221	-0.064937	-0.058339
	0.041930					
z641	-0.007888	0.015183	0.010533	0.034794	0.026778	-0.013525
	-0.017584	-0.039925	-0.028940	-0.008145	-0.011827	-0.015205
	0.032893	-0.008343	-0.022399	0.033336	0.009830	-0.009256
	-0.007536	0.002897	0.005937	-0.014425	0.012952	-0.001668
	-0.009336	0.055461	-0.018595	-0.003073	-0.022147	-0.029324
	0.015751					
x642	-0.018790	-0.007870	-0.000877	0.009694	-0.005236	-0.024416
	-0.013870	-0.041288	0.016153	-0.014443	-0.029186	0.000892
	0.058488	-0.000164	0.000522	-0.013000	0.033471	-0.009234
	-0.009990	-0.038018	0.011515	-0.036655	0.005735	-0.032535
	-0.007112	-0.000312	-0.021569	0.032282	0.015955	0.002263
	0.009698					
y642	0.026684	0.033301	-0.028091	0.021260	-0.027760	0.025515
	0.025466	-0.011014	-0.044204	-0.011252	-0.000696	-0.009272
	0.004533	0.021987	0.003986	0.002302	0.004321	-0.025622
	0.023778	0.025582	-0.013156	-0.008702	-0.002013	0.010609
	-0.013508	0.021625	0.013808	-0.083410	-0.062296	-0.055342
	0.039144					
z642	-0.010267	0.006187	0.017197	0.028429	0.031374	-0.016503
	-0.015552	-0.036463	-0.020861	-0.009274	-0.011653	-0.015183
	0.040151	-0.007672	-0.021376	0.032459	0.010085	-0.007946
	-0.005526	0.002752	0.010074	-0.017389	0.009524	0.002635
	-0.013801	0.053111	-0.017961	-0.006705	-0.017083	-0.021704
	0.007336					
x643	-0.019271	-0.006458	0.002178	0.010005	-0.009748	-0.029178
	-0.012162	-0.039608	0.017528	-0.010207	-0.030665	-0.001395
	0.056913	-0.003640	0.002956	-0.010444	0.034377	-0.010552
	-0.003321	-0.040977	0.004483	-0.034632	0.006254	-0.030073
	-0.001573	-0.006782	-0.022559	0.022479	0.008006	0.000222
	0.016692					
y643	0.032395	0.028492	-0.031442	0.022384	-0.023624	0.027577
	0.021473	-0.011273	-0.045739	-0.012881	0.000392	-0.009234
	0.005031	0.025247	0.009027	-0.001527	0.006040	-0.026190
	0.020787	0.028233	-0.013004	0.006095	-0.002235	0.004842
	-0.015800	0.013392	0.009371	-0.082320	-0.056660	-0.047967
	0.036724					

z643	-0.011713	-0.002822	0.023199	0.023178	0.034580	-0.020404
	-0.012667	-0.028627	-0.013989	-0.008941	-0.011984	-0.011262
	0.041893	-0.004689	-0.021824	0.030326	0.013300	-0.007896
	-0.003375	-0.000340	0.011327	-0.013534	0.004427	0.005832
	-0.012936	0.050106	-0.017711	-0.011777	-0.023283	-0.020920
	0.008891					
x644	-0.019442	-0.003788	0.003386	0.009733	-0.014660	-0.033407
	-0.011707	-0.036779	0.019990	-0.009274	-0.029767	-0.001745
	0.055742	-0.009548	0.006121	-0.008364	0.029140	-0.016682
	0.000129	-0.039658	-0.002933	-0.032542	0.003500	-0.023168
	-0.003180	-0.016302	-0.018035	0.011170	0.006235	-0.001413
	0.016967					
y644	0.037392	0.024390	-0.034495	0.019198	-0.014806	0.027264
	0.019014	-0.008765	-0.042445	-0.013797	-0.002762	-0.011557
	-0.005330	0.017489	0.013998	-0.002947	0.003316	-0.021299
	0.018866	0.018588	-0.010455	0.007619	0.015026	0.014979
	-0.012294	-0.007401	0.010031	-0.056510	-0.029057	-0.028414
	0.011454					
z644	-0.012939	-0.011718	0.028721	0.015449	0.036441	-0.023522
	-0.008367	-0.019170	-0.006156	-0.010116	-0.011158	-0.007414
	0.039947	-0.006784	-0.022651	0.027050	0.010468	-0.011559
	-0.003262	-0.005861	0.012092	-0.010008	0.002379	0.013409
	-0.014567	0.042958	-0.011857	-0.006955	-0.018159	-0.015703
	0.001098					
x645	-0.017510	0.000614	0.004256	0.013300	-0.020808	-0.036575
	-0.011201	-0.036438	0.022368	-0.006771	-0.032882	-0.002148
	0.052305	-0.013435	0.016239	-0.002998	0.022395	-0.016444
	0.007750	-0.034139	-0.009603	-0.028496	0.001365	-0.017123
	-0.000470	-0.025739	-0.016131	0.001465	-0.004749	-0.002086
	0.021946					
y645	0.041587	0.015273	-0.033948	0.012657	0.001090	0.026774
	0.018471	-0.011487	-0.037664	-0.014003	0.002102	-0.014983
	-0.003845	0.013847	0.007995	-0.011966	0.001122	-0.010456
	0.021374	0.005814	-0.011891	-0.006184	0.025647	0.017065
	-0.013196	-0.031557	0.012673	-0.039743	-0.025538	-0.016372
	0.013761					
z645	-0.013076	-0.020692	0.035719	0.006181	0.034995	-0.022855
	-0.003024	-0.014187	0.002789	-0.011579	-0.011344	-0.005627
	0.037759	-0.004076	-0.019051	0.025823	0.005353	-0.012616
	-0.000645	-0.011281	0.013877	-0.007856	-0.002192	0.020209
	-0.012853	0.041241	-0.009099	-0.001168	-0.020986	-0.011302
	0.001490					

x646	-0.013255	0.005551	0.002803	0.021659	-0.027227	-0.040569
	-0.009360	-0.033687	0.021625	-0.003848	-0.033766	0.001724
	0.046185	-0.014835	0.024452	-0.002856	0.016016	-0.018533
	0.011697	-0.023292	-0.011723	-0.016208	-0.002102	-0.023499
	-0.002417	-0.031168	-0.009426	-0.011333	-0.013616	-0.005297
	0.027537					
y646	0.044455	0.005194	-0.033503	0.008832	0.010893	0.026669
	0.014571	-0.013386	-0.036986	-0.011753	0.010586	-0.014953
	0.004466	0.019436	0.002938	-0.024545	0.006020	-0.005295
	0.018164	0.003924	-0.010197	-0.009059	0.023231	0.005437
	-0.020886	-0.044104	0.011790	-0.040448	-0.029261	-0.014262
	0.027436					
z646	-0.011903	-0.027535	0.041455	-0.000257	0.028706	-0.022319
	0.005883	-0.008962	0.007518	-0.012467	-0.010470	-0.002733
	0.029763	-0.002368	-0.016904	0.021273	-0.002252	-0.015848
	-0.000125	-0.013382	0.018021	0.001603	-0.004517	0.018918
	-0.009200	0.043647	-0.001321	0.007696	-0.021408	-0.008916
	0.004349					
x647	-0.008467	0.011506	-0.000778	0.030217	-0.032743	-0.042556
	-0.008545	-0.030595	0.021051	-0.000973	-0.035627	0.004745
	0.039652	-0.013870	0.032154	-0.001735	0.010996	-0.017726
	0.011738	-0.017073	-0.009238	-0.003650	-0.005438	-0.030046
	-0.006554	-0.036442	-0.003269	-0.023531	-0.020065	-0.005252
	0.027005					
y647	0.045868	-0.005082	-0.031962	0.003842	0.017420	0.027851
	0.010480	-0.013893	-0.036806	-0.008180	0.020238	-0.014960
	0.011623	0.025032	0.000612	-0.035434	0.010952	0.000123
	0.011654	0.005718	-0.004952	-0.005557	0.016031	-0.008469
	-0.026992	-0.048498	0.011743	-0.042242	-0.025481	-0.015448
	0.039901					
z647	-0.010080	-0.032064	0.044620	-0.006113	0.020718	-0.019426
	0.014229	-0.005110	0.010587	-0.015216	-0.009122	-0.001371
	0.020784	0.002288	-0.015936	0.019344	-0.010597	-0.018031
	-0.003094	-0.021314	0.023867	0.010001	-0.005615	0.017524
	-0.005644	0.046688	0.004754	0.016959	-0.022899	-0.001563
	0.000038					
x648	-0.003732	0.017080	-0.004646	0.036541	-0.036651	-0.043285
	-0.006470	-0.029687	0.021781	-0.000966	-0.038505	0.006133
	0.033866	-0.016072	0.038422	-0.000108	0.011617	-0.011007
	0.007285	-0.007977	-0.011459	0.008205	-0.009153	-0.035822
	-0.007917	-0.042788	0.004991	-0.034208	-0.019472	-0.007010
	0.024573					

y648	0.046180	-0.015736	-0.029777	-0.002037	0.022420	0.030664
	0.007300	-0.013026	-0.037366	-0.004616	0.031971	-0.015642
	0.017656	0.030637	0.000176	-0.041759	0.012924	0.005388
	0.005685	0.008218	0.001642	0.002162	0.006760	-0.022909
	-0.029448	-0.044500	0.012083	-0.042635	-0.013280	-0.016036
	0.048656					
z648	-0.007129	-0.034299	0.045378	-0.009155	0.012324	-0.017316
	0.024541	-0.003840	0.012464	-0.021839	-0.007474	0.000597
	0.011848	0.003293	-0.014900	0.020225	-0.014146	-0.014532
	-0.005881	-0.025092	0.018897	0.016366	-0.006103	0.019009
	0.004605	0.042976	0.008605	0.027842	-0.021325	0.009327
	-0.004649					
x649	0.002928	0.016498	-0.008313	0.039831	-0.036027	-0.039225
	-0.006443	-0.027147	0.018392	-0.001400	-0.033549	0.006307
	0.028175	-0.016564	0.043793	-0.003681	0.004903	-0.007109
	0.003869	-0.004743	-0.010735	0.017781	-0.006491	-0.039289
	-0.008637	-0.041385	0.008851	-0.040698	-0.014132	-0.013502
	0.023521					
y649	0.043843	-0.021769	-0.026440	-0.008257	0.023271	0.031305
	0.005687	-0.014441	-0.032877	-0.001726	0.035536	-0.017224
	0.021767	0.032572	-0.000425	-0.041311	0.016111	0.006046
	-0.000377	0.013081	0.005733	0.009486	-0.002647	-0.030103
	-0.028133	-0.038745	0.014999	-0.043261	-0.003985	-0.018281
	0.050486					
z649	-0.004682	-0.032885	0.043438	-0.009837	0.002804	-0.014525
	0.029703	-0.003184	0.013921	-0.029912	-0.013724	0.003452
	0.004358	0.002708	-0.010823	0.018139	-0.015238	-0.012297
	-0.003037	-0.032159	0.014709	0.019714	0.002802	0.026758
	0.010789	0.036345	0.009699	0.037118	-0.017055	0.014839
	-0.007918					
x650	0.011216	0.010945	-0.011250	0.041125	-0.032065	-0.033299
	-0.003850	-0.024208	0.011103	-0.001117	-0.021005	0.004031
	0.021868	-0.015444	0.042480	-0.009655	-0.005635	-0.003155
	-0.004315	-0.001432	-0.010808	0.024676	-0.005800	-0.042976
	-0.007495	-0.039728	0.009804	-0.044339	-0.011116	-0.018895
	0.023764					
y650	0.039014	-0.022042	-0.022384	-0.014265	0.021342	0.031376
	0.004075	-0.018271	-0.022074	-0.000785	0.026199	-0.018173
	0.024144	0.031695	-0.004666	-0.036696	0.022459	0.008453
	-0.002770	0.015547	0.010901	0.014256	-0.007443	-0.035526
	-0.026310	-0.028887	0.025101	-0.040767	0.006698	-0.019838
	0.042694					

z650	-0.002486	-0.028907	0.039455	-0.006057	-0.008399	-0.013552
	0.035964	-0.002561	0.013281	-0.037885	-0.024057	0.006581
	-0.003297	0.005180	-0.010599	0.012836	-0.012807	-0.009054
	-0.006769	-0.031640	0.007078	0.020722	0.008351	0.028570
	0.011566	0.021345	0.006825	0.042485	-0.020236	0.020991
	-0.007676					
x651	0.018651	0.004985	-0.011933	0.037449	-0.027038	-0.026144
	-0.000569	-0.024483	0.006930	-0.000780	-0.012120	-0.002355
	0.016789	-0.015492	0.039958	-0.012676	-0.015904	-0.000088
	-0.011385	0.001113	-0.010661	0.027797	-0.006988	-0.041392
	-0.005008	-0.037282	0.010801	-0.046187	-0.005252	-0.023823
	0.014605					
y651	0.033642	-0.020387	-0.018569	-0.017605	0.018458	0.029918
	0.005147	-0.021463	-0.010523	-0.000099	0.015162	-0.019001
	0.024025	0.028990	-0.006734	-0.028645	0.026551	0.007807
	-0.001808	0.018571	0.013832	0.016805	-0.010446	-0.033809
	-0.019610	-0.020463	0.029504	-0.035641	0.011878	-0.019201
	0.032776					
z651	0.000518	-0.025179	0.036063	-0.000493	-0.020326	-0.010504
	0.037371	-0.001533	0.008066	-0.046713	-0.030507	0.009492
	-0.009259	0.011417	-0.006445	0.007754	-0.012644	-0.003277
	-0.008921	-0.027077	0.001864	0.020919	0.006781	0.025740
	0.014364	0.008172	-0.000679	0.044118	-0.017558	0.022564
	-0.008503					
x652	0.025642	-0.002347	-0.011830	0.030905	-0.018093	-0.018279
	0.004260	-0.028335	0.007290	-0.000329	-0.004676	-0.008587
	0.012239	-0.019630	0.029891	-0.010308	-0.025616	0.001662
	-0.018891	0.001992	-0.012473	0.026090	-0.001617	-0.033732
	-0.001669	-0.032500	0.013145	-0.044353	-0.005195	-0.027086
	0.002697					
y652	0.028132	-0.018599	-0.013857	-0.018618	0.015693	0.027901
	0.008091	-0.023287	-0.000610	-0.000137	0.003592	-0.018859
	0.021349	0.025298	-0.008537	-0.021210	0.026689	0.006105
	0.000108	0.019575	0.015839	0.015777	-0.008710	-0.029298
	-0.009936	-0.010696	0.030628	-0.026754	0.011817	-0.016391
	0.022023					
z652	0.003956	-0.023925	0.031851	0.007543	-0.028186	-0.006461
	0.035474	-0.001745	0.002245	-0.055921	-0.031843	0.016624
	-0.016932	0.019037	-0.009157	0.004214	-0.016014	0.001680
	-0.017159	-0.019368	-0.005388	0.019256	0.009923	0.024106
	0.014574	-0.003146	-0.019375	0.039372	-0.017898	0.016208
	-0.000645					

x653	0.032471	-0.011415	-0.009525	0.021262	-0.006149	-0.007975
	0.010901	-0.033154	0.017944	0.002130	0.002575	-0.016155
	0.011930	-0.025381	0.013135	-0.000980	-0.035907	-0.000239
	-0.026503	0.000144	-0.022011	0.021843	0.004396	-0.022518
	-0.001271	-0.029986	0.023622	-0.036112	-0.011164	-0.018362
	-0.013391					
y653	0.023077	-0.017849	-0.006705	-0.018339	0.013446	0.027495
	0.011914	-0.022794	0.009422	0.000157	-0.006266	-0.018583
	0.017539	0.021853	-0.013033	-0.013056	0.020261	0.002486
	0.000659	0.018818	0.010314	0.014277	-0.006365	-0.022971
	-0.003719	-0.002189	0.026704	-0.014457	0.004183	-0.007996
	0.013698					
z653	0.007110	-0.025132	0.027629	0.016906	-0.034901	-0.004055
	0.030862	0.001824	-0.001044	-0.060114	-0.031875	0.023942
	-0.025787	0.032280	-0.005609	-0.002853	-0.018265	0.001257
	-0.023874	-0.014036	-0.004995	0.009351	0.010283	0.016274
	0.013285	-0.016801	-0.037721	0.032715	-0.013016	0.007072
	0.013389					
x654	0.037850	-0.014069	-0.008805	0.009795	0.001575	-0.001552
	0.009988	-0.035483	0.029221	-0.003490	0.011067	-0.020042
	0.008290	-0.022469	0.004940	-0.006123	-0.037742	-0.008939
	-0.020680	-0.009392	-0.017850	0.013869	0.011274	-0.009503
	0.007967	-0.033678	0.015159	-0.036730	-0.010141	-0.021779
	-0.011688					
y654	0.015755	-0.013598	0.000540	-0.014500	0.006323	0.022380
	0.011897	-0.018318	0.014821	-0.002954	-0.016390	-0.014532
	0.007303	0.025639	-0.010542	-0.012592	0.016972	-0.001152
	0.003996	0.012909	0.012287	0.008510	-0.000461	-0.018318
	0.006110	-0.000918	0.005431	-0.007619	0.002858	-0.001176
	0.014050					
z654	0.008528	-0.028861	0.022257	0.024506	-0.033584	0.001564
	0.022200	-0.002357	-0.006708	-0.057414	-0.026046	0.030164
	-0.024582	0.030478	-0.009882	-0.003360	-0.019389	0.001297
	-0.024051	-0.011134	-0.011169	-0.002832	0.015498	0.018610
	0.007506	-0.022549	-0.032201	0.027659	-0.021026	0.003628
	0.019959					
x655	0.041583	-0.015032	-0.009555	0.002066	0.007626	0.001611
	0.007451	-0.038906	0.034605	-0.005715	0.015148	-0.021451
	-0.000792	-0.020692	0.000932	-0.013972	-0.031908	-0.013835
	-0.011355	-0.017598	-0.005742	-0.001351	0.015867	-0.002357
	0.018097	-0.029156	0.007983	-0.033242	-0.004328	-0.028836
	-0.007627					

y655	0.009451	-0.010786	0.005707	-0.010425	-0.000201	0.016241
	0.010789	-0.013909	0.012450	-0.001676	-0.023982	-0.011980
	-0.002668	0.030315	-0.007010	-0.016970	0.013940	-0.002905
	0.006262	0.004860	0.016510	0.002047	0.004457	-0.018530
	0.011134	-0.001626	-0.015605	-0.002932	0.003555	0.000830
	0.019880					
z655	0.009529	-0.032216	0.016248	0.032216	-0.031124	0.004394
	0.013848	-0.006986	-0.012154	-0.051088	-0.020063	0.032282
	-0.025135	0.022711	-0.015334	-0.003539	-0.010005	0.003543
	-0.020246	-0.004792	-0.013709	-0.011971	0.014362	0.020291
	0.000763	-0.027221	-0.021154	0.021355	-0.028627	-0.006447
	0.028307					
x656	0.042821	-0.015285	-0.009688	-0.004037	0.012035	0.001974
	0.005055	-0.040981	0.037324	-0.005221	0.015419	-0.022850
	-0.008873	-0.021870	0.000323	-0.019006	-0.030012	-0.015060
	-0.007491	-0.024261	0.006114	-0.013967	0.015567	0.002827
	0.023968	-0.017800	0.003385	-0.024842	0.003239	-0.024748
	-0.004165					
y656	0.003680	-0.009021	0.008536	-0.007966	-0.005243	0.009245
	0.009144	-0.009381	0.005483	0.001881	-0.028958	-0.011836
	-0.010423	0.034168	-0.003933	-0.022994	0.009239	-0.003149
	0.005266	-0.004533	0.023277	-0.002511	0.007556	-0.020850
	0.012296	-0.002809	-0.033288	0.000895	0.007344	0.000468
	0.026975					
z656	0.009096	-0.033396	0.011812	0.036963	-0.026675	0.005171
	0.005227	-0.010617	-0.016351	-0.042670	-0.014804	0.031552
	-0.023636	0.015243	-0.016864	-0.000953	-0.011707	0.014247
	-0.025971	-0.002232	-0.006369	-0.014413	0.010107	0.018675
	-0.009355	-0.018495	-0.009699	0.018990	-0.020797	-0.003057
	0.030797					
x657	0.041705	-0.014807	-0.010729	-0.007456	0.015538	0.001733
	0.002350	-0.042260	0.034911	-0.003543	0.009404	-0.024843
	-0.017787	-0.023824	0.000521	-0.021940	-0.027573	-0.010846
	-0.006233	-0.027349	0.013985	-0.023777	0.011020	0.006599
	0.028581	-0.007145	0.001850	-0.014378	0.005799	-0.020605
	0.001457					
y657	-0.001731	-0.007997	0.009457	-0.007655	-0.007242	0.002728
	0.006450	-0.006694	-0.005405	0.005003	-0.031737	-0.014000
	-0.017313	0.036908	-0.002495	-0.030465	0.004590	0.000265
	0.000531	-0.015362	0.034384	-0.005810	0.008238	-0.023530
	0.013141	-0.007473	-0.047513	0.002293	0.013080	-0.008393
	0.033178					

z657	0.007268	-0.031680	0.008303	0.041049	-0.020115	0.006177
	-0.004224	-0.014587	-0.017843	-0.035356	-0.013237	0.028772
	-0.023775	0.006425	-0.017263	0.003200	-0.012528	0.031069
	-0.029790	0.002549	-0.001993	-0.015806	0.005409	0.020304
	-0.015700	-0.014324	0.008898	0.019745	-0.018447	0.004749
	0.029705					
x658	0.039150	-0.014615	-0.011824	-0.008268	0.018387	0.001317
	0.000514	-0.039554	0.028876	-0.000681	0.004750	-0.024520
	-0.021742	-0.024972	0.000896	-0.021707	-0.021859	-0.011177
	-0.001241	-0.029029	0.016816	-0.029248	0.004680	0.004448
	0.033277	-0.000815	0.001181	-0.008797	0.008626	-0.021178
	0.008310					
y658	-0.008211	-0.006318	0.012505	-0.003841	-0.011234	-0.000629
	0.003212	-0.000790	-0.009155	0.006528	-0.030498	-0.010436
	-0.015768	0.036904	-0.003574	-0.026909	0.002971	0.000071
	0.001770	-0.015141	0.032343	-0.005337	0.008548	-0.021457
	0.008607	-0.015363	-0.044621	0.001080	0.011606	-0.006978
	0.030320					
z658	0.006473	-0.030055	0.005300	0.041799	-0.010863	0.006230
	-0.011614	-0.016262	-0.018028	-0.026485	-0.001471	0.023296
	-0.017940	0.000583	-0.016842	0.006320	-0.006974	0.030076
	-0.022097	0.002075	0.006030	-0.016990	0.006105	0.012398
	-0.014124	-0.015386	0.020936	0.010899	-0.013210	0.004677
	0.027598					
x659	0.034105	-0.013414	-0.010557	-0.004821	0.018143	0.002018
	-0.003388	-0.031600	0.026025	0.003367	-0.000904	-0.018010
	-0.018935	-0.026157	0.000875	-0.015536	-0.015140	-0.011392
	0.004500	-0.024557	0.009658	-0.031044	-0.001274	0.004158
	0.030215	0.003458	0.010915	-0.000730	0.008852	-0.020589
	0.012096					
y659	-0.015147	-0.003733	0.017523	0.001595	-0.015288	-0.000740
	-0.001739	0.005722	-0.006846	0.006331	-0.026556	-0.001921
	-0.009864	0.033475	-0.005845	-0.016513	0.001272	0.001336
	0.007624	-0.008627	0.021770	-0.002514	0.008715	-0.016714
	-0.000677	-0.023192	-0.028612	0.000723	0.006693	0.004154
	0.019372					
z659	0.005976	-0.027407	0.002206	0.038902	-0.000947	0.004506
	-0.022107	-0.017764	-0.016698	-0.014153	0.012241	0.016547
	-0.009816	-0.007070	-0.012933	0.005015	0.000451	0.021406
	-0.014199	-0.003151	0.017165	-0.019583	0.009823	0.002034
	-0.016513	-0.009063	0.029463	0.009812	-0.004216	-0.002203
	0.024123					

x660	0.027410	-0.011048	-0.007540	-0.001259	0.017991	0.004110
	-0.007773	-0.023780	0.022403	0.004656	-0.007855	-0.009302
	-0.018097	-0.027956	-0.002173	-0.010236	-0.011195	-0.003085
	0.006913	-0.024675	0.002073	-0.026467	-0.011592	0.005027
	0.022014	0.003599	0.017022	-0.000666	0.006043	-0.015735
	0.017824					
y660	-0.020642	-0.001675	0.020855	0.003658	-0.015772	-0.002616
	-0.007834	0.008219	-0.007410	0.008413	-0.018098	0.001175
	-0.007044	0.026877	-0.007821	-0.009486	0.000162	-0.000513
	0.010576	-0.005532	0.018467	-0.001092	0.008564	-0.015535
	-0.005577	-0.030590	-0.018572	0.002933	0.005178	0.006908
	0.009317					
z660	0.004988	-0.021495	-0.000158	0.032552	0.008819	0.001915
	-0.030714	-0.021784	-0.013784	-0.006787	0.022646	0.009124
	-0.005475	-0.016286	-0.008403	0.001586	0.003342	0.017870
	-0.013301	-0.012635	0.022730	-0.019014	0.003415	-0.003505
	-0.022181	-0.002193	0.023659	0.001702	-0.004147	-0.001311
	0.029508					
x661	0.018729	-0.008350	-0.003502	0.001961	0.019406	0.006069
	-0.013635	-0.017582	0.019757	0.006535	-0.013981	-0.000651
	-0.017317	-0.029761	-0.007655	-0.006132	-0.011482	0.007636
	0.006910	-0.021159	-0.004705	-0.020341	-0.016788	0.002479
	0.014395	0.000839	0.022331	-0.000150	0.003683	-0.012608
	0.022892					
y661	-0.025180	-0.000257	0.022234	0.003773	-0.014312	-0.006438
	-0.014812	0.008742	-0.010560	0.012192	-0.006932	0.000438
	-0.005027	0.017383	-0.008855	-0.004288	0.000391	-0.007546
	0.012745	-0.003046	0.018322	-0.000494	0.006361	-0.017145
	-0.010933	-0.033274	-0.011805	0.006537	0.004565	0.004396
	0.001682					
z661	0.002517	-0.013824	-0.002098	0.025678	0.018936	-0.000364
	-0.037419	-0.026228	-0.007918	-0.000691	0.029418	0.003086
	-0.001544	-0.025382	-0.003383	-0.000644	0.001967	0.012623
	-0.013794	-0.012431	0.024012	-0.020395	-0.000220	-0.012086
	-0.026298	0.005653	0.021677	-0.001776	-0.005523	-0.001011
	0.033747					
x662	0.009350	-0.006667	0.001338	0.003664	0.020963	0.008757
	-0.019309	-0.012293	0.016369	0.009096	-0.015026	0.001574
	-0.015497	-0.027736	-0.018478	-0.002525	-0.008743	0.009052
	0.011555	-0.016730	-0.007983	-0.014578	-0.014589	-0.005990
	0.012040	-0.004765	0.025270	-0.000378	0.005791	-0.016904
	0.019426					

y662	-0.029705	0.001052	0.021788	0.003124	-0.011349	-0.012108
	-0.021572	0.008045	-0.015091	0.016729	0.004368	0.000373
	-0.001716	0.004441	-0.008529	0.002539	-0.000362	-0.014826
	0.011255	0.003219	0.017119	0.000211	-0.002606	-0.018094
	-0.017979	-0.029530	-0.005501	0.011193	0.000438	0.001126
	0.000005					
z662	-0.000176	-0.005126	-0.003150	0.018524	0.027826	0.000721
	-0.040217	-0.028556	-0.002148	0.003105	0.035501	-0.006809
	0.004782	-0.027509	-0.002384	0.000959	0.001865	-0.001161
	-0.005892	-0.006295	0.024747	-0.025178	-0.001223	-0.022316
	-0.021667	0.016191	0.022889	-0.000561	-0.004814	-0.007249
	0.026929					
x663	0.000682	-0.005538	0.004309	0.003485	0.022166	0.009035
	-0.023206	-0.007544	0.013038	0.012075	-0.015893	0.002997
	-0.012519	-0.025849	-0.028185	0.001577	-0.007803	0.013015
	0.016799	-0.012499	-0.013472	-0.008225	-0.012439	-0.013544
	0.009375	-0.004487	0.024250	-0.001119	-0.001599	-0.014458
	0.022575					
y663	-0.032035	0.002903	0.023024	0.004321	-0.009840	-0.011724
	-0.026701	0.001004	-0.015797	0.010150	0.010876	-0.003298
	-0.000160	-0.001031	-0.009899	0.003884	-0.000569	-0.022524
	-0.004352	0.019065	0.013207	0.004301	-0.001492	-0.015991
	-0.026143	-0.036393	-0.011601	0.006518	0.021624	0.003940
	0.003045					
z663	-0.002915	0.002576	-0.003037	0.013863	0.033165	0.003600
	-0.040418	-0.030256	0.004653	-0.001314	0.035683	-0.012991
	0.010547	-0.022332	-0.006235	0.003406	-0.001376	-0.004570
	-0.005631	0.004210	0.018297	-0.024956	0.002065	-0.029044
	-0.019611	0.023204	0.017332	-0.005553	-0.001464	0.004281
	0.031291					
x664	-0.007175	-0.004411	0.007212	0.002290	0.023091	0.008787
	-0.024449	-0.003416	0.010109	0.017398	-0.017091	0.002024
	-0.010618	-0.021254	-0.036365	0.003080	-0.009451	0.011591
	0.016991	-0.007572	-0.020130	-0.003751	-0.012243	-0.017108
	0.007726	-0.004441	0.022164	-0.001925	-0.006918	-0.012845
	0.015878					
y664	-0.033449	0.005133	0.024171	0.005390	-0.008630	-0.011061
	-0.032221	-0.004512	-0.016444	0.005552	0.019069	-0.008204
	0.002445	-0.006792	-0.006406	0.003743	0.003528	-0.028966
	-0.014094	0.030922	0.009853	0.010222	0.003939	-0.015106
	-0.031609	-0.041375	-0.013704	-0.003217	0.037464	0.003126
	0.001883					

z664	-0.004850	0.009558	-0.001392	0.009342	0.037611	0.007371
	-0.038616	-0.029080	0.013114	-0.003994	0.034822	-0.018206
	0.013731	-0.013052	-0.008850	0.002418	-0.008559	-0.013105
	-0.004707	0.013497	0.012772	-0.027904	0.005853	-0.022439
	-0.013257	0.027159	0.011568	-0.011134	0.003158	0.015462
	0.020190					
x665	-0.015058	-0.002266	0.009612	0.001807	0.022945	0.006897
	-0.024681	-0.001414	0.006660	0.022770	-0.019282	-0.001178
	-0.009739	-0.018894	-0.040825	0.004956	-0.010986	0.016900
	0.011868	-0.002274	-0.027291	0.003331	-0.019877	-0.022642
	0.012094	-0.005199	0.017618	0.005665	-0.012334	-0.014390
	0.006874					
y665	-0.033747	0.007661	0.024960	0.006602	-0.007551	-0.010491
	-0.036880	-0.007909	-0.016310	0.002507	0.027859	-0.013112
	0.007467	-0.009146	-0.001503	0.000655	0.009227	-0.037747
	-0.018397	0.039792	0.009321	0.015359	0.008665	-0.011398
	-0.034839	-0.043975	-0.016735	-0.017206	0.044299	0.001303
	-0.003314					
z665	-0.007305	0.015975	0.000578	0.006350	0.040399	0.009371
	-0.036362	-0.027907	0.021282	-0.008215	0.032515	-0.021682
	0.014468	-0.007247	-0.009774	0.004513	-0.014443	-0.010884
	-0.004670	0.021145	0.010048	-0.029452	0.001947	-0.018706
	0.002781	0.025791	0.000073	-0.001830	0.008369	0.020942
	0.010090					
x666	-0.022104	0.000369	0.010690	0.002069	0.021186	0.004453
	-0.022673	-0.000867	0.001181	0.028429	-0.022154	-0.006682
	-0.009649	-0.015806	-0.041180	0.002723	-0.012800	0.020633
	0.005916	-0.000660	-0.034365	0.015948	-0.025451	-0.028812
	0.006902	-0.005251	0.011799	0.004208	-0.022592	-0.018709
	0.000828					
y666	-0.033044	0.009824	0.027117	0.008503	-0.005928	-0.009731
	-0.041730	-0.008416	-0.013780	-0.000052	0.036543	-0.012950
	0.013340	-0.009013	0.004160	-0.000766	0.012524	-0.046712
	-0.019934	0.047961	0.010201	0.015146	0.014952	-0.002617
	-0.030634	-0.043563	-0.020896	-0.028472	0.046193	-0.001610
	-0.008658					
z666	-0.008733	0.021035	-0.000110	0.002916	0.040489	0.012011
	-0.031524	-0.027066	0.028053	-0.016756	0.027810	-0.026401
	0.015403	-0.001045	-0.010455	0.001100	-0.015863	-0.011875
	-0.002624	0.023446	0.012165	-0.025821	0.005516	-0.013363
	0.002895	0.021861	-0.013351	-0.009615	0.013238	0.031838
	0.008183					

x667	-0.028159	0.003499	0.015603	0.002950	0.020410	0.008073
	-0.026326	0.001542	0.002279	0.023706	-0.018979	-0.008605
	-0.007005	-0.008880	-0.038524	-0.002159	-0.010255	0.016793
	0.001282	0.004048	-0.034497	0.021630	-0.020859	-0.026929
	0.005509	-0.012063	0.001658	-0.004441	-0.023684	-0.023226
	-0.003476					
y667	-0.031082	0.010620	0.026651	0.008898	-0.004546	-0.011655
	-0.041339	-0.008481	-0.012612	-0.000729	0.040694	-0.011678
	0.018852	-0.007701	0.009677	-0.002802	0.012509	-0.050060
	-0.019232	0.050210	0.011463	0.015500	0.016119	0.006624
	-0.025156	-0.038094	-0.020889	-0.037086	0.037445	-0.002531
	-0.013774					
z667	-0.010183	0.022260	-0.002435	-0.002200	0.039281	0.010782
	-0.023285	-0.023242	0.035694	-0.018367	0.017876	-0.030426
	0.012513	0.005034	-0.011787	0.002678	-0.017404	-0.008721
	-0.004623	0.023297	0.008982	-0.023374	0.001455	-0.010656
	0.008208	0.015987	-0.015622	-0.007519	0.015227	0.027760
	0.002622					
x668	-0.032623	0.007300	0.023862	0.004857	0.019662	0.016437
	-0.032029	0.003607	0.007001	0.009273	-0.009523	-0.004693
	-0.002614	0.000745	-0.033450	-0.008076	-0.005844	0.011845
	-0.001037	0.007602	-0.033048	0.021283	-0.009164	-0.018223
	0.006137	-0.021475	-0.012736	-0.017812	-0.024298	-0.020474
	-0.001856					
y668	-0.027644	0.008397	0.020971	0.007002	-0.004027	-0.019786
	-0.033072	-0.007453	-0.013830	0.005521	0.036668	-0.011269
	0.020828	-0.006134	0.013757	-0.001994	0.008021	-0.045341
	-0.020616	0.045523	0.009363	0.017979	0.008335	0.012130
	-0.017911	-0.026252	-0.012842	-0.035681	0.018325	-0.004221
	-0.020125					
z668	-0.011242	0.021051	-0.006080	-0.007213	0.036135	0.006404
	-0.009807	-0.020043	0.038410	-0.015557	0.006323	-0.030638
	0.007823	0.010266	-0.015686	0.007278	-0.019914	0.002844
	-0.003971	0.017425	-0.003083	-0.019618	-0.009220	-0.009863
	0.016630	0.008876	-0.010042	0.001766	0.002650	0.016061
	0.000981					
x669	-0.036118	0.010614	0.031094	0.007229	0.018616	0.020746
	-0.032563	0.006177	0.013334	-0.003807	-0.001414	-0.001433
	-0.005925	0.011291	-0.023654	-0.012447	-0.005310	0.012401
	-0.007926	0.010387	-0.030273	0.022374	-0.001511	-0.011016
	0.008159	-0.028155	-0.020807	-0.029975	-0.029111	-0.020327
	-0.003978					

y669	-0.022754	0.004092	0.012568	0.002560	-0.003198	-0.029752
	-0.022410	-0.005920	-0.016234	0.010273	0.029372	-0.010206
	0.023262	-0.003068	0.015441	-0.001584	0.002831	-0.040514
	-0.019128	0.040001	0.007390	0.018321	0.000913	0.017696
	-0.009324	-0.014242	-0.004958	-0.026917	0.000939	-0.000547
	-0.021531					
z669	-0.013369	0.020160	-0.009418	-0.011963	0.033139	0.000371
	0.004054	-0.014929	0.043383	-0.013147	-0.003491	-0.030351
	-0.001974	0.013915	-0.018545	0.012093	-0.020868	0.011551
	-0.004952	0.016960	-0.009399	-0.015296	-0.015354	-0.009375
	0.028874	-0.001499	0.000394	0.001913	0.001147	0.001680
	-0.006575					
x670	-0.038560	0.012839	0.036163	0.008065	0.017464	0.022200
	-0.030554	0.008025	0.018667	-0.016391	0.007636	0.001871
	-0.009455	0.023697	-0.010900	-0.013631	-0.005835	0.016811
	-0.015862	0.008577	-0.027473	0.023894	0.003447	-0.007496
	0.004203	-0.031607	-0.031018	-0.031487	-0.033508	-0.015661
	-0.005630					
y670	-0.016781	-0.001199	0.002298	-0.003043	-0.001475	-0.039797
	-0.011867	-0.003785	-0.017935	0.012502	0.020219	-0.007492
	0.025266	-0.000088	0.014255	-0.001042	-0.004199	-0.038216
	-0.014763	0.035450	0.006863	0.014948	-0.005010	0.022523
	0.002254	-0.004424	0.000892	-0.012167	-0.006582	0.008111
	-0.017239					
z670	-0.016683	0.019495	-0.013091	-0.018280	0.029563	-0.006126
	0.014852	-0.010382	0.045405	-0.009544	-0.008289	-0.032252
	-0.006272	0.017010	-0.020831	0.020273	-0.014749	0.019451
	-0.002751	0.010921	-0.014227	-0.007379	-0.014655	-0.015219
	0.032761	-0.010424	0.004936	0.001345	0.006202	-0.011548
	-0.015490					
x671	-0.038878	0.010828	0.034194	0.003528	0.019029	0.012182
	-0.022260	0.007899	0.018629	-0.021793	0.013885	0.006129
	-0.012659	0.035278	-0.008614	-0.007231	-0.007351	0.037007
	-0.018322	0.013176	-0.024038	0.018279	-0.001718	-0.009689
	-0.006975	-0.043892	-0.025106	-0.022578	-0.029499	-0.020232
	0.001302					
y671	-0.010590	-0.008103	-0.010555	-0.008938	0.001668	-0.050023
	-0.001584	-0.000741	-0.020468	0.015721	0.009699	-0.004790
	0.025891	0.000445	0.006605	0.006162	-0.012034	-0.030569
	-0.009113	0.034359	0.007344	0.008904	-0.017293	0.022824
	0.008655	-0.003930	0.010346	0.006685	0.004533	0.013127
	-0.008981					

z671	-0.019302	0.021656	-0.014827	-0.024180	0.023606	-0.014059
	0.025302	-0.009357	0.046814	-0.004086	-0.010395	-0.035358
	-0.002540	0.017876	-0.025802	0.025645	0.000328	0.020877
	0.004517	0.005873	-0.023239	-0.012542	-0.001468	-0.020081
	0.045521	-0.008158	-0.001066	-0.004782	-0.005979	-0.021504
	-0.009012					
x672	-0.038713	0.010450	0.025999	-0.008078	0.022932	-0.010099
	-0.012961	0.007918	0.017537	-0.010533	0.017815	0.012927
	-0.015270	0.037456	-0.000346	0.001957	-0.002931	0.034296
	-0.017651	0.016083	-0.017818	0.009556	0.001714	0.007156
	-0.013943	-0.031079	-0.022829	-0.023226	-0.028918	-0.040353
	-0.019542					
y672	-0.004652	-0.013255	-0.026530	-0.013725	0.004587	-0.057158
	0.009182	0.000419	-0.024102	0.021005	-0.003342	-0.005255
	0.028995	-0.002420	0.000773	0.012158	-0.017178	-0.027815
	-0.002327	0.031997	0.006273	0.000182	-0.025357	0.024640
	0.018492	-0.001453	0.008987	0.021717	0.012183	0.016441
	-0.000642					
z672	-0.021332	0.025038	-0.015329	-0.031095	0.015715	-0.025232
	0.032758	-0.006582	0.047277	0.002254	-0.013326	-0.036432
	-0.003839	0.019017	-0.028690	0.034182	0.003790	0.024995
	0.007818	0.001634	-0.027369	-0.022999	0.013428	-0.034059
	0.040577	-0.003784	0.004292	-0.006575	-0.005028	-0.018455
	-0.007732					
x673	-0.037757	0.010333	0.019806	-0.016700	0.025043	-0.025480
	-0.008305	0.008749	0.015572	-0.005735	0.019211	0.018256
	-0.016101	0.038409	0.007234	0.000758	0.003342	0.019651
	-0.014682	0.020669	-0.011915	0.001172	0.011822	0.026137
	-0.019870	-0.020567	-0.031263	-0.028232	-0.038472	-0.040760
	-0.023686					
y673	0.000112	-0.013364	-0.036256	-0.013643	0.005310	-0.055840
	0.015682	0.001291	-0.023484	0.021648	-0.012295	-0.009045
	0.027580	-0.006943	-0.002410	0.016684	-0.018292	-0.023967
	0.003660	0.027887	0.009336	-0.005907	-0.033587	0.023079
	0.023490	0.002418	0.005674	0.027812	0.019524	0.017082
	0.006362					
z673	-0.022005	0.029168	-0.018190	-0.036194	0.009929	-0.033609
	0.034992	-0.003109	0.043144	0.008875	-0.016740	-0.037113
	-0.002857	0.017375	-0.031694	0.036429	0.007667	0.028054
	0.017148	0.007053	-0.022623	-0.031635	0.012308	-0.036619
	0.012020	-0.002769	0.005378	-0.020561	-0.005172	-0.015253
	0.002560					

x674	-0.036509	0.009067	0.012572	-0.022931	0.025506	-0.037077
	-0.002958	0.008450	0.013235	-0.002669	0.014866	0.021451
	-0.018661	0.036748	0.008482	0.005769	0.004388	0.006183
	-0.016052	0.022074	-0.007333	-0.001709	0.018206	0.037044
	-0.020440	-0.018706	-0.029463	-0.022165	-0.030494	-0.031151
	-0.020824					
y674	0.004185	-0.009523	-0.041671	-0.010201	0.004953	-0.048134
	0.018280	0.003265	-0.019458	0.020107	-0.015934	-0.014584
	0.024404	-0.011896	-0.001441	0.017024	-0.013809	-0.019072
	0.010480	0.023214	0.015543	-0.011674	-0.041916	0.018906
	0.022040	0.008612	0.001902	0.023397	0.020841	0.011866
	0.008904					
z674	-0.021687	0.035254	-0.018009	-0.036809	0.003212	-0.031911
	0.033826	-0.004190	0.040569	0.006391	-0.022372	-0.034734
	-0.005639	0.012146	-0.031312	0.039926	0.004497	0.033189
	0.016082	0.005339	-0.017174	-0.022574	0.005151	-0.037132
	-0.009218	0.004734	0.000210	-0.021734	0.009965	0.000289
	0.011089					
x675	-0.034186	0.007650	0.005073	-0.026013	0.025373	-0.044634
	0.000661	0.008022	0.010268	0.000672	0.008743	0.021505
	-0.019974	0.032673	0.008273	0.009188	0.003618	-0.003903
	-0.017839	0.018435	-0.003619	-0.007654	0.025947	0.041158
	-0.015499	-0.018769	-0.021943	-0.016586	-0.027787	-0.024851
	-0.010311					
y675	0.007683	-0.002597	-0.043967	-0.005344	0.003209	-0.036353
	0.018759	0.005314	-0.012893	0.016625	-0.016006	-0.019054
	0.019890	-0.016466	0.002328	0.015400	-0.007371	-0.011697
	0.015516	0.016801	0.020732	-0.011980	-0.051703	0.013316
	0.014716	0.018489	-0.005873	0.014799	0.023309	0.005670
	0.005745					
z675	-0.019333	0.041582	-0.015773	-0.034943	-0.002707	-0.025066
	0.027902	-0.006278	0.038606	0.000033	-0.024858	-0.031934
	-0.004465	0.006648	-0.027148	0.037162	0.002972	0.039814
	0.015633	-0.002862	-0.012685	-0.018305	0.003969	-0.023308
	-0.010274	0.016011	-0.009563	-0.023507	-0.001740	0.003694
	0.023476					
x676	-0.031894	0.006667	-0.001700	-0.026746	0.025315	-0.049662
	0.002684	0.007403	0.007805	0.003495	0.001033	0.017704
	-0.022800	0.026907	0.008571	0.010480	0.000131	-0.012064
	-0.021584	0.018365	0.002327	-0.011706	0.030395	0.033680
	-0.013167	-0.016695	-0.006706	-0.012213	-0.020159	-0.021917
	-0.007598					

y676	0.011266	0.006189	-0.044271	-0.000400	0.000590	-0.021776
	0.018100	0.007568	-0.005315	0.011300	-0.013471	-0.020730
	0.016088	-0.020002	0.008102	0.012325	0.000399	-0.001478
	0.020006	0.007884	0.022653	-0.010052	-0.061575	0.013886
	0.009302	0.028858	-0.019651	0.004494	0.020441	-0.001902
	0.003206					
z676	-0.018206	0.045483	-0.011111	-0.033249	-0.005829	-0.019852
	0.022164	-0.008772	0.037130	-0.006887	-0.027153	-0.028100
	-0.003962	0.004222	-0.024819	0.032827	-0.000121	0.042682
	0.011003	0.005088	-0.002417	-0.011307	0.002552	-0.018205
	-0.012096	0.028499	-0.003464	-0.018988	-0.004743	0.002435
	0.016742					
x677	-0.028814	0.007660	-0.008010	-0.026241	0.024343	-0.051223
	0.003299	0.006501	0.006480	0.004837	-0.004569	0.011913
	-0.024113	0.020038	0.010417	0.009030	-0.004873	-0.015625
	-0.023722	0.013585	0.004843	-0.017085	0.029840	0.024208
	-0.010901	-0.011604	0.003218	-0.012071	-0.012883	-0.021774
	-0.004834					
y677	0.014664	0.014871	-0.042925	0.004066	-0.001959	-0.005833
	0.018033	0.010522	0.002348	0.004832	-0.010332	-0.018805
	0.012236	-0.021479	0.014866	0.010425	0.009025	0.010225
	0.023233	0.002640	0.023675	-0.005712	-0.070141	0.018305
	0.004275	0.034634	-0.033772	-0.002850	0.017956	-0.007046
	0.002503					
z677	-0.016968	0.046660	-0.005986	-0.034059	-0.008177	-0.016782
	0.015543	-0.012441	0.033472	-0.013946	-0.025952	-0.019800
	0.002516	0.006508	-0.025960	0.028935	-0.003092	0.041096
	0.009506	0.013417	0.001795	-0.009336	0.001636	-0.009764
	-0.011863	0.036793	-0.001079	-0.015510	-0.007529	-0.005264
	0.014998					
x678	-0.024610	0.011878	-0.013404	-0.023278	0.022052	-0.047172
	0.004592	0.008091	0.007288	0.004882	-0.008344	0.004815
	-0.027103	0.011508	0.019715	0.004316	-0.010128	-0.012039
	-0.023632	0.005184	0.009482	-0.019289	0.023371	0.017741
	-0.009329	-0.004916	0.007415	-0.013611	-0.004598	-0.013465
	-0.005262					
y678	0.017822	0.021194	-0.040673	0.006630	-0.003956	0.009890
	0.018467	0.014074	0.008613	-0.001722	-0.006539	-0.012011
	0.010177	-0.018835	0.019841	0.010412	0.019290	0.021108
	0.024948	0.002302	0.023105	0.000391	-0.074623	0.022929
	-0.002876	0.033462	-0.043189	-0.010079	0.018313	-0.009119
	0.006349					

z678	-0.015230	0.045471	-0.001400	-0.035456	-0.012118	-0.014166
	0.009736	-0.014518	0.026643	-0.022452	-0.024855	-0.012143
	0.007334	0.010080	-0.025469	0.025012	-0.007161	0.040218
	0.009626	0.020404	0.013055	-0.000618	0.006393	0.001688
	-0.013659	0.041186	0.000617	-0.006200	-0.000227	-0.000402
	0.009981					
x679	-0.018911	0.018742	-0.017662	-0.018320	0.019036	-0.036603
	0.006915	0.013256	0.010509	0.005340	-0.009350	-0.000791
	-0.034343	0.001433	0.038441	-0.004467	-0.011935	-0.002076
	-0.024338	-0.006928	0.014161	-0.019201	0.010230	0.011439
	-0.010613	-0.002609	0.011733	-0.022504	0.000878	-0.006948
	-0.008099					
y679	0.020600	0.023714	-0.038520	0.006847	-0.006082	0.023680
	0.019667	0.017109	0.012526	-0.007978	-0.003748	-0.002240
	0.009254	-0.012344	0.019902	0.013634	0.029808	0.030487
	0.025520	0.006489	0.021217	0.008005	-0.071361	0.023027
	-0.012174	0.023482	-0.046442	-0.014149	0.025215	-0.009343
	0.015422					
z679	-0.013874	0.040665	0.002129	-0.038679	-0.017319	-0.015175
	0.002463	-0.015997	0.018944	-0.031530	-0.024683	-0.004915
	0.003562	0.012418	-0.027175	0.022766	-0.009915	0.036505
	0.007719	0.024498	0.023431	0.011502	0.016540	0.009461
	-0.014141	0.027792	0.006884	0.001476	0.008873	-0.002870
	0.009428					
x680	-0.010466	0.026816	-0.021613	-0.010226	0.014484	-0.017902
	0.010603	0.020985	0.014716	0.009622	-0.008182	-0.002855
	-0.038606	-0.005695	0.065966	-0.017366	-0.011022	0.014008
	-0.026852	-0.018673	0.015366	-0.019184	-0.008737	0.004593
	-0.015483	0.000639	0.020590	-0.040727	0.000003	0.003023
	-0.017044					
y680	0.022388	0.021700	-0.036774	0.004292	-0.008637	0.032348
	0.020779	0.018274	0.014127	-0.015618	-0.002392	0.009525
	0.008820	-0.001630	0.010711	0.024830	0.038349	0.039574
	0.025497	0.015265	0.013301	0.016778	-0.057212	0.014845
	-0.018576	0.005144	-0.039195	-0.013159	0.043528	-0.007098
	0.032483					
z680	-0.012798	0.031000	0.005710	-0.039478	-0.024433	-0.017970
	-0.003266	-0.016322	0.007563	-0.039908	-0.024930	0.002132
	0.000798	0.013282	-0.041512	0.018487	-0.021657	0.032221
	0.008045	0.031219	0.020115	0.014404	0.038753	0.015108
	-0.006143	0.017581	0.009748	0.022824	0.015101	0.009360
	0.017175					

x681	-0.007960	0.026779	-0.023525	-0.013636	0.014320	-0.012966
	0.013146	0.025786	0.010114	0.001046	-0.007150	0.000786
	-0.032536	-0.010328	0.055721	-0.011770	-0.007171	0.014120
	-0.025103	-0.012485	0.018450	-0.012149	-0.011754	0.007837
	-0.015293	-0.003580	0.016348	-0.029125	0.011131	0.005568
	0.001961					
y681	0.019771	0.021885	-0.031376	0.003868	-0.005659	0.033734
	0.018368	0.016382	0.012709	-0.017564	-0.002983	0.012348
	0.011449	0.003110	0.009920	0.023447	0.043502	0.035786
	0.017963	0.018156	0.014259	0.024062	-0.052619	0.016638
	-0.022823	-0.002300	-0.031279	-0.011136	0.039783	-0.004345
	0.030060					
z681	-0.011460	0.027336	0.007711	-0.036798	-0.026220	-0.017816
	-0.008419	-0.017916	0.005408	-0.038614	-0.021174	0.004430
	-0.008381	0.016513	-0.033560	0.015648	-0.024915	0.030705
	0.004855	0.026357	0.018423	0.014616	0.041221	0.006746
	-0.005975	0.017474	0.017118	0.021019	0.019287	0.001588
	0.005319					
x682	-0.004331	0.026335	-0.025466	-0.016326	0.014145	-0.007422
	0.015054	0.029823	0.006058	-0.002561	-0.004845	0.005158
	-0.025064	-0.013412	0.049798	-0.007371	-0.002947	0.013877
	-0.024539	-0.008538	0.023313	-0.007151	-0.016229	0.012217
	-0.016908	-0.009731	0.010571	-0.020889	0.016196	0.013648
	0.019172					
y682	0.017324	0.021659	-0.025419	0.004014	-0.003242	0.034078
	0.015600	0.013500	0.012684	-0.017346	-0.003252	0.013644
	0.012419	0.007212	0.010220	0.021600	0.044500	0.032649
	0.011780	0.020165	0.014561	0.027009	-0.046995	0.016883
	-0.024544	-0.009556	-0.026082	-0.006334	0.034606	-0.000597
	0.028435					
z682	-0.009295	0.024514	0.009376	-0.032032	-0.028026	-0.017576
	-0.012522	-0.019199	0.002337	-0.036591	-0.017619	0.006190
	-0.015244	0.018388	-0.027537	0.011843	-0.030820	0.030915
	0.002338	0.019718	0.020057	0.010568	0.042985	0.003209
	-0.007089	0.017078	0.016044	0.020986	0.019831	0.001342
	0.000392					
x683	-0.001566	0.024980	-0.027145	-0.018913	0.015060	-0.002483
	0.017303	0.032467	0.002604	-0.003715	-0.004610	0.008060
	-0.018347	-0.016011	0.047060	-0.005562	0.001868	0.012526
	-0.022046	-0.002692	0.026785	-0.002422	-0.019495	0.013817
	-0.015518	-0.014153	0.011132	-0.018203	0.018039	0.019661
	0.030487					

y683	0.014695	0.021082	-0.019128	0.004862	-0.000885	0.033321
	0.012820	0.009602	0.013501	-0.016422	-0.004299	0.012872
	0.011525	0.010322	0.010592	0.018514	0.042394	0.030176
	0.007726	0.022382	0.013743	0.026552	-0.040002	0.014790
	-0.023198	-0.015764	-0.021122	-0.001474	0.027447	0.001995
	0.024866					
z683	-0.008854	0.021196	0.009907	-0.027976	-0.027897	-0.017156
	-0.014660	-0.020868	-0.002294	-0.034834	-0.018169	0.007567
	-0.018057	0.018318	-0.024017	0.003757	-0.034068	0.031794
	0.004836	0.017211	0.020123	0.007635	0.045407	-0.000037
	-0.005551	0.023408	0.018360	0.015708	0.022936	-0.002232
	-0.006470					
x684	0.001298	0.023717	-0.027660	-0.021167	0.015726	0.002021
	0.018159	0.034235	0.000587	-0.002329	-0.003799	0.010430
	-0.014590	-0.018647	0.047651	-0.002679	0.004253	0.011876
	-0.020541	0.000521	0.027296	-0.000162	-0.020513	0.014376
	-0.013580	-0.018247	0.011767	-0.016161	0.015174	0.025743
	0.038811					
y684	0.011901	0.020531	-0.012625	0.006619	0.000896	0.031866
	0.009258	0.005200	0.014940	-0.014066	-0.005736	0.012024
	0.009205	0.012672	0.011522	0.015399	0.036575	0.027798
	0.005344	0.022583	0.011378	0.024227	-0.031806	0.011452
	-0.021115	-0.019926	-0.017522	0.002820	0.019057	0.003266
	0.018430					
z684	-0.007261	0.018856	0.010549	-0.025244	-0.028938	-0.016643
	-0.018901	-0.022903	-0.004833	-0.035210	-0.015379	0.008974
	-0.023790	0.016726	-0.018483	0.000457	-0.036105	0.033702
	0.003469	0.014751	0.017382	0.004539	0.045403	-0.000877
	-0.002865	0.024873	0.021963	0.013509	0.024380	-0.005595
	-0.013434					
x685	0.003374	0.022806	-0.027506	-0.022404	0.017248	0.005711
	0.019067	0.036097	-0.001460	0.001514	-0.004355	0.012486
	-0.012199	-0.019940	0.049010	0.000006	0.004629	0.011642
	-0.017930	0.000234	0.028243	0.001442	-0.021842	0.013704
	-0.011503	-0.022304	0.013392	-0.014692	0.009560	0.028597
	0.041748					
y685	0.008907	0.020255	-0.006093	0.009085	0.002184	0.029494
	0.005831	0.000471	0.016402	-0.011023	-0.007518	0.010591
	0.006272	0.014964	0.011417	0.012287	0.029387	0.026532
	0.004099	0.021944	0.008229	0.021199	-0.024163	0.007769
	-0.018870	-0.023309	-0.013925	0.006367	0.010395	0.001516
	0.008242					

z685	-0.006731	0.017559	0.010278	-0.021722	-0.027986	-0.015948
	-0.022444	-0.022567	-0.008470	-0.034126	-0.013555	0.010513
	-0.025667	0.016841	-0.014554	0.000204	-0.035319	0.035622
	0.004866	0.008936	0.019531	0.001912	0.043768	-0.003125
	-0.004077	0.022746	0.022369	0.012502	0.026721	-0.006516
	-0.017458					
x686	0.004866	0.022279	-0.026773	-0.023159	0.018525	0.009283
	0.019778	0.035641	-0.002528	0.006532	-0.005561	0.013270
	-0.010408	-0.020349	0.049163	0.001397	0.002665	0.009159
	-0.014992	-0.002683	0.026169	0.001607	-0.019827	0.013330
	-0.010716	-0.021771	0.014997	-0.018676	0.003735	0.029520
	0.042666					
y686	0.005569	0.020293	0.000135	0.011910	0.002993	0.026654
	0.002230	-0.004953	0.017931	-0.007988	-0.009262	0.008845
	0.002909	0.017124	0.010939	0.009215	0.022023	0.025376
	0.003449	0.021137	0.003910	0.018205	-0.016361	0.004788
	-0.017706	-0.025109	-0.009530	0.007575	0.002706	-0.001328
	-0.003898					
z686	-0.005265	0.016479	0.009547	-0.019213	-0.026763	-0.014782
	-0.026016	-0.025516	-0.009822	-0.032403	-0.008736	0.009238
	-0.025858	0.015131	-0.013477	-0.001184	-0.031884	0.032322
	0.006027	0.005190	0.022793	-0.006412	0.043389	-0.002440
	-0.007393	0.020826	0.020390	0.004803	0.031182	-0.004042
	-0.016751					
x687	0.004859	0.022297	-0.025200	-0.023498	0.019377	0.012186
	0.020233	0.035622	-0.004114	0.010554	-0.009238	0.015846
	-0.007587	-0.017961	0.047538	0.000850	-0.001312	0.010626
	-0.013107	-0.006348	0.021497	0.007667	-0.019914	0.014725
	-0.009627	-0.017733	0.016788	-0.020714	-0.001788	0.027152
	0.034265					
y687	0.001996	0.020434	0.006201	0.014801	0.003301	0.023760
	-0.001752	-0.010441	0.019109	-0.005379	-0.010256	0.007375
	0.000029	0.020070	0.009872	0.006685	0.015151	0.024743
	0.003555	0.020643	-0.001206	0.015872	-0.009163	0.001555
	-0.016725	-0.026158	-0.004344	0.009011	-0.002974	-0.005853
	-0.017940					
z687	-0.004262	0.015415	0.008760	-0.017381	-0.025099	-0.014092
	-0.030660	-0.027552	-0.011930	-0.031728	-0.004064	0.010725
	-0.022887	0.015249	-0.014338	-0.003021	-0.027154	0.035896
	0.006221	0.002663	0.028047	-0.007552	0.039195	-0.003395
	-0.011506	0.020162	0.013567	0.001731	0.037756	0.001718
	-0.016921					

x688	0.003869	0.022752	-0.023105	-0.023292	0.019436	0.014796
	0.021639	0.035729	-0.006269	0.012959	-0.014402	0.019350
	-0.006556	-0.014739	0.044914	0.001463	-0.003933	0.011619
	-0.009607	-0.008971	0.013424	0.016195	-0.017702	0.016651
	-0.006296	-0.014729	0.025434	-0.020888	-0.003262	0.017803
	0.021971					
y688	-0.001767	0.020389	0.011888	0.017230	0.003345	0.020603
	-0.006413	-0.016644	0.019694	-0.003298	-0.009453	0.005646
	-0.002467	0.022752	0.008695	0.005461	0.010417	0.023831
	0.004830	0.020482	-0.007062	0.013406	-0.001934	-0.002054
	-0.015744	-0.027924	0.002421	0.010238	-0.006184	-0.012337
	-0.032234					
z688	-0.002505	0.013947	0.008290	-0.016033	-0.022784	-0.014455
	-0.033742	-0.029892	-0.013094	-0.031591	0.002527	0.011697
	-0.022418	0.011799	-0.015290	-0.003162	-0.016167	0.037792
	0.006846	0.003760	0.033385	-0.010618	0.036210	-0.004948
	-0.006720	0.009013	0.011867	0.003908	0.043208	0.003774
	-0.014785					
x689	0.001179	0.023846	-0.020740	-0.022238	0.019443	0.018268
	0.021374	0.036673	-0.010333	0.012788	-0.021810	0.025285
	-0.002314	-0.009371	0.041221	0.001918	-0.006117	0.013126
	-0.005544	-0.011536	0.000755	0.025426	-0.017053	0.017937
	-0.008257	-0.006919	0.031000	-0.023109	0.000579	0.003895
	0.014046					
y689	-0.005446	0.019480	0.016824	0.019148	0.003310	0.017374
	-0.012577	-0.024338	0.019096	-0.001343	-0.005506	0.003744
	-0.004597	0.025653	0.008489	0.005365	0.007933	0.021631
	0.007716	0.019614	-0.011579	0.010577	0.005285	-0.007579
	-0.014898	-0.027517	0.007710	0.011248	-0.006115	-0.020310
	-0.044956					
z689	-0.001244	0.012803	0.007604	-0.012636	-0.019204	-0.014273
	-0.038642	-0.029795	-0.014370	-0.029363	0.006027	0.014390
	-0.017474	0.008750	-0.014830	-0.006076	-0.006395	0.036673
	0.006165	0.006221	0.037776	-0.015989	0.029600	-0.011362
	-0.008386	0.003239	-0.005099	0.003367	0.047342	0.011679
	-0.002107					
x690	-0.001151	0.024191	-0.016723	-0.020967	0.018975	0.016624
	0.016623	0.035302	-0.008679	0.011310	-0.023950	0.030203
	-0.004869	-0.006135	0.038135	0.000570	-0.009217	0.011188
	0.001472	-0.016273	-0.009293	0.026829	-0.015098	0.016485
	-0.004906	-0.012963	0.033885	-0.019054	0.003050	-0.004390
	0.013042					

y690	-0.006821	0.017504	0.017202	0.021844	0.003132	0.015341
	-0.020620	-0.030421	0.023937	-0.002035	-0.001233	0.008407
	-0.003087	0.028507	0.008957	0.004727	0.008063	0.020995
	0.003940	0.013708	-0.002189	0.005269	0.002121	-0.007855
	-0.012194	-0.023908	-0.003875	0.013701	0.001969	-0.011876
	-0.037804					
z690	-0.000232	0.013730	0.005715	-0.012701	-0.014542	-0.013003
	-0.036438	-0.030214	-0.015542	-0.026173	0.009278	0.015465
	-0.011032	0.002802	-0.009969	-0.005171	0.001456	0.035395
	0.001915	0.004503	0.036692	-0.013000	0.024491	-0.012415
	-0.005650	0.001901	-0.011471	0.002581	0.044739	0.017236
	-0.004146					
x691	-0.003713	0.023483	-0.013444	-0.019588	0.019968	0.015534
	0.011486	0.033321	-0.006861	0.009974	-0.025374	0.035607
	-0.004709	-0.001615	0.034911	-0.002226	-0.013016	0.008971
	0.006900	-0.019066	-0.014347	0.028237	-0.013284	0.013359
	-0.001394	-0.015305	0.035749	-0.017818	0.003373	-0.011333
	0.015145					
y691	-0.008000	0.015506	0.017239	0.024051	0.002787	0.013655
	-0.028155	-0.036287	0.026629	-0.003464	0.004198	0.012232
	-0.002596	0.031273	0.010129	0.004945	0.009251	0.019976
	0.002149	0.006812	0.006373	0.001574	-0.000988	-0.009448
	-0.009643	-0.019123	-0.014809	0.016514	0.011889	-0.007035
	-0.028816					
z691	0.000742	0.014241	0.003464	-0.012090	-0.009850	-0.012666
	-0.033449	-0.028888	-0.018195	-0.022127	0.010595	0.017684
	-0.002699	0.001001	-0.007329	-0.006874	0.006663	0.034376
	-0.000636	0.006283	0.038006	-0.006931	0.021043	-0.016066
	-0.003670	0.005054	-0.015735	0.000538	0.041335	0.020967
	-0.001788					
x692	-0.006433	0.022017	-0.010636	-0.019115	0.021446	0.014570
	0.005702	0.029393	-0.003825	0.007340	-0.023796	0.039250
	-0.004697	0.001007	0.032025	-0.003239	-0.014750	0.007067
	0.010074	-0.021210	-0.016957	0.028451	-0.011962	0.008969
	0.000599	-0.018217	0.034826	-0.019002	0.003222	-0.015058
	0.016813					
y692	-0.009093	0.014010	0.017114	0.025556	0.002310	0.012019
	-0.034519	-0.040981	0.027879	-0.004985	0.009453	0.015743
	-0.003265	0.033574	0.011484	0.006372	0.010706	0.018714
	0.002586	-0.000658	0.012443	-0.000621	-0.003439	-0.009839
	-0.005862	-0.016358	-0.023820	0.021147	0.022168	-0.005369
	-0.019038					

z692	0.001256	0.014724	0.001111	-0.013129	-0.005705	-0.013331
	-0.030179	-0.027340	-0.019745	-0.018593	0.012962	0.018689
	0.004022	-0.000761	-0.004627	-0.005917	0.011631	0.033786
	-0.001779	0.007070	0.037113	-0.002857	0.018849	-0.019242
	-0.002243	0.003868	-0.018996	-0.002238	0.036051	0.024407
	0.000628					
x693	-0.008855	0.020708	-0.008110	-0.018713	0.023611	0.013827
	-0.000390	0.025494	-0.000653	0.004418	-0.021082	0.043523
	-0.005474	0.002299	0.028870	-0.004884	-0.016241	0.004811
	0.011838	-0.025038	-0.016985	0.028397	-0.012379	0.005581
	0.000020	-0.020579	0.031346	-0.016662	0.003737	-0.018763
	0.019579					
y693	-0.009953	0.013145	0.016996	0.026858	0.001634	0.010404
	-0.039624	-0.044365	0.027995	-0.005997	0.013981	0.018832
	-0.004599	0.036479	0.012677	0.008131	0.012154	0.017080
	0.004126	-0.007135	0.016080	-0.002279	-0.005227	-0.009666
	-0.002476	-0.013350	-0.030298	0.024639	0.031854	-0.006866
	-0.010634					
z693	0.002252	0.015516	-0.001051	-0.014207	-0.001555	-0.013952
	-0.026450	-0.024115	-0.021978	-0.014645	0.014160	0.020686
	0.009689	-0.002630	-0.002423	-0.006321	0.014899	0.032612
	-0.003047	0.006637	0.035551	0.000223	0.015869	-0.020627
	-0.003747	0.004024	-0.021394	0.000673	0.031174	0.024443
	0.002571					
x694	-0.010919	0.019677	-0.005274	-0.018556	0.025058	0.012781
	-0.006788	0.020560	0.003249	0.001939	-0.017426	0.047869
	-0.006653	0.005412	0.025626	-0.005949	-0.018957	0.003527
	0.013027	-0.030871	-0.016516	0.028413	-0.013123	0.003534
	0.001254	-0.023540	0.023647	-0.013603	0.007309	-0.018274
	0.021044					
y694	-0.010853	0.012962	0.017017	0.028066	0.001102	0.009031
	-0.043270	-0.046036	0.027402	-0.006122	0.017159	0.021472
	-0.006503	0.039840	0.014386	0.010433	0.013783	0.014550
	0.005327	-0.011239	0.017681	-0.003943	-0.007440	-0.009688
	0.000818	-0.010791	-0.033006	0.026737	0.037218	-0.010476
	-0.004234					
z694	0.003571	0.016952	-0.002700	-0.015881	0.001093	-0.015028
	-0.022253	-0.020943	-0.023797	-0.009317	0.014276	0.022701
	0.014972	-0.000991	0.000301	-0.005957	0.015023	0.031784
	-0.004471	0.005002	0.032139	0.002485	0.012798	-0.020856
	-0.002076	0.002096	-0.025205	0.004674	0.026534	0.027652
	0.001062					

x695	-0.013758	0.018101	-0.002405	-0.018603	0.027305	0.010421
	-0.012761	0.016571	0.007493	-0.001802	-0.015215	0.050546
	-0.010071	0.008243	0.022146	-0.010008	-0.021351	0.002134
	0.014874	-0.032480	-0.013385	0.030670	-0.015809	0.002879
	0.002635	-0.025294	0.022522	-0.012206	0.010780	-0.022467
	0.019956					
y695	-0.011379	0.013452	0.017160	0.029036	0.000393	0.008753
	-0.045238	-0.047098	0.026561	-0.004417	0.019487	0.024198
	-0.008464	0.043301	0.015759	0.014644	0.015016	0.010961
	0.003852	-0.013764	0.017071	-0.007179	-0.008129	-0.009690
	0.002476	-0.006392	-0.035930	0.028468	0.036243	-0.013771
	0.001352					
z695	0.004108	0.017677	-0.004315	-0.018342	0.004378	-0.017618
	-0.016606	-0.016294	-0.025545	-0.004826	0.011379	0.022369
	0.018708	-0.000107	0.002702	-0.008392	0.013267	0.029854
	-0.005634	0.009606	0.030453	0.005177	0.010130	-0.019452
	-0.000552	0.003524	-0.019720	0.006019	0.018817	0.022405
	-0.002580					
x696	-0.016560	0.017184	0.000510	-0.017927	0.029242	0.008731
	-0.019221	0.011648	0.012003	-0.005714	-0.010820	0.052844
	-0.014598	0.012607	0.018038	-0.011519	-0.022957	0.001257
	0.016677	-0.037910	-0.011566	0.032323	-0.017894	0.002148
	0.002645	-0.027289	0.014726	-0.011214	0.018245	-0.020330
	0.021964					
y696	-0.011564	0.014217	0.017073	0.029580	0.000073	0.009125
	-0.045227	-0.047094	0.025626	-0.000644	0.020150	0.025913
	-0.009340	0.046724	0.017361	0.019821	0.016818	0.007240
	-0.001259	-0.012103	0.017568	-0.011351	-0.008717	-0.010519
	0.003265	-0.000660	-0.035050	0.029678	0.027934	-0.015950
	0.001453					
z696	0.004718	0.018724	-0.006644	-0.020491	0.007968	-0.019613
	-0.010633	-0.011847	-0.027736	-0.000186	0.009878	0.021034
	0.023500	0.002252	0.004181	-0.008244	0.012574	0.029151
	-0.006391	0.011686	0.027617	0.005821	0.009139	-0.019139
	0.001029	0.003683	-0.017274	0.006236	0.017094	0.024001
	-0.005145					
x697	-0.019692	0.016213	0.003572	-0.017006	0.031270	0.006968
	-0.025468	0.005731	0.016953	-0.009180	-0.006408	0.055619
	-0.020833	0.015833	0.013136	-0.011662	-0.024467	-0.000093
	0.017030	-0.043357	-0.009956	0.035092	-0.018820	-0.000128
	0.001028	-0.027879	0.005406	-0.011390	0.020363	-0.017209
	0.024816					

y697	-0.011276	0.014899	0.016525	0.029288	0.000271	0.009987
	-0.043553	-0.046545	0.024515	0.005337	0.019600	0.026423
	-0.008787	0.049654	0.018497	0.026032	0.018038	0.002397
	-0.010031	-0.007442	0.020624	-0.016906	-0.007298	-0.010852
	0.001869	0.005886	-0.031032	0.029319	0.011328	-0.017456
	-0.002588					
z697	0.004937	0.018672	-0.009873	-0.023463	0.012318	-0.022576
	-0.003786	-0.008231	-0.030130	0.004422	0.007373	0.021525
	0.028261	0.002015	0.004929	-0.008372	0.009991	0.026743
	-0.006968	0.014617	0.023922	0.006642	0.011734	-0.022193
	0.003565	0.003782	-0.013765	0.004187	0.011042	0.022459
	-0.003375					
x698	-0.022283	0.015884	0.006955	-0.015123	0.032263	0.005947
	-0.031882	-0.001603	0.021674	-0.010452	-0.001189	0.057805
	-0.026931	0.021560	0.008956	-0.008722	-0.026883	-0.000751
	0.013282	-0.048701	-0.004018	0.036519	-0.020234	-0.003433
	0.000987	-0.023203	-0.006568	-0.012768	0.012207	-0.014142
	0.024812					
y698	-0.010150	0.015025	0.014313	0.027351	0.001219	0.010718
	-0.039608	-0.044717	0.022220	0.013022	0.017782	0.026681
	-0.004886	0.051618	0.020946	0.033082	0.017298	-0.003660
	-0.022150	0.003526	0.027937	-0.024433	-0.004803	-0.010610
	0.001251	0.015814	-0.024565	0.027060	-0.014583	-0.019065
	-0.009028					
z698	0.005331	0.018152	-0.013735	-0.028279	0.017717	-0.028280
	0.002802	-0.005497	-0.032223	0.008333	0.005296	0.023224
	0.032460	0.002048	0.007251	-0.009528	0.003912	0.023203
	-0.003952	0.016268	0.020197	0.009724	0.012646	-0.023659
	0.009466	0.001346	-0.011062	0.002151	0.004340	0.020545
	0.003649					
x699	-0.022773	0.015796	0.008758	-0.010480	0.026940	0.006792
	-0.032664	-0.003372	0.016934	-0.008516	0.001300	0.058283
	-0.027326	0.030351	0.004571	-0.004217	-0.024078	0.003945
	0.004302	-0.036731	0.012845	0.032711	-0.018503	-0.012456
	0.001351	-0.002721	-0.022756	-0.014950	-0.007900	-0.019353
	0.019506					
y699	-0.008662	0.013665	0.009747	0.024136	0.004032	0.006077
	-0.027124	-0.035326	0.011711	0.018165	0.013550	0.030303
	0.002162	0.050189	0.021126	0.029996	0.006950	0.004685
	-0.017802	0.019705	0.036735	-0.027122	-0.009986	-0.005578
	-0.003101	0.031116	-0.023677	0.037348	-0.028309	-0.019063
	0.000121					

z699	0.003635	0.015339	-0.017147	-0.036455	0.024605	-0.040060
	0.009373	-0.003375	-0.030619	0.007179	0.006557	0.023866
	0.031544	-0.000238	0.007901	-0.007571	-0.000315	0.021399
	0.005635	0.011353	0.008278	0.022771	0.014295	-0.017565
	0.010830	-0.012183	0.000938	0.013943	0.016418	0.021348
	0.004657					
x700	-0.023214	0.015038	0.009486	-0.008423	0.023340	0.004595
	-0.033301	-0.007406	0.013457	-0.005099	0.001541	0.058309
	-0.026543	0.037751	0.000133	0.002397	-0.022484	0.003593
	-0.003338	-0.029741	0.028385	0.026429	-0.012129	-0.017731
	-0.000110	0.013276	-0.043681	-0.020851	-0.028979	-0.021173
	0.010357					
y700	-0.006388	0.009976	0.001300	0.019366	0.008489	-0.001591
	-0.015408	-0.027354	-0.001048	0.024936	0.005020	0.032963
	0.014203	0.047687	0.024194	0.027326	-0.003930	0.006329
	-0.019645	0.028512	0.035660	-0.028608	-0.009395	-0.002993
	0.000930	0.034993	-0.018056	0.044074	-0.035930	-0.019591
	0.003113					
z700	0.001660	0.013026	-0.019199	-0.043671	0.029628	-0.050047
	0.016338	-0.002321	-0.028810	0.007327	0.009297	0.027341
	0.031570	0.000042	0.006958	-0.009008	-0.004134	0.019187
	0.019207	0.008653	-0.000158	0.029836	0.012939	-0.008496
	0.001463	-0.015764	0.006838	0.023182	0.023212	0.017814
	0.016987					
x701	-0.023835	0.012573	0.007652	-0.009582	0.022970	-0.003826
	-0.035235	-0.012139	0.011043	-0.000056	-0.000469	0.055836
	-0.027057	0.041817	-0.004435	0.008403	-0.019756	-0.004061
	-0.008544	-0.025864	0.039562	0.024609	-0.003144	-0.023193
	0.002881	0.018478	-0.060188	-0.026522	-0.038436	-0.015204
	-0.005283					
y701	-0.003858	0.004967	-0.008138	0.013665	0.012570	-0.011178
	-0.005142	-0.021027	-0.013249	0.031206	-0.004679	0.035325
	0.026838	0.045292	0.025590	0.026138	-0.014516	0.004770
	-0.021661	0.033377	0.026959	-0.027147	-0.007317	-0.000935
	0.005239	0.030190	-0.011602	0.048681	-0.032503	-0.021235
	0.006920					
z701	-0.000077	0.011249	-0.019898	-0.048027	0.034225	-0.056947
	0.018584	-0.001481	-0.026364	0.008944	0.012618	0.033618
	0.030256	0.000797	0.009737	-0.012594	-0.005246	0.014423
	0.031251	0.006931	-0.001588	0.035275	0.012785	-0.008786
	-0.004422	-0.014847	0.014353	0.026481	0.028957	0.015787
	0.030338					

x702	-0.023807	0.008530	0.002516	-0.014700	0.023077	-0.018632
	-0.036222	-0.017475	0.009379	0.005420	-0.001558	0.048552
	-0.029016	0.041249	-0.012740	0.014601	-0.014814	-0.012313
	-0.011333	-0.021825	0.045873	0.020543	0.003183	-0.023419
	0.009611	0.014550	-0.068713	-0.028219	-0.034451	-0.009568
	-0.018309					
y702	-0.001816	-0.000008	-0.016882	0.008397	0.016087	-0.019514
	0.004091	-0.015386	-0.023826	0.036059	-0.014330	0.036367
	0.037370	0.040686	0.024659	0.025074	-0.024181	0.002136
	-0.024087	0.037297	0.016843	-0.026560	-0.005535	0.000125
	0.008031	0.021730	-0.006143	0.051125	-0.022927	-0.021577
	0.015304					
z702	-0.000495	0.010704	-0.019707	-0.049442	0.034384	-0.060142
	0.020214	-0.001471	-0.022102	0.011006	0.018248	0.039322
	0.028830	-0.000744	0.012438	-0.014159	-0.001319	0.016049
	0.035814	0.002552	-0.001799	0.036174	0.014149	-0.004707
	-0.006121	-0.011023	0.018561	0.028239	0.029371	0.012048
	0.031880					
x703	-0.024031	0.003732	-0.003900	-0.021771	0.024662	-0.035664
	-0.036672	-0.021365	0.007716	0.009843	-0.002603	0.038016
	-0.031693	0.038975	-0.023539	0.019494	-0.010223	-0.021706
	-0.007967	-0.013070	0.050034	0.014552	0.002987	-0.018861
	0.014428	0.007287	-0.065237	-0.026888	-0.023419	-0.004366
	-0.020392					
y703	-0.000044	-0.004018	-0.023513	0.004426	0.018912	-0.024684
	0.011467	-0.010657	-0.031414	0.038823	-0.022156	0.037094
	0.045197	0.033826	0.023291	0.024690	-0.033531	-0.000608
	-0.027531	0.038476	0.007477	-0.025611	-0.001461	0.000200
	0.010607	0.013727	-0.005811	0.051013	-0.016003	-0.021041
	0.020298					
z703	-0.000386	0.011059	-0.018148	-0.048940	0.033573	-0.059653
	0.020421	-0.002520	-0.017693	0.011458	0.024447	0.044758
	0.027992	-0.002257	0.013550	-0.014127	0.001066	0.018613
	0.038589	-0.003493	-0.002878	0.037608	0.014954	-0.001191
	-0.006153	-0.007699	0.018818	0.028380	0.022215	0.010308
	0.031858					
x704	-0.024305	-0.001501	-0.010013	-0.029198	0.027577	-0.052974
	-0.036384	-0.023754	0.006793	0.012110	-0.004522	0.025612
	-0.034134	0.037591	-0.035271	0.024182	-0.005573	-0.032306
	-0.002718	-0.000273	0.053141	0.010430	-0.001271	-0.013314
	0.016170	0.001481	-0.055682	-0.024041	-0.013417	0.000806
	-0.019884					

y704	0.001995	-0.007430	-0.029024	0.000763	0.021432	-0.028743
	0.018368	-0.006949	-0.037831	0.041419	-0.028190	0.037575
	0.052040	0.026570	0.021745	0.026572	-0.042287	-0.004251
	-0.032621	0.035915	-0.001980	-0.023216	0.005254	-0.002354
	0.011613	0.007122	-0.010895	0.050082	-0.010818	-0.020529
	0.023818					
z704	-0.000220	0.011818	-0.014850	-0.047535	0.032564	-0.057534
	0.020650	-0.003746	-0.012657	0.010166	0.031303	0.049311
	0.027350	-0.000948	0.013901	-0.014596	0.004062	0.018137
	0.038965	-0.010084	-0.003768	0.040391	0.015373	-0.003339
	-0.005474	-0.004168	0.014611	0.027485	0.009252	0.006805
	0.029515					
x705	-0.024585	-0.008199	-0.017264	-0.039433	0.031158	-0.073536
	-0.036048	-0.025393	0.006272	0.014249	-0.005880	0.009465
	-0.036930	0.035429	-0.050231	0.029347	-0.001858	-0.045205
	0.005895	0.014176	0.053287	0.006942	-0.005135	-0.001244
	0.017724	-0.004377	-0.042022	-0.022080	0.000487	0.008892
	-0.018165					
y705	0.003968	-0.011156	-0.035424	-0.005203	0.024895	-0.033075
	0.026437	-0.002473	-0.044814	0.045392	-0.034576	0.037599
	0.059539	0.017167	0.022923	0.029179	-0.053991	-0.007629
	-0.038950	0.030454	-0.014403	-0.022909	0.014819	-0.002713
	0.011204	-0.001765	-0.017208	0.046699	-0.002733	-0.018484
	0.026231					
z705	-0.000307	0.013516	-0.010048	-0.046477	0.029975	-0.054392
	0.019680	-0.005116	-0.003547	0.010162	0.041806	0.053356
	0.026676	0.003372	0.013932	-0.017286	0.005415	0.016779
	0.038204	-0.020227	-0.006819	0.043201	0.016965	-0.003652
	-0.005627	0.000763	0.004074	0.024310	-0.004134	0.005120
	0.025407					
x706	-0.025838	-0.018048	-0.026219	-0.051437	0.037081	-0.098979
	-0.035336	-0.024068	0.004710	0.011948	-0.009811	-0.012933
	-0.040488	0.033345	-0.066000	0.033558	0.005636	-0.057718
	0.023097	0.037078	0.050653	0.012170	-0.014156	0.020274
	0.017387	-0.008397	-0.002068	-0.018202	0.015150	0.013321
	-0.026619					
y706	0.005469	-0.015120	-0.042634	-0.014936	0.028420	-0.038780
	0.037302	-0.000507	-0.054281	0.047476	-0.038282	0.039586
	0.065757	0.008950	0.030075	0.037773	-0.064389	-0.007174
	-0.045804	0.022031	-0.035593	-0.021347	0.026860	-0.000342
	0.012976	-0.014983	-0.013055	0.047651	0.004313	-0.017688
	0.027126					

z706	-0.002659	0.014988	-0.003438	-0.044228	0.025385	-0.050635
	0.018076	-0.006733	0.006872	0.006230	0.056619	0.059597
	0.021475	0.011564	0.011040	-0.024483	0.013657	0.016115
	0.041886	-0.021703	-0.009852	0.052479	0.011810	-0.003559
	-0.002078	0.000035	0.003317	0.024387	-0.027173	-0.001636
	0.001792					
x707	-0.025902	-0.026327	-0.033180	-0.059193	0.043794	-0.111670
	-0.034382	-0.025149	0.004396	0.010170	-0.010424	-0.036976
	-0.044045	0.017725	-0.072767	0.038229	0.010004	-0.076314
	0.027150	0.041685	0.042425	0.019569	-0.013660	0.041808
	0.006920	-0.008286	0.035486	-0.015285	0.023153	0.017908
	-0.023416					
y707	0.006140	-0.017847	-0.046548	-0.025328	0.027444	-0.043313
	0.047887	0.002006	-0.061902	0.046592	-0.037816	0.046826
	0.071037	0.010048	0.044083	0.047418	-0.062138	0.009813
	-0.052335	0.015017	-0.054368	-0.010703	0.031450	0.003533
	0.023398	-0.008823	0.003480	0.044646	0.002765	-0.026013
	0.025111					
z707	-0.002096	0.017961	0.005884	-0.035020	0.020403	-0.036088
	0.015561	-0.006603	0.014839	0.004620	0.078313	0.069742
	0.017734	0.016649	0.016961	-0.029872	0.020072	0.020937
	0.039364	-0.037987	-0.008113	0.060526	0.005914	-0.006600
	0.007491	0.003358	-0.028243	0.018898	-0.062365	-0.003105
	-0.013510					
x708	-0.028999	0.012643	-0.009093	0.009229	0.010916	0.003210
	0.019201	-0.014481	-0.032349	-0.017861	-0.014491	-0.002237
	0.002692	0.027784	-0.019813	-0.011928	0.027997	-0.018942
	0.001942	-0.032548	0.076975	-0.010243	-0.029660	0.001466
	0.046265	0.047471	-0.012706	0.005418	0.019965	-0.012651
	-0.010971					
y708	-0.005495	0.004115	-0.009098	0.008930	-0.007502	-0.019988
	0.006327	0.009525	0.000940	-0.000108	-0.016159	0.004735
	-0.000537	0.026554	-0.007291	-0.015858	-0.001751	0.013490
	0.008290	0.017943	0.025208	-0.011798	0.034342	-0.040480
	-0.014342	-0.005494	0.011372	-0.028873	-0.008198	0.001172
	-0.034875					
z708	0.006572	0.024663	0.012423	-0.003190	-0.005151	0.004860
	0.015154	0.019979	-0.012388	0.009567	0.001832	-0.037973
	-0.013984	0.037800	0.032819	0.017584	-0.024918	-0.005486
	0.039547	0.018438	0.046530	0.016302	0.007692	-0.004452
	0.017106	0.028179	0.013305	-0.031727	0.014329	0.040288
	0.035508					

x709	-0.028460	0.016633	-0.002128	0.007816	0.010294	-0.001484
	0.024434	-0.022033	-0.022605	-0.013146	-0.007235	-0.001478
	0.007896	0.030778	-0.028666	0.000928	0.027734	-0.023597
	-0.000949	-0.021142	0.067643	-0.010290	-0.034753	-0.007177
	0.039087	0.042089	0.002443	0.006792	0.046408	-0.015091
	-0.021912					
y709	-0.007089	0.003550	-0.005827	0.012792	-0.009184	-0.014416
	0.005993	0.014449	0.005251	-0.005960	-0.013057	0.007367
	0.006363	0.026533	-0.013169	-0.017305	0.003612	0.005858
	0.009459	0.014745	0.023469	-0.003601	0.020541	-0.029616
	-0.016890	-0.010364	0.017187	-0.004154	0.011847	-0.002585
	-0.040736					
z709	0.006779	0.023109	0.014862	-0.001126	-0.001488	0.012603
	0.013973	0.021007	-0.011248	0.008217	-0.000432	-0.025559
	-0.006572	0.029007	0.041721	0.019467	-0.031343	-0.002911
	0.041495	0.021505	0.038930	0.012335	0.013653	0.001908
	0.007865	0.021546	0.000496	-0.034132	-0.005107	0.028014
	0.042125					
x710	-0.032342	0.018355	0.003370	0.010108	0.010095	-0.003107
	0.023513	-0.023928	-0.030987	-0.007321	-0.016166	0.001463
	0.019616	0.042081	-0.020047	0.007806	0.018530	-0.019047
	0.009916	-0.018612	0.057347	-0.012960	-0.022473	-0.010262
	0.020825	0.038215	0.003724	0.002460	0.054966	-0.016857
	-0.035976					
y710	-0.009409	0.003607	-0.003048	0.017153	-0.006942	-0.009000
	0.004184	0.017855	0.002667	-0.011537	-0.017740	0.010247
	0.010562	0.029397	-0.012193	-0.018638	0.003337	-0.000338
	0.013556	0.011212	0.018792	-0.000327	0.016668	-0.019041
	-0.026315	-0.012268	0.024139	0.010209	0.015126	-0.008331
	-0.041541					
z710	0.009840	0.021983	0.017344	-0.002557	0.001094	0.020581
	0.016960	0.024939	-0.000901	0.005436	0.005568	-0.018450
	0.002758	0.010569	0.040494	0.025412	-0.039062	-0.008620
	0.034371	0.022025	0.036563	0.014071	0.007643	0.003398
	-0.007702	0.013629	-0.003851	-0.028859	-0.007244	0.018121
	0.053221					
x711	-0.031494	0.019195	0.010753	0.012245	0.012999	-0.004008
	0.026300	-0.022563	-0.037324	-0.007606	-0.019221	0.002029
	0.030435	0.038553	-0.016728	0.009826	0.018791	-0.020260
	0.009648	-0.015936	0.046200	-0.007932	-0.027484	-0.008539
	-0.003938	0.032806	0.003541	0.007536	0.046090	-0.017736
	-0.031236					

y711	-0.010582	0.003760	0.000636	0.019953	-0.005578	-0.002723
	0.003819	0.020484	-0.000020	-0.012860	-0.017851	0.015569
	0.013053	0.027293	-0.011481	-0.020360	0.004738	-0.006029
	0.013586	0.008813	0.016697	-0.000344	0.013851	-0.013392
	-0.028825	-0.014546	0.027636	0.017400	0.007966	-0.013970
	-0.044164					
z711	0.011187	0.022721	0.018830	-0.003008	0.000573	0.026935
	0.017206	0.021627	0.004184	0.002311	0.010276	-0.010172
	0.009316	0.005083	0.037662	0.034779	-0.045290	-0.007881
	0.031407	0.025739	0.040349	0.009383	0.007132	0.006247
	-0.014847	0.003311	-0.002862	-0.017322	0.002228	0.008468
	0.055551					
x712	-0.030512	0.020111	0.018811	0.014408	0.012035	0.001165
	0.032047	-0.020315	-0.040422	-0.013315	-0.024913	0.001858
	0.039504	0.032328	-0.015566	0.015308	0.016512	-0.012341
	0.013399	-0.010473	0.039308	-0.002951	-0.027467	-0.010725
	-0.030750	0.019534	0.006454	0.013538	0.031025	-0.023362
	-0.029192					
y712	-0.012053	0.004831	0.004558	0.022006	-0.002789	0.002809
	0.002800	0.022703	-0.001177	-0.011780	-0.013920	0.021179
	0.011659	0.022914	-0.013169	-0.018914	0.004766	-0.009369
	0.011994	0.005848	0.013559	-0.003238	0.015713	-0.010215
	-0.026622	-0.009946	0.034316	0.013467	0.000507	-0.015155
	-0.041571					
z712	0.012649	0.023772	0.019378	-0.004277	0.002236	0.028962
	0.015839	0.016704	0.006011	0.004021	0.014763	-0.003453
	0.017601	0.002238	0.037129	0.042766	-0.051568	-0.014014
	0.028617	0.027406	0.045998	0.004917	-0.000599	0.011543
	-0.023378	-0.000680	-0.002240	0.000548	0.014178	0.002458
	0.068497					
x713	-0.029473	0.022367	0.027922	0.015402	0.008807	0.009133
	0.035213	-0.017947	-0.040815	-0.016757	-0.022170	0.003372
	0.050532	0.027033	-0.014619	0.031969	0.008351	-0.010422
	0.017181	-0.004980	0.036192	-0.012549	-0.037832	-0.013986
	-0.058150	0.003615	0.001898	0.019678	0.045200	-0.018744
	-0.001514					
y713	-0.012969	0.007658	0.007253	0.021902	-0.001027	0.008936
	0.000345	0.023692	-0.002493	-0.009741	-0.005061	0.027004
	0.008218	0.018339	-0.010505	-0.013164	0.002056	-0.017535
	0.008816	0.000702	0.011817	-0.001058	0.018606	-0.008993
	-0.022983	-0.004032	0.030125	0.009935	0.002237	-0.007977
	-0.053722					

z713	0.013027	0.024140	0.019146	-0.004983	0.006218	0.028184
	0.015832	0.012789	0.005693	0.004668	0.013993	0.000432
	0.022846	-0.000530	0.037479	0.042438	-0.052856	-0.016871
	0.026891	0.027236	0.053465	0.011382	-0.001559	0.017087
	-0.027325	0.003194	0.005736	0.018571	0.001526	-0.005131
	0.059417					
x714	-0.028046	0.026054	0.036215	0.014642	0.005974	0.019098
	0.037594	-0.013508	-0.043696	-0.020167	-0.019693	0.011785
	0.055742	0.020817	-0.019426	0.046485	-0.004915	-0.004056
	0.014621	-0.004256	0.035716	-0.016088	-0.053968	-0.012683
	-0.084888	-0.009112	-0.007205	0.030410	0.058229	-0.011114
	0.024089					
y714	-0.013311	0.010085	0.010030	0.021107	0.001521	0.013175
	-0.004629	0.022066	-0.003612	-0.007166	0.002006	0.032378
	0.005649	0.013284	-0.006066	-0.008393	-0.000070	-0.023925
	0.004208	-0.004801	0.009282	0.004376	0.022389	-0.008579
	-0.021038	0.006172	0.023591	0.011991	0.004918	0.002232
	-0.063989					
z714	0.011157	0.022501	0.018257	-0.004749	0.011252	0.023792
	0.015139	0.010524	0.007213	0.004948	0.009894	-0.003803
	0.027623	-0.004661	0.043348	0.037332	-0.049367	-0.021489
	0.029602	0.036847	0.063775	0.016966	-0.002759	0.018916
	-0.026328	0.009750	0.026391	0.033246	-0.016481	-0.012276
	0.039860					
x715	-0.029545	0.025224	0.043098	0.012271	0.007446	0.022913
	0.033031	-0.010141	-0.042843	-0.015414	-0.013827	0.012827
	0.052988	0.003524	-0.006062	0.047657	-0.022951	-0.011884
	-0.001144	-0.006001	0.037542	-0.007342	-0.055784	-0.012751
	-0.093107	0.024255	-0.019521	0.049386	0.043218	0.023902
	0.011443					
y715	-0.016406	0.010894	0.012689	0.019525	0.005957	0.016087
	-0.008419	0.027158	-0.006780	-0.011776	0.001688	0.041151
	0.001574	0.006604	-0.006700	-0.013170	0.000442	-0.027593
	0.006106	0.009291	0.015150	0.008990	0.012501	-0.005950
	-0.023807	0.005476	0.041113	0.006040	0.004227	-0.003023
	-0.085598					
z715	0.010916	0.026967	0.018417	-0.001877	0.012469	0.027829
	0.017957	0.004292	0.007830	0.000674	0.007583	0.001026
	0.045534	-0.003641	0.037828	0.054394	-0.053016	-0.020085
	0.039794	0.034773	0.070049	0.011427	-0.007224	0.024714
	-0.046952	-0.008472	0.024704	0.057348	-0.011005	-0.030486
	0.070860					

x716	-0.038417	0.022315	0.050324	0.010778	0.009538	0.028880
	0.037165	0.009268	-0.039105	-0.028773	-0.018051	0.010936
	0.058955	-0.006846	-0.001225	0.046298	-0.021892	-0.013579
	0.008814	0.032370	0.076051	0.002247	-0.091520	-0.007795
	-0.122048	0.001645	0.014352	0.063294	0.034083	0.007408
	-0.002369					
y716	-0.019641	0.010001	0.014506	0.017542	0.012644	0.016061
	-0.014769	0.036387	-0.010262	-0.010835	0.002203	0.046050
	-0.002358	-0.005004	-0.006275	-0.025440	0.005163	-0.033283
	0.007777	0.026434	0.013227	0.013146	-0.002452	-0.008847
	-0.006182	0.000313	0.074142	-0.009488	-0.004028	-0.008382
	-0.131036					
z716	0.008022	0.030067	0.017443	0.001726	0.017529	0.027785
	0.018944	-0.003048	0.007442	-0.000295	0.001097	0.002494
	0.061736	-0.008181	0.039447	0.066539	-0.061339	-0.019623
	0.040445	0.040429	0.079966	0.009938	-0.011024	0.030137
	-0.063165	-0.004000	0.029558	0.091696	-0.010611	-0.025941
	0.086060					
x717	-0.045406	0.018209	0.057285	0.011270	0.012369	0.027268
	0.040416	0.023517	-0.032083	-0.033130	-0.028216	0.009820
	0.053798	-0.017813	-0.000172	0.048975	-0.032285	-0.013627
	0.012255	0.073941	0.099653	0.000563	-0.114779	-0.008105
	-0.127176	-0.002149	0.048115	0.059068	0.023653	-0.016305
	-0.012250					
y717	-0.017014	0.011530	0.014849	0.015373	0.012901	0.020594
	-0.020502	0.035159	-0.014054	-0.012778	0.006728	0.053785
	-0.003034	-0.009145	-0.006926	-0.027325	0.005150	-0.040011
	0.003997	0.013464	0.001807	0.014256	0.004066	-0.009015
	-0.006134	0.002178	0.065214	-0.018489	-0.010406	-0.005054
	-0.134954					
z717	0.003539	0.030620	0.015126	0.004409	0.025329	0.026626
	0.018625	-0.001587	0.005981	-0.005105	-0.009651	0.002782
	0.075708	-0.010618	0.042396	0.062112	-0.055137	-0.019577
	0.045994	0.053975	0.092363	0.018570	-0.027755	0.034533
	-0.075708	-0.018488	0.061621	0.117134	-0.008131	-0.027609
	0.065899					

Phylogenetic mapping

Dataset: PC scores, CovMatrix, BE_All, Procrustes coordinates

Data type: PC scores

Phylogeny: Phylogeny

Method: weighted squared-change parsimony

The tree is rooted.

Tree length: 0.10571257

Permutation test against the null hypothesis of no phylogenetic signal

Number of randomization rounds: 10000

P-value: <.0001

Canonical Variate Analysis: CVA ...BE_All

Dataset: BE_All

Classification criterion: environment

Groups	Observations	
1.	coastal	15
2.	continental	8
3.	marine	7
4.	polar	2

Variation among groups, scaled by the inverse of the within-group variation

	Eigenvalues	% Variance	Cumulative %
1.	2.86408711	51.315	51.315
2.	1.57043558	28.137	79.452

3.	1.14689561	20.548	100.000

Mahalanobis distances among groups:

	coastal	continental	marine
continental	3.2857		
marine	3.1473	4.1787	
polar	5.1982	4.6536	6.1549

P-values from permutation tests (10000 permutation rounds) for Mahalanobis distances among groups:

	coastal	continental	marine
continental	0.0001		
marine	0.0010	0.0003	
polar	<.0001	0.0842	0.0251

Procrustes distances among groups:

	coastal	continental	marine
continental	0.0634		
marine	0.0642	0.0960	
polar	0.1145	0.0963	0.1307

P-values from permutation tests (10000 permutation rounds) for Procrustes distances among groups:

	coastal	continental	marine
continental	0.0199		

marine	0.0439	0.0003	
polar	0.0161	0.0645	0.0096

Canonical coefficients:

	CV1	CV2	CV3
x1	-1.8647	1.9329	1.5132
y1	2.1312	0.7253	-0.5788
z1	0.7526	1.4203	-0.9144
x2	-2.3774	0.7134	-2.8843
y2	3.3833	-1.9699	2.6434
z2	0.7780	1.1064	2.2453
x3	-2.4518	0.4339	-3.0226
y3	3.5214	-1.8344	2.5107
z3	0.6669	0.7251	2.1032
x4	-2.4577	-0.0924	-3.0972
y4	3.5454	-2.1450	2.4447
z4	0.7068	0.6793	1.9926
x5	-2.7297	-0.7882	-3.7962
y5	3.5129	-2.2842	2.5185
z5	0.8452	0.3843	2.0951
x6	-2.5862	-1.2841	-3.7817
y6	3.5685	-2.5977	2.4762
z6	1.0491	0.2418	2.3298
x7	-2.2651	-1.1429	-3.1364
y7	3.6149	-2.5057	2.3467
z7	1.2108	0.1291	2.4272
x8	-2.1557	-1.7104	-2.4772

y8	3.5157	-2.6323	2.4022
z8	1.3539	0.0392	2.6184
x9	-2.0066	-2.2103	-1.9133
y9	3.4488	-2.6716	2.4126
z9	1.4663	-0.1210	2.8117
x10	-2.0847	-2.5212	-1.4908
y10	3.3098	-2.6036	2.1953
z10	1.6301	0.0134	3.0336
x11	-2.0575	-2.6696	-1.2120
y11	3.3803	-2.4287	1.6992
z11	1.9088	-0.1942	2.9201
x12	-1.6161	-2.3552	-0.1598
y12	3.0458	-2.7338	1.1017
z12	1.6347	-0.9416	2.5204
x13	-0.6982	-1.4649	1.6923
y13	3.1946	-1.8705	1.7180
z13	1.6126	-1.0503	2.5371
x14	-0.0449	-1.5434	2.5354
y14	3.0213	-1.9512	1.4406
z14	1.5575	-1.6859	2.3025
x15	0.1445	-1.2458	2.2818
y15	2.8195	-1.7027	1.0743
z15	1.2743	-2.1983	1.6359
x16	0.3473	-0.3906	2.5525
y16	2.6282	-1.2939	0.6849
z16	1.2303	-2.0429	1.5433
x17	0.5435	0.2477	2.5940
y17	2.4100	-0.9495	0.4746

z17	1.1651	-2.2201	1.7563
x18	0.7134	-0.0693	2.3431
y18	2.2664	-1.0127	0.2958
z18	0.8762	-3.5783	1.3983
x19	0.7016	0.2600	2.1059
y19	2.1721	-0.9530	0.0752
z19	0.6012	-3.1545	1.5861
x20	0.4340	-0.4910	1.5706
y20	2.1111	-0.5497	0.0070
z20	0.7143	-1.4560	2.1708
x21	0.3067	-1.2582	1.8912
y21	2.0091	0.0884	-0.2525
z21	1.0123	0.2219	1.9570
x22	-0.4360	-1.6421	1.0253
y22	2.1260	0.3787	-0.2355
z22	1.0435	1.2342	1.8647
x23	-0.4313	-1.4733	0.5890
y23	1.9900	0.4161	-0.2149
z23	1.0603	1.9424	1.4854
x24	-0.5912	-1.2167	0.1705
y24	1.9944	0.2236	-0.0491
z24	0.9517	2.1356	1.1233
x25	-1.0696	-1.0386	-0.4584
y25	2.7119	0.2066	0.6308
z25	0.5661	1.8197	1.0906
x26	-1.0951	-1.0580	-0.9034
y26	2.7956	0.1400	1.6964
z26	-0.0357	1.3983	1.1209

x27	-0.8082	-0.1434	-0.7014
y27	2.3085	-1.3656	1.2069
z27	-0.2958	0.9029	1.5610
x28	-0.7226	0.6721	-0.9316
y28	2.0982	-2.9902	0.7754
z28	-0.1736	0.4116	2.2249
x29	0.0065	0.7138	-1.3744
y29	3.1947	-2.0228	2.8450
z29	-0.3692	0.3645	2.9373
x30	0.5033	-1.1431	-0.7846
y30	3.9145	-0.9135	2.5681
z30	-0.2756	1.1982	2.5220
x31	0.5900	-1.8670	-0.3940
y31	3.5303	0.1708	1.6448
z31	-0.5786	1.8130	1.7765
x32	0.3019	-1.9307	0.7495
y32	3.0775	0.9997	1.0233
z32	-0.9581	2.6094	1.5641
x33	-0.2392	-2.1655	1.4540
y33	2.6509	1.0050	0.6116
z33	-1.6603	2.5818	0.5901
x34	-0.6814	-2.4642	1.7362
y34	2.3509	0.4302	0.2524
z34	-2.3280	1.9447	-0.2741
x35	-0.7377	-2.3067	1.3514
y35	1.8872	-0.3030	-0.3923
z35	-2.4597	1.3786	-1.2962
x36	-0.6485	-1.6560	0.8056

y36	1.2441	-1.1600	-1.1302
z36	-2.5246	0.8837	-1.8688
x37	-0.2839	-0.0171	1.0803
y37	0.7825	-1.9034	-1.4401
z37	-1.6685	1.2283	-0.9898
x38	0.2042	2.5125	1.1155
y38	0.4417	-3.0167	-1.4579
z38	-0.9660	1.2542	-0.6939
x39	-0.3266	3.2606	0.5871
y39	0.7397	-3.2232	-1.4054
z39	-0.6017	1.2830	0.0220
x40	-0.6525	3.3048	0.3402
y40	1.1361	-3.4112	-1.3377
z40	-0.3526	1.5357	0.5948
x41	-0.8644	3.0720	0.0437
y41	1.6998	-3.3005	-0.9653
z41	-0.2859	1.5980	0.9536
x42	-1.0745	3.1131	-0.0804
y42	2.2173	-3.1643	-0.3698
z42	-0.2434	1.3834	0.8615
x43	-1.3633	3.0585	-0.4894
y43	2.6132	-3.0273	0.0842
z43	-0.1296	1.1534	1.0665
x44	-1.7816	2.5777	-1.2512
y44	2.9596	-2.6623	0.5939
z44	0.1132	0.8981	1.5774
x45	-2.1105	1.9322	-2.0408
y45	3.2253	-2.3026	1.2753

z45	0.4082	1.0780	1.8154
x46	-2.2537	1.2381	-2.5156
y46	3.4056	-2.1093	1.8770
z46	0.7376	1.1415	1.9019
x47	-1.7892	1.7151	-1.7018
y47	3.6024	2.6746	2.4131
z47	-2.2833	0.9975	-2.5483
x48	-2.0679	1.4716	-2.0092
y48	3.4579	1.8623	2.2000
z48	-1.9183	1.0041	-2.2498
x49	-2.2813	1.3111	-2.1819
y49	3.7330	1.7177	2.5363
z49	-1.5165	1.2011	-1.6496
x50	-2.5603	0.9610	-2.2845
y50	3.7761	0.7181	2.5240
z50	-0.9941	1.1537	-1.2659
x51	-2.8166	0.6555	-2.5084
y51	4.1966	0.0166	2.6533
z51	-0.3692	1.0925	-0.7700
x52	-3.0045	0.2765	-2.4834
y52	4.2659	-0.5926	2.7280
z52	0.2152	1.0517	-0.1625
x53	-3.0788	-0.2399	-2.3705
y53	4.6663	-1.2257	3.1618
z53	0.8358	0.9467	0.5112
x54	-3.1529	-0.6663	-2.2866
y54	4.9803	-1.6285	3.4912
z54	1.3050	0.9197	1.1120

x55	-3.2301	-1.3539	-2.1496
y55	5.2523	-2.3856	3.5939
z55	1.8882	0.5494	1.6537
x56	-3.1984	-1.8484	-1.7669
y56	5.2763	-2.7692	3.7489
z56	2.2691	0.3093	2.1884
x57	-3.1743	-1.8205	-1.5732
y57	4.8775	-2.4629	3.3223
z57	2.3169	0.2581	2.3035
x58	-3.0820	-1.9954	-1.1303
y58	4.6886	-2.7519	3.5270
z58	2.4716	-0.0449	2.8468
x59	-2.7210	-1.7827	-0.6705
y59	4.8230	-2.5054	3.5408
z59	2.7917	-0.0082	3.2270
x60	-2.5309	-1.6589	-0.2941
y60	4.4691	-2.5648	3.1951
z60	2.7051	-0.3380	3.2278
x61	-2.3447	-1.4806	0.1919
y61	4.4067	-2.5719	3.1385
z61	2.8623	-0.7407	3.5284
x62	-2.1997	-1.4721	0.5412
y62	4.1957	-2.9746	2.7237
z62	2.8187	-1.5179	3.2980
x63	-1.8267	-1.1623	1.2054
y63	4.0027	-3.2975	2.5438
z63	2.6584	-2.3434	3.0992
x64	-1.6881	-0.7121	1.5789

y64	3.7219	-2.7416	2.6178
z64	2.5459	-2.2348	3.4525
x65	-1.4547	-0.4091	2.1764
y65	3.4693	-2.3252	2.4684
z65	2.3623	-2.2471	3.4187
x66	-1.3292	-0.4956	2.4092
y66	3.2707	-1.9161	1.9571
z66	2.1235	-2.2435	2.8845
x67	-1.1437	-0.3419	2.4983
y67	3.0127	-1.6664	1.2782
z67	1.8950	-2.3615	2.3017
x68	-1.0978	-0.4216	2.4991
y68	2.7716	-1.5741	0.9121
z68	1.6631	-2.6385	2.2040
x69	-1.0975	-0.5056	2.4754
y69	2.6385	-0.9827	0.8705
z69	1.6601	-1.9327	2.5902
x70	-1.1968	-0.4425	2.2950
y70	2.7412	-0.5598	0.9029
z70	2.2223	-1.8966	3.3334
x71	-1.1752	-0.5452	2.4441
y71	2.6245	-0.5245	0.7638
z71	2.2902	-2.0866	4.2688
x72	-1.0519	-0.1821	2.6423
y72	2.3989	-0.3440	0.4002
z72	1.9379	-1.4433	3.7185
x73	-0.7382	-0.6416	3.0470
y73	2.2391	-0.4173	0.1992

z73	2.1723	-2.2036	4.0298
x74	-0.8030	-0.9928	2.5043
y74	2.2764	0.1442	0.4013
z74	2.1357	-1.3906	4.4061
x75	-1.1771	-0.7956	1.9806
y75	2.2420	0.3154	0.3182
z75	2.2301	-0.5632	4.1942
x76	-1.3195	-0.5598	1.9946
y76	2.2211	0.4004	0.0360
z76	2.2213	0.6052	4.0265
x77	-1.3526	-0.0448	2.1977
y77	2.1500	0.5299	-0.2838
z77	2.0142	0.4958	3.3193
x78	-1.4012	0.6864	2.3279
y78	2.0578	0.6884	-0.5034
z78	1.7791	0.2537	2.7861
x79	-1.3447	1.0668	2.4468
y79	2.0667	0.8096	-0.6452
z79	1.4551	0.3612	2.1377
x80	-1.3883	1.3178	2.3300
y80	1.9857	0.5095	-0.9722
z80	1.3945	1.1004	1.6727
x81	-1.4265	1.8558	2.0787
y81	1.9359	0.4692	-1.2968
z81	1.3967	0.9873	1.2454
x82	-1.5438	2.3000	1.6601
y82	1.7674	0.2574	-1.6578
z82	1.4662	1.0376	0.5749

x83	-1.5127	3.1429	1.0809
y83	1.5655	0.1858	-2.2236
z83	1.6639	0.8372	0.1959
x84	-1.6807	-1.2394	-1.3006
y84	-3.5576	-1.4851	-1.5970
z84	1.6959	2.7735	0.6082
x85	-1.5709	-0.7325	-1.4603
y85	-3.2202	-1.5408	-1.5205
z85	1.5522	2.6313	0.8496
x86	-1.4814	-0.4298	-1.4555
y86	-3.0034	-1.4066	-1.4892
z86	1.2784	2.5344	0.6797
x87	-1.3361	-0.5672	-1.1761
y87	-2.8303	-1.1912	-1.4605
z87	1.0402	2.8134	0.2158
x88	-0.9970	-0.3795	-0.6142
y88	-2.6811	-1.0094	-1.3221
z88	0.6479	2.4514	-0.4860
x89	-1.0861	-0.4264	-0.8632
y89	-2.6406	-1.0259	-1.4421
z89	0.7240	2.3907	-0.3561
x90	-1.0175	-0.4238	-0.5935
y90	-2.5693	-0.9005	-1.5061
z90	0.6273	2.3619	-0.6049
x91	-0.8536	-0.3100	-0.1967
y91	-2.5061	-0.5930	-1.4893
z91	0.5922	2.2635	-0.8184
x92	-0.8807	-0.6229	-0.4069

y92	-2.5957	-0.5953	-1.9814
z92	0.7170	2.3538	-0.5597
x93	-0.8844	-1.2207	-0.4299
y93	-2.2375	-0.3438	-1.7533
z93	0.6669	2.4274	-0.5682
x94	-0.7968	-1.4865	-0.3255
y94	-1.8231	0.0524	-1.3728
z94	0.6610	2.3806	-0.5412
x95	-0.8894	-1.3692	-0.2418
y95	-1.6180	0.2200	-1.2993
z95	0.8595	2.3090	-0.3533
x96	-0.7528	-1.2603	0.1429
y96	-1.2907	0.6253	-1.0604
z96	0.9213	2.1199	-0.3175
x97	-0.9197	-0.9965	0.3536
y97	-1.0609	0.8221	-0.9216
z97	0.9254	1.9288	-0.4201
x98	-1.1783	-0.4344	0.0014
y98	-0.8513	1.1917	-0.8698
z98	0.9815	1.7442	-0.5163
x99	-0.7871	0.5379	0.5248
y99	-0.3755	2.0475	-0.3052
z99	1.1020	1.5289	-0.5363
x100	-0.8132	0.3242	0.5596
y100	-0.1085	1.9419	-0.0909
z100	1.1059	1.2638	-0.6899
x101	-1.0117	0.3496	0.4163
y101	0.1289	2.0001	0.1272

z101	1.0688	0.9365	-0.9639
x102	-1.0851	0.3899	0.5065
y102	0.5907	1.8038	0.6373
z102	0.9262	0.9232	-1.2865
x103	-0.9055	0.4551	0.7198
y103	1.2730	1.7698	1.1088
z103	0.6123	0.6922	-1.4471
x104	-0.9207	0.7806	1.2322
y104	1.7046	1.9353	1.6111
z104	0.2719	0.3992	-1.4666
x105	-1.0942	1.1760	1.1872
y105	1.7521	1.9864	1.2656
z105	0.0092	0.6327	-1.6199
x106	-1.2074	1.4567	1.2794
y106	1.4197	1.2361	0.0275
z106	-0.1530	0.8588	-1.5170
x107	-1.3327	1.5462	1.2999
y107	1.4925	0.9992	0.1514
z107	-0.2222	0.9902	-1.2544
x108	-1.2884	1.6908	1.3901
y108	1.4857	1.0958	0.6848
z108	-0.2796	1.2494	-1.1258
x109	-1.3650	1.7132	1.3018
y109	1.4457	0.6152	0.5920
z109	-0.2311	1.1607	-1.1851
x110	-1.3977	1.6277	1.4029
y110	1.1769	1.0817	0.1697
z110	-0.2255	0.9996	-1.3294

x111	-1.5374	1.7346	1.4663
y111	0.8058	1.0134	0.0091
z111	0.0539	1.0378	-1.5089
x112	-1.4523	1.6342	1.6289
y112	0.3814	0.2425	-0.0686
z112	-0.2113	1.1820	-1.3841
x113	-1.2002	1.7477	2.1626
y113	-0.1194	-0.0778	-0.6432
z113	-0.5055	1.3620	-0.9938
x114	-1.0855	1.9628	1.6723
y114	-0.5689	-1.0307	-0.9945
z114	-0.7587	1.3581	-0.8608
x115	-1.3369	2.3758	0.7029
y115	-0.6761	-0.7410	-0.4715
z115	-0.9562	1.5537	-1.0142
x116	-1.6350	2.9885	0.0949
y116	-0.7310	-0.4706	-0.0923
z116	-1.1768	1.3746	-1.6835
x117	-1.9929	3.7106	-0.3097
y117	-0.6155	-0.9483	-0.0145
z117	-1.5056	1.9840	-2.2345
x118	-2.0579	4.0167	-0.6793
y118	-0.5202	0.0339	0.8560
z118	-1.8119	2.0987	-3.1277
x119	-1.8182	4.1685	-0.5531
y119	-1.0314	0.9298	1.1163
z119	-1.3296	3.0705	-2.1396
x120	-1.5040	4.2624	-0.1087

y120	-1.8860	1.5865	1.1476
z120	-0.7849	3.0452	-1.8978
x121	-1.5957	3.5620	-0.2120
y121	-2.6104	1.2958	0.1598
z121	-1.2615	2.7525	-2.6061
x122	-1.5170	3.0368	-0.1657
y122	-2.8612	1.2101	-0.1971
z122	-1.1705	2.3943	-2.2944
x123	-1.6052	2.2169	-0.4312
y123	-3.0320	0.7175	-0.5580
z123	-0.6218	1.9299	-1.4724
x124	-1.5875	1.6218	-0.4907
y124	-3.2793	0.0972	-1.0655
z124	-0.3953	1.3055	-1.5987
x125	-1.5139	0.9884	-0.5212
y125	-3.5249	-0.4394	-1.4677
z125	-0.2603	0.8726	-1.7246
x126	-1.3566	0.4001	-0.6226
y126	-3.6677	-0.9006	-1.7475
z126	0.1353	0.9283	-1.8716
x127	-1.2019	-0.2122	-0.7094
y127	-3.7380	-1.1535	-1.8525
z127	0.6396	1.4428	-1.6705
x128	-1.0984	-0.7655	-0.6487
y128	-3.7976	-1.2060	-1.8643
z128	1.1416	2.7267	-0.8525
x129	0.8270	3.3865	-1.5368
y129	-0.3670	0.6314	0.0299

z129	-0.6750	2.2031	-0.5473
x130	0.3898	2.8462	-1.6107
y130	-0.7234	0.2392	-0.2879
z130	-0.8221	2.5578	-0.7772
x131	0.3298	2.2039	-1.4882
y131	-1.3033	-0.1209	-0.7202
z131	-0.8092	2.8456	-0.8792
x132	0.1605	2.0037	-1.3381
y132	-1.9044	-0.9441	-1.3221
z132	-0.7551	2.6207	-0.9374
x133	-0.3056	1.7842	-1.5957
y133	-2.1212	-1.2767	-1.4107
z133	-0.5642	1.9187	-0.7426
x134	-0.7506	1.3910	-1.9719
y134	-2.2291	-0.8133	-1.2687
z134	-0.4638	1.4717	-0.6991
x135	-0.8656	1.1606	-2.1389
y135	-2.5257	-0.3156	-1.3505
z135	-0.0934	1.2379	-0.5456
x136	-0.8970	0.9008	-1.8984
y136	-2.8919	0.0591	-2.0056
z136	0.2069	0.9338	-0.2346
x137	-1.0763	0.7878	-1.7726
y137	-2.8705	0.2835	-2.3627
z137	0.3495	0.7458	0.0379
x138	-0.8100	-0.0099	-1.1607
y138	-3.1336	0.8722	-2.7157
z138	0.3025	0.9600	-0.1774

x139	-0.5905	-0.4945	-0.7413
y139	-3.0353	1.2493	-2.4538
z139	0.2293	0.9473	-0.5890
x140	-0.5650	-0.6255	-0.4709
y140	-2.9942	1.4006	-2.3913
z140	0.3467	0.9104	-0.7352
x141	-0.9053	-1.6809	-1.0512
y141	-2.7317	1.8512	-1.7221
z141	0.3474	0.8708	-1.1496
x142	-0.9277	-2.2367	-1.1207
y142	-2.5858	2.0875	-1.3937
z142	0.5656	0.9125	-1.1928
x143	-0.7941	-1.4842	-0.7967
y143	-2.4823	1.9498	-1.3386
z143	0.8502	0.9681	-1.0077
x144	-0.8538	-0.9463	-0.4022
y144	-2.2688	1.9377	-1.0433
z144	0.9878	1.1926	-0.9553
x145	-0.6052	-0.1774	-0.2050
y145	-2.1801	1.5541	-1.0425
z145	1.1587	1.2221	-0.7756
x146	-0.7306	-0.3050	-0.0735
y146	-1.8193	1.6740	-0.7665
z146	1.1834	1.1098	-1.0165
x147	-0.6661	-0.6764	0.0474
y147	-1.5699	1.9575	-0.4995
z147	1.3250	0.7793	-0.9824
x148	-0.6694	0.2014	0.3380

y148	-1.4253	2.1064	-0.2121
z148	1.6034	1.0100	-0.7611
x149	-0.9268	-0.0345	0.4602
y149	-1.2613	2.3727	-0.0931
z149	1.7050	0.8009	-0.5452
x150	-0.8925	0.3608	0.8386
y150	-0.9878	2.6105	0.0525
z150	1.8587	0.9775	-0.1164
x151	-0.9672	0.0051	0.7884
y151	-0.6403	2.8362	0.2298
z151	1.8279	0.5369	-0.1241
x152	-1.0788	-0.1462	0.7745
y152	-0.3601	3.0243	0.3416
z152	1.8386	0.2951	0.0153
x153	-1.2666	0.1007	1.0056
y153	-0.1583	3.0767	0.3440
z153	1.8735	0.6153	0.7067
x154	-1.2014	0.9545	1.1574
y154	-0.1172	3.1862	0.2457
z154	2.2833	1.3873	1.2769
x155	-1.3237	0.8798	1.2861
y155	-0.0760	2.9361	0.1328
z155	2.2410	0.8674	1.6061
x156	-1.4111	1.0001	1.6359
y156	0.0216	3.1306	0.5058
z156	2.2650	0.2846	1.6970
x157	-1.4662	1.2440	2.0438
y157	0.2634	2.8190	1.1464

z157	2.2478	0.7590	1.6356
x158	-1.7209	1.1798	2.1141
y158	0.5972	2.2213	1.1763
z158	1.8325	0.4853	1.2259
x159	-1.8648	1.2542	2.2241
y159	0.8293	1.3036	0.5999
z159	1.8294	1.2289	1.7191
x160	-2.0164	1.4027	2.1922
y160	1.0176	0.7890	0.0736
z160	1.8323	1.1795	1.6342
x161	-2.0862	1.4264	2.3096
y161	1.2969	0.2524	-0.0857
z161	1.8444	1.0025	1.3865
x162	-1.9831	1.6133	2.4220
y162	1.5126	-0.1465	-0.1734
z162	1.8101	1.1047	0.7948
x163	-1.9823	1.7460	2.0468
y163	1.5573	-0.1305	-0.7432
z163	1.7303	1.6985	0.5937
x164	-1.7133	2.5296	1.9173
y164	1.5516	-0.0153	-1.4764
z164	1.5547	0.6152	-0.1472
x165	-1.5127	3.1429	1.0809
y165	1.5655	0.1858	-2.2236
z165	1.6639	0.8372	0.1959
x166	1.1796	-0.0464	-1.8173
y166	0.1156	-3.0661	-0.3726
z166	0.1007	0.2512	-0.3540

x167	0.9422	-0.7160	-2.3805
y167	-0.1767	-2.1880	-0.1825
z167	0.0674	-0.0547	-0.6072
x168	0.8310	-0.9142	-2.3435
y168	-0.3854	-1.5806	-0.2929
z168	0.2207	-0.0909	-0.0528
x169	0.8253	-0.8985	-2.0084
y169	-0.4722	-1.6032	-0.4173
z169	0.4679	0.6190	0.8225
x170	0.8779	-1.0210	-1.6533
y170	-0.5261	-1.7120	-0.3493
z170	0.5976	0.8408	0.8614
x171	1.0421	-1.1330	-1.1213
y171	-0.3728	-1.8694	-0.0839
z171	0.4654	0.7340	0.3470
x172	1.1681	-1.3656	-0.7405
y172	-0.2642	-2.0574	0.1172
z172	0.3259	0.7011	0.0350
x173	1.1706	-1.5336	-0.5961
y173	-0.2454	-2.1516	0.2239
z173	0.6368	0.8144	0.4124
x174	1.1165	-1.7943	-0.4723
y174	-0.4360	-2.2563	0.1873
z174	1.0639	1.1370	0.7381
x175	1.1557	-1.6059	-0.2518
y175	-0.6355	-2.0994	0.1820
z175	1.3528	1.3060	0.8432
x176	1.5712	-1.5027	0.2100

y176	-0.5828	-2.1494	0.2865
z176	1.2847	1.2516	0.7330
x177	1.7409	-1.5822	0.5001
y177	-0.5441	-2.2244	0.3905
z177	1.3948	1.3334	0.6939
x178	1.7613	-1.5472	0.6925
y178	-0.5545	-2.1615	0.5013
z178	1.4730	1.3931	0.6409
x179	1.7404	-1.4393	0.8563
y179	-0.5156	-2.0644	0.5987
z179	1.6144	1.5882	0.6097
x180	1.6536	-1.4997	0.7839
y180	-0.5046	-2.0382	0.5868
z180	1.7722	1.9611	0.7265
x181	1.4869	-1.3702	0.7508
y181	-0.4946	-1.8631	0.6134
z181	1.8479	2.3844	0.6291
x182	1.4315	-0.8322	0.7800
y182	-0.6875	-1.7867	0.4773
z182	1.5698	2.4675	0.2403
x183	1.6648	-0.2352	0.7295
y183	-0.9019	-1.6875	0.3223
z183	1.2988	1.9143	-0.0258
x184	1.8558	-0.3348	0.7414
y184	-0.6885	-1.3016	0.5809
z184	1.5509	2.2294	0.1601
x185	1.6437	-0.2828	0.6624
y185	-0.4608	-0.6718	0.6807

z185	1.8623	2.5327	-0.0555
x186	1.0965	0.2541	-0.1247
y186	-0.5566	-0.2836	0.8979
z186	1.4338	2.4717	-0.3783
x187	0.7513	0.5568	-0.7011
y187	-0.5817	0.0748	1.1411
z187	1.2483	2.9840	-0.1495
x188	0.5021	0.6484	-0.9951
y188	-0.6443	0.7579	1.2754
z188	1.0613	3.3511	0.0233
x189	0.4256	0.5721	-1.1737
y189	-0.7661	0.5996	1.0700
z189	0.9921	3.1120	0.1097
x190	0.1586	0.1999	-1.9434
y190	-0.2390	1.6246	2.1029
z190	1.5085	3.9766	1.1773
x191	0.1427	0.9354	-1.9763
y191	-0.1472	1.3731	1.8915
z191	1.8159	3.9707	1.5227
x192	0.2007	0.7575	-2.0483
y192	-0.2381	1.6447	1.8496
z192	1.8769	3.5188	1.8937
x193	0.3585	1.2854	-1.6506
y193	-0.6549	1.0747	1.5798
z193	1.5891	3.2722	2.4760
x194	-0.1017	2.1072	-2.1927
y194	-1.0425	0.6081	1.5207
z194	0.6634	3.7410	2.3680

x195	-0.1151	2.5802	-2.2288
y195	-1.3406	0.7771	1.1649
z195	-0.0408	3.8928	2.1323
x196	-0.4483	2.9324	-2.5953
y196	-0.9295	0.4312	1.3925
z196	-0.5082	3.5812	1.6887
x197	-0.5496	2.6862	-2.7375
y197	-0.9246	0.6461	1.5745
z197	-1.0006	2.5924	0.8524
x198	-0.6011	2.6469	-2.6577
y198	-1.1599	-0.0430	1.9889
z198	-1.2054	1.4739	0.2741
x199	-0.5969	2.6165	-2.4303
y199	-1.1516	-0.7189	2.2362
z199	-1.2087	0.5238	-0.1761
x200	-0.4219	2.8008	-1.9217
y200	-0.9311	-1.5789	1.6181
z200	-1.1449	-0.7492	-1.0064
x201	0.0359	3.6461	-0.8393
y201	0.7242	-0.0635	3.3932
z201	-0.8391	-2.1096	-1.4332
x202	-0.0376	3.6833	-0.5133
y202	1.6233	0.0274	3.6507
z202	-0.4510	-3.4999	-2.0318
x203	-0.5075	2.7452	-0.5991
y203	1.4514	-1.1295	2.0874
z203	-1.1696	-4.3220	-2.8439
x204	-0.8600	2.6633	-0.2615

y204	1.5393	-1.0143	1.4885
z204	-1.5912	-4.1896	-2.7770
x205	-0.4433	3.3070	0.9967
y205	1.7183	-0.6721	0.8137
z205	-1.4691	-3.0177	-2.1132
x206	-0.3961	2.4390	1.4489
y206	1.8633	-0.5642	0.4110
z206	-1.6203	-2.5585	-2.1102
x207	-0.4809	1.0618	1.4863
y207	1.7814	-0.5910	-0.0830
z207	-1.8324	-2.2880	-2.1775
x208	-0.1960	-0.2288	1.8089
y208	1.5533	-0.2850	-0.4511
z208	-2.0040	-1.8958	-2.3140
x209	0.2461	-0.4613	2.1329
y209	1.1041	0.2273	-0.9191
z209	-2.4049	-0.8464	-2.7502
x210	0.8335	-0.0443	2.1547
y210	0.4100	0.5964	-1.3591
z210	-2.7982	0.4798	-3.2278
x211	1.1808	0.0204	1.7146
y211	-0.1196	1.5385	-1.3080
z211	-3.2052	1.4229	-3.8562
x212	1.0576	0.0250	1.7622
y212	0.2573	2.1449	-1.3164
z212	-3.1465	1.7456	-3.2503
x213	0.9257	0.5598	1.6949
y213	0.5282	1.9338	-1.2914

z213	-2.9335	2.5010	-2.6096
x214	0.6648	0.9066	1.4739
y214	0.8063	1.6280	-1.1709
z214	-2.7359	2.9944	-1.9828
x215	0.5428	1.0530	1.1194
y215	0.9404	1.1967	-0.9938
z215	-2.3862	3.0786	-1.5705
x216	0.3673	1.6908	0.5033
y216	1.0991	0.1008	-0.9000
z216	-2.1994	2.9154	-1.7471
x217	0.5938	1.7520	0.0330
y217	1.1251	-0.5544	-0.5211
z217	-1.8275	2.6196	-1.6506
x218	0.7209	1.4720	-0.9511
y218	0.8396	-1.7921	-0.6961
z218	-1.5383	1.9031	-1.7763
x219	1.2540	0.9235	-1.3092
y219	0.4000	-2.2985	-0.5713
z219	-1.1948	0.9745	-1.5963
x220	3.1275	-2.3392	-3.4039
y220	-0.7664	-1.0872	-1.7661
z220	-1.5082	-1.1742	-0.1588
x221	2.6550	-2.2283	-3.4361
y221	-0.9441	-0.7738	-1.7200
z221	-1.3510	-1.6029	-0.1251
x222	2.2334	-1.9486	-3.4459
y222	-1.1235	-0.4075	-1.8115
z222	-1.1967	-1.8808	-0.0401

x223	1.9845	-1.7511	-3.3735
y223	-1.1464	-0.5749	-2.0714
z223	-1.1765	-1.7454	0.1270
x224	1.9494	-1.5347	-2.9663
y224	-0.9318	-0.7164	-1.8681
z224	-1.0281	-1.5724	0.4739
x225	1.7672	-1.2398	-2.5354
y225	-1.0582	-0.8143	-1.7745
z225	-0.9092	-1.1407	0.6695
x226	1.7389	-0.8363	-1.9773
y226	-1.0888	-0.7039	-1.5307
z226	-0.7325	-0.7127	0.7849
x227	1.5841	-0.2727	-1.4866
y227	-1.2294	-0.4414	-1.3458
z227	-0.6158	-0.5165	0.6359
x228	1.6242	-0.0370	-1.1395
y228	-1.2250	-0.2640	-1.1360
z228	-0.4758	-0.4187	0.3654
x229	1.5814	-0.4942	-1.2758
y229	-1.2568	-0.9130	-1.4452
z229	-0.2217	-0.1073	0.3055
x230	1.5471	-0.6706	-1.0037
y230	-1.3903	-1.1011	-1.3893
z230	-0.1539	0.4481	0.2298
x231	1.5997	-0.5827	-0.4807
y231	-1.5702	-1.1778	-1.1204
z231	-0.1012	0.7702	0.1577
x232	1.8824	-0.6297	-0.1860

y232	-1.4590	-1.4358	-1.0559
z232	0.0539	1.1733	0.2314
x233	2.0042	-0.4916	0.0846
y233	-1.5749	-1.5405	-1.0385
z233	0.0681	1.5037	0.0685
x234	2.1287	-0.5853	0.5507
y234	-1.8127	-1.7488	-1.0067
z234	-0.1308	1.6421	-0.2420
x235	2.4183	0.2028	0.6896
y235	-1.7622	-1.4677	-1.0669
z235	-0.2648	1.3842	-0.3963
x236	2.2952	-0.0812	0.4522
y236	-1.9847	-1.8745	-1.3281
z236	-0.1908	1.3516	-0.4096
x237	2.0650	-0.4393	0.5755
y237	-2.2171	-2.2571	-1.3854
z237	-0.3051	1.2653	-0.5695
x238	2.3314	0.5768	1.0867
y238	-2.1374	-2.0674	-1.2427
z238	-0.3027	0.9841	-0.7494
x239	2.0112	1.0885	1.2642
y239	-2.1950	-1.9807	-1.3978
z239	-0.2655	0.9474	-1.0014
x240	1.7465	0.5564	1.2532
y240	-2.3694	-2.3210	-1.7363
z240	-0.3832	1.0093	-1.3172
x241	1.0843	-0.0027	1.3735
y241	-2.5484	-2.5001	-1.9323

z241	-0.5412	1.0467	-1.5904
x242	1.0028	0.8590	1.7802
y242	-2.7190	-2.8395	-2.2157
z242	-0.6395	0.8397	-1.7403
x243	1.1032	1.1507	2.4483
y243	-2.7642	-2.7629	-2.1301
z243	-0.6072	0.7921	-1.5990
x244	0.6957	0.6896	1.6105
y244	-2.8119	-2.4755	-1.9586
z244	-0.6819	1.1544	-1.4389
x245	0.7824	1.2156	1.4681
y245	-2.9451	-2.2146	-1.8728
z245	-0.7509	1.3414	-1.2774
x246	0.8719	1.6793	1.6840
y246	-3.0234	-2.1693	-1.9461
z246	-0.7667	1.4260	-1.0653
x247	0.3851	1.4230	0.6299
y247	-2.8010	-1.5828	-1.2963
z247	-0.8638	1.5429	-0.7340
x248	0.2632	1.9024	0.1995
y248	-2.7560	-1.4766	-0.8655
z248	-0.8597	1.6332	-0.2227
x249	0.4388	2.4900	-0.0839
y249	-2.9439	-1.6736	-1.0925
z249	-0.8273	1.7423	0.0084
x250	0.4969	2.2206	-0.7453
y250	-3.0111	-1.1206	-0.8749
z250	-1.0611	1.4557	0.0297

x251	0.1683	1.8888	-1.3549
y251	-3.0719	-0.8335	-0.6133
z251	-1.3905	1.0653	0.1981
x252	0.1139	2.2636	-1.5237
y252	-3.0899	-1.4361	-0.8006
z252	-1.1543	0.7919	0.6917
x253	-0.0051	2.9156	-2.0793
y253	-3.0998	-1.6662	-0.6076
z253	-1.1097	0.9578	0.5694
x254	0.0317	3.1888	-2.0579
y254	-3.3407	-1.6828	-1.0734
z254	-0.8882	0.8185	0.8829
x255	-0.3248	3.1703	-2.7100
y255	-3.2409	-1.5744	-0.7907
z255	-0.7857	0.5344	0.8551
x256	-0.7325	3.7449	-3.4954
y256	-3.1892	-2.1257	-0.5501
z256	-0.6454	0.4398	0.3838
x257	-2.4359	-0.6056	-0.5182
y257	5.8431	7.1183	6.1853
z257	-4.3185	-0.4172	-7.9480
x258	-0.9097	0.5130	-0.3191
y258	4.3895	5.9907	5.4236
z258	-4.3923	-0.2091	-7.0647
x259	-0.0424	0.9232	0.0431
y259	3.1967	5.1996	4.5237
z259	-4.4077	0.0491	-6.6684
x260	0.8002	1.4471	0.5218

y260	1.8327	4.2182	3.4215
z260	-4.2982	0.2633	-6.3108
x261	1.2680	1.4858	0.7167
y261	0.3315	3.4548	2.3677
z261	-4.2922	0.0782	-5.5901
x262	1.5317	1.7236	0.3691
y262	-0.8568	2.3704	1.5121
z262	-4.4628	0.1308	-4.6540
x263	1.1270	2.1007	-0.1731
y263	-1.5712	1.1127	0.2049
z263	-4.4608	0.5400	-3.4749
x264	0.2934	1.9255	-0.8777
y264	-1.6947	1.0177	-0.7748
z264	-4.2538	0.6342	-2.1495
x265	-0.4558	1.3033	-1.3922
y265	-1.8366	0.4571	-2.2624
z265	-4.3936	0.8232	-1.2861
x266	-1.6151	1.2266	-2.6317
y266	-2.1754	-0.0580	-2.9644
z266	-3.3709	-0.2362	0.3737
x267	-2.5441	0.2276	-3.4508
y267	-1.7920	-2.2477	-1.4970
z267	-2.6461	-2.7409	2.8429
x268	-2.7187	-0.6757	-3.5851
y268	-0.8519	-3.1759	-0.2491
z268	-2.6007	-4.8473	4.4896
x269	-2.2581	-2.6745	-3.0863
y269	-0.7590	-0.9547	-0.5642

z269	-2.4854	-6.7543	5.5632
x270	-1.9090	-2.6290	-2.7896
y270	-0.3229	-0.5651	-0.5283
z270	-2.6436	-7.1740	6.1561
x271	-1.2054	-4.2314	-1.8141
y271	0.1750	-1.2519	0.1711
z271	-2.9965	-5.7597	5.9736
x272	-0.6072	-5.4867	-1.2719
y272	0.2765	-0.8098	0.0516
z272	-3.4000	-3.9039	5.1570
x273	-0.0460	-6.6226	-0.0291
y273	0.1377	0.9257	0.2161
z273	-3.6981	-1.5927	4.5358
x274	0.7515	-7.3880	0.8953
y274	0.3504	0.9856	0.7425
z274	-3.2719	-1.3548	4.0445
x275	0.8280	-6.8049	0.6521
y275	0.2750	1.0017	1.0968
z275	-2.2130	-1.2559	3.9409
x276	1.2371	-6.0405	0.5800
y276	0.4563	0.2799	1.3656
z276	-2.1714	0.4211	3.5464
x277	1.3045	-6.7213	0.7203
y277	0.6995	0.3901	1.4490
z277	-2.9037	0.3618	2.9606
x278	1.5034	-6.9275	1.7588
y278	0.5226	0.3708	0.5353
z278	-2.9346	0.6316	3.3644

x279	1.0801	-5.8895	1.1249
y279	0.9072	-0.6148	0.7095
z279	-3.1632	1.2041	3.5122
x280	0.9363	-4.6584	1.0507
y280	0.8350	-1.5849	0.1949
z280	-3.4431	1.0450	3.0774
x281	0.5639	-3.5327	0.6517
y281	0.9314	-2.3445	-0.0584
z281	-3.4903	1.2641	2.6755
x282	0.4528	-3.3901	0.6993
y282	0.0851	-1.2456	-1.7940
z282	-2.8945	0.5387	2.2135
x283	0.1771	-3.3281	0.6680
y283	0.0363	0.8415	-2.6386
z283	-2.4242	-0.5291	1.4461
x284	-0.2556	-3.0752	0.3494
y284	0.5032	1.0808	-3.0772
z284	-2.1922	-0.5538	0.4896
x285	-0.6853	-2.6820	0.0007
y285	0.3228	0.9447	-2.6715
z285	-1.9379	0.7851	0.8428
x286	-0.5056	-2.3070	-0.5606
y286	-0.0902	1.9934	-2.0498
z286	-1.1634	1.2414	0.8005
x287	-0.8893	-2.1105	-0.8994
y287	-0.3593	1.8427	-1.1321
z287	-0.6372	1.0775	0.6251
x288	-1.1780	-2.4244	-1.6417

y288	-0.2669	1.5685	-0.8008
z288	-0.2380	0.9747	0.2954
x289	-1.8108	-2.4624	-2.2341
y289	0.2974	2.1230	-0.6315
z289	-0.0297	1.2957	-0.8356
x290	-2.4584	-2.6152	-2.2522
y290	1.3313	2.8652	0.0185
z290	0.0157	1.0445	-2.0838
x291	-3.1680	-2.8330	-1.8275
y291	2.8346	4.3081	1.6824
z291	-1.1063	0.2246	-3.8137
x292	-4.1549	-2.4570	-1.6572
y292	5.0389	7.0260	4.0126
z292	-2.7791	0.1743	-6.3123
x293	-1.4655	2.2068	-1.5905
y293	2.7683	6.7959	2.4429
z293	-0.1905	1.4078	-1.3369
x294	-1.8539	3.3922	-2.3884
y294	1.6498	6.1183	2.7111
z294	0.6066	1.8676	-0.6682
x295	-2.2516	4.0720	-2.3031
y295	0.0264	5.1756	1.8035
z295	1.1811	2.4886	1.2087
x296	-2.0713	4.7648	-1.7754
y296	-1.4385	4.1928	0.8466
z296	0.7940	2.7874	2.0691
x297	-1.9805	5.6195	-1.5557
y297	-2.6688	3.6130	-0.3873

z297	0.6684	2.6300	2.6727
x298	-2.4644	6.0066	-1.8421
y298	-2.5087	3.3582	-0.5378
z298	0.2861	2.8008	3.1634
x299	-2.8190	5.8759	-3.1591
y299	-1.9225	2.4762	-0.1750
z299	-0.3857	3.4100	3.4961
x300	-2.4750	5.8815	-3.5865
y300	-0.6247	1.0930	0.6462
z300	-1.0418	4.2171	4.1815
x301	-1.7367	3.9029	-3.9992
y301	0.7792	-2.8270	1.8222
z301	-0.8315	2.7192	4.1126
x302	-1.4980	1.6846	-2.9103
y302	1.0543	-2.7790	1.2810
z302	-0.3724	3.2506	4.0327
x303	-0.5797	1.1480	-0.5396
y303	-0.0358	-1.9245	-0.5951
z303	-0.5767	2.9084	4.1058
x304	-0.0641	2.2293	0.2755
y304	-0.3399	-3.3310	-0.7454
z304	-0.8479	0.6721	3.6857
x305	0.5979	2.1533	1.0088
y305	-0.3560	-3.0198	-1.1380
z305	-1.6419	-0.9008	3.2054
x306	1.1953	1.9116	1.5900
y306	-0.1209	-2.6215	-1.3511
z306	-2.5396	-1.8065	2.7667

x307	1.5077	2.2293	1.6278
y307	-0.1870	-2.0766	-1.9681
z307	-2.8822	-1.9460	2.6510
x308	1.8030	2.8025	1.0178
y308	-0.0260	-1.6079	-2.8374
z308	-2.7604	-0.9326	2.9275
x309	2.1788	3.0479	0.0756
y309	0.4100	-1.4386	-3.4566
z309	-2.4498	0.8994	3.4292
x310	1.6943	1.1332	-1.8834
y310	0.1552	-3.0418	-4.4982
z310	-2.3582	2.0986	3.6080
x311	1.0773	-1.5969	-3.3627
y311	-0.3820	-4.8722	-5.8620
z311	-2.3873	1.4959	2.1386
x312	-1.8665	1.8195	-1.7661
y312	1.0486	-3.9081	2.1305
z312	2.9745	-6.2602	-1.0450
x313	-1.6153	2.2058	-1.6599
y313	1.7495	-2.8646	1.6606
z313	1.8430	-4.4268	-1.3662
x314	-0.6913	1.9189	-0.8954
y314	1.3694	-2.2277	0.1819
z314	0.8986	-3.6368	-2.2562
x315	-0.2499	1.7080	-0.3816
y315	0.9460	-1.4604	-0.4458
z315	1.3158	-2.4211	-1.6466
x316	0.4923	1.5949	0.0439

y316	0.8726	-1.0610	-0.6679
z316	1.1888	-1.6251	-1.3115
x317	0.6045	0.8909	-1.1756
y317	0.9118	-0.7438	-0.3194
z317	1.1745	-1.0770	-0.9739
x318	1.1873	0.4335	-1.4959
y318	0.5610	-0.5510	-0.3094
z318	0.9236	-0.5162	-0.8092
x319	2.2039	0.9725	0.8961
y319	0.0503	-0.4006	-1.4410
z319	0.6939	0.3781	-1.2322
x320	3.6242	1.2573	3.2862
y320	-0.8004	0.0153	-1.9214
z320	0.8973	1.7159	-1.0077
x321	2.8743	-0.5084	1.9634
y321	-0.8213	0.8680	-0.4194
z321	1.2798	3.1902	-0.0049
x322	3.0952	-0.8776	2.6428
y322	-1.3939	-0.4104	-1.0978
z322	1.7281	0.5658	-0.3332
x323	2.8006	-0.9537	2.5946
y323	-1.7388	-0.9353	-1.2992
z323	2.3041	-0.4107	-0.2363
x324	2.6305	-0.9817	2.5507
y324	-1.8114	-1.7438	-1.4113
z324	2.6301	-1.4615	-0.2079
x325	2.4238	-1.2569	2.2280
y325	-1.8127	-2.3789	-1.5039

z325	2.7907	-2.0397	-0.0712
x326	2.0205	-1.4013	1.5424
y326	-1.6019	-2.9306	-1.5311
z326	2.4806	-2.1716	-0.1159
x327	1.5638	-0.6969	0.7093
y327	-1.3358	-3.7680	-1.6868
z327	1.5832	-2.0460	-0.4734
x328	1.7959	-0.2659	0.0317
y328	-1.2667	-4.6818	-2.0445
z328	0.4262	-1.9077	-0.8547
x329	2.5578	-0.1017	-0.4317
y329	-1.2058	-5.6708	-2.4280
z329	-0.8383	-2.0145	-1.1451
x330	2.9876	-0.4655	-1.1750
y330	-0.4387	-6.0217	-1.7577
z330	-2.1013	-2.5654	-2.4240
x331	-0.6467	2.6637	-1.5473
y331	1.3505	-0.5111	1.7735
z331	0.7594	-6.2371	4.6559
x332	-0.4677	0.5730	-0.4827
y332	0.9484	-0.9213	1.6320
z332	1.0252	-6.0644	4.5580
x333	-0.8101	-0.9067	-0.2029
y333	0.3143	-0.9597	1.3214
z333	1.3252	-4.5043	4.5208
x334	-1.6353	-2.2031	0.0932
y334	-0.7724	-1.2449	1.0161
z334	0.9565	-3.5119	4.4444

x335	-1.5785	-1.8947	0.7922
y335	-1.0249	-0.7207	0.8984
z335	1.1179	-2.4196	4.4918
x336	-1.7061	-1.1482	0.1738
y336	-1.4792	0.4698	-0.5865
z336	1.0806	-1.5594	3.9506
x337	-1.6764	-0.5297	-0.7080
y337	-1.5040	1.3621	-1.9932
z337	1.0101	-0.6650	3.5292
x338	-1.4259	-0.0821	-1.2150
y338	-1.1274	1.9570	-2.6794
z338	0.9852	0.2645	3.3210
x339	-1.0435	0.3073	-1.0562
y339	-0.5475	2.5107	-2.1617
z339	0.7969	1.1390	2.9220
x340	-1.3404	0.9160	-1.3690
y340	-0.5509	4.4403	-2.2835
z340	0.7789	2.0973	2.3174
x341	-1.3790	0.6875	-0.2003
y341	-0.2930	4.3837	-1.2406
z341	0.8695	2.4554	2.6319
x342	-1.3866	0.7863	0.0975
y342	-0.2729	4.2744	-0.7861
z342	0.7267	2.8959	2.5464
x343	-1.1877	0.8795	0.4850
y343	-0.0691	4.4223	-0.2620
z343	0.6308	3.3563	2.5069
x344	-1.1548	0.9055	0.4811

y344	-0.1060	4.5967	-0.3876
z344	0.5732	3.8798	2.5659
x345	-0.9734	0.7697	0.4885
y345	0.2594	5.1381	0.1335
z345	0.4413	3.9412	2.3049
x346	-0.8392	0.2239	0.1884
y346	0.2785	5.4687	0.2140
z346	0.4903	3.5601	2.1115
x347	-0.5830	-0.7056	-0.1535
y347	0.5637	5.4585	0.4711
z347	0.1862	3.1264	1.7184
x348	-0.3749	-1.6505	-0.5348
y348	1.1239	4.7951	1.0058
z348	-0.4937	3.1538	1.1155
x349	-0.3361	-2.5326	-1.1003
y349	1.5509	4.3490	1.3405
z349	-1.0766	2.9318	0.9673
x350	-0.6874	3.9437	-1.6118
y350	-1.3387	1.7018	1.5871
z350	1.9561	0.0481	1.1941
x351	-0.4677	2.6336	-1.2538
y351	-1.0395	1.4649	1.7303
z351	2.1353	2.2932	0.3272
x352	-0.4005	1.8691	-1.1410
y352	-0.9068	2.6098	1.3069
z352	2.4947	1.9744	-0.0373
x353	-0.3445	1.2323	-0.8869
y353	-0.9110	3.2960	1.0324

z353	2.8972	1.2648	-0.1374
x354	-0.2645	0.6197	-0.5954
y354	-0.9594	2.5044	1.2780
z354	3.1066	1.5395	-0.2723
x355	-0.3806	-0.0537	-0.0999
y355	-1.3793	1.0431	2.0917
z355	2.8039	1.6462	-0.1750
x356	-0.5670	-0.4355	0.2310
y356	-1.6536	-0.2709	2.7420
z356	2.0415	0.9830	0.5577
x357	-0.5947	-0.2238	0.1664
y357	-1.6067	-1.5142	2.9811
z357	1.1735	0.2428	1.5914
x358	-0.3218	0.3688	-0.4079
y358	-1.1067	-3.2287	3.0098
z358	0.5584	-0.6284	2.8402
x359	0.2600	1.0202	-1.6466
y359	-0.3112	-5.1064	2.5712
z359	0.2754	-1.6336	3.1097
x360	-0.8991	-0.6672	1.1049
y360	0.1429	0.9727	1.1350
z360	1.5098	1.1269	-0.7566
x361	-0.4439	-0.6181	1.2721
y361	0.0732	0.8852	0.6346
z361	0.1534	1.2587	-2.3402
x362	0.0708	-0.3893	1.9366
y362	0.1735	0.7605	0.1555
z362	-0.7139	1.2846	-3.0036

x363	0.3161	-0.5477	2.1635
y363	0.3423	1.0026	0.0804
z363	-0.9215	2.1143	-2.3033
x364	0.4247	-0.7605	2.6893
y364	0.4549	1.4616	-0.3368
z364	-0.9630	2.7764	-2.1055
x365	0.6957	-1.3522	3.2132
y365	0.4639	1.9004	-0.7250
z365	-0.8618	3.2416	-1.6231
x366	1.0856	-1.3936	3.5738
y366	0.4125	2.1503	-0.9676
z366	-0.5276	3.3017	-0.7071
x367	1.0838	-1.9559	3.4352
y367	0.5110	2.6766	-0.8901
z367	-0.1133	2.9776	0.1926
x368	0.8363	-1.9924	2.5464
y368	0.6396	2.9828	-0.6094
z368	0.3075	2.6894	0.9175
x369	0.4523	-2.6703	1.2039
y369	0.7212	3.1378	-0.3113
z369	0.7015	1.5226	1.0908
x370	0.3392	-2.6468	1.0317
y370	-0.9136	2.9586	-2.1305
z370	1.0485	-0.4776	-1.9633
x371	1.0554	-1.7142	0.7499
y371	-1.0568	3.8662	-1.8948
z371	-0.1006	0.6694	-2.9978
x372	1.6449	-1.7520	0.9517

y372	-1.3034	3.8547	-1.2859
z372	-0.9408	1.6662	-3.1563
x373	1.9199	-1.1300	0.8018
y373	-1.9215	4.3190	-1.3851
z373	-1.7236	2.8453	-3.3342
x374	2.0870	-0.1443	0.5976
y374	-2.0984	5.4348	-1.2041
z374	-2.2157	3.7326	-3.3874
x375	2.2111	0.1185	0.3663
y375	-1.9505	6.4089	-1.3572
z375	-2.3443	3.8629	-2.9909
x376	2.3250	-0.3708	0.1795
y376	-1.7132	6.4724	-1.8543
z376	-2.2116	3.4900	-2.1019
x377	2.1022	-1.2955	-0.2291
y377	-2.0624	5.7729	-2.1246
z377	-1.5344	2.9162	-1.3812
x378	1.5956	-2.5212	-0.7982
y378	-2.4926	4.7662	-2.7144
z378	-0.5258	1.8253	-0.3726
x379	0.5375	-2.6839	-1.8135
y379	-2.5402	3.7172	-3.3146
z379	0.1503	1.3861	-0.3176
x380	-1.5444	1.3930	-2.6029
y380	-9.4915	-9.4377	1.7764
z380	0.5324	-13.4196	-2.0084
x381	-2.3758	0.3367	0.3162
y381	-7.9499	-6.0535	0.1668

z381	-1.1545	-7.8622	-4.2878
x382	-2.4307	-2.5559	-1.1443
y382	-6.4314	-6.1250	1.6127
z382	-1.8690	-3.5549	-4.7171
x383	-2.4857	-6.6911	0.4071
y383	-3.7584	-6.7863	3.6141
z383	-1.9347	-4.6062	-4.5473
x384	-2.8412	-6.6094	-0.1467
y384	-1.8569	-3.1488	3.7474
z384	-5.7484	-4.6221	-9.1905
x385	-0.3821	-7.1978	1.8731
y385	-2.5252	0.3071	1.4120
z385	-7.5872	-0.3454	-10.7163
x386	-1.1565	-9.1668	1.5901
y386	-1.4652	4.2053	0.1333
z386	-5.7742	5.4949	-8.7414
x387	-1.4261	-8.7164	-0.5459
y387	1.2462	6.4474	2.3586
z387	-3.8332	8.4850	-6.2549
x388	-1.7741	-6.8159	-2.8684
y388	3.2509	3.3144	4.8825
z388	-2.7023	4.7089	-6.6104
x389	-0.9869	-6.0414	-3.9743
y389	1.0185	3.6727	4.9715
z389	-0.9900	3.4248	-5.9646
x390	0.0322	-4.7043	-3.1047
y390	0.1695	4.8438	2.4148
z390	0.5914	4.6576	-4.7659

x391	-0.1252	-3.6978	-3.6980
y391	-1.7031	4.4943	-0.0636
z391	0.0464	4.1291	-5.2887
x392	-0.2732	-2.4964	-4.0000
y392	-1.6333	4.3896	-0.2963
z392	-0.1878	3.6323	-5.0160
x393	-0.3252	-1.3204	-4.0369
y393	-1.3227	5.4890	-0.4200
z393	-0.1963	3.4155	-4.1734
x394	-0.6699	-0.6167	-4.6201
y394	-1.4104	5.6572	-2.8456
z394	-0.2247	1.9528	-4.4091
x395	-0.5785	-0.2910	-4.2779
y395	-0.9898	4.7919	-3.0493
z395	0.0552	0.9425	-3.1236
x396	-0.2957	-0.0413	-3.0400
y396	0.3055	4.7035	-1.6263
z396	0.0934	-0.0123	-1.5562
x397	-0.2757	0.7267	-2.4241
y397	0.8810	6.4861	-1.2947
z397	0.2029	-0.9572	-0.5858
x398	-0.4313	0.2278	-2.3641
y398	1.2696	4.4880	-0.1558
z398	0.3688	-1.2203	-0.6204
x399	0.8415	1.9868	-1.2961
y399	2.4488	4.0002	1.9647
z399	1.1537	-1.0142	-0.8493
x400	0.9133	0.7280	-1.3681

y400	2.5998	1.3998	2.2825
z400	0.0002	-3.1597	-2.4085
x401	3.6122	3.8525	2.7439
y401	3.7057	1.5443	3.5290
z401	0.8161	-2.4945	-0.0260
x402	4.3534	4.8185	3.4245
y402	3.6773	1.1247	3.6913
z402	0.6906	-3.2424	-0.3806
x403	4.3730	5.0181	3.6274
y403	3.1149	0.4663	3.0153
z403	0.5770	-3.5661	0.2605
x404	4.0316	4.8819	3.9419
y404	2.3779	-0.2567	2.3266
z404	-0.9898	-4.6055	-1.4449
x405	2.9304	3.9541	3.5411
y405	1.4708	-1.0512	1.4597
z405	-1.7214	-5.4054	-2.2216
x406	0.7805	3.4676	3.4621
y406	-0.2767	-2.3102	-0.1679
z406	-3.8250	-6.9440	-4.6022
x407	-0.0896	2.0417	2.6270
y407	-0.7353	-2.3433	-1.4098
z407	-6.1944	-7.8890	-7.3033
x408	1.3774	1.7230	4.1600
y408	-1.0956	-2.2272	-2.4767
z408	-3.4320	-5.0357	-4.8007
x409	1.9093	1.7002	4.3721
y409	-1.0554	-2.6433	-3.0540

z409	-2.3897	-4.7047	-4.9163
x410	1.5522	0.9575	2.9847
y410	-1.1176	-3.2257	-4.2812
z410	-2.1506	-5.4886	-6.7563
x411	1.4072	1.6471	2.5745
y411	-0.9913	-3.8942	-4.3616
z411	-0.5505	-3.2848	-3.4951
x412	1.3778	2.2641	2.3645
y412	-0.8587	-4.0570	-4.0120
z412	0.2585	-2.4898	-1.9521
x413	0.9448	2.4093	2.3700
y413	-0.7765	-4.0245	-4.5475
z413	0.4912	-2.3665	-0.5827
x414	0.9391	1.9995	2.0198
y414	-0.7286	-2.2348	-3.4522
z414	1.0199	-2.9663	0.0489
x415	0.2864	0.9745	0.7209
y415	0.0368	-2.0075	-0.5328
z415	0.6630	-3.6309	-0.5273
x416	0.1484	0.2443	0.8270
y416	-0.2930	-2.9333	-0.7537
z416	1.6289	-2.8254	2.0573
x417	-0.2702	-1.3611	0.4102
y417	-0.4267	-2.8831	-1.0670
z417	1.7094	-4.1621	2.3120
x418	-0.9296	-2.7317	0.4960
y418	-0.3414	-3.8107	-0.7108
z418	1.2083	-3.9433	2.1116

x419	-1.5640	-3.4636	0.7491
y419	-1.0184	-3.4269	-0.3555
z419	1.7143	-4.8734	1.8869
x420	-1.4986	-3.1059	1.5761
y420	0.0959	-2.1243	0.8033
z420	0.3640	-6.0535	0.7716
x421	-1.5341	-3.9863	2.2211
y421	-0.0636	-2.5302	1.2613
z421	0.7007	-4.9950	0.4777
x422	-1.4022	-3.5377	2.8908
y422	-0.0015	-1.9799	1.6390
z422	0.8201	-5.3486	0.3023
x423	-0.5338	-2.5074	3.4643
y423	0.5420	-1.4490	1.6962
z423	-0.2022	-5.6014	0.1934
x424	-0.3116	-2.7757	3.2696
y424	0.3309	-1.5486	1.6855
z424	1.0778	-3.9879	0.4823
x425	1.2644	-3.1538	3.4907
y425	-0.3598	-1.3092	1.3210
z425	1.7752	-4.1989	1.3870
x426	2.6314	-1.9800	4.3536
y426	-0.9623	-1.9027	0.6404
z426	1.7662	-2.7848	2.5953
x427	2.6995	-1.5119	4.0217
y427	-1.1667	-1.9867	0.8380
z427	2.8341	-2.3562	2.2529
x428	2.5189	-1.0700	3.0599

y428	-1.8644	-2.7140	1.3586
z428	2.2443	-5.5767	1.8444
x429	2.7088	-0.6847	2.5346
y429	-1.8416	-2.2196	1.9897
z429	2.3918	-5.5321	1.2361
x430	2.2441	-0.0943	1.7020
y430	-1.1691	-2.1296	2.9989
z430	2.5368	-5.7367	-0.2616
x431	1.9859	0.9380	1.6870
y431	-1.0201	-2.9141	2.7007
z431	2.5290	-7.0536	-2.2912
x432	1.8883	1.7442	1.7270
y432	-0.4040	-3.1452	2.6377
z432	2.2680	-6.4685	-3.7601
x433	1.8669	2.6278	1.8713
y433	-0.5132	-2.6445	2.4854
z433	1.8208	-4.4866	-4.6658
x434	1.0576	3.1829	1.8336
y434	-1.4211	-0.3478	1.0412
z434	0.7546	-0.9238	-6.5669
x435	1.4735	1.5256	3.2369
y435	-1.1524	3.5012	0.1878
z435	0.8513	2.5802	-6.1404
x436	1.7581	1.5937	5.3569
y436	-0.3843	4.7085	1.3247
z436	1.6460	6.4704	-4.3191
x437	1.9249	0.9918	5.7472
y437	-0.6142	2.0145	0.7173

z437	1.5377	3.8669	-3.1768
x438	1.4719	3.3501	5.6200
y438	-1.2923	1.5097	-0.1610
z438	2.3141	6.4955	-1.5162
x439	0.9350	4.7251	4.2878
y439	-1.6040	2.3799	-1.8667
z439	3.0261	6.3257	-0.0864
x440	1.9312	4.0002	3.8082
y440	-2.3923	2.4384	-3.7213
z440	2.3582	2.0432	-0.3418
x441	2.1937	2.0676	3.7105
y441	-2.8867	3.9807	-5.5183
z441	2.6861	-4.2632	0.4429
x442	2.3171	4.1752	3.8815
y442	-1.1081	3.3179	-4.6526
z442	3.0497	-5.1514	2.9765
x443	3.5709	3.3180	3.4331
y443	-0.7555	4.2775	-2.4898
z443	3.7517	-2.1230	2.7080
x444	2.5252	2.7630	5.0218
y444	-1.0870	5.7291	-3.6616
z444	2.9447	-1.1898	5.3195
x445	1.3121	2.5078	4.1943
y445	-2.2180	4.9819	-5.3825
z445	2.2905	-0.0393	5.5527
x446	2.0914	0.7597	3.0151
y446	-1.8689	3.0469	-6.4527
z446	3.3307	0.7029	3.8517

x447	1.4335	-1.3595	1.1584
y447	-0.6114	2.5961	-5.1909
z447	3.9697	0.1729	2.4227
x448	2.5477	-1.8980	0.9805
y448	-0.0082	2.9002	-4.7103
z448	3.0645	-1.0217	0.6974
x449	4.0175	-0.4529	2.5540
y449	0.0737	1.9018	-5.2955
z449	2.4064	1.1346	-0.2455
x450	5.2890	0.5245	3.8925
y450	0.8496	2.6590	-4.8886
z450	1.1067	2.8931	-1.7089
x451	5.4845	1.9389	2.1939
y451	2.0778	2.5426	-1.3407
z451	-0.4654	5.3374	-4.3696
x452	5.0247	3.7982	0.6635
y452	3.7939	-2.8853	2.7052
z452	-1.2049	8.4701	-7.0752
x453	5.2516	5.2957	-0.5973
y453	-2.1832	-2.2485	-0.2989
z453	5.6810	2.8355	-3.9284
x454	4.9775	4.2703	-0.2440
y454	-1.8959	-2.3330	0.6550
z454	3.7587	2.5271	-5.8499
x455	4.8951	3.6170	1.0146
y455	-0.8588	-1.7130	2.1738
z455	2.8069	3.0873	-6.2640
x456	3.4949	2.5897	0.5147

y456	-1.4026	-1.0584	1.0596
z456	1.4967	1.3558	-7.4134
x457	2.7047	2.2401	0.7516
y457	-1.0579	0.7884	0.2055
z457	1.8135	1.0622	-5.3943
x458	1.3048	1.3432	0.8424
y458	-0.7161	2.4844	-0.0549
z458	1.0948	-1.0798	-3.9043
x459	0.3353	2.2498	0.8524
y459	-0.5354	3.6871	0.2116
z459	0.5022	0.6763	-2.4110
x460	-0.0384	2.3743	0.3678
y460	0.5900	5.0452	1.4621
z460	0.4558	1.4278	-1.6400
x461	-0.7357	2.9230	-0.0708
y461	1.8551	6.4303	2.6316
z461	0.2814	2.2878	-0.3597
x462	-1.5078	2.1590	-1.6000
y462	2.7103	6.7205	2.4498
z462	-0.2128	1.3986	-1.3169
x463	-2.0342	1.4001	-2.1641
y463	2.6970	4.6052	3.5149
z463	-0.7238	-2.7199	-3.3294
x464	-1.7474	1.9964	-1.8095
y464	3.0572	3.0127	3.8631
z464	0.1872	-3.8358	-3.6940
x465	-2.0633	1.7724	-2.0662
y465	3.1489	1.5922	3.6226

z465	0.4243	-5.6497	-4.9496
x466	-2.8392	1.8713	-2.3590
y466	2.7270	0.6354	3.3399
z466	1.5412	-6.3396	-3.3419
x467	-3.5534	2.7355	-2.0907
y467	2.2864	0.0921	2.9840
z467	2.0576	-5.6439	-2.2596
x468	-3.9154	2.5994	-3.2528
y468	1.8883	-0.7714	2.2855
z468	2.3354	-6.3097	-2.9981
x469	-4.0858	2.8341	-3.1530
y469	1.6369	-1.5070	2.1112
z469	2.5008	-5.9659	-1.9705
x470	-2.4861	2.5132	-2.4504
y470	1.6051	-2.3468	2.1505
z470	3.7957	-6.2937	-0.8209
x471	-1.7792	1.7420	-1.7157
y471	1.0209	-3.8758	2.1124
z471	2.9261	-6.2185	-1.0792
x472	-0.9614	3.6066	0.0080
y472	2.1958	-4.1691	2.5400
z472	3.2879	-3.4801	2.1584
x473	-1.4388	3.3608	-0.5258
y473	3.2779	-4.3624	3.2015
z473	2.2912	-3.2137	1.4037
x474	-1.4243	2.6402	0.6279
y474	3.5761	-4.7405	2.9214
z474	2.1823	-4.0020	2.7237

x475	-1.5813	1.4736	0.4571
y475	3.7161	-4.3230	2.6096
z475	1.4816	-5.4021	2.2100
x476	-1.2639	1.0011	0.2903
y476	3.6284	-3.6862	1.8753
z476	1.7491	-5.8311	2.2022
x477	-1.2821	1.1774	0.8320
y477	3.3549	-3.2876	1.0685
z477	1.3043	-5.5658	3.4490
x478	-1.0250	1.2628	0.3989
y478	2.9764	-2.4159	0.6846
z478	1.0495	-5.8801	3.6164
x479	-0.7212	1.6740	-0.8233
y479	2.4443	-1.1615	1.4777
z479	0.8597	-6.2399	2.9088
x480	-0.6444	2.5843	-1.4638
y480	1.3739	-0.6905	1.9553
z480	0.7705	-6.2849	4.6999
x481	-1.4061	0.2671	-0.7505
y481	1.0673	-0.6508	2.1070
z481	0.5451	-6.4288	3.7105
x482	-1.8138	-0.6324	-0.0158
y482	0.6063	-1.2984	1.3296
z482	0.9247	-5.1589	3.9274
x483	-2.4387	-1.5807	-0.2648
y483	0.6693	-0.9900	1.6997
z483	0.4923	-5.4779	2.3815
x484	-2.8050	-2.6275	-0.5101

y484	0.6633	-0.6877	2.0264
z484	-0.0006	-6.6171	1.3548
x485	-2.6699	-3.1995	-0.9909
y485	0.5203	-0.5183	2.1139
z485	0.2918	-7.3266	0.8340
x486	-1.7760	-2.5017	-0.9988
y486	0.0764	-0.8398	2.0066
z486	1.3309	-6.4218	0.9350
x487	-1.5213	-2.2865	-0.6390
y487	0.0593	-1.0747	1.8190
z487	1.4742	-6.3636	2.2665
x488	-1.5775	-2.2127	-1.1138
y488	0.1135	-1.2209	2.0991
z488	1.3627	-6.5127	2.4250
x489	-1.1987	-2.1997	-1.1846
y489	0.0123	-1.2412	2.4090
z489	1.8025	-6.6052	1.8955
x490	-0.8538	-2.7153	-0.8434
y490	0.2116	-0.4858	2.6576
z490	1.1002	-7.3970	1.0409
x491	-0.4509	-2.6082	-0.1855
y491	0.1816	0.0429	2.5030
z491	0.6202	-6.6949	1.2906
x492	-0.0994	-2.2154	0.1215
y492	0.0632	0.6966	2.3393
z492	0.3246	-6.0513	1.4762
x493	0.3861	-1.6198	0.1593
y493	-0.4346	1.4623	2.0510

z493	0.6797	-5.4332	1.3325
x494	0.6441	-0.5132	0.2688
y494	-0.9640	2.0381	1.6258
z494	0.7179	-4.0577	1.3991
x495	0.5107	0.8309	0.0191
y495	-1.4498	2.3119	1.3597
z495	0.8492	-3.0352	1.1933
x496	0.0814	2.2825	-0.2611
y496	-1.7450	2.0563	1.0086
z496	1.0701	-1.2375	1.4249
x497	-0.4389	3.2253	-0.9757
y497	-1.5312	1.9140	1.4237
z497	0.9083	-0.4317	0.3044
x498	-0.6587	3.8259	-1.4513
y498	-1.3332	1.5888	1.6868
z498	1.9854	0.1494	1.1669
x499	-0.1780	3.6217	0.3038
y499	-0.9798	2.3508	2.1294
z499	2.3246	2.4644	0.3631
x500	0.2176	3.6599	1.4568
y500	-0.7031	2.9926	2.2633
z500	2.1019	2.3906	0.1503
x501	0.6183	4.2247	2.5156
y501	-0.6474	3.2497	2.3585
z501	2.5778	2.4320	1.3926
x502	0.9868	4.6130	3.0883
y502	-0.6132	2.9686	2.3760
z502	2.1958	1.9460	0.8565

x503	1.0930	4.4011	3.2281
y503	-0.6936	2.2949	2.0217
z503	1.6971	1.1905	0.4476
x504	0.9157	3.7555	3.0487
y504	-0.7438	1.8434	1.6129
z504	1.3211	1.5650	0.2878
x505	0.3409	2.5385	2.4364
y505	-0.7611	1.1689	0.9031
z505	1.5533	0.7490	-0.1842
x506	-0.2597	0.9550	1.7646
y506	-0.5163	1.2957	0.5381
z506	1.4549	1.4253	-0.9538
x507	-0.8265	-0.5238	1.0583
y507	0.1579	1.0332	1.1034
z507	1.4597	1.0030	-0.8087
x508	-0.6785	-0.6100	0.1492
y508	0.1357	1.7918	0.3597
z508	0.7006	-0.4369	-1.9575
x509	-0.4790	-0.8462	-0.4212
y509	0.5576	2.9091	0.5247
z509	0.5212	-1.0211	-2.4364
x510	-0.0794	-1.0285	-0.5333
y510	0.8499	3.8682	0.6718
z510	0.5128	-0.9798	-2.7847
x511	0.2637	-1.3589	-0.3785
y511	0.5804	4.3508	0.4999
z511	-0.1056	-1.2945	-3.3647
x512	0.6382	-1.6599	0.1098

y512	0.3461	4.6127	0.2040
z512	-0.2535	-1.4763	-3.4770
x513	1.0059	-1.9491	0.6299
y513	0.0015	4.7674	-0.2100
z513	-0.0393	-1.3995	-3.1207
x514	1.1027	-2.0943	1.1472
y514	-0.1957	4.5137	-0.4974
z514	0.2537	-1.4339	-2.7445
x515	0.9262	-2.4930	1.2495
y515	-0.6673	3.9395	-1.4203
z515	0.5565	-1.4535	-2.5562
x516	0.1796	-2.4906	0.5101
y516	-1.0307	3.4212	-2.3655
z516	1.0764	-0.6867	-1.8842
x517	0.6533	-1.8061	0.6155
y517	-0.5258	3.3573	-2.5826
z517	1.4983	-0.8149	-0.4719
x518	1.0019	-1.3210	0.8783
y518	0.0581	3.3217	-2.7435
z518	1.7073	-0.7310	0.1583
x519	0.5492	-0.0408	0.5787
y519	0.8228	3.4546	-2.3502
z519	1.4156	-0.3127	0.3814
x520	0.7979	-0.4330	0.9251
y520	1.9535	3.4183	-1.5087
z520	1.4749	-0.6511	0.6870
x521	0.8230	1.0531	0.6496
y521	3.1600	3.2887	-0.6174

z521	0.7847	0.5359	-0.0638
x522	1.0063	2.1530	-0.1435
y522	4.3413	2.0019	0.4823
z522	-0.4482	1.8421	-1.7501
x523	2.8881	2.7768	0.3373
y523	5.2126	0.7426	1.8991
z523	-1.4956	4.2323	-4.1011
x524	5.1663	3.1251	1.3861
y524	4.4160	0.1755	1.9571
z524	-1.5600	7.2520	-6.0207
x525	5.0247	3.7982	0.6635
y525	3.7939	-2.8853	2.7052
z525	-1.2049	8.4701	-7.0752
x526	3.2386	5.8050	2.1760
y526	-0.0222	-2.4888	5.1464
z526	2.0601	-0.0549	6.8065
x527	2.8551	4.2963	1.9419
y527	-0.1415	-3.1744	4.2589
z527	1.1852	-1.5342	5.7928
x528	2.3724	3.5763	1.2592
y528	-0.4775	-3.2884	2.9160
z528	0.3748	-2.1351	4.7401
x529	1.3415	1.8044	0.7532
y529	-0.5649	-1.9736	1.2838
z529	-0.1952	-1.9411	3.7001
x530	1.2447	1.9286	1.1444
y530	-0.9537	-1.3339	-0.3644
z530	-1.1406	-2.5854	2.3235

x531	0.5048	1.3457	0.4053
y531	-1.0470	-0.3513	-1.2398
z531	-1.5884	-2.5476	1.7807
x532	-0.1053	1.0997	-0.1183
y532	-1.0526	0.4377	-1.9292
z532	-2.0001	-2.7601	1.2036
x533	-0.6090	0.6828	-0.5212
y533	-0.9297	1.0934	-2.4654
z533	-2.2768	-2.7455	0.7767
x534	-1.2648	0.3154	-1.0236
y534	-0.7457	1.3367	-2.6728
z534	-2.2041	-2.5028	0.5916
x535	-1.7293	-0.3732	-1.4596
y535	-1.4702	0.1069	-2.7403
z535	-1.9688	-2.1440	0.4894
x536	-1.8845	-0.3205	-1.9560
y536	-1.5499	-0.1761	-1.7677
z536	-2.3719	-2.6937	-0.0627
x537	-1.8346	-0.0287	-2.0111
y537	-1.2589	-0.1850	-1.0961
z537	-2.9278	-3.3708	-0.7634
x538	-1.9659	-0.2153	-2.1940
y538	-1.1498	-0.5750	-0.7345
z538	-2.6875	-2.4427	-0.7851
x539	-2.0541	-0.1169	-2.2515
y539	-1.1069	-1.2279	-0.5784
z539	-2.3327	-1.6496	-0.9312
x540	-1.9452	0.1356	-2.4284

y540	-1.1653	-1.9736	-0.5198
z540	-2.3139	-1.0396	-0.9410
x541	-1.6259	0.3006	-2.8311
y541	-1.3981	-2.6680	-0.3896
z541	-2.2855	0.0595	-0.6553
x542	-0.9893	0.4419	-3.0022
y542	-1.7545	-3.2120	-0.5649
z542	-2.2136	0.7926	-0.9009
x543	-0.3490	0.4083	-2.9232
y543	-2.0406	-3.5160	-0.8864
z543	-1.8082	1.4147	-1.0786
x544	-0.2877	-0.5419	-2.8307
y544	-1.8589	-3.0856	-1.0787
z544	-1.5925	2.4604	-1.2703
x545	0.1126	-0.5573	-1.5312
y545	-2.2749	-3.9236	-2.4335
z545	-1.9563	1.9108	-2.2086
x546	0.6259	-1.1262	-0.9395
y546	-2.3187	-3.7409	-2.3648
z546	-1.8276	2.3562	-1.5441
x547	0.5128	-1.4358	-0.4889
y547	-2.2318	-4.2646	-2.8184
z547	-1.6836	1.8415	-1.6006
x548	0.6382	-1.3563	0.1588
y548	-2.1390	-4.7871	-3.4483
z548	-1.5301	1.3820	-1.7646
x549	0.7453	-1.7604	0.1791
y549	-1.6176	-4.4135	-3.2700

z549	-1.1820	1.2156	-1.5575
x550	0.6584	-2.2017	0.0345
y550	-0.9014	-3.7494	-2.8742
z550	-0.9278	1.0297	-1.2895
x551	0.9387	-1.9778	0.3292
y551	-0.9498	-4.0962	-3.1178
z551	-0.9593	0.5586	-1.1448
x552	1.1465	-2.1223	0.3467
y552	-1.3518	-3.4225	-3.0160
z552	-1.4208	0.2221	-1.0866
x553	1.4785	-2.4109	1.1725
y553	-1.2628	-3.0735	-3.6115
z553	-1.4343	-0.2504	-0.7828
x554	1.8095	-2.5780	1.9201
y554	-0.9506	-2.9671	-3.5912
z554	-1.4095	-0.9986	-0.0659
x555	1.5755	-2.2982	2.0699
y555	-0.8426	-2.7605	-3.0925
z555	-1.7619	-1.3427	0.5098
x556	1.2802	-1.9261	2.1730
y556	-0.9777	-3.2170	-3.5487
z556	-2.0886	-1.5347	1.1145
x557	1.2298	-1.4206	2.1296
y557	-0.1737	-2.5486	-3.0051
z557	-2.2404	-1.5508	1.7252
x558	1.1972	-0.8197	2.0912
y558	0.2286	-2.4959	-2.4128
z558	-2.3027	-1.4804	2.2698

x559	1.0269	-0.3802	1.9038
y559	0.3181	-2.7297	-2.0664
z559	-2.4017	-1.4852	2.6824
x560	1.0374	0.3343	1.5483
y560	0.4608	-2.0164	-1.5714
z560	-2.5725	-1.8092	2.8485
x561	1.0763	1.0697	1.4173
y561	0.1981	-1.9219	-1.6060
z561	-2.6667	-2.1107	2.9016
x562	1.3146	1.8293	1.6467
y562	-0.1518	-2.3891	-1.5731
z562	-2.8688	-2.2058	2.6283
x563	1.6538	1.8552	0.6891
y563	0.7871	-2.0396	0.3325
z563	-1.7792	-1.3806	2.7335
x564	2.0823	2.1425	0.4090
y564	1.3057	-2.1120	1.0649
z564	-0.9291	-0.7197	2.2168
x565	2.3680	2.1630	0.4189
y565	1.4684	-2.5127	1.2904
z565	-0.1816	-0.2392	1.7351
x566	2.4066	2.9166	0.7411
y566	1.2579	-2.2538	1.3535
z566	0.4459	-0.6277	1.0482
x567	2.7984	2.5562	1.1505
y567	1.0198	-2.4063	1.0934
z567	0.8277	-0.6765	0.5643
x568	2.7252	1.3511	1.2003

y568	0.4705	-2.7007	0.4263
z568	1.3182	-0.5345	0.3414
x569	2.9851	1.3679	1.7727
y569	-0.1551	-2.8295	-0.3900
z569	1.6802	-1.3337	0.0815
x570	2.9179	-0.2592	1.9463
y570	-0.8987	-2.8573	-1.1745
z570	2.2227	-1.7798	0.1115
x571	2.2901	-1.4227	1.9672
y571	-1.7318	-2.6801	-1.5545
z571	2.8179	-2.3156	0.0008
x572	1.9986	-1.1072	1.9389
y572	-0.9117	-1.8184	-2.1740
z572	2.0249	-0.5642	-0.9585
x573	2.0443	-1.5328	1.6775
y573	-0.6194	-0.3374	-2.3713
z573	1.1236	1.0491	-1.8389
x574	1.7486	-1.7338	1.4464
y574	-0.4750	1.0238	-2.4902
z574	0.4284	2.2197	-2.3819
x575	1.6502	-0.9989	1.4920
y575	-0.8313	1.2577	-2.8270
z575	-0.1006	3.0041	-2.5262
x576	1.0972	-0.4051	1.0079
y576	-0.6954	1.8216	-2.4650
z576	-0.4883	3.6741	-2.4571
x577	0.6577	0.1002	0.7839
y577	-0.9459	2.6446	-2.6040

z577	-0.6694	4.1372	-1.9673
x578	0.0690	0.5064	0.5112
y578	-0.6082	4.0025	-2.3617
z578	-0.5280	4.3899	-1.0129
x579	-0.6562	0.6731	0.2220
y579	-0.2061	4.8877	-0.5983
z579	-0.2489	4.0529	0.1016
x580	-1.0996	0.9269	0.5342
y580	0.0328	4.8051	-0.1451
z580	0.4846	4.1356	2.5070
x581	-0.2313	0.2552	0.8011
y581	0.6900	5.1916	1.5792
z581	1.6488	2.1704	2.7154
x582	0.2776	-0.4666	0.8649
y582	0.6127	4.5756	2.0252
z582	2.6135	0.7999	2.9191
x583	0.5093	-1.7714	0.9999
y583	0.3471	3.1548	2.0421
z583	3.3324	0.1195	3.1239
x584	0.5743	-2.3965	0.5764
y584	0.0670	2.4911	1.6937
z584	3.6771	-0.7465	3.2121
x585	0.8542	-3.0825	0.5251
y585	0.1306	1.8161	1.9585
z585	3.4180	-1.6007	2.2305
x586	0.7587	-2.3309	0.0571
y586	0.1610	1.5752	2.0698
z586	3.1281	-2.9620	1.7176

x587	0.7263	-2.4017	-1.0122
y587	0.1002	0.8583	1.8653
z587	3.1150	-2.9382	2.3941
x588	0.6601	-2.5618	-1.8794
y588	-0.0335	0.3966	1.6754
z588	2.5913	-2.3565	2.8133
x589	0.4248	-1.9630	-2.4465
y589	0.0412	0.1217	1.8568
z589	0.6746	-2.2498	1.6389
x590	-0.0691	-2.3511	-2.8671
y590	0.3223	0.6641	2.4036
z590	-0.0513	-2.5659	2.1465
x591	-0.4864	-2.6387	-3.3958
y591	0.5578	1.1728	3.1327
z591	-0.3282	-3.0542	2.4026
x592	-0.4253	-3.1011	-3.4495
y592	0.2553	2.2103	3.2561
z592	-0.6845	-2.9190	2.0639
x593	-0.1414	-2.8495	-3.0248
y593	-0.2540	2.5502	2.9450
z593	-0.7731	-2.8823	1.6622
x594	0.0056	-2.2485	-2.9010
y594	-0.4447	2.1589	3.3110
z594	-0.6502	-2.9247	1.2111
x595	0.1384	-1.6186	-2.7227
y595	-0.7639	1.8346	3.5721
z595	-0.2183	-2.4360	0.8654
x596	0.1526	-0.7587	-2.3383

y596	-1.1359	0.9787	2.8475
z596	0.5152	-1.3930	0.2450
x597	-0.0080	-0.1037	-1.6809
y597	-1.3354	1.0383	2.4235
z597	1.7079	0.1925	-0.2889
x598	-0.2002	0.3004	-0.4938
y598	-1.1239	1.5439	1.4510
z598	3.1819	1.8730	-0.2991
x599	-0.1188	1.3637	-0.0454
y599	-1.0611	1.7817	1.7656
z599	2.6892	1.1380	-0.8312
x600	-0.1364	1.4979	0.4312
y600	-1.1624	1.0250	1.5779
z600	2.2270	0.8868	-1.0471
x601	-0.2554	1.3212	0.7918
y601	-1.1909	0.5138	1.0799
z601	1.6693	0.6624	-1.2382
x602	-0.2965	1.1153	1.4974
y602	-1.0649	0.4007	0.8937
z602	1.1826	0.7517	-1.4405
x603	-0.4834	0.7545	1.9550
y603	-0.9367	0.3716	0.5055
z603	0.8314	1.2002	-1.6837
x604	-0.4064	0.6241	2.2406
y604	-0.6235	0.5992	0.0512
z604	0.3292	1.7864	-1.9210
x605	-0.4746	-0.7212	2.0618
y605	-0.2150	0.9015	-0.3779

z605	0.0672	2.8007	-1.8767
x606	0.0233	-1.1035	2.7532
y606	0.1664	1.3818	-0.6689
z606	-0.4529	3.1885	-1.9436
x607	0.5605	-0.9351	3.0919
y607	0.4647	1.6442	-0.6087
z607	-1.0406	3.0262	-2.1351
x608	0.9775	-0.9716	3.3592
y608	0.5315	2.0829	-0.6700
z608	-0.7868	3.7983	-1.0333
x609	1.3600	-0.9036	3.4715
y609	0.4509	2.1486	-0.8743
z609	-0.7475	4.1585	-0.5748
x610	1.5742	-0.8748	3.1920
y610	0.4473	2.0540	-0.7950
z610	-0.8752	4.3223	-0.3633
x611	1.7816	-1.3108	2.7636
y611	0.4213	2.7884	-0.7103
z611	-1.0339	4.6635	-0.3380
x612	1.9676	-1.5997	2.2453
y612	0.2614	3.6411	-0.6150
z612	-1.3154	4.8632	-0.6326
x613	2.1577	-1.5922	1.8174
y613	-0.0718	4.5341	-1.1652
z613	-1.6412	4.8936	-1.0724
x614	2.3440	-1.2566	1.4023
y614	-0.6307	4.6832	-1.4108
z614	-1.9387	4.8291	-1.6073

x615	2.4419	-0.6961	0.9661
y615	-1.3060	5.2343	-1.5090
z615	-2.2059	4.5589	-2.3449
x616	2.0716	0.2639	0.4538
y616	-1.9675	6.0193	-0.9651
z616	-2.4126	4.0129	-3.4344
x617	1.2724	1.1104	-0.4170
y617	-2.1277	5.6513	-1.5133
z617	-0.2149	2.9621	-2.2174
x618	0.8692	1.6684	-0.5649
y618	-1.1867	4.9697	-0.8401
z618	1.9718	2.0830	-1.1843
x619	0.7417	2.2069	-0.5113
y619	0.0540	4.5063	0.2098
z619	2.2444	0.2812	-1.3934
x620	0.3583	2.3458	-1.1545
y620	1.6789	4.0575	0.9740
z620	1.5279	-1.0680	-1.9298
x621	0.4084	2.7439	-2.0090
y621	2.6222	2.8605	1.4038
z621	3.2009	0.1164	-1.4684
x622	1.1713	3.6580	-2.4137
y622	3.1895	1.2103	1.6331
z622	3.4213	0.8867	-1.6195
x623	2.7053	4.0087	-1.2691
y623	3.7876	-0.0635	1.6773
z623	3.0563	2.6829	-2.4429
x624	4.5319	3.8610	0.5254

y624	4.4862	-2.1254	2.5552
z624	0.9489	4.4913	-4.1563
x625	5.0414	3.8781	0.6521
y625	3.6835	-3.4122	2.7809
z625	-1.6305	6.4389	-6.7836
x626	-6.5256	-3.0436	-8.4923
y626	0.7156	-0.3023	-0.2683
z626	-2.9998	-1.1886	3.2551
x627	-5.1640	-3.8299	-6.5889
y627	0.1073	0.3749	0.6284
z627	-2.3068	-2.2116	2.6708
x628	-4.0503	-3.7864	-4.3871
y628	-0.4869	0.5355	0.5011
z628	-1.8048	-2.3413	1.4383
x629	-3.0872	-2.7316	-2.6819
y629	-1.0341	0.6816	0.1447
z629	-1.5196	-2.4736	0.2131
x630	-2.1785	-1.8998	-1.6635
y630	-1.3950	0.0846	-0.5506
z630	-1.5792	-2.1752	-0.4736
x631	-1.3548	-1.1806	-1.1501
y631	-1.9149	-0.5256	-1.1119
z631	-1.3790	-2.2645	-0.6594
x632	-0.7281	-0.4977	-1.0323
y632	-2.2964	-1.8024	-1.6647
z632	-1.1902	-1.6075	-0.4024
x633	-0.3499	-0.2835	-1.2825
y633	-2.3543	-2.7012	-1.7634

z633	-1.6419	-2.2322	-1.0473
x634	0.0653	-0.0500	-1.1507
y634	-2.5742	-4.0676	-2.2639
z634	-1.5186	-2.2410	-0.6494
x635	-0.0353	-1.1904	-1.5635
y635	-2.5234	-3.8303	-2.0377
z635	-1.7237	-0.6809	0.0504
x636	0.1071	-2.3408	-1.5374
y636	-2.8390	-3.4154	-2.0152
z636	-2.0430	0.1432	0.8402
x637	0.0473	-2.5163	-1.4516
y637	-3.2005	-4.5756	-2.6960
z637	-2.8920	-1.0461	-0.4707
x638	0.2856	-1.5097	-0.1858
y638	-3.6217	-5.6625	-3.7534
z638	-3.1900	-0.4607	-0.2239
x639	0.2215	-0.8304	0.6573
y639	-4.1823	-6.4650	-5.0891
z639	-3.6348	-0.1805	-0.2462
x640	0.3055	-1.0002	0.9987
y640	-4.7458	-6.9738	-6.5454
z640	-3.8139	-0.6147	-0.5506
x641	-0.3740	-1.6714	0.3171
y641	-5.0661	-7.2452	-7.6998
z641	-4.7355	-1.4331	-1.6028
x642	0.2820	-0.5957	1.1689
y642	-4.9634	-6.9229	-8.1452
z642	-4.3339	-0.3753	-0.7363

x643	0.3593	-0.8946	0.2621
y643	-4.4541	-6.3448	-8.1430
z643	-4.2926	-0.5097	-1.0806
x644	0.8923	-0.7727	0.0558
y644	-1.5211	-3.4640	-5.1199
z644	-3.3372	0.1870	0.0585
x645	1.0273	-0.7066	-0.9969
y645	0.0108	-2.7303	-4.6769
z645	-3.0940	0.4817	0.4397
x646	0.7298	-1.1542	-2.2913
y646	0.0163	-3.4668	-5.7780
z646	-3.0877	0.3080	0.8880
x647	0.4940	-1.2284	-3.3832
y647	-0.1097	-4.3075	-6.2329
z647	-3.2020	0.7820	1.2054
x648	0.7386	-1.3507	-3.6929
y648	-0.2988	-4.7936	-5.8358
z648	-2.8640	1.5269	1.7239
x649	0.9323	-1.6612	-3.7901
y649	-0.2334	-4.8856	-5.0188
z649	-2.0908	1.7086	2.2973
x650	0.9079	-2.0432	-4.1334
y650	0.1163	-4.6567	-3.3111
z650	-1.6272	1.1533	1.9226
x651	1.2933	-1.7296	-3.6982
y651	0.4746	-4.0290	-1.8444
z651	-1.3033	0.4633	1.5311
x652	1.7778	-0.8679	-3.1001

y652	0.6189	-3.2228	-0.6677
z652	-1.3502	-1.0965	0.2103
x653	2.4403	1.1659	-1.9396
y653	0.3602	-2.2824	0.0943
z653	-1.3314	-3.2209	-1.2635
x654	3.2623	1.1585	-2.0446
y654	0.0331	-2.2948	-0.0989
z654	-1.4719	-3.5173	-2.5411
x655	3.6747	0.6523	-2.0650
y655	-0.3933	-3.0151	-0.7023
z655	-1.5433	-4.1303	-3.7027
x656	3.5878	0.5863	-1.4885
y656	-0.7290	-4.0070	-1.1872
z656	-1.8827	-3.9241	-3.2370
x657	3.2032	0.0471	-1.1192
y657	-0.7479	-5.4307	-1.7127
z657	-1.9991	-3.2162	-2.7352
x658	2.8473	-0.7773	-0.8704
y658	-0.6997	-5.0962	-1.7677
z658	-1.6818	-2.1447	-2.6417
x659	2.5574	-1.2169	-0.0732
y659	-0.6333	-3.2364	-1.2733
z659	-1.1985	-0.9741	-2.0320
x660	1.8668	-1.9364	0.1179
y660	-0.2712	-1.5567	-0.8809
z660	-1.5706	-0.4086	-2.5157
x661	1.3730	-2.6044	0.2781
y661	-0.0030	-0.0815	-0.5718

z661	-1.8501	0.1861	-2.6306
x662	1.4095	-2.7821	0.6335
y662	-0.1914	1.0113	-0.4041
z662	-1.8304	1.0761	-2.0004
x663	0.7871	-2.9481	0.2173
y663	0.3746	1.2905	0.8574
z663	-2.3386	1.2684	-1.5983
x664	0.4846	-2.6523	0.0987
y664	0.9421	2.0202	1.6172
z664	-2.2136	2.2185	-0.8334
x665	0.3576	-2.3780	0.1800
y665	1.2413	2.9565	1.6332
z665	-1.6755	2.8246	0.2197
x666	-0.2349	-2.4616	-0.6286
y666	1.4992	3.8943	1.4864
z666	-1.6295	2.8688	0.3951
x667	-0.2878	-2.3314	-1.1617
y667	1.2890	4.5466	0.7614
z667	-0.8681	2.1806	0.9809
x668	-0.5790	-1.8620	-2.0777
y668	0.8407	4.6829	-0.1733
z668	-0.3761	0.8027	0.6689
x669	-0.9813	-1.3047	-3.2552
y669	0.4850	4.4496	-0.8046
z669	0.8017	-0.2357	0.9302
x670	-1.6354	-0.3824	-4.2336
y670	0.5393	3.8105	-0.6474
z670	1.9448	-0.5430	1.4580

x671	-1.4022	-0.8797	-4.3260
y671	1.4920	2.3407	0.9678
z671	1.9382	-1.1594	0.3950
x672	-0.5338	0.5421	-4.0017
y672	2.3589	0.7934	2.1758
z672	1.9135	-0.7782	0.5789
x673	-0.6092	1.5696	-4.8623
y673	2.5974	-0.5459	3.0083
z673	1.2174	-1.4646	0.1737
x674	0.0312	1.9627	-3.9880
y674	2.2167	-1.5302	3.0394
z674	0.4005	-1.7538	1.2923
x675	0.5486	1.4656	-3.5819
y675	1.4626	-2.0151	2.9992
z675	-0.8364	-2.5050	0.3314
x676	1.1210	1.1124	-2.6425
y676	0.4703	-2.4694	2.4244
z676	-1.4281	-2.1807	0.7359
x677	1.3916	0.5028	-1.9664
y677	-0.3912	-2.8365	1.9592
z677	-1.7553	-2.3739	0.8666
x678	1.2905	0.4168	-1.2730
y678	-0.9850	-3.2869	1.6447
z678	-1.7011	-1.7483	1.8141
x679	1.0247	0.4273	-1.1051
y679	-1.0155	-3.9737	1.7784
z679	-0.5954	-1.6349	2.3839
x680	0.2682	0.9511	-1.5301

y680	-0.2234	-5.0292	2.7529
z680	0.3186	-1.6465	3.1296
x681	0.4820	-0.6595	-0.1715
y681	-0.2100	-4.5427	2.6772
z681	0.6556	-0.7892	2.9114
x682	0.4828	-1.6613	0.5366
y682	-0.1875	-3.9267	2.4559
z682	0.5620	-0.2129	2.3596
x683	0.5197	-2.2976	0.9613
y683	-0.1430	-3.2030	2.0627
z683	0.4241	-0.0807	2.1488
x684	0.5081	-2.4035	0.9691
y684	-0.1164	-2.2868	1.5357
z684	0.5747	0.4878	1.9066
x685	0.4987	-2.3335	0.7658
y685	-0.0316	-1.3176	0.9917
z685	0.6120	0.8394	1.7299
x686	0.3157	-2.2503	0.3728
y686	0.0437	-0.2809	0.4656
z686	0.5686	1.1822	1.4462
x687	0.1800	-2.0443	0.2436
y687	0.1862	0.7992	0.1025
z687	0.4248	1.5474	1.4186
x688	0.4583	-1.9866	0.5997
y688	0.4749	1.9539	-0.1256
z688	0.9865	1.9375	1.4495
x689	0.4453	-2.7901	1.2350
y689	0.7171	3.1892	-0.3308

z689	0.7086	1.5720	1.0826
x690	0.9817	-3.1496	1.3023
y690	0.4415	3.3771	-0.4367
z690	0.6344	2.3293	0.9974
x691	1.1553	-3.6925	1.0626
y691	0.1381	3.3337	-0.4864
z691	0.2404	2.3480	0.7781
x692	1.2332	-3.7425	0.6401
y692	0.0311	3.0976	-0.4161
z692	0.0367	2.5585	0.3781
x693	1.2440	-3.8498	0.3061
y693	-0.0252	2.7257	-0.3114
z693	-0.1218	2.4678	0.2694
x694	1.3269	-3.6560	0.0805
y694	-0.1233	2.3313	-0.3652
z694	-0.1101	2.6837	0.2006
x695	1.5134	-3.7101	-0.0436
y695	-0.4883	2.0603	-0.7079
z695	-0.0367	2.4064	0.1709
x696	1.5122	-3.5726	-0.1727
y696	-1.0101	2.2132	-1.2281
z696	0.0612	2.3894	0.4124
x697	1.2047	-3.2886	-0.7556
y697	-1.6904	2.7377	-2.0661
z697	0.1055	2.0198	0.2314
x698	0.3130	-2.6091	-2.1126
y698	-2.5956	3.4682	-3.2599
z698	0.2845	1.3507	-0.2228

x699	-1.6263	-2.2901	-3.9094
y699	-3.7213	2.1362	-3.3083
z699	1.9130	1.5592	1.1361
x700	-3.1891	-1.3380	-5.8573
y700	-3.5444	1.1214	-2.9620
z700	2.4070	0.7011	1.7818
x701	-3.4406	0.4992	-6.9075
y701	-2.4991	-0.0795	-2.0325
z701	2.4672	0.0067	1.9633
x702	-2.4135	2.0558	-6.8098
y702	-1.2897	-1.6132	-0.8362
z702	2.5048	0.2075	1.9329
x703	-1.0558	2.5799	-5.9926
y703	-0.2567	-2.6365	0.0564
z703	2.1885	0.6664	1.3123
x704	0.1375	2.7721	-4.9903
y704	0.5799	-3.2965	0.7032
z704	1.5410	1.2274	0.1523
x705	1.6644	2.9808	-3.5591
y705	1.7204	-3.7473	1.5743
z705	0.7791	2.0991	-1.2583
x706	3.7534	3.5042	-1.2367
y706	3.2347	-3.5739	2.5207
z706	0.2692	4.2156	-3.1353
x707	5.0414	3.8781	0.6521
y707	3.6835	-3.4122	2.7809
z707	-1.6305	6.4389	-6.7836
x708	-3.5702	-3.4866	0.3897

y708	-0.0332	0.9365	-2.0138
z708	-3.3662	-0.5015	-0.0216
x709	-2.4696	-2.3494	2.9899
y709	0.9349	0.6036	0.7712
z709	-3.6444	-0.9185	-1.3118
x710	-2.3059	-1.3296	3.7417
y710	0.9964	-0.0726	1.8319
z710	-3.3112	-1.6454	-0.4908
x711	-3.0064	-1.5041	3.7865
y711	0.8284	-0.1822	1.8706
z711	-2.4472	-1.8108	0.8659
x712	-3.4886	-1.7245	3.6733
y712	0.2478	-0.1698	1.4827
z712	-2.0210	-2.5671	2.3516
x713	-3.8841	-3.0713	5.1885
y713	0.0237	1.7523	1.7032
z713	-2.0208	-1.9220	2.4889
x714	-4.4934	-4.6523	6.7104
y714	-0.3079	3.8156	2.0925
z714	-1.8751	-0.4621	2.5659
x715	-6.5348	-0.8776	7.2507
y715	0.0347	4.8498	2.7329
z715	-1.6373	-3.2660	3.6107
x716	-6.0708	-1.2391	9.1243
y716	1.3557	7.4496	3.1342
z716	-2.1871	-3.7696	5.3147
x717	-5.7337	-2.3200	10.1674
y717	1.1075	8.2700	2.1742

z717	-1.0233	-3.0666	7.5868

New Procrustes fit:

Dataset 'BE_Canals'

256 landmarks in 3 dimensions.

The dataset contains 32 observations, of which 32 are included for analyses.

Average shape:

Lmk.	Axis 1 (x)	Axis 2 (y)	Axis 3 (z)
1	-0.04141995	0.02935969	0.00749175
2	0.04263838	0.05411971	-0.00301215
3	0.04174563	0.05473665	-0.00035387
4	0.03980603	0.05547906	0.00236344
5	0.03718705	0.05620669	0.00465257
6	0.03419929	0.05677374	0.00674637
7	0.03089988	0.05718168	0.00870453
8	0.02725099	0.05729037	0.01053089
9	0.02331524	0.05726811	0.01210709
10	0.01921243	0.05708083	0.01363220
11	0.01481087	0.05682857	0.01506394
12	0.01027788	0.05635569	0.01607772
13	0.00588568	0.05564811	0.01677962
14	0.00146915	0.05478802	0.01711935
15	-0.00283056	0.05367537	0.01708481
16	-0.00711146	0.05242784	0.01656877
17	-0.01127782	0.05093795	0.01580340
18	-0.01517331	0.04932435	0.01452689
19	-0.01871124	0.04755739	0.01275216
20	-0.02229628	0.04563512	0.01071807
21	-0.02662878	0.04305404	0.00723379

22	-0.03003878	0.04037557	0.00352860
23	-0.03271640	0.03763834	-0.00064113
24	-0.03409231	0.03506855	-0.00569782
25	-0.03396297	0.03297598	-0.01110329
26	-0.03302098	0.03076462	-0.01726775
27	-0.03104222	0.02831511	-0.02414112
28	-0.02777722	0.02620050	-0.03155475
29	-0.02322985	0.02480699	-0.03903098
30	-0.01841769	0.02396839	-0.04343437
31	-0.01235468	0.02452350	-0.04619867
32	-0.00652648	0.02600868	-0.04684400
33	-0.00039998	0.02790652	-0.04656777
34	0.00611680	0.03008515	-0.04547111
35	0.01302220	0.03245916	-0.04356053
36	0.02012250	0.03489250	-0.04097670
37	0.02726003	0.03722580	-0.03759602
38	0.03248973	0.04030713	-0.03286818
39	0.03353623	0.04211001	-0.03006134
40	0.03427540	0.04399113	-0.02664891
41	0.03504705	0.04591501	-0.02302587
42	0.03606300	0.04774068	-0.01958165
43	0.03742973	0.04933233	-0.01626169
44	0.03923010	0.05056043	-0.01294660
45	0.04111709	0.05154230	-0.00964424
46	0.04243682	0.05266090	-0.00611404
47	0.06219070	0.05896799	0.00028097
48	0.06073710	0.05921882	0.00191550
49	0.05899481	0.05948287	0.00359692

50	0.05675810	0.05982849	0.00549356
51	0.05417012	0.06016242	0.00740483
52	0.05116089	0.06038283	0.00934500
53	0.04789119	0.06063020	0.01135801
54	0.04440868	0.06086716	0.01329498
55	0.04077485	0.06096902	0.01524031
56	0.03666842	0.06132823	0.01736874
57	0.03259543	0.06144913	0.01913898
58	0.02855463	0.06142749	0.02078117
59	0.02464428	0.06124747	0.02220724
60	0.02070302	0.06097765	0.02347843
61	0.01679002	0.06046373	0.02429342
62	0.01294095	0.06017878	0.02536622
63	0.00921906	0.05963516	0.02602802
64	0.00540200	0.05892663	0.02648693
65	0.00162103	0.05832040	0.02675589
66	-0.00192145	0.05762950	0.02670003
67	-0.00543484	0.05689367	0.02647047
68	-0.00885962	0.05602967	0.02599774
69	-0.01220296	0.05511595	0.02529541
70	-0.01541971	0.05414327	0.02435090
71	-0.01843032	0.05291685	0.02314905
72	-0.02149032	0.05147967	0.02164975
73	-0.02452244	0.05015891	0.02029506
74	-0.02717483	0.04866344	0.01844178
75	-0.02938572	0.04726557	0.01671187
76	-0.03173670	0.04590149	0.01501244
77	-0.03397371	0.04432921	0.01299760

78	-0.03607791	0.04259073	0.01063406
79	-0.03822764	0.04072211	0.00798021
80	-0.04042997	0.03886523	0.00514962
81	-0.04287176	0.03712860	0.00213291
82	-0.04550669	0.03540696	-0.00114185
83	-0.04795965	0.03356039	-0.00473204
84	-0.04465184	-0.03316798	-0.01390263
85	-0.04429866	-0.03409316	-0.01170400
86	-0.04415302	-0.03461194	-0.00894954
87	-0.04432117	-0.03449488	-0.00623640
88	-0.04464684	-0.03400414	-0.00352368
89	-0.04505660	-0.03317170	-0.00110964
90	-0.04558605	-0.03210176	0.00126006
91	-0.04621195	-0.03080464	0.00339530
92	-0.04683791	-0.02939435	0.00555162
93	-0.04748008	-0.02765229	0.00762184
94	-0.04796475	-0.02565539	0.00967304
95	-0.04850300	-0.02344599	0.01141944
96	-0.04908280	-0.02096578	0.01289290
97	-0.04954086	-0.01834074	0.01417511
98	-0.04994605	-0.01551603	0.01522256
99	-0.05033822	-0.01251259	0.01602543
100	-0.05061099	-0.00928531	0.01646293
101	-0.05080568	-0.00597082	0.01647199
102	-0.05111353	-0.00269187	0.01603443
103	-0.05101302	0.00109964	0.01496078
104	-0.05067126	0.00464523	0.01318066
105	-0.05024427	0.00781754	0.01116630

106	-0.04982913	0.01060805	0.00882345
107	-0.04918682	0.01318829	0.00615765
108	-0.04847038	0.01513199	0.00349254
109	-0.04760015	0.01705868	0.00029368
110	-0.04683251	0.01827800	-0.00325955
111	-0.04608691	0.01886940	-0.00706846
112	-0.04357131	0.01816739	-0.01539512
113	-0.04186544	0.01603132	-0.02268167
114	-0.04032061	0.01367715	-0.02861545
115	-0.03949728	0.01030387	-0.03325136
116	-0.03899987	0.00714856	-0.03625343
117	-0.03832724	0.00360684	-0.03864428
118	-0.03830087	-0.00069653	-0.03981432
119	-0.03931824	-0.00599553	-0.03924776
120	-0.04121863	-0.01078293	-0.03693943
121	-0.04334156	-0.01321702	-0.03503727
122	-0.04536857	-0.01550923	-0.03244715
123	-0.04697523	-0.01776204	-0.02962731
124	-0.04796169	-0.02025596	-0.02685709
125	-0.04826189	-0.02297413	-0.02415997
126	-0.04781179	-0.02592044	-0.02169801
127	-0.04691355	-0.02880146	-0.01919779
128	-0.04591099	-0.03101338	-0.01697088
129	-0.04650941	-0.05072280	-0.01801442
130	-0.04597095	-0.05062362	-0.01552130
131	-0.04554710	-0.05040437	-0.01314335
132	-0.04509578	-0.04982539	-0.01023914
133	-0.04484715	-0.04925445	-0.00731912

134	-0.04485058	-0.04876331	-0.00458298
135	-0.04484760	-0.04806500	-0.00200382
136	-0.04493916	-0.04706657	0.00052912
137	-0.04518442	-0.04598884	0.00300851
138	-0.04568263	-0.04465803	0.00533570
139	-0.04663593	-0.04281305	0.00827796
140	-0.04740078	-0.04080127	0.01113918
141	-0.04806092	-0.03857832	0.01365858
142	-0.04871322	-0.03649461	0.01593642
143	-0.04925982	-0.03408514	0.01783141
144	-0.04981617	-0.03144501	0.01965235
145	-0.05041730	-0.02891573	0.02158394
146	-0.05098142	-0.02643322	0.02314952
147	-0.05149496	-0.02356574	0.02449155
148	-0.05194269	-0.02053281	0.02538550
149	-0.05249890	-0.01742140	0.02623767
150	-0.05286831	-0.01425125	0.02664764
151	-0.05317580	-0.01084149	0.02692065
152	-0.05348134	-0.00749730	0.02692366
153	-0.05372683	-0.00415490	0.02656524
154	-0.05390877	-0.00085218	0.02596911
155	-0.05398993	0.00249039	0.02496143
156	-0.05394814	0.00562961	0.02405383
157	-0.05382760	0.00942568	0.02204858
158	-0.05363764	0.01310951	0.01938789
159	-0.05334229	0.01652624	0.01656810
160	-0.05265093	0.01971405	0.01335286
161	-0.05170712	0.02268356	0.01008197

162	-0.05082148	0.02544972	0.00683779
163	-0.04987199	0.02814909	0.00323233
164	-0.04894866	0.03092117	-0.00067780
165	-0.04795965	0.03356039	-0.00473204
166	0.07056616	-0.01068682	-0.00892163
167	0.07175760	-0.01321056	-0.00724446
168	0.07214541	-0.01605492	-0.00528434
169	0.07200054	-0.01885704	-0.00341407
170	0.07121121	-0.02165384	-0.00146916
171	0.07002864	-0.02455456	0.00041394
172	0.06842070	-0.02749732	0.00220899
173	0.06622290	-0.03051919	0.00399487
174	0.06365187	-0.03361853	0.00576386
175	0.06062475	-0.03667317	0.00740956
176	0.05810666	-0.03888812	0.00864993
177	0.05539227	-0.04102518	0.00972933
178	0.05251385	-0.04293453	0.01062575
179	0.04947033	-0.04447234	0.01132218
180	0.04642511	-0.04589815	0.01184298
181	0.04320319	-0.04726567	0.01220685
182	0.03983977	-0.04836468	0.01239410
183	0.03637054	-0.04916877	0.01236580
184	0.03235232	-0.04979441	0.01213765
185	0.02863375	-0.04997766	0.01174764
186	0.02493824	-0.04999175	0.01110022
187	0.02125453	-0.04978633	0.01015159
188	0.01735602	-0.04949413	0.00890364
189	0.01385677	-0.04875699	0.00765412

190	0.01043524	-0.04790143	0.00619627
191	0.00706795	-0.04680922	0.00456216
192	0.00351097	-0.04561828	0.00249403
193	0.00033874	-0.04461140	0.00024054
194	-0.00449681	-0.04303065	-0.00337946
195	-0.00845861	-0.04072390	-0.00721210
196	-0.01160677	-0.03805440	-0.01134756
197	-0.01395775	-0.03518073	-0.01565911
198	-0.01535183	-0.03186341	-0.02027666
199	-0.01577705	-0.02802772	-0.02503512
200	-0.01564063	-0.02404369	-0.02992133
201	-0.01477189	-0.01960726	-0.03471592
202	-0.01291118	-0.01444753	-0.03978204
203	-0.00894108	-0.00823206	-0.04355956
204	-0.00344960	-0.00269439	-0.04530121
205	0.00212031	0.00183929	-0.04561739
206	0.00823427	0.00553474	-0.04528930
207	0.01477786	0.00833916	-0.04455672
208	0.02187281	0.01030498	-0.04264590
209	0.02929199	0.01122686	-0.03923269
210	0.03659574	0.01098425	-0.03439908
211	0.04329808	0.00953429	-0.02815946
212	0.04660998	0.00773075	-0.02465190
213	0.04966285	0.00543132	-0.02139387
214	0.05275201	0.00300575	-0.01845855
215	0.05610996	0.00065121	-0.01602961
216	0.05949316	-0.00146475	-0.01420430
217	0.06287148	-0.00367320	-0.01264192

218	0.06607082	-0.00574278	-0.01151598
219	0.06852679	-0.00805867	-0.01032698
220	0.09154619	-0.00256635	-0.00731781
221	0.09106538	-0.00656481	-0.00538836
222	0.09019448	-0.01044118	-0.00359890
223	0.08911599	-0.01413545	-0.00188788
224	0.08778662	-0.01761937	-0.00034000
225	0.08633178	-0.02100194	0.00116468
226	0.08474899	-0.02418458	0.00248270
227	0.08304786	-0.02712416	0.00377989
228	0.08126539	-0.02993694	0.00513549
229	0.07954950	-0.03233102	0.00646558
230	0.07747552	-0.03498296	0.00806846
231	0.07522930	-0.03746770	0.00962097
232	0.07284833	-0.03992759	0.01118821
233	0.07027600	-0.04240745	0.01248552
234	0.06759303	-0.04471699	0.01375292
235	0.06475657	-0.04697978	0.01495018
236	0.06182680	-0.04909507	0.01608286
237	0.05871545	-0.05111639	0.01702674
238	0.05565401	-0.05307950	0.01793772
239	0.05156252	-0.05495752	0.01851156
240	0.04744259	-0.05669657	0.01915729
241	0.04336201	-0.05795303	0.01937187
242	0.03884108	-0.05889288	0.01931769
243	0.03440076	-0.05963730	0.01918322
244	0.02984034	-0.06013742	0.01878179
245	0.02511095	-0.06023239	0.01808324

246	0.02021849	-0.05989344	0.01710913
247	0.01544639	-0.05921503	0.01579203
248	0.01047831	-0.05829041	0.01404729
249	0.00579382	-0.05694782	0.01182498
250	0.00132668	-0.05559396	0.00950192
251	-0.00322095	-0.05404962	0.00702342
252	-0.00736431	-0.05217016	0.00419274
253	-0.01161348	-0.05007272	0.00095462
254	-0.01578702	-0.04769761	-0.00254514
255	-0.02007519	-0.04537773	-0.00604999
256	-0.02421384	-0.04295314	-0.00937086

Procrustes sums of squares: 0.32630358941122606

Tangent sums of squares: 0.3223423480086

Data matrices in this dataset:

- BE_Canals, raw data
- BE_Canals, centroid size
- BE_Canals, Procrustes coordinates

New Procrustes fit:

Dataset 'BE_Canals'

256 landmarks in 3 dimensions.

The dataset contains 32 observations, of which 32 are included for analyses.

Average shape:

Lmk.	Axis 1 (x)	Axis 2 (y)	Axis 3 (z)
1	-0.04141995	0.02935969	0.00749175
2	0.04263838	0.05411971	-0.00301215
3	0.04174563	0.05473665	-0.00035387
4	0.03980603	0.05547906	0.00236344
5	0.03718705	0.05620669	0.00465257
6	0.03419929	0.05677374	0.00674637
7	0.03089988	0.05718168	0.00870453
8	0.02725099	0.05729037	0.01053089
9	0.02331524	0.05726811	0.01210709
10	0.01921243	0.05708083	0.01363220
11	0.01481087	0.05682857	0.01506394
12	0.01027788	0.05635569	0.01607772
13	0.00588568	0.05564811	0.01677962
14	0.00146915	0.05478802	0.01711935
15	-0.00283056	0.05367537	0.01708481
16	-0.00711146	0.05242784	0.01656877
17	-0.01127782	0.05093795	0.01580340
18	-0.01517331	0.04932435	0.01452689
19	-0.01871124	0.04755739	0.01275216
20	-0.02229628	0.04563512	0.01071807
21	-0.02662878	0.04305404	0.00723379
22	-0.03003878	0.04037557	0.00352860
23	-0.03271640	0.03763834	-0.00064113
24	-0.03409231	0.03506855	-0.00569782
25	-0.03396297	0.03297598	-0.01110329

26	-0.03302098	0.03076462	-0.01726775
27	-0.03104222	0.02831511	-0.02414112
28	-0.02777722	0.02620050	-0.03155475
29	-0.02322985	0.02480699	-0.03903098
30	-0.01841769	0.02396839	-0.04343437
31	-0.01235468	0.02452350	-0.04619867
32	-0.00652648	0.02600868	-0.04684400
33	-0.00039998	0.02790652	-0.04656777
34	0.00611680	0.03008515	-0.04547111
35	0.01302220	0.03245916	-0.04356053
36	0.02012250	0.03489250	-0.04097670
37	0.02726003	0.03722580	-0.03759602
38	0.03248973	0.04030713	-0.03286818
39	0.03353623	0.04211001	-0.03006134
40	0.03427540	0.04399113	-0.02664891
41	0.03504705	0.04591501	-0.02302587
42	0.03606300	0.04774068	-0.01958165
43	0.03742973	0.04933233	-0.01626169
44	0.03923010	0.05056043	-0.01294660
45	0.04111709	0.05154230	-0.00964424
46	0.04243682	0.05266090	-0.00611404
47	0.06219070	0.05896799	0.00028097
48	0.06073710	0.05921882	0.00191550
49	0.05899481	0.05948287	0.00359692
50	0.05675810	0.05982849	0.00549356
51	0.05417012	0.06016242	0.00740483
52	0.05116089	0.06038283	0.00934500
53	0.04789119	0.06063020	0.01135801

54	0.04440868	0.06086716	0.01329498
55	0.04077485	0.06096902	0.01524031
56	0.03666842	0.06132823	0.01736874
57	0.03259543	0.06144913	0.01913898
58	0.02855463	0.06142749	0.02078117
59	0.02464428	0.06124747	0.02220724
60	0.02070302	0.06097765	0.02347843
61	0.01679002	0.06046373	0.02429342
62	0.01294095	0.06017878	0.02536622
63	0.00921906	0.05963516	0.02602802
64	0.00540200	0.05892663	0.02648693
65	0.00162103	0.05832040	0.02675589
66	-0.00192145	0.05762950	0.02670003
67	-0.00543484	0.05689367	0.02647047
68	-0.00885962	0.05602967	0.02599774
69	-0.01220296	0.05511595	0.02529541
70	-0.01541971	0.05414327	0.02435090
71	-0.01843032	0.05291685	0.02314905
72	-0.02149032	0.05147967	0.02164975
73	-0.02452244	0.05015891	0.02029506
74	-0.02717483	0.04866344	0.01844178
75	-0.02938572	0.04726557	0.01671187
76	-0.03173670	0.04590149	0.01501244
77	-0.03397371	0.04432921	0.01299760
78	-0.03607791	0.04259073	0.01063406
79	-0.03822764	0.04072211	0.00798021
80	-0.04042997	0.03886523	0.00514962
81	-0.04287176	0.03712860	0.00213291

82	-0.04550669	0.03540696	-0.00114185
83	-0.04795965	0.03356039	-0.00473204
84	-0.04465184	-0.03316798	-0.01390263
85	-0.04429866	-0.03409316	-0.01170400
86	-0.04415302	-0.03461194	-0.00894954
87	-0.04432117	-0.03449488	-0.00623640
88	-0.04464684	-0.03400414	-0.00352368
89	-0.04505660	-0.03317170	-0.00110964
90	-0.04558605	-0.03210176	0.00126006
91	-0.04621195	-0.03080464	0.00339530
92	-0.04683791	-0.02939435	0.00555162
93	-0.04748008	-0.02765229	0.00762184
94	-0.04796475	-0.02565539	0.00967304
95	-0.04850300	-0.02344599	0.01141944
96	-0.04908280	-0.02096578	0.01289290
97	-0.04954086	-0.01834074	0.01417511
98	-0.04994605	-0.01551603	0.01522256
99	-0.05033822	-0.01251259	0.01602543
100	-0.05061099	-0.00928531	0.01646293
101	-0.05080568	-0.00597082	0.01647199
102	-0.05111353	-0.00269187	0.01603443
103	-0.05101302	0.00109964	0.01496078
104	-0.05067126	0.00464523	0.01318066
105	-0.05024427	0.00781754	0.01116630
106	-0.04982913	0.01060805	0.00882345
107	-0.04918682	0.01318829	0.00615765
108	-0.04847038	0.01513199	0.00349254
109	-0.04760015	0.01705868	0.00029368

110	-0.04683251	0.01827800	-0.00325955
111	-0.04608691	0.01886940	-0.00706846
112	-0.04357131	0.01816739	-0.01539512
113	-0.04186544	0.01603132	-0.02268167
114	-0.04032061	0.01367715	-0.02861545
115	-0.03949728	0.01030387	-0.03325136
116	-0.03899987	0.00714856	-0.03625343
117	-0.03832724	0.00360684	-0.03864428
118	-0.03830087	-0.00069653	-0.03981432
119	-0.03931824	-0.00599553	-0.03924776
120	-0.04121863	-0.01078293	-0.03693943
121	-0.04334156	-0.01321702	-0.03503727
122	-0.04536857	-0.01550923	-0.03244715
123	-0.04697523	-0.01776204	-0.02962731
124	-0.04796169	-0.02025596	-0.02685709
125	-0.04826189	-0.02297413	-0.02415997
126	-0.04781179	-0.02592044	-0.02169801
127	-0.04691355	-0.02880146	-0.01919779
128	-0.04591099	-0.03101338	-0.01697088
129	-0.04650941	-0.05072280	-0.01801442
130	-0.04597095	-0.05062362	-0.01552130
131	-0.04554710	-0.05040437	-0.01314335
132	-0.04509578	-0.04982539	-0.01023914
133	-0.04484715	-0.04925445	-0.00731912
134	-0.04485058	-0.04876331	-0.00458298
135	-0.04484760	-0.04806500	-0.00200382
136	-0.04493916	-0.04706657	0.00052912
137	-0.04518442	-0.04598884	0.00300851

138	-0.04568263	-0.04465803	0.00533570
139	-0.04663593	-0.04281305	0.00827796
140	-0.04740078	-0.04080127	0.01113918
141	-0.04806092	-0.03857832	0.01365858
142	-0.04871322	-0.03649461	0.01593642
143	-0.04925982	-0.03408514	0.01783141
144	-0.04981617	-0.03144501	0.01965235
145	-0.05041730	-0.02891573	0.02158394
146	-0.05098142	-0.02643322	0.02314952
147	-0.05149496	-0.02356574	0.02449155
148	-0.05194269	-0.02053281	0.02538550
149	-0.05249890	-0.01742140	0.02623767
150	-0.05286831	-0.01425125	0.02664764
151	-0.05317580	-0.01084149	0.02692065
152	-0.05348134	-0.00749730	0.02692366
153	-0.05372683	-0.00415490	0.02656524
154	-0.05390877	-0.00085218	0.02596911
155	-0.05398993	0.00249039	0.02496143
156	-0.05394814	0.00562961	0.02405383
157	-0.05382760	0.00942568	0.02204858
158	-0.05363764	0.01310951	0.01938789
159	-0.05334229	0.01652624	0.01656810
160	-0.05265093	0.01971405	0.01335286
161	-0.05170712	0.02268356	0.01008197
162	-0.05082148	0.02544972	0.00683779
163	-0.04987199	0.02814909	0.00323233
164	-0.04894866	0.03092117	-0.00067780
165	-0.04795965	0.03356039	-0.00473204

166	0.07056616	-0.01068682	-0.00892163
167	0.07175760	-0.01321056	-0.00724446
168	0.07214541	-0.01605492	-0.00528434
169	0.07200054	-0.01885704	-0.00341407
170	0.07121121	-0.02165384	-0.00146916
171	0.07002864	-0.02455456	0.00041394
172	0.06842070	-0.02749732	0.00220899
173	0.06622290	-0.03051919	0.00399487
174	0.06365187	-0.03361853	0.00576386
175	0.06062475	-0.03667317	0.00740956
176	0.05810666	-0.03888812	0.00864993
177	0.05539227	-0.04102518	0.00972933
178	0.05251385	-0.04293453	0.01062575
179	0.04947033	-0.04447234	0.01132218
180	0.04642511	-0.04589815	0.01184298
181	0.04320319	-0.04726567	0.01220685
182	0.03983977	-0.04836468	0.01239410
183	0.03637054	-0.04916877	0.01236580
184	0.03235232	-0.04979441	0.01213765
185	0.02863375	-0.04997766	0.01174764
186	0.02493824	-0.04999175	0.01110022
187	0.02125453	-0.04978633	0.01015159
188	0.01735602	-0.04949413	0.00890364
189	0.01385677	-0.04875699	0.00765412
190	0.01043524	-0.04790143	0.00619627
191	0.00706795	-0.04680922	0.00456216
192	0.00351097	-0.04561828	0.00249403
193	0.00033874	-0.04461140	0.00024054

194	-0.00449681	-0.04303065	-0.00337946
195	-0.00845861	-0.04072390	-0.00721210
196	-0.01160677	-0.03805440	-0.01134756
197	-0.01395775	-0.03518073	-0.01565911
198	-0.01535183	-0.03186341	-0.02027666
199	-0.01577705	-0.02802772	-0.02503512
200	-0.01564063	-0.02404369	-0.02992133
201	-0.01477189	-0.01960726	-0.03471592
202	-0.01291118	-0.01444753	-0.03978204
203	-0.00894108	-0.00823206	-0.04355956
204	-0.00344960	-0.00269439	-0.04530121
205	0.00212031	0.00183929	-0.04561739
206	0.00823427	0.00553474	-0.04528930
207	0.01477786	0.00833916	-0.04455672
208	0.02187281	0.01030498	-0.04264590
209	0.02929199	0.01122686	-0.03923269
210	0.03659574	0.01098425	-0.03439908
211	0.04329808	0.00953429	-0.02815946
212	0.04660998	0.00773075	-0.02465190
213	0.04966285	0.00543132	-0.02139387
214	0.05275201	0.00300575	-0.01845855
215	0.05610996	0.00065121	-0.01602961
216	0.05949316	-0.00146475	-0.01420430
217	0.06287148	-0.00367320	-0.01264192
218	0.06607082	-0.00574278	-0.01151598
219	0.06852679	-0.00805867	-0.01032698
220	0.09154619	-0.00256635	-0.00731781
221	0.09106538	-0.00656481	-0.00538836

222	0.09019448	-0.01044118	-0.00359890
223	0.08911599	-0.01413545	-0.00188788
224	0.08778662	-0.01761937	-0.00034000
225	0.08633178	-0.02100194	0.00116468
226	0.08474899	-0.02418458	0.00248270
227	0.08304786	-0.02712416	0.00377989
228	0.08126539	-0.02993694	0.00513549
229	0.07954950	-0.03233102	0.00646558
230	0.07747552	-0.03498296	0.00806846
231	0.07522930	-0.03746770	0.00962097
232	0.07284833	-0.03992759	0.01118821
233	0.07027600	-0.04240745	0.01248552
234	0.06759303	-0.04471699	0.01375292
235	0.06475657	-0.04697978	0.01495018
236	0.06182680	-0.04909507	0.01608286
237	0.05871545	-0.05111639	0.01702674
238	0.05565401	-0.05307950	0.01793772
239	0.05156252	-0.05495752	0.01851156
240	0.04744259	-0.05669657	0.01915729
241	0.04336201	-0.05795303	0.01937187
242	0.03884108	-0.05889288	0.01931769
243	0.03440076	-0.05963730	0.01918322
244	0.02984034	-0.06013742	0.01878179
245	0.02511095	-0.06023239	0.01808324
246	0.02021849	-0.05989344	0.01710913
247	0.01544639	-0.05921503	0.01579203
248	0.01047831	-0.05829041	0.01404729
249	0.00579382	-0.05694782	0.01182498

250	0.00132668	-0.05559396	0.00950192
251	-0.00322095	-0.05404962	0.00702342
252	-0.00736431	-0.05217016	0.00419274
253	-0.01161348	-0.05007272	0.00095462
254	-0.01578702	-0.04769761	-0.00254514
255	-0.02007519	-0.04537773	-0.00604999
256	-0.02421384	-0.04295314	-0.00937086

Procrustes sums of squares: 0.32630358941122606

Tangent sums of squares: 0.3223423480086

Data matrices in this dataset:

- BE_Canals, raw data
- BE_Canals, centroid size
- BE_Canals, Procrustes coordinates

Principal Component Analysis: PCA: CovMatrix, BE_Canals, Procrustes coordinates

	Eigenvalues	% Variance	Cumulative %
1.	0.00225468	21.684	21.684
2.	0.00119446	11.487	33.171
3.	0.00098577	9.480	42.651
4.	0.00088305	8.492	51.143
5.	0.00084313	8.108	59.252
6.	0.00071581	6.884	66.136

7.	0.00057872	5.566	71.701
8.	0.00039165	3.767	75.468
9.	0.00034167	3.286	78.754
10.	0.00030818	2.964	81.718
11.	0.00026469	2.546	84.263
12.	0.00023382	2.249	86.512
13.	0.00018752	1.803	88.315
14.	0.00017362	1.670	89.985
15.	0.00015452	1.486	91.471
16.	0.00012222	1.175	92.646
17.	0.00010993	1.057	93.704
18.	0.00010549	1.015	94.718
19.	0.00007525	0.724	95.442
20.	0.00007101	0.683	96.125
21.	0.00006229	0.599	96.724
22.	0.00005770	0.555	97.279
23.	0.00005034	0.484	97.763
24.	0.00004825	0.464	98.227
25.	0.00003853	0.371	98.597
26.	0.00003361	0.323	98.921
27.	0.00003005	0.289	99.210
28.	0.00002664	0.256	99.466
29.	0.00002236	0.215	99.681
30.	0.00001769	0.170	99.851
31.	0.00001550	0.149	100.000

Total variance: 0.01039814

Variance of the eigenvalues: 0.0000000135653

Eigenvalue variance scaled by total variance: 0.00013

Eigenvalue variance scaled by total variance and number of variables: 0.09560

Note: throughout all calculations of eigenvalue variances, the dimensionality used was 761.

Please double-check because this dimensionality may not be appropriate for all situations.

Warning: There appear to be fewer PCs than dimensions in shape space. This can have the following reasons, among others:

-- the degrees of freedom are less than the shape dimensionality (sample size, etc.).

-- there is complete integration in the data.

Interpret the results cautiously and check for reasons why this is the case.

Principal Component Coefficients

PC1 PC2 PC3 PC4 PC5 PC6 PC7 PC8 PC9 PC10 PC11 PC12
PC13 PC14 PC15 PC16 PC17 PC18 PC19 PC20 PC21 PC22 PC23 PC24
PC25 PC26 PC27 PC28 PC29 PC30 PC31

x1	0.026403	-0.019002	-0.034580	0.007072	-0.012084	-0.025777
	-0.041616	-0.056501	-0.029101	0.005229	-0.020307	-0.020569
	0.101467	0.054151	0.019755	0.037411	0.057597	-0.032692
	-0.004219	-0.049813	-0.055779	0.012544	-0.002786	0.017641
	0.067604	0.020143	-0.033551	-0.053433	-0.053287	0.024824
	-0.048231					
y1	0.003595	0.037485	0.004710	0.001248	-0.046245	-0.011507
	0.026654	-0.012835	-0.008278	-0.012496	-0.018361	0.002008
	0.036340	-0.002667	-0.049800	-0.036287	0.045968	0.013530
	0.010137	0.070384	-0.010519	-0.080986	-0.027379	-0.003123
	0.025835	0.031435	-0.058756	0.121000	0.031156	-0.021472
	-0.068502					
z1	0.001032	-0.051179	0.089875	-0.043940	0.028798	0.009900
	-0.004347	0.057697	-0.051193	-0.068659	0.004789	-0.055980
	0.002401	-0.015728	0.045940	-0.018699	-0.018194	0.113924
	-0.002685	0.014769	-0.024052	0.059698	-0.015036	0.009528
	-0.030852	-0.039994	-0.077589	-0.018017	0.074114	-0.005366
	-0.013976					
x2	-0.035790	-0.011621	-0.065957	-0.024634	0.036469	0.028233
	0.046887	-0.009648	0.039684	-0.010929	0.017092	0.047037

	-0.055508	-0.055753	0.052038	-0.035105	-0.058393	0.013948
	-0.014741	0.009123	0.032743	0.005727	-0.001958	0.042324
	0.005142	0.012010	0.042338	-0.079139	-0.022449	-0.022650
	-0.056866					
y2	-0.053831	-0.001939	-0.010159	0.013016	0.041883	-0.017814
	-0.071316	-0.029305	0.016543	-0.014017	-0.014124	-0.017118
	-0.018080	0.016296	0.033667	0.021585	-0.029118	0.008418
	0.053174	-0.022912	0.044662	-0.015766	0.010917	-0.012721
	0.063670	0.020187	0.064919	0.075831	-0.055612	-0.055755
	-0.045748					
z2	0.019937	0.046467	-0.042921	-0.016346	0.013774	-0.029000
	0.017621	-0.048115	-0.005824	0.058693	-0.040330	-0.006607
	0.038026	-0.002916	0.036254	-0.022883	-0.067312	0.076297
	0.021876	0.003204	-0.043336	0.102250	0.048136	-0.047582
	0.047348	-0.070837	0.032751	-0.048108	-0.000373	-0.014159
	-0.062944					
x3	-0.039808	-0.020032	-0.061959	-0.015976	0.038310	0.034413
	0.045151	-0.007425	0.047332	-0.014236	0.026244	0.046431
	-0.052915	-0.046094	0.041673	-0.037008	-0.046862	0.017526
	-0.014291	0.026413	0.040295	-0.007203	-0.007267	0.031804
	-0.010802	-0.005536	0.033372	-0.048873	-0.009924	-0.032433
	-0.039443					
y3	-0.063296	-0.001521	-0.009865	0.014079	0.046431	-0.014864
	-0.074481	-0.026871	0.025080	-0.008197	-0.008265	-0.036423
	-0.020258	0.001744	0.030768	0.014720	-0.022700	0.015935
	0.039688	-0.006464	0.045071	0.000906	0.035299	-0.029890
	0.023908	0.014974	0.056966	0.063851	-0.039717	-0.051337
	-0.017084					
z3	0.015810	0.039387	-0.042255	-0.016382	0.016075	-0.025956
	0.020244	-0.046881	0.005204	0.054258	-0.031325	0.006861
	0.025265	0.001880	0.024124	-0.022148	-0.052828	0.065425
	0.017281	0.009819	-0.037336	0.067937	0.026833	-0.033705
	0.033985	-0.074763	0.008807	-0.036734	-0.010351	-0.007391
	-0.042016					
x4	-0.040223	-0.026623	-0.058839	-0.011895	0.037666	0.038593
	0.043098	-0.001486	0.048623	-0.014354	0.034490	0.045994
	-0.050727	-0.043964	0.028601	-0.036703	-0.034051	0.014581
	-0.013557	0.029153	0.047277	-0.001240	-0.001379	0.020636
	-0.019144	-0.010536	0.013778	-0.013495	-0.009976	-0.032858
	-0.010618					

y4	-0.071046	0.000093	-0.011119	0.014521	0.047254	-0.014846
	-0.073426	-0.027090	0.037107	-0.002946	-0.005898	-0.045862
	-0.021291	-0.006109	0.030972	0.008965	-0.013362	0.016725
	0.028078	0.004501	0.051462	0.011521	0.049113	-0.039673
	-0.011383	0.009787	0.038701	0.050936	-0.025037	-0.042451
	-0.002548					
z4	0.009887	0.030269	-0.040996	-0.015720	0.019735	-0.019806
	0.022133	-0.043450	0.014108	0.049459	-0.019731	0.015729
	0.012065	0.001884	0.010038	-0.020143	-0.040941	0.050489
	0.012928	0.018540	-0.025467	0.050391	0.016124	-0.025730
	0.034430	-0.083028	-0.016528	-0.009668	-0.009087	-0.003087
	-0.013875					
x5	-0.038502	-0.031038	-0.058157	-0.011632	0.032440	0.040635
	0.039618	0.001635	0.043478	-0.007402	0.043611	0.045385
	-0.048409	-0.043038	0.018957	-0.042531	-0.025795	0.010508
	-0.018413	0.028161	0.036496	-0.001577	0.005589	0.018759
	-0.023089	-0.014211	0.012557	-0.002825	-0.007097	-0.022626
	-0.008980					
y5	-0.074255	0.001115	-0.011753	0.013880	0.043434	-0.017223
	-0.070271	-0.030015	0.049978	0.002789	-0.006089	-0.047727
	-0.018472	0.001802	0.041041	0.015958	0.001636	0.007385
	0.015023	-0.001045	0.059649	0.014654	0.057732	-0.043054
	-0.034305	0.008038	0.015000	0.056979	-0.017265	-0.041675
	0.009824					
z5	0.002457	0.021577	-0.041190	-0.017510	0.020516	-0.011132
	0.023617	-0.040143	0.015098	0.051127	-0.009175	0.026527
	0.000562	-0.000292	-0.009682	-0.027920	-0.031592	0.041498
	0.008076	0.036833	-0.036526	0.040026	0.018001	-0.012400
	0.037390	-0.084800	-0.017728	-0.014996	-0.006422	0.012862
	-0.005718					
x6	-0.036638	-0.034667	-0.056328	-0.010820	0.029919	0.042229
	0.032966	0.003288	0.035989	-0.004335	0.047163	0.039758
	-0.046210	-0.041169	0.007104	-0.042165	-0.016189	0.011336
	-0.022969	0.033261	0.028276	-0.001625	0.011223	0.022573
	-0.016867	-0.013629	0.011545	0.008112	-0.002161	-0.015799
	0.002274					
y6	-0.075940	0.002089	-0.011751	0.012515	0.038050	-0.017788
	-0.066612	-0.034362	0.057084	0.009256	-0.006223	-0.045327
	-0.017158	0.006167	0.043711	0.017239	0.012527	0.000104
	0.002657	-0.000545	0.063161	0.019450	0.065412	-0.044022
	-0.040239	0.001108	0.000697	0.057406	-0.003946	-0.048031
	0.016300					

z6	-0.004552	0.012585	-0.039237	-0.017785	0.024584	-0.003594
	0.022525	-0.038406	0.016576	0.049677	-0.002354	0.026852
	-0.008104	0.006141	-0.017489	-0.021628	-0.023819	0.033882
	-0.002276	0.051491	-0.037818	0.026525	0.021098	0.009896
	0.049333	-0.077076	-0.018979	-0.004831	0.001457	0.023173
	0.005337					
x7	-0.033470	-0.036579	-0.055016	-0.009169	0.028134	0.042098
	0.028185	0.003101	0.025790	-0.001649	0.049053	0.033369
	-0.037854	-0.036113	-0.000672	-0.044448	-0.019198	0.016398
	-0.023607	0.028109	0.025515	0.002491	0.015462	0.020493
	-0.018155	-0.014141	0.010357	0.012834	0.002250	-0.001653
	-0.002448					
y7	-0.077725	0.002641	-0.011371	0.009089	0.034233	-0.019271
	-0.062263	-0.040018	0.061548	0.013077	-0.012268	-0.034645
	-0.016008	0.008971	0.035623	0.014041	0.023823	-0.005231
	-0.001386	0.010202	0.064944	0.025873	0.062335	-0.047283
	-0.034072	-0.014848	-0.009132	0.058770	0.006335	-0.055225
	0.022399					
z7	-0.010929	0.005501	-0.037905	-0.016803	0.031139	0.000309
	0.024844	-0.036488	0.017242	0.046528	0.004982	0.023293
	-0.007882	0.017731	-0.018154	-0.018029	-0.034124	0.032159
	-0.006962	0.044419	-0.027615	0.024099	0.022317	0.018786
	0.036696	-0.067657	-0.016164	-0.003505	0.011311	0.048275
	-0.007750					
x8	-0.029065	-0.036292	-0.052937	-0.007472	0.023649	0.041268
	0.020081	0.002466	0.017428	-0.001656	0.046640	0.032500
	-0.034398	-0.028627	-0.012249	-0.044285	-0.016653	0.017212
	-0.009446	0.022267	0.017082	0.004989	0.016224	0.008152
	-0.020898	0.000537	0.012687	0.014950	0.002215	0.001602
	-0.001019					
y8	-0.079170	0.001657	-0.011704	0.005916	0.029802	-0.022816
	-0.053838	-0.039884	0.063572	0.017334	-0.018303	-0.021256
	-0.010979	0.011961	0.029561	0.010929	0.024262	-0.013431
	-0.001335	0.007185	0.066264	0.038082	0.052918	-0.048214
	-0.031679	-0.034804	-0.009366	0.051278	0.010010	-0.057053
	0.004802					
z8	-0.014803	0.003118	-0.036326	-0.014759	0.029393	0.004882
	0.022819	-0.040044	0.024533	0.038830	0.001951	0.034474
	-0.015600	0.032445	-0.025460	-0.009722	-0.029003	0.019670
	0.011589	0.036000	-0.024051	0.023108	0.015818	0.008917
	0.030223	-0.039456	-0.014345	-0.007932	0.013164	0.041299
	-0.002269					

x9	-0.026251	-0.035916	-0.052308	-0.005478	0.019738	0.038738
	0.012219	0.004633	0.008345	-0.000304	0.045727	0.028439
	-0.029987	-0.024232	-0.021348	-0.045427	-0.017191	0.017273
	-0.003706	0.017319	0.014875	0.008375	0.015560	-0.000850
	-0.020128	0.007534	0.011848	0.013693	0.005607	-0.002498
	-0.000711					
y9	-0.080909	-0.000462	-0.011915	-0.000029	0.024868	-0.026142
	-0.044674	-0.038545	0.060515	0.022180	-0.021403	-0.002480
	-0.006268	0.015763	0.020954	0.009000	0.028150	-0.021926
	-0.000323	-0.002998	0.066531	0.042102	0.040084	-0.054098
	-0.025263	-0.054197	-0.006941	0.047400	0.015392	-0.062205
	-0.008459					
z9	-0.022860	0.001537	-0.036920	-0.013986	0.027855	0.006572
	0.023574	-0.036094	0.034385	0.030834	0.003717	0.046529
	-0.024452	0.038048	-0.026727	-0.004569	-0.024803	-0.010386
	0.009983	0.030273	-0.018655	0.025390	0.005695	0.001076
	0.026483	-0.025284	-0.009010	-0.009257	0.021948	0.015107
	0.023634					
x10	-0.022854	-0.035136	-0.050661	-0.004219	0.015838	0.038240
	0.005536	0.008122	-0.001427	0.001723	0.048950	0.022218
	-0.023738	-0.021859	-0.025903	-0.045229	-0.015376	0.020307
	-0.003426	0.017443	0.003058	0.011321	0.012445	-0.003239
	-0.016466	0.021713	0.008774	0.009167	0.006596	0.010373
	-0.000347					
y10	-0.084454	-0.003697	-0.010569	-0.005478	0.022287	-0.028850
	-0.036992	-0.034244	0.051370	0.026583	-0.020346	0.014477
	-0.002106	0.018130	0.011792	0.004652	0.036006	-0.030624
	0.002363	-0.007290	0.057470	0.042161	0.029109	-0.058872
	-0.020962	-0.064062	0.006848	0.050747	0.035480	-0.062690
	-0.023459					
z10	-0.030884	-0.002677	-0.033257	-0.015930	0.031318	0.011971
	0.023642	-0.016874	0.040171	0.023956	0.017083	0.041278
	-0.030990	0.034122	-0.025410	0.002523	-0.021329	-0.020880
	0.000579	0.031897	-0.033434	0.025325	0.000803	0.008360
	0.006634	0.021364	0.019760	-0.028243	0.023870	0.053970
	0.017414					
x11	-0.017554	-0.035135	-0.049806	-0.002494	0.011446	0.036226
	-0.002153	0.007389	-0.012890	0.005109	0.048218	0.013068
	-0.013162	-0.020355	-0.030214	-0.044196	-0.013272	0.028487
	-0.004902	0.018938	0.004370	0.013799	0.008623	-0.007053
	-0.012257	0.026825	0.002159	0.010006	0.011830	0.009338
	-0.002954					

y11	-0.090731	-0.006610	-0.011165	-0.007002	0.021013	-0.023020
	-0.032085	-0.032110	0.041195	0.016673	-0.016937	0.032558
	0.011882	0.016855	-0.002952	0.002093	0.039781	-0.042870
	0.011985	-0.006572	0.038353	0.050329	0.001855	-0.074978
	-0.023383	-0.065292	0.034579	0.044940	0.052976	-0.055511
	-0.006394					
z11	-0.035363	-0.007322	-0.030548	-0.016030	0.034214	0.021276
	0.021595	-0.018420	0.041647	0.018109	0.021595	0.037807
	-0.031295	0.025551	-0.024990	0.008590	-0.003265	-0.023105
	-0.006802	0.049247	-0.016697	0.018497	0.002390	0.013249
	0.008784	0.035539	0.015772	-0.035541	0.030693	0.032770
	0.012629					
x12	-0.013547	-0.034487	-0.048835	0.000384	0.008806	0.034557
	-0.014234	0.001812	-0.022701	0.006777	0.044217	0.001296
	-0.007733	-0.017903	-0.030379	-0.041365	-0.009692	0.031205
	-0.000649	0.018350	0.007824	0.018486	-0.000480	-0.014360
	0.006902	0.027989	-0.004557	0.016839	0.023826	-0.003044
	0.003615					
y12	-0.087233	-0.004411	-0.009513	-0.003205	0.011212	-0.022549
	-0.017340	-0.028595	0.038933	0.016323	-0.015749	0.056308
	0.020862	0.012031	-0.023555	0.009398	0.052039	-0.041971
	0.006973	0.000364	0.001386	0.046778	-0.006526	-0.055312
	-0.042533	-0.068480	0.022064	0.004987	0.032649	-0.033466
	0.015990					
z12	-0.040419	-0.012002	-0.030495	-0.013168	0.039456	0.027800
	0.017501	-0.035483	0.042402	0.017835	0.018081	0.037872
	-0.042666	0.032395	-0.016038	0.018503	0.017135	-0.032340
	-0.010505	0.050378	0.009434	0.015427	-0.006312	0.008578
	0.041058	0.045453	0.012070	-0.036939	0.031326	0.002649
	0.017158					
x13	-0.007897	-0.033587	-0.048117	0.003298	0.003982	0.028885
	-0.023773	-0.001362	-0.027582	0.007736	0.046967	-0.014405
	0.002071	-0.008901	-0.014638	-0.032457	-0.016362	0.028643
	0.002063	0.000433	0.011620	0.019543	-0.008569	-0.021666
	0.007262	0.033853	-0.009837	0.036122	0.035786	-0.001694
	-0.001413					
y13	-0.082431	-0.003535	-0.003566	-0.002985	0.006476	-0.027711
	-0.006317	-0.022645	0.035950	0.020152	-0.019455	0.068284
	0.018743	0.012724	-0.035703	0.010646	0.060965	-0.038513
	0.002305	0.005222	-0.020011	0.034533	-0.012689	-0.040374
	-0.035944	-0.077510	0.022301	-0.010505	0.026194	-0.029781
	0.005804					

z13	-0.043852	-0.014342	-0.032028	-0.011378	0.044204	0.035684
	0.014441	-0.036162	0.050325	0.013932	0.033406	0.020834
	-0.040116	0.045398	0.010267	0.040286	0.017728	-0.046529
	-0.004975	0.025507	0.018045	-0.000787	-0.003808	-0.007942
	0.030912	0.042737	0.007357	0.001380	0.043380	-0.008106
	-0.017728					
x14	-0.004599	-0.030978	-0.045246	0.005369	-0.000789	0.023783
	-0.030446	-0.003578	-0.038716	0.007473	0.044641	-0.021776
	0.012734	-0.003954	-0.012100	-0.032973	-0.019288	0.029250
	0.004747	-0.006050	0.009967	0.026160	-0.009735	-0.028097
	0.010561	0.039959	-0.008797	0.040140	0.039827	0.002481
	-0.002591					
y14	-0.076621	-0.003358	-0.002857	-0.001796	0.001177	-0.025723
	-0.000119	-0.014721	0.036352	0.021919	-0.019096	0.070677
	0.010840	0.010592	-0.052340	0.018640	0.069828	-0.031854
	0.003952	0.015356	-0.024798	0.024867	-0.028642	-0.029550
	-0.038676	-0.075140	0.011446	-0.026023	0.018780	-0.018416
	0.014670					
z14	-0.050140	-0.015236	-0.020431	-0.006548	0.042587	0.038955
	0.012271	-0.042313	0.043724	0.004578	0.042837	0.012657
	-0.042035	0.046909	0.015948	0.042190	0.020273	-0.059320
	-0.009960	0.039418	0.035006	0.006982	0.027364	0.006983
	0.022895	0.053277	0.010217	-0.006118	0.047145	-0.043886
	-0.028619					
x15	-0.000287	-0.028723	-0.042956	0.007877	-0.006866	0.016691
	-0.037681	-0.006835	-0.048201	0.007930	0.041600	-0.029517
	0.021732	-0.000600	-0.005075	-0.029440	-0.017984	0.026794
	0.008897	-0.010930	0.008142	0.024130	-0.009732	-0.034040
	0.009650	0.039931	-0.013597	0.045733	0.048820	0.003886
	-0.007186					
y15	-0.069679	-0.003570	-0.002633	-0.000347	-0.002158	-0.026295
	0.007407	-0.007148	0.038215	0.025739	-0.017401	0.067193
	0.005405	0.010211	-0.059975	0.023575	0.077506	-0.023827
	0.011201	0.016375	-0.031865	0.019982	-0.038674	-0.018304
	-0.028553	-0.070640	0.005355	-0.030455	0.011368	-0.004356
	0.023341					
z15	-0.055584	-0.019813	-0.011100	-0.007369	0.043217	0.038541
	0.013422	-0.038523	0.035793	0.001063	0.039631	0.006928
	-0.040072	0.025525	0.003515	0.030395	0.031774	-0.052572
	-0.013183	0.072334	0.025923	0.003323	0.045152	0.009746
	0.012930	0.041337	0.016180	-0.012021	0.052023	-0.040858
	-0.024619					

x16	0.003893	-0.026446	-0.042087	0.010136	-0.012073	0.010974
	-0.043982	-0.008050	-0.056013	0.008222	0.037413	-0.034225
	0.027802	-0.002253	-0.005410	-0.023452	-0.010649	0.026502
	0.012363	-0.009254	0.002613	0.021227	-0.007388	-0.038849
	0.008061	0.032571	-0.023750	0.044180	0.052174	0.009210
	-0.007887					
y16	-0.064523	-0.001408	0.001348	0.000470	-0.005025	-0.028357
	0.014886	-0.002911	0.030561	0.031344	-0.019588	0.064490
	0.004728	0.014675	-0.067646	0.015223	0.075357	-0.010161
	0.023113	0.017106	-0.034553	0.021092	-0.046040	-0.006420
	-0.010800	-0.059673	0.009761	-0.035489	0.000482	0.001156
	0.021637					
z16	-0.053746	-0.026110	-0.005292	-0.010982	0.041855	0.042344
	0.006296	-0.036339	0.037470	-0.007849	0.042035	-0.005062
	-0.035419	0.014827	0.007639	0.020461	0.030674	-0.057471
	-0.022209	0.069866	0.032431	-0.004524	0.034439	0.008470
	0.001891	0.036857	0.035475	-0.026278	0.062939	-0.040547
	-0.051231					
x17	0.008452	-0.023677	-0.040884	0.011770	-0.016971	0.003321
	-0.049963	-0.007368	-0.062949	0.010044	0.033324	-0.037421
	0.035388	-0.004504	-0.005690	-0.019158	-0.003350	0.026163
	0.014302	-0.003172	-0.001590	0.020385	-0.002604	-0.037960
	0.011735	0.019747	-0.023497	0.033801	0.058034	0.020222
	-0.016458					
y17	-0.058777	-0.000697	0.003028	0.001568	-0.010327	-0.027127
	0.022172	0.001457	0.025238	0.037120	-0.019023	0.058684
	-0.002973	0.018385	-0.073545	0.014081	0.077214	-0.006313
	0.042890	0.009087	-0.036515	0.019307	-0.056871	-0.010761
	0.003057	-0.035956	0.005151	-0.026825	-0.002580	-0.002331
	0.025351					
z17	-0.052581	-0.031180	0.001208	-0.013427	0.043119	0.048430
	0.001727	-0.036030	0.033240	-0.022696	0.041950	-0.003490
	-0.021974	0.011271	0.012396	0.008295	0.019076	-0.054162
	-0.036845	0.052718	0.019055	-0.015987	0.027987	0.002678
	-0.016543	0.036599	0.023940	-0.032020	0.050419	-0.040889
	-0.062240					
x18	0.013779	-0.019166	-0.039508	0.015131	-0.022213	-0.003758
	-0.057173	-0.010201	-0.066495	0.007367	0.031100	-0.042590
	0.044947	-0.006295	0.007418	-0.012711	0.003335	0.021104
	0.010886	-0.008024	0.002427	0.013141	-0.003933	-0.043814
	0.012597	0.005125	-0.035374	0.030384	0.072367	0.013597
	-0.035062					

y18	-0.052933	-0.001367	0.005461	0.002561	-0.014901	-0.028311
	0.027958	0.010257	0.022688	0.041195	-0.019106	0.054397
	-0.007138	0.019497	-0.081222	0.011936	0.074727	-0.001805
	0.057067	-0.001328	-0.041721	0.023393	-0.066544	-0.010624
	0.026556	0.001961	0.001938	-0.022273	-0.013608	0.005186
	0.037973					
z18	-0.051961	-0.039122	0.007237	-0.017367	0.044351	0.050035
	0.011541	-0.027817	0.030801	-0.028018	0.040116	0.006209
	-0.019747	0.026579	-0.008535	0.006528	0.008736	-0.034999
	-0.038641	0.082649	-0.000585	-0.022977	0.058226	0.049196
	-0.043972	0.036091	0.047299	-0.030118	0.009240	-0.020225
	-0.029685					
x19	0.015875	-0.015921	-0.038239	0.017987	-0.021398	-0.007682
	-0.061130	-0.011105	-0.066486	0.000990	0.026030	-0.052418
	0.045944	-0.016139	0.010328	-0.008981	-0.000908	0.025281
	0.010925	0.013936	0.004223	0.001063	0.006486	-0.025680
	0.026748	0.004971	-0.032214	0.012411	0.076750	-0.012601
	-0.014501					
y19	-0.048230	-0.001304	0.007700	0.002326	-0.018353	-0.026495
	0.034103	0.011786	0.012386	0.045419	-0.018662	0.044504
	-0.012571	0.018815	-0.078072	0.011343	0.067160	0.010512
	0.071452	-0.017978	-0.046937	0.019695	-0.080782	-0.014414
	0.038487	0.026403	0.007670	-0.009358	-0.014682	0.006185
	0.021394					
z19	-0.047192	-0.039902	0.019613	-0.019510	0.040257	0.045956
	0.013453	-0.021054	0.020108	-0.032289	0.037422	0.024469
	0.004257	0.049374	-0.020700	-0.005232	0.024025	-0.034794
	-0.048053	0.061971	-0.011520	-0.000117	0.069441	0.041131
	-0.061285	0.006304	0.043292	-0.028784	-0.003164	0.025023
	-0.046433					
x20	0.019449	-0.012067	-0.038812	0.019689	-0.027723	-0.017488
	-0.061141	-0.005780	-0.062997	0.006464	0.020598	-0.050211
	0.052747	-0.016284	0.003124	-0.014866	0.019124	0.011362
	0.009223	0.023768	0.009863	-0.001268	0.012145	0.008705
	0.024204	0.028944	-0.044037	-0.010425	0.039256	0.005050
	0.015036					
y20	-0.040050	-0.002705	0.008258	0.001666	-0.022400	-0.021452
	0.032837	0.006307	0.001668	0.041536	-0.014358	0.032521
	-0.005755	0.023814	-0.067696	0.018663	0.055246	0.022506
	0.073509	-0.038870	-0.042460	0.004755	-0.094169	-0.054301
	0.046436	0.014605	0.018676	0.009302	0.037288	0.014270
	-0.046031					

z20	-0.041134	-0.040613	0.032309	-0.021448	0.045939	0.046287
	0.016630	-0.017797	0.012113	-0.045769	0.034926	0.013819
	0.009676	0.045992	-0.018977	-0.001829	0.000232	-0.012303
	-0.050343	0.054079	-0.031463	0.004921	0.071127	0.047228
	-0.048685	-0.012843	0.049170	-0.023800	-0.004299	0.011199
	-0.037284					
x21	0.021026	-0.001809	-0.041493	0.015130	-0.034825	-0.030232
	-0.050882	0.004163	-0.067240	0.006407	0.018990	-0.054115
	0.047973	-0.021616	0.000152	-0.003010	0.019160	0.002865
	-0.003709	0.001451	0.012186	-0.008598	0.008937	0.037229
	0.035993	0.053990	-0.023249	-0.025458	0.030024	0.023885
	0.031741					
y21	-0.028432	0.000154	0.012406	0.004926	-0.021488	-0.010071
	0.030613	0.007614	-0.006731	0.041904	-0.013591	0.026639
	0.010614	0.023591	-0.053402	0.002195	0.044936	0.042355
	0.063426	-0.013016	-0.044041	-0.000887	-0.081967	-0.062010
	0.021867	-0.022456	0.016182	0.031887	0.069497	-0.011907
	-0.102921					
z21	-0.034117	-0.049284	0.041201	-0.017285	0.046123	0.040728
	0.012015	-0.022781	0.005599	-0.059018	0.028044	0.002518
	0.020607	0.055159	-0.021948	0.003386	0.012413	0.000589
	-0.032371	0.051038	-0.040252	-0.013245	0.063814	0.044463
	-0.036855	-0.025188	0.009034	-0.032633	-0.025375	0.021432
	-0.033779					
x22	0.022358	0.003890	-0.049034	0.013070	-0.042741	-0.034690
	-0.042102	-0.002866	-0.061094	0.006040	0.016380	-0.045295
	0.050993	-0.012195	-0.007858	0.016918	0.018072	-0.014003
	-0.015557	-0.002002	0.010920	-0.038387	0.003876	0.052365
	0.051291	0.045558	-0.007671	-0.050977	0.018018	0.027135
	0.045540					
y22	-0.019930	0.001292	0.016952	0.006662	-0.020897	-0.006673
	0.032771	0.011084	-0.015663	0.040774	-0.009360	0.018862
	0.024996	0.036014	-0.033020	-0.008160	0.036529	0.049137
	0.055009	-0.010947	-0.047122	-0.027255	-0.059966	-0.038273
	-0.020951	-0.012194	0.042176	0.042711	0.053412	0.003888
	-0.119818					
z22	-0.025754	-0.049019	0.052873	-0.013627	0.048290	0.032839
	0.007023	-0.014839	-0.006772	-0.068091	0.021457	-0.017275
	0.018283	0.034150	-0.018405	-0.006527	0.022698	0.022313
	-0.022870	0.048065	-0.032490	0.012540	0.048421	0.033425
	-0.012164	-0.031625	-0.035700	-0.019368	-0.011633	0.001784
	-0.035036					

x23	0.018803	0.004767	-0.054375	0.014708	-0.042371	-0.037247
	-0.038496	0.000295	-0.054387	0.008488	0.015284	-0.050166
	0.043461	-0.003941	-0.026563	0.031534	0.017367	-0.031082
	-0.013380	-0.007076	-0.014359	-0.054061	0.001556	0.044910
	0.045680	0.049254	0.011629	-0.074494	0.007519	0.041105
	0.033931					
y23	-0.009587	0.001582	0.017384	0.007211	-0.021762	-0.005961
	0.035463	0.010375	-0.026242	0.045475	-0.007619	0.032466
	0.023579	0.044446	-0.018611	-0.021725	0.040198	0.053876
	0.050151	-0.012389	-0.065047	-0.053142	-0.045867	-0.009972
	-0.052780	-0.011718	0.049852	0.048501	0.020538	0.001019
	-0.139241					
z23	-0.019225	-0.042376	0.061194	-0.008621	0.043789	0.027891
	0.006264	-0.009781	-0.012105	-0.081185	0.016847	-0.030639
	0.032275	0.020637	-0.007846	-0.010410	0.031238	0.031823
	-0.027352	0.049395	0.007240	0.025981	0.034839	0.029454
	0.014910	-0.035930	-0.051535	-0.017853	0.000986	-0.016788
	-0.008443					
x24	0.010903	0.005381	-0.055254	0.019938	-0.037628	-0.030719
	-0.036166	-0.003678	-0.043246	0.003434	0.015918	-0.060433
	0.037681	-0.002308	-0.041841	0.030912	0.016906	-0.042812
	-0.010436	-0.013018	-0.020990	-0.048150	0.009519	0.013799
	0.023239	0.032182	0.010658	-0.085293	0.012813	0.010867
	0.028118					
y24	-0.004170	-0.001065	0.019482	0.011514	-0.016492	-0.001559
	0.031156	0.004540	-0.029753	0.041698	-0.002891	0.024960
	0.020562	0.041115	-0.008832	-0.040937	0.040754	0.060092
	0.042883	-0.010390	-0.065118	-0.050931	-0.017722	0.001361
	-0.086688	0.006793	0.054822	0.052880	0.016158	-0.010145
	-0.139983					
z24	-0.016324	-0.032631	0.059007	-0.001718	0.033904	0.019703
	0.007902	-0.003633	-0.014226	-0.078216	0.010437	-0.021122
	0.039606	0.018721	-0.007420	-0.001319	0.034523	0.018090
	-0.032747	0.054466	0.029663	0.010160	0.011730	0.038150
	0.040641	-0.034429	-0.038279	-0.022711	-0.013039	-0.005911
	0.010410					
x25	-0.001206	0.011511	-0.044287	0.026321	-0.029347	-0.029432
	-0.033065	-0.006639	-0.038018	-0.014190	0.008353	-0.068822
	0.042696	0.008753	-0.045325	0.008466	0.018409	-0.038397
	-0.003188	-0.001977	-0.015338	-0.038983	0.024598	-0.009895
	0.023107	0.023646	0.021527	-0.055633	0.011623	-0.057149
	0.020449					

y25	-0.005164	-0.001630	0.028333	0.015976	-0.006697	-0.001422
	0.026390	0.000010	-0.036493	0.028951	-0.004849	0.007904
	0.020314	0.049210	0.003681	-0.064529	0.041983	0.060822
	0.042417	-0.011754	-0.061498	-0.047403	0.010807	0.011409
	-0.092416	0.037586	0.082399	0.066074	0.011252	-0.038484
	-0.111135					
z25	-0.017090	-0.026551	0.051152	0.010809	0.026985	0.013305
	0.005678	-0.001967	-0.014980	-0.067259	0.010288	-0.003339
	0.043978	0.014240	-0.020871	0.007100	0.020251	-0.002632
	-0.035497	0.053209	0.046193	-0.004447	0.000246	0.031084
	0.055331	-0.041495	-0.027161	-0.033324	-0.010067	0.001349
	0.013364					
x26	-0.013027	0.010611	-0.042331	0.031089	-0.036036	-0.028391
	-0.023845	-0.011517	-0.024113	-0.035319	-0.016890	-0.071467
	0.031279	0.033684	-0.065637	0.024308	0.005788	-0.037194
	0.007976	0.001277	-0.013577	-0.045336	0.022758	-0.025451
	-0.001596	-0.002626	0.016783	-0.062418	0.022039	-0.048665
	-0.027007					
y26	-0.004945	-0.009967	0.030314	0.017179	-0.010374	-0.010166
	0.032377	-0.004137	-0.039900	0.020092	-0.017637	0.007955
	0.005926	0.070871	0.000086	-0.057501	0.052416	0.043847
	0.022085	-0.025951	-0.072630	-0.060710	0.022842	0.014284
	-0.124184	0.042785	0.073528	0.036664	0.003565	-0.009675
	-0.107615					
z26	-0.019643	-0.022500	0.040940	0.026310	0.025040	0.006145
	0.000884	0.004919	-0.017076	-0.052297	0.017187	0.007614
	0.042103	0.002997	-0.037468	0.009172	0.003345	-0.023531
	-0.026417	0.043923	0.060866	-0.025097	-0.001910	0.017175
	0.051228	-0.033317	-0.020377	-0.029952	-0.012487	-0.001339
	0.013589					
x27	-0.024023	0.016681	-0.041905	0.033120	-0.035739	-0.019011
	-0.014346	-0.013812	-0.018452	-0.038604	-0.004369	-0.067725
	0.016739	0.047400	-0.086133	0.014899	0.011367	-0.030415
	0.007903	0.021438	-0.021059	-0.038349	0.013705	-0.021849
	-0.015936	-0.023557	-0.010938	-0.072002	0.005833	-0.034469
	0.017985					
y27	-0.004385	-0.016311	0.028539	0.020766	-0.007222	-0.014380
	0.035324	-0.008556	-0.053029	0.020224	-0.009806	0.007757
	-0.003437	0.081195	-0.015405	-0.067581	0.074640	0.026896
	0.010050	-0.041627	-0.084528	-0.068707	0.022319	0.027035
	-0.115281	0.061856	0.048432	-0.002278	-0.041150	0.003143
	-0.003792					

z27	-0.021726	-0.022324	0.029346	0.044854	0.020512	-0.003819
	-0.003682	0.013560	-0.021246	-0.036174	0.023623	0.018215
	0.041695	-0.003183	-0.054179	0.012187	-0.022490	-0.041870
	-0.016964	0.023327	0.074472	-0.048098	0.006306	0.004844
	0.033010	-0.035204	-0.013669	-0.021128	-0.004949	-0.004839
	-0.008161					
x28	-0.036747	0.021819	-0.042308	0.031707	-0.037941	-0.008234
	0.002756	-0.007018	-0.019975	-0.033167	0.020811	-0.047704
	0.000801	0.048940	-0.088100	0.012185	0.001831	-0.014904
	-0.005482	0.061078	-0.024063	-0.027865	0.031114	-0.011374
	-0.029834	-0.034608	-0.050136	-0.050076	-0.004184	-0.014713
	0.049655					
y28	-0.002414	-0.026484	0.021285	0.021029	-0.001914	-0.018451
	0.046031	-0.009409	-0.070072	0.025401	0.013860	0.021188
	-0.005969	0.090716	0.000699	-0.053641	0.083100	0.008211
	-0.007339	-0.045240	-0.075145	-0.089687	0.028702	0.044483
	-0.075789	0.093831	-0.004686	-0.005935	-0.077297	-0.013621
	0.076955					
z28	-0.023329	-0.023662	0.016338	0.062425	0.015839	-0.012797
	-0.007392	0.021624	-0.027345	-0.022258	0.032920	0.028569
	0.043908	-0.007764	-0.075795	0.013519	-0.048721	-0.052179
	-0.005790	0.002135	0.081517	-0.066746	0.019700	-0.002519
	0.002577	-0.038101	-0.010281	-0.010995	0.001591	-0.011271
	-0.020988					
x29	-0.048248	0.022229	-0.038865	0.043499	-0.042479	0.002099
	0.010755	0.014040	-0.014717	-0.053129	0.040780	-0.035694
	0.011368	0.061724	-0.072639	0.021479	-0.039758	0.004007
	-0.007240	0.049619	0.002041	-0.029908	0.031562	-0.013740
	-0.034552	-0.039084	-0.029468	-0.034421	0.002623	-0.014901
	0.045738					
y29	0.002698	-0.039757	0.019959	0.016756	0.003924	-0.039418
	0.048408	-0.010905	-0.083276	0.033540	0.022668	0.033097
	-0.016809	0.095349	0.051426	-0.045641	0.050319	-0.019904
	-0.025264	-0.090822	-0.057624	-0.104409	0.018027	0.009196
	0.015260	0.096916	-0.004494	-0.005596	-0.070222	-0.121518
	0.049134					
z29	-0.023291	-0.027488	0.000865	0.087526	0.006425	-0.017200
	-0.008155	0.026757	-0.025087	-0.029575	0.049146	0.031182
	0.070710	0.009454	-0.091799	0.042202	-0.086226	-0.043313
	0.008955	-0.043217	0.114814	-0.068511	0.019093	0.003408
	-0.041195	-0.032841	0.011222	0.007691	0.012731	-0.012206
	-0.022364					

x30	-0.056076	0.026077	-0.035787	0.053145	-0.032543	0.019286
	0.017079	0.025826	-0.013877	-0.071762	0.057646	-0.014491
	0.026774	0.076198	-0.064997	0.012427	-0.055694	0.027224
	-0.032419	0.021498	-0.011358	-0.052629	0.031729	-0.008007
	-0.032669	-0.045439	-0.024270	-0.019416	0.020559	-0.034749
	0.049628					
y30	0.014421	-0.043539	0.020042	0.024324	0.005351	-0.058489
	0.039152	-0.006332	-0.092127	0.031664	0.001479	0.042477
	-0.010974	0.082980	0.063057	-0.056265	0.011129	-0.071172
	-0.033395	-0.065174	-0.011153	-0.122979	-0.004780	0.017632
	0.057633	0.040311	-0.011364	0.003863	-0.031977	-0.130566
	-0.038549					
z30	-0.027429	-0.028948	-0.011998	0.102606	0.001199	-0.011826
	-0.002498	0.022137	-0.018139	-0.043948	0.070424	0.033817
	0.102025	0.030221	-0.111135	0.061884	-0.085169	0.006235
	0.012233	-0.091406	0.099440	-0.063626	0.030805	0.020263
	-0.068390	-0.022899	0.032502	0.012891	0.012600	-0.038279
	0.020169					
x31	-0.057759	0.029472	-0.030580	0.048956	-0.023165	0.024772
	0.018205	0.037315	-0.013133	-0.067443	0.064356	-0.007030
	0.028548	0.062447	-0.040275	0.005554	-0.044664	0.026615
	-0.033478	0.018785	-0.030129	-0.050272	0.034318	0.000446
	-0.033563	-0.054468	-0.021203	-0.012697	0.037508	-0.016019
	0.053913					
y31	0.029159	-0.044343	0.013442	0.020652	0.005361	-0.077767
	0.023362	-0.013862	-0.092244	0.028716	-0.018684	0.029418
	-0.012661	0.056124	0.069008	-0.053527	0.002987	-0.111520
	-0.020891	-0.036290	0.027307	-0.100464	-0.044797	0.028482
	0.041569	0.020888	0.005734	-0.023007	-0.003476	-0.092542
	-0.057470					
z31	-0.021947	-0.024357	-0.018492	0.097633	-0.002096	-0.029287
	0.002202	0.014704	-0.005291	-0.031171	0.074054	0.012239
	0.095709	0.022003	-0.102968	0.068696	-0.075907	0.016666
	0.021405	-0.096753	0.102979	-0.050371	0.028560	0.046100
	-0.074335	-0.019999	0.050766	0.005943	0.013453	-0.018553
	0.022051					
x32	-0.057224	0.036329	-0.029265	0.041577	-0.011791	0.033288
	0.017752	0.044961	-0.011586	-0.062299	0.069069	-0.000486
	0.031305	0.050313	-0.022907	0.001518	-0.029517	0.023653
	-0.026996	0.010775	-0.045204	-0.046058	0.027647	0.005052
	-0.024400	-0.064899	-0.019737	-0.019156	0.040904	-0.003938
	0.053882					

y32	0.043643	-0.041330	0.007737	0.016462	-0.004240	-0.095125
	0.002994	-0.021097	-0.093853	0.019232	-0.045469	0.011743
	-0.013121	0.031330	0.065367	-0.059218	0.000252	-0.133911
	-0.004845	0.006003	0.060902	-0.070291	-0.068675	0.023291
	0.035379	0.011227	0.036403	-0.039414	0.008798	-0.081540
	-0.077655					
z32	-0.009991	-0.015460	-0.019547	0.088898	-0.020815	-0.053677
	-0.001491	-0.003175	0.010826	-0.019197	0.074373	-0.009759
	0.074149	0.010813	-0.066048	0.090900	-0.061054	0.028233
	0.035088	-0.115923	0.111129	-0.014033	0.017941	0.044363
	-0.072899	0.006718	0.074739	0.026962	0.012881	-0.039877
	-0.037186					
x33	-0.053831	0.040550	-0.028212	0.033736	-0.002564	0.039302
	0.016551	0.046868	-0.014319	-0.059445	0.071231	0.001549
	0.031607	0.035834	0.003231	0.005263	-0.011092	0.015106
	-0.013218	-0.008235	-0.059310	-0.044932	0.031477	-0.003344
	-0.013846	-0.059866	-0.026424	-0.023529	0.049042	-0.020752
	0.051355					
y33	0.056253	-0.037483	0.002944	0.015419	-0.006817	-0.108537
	-0.016334	-0.030847	-0.082193	0.004637	-0.070479	-0.008572
	-0.012547	0.013302	0.049244	-0.075883	-0.016221	-0.151158
	-0.000206	0.051292	0.080879	-0.038297	-0.082955	0.025375
	0.020182	0.021971	0.035536	-0.035364	0.005382	-0.038285
	-0.061377					
z33	-0.001313	-0.004122	-0.018499	0.079533	-0.030787	-0.079168
	-0.002821	-0.018813	0.029695	-0.009901	0.067071	-0.029882
	0.048138	-0.009103	-0.047339	0.094801	-0.047021	0.036246
	0.037274	-0.123292	0.115983	0.013263	0.009107	0.032611
	-0.056297	0.046503	0.055886	0.038519	0.002316	-0.035024
	-0.053245					
x34	-0.045385	0.044700	-0.030488	0.024360	0.004635	0.042059
	0.015147	0.047657	-0.007008	-0.057381	0.071371	0.004243
	0.036181	0.026248	0.027940	0.007215	0.009510	0.004338
	0.000704	-0.017866	-0.053507	-0.048729	0.034649	0.001328
	0.006484	-0.052990	-0.016152	-0.024921	0.053408	-0.022855
	0.045626					
y34	0.064159	-0.034530	0.003240	0.019100	-0.005632	-0.112512
	-0.034910	-0.044696	-0.072215	-0.011512	-0.085683	-0.029676
	-0.014984	-0.001470	0.028056	-0.087029	-0.041682	-0.155738
	0.001653	0.087092	0.065927	-0.001082	-0.083331	0.035384
	-0.019119	0.033493	0.014052	-0.012944	-0.007623	-0.006310
	-0.041921					

z34	0.000455	0.002024	-0.009100	0.073032	-0.035979	-0.104302
	0.001458	-0.038303	0.030392	-0.006633	0.054829	-0.058042
	0.015738	-0.035103	-0.045750	0.088079	-0.039881	0.037321
	0.033197	-0.113829	0.083132	0.034894	0.018334	0.030451
	-0.073517	0.054839	-0.005664	0.052998	-0.016348	-0.032889
	-0.024641					
x35	-0.032381	0.044921	-0.034106	0.010584	0.008193	0.034372
	0.016102	0.034051	0.003204	-0.053180	0.062639	0.001336
	0.021903	0.003874	0.045036	0.010025	0.023235	-0.007500
	-0.005703	-0.018260	-0.055903	-0.035768	0.047376	0.009038
	0.022809	-0.040382	0.003733	-0.010184	0.058642	-0.024395
	-0.011492					
y35	0.067958	-0.031375	0.005490	0.025819	-0.001854	-0.103150
	-0.053648	-0.055065	-0.062230	-0.026971	-0.086312	-0.036171
	-0.011709	-0.012727	0.002639	-0.085582	-0.071238	-0.140411
	0.002319	0.101740	0.046437	0.033923	-0.083579	0.029944
	-0.060666	0.030952	-0.006293	0.007913	-0.014285	0.007200
	0.006826					
z35	-0.001657	0.008245	-0.001240	0.066372	-0.036664	-0.110983
	0.005801	-0.032203	0.029529	-0.001522	0.050341	-0.060348
	0.011384	-0.050599	-0.049708	0.066587	-0.026105	0.032867
	0.057887	-0.089426	0.073786	0.042174	0.002624	0.027740
	-0.068502	0.017836	-0.059437	0.030491	-0.027238	-0.007258
	0.065781					
x36	-0.016649	0.045726	-0.038815	-0.005430	0.009284	0.020282
	0.010435	0.013385	0.013155	-0.044931	0.050576	-0.006787
	0.001395	-0.025760	0.053131	0.008412	0.026619	-0.028085
	-0.016814	-0.011160	-0.055202	-0.027267	0.048428	0.013664
	0.040439	-0.038613	0.016241	-0.010036	0.053538	-0.049549
	-0.045328					
y36	0.064323	-0.028684	0.009998	0.034354	0.006228	-0.081276
	-0.063789	-0.059549	-0.054932	-0.043828	-0.082683	-0.033450
	-0.008275	-0.025874	-0.024865	-0.071365	-0.096305	-0.101495
	-0.008379	0.082389	0.005684	0.059593	-0.074914	0.014931
	-0.099724	0.031040	-0.039268	0.024163	-0.011732	0.033564
	0.032726					
z36	-0.006057	0.012897	0.000718	0.056989	-0.029430	-0.105186
	0.022563	-0.013670	0.029978	0.002623	0.044554	-0.047726
	0.014495	-0.061427	-0.049278	0.040070	-0.012071	0.036184
	0.081669	-0.064269	0.069551	0.049810	-0.014364	0.031293
	-0.049517	-0.008747	-0.075773	0.015202	-0.035497	0.056476
	0.088190					

x37	-0.001863	0.042674	-0.044706	-0.025520	0.011338	0.002559
	0.005473	-0.009612	0.029221	-0.034239	0.034255	-0.022392
	-0.024959	-0.072580	0.047025	0.007544	0.020277	-0.053543
	-0.022507	-0.009604	-0.048006	-0.011956	0.020808	0.008222
	0.050325	-0.040014	-0.010278	-0.043793	0.035186	-0.059155
	-0.049184					
y37	0.054405	-0.021480	0.014177	0.046474	0.014240	-0.045867
	-0.070132	-0.054327	-0.057918	-0.060134	-0.074888	-0.023035
	0.001210	-0.027551	-0.047907	-0.056128	-0.094505	-0.047396
	-0.016409	0.051659	-0.042883	0.055131	-0.033700	-0.007785
	-0.122014	0.026137	-0.049389	0.046919	-0.015946	0.025535
	0.047238					
z37	-0.003831	0.019462	-0.001019	0.048309	-0.021969	-0.094182
	0.043570	-0.001974	0.022286	0.003343	0.041622	-0.032055
	0.012498	-0.046427	-0.010646	0.017402	0.008077	0.028029
	0.096569	-0.045767	0.084575	0.046885	-0.004148	0.032379
	-0.020181	-0.025675	-0.030196	0.050219	-0.016832	0.070021
	0.028076					
x38	0.007333	0.032968	-0.045366	-0.041606	0.003651	-0.013717
	0.009588	-0.019770	0.045628	-0.024536	0.018737	-0.028565
	-0.052241	-0.119808	0.038603	0.014577	0.021490	-0.074749
	-0.004412	-0.035451	-0.053233	0.017875	0.009493	0.014687
	0.056460	-0.066971	-0.056253	-0.036051	0.026634	-0.047338
	-0.059220					
y38	0.040552	-0.017125	0.017348	0.055992	0.022884	-0.009142
	-0.077632	-0.077355	-0.067419	-0.070414	-0.063794	-0.013397
	-0.003614	-0.038588	-0.087736	-0.043631	-0.093781	0.019919
	-0.040262	0.011415	-0.079280	0.062206	0.026715	-0.048275
	-0.139203	0.037891	-0.057722	0.065778	-0.024899	-0.022643
	0.034113					
z38	-0.000247	0.027451	-0.010105	0.036671	-0.002720	-0.081627
	0.059833	0.010837	0.014010	0.006724	0.037540	-0.016165
	0.024377	-0.014816	0.024465	-0.000963	0.011830	-0.001675
	0.083704	-0.005333	0.108387	0.016422	-0.035218	0.035137
	0.002027	-0.035523	0.045260	0.029011	-0.023389	0.080254
	0.004501					
x39	0.015496	0.019373	-0.039336	-0.041072	0.007485	-0.017491
	0.024652	-0.028563	0.039268	-0.033750	0.017351	-0.033220
	-0.057141	-0.143252	0.017933	0.027062	0.010246	-0.085184
	0.006967	-0.025484	-0.047721	0.026638	0.004384	0.018532
	0.022234	-0.062018	-0.049209	-0.003881	0.023532	-0.026797
	-0.058047					

y39	0.027590	-0.017609	0.019869	0.051771	0.023245	-0.003027
	-0.075349	-0.088559	-0.069172	-0.077455	-0.043100	-0.011036
	-0.011308	-0.032053	-0.080066	-0.041430	-0.088067	-0.004906
	-0.032875	-0.006007	-0.067132	0.080538	0.024597	-0.026144
	-0.085230	0.034550	-0.034444	0.060100	-0.028533	-0.007285
	0.007195					
z39	0.002940	0.034480	-0.018483	0.035277	0.003714	-0.076235
	0.051718	0.015300	0.016674	0.020754	0.022840	-0.004430
	0.030832	-0.001483	0.040270	-0.001902	0.003824	0.018670
	0.071694	0.018631	0.089479	0.008964	-0.034596	0.012144
	-0.011748	-0.033507	0.049905	0.029994	-0.019348	0.056744
	0.018558					
x40	0.016352	0.009866	-0.038928	-0.042448	0.008174	-0.020374
	0.029463	-0.023158	0.027900	-0.037407	0.025152	-0.029547
	-0.056306	-0.162338	0.005280	0.026012	0.012066	-0.080104
	0.024418	-0.029961	-0.050608	0.014177	-0.002458	0.031463
	0.030283	-0.049698	-0.036552	-0.015710	0.017859	-0.024351
	-0.063733					
y40	0.015876	-0.011819	0.016938	0.043152	0.021179	0.004315
	-0.074345	-0.082465	-0.063618	-0.097044	-0.025342	-0.006342
	-0.010962	-0.018652	-0.068814	-0.047740	-0.084300	-0.027258
	-0.028833	-0.015273	-0.066081	0.090738	0.000266	-0.013090
	-0.031966	0.014490	-0.008435	0.058453	-0.033346	0.028912
	0.018587					
z40	0.004561	0.036443	-0.021401	0.032079	0.009040	-0.070591
	0.045870	0.008279	0.022627	0.038378	0.009638	0.001689
	0.032496	0.004167	0.046460	0.002178	-0.004948	0.036882
	0.058864	0.031052	0.077105	0.018462	-0.014550	-0.003294
	-0.020411	-0.035649	0.059354	0.032281	-0.018212	0.022144
	0.012819					
x41	0.015039	0.005327	-0.040798	-0.037849	0.014140	-0.018843
	0.033967	-0.016403	0.019798	-0.039500	0.028657	-0.017976
	-0.044221	-0.161094	0.007302	0.028861	0.004090	-0.068782
	0.030561	-0.025973	-0.032931	0.012336	-0.002120	0.042793
	0.039050	-0.041000	-0.021555	-0.005478	0.003941	-0.027970
	-0.044310					
y41	0.004217	-0.007898	0.014465	0.033931	0.019702	0.009856
	-0.073202	-0.073416	-0.058344	-0.111898	-0.013383	-0.002600
	-0.009675	-0.007971	-0.052972	-0.051970	-0.079223	-0.042373
	-0.016703	-0.023590	-0.062944	0.092924	-0.019093	-0.002869
	0.012228	0.003100	0.016698	0.050849	-0.040068	0.068696
	0.016548					

z41	0.005946	0.037466	-0.025578	0.026529	0.012152	-0.064618
	0.040360	-0.001398	0.026578	0.053300	-0.001439	0.002543
	0.034296	0.008049	0.047674	0.000905	-0.010122	0.051361
	0.046466	0.037063	0.065234	0.024204	0.006553	-0.014553
	-0.021158	-0.043546	0.064980	0.028724	-0.018799	-0.014613
	0.005918					
x42	0.007459	0.001525	-0.042050	-0.033503	0.020109	-0.012682
	0.037397	-0.013044	0.015837	-0.043937	0.032665	-0.003913
	-0.042057	-0.151195	0.019034	0.029903	-0.013649	-0.058568
	0.042221	-0.034370	-0.020812	0.014412	-0.007477	0.053065
	0.025935	-0.029361	-0.000019	-0.012374	0.003885	-0.015107
	-0.043030					
y42	-0.005619	-0.004978	0.010353	0.026931	0.018541	0.012377
	-0.070347	-0.068834	-0.048800	-0.114970	-0.008211	0.002026
	-0.005557	0.000679	-0.038707	-0.052103	-0.064664	-0.050506
	-0.011079	-0.021667	-0.052000	0.086384	-0.032528	0.013683
	0.070290	-0.008216	0.025501	0.053216	-0.052375	0.075453
	0.021368					
z42	0.007792	0.038781	-0.029317	0.019824	0.015368	-0.059186
	0.035286	-0.009442	0.025970	0.062285	-0.011267	0.000519
	0.037657	0.010491	0.047823	-0.002206	-0.016539	0.064161
	0.033857	0.039375	0.051991	0.033688	0.026203	-0.029059
	-0.027503	-0.044331	0.068791	0.013933	-0.016653	-0.030419
	-0.008783					
x43	-0.003749	0.001239	-0.046028	-0.032947	0.028975	-0.007124
	0.039183	-0.008515	0.012587	-0.042854	0.030697	0.009311
	-0.041297	-0.138864	0.031111	0.016005	-0.028610	-0.044101
	0.040815	-0.033845	-0.011029	0.014385	-0.012647	0.064325
	0.024622	-0.026542	0.007210	-0.032066	-0.006350	-0.004432
	-0.049776					
y43	-0.014999	-0.005215	0.006048	0.021889	0.017410	0.012517
	-0.067617	-0.065042	-0.037810	-0.108308	-0.004421	0.001927
	-0.002481	0.007996	-0.025315	-0.043574	-0.048358	-0.048379
	-0.003752	-0.020463	-0.039375	0.073206	-0.036532	0.024654
	0.109967	-0.009845	0.035422	0.060297	-0.060923	0.065270
	0.024686					
z43	0.011152	0.040323	-0.032240	0.014651	0.016921	-0.053204
	0.032109	-0.017849	0.023617	0.064486	-0.019846	0.000875
	0.040698	0.012348	0.047075	-0.004426	-0.022749	0.073948
	0.023421	0.039910	0.036502	0.047519	0.038826	-0.041060
	-0.029444	-0.043197	0.072459	0.002746	-0.012841	-0.032198
	-0.020220					

x44	-0.012849	-0.001538	-0.049171	-0.034159	0.034194	-0.001218
	0.040532	-0.006852	0.012732	-0.039117	0.024136	0.019440
	-0.038637	-0.128439	0.039676	-0.001779	-0.037563	-0.019996
	0.029704	-0.023471	0.002118	0.014882	-0.008370	0.064152
	0.025647	-0.025869	0.014308	-0.058430	-0.008135	-0.001773
	-0.075564					
y44	-0.026702	-0.006527	0.000789	0.019201	0.020687	0.008330
	-0.065993	-0.054861	-0.026540	-0.093718	-0.001069	-0.005187
	-0.000824	0.017260	-0.010741	-0.029023	-0.038726	-0.038546
	0.007625	-0.025717	-0.027217	0.054673	-0.032697	0.027857
	0.116047	-0.003479	0.054358	0.072914	-0.067199	0.058450
	0.021391					
z44	0.015166	0.043045	-0.034766	0.008748	0.016547	-0.048662
	0.030036	-0.026258	0.017799	0.061135	-0.028528	0.003615
	0.041740	0.011063	0.046896	-0.009437	-0.029310	0.080921
	0.017213	0.034948	0.016621	0.066926	0.044724	-0.044436
	-0.017061	-0.040304	0.075665	-0.005085	-0.015422	-0.025104
	-0.017951					
x45	-0.023218	-0.004248	-0.054230	-0.035106	0.036707	0.004933
	0.044905	-0.006748	0.015217	-0.031745	0.017691	0.029874
	-0.043146	-0.103208	0.047834	-0.012323	-0.046609	0.002715
	0.009601	-0.012560	0.008403	0.023845	0.001626	0.066995
	0.027433	-0.019576	0.035808	-0.070000	-0.019672	0.003565
	-0.088816					
y45	-0.036760	-0.008573	-0.003208	0.017790	0.026234	-0.000223
	-0.067820	-0.039384	-0.013183	-0.074871	0.000041	-0.016179
	0.000712	0.018000	0.002595	-0.016570	-0.029999	-0.020217
	0.024860	-0.035408	-0.009604	0.032199	-0.023099	0.020405
	0.092836	0.000908	0.071632	0.079867	-0.064070	0.038647
	0.010323					
z45	0.019512	0.045182	-0.036574	0.001224	0.015527	-0.046106
	0.026941	-0.034076	0.009371	0.053571	-0.035482	0.003350
	0.046783	0.003256	0.045523	-0.015761	-0.037057	0.089827
	0.014452	0.025621	-0.003278	0.087224	0.048600	-0.045714
	0.003409	-0.038373	0.077763	-0.014412	-0.012762	-0.011473
	-0.028194					
x46	-0.031142	-0.005600	-0.062010	-0.034322	0.039963	0.015101
	0.046027	-0.008411	0.022570	-0.020419	0.014779	0.042616
	-0.049083	-0.076361	0.058518	-0.020286	-0.059983	0.020311
	-0.006232	-0.007091	0.009972	0.025584	0.007078	0.068488
	0.019459	-0.007886	0.047046	-0.079009	-0.024468	0.011010
	-0.085346					

y46	-0.045463	-0.007287	-0.005628	0.016511	0.031666	-0.011360
	-0.070419	-0.030565	0.001132	-0.054970	-0.004239	-0.020834
	-0.001073	0.016869	0.012677	-0.007704	-0.020182	-0.002197
	0.038294	-0.034890	0.013964	0.013281	-0.007163	0.010054
	0.070560	-0.000170	0.080671	0.085736	-0.062220	-0.004769
	-0.004541					
z46	0.020849	0.044968	-0.037292	-0.007908	0.012153	-0.043738
	0.026047	-0.042451	-0.001923	0.044084	-0.040634	-0.000784
	0.053973	-0.001114	0.041084	-0.019991	-0.044387	0.100711
	0.013181	0.012202	-0.025510	0.112581	0.054168	-0.040556
	0.038587	-0.048388	0.085663	-0.019642	-0.017760	0.002286
	-0.041526					
x47	-0.001018	-0.044840	-0.053619	-0.033896	-0.022627	-0.001410
	0.076952	0.093860	-0.015854	0.028401	-0.043590	-0.028021
	0.051197	-0.050752	0.133483	-0.028702	-0.016167	-0.001490
	0.032733	-0.032931	0.049613	-0.011826	-0.000229	-0.041753
	0.017120	0.093569	-0.025378	0.012215	-0.126970	0.008389
	-0.011086					
y47	-0.064350	-0.009032	-0.009205	0.005050	0.092691	-0.015228
	-0.126565	-0.010571	0.001059	0.066239	0.021703	-0.039988
	-0.010631	-0.061960	0.020700	0.027082	0.006358	0.049173
	-0.072543	-0.039849	-0.102500	-0.123448	-0.084647	0.026760
	0.044238	-0.095345	-0.095148	-0.053404	0.009992	0.003318
	-0.020994					
z47	0.084567	0.031914	-0.058384	-0.022999	-0.001865	-0.091711
	0.019738	-0.083821	-0.005682	0.043388	-0.044844	-0.004407
	-0.022170	-0.066313	0.080043	-0.039724	-0.040696	0.010827
	-0.001591	0.059459	-0.005497	-0.137452	-0.012072	-0.020920
	-0.100293	0.007414	-0.001139	-0.015771	0.013815	-0.047799
	0.027163					
x48	-0.000849	-0.048822	-0.050069	-0.035790	-0.016822	0.005853
	0.070699	0.095350	-0.011310	0.029532	-0.038500	-0.021066
	0.040192	-0.045871	0.110539	-0.022032	-0.022478	-0.004509
	0.034593	-0.041088	0.047025	-0.015816	-0.016109	-0.031740
	0.020247	0.077375	-0.030224	-0.000371	-0.126978	0.021271
	0.004272					
y48	-0.070427	-0.004571	-0.012704	0.005046	0.085013	-0.020326
	-0.119982	-0.011020	0.004006	0.062659	0.021854	-0.037161
	-0.004714	-0.050782	0.019960	0.030140	0.003728	0.051427
	-0.077515	-0.026845	-0.099019	-0.110852	-0.078495	0.031372
	0.040681	-0.062027	-0.065560	-0.045644	-0.001568	0.009873
	-0.014451					

z48	0.079894	0.028749	-0.054226	-0.025633	-0.000922	-0.082590
	0.016740	-0.069388	-0.006733	0.048869	-0.043126	-0.002588
	-0.019038	-0.053936	0.060223	-0.036457	-0.043058	0.010244
	0.000971	0.046176	-0.017505	-0.116535	-0.031333	-0.021431
	-0.080001	-0.003603	-0.017466	-0.012641	0.013845	-0.035328
	0.025739					
x49	-0.004037	-0.050243	-0.043588	-0.036886	-0.011191	0.010790
	0.066353	0.091156	-0.010151	0.028363	-0.034533	-0.010366
	0.037893	-0.034719	0.096316	-0.024769	-0.029658	-0.001282
	0.028503	-0.043083	0.043698	-0.011005	-0.014129	-0.026844
	0.025157	0.061661	-0.022489	0.001890	-0.116538	0.022272
	-0.000793					
y49	-0.078132	-0.001190	-0.012280	0.002925	0.075414	-0.028087
	-0.110993	-0.008649	0.001837	0.060980	0.018616	-0.034059
	0.008243	-0.038493	0.029583	0.024903	-0.004640	0.061665
	-0.080655	-0.022105	-0.093296	-0.092795	-0.051835	0.031201
	0.031053	-0.032063	-0.036058	-0.033258	-0.008080	0.018532
	-0.034239					
z49	0.074152	0.024271	-0.048684	-0.028609	-0.001608	-0.075619
	0.015269	-0.053344	-0.009629	0.052407	-0.042904	-0.004839
	-0.012451	-0.043545	0.047778	-0.032028	-0.045639	0.014442
	0.002822	0.027627	-0.027654	-0.097567	-0.037112	-0.027872
	-0.071859	-0.013240	-0.028029	-0.004330	0.014809	-0.017064
	0.012073					
x50	-0.006677	-0.052213	-0.046569	-0.033498	-0.007493	0.016760
	0.062435	0.088840	-0.002591	0.026480	-0.025659	-0.004480
	0.025024	-0.028248	0.076499	-0.024413	-0.024460	-0.002847
	0.026094	-0.043920	0.045628	-0.001219	-0.019670	-0.025148
	0.014544	0.047399	-0.031701	0.000677	-0.110009	0.001026
	0.019931					
y50	-0.084131	0.001556	-0.018333	0.004523	0.067609	-0.029132
	-0.106749	-0.003747	0.007691	0.054982	0.019257	-0.041664
	0.014736	-0.038429	0.033124	0.019399	-0.001274	0.062909
	-0.072986	-0.010028	-0.067246	-0.069675	-0.038629	0.026559
	0.026904	-0.023714	-0.039604	-0.032024	-0.009376	-0.023510
	-0.013902					
z50	0.069154	0.020023	-0.048090	-0.029162	-0.003106	-0.065937
	0.015700	-0.037398	-0.010706	0.053976	-0.041473	-0.007542
	-0.009478	-0.034188	0.030333	-0.027962	-0.037808	0.010560
	0.011619	0.012448	-0.034363	-0.075233	-0.047567	-0.033517
	-0.059607	-0.032138	-0.053345	-0.000561	0.007192	-0.025278
	0.023358					

x51	-0.010527	-0.053505	-0.047756	-0.031355	-0.004845	0.023654
	0.057152	0.081595	0.004032	0.020863	-0.016642	0.000721
	0.020417	-0.021361	0.057147	-0.021188	-0.016606	-0.000747
	0.019238	-0.038905	0.036321	0.006628	-0.018150	-0.018418
	-0.003318	0.030842	-0.024667	0.009856	-0.102124	-0.004341
	0.025901					
y51	-0.090464	0.003121	-0.019080	0.000732	0.060345	-0.027567
	-0.104976	-0.001469	0.011976	0.045334	0.015546	-0.047984
	0.026907	-0.033942	0.031313	0.015988	0.002424	0.060313
	-0.059443	-0.002695	-0.065348	-0.057123	-0.021588	0.030398
	0.016556	-0.021303	-0.021350	-0.020398	-0.019135	-0.037832
	-0.007980					
z51	0.062786	0.016029	-0.044893	-0.031383	-0.004614	-0.055242
	0.015551	-0.024906	-0.012701	0.051144	-0.041069	-0.010805
	-0.000661	-0.026572	0.011903	-0.023102	-0.028196	0.009165
	0.016637	0.003217	-0.051181	-0.055202	-0.049125	-0.032432
	-0.050814	-0.053552	-0.059894	0.008145	-0.001733	-0.019917
	0.025642					
x52	-0.012505	-0.055923	-0.047551	-0.025376	-0.000645	0.028498
	0.055214	0.073891	0.007229	0.015175	-0.007069	0.005110
	0.006667	-0.017083	0.037913	-0.023692	-0.013278	0.002428
	0.018331	-0.038663	0.033181	0.001529	-0.021421	-0.013343
	-0.010835	0.022865	-0.010348	0.014040	-0.091393	0.001354
	0.038134					
y52	-0.093312	0.002741	-0.016567	-0.000186	0.056010	-0.026627
	-0.095787	-0.000851	0.011658	0.039507	0.012110	-0.058343
	0.032293	-0.034860	0.028452	0.013390	-0.004624	0.059231
	-0.044237	-0.002350	-0.046451	-0.060346	-0.002250	0.033509
	0.016135	-0.015158	-0.018005	-0.019255	-0.021788	-0.018679
	-0.001706					
z52	0.056772	0.009080	-0.040615	-0.030562	-0.005029	-0.047801
	0.019754	-0.013570	-0.017319	0.047552	-0.037471	-0.018592
	-0.000097	-0.018295	-0.005507	-0.018930	-0.023137	0.011777
	0.025226	-0.014691	-0.057651	-0.047219	-0.046954	-0.030465
	-0.035855	-0.061717	-0.056095	0.016132	-0.010920	0.002399
	0.035407					
x53	-0.015653	-0.057422	-0.049071	-0.023158	0.002025	0.034110
	0.049701	0.064652	0.012793	0.008841	0.000905	0.008613
	-0.001165	-0.013194	0.022691	-0.029435	-0.013574	0.003293
	0.012614	-0.036126	0.032541	0.003243	-0.013497	-0.012907
	-0.016175	0.016638	-0.006855	0.022056	-0.077107	-0.002306
	0.044820					

y53	-0.098142	0.002161	-0.016708	-0.006894	0.050298	-0.021735
	-0.089851	-0.001088	0.014697	0.032277	0.005772	-0.066029
	0.037546	-0.031995	0.030595	0.002824	-0.017926	0.056284
	-0.032040	-0.007678	-0.027058	-0.058663	0.016104	0.030442
	0.018592	-0.019072	-0.028440	-0.005240	-0.015668	-0.011754
	0.009049					
z53	0.048766	0.002557	-0.038482	-0.033057	-0.005900	-0.036889
	0.021496	-0.008277	-0.017911	0.042563	-0.036038	-0.021957
	0.002495	-0.008412	-0.015066	-0.015484	-0.022900	0.009153
	0.029086	-0.027327	-0.062161	-0.038057	-0.036497	-0.030516
	-0.015337	-0.066326	-0.065293	0.033247	-0.015636	0.013948
	0.046390					
x54	-0.019242	-0.059429	-0.048590	-0.021159	0.004377	0.036662
	0.045187	0.056704	0.013286	0.004445	0.008930	0.011991
	-0.005712	-0.009996	0.010480	-0.038057	-0.010755	0.007323
	0.008005	-0.033612	0.028792	0.006072	-0.001033	-0.010457
	-0.017086	0.011343	0.012440	0.029249	-0.062975	0.003166
	0.045325					
y54	-0.104050	0.001241	-0.014137	-0.015145	0.048128	-0.020756
	-0.080458	-0.000015	0.010781	0.031554	0.001047	-0.065991
	0.043450	-0.030059	0.034789	-0.014075	-0.019797	0.057347
	-0.024984	-0.014794	-0.015695	-0.061972	0.044421	0.031752
	0.013600	-0.013617	-0.023219	-0.000496	-0.019512	0.006570
	0.008115					
z54	0.037885	-0.006532	-0.035328	-0.035512	-0.004319	-0.029124
	0.025234	-0.000362	-0.023173	0.039111	-0.033584	-0.024070
	0.002114	-0.003623	-0.026594	-0.018257	-0.015998	0.014680
	0.034039	-0.032766	-0.068103	-0.028368	-0.016895	-0.029604
	-0.005086	-0.063376	-0.048294	0.039187	-0.022147	0.046269
	0.048729					
x55	-0.019643	-0.058782	-0.048537	-0.018215	0.004879	0.039914
	0.042562	0.044836	0.009582	0.004989	0.015799	0.021234
	-0.008990	-0.006577	0.002707	-0.043883	-0.002499	0.007368
	0.011474	-0.027166	0.026138	0.013387	0.004027	-0.006282
	-0.005472	0.005817	0.010853	0.047704	-0.062848	0.002256
	0.066919					
y55	-0.106390	0.002590	-0.009782	-0.015406	0.034859	-0.019655
	-0.068990	-0.000208	0.018511	0.022591	-0.018674	-0.048890
	0.055560	-0.025147	0.033478	-0.019414	-0.040490	0.034676
	0.001345	-0.022188	0.007990	-0.038128	0.047658	0.032916
	0.041619	-0.017783	-0.006783	0.004780	0.002789	-0.004356
	0.024294					

z55	0.029996	-0.012144	-0.034225	-0.032194	-0.011740	-0.020822
	0.031089	0.000248	-0.018633	0.029680	-0.039338	-0.014390
	0.001098	0.002104	-0.034573	-0.010372	-0.014997	-0.000736
	0.046593	-0.040125	-0.064500	-0.009376	-0.013991	-0.018831
	0.039494	-0.071660	-0.033714	0.046803	-0.020549	0.031200
	0.079272					
x56	-0.019923	-0.059229	-0.049140	-0.015081	0.007812	0.042304
	0.034400	0.037027	0.007693	0.006287	0.023581	0.018988
	-0.013371	-0.005297	-0.005537	-0.043839	-0.004898	0.009676
	0.011991	-0.017543	0.022763	0.019933	0.008664	-0.017726
	0.001075	0.009929	0.010826	0.048090	-0.057957	-0.003800
	0.059085					
y56	-0.108412	0.000577	-0.009150	-0.017842	0.037866	-0.016165
	-0.061719	-0.001043	0.017049	0.032024	-0.019403	-0.051731
	0.056435	-0.010106	0.034432	-0.015734	-0.053555	0.027091
	0.014942	-0.010146	0.025016	-0.019931	0.058233	0.030559
	0.041883	-0.018373	-0.021974	0.014729	-0.028927	0.044346
	0.011485					
z56	0.019699	-0.019563	-0.033027	-0.032099	-0.005723	-0.013745
	0.033495	-0.001649	-0.017882	0.028937	-0.032784	-0.019798
	-0.006858	0.013078	-0.035912	-0.001335	-0.021438	-0.008534
	0.046305	-0.035438	-0.063960	0.000585	-0.007375	-0.026116
	0.061516	-0.056271	-0.034508	0.049172	-0.026658	0.027822
	0.069819					
x57	-0.018551	-0.058993	-0.047984	-0.014384	0.007352	0.041820
	0.028669	0.032494	0.003414	0.012006	0.029102	0.016660
	-0.014395	-0.003795	-0.014206	-0.051054	-0.003706	0.012087
	0.010204	-0.010038	0.016529	0.013346	0.000458	-0.021979
	0.000397	0.015296	0.018465	0.040916	-0.051055	-0.004603
	0.032340					
y57	-0.107206	-0.001829	-0.013763	-0.019802	0.033802	-0.013193
	-0.055150	0.003894	0.019744	0.037423	-0.009036	-0.050262
	0.049514	-0.010814	0.028845	-0.010626	-0.037125	0.030923
	0.025906	-0.012932	0.038940	-0.034877	0.033402	0.044625
	0.015024	-0.013275	-0.039492	-0.002513	-0.025732	0.066569
	-0.025398					
z57	0.013629	-0.026502	-0.029916	-0.034577	-0.005404	-0.011770
	0.036941	0.006837	-0.014161	0.035823	-0.027173	-0.023990
	-0.011655	0.016285	-0.041074	-0.005271	-0.017542	-0.008278
	0.043754	-0.028672	-0.059032	-0.008164	-0.026471	-0.024618
	0.055149	-0.034731	-0.020972	0.032822	-0.028604	0.017101
	0.032750					

x58	-0.016373	-0.059311	-0.047271	-0.012728	0.004971	0.039521
	0.022670	0.028819	-0.000486	0.014048	0.033056	0.016624
	-0.014903	-0.003845	-0.021488	-0.050660	-0.002950	0.015708
	0.006386	-0.009389	0.011006	0.017231	0.006072	-0.024544
	-0.004489	0.020492	0.022713	0.043145	-0.043093	-0.005662
	0.025874					
y58	-0.107040	-0.001504	-0.017838	-0.020739	0.026129	-0.015493
	-0.046306	0.005634	0.021111	0.030363	-0.015980	-0.033497
	0.043584	-0.009982	0.029176	-0.003711	-0.030970	0.033772
	0.023588	-0.031148	0.052575	-0.029562	0.040945	0.049395
	-0.003189	-0.013239	-0.031233	-0.023531	-0.015412	0.045167
	-0.028278					
z58	0.008175	-0.033934	-0.027620	-0.035076	-0.009379	-0.012609
	0.041806	0.011243	-0.009254	0.034647	-0.026575	-0.019377
	-0.016564	0.021368	-0.042769	0.004823	-0.016617	-0.013511
	0.033622	-0.038574	-0.056054	0.003939	-0.013876	-0.019309
	0.050463	-0.018452	-0.006725	0.033782	-0.032419	0.016886
	0.022196					
x59	-0.015007	-0.058095	-0.046800	-0.009856	0.004198	0.040534
	0.015050	0.024318	-0.008258	0.016141	0.035899	0.010198
	-0.010643	-0.001495	-0.025801	-0.048719	-0.003570	0.023666
	0.009242	-0.003910	0.015410	0.021845	0.002770	-0.026068
	-0.008787	0.023563	0.021377	0.043770	-0.035593	0.003167
	0.020845					
y59	-0.109064	-0.001440	-0.019162	-0.019338	0.018917	-0.011515
	-0.040262	0.008077	0.024153	0.018756	-0.010896	-0.023951
	0.042741	-0.011775	0.026900	-0.006259	-0.017783	0.010399
	0.034037	-0.028704	0.063045	-0.016466	0.022615	0.030152
	-0.008300	-0.009991	-0.024452	-0.019307	-0.001788	0.053383
	-0.001694					
z59	-0.000428	-0.037375	-0.025042	-0.032667	-0.008257	-0.004604
	0.041851	0.014401	-0.005840	0.029385	-0.019794	-0.029203
	-0.014110	0.023562	-0.033955	0.010557	-0.013429	-0.026330
	0.038584	-0.028137	-0.035868	0.012306	-0.027670	-0.018694
	0.054108	-0.010804	0.010431	0.036936	-0.023136	0.030283
	0.043608					
x60	-0.012438	-0.055638	-0.046168	-0.007095	0.002587	0.040697
	0.007940	0.019113	-0.013100	0.018730	0.037466	0.008829
	-0.005187	0.001313	-0.028000	-0.045037	0.000563	0.027530
	0.006978	-0.000788	0.014011	0.026672	-0.002537	-0.028909
	-0.003613	0.018392	0.013336	0.039873	-0.027955	-0.005273
	0.030056					

y60	-0.106495	-0.001312	-0.021219	-0.015859	0.012039	-0.012622
	-0.035954	0.007261	0.028651	0.013407	-0.008416	-0.012616
	0.042300	-0.006009	0.021125	-0.006529	-0.001009	-0.003772
	0.028430	-0.027905	0.063860	-0.001587	-0.000421	0.009729
	-0.014133	-0.008931	-0.045861	-0.025119	0.018516	0.028019
	0.038340					
z60	-0.006036	-0.040769	-0.023202	-0.029231	-0.007775	-0.000678
	0.040314	0.010180	0.000750	0.029488	-0.014472	-0.030514
	-0.013597	0.029253	-0.024133	0.020864	-0.002540	-0.036672
	0.028451	-0.021103	-0.025170	0.027205	-0.044662	-0.020163
	0.063123	-0.012339	0.000135	0.022477	-0.004060	0.015861
	0.084872					
x61	-0.010329	-0.053737	-0.045061	-0.006140	0.001940	0.038575
	-0.000467	0.014499	-0.019436	0.020692	0.038935	0.007911
	-0.000585	-0.000039	-0.028863	-0.043618	0.001490	0.027295
	0.004395	-0.001866	0.009516	0.028046	-0.005977	-0.031387
	0.000848	0.019620	0.014623	0.040749	-0.015515	-0.004292
	0.024117					
y61	-0.103908	-0.001956	-0.020867	-0.016661	0.008480	-0.016122
	-0.029939	0.001664	0.020303	0.011153	-0.023239	0.003878
	0.048350	0.004330	0.010835	-0.004829	0.000001	0.000264
	0.023714	-0.036611	0.062736	0.004732	0.002352	0.007426
	-0.018421	0.017636	-0.029783	-0.033793	0.027872	0.001400
	0.004730					
z61	-0.011467	-0.046072	-0.021895	-0.031042	-0.006108	-0.000231
	0.036401	0.007822	-0.001839	0.027241	-0.017768	-0.023152
	-0.013013	0.028653	-0.016377	0.033827	-0.003568	-0.041641
	0.020356	-0.037285	-0.018703	0.031713	-0.035771	-0.013307
	0.062879	0.010591	0.020136	0.010673	0.019212	0.012807
	0.053930					
x62	-0.007115	-0.052374	-0.044410	-0.003573	0.000629	0.036671
	-0.009188	0.009056	-0.026663	0.023925	0.038386	0.005151
	0.002935	0.002001	-0.029202	-0.036705	-0.000145	0.034226
	0.002723	-0.002563	0.008827	0.029912	-0.008051	-0.029990
	0.003407	0.019584	0.008022	0.042685	-0.008633	-0.003658
	0.017932					
y62	-0.102758	-0.001610	-0.019539	-0.022967	0.002204	-0.017592
	-0.017220	0.001153	0.018256	0.006755	-0.028109	0.019004
	0.041787	0.005314	-0.001984	-0.007898	0.006364	-0.009588
	0.008859	-0.017315	0.040091	0.013575	0.005379	0.019718
	-0.017082	0.028968	0.008207	-0.016230	0.034747	-0.013550
	0.037314					

z62	-0.015932	-0.050919	-0.019865	-0.031669	-0.005942	0.003033
	0.036206	0.006023	-0.000735	0.025841	-0.015627	-0.017002
	-0.023434	0.025173	-0.016205	0.044687	-0.004170	-0.048398
	0.007073	-0.022456	-0.022500	0.040072	-0.029002	0.006054
	0.068121	0.029053	0.040508	0.031077	0.024434	0.013139
	0.065342					
x63	-0.004801	-0.049867	-0.043142	-0.000545	-0.001074	0.034934
	-0.017540	0.004928	-0.030327	0.024991	0.038975	0.001310
	0.008940	0.005537	-0.025232	-0.028628	-0.000836	0.033397
	0.004848	-0.007989	0.008948	0.031138	-0.011109	-0.033678
	0.005401	0.017707	0.002940	0.046118	0.000525	0.000817
	0.021880					
y63	-0.101575	-0.001139	-0.022833	-0.022540	0.000642	-0.018486
	-0.003221	-0.000597	0.016876	0.002783	-0.035216	0.026626
	0.038929	0.009545	-0.024423	-0.004236	0.013129	-0.014768
	0.015577	-0.000575	0.018059	0.025822	0.001713	0.017881
	-0.031417	0.031052	0.010318	-0.037955	-0.008173	-0.007406
	0.051120					
z63	-0.023030	-0.053673	-0.020338	-0.028814	-0.001243	0.008998
	0.036625	0.005538	0.001295	0.023371	-0.015077	-0.016717
	-0.025248	0.026990	-0.017231	0.058585	-0.004573	-0.055943
	0.016155	-0.015618	-0.025212	0.045340	-0.027719	0.009686
	0.060772	0.029949	0.040223	0.013824	0.004265	0.029948
	0.077700					
x64	-0.002196	-0.046967	-0.042318	0.001087	-0.001483	0.032437
	-0.025853	0.000886	-0.035487	0.027242	0.041432	-0.001911
	0.011958	0.005818	-0.023726	-0.028250	0.002440	0.032507
	0.006130	-0.007069	0.005480	0.028857	-0.014056	-0.034075
	0.007678	0.016001	-0.000897	0.047297	0.001508	0.005181
	0.016119					
y64	-0.098056	-0.002199	-0.021502	-0.018251	-0.007339	-0.021678
	0.007427	-0.004172	0.020168	-0.003695	-0.046322	0.033542
	0.037402	0.012809	-0.028346	0.002522	0.002284	-0.016372
	0.020287	-0.005201	0.015740	0.032349	-0.014610	-0.004357
	-0.037118	0.025783	0.004683	-0.042956	-0.001730	-0.029348
	0.052013					
z64	-0.027584	-0.057281	-0.019244	-0.023050	-0.000705	0.012246
	0.032062	0.002296	0.006385	0.016220	-0.016908	-0.013869
	-0.033435	0.018656	-0.004432	0.064250	-0.017547	-0.061794
	0.020351	-0.029688	-0.012096	0.037872	-0.039026	-0.008388
	0.062847	0.026040	0.040324	-0.001011	0.027565	0.006465
	0.054626					

x65	-0.000123	-0.043110	-0.040812	0.004050	-0.002260	0.030857
	-0.034523	-0.005459	-0.038336	0.024171	0.040193	-0.005704
	0.015218	0.006239	-0.022270	-0.028835	0.002963	0.032523
	0.012300	-0.007378	0.004810	0.030294	-0.016121	-0.040050
	0.009524	0.013848	0.000907	0.044163	0.004408	0.005981
	0.005694					
y65	-0.093785	-0.004574	-0.020309	-0.020709	-0.011740	-0.025797
	0.019414	0.000236	0.011788	0.008110	-0.040790	0.030259
	0.035535	0.008100	-0.032126	0.013910	0.004444	-0.020162
	0.000650	0.006545	-0.004490	0.026056	-0.022695	-0.010092
	-0.038111	0.030776	-0.019633	-0.020795	0.011967	-0.023580
	0.069517					
z65	-0.029348	-0.060812	-0.016401	-0.021277	0.001603	0.016680
	0.026391	0.001899	0.004826	0.014956	-0.008179	-0.025630
	-0.043181	0.007782	0.002984	0.069736	-0.015680	-0.063980
	0.010627	-0.039732	-0.004741	0.023138	-0.028384	-0.021118
	0.059568	0.048436	0.038180	0.005340	0.076789	0.006197
	0.018064					
x66	0.001525	-0.039782	-0.038713	0.007295	-0.002319	0.029110
	-0.042721	-0.009777	-0.039945	0.021774	0.040777	-0.014137
	0.017878	0.007144	-0.019329	-0.028759	0.003358	0.032158
	0.019017	-0.006014	0.005227	0.032042	-0.017692	-0.041731
	0.013252	0.011843	-0.000277	0.046665	0.018624	0.001728
	-0.001279					
y66	-0.087953	-0.006049	-0.021345	-0.023394	-0.015447	-0.028015
	0.023466	0.001869	0.002021	0.011081	-0.043214	0.038268
	0.036327	0.003021	-0.042162	0.025276	0.006634	-0.021606
	-0.011658	0.012946	-0.005016	0.014307	-0.037974	-0.014431
	-0.043196	0.019749	-0.014271	-0.035221	0.015341	-0.019193
	0.084224					
z66	-0.033672	-0.060116	-0.009732	-0.022554	0.007496	0.023993
	0.019828	0.001207	0.002371	0.007730	-0.005045	-0.032613
	-0.047207	-0.006129	-0.001577	0.071341	0.008925	-0.050235
	-0.001651	-0.029275	-0.001754	0.007076	-0.021826	-0.036174
	0.052756	0.054954	0.038624	-0.013606	0.089336	-0.025562
	0.013573					
x67	0.003099	-0.036588	-0.035456	0.010174	-0.003000	0.026330
	-0.050322	-0.013246	-0.044600	0.020212	0.040383	-0.020530
	0.019043	0.006649	-0.018639	-0.025221	0.005768	0.030367
	0.019636	-0.003340	0.001388	0.029506	-0.017885	-0.041284
	0.014850	0.007926	-0.001627	0.046783	0.033882	-0.003670
	-0.003674					

y67	-0.079733	-0.006069	-0.026354	-0.027425	-0.018590	-0.028451
	0.028330	0.001012	0.001533	0.017524	-0.039858	0.046078
	0.041257	0.003175	-0.050091	0.017927	0.013038	-0.010239
	-0.002208	0.021854	-0.003947	0.013155	-0.045958	-0.012948
	-0.050702	0.019014	-0.013892	-0.043798	-0.016895	0.011139
	0.083169					
z67	-0.035947	-0.058133	-0.006961	-0.025471	0.011347	0.028945
	0.015877	0.000479	0.007676	0.009798	0.002923	-0.033314
	-0.040222	-0.010432	-0.000883	0.064463	0.026485	-0.039205
	-0.000680	-0.003512	-0.009708	0.003210	-0.022672	-0.031929
	0.044062	0.052953	0.030540	-0.008770	0.061585	-0.011635
	0.014473					
x68	0.004445	-0.032813	-0.032891	0.011627	-0.004409	0.023887
	-0.057064	-0.017119	-0.049101	0.018618	0.037717	-0.025097
	0.021081	0.004985	-0.021248	-0.022734	0.014036	0.028858
	0.020339	0.004055	-0.004603	0.024792	-0.016081	-0.043459
	0.017245	-0.003327	-0.000330	0.045793	0.041103	-0.013094
	-0.006903					
y68	-0.072285	-0.007437	-0.028543	-0.027050	-0.021624	-0.029093
	0.032978	0.004211	0.000930	0.021680	-0.035164	0.051760
	0.042786	0.006088	-0.048986	0.021170	0.009297	-0.004486
	0.003270	0.014502	0.008101	0.022335	-0.057035	-0.004547
	-0.052775	0.029874	-0.021097	-0.052939	-0.027977	0.044455
	0.084283					
z68	-0.037290	-0.058109	-0.002634	-0.025001	0.015319	0.031990
	0.013832	0.004815	0.012965	0.008124	0.010828	-0.034805
	-0.039355	-0.010849	0.001553	0.055329	0.029122	-0.030236
	0.003513	0.004089	-0.014322	0.005692	-0.022005	-0.020959
	0.032111	0.079935	0.016411	-0.008310	0.047044	0.009523
	0.018353					
x69	0.004977	-0.029229	-0.031921	0.014568	-0.006144	0.022129
	-0.062995	-0.016707	-0.052154	0.016226	0.035603	-0.027919
	0.023073	0.002963	-0.024908	-0.017908	0.021877	0.031403
	0.025019	0.006238	-0.011100	0.025877	-0.019955	-0.038615
	0.015648	-0.009891	-0.004543	0.047835	0.039564	-0.002626
	0.016878					
y69	-0.063828	-0.008748	-0.024553	-0.027345	-0.024544	-0.033382
	0.036449	-0.001009	-0.005219	0.027711	-0.033044	0.053802
	0.043959	0.011584	-0.038733	0.018873	0.000332	-0.005857
	-0.000003	0.003150	0.020269	0.019365	-0.054813	-0.009279
	-0.044492	0.026140	-0.011642	-0.063503	-0.021908	0.034226
	0.033429					

z69	-0.036098	-0.058885	0.003846	-0.027923	0.018479	0.032524
	0.011397	0.001450	0.017903	0.006338	0.016251	-0.035795
	-0.038592	-0.010737	0.003628	0.047170	0.027115	-0.032177
	-0.002973	0.018385	-0.019383	-0.006671	-0.007182	-0.030314
	0.017315	0.087802	0.003589	-0.013496	0.049246	-0.007072
	-0.028863					
x70	0.007586	-0.025083	-0.030071	0.015924	-0.006234	0.018927
	-0.071053	-0.016948	-0.053236	0.015019	0.035042	-0.029762
	0.023864	-0.005715	-0.025939	-0.017122	0.028155	0.038766
	0.026988	0.018630	-0.022489	0.023565	-0.028476	-0.043258
	0.016614	-0.005123	-0.000650	0.034921	0.042658	-0.002855
	0.014054					
y70	-0.059585	-0.009549	-0.022628	-0.025672	-0.029602	-0.033510
	0.042380	-0.003766	-0.010706	0.028390	-0.033538	0.053313
	0.049177	0.026057	-0.032564	0.019756	-0.006817	-0.020140
	0.003179	-0.012426	0.038834	0.021178	-0.037759	0.005083
	-0.038336	0.005915	-0.008991	-0.052900	-0.020231	0.036675
	0.020774					
z70	-0.037173	-0.059089	0.007971	-0.028834	0.017944	0.035146
	0.014405	-0.001130	0.016155	0.002867	0.017701	-0.032182
	-0.031701	-0.000459	0.002614	0.046188	0.031295	-0.038043
	-0.007126	0.013636	-0.016328	-0.014111	0.003874	-0.020428
	-0.000037	0.068908	-0.011636	-0.000655	0.044980	-0.003659
	-0.036208					
x71	0.011106	-0.022238	-0.029420	0.014288	-0.006657	0.014129
	-0.078438	-0.019563	-0.052120	0.016915	0.029779	-0.028744
	0.026739	-0.007701	-0.030985	-0.017535	0.035921	0.033595
	0.023386	0.047589	-0.020828	0.020912	-0.035217	-0.028777
	0.027238	-0.009963	-0.002489	0.031423	0.039743	0.001535
	0.010274					
y71	-0.055722	-0.008150	-0.019414	-0.020496	-0.034219	-0.032588
	0.047392	-0.000790	-0.014337	0.025024	-0.028331	0.051632
	0.046469	0.030501	-0.031209	0.025363	-0.008935	-0.016621
	0.011639	-0.035059	0.040325	0.025174	-0.034350	0.006993
	-0.039261	0.003833	0.005728	-0.058021	-0.023732	0.041143
	0.019066					
z71	-0.038299	-0.058533	0.012691	-0.025580	0.017249	0.039241
	0.018073	0.001753	0.012603	-0.003847	0.019897	-0.031274
	-0.028061	0.006987	0.005378	0.038690	0.030073	-0.027166
	-0.001624	-0.012623	-0.033189	-0.013080	0.017875	-0.024737
	-0.025115	0.074509	-0.019458	0.003322	0.038183	-0.004000
	-0.032183					

x72	0.012818	-0.019715	-0.028016	0.018187	-0.009053	0.013379
	-0.083059	-0.018822	-0.052037	0.013966	0.033603	-0.030823
	0.032309	-0.008623	-0.028501	-0.009959	0.042037	0.034734
	0.032359	0.052399	-0.021482	0.029080	-0.023327	-0.029893
	0.030756	0.003815	-0.005543	0.033217	0.032980	0.002920
	0.004999					
y72	-0.051764	-0.006224	-0.016794	-0.021474	-0.035706	-0.033422
	0.052079	0.001831	-0.019571	0.026626	-0.036727	0.053090
	0.036232	0.026410	-0.037377	0.015372	-0.016227	-0.011405
	0.006008	-0.028847	0.041354	0.011006	-0.039304	0.018982
	-0.028860	-0.020329	0.018099	-0.075064	-0.015507	0.035179
	0.017941					
z72	-0.036444	-0.056572	0.018448	-0.028935	0.019135	0.035554
	0.018645	-0.001103	0.007629	-0.006347	0.013027	-0.026778
	-0.021374	0.016685	0.004090	0.032346	0.032931	-0.027243
	-0.011059	-0.022088	-0.041717	-0.019318	0.007288	-0.015897
	-0.041042	0.056328	-0.018963	0.000064	0.032460	0.003723
	-0.031121					
x73	0.014321	-0.014318	-0.027032	0.016972	-0.009335	0.008785
	-0.084049	-0.020355	-0.057978	0.018140	0.024685	-0.020147
	0.029090	-0.013172	-0.023127	-0.003409	0.042099	0.032499
	0.027401	0.059699	-0.020899	0.025363	-0.023041	-0.019963
	0.029852	-0.013814	0.007117	0.014209	0.034323	0.005541
	-0.004290					
y73	-0.047087	-0.008073	-0.015240	-0.018744	-0.042566	-0.031434
	0.053237	0.008651	-0.018739	0.022549	-0.031328	0.041938
	0.036283	0.029614	-0.044396	0.009436	-0.009719	-0.005986
	0.014868	-0.029930	0.039436	0.011760	-0.034994	0.015947
	-0.016683	-0.009427	0.001830	-0.066102	-0.019381	0.041156
	0.016808					
z73	-0.034795	-0.059772	0.025539	-0.024800	0.021684	0.036177
	0.017201	0.002532	0.011189	-0.021122	0.018962	-0.029306
	-0.011581	0.031432	-0.009763	0.025586	0.023624	-0.022760
	-0.002633	-0.022384	-0.052903	-0.028587	0.029217	-0.019990
	-0.042558	0.095178	-0.037339	0.022025	0.010341	-0.025533
	-0.025750					
x74	0.013757	-0.007949	-0.022719	0.013557	-0.009834	0.003375
	-0.088044	-0.019623	-0.062670	0.015454	0.014992	-0.013536
	0.028188	-0.007169	-0.029436	-0.009799	0.036252	0.031662
	0.025415	0.062002	-0.024037	0.027915	-0.016646	-0.009922
	0.037800	-0.006125	0.023246	0.023195	0.023755	-0.025747
	-0.013728					

y74	-0.039169	-0.008867	-0.014998	-0.014350	-0.047231	-0.031054
	0.051391	0.011313	-0.014676	0.019279	-0.032766	0.025480
	0.037975	0.035396	-0.018883	0.016069	0.000413	-0.005567
	0.016809	-0.043454	0.052989	0.010972	-0.034330	0.017808
	-0.013842	-0.022086	-0.022042	-0.082687	0.011491	0.088501
	-0.012980					
z74	-0.031282	-0.061404	0.029734	-0.021483	0.023054	0.035995
	0.024504	-0.001552	0.008570	-0.021830	0.030084	-0.029369
	0.002734	0.027901	-0.010373	0.030024	0.031142	-0.021725
	-0.004928	-0.020805	-0.060259	-0.037154	0.023545	-0.015536
	-0.058914	0.087396	-0.041327	0.019991	0.000445	-0.004194
	-0.010992					
x75	0.013913	-0.007298	-0.024055	0.014685	-0.015782	0.000169
	-0.091346	-0.022082	-0.061088	0.019458	0.012458	-0.011887
	0.026776	-0.002439	-0.030493	-0.004480	0.043341	0.020756
	0.023863	0.063136	-0.024473	0.035333	-0.019791	-0.003453
	0.045278	0.005168	0.027608	0.012109	0.009816	-0.025014
	-0.013594					
y75	-0.032569	-0.002261	-0.011906	-0.011770	-0.048461	-0.029198
	0.055979	0.015180	-0.016326	0.009514	-0.035170	0.019933
	0.039445	0.026389	-0.012265	0.026023	0.002312	0.002115
	0.022017	-0.046955	0.053004	-0.010319	-0.025890	0.039321
	-0.013894	-0.025055	-0.030077	-0.066737	0.037818	0.099553
	-0.043197					
z75	-0.027507	-0.061239	0.038197	-0.024855	0.028596	0.035780
	0.027991	0.002126	0.002028	-0.025574	0.030846	-0.032746
	0.013343	0.027859	-0.016217	0.015073	0.027101	-0.007014
	-0.010447	-0.022347	-0.065598	-0.034536	0.018859	-0.029891
	-0.057527	0.054390	-0.046719	0.011558	-0.014517	0.002351
	-0.015408					
x76	0.014068	-0.001766	-0.026490	0.012236	-0.019187	-0.002361
	-0.085072	-0.022869	-0.061701	0.020115	0.003432	-0.008877
	0.028926	-0.009852	-0.031366	0.008060	0.046367	0.012165
	0.023595	0.065531	-0.035620	0.032987	-0.004632	0.023307
	0.036518	0.008778	0.024503	-0.010121	-0.006906	-0.011859
	-0.012158					
y76	-0.027727	0.000610	-0.008671	-0.002478	-0.048534	-0.026543
	0.054755	0.013871	-0.017947	0.008202	-0.024835	0.013221
	0.041170	0.019764	-0.013771	0.013777	0.011372	0.004027
	0.017742	-0.038667	0.052970	-0.023385	-0.033178	0.032659
	-0.002468	-0.017000	-0.025270	-0.018094	0.058341	0.088294
	-0.047559					

z76	-0.023591	-0.063482	0.046382	-0.025641	0.034590	0.034871
	0.021641	0.006055	-0.002403	-0.035335	0.027007	-0.039781
	0.014632	0.036143	-0.012047	0.010575	0.019928	0.013727
	-0.010238	-0.022989	-0.051646	-0.030431	0.005156	-0.040298
	-0.049154	0.028297	-0.044655	0.000063	-0.005704	-0.010077
	-0.019103					
x77	0.014018	0.002815	-0.028799	0.013508	-0.019149	-0.004559
	-0.075089	-0.019482	-0.060090	0.015580	-0.000842	-0.001163
	0.028862	-0.003527	-0.022390	0.029141	0.040010	0.013034
	0.008335	0.067410	-0.027206	0.011694	0.011764	0.031222
	0.012871	0.005189	0.035132	-0.015672	-0.006631	-0.018056
	-0.023329					
y77	-0.022493	0.002578	-0.004194	0.001389	-0.050190	-0.024658
	0.049600	0.011515	-0.022184	0.006485	-0.016468	0.002455
	0.042065	0.003398	-0.023056	-0.011112	0.025997	0.001072
	0.027048	-0.022628	0.029991	-0.020501	-0.034106	0.034607
	0.021977	-0.008835	-0.043465	0.012340	0.065486	0.090316
	-0.042979					
z77	-0.018032	-0.063613	0.053093	-0.029716	0.037101	0.032056
	0.017213	0.007341	-0.007482	-0.040358	0.018373	-0.047573
	0.017070	0.036019	-0.006880	0.013063	0.015703	0.028616
	-0.003877	-0.020631	-0.045999	-0.025243	-0.004426	-0.024414
	-0.041542	0.007315	-0.037850	-0.019805	-0.013922	-0.010988
	-0.006368					
x78	0.016004	0.004648	-0.034075	0.017562	-0.023507	-0.008372
	-0.071769	-0.029041	-0.052674	0.017761	-0.009576	-0.002468
	0.034363	0.005129	-0.019156	0.036381	0.037688	0.012119
	0.004359	0.058274	-0.005587	-0.001115	0.003344	0.024851
	0.010269	-0.001210	0.037787	0.002072	0.001603	0.002280
	-0.011071					
y78	-0.018587	0.008815	0.003383	-0.000249	-0.044198	-0.022692
	0.052935	0.017234	-0.029723	0.000561	-0.006397	-0.004293
	0.036100	-0.012972	-0.021184	-0.023621	0.037889	0.000884
	0.025133	-0.001099	-0.003317	-0.025135	-0.019499	0.049546
	0.021898	0.009258	-0.052486	0.018914	0.060327	0.076009
	-0.041098					
z78	-0.013005	-0.062529	0.060774	-0.032496	0.039052	0.029236
	0.013143	0.013984	-0.015338	-0.044984	0.018997	-0.047171
	0.022017	0.037350	0.000544	0.011364	0.020454	0.037598
	-0.003684	-0.024340	-0.043473	-0.018688	-0.009175	-0.017305
	-0.038709	-0.008514	-0.042381	-0.029796	-0.021972	-0.008924
	-0.013401					

x79	0.014112	0.006676	-0.036296	0.018346	-0.021567	-0.015697
	-0.072916	-0.035871	-0.041396	0.015143	-0.012537	-0.014600
	0.038022	0.002469	-0.017322	0.046776	0.039156	-0.007265
	-0.001355	0.055603	-0.003506	-0.005753	-0.013797	0.025068
	0.002282	0.015067	0.038711	-0.003155	0.000361	-0.006963
	-0.017525					
y79	-0.011224	0.012220	0.009935	0.000965	-0.042848	-0.018112
	0.058629	0.021592	-0.037676	-0.003793	-0.008968	-0.003621
	0.034681	-0.015336	-0.024012	-0.035694	0.044216	0.012223
	0.027136	0.009906	-0.020382	-0.032263	-0.005387	0.055929
	0.020893	0.005833	-0.047908	0.030757	0.058315	0.069202
	-0.039923					
z79	-0.007709	-0.060515	0.066166	-0.032274	0.036527	0.027289
	0.011532	0.019486	-0.025030	-0.048694	0.015823	-0.042468
	0.029010	0.039982	0.007893	0.010647	0.022650	0.049861
	-0.003297	-0.031051	-0.029489	-0.011841	-0.009117	-0.005064
	-0.024795	-0.030126	-0.045763	-0.035994	-0.033684	0.002021
	-0.014459					
x80	0.011038	0.009120	-0.043138	0.024627	-0.020836	-0.016942
	-0.063276	-0.045145	-0.033543	0.008425	-0.018555	-0.011820
	0.037253	0.004680	-0.018378	0.053703	0.046016	-0.023257
	-0.001775	0.045101	0.004056	-0.014617	-0.035909	0.027097
	0.012054	0.003855	0.009309	-0.017140	-0.026062	-0.047800
	0.003664					
y80	-0.003114	0.015342	0.019688	-0.002130	-0.040133	-0.014595
	0.058213	0.025654	-0.040958	-0.008076	-0.009052	-0.008997
	0.035476	-0.021755	-0.028394	-0.048096	0.041715	0.020229
	0.024697	0.026431	-0.037088	-0.036472	0.015643	0.055976
	0.006101	0.018777	-0.028059	0.046086	0.068126	0.076486
	-0.051464					
z80	-0.003315	-0.055532	0.070923	-0.032540	0.031880	0.023577
	0.007577	0.023933	-0.030401	-0.049018	0.014443	-0.037826
	0.036933	0.040612	0.018037	0.011363	0.028696	0.055593
	-0.005651	-0.024891	-0.016190	-0.001826	-0.011565	0.011652
	-0.011211	-0.039989	-0.030629	-0.036856	-0.043865	0.031902
	-0.015458					
x81	0.007288	0.011342	-0.050517	0.028664	-0.022377	-0.017260
	-0.049435	-0.042133	-0.027833	0.004302	-0.016893	-0.004350
	0.048003	0.010325	-0.016219	0.067665	0.057961	-0.048933
	-0.013765	0.046981	0.001455	-0.005579	-0.048874	0.017443
	-0.008865	-0.003063	0.004156	-0.034422	-0.032648	-0.028219
	-0.009789					

y81	0.003279	0.019152	0.031391	-0.002708	-0.033225	-0.005317
	0.054406	0.021529	-0.040310	-0.017860	-0.016631	-0.027100
	0.022433	-0.037351	-0.033342	-0.054185	0.025949	0.038987
	0.028133	0.037923	-0.038582	-0.050554	0.032663	0.070220
	0.013103	0.020177	0.002811	0.046297	0.066694	0.035329
	-0.024835					
z81	-0.001754	-0.047936	0.074172	-0.027019	0.027866	0.023529
	0.002820	0.025105	-0.033083	-0.051617	0.012440	-0.031537
	0.040748	0.037721	0.026439	0.014703	0.037276	0.055430
	-0.005379	-0.012255	0.008864	0.004867	-0.021540	0.025405
	0.006896	-0.047450	-0.016482	-0.039036	-0.043142	0.044337
	-0.005432					
x82	0.004191	0.015761	-0.054725	0.031328	-0.024682	-0.013606
	-0.032843	-0.033990	-0.021229	-0.005089	-0.015154	-0.002308
	0.047696	0.006780	-0.016332	0.077773	0.066295	-0.079290
	-0.037806	0.039406	-0.000375	-0.000060	-0.049304	0.016474
	-0.011674	0.014617	0.017257	-0.061768	-0.034491	0.010188
	-0.044363					
y82	0.010056	0.021103	0.039651	-0.002048	-0.026544	0.004686
	0.048941	0.009999	-0.037239	-0.021429	-0.024641	-0.035057
	0.017867	-0.040272	-0.039484	-0.052673	0.016871	0.054853
	0.037464	0.048418	-0.036078	-0.062731	0.034688	0.082542
	0.007843	0.001680	0.019265	0.040892	0.053267	-0.018289
	0.010245					
z82	-0.003990	-0.034580	0.072211	-0.017589	0.020210	0.024707
	0.001452	0.021367	-0.034148	-0.049826	0.009235	-0.013111
	0.045644	0.040571	0.037658	0.023322	0.064211	0.043176
	-0.006138	0.013143	0.034748	0.011786	-0.049575	0.042556
	0.025192	-0.048809	-0.013059	-0.043578	-0.050348	0.061717
	0.004454					
x83	0.002952	0.022854	-0.059426	0.031430	-0.017960	-0.008463
	-0.019286	-0.027084	-0.007996	-0.009900	-0.024788	-0.005144
	0.045323	0.016032	-0.019532	0.084160	0.051310	-0.085754
	-0.056085	0.046639	0.007100	0.007216	-0.065545	0.005161
	-0.025773	0.006991	0.015678	-0.073851	-0.038546	0.023145
	-0.043791					
y83	0.016589	0.019315	0.048503	-0.003008	-0.027677	0.013066
	0.051468	-0.000076	-0.037409	-0.029677	-0.028376	-0.037053
	0.017235	-0.051286	-0.045323	-0.046112	0.027927	0.050791
	0.047842	0.044722	-0.035103	-0.072035	0.043417	0.099312
	0.006473	-0.006887	0.042797	-0.000410	0.033065	-0.043252
	0.014238					

z83	-0.007060	-0.020765	0.065808	-0.006925	0.006638	0.021299
	0.005274	0.019828	-0.040834	-0.042268	0.006951	0.012519
	0.059829	0.042725	0.058802	0.034627	0.106011	0.021076
	-0.012451	0.047621	0.060293	0.013834	-0.074872	0.061510
	0.038740	-0.038453	-0.001504	-0.038991	-0.067238	0.095872
	0.012275					
x84	-0.036501	0.021051	0.040679	0.000576	0.017510	0.015517
	0.059663	-0.005373	-0.005500	-0.026269	-0.054146	-0.008322
	-0.056291	-0.080799	-0.020667	0.023603	-0.078159	0.014469
	-0.017042	-0.007761	-0.082745	-0.001476	-0.019290	0.005112
	-0.013220	0.063587	-0.101936	-0.105148	0.000934	0.000813
	-0.018938					
y84	0.011303	-0.021195	0.054882	0.022605	-0.022358	-0.013977
	-0.039340	0.028706	-0.000373	0.022187	-0.032424	0.000897
	-0.038087	-0.012816	0.010435	-0.025951	0.018875	-0.013650
	-0.008374	-0.020438	0.018535	0.023711	0.034718	0.004851
	0.071605	-0.052458	-0.012473	-0.005794	0.072986	-0.036444
	-0.001078					
z84	0.008076	0.055595	-0.071696	0.031066	0.097817	-0.022656
	0.002410	-0.004380	-0.057594	0.009941	0.041573	0.039644
	-0.036612	0.027067	-0.006317	0.090759	-0.099987	-0.010205
	0.008784	-0.068527	-0.038122	0.045635	-0.018229	0.046029
	-0.011444	0.052658	-0.011278	-0.018898	0.044244	0.015025
	-0.023287					
x85	-0.032673	0.016488	0.040732	-0.005239	0.014223	0.018089
	0.053318	-0.012442	-0.007537	-0.011456	-0.053300	-0.001474
	-0.037942	-0.066054	-0.034319	0.017485	-0.069110	0.037323
	-0.010810	-0.006740	-0.054243	-0.017809	-0.003799	-0.005613
	-0.013075	0.049107	-0.092991	-0.066775	-0.000537	-0.007635
	0.002885					
y85	0.011932	-0.022832	0.058674	0.028939	-0.009582	-0.015173
	-0.036120	0.033190	-0.002866	0.020021	-0.020667	-0.005252
	-0.041746	-0.012791	0.011621	0.004651	0.007490	-0.024252
	0.006873	-0.029998	0.009371	0.036230	0.032212	0.022288
	0.068941	-0.031732	-0.022554	-0.020118	0.081500	-0.003829
	-0.009527					
z85	0.011732	0.051821	-0.064446	0.021666	0.091501	-0.022949
	0.008052	-0.001638	-0.050074	0.005596	0.031369	0.034069
	-0.029458	0.027844	0.000882	0.073913	-0.084174	-0.009409
	0.001648	-0.058941	-0.037874	0.039088	-0.015424	0.045073
	-0.018883	0.045617	-0.010369	0.007563	0.023759	0.001054
	-0.014275					

x86	-0.029551	0.011438	0.041802	-0.010153	0.011580	0.019144
	0.048126	-0.014811	-0.006274	-0.003611	-0.047484	0.003420
	-0.020438	-0.043674	-0.043055	0.010562	-0.067669	0.058987
	-0.009487	-0.015045	-0.034798	-0.027664	0.003531	-0.004792
	-0.018249	0.045986	-0.088865	-0.034750	0.005073	-0.002087
	-0.000190					
y86	0.013952	-0.023443	0.061351	0.031330	0.001806	-0.015561
	-0.033293	0.033539	-0.007551	0.028403	-0.016301	-0.013807
	-0.045440	-0.016116	0.010126	0.036034	0.003465	-0.026280
	0.024940	-0.015449	0.002204	0.038030	0.028551	0.020390
	0.061187	-0.023976	-0.023513	-0.038511	0.076219	-0.000151
	0.002719					
z86	0.014818	0.050363	-0.058186	0.011450	0.078860	-0.020597
	0.013069	-0.003894	-0.042446	0.003489	0.021726	0.031565
	-0.027034	0.029679	0.005901	0.058327	-0.065028	-0.005303
	-0.004918	-0.047149	-0.033397	0.023862	-0.012047	0.042169
	-0.018970	0.036838	-0.023389	0.028297	0.013081	-0.001797
	-0.004927					
x87	-0.028788	0.007590	0.041890	-0.012681	0.006951	0.022033
	0.043571	-0.016106	-0.004763	0.001818	-0.049400	0.009882
	-0.015842	-0.023429	-0.050121	0.013400	-0.068086	0.070251
	-0.003516	-0.023727	-0.013957	-0.036575	0.013967	-0.003329
	-0.013250	0.032442	-0.094454	-0.020915	0.023340	-0.010926
	-0.009935					
y87	0.018596	-0.021672	0.066033	0.029446	0.014726	-0.018268
	-0.028692	0.033417	-0.012134	0.034528	-0.003375	-0.021374
	-0.034355	-0.017476	0.010233	0.042962	0.000977	-0.015636
	0.029131	-0.005643	-0.002525	0.044709	0.014426	0.011963
	0.036515	-0.010248	-0.004652	-0.046327	0.050162	0.000710
	0.014343					
z87	0.015785	0.051517	-0.053270	0.003942	0.063859	-0.018522
	0.015290	-0.007688	-0.035165	0.000341	0.014006	0.030780
	-0.026424	0.034686	0.008457	0.049143	-0.055822	0.001514
	-0.009364	-0.039059	-0.024036	0.011532	-0.007952	0.044053
	-0.015280	0.028736	-0.030800	0.036543	0.006505	-0.007445
	-0.007638					
x88	-0.027833	0.004570	0.043163	-0.015711	0.006642	0.020865
	0.039369	-0.013934	-0.001927	0.006558	-0.047503	0.008787
	-0.012676	-0.006212	-0.047903	0.016677	-0.071216	0.071574
	-0.000544	-0.034805	0.002260	-0.046351	0.019717	0.000325
	-0.024874	0.024109	-0.080355	-0.004919	0.040781	-0.014364
	-0.010082					

y88	0.021855	-0.020387	0.067896	0.028843	0.023025	-0.016539
	-0.020998	0.031865	-0.013954	0.041262	0.004422	-0.021496
	-0.026137	-0.015681	0.003031	0.038433	0.004352	0.007709
	0.031510	0.000354	-0.002773	0.060262	0.012636	0.007407
	0.033815	0.001445	0.002524	-0.056379	0.019614	-0.005828
	0.014657					
z88	0.015184	0.054141	-0.049188	-0.003911	0.049888	-0.016423
	0.016944	-0.011734	-0.027626	-0.001220	0.011717	0.029370
	-0.027665	0.037539	0.012018	0.037796	-0.046751	0.004048
	-0.014312	-0.030369	-0.016469	-0.002515	-0.000722	0.045407
	-0.014603	0.023129	-0.037623	0.045674	0.001282	-0.010766
	-0.004011					
x89	-0.027337	0.003995	0.042199	-0.017303	0.009431	0.022938
	0.034283	-0.014437	-0.002864	0.018262	-0.043814	0.011771
	-0.007428	0.007818	-0.056970	0.006144	-0.067697	0.089317
	0.002935	-0.036927	0.003610	-0.045610	0.019627	0.004220
	-0.032435	0.022155	-0.085016	-0.004977	0.039048	-0.014912
	-0.029705					
y89	0.025529	-0.021236	0.069881	0.028174	0.027381	-0.019277
	-0.017697	0.031375	-0.014027	0.039339	0.004487	-0.027773
	-0.021456	-0.010988	0.006511	0.047462	0.002087	0.004585
	0.031698	-0.003360	0.003526	0.063638	0.008797	0.011466
	0.024539	0.007264	0.003403	-0.058327	0.000186	-0.004575
	0.035500					
z89	0.013327	0.058325	-0.046085	-0.009835	0.037605	-0.013505
	0.016877	-0.016355	-0.022486	0.000888	0.012660	0.030964
	-0.026560	0.038504	0.011068	0.021335	-0.037798	0.012438
	-0.018703	-0.020482	-0.012725	-0.010744	0.002756	0.042862
	-0.012052	0.022707	-0.049869	0.051142	-0.004822	-0.011620
	-0.008978					
x90	-0.025344	0.002179	0.041370	-0.017207	0.011193	0.021534
	0.029421	-0.015490	0.005177	0.027610	-0.037902	0.009098
	-0.000278	0.024620	-0.056777	-0.004572	-0.070961	0.089100
	-0.001988	-0.046247	0.013509	-0.038682	0.012366	0.014501
	-0.023148	0.020972	-0.086739	-0.000236	0.034918	-0.014936
	-0.043548					
y90	0.027595	-0.018670	0.071811	0.027004	0.033125	-0.018751
	-0.013833	0.029556	-0.019468	0.035466	0.005205	-0.023647
	-0.020056	-0.013622	0.005205	0.050772	0.008346	-0.000194
	0.037380	0.012377	-0.010998	0.065394	0.002612	0.003897
	-0.001042	0.013349	0.002527	-0.056426	-0.003119	-0.010972
	0.048633					

z90	0.012269	0.061697	-0.043239	-0.013275	0.024205	-0.011163
	0.015336	-0.022132	-0.015151	0.004073	0.011724	0.028510
	-0.022350	0.043074	0.014461	0.008715	-0.036160	0.022950
	-0.026005	-0.022054	0.003422	-0.016241	0.005790	0.041405
	-0.001679	0.023152	-0.067809	0.051495	-0.019228	-0.003115
	-0.021617					
x91	-0.023039	-0.000027	0.041709	-0.015109	0.012274	0.020729
	0.022702	-0.016966	0.016509	0.030336	-0.029290	0.007713
	0.007217	0.038244	-0.050800	-0.020476	-0.069288	0.075429
	0.001005	-0.054213	0.004981	-0.022801	-0.005025	0.019047
	-0.017872	0.026760	-0.086779	0.000006	0.039641	-0.012227
	-0.064141					
y91	0.029255	-0.013807	0.069741	0.023045	0.040676	-0.018289
	-0.009632	0.022988	-0.023891	0.040984	0.006620	-0.025733
	-0.017444	-0.016465	0.000500	0.047845	0.012162	0.005739
	0.029922	0.019232	-0.009362	0.049893	0.003613	-0.013329
	0.003164	0.009271	-0.002967	-0.054010	-0.020730	-0.027431
	0.051627					
z91	0.009850	0.063413	-0.037635	-0.014770	0.006824	-0.009679
	0.009192	-0.025212	-0.002715	-0.002306	0.015086	0.030314
	-0.017179	0.044912	0.015325	-0.007132	-0.028165	0.006281
	-0.023054	-0.026047	0.004497	-0.003122	-0.005137	0.031357
	0.005578	0.025020	-0.070486	0.057021	-0.010633	0.012150
	-0.026839					
x92	-0.022342	-0.000889	0.040410	-0.015299	0.016176	0.022274
	0.016825	-0.019593	0.019963	0.033282	-0.026440	0.009732
	0.010919	0.041480	-0.055084	-0.040775	-0.051705	0.065594
	0.002028	-0.040974	-0.006852	-0.012735	-0.019636	0.023851
	-0.006876	0.031066	-0.088845	-0.010364	0.034079	-0.018618
	-0.064449					
y92	0.031119	-0.009317	0.067485	0.023198	0.046439	-0.016886
	-0.003774	0.015126	-0.022719	0.045128	0.011715	-0.028463
	-0.010935	-0.011093	0.005481	0.041586	-0.004709	0.008892
	0.022747	0.019272	-0.013171	0.048742	0.003695	-0.021034
	0.003492	-0.001170	-0.026391	-0.060754	-0.037262	-0.044423
	0.029093					
z92	0.005938	0.067060	-0.032247	-0.020662	-0.008437	-0.007438
	0.005382	-0.025080	0.004749	-0.008201	0.013679	0.033200
	-0.018603	0.033810	0.010189	-0.018978	-0.009503	-0.003699
	-0.024705	-0.014033	0.003087	-0.003155	-0.009356	0.029053
	0.010747	0.032189	-0.052653	0.059041	-0.009282	0.019935
	-0.003109					

x93	-0.022759	-0.003818	0.038689	-0.014576	0.019357	0.019088
	0.015196	-0.016390	0.024842	0.033090	-0.018356	0.001378
	0.013846	0.033630	-0.041613	-0.054041	-0.046459	0.045412
	0.002627	-0.025835	-0.009493	-0.008586	-0.015278	0.028154
	-0.000288	0.044273	-0.068165	-0.015998	0.041073	-0.039422
	-0.023268					
y93	0.030585	-0.002218	0.067682	0.021878	0.051651	-0.015488
	0.000185	0.012197	-0.019019	0.042179	0.013884	-0.032141
	-0.008978	-0.007794	0.009173	0.043232	-0.012210	0.002603
	0.017707	0.017749	-0.023560	0.040688	-0.000320	-0.021364
	-0.009763	0.001452	-0.014876	-0.050489	-0.030788	-0.024479
	0.009179					
z93	0.003428	0.069220	-0.029220	-0.025516	-0.024921	-0.006398
	0.003811	-0.027427	0.012838	-0.011398	0.014963	0.034822
	-0.017677	0.024661	0.012978	-0.029978	-0.006495	-0.011556
	-0.027762	-0.002856	0.010270	-0.001792	-0.004028	0.022505
	0.023218	0.033866	-0.045163	0.050168	-0.015640	-0.003418
	0.036663					
x94	-0.022038	-0.006848	0.041298	-0.016154	0.015763	0.018803
	0.013808	-0.013444	0.025453	0.030679	-0.010067	-0.000369
	0.011590	0.026705	-0.022856	-0.058838	-0.046584	0.025923
	0.008562	-0.012737	0.009079	-0.009638	-0.013044	0.037759
	0.004155	0.036837	-0.052370	-0.021425	0.047542	-0.044429
	0.022518					
y94	0.026594	-0.000003	0.069903	0.017946	0.052917	-0.016582
	0.005893	0.019337	-0.016343	0.036639	0.017739	-0.041459
	-0.007143	-0.010761	0.003830	0.038720	-0.023568	0.001403
	0.018200	0.012467	-0.024981	0.039717	-0.000687	-0.030445
	-0.012924	0.008626	0.017800	-0.050027	-0.021898	0.013984
	0.003869					
z94	0.002961	0.074138	-0.025903	-0.029252	-0.040050	-0.004625
	0.000111	-0.034925	0.021356	-0.012678	0.017098	0.037181
	-0.017412	0.019114	0.023329	-0.033012	-0.000242	-0.023371
	-0.034335	0.007927	0.017712	-0.008192	0.005254	0.027341
	0.018442	0.027318	-0.047810	0.046014	-0.016967	-0.025442
	0.059021					
x95	-0.020839	-0.009607	0.043608	-0.017742	0.013299	0.021501
	0.008130	-0.011341	0.028281	0.026145	-0.008768	0.003514
	0.009411	0.025563	-0.017800	-0.068056	-0.048729	0.022131
	0.020020	-0.006187	0.010502	-0.004994	-0.016055	0.042121
	0.005876	0.030983	-0.036143	-0.043008	0.053552	-0.032438
	0.030259					

y95	0.024600	0.002253	0.068931	0.015237	0.053323	-0.015923
	0.012022	0.014850	-0.017860	0.034462	0.019479	-0.045686
	-0.004554	-0.005829	0.005377	0.033082	-0.029189	-0.012477
	0.008238	0.018250	-0.013110	0.038729	-0.004342	-0.034706
	0.006613	-0.009879	0.001723	-0.039353	-0.035385	-0.002005
	0.021095					
z95	0.000813	0.079086	-0.021024	-0.031791	-0.054496	-0.003332
	-0.004207	-0.035660	0.032564	-0.017767	0.014383	0.037024
	-0.018427	0.009347	0.022344	-0.033031	-0.000266	-0.018619
	-0.029343	0.009898	0.020903	-0.016534	0.012589	0.031173
	0.001205	0.037597	-0.021606	0.032600	0.001115	-0.004611
	0.048715					
x96	-0.019172	-0.015143	0.043870	-0.016249	0.011546	0.020386
	0.000971	-0.009861	0.028731	0.023748	-0.009716	0.003144
	0.014179	0.028992	-0.010465	-0.076915	-0.051173	0.019454
	0.028633	-0.004256	0.021507	0.003461	-0.013803	0.035535
	0.008244	0.022612	-0.029040	-0.050993	0.053085	-0.020899
	0.029978					
y96	0.024620	0.007228	0.068869	0.011023	0.052481	-0.013208
	0.016458	0.009621	-0.016757	0.030942	0.019225	-0.046323
	-0.003200	-0.002683	0.006861	0.027286	-0.033121	-0.019689
	0.001985	0.021895	-0.002336	0.036086	-0.009044	-0.036857
	0.016610	-0.012926	-0.011106	-0.036576	-0.024129	0.003070
	0.023620					
z96	-0.002918	0.080965	-0.019333	-0.029614	-0.068237	-0.003782
	-0.009109	-0.037551	0.040731	-0.019556	0.014322	0.033115
	-0.013817	0.004130	0.019795	-0.033574	-0.004416	-0.015879
	-0.025774	0.011062	0.024051	-0.021716	0.022045	0.022964
	-0.007231	0.033573	0.005752	0.033139	-0.014136	-0.007660
	0.049215					
x97	-0.015301	-0.020269	0.043967	-0.015440	0.010619	0.021249
	-0.003706	-0.017487	0.029601	0.026212	-0.006878	0.004818
	0.018285	0.031421	0.005596	-0.085961	-0.045904	0.009353
	0.030569	-0.005475	0.033007	0.008973	-0.011731	0.029282
	0.027716	0.008307	-0.055747	-0.045587	0.052599	-0.019327
	0.049075					
y97	0.021442	0.012547	0.065961	0.006277	0.049271	-0.009431
	0.018499	0.004543	-0.014883	0.025939	0.021010	-0.046942
	-0.000131	-0.001514	0.000183	0.018506	-0.035326	-0.020340
	-0.003336	0.024149	0.003799	0.033393	-0.009542	-0.040271
	0.023063	-0.015880	-0.005166	-0.034727	-0.019503	0.001189
	0.018395					

z97	-0.000921	0.082174	-0.013067	-0.026539	-0.078580	-0.002495
	-0.008330	-0.042250	0.048127	-0.015405	0.017352	0.031362
	-0.010854	-0.003504	0.031786	-0.035294	-0.006371	-0.021095
	-0.024997	0.014822	0.028859	-0.027376	0.025051	0.017604
	-0.006257	0.034041	-0.010488	0.033291	-0.020237	0.010973
	0.063131					
x98	-0.012590	-0.025247	0.043203	-0.015308	0.009761	0.022483
	-0.011208	-0.024163	0.028042	0.025343	-0.009834	0.007151
	0.021277	0.034088	0.013598	-0.094388	-0.039425	0.000394
	0.030546	-0.004079	0.038527	0.014956	-0.012480	0.017706
	0.032387	-0.006657	-0.069778	-0.044479	0.051423	-0.013321
	0.054192					
y98	0.018934	0.018140	0.062570	0.002598	0.045121	-0.005766
	0.021262	-0.002547	-0.010662	0.022408	0.024983	-0.046420
	0.003196	0.000815	-0.000091	0.016151	-0.036948	-0.021959
	-0.008607	0.017305	0.009466	0.029067	-0.009357	-0.042580
	0.032398	-0.016147	-0.005099	-0.024402	-0.013687	-0.003388
	0.021965					
z98	0.000506	0.083724	-0.006717	-0.026161	-0.085414	0.000300
	-0.012427	-0.044954	0.050742	-0.017117	0.012012	0.028680
	-0.008182	-0.009653	0.029697	-0.042795	-0.004917	-0.026694
	-0.022024	0.036274	0.029277	-0.031202	0.016482	0.008121
	-0.018897	0.030436	-0.009199	0.022526	-0.023018	0.032045
	0.046925					
x99	-0.010950	-0.030430	0.041301	-0.014861	0.012410	0.019343
	-0.020281	-0.022097	0.026901	0.022322	-0.014438	0.005413
	0.027104	0.037838	0.027337	-0.092266	-0.041304	0.000254
	0.035486	-0.008467	0.035665	0.019656	-0.005751	0.006582
	0.011471	-0.004481	-0.045103	-0.046271	0.060868	0.012134
	0.036046					
y99	0.017713	0.023906	0.059795	-0.000791	0.039687	-0.002339
	0.026553	-0.007503	-0.006245	0.019949	0.026342	-0.047319
	0.003702	-0.001306	-0.003134	0.013470	-0.042613	-0.025114
	-0.017720	0.016080	0.018877	0.023385	-0.007355	-0.043259
	0.034395	-0.014629	-0.007335	-0.021722	-0.011925	0.002052
	0.028334					
z99	-0.003422	0.083732	-0.003186	-0.026286	-0.089339	-0.000777
	-0.020533	-0.041921	0.051454	-0.022857	0.012867	0.018976
	0.002520	-0.005275	0.033665	-0.041693	-0.006302	-0.020590
	-0.004486	0.037624	0.024168	-0.031154	0.012057	0.003141
	-0.030590	0.035751	0.038516	0.019486	-0.007196	0.047012
	0.004955					

x100	-0.008403	-0.031838	0.036611	-0.014541	0.014451	0.019958
	-0.025821	-0.030589	0.024900	0.023379	-0.020698	0.009085
	0.027831	0.033464	0.042301	-0.090696	-0.040659	0.001587
	0.034218	-0.004263	0.026144	0.019968	0.008624	0.004625
	0.004258	0.000811	-0.043658	-0.040491	0.050246	0.003071
	0.051440					
y100	0.017004	0.030320	0.056975	-0.004656	0.033705	0.001772
	0.030772	-0.015685	-0.000220	0.015997	0.027177	-0.046600
	0.006035	-0.001087	-0.005342	0.011049	-0.046463	-0.029075
	-0.025143	0.016287	0.023874	0.014429	-0.008282	-0.042702
	0.038477	-0.016805	-0.003917	-0.016736	-0.009410	0.002692
	0.026045					
z100	-0.006402	0.085184	-0.001573	-0.025490	-0.093746	0.004184
	-0.025891	-0.044904	0.050286	-0.022003	0.012381	0.016607
	0.001464	-0.014385	0.038329	-0.041336	-0.000139	-0.011092
	0.005556	0.046987	0.019955	-0.029845	0.010698	0.004220
	-0.036788	0.050035	0.035830	0.024135	-0.019076	0.025006
	0.022518					
x101	-0.005705	-0.033579	0.033817	-0.014748	0.014609	0.020360
	-0.030833	-0.037921	0.027305	0.023899	-0.024060	0.010012
	0.029907	0.024642	0.053114	-0.087973	-0.041580	0.003837
	0.033656	-0.003801	0.006227	0.019272	0.030398	0.000337
	-0.001805	-0.015655	-0.041489	-0.036815	0.036132	-0.000033
	0.050024					
y101	0.015726	0.037799	0.052856	-0.007152	0.026017	0.005065
	0.034521	-0.024504	0.006453	0.011471	0.028194	-0.045962
	0.009160	-0.000315	-0.006209	0.007172	-0.048287	-0.032311
	-0.029710	0.019505	0.024760	0.005792	-0.008989	-0.039326
	0.039264	-0.012548	0.000285	-0.010475	-0.007367	0.002323
	0.023204					
z101	-0.006051	0.082953	0.007023	-0.027544	-0.096622	0.012362
	-0.026969	-0.047939	0.055747	-0.019550	0.017913	0.011248
	-0.001348	-0.026583	0.035608	-0.037237	-0.003285	-0.010861
	0.011806	0.058109	0.021539	-0.030193	0.014858	0.007541
	-0.025328	0.008820	0.009968	0.020362	-0.029335	0.013388
	0.008789					
x102	-0.007802	-0.034415	0.026401	-0.011336	0.014564	0.017172
	-0.041355	-0.046267	0.027052	0.022881	-0.030794	0.010510
	0.037681	0.013147	0.056720	-0.077423	-0.034797	0.009303
	0.033427	-0.026545	-0.005179	0.020220	0.057215	-0.007505
	-0.010917	-0.016665	-0.027662	-0.016825	0.006913	-0.017635
	0.047897					

y102	0.015584	0.043997	0.048150	-0.009164	0.017368	0.009363
	0.037878	-0.033345	0.011416	0.006092	0.029127	-0.042550
	0.013670	-0.000308	-0.008611	0.004115	-0.048631	-0.038793
	-0.032849	0.017572	0.025660	-0.000720	-0.013511	-0.037871
	0.040789	-0.011854	0.005742	-0.000280	-0.003681	-0.004384
	0.027044					
z102	-0.009814	0.079472	0.011175	-0.024361	-0.096082	0.017421
	-0.030204	-0.051185	0.056734	-0.026078	0.029063	-0.002569
	-0.005800	-0.034643	0.033541	-0.036783	0.007837	-0.025984
	0.010721	0.053260	0.027704	-0.030173	-0.003741	0.008236
	0.000042	0.003265	0.013641	0.009336	-0.025982	0.008387
	-0.000274					
x103	-0.010268	-0.033337	0.021758	-0.011110	0.014804	0.012065
	-0.041269	-0.040177	0.025094	0.017355	-0.026664	0.002375
	0.049515	0.007632	0.058842	-0.056360	-0.051035	0.000743
	0.020948	-0.038808	0.009158	0.023117	0.068961	0.010293
	-0.024876	-0.024549	0.011167	-0.004988	-0.000114	-0.025180
	0.047569					
y103	0.014966	0.048994	0.042116	-0.012666	0.006568	0.013128
	0.042902	-0.038232	0.016087	-0.000205	0.026855	-0.038877
	0.017860	-0.000699	-0.013488	0.000713	-0.046959	-0.040587
	-0.037409	0.012869	0.019296	-0.006468	-0.014418	-0.034194
	0.034105	-0.006655	0.012562	0.001153	-0.004837	0.002102
	0.016535					
z103	-0.014745	0.067329	0.017506	-0.024371	-0.087210	0.024497
	-0.031340	-0.041639	0.055073	-0.041141	0.033674	-0.025190
	-0.011467	-0.048672	0.030373	-0.030409	0.000693	-0.016541
	0.012780	0.056475	0.025570	-0.029842	-0.008019	0.003892
	0.011223	0.003889	0.024956	-0.001779	0.003097	-0.017359
	0.009271					
x104	-0.009078	-0.028778	0.016025	-0.009974	0.012900	0.008346
	-0.039863	-0.041253	0.024482	0.011548	-0.024088	-0.000715
	0.056537	0.006504	0.071610	-0.037020	-0.055801	-0.013609
	0.004391	-0.039926	0.010102	0.018427	0.072043	0.027055
	-0.024602	-0.022111	0.034954	0.007901	0.004735	-0.023942
	0.039407					
y104	0.015062	0.054019	0.036800	-0.014292	-0.003076	0.018834
	0.046332	-0.046643	0.016631	-0.004406	0.021910	-0.030026
	0.021067	-0.000405	-0.015331	0.001611	-0.042577	-0.040574
	-0.042034	0.007379	0.012732	-0.010467	-0.014623	-0.031290
	0.033706	-0.005449	0.010850	0.010341	-0.003979	-0.006535
	0.001522					

z104	-0.014154	0.057547	0.022084	-0.023594	-0.081106	0.026177
	-0.026449	-0.037093	0.046836	-0.041234	0.030701	-0.018074
	-0.013193	-0.043905	0.039877	-0.013243	-0.000698	-0.018141
	0.005038	0.040613	0.015113	-0.030038	-0.011478	0.001760
	0.006164	0.005959	0.024566	0.007373	0.001169	-0.032284
	-0.008112					
x105	-0.007125	-0.025705	0.009429	-0.011062	0.010588	0.006358
	-0.038490	-0.038963	0.022141	0.005032	-0.021516	0.002185
	0.063216	0.005241	0.064523	-0.026972	-0.049686	-0.024031
	-0.009469	-0.039204	0.004872	0.022160	0.070093	0.035750
	-0.007767	-0.031837	0.044986	0.010464	-0.006108	-0.002219
	0.028993					
y105	0.016234	0.058183	0.033660	-0.013594	-0.005436	0.025108
	0.046396	-0.052969	0.018443	-0.010964	0.018664	-0.021141
	0.025481	-0.001035	-0.021600	-0.002676	-0.042965	-0.037750
	-0.045156	0.009217	0.008643	-0.004921	-0.019601	-0.037856
	0.037274	-0.014207	0.013636	0.022459	0.001831	-0.004357
	-0.000564					
z105	-0.011765	0.045903	0.029674	-0.020719	-0.063606	0.032048
	-0.026983	-0.029166	0.039580	-0.047687	0.033086	-0.012782
	-0.007717	-0.044626	0.035578	-0.012316	-0.008606	-0.012560
	-0.001563	0.037556	0.011154	-0.010922	-0.025428	-0.019340
	0.021319	-0.015511	0.029449	0.009979	0.002603	-0.017968
	-0.002090					
x106	-0.009282	-0.022319	0.002456	-0.009275	0.008226	0.004996
	-0.037771	-0.037137	0.018545	-0.000714	-0.023083	0.010210
	0.066005	-0.004156	0.041386	-0.023270	-0.035869	-0.027912
	-0.023047	-0.017068	-0.006343	0.031344	0.066541	0.035235
	0.008691	-0.022454	0.047664	0.010151	-0.004751	0.003239
	0.013597					
y106	0.013708	0.061288	0.032133	-0.014384	-0.009071	0.032572
	0.047482	-0.055582	0.017648	-0.018719	0.012507	-0.009675
	0.026743	-0.010795	-0.041072	-0.014162	-0.035122	-0.030476
	-0.050480	0.019503	0.000612	0.001134	-0.017531	-0.046744
	0.036432	-0.007067	0.019540	0.013389	0.021341	0.002214
	-0.011032					
z106	-0.013181	0.035076	0.037574	-0.017951	-0.050647	0.035529
	-0.025381	-0.019313	0.034549	-0.054470	0.034302	-0.004688
	-0.004035	-0.049073	0.026654	-0.013841	-0.007599	-0.007397
	-0.013934	0.037390	0.006206	0.003226	-0.032984	-0.040575
	0.023513	0.001709	0.026167	0.002610	0.029054	-0.001059
	-0.014109					

x107	-0.007186	-0.018576	-0.003358	-0.007920	0.004492	0.000035
	-0.034314	-0.035460	0.018395	-0.004716	-0.023527	0.013073
	0.069781	0.003990	0.034544	-0.008415	-0.026348	-0.037016
	-0.028670	-0.025364	-0.011362	0.030279	0.057579	0.036785
	0.026634	-0.025756	0.047743	0.018334	-0.003279	0.003447
	-0.000956					
y107	0.013192	0.063059	0.030166	-0.019407	-0.013138	0.033631
	0.052234	-0.055985	0.020131	-0.021955	0.004637	-0.005221
	0.030495	-0.004531	-0.043696	-0.021886	-0.030154	-0.029838
	-0.054032	-0.004040	-0.004257	0.000558	-0.012823	-0.044906
	0.030359	-0.012965	0.032992	0.016036	0.035224	0.010047
	-0.015560					
z107	-0.013583	0.025220	0.045204	-0.018991	-0.038516	0.034468
	-0.021632	-0.006664	0.031254	-0.055540	0.030372	-0.001771
	-0.001872	-0.040998	0.023232	-0.019907	-0.003941	-0.007822
	-0.017082	0.011935	0.004799	0.011905	-0.031241	-0.045945
	0.024848	-0.007036	0.029872	-0.000204	0.041586	0.015355
	-0.006313					
x108	-0.004001	-0.014523	-0.012798	-0.009506	-0.000866	-0.003846
	-0.024817	-0.027678	0.021753	-0.006143	-0.027466	0.013766
	0.066810	0.012345	0.032629	0.023114	-0.020750	-0.043337
	-0.036031	-0.032687	-0.014093	0.004428	0.047166	0.040399
	0.027485	-0.034782	0.049489	0.003343	-0.014794	-0.004484
	0.001356					
y108	0.014851	0.061029	0.020622	-0.024678	-0.018154	0.034539
	0.059700	-0.047238	0.022670	-0.023239	-0.000254	-0.006867
	0.026413	-0.011185	-0.038203	-0.004001	-0.025988	-0.038500
	-0.059309	-0.019253	-0.012595	-0.018385	-0.031174	-0.032635
	-0.013348	-0.032143	0.024764	-0.003699	0.009021	-0.001890
	-0.025427					
z108	-0.013597	0.017221	0.048176	-0.021934	-0.026113	0.033021
	-0.012659	0.010739	0.027459	-0.059728	0.024121	-0.003760
	-0.006360	-0.044678	0.032278	-0.000113	-0.012599	-0.008059
	-0.024483	0.008963	0.004666	-0.007453	-0.035438	-0.032524
	0.011506	-0.028172	0.041240	-0.029559	0.028895	-0.013553
	0.023123					
x109	-0.003263	-0.009397	-0.022765	-0.005555	-0.005093	-0.009189
	-0.015493	-0.027018	0.022485	-0.007039	-0.034852	0.020730
	0.063969	0.028118	0.026069	0.047916	-0.010932	-0.037830
	-0.040433	-0.024646	-0.019987	-0.007024	0.034447	0.037944
	0.038583	-0.036606	0.035080	-0.001027	-0.016681	-0.018005
	-0.000463					

y109	0.014738	0.060429	0.017974	-0.028915	-0.016117	0.036594
	0.068339	-0.050115	0.018146	-0.027939	-0.013370	0.006543
	0.027271	-0.006170	-0.032977	0.002154	-0.027561	-0.029091
	-0.060766	-0.012118	-0.029040	-0.013337	-0.021538	-0.007304
	-0.030561	-0.048365	0.018875	-0.002155	-0.000248	-0.003726
	-0.025765					
z109	-0.015053	0.009762	0.049701	-0.019947	-0.014298	0.031645
	-0.004898	0.018712	0.021582	-0.061461	0.014418	0.008074
	-0.006909	-0.037591	0.033407	0.014875	-0.008670	-0.002219
	-0.027294	0.022348	0.001769	-0.014145	-0.030723	-0.015226
	0.008531	-0.044449	0.029851	-0.038302	0.015300	-0.022236
	0.047741					
x110	-0.001683	-0.007436	-0.029791	0.005844	-0.011684	-0.014498
	-0.016662	-0.027774	0.027511	-0.007133	-0.032831	0.017471
	0.066955	0.043851	0.013420	0.054168	0.005342	-0.042373
	-0.031576	-0.026931	-0.017492	0.003108	-0.004588	-0.002407
	0.050385	-0.028787	0.007029	0.012590	0.008377	-0.001611
	-0.040549					
y110	0.019017	0.054706	0.019024	-0.026188	-0.015486	0.038860
	0.060109	-0.053678	0.011915	-0.027759	-0.013919	0.005040
	0.032773	-0.005177	-0.038486	-0.014225	-0.013788	-0.023448
	-0.054097	-0.017980	-0.021459	0.015112	-0.027597	-0.016309
	-0.039748	-0.042439	-0.002850	0.025992	0.020908	0.039986
	-0.081803					
z110	-0.014817	0.003239	0.048299	-0.012833	-0.007745	0.027069
	-0.004037	0.026093	0.017028	-0.057591	0.008353	0.013602
	-0.006156	-0.032009	0.028179	0.023147	0.005487	-0.004079
	-0.023702	0.031557	0.008043	-0.022387	-0.033019	-0.014287
	0.008387	-0.045458	0.018283	-0.040293	0.009549	-0.016886
	0.043701					
x111	-0.003125	-0.002797	-0.047845	0.014688	-0.023907	-0.014481
	-0.011847	-0.021717	0.047137	-0.011720	-0.032819	0.010454
	0.044164	0.039071	0.005859	0.084329	0.011979	-0.049940
	-0.024021	-0.015375	-0.020662	-0.008454	-0.050005	-0.029068
	0.037853	-0.013012	-0.004100	0.003763	0.024452	-0.000078
	-0.026024					
y111	0.021442	0.045934	0.015241	-0.022363	-0.009360	0.038270
	0.058816	-0.059453	0.000162	-0.021102	-0.007856	0.003176
	0.035912	0.001594	-0.027636	-0.009993	-0.008882	-0.009979
	-0.074502	-0.011647	-0.003097	0.027229	-0.018415	0.010544
	-0.035748	-0.026414	-0.030929	0.036950	0.047512	0.072501
	-0.106676					

z111	-0.016257	-0.000207	0.047478	-0.003496	-0.001758	0.020173
	-0.002299	0.028724	0.009058	-0.049254	0.002464	0.024769
	0.005944	-0.018448	0.020521	0.018717	0.014674	-0.005780
	-0.022417	0.036651	0.013801	-0.028001	-0.032413	-0.021684
	0.026062	-0.039463	0.012019	-0.043284	-0.003966	-0.005901
	0.029686					
x112	-0.017544	0.015270	-0.056499	0.008729	-0.041835	-0.001249
	0.005799	-0.002993	0.045155	-0.028340	-0.036642	0.008333
	0.010013	0.043049	-0.020494	0.071564	0.013275	-0.043544
	-0.017295	0.018537	-0.017564	-0.005754	-0.040408	-0.062966
	-0.011497	-0.024456	-0.003322	0.011368	0.008095	-0.020752
	-0.006518					
y112	0.020290	0.036401	0.016019	-0.015796	-0.000912	0.027357
	0.047278	-0.059657	-0.012980	-0.016843	0.006350	0.010070
	0.011999	-0.008890	-0.003234	-0.008991	0.004185	0.005328
	-0.085246	0.017814	-0.005577	0.044505	0.000998	0.026987
	-0.019708	-0.003965	0.003081	0.057351	0.066133	0.089196
	-0.015630					
z112	-0.022471	-0.005164	0.037463	0.015840	0.001150	0.009561
	-0.003491	0.041463	-0.003819	-0.033428	-0.006630	0.040208
	0.007185	-0.014821	-0.004248	0.009202	0.009895	-0.035714
	-0.022535	0.036590	0.032399	-0.060186	-0.012332	-0.037065
	0.007922	-0.023530	0.000601	-0.033906	-0.027963	-0.003176
	0.006802					
x113	-0.030732	0.026484	-0.054613	0.007094	-0.048232	0.008935
	0.022788	0.009499	0.033014	-0.043350	-0.047319	0.015503
	0.002155	0.064951	-0.037455	0.048095	0.008785	0.004879
	-0.018809	0.049650	-0.018215	0.007736	-0.029196	-0.077281
	-0.030341	-0.046454	-0.019916	0.011066	-0.026892	-0.043774
	-0.041044					
y113	0.018072	0.026195	0.016809	-0.007097	0.004111	0.023888
	0.037558	-0.047476	-0.035288	-0.015183	0.019562	0.035299
	0.009407	-0.005945	-0.001711	-0.019624	0.005808	0.036096
	-0.095626	0.029491	-0.035323	0.070581	0.023338	0.032138
	-0.019574	-0.004453	0.041909	0.049056	0.055645	0.086151
	-0.017387					
z113	-0.027582	-0.009864	0.030093	0.034101	0.003304	-0.003563
	-0.005333	0.056009	-0.016045	-0.016326	-0.019444	0.054867
	0.004269	-0.010995	-0.021409	-0.003794	-0.002835	-0.058304
	-0.015094	0.021478	0.044918	-0.093846	0.006057	-0.057112
	-0.008163	-0.009795	-0.014499	-0.020471	-0.040853	-0.009242
	-0.018370					

x114	-0.037638	0.040909	-0.055794	0.008448	-0.056479	0.019100
	0.034973	-0.009266	0.034575	-0.062660	-0.066242	0.024520
	-0.013973	0.093927	-0.037359	0.045808	0.011775	0.037602
	-0.019536	0.078684	0.004160	0.011497	-0.042277	-0.076477
	-0.008997	-0.093958	-0.087170	0.031821	-0.058641	-0.101284
	-0.020429					
y114	0.021524	0.024601	0.008057	-0.001668	0.001079	0.013469
	0.033461	-0.062791	-0.033472	-0.016798	0.013833	0.061354
	-0.000740	0.018823	0.017913	0.015152	0.004618	0.053120
	-0.096291	0.028435	-0.025448	0.072032	0.012205	0.054616
	-0.017476	-0.020665	0.017313	0.054624	0.032143	0.043523
	0.025528					
z114	-0.031171	-0.013870	0.025661	0.047458	0.005962	-0.007449
	-0.007962	0.070198	-0.031405	-0.001550	-0.029266	0.067322
	-0.002436	-0.019241	-0.040801	-0.028056	-0.006590	-0.077677
	-0.010413	0.016934	0.046610	-0.114934	0.023225	-0.074451
	-0.008925	-0.007363	-0.036555	-0.005368	-0.050496	-0.024214
	-0.034304					
x115	-0.049334	0.046153	-0.049162	0.013521	-0.060461	0.018990
	0.040288	-0.001841	0.034907	-0.078351	-0.079017	0.000225
	-0.029673	0.106516	-0.034190	0.038101	0.012075	0.078805
	-0.013946	0.082145	0.017852	0.014667	-0.039509	-0.071379
	-0.021893	-0.101517	-0.087459	0.026827	-0.058396	-0.076206
	-0.026178					
y115	0.017121	0.016726	0.004198	0.003701	-0.009705	0.002097
	0.023613	-0.047482	-0.034599	-0.012044	0.005613	0.064426
	-0.001838	0.029313	0.029216	0.032097	0.011670	0.070386
	-0.080691	0.014970	-0.025850	0.068805	0.008251	0.067983
	-0.032737	0.001189	0.032231	0.020557	0.036746	0.064622
	0.036342					
z115	-0.032511	-0.014442	0.022635	0.055798	0.011654	-0.011443
	-0.011147	0.082663	-0.044165	0.002787	-0.038585	0.074902
	-0.019160	-0.032349	-0.052653	-0.047695	-0.015733	-0.082359
	-0.012390	0.020232	0.049448	-0.113209	0.033387	-0.096010
	-0.007370	-0.020346	-0.045280	0.026591	-0.053504	-0.025304
	-0.058051					
x116	-0.060681	0.044229	-0.039371	0.021063	-0.054736	0.011793
	0.037945	0.016715	0.034287	-0.092324	-0.075042	-0.038189
	-0.045900	0.101956	-0.031690	0.020478	0.005904	0.110277
	-0.006419	0.077452	0.018209	0.014081	-0.016941	-0.074962
	-0.055623	-0.070294	-0.039295	0.015411	-0.059486	-0.015454
	-0.038090					

y116	0.012889	0.008634	0.002684	0.008737	-0.017379	-0.002863
	0.011601	-0.035155	-0.033137	-0.007401	0.000329	0.067010
	-0.003833	0.023353	0.028639	0.027506	0.018663	0.078160
	-0.062930	0.026594	-0.028127	0.054578	0.016826	0.063181
	-0.027703	0.019238	0.030946	0.000946	0.027546	0.052371
	0.055691					
z116	-0.030698	-0.009647	0.016871	0.061263	0.013428	-0.009322
	-0.013461	0.076615	-0.051781	0.001717	-0.051662	0.087102
	-0.037580	-0.037893	-0.051390	-0.053842	-0.017202	-0.088423
	-0.015418	0.030289	0.054435	-0.100645	0.022730	-0.101121
	0.022595	-0.057668	-0.072087	0.051911	-0.043851	-0.046856
	-0.058002					
x117	-0.062485	0.050114	-0.031728	0.034802	-0.050952	0.018779
	0.037750	-0.012190	0.046373	-0.101165	-0.073767	-0.053695
	-0.056619	0.101309	-0.004950	0.018193	0.019050	0.092512
	-0.012998	0.075044	0.029330	-0.004930	-0.026027	-0.041108
	-0.004533	-0.064056	-0.077205	0.041869	-0.051522	-0.055596
	0.015503					
y117	0.012786	0.005835	0.002551	0.016594	-0.023027	-0.002106
	0.002526	-0.032487	-0.024439	-0.001490	-0.008855	0.071558
	-0.004661	0.016212	0.037168	0.035688	0.020599	0.071840
	-0.056533	0.035201	-0.011585	0.038103	0.015350	0.066260
	-0.001641	0.027439	0.002261	0.013671	0.035974	0.013041
	0.064699					
z117	-0.032315	-0.011428	0.010473	0.063053	0.017877	-0.015134
	-0.021269	0.084581	-0.062601	-0.002046	-0.058089	0.085936
	-0.057501	-0.047291	-0.052592	-0.070573	-0.021507	-0.083905
	-0.004624	0.034032	0.045315	-0.070384	0.021827	-0.118024
	0.008707	-0.071247	-0.047715	0.040255	-0.024232	-0.001204
	-0.074850					
x118	-0.065420	0.047533	-0.019129	0.049125	-0.042012	0.014581
	0.032935	-0.015808	0.050388	-0.105908	-0.073025	-0.087344
	-0.067334	0.090357	0.022698	0.017355	0.019978	0.073106
	-0.020309	0.029831	0.039222	-0.009055	-0.029669	-0.012370
	0.000515	-0.032855	-0.034468	0.050378	-0.023014	-0.018700
	0.015308					
y118	0.011517	0.001857	0.005762	0.023882	-0.026763	-0.002342
	-0.008281	-0.019674	-0.018266	0.007040	-0.021801	0.073601
	-0.010154	0.003188	0.042947	0.041648	0.012956	0.070961
	-0.049804	0.032290	0.004731	0.027194	0.015203	0.058589
	0.002575	0.038397	0.002655	0.019065	0.042523	0.007344
	0.049001					

z118	-0.032754	-0.008635	0.001591	0.063690	0.020390	-0.023547
	-0.027552	0.084176	-0.065338	-0.010962	-0.059034	0.084072
	-0.067770	-0.054264	-0.047886	-0.080007	-0.013872	-0.078530
	0.009034	0.047063	0.034016	-0.049154	0.017054	-0.128144
	0.006777	-0.073693	-0.037498	0.036288	-0.002878	0.043990
	-0.063595					
x119	-0.066097	0.043373	-0.012988	0.057603	-0.033042	0.012042
	0.029918	-0.009851	0.036994	-0.108788	-0.088689	-0.106144
	-0.085975	0.067902	0.020029	0.003675	0.038694	0.054054
	-0.039627	0.010742	0.041301	0.009017	-0.019752	0.012548
	0.009260	-0.036529	0.025405	0.024377	-0.012191	0.043754
	0.024455					
y119	0.009350	-0.000935	0.012007	0.029108	-0.033963	-0.004528
	-0.022369	-0.003311	-0.011416	0.016096	-0.033787	0.082007
	-0.017986	-0.009110	0.053334	0.049288	0.001049	0.075035
	-0.042701	0.025787	0.014997	0.012591	0.013653	0.038338
	0.005419	0.047656	-0.000745	0.027634	0.034945	0.012899
	0.032163					
z119	-0.032727	-0.002753	-0.001442	0.067376	0.024570	-0.043032
	-0.032190	0.079623	-0.057086	-0.018164	-0.051501	0.069139
	-0.072206	-0.049913	-0.015051	-0.065103	-0.013696	-0.070724
	0.014882	0.020535	0.028282	-0.047942	-0.007713	-0.110213
	0.008444	-0.050321	-0.038117	0.052748	0.037437	0.103150
	-0.020344					
x120	-0.068117	0.039110	-0.004194	0.068425	-0.018858	0.003738
	0.034278	-0.005846	0.031151	-0.108248	-0.101658	-0.121702
	-0.110146	0.054110	0.031999	-0.012029	0.047842	0.030274
	-0.059917	-0.032985	0.035444	0.016422	-0.048803	0.043342
	0.006107	-0.018420	0.055645	0.012588	0.027109	0.123877
	0.037183					
y120	0.005262	-0.002235	0.018973	0.032500	-0.040658	-0.002551
	-0.032946	0.012599	-0.010087	0.029518	-0.048555	0.097020
	-0.026575	-0.025805	0.053369	0.053926	0.001401	0.092596
	-0.039988	0.034403	0.022700	-0.005437	0.029451	0.023019
	0.004469	0.063546	0.026608	0.019524	0.021768	-0.006069
	0.022645					
z120	-0.032104	0.003244	-0.008948	0.069496	0.034998	-0.052439
	-0.031553	0.081980	-0.051224	-0.010414	-0.061214	0.053308
	-0.072193	-0.047929	0.009659	-0.038933	-0.000790	-0.047181
	0.002469	-0.009532	0.026024	-0.041035	0.005218	-0.070952
	-0.013744	-0.022761	0.020804	0.041028	0.098557	0.134282
	0.025557					

x121	-0.074740	0.041932	-0.002300	0.068975	-0.008492	0.007535
	0.035678	-0.014252	0.012651	-0.093312	-0.088284	-0.117542
	-0.123031	0.042565	0.029470	-0.038551	0.048937	0.027837
	-0.026991	-0.059838	0.024672	0.007942	-0.075143	0.052070
	0.025547	-0.020126	0.075072	0.027807	0.041123	0.059034
	0.039523					
y121	0.004523	-0.004409	0.025696	0.039384	-0.041554	-0.001831
	-0.037103	0.019934	-0.008467	0.038001	-0.065641	0.107413
	-0.033302	-0.037126	0.050506	0.051368	0.009244	0.119217
	-0.060833	0.059927	0.026713	-0.014379	0.039348	0.024947
	0.010639	0.072414	0.045595	0.011008	-0.014227	0.026693
	0.042277					
z121	-0.034056	0.013090	-0.016648	0.069639	0.046251	-0.039557
	-0.031558	0.070342	-0.053159	0.004856	-0.064152	0.069869
	-0.084792	-0.046903	0.006852	-0.034070	0.001335	-0.007509
	-0.007721	-0.016557	0.015050	-0.050986	-0.001797	-0.049878
	0.011027	-0.019106	0.038326	0.039131	0.098260	0.097568
	0.039432					
x122	-0.080684	0.041578	0.004346	0.069624	-0.000553	0.005297
	0.029451	-0.016245	-0.009025	-0.083544	-0.077227	-0.115631
	-0.130645	0.016684	0.024265	-0.056597	0.050406	0.034182
	-0.008801	-0.064221	0.016593	-0.004781	-0.089760	0.051418
	0.027209	-0.000513	0.106478	0.029497	0.007721	0.031680
	0.029987					
y122	0.007142	-0.000153	0.026808	0.040386	-0.041666	0.002569
	-0.039435	0.009764	-0.006778	0.044197	-0.073910	0.117121
	-0.032038	-0.026749	0.053308	0.052004	0.010558	0.119850
	-0.071831	0.065650	0.027723	-0.021864	0.042932	0.019108
	0.014094	0.073529	0.007068	0.001632	-0.034724	0.008802
	0.028101					
z122	-0.031029	0.026073	-0.027267	0.066644	0.051587	-0.033815
	-0.033384	0.051481	-0.057969	0.010742	-0.056312	0.078353
	-0.085755	-0.032966	0.008024	-0.014001	-0.004878	0.010308
	-0.009722	-0.027226	0.011705	-0.060307	-0.002156	-0.037975
	0.019383	-0.003792	0.044101	0.026736	0.071508	0.065617
	0.026424					
x123	-0.082504	0.042370	0.011277	0.069709	0.006876	0.006534
	0.026577	-0.019995	-0.026734	-0.071872	-0.073031	-0.103733
	-0.136399	-0.008513	0.020239	-0.070470	0.051152	0.036591
	0.006957	-0.053678	0.005305	-0.016298	-0.107312	0.045291
	0.028160	0.021106	0.107729	0.021720	-0.024814	-0.002080
	0.030933					

y123	0.008563	0.000719	0.027621	0.036954	-0.039424	0.002153
	-0.041579	0.008608	-0.009935	0.044944	-0.070249	0.109319
	-0.028307	-0.016792	0.046080	0.053394	0.006495	0.109614
	-0.069024	0.047848	0.025931	-0.023912	0.056076	0.016542
	0.016512	0.058572	0.002491	-0.022327	-0.050636	-0.014372
	0.007251					
z123	-0.026491	0.037922	-0.037987	0.059711	0.057024	-0.027085
	-0.033729	0.034282	-0.063469	0.012998	-0.045194	0.081969
	-0.083227	-0.018869	0.001843	0.006768	-0.014623	0.020864
	-0.005673	-0.040181	0.005542	-0.064699	0.000733	-0.026413
	0.030848	-0.000933	0.045711	0.001532	0.039375	0.019146
	0.021886					
x124	-0.080873	0.040093	0.019115	0.066806	0.014573	0.007425
	0.025095	-0.016361	-0.038061	-0.062543	-0.065762	-0.095282
	-0.136678	-0.028006	0.017084	-0.075363	0.039654	0.034411
	0.019799	-0.052440	-0.005478	-0.028085	-0.107827	0.034594
	0.019063	0.043526	0.106132	0.000682	-0.053027	-0.023328
	0.019452					
y124	0.008761	0.002203	0.027243	0.032608	-0.037802	0.000876
	-0.040336	0.006517	-0.015267	0.047885	-0.069224	0.101112
	-0.025508	-0.014838	0.029986	0.044145	0.012838	0.099502
	-0.059627	0.042425	0.017857	-0.015434	0.052749	0.014953
	0.031716	0.037329	-0.009697	-0.050854	-0.055431	-0.047245
	0.000167					
z124	-0.021231	0.048854	-0.048828	0.050586	0.061728	-0.022351
	-0.030791	0.018098	-0.068099	0.015901	-0.035796	0.081259
	-0.076561	-0.010158	-0.005594	0.022453	-0.021188	0.029986
	-0.000289	-0.044575	-0.004011	-0.055859	-0.002594	-0.013199
	0.041754	0.000737	0.036944	-0.027686	0.021408	-0.022037
	0.020535					
x125	-0.075965	0.037009	0.026137	0.058924	0.020428	0.006097
	0.028820	-0.010699	-0.040571	-0.053768	-0.057226	-0.087668
	-0.130629	-0.042207	0.014562	-0.070987	0.021438	0.028722
	0.032286	-0.050332	-0.017466	-0.026754	-0.103976	0.032621
	-0.000719	0.056700	0.088128	-0.019707	-0.072283	-0.025867
	0.008736					
y125	0.008744	0.000680	0.028073	0.030675	-0.035164	0.001400
	-0.041171	0.006641	-0.020508	0.048117	-0.069654	0.092799
	-0.025453	-0.016889	0.014416	0.027540	0.018894	0.085877
	-0.055103	0.033616	0.013478	-0.013633	0.048580	0.001087
	0.058535	0.021052	-0.009978	-0.081130	-0.054971	-0.073841
	0.001716					

z125	-0.015595	0.057115	-0.059666	0.043589	0.067434	-0.017172
	-0.027804	0.002586	-0.070887	0.016563	-0.025611	0.080717
	-0.068879	-0.001759	-0.013087	0.034305	-0.031649	0.034524
	-0.002879	-0.049845	-0.012115	-0.047873	-0.001203	-0.008684
	0.056281	0.008613	0.029393	-0.054673	0.017817	-0.055238
	0.021855					
x126	-0.068081	0.033355	0.031354	0.048282	0.025042	0.006790
	0.036465	-0.010066	-0.036703	-0.043712	-0.047380	-0.073442
	-0.122380	-0.053851	0.017469	-0.055737	0.003835	0.018243
	0.031137	-0.047313	-0.032831	-0.020483	-0.086704	0.027088
	-0.013243	0.069564	0.044346	-0.028531	-0.076432	-0.020818
	0.007541					
y126	0.007947	-0.003656	0.031620	0.029649	-0.034464	-0.000221
	-0.042363	0.014104	-0.021294	0.043243	-0.071192	0.077038
	-0.025742	-0.016053	-0.003318	0.005639	0.018512	0.068924
	-0.041905	0.022237	0.015578	-0.009337	0.032094	-0.000399
	0.075664	-0.004520	0.001718	-0.105506	-0.046299	-0.090335
	-0.001079					
z126	-0.010188	0.062417	-0.067846	0.038933	0.071655	-0.014348
	-0.023023	-0.006268	-0.070326	0.011918	-0.018085	0.074084
	-0.055578	0.011096	-0.022993	0.045962	-0.051773	0.034451
	0.000126	-0.054188	-0.015330	-0.034885	-0.012312	0.010262
	0.059002	0.007383	0.025061	-0.076597	0.030567	-0.080190
	0.015158					
x127	-0.059300	0.028927	0.035857	0.036800	0.025893	0.009650
	0.046776	-0.007474	-0.029946	-0.036783	-0.046163	-0.054628
	-0.109001	-0.063802	0.011823	-0.030436	-0.019909	0.013454
	0.022686	-0.044491	-0.050672	-0.010941	-0.062401	0.020618
	-0.019794	0.081704	-0.002934	-0.045537	-0.051102	-0.012403
	-0.005517					
y127	0.007994	-0.010002	0.037288	0.027906	-0.031884	-0.004030
	-0.043139	0.022016	-0.016095	0.039219	-0.064564	0.056912
	-0.028556	-0.014827	-0.008896	-0.020178	0.023880	0.043275
	-0.028820	0.012148	0.022430	-0.006104	0.014942	0.002055
	0.084019	-0.029489	0.007599	-0.100013	-0.039063	-0.076286
	0.004579					
z127	-0.004021	0.064586	-0.073332	0.033070	0.079280	-0.013919
	-0.016552	-0.014590	-0.066878	0.011626	0.004109	0.063128
	-0.044283	0.021680	-0.020835	0.056363	-0.064818	0.019211
	0.001557	-0.054638	-0.015728	-0.018662	-0.022632	0.029187
	0.050267	0.009672	0.006946	-0.061195	0.037326	-0.075066
	0.016219					

x128	-0.050160	0.024927	0.036359	0.026277	0.021173	0.015181
	0.055292	-0.010559	-0.020646	-0.031566	-0.055313	-0.027933
	-0.090771	-0.069408	-0.005647	-0.001134	-0.045602	0.013157
	0.011712	-0.036557	-0.063008	-0.005212	-0.043381	0.016367
	-0.008867	0.077639	-0.045154	-0.073162	-0.027649	-0.020634
	-0.013008					
y128	0.009650	-0.016243	0.047249	0.025971	-0.024766	-0.010590
	-0.043100	0.029074	-0.009974	0.036596	-0.047442	0.032201
	-0.034635	-0.017124	0.004465	-0.044665	0.034369	0.014230
	-0.022052	0.001865	0.018528	0.000675	0.005568	-0.000144
	0.078849	-0.042984	-0.000652	-0.049893	-0.008232	-0.033806
	0.014546					
z128	0.000028	0.060863	-0.070872	0.029208	0.088065	-0.020580
	-0.009759	-0.009693	-0.063859	0.009732	0.030237	0.044511
	-0.037378	0.023812	-0.015316	0.070133	-0.085755	-0.000090
	0.002394	-0.065811	-0.028676	0.015941	-0.021376	0.036942
	0.017475	0.033104	0.002905	-0.018114	0.072834	-0.018836
	-0.015614					
x129	-0.049771	0.065716	0.061011	-0.010757	0.042057	-0.015394
	0.029618	0.032205	0.002144	-0.038457	-0.083458	-0.017309
	0.090203	-0.072260	0.009108	-0.005609	0.151497	-0.036698
	0.112580	0.035004	-0.092512	0.001648	0.089313	-0.027413
	0.023639	0.065686	0.012525	0.026439	-0.080284	0.018956
	0.000098					
y129	-0.008834	-0.015667	0.035129	0.062728	-0.049004	0.028136
	-0.022970	-0.001812	0.033521	0.031968	0.067692	0.063580
	-0.101054	-0.020298	-0.015973	0.066154	-0.015345	-0.025463
	0.020789	-0.076833	0.054066	-0.030907	-0.028624	0.070249
	0.022652	-0.141099	-0.037893	0.110643	-0.025359	0.029711
	-0.054155					
z129	0.033817	0.101742	-0.095775	-0.031065	0.176682	-0.020686
	0.028597	0.006145	-0.036007	0.015442	-0.057149	-0.111434
	0.008042	0.043614	0.024468	-0.035317	0.150372	-0.019549
	0.084253	0.022203	0.024464	-0.007227	0.099291	-0.042590
	-0.058989	-0.024028	0.001493	-0.027907	0.044551	0.042233
	-0.019562					
x130	-0.049597	0.057177	0.060892	-0.005601	0.038403	-0.001360
	0.030708	0.028699	0.006898	-0.025329	-0.069564	0.011785
	0.095081	-0.066120	-0.002233	0.020683	0.120421	-0.050579
	0.089911	0.006090	-0.080150	0.011340	0.056018	-0.024192
	0.023624	0.057320	-0.022019	0.043150	-0.060699	-0.013104
	0.016386					

y130	-0.004846	-0.007852	0.033955	0.051020	-0.036109	0.027309
	-0.019771	-0.010913	0.029847	0.031269	0.069688	0.053802
	-0.101446	-0.021176	0.004741	0.052772	0.010459	-0.021210
	0.030092	-0.058260	0.042671	-0.043253	-0.025575	0.070973
	0.025919	-0.143799	-0.064640	0.103891	-0.030215	0.022463
	-0.040327					
z130	0.035426	0.089282	-0.086616	-0.031298	0.162471	-0.020278
	0.030457	0.008754	-0.037429	0.014271	-0.048506	-0.096471
	-0.000054	0.045261	0.017097	-0.031030	0.129151	-0.014404
	0.056079	0.010352	0.014008	0.009838	0.077975	-0.031761
	-0.053571	-0.019731	0.013880	-0.021206	0.036931	0.038261
	-0.027085					
x131	-0.048693	0.048654	0.062364	-0.002425	0.035367	0.004101
	0.031500	0.029341	0.009653	-0.010088	-0.055828	0.029072
	0.095306	-0.067027	-0.014165	0.039678	0.092068	-0.054051
	0.063127	-0.012373	-0.074311	0.032764	0.042391	-0.025993
	0.011269	0.067843	-0.042392	0.056742	-0.042335	-0.009851
	0.031170					
y131	-0.000415	-0.002115	0.036329	0.044004	-0.022866	0.017870
	-0.016956	-0.007343	0.024483	0.035252	0.072856	0.037948
	-0.101206	-0.032645	0.018962	0.040253	0.025803	-0.012194
	0.025576	-0.040666	0.025320	-0.041241	-0.007350	0.064207
	0.011891	-0.120295	-0.089040	0.097145	-0.025361	0.056111
	-0.021034					
z131	0.037150	0.077889	-0.080313	-0.026925	0.143411	-0.017084
	0.031052	0.006293	-0.033599	0.006137	-0.058873	-0.077140
	-0.000750	0.053837	0.005271	-0.015460	0.097364	-0.011515
	0.042058	-0.003427	0.015133	0.024114	0.053629	-0.017627
	-0.027993	-0.027386	0.025873	-0.034062	0.037535	0.009676
	-0.047087					
x132	-0.046587	0.041836	0.062047	-0.000170	0.030463	0.012878
	0.035492	0.023857	0.013198	0.000657	-0.047735	0.050112
	0.091567	-0.058071	-0.023881	0.056272	0.072001	-0.055793
	0.046182	-0.024375	-0.057028	0.047364	0.007665	-0.018943
	0.017513	0.075018	-0.062235	0.062795	-0.020766	-0.009602
	0.041192					
y132	0.003781	0.004685	0.039675	0.035548	-0.009204	0.014932
	-0.009071	-0.014693	0.024711	0.031379	0.072413	0.029202
	-0.100028	-0.026934	0.031039	0.032773	0.064782	-0.005486
	0.035567	-0.013263	0.015857	-0.035117	-0.004830	0.066780
	0.018432	-0.075200	-0.120308	0.086430	-0.026457	0.070961
	0.013579					

z132	0.038742	0.065257	-0.071986	-0.026602	0.126991	-0.015760
	0.033199	0.007273	-0.036125	0.007596	-0.050656	-0.060090
	-0.003306	0.052596	0.001447	-0.010738	0.074820	-0.009120
	0.019589	-0.012839	0.007129	0.032130	0.043757	-0.006543
	-0.022992	-0.027387	0.027103	-0.022706	0.021612	-0.012362
	-0.036236					
x133	-0.043025	0.035780	0.058777	-0.001848	0.028211	0.017986
	0.037534	0.018528	0.013714	0.009850	-0.045998	0.063893
	0.087469	-0.048929	-0.027769	0.064195	0.052447	-0.049761
	0.034100	-0.028956	-0.032296	0.057418	-0.016097	-0.020912
	0.014769	0.067231	-0.052096	0.070788	-0.014903	-0.030714
	0.020541					
y133	0.006584	0.007230	0.039862	0.031190	0.000195	0.011777
	-0.004712	-0.019166	0.030874	0.023361	0.054529	0.018802
	-0.089091	-0.014117	0.030477	0.036032	0.077730	0.005715
	0.049964	-0.000239	0.022019	-0.007449	0.011395	0.056722
	0.026369	-0.039670	-0.098168	0.081668	-0.045903	0.036298
	0.005373					
z133	0.039967	0.055738	-0.063935	-0.028470	0.110930	-0.012535
	0.037895	0.006733	-0.038245	0.010688	-0.035576	-0.044491
	-0.009493	0.048303	0.003287	-0.011977	0.067030	-0.010961
	-0.000443	-0.015411	-0.001809	0.032113	0.037467	0.005787
	-0.016203	-0.025511	0.011945	-0.009219	0.009457	-0.022349
	-0.013119					
x134	-0.039790	0.029957	0.060080	-0.005066	0.021560	0.017575
	0.039581	0.020126	0.015450	0.010919	-0.042313	0.070013
	0.081918	-0.054664	-0.020942	0.064217	0.024758	-0.059859
	0.017894	-0.036599	-0.020498	0.059799	-0.029775	-0.023195
	0.013976	0.058211	-0.016348	0.067221	0.009497	-0.031167
	0.016447					
y134	0.010531	0.007271	0.047113	0.035401	0.005177	0.003911
	-0.005110	-0.010278	0.030792	0.012512	0.026644	0.007368
	-0.072365	-0.012215	0.033723	0.043438	0.054304	0.007497
	0.057252	-0.000414	0.019592	0.012811	0.028004	0.044426
	0.020902	0.003761	-0.073104	0.042579	-0.035015	0.053668
	-0.000985					
z134	0.040188	0.049502	-0.058383	-0.030365	0.097390	-0.010522
	0.039474	0.008788	-0.040419	0.013197	-0.026789	-0.031083
	-0.015823	0.046531	0.001804	-0.010175	0.059882	-0.009750
	-0.010301	-0.014895	-0.008004	0.031993	0.025349	0.013779
	-0.017652	-0.024374	-0.003259	-0.001307	0.003789	-0.017543
	0.000869					

x135	-0.034657	0.022582	0.059066	-0.008195	0.015214	0.014409
	0.043859	0.010962	0.013895	0.014536	-0.042538	0.074614
	0.065699	-0.062244	-0.016990	0.063704	-0.000816	-0.050916
	0.008575	-0.036045	-0.016280	0.064610	-0.040352	-0.024029
	0.007885	0.040471	0.030358	0.069476	0.032292	-0.053475
	0.008437					
y135	0.014247	0.003610	0.047924	0.038460	0.009608	-0.004489
	-0.006003	-0.005099	0.032178	0.010362	0.017895	-0.002766
	-0.064743	-0.017428	0.031979	0.054813	0.039729	0.021261
	0.073282	-0.000447	0.014796	0.023212	0.033178	0.030866
	-0.004844	0.025696	-0.035737	0.011594	-0.025813	0.035517
	-0.002956					
z135	0.038966	0.045615	-0.052557	-0.033908	0.085632	-0.007473
	0.040148	0.012357	-0.043564	0.015422	-0.018770	-0.020250
	-0.012253	0.051393	0.004360	-0.015689	0.055595	-0.008541
	-0.019228	-0.014871	-0.004803	0.022414	0.014614	0.023217
	-0.006301	-0.025395	-0.018744	0.010306	-0.010425	-0.007979
	0.009991					
x136	-0.028773	0.018325	0.053663	-0.012001	0.014956	0.017316
	0.050350	0.001883	0.007781	0.021535	-0.041917	0.080726
	0.057961	-0.062169	-0.013623	0.049509	-0.010538	-0.030120
	0.001628	-0.028071	-0.002681	0.053485	-0.048854	-0.015163
	0.016838	0.013017	0.043018	0.068952	0.035093	-0.056059
	-0.009642					
y136	0.016178	0.002250	0.045151	0.036851	0.019568	-0.004406
	-0.004468	-0.001721	0.032407	0.015553	0.029780	-0.004393
	-0.056553	-0.025617	0.029850	0.049157	0.043290	0.029057
	0.071763	-0.002324	0.010683	0.021727	0.036816	0.026698
	-0.012168	0.009216	-0.035066	0.008936	-0.041777	0.024089
	-0.003531					
z136	0.037649	0.042222	-0.046061	-0.035831	0.068456	-0.008100
	0.040000	0.010865	-0.042128	0.011992	-0.021726	-0.011268
	-0.013685	0.053576	0.005635	-0.013486	0.039787	-0.009459
	-0.023523	-0.015434	-0.005080	0.024297	0.005440	0.028285
	0.008708	-0.018777	-0.011100	0.010615	-0.006026	-0.017116
	0.014363					
x137	-0.021860	0.018517	0.046625	-0.016949	0.013689	0.022671
	0.054680	-0.008881	0.006901	0.023476	-0.046954	0.082201
	0.058266	-0.052823	-0.007257	0.033465	-0.009875	-0.019396
	0.002689	-0.014120	0.011704	0.030764	-0.057072	-0.010411
	0.047460	0.002625	0.047115	0.062307	0.044252	-0.066657
	-0.010521					

y137	0.020609	0.007206	0.044096	0.033906	0.027018	-0.004653
	-0.008857	-0.000620	0.028749	0.018653	0.028462	-0.010242
	-0.042152	-0.023717	0.032895	0.038305	0.050472	0.027207
	0.075949	0.003587	0.015011	0.021839	0.045996	0.003573
	-0.008876	0.038712	-0.045468	-0.001915	-0.050112	0.019052
	-0.001954					
z137	0.035027	0.038762	-0.039221	-0.039544	0.054492	-0.008773
	0.041874	0.011766	-0.040233	0.009039	-0.017031	-0.005287
	-0.017916	0.050434	0.007438	-0.012422	0.027506	-0.010217
	-0.027173	-0.016890	-0.008860	0.023173	-0.004788	0.035303
	0.011867	-0.023077	-0.000949	0.014880	-0.007965	-0.018507
	0.017169					
x138	-0.016759	0.017407	0.046593	-0.022149	0.016055	0.022317
	0.058519	-0.010840	0.008237	0.020550	-0.049465	0.081958
	0.056806	-0.043854	-0.011500	0.020211	-0.007743	-0.005737
	0.005372	-0.026764	0.006405	0.024661	-0.066138	-0.007293
	0.065077	0.008835	0.090121	0.071331	0.064239	-0.036625
	-0.007863					
y138	0.024983	0.009859	0.045846	0.036494	0.033108	-0.002753
	-0.010083	0.000228	0.030608	0.017612	0.028897	-0.004701
	-0.040075	-0.026760	0.029502	0.040916	0.047084	0.017575
	0.064523	0.021928	-0.005167	0.035324	0.019923	-0.017130
	-0.029212	0.028201	-0.026638	-0.008103	-0.042031	0.002452
	-0.027605					
z138	0.032501	0.038353	-0.033926	-0.044570	0.044000	-0.009865
	0.043159	0.009306	-0.043802	0.010702	-0.012637	-0.002629
	-0.017930	0.050722	0.012594	-0.016291	0.021023	-0.002599
	-0.026029	-0.023701	-0.004493	0.018507	0.001585	0.045656
	0.020848	-0.008954	-0.014900	0.026360	-0.018874	-0.003531
	0.035839					
x139	-0.009857	0.014738	0.054834	-0.024532	0.017727	0.024785
	0.052205	-0.012243	0.013840	0.019893	-0.044947	0.068855
	0.057670	-0.030339	-0.007781	-0.000755	-0.007010	-0.005203
	0.017892	-0.046811	0.019994	0.024136	-0.050697	-0.002336
	0.046525	-0.001102	0.078170	0.059355	0.052514	-0.004258
	-0.018748					
y139	0.028574	0.015014	0.048258	0.035200	0.042752	-0.003411
	-0.006879	-0.000708	0.028552	0.020313	0.028859	-0.005252
	-0.038570	-0.029551	0.037252	0.022947	0.048921	0.013295
	0.055341	0.030623	-0.005239	0.023561	0.012369	-0.038310
	-0.033715	0.040418	-0.014484	0.004504	-0.054680	0.006260
	0.019535					

z139	0.028281	0.039258	-0.026898	-0.045268	0.025610	-0.010939
	0.035443	0.006072	-0.044453	0.010355	-0.007666	-0.001233
	-0.015330	0.055947	0.007874	-0.012845	0.016721	-0.000607
	-0.019189	-0.022856	-0.004639	0.031435	0.004675	0.050785
	0.019794	-0.012190	-0.036305	0.016402	-0.017743	-0.005430
	0.009237					
x140	-0.003964	0.009516	0.058631	-0.026306	0.017115	0.026129
	0.048026	-0.018215	0.018889	0.021238	-0.050202	0.060751
	0.049783	-0.016894	-0.008334	-0.007980	-0.012553	-0.001101
	0.032594	-0.046354	0.023755	0.027363	-0.042463	0.006876
	0.035071	-0.025562	0.062668	0.032430	0.059875	-0.018154
	-0.054675					
y140	0.030265	0.018350	0.048156	0.029869	0.049649	-0.004120
	0.000357	-0.005955	0.027427	0.021639	0.025313	-0.011767
	-0.037147	-0.023293	0.032352	0.017550	0.044689	0.017791
	0.051571	0.024579	-0.018540	0.015618	-0.001124	-0.027915
	-0.032840	0.028219	-0.007361	0.003099	-0.052867	-0.017863
	0.002244					
z140	0.024160	0.040104	-0.018256	-0.047513	0.007747	-0.011437
	0.027403	0.004872	-0.042179	0.010186	0.002269	0.004200
	-0.015204	0.050675	0.014108	-0.016621	0.015957	-0.006926
	-0.018592	-0.009875	-0.002328	0.037764	0.008696	0.039826
	0.021778	-0.007935	-0.050420	0.016102	-0.019938	-0.004248
	0.029467					
x141	0.000594	0.004555	0.060685	-0.025458	0.018033	0.027155
	0.040365	-0.024426	0.024388	0.020031	-0.048919	0.048920
	0.041245	-0.000241	-0.000922	-0.011350	-0.016682	-0.010084
	0.045483	-0.048158	0.033931	0.028612	-0.033335	-0.002215
	0.032130	-0.038054	0.052603	0.016334	0.060893	-0.029887
	-0.068829					
y141	0.030895	0.019472	0.048668	0.025328	0.057716	-0.006365
	0.001117	-0.006086	0.027223	0.021960	0.033211	-0.015454
	-0.032045	-0.016192	0.037662	0.017205	0.037670	0.012002
	0.048862	0.016203	-0.016925	0.005624	0.004170	-0.050477
	-0.036974	0.016462	0.019660	0.004935	-0.045331	-0.024987
	-0.019138					
z141	0.020412	0.042887	-0.011697	-0.050673	-0.009577	-0.009426
	0.024081	0.001517	-0.043376	0.012701	0.000969	0.006501
	-0.017299	0.042128	0.008519	-0.024973	0.018763	-0.000122
	-0.017631	0.006035	-0.006342	0.045756	0.004099	0.049147
	0.027502	-0.001403	-0.076034	0.008533	-0.037682	-0.007643
	0.054563					

x142	0.003779	0.000502	0.061218	-0.028728	0.018686	0.028822
	0.036148	-0.027171	0.027474	0.024046	-0.040273	0.045588
	0.032475	0.006689	0.011398	-0.027080	-0.012156	-0.011755
	0.051502	-0.044420	0.028612	0.025077	-0.022740	-0.000309
	0.013860	-0.035513	0.033046	0.018908	0.054993	-0.027749
	-0.064487					
y142	0.030500	0.016976	0.050644	0.021807	0.064291	-0.009163
	0.006906	0.001669	0.025366	0.022594	0.038518	-0.017709
	-0.031847	-0.011968	0.040872	0.014097	0.021361	0.005633
	0.038314	0.010965	-0.013663	0.003051	0.007044	-0.049522
	-0.054963	0.013993	0.018702	0.016864	-0.039854	-0.007130
	-0.037087					
z142	0.017598	0.048363	-0.008218	-0.049680	-0.030529	-0.008647
	0.016069	-0.008136	-0.040483	0.007753	-0.013829	0.003572
	-0.010452	0.042157	0.000211	-0.014328	0.012987	0.007793
	-0.003874	0.007576	0.001059	0.059678	-0.003274	0.046472
	0.054563	-0.009784	-0.061925	-0.026273	-0.032554	-0.030739
	0.053312					
x143	0.004514	-0.001737	0.061320	-0.031762	0.019420	0.031283
	0.033620	-0.032128	0.026291	0.026261	-0.032450	0.040468
	0.019746	0.008935	0.019192	-0.037445	-0.007126	-0.013708
	0.047477	-0.023800	0.027709	0.015326	-0.015443	0.001707
	0.001201	-0.035691	0.021153	0.014483	0.046612	-0.036460
	-0.038834					
y143	0.028302	0.018521	0.053249	0.016521	0.065285	-0.006515
	0.012845	-0.004423	0.023354	0.016322	0.043078	-0.018993
	-0.033670	-0.008263	0.033195	0.013353	0.011677	0.000528
	0.021475	0.016355	-0.028314	-0.004097	0.011867	-0.044982
	-0.053530	0.011412	0.019812	0.024573	-0.044777	-0.019572
	-0.004655					
z143	0.016066	0.049401	-0.007191	-0.045263	-0.047528	-0.011819
	0.006885	-0.006259	-0.036009	0.006461	-0.030522	-0.001988
	0.001127	0.045260	-0.002942	-0.006573	-0.001946	0.014711
	0.014126	-0.004492	0.022872	0.073486	-0.011519	0.037638
	0.062334	-0.017906	-0.045818	-0.059870	-0.013351	-0.023826
	-0.003028					
x144	0.004625	-0.003370	0.058896	-0.031951	0.018148	0.032671
	0.029315	-0.039706	0.027936	0.029852	-0.032177	0.035845
	0.012571	0.019158	0.025761	-0.035714	-0.004328	-0.010602
	0.055870	-0.010557	0.027269	0.010425	-0.010843	0.002097
	-0.006777	-0.029946	0.020076	-0.005941	0.042255	-0.045967
	-0.029926					

y144	0.025389	0.022157	0.055408	0.012502	0.066194	-0.004524
	0.018581	-0.013293	0.022453	0.015495	0.042890	-0.018946
	-0.029380	0.004338	0.027840	0.026306	0.002816	-0.000946
	0.006827	0.020126	-0.021618	0.000778	0.010966	-0.037359
	-0.045674	-0.003900	0.021304	0.014764	-0.035401	-0.027402
	0.010510					
z144	0.013582	0.047482	-0.004162	-0.046372	-0.059101	-0.017515
	-0.000925	0.008862	-0.035529	0.005787	-0.026754	-0.015009
	0.000989	0.028759	0.000263	-0.021267	-0.012184	0.016380
	0.026729	-0.015238	0.016818	0.069810	-0.008233	0.019157
	0.040899	0.010309	-0.022765	-0.059806	-0.015715	0.007339
	-0.028488					
x145	0.003579	-0.005865	0.054061	-0.033495	0.018443	0.033481
	0.022212	-0.045878	0.027955	0.030597	-0.035412	0.032945
	0.005668	0.025282	0.020283	-0.038651	0.006252	-0.013616
	0.063229	0.007586	0.022089	0.006656	-0.012673	-0.007837
	-0.014933	-0.017962	0.028611	-0.025875	0.040929	-0.049840
	-0.003289					
y145	0.021371	0.023463	0.058360	0.005790	0.069483	-0.008667
	0.022068	-0.009939	0.016363	0.013799	0.040445	-0.032409
	-0.032426	0.008707	0.008495	0.020019	-0.009762	-0.007360
	-0.005659	0.015769	-0.012163	0.007815	0.014941	-0.047840
	-0.045853	0.020105	0.034741	-0.011174	-0.035265	-0.009424
	0.041159					
z145	0.011829	0.050026	-0.004839	-0.042924	-0.076730	-0.013906
	-0.003839	0.004475	-0.031375	0.007073	-0.025803	-0.008930
	0.007961	0.018627	0.020709	-0.015608	-0.013719	0.028602
	0.035639	-0.004645	0.012213	0.060049	-0.007620	0.030068
	0.041220	-0.006799	-0.037149	-0.037465	-0.020578	-0.008865
	-0.044565					
x146	0.003709	-0.008084	0.046863	-0.031305	0.014241	0.031708
	0.019890	-0.051085	0.028967	0.035724	-0.033705	0.026639
	0.007962	0.027640	0.036182	-0.047404	-0.001084	-0.014887
	0.065937	0.007366	0.021844	-0.003340	-0.001503	-0.007392
	-0.013390	-0.007069	0.027524	-0.039391	0.051480	-0.045103
	0.012036					
y146	0.018640	0.024079	0.057477	0.004251	0.068037	-0.010660
	0.032427	-0.009012	0.012990	0.018379	0.040791	-0.039686
	-0.029428	0.012571	0.004046	0.012935	-0.024952	-0.032343
	-0.033707	0.015583	0.031148	-0.000572	0.016169	-0.047070
	-0.011778	-0.005692	0.006813	-0.008383	-0.016798	-0.004347
	0.055514					

z146	0.008004	0.051924	-0.000533	-0.044084	-0.084590	-0.016573
	-0.009843	0.004760	-0.036646	0.004492	-0.029075	-0.008937
	0.007123	0.010594	0.021783	-0.007905	-0.013808	0.052269
	0.045151	0.009545	-0.000473	0.062615	-0.002354	0.028760
	0.021770	0.018447	-0.021568	-0.031926	-0.037727	-0.005160
	-0.039897					
x147	0.004195	-0.013008	0.041187	-0.028719	0.010939	0.028501
	0.017505	-0.052303	0.030039	0.042408	-0.027705	0.021605
	0.005613	0.018505	0.050286	-0.048440	-0.005711	-0.012892
	0.053723	0.011845	0.029118	-0.027045	-0.003412	-0.003900
	-0.018464	-0.015642	-0.001901	-0.023949	0.034837	-0.025642
	0.036163					
y147	0.015778	0.027651	0.056203	0.001628	0.065873	-0.008075
	0.035402	-0.008717	0.014758	0.020272	0.044632	-0.038075
	-0.025525	0.013373	0.006982	0.016719	-0.034968	-0.027902
	-0.040341	0.013368	0.030392	-0.004563	0.009583	-0.044838
	-0.008565	-0.011422	0.006013	-0.004306	-0.017059	-0.008999
	0.046030					
z147	0.007524	0.051415	0.001048	-0.043011	-0.099054	-0.016806
	-0.012403	0.001737	-0.034756	-0.002758	-0.036329	-0.019421
	0.009341	-0.000370	0.013981	-0.010529	-0.019214	0.037251
	0.047783	0.010434	-0.001816	0.058469	0.004211	0.019620
	0.025366	0.017462	-0.019452	-0.040184	-0.030958	-0.006309
	-0.005958					
x148	0.003861	-0.018721	0.038676	-0.027589	0.008047	0.028207
	0.005715	-0.059644	0.031357	0.047722	-0.018272	0.016799
	0.001668	0.006889	0.047554	-0.047024	0.009923	-0.010643
	0.051453	-0.003453	0.020056	-0.041720	-0.008226	-0.009479
	-0.048710	-0.022422	-0.027735	-0.002614	0.003003	0.008556
	0.046885					
y148	0.014014	0.030217	0.055441	-0.001874	0.060550	-0.003996
	0.035577	-0.012331	0.014099	0.020095	0.045550	-0.037501
	-0.020227	0.011676	0.002678	0.020576	-0.034504	-0.025425
	-0.035674	0.007630	0.024635	-0.011921	0.005134	-0.043568
	-0.003012	-0.009148	0.003575	0.005130	-0.025379	-0.003190
	0.054758					
z148	0.005098	0.049867	0.001753	-0.038192	-0.109958	-0.020220
	-0.016624	0.007868	-0.026060	-0.008052	-0.036588	-0.029280
	0.009875	-0.013059	0.017906	-0.012713	-0.027714	0.013770
	0.042380	0.011860	0.004285	0.061323	-0.000075	-0.000601
	0.009686	0.000449	-0.012577	-0.044222	-0.003239	-0.008306
	-0.024072					

x149	0.003953	-0.021534	0.034107	-0.028641	0.010046	0.027806
	-0.005725	-0.069248	0.027935	0.048307	-0.014927	0.014596
	0.000695	0.004254	0.047811	-0.054782	0.017894	-0.015901
	0.050823	-0.010961	0.013372	-0.046316	-0.015579	-0.018159
	-0.059496	-0.020317	-0.036017	-0.001195	-0.000254	0.024040
	0.024677					
y149	0.012055	0.033060	0.054614	-0.006312	0.055512	-0.004143
	0.035848	-0.014662	0.009467	0.018229	0.044501	-0.042020
	-0.014244	0.008800	0.001964	0.014553	-0.037868	-0.027971
	-0.033342	0.005409	0.022851	-0.019926	0.003745	-0.047714
	0.000231	-0.004150	0.005351	0.007103	-0.019526	0.003818
	0.039421					
z149	0.003326	0.050135	0.003248	-0.033328	-0.117995	-0.012250
	-0.021689	0.005456	-0.012994	-0.004594	-0.031673	-0.021761
	0.006931	-0.018826	0.018213	0.004290	-0.028489	0.015004
	0.039366	0.005446	-0.001554	0.059008	-0.007569	-0.002608
	0.007434	-0.020294	-0.016866	-0.036207	-0.012172	-0.033474
	0.006952					
x150	0.003227	-0.022732	0.033979	-0.027510	0.014583	0.023610
	-0.011987	-0.075107	0.026152	0.050018	-0.017962	0.015208
	0.005032	0.012869	0.046731	-0.060484	0.019018	-0.015568
	0.048523	-0.011974	0.020066	-0.030435	-0.014316	-0.015985
	-0.064634	-0.024749	-0.043807	-0.009843	0.008973	0.028704
	0.013754					
y150	0.010449	0.036136	0.053921	-0.008589	0.049866	-0.003232
	0.038079	-0.016732	0.007719	0.016827	0.040942	-0.044238
	-0.009338	0.009650	0.001473	0.014821	-0.040115	-0.029597
	-0.031973	0.006524	0.026753	-0.017800	0.001559	-0.048120
	0.004254	-0.001387	0.002490	0.004411	-0.011994	0.000049
	0.038881					
z150	0.000195	0.045469	0.003913	-0.033152	-0.123779	-0.009517
	-0.031624	0.012852	-0.012488	-0.004526	-0.021294	-0.028153
	0.006711	-0.036979	0.019480	0.011052	-0.029175	0.017307
	0.037133	-0.012078	-0.017626	0.035034	-0.002973	-0.016588
	-0.016982	-0.018440	0.005233	-0.021439	-0.018477	-0.009097
	-0.002411					
x151	0.001233	-0.024107	0.036991	-0.027374	0.016441	0.019074
	-0.020844	-0.080605	0.023102	0.049372	-0.017657	0.018856
	0.014426	0.015915	0.049982	-0.063103	0.008689	-0.015121
	0.039914	-0.018050	0.014204	-0.027311	-0.012411	-0.016808
	-0.050729	-0.038686	-0.028181	-0.020647	0.000769	0.037901
	0.018501					

y151	0.008627	0.038975	0.052532	-0.011162	0.042687	-0.002248
	0.039382	-0.017707	0.006881	0.015146	0.039317	-0.047189
	-0.004108	0.008299	0.000090	0.017426	-0.044984	-0.028672
	-0.031038	0.004448	0.026836	-0.020601	-0.000965	-0.048593
	0.004206	-0.001838	0.005712	0.001444	-0.005380	0.000670
	0.035785					
z151	0.000589	0.045314	-0.000594	-0.032109	-0.127592	-0.001620
	-0.037888	0.007266	-0.012630	-0.001802	-0.021513	-0.027365
	0.001368	-0.043005	0.019060	0.014059	-0.010412	0.013481
	0.042565	-0.009486	-0.021760	0.015210	-0.005974	-0.016497
	-0.026068	-0.012388	-0.027579	-0.000395	-0.018400	-0.040283
	0.006790					
x152	0.001750	-0.025284	0.033182	-0.027473	0.018352	0.013703
	-0.027242	-0.085547	0.019231	0.050036	-0.017148	0.021370
	0.021598	0.019899	0.045698	-0.063461	0.001585	-0.017418
	0.025652	-0.029081	0.003944	-0.034542	-0.015057	-0.015011
	-0.050058	-0.037801	-0.010129	-0.026104	0.006654	0.054487
	0.019787					
y152	0.007259	0.042555	0.051150	-0.013609	0.035243	-0.001877
	0.040956	-0.019878	0.005260	0.013422	0.035939	-0.047004
	0.001842	0.009406	-0.000735	0.017226	-0.048567	-0.028415
	-0.031740	0.000070	0.023394	-0.026901	-0.001248	-0.047275
	0.003713	-0.000279	0.010359	0.001246	-0.002707	0.000713
	0.031067					
z152	-0.002099	0.045512	0.001858	-0.031152	-0.127001	0.008561
	-0.043401	0.006334	-0.009239	-0.001125	-0.016508	-0.028221
	-0.002542	-0.046549	0.018853	0.022174	-0.003862	0.014717
	0.038439	-0.006657	-0.016567	0.009170	-0.011508	-0.017454
	-0.023389	-0.026578	-0.050143	0.010117	-0.029028	-0.065661
	0.010997					
x153	0.002623	-0.025691	0.029144	-0.025127	0.021453	0.010456
	-0.032485	-0.088676	0.013804	0.050564	-0.016863	0.027250
	0.030310	0.019494	0.033869	-0.061570	-0.007823	-0.010312
	0.016263	-0.031874	0.000438	-0.033375	-0.019896	-0.004306
	-0.050710	-0.042590	-0.004492	-0.028728	-0.009488	0.067163
	0.012765					
y153	0.005875	0.045946	0.050225	-0.015531	0.027898	-0.001280
	0.042325	-0.019958	0.003891	0.010833	0.033151	-0.046147
	0.007295	0.009319	-0.002138	0.018427	-0.054365	-0.029551
	-0.032074	-0.006686	0.017983	-0.028506	-0.000562	-0.046552
	0.000050	0.003305	0.022396	0.002625	0.002537	0.002670
	0.026117					

z153	-0.007029	0.044428	0.007069	-0.034069	-0.124052	0.012953
	-0.050391	0.013687	-0.004161	-0.000262	-0.006511	-0.038754
	-0.011956	-0.050387	0.019918	0.029174	-0.005475	0.015417
	0.027829	-0.020662	-0.021026	-0.002505	-0.011996	-0.035670
	-0.035128	-0.014883	-0.024522	0.008449	-0.007842	-0.049272
	0.010444					
x154	0.002234	-0.029614	0.020438	-0.022519	0.020302	0.012379
	-0.041915	-0.085702	0.021386	0.056432	-0.004652	0.024434
	0.031075	0.006042	0.027432	-0.062518	-0.005496	-0.005637
	-0.001067	-0.040278	-0.000944	-0.032348	-0.028248	-0.002213
	-0.055080	-0.043057	-0.025760	-0.027257	-0.029514	0.080916
	0.021050					
y154	0.006423	0.048786	0.045883	-0.014951	0.018511	0.001169
	0.043335	-0.021855	0.003948	0.009121	0.027634	-0.046989
	0.010773	0.006264	-0.005021	0.025643	-0.058799	-0.031051
	-0.025935	-0.008918	0.014428	-0.035786	-0.005510	-0.048464
	-0.007493	0.004882	0.021657	0.002697	0.008781	0.009183
	0.019281					
z154	-0.007706	0.046656	0.015451	-0.032537	-0.121326	0.014516
	-0.051060	0.014547	-0.012671	-0.007152	-0.015739	-0.039084
	-0.006033	-0.047828	0.021726	0.039193	-0.020103	0.017244
	0.035966	-0.020011	-0.030545	-0.018211	-0.010111	-0.051398
	-0.044527	-0.014926	0.022622	0.011347	0.023588	-0.024050
	-0.025361					
x155	0.002495	-0.028368	0.015387	-0.018508	0.021253	0.012655
	-0.052974	-0.086543	0.020333	0.050084	-0.005002	0.020764
	0.029488	0.008103	0.015143	-0.043991	-0.003425	0.004870
	-0.014570	-0.035745	-0.003416	-0.043001	-0.041804	0.000739
	-0.051017	-0.034519	-0.007465	-0.014826	-0.022551	0.098893
	0.003073					
y155	0.006103	0.052765	0.041230	-0.013230	0.005152	0.004786
	0.050577	-0.025429	0.005953	0.011301	0.032501	-0.043353
	0.019508	-0.002724	-0.000067	0.015921	-0.076257	-0.028347
	-0.027741	-0.016980	0.005495	-0.031409	0.003648	-0.039133
	-0.002271	0.005746	0.012293	-0.005601	0.002380	0.003502
	0.028518					
z155	-0.009735	0.044791	0.019994	-0.033293	-0.118899	0.019730
	-0.043059	0.014898	-0.009455	-0.004750	-0.002438	-0.034139
	0.000310	-0.063619	0.029316	0.026859	-0.043822	0.016319
	0.038435	-0.042702	-0.050191	-0.007668	-0.001755	-0.046622
	-0.051706	-0.011295	0.033525	-0.013254	0.003434	-0.034180
	-0.019496					

x156	0.005189	-0.027646	0.012557	-0.012242	0.023739	0.009388
	-0.054561	-0.079430	0.024514	0.051782	0.012386	0.004852
	0.035334	0.002297	0.020514	-0.042090	-0.003953	0.009252
	-0.022240	-0.038233	-0.017043	-0.036238	-0.048372	-0.011921
	-0.074652	-0.013772	0.006901	0.008365	-0.045353	0.106898
	-0.013897					
y156	0.005815	0.055360	0.037547	-0.015686	-0.001737	0.005843
	0.050733	-0.030485	0.007702	0.011225	0.026529	-0.040854
	0.021902	0.005621	-0.005886	0.021265	-0.074863	-0.029892
	-0.028626	-0.021052	-0.000783	-0.036060	-0.001301	-0.027620
	-0.002196	0.004626	0.016040	-0.013530	-0.000359	0.004208
	0.025037					
z156	-0.008437	0.042996	0.023929	-0.038114	-0.111201	0.021841
	-0.039941	0.003843	-0.003178	-0.003885	-0.005133	-0.035962
	-0.002756	-0.042112	0.031246	0.042409	-0.037445	0.005911
	0.030407	-0.054562	-0.056316	-0.016002	-0.018889	-0.026405
	-0.035473	-0.020512	0.034123	-0.027967	-0.005516	-0.044431
	-0.030772					
x157	0.004574	-0.024260	0.006017	-0.008854	0.026039	0.013664
	-0.065082	-0.074485	0.031191	0.041864	0.010941	0.001205
	0.024330	-0.000718	0.021451	-0.013835	0.017474	-0.001189
	-0.024880	-0.056775	-0.038491	-0.034590	-0.049746	-0.006987
	-0.074299	-0.019186	0.013913	0.015751	-0.056566	0.062598
	-0.014788					
y157	0.006884	0.057467	0.035422	-0.015114	-0.009860	0.007356
	0.049656	-0.030008	0.009385	0.010189	0.022880	-0.040996
	0.025676	0.008869	-0.010768	0.019182	-0.073644	-0.023979
	-0.025986	-0.024483	-0.006437	-0.040388	0.001522	-0.024589
	-0.016856	0.012142	0.024817	-0.006811	0.004767	0.011198
	0.030397					
z157	-0.007680	0.036578	0.034062	-0.039754	-0.094320	0.019362
	-0.043987	0.008051	0.002117	-0.009445	-0.007391	-0.037252
	-0.006083	-0.017309	0.023876	0.050399	-0.022894	0.006645
	0.029598	-0.060064	-0.059131	-0.015469	-0.030499	-0.027054
	-0.057361	-0.010463	0.068293	-0.015788	-0.008100	-0.028508
	-0.027099					
x158	0.000114	-0.022113	-0.004913	-0.003590	0.026611	0.014643
	-0.075169	-0.064035	0.032017	0.031085	0.003766	0.001879
	0.022854	-0.009854	0.017725	0.003312	0.016296	-0.006326
	-0.032666	-0.064285	-0.044932	-0.022322	-0.040405	-0.002143
	-0.063900	-0.009553	0.039876	0.039465	-0.035572	0.047271
	-0.013820					

y158	0.008138	0.060683	0.033053	-0.013320	-0.020622	0.013547
	0.049910	-0.035609	0.006243	0.000507	0.017500	-0.030338
	0.027036	0.004408	-0.016417	0.008156	-0.059519	-0.021073
	-0.013957	-0.020147	-0.010958	-0.042161	0.008754	-0.016397
	-0.022270	0.011804	0.022477	-0.006115	0.001942	-0.009152
	0.029314					
z158	-0.005550	0.031219	0.042009	-0.037132	-0.084826	0.027161
	-0.037447	0.006915	0.001139	-0.018114	-0.001607	-0.024278
	-0.007391	-0.015588	0.024331	0.035597	-0.011396	0.009278
	0.033835	-0.062835	-0.059471	-0.008371	-0.025328	-0.024883
	-0.060358	-0.019372	0.056784	-0.027598	-0.028373	-0.038906
	-0.013599					
x159	-0.003686	-0.015779	-0.011656	-0.000049	0.019520	0.015565
	-0.074588	-0.058828	0.031759	0.017618	-0.010589	0.016214
	0.020202	-0.020627	-0.006251	0.010478	0.017655	-0.022993
	-0.028786	-0.044611	-0.045639	-0.003031	-0.025264	0.007856
	-0.042604	-0.032805	0.058537	0.032877	-0.033373	0.013988
	0.007673					
y159	0.006648	0.059107	0.031631	-0.011369	-0.024767	0.016873
	0.044249	-0.031866	0.002701	-0.011599	0.008728	-0.030135
	0.028454	-0.002790	-0.028215	0.000193	-0.045536	-0.012902
	-0.006428	-0.023471	-0.014143	-0.048893	0.011170	-0.012502
	-0.024578	0.022681	0.035128	0.001597	0.019931	-0.001216
	0.019976					
z159	-0.006441	0.020566	0.050314	-0.034867	-0.064492	0.029641
	-0.038307	0.015374	-0.003397	-0.027586	0.002976	-0.026377
	-0.010228	-0.012858	0.023632	0.029703	0.002829	0.016829
	0.030698	-0.070606	-0.052477	-0.008809	-0.030005	-0.028040
	-0.066343	-0.011827	0.057519	-0.016633	-0.018069	-0.020229
	-0.006181					
x160	-0.002568	-0.011078	-0.018683	0.002471	0.013547	0.013042
	-0.072167	-0.055995	0.028992	0.015031	-0.016474	0.014224
	0.025929	-0.019282	-0.013914	0.019755	0.029153	-0.037353
	-0.036502	-0.039755	-0.042475	-0.000772	-0.021002	0.010582
	-0.045194	-0.036772	0.065873	0.021127	-0.043889	0.003858
	0.013862					
y160	0.006187	0.056674	0.030656	-0.008360	-0.028182	0.020191
	0.042036	-0.028078	-0.001932	-0.024498	0.000491	-0.024922
	0.025634	-0.011031	-0.036148	-0.005755	-0.037246	-0.012809
	0.004330	-0.011737	-0.011154	-0.045795	0.011441	0.001613
	-0.007486	0.014187	0.021394	0.020052	0.030625	-0.009015
	0.009125					

z160	-0.007629	0.008637	0.060389	-0.029711	-0.044827	0.032651
	-0.035843	0.025203	-0.009161	-0.040126	0.005517	-0.024650
	-0.012904	-0.014151	0.022496	0.021953	0.003849	0.020983
	0.031249	-0.059927	-0.037223	0.005102	-0.031956	-0.025426
	-0.047741	-0.022306	0.043377	-0.009189	-0.004993	-0.011330
	-0.001086					
x161	0.001054	-0.004868	-0.029332	0.005027	0.010214	0.013871
	-0.065977	-0.053097	0.021110	0.011213	-0.027279	0.009710
	0.024709	-0.018777	-0.003197	0.045712	0.030444	-0.046867
	-0.053862	-0.027289	-0.035510	-0.009084	-0.000611	0.032573
	-0.042329	-0.045207	0.077763	-0.008845	-0.071567	-0.000867
	0.028391					
y161	0.004989	0.052111	0.032088	-0.007154	-0.032596	0.019959
	0.041704	-0.022944	-0.002447	-0.034376	-0.001550	-0.026361
	0.025418	-0.017600	-0.049540	-0.015225	-0.023078	-0.007021
	0.017702	-0.003641	-0.007613	-0.049570	0.009999	0.012214
	0.006211	0.015598	0.021151	0.033010	0.051567	-0.027280
	-0.005064					
z161	-0.009269	-0.002551	0.070136	-0.028517	-0.026635	0.031736
	-0.028324	0.035496	-0.014689	-0.051648	0.008230	-0.023742
	-0.014190	-0.013251	0.024114	0.021071	0.003942	0.031410
	0.027092	-0.049881	-0.021897	0.007493	-0.022999	-0.010177
	-0.031365	-0.029402	0.048654	-0.019288	-0.001879	-0.012963
	0.008529					
x162	0.002992	0.003669	-0.038899	0.006190	-0.000207	0.004800
	-0.049620	-0.047930	0.015453	0.007912	-0.032065	0.014777
	0.025138	-0.010776	-0.000094	0.080583	0.032455	-0.057500
	-0.068365	-0.027075	-0.025434	-0.033513	0.010683	0.057496
	-0.053014	-0.037274	0.096122	-0.033895	-0.107567	0.005240
	0.028686					
y162	0.007488	0.045623	0.033245	-0.004408	-0.031685	0.023639
	0.040742	-0.020037	-0.006556	-0.039419	-0.009535	-0.026564
	0.024316	-0.022280	-0.051039	-0.031351	-0.008646	-0.003855
	0.033511	0.018582	-0.014736	-0.040357	0.011365	0.021453
	0.019478	0.005739	0.003063	0.037802	0.066081	-0.049381
	-0.014415					
z162	-0.009226	-0.012043	0.078103	-0.025468	-0.006955	0.035025
	-0.023227	0.040326	-0.020281	-0.060475	0.006959	-0.027654
	-0.013074	-0.012828	0.034558	0.018959	0.002964	0.041044
	0.027232	-0.022699	-0.004900	0.015368	-0.018301	0.006269
	-0.013329	-0.044672	0.043578	-0.028372	0.010648	-0.024837
	0.029106					

x163	0.003378	0.011175	-0.051743	0.010061	-0.010334	-0.001060
	-0.034370	-0.054374	0.014672	-0.002370	-0.028683	0.022573
	0.037305	-0.002711	-0.010466	0.091864	0.040616	-0.077727
	-0.056177	-0.021012	-0.015296	-0.033140	-0.027297	0.041053
	-0.041942	-0.022930	0.064904	-0.040667	-0.111085	0.020988
	0.023992					
y163	0.009727	0.036284	0.041544	-0.000355	-0.027472	0.020099
	0.038189	-0.000898	-0.017133	-0.038545	-0.017590	-0.039743
	0.021300	-0.033407	-0.051683	-0.045050	-0.003395	0.014502
	0.036952	0.030757	-0.012496	-0.036328	0.029889	0.031536
	0.006475	0.003949	0.019425	0.035002	0.077400	-0.030616
	-0.029618					
z163	-0.008200	-0.020177	0.081240	-0.021537	0.005560	0.032027
	-0.016311	0.042421	-0.026194	-0.060672	0.005372	-0.021179
	0.003698	-0.002346	0.036658	0.011783	0.020323	0.040274
	0.023773	-0.008111	0.017336	0.025603	-0.033372	0.010055
	-0.004006	-0.057225	0.023883	-0.030125	-0.001169	0.011094
	0.031340					
x164	0.004544	0.017128	-0.055643	0.021830	-0.015891	-0.004360
	-0.032166	-0.046279	0.005275	-0.010197	-0.020734	0.008907
	0.045366	0.010166	-0.010193	0.087463	0.047218	-0.090718
	-0.052174	-0.005514	0.002371	-0.014354	-0.053999	0.006420
	-0.028677	0.002526	0.040413	-0.052737	-0.065731	0.060270
	-0.019345					
y164	0.012448	0.026628	0.046137	-0.001127	-0.026418	0.018637
	0.045725	0.002109	-0.026806	-0.033894	-0.025018	-0.038122
	0.020014	-0.047814	-0.059019	-0.051573	0.015374	0.035173
	0.042188	0.046633	-0.024465	-0.045748	0.038132	0.067968
	0.015968	-0.013464	0.016789	0.023976	0.049041	-0.063597
	-0.007388					
z164	-0.007458	-0.023760	0.076312	-0.015419	0.008333	0.028191
	-0.005649	0.032972	-0.032510	-0.052951	0.005530	-0.005167
	0.029287	0.017320	0.040447	0.019140	0.060459	0.030984
	0.009090	0.014897	0.037133	0.020518	-0.052167	0.034683
	0.018821	-0.055315	0.007945	-0.036233	-0.033479	0.037547
	0.026440					
x165	0.002952	0.022854	-0.059426	0.031430	-0.017960	-0.008463
	-0.019286	-0.027084	-0.007996	-0.009900	-0.024788	-0.005144
	0.045323	0.016032	-0.019532	0.084160	0.051310	-0.085754
	-0.056085	0.046639	0.007100	0.007216	-0.065545	0.005161
	-0.025773	0.006991	0.015678	-0.073851	-0.038546	0.023145
	-0.043791					

y165	0.016589	0.019315	0.048503	-0.003008	-0.027677	0.013066
	0.051468	-0.000076	-0.037409	-0.029677	-0.028376	-0.037053
	0.017235	-0.051286	-0.045323	-0.046112	0.027927	0.050791
	0.047842	0.044722	-0.035103	-0.072035	0.043417	0.099312
	0.006473	-0.006887	0.042797	-0.000410	0.033065	-0.043252
	0.014238					
z165	-0.007060	-0.020765	0.065808	-0.006925	0.006638	0.021299
	0.005274	0.019828	-0.040834	-0.042268	0.006951	0.012519
	0.059829	0.042725	0.058802	0.034627	0.106011	0.021076
	-0.012451	0.047621	0.060293	0.013834	-0.074872	0.061510
	0.038740	-0.038453	-0.001504	-0.038991	-0.067238	0.095872
	0.012275					
x166	0.060511	0.016132	0.009939	0.004848	0.034299	-0.030266
	-0.016437	-0.020747	-0.014012	-0.095464	-0.009882	0.079423
	-0.006053	0.020612	0.023431	0.048974	-0.005699	0.073791
	0.026563	-0.015578	0.024690	-0.018276	0.053977	-0.077631
	0.021995	0.076520	-0.025160	-0.003974	-0.038650	-0.003248
	-0.010423					
y166	0.022991	0.007375	-0.019480	0.069706	-0.046271	0.050465
	0.028147	0.026326	-0.006654	-0.012149	0.010638	-0.041211
	0.057244	-0.039093	0.008212	-0.023550	-0.060426	-0.060601
	0.025952	-0.027687	-0.040122	-0.004837	0.076485	0.028396
	0.051834	0.045122	0.009471	-0.087459	0.050219	0.004920
	0.042695					
z166	0.020278	0.036597	0.026969	-0.034115	0.011907	0.033354
	-0.037218	-0.005379	0.023719	-0.005333	-0.003764	0.009414
	0.050714	-0.005153	-0.009528	0.001795	0.050601	-0.020538
	-0.028003	0.007589	0.034603	-0.010055	-0.043304	0.000435
	0.079116	0.082640	-0.086347	-0.010652	0.070662	0.053616
	0.014940					
x167	0.063573	0.017676	0.011756	0.009896	0.023978	-0.034099
	-0.019150	-0.016038	-0.002897	-0.080642	-0.005168	0.067798
	-0.006898	0.028074	0.026018	0.030536	-0.012814	0.056970
	0.000793	-0.028889	0.038066	-0.027668	0.056764	-0.078076
	0.018614	0.072213	0.003048	-0.009519	-0.028655	0.037067
	-0.013297					
y167	0.020258	0.001474	-0.023279	0.066913	-0.049018	0.049719
	0.032569	0.016232	-0.018887	-0.010173	0.016140	-0.028524
	0.047064	-0.031396	0.019160	-0.014605	-0.045526	-0.047763
	0.017174	-0.035830	-0.018338	-0.000541	0.064054	0.016991
	0.039439	0.020914	0.012322	-0.055744	0.039279	0.005935
	0.022946					

z167	0.018002	0.030204	0.028063	-0.034633	0.010163	0.039936
	-0.036033	0.001069	0.004565	-0.008596	-0.001519	0.022439
	0.043476	-0.018715	-0.023714	-0.000152	0.047204	-0.011497
	-0.022359	0.000474	0.040526	-0.000081	-0.035352	0.013267
	0.059324	0.044946	-0.076749	0.005353	0.019937	0.034148
	-0.008095					
x168	0.066796	0.016624	0.014608	0.016569	0.017431	-0.032574
	-0.018863	-0.015751	0.004130	-0.063746	-0.004160	0.062362
	-0.003291	0.030786	0.019369	0.007697	-0.015924	0.048378
	-0.007255	-0.026623	0.047196	-0.027185	0.036384	-0.067569
	0.008846	0.074083	0.000092	0.000102	-0.031000	0.067658
	-0.025772					
y168	0.017453	-0.004501	-0.026084	0.061883	-0.050082	0.046977
	0.035815	0.009016	-0.027526	-0.010637	0.021255	-0.016241
	0.037846	-0.028096	0.025863	-0.006508	-0.033615	-0.036494
	0.014869	-0.037244	-0.005754	0.003114	0.052873	0.012370
	0.030048	0.000592	0.015302	-0.026649	0.025782	-0.002567
	0.010677					
z168	0.016360	0.023584	0.029846	-0.033287	0.005187	0.043321
	-0.033424	0.004674	-0.008976	-0.010698	0.003709	0.028009
	0.035026	-0.019033	-0.026899	0.001079	0.043579	-0.009421
	-0.017657	-0.017817	0.046117	0.002443	-0.020800	0.011384
	0.042556	0.013661	-0.056299	0.025739	-0.013410	0.026767
	-0.023860					
x169	0.068926	0.017907	0.015279	0.019687	0.010406	-0.031109
	-0.013865	-0.017783	0.012679	-0.053470	0.002833	0.054578
	-0.006376	0.035910	0.001814	-0.010101	-0.008403	0.034827
	-0.014586	-0.010980	0.052437	-0.025369	0.022642	-0.037948
	0.033669	0.067818	0.005132	-0.004426	-0.039500	0.082731
	-0.023484					
y169	0.015949	-0.010561	-0.028536	0.055419	-0.050114	0.042371
	0.036460	0.004195	-0.031606	-0.011219	0.026443	-0.007750
	0.033002	-0.025839	0.032086	0.000745	-0.026951	-0.025405
	0.015668	-0.035651	0.002098	0.006530	0.042910	0.009270
	0.023250	-0.010792	0.015914	-0.000545	0.013768	-0.007972
	0.000874					
z169	0.014004	0.014977	0.032463	-0.030663	0.002091	0.043750
	-0.031030	0.010374	-0.021439	-0.010964	0.007536	0.026554
	0.029049	-0.017429	-0.024426	0.005215	0.035267	-0.006033
	-0.010607	-0.040814	0.047349	0.002328	-0.003519	0.002788
	0.015606	-0.002107	-0.025343	0.044645	-0.038949	0.031451
	-0.040575					

x170	0.067592	0.015683	0.018211	0.024845	0.005256	-0.032488
	-0.007920	-0.012123	0.019298	-0.047013	0.007024	0.049346
	-0.010233	0.036903	-0.007824	-0.016762	-0.004542	0.016253
	-0.017413	-0.007249	0.043469	-0.015124	0.018371	-0.022682
	0.038854	0.055530	0.012874	-0.005386	-0.059163	0.086305
	-0.008484					
y170	0.016218	-0.014867	-0.033152	0.046778	-0.046975	0.040898
	0.036152	-0.004568	-0.037093	-0.009742	0.028991	-0.001807
	0.031375	-0.021644	0.034257	0.007374	-0.020077	-0.005190
	0.014260	-0.029797	0.011562	0.004805	0.029069	0.006266
	0.026676	-0.019536	-0.003924	0.013418	0.003277	-0.025277
	-0.013904					
z170	0.013700	0.007878	0.032500	-0.027974	0.000207	0.047883
	-0.027102	0.003526	-0.030454	-0.009952	0.008951	0.026513
	0.023918	-0.011274	-0.022637	0.008286	0.030355	-0.004487
	-0.003309	-0.048760	0.044542	0.000390	0.003887	0.005629
	0.014439	-0.020718	-0.021729	0.057271	-0.065454	0.016435
	-0.053432					
x171	0.064362	0.013359	0.019585	0.025251	0.004708	-0.027774
	-0.000894	-0.011500	0.020812	-0.043301	0.005277	0.043450
	-0.016801	0.035706	-0.012526	-0.013484	0.003079	0.007536
	-0.009681	-0.021012	0.034349	-0.019559	0.020893	-0.020554
	0.035209	0.035605	0.019073	-0.005376	-0.049310	0.050856
	-0.009058					
y171	0.017090	-0.018508	-0.036764	0.037835	-0.044691	0.035775
	0.033541	-0.008155	-0.036655	-0.008405	0.034232	0.002681
	0.031831	-0.018021	0.031550	0.009398	-0.016537	0.006472
	0.008054	-0.014586	0.016174	0.009859	0.015137	0.011146
	0.030472	-0.018923	-0.018300	0.021383	-0.015293	-0.024902
	-0.016380					
z171	0.012736	0.002059	0.031998	-0.025415	-0.000866	0.050858
	-0.024215	-0.004091	-0.035691	-0.007978	0.009570	0.025643
	0.019751	-0.000973	-0.023961	0.011916	0.026585	-0.004851
	0.003955	-0.050657	0.034376	-0.000107	0.009299	0.007936
	0.012705	-0.030629	-0.020584	0.061792	-0.087999	0.004469
	-0.059721					
x172	0.064434	0.014206	0.020068	0.027878	0.002201	-0.022450
	0.003467	-0.014194	0.025750	-0.032391	0.008458	0.033950
	-0.015489	0.031079	-0.017989	-0.012815	0.002719	0.004360
	-0.004772	-0.025581	0.031171	-0.028589	0.039452	-0.020443
	0.030442	0.023306	0.026741	-0.011888	-0.053379	0.036725
	0.008043					

y172 0.016025 -0.023824 -0.040002 0.025194 -0.039483 0.030302
0.031579 -0.006789 -0.037727 -0.012642 0.036728 0.007211
0.030717 -0.014477 0.028844 0.014226 -0.011363 0.011923
0.000859 -0.003199 0.013140 0.017293 -0.009937 0.006766
0.032879 -0.009394 -0.026222 0.023706 -0.015446 -0.031403
-0.027363

z172 0.011325 -0.001965 0.030629 -0.022932 -0.002150 0.054104
-0.020424 -0.012320 -0.037156 -0.004869 0.012972 0.024858
0.017025 0.008078 -0.023948 0.011222 0.017463 -0.005098
0.009819 -0.045686 0.020222 0.000192 0.017902 0.006482
0.016221 -0.026852 -0.017419 0.053864 -0.098124 0.006077
-0.058699

x173 0.064627 0.014062 0.020514 0.028804 0.000791 -0.015401
0.007170 -0.014174 0.028751 -0.021775 0.016752 0.032026
-0.014937 0.023753 -0.023493 -0.018095 0.010450 -0.005778
-0.003081 -0.018455 0.021235 -0.025625 0.037899 -0.025397
0.030240 0.020223 0.013860 -0.011695 -0.051692 0.025463
0.017942

y173 0.014766 -0.026882 -0.041643 0.012919 -0.033200 0.021641
0.028276 -0.005834 -0.035687 -0.014653 0.037593 0.001267
0.028173 -0.007559 0.028537 0.023458 -0.014916 0.023115
-0.000283 0.002068 0.014476 0.012429 -0.011915 0.010027
0.023026 -0.008271 -0.024157 0.018888 -0.018976 -0.035868
-0.038159

z173 0.009782 -0.004535 0.030238 -0.019712 -0.000572 0.055893
-0.019309 -0.022993 -0.035585 0.000031 0.017321 0.019821
0.014790 0.020692 -0.021086 0.011210 0.007527 -0.008063
0.017940 -0.035442 0.004890 -0.004174 0.028659 0.003837
0.013185 -0.016518 -0.016005 0.041381 -0.100484 0.011923
-0.059828

x174 0.063129 0.014038 0.019349 0.030416 0.000986 -0.010055
0.010296 -0.013447 0.029609 -0.008776 0.030724 0.031966
-0.024891 0.017673 -0.025861 -0.027193 0.021744 -0.009661
0.001464 -0.023203 0.006438 -0.022079 0.031007 -0.014607
0.026551 0.021156 0.005770 -0.004371 -0.034951 0.034009
0.046309

y174 0.014653 -0.030245 -0.041653 -0.000743 -0.027109 0.014176
0.024531 -0.004093 -0.030481 -0.018342 0.034982 -0.007797
0.030207 -0.001587 0.024070 0.032963 -0.020914 0.026324
-0.002763 0.015263 0.015447 0.005452 -0.015564 0.001645
0.017243 -0.011363 -0.017186 0.007034 -0.027307 -0.049470
-0.053781

z174	0.008323	-0.005707	0.029519	-0.016707	0.001902	0.059088
	-0.019823	-0.036163	-0.029854	0.005604	0.021305	0.014849
	0.010849	0.033415	-0.019874	0.010530	-0.001943	-0.017418
	0.027385	-0.022145	-0.014010	-0.013285	0.035134	-0.000741
	0.016146	-0.007433	-0.012465	0.023020	-0.093873	0.013445
	-0.046943					
x175	0.059948	0.015560	0.019392	0.034554	-0.002390	-0.009738
	0.012733	-0.011724	0.032949	-0.002174	0.032465	0.029323
	-0.034324	0.016926	-0.032488	-0.023356	0.020948	-0.008700
	0.004515	-0.024897	0.007598	-0.028703	0.027638	-0.011337
	0.021041	0.015099	0.020690	-0.018071	-0.010575	0.030235
	0.058509					
y175	0.015314	-0.034803	-0.041308	-0.018032	-0.017770	0.010019
	0.021129	-0.000953	-0.026913	-0.014415	0.042752	-0.014765
	0.032533	-0.001908	0.022897	0.028975	-0.020544	0.021948
	-0.006976	0.024844	0.003277	0.005888	-0.026943	-0.000138
	0.019203	-0.005532	-0.021460	0.014918	-0.034910	-0.044162
	-0.054567					
z175	0.003825	-0.003977	0.028413	-0.010350	0.002515	0.057406
	-0.020948	-0.050477	-0.021459	0.012496	0.018218	0.014656
	0.005011	0.047052	-0.015970	0.007798	-0.012683	-0.034569
	0.038272	0.000193	-0.030616	-0.026621	0.030617	-0.004834
	0.033079	0.008456	0.009034	0.014478	-0.062319	0.008183
	-0.036469					
x176	0.058776	0.014161	0.016555	0.032605	-0.006242	-0.007456
	0.014312	-0.015158	0.033561	0.001874	0.026679	0.030079
	-0.035195	0.019195	-0.030871	-0.012632	0.021586	-0.008941
	0.014430	-0.013630	0.004330	-0.037353	0.032175	-0.005667
	0.016441	0.000218	0.013796	-0.008882	-0.005828	0.012964
	0.051387					
y176	0.014324	-0.037372	-0.038948	-0.028053	-0.010962	0.003194
	0.018111	0.009252	-0.024812	-0.010346	0.052609	-0.020386
	0.028055	-0.006415	0.018228	0.020752	-0.015965	0.023351
	-0.012908	0.015603	0.004661	0.003643	-0.033689	0.006443
	0.022112	0.007389	-0.014745	0.017770	-0.027367	-0.025832
	-0.035246					
z176	-0.000344	-0.005436	0.024395	-0.004607	0.000374	0.052440
	-0.020748	-0.053796	-0.016785	0.017976	0.005507	0.016396
	0.001151	0.057511	-0.010366	0.011978	-0.011513	-0.036114
	0.052860	0.010992	-0.032396	-0.040185	0.033272	0.005222
	0.046145	0.026370	0.017448	0.034565	-0.027467	-0.001133
	-0.024747					

x177	0.057464	0.013971	0.012942	0.030185	-0.009957	-0.004491
	0.012374	-0.016976	0.033884	0.009390	0.021954	0.028431
	-0.036672	0.020496	-0.030921	-0.006226	0.021512	-0.009547
	0.012844	-0.009443	0.002783	-0.036672	0.031222	-0.006075
	0.012193	-0.000878	0.004565	-0.001823	-0.005374	0.006121
	0.049903					
y177	0.013864	-0.041644	-0.035448	-0.036530	-0.004041	-0.004176
	0.017649	0.017357	-0.021902	-0.014914	0.060643	-0.026009
	0.025797	-0.008439	0.014835	0.019234	-0.014774	0.025946
	-0.006537	0.016699	0.004124	-0.002778	-0.031167	0.016213
	0.019634	0.006654	-0.008664	0.015610	-0.021128	-0.017713
	-0.033016					
z177	-0.001904	-0.008879	0.020465	0.001240	-0.001332	0.050963
	-0.022085	-0.056815	-0.009956	0.017232	-0.001216	0.015494
	-0.000872	0.063415	-0.006721	0.019177	-0.012421	-0.035175
	0.062749	0.020085	-0.031751	-0.045156	0.038427	0.009287
	0.048916	0.034279	0.015102	0.029338	-0.009566	-0.006768
	-0.017301					
x178	0.054938	0.013897	0.011452	0.026986	-0.012783	-0.004528
	0.008844	-0.013624	0.035189	0.016454	0.021283	0.023879
	-0.038464	0.017112	-0.036183	0.000562	0.021846	-0.008637
	0.010355	-0.010073	0.001012	-0.035455	0.032205	-0.012600
	0.006186	0.002104	0.012670	-0.000394	0.007726	0.009712
	0.050489					
y178	0.015139	-0.046196	-0.033832	-0.044023	0.001177	-0.009982
	0.020862	0.021594	-0.021865	-0.019387	0.062050	-0.028932
	0.025582	-0.007175	0.020300	0.017686	-0.018851	0.029631
	0.001746	0.022484	0.005442	-0.010759	-0.028365	0.032543
	0.015113	0.002644	-0.019336	0.018718	-0.033546	-0.012289
	-0.033159					
z178	-0.001973	-0.012628	0.017603	0.006652	-0.003366	0.048392
	-0.021225	-0.055784	-0.003491	0.016240	-0.006711	0.014316
	-0.002793	0.063564	-0.004297	0.025672	-0.016895	-0.030116
	0.070086	0.024132	-0.029155	-0.048180	0.043836	0.012025
	0.046347	0.036271	0.012854	0.020290	0.001134	-0.006524
	-0.008139					
x179	0.051244	0.012351	0.010703	0.023083	-0.015534	-0.006791
	0.008378	-0.008188	0.032113	0.023455	0.023464	0.015129
	-0.040287	0.014979	-0.040120	0.006325	0.017373	-0.006269
	0.009204	-0.011508	0.001763	-0.030193	0.029676	-0.007683
	-0.005762	0.010087	0.010645	-0.002908	0.009460	0.016995
	0.034207					

y179	0.017693	-0.048295	-0.032882	-0.051185	0.006501	-0.011354
	0.017591	0.020418	-0.016461	-0.026576	0.056142	-0.024583
	0.024610	-0.007648	0.024114	0.020510	-0.012171	0.025686
	0.008961	0.032908	0.001972	-0.023968	-0.026672	0.033625
	0.021479	-0.015437	-0.017625	0.030098	-0.033947	-0.022758
	-0.008026					
z179	-0.001857	-0.016527	0.015982	0.011712	-0.004576	0.046258
	-0.019996	-0.053027	0.002368	0.012904	-0.012252	0.011819
	-0.004948	0.059032	-0.001072	0.029146	-0.025595	-0.026973
	0.077724	0.030226	-0.029294	-0.048031	0.048458	0.015793
	0.035818	0.039655	0.016380	0.014634	0.007421	0.000786
	-0.002048					
x180	0.049271	0.012764	0.009033	0.019622	-0.019884	-0.004028
	0.008367	-0.012584	0.032937	0.029287	0.024070	0.013369
	-0.043595	0.013959	-0.039485	0.012823	0.019013	-0.010056
	0.014076	-0.000312	-0.002509	-0.031121	0.025311	-0.004977
	-0.001189	0.001351	-0.008891	0.001574	0.005417	0.004842
	0.035789					
y180	0.018044	-0.052775	-0.029725	-0.059447	0.014507	-0.020521
	0.014231	0.033111	-0.018380	-0.031735	0.051237	-0.027585
	0.026917	-0.008428	0.021746	0.022301	-0.012358	0.029815
	0.000292	0.025748	0.005801	-0.026376	-0.025558	0.039394
	0.009362	-0.008725	0.011355	0.029660	-0.027045	-0.010938
	-0.007727					
z180	-0.000846	-0.019436	0.015080	0.017025	-0.005580	0.045998
	-0.017312	-0.054186	0.007435	0.010844	-0.018825	0.013026
	-0.006375	0.055634	0.003062	0.031685	-0.034026	-0.025933
	0.083319	0.042517	-0.033231	-0.050683	0.055357	0.020364
	0.030893	0.036662	0.017170	0.014960	0.006400	0.009458
	0.003581					
x181	0.045807	0.011120	0.005603	0.014366	-0.022575	-0.003858
	0.007981	-0.011863	0.031959	0.032509	0.027897	0.006807
	-0.045145	0.008374	-0.038883	0.019375	0.023075	-0.008602
	0.011850	0.001963	-0.003666	-0.032617	0.024419	-0.003760
	0.002242	0.007036	-0.009911	0.000992	-0.000856	0.019331
	0.023327					
y181	0.019364	-0.054683	-0.021477	-0.064739	0.019452	-0.028766
	0.010749	0.040693	-0.018014	-0.033402	0.041409	-0.025900
	0.028271	-0.002848	0.019242	0.019707	-0.022100	0.027332
	0.004909	0.026632	0.004891	-0.021246	-0.023246	0.047217
	-0.004111	-0.025810	0.020367	0.036588	-0.016335	-0.029134
	0.003634					

z181	0.000970	-0.022802	0.013821	0.023213	-0.005148	0.045267
	-0.015202	-0.053214	0.010999	0.006267	-0.024257	0.016167
	-0.005611	0.047176	0.009681	0.030271	-0.044472	-0.022822
	0.084722	0.052809	-0.036705	-0.047798	0.062305	0.020520
	0.024182	0.041082	0.026478	0.017679	0.004121	0.036358
	0.000938					
x182	0.040884	0.008367	0.003772	0.008885	-0.024783	-0.007169
	0.005483	-0.006425	0.029190	0.036029	0.033313	-0.002334
	-0.046088	0.002145	-0.038748	0.025528	0.024060	-0.005830
	0.008799	0.001190	-0.001222	-0.032714	0.021014	-0.000028
	-0.001043	0.005818	-0.004534	0.000387	-0.008908	0.017109
	0.014624					
y182	0.023258	-0.053680	-0.019893	-0.069240	0.019586	-0.027728
	0.012925	0.035533	-0.010037	-0.033653	0.031134	-0.019744
	0.024249	0.000117	0.016272	0.016964	-0.022496	0.023381
	0.008738	0.031807	0.000910	-0.019702	-0.017855	0.047679
	0.000438	-0.029076	0.002871	0.047767	0.004173	-0.014440
	0.012787					
z182	0.003785	-0.026295	0.012243	0.032558	-0.005316	0.043333
	-0.015092	-0.048844	0.015529	0.002888	-0.027019	0.020520
	-0.005609	0.033461	0.016762	0.025080	-0.051362	-0.017984
	0.081531	0.060219	-0.038727	-0.040816	0.059827	0.020284
	0.006128	0.041711	0.029088	0.017228	-0.001256	0.049750
	-0.006495					
x183	0.036577	0.005803	0.003127	0.003780	-0.025726	-0.009272
	0.005092	-0.001837	0.026774	0.040624	0.038110	-0.007883
	-0.044741	-0.001350	-0.037286	0.031558	0.020065	-0.001062
	0.007620	0.004647	-0.002326	-0.033720	0.020601	0.002363
	-0.015685	-0.000496	0.007022	0.008376	0.004637	0.022547
	-0.002213					
y183	0.026389	-0.052126	-0.020108	-0.071713	0.017478	-0.029394
	0.007987	0.033983	-0.006786	-0.037734	0.017853	-0.018302
	0.011243	-0.005210	0.012911	0.021361	-0.010785	0.014314
	0.002795	0.036268	0.012601	-0.023285	-0.016957	0.031870
	0.027142	-0.014905	-0.018847	0.047284	-0.007715	0.009315
	0.050618					
z183	0.006282	-0.033197	0.011067	0.040189	-0.004024	0.041724
	-0.014416	-0.045607	0.020022	0.000608	-0.026093	0.016483
	-0.006224	0.018982	0.016793	0.023396	-0.053952	-0.017361
	0.081841	0.067368	-0.039891	-0.037528	0.050305	0.033330
	0.001129	0.029978	0.022046	0.030611	-0.001046	0.043407
	-0.025957					

x184	0.032207	0.003600	0.002456	-0.003286	-0.024305	-0.011268
	0.002940	0.001835	0.023288	0.042271	0.037166	-0.014068
	-0.049015	-0.006341	-0.035682	0.046258	0.019181	-0.001132
	0.006006	0.011100	-0.002025	-0.036653	0.017442	0.008375
	-0.023757	-0.007201	0.009977	0.013290	0.007194	0.019087
	-0.001130					
y184	0.027147	-0.051933	-0.012138	-0.077658	0.016715	-0.035122
	0.005067	0.041975	-0.004284	-0.041800	0.013004	-0.022015
	0.015503	0.004904	0.013771	0.006870	-0.010963	0.016212
	0.011795	0.025552	0.003925	-0.010692	-0.023265	0.029880
	0.017475	0.007769	0.004313	0.052454	-0.001336	0.005704
	0.013446					
z184	0.003888	-0.036960	0.007757	0.050160	-0.010872	0.034412
	-0.013659	-0.040277	0.020922	0.004207	-0.017970	-0.008550
	0.001944	0.008297	0.020179	-0.001899	-0.060977	-0.019653
	0.079667	0.068577	-0.019980	-0.029976	0.058490	0.032171
	0.020026	0.056480	0.016481	0.038639	0.000763	0.042087
	-0.050293					
x185	0.027519	0.001434	0.002627	-0.010223	-0.024876	-0.013802
	-0.000318	0.007332	0.021555	0.043446	0.036920	-0.021090
	-0.053989	-0.009780	-0.035322	0.059120	0.018142	0.002905
	0.005904	0.011995	-0.011601	-0.033537	0.010062	0.006371
	-0.044108	0.002835	0.021034	0.011190	0.013616	0.029048
	-0.010755					
y185	0.026133	-0.052039	-0.010253	-0.080227	0.018566	-0.040947
	0.006401	0.047656	-0.006738	-0.038407	0.010469	-0.025549
	0.024188	0.016202	0.016072	-0.007209	-0.011955	0.003650
	-0.007346	0.033213	0.019246	-0.011846	-0.019731	0.038009
	0.036557	-0.037686	-0.001452	0.058045	-0.019094	-0.057624
	0.000464					
z185	0.003688	-0.038718	0.001402	0.052361	-0.016858	0.030467
	-0.003612	-0.033362	0.026358	0.014788	-0.018483	-0.027951
	0.018368	0.008606	0.022189	-0.026997	-0.061314	-0.019785
	0.071049	0.083575	0.005496	-0.031060	0.050874	0.018471
	0.048169	0.070228	0.002246	0.023566	-0.016222	0.031879
	-0.040511					
x186	0.022518	-0.000294	0.002547	-0.014696	-0.025277	-0.016261
	-0.002227	0.013741	0.018004	0.044376	0.039552	-0.034065
	-0.056269	-0.014425	-0.029893	0.065857	0.008543	0.002138
	0.002566	0.013506	0.001298	-0.029590	0.006754	0.005402
	-0.055314	0.011993	0.021531	0.017171	0.026650	0.033827
	-0.013743					

y186	0.031944	-0.045278	-0.014198	-0.083771	0.015144	-0.041320
	0.004720	0.042458	-0.000305	-0.028971	0.006988	-0.022562
	0.021026	0.037388	0.024646	-0.009556	-0.018684	-0.007992
	-0.021834	0.020508	0.019937	-0.016860	-0.015979	0.043796
	0.047600	-0.030090	-0.007171	0.087050	-0.024686	-0.068942
	0.006216					
z186	0.008227	-0.041762	-0.001649	0.054246	-0.015843	0.031028
	0.003344	-0.025267	0.037110	0.017140	-0.024629	-0.015994
	0.025636	0.007211	0.017706	-0.025906	-0.045614	-0.016232
	0.050498	0.095230	-0.009121	-0.032225	0.025515	0.007228
	0.040409	0.045224	-0.002077	-0.004140	-0.042135	0.029145
	-0.050106					
x187	0.017188	0.000052	0.003584	-0.021146	-0.026950	-0.018654
	-0.001725	0.016208	0.011598	0.046621	0.038481	-0.036813
	-0.053848	-0.013304	-0.027648	0.065233	0.002559	-0.000323
	-0.002679	0.018502	0.006244	-0.021882	0.005389	0.010483
	-0.054009	0.014774	0.020318	0.020490	0.023014	0.024058
	-0.016230					
y187	0.032805	-0.046745	-0.019369	-0.088502	0.008048	-0.046773
	-0.001742	0.057967	0.011765	-0.025086	0.009154	-0.049309
	0.017764	0.035575	0.021553	-0.007768	-0.017254	-0.003374
	-0.013515	0.000119	0.003009	-0.018468	-0.015466	0.045151
	0.015024	-0.011087	0.027929	0.082592	-0.019079	-0.043278
	-0.028894					
z187	0.011337	-0.047459	-0.004035	0.055995	-0.011017	0.030760
	0.006212	-0.007983	0.047360	0.014424	-0.028459	-0.021102
	0.027977	0.001223	0.008634	-0.018712	-0.035163	-0.011130
	0.038883	0.094602	-0.021328	-0.039429	0.006915	-0.006512
	0.021823	0.033626	0.011077	-0.022660	-0.048118	0.049884
	-0.067116					
x188	0.011403	-0.000025	0.007039	-0.026659	-0.025325	-0.022779
	-0.000662	0.024603	0.001875	0.048664	0.034851	-0.039348
	-0.050519	-0.011474	-0.024804	0.063711	-0.006841	-0.002016
	-0.006959	0.025990	0.013736	-0.010940	0.005306	0.010790
	-0.063509	0.024949	0.026421	0.019000	0.022995	0.032421
	-0.025732					
y188	0.033625	-0.049679	-0.024211	-0.083793	0.004285	-0.050156
	-0.003419	0.067035	0.017435	-0.024090	0.006123	-0.063521
	0.008375	0.031912	0.025847	0.008770	-0.008499	-0.007229
	-0.016176	0.002950	0.019870	-0.031530	-0.014021	0.032521
	-0.001170	-0.003785	0.022568	0.081904	-0.009552	-0.019720
	-0.052067					

z188	0.014111	-0.053777	-0.007718	0.057598	-0.009719	0.029458
	0.011060	0.003016	0.054689	0.011226	-0.030952	-0.028802
	0.028034	-0.008940	0.002490	-0.008275	-0.023521	-0.008322
	0.027182	0.088202	-0.026109	-0.053434	-0.007363	-0.017551
	0.015653	0.019198	0.015828	-0.032729	-0.053805	0.060323
	-0.069088					
x189	0.008530	0.002342	0.007294	-0.032207	-0.026356	-0.020673
	0.000349	0.021496	-0.004233	0.053314	0.029672	-0.034274
	-0.049897	-0.007503	-0.022025	0.064320	-0.007987	-0.010301
	-0.011914	0.041905	0.023363	-0.010056	-0.005317	0.013375
	-0.053861	0.019660	0.001460	0.022414	0.018325	0.013490
	-0.005290					
y189	0.041944	-0.048328	-0.029844	-0.083379	-0.008589	-0.041304
	-0.004012	0.050886	0.027364	-0.018872	-0.007808	-0.050323
	0.001679	0.041348	0.044295	0.027043	0.004469	-0.030210
	-0.021067	0.012368	0.039104	-0.060669	-0.025219	0.032230
	0.018177	-0.030654	-0.033716	0.074036	0.003278	-0.047019
	0.012311					
z189	0.017830	-0.057796	-0.012724	0.058812	-0.011212	0.032091
	0.016135	-0.003368	0.062640	0.006489	-0.032874	-0.025747
	0.028338	-0.015077	0.005704	-0.000030	-0.011823	-0.015193
	0.013948	0.084677	-0.027690	-0.070546	-0.013966	-0.012965
	0.039414	-0.010127	-0.010468	-0.031632	-0.058992	0.026546
	-0.042699					
x190	0.004067	0.002432	0.009046	-0.037443	-0.027187	-0.022849
	0.002118	0.027351	-0.012829	0.057911	0.025784	-0.036466
	-0.049745	-0.001690	-0.021715	0.060151	-0.013441	-0.017098
	-0.013760	0.052562	0.032284	-0.008735	-0.012196	0.010015
	-0.058300	0.022203	-0.008393	0.019199	0.008132	-0.001236
	0.000313					
y190	0.041184	-0.054310	-0.022444	-0.082663	-0.015890	-0.051526
	-0.009064	0.068575	0.023223	-0.020157	-0.008940	-0.068448
	0.010484	0.057136	0.059789	0.013361	-0.009465	-0.029946
	-0.001023	-0.021599	0.047177	-0.056341	0.002486	0.004136
	-0.022099	-0.009965	0.016611	0.044636	0.040043	-0.012548
	-0.010814					
z190	0.020065	-0.060825	-0.011732	0.060023	-0.009556	0.029826
	0.019474	-0.007127	0.064504	-0.001179	-0.036419	-0.024457
	0.041681	-0.017618	0.018563	0.000561	-0.008452	-0.016886
	0.002381	0.072651	-0.032945	-0.069137	-0.004580	-0.011579
	0.050806	-0.026850	-0.004259	-0.026958	-0.039613	0.027640
	-0.042377					

x191	-0.002425	0.004330	0.011688	-0.043012	-0.024920	-0.023471
	0.003709	0.030546	-0.020456	0.063457	0.022693	-0.036940
	-0.049998	0.003879	-0.030283	0.054777	-0.021318	-0.017945
	-0.022287	0.069700	0.033477	0.000322	-0.018891	0.007603
	-0.049544	0.027112	-0.013412	0.019464	-0.009875	-0.014575
	0.004207					
y191	0.038612	-0.047589	-0.019958	-0.077205	-0.021294	-0.044637
	-0.014104	0.054448	0.027236	-0.020123	-0.014056	-0.063721
	0.014188	0.067887	0.048999	0.002439	-0.014743	-0.028041
	-0.006716	-0.015970	0.034566	-0.042746	0.015282	-0.005396
	0.032275	-0.009639	0.022504	0.064503	0.038663	-0.043984
	-0.002506					
z191	0.021636	-0.062825	-0.009437	0.062661	-0.006256	0.027599
	0.024060	-0.006489	0.063938	-0.010856	-0.038736	-0.020088
	0.054525	-0.024827	0.024042	-0.001655	-0.005790	-0.011848
	-0.012043	0.057175	-0.035598	-0.066447	0.005528	-0.006223
	0.062311	-0.043012	0.013103	-0.011362	-0.016668	0.031255
	-0.048070					
x192	-0.010161	0.005524	0.019467	-0.049644	-0.020187	-0.025080
	0.004422	0.040557	-0.033470	0.066330	0.019480	-0.038416
	-0.045490	0.008419	-0.038250	0.043898	-0.036117	-0.015641
	-0.033090	0.073948	0.034879	0.013606	-0.021849	0.002044
	-0.055713	0.023180	0.013810	0.004872	-0.027896	0.004734
	-0.011657					
y192	0.036263	-0.044969	-0.018434	-0.075433	-0.028818	-0.034812
	-0.020967	0.052686	0.028977	-0.023134	-0.017413	-0.059106
	0.013705	0.061724	0.038877	-0.000100	-0.012441	-0.028595
	-0.013301	-0.042011	0.018701	-0.038957	0.029824	-0.029160
	0.033085	-0.031457	0.047485	0.048040	0.044426	-0.026907
	0.007691					
z192	0.024697	-0.065356	-0.015310	0.064685	-0.007626	0.025228
	0.029481	-0.009071	0.067939	-0.017294	-0.042936	-0.016335
	0.058313	-0.034855	0.029230	0.006381	0.006311	-0.012369
	-0.023507	0.043621	-0.039723	-0.071178	0.014367	-0.000206
	0.066266	-0.037774	-0.007355	0.010986	0.006772	0.015002
	-0.018147					
x193	-0.016003	0.009244	0.018679	-0.049511	-0.018511	-0.019748
	0.001781	0.039728	-0.036313	0.064146	0.010806	-0.029431
	-0.048711	0.003788	-0.040162	0.038507	-0.024879	-0.025542
	-0.032232	0.096713	0.035269	0.008454	-0.022188	-0.003269
	-0.034213	0.019776	0.000742	0.003272	-0.022085	0.001038
	0.005491					

y193	0.036403	-0.037681	-0.027331	-0.068273	-0.039592	-0.020062
	-0.027957	0.053362	0.039221	-0.022018	-0.022582	-0.045814
	0.002194	0.052676	0.030907	-0.001559	0.005957	-0.038924
	-0.012307	-0.046134	-0.012237	-0.044055	0.034344	-0.066796
	0.018593	-0.007750	0.020718	0.025278	0.051693	-0.027021
	0.052032					
z193	0.028142	-0.068424	-0.011801	0.059268	-0.003735	0.016951
	0.038543	-0.002738	0.062093	-0.017235	-0.042380	-0.020958
	0.070017	-0.029847	0.032398	0.008230	-0.013297	0.005499
	-0.048095	0.003957	-0.056753	-0.048561	0.021564	-0.003121
	0.039281	-0.015652	0.001547	0.020988	0.006484	0.008913
	-0.017152					
x194	-0.028020	0.008324	0.021125	-0.049093	-0.014086	-0.016377
	0.005449	0.048569	-0.047502	0.057189	0.006986	-0.027819
	-0.044811	0.002363	-0.041212	0.025718	-0.035234	-0.050466
	-0.041755	0.095647	0.031666	0.029522	-0.008249	0.001127
	-0.035803	0.043800	-0.022349	0.015366	-0.010006	-0.001880
	-0.005824					
y194	0.027897	-0.027401	-0.032216	-0.060698	-0.044567	-0.015725
	-0.038049	0.062214	0.039567	-0.009573	-0.030388	-0.041366
	-0.008602	0.042728	0.033053	-0.005279	0.001990	-0.042113
	-0.015657	-0.035929	-0.039843	-0.015491	0.052253	-0.120329
	-0.007396	0.061788	0.012101	-0.017140	0.068359	0.002354
	0.073651					
z194	0.032493	-0.066717	-0.008626	0.050666	0.005425	0.005049
	0.044094	0.008443	0.054835	-0.004706	-0.052869	-0.024830
	0.072245	-0.036371	0.028745	0.019264	-0.018258	0.048321
	-0.072947	-0.004609	-0.062150	-0.048480	0.020568	-0.026239
	0.021867	-0.016884	0.025894	-0.000409	-0.000978	0.036440
	0.014046					
x195	-0.037083	0.010426	0.032475	-0.050987	-0.002813	-0.014426
	0.010347	0.055774	-0.065845	0.046696	-0.000155	-0.028204
	-0.032021	0.008747	-0.036876	-0.000013	-0.050932	-0.055731
	-0.054657	0.084997	0.017367	0.065006	0.004010	0.012180
	-0.026218	0.036703	-0.019152	0.014182	-0.023915	-0.004423
	-0.040596					
y195	0.028760	-0.016931	-0.032232	-0.055955	-0.043573	-0.007948
	-0.046028	0.068576	0.044988	-0.000889	-0.039907	-0.044601
	-0.018742	0.036583	0.029526	-0.006307	-0.007213	-0.035916
	-0.022584	-0.031263	-0.061656	0.020553	0.057013	-0.124365
	-0.018751	0.062541	0.015114	-0.041146	0.056526	0.040720
	0.048512					

z195	0.033445	-0.064021	-0.010074	0.047615	0.009134	-0.011237
	0.047858	0.016806	0.051952	0.002406	-0.061694	-0.026951
	0.070102	-0.046125	0.025572	0.029391	-0.019407	0.067758
	-0.089065	-0.000412	-0.059475	-0.049060	0.028317	-0.038309
	0.022383	-0.003354	0.049440	0.022755	0.016747	0.061372
	0.040609					
x196	-0.046085	0.011152	0.038863	-0.052428	0.003252	-0.010130
	0.014030	0.061865	-0.079568	0.035437	-0.001442	-0.023541
	-0.018149	0.021552	-0.032173	-0.018246	-0.060358	-0.055040
	-0.055062	0.059877	0.020734	0.071312	0.020794	0.018627
	0.010529	0.010277	-0.024027	0.028834	-0.048162	-0.019520
	-0.072711					
y196	0.029422	-0.012260	-0.038087	-0.052352	-0.047192	0.004289
	-0.048301	0.068709	0.051142	0.004977	-0.039227	-0.044417
	-0.017377	0.036237	0.018746	0.011047	-0.008383	-0.031385
	-0.014846	-0.046977	-0.049687	0.019984	0.048028	-0.110023
	0.003954	0.035208	-0.006903	-0.022076	0.021803	0.039953
	0.003471					
z196	0.031801	-0.059452	-0.013024	0.046416	0.012110	-0.027679
	0.050212	0.020798	0.047312	0.009078	-0.064104	-0.030122
	0.066408	-0.059865	0.016495	0.042022	-0.014933	0.076336
	-0.095742	0.005064	-0.060480	-0.041322	0.023786	-0.033776
	0.022532	0.007094	0.054607	0.051156	0.016286	0.064367
	0.041233					
x197	-0.054500	0.019857	0.033527	-0.044095	-0.001331	-0.014678
	0.008978	0.062164	-0.076994	0.028972	0.000484	-0.020978
	-0.011388	0.030693	-0.015363	-0.024714	-0.049236	-0.047487
	-0.065713	0.033753	0.028221	0.048870	0.017641	-0.008966
	0.040909	0.012568	-0.034515	0.044328	-0.039084	-0.016088
	-0.084873					
y197	0.029435	-0.002959	-0.047596	-0.038934	-0.053663	0.008879
	-0.054156	0.058069	0.061795	0.012672	-0.037033	-0.039280
	-0.014987	0.037567	0.006752	0.022322	0.005212	-0.026967
	-0.021936	-0.065685	-0.035181	0.013673	0.007341	-0.122134
	0.033805	0.043345	-0.038913	0.012622	0.013400	0.037467
	-0.034271					
z197	0.026421	-0.049577	-0.017026	0.046553	0.014136	-0.038747
	0.046302	0.022449	0.043730	0.012284	-0.060276	-0.026434
	0.056755	-0.069144	0.000447	0.044991	-0.006542	0.071154
	-0.091444	-0.000501	-0.062637	-0.033258	0.016926	-0.025195
	0.032303	0.010521	0.040128	0.066267	0.009195	0.056617
	0.039517					

x198	-0.055612	0.020857	0.036816	-0.032689	-0.009397	-0.009259
	0.010316	0.071531	-0.069660	0.021898	-0.010545	-0.019221
	-0.015434	0.028243	-0.003406	-0.016430	-0.037969	-0.033397
	-0.065222	-0.011649	0.041953	0.019199	0.032855	-0.009470
	0.056736	0.006215	-0.019768	0.016267	-0.027583	0.039651
	-0.068264					
y198	0.036360	-0.002087	-0.045875	-0.028034	-0.053922	0.024350
	-0.050787	0.056355	0.066075	0.012588	-0.058261	-0.034855
	-0.019294	0.029914	-0.001705	0.047711	0.003917	-0.018799
	-0.020146	-0.096519	-0.011726	0.011084	0.007582	-0.088744
	0.037233	0.042448	-0.057461	0.002253	0.012490	0.044691
	-0.038320					
z198	0.016989	-0.038317	-0.018972	0.047126	0.016063	-0.046409
	0.037580	0.028344	0.039845	0.012261	-0.054685	-0.017963
	0.038493	-0.071402	-0.009508	0.039860	0.001915	0.055187
	-0.076621	-0.007725	-0.063656	-0.016593	0.027025	-0.008400
	0.023045	0.021575	0.017943	0.052714	0.018958	0.062019
	0.035866					
x199	-0.062287	0.023326	0.042694	-0.018784	-0.020742	-0.015794
	0.017888	0.077339	-0.068149	0.011245	-0.021544	-0.009171
	-0.019109	0.028659	0.007451	-0.025429	-0.036271	-0.016510
	-0.069070	-0.022863	0.049646	0.003076	0.073923	-0.022923
	0.062894	0.000546	0.013104	-0.004260	-0.016385	0.087529
	-0.024148					
y199	0.040103	-0.001750	-0.044781	-0.016439	-0.051775	0.031368
	-0.040534	0.048693	0.066089	0.009232	-0.078867	-0.031029
	-0.022758	0.023458	-0.008040	0.054892	-0.001040	-0.018838
	-0.012824	-0.100012	0.010008	0.017445	0.022876	-0.049810
	0.017739	0.033392	-0.073148	-0.002246	0.018024	0.051949
	-0.019448					
z199	0.003371	-0.030597	-0.019755	0.049107	0.012416	-0.058544
	0.028609	0.033950	0.038038	0.009961	-0.049920	-0.009021
	0.021110	-0.071624	-0.018650	0.021995	0.010111	0.028299
	-0.054074	-0.001779	-0.062616	0.006655	0.044081	0.013722
	0.002510	0.023567	-0.002685	0.035175	0.034170	0.067349
	0.035598					
x200	-0.072433	0.020059	0.046309	0.000902	-0.038532	-0.025460
	0.017886	0.084147	-0.070432	0.011736	-0.025892	-0.010604
	-0.026836	0.022065	0.024028	-0.022377	-0.014525	0.007767
	-0.092756	0.013462	0.049021	-0.005538	0.116416	-0.024973
	0.051197	0.018230	0.040774	-0.004446	0.024731	0.061211
	-0.016332					

y200	0.037659	-0.005061	-0.045530	-0.002554	-0.052094	0.040126
	-0.035207	0.042076	0.054576	0.013410	-0.095597	-0.027305
	-0.029556	0.016493	-0.027081	0.046635	0.013098	-0.009447
	-0.022752	-0.060515	0.022024	0.031008	0.049982	0.012205
	0.008090	0.043909	-0.074383	-0.008134	0.038175	0.027158
	-0.038806					
z200	-0.013382	-0.032473	-0.016041	0.051866	0.000376	-0.070914
	0.017772	0.042585	0.032073	0.011796	-0.044146	-0.005865
	0.008882	-0.078051	-0.034560	-0.009250	0.028742	0.004345
	-0.034228	0.022590	-0.057169	0.034649	0.069819	0.050592
	-0.017420	0.019526	-0.018982	0.017416	0.053437	0.047253
	0.007206					
x201	-0.078686	0.018335	0.051184	0.013731	-0.051682	-0.050809
	0.014134	0.083404	-0.065023	0.002611	-0.015791	-0.022931
	-0.024273	0.026647	0.083345	-0.003703	-0.004451	0.012487
	-0.083743	0.019533	0.044285	-0.001696	0.116087	-0.026286
	-0.018700	-0.002586	0.050505	0.025728	0.060696	0.007665
	-0.025987					
y201	0.038996	-0.008048	-0.046694	0.006132	-0.046518	0.039979
	-0.029636	0.028520	0.055656	0.008647	-0.101424	-0.034809
	-0.027368	0.024108	-0.021040	0.035645	0.001818	-0.017930
	-0.006917	-0.057353	0.053874	0.044484	0.058558	0.088253
	-0.031118	0.048205	-0.074031	0.012744	0.051359	0.026883
	-0.055297					
z201	-0.024589	-0.035885	-0.008667	0.051981	-0.013114	-0.094464
	0.005483	0.044927	0.042407	0.009571	-0.025271	-0.013939
	0.011154	-0.068306	-0.013602	-0.037717	0.021810	-0.033736
	-0.002569	0.011428	-0.030980	0.059502	0.071722	0.099277
	-0.059720	0.019646	-0.025872	0.037972	0.068447	0.034693
	-0.028008					
x202	-0.077566	0.019819	0.047333	0.024022	-0.056306	-0.062045
	0.009706	0.087238	-0.038498	0.002042	0.016484	-0.015298
	-0.010212	0.019964	0.122632	-0.000808	0.002417	0.012341
	-0.062941	0.028640	0.008832	0.019166	0.105229	-0.031946
	-0.054309	-0.022869	0.036366	0.039480	0.053892	-0.002308
	-0.023168					
y202	0.039639	-0.008573	-0.048095	0.011076	-0.034428	0.047386
	-0.022564	0.012736	0.046851	0.004119	-0.111817	-0.031746
	-0.026503	0.033305	-0.020076	0.016559	0.006954	-0.042184
	0.002494	-0.039076	0.059780	0.061134	0.056137	0.177018
	-0.074352	0.033591	-0.084638	0.029676	0.019001	-0.037568
	-0.079411					

z202	-0.039392	-0.032203	0.000428	0.046864	-0.019172	-0.107155
	-0.007364	0.046396	0.049078	0.014499	-0.000230	0.006560
	0.014889	-0.061463	-0.013144	-0.085535	0.036047	-0.069525
	0.025629	0.026392	-0.049228	0.104668	0.065468	0.156613
	-0.112942	0.024175	-0.023858	0.049956	0.027671	-0.017818
	-0.059095					
x203	-0.069651	0.018605	0.039431	0.019684	-0.062374	-0.067513
	0.005258	0.090701	-0.033085	-0.008395	0.043236	0.018273
	-0.000575	0.036799	0.132904	-0.007024	0.003291	-0.021267
	-0.038395	0.047385	-0.025555	0.028780	0.054882	-0.012132
	-0.033270	0.016545	0.033479	0.044368	0.084379	0.031692
	0.014647					
y203	0.040051	0.005954	-0.058523	0.014915	-0.008531	0.071496
	-0.009401	0.005869	0.037059	-0.000739	-0.110422	-0.033752
	-0.020302	0.017428	-0.044302	0.012304	0.026538	-0.026185
	-0.009922	-0.017458	0.075235	0.031051	0.067795	0.141382
	-0.061929	0.012304	-0.070048	-0.021133	-0.021970	-0.074905
	-0.043379					
z203	-0.050543	-0.016727	-0.002758	0.041547	-0.016089	-0.106884
	-0.007371	0.040412	0.054609	0.007584	0.044968	0.024464
	0.016810	-0.072823	-0.034970	-0.104462	0.065111	-0.072290
	0.023197	0.061564	-0.071493	0.105561	0.048367	0.105842
	-0.057245	0.059968	-0.013937	-0.014460	-0.022801	-0.038047
	0.013743					
x204	-0.058637	0.018991	0.034817	0.017569	-0.055341	-0.067780
	0.002466	0.094237	-0.039022	-0.012401	0.054080	0.037919
	0.008596	0.052008	0.130983	-0.000935	-0.003521	-0.032604
	-0.021346	0.034979	-0.049426	0.005618	0.019772	-0.000489
	-0.031353	0.010151	0.022809	0.032453	0.090224	0.024424
	0.022946					
y204	0.044089	0.019901	-0.068939	0.017791	0.015078	0.092523
	-0.002769	0.004114	0.035720	-0.002029	-0.094847	-0.042262
	-0.006269	0.003133	-0.044345	0.016504	0.033463	-0.019722
	-0.010888	-0.014217	0.100097	0.001267	0.062317	0.104282
	-0.040888	0.013633	-0.062765	-0.037055	-0.036787	-0.075668
	-0.028591					
z204	-0.054566	0.000843	0.000036	0.044157	-0.003723	-0.110321
	-0.007468	0.033557	0.064360	-0.001817	0.088064	0.019052
	0.018792	-0.079320	-0.035990	-0.098746	0.074525	-0.044469
	0.027365	0.042875	-0.066189	0.076538	0.030298	0.020392
	-0.022343	0.074010	-0.017691	-0.035095	-0.020438	-0.038757
	0.059786					

x205	-0.046844	0.018638	0.029809	0.015771	-0.048781	-0.064614
	-0.000933	0.090963	-0.047764	-0.017964	0.053149	0.053049
	0.013535	0.062677	0.112204	-0.000437	-0.003293	-0.043096
	-0.006953	0.026121	-0.059373	-0.010244	0.000507	0.016221
	-0.032479	-0.017953	-0.001265	0.017252	0.074748	0.030921
	0.014184					
y205	0.047503	0.033268	-0.075695	0.019889	0.035610	0.108187
	-0.002516	0.010414	0.039667	-0.002671	-0.079930	-0.050674
	0.008061	-0.020137	-0.044268	0.011048	0.041828	-0.011595
	-0.010344	0.002332	0.114325	-0.022351	0.051101	0.075336
	-0.027497	0.026690	-0.037398	-0.048502	-0.030820	-0.060153
	0.007623					
z205	-0.048808	0.011427	0.008638	0.053437	0.002115	-0.129404
	-0.014424	0.017963	0.073748	-0.019035	0.112753	-0.007845
	0.020871	-0.043936	-0.030903	-0.084035	0.078253	-0.042286
	0.020337	0.012737	-0.024274	0.052559	0.016142	-0.012698
	0.006662	0.051115	-0.018035	0.000341	-0.010208	-0.015340
	0.017491					
x206	-0.032517	0.020736	0.024259	0.014292	-0.038134	-0.059808
	-0.003826	0.083963	-0.045728	-0.023985	0.051166	0.059270
	0.017230	0.060917	0.110845	0.009688	-0.004358	-0.054558
	0.002641	0.013169	-0.058033	-0.022697	-0.013268	0.042097
	-0.043212	-0.031298	-0.021973	0.008062	0.059552	0.023150
	0.013039					
y206	0.049466	0.044016	-0.080214	0.020906	0.050375	0.122246
	-0.010372	0.020449	0.042349	-0.006078	-0.074862	-0.058560
	0.019087	-0.039559	-0.044109	0.002058	0.045191	-0.009292
	-0.002134	0.011271	0.114134	-0.032616	0.042360	0.038199
	-0.014169	0.032987	-0.002814	-0.040787	0.005402	-0.027355
	0.042568					
z206	-0.049112	0.020776	0.011577	0.058339	-0.004647	-0.130414
	-0.005067	-0.003773	0.066223	-0.020632	0.107442	-0.023327
	0.020224	-0.026783	-0.044513	-0.086362	0.064771	-0.024723
	0.009174	-0.001070	-0.012254	0.029530	0.041127	-0.026588
	0.043567	0.022765	-0.047218	-0.019412	-0.034058	0.002946
	-0.018879					
x207	-0.017146	0.024588	0.018888	0.013258	-0.023684	-0.053005
	-0.007930	0.073964	-0.035433	-0.032059	0.049583	0.061653
	0.018649	0.045775	0.115160	0.019452	-0.002588	-0.061481
	0.009400	0.005740	-0.054015	-0.024202	-0.022626	0.059428
	-0.044623	-0.039944	-0.036072	0.001814	0.048040	0.011933
	0.021165					

y207	0.051710	0.053257	-0.080260	0.021060	0.055808	0.132673
	-0.020980	0.030550	0.041338	-0.005519	-0.076499	-0.062429
	0.023379	-0.048619	-0.042313	-0.005954	0.045281	-0.009868
	0.012998	0.015339	0.101306	-0.037473	0.032075	0.011949
	-0.003602	0.038567	0.016820	-0.023129	0.052755	0.023156
	0.046768					
z207	-0.051584	0.030921	0.004930	0.053205	-0.012479	-0.118264
	0.007098	-0.003619	0.054578	-0.019207	0.100049	-0.036888
	0.018063	-0.009457	-0.067722	-0.098185	0.026953	0.015218
	0.001010	-0.005094	-0.016909	0.024329	0.061510	-0.047639
	0.068980	0.010072	-0.063055	-0.037334	-0.054489	0.024138
	-0.010872					
x208	0.000187	0.028190	0.014481	0.009879	-0.008248	-0.047490
	-0.013659	0.061725	-0.019069	-0.041523	0.043656	0.060317
	0.017020	0.027541	0.116329	0.028901	0.000638	-0.061398
	0.015837	-0.007725	-0.046099	-0.026543	-0.026910	0.068003
	-0.046493	-0.042071	-0.042376	0.001246	0.044702	0.018798
	0.024058					
y208	0.056253	0.063913	-0.076262	0.021586	0.054496	0.138072
	-0.034974	0.046900	0.035584	0.002900	-0.070344	-0.057765
	0.022397	-0.047878	-0.036652	-0.013103	0.043649	-0.003143
	0.027303	0.020082	0.087923	-0.039019	0.012439	-0.016073
	0.005900	0.034150	0.018526	0.010033	0.079093	0.067034
	0.039047					
z208	-0.055990	0.043681	-0.004174	0.047158	-0.009714	-0.093488
	0.018338	-0.002205	0.049336	-0.012407	0.094054	-0.042024
	0.015543	0.014400	-0.074097	-0.099938	0.009853	0.024999
	-0.009971	0.003325	-0.029781	0.029047	0.061175	-0.051243
	0.091042	0.006250	-0.064480	-0.061242	-0.049019	-0.009251
	-0.013367					
x209	0.017466	0.031367	0.008975	0.001760	0.005133	-0.041652
	-0.018281	0.046131	-0.004834	-0.054281	0.028761	0.053791
	0.006039	0.023930	0.111875	0.032107	-0.000135	-0.059406
	0.030880	-0.006232	-0.054818	-0.025760	-0.011965	0.069198
	-0.046246	-0.061687	-0.056081	-0.003124	0.044099	0.039310
	0.020950					
y209	0.058026	0.079313	-0.069075	0.029055	0.050550	0.138161
	-0.047125	0.070465	0.028001	0.017679	-0.050585	-0.038928
	0.012721	-0.040120	-0.020817	-0.019360	0.044329	0.004266
	0.035238	0.040326	0.060395	-0.028487	-0.014614	-0.022921
	0.007972	0.018103	-0.007793	0.038974	0.072788	0.071507
	0.008343					

z209	-0.051576	0.051881	-0.004621	0.045312	-0.005464	-0.071299
	0.023848	0.005787	0.053171	0.002906	0.095974	-0.034084
	0.027335	0.010938	-0.056512	-0.091769	0.005958	0.034448
	-0.038645	-0.011979	0.000080	0.024804	0.023970	-0.058771
	0.103198	0.042796	-0.031828	-0.058633	-0.034508	-0.052640
	-0.003053					
x210	0.031292	0.031075	0.004248	-0.003691	0.022674	-0.024768
	-0.011463	0.026984	0.004788	-0.064487	0.014909	0.052237
	-0.001329	0.024601	0.106826	0.032171	-0.005272	-0.054902
	0.044071	0.005573	-0.053628	-0.019299	0.011510	0.048867
	-0.037593	-0.071513	-0.067097	-0.024927	0.034595	0.035474
	0.019192					
y210	0.054873	0.094203	-0.062027	0.042349	0.046703	0.141117
	-0.053210	0.095748	0.020275	0.037251	-0.019070	-0.008045
	0.000979	-0.026234	0.004493	-0.022973	0.037484	0.004758
	0.026249	0.058695	0.011542	0.000552	-0.045640	-0.018207
	0.008506	-0.005037	-0.040335	0.057424	0.038484	0.027261
	-0.021892					
z210	-0.038066	0.059816	-0.002777	0.037772	-0.007474	-0.067730
	0.010332	0.023364	0.057540	0.014620	0.091462	-0.020604
	0.034188	0.011400	-0.036485	-0.080332	0.012189	0.040271
	-0.060944	-0.030593	0.006186	0.024834	-0.032694	-0.040396
	0.081692	0.079694	0.010469	-0.015009	-0.010327	-0.057031
	-0.022520					
x211	0.044240	0.022959	0.004621	-0.003504	0.040617	-0.014598
	0.001808	0.018617	0.017915	-0.077312	0.010580	0.052374
	0.000743	0.033513	0.102087	0.044715	-0.024314	-0.037644
	0.046590	0.002111	-0.025837	-0.008879	0.016848	0.001010
	-0.029904	-0.041095	-0.037221	-0.044857	0.049954	0.024389
	0.038124					
y211	0.049128	0.105548	-0.052872	0.060807	0.041407	0.138192
	-0.055736	0.130954	0.013812	0.053209	0.019892	0.025086
	-0.000559	-0.003450	0.032482	-0.027470	-0.002360	-0.000852
	0.007017	0.059416	-0.061690	0.041266	-0.084523	-0.021817
	-0.005024	-0.030261	-0.041729	0.070103	0.007885	-0.034074
	-0.016114					
z211	-0.020883	0.073833	-0.003332	0.027675	-0.008198	-0.052155
	-0.014563	0.031795	0.054427	0.026684	0.080194	0.001141
	0.032896	0.015300	-0.011796	-0.081908	0.029799	0.034883
	-0.073476	-0.030987	-0.020806	0.036105	-0.072139	0.001165
	0.050895	0.066767	0.006972	0.029344	-0.025504	-0.064213
	-0.071995					

x212	0.049363	0.020648	0.004047	0.004198	0.046069	-0.020398
	0.004396	0.015966	0.022790	-0.097113	-0.000966	0.048531
	0.021082	0.028051	0.094514	0.039035	-0.021414	-0.030324
	0.038220	-0.005076	-0.025919	-0.004174	0.000278	-0.010275
	-0.037182	-0.019140	-0.049114	-0.035187	0.057489	0.021972
	0.040654					
y212	0.049039	0.099645	-0.043777	0.072563	0.042278	0.131306
	-0.048012	0.148970	0.014601	0.033692	0.028331	0.033054
	0.021676	0.020532	0.026079	-0.038726	-0.031058	-0.003783
	0.020327	0.038815	-0.070256	0.052131	-0.094501	-0.015347
	-0.034989	-0.038028	0.001372	0.025196	0.002209	-0.027227
	-0.010091					
z212	-0.009713	0.073057	0.001249	0.020034	-0.004914	-0.035658
	-0.028444	0.039034	0.052151	0.033810	0.078225	0.009789
	0.024719	0.033939	-0.003668	-0.070943	0.022222	0.034431
	-0.064368	-0.032909	-0.020789	0.027531	-0.075023	0.009303
	0.026886	0.063434	0.035978	0.006436	-0.025394	-0.055494
	-0.076352					
x213	0.048242	0.015713	0.005922	0.008215	0.050602	-0.028624
	0.004905	0.017465	0.025561	-0.106994	-0.012101	0.046943
	0.028709	0.017750	0.077294	0.041843	-0.022287	-0.020017
	0.038794	0.001569	-0.020879	0.020435	-0.001835	-0.006858
	-0.031210	-0.009282	-0.051906	-0.013994	0.028804	-0.003941
	0.035488					
y213	0.043579	0.087775	-0.036861	0.080670	0.039303	0.123778
	-0.038299	0.156178	0.023480	0.027397	0.033524	0.032387
	0.033951	0.026230	0.021675	-0.034658	-0.047831	-0.005881
	0.036756	0.038865	-0.059676	0.053106	-0.099053	0.001808
	-0.055810	-0.029225	0.029432	0.013033	-0.027274	-0.020845
	0.002311					
z213	0.002521	0.069782	0.005677	0.013926	-0.002444	-0.017239
	-0.040252	0.038038	0.053755	0.039814	0.075569	0.017529
	0.020604	0.045994	0.016096	-0.061309	0.025436	0.031531
	-0.056837	-0.033640	-0.009640	0.000800	-0.092218	0.008838
	0.003272	0.080659	0.044691	-0.014091	-0.011275	-0.039094
	-0.078095					
x214	0.046191	0.012016	0.007750	0.011425	0.055078	-0.038295
	0.000486	0.016620	0.020436	-0.117558	-0.021731	0.047650
	0.030376	0.016898	0.053062	0.052042	-0.012071	-0.009275
	0.045826	0.013126	-0.020914	0.033942	-0.003543	-0.007162
	-0.025682	-0.000054	-0.044431	0.001860	0.010545	-0.019440
	0.026857					

y214	0.037905	0.074676	-0.030498	0.086466	0.034675	0.112760
	-0.028968	0.156302	0.027208	0.020003	0.036732	0.026526
	0.044950	0.029061	0.016428	-0.028721	-0.059929	-0.008928
	0.049691	0.037852	-0.053952	0.050880	-0.090243	0.016605
	-0.067627	-0.019800	0.049111	-0.000192	-0.043204	-0.007968
	0.015446					
z214	0.014495	0.065645	0.010190	0.008185	-0.001181	-0.000039
	-0.046711	0.033546	0.060753	0.043926	0.072501	0.019566
	0.020807	0.047048	0.036671	-0.058189	0.020322	0.031047
	-0.057905	-0.040610	0.003736	-0.016484	-0.102561	0.005977
	-0.016626	0.100627	0.038637	-0.024113	-0.002394	-0.019149
	-0.071427					
x215	0.045495	0.010168	0.010539	0.010607	0.055393	-0.044420
	-0.009417	0.013447	0.009830	-0.123109	-0.026710	0.053394
	0.025202	0.023025	0.032538	0.062356	0.000847	0.002765
	0.050483	0.020900	-0.023997	0.023932	-0.001624	-0.019628
	-0.013450	0.016785	-0.034250	0.016845	0.009394	-0.020199
	0.023727					
y215	0.032531	0.061783	-0.024649	0.088526	0.025828	0.102302
	-0.021053	0.150488	0.024194	0.013941	0.038708	0.020817
	0.051401	0.029233	0.010368	-0.023984	-0.069723	-0.014835
	0.055632	0.035575	-0.048941	0.037766	-0.071250	0.025716
	-0.062473	-0.009367	0.057200	-0.017094	-0.038984	0.009069
	0.027904					
z215	0.025091	0.061662	0.013486	0.003099	0.002316	0.014203
	-0.047094	0.025660	0.070102	0.041545	0.065996	0.014403
	0.027153	0.041199	0.051390	-0.053726	0.015210	0.026159
	-0.062249	-0.047393	0.023691	-0.013649	-0.108438	0.009545
	-0.031247	0.110844	0.020104	-0.027899	0.000910	-0.003517
	-0.061445					
x216	0.048061	0.008932	0.012332	0.008710	0.053654	-0.042694
	-0.016285	-0.000106	0.002081	-0.124963	-0.025312	0.060431
	0.017511	0.032908	0.028072	0.071111	0.007815	0.002425
	0.059467	0.020264	-0.021223	0.015827	0.003461	-0.018543
	0.001999	0.021398	-0.044508	0.037103	-0.002569	-0.066827
	0.020103					
y216	0.028895	0.050894	-0.022126	0.087691	0.012953	0.099271
	-0.010162	0.128961	0.018233	0.009462	0.039393	0.021204
	0.051473	0.025531	0.005021	-0.022788	-0.071871	-0.031711
	0.062304	0.039795	-0.042127	0.026358	-0.049187	0.042357
	-0.035210	-0.015887	0.037790	-0.030590	-0.043382	-0.024672
	0.046655					

z216	0.032119	0.058221	0.017999	-0.003757	0.008787	0.021380
	-0.050621	0.022782	0.071904	0.034173	0.048034	0.008106
	0.036595	0.031861	0.048221	-0.045075	0.020101	0.022239
	-0.074687	-0.042111	0.034714	-0.012038	-0.114055	0.003763
	-0.038482	0.117386	0.004552	-0.036071	0.019239	0.046622
	-0.044355					
x217	0.051699	0.005952	0.013179	0.006390	0.055328	-0.041432
	-0.021430	-0.005791	-0.008737	-0.128795	-0.024576	0.058764
	0.012586	0.038868	0.023197	0.072137	0.016084	0.018657
	0.059869	0.010720	-0.017040	0.012418	0.020706	-0.029888
	-0.008553	0.052452	-0.029172	0.036293	-0.009206	-0.058262
	-0.015444					
y217	0.024674	0.037404	-0.020594	0.085619	0.001609	0.090539
	0.002010	0.113736	0.005476	0.003934	0.037484	0.009491
	0.051084	0.013352	-0.000151	-0.024264	-0.073951	-0.030654
	0.064311	0.031169	-0.035209	0.021044	-0.011792	0.045400
	-0.034252	0.004655	0.046473	-0.062764	-0.037814	0.000659
	0.023106					
z217	0.034291	0.054834	0.022579	-0.013759	0.012681	0.026333
	-0.053813	0.015213	0.074867	0.028261	0.033339	0.001118
	0.043870	0.028540	0.043146	-0.027016	0.022676	0.001157
	-0.085647	-0.042638	0.049116	-0.016114	-0.124793	0.010205
	-0.008050	0.096512	-0.026495	-0.018011	0.038057	0.054001
	-0.007034					
x218	0.058234	0.008020	0.012776	0.004864	0.053073	-0.036956
	-0.025248	-0.020603	-0.013950	-0.125137	-0.021383	0.061653
	0.010860	0.041147	0.026173	0.072517	0.015911	0.030932
	0.050859	-0.002284	-0.019156	-0.002735	0.027978	-0.036439
	0.005593	0.062017	-0.043930	0.038575	-0.015433	-0.074570
	-0.011244					
y218	0.024246	0.029451	-0.021507	0.082818	-0.015896	0.083909
	0.015058	0.080740	-0.001195	0.003968	0.034478	0.005790
	0.049299	-0.001044	0.006011	-0.021024	-0.073163	-0.038201
	0.060700	0.021677	-0.032425	0.006214	0.016186	0.048576
	0.000749	0.001547	0.021607	-0.075968	-0.028121	-0.020638
	0.044520					
z218	0.029864	0.047909	0.026203	-0.024805	0.020339	0.031715
	-0.053595	0.018350	0.062130	0.016952	0.012219	-0.000178
	0.045812	0.017202	0.009412	-0.020094	0.040638	-0.006383
	-0.082444	-0.020190	0.054422	-0.007720	-0.114493	0.008574
	0.010888	0.087814	-0.033090	-0.023275	0.043548	0.085319
	-0.000299					

x219	0.062870	0.009978	0.012065	0.006310	0.050718	-0.033123
	-0.024479	-0.020462	-0.012600	-0.115196	-0.012108	0.056345
	0.012054	0.035964	0.032971	0.064503	-0.003419	0.053687
	0.034324	-0.026732	-0.009063	-0.004078	0.035851	-0.050105
	0.005878	0.080375	-0.040351	0.029109	-0.020269	-0.038310
	-0.021519					
y219	0.021351	0.020455	-0.021772	0.079664	-0.034809	0.069197
	0.029198	0.063963	-0.005580	0.006792	0.032928	-0.005727
	0.046241	-0.028371	0.006047	-0.025859	-0.084855	-0.029732
	0.055172	0.006866	-0.020893	0.003402	0.053767	0.037300
	0.019824	0.019332	0.032047	-0.103639	-0.007764	0.014509
	0.048049					
z219	0.027219	0.043102	0.028253	-0.031787	0.023338	0.037306
	-0.049887	0.006825	0.045636	0.003786	-0.001662	0.004095
	0.052664	0.015375	-0.004087	-0.008098	0.056858	-0.020579
	-0.066559	-0.008884	0.041904	-0.010312	-0.097339	0.009675
	0.039412	0.079178	-0.061253	-0.013476	0.048459	0.068345
	0.009334					
x220	0.064637	-0.004926	0.054598	-0.008027	0.043477	-0.068047
	-0.057694	0.006258	0.016580	-0.065006	-0.017473	-0.000829
	0.013794	-0.081448	-0.002167	0.056102	-0.025362	0.087656
	0.029719	-0.020182	0.069346	0.048522	-0.007474	-0.042223
	0.026055	0.043809	-0.012862	-0.064581	0.091680	0.034116
	-0.124073					
y220	0.006609	-0.013164	-0.019435	0.071576	-0.046024	0.060288
	0.092339	-0.092609	-0.106978	0.044100	0.041146	-0.012478
	-0.003129	0.007035	0.097642	-0.052546	0.150272	-0.056872
	-0.129668	-0.062653	-0.013823	0.075447	0.041798	-0.028303
	-0.030948	0.013066	-0.048227	0.022894	0.023429	-0.081902
	-0.003225					
z220	0.030940	0.052341	0.016566	-0.129201	0.005837	0.017790
	-0.078401	0.017394	-0.020850	-0.016020	-0.006258	0.061134
	0.034282	-0.119138	-0.033006	-0.041910	0.065470	0.003368
	-0.069489	-0.014727	0.044693	-0.040527	0.001378	0.024491
	0.003215	0.074567	0.001261	0.085040	0.011904	-0.152669
	0.149664					
x221	0.066421	-0.007848	0.058206	0.002190	0.039439	-0.056636
	-0.048988	0.008724	0.024087	-0.053591	-0.014970	-0.013973
	0.015554	-0.065817	-0.001068	0.034820	-0.014402	0.067016
	-0.001258	-0.009437	0.060166	0.040453	-0.011942	-0.055302
	0.015413	0.031141	-0.010586	-0.060503	0.062119	0.027189
	-0.116471					

y221	0.005394	-0.012717	-0.024400	0.064236	-0.047651	0.055419
	0.084461	-0.086273	-0.098542	0.034386	0.041017	-0.004923
	-0.005685	-0.005585	0.082735	-0.042056	0.118395	-0.043327
	-0.105232	-0.062043	-0.012186	0.061235	0.053622	-0.033581
	-0.024015	0.004240	-0.032352	0.004116	0.013435	-0.042438
	-0.010221					
z221	0.026813	0.042105	0.020899	-0.120940	0.008414	0.028169
	-0.073344	0.015723	-0.034906	-0.016691	0.000126	0.054761
	0.016691	-0.116036	-0.044189	-0.037891	0.059085	0.005861
	-0.067796	-0.022231	0.041740	-0.028388	0.024768	0.023048
	-0.004420	0.032445	0.003874	0.073414	-0.000241	-0.094263
	0.110762					
x222	0.069429	-0.007076	0.056524	0.010732	0.032356	-0.047604
	-0.035555	0.012284	0.030314	-0.044256	-0.013774	-0.013481
	0.018585	-0.048752	0.000189	0.014064	-0.001312	0.030650
	-0.026158	-0.001550	0.049273	0.040617	-0.020602	-0.060509
	0.005224	0.002653	-0.003340	-0.053998	0.043556	0.028563
	-0.078420					
y222	0.004935	-0.011954	-0.030417	0.058449	-0.048489	0.052351
	0.076876	-0.081270	-0.091005	0.025285	0.040616	0.002362
	-0.007902	-0.012963	0.070924	-0.033212	0.091615	-0.029996
	-0.080612	-0.059467	-0.010414	0.048119	0.060892	-0.036410
	-0.016250	-0.002768	-0.023012	-0.009884	0.011761	-0.008463
	-0.016716					
z222	0.025095	0.035132	0.021281	-0.110631	0.009194	0.038933
	-0.064226	0.015475	-0.044768	-0.017416	0.002814	0.054549
	0.004673	-0.105806	-0.047130	-0.035084	0.052597	-0.001875
	-0.061153	-0.027176	0.039491	-0.018377	0.037582	0.018200
	-0.015227	-0.010626	-0.001095	0.056210	-0.003666	-0.039282
	0.093710					
x223	0.074172	-0.006568	0.052098	0.019806	0.027721	-0.038351
	-0.027030	0.010830	0.031217	-0.040154	-0.011454	-0.003649
	0.022573	-0.034698	-0.000437	-0.002150	0.014635	0.012370
	-0.043219	0.001661	0.047246	0.041977	-0.021796	-0.042378
	0.008504	-0.029491	-0.006238	-0.035507	0.037663	-0.021690
	-0.036189					
y223	0.005073	-0.010990	-0.036080	0.052696	-0.048867	0.049923
	0.071272	-0.078983	-0.083435	0.017370	0.039684	0.011056
	-0.009907	-0.017506	0.061763	-0.025113	0.068928	-0.020288
	-0.058461	-0.055149	-0.008319	0.036536	0.060594	-0.041008
	-0.004228	-0.009823	-0.020292	-0.018455	0.011023	0.016942
	-0.011942					

z223	0.025108	0.027892	0.021759	-0.098612	0.011066	0.048978
	-0.056060	0.015646	-0.054806	-0.018375	0.001339	0.053391
	-0.002436	-0.090995	-0.045698	-0.029492	0.043935	-0.003137
	-0.055133	-0.029232	0.037837	-0.010130	0.046102	0.014363
	-0.023167	-0.049879	-0.002699	0.029416	-0.003117	-0.027744
	0.086919					
x224	0.074875	-0.009197	0.047205	0.025475	0.024243	-0.035334
	-0.020609	0.017158	0.026618	-0.032961	-0.005231	-0.007245
	0.028938	-0.020239	-0.014435	-0.016522	0.022091	0.004642
	-0.057354	-0.002746	0.049701	0.045232	-0.007546	-0.036390
	0.000966	-0.051469	0.007027	-0.041669	0.024679	-0.041943
	-0.030237					
y224	0.004072	-0.010823	-0.041916	0.045709	-0.049370	0.046480
	0.065396	-0.075778	-0.075062	0.009620	0.043094	0.014378
	-0.009631	-0.020093	0.052356	-0.018734	0.050901	-0.012267
	-0.038963	-0.056260	-0.006806	0.029067	0.058851	-0.044602
	0.007477	-0.013264	-0.012057	-0.026278	0.010093	0.031887
	-0.011865					
z224	0.020397	0.016849	0.020440	-0.090363	0.011179	0.051953
	-0.051599	0.023524	-0.063521	-0.017967	0.007171	0.037017
	-0.002242	-0.075528	-0.055645	-0.028448	0.031768	0.000726
	-0.049871	-0.052473	0.041902	0.002211	0.061842	0.006886
	-0.035363	-0.076656	0.023840	-0.007269	-0.009903	-0.010793
	0.058540					
x225	0.075394	-0.010450	0.045824	0.029641	0.019625	-0.032544
	-0.012344	0.023784	0.021663	-0.027268	0.000247	-0.009793
	0.022955	-0.009996	-0.020594	-0.023364	0.031223	0.010641
	-0.063337	-0.011545	0.050983	0.041681	-0.007034	-0.021614
	-0.013240	-0.041771	-0.004494	-0.037650	0.034402	-0.055740
	-0.038402					
y225	0.003168	-0.011216	-0.046568	0.039969	-0.048813	0.043129
	0.061065	-0.073461	-0.067190	0.002730	0.044695	0.019602
	-0.010095	-0.022071	0.045945	-0.013120	0.035594	-0.005866
	-0.024918	-0.048450	-0.004913	0.023063	0.048697	-0.049471
	0.016026	-0.011399	-0.007066	-0.030517	0.005805	0.046565
	-0.009856					
z225	0.015887	0.007288	0.022074	-0.082593	0.009624	0.053162
	-0.043479	0.032894	-0.071090	-0.016881	0.012144	0.022319
	-0.011671	-0.064964	-0.056008	-0.020151	0.022340	0.010014
	-0.044216	-0.067034	0.040653	0.006852	0.059578	0.004222
	-0.061424	-0.068132	0.036533	-0.034666	0.010785	0.004123
	0.026229					

x226	0.075693	-0.010832	0.042425	0.033915	0.016134	-0.032364
	-0.005415	0.025047	0.018960	-0.016565	0.001246	-0.016234
	0.018219	0.002265	-0.023880	-0.025782	0.034594	0.008093
	-0.062267	-0.004833	0.043033	0.035010	-0.010086	-0.019287
	-0.024496	-0.039555	-0.009220	-0.033710	-0.000442	-0.050011
	-0.033385					
y226	0.002687	-0.011826	-0.049885	0.035967	-0.047189	0.039651
	0.056406	-0.069332	-0.060939	-0.003590	0.046567	0.025309
	-0.009404	-0.024296	0.041316	-0.008797	0.025349	0.001470
	-0.012670	-0.039809	0.000765	0.018864	0.040169	-0.056211
	0.016949	-0.008557	-0.002074	-0.033507	0.007546	0.051130
	-0.006967					
z226	0.011182	0.000533	0.019778	-0.069809	0.010335	0.048054
	-0.038307	0.037877	-0.076766	-0.013544	0.012674	0.010976
	-0.017378	-0.053133	-0.049404	-0.011685	0.013520	0.009574
	-0.029642	-0.058674	0.035402	0.010747	0.057192	-0.003252
	-0.097742	-0.053866	0.054813	-0.043046	-0.005042	0.031612
	0.008643					
x227	0.076393	-0.012875	0.041653	0.037108	0.011965	-0.034109
	-0.000997	0.032979	0.020498	-0.004408	0.005358	-0.024758
	0.015708	0.010230	-0.028063	-0.030616	0.039110	0.007548
	-0.059503	-0.014837	0.037215	0.034216	-0.011273	-0.023201
	-0.034589	-0.031907	0.000851	-0.030269	-0.009860	-0.021385
	-0.037416					
y227	0.002173	-0.012289	-0.053988	0.031519	-0.045163	0.037115
	0.053403	-0.069466	-0.056280	-0.008884	0.046834	0.030931
	-0.009066	-0.025097	0.037741	-0.003777	0.019231	0.006552
	-0.002202	-0.023850	0.003846	0.013866	0.028846	-0.057659
	0.022201	-0.007861	-0.005201	-0.034732	0.004077	0.037416
	0.002304					
z227	0.009463	-0.008909	0.019626	-0.058357	0.007818	0.043180
	-0.036907	0.043934	-0.077807	-0.004939	0.015943	-0.001986
	-0.014412	-0.041929	-0.051193	-0.014575	0.017108	0.007936
	-0.014012	-0.068582	0.033159	0.023567	0.049115	-0.024801
	-0.115877	-0.038370	0.065873	-0.052020	-0.002276	0.072519
	-0.018031					
x228	0.077530	-0.014604	0.043777	0.038268	0.009569	-0.030083
	0.002140	0.034319	0.022513	0.003759	0.008229	-0.029875
	0.013879	0.016540	-0.029127	-0.034151	0.038227	-0.002324
	-0.052666	-0.024142	0.028582	0.026376	-0.015146	-0.024147
	-0.019760	-0.035330	0.004791	-0.031891	-0.018332	-0.000674
	-0.035898					

y228	0.001053	-0.013645	-0.058123	0.027037	-0.043212	0.031449
	0.051324	-0.064218	-0.053082	-0.013824	0.047634	0.032787
	-0.005726	-0.027500	0.034901	-0.000866	0.018157	0.015652
	0.006700	-0.009047	0.009711	0.010772	0.017333	-0.061714
	0.015423	0.002736	-0.002048	-0.040353	0.001416	0.029142
	0.004535					
z228	0.008940	-0.016220	0.020238	-0.048044	0.004817	0.045819
	-0.034450	0.039849	-0.079932	-0.000033	0.016296	-0.009111
	-0.005300	-0.028470	-0.048203	-0.017649	0.022800	-0.004189
	0.006906	-0.067055	0.029156	0.021759	0.030942	-0.035468
	-0.094922	-0.032353	0.060776	-0.065027	-0.009492	0.083175
	-0.025210					
x229	0.076368	-0.010927	0.039154	0.040983	0.006893	-0.025300
	0.007020	0.031512	0.020925	0.009168	0.006839	-0.020827
	0.002783	0.021701	-0.032474	-0.044412	0.039679	0.005597
	-0.041141	-0.014888	0.019692	0.013847	-0.004803	-0.003861
	-0.001802	-0.041074	-0.005661	-0.031585	-0.015217	-0.026390
	-0.001525					
y229	0.002879	-0.017723	-0.058679	0.020160	-0.043135	0.026776
	0.046779	-0.063809	-0.047581	-0.020087	0.047179	0.030117
	-0.000763	-0.031662	0.034955	0.004615	0.024157	0.014730
	0.019936	0.005890	0.019598	0.000261	0.002604	-0.064635
	0.020614	-0.000544	-0.007505	-0.036351	-0.012058	0.014661
	0.013849					
z229	0.006132	-0.016768	0.015970	-0.036298	-0.002809	0.043271
	-0.033598	0.028858	-0.077329	0.000797	0.011723	-0.006679
	-0.008608	-0.018305	-0.039807	-0.020041	0.033382	0.000908
	0.030941	-0.031070	0.032216	0.007344	0.025229	-0.024656
	-0.073648	-0.028586	0.044144	-0.062744	-0.013524	0.024344
	0.007604					
x230	0.074691	-0.008991	0.034685	0.045200	0.003088	-0.021528
	0.009850	0.025290	0.019650	0.015784	0.010486	-0.021415
	-0.008269	0.030242	-0.036449	-0.051460	0.030919	0.007475
	-0.031677	-0.019074	0.016980	0.008836	0.006158	0.006723
	0.013468	-0.040743	-0.005294	-0.026360	0.000693	-0.023905
	0.017330					
y230	0.004854	-0.022522	-0.056362	0.008531	-0.038379	0.019650
	0.041627	-0.056591	-0.041171	-0.027336	0.048066	0.028372
	0.003078	-0.039909	0.037484	0.011477	0.029254	0.021607
	0.025036	0.017735	0.025593	-0.004255	-0.010746	-0.070163
	0.019824	-0.000885	-0.007906	-0.035168	-0.022322	0.011813
	0.008749					

z230	0.005982	-0.023386	0.018170	-0.030591	-0.008667	0.040922
	-0.033965	0.017780	-0.073019	-0.000053	0.007090	-0.011676
	-0.012407	-0.010546	-0.023264	-0.018128	0.026090	0.011062
	0.046050	-0.017244	0.034900	0.000995	0.027808	-0.023487
	-0.037293	-0.031871	0.029744	-0.060516	-0.015296	0.015341
	0.015620					
x231	0.073427	-0.009372	0.034373	0.045559	-0.003864	-0.021304
	0.015732	0.019693	0.024347	0.019428	0.010663	-0.017476
	-0.014546	0.030211	-0.033965	-0.044511	0.029762	-0.001806
	-0.032283	-0.032766	0.009991	0.003752	0.001208	0.018897
	0.012575	-0.044556	-0.007579	-0.026632	0.012355	-0.025510
	0.021947					
y231	0.006451	-0.028020	-0.055429	-0.002819	-0.032058	0.010429
	0.038022	-0.050562	-0.042420	-0.031659	0.046436	0.022703
	-0.000824	-0.046566	0.034010	0.014254	0.032127	0.033292
	0.029720	0.030223	0.025466	-0.003514	-0.010701	-0.075612
	0.007500	0.010018	-0.006010	-0.034618	-0.019214	0.019189
	-0.001887					
z231	0.006489	-0.033229	0.021080	-0.028547	-0.015700	0.031544
	-0.028091	0.012530	-0.072392	-0.002057	-0.006135	-0.014243
	-0.018214	-0.005802	-0.013031	-0.006162	0.026112	0.008421
	0.053906	-0.021630	0.027855	0.001278	0.021835	-0.014111
	-0.041134	-0.029656	0.029356	-0.075329	0.001195	0.007468
	0.002727					
x232	0.070299	-0.008741	0.032487	0.043574	-0.008488	-0.019788
	0.014788	0.013396	0.028958	0.024860	0.017916	-0.015153
	-0.019790	0.029799	-0.038335	-0.045683	0.028272	-0.001925
	-0.032268	-0.035547	0.004393	0.000179	0.000765	0.041531
	0.031929	-0.054510	0.005689	-0.003190	-0.002119	-0.014162
	0.035736					
y232	0.008015	-0.031869	-0.054879	-0.011773	-0.026191	0.002563
	0.035355	-0.043297	-0.040550	-0.037358	0.045338	0.020478
	-0.003808	-0.048894	0.030916	0.017111	0.036558	0.041846
	0.033364	0.035393	0.031183	-0.004818	-0.013668	-0.082739
	-0.005756	0.017034	-0.012267	-0.047893	-0.011735	0.006387
	-0.011223					
z232	0.004489	-0.039098	0.022089	-0.024999	-0.020056	0.024600
	-0.031069	0.002360	-0.070903	-0.002928	-0.009610	-0.015717
	-0.025676	0.001365	-0.004210	-0.005095	0.028989	0.010932
	0.061539	-0.008621	0.027466	-0.002314	0.022085	0.003435
	-0.020794	-0.030572	0.032400	-0.057089	-0.006293	0.001624
	0.007853					

x233	0.068325	-0.007172	0.028557	0.044277	-0.011985	-0.018446
	0.013128	0.014096	0.029767	0.030527	0.025103	-0.012321
	-0.019677	0.033297	-0.038789	-0.044111	0.027087	0.003470
	-0.027568	-0.045083	0.007343	-0.001570	0.000259	0.039960
	0.037141	-0.049571	-0.002020	-0.000357	-0.002254	-0.028777
	0.044885					
y233	0.009174	-0.034837	-0.053490	-0.021561	-0.019022	0.000289
	0.034482	-0.038429	-0.033721	-0.040867	0.048200	0.019538
	-0.007763	-0.049643	0.024741	0.019051	0.029942	0.044439
	0.030894	0.041571	0.026698	-0.006666	-0.022016	-0.077630
	-0.010491	0.017363	-0.008232	-0.045463	-0.010134	0.022615
	-0.016009					
z233	0.001182	-0.040556	0.017016	-0.015457	-0.022552	0.022865
	-0.033203	-0.001863	-0.066696	0.000300	-0.005080	-0.011699
	-0.026122	0.019451	0.004468	-0.004441	0.021348	0.016553
	0.059912	-0.004056	0.028878	0.002428	0.004794	0.000654
	-0.007646	-0.011244	0.023254	-0.043918	0.008945	-0.020662
	0.010192					
x234	0.067515	-0.006625	0.025808	0.043618	-0.013492	-0.019227
	0.012585	0.014122	0.024714	0.036727	0.025312	-0.017008
	-0.019196	0.034131	-0.045975	-0.038476	0.019517	0.001644
	-0.020528	-0.051609	0.003000	-0.004210	0.010825	0.032537
	0.030862	-0.029762	0.000713	-0.004799	0.005352	-0.016677
	0.040291					
y234	0.010008	-0.036964	-0.052003	-0.029479	-0.012484	-0.003216
	0.032433	-0.035267	-0.023406	-0.044829	0.053716	0.025308
	-0.014566	-0.051579	0.019482	0.019919	0.032580	0.047241
	0.019983	0.046993	0.026106	-0.004609	-0.036835	-0.071296
	-0.019265	0.010659	-0.005901	-0.039850	-0.002550	0.015827
	-0.013762					
z234	0.002322	-0.044139	0.017005	-0.008323	-0.021697	0.021162
	-0.034165	-0.008943	-0.064980	0.002610	-0.010006	-0.010742
	-0.031175	0.032062	-0.002867	0.006120	0.006573	0.009898
	0.051210	-0.010976	0.023926	0.009559	-0.006865	-0.003764
	-0.009473	0.009829	0.029260	-0.045790	0.033394	-0.025368
	0.004193					
x235	0.064905	-0.007278	0.026574	0.041233	-0.015189	-0.017710
	0.006467	0.012773	0.025394	0.038564	0.026065	-0.015756
	-0.013393	0.037184	-0.043386	-0.033796	0.017490	0.011056
	-0.009304	-0.043134	0.000480	-0.009841	0.006511	0.031185
	0.031078	-0.033228	0.011214	0.011367	0.011615	-0.007420
	0.023180					

y235	0.011627	-0.037219	-0.051730	-0.036808	-0.004681	-0.007349
	0.030831	-0.029842	-0.016581	-0.049054	0.056398	0.026604
	-0.024475	-0.049438	0.007180	0.021187	0.035865	0.041031
	0.015807	0.046137	0.024002	-0.003902	-0.044782	-0.065876
	-0.026386	0.015812	-0.005402	-0.043563	0.006441	0.007141
	-0.000949					
z235	0.001480	-0.047350	0.021776	-0.002982	-0.020753	0.022048
	-0.043228	-0.015241	-0.057370	0.000468	-0.013271	-0.004831
	-0.028946	0.052201	-0.001929	0.009842	0.003390	0.014292
	0.055832	-0.001622	0.023384	0.011504	-0.039079	0.000131
	0.002254	0.011618	0.036022	-0.017850	0.053360	-0.038778
	-0.005262					
x236	0.063411	-0.005276	0.023824	0.037092	-0.015494	-0.016844
	0.009218	0.009498	0.023393	0.044453	0.020490	-0.016047
	-0.015600	0.033829	-0.047226	-0.022613	0.020075	0.013844
	-0.008033	-0.029818	-0.006787	-0.013466	0.009291	0.040864
	0.030778	-0.030736	0.018603	0.003554	-0.006404	-0.009410
	0.018490					
y236	0.013111	-0.039895	-0.050211	-0.040624	-0.001945	-0.011697
	0.028477	-0.026564	-0.007163	-0.052758	0.061727	0.029232
	-0.026397	-0.051105	0.004254	0.015284	0.028970	0.039322
	0.013765	0.042616	0.029406	-0.011128	-0.043009	-0.060624
	-0.033594	0.020642	-0.010367	-0.036606	0.026238	0.004491
	0.013438					
z236	0.003550	-0.050015	0.020514	0.003576	-0.023547	0.020084
	-0.034158	-0.025224	-0.055785	0.006027	-0.021240	-0.002737
	-0.030512	0.049027	0.001772	0.015811	-0.001164	0.009715
	0.046482	0.014497	0.021476	0.006216	-0.037454	0.025987
	0.005923	0.014317	0.033246	-0.026026	0.043430	-0.048377
	0.012661					
x237	0.060272	-0.003944	0.022297	0.030770	-0.017530	-0.015847
	0.008613	0.009873	0.021071	0.047010	0.020377	-0.015337
	-0.021674	0.028765	-0.050111	-0.016630	0.027427	0.017458
	0.001224	-0.019021	-0.009031	-0.006196	0.020517	0.027774
	0.040729	-0.024779	0.023963	0.006624	-0.008524	-0.009838
	0.013255					
y237	0.016040	-0.042264	-0.049236	-0.043191	0.002423	-0.015049
	0.025212	-0.024799	0.003246	-0.052343	0.063461	0.029671
	-0.027463	-0.049353	-0.002240	0.017445	0.018317	0.036080
	0.006761	0.034245	0.030305	-0.020678	-0.052181	-0.040813
	-0.047427	0.017249	-0.020791	-0.032253	0.033004	0.011447
	0.017923					

z237	0.004457	-0.052560	0.019991	0.009518	-0.028589	0.023625
	-0.028444	-0.028830	-0.051380	0.013331	-0.014091	-0.002265
	-0.040450	0.040805	0.004042	0.014011	-0.007772	0.014463
	0.042445	0.013795	0.027513	0.019921	-0.019308	0.017832
	0.021969	0.013164	0.009705	-0.020554	0.045479	-0.049997
	0.020012					
x238	0.060188	-0.003998	0.022718	0.025841	-0.019792	-0.014676
	0.009289	0.005442	0.023713	0.051767	0.020932	-0.014845
	-0.018361	0.028106	-0.037123	-0.006881	0.027375	0.013944
	0.007831	-0.017286	-0.007570	-0.020044	0.012939	0.028388
	0.037942	-0.033008	0.022901	0.001909	-0.005579	-0.017558
	0.016906					
y238	0.014986	-0.041012	-0.048891	-0.045853	0.010425	-0.019378
	0.021203	-0.015843	0.006891	-0.056730	0.063109	0.029750
	-0.032268	-0.047755	-0.024019	0.012842	0.020236	0.034200
	0.000397	0.031787	0.023707	-0.017180	-0.041390	-0.033995
	-0.050769	0.033717	-0.010937	-0.014354	0.028513	0.019960
	0.005130					
z238	0.006017	-0.054578	0.027494	0.015325	-0.025130	0.024072
	-0.024725	-0.026245	-0.046619	0.016981	-0.009583	0.000085
	-0.034492	0.041348	0.020175	0.016365	-0.011864	-0.000547
	0.037189	0.002645	0.026802	0.012069	-0.027208	0.033183
	0.013777	0.005357	0.020173	-0.018678	0.032104	-0.058391
	0.017684					
x239	0.056227	-0.003538	0.022356	0.018637	-0.021838	-0.013897
	0.010210	0.006738	0.025866	0.057408	0.025282	-0.014115
	-0.021242	0.019161	-0.036434	0.003132	0.026742	0.016348
	0.009120	-0.011792	-0.009441	-0.024673	0.017226	0.026081
	0.020628	-0.032396	0.039216	0.003170	0.002067	-0.002790
	0.009181					
y239	0.016999	-0.039041	-0.045438	-0.049236	0.018864	-0.025156
	0.015991	-0.014002	0.007834	-0.064718	0.053411	0.036299
	-0.024825	-0.029923	-0.032359	-0.000417	0.020190	0.027699
	0.001607	0.039954	0.018513	-0.012677	-0.045141	-0.018475
	-0.027679	0.035654	-0.017951	-0.007679	0.017829	0.002812
	0.002790					
z239	0.005428	-0.058841	0.024943	0.021993	-0.026951	0.022730
	-0.018655	-0.014734	-0.035299	0.021135	-0.000358	-0.000633
	-0.039843	0.026901	0.024973	0.018873	-0.012863	-0.011424
	0.027160	0.013541	0.023186	0.018784	-0.033755	0.033692
	-0.022029	0.010194	0.026315	-0.010552	0.031261	-0.031536
	0.012831					

x240	0.053428	-0.001178	0.019867	0.013145	-0.024245	-0.010847
	0.009231	-0.001292	0.025790	0.060567	0.027277	-0.011820
	-0.024304	0.012942	-0.033994	0.013731	0.029072	0.011693
	0.011679	-0.002246	-0.012170	-0.027612	0.016749	0.027983
	0.031885	-0.038423	0.023744	0.009541	-0.004986	-0.014226
	0.022104					
y240	0.019281	-0.040181	-0.039293	-0.055870	0.026098	-0.031667
	0.013107	-0.002436	0.014376	-0.068650	0.044453	0.040954
	-0.018950	-0.017322	-0.047808	-0.016956	0.014860	0.034753
	0.003253	0.039892	0.016041	-0.009736	-0.043375	-0.004904
	-0.036760	0.042109	0.015211	-0.017906	0.023910	0.020613
	-0.017553					
z240	0.006192	-0.062064	0.019109	0.030264	-0.029841	0.026471
	-0.017198	-0.023516	-0.029748	0.026789	0.005861	0.002014
	-0.046798	0.021026	0.027082	0.024090	-0.008684	-0.017763
	0.022525	0.022537	0.019969	0.028181	-0.045679	0.032966
	-0.010147	-0.000526	-0.021554	0.002764	0.015589	-0.045650
	0.026588					
x241	0.050464	0.000274	0.017449	0.006841	-0.026118	-0.009887
	0.010094	-0.004547	0.024811	0.063482	0.027229	-0.011907
	-0.027141	0.006806	-0.030792	0.021806	0.033961	0.008937
	0.015525	0.004444	-0.013976	-0.029601	0.019249	0.027063
	0.035374	-0.033697	0.017527	0.015976	-0.009023	-0.011439
	0.028683					
y241	0.021789	-0.043526	-0.034365	-0.059471	0.028109	-0.037292
	0.006646	0.007898	0.017127	-0.075375	0.035918	0.039889
	-0.010801	-0.016038	-0.058887	-0.030866	0.009051	0.037222
	0.015286	0.044027	0.016884	-0.016344	-0.040140	0.009792
	-0.042696	0.029338	0.032245	-0.035864	0.026637	0.029727
	-0.028037					
z241	0.005249	-0.060649	0.014014	0.034561	-0.026418	0.030536
	-0.013029	-0.027255	-0.015353	0.037684	0.015036	0.005242
	-0.061366	0.027430	0.026560	0.029198	-0.011520	-0.015242
	-0.002540	0.016317	0.011011	0.054239	-0.059047	0.032077
	0.003371	0.011907	-0.033400	0.022349	0.003552	-0.041318
	0.026671					
x242	0.046085	0.001323	0.015610	0.001138	-0.026340	-0.008383
	0.009373	-0.006132	0.023683	0.063990	0.028711	-0.013717
	-0.029619	0.000259	-0.031742	0.030905	0.030067	0.009773
	0.016881	0.014458	-0.015087	-0.031985	0.019390	0.024033
	0.028454	-0.028889	0.019002	0.021653	-0.000671	-0.009405
	0.022868					

y242	0.027168	-0.040320	-0.035317	-0.063053	0.026669	-0.036873
	0.004093	0.000387	0.025751	-0.075242	0.026300	0.054163
	-0.009192	-0.008211	-0.060608	-0.043098	0.023752	0.027350
	0.018594	0.048612	0.011454	-0.022767	-0.039189	0.030784
	0.001226	0.003140	0.002474	-0.043282	-0.005400	0.001069
	0.002964					
z242	0.004165	-0.066189	0.017113	0.038022	-0.020978	0.029343
	-0.014528	-0.016973	-0.004653	0.035024	0.019986	-0.002815
	-0.067353	0.030396	0.013079	0.037507	-0.033525	-0.007152
	-0.017271	0.015171	0.006299	0.062816	-0.062737	0.018497
	-0.028972	0.024588	-0.009659	0.032479	0.033574	-0.027444
	-0.009543					
x243	0.041081	0.001791	0.014209	-0.004386	-0.024206	-0.009374
	0.007017	-0.002865	0.021820	0.062411	0.029917	-0.019498
	-0.031806	-0.007412	-0.032667	0.037579	0.026073	0.012378
	0.017910	0.019917	-0.012881	-0.030585	0.021235	0.020964
	0.017647	-0.020462	0.031636	0.017616	0.001179	0.006335
	0.013670					
y243	0.030801	-0.037382	-0.033370	-0.067255	0.025651	-0.036729
	0.001536	-0.000783	0.032886	-0.068718	0.018816	0.062516
	-0.009409	0.006067	-0.066640	-0.045719	0.022124	0.027528
	0.022841	0.047004	-0.010646	-0.033207	-0.047632	0.049075
	0.013837	-0.004808	-0.004503	-0.024149	-0.003316	-0.010826
	0.005595					
z243	0.001978	-0.071431	0.018325	0.041165	-0.020434	0.028525
	-0.013924	-0.012454	0.002105	0.025627	0.019744	-0.011602
	-0.066423	0.017849	0.015542	0.029629	-0.038220	-0.007096
	-0.019493	-0.000530	0.021225	0.078363	-0.034008	-0.013459
	-0.023010	0.007437	-0.007441	0.011711	0.033089	-0.030455
	-0.016508					
x244	0.037036	0.004345	0.012822	-0.011732	-0.022615	-0.008187
	0.005698	-0.004628	0.019324	0.061453	0.027586	-0.016996
	-0.033801	-0.009836	-0.034097	0.046446	0.028782	0.009727
	0.017499	0.027175	-0.011731	-0.032611	0.015380	0.022243
	0.016830	-0.020804	0.028122	0.016876	0.002500	-0.002422
	0.013275					
y244	0.031072	-0.041446	-0.027480	-0.071247	0.029606	-0.043415
	-0.006187	0.011896	0.038420	-0.069293	0.016373	0.043384
	-0.015464	0.008553	-0.069554	-0.048270	0.000551	0.036273
	0.041379	0.031967	-0.024294	-0.026529	-0.031457	0.043956
	-0.006322	0.009700	0.061272	-0.013150	0.005397	0.023509
	-0.026405					

z244	0.003944	-0.071298	0.014269	0.051570	-0.029207	0.029939
	-0.009554	-0.013649	0.012647	0.027727	0.014944	-0.000053
	-0.063528	0.014135	0.021537	0.012717	-0.030682	-0.006577
	-0.036597	0.008353	0.023620	0.078614	-0.038123	-0.009203
	0.000971	-0.017350	-0.058974	0.003615	0.043697	-0.031726
	0.022584					
x245	0.031263	0.006432	0.012327	-0.018856	-0.021271	-0.009666
	0.005354	0.000179	0.014570	0.061943	0.027218	-0.017582
	-0.032248	-0.014849	-0.034856	0.050555	0.023262	0.012528
	0.016066	0.029381	-0.007635	-0.029582	0.015161	0.020963
	0.010624	-0.011723	0.035905	0.010374	0.006233	0.008244
	0.002680					
y245	0.031130	-0.042527	-0.023478	-0.072165	0.026656	-0.051751
	-0.012180	0.023646	0.043645	-0.070799	0.011160	0.041187
	-0.028144	0.009687	-0.071592	-0.051538	-0.009335	0.038929
	0.037591	0.015712	-0.000135	-0.016550	-0.019319	0.042489
	0.007954	0.008496	0.070659	-0.026672	0.019582	0.037740
	-0.019928					
z245	0.008069	-0.076356	0.011248	0.058584	-0.030584	0.030942
	-0.008246	-0.015881	0.027235	0.029644	0.006076	-0.006508
	-0.061091	0.015587	0.022733	0.005212	-0.017187	-0.013378
	-0.031102	0.025768	-0.001372	0.065111	-0.045030	-0.008422
	-0.019323	-0.025117	-0.046668	0.026693	0.047839	-0.030661
	0.031388					
x246	0.025943	0.008933	0.011467	-0.025025	-0.019694	-0.009580
	0.006277	0.001723	0.009135	0.062770	0.024759	-0.015452
	-0.029501	-0.017816	-0.034255	0.053225	0.020984	0.011878
	0.014865	0.037761	-0.006726	-0.026696	0.011153	0.021058
	0.010692	-0.006133	0.033497	0.008496	0.007348	0.007387
	0.001210					
y246	0.034556	-0.038565	-0.018913	-0.073771	0.015396	-0.051948
	-0.015257	0.017530	0.049982	-0.064138	-0.000617	0.047745
	-0.029178	0.014017	-0.061321	-0.055095	0.000316	0.036752
	0.040971	0.008877	-0.007750	-0.004783	-0.019755	0.046359
	0.038693	-0.017288	0.058358	-0.024006	0.027521	-0.000166
	0.011956					
z246	0.011372	-0.082985	0.009814	0.059902	-0.030554	0.024530
	-0.008053	-0.008385	0.039764	0.022409	-0.000035	-0.016659
	-0.059236	0.012739	0.017278	-0.001342	-0.026027	-0.015843
	-0.024509	0.035610	-0.001060	0.051295	-0.023494	-0.021392
	-0.030608	-0.021225	-0.034947	0.024060	0.040528	0.006401
	0.019810					

x247	0.019690	0.010481	0.012586	-0.031266	-0.016185	-0.012163
	0.004930	0.006674	0.000524	0.059969	0.020589	-0.018823
	-0.029659	-0.016382	-0.033816	0.064956	0.014495	0.006960
	0.015500	0.050388	0.000203	-0.023675	0.010671	0.015378
	-0.003125	0.003506	0.042268	0.013046	0.011506	0.017937
	-0.004468					
y247	0.034263	-0.040258	-0.014726	-0.073081	0.009154	-0.061156
	-0.016530	0.021520	0.048949	-0.061024	-0.014159	0.043217
	-0.030739	0.030839	-0.044483	-0.039198	-0.008626	0.030195
	0.048079	0.006666	-0.020368	-0.001145	0.009389	0.019666
	0.050737	-0.011211	0.066078	-0.005472	0.033572	0.021297
	0.021337					
z247	0.015824	-0.084192	0.006839	0.061736	-0.035785	0.022082
	-0.001738	-0.003694	0.056135	0.022277	-0.007887	-0.010216
	-0.044073	0.002534	0.021207	-0.030695	-0.013042	-0.007446
	-0.027842	0.025232	-0.011701	0.046474	-0.020799	-0.021516
	-0.013353	-0.021292	-0.039176	-0.010885	0.023178	-0.012201
	0.025918					
x248	0.012211	0.012092	0.014093	-0.038030	-0.016152	-0.010958
	0.006655	0.010951	-0.005270	0.057274	0.020551	-0.019049
	-0.028236	-0.016600	-0.037171	0.057906	0.003734	0.003781
	0.014586	0.060070	0.008035	-0.018959	0.013961	0.011386
	0.001172	0.008213	0.047623	0.007809	-0.000232	0.020178
	-0.015850					
y248	0.030996	-0.037409	-0.020852	-0.071523	0.001155	-0.059023
	-0.019866	0.023773	0.060199	-0.058269	-0.014665	0.043121
	-0.036836	0.047559	-0.037230	-0.035367	-0.009338	0.030937
	0.037162	0.006494	-0.031539	0.002246	0.018136	0.000609
	0.060206	0.007626	0.057840	-0.009687	0.030131	-0.000825
	0.016484					
z248	0.019658	-0.082639	-0.001353	0.061888	-0.028933	0.017610
	0.000033	-0.001584	0.062429	0.022917	-0.025710	-0.009183
	-0.030831	0.003941	0.028019	-0.013999	0.004668	-0.002891
	-0.044229	0.018288	-0.030562	0.037826	-0.036102	-0.024234
	-0.026338	-0.010554	-0.046514	-0.016700	0.036707	-0.030841
	0.034225					
x249	0.006734	0.016154	0.015874	-0.044728	-0.015436	-0.006754
	0.006445	0.013872	-0.010510	0.057753	0.016637	-0.013006
	-0.021878	-0.019870	-0.041267	0.058192	-0.002036	0.001810
	0.007900	0.060389	0.001438	-0.023239	0.011507	0.007086
	0.012092	-0.000047	0.027204	-0.009186	-0.009622	0.020260
	0.000420					

y249	0.031723	-0.032066	-0.023454	-0.069193	-0.010448	-0.047079
	-0.024268	0.023061	0.071781	-0.049250	-0.011074	0.045713
	-0.042721	0.050787	-0.036515	-0.031675	-0.009872	0.025201
	0.018227	-0.004570	-0.042789	0.003252	0.017581	-0.003261
	0.056268	0.002065	0.029049	-0.018056	0.019912	-0.010076
	0.022704					
z249	0.021943	-0.083967	-0.007690	0.063389	-0.024542	0.006417
	0.006938	0.000305	0.068401	0.019796	-0.036083	-0.017002
	-0.026175	0.009343	0.030864	-0.018769	0.005291	-0.004019
	-0.056614	0.045642	-0.021973	0.052895	-0.044235	0.001646
	-0.038629	0.007879	-0.014653	0.012292	0.038959	-0.024378
	0.011630					
x250	-0.001957	0.018691	0.023102	-0.052373	-0.007816	-0.010380
	0.005421	0.019256	-0.019042	0.057966	0.011624	-0.012086
	-0.021315	-0.007371	-0.036832	0.052873	-0.012083	-0.002213
	-0.009420	0.071141	0.001648	-0.005225	0.011164	0.001670
	0.017548	0.004547	0.047000	0.005397	-0.003067	0.021363
	-0.001226					
y250	0.030285	-0.029641	-0.021891	-0.069088	-0.014848	-0.039483
	-0.030460	0.032986	0.076500	-0.039938	-0.001314	0.037318
	-0.056343	0.058086	-0.029952	-0.026595	-0.021151	0.023904
	-0.006846	-0.019548	-0.045441	0.016964	0.021870	-0.010895
	0.029966	0.002192	0.038290	-0.017150	0.014422	0.015678
	0.013838					
z250	0.028648	-0.084009	-0.014553	0.062718	-0.024205	0.009778
	0.018045	0.003008	0.066301	0.016141	-0.044890	-0.019614
	-0.009188	-0.010439	0.022562	-0.016007	0.008965	0.000916
	-0.051266	0.030047	-0.017730	0.038823	-0.038946	0.012562
	-0.048156	-0.006718	-0.032301	-0.011766	0.008576	-0.015220
	0.003613					
x251	-0.009287	0.023276	0.023294	-0.060404	-0.003232	-0.010041
	0.006420	0.024911	-0.025382	0.060364	-0.001719	-0.015200
	-0.017740	-0.001945	-0.031547	0.047907	-0.023171	-0.010150
	-0.025910	0.080992	0.008228	0.006909	0.020186	0.005502
	0.008551	0.011923	0.064492	0.010171	-0.002826	0.044845
	-0.004894					
y251	0.029557	-0.025721	-0.022348	-0.067781	-0.022639	-0.032967
	-0.034198	0.042014	0.077475	-0.031904	-0.003813	0.032147
	-0.065692	0.055072	-0.026958	-0.022193	-0.028555	0.011610
	-0.021161	-0.031883	-0.043877	0.021025	0.041181	-0.017826
	0.001265	0.015759	0.055310	-0.024301	0.002480	0.037493
	0.004045					

z251	0.035123	-0.087710	-0.011011	0.061493	-0.019200	0.010183
	0.027165	0.008911	0.062769	0.008085	-0.042261	-0.013263
	-0.003602	-0.024535	0.008979	-0.010986	0.005122	0.009619
	-0.056063	0.000684	-0.022550	0.035360	-0.041246	0.009045
	-0.045860	-0.025151	-0.044642	-0.023996	-0.013107	-0.042713
	-0.001882					
x252	-0.015654	0.027288	0.022697	-0.065463	-0.005500	-0.007468
	0.007109	0.015194	-0.036960	0.063359	-0.013601	-0.013874
	-0.017932	0.001467	-0.031159	0.049515	-0.036420	-0.008010
	-0.039286	0.088897	0.007077	0.014119	0.011098	0.015132
	0.024601	-0.017388	0.035467	-0.005281	-0.024307	0.017781
	0.012216					
y252	0.028346	-0.021639	-0.024243	-0.065168	-0.039015	-0.021769
	-0.039630	0.039178	0.080434	-0.030474	-0.008284	0.038782
	-0.074435	0.053778	-0.027131	-0.018763	-0.031557	-0.007777
	-0.023315	-0.036302	-0.048089	0.017507	0.048691	-0.007405
	0.018113	-0.014210	0.039733	-0.042513	-0.021824	-0.002255
	0.025135					
z252	0.039158	-0.088995	-0.008939	0.058918	-0.008261	0.007824
	0.036936	0.023802	0.065347	-0.002663	-0.037947	-0.005757
	0.009897	-0.031489	0.001200	-0.012725	0.008114	-0.000223
	-0.065528	-0.012372	-0.026561	0.025648	-0.031138	0.008135
	-0.042955	-0.004291	-0.021412	0.005143	-0.017851	-0.041123
	-0.013845					
x253	-0.020526	0.029563	0.024694	-0.070485	0.003450	-0.007104
	0.005696	0.019652	-0.043618	0.060702	-0.010370	-0.013044
	-0.013423	0.001774	-0.029334	0.041603	-0.041776	0.003379
	-0.041115	0.077890	-0.017617	0.020711	0.023296	0.006430
	0.021331	0.008060	0.030434	0.005373	-0.056266	0.017207
	-0.017854					
y253	0.027327	-0.016827	-0.022381	-0.063995	-0.046362	-0.012699
	-0.048687	0.043865	0.079932	-0.026024	-0.002205	0.040888
	-0.079424	0.045486	-0.027864	-0.022818	-0.019451	-0.019619
	-0.022403	-0.051180	-0.068605	0.024440	0.059310	-0.006765
	0.005684	-0.006501	0.043285	-0.041732	-0.047601	0.000199
	0.003236					
z253	0.040922	-0.089919	-0.006450	0.055752	-0.004298	0.003150
	0.049381	0.029405	0.056820	-0.007664	-0.050468	-0.005870
	0.020553	-0.041925	-0.005562	-0.004138	0.005055	-0.009288
	-0.083238	-0.013374	-0.016104	0.029688	-0.036800	0.022198
	-0.043825	-0.029721	0.011754	0.013156	-0.000679	-0.027915
	0.000512					

x254	-0.023726	0.029752	0.027040	-0.073923	0.015918	-0.005272
	0.005133	0.023076	-0.049781	0.066005	-0.000606	-0.008753
	-0.018602	0.000766	-0.019402	0.021978	-0.036336	-0.011682
	-0.065957	0.090949	-0.033698	0.033397	0.026380	0.018055
	0.009591	0.003461	-0.004951	0.038564	-0.066062	0.039728
	0.000318					
y254	0.027452	-0.014084	-0.019523	-0.057773	-0.051840	0.001244
	-0.057713	0.042914	0.072492	-0.017623	0.006799	0.055001
	-0.086384	0.041614	-0.028816	-0.031025	0.004734	-0.041211
	-0.025182	-0.054925	-0.081865	0.026254	0.067993	-0.005050
	-0.000159	-0.021702	0.001959	-0.033972	-0.057275	0.007468
	-0.025967					
z254	0.041297	-0.089149	-0.003417	0.051115	0.002412	-0.002649
	0.066174	0.034230	0.039418	-0.017788	-0.069244	-0.007491
	0.036278	-0.053487	-0.014829	0.015924	-0.006518	0.009182
	-0.089991	-0.029579	-0.012414	0.018680	-0.025186	0.021261
	-0.033118	-0.033071	0.050715	0.015338	-0.009118	-0.040822
	-0.017647					
x255	-0.030335	0.029460	0.025464	-0.071159	0.021449	-0.004654
	0.007511	0.030599	-0.051225	0.058725	-0.015877	-0.019429
	-0.020346	-0.006374	-0.015637	0.019414	-0.064970	-0.019693
	-0.072854	0.078132	-0.031609	0.060794	0.034196	0.027752
	-0.017516	0.006194	0.001868	0.033060	-0.085226	0.078537
	-0.053772					
y255	0.023167	-0.014082	-0.016483	-0.047765	-0.060271	0.004966
	-0.062996	0.056186	0.070070	-0.020336	0.001847	0.059672
	-0.087975	0.034289	-0.039158	-0.033821	-0.002071	-0.050591
	-0.011193	-0.075997	-0.085470	0.042934	0.089232	0.001134
	-0.022426	-0.011797	0.008317	-0.059333	-0.061536	0.024516
	-0.118176					
z255	0.043948	-0.088719	0.002829	0.046238	0.014945	-0.011797
	0.079768	0.037081	0.018212	-0.019677	-0.075576	0.000255
	0.047863	-0.058739	-0.023000	0.023220	0.002042	0.027810
	-0.108859	-0.028157	-0.021648	0.007455	-0.011375	0.023739
	-0.010044	-0.025318	0.071660	0.038727	-0.000909	-0.068317
	0.000562					
x256	-0.034818	0.029311	0.023166	-0.064589	0.025839	-0.004812
	0.019054	0.025684	-0.036205	0.045664	-0.017631	-0.036240
	-0.022680	-0.002849	-0.018260	0.030997	-0.094266	-0.018691
	-0.088783	0.060040	-0.024279	0.071912	0.046402	0.030405
	-0.015010	0.012362	-0.005262	0.025279	-0.072482	0.108329
	-0.083455					

y256	0.019969	-0.011038	-0.009937	-0.040839	-0.070114	0.012465
	-0.067657	0.060735	0.070537	-0.027972	-0.000240	0.074011
	-0.086407	0.032381	-0.060776	-0.039728	-0.004413	-0.066631
	-0.001453	-0.098616	-0.081207	0.057496	0.114994	-0.004071
	-0.009824	-0.015845	0.014887	-0.083592	-0.062330	0.023876
	-0.174772					
z256	0.046290	-0.089015	0.013923	0.035498	0.033740	-0.021043
	0.085688	0.047330	-0.024962	-0.015207	-0.086848	0.007366
	0.053628	-0.081252	-0.021192	0.022023	0.026017	0.038620
	-0.116347	-0.013305	-0.042203	-0.001589	-0.001770	0.034642
	-0.014904	-0.017910	0.104311	0.078551	-0.038156	-0.079101
	0.030404					

Phylogenetic mapping

Dataset: PC scores, CovMatrix, BE_Canals, Procrustes coordinates

Data type: PC scores

Phylogeny: Phylogeny_BE_Canals

Method: weighted squared-change parsimony

The tree is rooted.

Tree length: 0.11770675

Permutation test against the null hypothesis of no phylogenetic signal

Number of randomization rounds: 10000

P-value: 0.1624

Canonical Variate Analysis: CVA ...BE_Canals

Dataset: BE_Canals

Classification criterion: environment

Groups	Observations
1. coastal	15
2. continental	8
3. marine	7
4. polar	2

Variation among groups, scaled by the inverse of the within-group variation

	Eigenvalues	% Variance	Cumulative %
1.	3.40290548	56.916	56.916
2.	1.52561791	25.517	82.434
3.	1.05025811	17.566	100.000

Mahalanobis distances among groups:

	coastal	continental	marine
continental	3.8492		
marine	2.6440	4.2482	
polar	5.1355	6.6419	4.9089

P-values from permutation tests (10000 permutation rounds) for Mahalanobis distances among groups:

	coastal	continental	marine
continental	<.0001		
marine	0.0285	0.0004	
polar	0.0032	<.0001	0.0555

Procrustes distances among groups:

	coastal	continental	marine
continental	0.0550		
marine	0.0532	0.0750	
polar	0.0988	0.1204	0.0991

P-values from permutation tests (10000 permutation rounds) for Procrustes distances among groups:

	coastal	continental	marine
continental	0.0637		
marine	0.1618	0.0002	
polar	0.1364	0.0413	0.1772

Canonical coefficients:

	CV1	CV2	CV3
x1	-3.8117	2.6740	2.5468
y1	4.1426	5.2789	-7.8954
z1	1.4158	-4.2567	-0.7465
x2	-12.3712	-2.7364	-3.5842
y2	-3.5398	2.2586	-8.6627
z2	-6.4206	-2.8162	-6.1507
x3	-10.1597	-1.7272	-4.3538
y3	-0.7704	2.6024	-6.7038
z3	-4.7405	-1.4020	-5.5701
x4	-6.8298	-0.9708	-4.3324

y4	0.7449	3.8629	-5.0912
z4	-1.6094	-0.1929	-5.1166
x5	-6.2714	0.2002	-3.1650
y5	2.1370	5.3569	-4.4192
z5	-1.4678	0.7870	-3.4122
x6	-4.4582	0.6262	-2.2792
y6	3.0404	5.6036	-4.2572
z6	-0.2907	1.9547	-2.3844
x7	-4.2475	1.4463	-1.6364
y7	3.8197	5.5458	-4.5275
z7	-1.6298	4.2135	-1.1371
x8	-3.9751	1.0452	-1.8662
y8	1.6711	5.5135	-4.5446
z8	-2.1777	3.2557	-1.9960
x9	-3.7638	-0.0177	-2.3134
y9	0.0188	4.8399	-4.6411
z9	-1.4506	0.8305	-2.2761
x10	-3.6249	0.1583	-1.5026
y10	-1.3488	4.8659	-4.2435
z10	-3.6349	3.1500	2.1082
x11	-3.0779	-0.2580	-1.9500
y11	-1.1796	3.8596	-2.2808
z11	-3.5199	2.2428	1.1856
x12	-1.1451	-1.4495	-3.0595
y12	-1.7425	2.6580	0.6990
z12	-2.5353	1.0804	-0.7798
x13	-0.2847	-0.5040	-3.5507
y13	-3.0406	2.0003	1.4817

z13	-3.4746	3.3633	-2.4900
x14	0.1413	-0.2544	-3.4995
y14	-2.5516	1.3204	2.3698
z14	-4.4635	2.1071	-4.6989
x15	0.7746	0.3515	-3.6226
y15	-1.4650	1.1812	3.3201
z15	-3.2516	2.6584	-3.3078
x16	1.6582	0.7994	-3.2222
y16	-1.6605	0.4108	2.8779
z16	-4.8713	3.2810	-1.7758
x17	1.6652	1.6885	-2.0954
y17	-0.6817	-0.8816	1.6313
z17	-6.3939	2.7116	-1.3527
x18	1.5291	2.1221	-2.3590
y18	0.4176	-2.3000	1.5480
z18	-6.7340	3.5363	-0.0747
x19	3.5641	-1.1640	-3.3983
y19	-0.0404	-2.8077	0.2379
z19	-8.7752	7.6859	3.6301
x20	4.7181	-0.3735	-0.9436
y20	-2.2642	-0.9731	-0.8698
z20	-7.4831	4.5284	2.8386
x21	6.2094	-1.1591	2.0548
y21	-5.6979	0.1040	-4.4276
z21	-5.6862	4.5948	2.7576
x22	6.6720	-2.4945	3.7162
y22	-7.9977	3.5150	-3.5595
z22	-2.5572	2.5520	0.5608

x23	4.9995	-2.6686	5.7885
y23	-11.4356	5.3865	-4.4691
z23	1.8159	0.3095	0.1142
x24	4.7813	-5.1737	4.6626
y24	-11.7926	6.1196	-5.1797
z24	2.8837	0.4719	1.7830
x25	4.9554	-8.1177	-0.5746
y25	-9.3197	4.8701	-6.1061
z25	2.1742	-0.2395	2.7312
x26	1.4491	-6.7766	-1.7913
y26	-10.0094	6.8995	-2.6466
z26	1.5528	-1.3133	2.3957
x27	4.1245	-6.4958	-1.2168
y27	-3.2501	3.8632	1.4700
z27	-0.7610	-2.0184	1.4314
x28	4.9617	-4.2780	-2.3578
y28	1.5452	0.3265	0.5664
z28	-2.5711	-3.3204	0.0980
x29	2.2720	-5.2883	-3.7532
y29	-1.9675	-8.6973	-6.8721
z29	-3.9562	-4.8951	-1.2825
x30	1.5448	-8.4082	-5.0044
y30	-7.6594	-6.2431	-8.7793
z30	-1.4440	-10.2261	-2.7053
x31	2.1177	-6.4819	-2.5010
y31	-8.3120	-1.5529	-4.9313
z31	0.1131	-7.7909	-0.8574
x32	2.3070	-5.8173	-0.5648

y32	-9.0167	1.3115	-3.6226
z32	-2.7496	-4.7092	-3.9469
x33	2.9747	-7.3656	-0.7894
y33	-6.7056	6.0512	-0.9178
z33	-1.3779	-1.8867	-4.8807
x34	3.5788	-6.8162	-0.0023
y34	-4.5449	9.7831	-0.0066
z34	3.9476	-0.3338	-5.3523
x35	1.0292	-3.4151	-0.1884
y35	-0.9628	9.5418	0.1513
z35	11.9408	-1.1019	-2.1504
x36	0.0412	-2.8384	-1.2735
y36	1.3181	8.3324	1.0047
z36	13.6073	2.5099	2.3997
x37	1.3083	-4.0462	-0.6682
y37	3.0015	4.4620	-0.0780
z37	9.3204	8.4448	1.9146
x38	3.5096	-3.2128	0.5158
y38	3.2518	-1.7554	-4.9921
z38	3.8091	11.6979	3.8040
x39	4.9463	-0.4395	1.5807
y39	1.5365	0.4740	-3.7356
z39	2.3697	9.7379	1.4141
x40	4.6169	-1.8797	2.0420
y40	2.2727	3.0636	-0.0528
z40	0.2907	7.4389	-1.5920
x41	4.6843	-2.8945	1.2573
y41	1.7867	6.0245	3.3749

z41	-1.0776	4.6114	-4.3628
x42	2.2267	-2.5623	1.2627
y42	2.3957	6.9204	4.1477
z42	-2.9647	2.9468	-5.6279
x43	-1.1899	-2.3190	0.9188
y43	2.9747	6.7794	3.4899
z43	-4.3690	2.2259	-5.9486
x44	-5.5796	-2.1605	0.0001
y44	2.6194	7.2805	2.7501
z44	-4.5340	1.5448	-5.5013
x45	-9.8261	-1.1682	-0.7398
y45	1.8209	6.7504	1.2539
z45	-4.8726	1.1488	-4.7335
x46	-12.8477	-0.5453	-1.3700
y46	0.6628	5.0274	-2.2912
z46	-5.1711	0.7124	-3.9224
x47	-7.4304	-1.5374	-0.3542
y47	1.6972	-1.7596	3.2831
z47	5.6959	2.2261	-6.0001
x48	-6.5906	-2.6250	1.0677
y48	0.6277	-0.4643	3.3100
z48	5.1606	1.1612	-5.3505
x49	-7.2927	-2.7144	1.0045
y49	-2.4258	1.7713	2.7246
z49	3.9856	1.2767	-4.3715
x50	-6.0542	-4.8585	-0.1034
y50	-1.3512	-1.3212	-0.3086
z50	4.7065	-1.2891	-4.7025

x51	-5.5851	-4.4599	-0.1385
y51	-1.1210	-1.6283	-1.1421
z51	4.9684	-2.0181	-3.7528
x52	-4.1834	-3.9516	0.6418
y52	0.4382	-0.6127	-0.1267
z52	6.3712	-1.9133	-1.6601
x53	-3.0889	-3.7923	0.3863
y53	2.5407	-0.3673	-0.3875
z53	7.8545	-2.0169	-0.9553
x54	-2.5078	-2.6553	1.0552
y54	2.9310	1.3259	0.6376
z54	8.1042	-0.1303	1.6862
x55	-0.2903	-1.9724	0.5873
y55	3.8002	-1.1648	0.0873
z55	10.2123	-3.0788	1.7376
x56	-0.7241	-2.0615	-0.5624
y56	1.8497	3.9105	1.0484
z56	8.8319	-3.2329	0.7112
x57	-2.6857	-1.4242	-1.0860
y57	-0.9029	6.4092	1.4624
z57	4.6699	-3.4647	-0.1158
x58	-2.9627	-1.2435	-1.3075
y58	-2.2908	3.7466	0.6735
z58	2.6499	-3.2243	0.3852
x59	-2.8623	-0.3253	-1.4535
y59	-0.2744	4.5020	2.4580
z59	4.2340	-1.7552	2.3034
x60	-1.8296	-1.3451	-2.0056

y60	2.4566	1.6748	2.0017
z60	6.9309	-3.5537	2.9739
x61	-1.6885	-1.1434	-1.9388
y61	-1.3888	-0.5963	-0.5060
z61	3.9194	-3.7736	3.1839
x62	-1.6819	-1.0338	-2.5738
y62	0.2153	-1.7079	0.0908
z62	4.2105	-2.8048	3.5682
x63	-0.7186	-0.7114	-2.3868
y63	-0.9936	-2.2848	0.7475
z63	3.6969	-2.2204	5.0032
x64	-0.6072	0.1734	-2.3406
y64	-1.5866	-4.9695	-0.9951
z64	1.8021	-4.4139	3.4547
x65	-0.8769	0.6865	-2.5793
y65	0.8206	-4.6504	-0.5520
z65	1.4812	-3.5437	3.3592
x66	-0.3223	0.8633	-3.1485
y66	1.5558	-5.7041	0.8741
z66	2.3817	-6.1137	2.4760
x67	0.5353	0.6735	-3.3590
y67	0.4597	-3.2266	2.1225
z67	2.2206	-3.2396	2.9978
x68	1.2810	0.3968	-3.6490
y68	-0.3660	-1.4256	3.6377
z68	1.9214	-1.2670	3.6409
x69	3.7391	0.6662	-2.4310
y69	-4.4922	-1.1696	2.1342

z69	-1.7432	-0.5379	1.4150
x70	3.6399	0.3939	-2.1785
y70	-4.9651	-0.1059	2.0041
z70	-1.8695	1.1017	1.3114
x71	3.5108	1.7563	-1.9702
y71	-5.1640	-0.6772	2.6789
z71	-1.8840	0.4048	1.1526
x72	3.1854	2.2396	-2.4045
y72	-5.0629	-2.3451	2.9817
z72	-2.0485	1.2813	2.3586
x73	1.6535	2.4133	-1.7006
y73	-3.9036	-2.1966	2.7202
z73	-1.8713	-1.2941	-1.2323
x74	0.1930	0.3578	-4.2994
y74	-4.0878	2.1135	5.8740
z74	-0.5055	0.4925	1.0393
x75	-0.7633	0.5108	-3.8462
y75	-3.6185	3.5766	5.7187
z75	-0.6586	0.3723	1.8025
x76	-1.3061	0.5517	-2.3062
y76	-1.2084	5.0881	4.4709
z76	-0.7629	-1.2896	0.8297
x77	-3.1288	0.4609	-2.7095
y77	2.0638	6.3567	4.2629
z77	0.0502	-2.7122	1.3066
x78	-0.9219	2.8689	-1.4836
y78	3.0899	5.7415	3.5213
z78	-0.7240	-2.7004	1.5898

x79	-1.0378	2.9402	-1.2892
y79	3.8686	5.3928	2.9474
z79	-0.9282	-2.3183	2.2041
x80	0.1859	-1.0533	-3.5738
y80	3.8784	6.8315	3.4633
z80	-1.2938	0.6652	4.4543
x81	-2.3382	1.2410	-1.1748
y81	7.2055	2.0294	1.6391
z81	-0.5083	2.1499	5.7067
x82	-5.7802	4.1915	3.2734
y82	9.9366	-3.3628	-1.3427
z82	-0.5849	5.2847	6.9432
x83	-6.9635	4.4913	4.4243
y83	9.9288	-6.8598	-0.8229
z83	-1.4187	11.3648	9.5830
x84	-1.6437	-11.2633	-0.6431
y84	2.7010	-3.7929	2.0067
z84	-6.5823	-5.6143	-6.6949
x85	1.3657	-10.7079	-2.2286
y85	1.8054	-2.7949	3.7422
z85	-5.2094	-4.4860	-6.9106
x86	1.8613	-8.9567	-3.3582
y86	2.1211	-3.8974	3.8523
z86	-3.0239	-2.8170	-6.9241
x87	1.8394	-9.3457	-5.3039
y87	1.5560	-3.8250	4.1176
z87	-2.3346	-2.0351	-7.6289
x88	2.2166	-8.7752	-5.6579

y88	0.2100	-4.7083	3.0249
z88	-1.0832	-0.9674	-7.9579
x89	0.2441	-8.2791	-7.0606
y89	1.2409	-4.9548	3.5734
z89	-0.7848	0.1341	-8.6949
x90	-1.2425	-7.3520	-7.5971
y90	1.6445	-4.9951	3.2253
z90	-1.1775	1.6566	-9.1323
x91	-3.5423	-5.7678	-7.4147
y91	2.4626	-5.9961	1.7899
z91	-0.9664	4.0254	-7.5171
x92	-4.1920	-5.5089	-7.2336
y92	0.1058	-6.9612	-0.8610
z92	1.5292	5.0142	-5.1170
x93	-1.5152	-7.1423	-6.2429
y93	-0.9239	-4.2026	0.2329
z93	4.2913	2.4387	-5.0405
x94	1.6321	-7.5725	-4.6311
y94	-0.7210	-1.7304	3.6829
z94	6.0533	1.5036	-5.4179
x95	1.2619	-7.3185	-2.9379
y95	1.0882	-2.5794	2.5703
z95	5.7437	3.0708	-3.1981
x96	0.6135	-6.1432	-1.9355
y96	2.2834	-2.0230	2.5638
z96	5.4473	3.2726	-2.7136
x97	2.6609	-5.3237	-1.8110
y97	2.5580	-1.9619	2.2836

z97	6.9999	5.0375	-1.2326
x98	3.1997	-3.9179	-1.2478
y98	3.7997	-2.1132	1.7867
z98	5.7351	7.7172	0.2583
x99	1.0949	-0.8296	0.7917
y99	5.0180	-1.8654	2.1666
z99	2.4444	10.1799	1.2760
x100	1.5806	-0.9148	0.2014
y100	5.6172	-1.4420	2.1170
z100	3.4820	8.5306	-0.5181
x101	1.2848	-0.4334	0.2840
y101	6.0244	-0.8019	1.7956
z101	3.3955	7.8595	-1.6804
x102	1.2495	-0.8640	-0.5182
y102	6.9642	-0.8878	1.3635
z102	4.0343	7.5569	-0.6481
x103	0.7715	-1.2768	0.2499
y103	6.3661	-0.1622	1.7768
z103	5.9825	3.3240	-0.9467
x104	0.2567	-0.0011	1.0527
y104	5.5258	-0.2370	0.5860
z104	3.5855	2.3363	-2.0010
x105	-0.3373	1.9036	3.1578
y105	5.7400	0.2393	0.3765
z105	3.3936	2.2560	-0.2762
x106	-1.3282	2.4093	3.5550
y106	5.3287	0.0336	1.0063
z106	2.1437	2.4000	1.4050

x107	-1.7775	2.6311	3.5312
y107	5.6095	0.3322	2.3793
z107	2.7619	2.2824	4.0292
x108	-2.0219	0.6568	3.7192
y108	3.3329	-0.9815	2.6231
z108	3.0612	-2.6953	4.4076
x109	-2.4856	-0.9487	1.7780
y109	1.9559	-0.9239	1.7502
z109	3.3081	-4.4455	4.0873
x110	-3.9565	1.8334	0.9009
y110	-0.4757	5.5686	2.4689
z110	2.5858	-3.7039	4.1930
x111	-2.6892	0.9069	0.9146
y111	-1.5379	9.8426	2.7387
z111	0.6584	-2.4828	4.1881
x112	-2.0872	-0.6924	-1.7561
y112	4.7898	11.5370	5.5764
z112	-2.5515	-1.4180	3.4447
x113	-6.9046	-1.8514	-7.2033
y113	0.5933	11.0138	5.4754
z113	-5.5445	-1.6167	1.7478
x114	-5.0065	-5.0249	-15.4927
y114	1.1652	7.8740	0.6915
z114	-7.0034	-2.7999	-0.0223
x115	-4.3383	-2.9862	-15.1134
y115	-0.3484	7.4448	2.6968
z115	-8.3807	-1.6462	-1.4177
x116	-4.5006	1.8188	-10.2402

y116	-0.1807	4.9473	2.2309
z116	-7.3478	-2.3197	-4.2727
x117	3.2323	-0.0236	-12.3846
y117	1.1115	1.8441	-1.0996
z117	-9.1115	1.3754	-1.0994
x118	6.0760	3.7506	-6.6762
y118	-0.2489	1.2047	-1.6886
z118	-7.5904	5.2485	1.9465
x119	8.1796	7.7844	2.6255
y119	-2.3617	1.4710	-1.9696
z119	-1.8597	9.7901	6.3826
x120	11.1017	13.2946	12.2611
y120	-4.9193	-0.8079	-2.9277
z120	2.8710	10.3451	11.8494
x121	12.1971	7.8708	7.6274
y121	-5.0741	1.3721	0.1271
z121	3.2390	5.7506	8.5400
x122	10.7227	6.0403	5.7487
y122	-7.0375	0.4838	-2.5973
z122	0.8447	2.7228	4.3191
x123	9.3471	3.0223	2.9292
y123	-8.9505	-2.1923	-3.8612
z123	-0.9803	-2.7072	-0.2795
x124	6.5795	0.0398	1.2027
y124	-9.1626	-6.2364	-5.4084
z124	-2.0570	-7.9193	-4.3009
x125	3.9303	-1.6202	0.4470
y125	-8.4388	-10.3326	-5.5308

z125	-2.5825	-12.6942	-7.3525
x126	2.7516	-2.5787	-0.0785
y126	-7.8444	-13.3033	-4.6994
z126	-3.5826	-16.6403	-9.8455
x127	1.0998	-4.7153	-0.4383
y127	-5.5668	-11.5406	-1.9213
z127	-2.9773	-15.3479	-10.6271
x128	-0.5004	-9.2925	-1.7910
y128	-0.9817	-4.9099	2.2102
z128	-3.9202	-8.5744	-8.0629
x129	8.1941	4.1578	6.5732
y129	-2.8126	7.9762	-0.9047
z129	12.0702	9.0170	2.1183
x130	7.4839	-0.5140	3.8190
y130	-0.1207	8.7461	-1.7405
z130	9.0681	8.0142	2.0767
x131	6.9839	-1.9665	3.7302
y131	2.8697	11.7032	0.8014
z131	4.8304	3.4869	-0.1114
x132	6.3259	-3.4225	2.7570
y132	6.9117	13.4082	1.4904
z132	3.2379	0.8139	-1.4298
x133	2.9914	-4.5777	-0.0091
y133	5.6112	11.2810	-1.4769
z133	3.7245	-0.3142	-1.8504
x134	1.5765	-5.1335	1.2295
y134	3.6211	9.5663	0.5688
z134	3.6990	-0.3790	-1.8207

x135	0.0415	-6.6044	0.1198
y135	1.8783	6.0754	0.2031
z135	3.7042	0.3964	-1.7212
x136	-1.4696	-6.0101	-1.1855
y136	1.5991	5.2418	-0.1018
z136	3.0525	-1.1714	-2.4128
x137	-0.7879	-6.7333	-2.7288
y137	1.7534	4.5658	-0.6947
z137	2.5300	-1.4817	-2.2742
x138	0.6063	-4.7880	-0.0273
y138	-1.8537	4.3819	-1.1806
z138	4.2471	-0.7321	-2.1022
x139	0.4797	-1.6230	2.0832
y139	1.7500	4.9040	0.3469
z139	1.8604	-0.6402	-3.6275
x140	-2.4507	-2.3372	0.0857
y140	0.8527	3.0543	-1.3873
z140	2.9680	-0.2849	-3.4730
x141	-3.6169	-2.5186	-1.2164
y141	-0.6356	3.4379	-1.4141
z141	4.4912	-1.3903	-4.0733
x142	-3.5459	-0.8912	-1.7243
y142	-2.2109	5.6735	-0.6317
z142	4.1521	-5.5399	-5.3040
x143	-1.9464	-0.6886	-2.2378
y143	0.3566	3.9186	-0.7241
z143	-0.5138	-6.0729	-5.5484
x144	-2.2456	-1.3601	-3.2261

y144	1.4183	2.3555	-0.8843
z144	-2.4759	-3.5021	-3.2043
x145	-0.9959	-2.1201	-2.8242
y145	3.6104	0.9205	1.8585
z145	-3.5422	-2.8616	-5.9677
x146	-0.0210	-2.2909	-2.3132
y146	6.6550	0.6757	2.7721
z146	-4.1476	-2.3484	-6.6320
x147	2.3782	0.0377	-1.3372
y147	5.6934	0.2659	1.9053
z147	-0.4488	-4.1736	-4.9192
x148	4.3026	4.3967	0.9787
y148	6.9607	0.2446	2.0447
z148	-1.2257	-3.5231	-3.5616
x149	3.1372	7.1403	1.6286
y149	6.6742	0.9524	2.1523
z149	0.5966	-6.2102	-4.3269
x150	2.0491	8.1007	1.5630
y150	6.9801	0.2776	1.7031
z150	0.5306	-3.8994	-1.5236
x151	1.5645	8.2814	3.0374
y151	7.0857	-0.2106	1.7214
z151	2.5538	-5.2893	-4.3745
x152	1.4012	9.0200	5.0620
y152	6.9293	-0.4923	1.6535
z152	3.2909	-6.7746	-6.4149
x153	-0.0095	9.5850	5.5900
y153	6.5491	-0.7367	1.9457

z153	3.9571	-5.9844	-3.7150
x154	0.4647	10.3539	6.8995
y154	6.4128	-0.6682	2.0255
z154	1.7536	-3.7563	-1.9356
x155	-0.1560	12.2844	7.6900
y155	6.4345	-2.6454	1.4978
z155	0.0182	-6.8001	-1.4007
x156	-1.8586	14.9256	8.0762
y156	5.8630	-3.0205	1.5532
z156	-1.1427	-7.4901	-1.5741
x157	-1.8162	11.5539	6.4871
y157	6.4398	-2.6057	2.0477
z157	-1.4609	-4.8775	0.5093
x158	-1.3278	9.9145	6.2301
y158	6.4992	-3.8812	0.3289
z158	-1.5290	-5.9373	0.3072
x159	-0.2462	5.5889	5.2977
y159	7.2191	-3.3813	1.1343
z159	-0.1421	-4.1140	2.2643
x160	-0.1588	4.5071	5.8290
y160	7.7022	-3.4491	-0.3532
z160	0.7711	-3.5262	2.7537
x161	-0.5364	2.9709	6.8106
y161	8.4030	-4.4651	-1.9651
z161	1.2006	-4.5173	3.2881
x162	-2.3801	2.6983	7.8883
y162	8.4396	-5.5187	-4.1473
z162	2.6642	-5.9125	2.7712

x163	-2.4665	3.7536	7.4432
y163	7.7366	-3.9054	-2.2747
z163	2.5068	-1.8851	4.9982
x164	-4.4150	7.5915	8.4343
y164	9.0919	-7.5227	-3.6112
z164	1.0779	2.7070	6.5535
x165	-6.9635	4.4913	4.4243
y165	9.9288	-6.8598	-0.8229
z165	-1.4187	11.3648	9.5830
x166	-1.6762	-1.6841	-7.0054
y166	3.3527	-11.7986	5.9072
z166	7.9762	1.6235	4.4661
x167	-1.6146	2.1826	-2.0217
y167	1.7507	-8.1860	4.4132
z167	4.5120	1.0385	2.2752
x168	-2.5219	6.0734	0.0315
y168	0.5503	-5.9654	2.4517
z168	2.3878	1.7435	1.5114
x169	-1.8508	7.7751	1.8058
y169	-0.4515	-4.0332	0.6853
z169	0.1215	3.2321	1.8147
x170	-1.3915	8.1080	3.6308
y170	-1.4721	-3.8985	-2.6862
z170	-1.4963	3.0704	-0.1341
x171	-0.6982	4.9586	2.4271
y171	-2.2780	-2.7243	-4.0998
z171	-2.8799	2.7146	-1.7284
x172	1.1708	2.9477	2.7117

y172	-3.7965	-2.5853	-5.4256
z172	-3.8510	2.5417	-2.1902
x173	2.2026	1.9409	2.5505
y173	-4.9182	-2.5504	-6.4730
z173	-4.8235	3.0489	-2.4837
x174	5.1337	2.1907	3.9375
y174	-6.5971	-3.3820	-7.6192
z174	-4.4904	2.6102	-2.3992
x175	6.3415	0.6840	4.7223
y175	-6.5631	-2.2842	-7.2807
z175	-3.9879	2.3092	-2.6709
x176	6.0397	0.0970	2.6527
y176	-4.2942	-1.5715	-4.8722
z176	-2.2309	2.0955	-4.0553
x177	5.8875	-0.1047	1.6354
y177	-3.4825	-1.1427	-3.8818
z177	-1.6764	1.3437	-4.7755
x178	6.2994	-0.1712	2.3114
y178	-3.5257	-0.3284	-4.2602
z178	-1.1617	0.5307	-4.7851
x179	4.9077	0.3705	2.3460
y179	-0.9212	-0.8255	-4.3541
z179	-1.1063	0.5796	-4.2388
x180	5.2510	-0.1771	0.7362
y180	-1.0303	-0.2349	-2.1922
z180	-1.1008	1.2778	-3.8722
x181	4.6436	1.3112	1.4603
y181	-0.5326	-1.8301	-2.8510

z181	-1.9239	3.6882	-2.2259
x182	3.9264	1.3841	1.4675
y182	1.4157	-0.6701	-2.3430
z182	-3.5981	5.3274	-1.2795
x183	2.8524	2.6310	1.6341
y183	5.5179	0.1034	-0.1483
z183	-4.7523	6.0594	-2.0217
x184	3.1128	2.5876	1.4918
y184	2.1859	0.9107	-0.7336
z184	-5.3192	7.0131	-2.6851
x185	2.0202	3.4118	2.2647
y185	0.6493	-2.4723	-4.5275
z185	-4.5757	5.6256	-3.1185
x186	2.1761	3.9903	2.3571
y186	1.1287	-2.1064	-6.0697
z186	-7.5672	5.1820	-1.8185
x187	1.4809	3.4777	1.3357
y187	-1.1225	0.1788	-3.4017
z187	-9.5006	6.1491	1.3946
x188	-0.2522	4.3360	1.5292
y188	-1.8188	3.1442	-1.8877
z188	-9.1524	6.1755	3.9443
x189	0.9049	2.8403	-0.3775
y189	3.9640	0.0389	-3.0864
z189	-6.0365	2.8882	2.9120
x190	0.2795	1.6433	-1.7998
y190	1.5017	1.7693	0.8302
z190	-4.9457	3.1403	4.3425

x191	-0.6557	0.4241	-3.3941
y191	2.7422	-0.8944	-1.9097
z191	-3.9582	3.6493	5.9050
x192	-3.8005	1.4767	-1.6991
y192	3.8118	-1.5952	1.7138
z192	0.2867	1.9236	5.6862
x193	-2.1030	1.0178	-1.8011
y193	6.6556	-4.3910	3.2217
z193	-0.6696	0.0514	5.7665
x194	-3.3810	0.8688	-2.4454
y194	5.6190	-5.0720	5.7421
z194	0.9976	-0.8096	9.2528
x195	-7.4724	0.8713	-2.7894
y195	1.9916	-2.8586	8.2266
z195	4.3182	0.6773	11.5742
x196	-9.9854	-0.0670	-4.8157
y196	-0.7381	-2.1902	5.9376
z196	5.5485	1.8195	11.2514
x197	-9.2021	1.0466	-5.0661
y197	-1.6669	-0.5650	3.3321
z197	6.4559	1.5195	9.9416
x198	-6.4184	2.2475	1.1879
y198	-1.1143	-2.5764	3.7936
z198	6.1135	1.6314	9.7013
x199	-2.8973	3.8485	6.7850
y199	0.8505	-3.0822	4.2358
z199	5.5011	2.6628	9.7356
x200	-2.3857	3.4831	6.2495

y200	-0.7918	-3.9183	1.7778
z200	2.7585	3.2247	8.1322
x201	-3.5267	5.0887	2.5775
y201	-1.7660	-0.8572	0.0522
z201	-0.4120	7.4021	6.1960
x202	-5.3639	7.5782	1.6628
y202	-5.6197	-1.6347	-5.7675
z202	-7.2672	9.0040	1.7293
x203	-4.7878	9.7608	3.5408
y203	-2.7905	-7.8058	-6.2050
z203	-4.1314	3.9075	2.7515
x204	-5.4881	7.2169	2.7115
y204	-0.1322	-9.5348	-5.6169
z204	1.9542	1.0254	3.3970
x205	-6.5637	6.4785	2.4021
y205	4.1500	-10.1427	-2.7576
z205	3.6406	6.9381	3.3147
x206	-6.6299	5.2750	1.6607
y206	9.1162	-9.2589	1.4295
z206	3.3400	7.6626	2.4575
x207	-5.3296	3.9406	1.2638
y207	11.8193	-5.9872	5.2713
z207	4.1482	4.9088	1.5144
x208	-3.2614	4.1941	2.1483
y208	12.4789	-1.9709	6.9597
z208	2.5235	-0.5794	-1.7681
x209	-1.3500	5.6369	2.9535
y209	8.2914	0.7536	4.3490

z209	1.8414	-5.4130	-3.4645
x210	-0.9218	4.2842	1.8705
y210	0.5322	-0.3939	-1.2991
z210	-0.6526	-3.7483	-3.3298
x211	-0.0887	0.6871	1.3706
y211	-7.3044	-5.3916	-6.0871
z211	-6.2455	0.1914	-5.2425
x212	1.2136	0.2114	1.4485
y212	-11.0999	-7.9104	-3.6900
z212	-9.1684	-0.0295	-4.3124
x213	0.5873	-1.3208	-1.0839
y213	-12.8348	-8.0462	-2.5487
z213	-10.5536	1.0643	-3.0163
x214	0.1087	-1.8862	-3.0250
y214	-13.0071	-7.8091	-0.8387
z214	-10.4629	2.2624	-1.4740
x215	0.7458	-1.6754	-4.0333
y215	-11.7328	-7.8001	1.1606
z215	-9.2372	3.2642	-0.0934
x216	0.8880	-4.1897	-8.8484
y216	-9.4356	-11.2776	-0.8805
z216	-6.1986	6.0447	4.5347
x217	-1.6654	-2.5748	-9.4912
y217	-10.0768	-10.1009	1.2363
z217	-0.2508	5.5928	6.2169
x218	-0.5665	-4.3070	-11.3976
y218	-5.8592	-12.8009	0.3055
z218	2.2737	6.5825	9.1398

x219	-1.4083	-2.7727	-9.3396
y219	-3.2977	-12.0802	3.8169
z219	5.1376	4.5781	7.5128
x220	-2.9955	-0.5747	0.3994
y220	1.6785	4.4138	-0.2303
z220	18.6238	-15.2016	-4.2933
x221	-3.5235	0.7927	1.9430
y221	0.7198	4.7968	1.4027
z221	15.7348	-10.2443	-0.8307
x222	-1.9803	2.1121	5.0008
y222	0.0251	5.1892	2.3642
z222	13.8883	-6.1378	2.7614
x223	0.5382	-0.9917	3.3576
y223	0.2727	5.1332	2.8383
z223	11.9870	-6.2987	3.6753
x224	-0.0043	-2.5920	3.4452
y224	0.2351	4.5922	2.9952
z224	8.4107	-6.5515	5.7296
x225	-0.5359	-3.5314	2.0569
y225	-0.0826	4.7029	3.0186
z225	4.9681	-6.4596	6.6133
x226	-1.2824	-2.3196	1.8777
y226	-0.3524	4.3557	2.5746
z226	1.3985	-3.2059	7.6338
x227	-1.7669	0.1567	4.1194
y227	-0.2153	2.8663	0.7558
z227	-1.2801	0.6182	10.2336
x228	-1.4122	1.2488	6.0067

y228	-0.8061	2.0159	-0.4264
z228	-2.2292	1.3253	10.7156
x229	1.1918	-2.1240	3.6853
y229	0.2042	1.3951	-1.6544
z229	-0.2231	-2.5947	5.2944
x230	3.3394	-2.8991	3.2105
y230	-0.1338	1.7266	-1.9451
z230	0.7690	-3.0951	3.2014
x231	3.9739	-3.3729	3.6215
y231	-0.5864	2.7540	-1.9945
z231	-0.5132	-3.7717	2.8059
x232	5.5465	-1.8349	4.3328
y232	-1.3594	1.4868	-3.0644
z232	0.1319	-2.4575	1.4680
x233	6.0698	-3.6597	2.7903
y233	-1.7248	2.7466	-2.0852
z233	-0.6675	-3.5837	-1.6932
x234	5.9164	-3.7195	3.1881
y234	-1.6955	2.8274	-2.1390
z234	-1.7569	-4.6027	-2.2456
x235	4.8751	-1.7821	2.6277
y235	-0.6505	1.5003	-1.9527
z235	-2.6440	-3.8926	-4.5498
x236	3.8623	-1.9716	2.2312
y236	1.0601	0.9668	-1.8182
z236	-2.1392	-4.8115	-4.8389
x237	3.7465	-1.7893	1.6158
y237	1.6066	1.2130	-1.1935

z237	-1.0864	-5.3169	-5.6558
x238	3.9966	-2.2030	1.2199
y238	1.0724	2.3272	-0.7492
z238	-2.4126	-5.1132	-4.9885
x239	3.4316	-0.5714	2.3841
y239	0.4064	1.2714	-2.5831
z239	-3.8516	-1.4886	-2.3269
x240	4.8489	-1.3196	1.1201
y240	-2.1271	2.3431	-0.9161
z240	-2.7363	-1.8505	-4.3492
x241	5.8095	-0.8012	0.9648
y241	-3.2450	2.1986	0.4088
z241	-3.6262	-0.6861	-4.7920
x242	5.7038	-0.3033	0.5222
y242	-1.0121	-0.7432	-1.1572
z242	-6.7841	0.8918	-4.1229
x243	4.9723	0.9762	1.5294
y243	-0.6217	-1.6167	-2.6694
z243	-6.8602	-0.3704	-2.9069
x244	4.9669	0.3616	0.6752
y244	-3.0780	1.4208	0.0037
z244	-3.4384	-1.6058	-2.3683
x245	3.8177	0.8428	1.1748
y245	-2.3308	0.9108	1.8047
z245	-2.2302	0.6002	-1.3676
x246	3.3504	0.6133	0.7280
y246	0.6147	-2.7903	0.1915
z246	-2.7268	3.8108	1.3941

x247	2.5481	1.9196	0.9165
y247	1.4706	-0.8949	1.3443
z247	-3.1206	0.5498	2.0786
x248	0.8222	2.0351	0.4880
y248	-0.2249	-2.6802	-0.7482
z248	-2.5067	-1.5944	2.1046
x249	1.5591	-0.1121	0.8426
y249	-0.5333	-4.1837	-1.0326
z249	-4.6646	2.3997	1.9333
x250	0.4481	0.9600	0.8860
y250	-2.5456	-1.7639	1.3039
z250	-4.7777	1.1081	3.1755
x251	-0.7196	3.4339	2.6645
y251	-4.5343	-0.0907	3.8135
z251	-5.2181	-3.4916	2.1339
x252	-0.4702	-0.0192	0.2598
y252	-4.1060	-4.7495	2.1713
z252	-5.5459	-2.9577	3.2109
x253	-3.8140	0.4346	-1.2938
y253	-6.5308	-3.7791	2.8963
z253	-3.5369	-2.5550	6.5201
x254	-2.5311	4.6300	0.0240
y254	-8.5336	-1.1677	2.8537
z254	-4.2131	-5.4953	6.2365
x255	-8.1563	8.3516	1.8629
y255	-17.0657	0.4788	3.0490
z255	-1.5048	-8.5655	5.1149
x256	-9.5028	9.8653	3.2612

y256	-21.8978	-0.5523	3.0696
z256	0.9978	-8.3353	5.5677

New Procrustes fit:

Dataset 'BE_Cochlea'

451 landmarks in 3 dimensions.

The dataset contains 32 observations, of which 32 are included for analyses.

Average shape:

Lmk.	Axis 1 (x)	Axis 2 (y)	Axis 3 (z)
1	0.05877580	-0.04667531	0.01801701
2	0.05962066	-0.04487025	0.01793542
3	0.06005017	-0.04301460	0.01758805
4	0.06018825	-0.04102789	0.01698509
5	0.05997332	-0.03903239	0.01610827
6	0.05954585	-0.03723577	0.01506347
7	0.05862282	-0.03555502	0.01375282
8	0.05731222	-0.03408616	0.01225261
9	0.05560439	-0.03288497	0.01057820
10	0.05347188	-0.03194937	0.00861058
11	0.05062401	-0.03103028	0.00641017
12	0.04799972	-0.03034966	0.00466563
13	0.04585368	-0.02996126	0.00339922
14	0.04358156	-0.02989691	0.00228664
15	0.04166877	-0.03033148	0.00158216
16	0.03989504	-0.03105285	0.00121715
17	0.03831066	-0.03194019	0.00116767
18	0.03645372	-0.03322449	0.00146059
19	0.03481507	-0.03479820	0.00225492
20	0.03343377	-0.03681865	0.00327817
21	0.03252415	-0.03895647	0.00432437

22	0.03219559	-0.04106531	0.00547771
23	0.03233126	-0.04308833	0.00659864
24	0.03301622	-0.04508684	0.00786062
25	0.03419896	-0.04713879	0.00920107
26	0.03588820	-0.04913998	0.01052433
27	0.03802368	-0.05111676	0.01189757
28	0.04083396	-0.05300532	0.01310040
29	0.04390119	-0.05449422	0.01413075
30	0.04649016	-0.05535121	0.01525070
31	0.04880712	-0.05532383	0.01598656
32	0.05055348	-0.05488999	0.01645947
33	0.05244782	-0.05407417	0.01699630
34	0.05432022	-0.05282038	0.01748719
35	0.05599132	-0.05123296	0.01784864
36	0.05742757	-0.04918859	0.01799648
37	0.00796688	0.04727679	0.02916560
38	0.00772690	0.05210426	0.02890904
39	0.00718053	0.05698305	0.02631584
40	0.00675666	0.05940990	0.02318183
41	0.00649699	0.06003132	0.02070380
42	0.00626769	0.05971020	0.01831279
43	0.00606234	0.05869019	0.01597498
44	0.00570363	0.05724657	0.01357798
45	0.00526270	0.05660174	0.01168152
46	0.00496687	0.05615289	0.01025701
47	0.00503230	0.05872998	0.00909673
48	0.00489443	0.06075146	0.00746035
49	0.00474147	0.06167771	0.00576857

50	0.00449965	0.06200121	0.00401573
51	0.00414824	0.06193338	0.00218839
52	0.00368755	0.06131051	0.00013210
53	0.00310433	0.05953712	-0.00242719
54	0.00254840	0.05605564	-0.00462411
55	0.00211284	0.05224809	-0.00532753
56	-0.04519183	0.00435963	0.03018831
57	-0.04917633	0.00474008	0.03051463
58	-0.05363058	0.00512857	0.02919284
59	-0.05617507	0.00526689	0.02702704
60	-0.05715319	0.00530614	0.02485186
61	-0.05716557	0.00523439	0.02271843
62	-0.05667410	0.00515312	0.02039673
63	-0.05636188	0.00508541	0.01816311
64	-0.05615519	0.00509014	0.01588401
65	-0.05586330	0.00484961	0.01395646
66	-0.05820874	0.00501670	0.01263134
67	-0.05995323	0.00522247	0.01098426
68	-0.06077710	0.00535437	0.00926151
69	-0.06107289	0.00543619	0.00745131
70	-0.06093483	0.00548187	0.00549732
71	-0.06032544	0.00548634	0.00322566
72	-0.05867577	0.00540296	0.00052616
73	-0.05554794	0.00519083	-0.00204748
74	-0.05186728	0.00494762	-0.00332114
75	-0.01467786	-0.03179747	0.02380551
76	-0.01516309	-0.03507844	0.02373396
77	-0.01588242	-0.03831855	0.02254883

78	-0.01659572	-0.04058230	0.02069099
79	-0.01718341	-0.04180071	0.01866474
80	-0.01764450	-0.04219087	0.01678142
81	-0.01794346	-0.04221257	0.01536279
82	-0.01824473	-0.04231746	0.01365815
83	-0.01858067	-0.04258944	0.01140901
84	-0.01887968	-0.04294714	0.00928584
85	-0.01956109	-0.04457823	0.00785725
86	-0.02016834	-0.04577853	0.00634925
87	-0.02067851	-0.04647198	0.00472613
88	-0.02106955	-0.04663562	0.00298022
89	-0.02137919	-0.04642778	0.00106451
90	-0.02164541	-0.04571495	-0.00122092
91	-0.02181391	-0.04389651	-0.00399107
92	-0.02167689	-0.04056486	-0.00642813
93	-0.02117884	-0.03672599	-0.00767928
94	-0.00426540	0.01787991	-0.00097703
95	-0.00423682	0.02154090	-0.00415794
96	-0.00423341	0.02389732	-0.00801080
97	-0.00436424	0.02487388	-0.01132986
98	-0.00454856	0.02522002	-0.01402951
99	-0.00473563	0.02517144	-0.01618625
100	-0.00493962	0.02454844	-0.01825546
101	-0.00526950	0.02288157	-0.02029303
102	-0.00571272	0.01990598	-0.02189341
103	-0.00614702	0.01626074	-0.02229218
104	-0.02787472	0.00025317	-0.00432986
105	-0.03156823	0.00026259	-0.00622263

106	-0.03438858	0.00027500	-0.00862732
107	-0.03603570	0.00025737	-0.01108417
108	-0.03674463	0.00022646	-0.01329002
109	-0.03679689	0.00018927	-0.01520185
110	-0.03631442	0.00017372	-0.01709593
111	-0.03507344	0.00015179	-0.01902401
112	-0.03277391	0.00009061	-0.02088807
113	-0.02957059	-0.00001028	-0.02209240
114	-0.01674953	-0.01552812	-0.00680430
115	-0.01782179	-0.01873545	-0.00912564
116	-0.01868114	-0.02121717	-0.01123237
117	-0.01940247	-0.02293243	-0.01342088
118	-0.01990120	-0.02370586	-0.01549797
119	-0.02020714	-0.02380052	-0.01739484
120	-0.02037488	-0.02331463	-0.01930986
121	-0.02035667	-0.02187576	-0.02135302
122	-0.02004399	-0.01927457	-0.02319392
123	-0.01947303	-0.01592603	-0.02417448
124	0.06941700	-0.00591468	0.02208691
125	0.06559907	-0.00465598	0.02042177
126	0.05995088	-0.00385371	0.01957695
127	0.05383224	-0.00287838	0.01898104
128	0.04898213	-0.00056467	0.01753870
129	0.04641885	0.00283799	0.01545482
130	0.04562607	0.00603373	0.01384129
131	0.04347964	0.01006094	0.01216210
132	0.04022713	0.01391145	0.01109927
133	0.03688942	0.01749444	0.01113011

134	0.03478733	0.02023648	0.01147239
135	0.03217898	0.02264055	0.01192367
136	0.02931296	0.02497027	0.01248763
137	0.02624981	0.02702056	0.01311328
138	0.02317882	0.02873632	0.01375417
139	0.01966527	0.03017032	0.01466295
140	0.01581670	0.03126639	0.01562314
141	0.01181960	0.03198496	0.01663080
142	0.00785243	0.03250218	0.01762015
143	0.00055755	0.03210642	0.01855797
144	-0.00607250	0.03090562	0.01916962
145	-0.01173381	0.02893352	0.01967074
146	-0.01715667	0.02621488	0.02013400
147	-0.02197252	0.02297317	0.02024218
148	-0.02614652	0.01901930	0.02029874
149	-0.02952407	0.01447271	0.02021824
150	-0.03237551	0.00933827	0.01982166
151	-0.03404169	0.00324481	0.01973812
152	-0.03387699	-0.00121194	0.01947951
153	-0.03316556	-0.00538408	0.01916599
154	-0.03174791	-0.00920682	0.01884025
155	-0.02992206	-0.01245178	0.01862172
156	-0.02765636	-0.01527186	0.01831971
157	-0.02491728	-0.01767327	0.01792175
158	-0.02172262	-0.01983800	0.01743256
159	-0.01801512	-0.02138259	0.01699664
160	-0.01388377	-0.02234237	0.01659208
161	-0.01037269	-0.02266509	0.01576038

162	-0.00707156	-0.02234275	0.01492899
163	-0.00399334	-0.02165879	0.01405961
164	-0.00121345	-0.02047191	0.01312481
165	0.00116485	-0.01898633	0.01227293
166	0.00351098	-0.01722271	0.01099841
167	0.00549261	-0.01503375	0.00969936
168	0.00716002	-0.01245250	0.00847466
169	0.00841765	-0.00928536	0.00729322
170	0.00882584	-0.00628623	0.00578589
171	0.00869180	-0.00347116	0.00452545
172	0.00799667	-0.00104827	0.00372438
173	0.00689620	0.00134231	0.00275250
174	0.00542545	0.00346689	0.00183002
175	0.00354015	0.00529939	0.00100212
176	0.00117217	0.00692459	0.00031887
177	-0.00192464	0.00835286	-0.00029088
178	-0.00506912	0.00915777	-0.00074685
179	-0.00753937	0.00923147	-0.00187748
180	-0.01095734	0.00855800	-0.00298187
181	-0.01358449	0.00737560	-0.00367538
182	-0.01575355	0.00563300	-0.00400636
183	-0.01755849	0.00339540	-0.00427632
184	-0.01864235	0.00129523	-0.00478760
185	-0.01922021	-0.00103731	-0.00486530
186	-0.01912341	-0.00341676	-0.00430981
187	-0.01836855	-0.00608889	-0.00409070
188	-0.01715322	-0.00679821	-0.00554378
189	-0.01505891	-0.00775991	-0.00644198

190	-0.01259182	-0.00851436	-0.00609036
191	-0.01140929	-0.00870403	-0.00622596
192	-0.00958318	-0.00848526	-0.00715401
193	-0.00844862	-0.00786866	-0.00823375
194	-0.00731575	-0.00698256	-0.01042083
195	-0.00611975	-0.00598458	-0.01492912
196	-0.00612833	-0.00579702	-0.01886663
197	0.04651563	0.04282831	0.03047179
198	0.04649574	0.04257970	0.02893064
199	0.04602221	0.04227479	0.02734178
200	0.04435199	0.04233101	0.02553966
201	0.04156948	0.04262508	0.02450017
202	0.03733246	0.04315158	0.02434113
203	0.03176874	0.04392022	0.02491710
204	0.02490951	0.04484767	0.02632673
205	0.01681140	0.04594940	0.02777403
206	0.00795717	0.04730839	0.02915980
207	-0.00134778	0.04579223	0.02986678
208	-0.00995310	0.04341548	0.03008664
209	-0.01761028	0.04016320	0.03042438
210	-0.02427303	0.03604393	0.03069912
211	-0.03022195	0.03119593	0.03059668
212	-0.03526176	0.02550912	0.03062614
213	-0.03943259	0.01917814	0.03053576
214	-0.04279221	0.01208299	0.03033132
215	-0.04522728	0.00432484	0.03018943
216	-0.04454466	-0.00200636	0.02975461
217	-0.04311780	-0.00795560	0.02934352

218	-0.04099884	-0.01322927	0.02882747
219	-0.03815708	-0.01794628	0.02834977
220	-0.03475490	-0.02196916	0.02776404
221	-0.03063926	-0.02538186	0.02688708
222	-0.02579416	-0.02820326	0.02602098
223	-0.02048122	-0.03029040	0.02509449
224	-0.01470077	-0.03181495	0.02381435
225	-0.00937257	-0.03175902	0.02222195
226	-0.00446732	-0.03116002	0.02064212
227	0.00011413	-0.02991630	0.01921203
228	0.00418445	-0.02812396	0.01783879
229	0.00781840	-0.02577208	0.01632315
230	0.01091813	-0.02284388	0.01484105
231	0.01357570	-0.01927767	0.01321082
232	0.01569739	-0.01521238	0.01149110
233	0.01708608	-0.01081127	0.00948379
234	0.01724133	-0.00570197	0.00728473
235	0.01671911	-0.00111114	0.00533429
236	0.01562936	0.00299371	0.00373287
237	0.01394426	0.00674777	0.00254574
238	0.01163655	0.01004557	0.00156465
239	0.00856965	0.01292649	0.00076900
240	0.00478551	0.01532627	0.00019183
241	0.00037482	0.01699645	-0.00038878
242	-0.00453294	0.01794816	-0.00100638
243	-0.00845293	0.01756128	-0.00126253
244	-0.01216947	0.01678653	-0.00158934
245	-0.01558089	0.01557100	-0.00194028

246	-0.01863318	0.01394266	-0.00226457
247	-0.02127505	0.01187152	-0.00266783
248	-0.02353230	0.00941282	-0.00303326
249	-0.02540899	0.00659058	-0.00343200
250	-0.02684963	0.00349530	-0.00390104
251	-0.02801317	-0.00004773	-0.00438136
252	-0.02787426	-0.00277422	-0.00469069
253	-0.02750334	-0.00536901	-0.00496129
254	-0.02683638	-0.00771146	-0.00523555
255	-0.02584270	-0.00976487	-0.00552829
256	-0.02456148	-0.01149362	-0.00583492
257	-0.02304202	-0.01293164	-0.00607222
258	-0.02120587	-0.01407667	-0.00632979
259	-0.01912447	-0.01499400	-0.00653245
260	-0.01636703	-0.01567898	-0.00679349
261	-0.01427720	-0.01548459	-0.00695804
262	-0.01240205	-0.01527414	-0.00730579
263	-0.01047622	-0.01482033	-0.00790946
264	-0.00852160	-0.01407540	-0.00870025
265	-0.00687067	-0.01302190	-0.00969213
266	-0.00572542	-0.01162092	-0.01094398
267	-0.00494511	-0.00948841	-0.01302397
268	-0.00517429	-0.00692758	-0.01626404
269	-0.00612833	-0.00579702	-0.01886663
270	0.06969210	-0.01349819	0.01979498
271	0.07020888	-0.01457501	0.01710319
272	0.07049656	-0.01539571	0.01435687
273	0.07044143	-0.01594716	0.01146778

274	0.07016997	-0.01619773	0.00869077
275	0.06972318	-0.01618350	0.00583085
276	0.06917035	-0.01588037	0.00296012
277	0.06843317	-0.01527417	0.00012780
278	0.06760618	-0.01436134	-0.00269561
279	0.06666446	-0.01302851	-0.00553810
280	0.06565553	-0.01096430	-0.00840288
281	0.06463966	-0.00864347	-0.01092399
282	0.06369978	-0.00604125	-0.01315857
283	0.06276813	-0.00319299	-0.01509299
284	0.06183759	-0.00010722	-0.01672281
285	0.06099901	0.00317373	-0.01806970
286	0.06017080	0.00667550	-0.01903924
287	0.05944167	0.01030807	-0.01968852
288	0.05862305	0.01402412	-0.02001891
289	0.05778975	0.01784895	-0.01981314
290	0.05697143	0.02153840	-0.01933367
291	0.05587485	0.02519729	-0.01861552
292	0.05462691	0.02885766	-0.01774475
293	0.05327633	0.03246907	-0.01670759
294	0.05169288	0.03598446	-0.01550394
295	0.04982327	0.03946272	-0.01413197
296	0.04777881	0.04284470	-0.01264811
297	0.04531307	0.04593092	-0.01087382
298	0.04204507	0.04931141	-0.00869201
299	0.03834257	0.05229765	-0.00665737
300	0.03434476	0.05493889	-0.00480769
301	0.03005176	0.05712137	-0.00308056

302	0.02547324	0.05901879	-0.00150716
303	0.02052011	0.06046798	-0.00008790
304	0.01536202	0.06143009	0.00114700
305	0.00992789	0.06193953	0.00220629
306	0.00434664	0.06201137	0.00308067
307	-0.00759440	0.06065021	0.00475855
308	-0.01825783	0.05760458	0.00613187
309	-0.02769784	0.05326448	0.00714484
310	-0.03595828	0.04791591	0.00780559
311	-0.04324406	0.04164913	0.00815693
312	-0.04955982	0.03437339	0.00819086
313	-0.05491076	0.02599151	0.00791925
314	-0.05884766	0.01633124	0.00732050
315	-0.06104580	0.00546276	0.00648372
316	-0.06108409	-0.00349596	0.00668159
317	-0.05960575	-0.01171485	0.00674546
318	-0.05699936	-0.01910301	0.00662662
319	-0.05347862	-0.02569933	0.00631182
320	-0.04910220	-0.03161924	0.00579496
321	-0.04373667	-0.03678971	0.00510802
322	-0.03732505	-0.04118098	0.00423260
323	-0.02979705	-0.04453288	0.00319081
324	-0.02123232	-0.04656881	0.00202615
325	-0.01328587	-0.04686668	0.00090684
326	-0.00595188	-0.04585974	-0.00031227
327	0.00059669	-0.04388543	-0.00164335
328	0.00646181	-0.04112426	-0.00307071
329	0.01169428	-0.03772807	-0.00470285

330	0.01624462	-0.03335908	-0.00644225
331	0.02014895	-0.02816537	-0.00840435
332	0.02316691	-0.02207268	-0.01043602
333	0.02487581	-0.01518916	-0.01228329
334	0.02523452	-0.00803577	-0.01381920
335	0.02437678	-0.00155817	-0.01497083
336	0.02252940	0.00416591	-0.01579418
337	0.01990610	0.00917193	-0.01634715
338	0.01659768	0.01360712	-0.01667784
339	0.01261723	0.01757384	-0.01675086
340	0.00777608	0.02100475	-0.01651974
341	0.00202621	0.02374493	-0.01597368
342	-0.00465237	0.02521670	-0.01514266
343	-0.01062376	0.02530440	-0.01509515
344	-0.01615362	0.02432390	-0.01502300
345	-0.02099838	0.02253364	-0.01494561
346	-0.02511065	0.02018700	-0.01485578
347	-0.02864553	0.01735823	-0.01475561
348	-0.03165426	0.01398315	-0.01465970
349	-0.03410389	0.00997503	-0.01454631
350	-0.03593260	0.00538537	-0.01441575
351	-0.03681728	0.00019852	-0.01426754
352	-0.03688606	-0.00390312	-0.01436739
353	-0.03633330	-0.00776155	-0.01450749
354	-0.03525023	-0.01128342	-0.01470764
355	-0.03368765	-0.01439149	-0.01493409
356	-0.03174484	-0.01706312	-0.01519577
357	-0.02944260	-0.01933479	-0.01548214

358	-0.02678154	-0.02127103	-0.01578552
359	-0.02368308	-0.02282979	-0.01611051
360	-0.02007482	-0.02379117	-0.01648701
361	-0.01597488	-0.02409289	-0.01759634
362	-0.01212642	-0.02340088	-0.01852789
363	-0.00886268	-0.02199788	-0.01932027
364	-0.00647949	-0.02023273	-0.01992728
365	-0.00450245	-0.01786800	-0.02033606
366	-0.00324710	-0.01506459	-0.02066834
367	-0.00296013	-0.01152839	-0.02065185
368	-0.00426607	-0.00768396	-0.01991126
369	-0.00610085	-0.00580005	-0.01891464
370	0.05140993	-0.01842291	0.00566060
371	0.05257369	-0.01800865	0.00040687
372	0.05339873	-0.01663452	-0.00492395
373	0.05400986	-0.01420312	-0.00956555
374	0.05420271	-0.01103022	-0.01336769
375	0.05393292	-0.00735661	-0.01659678
376	0.05324835	-0.00314133	-0.01924953
377	0.05217755	0.00151823	-0.02140629
378	0.05080984	0.00671696	-0.02294572
379	0.04923591	0.01249247	-0.02373113
380	0.04832505	0.01641936	-0.02380675
381	0.04704530	0.02030713	-0.02355552
382	0.04549287	0.02414021	-0.02298578
383	0.04364571	0.02791058	-0.02201274
384	0.04147542	0.03161267	-0.02071826
385	0.03889399	0.03527949	-0.01929541

386	0.03601098	0.03881362	-0.01752911
387	0.03267524	0.04235080	-0.01565169
388	0.02893169	0.04574416	-0.01366844
389	0.02375214	0.04889568	-0.01133150
390	0.01809623	0.05111990	-0.00928561
391	0.01199648	0.05235305	-0.00748058
392	0.00551080	0.05256393	-0.00598406
393	-0.00120712	0.05173860	-0.00471686
394	-0.00811710	0.04994197	-0.00373254
395	-0.01536374	0.04708882	-0.00309627
396	-0.02287264	0.04321799	-0.00264602
397	-0.03057622	0.03844194	-0.00249842
398	-0.03797906	0.03066347	-0.00253508
399	-0.04383263	0.02309866	-0.00282986
400	-0.04818694	0.01569669	-0.00310478
401	-0.05102262	0.00849189	-0.00330495
402	-0.05238668	0.00143701	-0.00333108
403	-0.05224877	-0.00541040	-0.00336112
404	-0.05063517	-0.01209127	-0.00344116
405	-0.04758290	-0.01859946	-0.00373158
406	-0.04305755	-0.02498091	-0.00415554
407	-0.03852160	-0.02934234	-0.00471880
408	-0.03377762	-0.03268295	-0.00533001
409	-0.02890114	-0.03506147	-0.00621303
410	-0.02379669	-0.03643042	-0.00717208
411	-0.01853829	-0.03682693	-0.00821865
412	-0.01303560	-0.03625554	-0.00940837
413	-0.00729899	-0.03460375	-0.01068935

414	-0.00129997	-0.03183103	-0.01202545
415	0.00470018	-0.02792676	-0.01357854
416	0.00888596	-0.02104325	-0.01556246
417	0.01154658	-0.01473883	-0.01763760
418	0.01275749	-0.00900705	-0.01941793
419	0.01267239	-0.00381444	-0.02068568
420	0.01141103	0.00088810	-0.02152516
421	0.00898673	0.00523484	-0.02198211
422	0.00528378	0.00926679	-0.02224599
423	0.00022776	0.01299689	-0.02228899
424	-0.00627402	0.01639675	-0.02229567
425	-0.01023178	0.01634561	-0.02215636
426	-0.01393416	0.01584275	-0.02204436
427	-0.01730399	0.01487620	-0.02201940
428	-0.02030219	0.01343935	-0.02193570
429	-0.02290505	0.01154876	-0.02195094
430	-0.02512965	0.00922925	-0.02193345
431	-0.02697978	0.00649249	-0.02199986
432	-0.02848060	0.00338189	-0.02204046
433	-0.02962928	-0.00018222	-0.02209474
434	-0.02959244	-0.00300577	-0.02221722
435	-0.02927869	-0.00564607	-0.02237993
436	-0.02866663	-0.00803136	-0.02253198
437	-0.02775557	-0.01012331	-0.02274362
438	-0.02655140	-0.01190423	-0.02295235
439	-0.02510911	-0.01336694	-0.02321805
440	-0.02338677	-0.01454070	-0.02352926
441	-0.02148262	-0.01541095	-0.02385447

442	-0.01901863	-0.01601037	-0.02425300
443	-0.01581823	-0.01543605	-0.02482343
444	-0.01277439	-0.01457023	-0.02525146
445	-0.01043022	-0.01337640	-0.02553250
446	-0.00885076	-0.01202657	-0.02554968
447	-0.00793126	-0.01078536	-0.02534671
448	-0.00722975	-0.00961324	-0.02490340
449	-0.00647238	-0.00824311	-0.02398828
450	-0.00589181	-0.00679044	-0.02205003
451	-0.00610390	-0.00579934	-0.01891597

Procrustes sums of squares: 0.4268211667521373

Tangent sums of squares: 0.4201339065293579

Data matrices in this dataset:

- BE_Cochlea, raw data
- BE_Cochlea, centroid size
- BE_Cochlea, Procrustes coordinates

New Procrustes fit:

Dataset 'BE_Cochlea'

451 landmarks in 3 dimensions.

The dataset contains 32 observations, of which 32 are included for analyses.

Average shape:

Lmk.	Axis 1 (x)	Axis 2 (y)	Axis 3 (z)
1	0.05877580	-0.04667531	0.01801701
2	0.05962066	-0.04487025	0.01793542
3	0.06005017	-0.04301460	0.01758805
4	0.06018825	-0.04102789	0.01698509
5	0.05997332	-0.03903239	0.01610827
6	0.05954585	-0.03723577	0.01506347
7	0.05862282	-0.03555502	0.01375282
8	0.05731222	-0.03408616	0.01225261
9	0.05560439	-0.03288497	0.01057820
10	0.05347188	-0.03194937	0.00861058
11	0.05062401	-0.03103028	0.00641017
12	0.04799972	-0.03034966	0.00466563
13	0.04585368	-0.02996126	0.00339922
14	0.04358156	-0.02989691	0.00228664
15	0.04166877	-0.03033148	0.00158216
16	0.03989504	-0.03105285	0.00121715
17	0.03831066	-0.03194019	0.00116767
18	0.03645372	-0.03322449	0.00146059
19	0.03481507	-0.03479820	0.00225492
20	0.03343377	-0.03681865	0.00327817
21	0.03252415	-0.03895647	0.00432437
22	0.03219559	-0.04106531	0.00547771
23	0.03233126	-0.04308833	0.00659864
24	0.03301622	-0.04508684	0.00786062
25	0.03419896	-0.04713879	0.00920107
26	0.03588820	-0.04913998	0.01052433

27	0.03802368	-0.05111676	0.01189757
28	0.04083396	-0.05300532	0.01310040
29	0.04390119	-0.05449422	0.01413075
30	0.04649016	-0.05535121	0.01525070
31	0.04880712	-0.05532383	0.01598656
32	0.05055348	-0.05488999	0.01645947
33	0.05244782	-0.05407417	0.01699630
34	0.05432022	-0.05282038	0.01748719
35	0.05599132	-0.05123296	0.01784864
36	0.05742757	-0.04918859	0.01799648
37	0.00796688	0.04727679	0.02916560
38	0.00772690	0.05210426	0.02890904
39	0.00718053	0.05698305	0.02631584
40	0.00675666	0.05940990	0.02318183
41	0.00649699	0.06003132	0.02070380
42	0.00626769	0.05971020	0.01831279
43	0.00606234	0.05869019	0.01597498
44	0.00570363	0.05724657	0.01357798
45	0.00526270	0.05660174	0.01168152
46	0.00496687	0.05615289	0.01025701
47	0.00503230	0.05872998	0.00909673
48	0.00489443	0.06075146	0.00746035
49	0.00474147	0.06167771	0.00576857
50	0.00449965	0.06200121	0.00401573
51	0.00414824	0.06193338	0.00218839
52	0.00368755	0.06131051	0.00013210
53	0.00310433	0.05953712	-0.00242719
54	0.00254840	0.05605564	-0.00462411

55	0.00211284	0.05224809	-0.00532753
56	-0.04519183	0.00435963	0.03018831
57	-0.04917633	0.00474008	0.03051463
58	-0.05363058	0.00512857	0.02919284
59	-0.05617507	0.00526689	0.02702704
60	-0.05715319	0.00530614	0.02485186
61	-0.05716557	0.00523439	0.02271843
62	-0.05667410	0.00515312	0.02039673
63	-0.05636188	0.00508541	0.01816311
64	-0.05615519	0.00509014	0.01588401
65	-0.05586330	0.00484961	0.01395646
66	-0.05820874	0.00501670	0.01263134
67	-0.05995323	0.00522247	0.01098426
68	-0.06077710	0.00535437	0.00926151
69	-0.06107289	0.00543619	0.00745131
70	-0.06093483	0.00548187	0.00549732
71	-0.06032544	0.00548634	0.00322566
72	-0.05867577	0.00540296	0.00052616
73	-0.05554794	0.00519083	-0.00204748
74	-0.05186728	0.00494762	-0.00332114
75	-0.01467786	-0.03179747	0.02380551
76	-0.01516309	-0.03507844	0.02373396
77	-0.01588242	-0.03831855	0.02254883
78	-0.01659572	-0.04058230	0.02069099
79	-0.01718341	-0.04180071	0.01866474
80	-0.01764450	-0.04219087	0.01678142
81	-0.01794346	-0.04221257	0.01536279
82	-0.01824473	-0.04231746	0.01365815

83	-0.01858067	-0.04258944	0.01140901
84	-0.01887968	-0.04294714	0.00928584
85	-0.01956109	-0.04457823	0.00785725
86	-0.02016834	-0.04577853	0.00634925
87	-0.02067851	-0.04647198	0.00472613
88	-0.02106955	-0.04663562	0.00298022
89	-0.02137919	-0.04642778	0.00106451
90	-0.02164541	-0.04571495	-0.00122092
91	-0.02181391	-0.04389651	-0.00399107
92	-0.02167689	-0.04056486	-0.00642813
93	-0.02117884	-0.03672599	-0.00767928
94	-0.00426540	0.01787991	-0.00097703
95	-0.00423682	0.02154090	-0.00415794
96	-0.00423341	0.02389732	-0.00801080
97	-0.00436424	0.02487388	-0.01132986
98	-0.00454856	0.02522002	-0.01402951
99	-0.00473563	0.02517144	-0.01618625
100	-0.00493962	0.02454844	-0.01825546
101	-0.00526950	0.02288157	-0.02029303
102	-0.00571272	0.01990598	-0.02189341
103	-0.00614702	0.01626074	-0.02229218
104	-0.02787472	0.00025317	-0.00432986
105	-0.03156823	0.00026259	-0.00622263
106	-0.03438858	0.00027500	-0.00862732
107	-0.03603570	0.00025737	-0.01108417
108	-0.03674463	0.00022646	-0.01329002
109	-0.03679689	0.00018927	-0.01520185
110	-0.03631442	0.00017372	-0.01709593

111	-0.03507344	0.00015179	-0.01902401
112	-0.03277391	0.00009061	-0.02088807
113	-0.02957059	-0.00001028	-0.02209240
114	-0.01674953	-0.01552812	-0.00680430
115	-0.01782179	-0.01873545	-0.00912564
116	-0.01868114	-0.02121717	-0.01123237
117	-0.01940247	-0.02293243	-0.01342088
118	-0.01990120	-0.02370586	-0.01549797
119	-0.02020714	-0.02380052	-0.01739484
120	-0.02037488	-0.02331463	-0.01930986
121	-0.02035667	-0.02187576	-0.02135302
122	-0.02004399	-0.01927457	-0.02319392
123	-0.01947303	-0.01592603	-0.02417448
124	0.06941700	-0.00591468	0.02208691
125	0.06559907	-0.00465598	0.02042177
126	0.05995088	-0.00385371	0.01957695
127	0.05383224	-0.00287838	0.01898104
128	0.04898213	-0.00056467	0.01753870
129	0.04641885	0.00283799	0.01545482
130	0.04562607	0.00603373	0.01384129
131	0.04347964	0.01006094	0.01216210
132	0.04022713	0.01391145	0.01109927
133	0.03688942	0.01749444	0.01113011
134	0.03478733	0.02023648	0.01147239
135	0.03217898	0.02264055	0.01192367
136	0.02931296	0.02497027	0.01248763
137	0.02624981	0.02702056	0.01311328
138	0.02317882	0.02873632	0.01375417

139	0.01966527	0.03017032	0.01466295
140	0.01581670	0.03126639	0.01562314
141	0.01181960	0.03198496	0.01663080
142	0.00785243	0.03250218	0.01762015
143	0.00055755	0.03210642	0.01855797
144	-0.00607250	0.03090562	0.01916962
145	-0.01173381	0.02893352	0.01967074
146	-0.01715667	0.02621488	0.02013400
147	-0.02197252	0.02297317	0.02024218
148	-0.02614652	0.01901930	0.02029874
149	-0.02952407	0.01447271	0.02021824
150	-0.03237551	0.00933827	0.01982166
151	-0.03404169	0.00324481	0.01973812
152	-0.03387699	-0.00121194	0.01947951
153	-0.03316556	-0.00538408	0.01916599
154	-0.03174791	-0.00920682	0.01884025
155	-0.02992206	-0.01245178	0.01862172
156	-0.02765636	-0.01527186	0.01831971
157	-0.02491728	-0.01767327	0.01792175
158	-0.02172262	-0.01983800	0.01743256
159	-0.01801512	-0.02138259	0.01699664
160	-0.01388377	-0.02234237	0.01659208
161	-0.01037269	-0.02266509	0.01576038
162	-0.00707156	-0.02234275	0.01492899
163	-0.00399334	-0.02165879	0.01405961
164	-0.00121345	-0.02047191	0.01312481
165	0.00116485	-0.01898633	0.01227293
166	0.00351098	-0.01722271	0.01099841

167	0.00549261	-0.01503375	0.00969936
168	0.00716002	-0.01245250	0.00847466
169	0.00841765	-0.00928536	0.00729322
170	0.00882584	-0.00628623	0.00578589
171	0.00869180	-0.00347116	0.00452545
172	0.00799667	-0.00104827	0.00372438
173	0.00689620	0.00134231	0.00275250
174	0.00542545	0.00346689	0.00183002
175	0.00354015	0.00529939	0.00100212
176	0.00117217	0.00692459	0.00031887
177	-0.00192464	0.00835286	-0.00029088
178	-0.00506912	0.00915777	-0.00074685
179	-0.00753937	0.00923147	-0.00187748
180	-0.01095734	0.00855800	-0.00298187
181	-0.01358449	0.00737560	-0.00367538
182	-0.01575355	0.00563300	-0.00400636
183	-0.01755849	0.00339540	-0.00427632
184	-0.01864235	0.00129523	-0.00478760
185	-0.01922021	-0.00103731	-0.00486530
186	-0.01912341	-0.00341676	-0.00430981
187	-0.01836855	-0.00608889	-0.00409070
188	-0.01715322	-0.00679821	-0.00554378
189	-0.01505891	-0.00775991	-0.00644198
190	-0.01259182	-0.00851436	-0.00609036
191	-0.01140929	-0.00870403	-0.00622596
192	-0.00958318	-0.00848526	-0.00715401
193	-0.00844862	-0.00786866	-0.00823375
194	-0.00731575	-0.00698256	-0.01042083

195	-0.00611975	-0.00598458	-0.01492912
196	-0.00612833	-0.00579702	-0.01886663
197	0.04651563	0.04282831	0.03047179
198	0.04649574	0.04257970	0.02893064
199	0.04602221	0.04227479	0.02734178
200	0.04435199	0.04233101	0.02553966
201	0.04156948	0.04262508	0.02450017
202	0.03733246	0.04315158	0.02434113
203	0.03176874	0.04392022	0.02491710
204	0.02490951	0.04484767	0.02632673
205	0.01681140	0.04594940	0.02777403
206	0.00795717	0.04730839	0.02915980
207	-0.00134778	0.04579223	0.02986678
208	-0.00995310	0.04341548	0.03008664
209	-0.01761028	0.04016320	0.03042438
210	-0.02427303	0.03604393	0.03069912
211	-0.03022195	0.03119593	0.03059668
212	-0.03526176	0.02550912	0.03062614
213	-0.03943259	0.01917814	0.03053576
214	-0.04279221	0.01208299	0.03033132
215	-0.04522728	0.00432484	0.03018943
216	-0.04454466	-0.00200636	0.02975461
217	-0.04311780	-0.00795560	0.02934352
218	-0.04099884	-0.01322927	0.02882747
219	-0.03815708	-0.01794628	0.02834977
220	-0.03475490	-0.02196916	0.02776404
221	-0.03063926	-0.02538186	0.02688708
222	-0.02579416	-0.02820326	0.02602098

223	-0.02048122	-0.03029040	0.02509449
224	-0.01470077	-0.03181495	0.02381435
225	-0.00937257	-0.03175902	0.02222195
226	-0.00446732	-0.03116002	0.02064212
227	0.00011413	-0.02991630	0.01921203
228	0.00418445	-0.02812396	0.01783879
229	0.00781840	-0.02577208	0.01632315
230	0.01091813	-0.02284388	0.01484105
231	0.01357570	-0.01927767	0.01321082
232	0.01569739	-0.01521238	0.01149110
233	0.01708608	-0.01081127	0.00948379
234	0.01724133	-0.00570197	0.00728473
235	0.01671911	-0.00111114	0.00533429
236	0.01562936	0.00299371	0.00373287
237	0.01394426	0.00674777	0.00254574
238	0.01163655	0.01004557	0.00156465
239	0.00856965	0.01292649	0.00076900
240	0.00478551	0.01532627	0.00019183
241	0.00037482	0.01699645	-0.00038878
242	-0.00453294	0.01794816	-0.00100638
243	-0.00845293	0.01756128	-0.00126253
244	-0.01216947	0.01678653	-0.00158934
245	-0.01558089	0.01557100	-0.00194028
246	-0.01863318	0.01394266	-0.00226457
247	-0.02127505	0.01187152	-0.00266783
248	-0.02353230	0.00941282	-0.00303326
249	-0.02540899	0.00659058	-0.00343200
250	-0.02684963	0.00349530	-0.00390104

251	-0.02801317	-0.00004773	-0.00438136
252	-0.02787426	-0.00277422	-0.00469069
253	-0.02750334	-0.00536901	-0.00496129
254	-0.02683638	-0.00771146	-0.00523555
255	-0.02584270	-0.00976487	-0.00552829
256	-0.02456148	-0.01149362	-0.00583492
257	-0.02304202	-0.01293164	-0.00607222
258	-0.02120587	-0.01407667	-0.00632979
259	-0.01912447	-0.01499400	-0.00653245
260	-0.01636703	-0.01567898	-0.00679349
261	-0.01427720	-0.01548459	-0.00695804
262	-0.01240205	-0.01527414	-0.00730579
263	-0.01047622	-0.01482033	-0.00790946
264	-0.00852160	-0.01407540	-0.00870025
265	-0.00687067	-0.01302190	-0.00969213
266	-0.00572542	-0.01162092	-0.01094398
267	-0.00494511	-0.00948841	-0.01302397
268	-0.00517429	-0.00692758	-0.01626404
269	-0.00612833	-0.00579702	-0.01886663
270	0.06969210	-0.01349819	0.01979498
271	0.07020888	-0.01457501	0.01710319
272	0.07049656	-0.01539571	0.01435687
273	0.07044143	-0.01594716	0.01146778
274	0.07016997	-0.01619773	0.00869077
275	0.06972318	-0.01618350	0.00583085
276	0.06917035	-0.01588037	0.00296012
277	0.06843317	-0.01527417	0.00012780
278	0.06760618	-0.01436134	-0.00269561

279	0.06666446	-0.01302851	-0.00553810
280	0.06565553	-0.01096430	-0.00840288
281	0.06463966	-0.00864347	-0.01092399
282	0.06369978	-0.00604125	-0.01315857
283	0.06276813	-0.00319299	-0.01509299
284	0.06183759	-0.00010722	-0.01672281
285	0.06099901	0.00317373	-0.01806970
286	0.06017080	0.00667550	-0.01903924
287	0.05944167	0.01030807	-0.01968852
288	0.05862305	0.01402412	-0.02001891
289	0.05778975	0.01784895	-0.01981314
290	0.05697143	0.02153840	-0.01933367
291	0.05587485	0.02519729	-0.01861552
292	0.05462691	0.02885766	-0.01774475
293	0.05327633	0.03246907	-0.01670759
294	0.05169288	0.03598446	-0.01550394
295	0.04982327	0.03946272	-0.01413197
296	0.04777881	0.04284470	-0.01264811
297	0.04531307	0.04593092	-0.01087382
298	0.04204507	0.04931141	-0.00869201
299	0.03834257	0.05229765	-0.00665737
300	0.03434476	0.05493889	-0.00480769
301	0.03005176	0.05712137	-0.00308056
302	0.02547324	0.05901879	-0.00150716
303	0.02052011	0.06046798	-0.00008790
304	0.01536202	0.06143009	0.00114700
305	0.00992789	0.06193953	0.00220629
306	0.00434664	0.06201137	0.00308067

307	-0.00759440	0.06065021	0.00475855
308	-0.01825783	0.05760458	0.00613187
309	-0.02769784	0.05326448	0.00714484
310	-0.03595828	0.04791591	0.00780559
311	-0.04324406	0.04164913	0.00815693
312	-0.04955982	0.03437339	0.00819086
313	-0.05491076	0.02599151	0.00791925
314	-0.05884766	0.01633124	0.00732050
315	-0.06104580	0.00546276	0.00648372
316	-0.06108409	-0.00349596	0.00668159
317	-0.05960575	-0.01171485	0.00674546
318	-0.05699936	-0.01910301	0.00662662
319	-0.05347862	-0.02569933	0.00631182
320	-0.04910220	-0.03161924	0.00579496
321	-0.04373667	-0.03678971	0.00510802
322	-0.03732505	-0.04118098	0.00423260
323	-0.02979705	-0.04453288	0.00319081
324	-0.02123232	-0.04656881	0.00202615
325	-0.01328587	-0.04686668	0.00090684
326	-0.00595188	-0.04585974	-0.00031227
327	0.00059669	-0.04388543	-0.00164335
328	0.00646181	-0.04112426	-0.00307071
329	0.01169428	-0.03772807	-0.00470285
330	0.01624462	-0.03335908	-0.00644225
331	0.02014895	-0.02816537	-0.00840435
332	0.02316691	-0.02207268	-0.01043602
333	0.02487581	-0.01518916	-0.01228329
334	0.02523452	-0.00803577	-0.01381920

335	0.02437678	-0.00155817	-0.01497083
336	0.02252940	0.00416591	-0.01579418
337	0.01990610	0.00917193	-0.01634715
338	0.01659768	0.01360712	-0.01667784
339	0.01261723	0.01757384	-0.01675086
340	0.00777608	0.02100475	-0.01651974
341	0.00202621	0.02374493	-0.01597368
342	-0.00465237	0.02521670	-0.01514266
343	-0.01062376	0.02530440	-0.01509515
344	-0.01615362	0.02432390	-0.01502300
345	-0.02099838	0.02253364	-0.01494561
346	-0.02511065	0.02018700	-0.01485578
347	-0.02864553	0.01735823	-0.01475561
348	-0.03165426	0.01398315	-0.01465970
349	-0.03410389	0.00997503	-0.01454631
350	-0.03593260	0.00538537	-0.01441575
351	-0.03681728	0.00019852	-0.01426754
352	-0.03688606	-0.00390312	-0.01436739
353	-0.03633330	-0.00776155	-0.01450749
354	-0.03525023	-0.01128342	-0.01470764
355	-0.03368765	-0.01439149	-0.01493409
356	-0.03174484	-0.01706312	-0.01519577
357	-0.02944260	-0.01933479	-0.01548214
358	-0.02678154	-0.02127103	-0.01578552
359	-0.02368308	-0.02282979	-0.01611051
360	-0.02007482	-0.02379117	-0.01648701
361	-0.01597488	-0.02409289	-0.01759634
362	-0.01212642	-0.02340088	-0.01852789

363	-0.00886268	-0.02199788	-0.01932027
364	-0.00647949	-0.02023273	-0.01992728
365	-0.00450245	-0.01786800	-0.02033606
366	-0.00324710	-0.01506459	-0.02066834
367	-0.00296013	-0.01152839	-0.02065185
368	-0.00426607	-0.00768396	-0.01991126
369	-0.00610085	-0.00580005	-0.01891464
370	0.05140993	-0.01842291	0.00566060
371	0.05257369	-0.01800865	0.00040687
372	0.05339873	-0.01663452	-0.00492395
373	0.05400986	-0.01420312	-0.00956555
374	0.05420271	-0.01103022	-0.01336769
375	0.05393292	-0.00735661	-0.01659678
376	0.05324835	-0.00314133	-0.01924953
377	0.05217755	0.00151823	-0.02140629
378	0.05080984	0.00671696	-0.02294572
379	0.04923591	0.01249247	-0.02373113
380	0.04832505	0.01641936	-0.02380675
381	0.04704530	0.02030713	-0.02355552
382	0.04549287	0.02414021	-0.02298578
383	0.04364571	0.02791058	-0.02201274
384	0.04147542	0.03161267	-0.02071826
385	0.03889399	0.03527949	-0.01929541
386	0.03601098	0.03881362	-0.01752911
387	0.03267524	0.04235080	-0.01565169
388	0.02893169	0.04574416	-0.01366844
389	0.02375214	0.04889568	-0.01133150
390	0.01809623	0.05111990	-0.00928561

391	0.01199648	0.05235305	-0.00748058
392	0.00551080	0.05256393	-0.00598406
393	-0.00120712	0.05173860	-0.00471686
394	-0.00811710	0.04994197	-0.00373254
395	-0.01536374	0.04708882	-0.00309627
396	-0.02287264	0.04321799	-0.00264602
397	-0.03057622	0.03844194	-0.00249842
398	-0.03797906	0.03066347	-0.00253508
399	-0.04383263	0.02309866	-0.00282986
400	-0.04818694	0.01569669	-0.00310478
401	-0.05102262	0.00849189	-0.00330495
402	-0.05238668	0.00143701	-0.00333108
403	-0.05224877	-0.00541040	-0.00336112
404	-0.05063517	-0.01209127	-0.00344116
405	-0.04758290	-0.01859946	-0.00373158
406	-0.04305755	-0.02498091	-0.00415554
407	-0.03852160	-0.02934234	-0.00471880
408	-0.03377762	-0.03268295	-0.00533001
409	-0.02890114	-0.03506147	-0.00621303
410	-0.02379669	-0.03643042	-0.00717208
411	-0.01853829	-0.03682693	-0.00821865
412	-0.01303560	-0.03625554	-0.00940837
413	-0.00729899	-0.03460375	-0.01068935
414	-0.00129997	-0.03183103	-0.01202545
415	0.00470018	-0.02792676	-0.01357854
416	0.00888596	-0.02104325	-0.01556246
417	0.01154658	-0.01473883	-0.01763760
418	0.01275749	-0.00900705	-0.01941793

419	0.01267239	-0.00381444	-0.02068568
420	0.01141103	0.00088810	-0.02152516
421	0.00898673	0.00523484	-0.02198211
422	0.00528378	0.00926679	-0.02224599
423	0.00022776	0.01299689	-0.02228899
424	-0.00627402	0.01639675	-0.02229567
425	-0.01023178	0.01634561	-0.02215636
426	-0.01393416	0.01584275	-0.02204436
427	-0.01730399	0.01487620	-0.02201940
428	-0.02030219	0.01343935	-0.02193570
429	-0.02290505	0.01154876	-0.02195094
430	-0.02512965	0.00922925	-0.02193345
431	-0.02697978	0.00649249	-0.02199986
432	-0.02848060	0.00338189	-0.02204046
433	-0.02962928	-0.00018222	-0.02209474
434	-0.02959244	-0.00300577	-0.02221722
435	-0.02927869	-0.00564607	-0.02237993
436	-0.02866663	-0.00803136	-0.02253198
437	-0.02775557	-0.01012331	-0.02274362
438	-0.02655140	-0.01190423	-0.02295235
439	-0.02510911	-0.01336694	-0.02321805
440	-0.02338677	-0.01454070	-0.02352926
441	-0.02148262	-0.01541095	-0.02385447
442	-0.01901863	-0.01601037	-0.02425300
443	-0.01581823	-0.01543605	-0.02482343
444	-0.01277439	-0.01457023	-0.02525146
445	-0.01043022	-0.01337640	-0.02553250
446	-0.00885076	-0.01202657	-0.02554968

447	-0.00793126	-0.01078536	-0.02534671
448	-0.00722975	-0.00961324	-0.02490340
449	-0.00647238	-0.00824311	-0.02398828
450	-0.00589181	-0.00679044	-0.02205003
451	-0.00610390	-0.00579934	-0.01891597

Procrustes sums of squares: 0.4268211667521373

Tangent sums of squares: 0.4201339065293579

Data matrices in this dataset:

- BE_Cochlea, raw data
- BE_Cochlea, centroid size
- BE_Cochlea, Procrustes coordinates

Principal Component Analysis: PCA: CovMatrix, BE_Cochlea, Procrustes coordinates

	Eigenvalues	% Variance	Cumulative %
1.	0.00248578	18.342	18.342
2.	0.00215649	15.912	34.253
3.	0.00183003	13.503	47.757
4.	0.00109268	8.062	55.819
5.	0.00083158	6.136	61.955
6.	0.00070890	5.231	67.186
7.	0.00068274	5.038	72.223
8.	0.00049196	3.630	75.853

9.	0.00040647	2.999	78.852
10.	0.00031477	2.323	81.175
11.	0.00029140	2.150	83.325
12.	0.00026449	1.952	85.277
13.	0.00022785	1.681	86.958
14.	0.00021244	1.568	88.525
15.	0.00018942	1.398	89.923
16.	0.00016587	1.224	91.147
17.	0.00014816	1.093	92.240
18.	0.00014574	1.075	93.316
19.	0.00012623	0.931	94.247
20.	0.00011599	0.856	95.103
21.	0.00009822	0.725	95.828
22.	0.00009482	0.700	96.527
23.	0.00008461	0.624	97.151
24.	0.00007187	0.530	97.682
25.	0.00006376	0.470	98.152
26.	0.00005824	0.430	98.582
27.	0.00004944	0.365	98.947
28.	0.00004536	0.335	99.281
29.	0.00003949	0.291	99.573
30.	0.00003113	0.230	99.803
31.	0.00002676	0.197	100.000

Total variance: 0.01355271

Variance of the eigenvalues: 0.0000000132516

Eigenvalue variance scaled by total variance: 0.00007

Eigenvalue variance scaled by total variance and number of variables: 0.09718

Note: throughout all calculations of eigenvalue variances, the dimensionality used was 1346.

Please double-check because this dimensionality may not be appropriate for all situations.

Warning: There appear to be fewer PCs than dimensions in shape space. This can have the following reasons, among others:

-- the degrees of freedom are less than the shape dimensionality (sample size, etc.).

-- there is complete integration in the data.

Interpret the results cautiously and check for reasons why this is the case.

Principal Component Coefficients

PC1 PC2 PC3 PC4 PC5 PC6 PC7 PC8 PC9 PC10 PC11 PC12
PC13 PC14 PC15 PC16 PC17 PC18 PC19 PC20 PC21 PC22 PC23 PC24
PC25 PC26 PC27 PC28 PC29 PC30 PC31

x1	-0.022048	-0.045012	0.026469	0.032407	0.060998	-0.018527
	0.017977	0.027430	-0.025881	-0.010905	-0.019048	-0.047341
	-0.012548	0.003773	0.048401	-0.051708	0.090533	-0.060278
	-0.018441	0.013835	0.022053	0.081951	0.052244	0.028803
	0.004238	-0.042401	-0.000789	0.008197	0.017918	-0.004756
	0.024741					
y1	0.014201	-0.003073	0.087824	-0.009727	0.078339	-0.045309
	0.101854	0.023964	-0.058459	-0.055184	-0.005566	-0.022622
	-0.019138	-0.086668	-0.056251	-0.013846	-0.043479	-0.138778
	0.019520	-0.001915	-0.043079	0.025686	0.026738	0.020322
	0.037307	-0.011196	0.030370	0.086480	0.058996	-0.026547
	0.053144					
z1	-0.019478	0.090395	-0.028370	-0.068754	0.024489	0.023955
	0.067716	-0.024337	-0.022693	-0.039310	0.000133	-0.067812
	-0.016159	0.039568	-0.017503	-0.007419	0.013502	0.004425
	0.006846	-0.016990	0.002009	0.013685	0.025829	-0.057505
	0.087222	0.018480	-0.006348	0.061694	0.013864	-0.017645
	-0.020142					
x2	-0.020017	-0.041883	0.016038	0.027871	0.049690	-0.010946
	0.014300	0.012301	-0.018202	0.003841	-0.023834	-0.034175
	0.002324	0.014227	0.042801	-0.056343	0.078608	-0.039041
	-0.030262	0.017821	0.019864	0.054036	0.037038	0.008492

	0.006203	-0.030083	-0.016300	0.008450	0.017758	-0.002087
	0.020783					
y2	0.014540	-0.002301	0.082642	-0.013225	0.073020	-0.039253
	0.096887	0.027105	-0.057861	-0.052226	-0.005046	-0.021511
	-0.018600	-0.079849	-0.049859	-0.008204	-0.032236	-0.132056
	0.018796	-0.008635	-0.050901	0.023610	0.016081	0.017013
	0.020137	-0.017746	0.033813	0.076362	0.062166	-0.021294
	0.038077					
z2	-0.018222	0.089802	-0.030863	-0.068345	0.018645	0.028154
	0.066213	-0.019082	-0.018361	-0.032468	0.000952	-0.070872
	-0.015465	0.043595	-0.007613	0.000608	0.011499	0.016927
	0.005185	-0.034512	0.000030	0.004419	0.021244	-0.052768
	0.067604	0.020734	-0.008823	0.044305	0.009742	-0.033783
	-0.024986					
x3	-0.018083	-0.040703	0.008558	0.025797	0.037708	-0.005646
	0.002690	-0.001970	-0.009997	0.014668	-0.031111	-0.020641
	0.017446	0.020905	0.046493	-0.051186	0.064933	-0.016934
	-0.040458	0.021742	0.024214	0.039636	0.035652	-0.001953
	0.021483	-0.011357	-0.040242	0.003777	0.014615	0.007687
	0.028303					
y3	0.015623	-0.001752	0.077202	-0.014890	0.068140	-0.031480
	0.090136	0.026828	-0.056773	-0.045060	-0.006156	-0.018622
	-0.013222	-0.074142	-0.038816	-0.003824	-0.024937	-0.118390
	0.015070	-0.018530	-0.053738	0.019649	0.010631	0.008947
	0.015307	-0.014626	0.031862	0.063069	0.059212	-0.017882
	0.028761					
z3	-0.016294	0.087319	-0.029762	-0.066446	0.015780	0.030837
	0.064379	-0.012054	-0.015500	-0.028318	0.003496	-0.072226
	-0.012054	0.047223	0.004207	0.009067	0.008493	0.023236
	0.003129	-0.046028	-0.001003	-0.001829	0.018032	-0.048335
	0.047852	0.020950	-0.009335	0.037106	0.005943	-0.038426
	-0.025587					
x4	-0.016326	-0.040310	0.002881	0.021670	0.026684	-0.002671
	-0.005691	-0.012137	-0.002797	0.021574	-0.036662	-0.005746
	0.025677	0.024230	0.050004	-0.044437	0.048771	0.000451
	-0.048853	0.027504	0.029874	0.025315	0.032471	-0.007779
	0.035592	0.002756	-0.059186	-0.005075	0.015039	0.019902
	0.021873					
y4	0.016100	-0.000170	0.071176	-0.018978	0.061962	-0.024854
	0.086929	0.026941	-0.057341	-0.036025	-0.005961	-0.015581
	-0.007395	-0.064913	-0.031533	-0.001110	-0.018892	-0.103188

	0.011928	-0.029189	-0.059602	0.012021	0.000371	0.001347
	0.003845	-0.012100	0.026850	0.051046	0.056052	-0.014202
	0.018175					
z4	-0.013825	0.083800	-0.026868	-0.064438	0.014306	0.032665
	0.063498	-0.002564	-0.012846	-0.024476	0.007118	-0.070729
	-0.011880	0.047309	0.014485	0.016690	0.003601	0.024341
	-0.000827	-0.052240	-0.000306	-0.008099	0.014443	-0.042389
	0.026969	0.016372	-0.006566	0.028153	0.003022	-0.037942
	-0.029399					
x5	-0.016625	-0.039070	-0.001321	0.015609	0.019809	-0.001186
	-0.009102	-0.019259	0.004408	0.021822	-0.039439	0.003371
	0.028302	0.027671	0.048747	-0.040839	0.038596	0.012562
	-0.053207	0.027066	0.033851	0.009300	0.024097	-0.006744
	0.042294	0.013705	-0.066819	-0.001859	0.025219	0.034083
	0.013945					
y5	0.016289	0.001489	0.064918	-0.023519	0.054554	-0.018495
	0.084645	0.027497	-0.057433	-0.026003	-0.005779	-0.011952
	-0.001460	-0.054131	-0.027265	0.000643	-0.014964	-0.087815
	0.007531	-0.039028	-0.064087	0.002680	-0.013374	-0.002751
	-0.011671	-0.009105	0.016837	0.040523	0.053153	-0.008539
	0.011665					
z5	-0.011431	0.079719	-0.023122	-0.060962	0.013933	0.035245
	0.060839	0.006763	-0.008560	-0.019143	0.009881	-0.067656
	-0.013367	0.043816	0.022690	0.021308	-0.002994	0.023522
	-0.005462	-0.058983	0.004463	-0.009299	0.012462	-0.034170
	0.010672	0.010677	-0.003358	0.014978	-0.004076	-0.033982
	-0.026881					
x6	-0.018521	-0.037187	-0.004310	0.007071	0.012668	-0.002394
	-0.002925	-0.024086	0.006607	0.019926	-0.040034	0.005406
	0.025317	0.034845	0.037276	-0.038504	0.031830	0.016883
	-0.048625	0.024912	0.028549	0.000265	0.007999	-0.000681
	0.028288	0.020618	-0.067690	0.016148	0.031732	0.044513
	0.006077					
y6	0.016344	0.002363	0.058593	-0.026666	0.046209	-0.012873
	0.080769	0.026091	-0.058653	-0.015869	-0.007560	-0.007357
	0.005817	-0.044288	-0.019848	0.003644	-0.014462	-0.071878
	0.006353	-0.048768	-0.061575	-0.001693	-0.021712	-0.001380
	-0.021383	-0.001469	0.006642	0.029713	0.042216	-0.001034
	0.007150					
z6	-0.009320	0.075827	-0.019682	-0.057397	0.013661	0.037063
	0.059684	0.013973	-0.006092	-0.013167	0.012219	-0.063735

	-0.014802	0.040782	0.028596	0.022709	-0.010142	0.019267
	-0.007537	-0.064088	0.011287	-0.006614	0.009513	-0.023182
	-0.006936	0.006798	0.002167	0.005767	-0.014405	-0.030393
	-0.023773					
x7	-0.021267	-0.037122	-0.006933	0.001981	0.005525	-0.002469
	-0.002673	-0.025629	0.008815	0.016090	-0.042290	0.006766
	0.022244	0.036930	0.027486	-0.034152	0.029591	0.019835
	-0.053363	0.022713	0.011778	-0.010866	-0.003587	0.009520
	0.022401	0.027814	-0.068342	0.025333	0.022557	0.034071
	0.007531					
y7	0.016048	0.002549	0.052431	-0.029529	0.037279	-0.008161
	0.072718	0.024093	-0.059456	-0.005926	-0.010624	0.000025
	0.015836	-0.035998	-0.013226	0.006263	-0.009056	-0.056529
	-0.001437	-0.056608	-0.065467	-0.008599	-0.029340	0.002938
	-0.026644	0.007390	-0.008274	0.013583	0.028069	0.001656
	0.004673					
z7	-0.006406	0.071206	-0.015924	-0.053401	0.014014	0.039523
	0.055346	0.019577	-0.002876	-0.006922	0.012576	-0.055618
	-0.014953	0.035332	0.033968	0.023418	-0.016351	0.014648
	-0.012753	-0.065116	0.019747	-0.002375	0.006460	-0.013141
	-0.017571	0.004910	0.009951	-0.005232	-0.026270	-0.024099
	-0.023236					
x8	-0.022930	-0.038901	-0.009364	-0.001248	-0.000997	-0.002116
	-0.005824	-0.025472	0.014904	0.012831	-0.043211	0.006677
	0.018377	0.036381	0.023372	-0.033230	0.022399	0.032643
	-0.054463	0.011021	-0.002564	-0.018034	-0.010063	0.017298
	0.015865	0.036821	-0.060953	0.029239	0.007882	0.024033
	0.020797					
y8	0.016606	0.001110	0.046274	-0.031969	0.029124	-0.004741
	0.063094	0.021563	-0.057266	0.004776	-0.013534	0.008656
	0.027625	-0.026819	-0.006321	0.003600	-0.008065	-0.036353
	-0.004539	-0.068157	-0.066602	-0.013554	-0.033530	0.006115
	-0.031197	0.016895	-0.019284	-0.003950	0.011379	0.006431
	0.014512					
z8	-0.002747	0.066217	-0.012111	-0.049279	0.014675	0.041970
	0.051103	0.022650	0.000825	-0.000001	0.012225	-0.046222
	-0.014022	0.029482	0.035812	0.019665	-0.021190	0.008641
	-0.016228	-0.063453	0.027625	0.002031	0.002095	-0.003851
	-0.029520	0.005022	0.016317	-0.013251	-0.035172	-0.012722
	-0.017255					

x9	-0.023199	-0.041113	-0.009038	-0.004658	-0.006880	-0.001984
	-0.007658	-0.024073	0.022162	0.012629	-0.037686	0.000625
	0.008545	0.030636	0.025661	-0.028714	0.011970	0.047858
	-0.051507	-0.003567	-0.011536	-0.024694	-0.007144	0.023154
	0.005101	0.049950	-0.046528	0.025075	-0.001465	0.020184
	0.017173					
y9	0.017035	-0.000538	0.041403	-0.035148	0.020863	-0.004430
	0.053887	0.021498	-0.053544	0.019587	-0.012622	0.017430
	0.032266	-0.023013	0.005054	0.002781	-0.016340	-0.014422
	-0.003929	-0.078420	-0.057717	-0.018639	-0.022671	0.009863
	-0.032920	0.025999	-0.027472	-0.031926	-0.007661	0.005675
	0.005215					
z9	0.001105	0.061239	-0.007939	-0.044521	0.016602	0.043552
	0.047900	0.023582	0.003519	0.006998	0.011698	-0.035599
	-0.014072	0.022145	0.035315	0.013515	-0.026720	-0.001010
	-0.017555	-0.056950	0.035251	0.004890	-0.000795	0.006556
	-0.037146	0.003982	0.021918	-0.017375	-0.043842	-0.009110
	-0.008433					
x10	-0.024425	-0.043801	-0.006756	-0.003331	-0.010767	-0.004800
	-0.006192	-0.020603	0.029905	0.006492	-0.029059	-0.008496
	-0.009232	0.024517	0.026266	-0.028550	-0.004001	0.059457
	-0.044959	-0.011289	-0.021159	-0.028950	-0.001785	0.033715
	-0.004811	0.057378	-0.033477	0.042868	-0.008146	-0.006959
	0.001774					
y10	0.019494	-0.005178	0.038844	-0.032351	0.013600	-0.003062
	0.042637	0.014484	-0.043018	0.028660	-0.013604	0.019879
	0.036157	-0.022626	0.014633	0.002272	-0.010527	-0.001550
	-0.016335	-0.077302	-0.047115	-0.023264	-0.015173	0.001179
	-0.021930	0.029328	-0.042957	-0.052371	-0.000310	0.003562
	-0.004003					
z10	0.006545	0.056570	-0.004618	-0.041385	0.018937	0.046916
	0.043392	0.019814	0.006858	0.010786	0.006075	-0.027522
	-0.009597	0.015587	0.033955	0.016213	-0.016370	-0.017469
	-0.023445	-0.044450	0.036710	0.006534	-0.006593	0.009403
	-0.031509	-0.003656	0.026035	-0.035549	-0.036719	0.016991
	0.002115					
x11	-0.021971	-0.043730	-0.003576	-0.002385	-0.012530	-0.005696
	-0.000797	-0.018846	0.035049	-0.000278	-0.026482	-0.008554
	-0.020572	0.019671	0.036891	-0.026621	-0.020880	0.062003
	-0.027305	-0.014906	-0.025639	-0.031646	0.008269	0.036924
	-0.013947	0.061895	-0.018349	0.064357	-0.017658	-0.019880
	-0.016586					

y11	0.024853	-0.008982	0.036461	-0.031589	0.006698	-0.002633
	0.034070	0.010977	-0.038145	0.028886	-0.017444	0.021665
	0.042061	-0.017185	0.020885	0.000536	-0.009591	-0.006128
	-0.015740	-0.068763	-0.039598	-0.022741	0.003948	-0.000300
	-0.001154	0.029488	-0.046493	-0.044051	0.005639	0.001535
	-0.028443					
z11	0.010579	0.048723	-0.001512	-0.038671	0.020535	0.050606
	0.028667	0.020627	0.017350	0.008267	0.007976	-0.031698
	-0.020379	0.012217	0.019067	0.018137	0.001290	-0.023596
	-0.036935	-0.034829	0.017445	-0.007085	-0.000171	0.013707
	-0.000269	-0.023245	0.035122	-0.078733	-0.020125	0.029131
	0.005708					
x12	-0.021212	-0.041955	-0.001249	-0.003270	-0.012440	-0.005759
	0.003035	-0.018649	0.036218	-0.006347	-0.027104	-0.011738
	-0.026479	0.017595	0.049146	-0.022422	-0.028102	0.060869
	-0.015127	-0.018461	-0.025364	-0.028024	0.013330	0.034733
	-0.024291	0.066951	-0.007869	0.074167	-0.020848	-0.017188
	-0.026216					
y12	0.028547	-0.010180	0.036291	-0.033467	0.008779	-0.008230
	0.025144	0.015261	-0.034299	0.021201	-0.023424	0.029890
	0.042055	-0.013962	0.022191	-0.000935	-0.014395	-0.009151
	-0.017597	-0.052934	-0.024589	-0.015654	0.016418	-0.004801
	0.006305	0.026034	-0.040951	-0.045117	0.005672	-0.000423
	-0.026325					
z12	0.014635	0.042830	0.001046	-0.034820	0.022261	0.056395
	0.014960	0.023617	0.025242	0.008972	0.012452	-0.029365
	-0.028186	0.012487	0.003712	0.010244	0.011884	-0.023064
	-0.042564	-0.025348	0.001936	-0.020885	0.005633	0.014870
	0.022963	-0.040020	0.039618	-0.110187	-0.009248	0.026908
	0.009813					
x13	-0.021340	-0.039223	0.001032	-0.004395	-0.009884	-0.005871
	0.003204	-0.017920	0.042552	-0.009599	-0.028973	-0.016496
	-0.036060	0.016273	0.048226	-0.017953	-0.023220	0.061130
	-0.014852	-0.022267	-0.034539	-0.036493	0.011362	0.023267
	-0.024425	0.059839	-0.012322	0.059754	-0.007615	-0.010854
	-0.019413					
y13	0.029956	-0.008871	0.036827	-0.034635	0.011222	-0.017372
	0.016361	0.019673	-0.022326	0.011783	-0.028627	0.036333
	0.033690	-0.018307	0.010937	0.002483	-0.011606	-0.008347
	-0.030138	-0.042810	-0.022671	-0.021498	0.018455	-0.011452
	0.016399	0.007202	-0.043221	-0.067849	0.011326	-0.002879
	-0.011285					

z13	0.018755	0.039046	0.003139	-0.033936	0.024344	0.061676
	0.002432	0.026687	0.030624	0.009263	0.017417	-0.023693
	-0.033163	0.012660	-0.007531	0.002738	0.017175	-0.019361
	-0.043353	-0.014232	-0.016585	-0.034941	0.013822	0.010143
	0.049029	-0.053705	0.037270	-0.137731	0.004060	0.022937
	0.010541					
x14	-0.021226	-0.036170	0.001152	-0.002525	-0.010757	-0.002465
	0.006626	-0.020429	0.043346	-0.010351	-0.028757	-0.022592
	-0.033653	0.017958	0.056680	-0.016499	-0.020955	0.057682
	-0.007368	-0.028106	-0.033627	-0.036606	0.007443	0.023438
	-0.028897	0.064957	-0.008224	0.062385	-0.007963	-0.003174
	-0.020496					
y14	0.032055	-0.007409	0.034864	-0.032992	0.012896	-0.022431
	0.009951	0.016577	-0.013798	0.003080	-0.027945	0.040716
	0.035714	-0.018408	0.007193	-0.000011	-0.008667	-0.014117
	-0.038427	-0.040224	-0.016211	-0.023893	0.018376	-0.009248
	0.020314	0.004214	-0.038050	-0.067254	0.005720	-0.004300
	0.004755					
z14	0.022312	0.035694	0.004263	-0.031146	0.025812	0.066547
	-0.005219	0.031095	0.028017	0.009244	0.019172	-0.016578
	-0.031370	0.012952	-0.008459	0.000176	0.019518	-0.017918
	-0.030676	-0.008326	-0.022258	-0.031641	0.016908	0.013324
	0.048832	-0.059484	0.041264	-0.146409	-0.002817	0.023913
	0.001152					
x15	-0.020137	-0.033094	0.001915	-0.002142	-0.008766	0.001966
	0.006059	-0.021615	0.048316	-0.010007	-0.030778	-0.029860
	-0.035974	0.021754	0.057866	-0.014147	-0.016679	0.056757
	-0.004428	-0.031402	-0.036862	-0.043508	0.007397	0.014442
	-0.019232	0.060679	-0.013256	0.048164	0.002260	0.004844
	-0.016465					
y15	0.033504	-0.006266	0.032924	-0.029661	0.009679	-0.020611
	0.006894	0.014873	-0.012110	-0.003576	-0.025148	0.042630
	0.046152	-0.017569	0.015320	-0.001732	-0.011777	-0.025976
	-0.031234	-0.039658	0.000422	-0.013780	0.013717	0.000759
	0.008220	0.002362	-0.020943	-0.041760	-0.013179	0.002966
	0.005159					
z15	0.024430	0.033683	0.004521	-0.028544	0.026415	0.067651
	-0.009182	0.031113	0.023131	0.005307	0.021632	-0.006918
	-0.025115	0.008974	-0.005865	-0.001284	0.021801	-0.013426
	-0.019916	-0.006101	-0.027919	-0.025316	0.014678	0.019144
	0.034205	-0.053000	0.045261	-0.140524	-0.013634	0.023271
	-0.004175					

x16	-0.019096	-0.030905	0.003364	-0.002762	-0.006114	0.005996
	0.004256	-0.021652	0.054137	-0.009521	-0.033010	-0.036378
	-0.039655	0.023864	0.057592	-0.010439	-0.010860	0.059817
	-0.005895	-0.032058	-0.048133	-0.054363	0.009092	0.000435
	-0.003468	0.056562	-0.022668	0.026849	0.011427	0.013844
	-0.014198					
y16	0.034602	-0.004143	0.031498	-0.026919	0.009501	-0.016348
	0.001778	0.013024	-0.009448	-0.006862	-0.019730	0.043195
	0.052075	-0.018902	0.019901	-0.001330	-0.012729	-0.037055
	-0.020925	-0.035147	0.012387	-0.001734	0.014003	0.009102
	-0.008310	-0.001889	-0.018315	-0.027214	-0.018972	-0.001545
	0.004154					
z16	0.026120	0.032623	0.004315	-0.024623	0.026672	0.065672
	-0.010244	0.027647	0.018211	-0.002205	0.020344	0.001091
	-0.016742	0.008881	-0.003546	-0.001923	0.021516	-0.011347
	-0.008956	-0.011440	-0.020966	-0.020491	0.007492	0.026721
	0.010320	-0.045825	0.059200	-0.117421	-0.026420	0.031129
	-0.004657					
x17	-0.017454	-0.029471	0.005229	-0.003907	-0.001231	0.011034
	0.001342	-0.019441	0.061557	-0.010705	-0.034078	-0.043590
	-0.045685	0.024926	0.054369	-0.007191	-0.007038	0.061252
	-0.010859	-0.026266	-0.060801	-0.064158	0.012342	-0.013565
	0.015982	0.049308	-0.032933	-0.003517	0.027454	0.021146
	-0.008603					
y17	0.035084	-0.002041	0.030375	-0.025753	0.008413	-0.012864
	-0.003045	0.008380	-0.003206	-0.008463	-0.017736	0.044769
	0.053145	-0.020406	0.021802	0.002071	-0.012625	-0.044310
	-0.009977	-0.039335	0.017161	-0.000672	0.012864	0.009366
	-0.017194	-0.012274	-0.013445	-0.014425	-0.020422	-0.001027
	0.004770					
z17	0.027977	0.033162	0.003929	-0.020269	0.027526	0.061136
	-0.010807	0.023774	0.011387	-0.009600	0.019551	0.005341
	-0.003924	0.013125	0.000437	-0.004783	0.023378	-0.011403
	-0.001473	-0.015072	-0.008310	-0.008559	0.004119	0.031900
	-0.023142	-0.034579	0.057136	-0.095260	-0.039545	0.031348
	-0.003856					
x18	-0.016538	-0.029234	0.005926	-0.000764	0.001094	0.018976
	0.002316	-0.018417	0.063874	-0.009595	-0.035653	-0.054157
	-0.044988	0.023135	0.063171	-0.002686	-0.006125	0.058969
	-0.004533	-0.021630	-0.061041	-0.066313	0.014036	-0.019470
	0.021084	0.051201	-0.035024	-0.016549	0.027539	0.026972
	-0.012390					

y18	0.035779	0.001350	0.029184	-0.026793	0.006720	-0.008514
	-0.006979	0.000285	0.003525	-0.012096	-0.013448	0.045543
	0.058319	-0.016058	0.023465	0.005039	-0.013725	-0.048848
	-0.004098	-0.049484	0.016670	0.004235	0.013093	0.009251
	-0.027808	-0.018046	-0.007625	0.002138	-0.024397	-0.001149
	0.001017					
z18	0.029932	0.037960	0.002185	-0.017632	0.026692	0.056442
	-0.011646	0.013170	0.007777	-0.013467	0.021623	0.009095
	0.009483	0.010905	0.006049	-0.011660	0.020189	-0.009937
	0.010501	-0.026295	0.003375	-0.005244	-0.000028	0.025316
	-0.046642	-0.029986	0.040716	-0.082260	-0.039539	0.029568
	0.011424					
x19	-0.015571	-0.030210	0.007443	0.003607	0.001917	0.026703
	0.003340	-0.017487	0.063529	-0.007908	-0.037746	-0.063744
	-0.041907	0.023884	0.073680	0.008025	-0.007072	0.054608
	0.005477	-0.018486	-0.056910	-0.063235	0.022751	-0.016409
	0.031025	0.050445	-0.024351	-0.014967	0.021877	0.034585
	-0.024945					
y19	0.035087	0.005139	0.028266	-0.028950	0.007150	-0.007298
	-0.008338	-0.008554	0.012075	-0.014606	-0.010989	0.044697
	0.060944	-0.012689	0.018678	0.006556	-0.015561	-0.050681
	-0.004396	-0.057519	0.005334	0.003442	0.008776	-0.001742
	-0.047464	-0.023797	-0.017869	0.004343	-0.022060	-0.012292
	-0.004255					
z19	0.030568	0.043442	0.001502	-0.016171	0.025193	0.046974
	-0.015816	-0.000364	0.006244	-0.018760	0.019969	0.015059
	0.021616	0.012281	0.006125	-0.016514	0.022469	-0.010013
	0.014073	-0.035462	0.012177	0.004789	-0.011130	0.016272
	-0.080775	-0.023663	0.012909	-0.058121	-0.023384	0.016492
	0.021784					
x20	-0.015164	-0.030665	0.008110	0.008033	0.005694	0.034792
	0.002466	-0.016936	0.065323	-0.008445	-0.034322	-0.070191
	-0.042727	0.020783	0.077791	0.018866	-0.010073	0.048292
	0.014684	-0.018183	-0.061981	-0.061151	0.021800	-0.014944
	0.033630	0.040111	-0.014874	-0.020675	0.022914	0.026453
	-0.048260					
y20	0.033071	0.006142	0.028613	-0.026837	0.006966	-0.004570
	-0.004681	-0.014859	0.019578	-0.021042	-0.016953	0.038997
	0.060806	-0.009294	0.013982	0.009312	-0.014512	-0.052741
	-0.008945	-0.050554	0.000769	-0.001263	0.007655	-0.002583
	-0.064903	-0.013004	-0.007998	0.009833	-0.027848	-0.015161
	-0.008065					

z20	0.028507	0.048551	0.002317	-0.011212	0.022347	0.041229
	-0.017795	-0.008960	0.006384	-0.029681	0.015786	0.011809
	0.029478	0.012469	-0.003462	-0.017889	0.024400	-0.012864
	0.010433	-0.035931	0.008385	0.004625	-0.028277	0.018648
	-0.108531	-0.008741	0.013809	-0.030918	-0.015945	-0.003451
	0.020104					
x21	-0.015095	-0.031886	0.010295	0.013519	0.009851	0.040631
	0.002652	-0.019887	0.068116	-0.007376	-0.032360	-0.073942
	-0.039280	0.023775	0.077332	0.020647	-0.010592	0.039253
	0.017258	-0.021282	-0.063918	-0.063536	0.015485	-0.008378
	0.030113	0.035073	-0.011749	-0.015320	0.012925	0.047067
	-0.039207					
y21	0.032931	0.007055	0.027473	-0.024080	0.008118	0.001234
	-0.001302	-0.017759	0.026916	-0.028109	-0.021079	0.032801
	0.058387	-0.010067	0.012894	0.013125	-0.010384	-0.054634
	-0.009525	-0.040574	-0.004686	-0.006353	0.008452	-0.001605
	-0.070025	-0.011089	0.008014	0.006661	-0.029378	-0.015979
	-0.013676					
z21	0.026656	0.053739	0.002762	-0.008076	0.022350	0.035969
	-0.018294	-0.012703	0.008096	-0.040832	0.015558	0.009777
	0.035429	0.016887	-0.011133	-0.022176	0.026427	-0.018827
	0.008116	-0.035881	0.005239	-0.001040	-0.033914	0.029555
	-0.115944	-0.009134	0.022368	-0.010520	-0.024266	0.012503
	0.046737					
x22	-0.014115	-0.033213	0.012837	0.015686	0.014304	0.041878
	0.002045	-0.017775	0.068726	-0.011494	-0.035566	-0.078713
	-0.039733	0.021141	0.076014	0.026267	-0.002272	0.032984
	0.022321	-0.018068	-0.060637	-0.061962	0.009070	0.004500
	0.024926	0.036212	-0.007969	-0.020361	0.006505	0.068749
	-0.040122					
y22	0.034204	0.007597	0.026211	-0.021467	0.009691	0.007197
	0.002280	-0.021484	0.031942	-0.030389	-0.023536	0.027582
	0.056614	-0.009279	0.012325	0.015081	-0.006567	-0.051285
	-0.010743	-0.034681	-0.015544	-0.012234	0.008036	0.000299
	-0.070233	-0.006421	0.013580	-0.000610	-0.030574	-0.012333
	-0.025729					
z22	0.025871	0.058361	0.001544	-0.006942	0.024341	0.031545
	-0.017333	-0.013886	0.008411	-0.047530	0.016679	0.006757
	0.036739	0.018648	-0.018749	-0.024339	0.028007	-0.016031
	0.009271	-0.035157	-0.007372	-0.003931	-0.040256	0.042117
	-0.112277	0.000522	0.026542	-0.004973	-0.026784	0.030509
	0.045055					

x23	-0.013699	-0.033581	0.015396	0.017861	0.017792	0.043411
	0.009103	-0.016892	0.067300	-0.010171	-0.040265	-0.084495
	-0.038693	0.018404	0.074995	0.031558	0.005364	0.029743
	0.017409	-0.010122	-0.060068	-0.051176	0.014618	-0.005144
	0.023800	0.031505	0.002578	-0.027115	0.002675	0.047882
	-0.052955					
y23	0.036092	0.007725	0.025533	-0.019410	0.011463	0.014998
	0.007360	-0.023899	0.034095	-0.033085	-0.026395	0.019960
	0.055170	-0.010764	0.011787	0.020376	-0.002900	-0.044481
	-0.014108	-0.025833	-0.025830	-0.019691	0.010807	0.002410
	-0.072351	0.000458	0.022444	-0.009770	-0.032422	-0.022103
	-0.039594					
z23	0.024159	0.062267	-0.000762	-0.006914	0.025646	0.029765
	-0.011877	-0.015194	0.006311	-0.051349	0.017125	0.002627
	0.035204	0.019721	-0.025221	-0.019074	0.029526	-0.004698
	0.008842	-0.034940	-0.023298	-0.003973	-0.039622	0.054619
	-0.105980	0.006195	0.029428	0.000376	-0.029485	0.024564
	0.036734					
x24	-0.012846	-0.033121	0.015463	0.020619	0.018624	0.043605
	0.010771	-0.016546	0.062948	-0.008997	-0.038407	-0.089072
	-0.039482	0.015976	0.073532	0.033581	0.012721	0.025494
	0.014012	0.002028	-0.046498	-0.046494	0.026353	-0.007536
	0.016008	0.035957	0.019709	-0.032076	0.008868	0.037388
	-0.067017					
y24	0.038371	0.008076	0.023629	-0.018337	0.011210	0.022152
	0.011510	-0.028206	0.036625	-0.035815	-0.031074	0.012378
	0.050138	-0.014159	0.013346	0.018892	0.000270	-0.039660
	-0.013400	-0.013499	-0.021811	-0.026204	0.018385	0.001173
	-0.070009	0.010339	0.031701	-0.019029	-0.024288	-0.029152
	-0.048736					
z24	0.020979	0.065345	-0.004114	-0.009701	0.025518	0.028232
	-0.003870	-0.018936	0.004731	-0.055009	0.013353	-0.001654
	0.030212	0.020424	-0.028555	-0.023772	0.032908	0.005581
	0.011645	-0.027873	-0.016274	-0.005712	-0.033322	0.057393
	-0.094250	0.011930	0.031051	0.007952	-0.029082	0.024320
	0.040811					
x25	-0.012795	-0.033661	0.017784	0.024586	0.019673	0.039899
	0.009659	-0.014641	0.054082	-0.009349	-0.036691	-0.084716
	-0.037338	0.019608	0.069346	0.032747	0.019327	0.015194
	0.011076	0.006093	-0.038347	-0.038309	0.033851	-0.001823
	0.013288	0.027428	0.031453	-0.033700	0.009219	0.031467
	-0.066698					

y25	0.040643	0.006957	0.023620	-0.015920	0.011343	0.028722
	0.012984	-0.032029	0.034913	-0.037910	-0.033777	0.010909
	0.049100	-0.012173	0.013467	0.017597	0.001615	-0.035781
	-0.012647	-0.009059	-0.024607	-0.034653	0.020608	0.006914
	-0.061397	0.009219	0.036382	-0.029616	-0.020774	-0.030882
	-0.055241					
z25	0.016633	0.067303	-0.008949	-0.012232	0.025658	0.028504
	0.004299	-0.022583	0.000528	-0.057648	0.009516	-0.004666
	0.026623	0.020862	-0.033319	-0.025117	0.035191	0.018743
	0.016833	-0.021939	-0.014588	-0.008732	-0.030520	0.061390
	-0.076735	0.016856	0.031093	0.013360	-0.026747	0.017737
	0.038523					
x26	-0.011551	-0.033498	0.019175	0.027669	0.022679	0.031399
	0.008247	-0.011285	0.046588	-0.010478	-0.034942	-0.082345
	-0.033460	0.017289	0.066211	0.029725	0.030026	0.004162
	0.008164	0.010116	-0.036376	-0.031292	0.039876	0.007477
	0.010502	0.027873	0.024462	-0.042782	0.020579	0.028110
	-0.051568					
y26	0.044430	0.005726	0.022954	-0.014225	0.012826	0.031957
	0.013028	-0.035769	0.036022	-0.039247	-0.038626	0.007638
	0.049935	-0.016363	0.015480	0.016893	0.004237	-0.032824
	-0.008249	-0.001377	-0.035092	-0.041175	0.020015	0.012797
	-0.045433	0.017477	0.019907	-0.047279	-0.001797	-0.022482
	-0.052437					
z26	0.011191	0.068856	-0.015864	-0.014861	0.026402	0.026821
	0.011733	-0.026446	-0.000661	-0.056542	0.003822	-0.005688
	0.020234	0.018211	-0.034254	-0.028383	0.036027	0.031441
	0.028637	-0.013296	-0.016773	-0.010344	-0.027565	0.056882
	-0.054997	0.024009	0.019146	0.021591	-0.015828	0.022099
	0.037241					
x27	-0.010440	-0.034296	0.020780	0.030056	0.026101	0.021898
	0.012570	-0.007730	0.037769	-0.009327	-0.031318	-0.076861
	-0.030512	0.015507	0.061823	0.026012	0.041944	-0.002375
	0.006799	0.010725	-0.031838	-0.021833	0.042997	0.022463
	0.012414	0.025948	0.026530	-0.050096	0.028526	0.020319
	-0.025222					
y27	0.048527	0.002749	0.023540	-0.013579	0.013988	0.035293
	0.017356	-0.039952	0.037768	-0.038587	-0.042555	0.007118
	0.051042	-0.019495	0.019471	0.016968	0.003175	-0.026585
	-0.000217	0.004272	-0.037850	-0.043051	0.020394	0.019817
	-0.019405	0.016445	0.015143	-0.066138	0.011050	-0.020832
	-0.035178					

z27	0.004998	0.069174	-0.022505	-0.018516	0.026130	0.025186
	0.019851	-0.030927	-0.001917	-0.055640	-0.004209	-0.006891
	0.015071	0.016351	-0.034392	-0.029088	0.034277	0.044854
	0.041882	-0.003052	-0.011221	-0.010188	-0.021370	0.049761
	-0.021158	0.023380	0.007341	0.022537	-0.009158	0.019027
	0.038813					
x28	-0.010326	-0.034861	0.023224	0.031518	0.031278	0.009449
	0.016887	0.001659	0.027727	-0.006675	-0.025067	-0.072689
	-0.029668	0.014979	0.054872	0.020234	0.045770	-0.010534
	0.007756	0.012131	-0.029515	-0.002557	0.044769	0.034618
	0.012966	0.017285	0.039023	-0.050965	0.032053	0.007629
	-0.017184					
y28	0.051323	-0.000489	0.024597	-0.013293	0.016628	0.036731
	0.023071	-0.038525	0.040092	-0.033418	-0.040467	0.002549
	0.048581	-0.019102	0.029541	0.014600	-0.002028	-0.025031
	-0.000519	0.006766	-0.044216	-0.036503	0.013450	0.022247
	0.000069	0.003391	0.016633	-0.080613	0.007160	-0.028835
	-0.046339					
z28	-0.002615	0.070359	-0.030890	-0.020150	0.024898	0.021289
	0.030191	-0.037402	0.001527	-0.054727	-0.012209	-0.011188
	0.014309	0.016457	-0.028252	-0.030278	0.043796	0.048167
	0.036318	0.003515	-0.010508	-0.005236	-0.024586	0.041286
	0.005600	0.015734	-0.007487	0.029036	-0.009780	0.012078
	0.040602					
x29	-0.011621	-0.036904	0.025906	0.029847	0.037720	0.002433
	0.021475	0.015068	0.009550	-0.012898	-0.023411	-0.075631
	-0.036742	0.016150	0.044613	0.010148	0.040995	-0.007956
	0.011247	0.012117	-0.021367	0.010136	0.043877	0.040874
	-0.007100	-0.002418	0.042551	-0.033680	0.029421	0.004225
	-0.009216					
y29	0.048808	-0.003887	0.030748	-0.014674	0.023091	0.036788
	0.032168	-0.031418	0.034329	-0.033711	-0.037365	-0.005588
	0.035000	-0.023490	0.029945	0.012217	-0.016238	-0.013756
	-0.003793	0.006597	-0.026158	-0.045207	0.003607	0.015119
	-0.007230	-0.005239	0.015124	-0.080767	-0.002295	-0.028883
	-0.051822					
z29	-0.012417	0.073061	-0.033772	-0.020060	0.028617	0.009919
	0.038083	-0.045993	0.009209	-0.044105	-0.013481	-0.006716
	0.016831	0.008187	-0.027101	-0.029502	0.051920	0.036675
	0.024105	0.012757	0.005622	0.003227	-0.025004	0.031980
	0.051470	0.032327	-0.011425	0.012774	-0.007676	-0.008262
	0.034361					

x30	-0.009614	-0.038886	0.026762	0.018871	0.042142	0.008705
	0.021448	0.024391	-0.014336	-0.023403	-0.027473	-0.092580
	-0.050672	0.026561	0.041500	-0.002210	0.030877	-0.006193
	0.018899	0.012592	-0.019395	0.017820	0.055942	0.042945
	-0.024294	-0.030963	0.010089	-0.007612	0.016450	0.013107
	-0.015562					
y30	0.052034	-0.006209	0.037707	-0.022765	0.032705	0.050400
	0.038660	-0.023428	0.018734	-0.031347	-0.034585	-0.022762
	0.026627	-0.017789	0.030238	0.006445	-0.033216	-0.019358
	0.008581	0.015824	-0.025371	-0.059067	0.006627	0.007858
	0.002084	-0.006448	-0.006955	-0.066999	-0.002456	-0.014353
	-0.047820					
z30	-0.020541	0.072573	-0.034223	-0.022977	0.034395	0.003896
	0.044985	-0.053423	0.006252	-0.037379	-0.016461	-0.002864
	0.014911	-0.000570	-0.036274	-0.024500	0.043501	0.033725
	0.025182	0.019696	0.019723	-0.000527	-0.020970	0.012687
	0.084964	0.049893	-0.015822	0.006522	0.004271	-0.021774
	0.021038					
x31	-0.011577	-0.039240	0.028400	0.021047	0.046928	0.001053
	0.024632	0.030194	-0.021125	-0.024984	-0.025607	-0.089984
	-0.046740	0.027384	0.037384	-0.010297	0.035537	-0.012762
	0.011999	0.013116	-0.020445	0.027778	0.055274	0.047494
	-0.022747	-0.041952	0.009789	0.003102	0.013831	0.003434
	-0.001704					
y31	0.044334	-0.006815	0.042408	-0.015746	0.035485	0.038574
	0.046567	-0.020720	0.011294	-0.028596	-0.028411	-0.011102
	0.025117	-0.022185	0.023419	0.010169	-0.035719	-0.030469
	0.010082	0.015751	-0.027955	-0.053757	0.000686	0.016864
	0.015417	-0.016031	-0.010141	-0.052877	0.008549	-0.022385
	-0.040908					
z31	-0.024886	0.074350	-0.034005	-0.028059	0.033941	0.001496
	0.052966	-0.052969	-0.001021	-0.029872	-0.014757	-0.003364
	0.010593	0.000905	-0.041850	-0.023198	0.039358	0.019393
	0.034820	0.024514	0.025519	0.005364	-0.016187	0.006120
	0.105935	0.050906	-0.022860	0.015957	0.016852	-0.023367
	0.010345					
x32	-0.011113	-0.040428	0.029487	0.019408	0.051321	0.001132
	0.024156	0.033672	-0.029351	-0.028417	-0.026875	-0.093368
	-0.046893	0.026471	0.037693	-0.018350	0.032485	-0.012903
	0.010211	0.011585	-0.021513	0.034774	0.062577	0.046093
	-0.019402	-0.054391	-0.002863	0.011940	0.003109	0.003451
	0.003766					

y32	0.036365	-0.005828	0.049527	-0.009846	0.038862	0.024445
	0.055753	-0.016311	0.005768	-0.029523	-0.022236	0.003668
	0.026210	-0.031790	0.010049	0.004112	-0.027877	-0.042593
	0.006845	0.018713	-0.016133	-0.048427	0.002935	0.023503
	0.026912	0.000140	0.002210	-0.039587	0.021169	-0.018010
	-0.023570					
z32	-0.026497	0.076880	-0.033577	-0.038261	0.032299	0.006113
	0.058373	-0.049555	-0.010674	-0.027887	-0.012671	-0.012049
	0.002721	0.006639	-0.041697	-0.020717	0.031120	0.010880
	0.044697	0.024509	0.023858	0.009593	-0.013085	0.002353
	0.110694	0.042322	-0.031005	0.030962	0.025039	-0.017389
	-0.011081					
x33	-0.012931	-0.041347	0.031414	0.022435	0.055473	-0.004485
	0.023703	0.037172	-0.033515	-0.029006	-0.024280	-0.088483
	-0.042866	0.022276	0.039023	-0.025568	0.037010	-0.014906
	0.004894	0.010101	-0.013166	0.043961	0.068043	0.046875
	-0.010601	-0.059974	-0.003661	0.009091	-0.002214	-0.001434
	0.020097					
y33	0.030839	-0.004850	0.056322	-0.005174	0.045052	0.011032
	0.064274	-0.011694	-0.003663	-0.030543	-0.017966	0.008077
	0.022988	-0.042223	-0.005934	0.001065	-0.021923	-0.057776
	0.011809	0.017232	-0.019979	-0.039978	0.002563	0.033085
	0.030432	0.017476	0.011602	-0.016814	0.035015	-0.016619
	-0.010455					
z33	-0.028829	0.079742	-0.033148	-0.046232	0.031515	0.006210
	0.064657	-0.043567	-0.017733	-0.026213	-0.008835	-0.018575
	-0.004194	0.010403	-0.038642	-0.018964	0.025211	0.002362
	0.047894	0.020117	0.027019	0.011857	-0.009111	-0.007481
	0.106063	0.034595	-0.026638	0.040943	0.026540	-0.014799
	-0.024379					
x34	-0.016250	-0.041538	0.033048	0.025932	0.057410	-0.013132
	0.024748	0.040355	-0.036975	-0.028522	-0.019823	-0.079121
	-0.038218	0.016892	0.037757	-0.032278	0.049771	-0.020117
	0.001752	0.008253	-0.005641	0.052967	0.066865	0.052811
	-0.003491	-0.062117	0.002442	0.008706	-0.000134	-0.001043
	0.033097					
y34	0.026699	-0.004958	0.063686	-0.002318	0.053462	-0.002354
	0.074427	-0.004933	-0.016880	-0.031790	-0.015352	0.006602
	0.016058	-0.049463	-0.023788	-0.003914	-0.027308	-0.072735
	0.017023	0.017393	-0.023486	-0.025648	0.005435	0.034972
	0.031769	0.022084	0.010986	0.005557	0.048507	-0.033609
	0.007788					

z34	-0.029478	0.082189	-0.031434	-0.051948	0.033231	0.007735
	0.068723	-0.038810	-0.022818	-0.026108	-0.004541	-0.025490
	-0.005535	0.012577	-0.033092	-0.014381	0.019778	-0.005638
	0.043433	0.015231	0.027991	0.012007	-0.003687	-0.019025
	0.105794	0.031596	-0.024235	0.044006	0.027788	-0.007642
	-0.022203					
x35	-0.019563	-0.041212	0.033068	0.030167	0.058673	-0.021938
	0.023989	0.041319	-0.038226	-0.026879	-0.015791	-0.068019
	-0.033469	0.012235	0.038242	-0.043084	0.067849	-0.030164
	0.002477	0.008165	0.004266	0.067947	0.059142	0.061070
	-0.005148	-0.057038	-0.000203	0.013114	0.009030	-0.010210
	0.045047					
y35	0.021404	-0.005394	0.072420	-0.001212	0.063659	-0.015843
	0.086471	0.003680	-0.032988	-0.033953	-0.013409	0.003662
	0.006968	-0.059357	-0.042092	-0.006510	-0.036270	-0.089054
	0.019067	0.014319	-0.028813	-0.015995	0.013917	0.033865
	0.042177	0.017142	0.018083	0.029308	0.054706	-0.030666
	0.025181					
z35	-0.027172	0.084624	-0.028637	-0.056781	0.032527	0.011522
	0.069760	-0.033554	-0.024581	-0.030670	-0.001163	-0.037666
	-0.006516	0.017962	-0.029078	-0.011960	0.019014	-0.005120
	0.034605	0.009051	0.021922	0.012278	0.002226	-0.030084
	0.106379	0.030616	-0.020087	0.050941	0.024616	-0.014009
	-0.010688					
x36	-0.022902	-0.041929	0.033167	0.035139	0.061640	-0.027351
	0.021492	0.039591	-0.036597	-0.021752	-0.013667	-0.054669
	-0.026003	0.008147	0.039005	-0.049002	0.083056	-0.042902
	-0.004828	0.010568	0.016758	0.080945	0.060917	0.058912
	0.001802	-0.052450	0.002169	0.008941	0.019038	-0.023973
	0.052243					
y36	0.016715	-0.005872	0.082708	-0.000276	0.074244	-0.033102
	0.099256	0.015284	-0.051696	-0.039606	-0.009070	-0.001374
	-0.003394	-0.072730	-0.059061	-0.012669	-0.043799	-0.104124
	0.027050	0.009673	-0.033638	-0.003332	0.015499	0.041455
	0.047345	0.009575	0.027329	0.053402	0.060750	-0.027426
	0.049979					
z36	-0.023602	0.088050	-0.028455	-0.063452	0.029555	0.016916
	0.069702	-0.027034	-0.023801	-0.035716	0.000520	-0.053870
	-0.012308	0.026262	-0.020915	-0.010839	0.014408	-0.002532
	0.020026	0.000418	0.014107	0.013789	0.015471	-0.045372
	0.098444	0.022857	-0.017259	0.059619	0.012013	-0.021231
	-0.014267					

x37	-0.003850	-0.007036	0.067944	-0.027456	0.057019	0.027393
	0.028562	-0.017999	-0.012840	-0.002595	0.024775	0.052577
	-0.040030	-0.029312	0.011804	0.002481	-0.007346	0.060216
	-0.068697	-0.028000	0.014080	0.038480	0.044819	-0.004761
	0.011817	0.072126	-0.054066	0.027781	0.001952	-0.015036
	0.041817					
y37	-0.024483	-0.039964	-0.000599	0.009919	0.031561	0.021915
	-0.007978	-0.036016	0.035979	0.033824	0.011830	0.040800
	-0.042741	0.001612	0.051157	-0.005650	0.046443	-0.039655
	0.009843	0.031729	0.020961	-0.004491	-0.025151	0.017608
	-0.022854	-0.048799	-0.027750	-0.020592	-0.066536	-0.090576
	0.058087					
z37	-0.043242	-0.022451	-0.019669	0.016243	0.046872	-0.056422
	0.015052	-0.074205	-0.012451	-0.023749	-0.062257	0.013348
	0.007170	-0.021191	0.012026	-0.036454	-0.001100	0.023266
	-0.045110	-0.033195	-0.047062	0.069817	-0.003638	-0.012660
	-0.029035	-0.014787	-0.006356	-0.017525	-0.025513	-0.076492
	-0.024902					
x38	-0.003835	-0.012412	0.078632	-0.027530	0.054432	0.035552
	0.036469	-0.016185	-0.004005	-0.003328	0.038688	0.044517
	-0.026650	-0.030362	0.011348	-0.011180	-0.013503	0.051428
	-0.065236	-0.020874	0.006967	0.047297	0.037105	-0.012903
	0.024603	0.046469	-0.033123	0.030621	-0.004601	-0.007973
	0.014497					
y38	-0.036660	-0.048368	0.006322	0.003887	0.039125	0.015690
	-0.004512	-0.040704	0.017530	0.021140	-0.002125	0.033971
	-0.034459	-0.010239	0.025971	0.000701	0.028686	-0.030852
	0.027484	0.027923	0.032944	0.000835	-0.010259	0.025547
	-0.033091	-0.062826	-0.023089	-0.009712	-0.049647	-0.050085
	0.026925					
z38	-0.030619	-0.010770	-0.024464	0.010969	0.041342	-0.061533
	0.014307	-0.061945	-0.016974	-0.030003	-0.066505	-0.004280
	0.023911	-0.013943	0.009146	-0.037818	-0.009717	0.038118
	-0.033317	-0.018069	-0.032034	0.057880	0.008153	-0.003373
	-0.028511	-0.034502	0.002211	-0.007461	-0.017651	-0.042149
	-0.033419					
x39	-0.006285	-0.019160	0.086958	-0.024483	0.051360	0.041718
	0.039515	-0.016621	0.007038	-0.002357	0.048726	0.042907
	-0.014764	-0.034225	0.011858	-0.023789	-0.015916	0.049268
	-0.061073	-0.019041	0.013082	0.061380	0.037351	-0.009481
	0.035753	0.023108	-0.019328	0.026462	-0.000042	0.005830
	-0.012377					

y39	-0.038869	-0.054387	0.011986	-0.004476	0.042973	0.013417
	-0.001555	-0.041856	-0.010484	0.009249	-0.016352	0.027017
	-0.026599	-0.017356	-0.001247	0.005777	0.009450	-0.024267
	0.052524	0.031020	0.049818	-0.000251	0.000624	0.035985
	-0.039811	-0.062965	-0.027159	-0.001497	-0.032897	-0.007053
	-0.003173					
z39	-0.008335	0.015229	-0.028952	0.005532	0.034038	-0.055936
	0.012948	-0.043215	-0.023903	-0.023996	-0.053893	-0.006140
	0.032186	-0.008285	0.013836	-0.032184	-0.013231	0.041940
	-0.018622	-0.008377	-0.020304	0.061437	0.007833	0.005888
	-0.006642	-0.043161	-0.014616	-0.022057	-0.021826	-0.021482
	-0.034767					
x40	-0.006026	-0.023741	0.087952	-0.020426	0.046169	0.042742
	0.038317	-0.018848	0.013817	-0.004656	0.053458	0.038131
	0.001876	-0.031076	0.012135	-0.029094	-0.011043	0.047629
	-0.063445	-0.014294	0.014227	0.055501	0.031312	-0.000031
	0.030216	0.004897	-0.015475	0.025638	0.013798	0.013399
	-0.029094					
y40	-0.037993	-0.058847	0.015272	-0.005907	0.047806	0.011464
	-0.003529	-0.046032	-0.027660	0.002009	-0.025935	0.026080
	-0.024288	-0.022577	-0.015861	0.015959	-0.005104	-0.018204
	0.062430	0.032699	0.053316	-0.011951	-0.000230	0.036990
	-0.040438	-0.041699	-0.022543	0.001600	-0.028294	0.014961
	-0.015367					
z40	0.008373	0.035033	-0.030830	0.006064	0.031681	-0.047334
	0.005452	-0.030409	-0.021869	-0.015972	-0.040341	-0.002174
	0.036008	-0.005689	0.022124	-0.021737	-0.013879	0.039862
	-0.017882	-0.000573	-0.021022	0.053507	0.009238	0.007060
	0.017117	-0.035735	-0.015506	-0.034677	-0.032634	-0.012767
	-0.032273					
x41	-0.004286	-0.025014	0.085016	-0.017145	0.036594	0.038285
	0.034921	-0.017586	0.015629	-0.008998	0.054005	0.031631
	0.014959	-0.024277	0.011610	-0.023210	-0.003113	0.049202
	-0.064016	-0.010263	0.012174	0.040199	0.026692	0.015350
	0.021176	-0.014835	-0.011828	0.023236	0.020634	0.016532
	-0.038294					
y41	-0.038463	-0.060512	0.020563	-0.007397	0.052941	0.011755
	-0.003432	-0.047311	-0.034354	0.000030	-0.031755	0.029220
	-0.026100	-0.026991	-0.028961	0.023852	-0.015697	-0.014876
	0.064989	0.031869	0.061669	-0.017665	-0.014713	0.032396
	-0.054835	-0.012100	-0.026160	-0.002036	-0.019914	0.016176
	-0.012242					

z41	0.019594	0.047491	-0.030258	0.005918	0.030531	-0.039644
	0.001285	-0.020116	-0.018912	-0.008233	-0.029725	0.001592
	0.038415	-0.005544	0.025742	-0.014590	-0.015615	0.036501
	-0.020302	0.007519	-0.018008	0.043994	0.006335	0.003474
	0.025469	-0.024942	-0.016273	-0.040650	-0.037205	-0.011450
	-0.025146					
x42	-0.002412	-0.024420	0.085095	-0.012810	0.033400	0.038162
	0.034062	-0.015642	0.015097	-0.009623	0.049168	0.026060
	0.027597	-0.021932	0.010725	-0.018784	0.007259	0.047722
	-0.063972	-0.014164	0.003669	0.036450	0.019767	0.014290
	0.015565	-0.030052	-0.000993	0.017545	0.013018	0.009561
	-0.036471					
y42	-0.039471	-0.060957	0.024041	-0.008852	0.052643	0.005636
	-0.003088	-0.038644	-0.032344	-0.006588	-0.036006	0.024751
	-0.021933	-0.029285	-0.035877	0.032771	-0.014691	-0.006600
	0.060303	0.027829	0.054172	-0.015814	-0.014735	0.041228
	-0.069014	0.004367	-0.027435	0.007452	-0.004107	0.017012
	-0.009183					
z42	0.028539	0.059143	-0.029405	0.004386	0.028158	-0.030820
	-0.001797	-0.011248	-0.015684	-0.004119	-0.019905	0.004433
	0.038386	-0.005567	0.028262	-0.009406	-0.013252	0.032104
	-0.023199	0.010742	-0.015487	0.042195	0.004899	0.002315
	0.033903	-0.019772	-0.015455	-0.046653	-0.043700	-0.013211
	-0.021948					
x43	0.000819	-0.021593	0.085938	-0.008213	0.035988	0.040338
	0.037548	-0.015112	0.012158	-0.007555	0.041726	0.022809
	0.041132	-0.020549	0.009974	-0.011771	0.011868	0.038826
	-0.067979	-0.007754	-0.004964	0.028225	0.009190	-0.001921
	0.005246	-0.036974	0.010564	0.023573	0.008836	-0.002596
	-0.024228					
y43	-0.030515	-0.061183	0.020071	-0.003850	0.042607	-0.009451
	-0.007175	-0.025593	-0.025391	-0.012599	-0.033206	0.024767
	-0.022170	-0.021985	-0.031731	0.026251	-0.013689	-0.005484
	0.046514	0.021607	0.039599	-0.021852	0.008739	0.044980
	-0.054725	0.023843	-0.028266	0.014194	-0.003515	0.036317
	-0.012174					
z43	0.037474	0.069751	-0.026904	0.002334	0.027179	-0.017457
	-0.002998	-0.003007	-0.015661	0.001131	-0.008724	0.004228
	0.043828	-0.007421	0.029861	-0.003191	-0.008118	0.023998
	-0.030520	0.026061	-0.014396	0.033296	0.005670	-0.002302
	0.035833	-0.011535	-0.007584	-0.043650	-0.053293	-0.013879
	-0.016468					

x44	0.002139	-0.016770	0.085131	-0.004772	0.035443	0.038443
	0.041774	-0.015085	0.006185	-0.007149	0.032880	0.022085
	0.046852	-0.022378	0.007798	0.000580	0.016318	0.025559
	-0.074336	-0.000324	-0.005349	0.019992	0.004454	-0.011404
	-0.003450	-0.036686	0.017109	0.031343	0.006287	-0.002298
	-0.014700					
y44	-0.005728	-0.051574	0.011027	0.009285	0.032663	-0.019154
	-0.012515	-0.013746	-0.010687	-0.016341	-0.027501	0.029894
	-0.024992	0.003327	-0.019882	0.005915	-0.024544	-0.011832
	0.033281	0.013484	0.017427	-0.007009	0.032471	0.039051
	-0.036155	0.015154	-0.027055	0.004223	-0.012236	0.035175
	-0.017081					
z44	0.039007	0.073346	-0.022639	-0.004772	0.021500	-0.007369
	-0.005193	0.009307	-0.023119	0.003799	0.008246	0.002352
	0.053454	-0.010110	0.029593	0.012617	0.007373	0.011048
	-0.039854	0.037112	-0.007093	0.025471	0.017796	0.010564
	0.035351	-0.004259	-0.007491	-0.040613	-0.043106	-0.020557
	-0.016126					
x45	0.002053	-0.018126	0.083885	0.002096	0.033425	0.035316
	0.044424	-0.017466	0.004922	-0.008849	0.025322	0.023215
	0.051136	-0.024188	0.005860	0.008180	0.016005	0.021945
	-0.077062	0.003834	0.001806	0.015361	0.002742	-0.015133
	0.008754	-0.025290	0.023831	0.043914	0.005362	-0.003639
	-0.007875					
y45	0.012787	-0.034518	0.009612	0.008629	0.024060	-0.013519
	-0.010927	-0.000746	-0.008320	-0.019672	-0.010436	0.035416
	-0.008892	0.012537	-0.005145	0.004557	-0.030148	-0.006408
	0.006790	0.022652	-0.015515	0.006226	0.051858	0.023919
	-0.033040	0.012408	-0.021172	-0.008321	-0.029825	0.000287
	-0.046340					
z45	0.032088	0.065358	-0.019930	-0.003988	0.021605	-0.012729
	-0.011431	0.012578	-0.021686	0.004164	0.018879	0.007342
	0.048643	-0.009828	0.028065	0.022588	0.009948	0.008479
	-0.028236	0.028417	0.006552	0.024368	0.015277	0.010349
	0.041277	0.016693	-0.016956	-0.026160	-0.012055	-0.017160
	0.015597					
x46	0.006294	-0.015704	0.082653	0.004577	0.032493	0.034032
	0.036826	-0.015095	0.006828	-0.014931	0.027033	0.022847
	0.041280	-0.021801	-0.002489	0.015962	0.021737	0.033565
	-0.067439	-0.006749	0.007361	0.006497	-0.006658	-0.019602
	0.008742	-0.007167	0.032880	0.022670	0.008880	-0.000046
	0.020724					

y46	0.020939	-0.025046	0.006679	0.006096	0.020764	-0.009016
	-0.009378	0.006200	-0.008126	-0.015125	-0.006801	0.036992
	0.000126	0.018518	-0.001257	0.003369	-0.032101	-0.005734
	0.003212	0.032968	-0.007227	0.009569	0.058328	0.024701
	-0.024019	0.019722	-0.022295	-0.004240	-0.022142	-0.004970
	-0.046411					
z46	0.028639	0.062295	-0.018903	-0.003170	0.023018	-0.017197
	-0.011337	0.015112	-0.016968	0.005170	0.020810	0.009530
	0.051190	-0.017443	0.032521	0.026200	0.006331	0.010481
	-0.030661	0.029228	0.005642	0.017107	0.017858	0.012120
	0.030470	0.025497	-0.020469	-0.022194	-0.006260	-0.012748
	0.015971					
x47	0.006636	-0.018785	0.084260	0.007936	0.033673	0.032938
	0.029944	-0.011586	0.012956	-0.016640	0.035101	0.018105
	0.041467	-0.024921	-0.012509	0.020778	0.031017	0.045243
	-0.063457	-0.015892	0.002273	-0.002023	-0.013194	-0.009777
	0.000922	-0.005762	0.013572	-0.000375	0.014486	0.012059
	0.009943					
y47	0.027651	-0.015936	0.002651	-0.001020	0.019770	-0.002747
	-0.012103	0.009751	-0.019616	-0.014309	-0.011228	0.034813
	-0.000571	0.024300	-0.008469	0.003433	-0.019560	-0.001146
	-0.002243	0.034453	0.005236	0.007269	0.034383	0.026353
	-0.010237	0.010988	-0.015036	-0.002702	-0.002633	0.019066
	-0.032871					
z47	0.034262	0.066698	-0.019426	0.001993	0.028576	-0.020091
	-0.014980	0.017027	-0.008552	0.009732	0.014666	0.001869
	0.038066	-0.012254	0.022589	0.024808	0.012763	0.016161
	-0.031944	0.014440	0.010569	0.013943	0.000506	0.022886
	0.033168	0.009753	-0.005553	-0.023565	0.000743	0.002246
	0.010527					
x48	0.006454	-0.021817	0.085220	0.015205	0.030855	0.030748
	0.034811	-0.012308	0.008691	-0.016508	0.039052	0.014539
	0.053338	-0.024348	-0.007733	0.021625	0.036455	0.041069
	-0.055174	-0.010876	0.009771	0.000755	-0.020047	-0.002394
	-0.015024	0.005404	-0.003192	0.009184	-0.004225	0.012405
	-0.005308					
y48	0.034758	-0.006640	-0.002095	-0.005369	0.019218	0.001611
	-0.012972	0.012542	-0.026096	-0.012662	-0.012755	0.035222
	0.001256	0.029337	-0.005003	0.007274	-0.011793	0.003341
	-0.007007	0.035906	0.011469	0.012906	0.016618	0.020849
	-0.010935	0.011328	-0.010823	0.007435	0.005030	0.026540
	-0.021744					

z48	0.034897	0.065162	-0.017382	0.007312	0.034114	-0.023938
	-0.018209	0.017999	0.003093	0.014227	0.015483	-0.003427
	0.026263	-0.013259	0.023661	0.024965	0.017299	0.015824
	-0.026573	0.005018	-0.003498	0.014647	-0.011160	0.023993
	0.025891	0.004798	-0.004125	-0.018238	0.006015	0.004005
	0.016582					
x49	0.007455	-0.024123	0.085269	0.019237	0.029440	0.027709
	0.036084	-0.012249	0.009394	-0.015860	0.041500	0.011302
	0.059192	-0.024949	-0.009670	0.023813	0.039761	0.039006
	-0.050878	-0.006837	0.010179	-0.005275	-0.023232	-0.003687
	-0.017274	0.006957	-0.021319	0.003480	-0.009203	0.011715
	-0.008233					
y49	0.039934	-0.000771	-0.003619	-0.006631	0.020862	0.002727
	-0.013054	0.016192	-0.024143	-0.013719	-0.012758	0.035571
	0.001135	0.031158	-0.009809	0.007844	-0.008585	0.011720
	-0.009729	0.033844	0.006218	0.017390	0.007229	0.013738
	-0.009757	0.010452	-0.001094	0.000678	0.006669	0.022302
	-0.007944					
z49	0.034161	0.062252	-0.013736	0.011184	0.037936	-0.025185
	-0.019339	0.019324	0.012171	0.016283	0.018290	-0.008076
	0.018411	-0.014151	0.022247	0.024434	0.020393	0.011357
	-0.018209	-0.002088	-0.014953	0.016135	-0.019208	0.024888
	0.019468	0.005464	0.004934	-0.016960	0.003273	0.000977
	0.026056					
x50	0.009026	-0.026075	0.083978	0.022504	0.027972	0.024924
	0.036254	-0.013511	0.010224	-0.015546	0.044800	0.008176
	0.063268	-0.026512	-0.011689	0.025523	0.039784	0.036593
	-0.043409	-0.006699	0.009182	-0.011167	-0.025646	-0.003255
	-0.016271	0.008099	-0.030362	-0.001012	-0.011785	0.013778
	-0.009316					
y50	0.043401	0.003355	-0.004833	-0.005695	0.025877	0.000906
	-0.013326	0.019709	-0.025265	-0.016666	-0.008424	0.034435
	0.003309	0.036840	-0.009215	0.008549	-0.001449	0.015743
	-0.008214	0.035074	-0.000872	0.015970	0.010622	0.012897
	-0.012380	0.012785	0.008499	-0.001665	0.007723	0.015279
	0.003102					
z50	0.033077	0.058977	-0.009894	0.014843	0.040702	-0.026157
	-0.019393	0.020667	0.018856	0.018352	0.020684	-0.012263
	0.012262	-0.013007	0.022036	0.023883	0.023705	0.004136
	-0.011540	-0.005577	-0.024049	0.018037	-0.023167	0.027992
	0.010321	0.008895	0.012688	-0.013637	-0.001514	-0.003688
	0.033456					

x51	0.011255	-0.027805	0.082342	0.024698	0.026224	0.021655
	0.035717	-0.015238	0.011002	-0.014761	0.046784	0.005792
	0.066713	-0.028029	-0.013260	0.027277	0.038623	0.033626
	-0.035198	-0.007211	0.007468	-0.015181	-0.026113	-0.004166
	-0.013530	0.011631	-0.032750	-0.002495	-0.014305	0.015735
	-0.012528					
y51	0.045761	0.007400	-0.005125	-0.003675	0.028994	0.001304
	-0.013599	0.022105	-0.024080	-0.014246	-0.004580	0.031810
	0.003509	0.036001	-0.011277	0.009434	0.001312	0.011552
	-0.010115	0.032848	-0.002508	0.016436	0.003748	0.018451
	-0.022040	0.011131	0.013877	-0.001684	0.005319	0.025198
	0.014541					
z51	0.033016	0.056819	-0.007536	0.016946	0.041184	-0.027066
	-0.017647	0.018899	0.024418	0.017982	0.021405	-0.015221
	0.008015	-0.013226	0.020991	0.021066	0.024405	-0.000862
	-0.007971	-0.002874	-0.023793	0.018272	-0.022337	0.030236
	0.005321	0.011064	0.017506	-0.010202	-0.003226	-0.007626
	0.030439					
x52	0.014469	-0.029006	0.080070	0.026129	0.024558	0.018210
	0.035085	-0.018932	0.012503	-0.013389	0.045665	0.003952
	0.068759	-0.029814	-0.015302	0.028156	0.035113	0.028236
	-0.027739	-0.006981	0.008325	-0.018092	-0.024205	-0.008201
	-0.006816	0.013527	-0.027365	-0.001226	-0.015238	0.018429
	-0.014227					
y52	0.049151	0.012371	-0.003782	0.000233	0.031550	-0.000020
	-0.012367	0.025079	-0.021083	-0.012571	0.000013	0.028426
	0.005491	0.037414	-0.014722	0.004406	0.007718	0.009881
	-0.007823	0.031696	0.002046	0.018148	0.005007	0.025372
	-0.027152	0.015700	0.017442	-0.009677	0.004851	0.032033
	0.023621					
z52	0.033771	0.054836	-0.007330	0.017582	0.038100	-0.027371
	-0.013988	0.013104	0.029872	0.015884	0.021233	-0.016605
	0.004611	-0.016151	0.017864	0.016089	0.019199	-0.003916
	-0.010265	0.007051	-0.014871	0.018949	-0.018470	0.033220
	0.004094	0.014381	0.021188	-0.004535	-0.000607	-0.010837
	0.015393					
x53	0.018914	-0.029386	0.076302	0.028283	0.024140	0.014398
	0.035401	-0.024063	0.014949	-0.010041	0.041579	0.001818
	0.071488	-0.030995	-0.016820	0.027547	0.028072	0.020291
	-0.018858	-0.007061	0.009859	-0.022734	-0.021946	-0.016490
	0.004258	0.014979	-0.014912	0.001306	-0.014357	0.021346
	-0.010870					

y53	0.053306	0.014264	-0.001613	0.003343	0.033477	-0.001524
	-0.008793	0.024671	-0.012514	-0.009154	0.010031	0.024359
	0.003807	0.041005	-0.020313	0.000401	0.010404	0.002217
	-0.008984	0.036921	0.011565	0.029898	0.003578	0.034650
	-0.036109	0.033027	0.026096	-0.012352	0.008013	0.028098
	0.033292					
z53	0.033231	0.052408	-0.007081	0.018487	0.032846	-0.027037
	-0.007588	0.004141	0.033235	0.011290	0.017275	-0.017541
	0.002377	-0.023039	0.011927	0.004160	0.013610	-0.007025
	-0.012683	0.019998	-0.001014	0.017234	-0.005185	0.040241
	0.002893	0.009213	0.022230	-0.000041	0.002309	-0.010820
	-0.013090					
x54	0.023685	-0.029125	0.070376	0.030758	0.025252	0.010515
	0.036436	-0.026957	0.017694	-0.005784	0.036514	0.001225
	0.074443	-0.030028	-0.017844	0.026013	0.018417	0.012748
	-0.005260	-0.011185	0.009781	-0.024799	-0.021827	-0.027635
	0.015059	0.017018	0.004293	0.006051	-0.012159	0.024271
	0.003452					
y54	0.058279	0.016219	0.000783	0.006951	0.033266	-0.004736
	-0.001118	0.012606	-0.001492	-0.005268	0.016287	0.017199
	0.002222	0.030845	-0.031241	-0.011263	0.004815	-0.005328
	-0.014708	0.052245	0.032512	0.031854	0.008615	0.049123
	-0.044301	0.045618	0.035287	-0.015388	0.003671	0.023777
	0.004422					
z54	0.026695	0.042726	-0.006615	0.016143	0.026169	-0.022729
	-0.001400	0.000304	0.035004	0.007099	0.019072	-0.015806
	-0.002914	-0.015899	0.009622	-0.004656	0.013309	-0.017497
	-0.008485	0.029420	0.010127	0.029192	0.005807	0.046824
	0.003433	0.009687	0.015412	0.008918	0.014393	-0.020285
	-0.016587					
x55	0.025106	-0.025976	0.061590	0.027703	0.019358	0.002600
	0.032306	-0.026223	0.020745	-0.007053	0.033010	0.002319
	0.067130	-0.025840	-0.018052	0.034599	0.011026	0.013481
	0.016065	-0.027635	0.011123	-0.017873	-0.019973	-0.017483
	0.024986	0.008219	0.016186	-0.000237	-0.008451	0.032346
	0.010320					
y55	0.061221	0.013942	0.001969	0.010513	0.033315	-0.006042
	0.005791	0.000413	0.007726	0.000472	0.022138	0.012977
	-0.000942	0.019886	-0.041041	-0.026804	-0.006055	-0.016077
	-0.019124	0.070893	0.055499	0.031636	0.014210	0.061926
	-0.048875	0.061581	0.041237	-0.012984	0.001672	0.016582
	-0.029406					

z55	0.019224	0.034635	-0.005120	0.010806	0.021346	-0.016408
	0.006091	0.000884	0.036981	0.001085	0.009567	-0.016172
	0.009114	-0.007471	0.014479	-0.003146	0.018292	-0.020261
	-0.000600	0.029644	-0.001109	0.029481	0.001823	0.030922
	0.009651	-0.003971	0.005680	0.015615	0.013021	-0.020148
	0.009799					
x56	-0.021056	0.061064	0.006022	-0.006758	-0.011487	-0.014234
	0.018295	0.057368	-0.005449	0.047636	-0.017403	0.017551
	-0.006766	0.044709	0.011848	-0.022721	0.028548	0.009448
	-0.018699	0.015007	-0.019871	-0.002264	0.031288	-0.045053
	-0.016882	-0.029952	0.027937	-0.047597	-0.028169	-0.067387
	-0.049306					
y56	0.000721	-0.017191	0.019469	0.045401	-0.031871	0.006703
	-0.034838	-0.003991	-0.018383	0.015149	-0.023253	-0.018928
	-0.016151	0.026495	0.021337	0.017903	-0.001690	-0.006024
	0.012289	-0.012274	-0.009167	0.002496	-0.016037	-0.019137
	0.053155	0.001659	0.011984	-0.009175	0.031039	0.006089
	-0.038960					
z56	-0.064457	0.012069	0.026792	-0.014638	0.025641	0.004862
	-0.009219	-0.028619	-0.000046	0.005207	0.007826	-0.026802
	-0.005997	-0.004853	0.022635	-0.007385	-0.015974	-0.013973
	0.048658	0.032527	-0.005900	-0.003294	-0.023147	-0.011982
	-0.011807	-0.067992	0.002644	0.003333	0.001040	0.036254
	-0.020035					
x57	-0.015270	0.066507	0.002306	-0.002331	-0.018301	-0.008910
	0.017506	0.047501	-0.003807	0.038117	-0.018607	0.019274
	-0.004298	0.055119	0.009171	-0.042672	0.046630	0.012982
	-0.016900	0.021584	-0.019965	-0.012107	0.024497	-0.043686
	-0.036061	-0.029908	0.014905	-0.040593	-0.021291	-0.065148
	-0.026677					
y57	0.000757	-0.014969	0.024698	0.043950	-0.035414	0.001440
	-0.030958	-0.001993	-0.024840	0.010827	-0.037050	-0.028924
	-0.007132	0.018801	0.018169	0.008111	0.014154	-0.012115
	0.017829	-0.002571	0.015644	0.008859	-0.010188	-0.019957
	0.052320	0.002570	0.014082	-0.022139	0.029479	-0.009382
	-0.009576					
z57	-0.063332	0.016860	0.027527	-0.013314	0.019817	0.002262
	-0.010151	-0.022263	-0.000259	0.012881	0.008511	-0.035198
	-0.005393	-0.008536	0.030989	-0.012185	0.003965	-0.022399
	0.024840	0.025633	0.016210	0.019525	-0.010006	-0.032457
	-0.008108	-0.085687	0.004801	-0.003316	-0.012732	0.034338
	-0.036987					

x58	-0.010172	0.067128	-0.004018	0.004543	-0.022804	-0.004090
	0.015750	0.035865	-0.000241	0.025878	-0.016044	0.018631
	0.002788	0.068893	0.005575	-0.063583	0.058403	0.018997
	-0.011003	0.025783	-0.027894	-0.021704	0.013026	-0.028165
	-0.067248	-0.029121	-0.006282	-0.034100	-0.001954	-0.055051
	-0.013087					
y58	0.001004	-0.013489	0.026692	0.042796	-0.034739	-0.001397
	-0.032941	-0.001333	-0.034454	0.007265	-0.046770	-0.036975
	-0.005707	0.008527	0.016263	0.000124	0.021446	-0.013497
	0.023843	0.000790	0.018566	0.014107	-0.004561	-0.014522
	0.048220	0.003169	0.012998	-0.029764	0.031787	-0.014506
	-0.009109					
z58	-0.057928	0.028736	0.026619	-0.010795	0.015410	0.003357
	-0.008374	-0.018710	-0.006058	0.018908	0.018479	-0.036546
	-0.010140	-0.018198	0.028484	-0.010395	0.016744	-0.038036
	0.009773	0.020519	0.026343	0.026199	-0.008563	-0.055802
	0.000886	-0.091101	0.019117	-0.009405	-0.009928	0.025580
	-0.037015					
x59	-0.007221	0.066301	-0.008865	0.009473	-0.027150	-0.002694
	0.015415	0.025533	-0.000770	0.013057	-0.012396	0.018338
	0.004680	0.080506	0.001761	-0.079057	0.067265	0.019650
	-0.007344	0.026239	-0.028717	-0.018805	0.004269	-0.029160
	-0.085728	-0.029565	-0.019585	-0.031731	0.005062	-0.047105
	0.008478					
y59	0.001950	-0.012483	0.024226	0.043487	-0.029909	-0.000808
	-0.034951	-0.002734	-0.039196	0.003318	-0.044382	-0.039325
	-0.008052	0.000966	0.008274	0.002925	0.010759	-0.005263
	0.030327	-0.005586	-0.010709	0.014650	-0.011008	0.002343
	0.042347	0.003371	0.019910	-0.026357	0.040422	-0.015077
	-0.022208					
z59	-0.052164	0.036541	0.026997	-0.011260	0.015471	0.005045
	-0.007224	-0.010743	-0.007671	0.026281	0.023266	-0.035637
	-0.009055	-0.024680	0.024945	-0.004711	0.016356	-0.043371
	0.003715	0.022584	0.032347	0.011591	-0.009093	-0.049523
	0.001443	-0.087133	0.022650	-0.010327	0.001562	0.019717
	-0.051548					
x60	-0.005377	0.065957	-0.012597	0.013328	-0.027062	-0.001398
	0.015572	0.017397	-0.000195	0.006561	-0.005837	0.013345
	0.004446	0.083908	-0.004009	-0.085508	0.071366	0.014829
	-0.001307	0.026797	-0.042156	-0.013979	-0.008364	-0.028859
	-0.100529	-0.035178	-0.023205	-0.025258	0.016560	-0.039179
	0.005699					

y60	0.001720	-0.011624	0.024544	0.040501	-0.030130	-0.004402
	-0.032350	-0.001172	-0.037920	-0.002303	-0.042949	-0.038112
	-0.005811	0.001897	-0.000371	0.001299	0.008774	0.002951
	0.035557	-0.002840	-0.021314	0.016325	-0.013405	0.011880
	0.038352	0.006124	0.027032	-0.025796	0.043363	-0.021902
	-0.017452					
z60	-0.047473	0.040973	0.026643	-0.011715	0.012937	0.006791
	-0.006247	-0.004174	-0.010491	0.028034	0.025205	-0.031822
	-0.006509	-0.024756	0.021586	-0.002821	0.015131	-0.042513
	-0.001304	0.022119	0.040297	-0.004299	-0.007851	-0.041648
	0.000572	-0.080133	0.021869	-0.009884	0.003489	0.016560
	-0.049389					
x61	-0.004406	0.064369	-0.012613	0.017466	-0.023309	-0.000650
	0.018155	0.011778	0.000993	0.007215	-0.003584	0.001933
	0.008497	0.062124	-0.006873	-0.092057	0.070777	-0.004817
	-0.004364	0.031494	-0.039340	-0.022212	-0.019167	-0.042229
	-0.089964	-0.039473	-0.000625	-0.007183	0.025117	-0.036364
	0.014130					
y61	0.002073	-0.010944	0.024475	0.038563	-0.029646	-0.006532
	-0.030512	-0.000309	-0.034536	-0.006503	-0.038860	-0.036077
	-0.003679	0.005284	-0.008417	0.002110	0.007010	0.008081
	0.038329	-0.001296	-0.034851	0.016645	-0.019893	0.020993
	0.033681	0.011951	0.032095	-0.024152	0.045629	-0.027781
	-0.016314					
z61	-0.042258	0.044616	0.024400	-0.010806	0.011526	0.012230
	-0.005147	-0.000674	-0.012798	0.026701	0.025496	-0.025614
	-0.000923	-0.021058	0.018247	-0.006322	0.014737	-0.039809
	-0.006462	0.020219	0.040315	-0.020586	-0.010027	-0.040108
	-0.003265	-0.069076	0.023582	-0.003768	0.005243	0.018572
	-0.038375					
x62	-0.009223	0.057185	-0.010338	0.017929	-0.018335	-0.001607
	0.020708	0.005715	-0.007013	0.005169	0.007776	-0.006197
	0.003200	0.029063	-0.019769	-0.077688	0.069822	-0.015731
	-0.003578	0.033826	-0.031840	-0.030953	-0.046596	-0.043715
	-0.076015	-0.021660	0.036587	0.009468	0.036571	-0.019628
	0.017421					
y62	0.003272	-0.009762	0.024022	0.035669	-0.028006	-0.007656
	-0.029207	-0.000481	-0.029679	-0.009209	-0.037835	-0.035857
	-0.002268	0.010582	-0.013488	0.001340	0.009964	0.004525
	0.034388	0.001738	-0.042641	0.015158	-0.027341	0.021558
	0.023570	0.014870	0.030295	-0.019204	0.045153	-0.032120
	-0.013348					

z62	-0.038850	0.047762	0.020564	-0.009947	0.008128	0.016796
	-0.005323	0.002453	-0.017289	0.022432	0.025452	-0.014459
	0.001828	-0.011744	0.013370	-0.005151	0.014190	-0.030903
	-0.007115	0.013109	0.041053	-0.034731	-0.011285	-0.034786
	-0.006764	-0.053021	0.019890	-0.001624	0.006322	0.012837
	-0.024015					
x63	-0.018148	0.044802	-0.005862	0.014235	-0.014536	-0.008198
	0.020149	0.003761	-0.004858	0.003695	0.017329	-0.005939
	0.001130	0.008885	-0.018410	-0.048022	0.053859	0.000231
	0.000305	0.033510	-0.002972	-0.013872	-0.068428	-0.004525
	-0.036750	0.003852	0.040441	-0.005760	0.038958	0.002176
	0.021375					
y63	0.004799	-0.008991	0.022685	0.034723	-0.026721	-0.006742
	-0.031550	-0.001271	-0.026797	-0.010309	-0.036320	-0.034287
	-0.004445	0.014169	-0.018056	0.001859	0.012650	-0.000515
	0.031077	0.002016	-0.049387	0.009607	-0.030426	0.018108
	0.019960	0.013333	0.030445	-0.015110	0.041944	-0.033219
	-0.011779					
z63	-0.038089	0.047297	0.018595	-0.010457	0.005031	0.013935
	-0.003912	0.006854	-0.021004	0.019317	0.024706	-0.006060
	0.004907	-0.002363	0.009436	0.009755	0.008264	-0.018398
	-0.007337	0.001488	0.037075	-0.043489	-0.014138	-0.024668
	-0.006653	-0.031980	0.011635	-0.000530	-0.001454	0.007882
	-0.016579					
x64	-0.026718	0.027986	-0.002298	0.006566	-0.009464	-0.024491
	0.027145	0.011694	-0.003945	0.001084	0.023014	-0.015572
	-0.003287	0.003559	-0.018994	-0.037050	0.032412	0.017630
	0.011117	0.017853	-0.017239	-0.012483	-0.084623	0.010825
	-0.020555	0.020923	0.027278	-0.025184	0.034867	0.013562
	0.007487					
y64	0.004980	-0.008476	0.021534	0.031085	-0.024054	-0.004499
	-0.030823	-0.002355	-0.024764	-0.012151	-0.034375	-0.032741
	-0.009654	0.018869	-0.027237	0.001544	0.011184	-0.003552
	0.028568	0.003570	-0.053048	0.000565	-0.035448	0.013867
	0.006178	0.004157	0.032972	-0.004259	0.042720	-0.032679
	-0.014019					
z64	-0.041934	0.043477	0.015144	-0.009916	0.005754	0.004355
	0.000073	0.011578	-0.021759	0.019697	0.025150	0.004299
	0.004606	-0.000311	0.010521	0.026149	-0.006130	-0.002597
	-0.009261	-0.009828	0.036859	-0.051490	-0.013905	-0.013763
	-0.012274	-0.009417	0.005124	-0.013692	-0.011175	-0.001024
	-0.021647					

x65	-0.030944	0.017372	0.001528	0.006054	-0.008396	-0.029069
	0.028252	-0.004543	0.009356	-0.013250	0.017497	-0.028260
	-0.010859	0.003925	-0.024792	-0.045778	0.022827	0.006652
	0.024820	0.021691	-0.011223	-0.000672	-0.084497	0.002923
	-0.007432	0.007332	0.038118	-0.005775	0.039427	0.015156
	0.030473					
y65	0.007823	-0.007337	0.019072	0.030640	-0.021370	-0.001532
	-0.035532	-0.002957	-0.020540	-0.009576	-0.033070	-0.025328
	-0.019708	0.022240	-0.027506	-0.003165	0.005621	-0.007617
	0.020866	0.012402	-0.057246	-0.004553	-0.035710	-0.002553
	0.009795	0.002843	0.018959	0.006124	0.039540	-0.029721
	-0.028536					
z65	-0.041773	0.044629	0.010865	-0.009314	0.002188	0.006894
	0.000363	0.012736	-0.021449	0.016810	0.022600	0.012453
	0.014367	0.004127	0.010336	0.029553	-0.010699	0.005976
	-0.013512	-0.017910	0.041568	-0.057782	-0.011811	-0.000629
	-0.012357	0.000634	0.004000	-0.019038	-0.014332	-0.001326
	-0.011787					
x66	-0.029606	0.009632	-0.000288	0.009075	-0.011712	-0.029773
	0.031998	-0.010038	0.013255	-0.006645	0.025712	-0.022702
	-0.007255	0.005201	-0.020969	-0.046011	0.002032	0.006061
	0.028952	0.034459	-0.010539	0.006893	-0.045407	0.017083
	-0.017438	0.014398	0.017503	-0.014182	0.030060	0.015824
	0.009135					
y66	0.009136	-0.005985	0.020006	0.031173	-0.020914	-0.003331
	-0.038416	-0.002784	-0.017633	-0.012217	-0.036203	-0.017785
	-0.018603	0.022166	-0.025875	-0.006494	-0.002715	-0.001144
	0.026668	0.017469	-0.051099	-0.001421	-0.031249	0.006406
	0.018068	-0.010553	0.011987	0.010154	0.041442	-0.015005
	-0.046171					
z66	-0.040219	0.047937	0.014138	-0.013470	0.005403	0.005090
	-0.001015	0.014786	-0.010190	0.008278	0.019932	0.006649
	0.001371	0.014201	-0.003715	0.024245	0.004707	0.008807
	-0.018678	-0.018481	0.030788	-0.069925	-0.033878	-0.011776
	-0.004236	-0.016671	0.000958	-0.021306	0.017591	0.004529
	0.007105					
x67	-0.029694	0.003136	-0.000955	0.012014	-0.016538	-0.031637
	0.035181	-0.012022	0.017881	-0.006282	0.027292	-0.020022
	-0.004282	0.009250	-0.018317	-0.045817	-0.008437	0.006544
	0.031142	0.036484	-0.005890	0.017312	-0.027838	0.021245
	-0.016560	0.014518	0.008042	-0.018831	0.018401	0.015510
	0.008862					

y67	0.009170	-0.004819	0.020952	0.028152	-0.021314	-0.004834
	-0.039513	-0.002337	-0.014649	-0.017692	-0.036295	-0.014549
	-0.016315	0.025258	-0.030948	-0.008556	-0.003688	0.001162
	0.028110	0.019521	-0.049886	0.000036	-0.029430	0.006654
	0.019922	-0.025041	0.006683	0.009946	0.049790	-0.001945
	-0.059609					
z67	-0.038763	0.046885	0.016510	-0.014511	0.009080	0.005070
	-0.001765	0.007183	-0.003983	0.003772	0.019274	0.001855
	-0.003218	0.018970	-0.008284	0.017829	0.002629	0.005990
	-0.014143	-0.012824	0.025738	-0.074830	-0.036077	-0.007355
	-0.011126	-0.015061	-0.006712	-0.010019	0.035016	0.004101
	0.010800					
x68	-0.030562	-0.000423	-0.000843	0.013929	-0.018314	-0.034338
	0.036620	-0.011881	0.018502	-0.008402	0.027426	-0.021371
	-0.003046	0.013365	-0.013145	-0.046641	-0.012931	0.004893
	0.031540	0.038533	-0.002329	0.025974	-0.018323	0.023736
	-0.014987	0.012888	0.000203	-0.021660	0.012392	0.009086
	0.003494					
y68	0.009319	-0.003558	0.021634	0.024714	-0.020757	-0.005248
	-0.039660	-0.002013	-0.012687	-0.022145	-0.035536	-0.010944
	-0.014035	0.025055	-0.036332	-0.011855	-0.002386	0.003905
	0.026160	0.020184	-0.048376	-0.000449	-0.030102	0.010629
	0.020250	-0.035795	0.002736	0.013047	0.051495	0.000534
	-0.067816					
z68	-0.037341	0.043942	0.017468	-0.014950	0.010126	0.005669
	-0.000988	-0.000591	0.002592	0.000463	0.019599	0.000587
	-0.005997	0.020953	-0.013665	0.014133	-0.003039	0.005655
	-0.008338	-0.010102	0.023322	-0.078643	-0.034434	-0.001261
	-0.013000	-0.011315	-0.011482	-0.001896	0.044162	0.008854
	0.020225					
x69	-0.030703	-0.003557	-0.001582	0.015986	-0.020144	-0.034233
	0.035496	-0.013007	0.017251	-0.008332	0.027575	-0.024106
	-0.008544	0.009972	-0.012929	-0.049827	-0.019903	0.000729
	0.036375	0.037581	-0.004557	0.028348	-0.010079	0.024200
	-0.011663	0.006621	0.004140	-0.022467	0.004051	0.007656
	-0.009447					
y69	0.009272	-0.002375	0.022031	0.021573	-0.019057	-0.004159
	-0.039242	-0.002469	-0.011868	-0.026237	-0.034324	-0.007393
	-0.010783	0.023655	-0.039903	-0.013473	0.000557	0.007793
	0.021062	0.020238	-0.046024	-0.001675	-0.032477	0.016265
	0.020550	-0.042922	0.000078	0.013480	0.051784	-0.001530
	-0.072515					

z69	-0.036205	0.040960	0.018459	-0.014928	0.010163	0.005447
	0.000574	-0.007421	0.008200	-0.003004	0.019697	0.000074
	-0.006571	0.024283	-0.016821	0.011062	-0.006852	0.005981
	-0.003231	-0.007200	0.021033	-0.080649	-0.031248	0.006176
	-0.014358	-0.006000	-0.017479	0.005128	0.050129	0.011400
	0.029626					
x70	-0.031107	-0.006657	-0.002406	0.016857	-0.021431	-0.034637
	0.034549	-0.014941	0.016419	-0.007837	0.025211	-0.025703
	-0.012439	0.006299	-0.011872	-0.054441	-0.020012	-0.001277
	0.039878	0.039116	-0.006470	0.034424	-0.003940	0.018279
	-0.007499	0.005985	0.008056	-0.018784	-0.001161	0.005305
	-0.018769					
y70	0.009091	-0.001205	0.021960	0.018834	-0.017089	-0.001974
	-0.038913	-0.003342	-0.011626	-0.029866	-0.033025	-0.005049
	-0.007571	0.020565	-0.042495	-0.015092	0.004824	0.012025
	0.013960	0.019121	-0.043512	-0.004242	-0.034950	0.022940
	0.020880	-0.046870	-0.000264	0.011907	0.048509	-0.008112
	-0.073233					
z70	-0.034494	0.038883	0.019345	-0.013157	0.008616	0.006211
	0.002298	-0.011754	0.015123	-0.006154	0.019202	-0.000323
	-0.010044	0.025119	-0.017570	0.006904	-0.009847	0.004841
	0.002664	-0.006713	0.015885	-0.077122	-0.027726	0.009437
	-0.013690	-0.000795	-0.015289	0.008191	0.045658	0.009183
	0.032335					
x71	-0.031056	-0.009349	-0.004791	0.017339	-0.022729	-0.035500
	0.032487	-0.017014	0.013484	-0.006184	0.022164	-0.026900
	-0.010301	0.002384	-0.005105	-0.050542	-0.021257	0.000539
	0.037311	0.041701	-0.006937	0.037014	0.001714	0.016800
	-0.006190	0.005519	0.008975	-0.011746	-0.013310	0.003216
	-0.028955					
y71	0.008722	-0.000017	0.021319	0.015985	-0.015087	0.000397
	-0.038865	-0.004352	-0.011284	-0.033399	-0.031362	-0.003428
	-0.005524	0.016633	-0.044666	-0.017279	0.011189	0.016999
	0.006050	0.017166	-0.041145	-0.008131	-0.037713	0.030583
	0.021368	-0.046581	0.000252	0.008884	0.042595	-0.018819
	-0.070518					
z71	-0.032034	0.038027	0.020084	-0.009473	0.004896	0.007930
	0.004362	-0.013694	0.024477	-0.008336	0.017879	-0.000215
	-0.017556	0.022933	-0.014617	0.000994	-0.011746	0.001439
	0.009656	-0.009019	0.006731	-0.066489	-0.023560	0.006801
	-0.010777	0.004142	-0.004360	0.006345	0.029108	0.001728
	0.026055					

x72	-0.031092	-0.010265	-0.008658	0.016043	-0.022650	-0.035837
	0.029187	-0.019302	0.003577	-0.006166	0.021224	-0.028191
	-0.003441	-0.001844	0.001037	-0.040165	-0.023823	-0.001426
	0.023162	0.041867	0.000295	0.026771	0.003622	0.019216
	-0.005048	-0.009695	0.008769	-0.004990	-0.015094	0.014740
	-0.034009					
y72	0.008136	0.001067	0.019542	0.012792	-0.013158	0.003063
	-0.039052	-0.005597	-0.010574	-0.036557	-0.028634	-0.001787
	-0.004087	0.012072	-0.044413	-0.020213	0.020756	0.023133
	-0.003939	0.015519	-0.036648	-0.014496	-0.039733	0.040244
	0.020270	-0.040992	0.002146	0.005612	0.032836	-0.035964
	-0.065103					
z72	-0.030007	0.037474	0.020473	-0.005265	0.000272	0.011209
	0.007193	-0.017484	0.034138	-0.012242	0.018338	0.000125
	-0.026569	0.018068	-0.009313	-0.005982	-0.016484	-0.006285
	0.015284	-0.012055	-0.001225	-0.054700	-0.018093	0.006882
	-0.005779	0.002984	0.010186	-0.000000	0.014159	-0.003473
	0.018331					
x73	-0.030952	-0.010040	-0.013982	0.013517	-0.019010	-0.039225
	0.026855	-0.018543	-0.012567	-0.003922	0.019319	-0.030845
	0.009654	0.001356	0.003576	-0.023415	-0.019192	-0.003028
	0.009229	0.039110	0.008577	0.011760	-0.000271	0.024108
	-0.012608	-0.023454	-0.005626	0.013119	-0.006800	0.027286
	-0.031664					
y73	0.007289	0.001895	0.016684	0.009866	-0.010912	0.007353
	-0.039198	-0.007613	-0.009984	-0.038888	-0.024214	0.000104
	-0.002235	0.005838	-0.040990	-0.024197	0.032586	0.029531
	-0.019163	0.014182	-0.029813	-0.024430	-0.039724	0.050666
	0.017973	-0.033011	0.007492	0.000953	0.018666	-0.060124
	-0.055709					
z73	-0.029519	0.035398	0.020052	-0.000849	-0.003660	0.012275
	0.012294	-0.020257	0.041992	-0.015292	0.019870	-0.001200
	-0.034177	0.018282	-0.005703	-0.011887	-0.017199	-0.013321
	0.029877	-0.015763	-0.012753	-0.036243	-0.012961	0.009960
	-0.005711	0.005509	0.013727	-0.002339	0.005874	-0.013214
	0.012770					
x74	-0.028018	-0.008279	-0.017903	0.009163	-0.013955	-0.043020
	0.025284	-0.016523	-0.026679	-0.006166	0.014799	-0.035759
	0.022757	0.006458	0.001950	-0.007652	-0.010958	-0.004837
	-0.004704	0.035776	0.021796	-0.006770	-0.002473	0.024351
	-0.015675	-0.033913	-0.012726	0.029475	0.003923	0.029595
	-0.012839					

y74	0.006686	0.001207	0.015893	0.010312	-0.002717	0.017823
	-0.035791	-0.012393	-0.011743	-0.037795	-0.021035	0.000847
	0.005504	-0.006970	-0.037445	-0.031614	0.042291	0.030052
	-0.039651	0.009854	-0.031938	-0.031567	-0.046388	0.047143
	0.018518	-0.020008	0.021364	0.003239	-0.002640	-0.088756
	-0.035494					
z74	-0.026590	0.032755	0.017275	-0.001157	-0.008495	0.009294
	0.019434	-0.022726	0.042482	-0.024259	0.017343	-0.009259
	-0.031738	0.023720	-0.007715	-0.013127	-0.016874	-0.018674
	0.040619	-0.010262	-0.002275	-0.032476	0.000228	0.008294
	-0.004784	0.003165	0.024321	0.005155	0.012976	-0.026554
	0.035470					
x75	-0.022173	0.028878	-0.003989	0.006728	-0.010087	0.065582
	-0.003784	-0.005631	-0.013702	0.014061	-0.050084	0.022374
	-0.007004	0.027110	-0.056994	0.063777	0.017470	-0.009520
	-0.033251	0.022814	0.017036	0.012665	0.006367	0.008699
	0.051144	0.006218	-0.001530	0.043993	0.012789	-0.009939
	-0.019340					
y75	-0.025711	0.019198	-0.018724	0.000982	0.022669	-0.043642
	-0.015854	0.006730	0.026156	0.040695	-0.026869	0.077453
	0.003217	-0.005923	0.027725	0.000117	0.029615	-0.024613
	-0.014290	-0.019654	-0.008136	0.025275	0.006102	0.011525
	0.029593	-0.023839	0.007120	-0.014303	-0.008079	0.063740
	0.006796					
z75	0.000733	0.008286	0.019579	-0.001665	0.014902	-0.016022
	-0.037338	-0.020561	-0.016690	0.033387	0.001361	-0.030217
	0.009436	-0.059179	0.019327	-0.005124	-0.009093	0.001991
	-0.014199	0.006951	0.007309	0.037372	-0.028453	0.039485
	0.012351	-0.018816	0.019582	-0.018948	0.027640	0.031075
	0.023446					
x76	-0.022078	0.026102	-0.003481	0.008178	-0.007564	0.068225
	-0.002987	-0.008475	-0.011133	0.025305	-0.049916	0.017220
	-0.011319	0.029765	-0.068686	0.059763	0.015210	-0.006032
	-0.037333	0.014374	0.015891	0.015833	0.000252	0.018013
	0.027707	-0.016534	-0.001011	0.037618	0.009272	0.011826
	-0.011758					
y76	-0.025663	0.022837	-0.017362	0.005630	0.016805	-0.049136
	-0.012963	0.006664	0.022052	0.039722	-0.025895	0.082417
	0.005123	-0.001486	0.031808	-0.012204	0.039562	-0.022294
	-0.021852	-0.025885	-0.009424	0.000312	0.008899	0.007902
	0.014418	-0.022237	0.005263	-0.008484	0.000526	0.048792
	-0.001485					

z76	0.005334	0.008507	0.023207	0.003671	0.014358	-0.020860
	-0.033916	-0.020969	-0.015395	0.038080	-0.000833	-0.035905
	0.004703	-0.056159	0.017969	-0.006634	-0.020740	0.008145
	-0.006433	0.017666	0.008479	0.062654	-0.033769	0.040712
	0.015943	-0.034089	0.005350	-0.023070	0.035171	0.035316
	0.024250					
x77	-0.021876	0.021400	-0.004021	0.009219	-0.004652	0.070354
	-0.004166	-0.012051	-0.005687	0.033293	-0.045691	0.012364
	-0.013077	0.029909	-0.072933	0.061848	0.011666	0.003066
	-0.038102	0.005694	0.015439	0.017754	-0.001995	0.022329
	0.018344	-0.031404	0.005991	0.030864	0.003723	0.023975
	-0.000284					
y77	-0.025983	0.026779	-0.016788	0.007586	0.009236	-0.056666
	-0.008246	0.006228	0.015932	0.036864	-0.024737	0.081304
	0.005601	0.002160	0.036457	-0.027266	0.050810	-0.023006
	-0.029668	-0.032812	-0.004222	-0.026238	0.012215	0.000189
	-0.012640	-0.026112	0.000852	-0.008449	0.009256	0.042107
	-0.009786					
z77	0.006374	0.006393	0.024531	0.009216	0.013702	-0.020643
	-0.031275	-0.016709	-0.011482	0.045088	0.003689	-0.040280
	0.001399	-0.051455	0.013851	0.008837	-0.036361	0.020867
	-0.002442	0.028985	0.009052	0.070624	-0.038878	0.044033
	0.033432	-0.013585	0.007573	-0.020120	0.037485	0.025932
	0.018501					
x78	-0.020522	0.017829	-0.005566	0.010344	-0.001491	0.069752
	-0.006107	-0.015440	-0.000798	0.037161	-0.042135	0.009780
	-0.014055	0.027335	-0.075048	0.059837	0.010580	0.013945
	-0.036012	0.001681	0.014189	0.019536	0.000231	0.023887
	0.007919	-0.039192	0.013461	0.023897	0.002645	0.028481
	0.004505					
y78	-0.026980	0.028254	-0.017247	0.008482	0.003015	-0.061066
	-0.001938	0.005896	0.012615	0.037341	-0.022443	0.078618
	0.006764	0.007093	0.041641	-0.032210	0.054958	-0.024804
	-0.036725	-0.037386	0.002017	-0.044308	0.007290	-0.005891
	-0.035294	-0.036624	-0.005431	-0.009659	0.023131	0.036140
	-0.022147					
z78	0.008314	0.006599	0.025285	0.014150	0.012524	-0.023443
	-0.033180	-0.013085	-0.012470	0.043368	0.006498	-0.043566
	-0.002793	-0.049558	0.006623	0.010785	-0.041102	0.033050
	-0.002247	0.034418	0.006067	0.068907	-0.029800	0.047320
	0.045146	0.012677	0.009877	-0.013761	0.030049	0.017841
	0.024744					

x79	-0.018837	0.015620	-0.007099	0.011583	0.001440	0.067287
	-0.008106	-0.017367	0.002899	0.039759	-0.040426	0.008924
	-0.013431	0.024844	-0.076686	0.056380	0.011982	0.023774
	-0.032314	0.000750	0.013859	0.020018	0.001833	0.026866
	-0.004835	-0.043815	0.016686	0.018868	-0.001121	0.029463
	-0.000953					
y79	-0.026966	0.026560	-0.017568	0.011217	0.000300	-0.060622
	-0.000583	0.005797	0.015494	0.038292	-0.019681	0.072306
	0.000459	0.006239	0.046088	-0.037299	0.052655	-0.020267
	-0.045249	-0.037473	0.004438	-0.051423	0.002597	-0.008048
	-0.039267	-0.029564	0.003167	-0.004554	0.035320	0.035614
	-0.016090					
z79	0.009248	0.007966	0.025553	0.016692	0.009062	-0.027910
	-0.032891	-0.009636	-0.017322	0.041564	0.008324	-0.042646
	0.000777	-0.043665	0.001330	0.015434	-0.044650	0.038542
	0.001528	0.034117	0.008104	0.060595	-0.022018	0.044681
	0.046874	0.020759	0.002332	-0.018973	0.020831	0.009869
	0.017679					
x80	-0.017669	0.013971	-0.008586	0.012547	0.005037	0.063536
	-0.010126	-0.018904	0.006086	0.039420	-0.039421	0.009273
	-0.013824	0.022936	-0.076237	0.050830	0.014265	0.030283
	-0.031154	-0.001089	0.013767	0.019290	0.001406	0.030623
	-0.013626	-0.046318	0.015650	0.020654	-0.000418	0.027205
	-0.003991					
y80	-0.028437	0.023470	-0.017069	0.011463	0.002730	-0.060400
	-0.001483	0.002165	0.022415	0.033603	-0.015408	0.065199
	-0.013829	-0.008071	0.040354	-0.032146	0.037013	0.001040
	-0.037154	-0.033076	0.012704	-0.047926	-0.009871	0.002855
	-0.025368	-0.009419	0.015880	-0.022821	0.041423	0.032800
	-0.009795					
z80	0.010223	0.009574	0.024264	0.019490	0.003362	-0.032611
	-0.031936	-0.006673	-0.022639	0.041038	0.006774	-0.040751
	0.005656	-0.036999	-0.000787	0.015113	-0.043073	0.034669
	-0.003323	0.030117	0.008088	0.048083	-0.011715	0.033938
	0.038172	0.017925	0.000166	-0.013474	0.013814	0.003131
	0.012753					
x81	-0.017176	0.012717	-0.009486	0.012911	0.009006	0.059371
	-0.011075	-0.019255	0.008058	0.038564	-0.038426	0.009463
	-0.015701	0.021109	-0.074711	0.047910	0.014657	0.034120
	-0.029549	-0.002872	0.014200	0.017989	0.001666	0.032198
	-0.014334	-0.047173	0.013135	0.023593	-0.000198	0.023181
	-0.005893					

y81	-0.033344	0.018775	-0.015008	0.007392	0.006571	-0.063314
	-0.002301	0.003136	0.027059	0.025623	-0.007364	0.054631
	-0.025636	-0.024740	0.041130	-0.017169	0.025353	0.028552
	-0.023268	-0.026388	0.014387	-0.042011	-0.019884	0.017565
	-0.014518	0.020054	0.025030	-0.039513	0.029721	0.027429
	-0.004464					
z81	0.010364	0.009475	0.022433	0.023268	0.000293	-0.039182
	-0.031575	-0.005378	-0.026879	0.042907	0.003626	-0.037080
	0.008896	-0.033460	-0.001216	0.019343	-0.041574	0.031522
	-0.009318	0.023753	0.008984	0.037001	-0.003591	0.021535
	0.023203	0.015821	-0.003594	-0.008680	0.003684	-0.007850
	0.008288					
x82	-0.016459	0.011040	-0.010050	0.011963	0.013481	0.053919
	-0.011110	-0.017396	0.008653	0.039137	-0.036180	0.009558
	-0.017841	0.017578	-0.071624	0.048522	0.010766	0.038042
	-0.022871	-0.004635	0.015482	0.017959	0.006341	0.030514
	-0.008262	-0.044306	0.012647	0.022855	-0.000443	0.017683
	-0.010715					
y82	-0.038469	0.010335	-0.012238	0.001109	0.011801	-0.067649
	-0.003968	0.003522	0.028763	0.019881	0.002475	0.043858
	-0.026383	-0.034687	0.040746	0.006350	0.023111	0.051720
	-0.002330	-0.021404	0.012176	-0.028909	-0.019747	0.035270
	-0.001648	0.044568	0.023619	-0.032371	0.004018	0.040276
	-0.000424					
z82	0.010276	0.008795	0.020930	0.025746	-0.005116	-0.042970
	-0.031064	-0.004319	-0.028892	0.047528	0.004583	-0.033623
	0.014108	-0.025005	-0.001796	0.023650	-0.038380	0.025649
	-0.014458	0.019836	0.007718	0.028122	0.000569	0.013718
	0.014145	0.014892	-0.010840	-0.003811	-0.005986	-0.011060
	0.005369					
x83	-0.015330	0.009224	-0.010908	0.009206	0.018569	0.047535
	-0.010106	-0.013696	0.009636	0.039000	-0.030176	0.007338
	-0.020030	0.014613	-0.067193	0.050364	0.008056	0.041191
	-0.010295	-0.006316	0.016170	0.019436	0.011806	0.022526
	-0.005851	-0.038355	0.014731	0.019976	-0.000846	0.010947
	-0.019907					
y83	-0.042465	0.000928	-0.012682	-0.005625	0.012361	-0.069310
	-0.001936	0.002626	0.029390	0.017977	0.011159	0.033381
	-0.022146	-0.028953	0.033690	0.014558	0.023771	0.047476
	0.012329	-0.029579	-0.005062	-0.012695	-0.001541	0.046598
	0.003339	0.048548	0.021330	-0.011120	0.000699	0.052975
	-0.005420					

z83	0.011057	0.008497	0.019828	0.029046	-0.012733	-0.045196
	-0.030911	-0.000303	-0.030638	0.052166	0.005263	-0.033217
	0.020310	-0.012017	0.000139	0.021930	-0.034246	0.022840
	-0.021711	0.018107	0.003776	0.018386	0.007080	0.010753
	0.006960	0.014588	-0.015831	0.000380	-0.007875	-0.015311
	0.003470					
x84	-0.013326	0.008448	-0.013337	0.008193	0.023390	0.047122
	-0.014819	-0.017702	0.013717	0.033217	-0.024610	0.001654
	-0.018928	0.010834	-0.061106	0.047255	0.015732	0.038327
	-0.005794	-0.002748	0.014913	0.027294	0.016878	0.016895
	-0.010987	-0.030341	0.011182	0.028037	0.003787	0.006793
	-0.016766					
y84	-0.043873	-0.004404	-0.013452	-0.005046	0.016512	-0.062303
	0.000753	-0.011491	0.035038	0.008802	0.012941	0.024092
	-0.029789	-0.031019	0.028162	0.000270	0.017719	0.035366
	0.019318	-0.030375	-0.004787	-0.009791	-0.009080	0.053664
	0.000326	0.041409	0.033658	-0.013016	0.012928	0.055123
	0.006156					
z84	0.013106	0.009341	0.018462	0.032519	-0.020936	-0.048947
	-0.028024	0.004035	-0.033566	0.055703	0.006861	-0.031183
	0.025788	-0.002188	-0.003083	0.020142	-0.036666	0.023482
	-0.025478	0.013369	0.004832	0.008187	0.015275	0.005192
	0.006562	0.011747	-0.010868	0.004022	-0.015629	-0.016528
	-0.001673					
x85	-0.013413	0.006074	-0.015738	0.007842	0.024645	0.043415
	-0.016119	-0.020244	0.018779	0.032649	-0.024729	0.002670
	-0.020330	0.006281	-0.061166	0.042725	0.021627	0.037400
	-0.000330	-0.002130	0.018447	0.019095	0.020381	0.015973
	-0.017335	-0.019769	0.012839	0.027524	-0.011723	0.006369
	-0.012120					
y85	-0.048903	-0.006432	-0.016322	-0.004359	0.014228	-0.051651
	0.002822	-0.014683	0.034060	0.013177	0.012260	0.018970
	-0.018659	-0.026926	0.030151	-0.001863	0.011855	0.023635
	0.028042	-0.015362	0.005806	-0.013374	0.006349	0.036451
	0.002161	0.041456	0.031606	-0.003608	0.003722	0.049685
	0.008020					
z85	0.017274	0.009273	0.020680	0.036474	-0.023118	-0.055981
	-0.027561	0.006194	-0.034020	0.049555	0.012377	-0.034282
	0.024375	0.004676	-0.007944	0.014374	-0.026085	0.029178
	-0.031183	0.001270	-0.003968	0.011004	0.009384	0.010822
	-0.005343	0.005922	-0.012108	0.000577	-0.008901	-0.014761
	-0.004945					

x86	-0.013533	0.003738	-0.016614	0.007625	0.026854	0.037724
	-0.014253	-0.019202	0.021356	0.034991	-0.025254	0.005445
	-0.020858	0.003095	-0.059233	0.039455	0.016040	0.037163
	0.007428	-0.005226	0.019943	0.007154	0.021754	0.014734
	-0.019167	-0.010159	0.022403	0.025991	-0.023051	0.006156
	-0.012560					
y86	-0.051263	-0.007513	-0.018269	-0.004669	0.011154	-0.048102
	0.005989	-0.011012	0.031810	0.014639	0.014448	0.018253
	-0.011550	-0.016860	0.026451	-0.003044	0.013119	0.015468
	0.032348	-0.014003	0.007438	-0.010650	0.016708	0.028011
	0.000342	0.035095	0.023282	-0.001171	0.005072	0.047511
	0.008823					
z86	0.018958	0.008675	0.021869	0.040947	-0.024616	-0.055843
	-0.029019	0.000294	-0.033483	0.044582	0.012087	-0.040462
	0.023623	0.005468	-0.007833	0.009155	-0.024940	0.031527
	-0.034199	0.003013	-0.003950	0.007893	0.008235	0.013355
	-0.011424	0.008178	-0.009444	0.004646	-0.013478	-0.019183
	-0.005523					
x87	-0.013102	0.001279	-0.017091	0.008512	0.029207	0.033820
	-0.013600	-0.019940	0.023903	0.035731	-0.025633	0.006830
	-0.019205	0.000524	-0.056593	0.035773	0.013417	0.037940
	0.013006	-0.006775	0.019247	-0.002428	0.020248	0.012000
	-0.022241	-0.001374	0.030605	0.025052	-0.029673	0.005568
	-0.010938					
y87	-0.052352	-0.009291	-0.020112	-0.003894	0.008080	-0.046085
	0.008497	-0.007825	0.032510	0.014499	0.016173	0.019486
	-0.007504	-0.009878	0.024384	-0.003320	0.011570	0.011148
	0.034370	-0.016874	0.009130	-0.003975	0.024420	0.025483
	0.002501	0.029169	0.018125	0.002655	0.005488	0.045565
	0.015411					
z87	0.020262	0.008403	0.022498	0.043732	-0.026071	-0.054651
	-0.029814	-0.004624	-0.033596	0.041491	0.011844	-0.046442
	0.023654	0.006791	-0.008187	0.003572	-0.022585	0.030705
	-0.036652	0.007220	-0.004492	0.003409	0.009669	0.011810
	-0.019116	0.011022	-0.009023	0.006437	-0.017420	-0.022739
	-0.009934					
x88	-0.012019	-0.000994	-0.017177	0.009268	0.031137	0.031385
	-0.013007	-0.021861	0.025628	0.035342	-0.025382	0.007142
	-0.017156	-0.000927	-0.053671	0.032728	0.011593	0.040215
	0.019425	-0.006044	0.018087	-0.006902	0.018215	0.008879
	-0.026093	0.003294	0.038133	0.022469	-0.033923	0.004294
	-0.009140					

y88	-0.052430	-0.012099	-0.021805	-0.004806	0.006217	-0.043802
	0.011441	-0.006503	0.032553	0.009382	0.016546	0.018773
	-0.010030	-0.003858	0.020945	-0.008342	0.008649	0.008286
	0.034943	-0.020377	0.012998	0.002340	0.026253	0.025137
	0.004546	0.022295	0.012733	-0.003738	0.004367	0.046105
	0.017468					
z88	0.021419	0.008330	0.022557	0.046301	-0.028104	-0.053675
	-0.030227	-0.008886	-0.032479	0.040170	0.011967	-0.050394
	0.024224	0.006970	-0.008070	-0.002411	-0.020428	0.027328
	-0.039706	0.010805	-0.005572	-0.000415	0.012540	0.009883
	-0.025522	0.014930	-0.008537	0.009058	-0.021811	-0.025704
	-0.014680					
x89	-0.010254	-0.003117	-0.017100	0.009761	0.032663	0.029951
	-0.012005	-0.024335	0.027699	0.034256	-0.024936	0.008175
	-0.015177	-0.000313	-0.051033	0.031230	0.010904	0.043173
	0.026981	-0.005373	0.014830	-0.007203	0.015097	0.006019
	-0.030831	0.004917	0.045144	0.020936	-0.033128	0.001838
	-0.005956					
y89	-0.052760	-0.015072	-0.022352	-0.005979	0.004681	-0.040107
	0.012807	-0.005050	0.035046	0.004559	0.016109	0.021736
	-0.014264	0.000675	0.017720	-0.007520	0.008491	0.010242
	0.036325	-0.023576	0.014566	0.004407	0.031746	0.030037
	0.002823	0.021146	0.007792	0.001463	0.007263	0.037955
	0.026970					
z89	0.022244	0.007658	0.021662	0.048145	-0.031048	-0.052356
	-0.028746	-0.012190	-0.027946	0.037625	0.010369	-0.049609
	0.020708	0.008912	-0.006158	-0.006580	-0.020065	0.021784
	-0.041441	0.007112	-0.007603	-0.003652	0.014250	0.006253
	-0.030701	0.018670	-0.008959	0.010507	-0.025654	-0.021239
	-0.014577					
x90	-0.007999	-0.005252	-0.017009	0.009325	0.034040	0.028412
	-0.010722	-0.027699	0.030011	0.033136	-0.024832	0.009387
	-0.013109	0.002698	-0.048049	0.029307	0.011273	0.046370
	0.035230	-0.004474	0.010355	-0.003398	0.009728	0.001651
	-0.035734	0.004324	0.051706	0.018416	-0.026321	-0.001111
	-0.003467					
y90	-0.053253	-0.018697	-0.022836	-0.008680	0.005711	-0.036869
	0.012413	-0.004689	0.035801	0.001650	0.016390	0.022146
	-0.016117	0.005766	0.015935	-0.008510	0.007991	0.016057
	0.039126	-0.024899	0.018207	0.005282	0.036524	0.031286
	0.004409	0.023398	0.005506	-0.002269	0.017338	0.022072
	0.027827					

z90	0.022453	0.006143	0.019242	0.048831	-0.034854	-0.051416
	-0.025099	-0.014634	-0.019703	0.034097	0.006994	-0.045188
	0.012739	0.013474	-0.002354	-0.010508	-0.021549	0.014887
	-0.041357	-0.004613	-0.010228	-0.003175	0.010937	-0.001127
	-0.032994	0.022061	-0.008865	0.007509	-0.027800	-0.007217
	-0.009030					
x91	-0.005051	-0.006476	-0.016488	0.008575	0.035302	0.026383
	-0.009549	-0.032011	0.031966	0.032074	-0.025445	0.010035
	-0.009743	0.004982	-0.041284	0.027265	0.012268	0.048719
	0.041440	-0.001852	0.004996	0.005611	0.002540	-0.001799
	-0.038921	-0.001059	0.055571	0.016825	-0.013220	-0.002281
	-0.000069					
y91	-0.052883	-0.019537	-0.023333	-0.012308	0.010026	-0.034003
	0.009516	-0.005327	0.033909	0.001249	0.015822	0.020369
	-0.012433	0.004192	0.016555	-0.003375	0.010896	0.018860
	0.038017	-0.018073	0.024834	0.007037	0.045994	0.040131
	0.010793	0.017267	0.001964	0.001225	0.020597	0.002241
	0.026764					
z91	0.022573	0.005123	0.015461	0.048307	-0.039379	-0.049647
	-0.020063	-0.018739	-0.008613	0.030893	0.001747	-0.039023
	0.002325	0.013495	0.003689	-0.014760	-0.024224	0.005335
	-0.041660	-0.015835	-0.013518	0.003797	0.007351	-0.007618
	-0.033288	0.022295	-0.006715	0.004881	-0.037464	0.014403
	-0.000287					
x92	-0.001823	-0.006498	-0.014185	0.008983	0.035888	0.026040
	-0.008168	-0.035067	0.030272	0.028712	-0.023771	0.008342
	-0.003969	0.008026	-0.032677	0.027796	0.012740	0.053413
	0.046267	0.002326	0.000289	0.016753	-0.004043	-0.003182
	-0.039181	-0.011860	0.058912	0.013663	0.002767	-0.006903
	0.003640					
y92	-0.050421	-0.017437	-0.021604	-0.016287	0.018474	-0.030491
	0.004915	-0.003725	0.024614	-0.000490	0.018789	0.014799
	-0.004758	0.004822	0.008272	0.003973	0.022462	0.022344
	0.037641	-0.004733	0.033162	-0.003203	0.060727	0.050997
	0.013864	0.000936	0.000199	0.010070	0.023112	-0.038236
	0.017198					
z92	0.022550	0.004963	0.012747	0.045322	-0.042789	-0.046002
	-0.013777	-0.021807	-0.000805	0.025785	-0.001321	-0.033171
	-0.008966	0.011785	0.002521	-0.018428	-0.021032	-0.003499
	-0.039660	-0.023023	-0.017439	0.008316	0.006992	-0.012987
	-0.037335	0.015069	-0.004013	0.003014	-0.053168	0.023819
	0.003093					

x93	0.001059	-0.004958	-0.011633	0.010835	0.036331	0.028521
	-0.005216	-0.035549	0.025671	0.021905	-0.018852	0.004032
	0.003518	0.012873	-0.026760	0.033079	0.008874	0.061686
	0.053238	0.009448	-0.003330	0.029167	-0.010121	-0.002226
	-0.036489	-0.025816	0.066707	0.008482	0.022465	-0.014756
	0.010099					
y93	-0.044902	-0.013756	-0.020409	-0.018752	0.029067	-0.027388
	0.000546	-0.000208	0.016377	-0.003290	0.022498	0.008638
	0.004296	0.007295	-0.000644	0.007687	0.029416	0.027275
	0.039107	0.010827	0.038443	-0.014133	0.072358	0.058960
	0.017490	-0.009246	0.002482	0.014448	0.028960	-0.073331
	0.009652					
z93	0.025291	0.005473	0.009922	0.040042	-0.039346	-0.043271
	-0.009777	-0.022159	0.004495	0.017459	0.000862	-0.026031
	-0.013873	0.008571	-0.000441	-0.020690	-0.022529	-0.000268
	-0.030913	-0.015917	-0.016221	0.012443	0.015425	-0.012643
	-0.029931	0.011809	-0.002256	-0.004410	-0.053869	0.011266
	0.008201					
x94	0.008925	0.014847	0.001174	0.015791	-0.012043	0.029599
	-0.007683	0.027882	-0.021607	-0.022621	-0.025678	0.002250
	-0.001230	0.017321	0.037034	-0.026557	-0.071448	-0.008775
	0.011068	0.013060	0.052212	-0.015434	0.031259	-0.046034
	-0.009986	0.034475	0.022099	-0.004996	-0.021129	0.010377
	0.004764					
y94	0.007768	0.023001	-0.015296	0.000488	-0.024586	0.006824
	0.028535	-0.005479	0.028984	0.050922	-0.005397	0.012342
	0.005391	-0.030124	-0.005621	0.006370	-0.039751	0.001512
	0.025099	0.016467	0.003224	0.002291	0.024191	-0.005945
	-0.009101	-0.000485	-0.014703	-0.011681	0.046364	0.013147
	0.016435					
z94	0.026099	-0.063587	-0.012084	-0.026711	-0.006661	0.030462
	-0.036734	0.019079	0.000351	0.005774	0.025920	0.005760
	0.009703	0.019473	0.000032	-0.034926	-0.035154	-0.010273
	-0.018044	0.004679	-0.036316	0.028764	-0.005788	-0.027883
	-0.035225	0.049923	0.026408	0.033015	0.021218	0.007415
	-0.047266					
x95	0.011873	0.016527	0.004143	0.023283	-0.003441	0.026396
	-0.009196	0.022946	-0.017955	-0.033184	-0.019888	-0.004374
	0.005577	0.026804	0.038935	-0.032312	-0.073845	-0.002623
	0.025307	0.008240	0.062882	-0.010287	0.037664	-0.038104
	-0.027555	0.023233	0.025925	-0.013464	-0.000625	0.002946
	0.020679					

y95	0.003189	0.020685	-0.018461	0.001661	-0.033338	0.021207
	0.023414	-0.010630	0.023584	0.043663	-0.001657	0.006399
	0.000787	-0.029432	0.000086	-0.006395	-0.036593	-0.000787
	0.021315	0.003666	-0.002314	0.021558	0.008525	-0.005075
	-0.017089	0.015476	-0.001085	0.006723	0.052506	0.008611
	0.004076					
z95	0.025937	-0.065900	-0.010960	-0.025344	-0.008341	0.023723
	-0.023889	0.023140	0.000942	0.014158	0.029925	0.008061
	0.008047	0.034217	-0.007483	-0.028269	-0.031108	-0.011854
	-0.002192	-0.005889	-0.024120	0.027539	-0.020733	-0.006949
	-0.014400	0.024622	0.023123	0.046828	0.016610	-0.003764
	-0.061904					
x96	0.013408	0.014616	0.005456	0.027618	0.003511	0.022606
	-0.008747	0.019406	-0.012343	-0.038553	-0.012003	-0.011973
	0.012150	0.035298	0.041026	-0.034325	-0.073549	-0.001601
	0.034449	0.000604	0.067750	-0.001075	0.037418	-0.026798
	-0.041791	0.016062	0.026909	-0.012326	0.009958	-0.000351
	0.033981					
y96	0.002756	0.022213	-0.021737	0.001331	-0.035368	0.027915
	0.024121	-0.016241	0.021012	0.036621	-0.001883	-0.003194
	0.000467	-0.033477	0.002437	-0.010508	-0.029947	-0.003589
	0.019169	-0.002785	-0.006278	0.023712	0.005295	-0.003329
	-0.004349	0.012471	-0.000782	0.002254	0.053728	0.019824
	0.012738					
z96	0.031125	-0.065696	-0.009297	-0.023845	-0.007775	0.011600
	-0.006854	0.021382	-0.000950	0.023987	0.027515	0.008767
	0.012072	0.042670	-0.012453	-0.017993	-0.017633	-0.013860
	0.010765	-0.010650	-0.001791	0.007857	-0.015145	0.007391
	0.014967	-0.020610	0.007972	0.040111	0.006716	0.003263
	-0.049951					
x97	0.014654	0.011811	0.005515	0.030470	0.008707	0.018846
	-0.008237	0.016864	-0.007250	-0.039747	-0.007300	-0.016352
	0.019848	0.038616	0.044239	-0.033813	-0.073559	0.000000
	0.040665	-0.005312	0.068807	0.006554	0.037874	-0.017095
	-0.046082	0.010541	0.027058	-0.012222	0.011710	0.002102
	0.041227					
y97	0.009426	0.029375	-0.022197	-0.000084	-0.029636	0.025151
	0.023844	-0.017771	0.017972	0.028483	-0.007501	-0.000987
	-0.003533	-0.040479	0.005010	-0.012419	-0.027462	0.005131
	0.012963	-0.010931	-0.005995	0.014346	-0.002892	-0.008231
	0.015590	0.004706	-0.005978	-0.002104	0.044221	0.034047
	0.020768					

z97	0.038554	-0.065457	-0.006565	-0.022946	-0.004376	-0.000072
	0.002953	0.022184	-0.001130	0.031468	0.024255	0.012232
	0.011717	0.046619	-0.016291	-0.011314	-0.008139	-0.009202
	0.017657	-0.015580	0.014501	-0.012055	-0.012893	0.015139
	0.038468	-0.056444	-0.008847	0.031698	-0.001229	0.007748
	-0.037979					
x98	0.015363	0.009608	0.005215	0.032812	0.013001	0.015374
	-0.007552	0.014440	-0.005704	-0.039618	-0.003897	-0.016327
	0.026353	0.040521	0.046301	-0.032147	-0.073985	0.001378
	0.046907	-0.009297	0.066402	0.009336	0.039709	-0.009747
	-0.046307	0.003685	0.027334	-0.012183	0.009768	0.004731
	0.040342					
y98	0.015251	0.031822	-0.019760	0.000694	-0.028401	0.023218
	0.023591	-0.016710	0.013653	0.026001	-0.010850	0.009477
	-0.008524	-0.038774	0.006206	-0.011561	-0.029852	0.014888
	0.006603	-0.021167	-0.001168	0.006561	-0.005405	-0.005077
	0.018395	-0.001431	-0.010909	0.008145	0.034154	0.044420
	0.013408					
z98	0.044576	-0.065720	-0.003875	-0.021125	-0.002831	-0.008232
	0.010209	0.023165	-0.000230	0.035750	0.021432	0.014382
	0.010146	0.050080	-0.018095	-0.004189	-0.002547	-0.004828
	0.021905	-0.019050	0.027402	-0.023241	-0.010372	0.022475
	0.049311	-0.076293	-0.021215	0.028310	-0.006683	0.007364
	-0.034671					
x99	0.015492	0.007874	0.003980	0.034298	0.016404	0.012906
	-0.006285	0.011253	-0.005889	-0.037057	-0.001159	-0.013500
	0.030816	0.041400	0.047655	-0.029919	-0.076569	0.001455
	0.053831	-0.010968	0.061317	0.011307	0.039434	-0.005566
	-0.043702	-0.003423	0.027667	-0.012365	0.007064	0.004740
	0.032887					
y99	0.019984	0.030522	-0.020372	0.002279	-0.030574	0.022909
	0.023978	-0.018495	0.014772	0.030794	-0.014648	0.019348
	-0.011244	-0.034520	0.008907	-0.019233	-0.033383	0.020463
	0.010728	-0.029509	0.004385	0.006830	-0.012745	-0.009972
	0.029155	-0.022473	-0.010029	0.018430	0.020905	0.050647
	-0.012447					
z99	0.048422	-0.064048	-0.001104	-0.017826	-0.001057	-0.012851
	0.013865	0.023010	0.002986	0.033316	0.021024	0.012333
	0.002776	0.046306	-0.019453	-0.000745	0.000868	-0.003282
	0.018324	-0.018361	0.032129	-0.022782	-0.009579	0.028317
	0.048224	-0.072863	-0.020703	0.021073	-0.004055	-0.003388
	-0.026187					

x100	0.015114	0.006130	0.001845	0.034827	0.018287	0.010506
	-0.003981	0.008556	-0.008388	-0.032763	0.001458	-0.010358
	0.035198	0.041159	0.047138	-0.026128	-0.079530	-0.000379
	0.059612	-0.011538	0.051142	0.011207	0.037708	-0.003034
	-0.040024	-0.010747	0.029593	-0.010109	0.001011	0.004166
	0.020878					
y100	0.024652	0.028810	-0.020358	0.003499	-0.032582	0.022558
	0.025079	-0.019698	0.015620	0.033312	-0.014967	0.019711
	-0.007240	-0.029268	0.010172	-0.023651	-0.034964	0.022833
	0.010989	-0.033064	0.012692	0.000780	-0.010380	-0.007386
	0.032751	-0.041923	-0.005669	0.021426	0.021842	0.038926
	-0.026164					
z100	0.050090	-0.060755	0.002003	-0.013396	0.002307	-0.015064
	0.015178	0.021365	0.009115	0.025972	0.024064	0.007283
	-0.011239	0.037765	-0.020803	-0.001417	0.001169	-0.005377
	0.008501	-0.013329	0.030418	-0.016181	-0.010769	0.036569
	0.037739	-0.048239	-0.013755	0.011307	0.002549	-0.018363
	-0.006985					
x101	0.013997	0.003889	-0.001073	0.035699	0.018650	0.008323
	-0.002205	0.004825	-0.011631	-0.026222	0.001699	-0.006174
	0.039491	0.038650	0.046594	-0.020666	-0.081700	-0.004104
	0.063378	-0.011179	0.036257	0.010505	0.035039	-0.002057
	-0.029992	-0.019289	0.030833	-0.004251	-0.014036	0.009767
	0.001486					
y101	0.029484	0.029709	-0.018760	0.006835	-0.032210	0.022814
	0.024752	-0.021325	0.017932	0.034388	-0.017660	0.013741
	-0.004518	-0.032166	0.013343	-0.025703	-0.035696	0.019746
	0.012570	-0.035544	0.021653	-0.001531	-0.004693	0.003881
	0.030832	-0.048288	-0.002837	0.025963	0.019923	0.028724
	-0.033943					
z101	0.050786	-0.058777	0.004683	-0.009779	0.005071	-0.016306
	0.016496	0.018851	0.016808	0.016169	0.029251	0.002201
	-0.029625	0.032292	-0.024395	-0.004390	-0.001414	-0.008820
	-0.007615	-0.007991	0.025573	-0.008247	-0.016805	0.043403
	0.021269	-0.019314	-0.003139	-0.000187	0.009885	-0.036260
	0.012526					
x102	0.011739	0.001210	-0.005354	0.035304	0.017229	0.006369
	0.000461	0.000662	-0.016826	-0.019804	-0.000255	-0.000484
	0.042911	0.035096	0.044205	-0.015420	-0.081011	-0.011435
	0.062906	-0.008611	0.012582	0.008346	0.026497	-0.002711
	-0.019121	-0.029077	0.033930	0.008436	-0.036398	0.017794
	-0.023380					

y102	0.035998	0.031760	-0.014953	0.009355	-0.030305	0.022879
	0.025758	-0.026727	0.022541	0.027934	-0.015470	-0.000210
	-0.013551	-0.034231	0.013622	-0.025553	-0.036000	0.002572
	0.001254	-0.029260	0.034805	-0.002509	-0.010097	0.019944
	0.007009	-0.039925	0.007725	0.028716	0.021856	-0.003722
	-0.028935					
z102	0.048552	-0.057297	0.005532	-0.004251	0.007126	-0.014559
	0.014725	0.021004	0.026432	0.011043	0.029722	0.000381
	-0.034663	0.022629	-0.022876	-0.010380	-0.006794	-0.002240
	-0.012377	-0.009110	0.015509	0.000618	-0.013124	0.052942
	0.017505	0.002212	-0.000017	-0.008703	0.014057	-0.036945
	0.016723					
x103	0.008585	-0.000645	-0.010742	0.033669	0.015385	0.006413
	0.003063	-0.000827	-0.024222	-0.013133	-0.002076	0.011047
	0.046935	0.029356	0.042530	-0.008201	-0.083286	-0.016207
	0.061847	-0.005881	-0.016338	0.005214	0.016270	-0.004402
	-0.001562	-0.035180	0.036859	0.017649	-0.064347	0.026918
	-0.048471					
y103	0.041635	0.032334	-0.009960	0.011907	-0.026466	0.024597
	0.024415	-0.032281	0.029279	0.019350	-0.012020	-0.018418
	-0.024485	-0.037275	0.015672	-0.023968	-0.030447	-0.017672
	-0.018650	-0.020254	0.045912	-0.001859	-0.019972	0.042340
	-0.024640	-0.024953	0.019270	0.030510	0.024972	-0.048966
	-0.020657					
z103	0.045834	-0.051595	0.004641	0.001340	0.005045	-0.015268
	0.018265	0.030609	0.030353	0.003756	0.031633	0.010098
	-0.036485	0.021174	-0.020442	-0.014283	-0.010397	0.010543
	-0.009985	-0.008615	0.007962	0.001228	-0.010372	0.042286
	0.026473	0.012680	-0.013296	-0.008146	0.003405	-0.008914
	0.027709					
x104	-0.013540	-0.004792	-0.026094	-0.020087	0.002639	0.016759
	-0.052227	0.004046	-0.038855	0.000567	-0.005388	-0.000361
	0.019461	-0.037425	-0.002497	-0.018070	-0.007194	-0.005037
	-0.028121	-0.031633	0.007060	-0.001498	0.022858	-0.010487
	-0.014497	0.017922	0.045159	-0.004313	-0.007428	-0.027396
	0.024353					
y104	-0.021334	-0.001130	-0.035896	0.011329	-0.022082	0.039459
	0.014384	-0.011579	-0.014928	0.001185	0.010771	-0.022081
	-0.024926	-0.004103	0.009842	0.001281	0.017200	-0.015900
	-0.039313	-0.009046	-0.016310	-0.002595	0.031621	-0.003480
	-0.050015	-0.007557	-0.023505	0.024602	0.033158	0.002545
	-0.003355					

z104	-0.015764	-0.015124	-0.030243	-0.033970	-0.041019	0.033783
	-0.037101	-0.016524	-0.015412	-0.043045	0.013682	0.020093
	0.003402	-0.004010	0.024737	0.007578	-0.031288	-0.030527
	-0.014174	-0.014599	-0.007970	0.003887	0.030017	0.012417
	0.000548	0.007615	0.001582	-0.021642	0.016486	-0.017330
	-0.001372					
x105	-0.008896	-0.012838	-0.020454	-0.012627	0.010432	0.005903
	-0.046900	0.011395	-0.016667	-0.003567	0.001296	-0.006410
	0.014782	-0.027394	-0.005133	-0.019170	-0.003448	-0.010703
	-0.020893	-0.018922	-0.007492	-0.012161	0.012012	-0.005496
	0.007414	0.006947	0.026335	-0.003433	-0.015500	-0.018231
	0.004225					
y105	-0.021620	0.000515	-0.039378	0.010436	-0.025814	0.042781
	0.010637	-0.011299	-0.017146	-0.007166	0.005290	-0.019835
	-0.018494	0.001427	0.014465	-0.007842	0.025092	-0.021956
	-0.040270	-0.009166	-0.016745	0.000465	0.033215	-0.000816
	-0.044370	-0.013692	-0.026322	0.023537	0.023159	0.012283
	0.006153					
z105	-0.015040	-0.010051	-0.021334	-0.027817	-0.043281	0.030201
	-0.024260	-0.008024	-0.015965	-0.041300	0.014460	0.013038
	0.009075	0.006268	0.014516	0.011722	-0.021334	-0.019115
	0.003792	-0.011060	-0.013543	0.011169	0.038163	0.007793
	-0.011167	-0.001700	-0.004395	-0.010856	0.005720	-0.034717
	0.006867					
x106	-0.006739	-0.020309	-0.016396	-0.006387	0.017757	-0.002542
	-0.044233	0.020042	-0.003537	-0.004495	0.004607	-0.008910
	0.010277	-0.018575	-0.006521	-0.018501	0.007760	-0.016379
	-0.018906	-0.003253	-0.024968	-0.014112	-0.000400	-0.004153
	0.014826	0.001628	0.004624	-0.003257	-0.023363	-0.012131
	-0.014616					
y106	-0.020722	0.002059	-0.041435	0.008530	-0.028609	0.044871
	0.008541	-0.010933	-0.018884	-0.014480	0.001091	-0.018303
	-0.013407	0.006332	0.017011	-0.015301	0.030218	-0.026123
	-0.037413	-0.009301	-0.014460	0.004106	0.035346	0.000696
	-0.037126	-0.016776	-0.027336	0.020713	0.013830	0.016170
	0.017204					
z106	-0.010895	-0.011114	-0.014721	-0.023934	-0.040186	0.027043
	-0.013724	-0.004170	-0.004771	-0.038990	0.016364	0.000208
	0.017131	0.013723	0.005602	0.009514	-0.011764	-0.009043
	0.024918	-0.014502	-0.012949	0.013329	0.047578	0.013289
	0.001278	-0.015119	-0.003121	-0.004035	-0.006814	-0.032734
	-0.001801					

x107	-0.005958	-0.025885	-0.014484	-0.000829	0.021500	-0.003223
	-0.041600	0.025983	-0.000281	-0.007483	0.006411	-0.010875
	0.008633	-0.009448	-0.012715	-0.018338	0.011768	-0.019664
	-0.007764	0.008992	-0.031388	-0.009617	-0.004340	-0.003308
	0.020189	-0.006151	-0.001782	-0.000343	-0.018830	-0.007151
	-0.018358					
y107	-0.019327	0.002955	-0.042098	0.005971	-0.029958	0.045182
	0.008277	-0.010917	-0.019471	-0.018461	-0.001999	-0.018346
	-0.008707	0.008834	0.019410	-0.019614	0.032972	-0.029407
	-0.033040	-0.009108	-0.012175	0.007575	0.037631	0.003316
	-0.029991	-0.018507	-0.029593	0.017264	0.006215	0.019271
	0.022756					
z107	-0.005226	-0.013925	-0.009575	-0.021852	-0.035344	0.021035
	-0.010003	0.000692	0.009543	-0.035229	0.018472	-0.008967
	0.022147	0.015816	0.004670	0.007093	0.002418	-0.000993
	0.034511	-0.018617	-0.018428	0.012723	0.048993	0.019313
	0.010360	-0.020012	-0.012884	-0.000803	-0.025372	-0.027633
	-0.022847					
x108	-0.005146	-0.028596	-0.013641	0.001011	0.024151	-0.003140
	-0.039960	0.032861	0.000946	-0.012043	0.002286	-0.012200
	0.010806	-0.008320	-0.013308	-0.012632	0.012061	-0.014825
	-0.001085	0.016814	-0.032403	0.000031	-0.009491	0.000778
	0.024360	-0.003849	-0.006403	-0.007444	-0.018076	0.001557
	-0.011165					
y108	-0.017616	0.003267	-0.041296	0.003728	-0.029672	0.044543
	0.007858	-0.011643	-0.019798	-0.020015	-0.001706	-0.018904
	-0.006786	0.008991	0.020720	-0.022158	0.034223	-0.031017
	-0.026470	-0.009563	-0.008618	0.007774	0.040523	0.005719
	-0.023643	-0.019005	-0.030892	0.014596	0.001261	0.020227
	0.025719					
z108	-0.000124	-0.017157	-0.006069	-0.018989	-0.032291	0.016943
	-0.008344	0.003604	0.019873	-0.032086	0.021991	-0.015617
	0.023227	0.019056	0.002310	0.004511	0.011708	0.001546
	0.042104	-0.021723	-0.022451	0.011471	0.050198	0.022453
	0.020087	-0.025673	-0.020252	0.005341	-0.036630	-0.024653
	-0.041563					
x109	-0.005713	-0.030657	-0.014055	0.001030	0.024292	0.000671
	-0.038215	0.037448	0.002849	-0.015722	-0.001708	-0.011686
	0.014140	-0.011179	-0.013431	-0.007248	0.006781	-0.010319
	0.001201	0.017755	-0.027620	0.006820	-0.013196	0.013324
	0.027296	-0.007531	-0.004926	-0.014876	-0.006096	0.009610
	-0.000655					

y109	-0.015393	0.003637	-0.039624	0.002349	-0.028304	0.042908
	0.007870	-0.013019	-0.020661	-0.020038	0.000657	-0.019574
	-0.007594	0.007591	0.020731	-0.022632	0.034054	-0.031655
	-0.020697	-0.009056	-0.004440	0.006547	0.042573	0.006084
	-0.017924	-0.016445	-0.032042	0.011317	-0.004737	0.016298
	0.026587					
z109	0.004755	-0.019326	-0.002337	-0.016604	-0.032598	0.014306
	-0.005361	0.007811	0.025621	-0.030552	0.021654	-0.015838
	0.019636	0.019132	-0.000171	0.004885	0.014426	0.003984
	0.041695	-0.025508	-0.023228	0.010392	0.047450	0.026065
	0.024415	-0.024758	-0.024180	0.001757	-0.038308	-0.017953
	-0.048786					
x110	-0.006828	-0.030873	-0.016220	0.000102	0.025685	0.002309
	-0.038137	0.039020	-0.000787	-0.018675	-0.006218	-0.008189
	0.017212	-0.011411	-0.010485	-0.001237	0.001657	-0.007454
	-0.000215	0.021833	-0.024674	0.009281	-0.015458	0.014915
	0.024117	-0.003796	-0.011743	-0.019493	0.000721	0.005413
	0.007372					
y110	-0.013213	0.003218	-0.036969	0.001408	-0.025741	0.041301
	0.008673	-0.014700	-0.020163	-0.017939	0.004699	-0.021608
	-0.009007	0.003535	0.019511	-0.022858	0.032383	-0.030178
	-0.013846	-0.008556	0.000421	0.004090	0.045804	0.008975
	-0.012925	-0.014244	-0.031853	0.008862	-0.007042	0.008787
	0.026193					
z110	0.010175	-0.020189	0.002001	-0.014206	-0.035666	0.012021
	-0.000399	0.012801	0.027317	-0.030539	0.017803	-0.008674
	0.009994	0.016721	-0.002304	0.008257	0.011412	0.005798
	0.033019	-0.029450	-0.019244	0.008390	0.039163	0.029385
	0.025873	-0.016392	-0.027092	-0.012984	-0.032442	-0.008893
	-0.045675					
x111	-0.007592	-0.030960	-0.019449	0.001676	0.028696	0.004202
	-0.039244	0.035002	-0.005319	-0.021585	-0.009919	-0.005021
	0.019505	-0.012648	-0.008369	0.002201	-0.006142	-0.005799
	0.003135	0.027460	-0.019877	0.012935	-0.013991	0.013584
	0.026497	-0.004926	-0.007865	-0.022179	-0.001574	0.006749
	0.021369					
y111	-0.010903	0.002442	-0.033316	0.000264	-0.021673	0.038890
	0.010229	-0.016561	-0.019372	-0.014488	0.010429	-0.024666
	-0.011491	-0.002217	0.018308	-0.020775	0.030134	-0.026689
	-0.006377	-0.008229	0.005995	-0.000673	0.050182	0.011729
	-0.008050	-0.010086	-0.031615	0.007497	-0.004913	-0.004187
	0.025169					

z111	0.016170	-0.020899	0.006598	-0.009861	-0.038419	0.008989
	0.005525	0.015102	0.027036	-0.031542	0.014877	-0.000640
	-0.004893	0.013276	-0.004944	0.011948	0.003383	0.006519
	0.024617	-0.032869	-0.010442	0.005306	0.028756	0.032156
	0.031794	-0.007990	-0.022585	-0.030754	-0.025267	0.004202
	-0.033141					
x112	-0.010936	-0.030957	-0.024326	0.000480	0.033856	0.007547
	-0.040791	0.030742	-0.011472	-0.018054	-0.013786	-0.002037
	0.029529	-0.006780	-0.004246	0.002441	-0.008781	-0.002034
	0.011312	0.034761	-0.018470	0.015163	-0.008823	0.006161
	0.021820	-0.004688	-0.011313	-0.010721	-0.012645	-0.000845
	0.030355					
y112	-0.007205	0.002153	-0.028212	-0.000298	-0.016105	0.034934
	0.013171	-0.019671	-0.018871	-0.010477	0.018002	-0.027908
	-0.017980	-0.010973	0.014678	-0.016605	0.024827	-0.021902
	0.000117	-0.008017	0.013253	-0.006300	0.053700	0.014028
	-0.003321	-0.003781	-0.029635	0.003856	-0.000563	-0.020520
	0.022716					
z112	0.021322	-0.021584	0.011669	-0.005435	-0.038752	0.006140
	0.012285	0.019291	0.023777	-0.027899	0.012119	0.008717
	-0.018849	0.016060	-0.005561	0.014719	-0.000906	0.007710
	0.016686	-0.032976	-0.001187	-0.002891	0.015920	0.025460
	0.033805	0.005852	-0.023110	-0.043240	-0.023897	0.009068
	-0.018790					
x113	-0.014326	-0.030736	-0.031702	-0.000209	0.039749	0.012132
	-0.044107	0.025117	-0.015079	-0.013755	-0.014457	0.002871
	0.041892	-0.001640	-0.001847	0.001459	-0.017829	0.000861
	0.026530	0.046319	-0.020320	0.023599	0.002279	0.005402
	0.016282	-0.006405	-0.015093	0.010192	-0.021546	-0.005828
	0.037402					
y113	-0.003257	0.002877	-0.021569	-0.000209	-0.009309	0.030283
	0.016726	-0.022988	-0.018898	-0.004237	0.025665	-0.030492
	-0.026926	-0.021505	0.008814	-0.009328	0.018293	-0.016595
	0.006602	-0.007979	0.023219	-0.013929	0.053981	0.011220
	0.001341	0.006789	-0.028776	-0.001380	-0.001204	-0.039821
	0.019013					
z113	0.027466	-0.023303	0.011374	0.002034	-0.033670	0.007009
	0.015657	0.023266	0.024134	-0.027032	0.013614	0.019953
	-0.027570	0.019840	-0.006887	0.019010	-0.014265	0.007639
	0.017570	-0.020517	0.007184	-0.004736	0.006251	0.026841
	0.036026	0.010320	-0.022386	-0.040079	-0.014629	0.017958
	0.007085					

x114	0.017201	0.010216	0.010869	-0.019005	-0.028899	-0.005981
	-0.049742	-0.026636	-0.002276	-0.002615	0.023848	-0.006934
	-0.010333	-0.039538	0.015298	-0.017374	0.018142	0.014329
	-0.019747	-0.030229	0.030012	-0.032282	0.050747	0.014602
	-0.004057	-0.007172	-0.068326	-0.006465	0.040493	-0.067836
	0.022289					
y114	-0.028536	-0.013265	-0.032673	-0.013873	0.023745	0.012487
	0.000587	0.009412	-0.011936	-0.005135	0.003503	-0.046234
	-0.017688	0.019721	-0.017543	-0.006826	-0.040087	-0.004744
	-0.031400	-0.024476	-0.020560	0.002157	-0.015194	-0.028644
	0.014456	0.032891	-0.037859	0.024277	-0.041984	0.044243
	0.018992					
z114	-0.007661	-0.024620	-0.038957	-0.008210	-0.021479	0.013115
	-0.010467	-0.034645	-0.019586	-0.041363	0.030648	-0.010861
	-0.047063	0.017276	0.015590	0.018230	-0.017888	-0.048854
	-0.020210	-0.013856	-0.014085	0.034283	-0.017212	-0.031642
	0.044893	0.024018	-0.030103	-0.003635	-0.000591	-0.014397
	0.034464					
x115	0.021715	0.009822	0.004285	-0.013526	-0.023339	-0.010042
	-0.048433	-0.024993	-0.005907	-0.016793	0.027508	-0.004501
	-0.023015	-0.039975	0.020545	0.007366	0.012356	0.008125
	-0.020682	-0.017489	0.026270	-0.037247	0.028301	0.001675
	0.000001	-0.008977	-0.070009	-0.011296	0.031549	-0.047167
	0.024221					
y115	-0.024304	-0.014453	-0.029780	-0.010292	0.027865	0.013515
	-0.003656	0.019051	-0.012133	-0.007498	0.004102	-0.043866
	-0.010950	0.015588	-0.018577	-0.007836	-0.032442	-0.001275
	-0.022975	-0.016713	-0.019801	-0.002052	-0.006745	-0.026813
	-0.006234	0.022022	-0.010309	0.022538	-0.028181	0.045583
	0.022580					
z115	-0.001723	-0.015764	-0.031973	0.002914	-0.023757	0.009754
	-0.003476	-0.021502	-0.015669	-0.038854	0.014766	-0.005586
	-0.040355	-0.000420	0.007457	0.024521	-0.017451	-0.025175
	-0.018281	-0.012490	-0.009813	0.035158	-0.004073	-0.028047
	0.023306	0.014594	-0.016306	0.008524	-0.007577	-0.017038
	0.017232					
x116	0.024134	0.008508	-0.001052	-0.008019	-0.018370	-0.012238
	-0.048406	-0.023179	-0.007248	-0.027456	0.032472	-0.004833
	-0.030484	-0.041661	0.024844	0.025210	0.008798	0.003411
	-0.014387	-0.007596	0.024365	-0.040091	0.011373	-0.008978
	0.005027	-0.013427	-0.071847	-0.013828	0.027056	-0.033134
	0.023473					

y116	-0.022266	-0.016531	-0.030424	-0.005035	0.028937	0.022463
	-0.006612	0.027464	-0.010974	0.000847	0.011633	-0.043024
	-0.010079	0.004951	-0.013435	0.005390	-0.022776	-0.004672
	-0.015452	-0.019925	-0.029358	-0.004229	-0.015281	-0.029232
	-0.015721	0.018338	0.014613	0.024303	-0.030002	0.034206
	0.008367					
z116	0.005107	-0.008781	-0.027424	0.009062	-0.022619	0.001877
	0.002876	-0.011258	-0.007608	-0.044047	-0.006641	-0.006835
	-0.015978	-0.011839	-0.008809	0.017023	-0.017673	-0.005315
	-0.010613	-0.002510	0.006071	0.041029	0.020276	-0.022426
	0.005952	0.005231	-0.013052	0.016001	-0.007805	-0.012400
	0.012897					
x117	0.026170	0.007329	-0.006461	-0.002158	-0.013204	-0.013749
	-0.046686	-0.021703	-0.008073	-0.037111	0.035355	-0.005686
	-0.034447	-0.043121	0.027119	0.040507	0.003552	0.002412
	-0.008177	0.001062	0.018405	-0.040426	-0.003805	-0.015717
	0.006432	-0.018382	-0.069578	-0.016749	0.022721	-0.019713
	0.018945					
y117	-0.019225	-0.014127	-0.029737	0.001431	0.029099	0.027819
	-0.006998	0.033119	-0.013235	0.006544	0.009048	-0.041826
	-0.006389	-0.008124	-0.017560	0.004814	-0.025182	0.000228
	-0.004404	-0.015240	-0.036032	-0.006476	-0.016236	-0.025671
	-0.028367	0.009972	0.027394	0.019405	-0.022131	0.031395
	-0.000172					
z117	0.010581	-0.006687	-0.026015	0.014605	-0.020886	0.000694
	0.006283	-0.001927	0.002111	-0.046420	-0.016015	-0.009856
	0.003971	-0.020440	-0.018065	0.019373	-0.010965	0.005671
	-0.006708	-0.003652	0.012621	0.047568	0.034355	-0.019799
	-0.005689	0.002070	-0.001371	0.024658	-0.015219	-0.012682
	-0.000269					
x118	0.026999	0.005599	-0.011089	0.002647	-0.008272	-0.014529
	-0.045279	-0.020648	-0.008376	-0.044634	0.036290	-0.005493
	-0.035945	-0.042943	0.030399	0.051402	-0.001506	0.003537
	-0.002288	0.009179	0.013196	-0.037162	-0.014583	-0.020486
	0.009576	-0.022028	-0.063344	-0.018483	0.017549	-0.010109
	0.016454					
y118	-0.018494	-0.013709	-0.028032	0.002441	0.031409	0.029684
	-0.009536	0.039678	-0.011421	0.009076	0.008200	-0.041959
	-0.003771	-0.014977	-0.015621	0.006358	-0.024247	0.007850
	0.002521	-0.011337	-0.041641	-0.007740	-0.014809	-0.025518
	-0.018462	0.006200	0.025391	0.015958	-0.014442	0.027558
	0.005672					

z118	0.015286	-0.005202	-0.025952	0.020831	-0.020748	0.002265
	0.009540	0.005196	0.006938	-0.047675	-0.022251	-0.012941
	0.017399	-0.028741	-0.028081	0.022389	-0.008670	0.008582
	-0.002330	-0.007285	0.014397	0.051775	0.039742	-0.017275
	-0.021271	-0.000248	0.013346	0.029884	-0.022925	-0.014042
	-0.013553					
x119	0.026810	0.003581	-0.014474	0.005701	-0.004662	-0.015427
	-0.043850	-0.020063	-0.007960	-0.049797	0.035011	-0.003520
	-0.037595	-0.040807	0.034241	0.054882	-0.004470	0.006640
	0.003318	0.018089	0.012296	-0.031901	-0.020055	-0.023701
	0.013665	-0.026914	-0.058655	-0.022911	0.012958	-0.003926
	0.019736					
y119	-0.019112	-0.014467	-0.026576	0.000363	0.031462	0.029558
	-0.011319	0.045252	-0.011112	0.009540	0.005396	-0.038736
	-0.003643	-0.018775	-0.011533	0.002619	-0.019339	0.013080
	0.001280	-0.010179	-0.039678	-0.003549	-0.011811	-0.024437
	-0.011044	0.000006	0.011605	0.005465	-0.015104	0.020690
	0.017719					
z119	0.018191	-0.005085	-0.025078	0.025068	-0.021127	0.003054
	0.011025	0.012639	0.010569	-0.048293	-0.026928	-0.010331
	0.023997	-0.032974	-0.030742	0.022657	-0.007278	0.013400
	0.000775	-0.009148	0.012847	0.053125	0.042200	-0.016855
	-0.024064	-0.002283	0.016790	0.023084	-0.027097	-0.011208
	-0.018185					
x120	0.025968	0.001383	-0.017161	0.008283	-0.000824	-0.015447
	-0.041669	-0.019670	-0.006585	-0.052620	0.033238	-0.000954
	-0.038466	-0.037502	0.038326	0.052668	-0.007070	0.011074
	0.009345	0.027565	0.011964	-0.025347	-0.023815	-0.025255
	0.018153	-0.031312	-0.052523	-0.028787	0.009392	-0.000190
	0.022453					
y120	-0.020233	-0.015220	-0.026361	-0.001993	0.032570	0.030479
	-0.011894	0.047517	-0.011386	0.008632	0.005123	-0.039099
	-0.003472	-0.018678	-0.008240	-0.001374	-0.017926	0.016572
	-0.000877	-0.011028	-0.035884	0.004130	-0.013595	-0.017239
	-0.006449	-0.002739	0.007126	-0.006960	-0.020451	0.011357
	0.020823					
z120	0.019262	-0.006360	-0.022738	0.026969	-0.022927	0.002084
	0.012901	0.021857	0.013225	-0.049075	-0.031413	-0.001526
	0.024118	-0.032829	-0.026963	0.017797	-0.004625	0.019590
	0.002382	-0.009382	0.009911	0.052759	0.042178	-0.017064
	-0.017767	-0.005499	0.009727	0.002855	-0.029485	-0.003617
	-0.014366					

x121	0.024142	-0.001464	-0.019426	0.011507	0.003736	-0.013288
	-0.038209	-0.018979	-0.004145	-0.051136	0.029614	0.001128
	-0.035816	-0.033056	0.041406	0.044445	-0.009182	0.017968
	0.017702	0.036681	0.009227	-0.018057	-0.028156	-0.025523
	0.019881	-0.035198	-0.041318	-0.029717	0.007946	0.000326
	0.022253					
y121	-0.021670	-0.017542	-0.029122	-0.004483	0.033510	0.033394
	-0.012970	0.046171	-0.012957	0.012988	0.006756	-0.045503
	-0.000428	-0.014646	-0.007166	-0.000079	-0.017789	0.016231
	-0.004542	-0.009866	-0.027265	-0.003091	-0.023102	-0.009152
	-0.004007	-0.000826	0.002890	-0.001715	-0.020259	0.005305
	0.015063					
z121	0.018397	-0.010682	-0.019644	0.029060	-0.025334	0.001482
	0.013737	0.032441	0.015730	-0.045737	-0.032998	0.008132
	0.022045	-0.030592	-0.018941	0.015752	-0.002563	0.023105
	0.005761	-0.007173	0.004279	0.043538	0.037795	-0.016295
	-0.009703	-0.004522	0.004461	-0.012930	-0.023358	0.004278
	-0.008458					
x122	0.021139	-0.004440	-0.020674	0.013392	0.008899	-0.009387
	-0.033225	-0.018469	0.000161	-0.044608	0.024091	0.002448
	-0.028767	-0.025339	0.042076	0.029840	-0.008233	0.027762
	0.029849	0.044171	0.005257	-0.006869	-0.030272	-0.020433
	0.016241	-0.037476	-0.028770	-0.024302	0.007983	-0.002476
	0.020860					
y122	-0.022746	-0.018852	-0.033095	-0.009242	0.036946	0.039370
	-0.012539	0.038557	-0.012078	0.021112	0.011580	-0.059527
	0.003762	-0.007090	-0.010143	0.003705	-0.016459	0.011751
	-0.007341	-0.013589	-0.012273	-0.009825	-0.037088	0.005594
	-0.002225	0.005583	-0.009780	0.014553	-0.019001	0.001057
	0.006057					
z122	0.016116	-0.017583	-0.014765	0.030334	-0.026048	0.002928
	0.015866	0.041672	0.022033	-0.034878	-0.031103	0.013397
	0.017553	-0.026237	-0.009044	0.013972	0.001044	0.020472
	0.013954	-0.011208	-0.003170	0.034772	0.027962	-0.009705
	-0.000720	0.001575	-0.006298	-0.021772	-0.011272	0.013869
	-0.001490					
x123	0.018805	-0.006082	-0.020012	0.012815	0.012738	-0.005912
	-0.029141	-0.019742	0.003028	-0.036269	0.019172	0.004774
	-0.022225	-0.014656	0.039678	0.015815	-0.005109	0.038216
	0.039758	0.051380	0.005105	0.008537	-0.029862	-0.012055
	0.012844	-0.035812	-0.013607	-0.015847	0.001129	-0.011557
	0.018996					

y123	-0.021496	-0.017908	-0.037927	-0.015780	0.041513	0.046181
	-0.011657	0.028490	-0.011998	0.027511	0.018436	-0.073674
	0.005228	0.001885	-0.013469	0.011761	-0.014609	0.008460
	-0.014820	-0.013652	0.007461	-0.015181	-0.048362	0.022413
	0.000814	0.012227	-0.024293	0.031511	-0.013524	-0.004406
	0.005792					
z123	0.019562	-0.021849	-0.014183	0.032263	-0.023143	0.004090
	0.018921	0.044968	0.023438	-0.023367	-0.027145	0.014464
	0.002650	-0.015017	-0.005231	0.022137	0.000113	0.008211
	0.011512	-0.012227	0.016626	0.028076	0.007143	-0.014009
	0.014533	0.015776	-0.014725	-0.011818	-0.009534	0.009299
	0.021556					
x124	-0.036846	-0.033852	-0.016521	-0.002765	0.010545	-0.104429
	0.080015	0.033866	-0.050240	-0.000751	-0.017157	-0.021300
	-0.023842	-0.032428	0.039587	-0.027955	-0.045037	-0.036421
	-0.046289	-0.027134	0.046981	-0.073618	-0.099918	-0.015295
	0.110478	0.031580	0.059085	-0.003150	-0.114123	-0.017386
	-0.018212					
y124	0.027193	-0.006729	-0.047311	-0.055041	-0.042655	-0.029938
	0.026990	0.031592	-0.049651	0.015078	0.120227	0.004710
	0.029480	0.061688	0.000718	0.031072	-0.031323	0.036110
	-0.081832	0.163447	0.039447	-0.186887	0.023299	0.051520
	-0.062464	-0.156006	0.010386	0.057842	0.156676	0.005683
	-0.052959					
z124	-0.070223	0.001471	0.027475	-0.099437	-0.024568	0.031340
	0.018807	0.097214	-0.002776	-0.005394	0.076394	0.085852
	-0.019981	0.012003	0.014172	0.039513	0.081363	0.075296
	0.007862	-0.025842	-0.120034	0.151826	-0.031173	-0.111217
	-0.044656	-0.006268	-0.056548	-0.086947	0.164181	0.027456
	0.012920					
x125	-0.017781	-0.025073	-0.020224	-0.000520	0.024398	-0.085035
	0.060123	0.037224	-0.016893	0.026274	-0.050000	-0.040717
	-0.034514	-0.000300	0.002672	-0.009278	-0.024128	-0.060942
	-0.025563	-0.041103	0.079883	-0.043436	-0.064107	-0.019751
	0.054142	0.006718	0.026886	-0.061520	-0.091181	-0.029494
	-0.011744					
y125	0.027980	-0.007591	-0.036682	-0.062705	-0.056770	-0.025335
	0.044108	0.014378	-0.038758	-0.007455	0.106142	0.012801
	0.067393	-0.003594	0.050980	-0.008663	-0.035568	0.061661
	-0.097538	0.175617	-0.018798	-0.135629	0.014525	0.009989
	0.003181	-0.066222	0.023848	0.012821	0.160803	-0.022524
	-0.007009					

z125	-0.064669	-0.004813	0.025256	-0.081394	-0.037970	0.021911
	0.031637	0.097966	-0.036172	0.027562	0.061283	0.056645
	-0.018961	0.014938	0.017132	0.073704	0.061974	0.080076
	0.008914	-0.023348	-0.094213	0.126163	0.028668	-0.035807
	-0.042360	-0.039989	-0.026241	-0.059250	0.123043	0.013175
	-0.024857					
x126	-0.015112	-0.020713	-0.020698	-0.009978	-0.001428	-0.072968
	0.041561	0.039433	-0.004945	0.004676	-0.040287	-0.010644
	-0.029048	-0.019868	-0.011004	-0.025312	-0.017301	-0.051985
	-0.034331	0.004692	0.025569	-0.059985	-0.022025	-0.055991
	0.076868	-0.002422	0.031237	-0.069488	-0.062107	0.000935
	-0.000977					
y126	0.024224	-0.007440	-0.036013	-0.059643	-0.062121	-0.042997
	0.058747	0.009122	0.003349	-0.023643	0.062564	-0.010145
	0.104829	-0.021060	0.105352	0.022169	-0.061161	0.091010
	-0.122819	0.170326	-0.047765	-0.083802	-0.007899	-0.001874
	0.021029	0.017165	0.014943	0.014485	0.118762	-0.028923
	0.024397					
z126	-0.049845	-0.016415	0.022572	-0.061597	-0.054056	0.008959
	0.032882	0.101540	-0.080838	0.071515	0.067965	0.023103
	-0.018538	-0.001472	0.023502	0.086258	0.045084	0.078831
	0.026989	-0.033253	-0.057677	0.063667	0.072882	0.016044
	-0.068963	-0.089256	0.020859	-0.034522	0.054666	0.031236
	-0.041081					
x127	-0.005184	-0.011504	-0.018245	-0.005639	-0.005991	-0.064339
	0.026915	0.038662	0.024879	0.013128	-0.057638	-0.015131
	-0.036154	-0.012314	-0.024552	-0.019931	-0.009772	-0.075477
	-0.029749	0.018128	0.023025	-0.042985	0.017458	-0.070173
	0.070692	-0.026146	0.020441	-0.088984	-0.047563	0.014554
	0.000632					
y127	0.033602	-0.005325	-0.041475	-0.044174	-0.065006	-0.064620
	0.076518	0.020049	0.044440	-0.015277	0.012205	-0.064528
	0.136032	-0.025466	0.119383	0.073093	-0.079747	0.058941
	-0.118126	0.151153	-0.023287	-0.040465	-0.000320	-0.029812
	0.011276	0.005900	0.041258	0.017313	0.039307	-0.017764
	0.040589					
z127	-0.052405	-0.015259	0.022144	-0.055460	-0.050763	0.023141
	0.033641	0.084924	-0.130552	0.093002	0.074296	0.041483
	0.000427	0.018698	0.054589	0.072454	0.030962	0.115004
	0.052000	-0.027715	-0.073574	0.045588	0.061899	0.048360
	-0.079054	-0.059271	0.019789	-0.034479	0.031814	0.045773
	0.033394					

x128	0.002170	-0.003751	-0.020599	-0.008286	-0.004995	-0.054601
	0.015316	0.042812	0.039842	0.022331	-0.072527	-0.003412
	-0.028457	-0.017156	-0.031755	-0.015032	-0.002023	-0.099510
	-0.035404	0.042949	0.002193	-0.035825	0.043471	-0.070461
	0.056292	-0.050272	-0.008037	-0.091675	-0.066599	0.056713
	0.006940					
y128	0.027839	-0.001690	-0.035043	-0.028960	-0.054292	-0.057033
	0.065497	0.019899	0.031465	0.000621	0.013077	-0.041887
	0.137695	-0.009001	0.100950	0.052687	-0.074060	0.029794
	-0.079252	0.116770	-0.006409	-0.023913	0.047063	0.005149
	-0.019985	-0.043926	-0.000672	0.031400	-0.008461	-0.027168
	0.085242					
z128	-0.044261	-0.019603	0.032751	-0.029252	-0.052423	0.019164
	0.047559	0.067389	-0.150774	0.107436	0.056503	0.010019
	0.001278	0.005081	0.075904	0.039860	0.044587	0.123365
	0.089016	-0.041199	-0.091386	0.026987	0.068705	0.023415
	-0.045096	-0.035340	0.090198	-0.001797	-0.020709	0.014061
	0.038863					
x129	-0.001397	-0.003884	-0.018266	0.002455	-0.011273	-0.034411
	0.003648	0.049773	0.011028	0.028586	-0.045913	0.017122
	-0.040198	-0.026576	-0.042902	-0.042285	0.021547	-0.086106
	0.009491	0.079807	-0.004215	-0.035643	0.054396	-0.073790
	0.013874	-0.021103	0.002830	-0.130788	0.029357	0.052923
	0.001810					
y129	0.019262	-0.003068	-0.028962	-0.014118	-0.054082	-0.046352
	0.059856	0.023965	0.028548	0.013201	0.017667	-0.038831
	0.119522	-0.014178	0.094637	0.034300	-0.057023	0.038719
	-0.057518	0.088387	-0.025752	0.010975	0.053814	-0.000804
	-0.029715	-0.027408	0.001383	0.013449	-0.001687	-0.022623
	0.072400					
z129	-0.043342	-0.021478	0.036099	-0.014621	-0.049713	0.015067
	0.052892	0.061150	-0.131232	0.111542	0.042674	-0.006174
	0.002918	0.000057	0.079870	0.037522	0.034010	0.096983
	0.081657	-0.055523	-0.044028	0.023276	0.062433	0.025832
	-0.019447	0.002560	0.084946	0.025897	-0.042382	-0.032340
	0.022751					
x130	0.003542	-0.012445	-0.019955	-0.003259	-0.018362	-0.017563
	0.009580	0.067953	-0.015789	0.039126	-0.021642	0.021313
	-0.066154	-0.013491	-0.058539	-0.064509	0.013312	-0.066036
	0.010269	0.064262	-0.028654	0.013362	0.064096	-0.043698
	-0.030731	0.019365	0.002176	-0.105815	0.121100	0.026428
	0.076103					

y130	0.012346	-0.006965	-0.022021	-0.001952	-0.054413	-0.028536
	0.045571	0.029954	0.031592	0.018764	0.029748	-0.050429
	0.093762	-0.015376	0.083439	0.024667	-0.053679	0.056210
	-0.028140	0.057464	-0.032752	0.013475	0.034371	0.007973
	-0.004095	0.003287	-0.003889	0.002715	0.000068	-0.048746
	0.054074					
z130	-0.045683	-0.023550	0.034052	-0.006761	-0.057190	0.009215
	0.043700	0.056181	-0.106025	0.114103	0.033209	-0.019142
	-0.005480	-0.007298	0.092887	0.058986	0.023136	0.057907
	0.047911	-0.024615	0.000114	0.002478	0.052833	0.040374
	-0.001656	0.029489	0.054485	0.049091	-0.069553	-0.030760
	-0.012181					
x131	0.012438	-0.020942	-0.012230	-0.010663	-0.028107	-0.005486
	0.013940	0.054037	-0.010384	0.044933	-0.011905	0.012956
	-0.048513	-0.010036	-0.044393	-0.040509	0.017412	-0.022347
	0.032878	0.025688	-0.047773	0.035244	0.038379	-0.017038
	-0.049801	-0.019544	0.002403	-0.055567	0.102317	-0.004482
	0.114984					
y131	0.004618	-0.009930	-0.012107	0.007698	-0.050146	-0.013318
	0.036470	0.031134	0.034326	0.024220	0.034897	-0.053600
	0.063478	-0.008159	0.060481	0.016203	-0.050303	0.062017
	-0.010310	0.021628	-0.050775	0.016709	0.021992	0.024391
	0.003803	0.025191	-0.001114	0.035959	-0.026318	-0.039307
	0.055079					
z131	-0.047270	-0.020741	0.035473	-0.000832	-0.053118	0.005736
	0.043778	0.053337	-0.090726	0.108361	0.010564	-0.017777
	-0.008592	-0.006987	0.081222	0.061221	0.009880	0.010866
	0.029120	0.002752	0.028621	-0.014190	0.031977	0.037673
	0.022662	0.061147	0.031113	0.049605	-0.068156	-0.038356
	-0.028222					
x132	0.023342	-0.019315	-0.007972	-0.017910	-0.034059	-0.006140
	0.024227	0.033375	-0.004420	0.050082	-0.009210	-0.014217
	-0.017061	0.001179	-0.039524	-0.033512	0.033019	-0.007067
	0.034394	-0.022210	-0.050882	0.018746	0.025871	-0.029755
	-0.055294	-0.037659	0.045040	-0.021390	0.061055	-0.049100
	0.082699					
y132	0.004737	-0.007418	-0.008920	0.014955	-0.040991	-0.011822
	0.031766	0.027530	0.039476	0.020872	0.033006	-0.048987
	0.050180	-0.001671	0.041940	-0.001354	-0.039162	0.054117
	0.002765	0.000944	-0.050426	0.017369	0.018249	0.018730
	-0.010063	0.008657	0.017733	0.045572	-0.034288	-0.085348
	0.029218					

z132	-0.043457	-0.016994	0.039131	0.004971	-0.040562	0.010927
	0.048067	0.039116	-0.082191	0.094746	-0.011823	0.009623
	-0.006819	-0.002128	0.057865	0.060809	-0.001007	-0.010604
	0.038191	0.049568	0.031694	0.000606	0.022891	0.011457
	0.046839	0.056166	-0.017127	0.049125	-0.033953	-0.037920
	0.017180					
x133	0.029259	-0.012306	-0.002834	-0.020153	-0.027624	-0.005179
	0.030042	0.013645	0.003721	0.061437	-0.022026	-0.011622
	-0.011324	0.012055	-0.029691	-0.026857	0.020616	-0.013226
	0.031117	-0.008581	-0.042658	0.021134	-0.001262	-0.059828
	-0.043498	-0.044814	0.008206	-0.010563	0.054799	-0.055852
	0.076793					
y133	0.004131	0.000288	-0.005496	0.023055	-0.031418	-0.011084
	0.023317	0.020658	0.043043	0.016834	0.021449	-0.020864
	0.032445	-0.001044	0.026266	-0.014818	-0.041119	0.037567
	0.004434	0.015232	-0.044177	0.017642	-0.018625	-0.013118
	-0.006427	0.004097	-0.036403	0.047594	-0.024828	-0.084288
	0.029400					
z133	-0.035505	-0.018068	0.032335	0.012716	-0.034041	0.018287
	0.046343	0.034064	-0.053910	0.092624	-0.029261	0.018813
	0.003158	0.010064	0.014214	0.058512	0.007539	-0.027741
	0.047960	0.054598	0.051463	-0.003399	-0.000334	-0.018704
	0.025926	0.009924	-0.035518	0.048670	-0.039532	-0.012352
	0.016988					
x134	0.033228	-0.009530	-0.005696	-0.025324	-0.026679	0.000939
	0.026101	0.018031	0.003693	0.051982	-0.021721	-0.006359
	-0.005620	0.008890	-0.040341	-0.023472	0.022967	-0.016597
	0.033179	-0.019042	-0.024918	0.014940	-0.007970	-0.052272
	-0.036261	-0.060048	-0.015409	-0.015086	0.026835	-0.015638
	0.077251					
y134	0.006881	-0.000460	-0.009326	0.024546	-0.025526	-0.002767
	0.013845	0.032602	0.043975	0.008270	0.018771	-0.003705
	0.024725	-0.011786	-0.005933	-0.022208	-0.019649	0.026136
	0.008331	0.001908	-0.024089	0.009353	-0.027624	-0.000220
	-0.006138	-0.013265	-0.062250	0.046511	-0.039085	-0.044689
	0.034690					
z134	-0.035799	-0.018498	0.029211	0.007485	-0.037503	0.018338
	0.037514	0.020159	-0.039217	0.077616	-0.033583	0.021851
	0.030199	0.011542	0.000986	0.069145	-0.000804	-0.046049
	0.036070	0.062952	0.025993	-0.009258	-0.022067	-0.034346
	0.012516	-0.004692	-0.021417	0.023468	-0.038814	0.000266
	-0.009322					

x135	0.035642	-0.006170	-0.005177	-0.033241	-0.022926	0.002903
	0.028443	0.013467	0.008753	0.044539	-0.031579	-0.000717
	-0.007111	0.014076	-0.041367	-0.015352	0.016375	-0.024335
	0.036334	-0.007425	-0.030446	0.002935	-0.025134	-0.035273
	-0.033356	-0.044519	-0.046442	-0.002585	0.023372	-0.018768
	0.069855					
y135	0.008522	0.000159	-0.008577	0.021188	-0.017760	-0.000395
	0.006800	0.029553	0.049549	0.004827	0.007777	0.003764
	0.014373	-0.006664	-0.018779	-0.021491	-0.016155	0.013343
	0.016509	0.015473	-0.028401	-0.005742	-0.052501	0.012047
	-0.021815	-0.003586	-0.095208	0.043717	-0.032107	-0.047246
	0.025891					
z135	-0.035990	-0.017586	0.021870	0.006148	-0.034426	0.023155
	0.033041	0.012795	-0.035660	0.050217	-0.037820	0.033149
	0.030429	0.012817	0.003554	0.070023	-0.002054	-0.050846
	0.028162	0.069496	0.011649	-0.002572	-0.010732	-0.040799
	0.029758	0.001111	-0.009732	0.014302	-0.032987	-0.002195
	0.000294					
x136	0.036971	-0.002709	-0.001013	-0.035557	-0.022296	0.004405
	0.030335	0.010706	0.011050	0.036631	-0.035923	0.001909
	-0.010539	0.011921	-0.030265	-0.004633	0.010010	-0.026738
	0.038521	0.006864	-0.045156	0.001138	-0.032130	-0.017154
	-0.025014	-0.021329	-0.052997	0.015017	0.022385	-0.018840
	0.061334					
y136	0.012042	0.000592	-0.006581	0.022388	-0.014956	0.000473
	-0.000229	0.030365	0.051719	0.000671	0.002022	0.004168
	0.001451	-0.011351	-0.020215	-0.015616	-0.009935	0.004642
	0.024723	0.031956	-0.047737	-0.009597	-0.063599	0.026204
	-0.025137	0.008473	-0.096470	0.057427	-0.027870	-0.041410
	0.022060					
z136	-0.035285	-0.016704	0.015091	0.001393	-0.028424	0.027922
	0.031354	0.004947	-0.033365	0.021240	-0.044420	0.038791
	0.027282	0.021636	0.003378	0.061600	0.001137	-0.051655
	0.026687	0.065732	0.001643	-0.009677	0.000804	-0.041417
	0.032391	0.013344	-0.019801	0.004042	-0.028913	-0.018244
	0.005169					
x137	0.035459	-0.000999	0.003414	-0.035413	-0.019620	0.010213
	0.033360	0.010196	0.008686	0.026926	-0.033659	0.005591
	-0.013751	0.011919	-0.019573	0.003646	0.008908	-0.025703
	0.039154	0.014474	-0.042237	-0.000650	-0.023775	-0.010202
	-0.025895	-0.006080	-0.057214	0.019556	0.022923	-0.013024
	0.060178					

y137	0.014829	-0.001877	-0.007333	0.022540	-0.013573	0.009216
	-0.002803	0.031138	0.049336	-0.005312	-0.001214	0.006732
	-0.006390	-0.005638	-0.017864	-0.012471	0.003859	0.005121
	0.036525	0.040699	-0.043012	-0.016872	-0.053432	0.025369
	-0.033388	0.015029	-0.095129	0.048774	-0.025511	-0.024115
	0.034675					
z137	-0.033738	-0.013256	0.010719	-0.002699	-0.021945	0.027625
	0.029875	0.000574	-0.030796	0.000798	-0.049825	0.041755
	0.020766	0.016754	0.004079	0.054790	0.006224	-0.059847
	0.020185	0.063628	-0.018845	-0.008636	-0.004588	-0.030503
	0.032071	0.028268	-0.033365	0.004897	-0.023585	-0.029907
	0.001799					
x138	0.033433	0.000607	0.009205	-0.035377	-0.014910	0.016060
	0.035315	0.008068	0.004631	0.017109	-0.029507	0.008275
	-0.012697	0.012744	-0.015565	0.008232	0.009307	-0.022774
	0.028039	0.011894	-0.031549	-0.001109	-0.015596	-0.004728
	-0.025541	0.004672	-0.062531	0.004822	0.016919	-0.017574
	0.060601					
y138	0.019822	-0.005184	-0.008574	0.018446	-0.010984	0.018293
	-0.004942	0.023591	0.045765	-0.010276	-0.003983	0.010824
	-0.008556	0.005645	-0.022497	-0.016319	0.017938	0.002822
	0.033691	0.034901	-0.031699	-0.020942	-0.042226	0.016596
	-0.040826	0.017931	-0.096686	-0.000791	-0.030112	-0.015085
	0.041281					
z138	-0.033224	-0.010129	0.006578	-0.004129	-0.014985	0.025706
	0.027461	0.005978	-0.025155	-0.014097	-0.054354	0.043422
	0.011862	0.004542	0.007847	0.057293	0.011182	-0.069314
	0.030555	0.075700	-0.037012	-0.010176	-0.007402	-0.018131
	0.022302	0.038426	-0.039201	0.033060	-0.011698	-0.023879
	0.006490					
x139	0.029749	0.002777	0.015855	-0.032975	-0.008343	0.021328
	0.034937	0.008137	0.001420	0.008109	-0.025098	0.012948
	-0.014488	0.011006	-0.009210	0.012384	0.006522	-0.017517
	0.018020	0.011453	-0.023981	0.002859	-0.010352	-0.002624
	-0.023108	0.014976	-0.059970	0.005119	0.017179	-0.014240
	0.052858					
y139	0.025366	-0.006546	-0.008495	0.015171	-0.007794	0.027077
	-0.010782	0.018757	0.045384	-0.019259	-0.010347	0.018605
	-0.014087	0.009836	-0.020985	-0.015444	0.029584	-0.004337
	0.036368	0.038998	-0.029836	-0.018637	-0.041262	0.009315
	-0.038633	0.018932	-0.084249	-0.016153	-0.035828	-0.010866
	0.030835					

z139	-0.032165	-0.006868	0.001830	-0.007925	-0.008747	0.021715
	0.027371	0.007306	-0.023192	-0.019955	-0.051384	0.037596
	0.010240	-0.004350	0.009896	0.050104	0.016647	-0.068593
	0.036905	0.073152	-0.045710	-0.014497	-0.000642	-0.002861
	0.012728	0.052707	-0.055141	0.039602	-0.006844	-0.013196
	0.007673					
x140	0.024177	0.005482	0.023150	-0.028870	-0.000889	0.025366
	0.032978	0.009309	-0.002336	0.001057	-0.018890	0.016125
	-0.016279	0.006735	-0.001382	0.012859	0.001123	-0.011656
	0.008398	0.008923	-0.017096	0.007026	-0.003926	-0.000783
	-0.022405	0.026681	-0.055739	0.008891	0.012892	-0.007472
	0.050611					
y140	0.027667	-0.005689	-0.006531	0.011181	-0.007672	0.033199
	-0.012421	0.014935	0.049921	-0.020475	-0.011349	0.018726
	-0.014315	0.010451	-0.015039	-0.021478	0.034931	-0.001248
	0.047024	0.039934	-0.036985	-0.029891	-0.028693	0.007780
	-0.036763	0.031946	-0.067728	0.003349	-0.049211	-0.009305
	0.034673					
z140	-0.029954	-0.005108	-0.001431	-0.011360	-0.000988	0.019850
	0.023049	0.012810	-0.025995	-0.029632	-0.048849	0.033734
	0.007999	-0.019377	0.009730	0.048576	0.025665	-0.075813
	0.036701	0.070884	-0.043775	-0.004560	-0.001801	0.008254
	0.005958	0.055242	-0.061285	0.029701	-0.011133	0.010669
	0.009864					
x141	0.017757	0.007925	0.029852	-0.024831	0.006360	0.027563
	0.031078	0.012424	-0.006955	-0.003209	-0.011368	0.017938
	-0.018256	0.003215	0.004243	0.013784	-0.004936	-0.004410
	-0.000521	0.004207	-0.008503	0.009164	0.001555	0.001197
	-0.025420	0.034541	-0.050814	0.009303	0.010989	0.000321
	0.053477					
y141	0.027837	-0.007115	-0.003450	0.007322	-0.004939	0.037316
	-0.015873	0.016664	0.048668	-0.016335	-0.010467	0.022969
	-0.012145	-0.002827	-0.002269	-0.012848	0.038514	-0.000938
	0.047387	0.042774	-0.031340	-0.034663	-0.019097	0.008481
	-0.034174	0.028466	-0.065594	-0.002310	-0.050796	-0.000975
	0.059762					
z141	-0.027397	-0.002496	-0.004466	-0.015319	0.002765	0.016140
	0.022059	0.013912	-0.024406	-0.034721	-0.042643	0.026334
	0.009145	-0.027328	0.002238	0.040691	0.035274	-0.069735
	0.038655	0.057604	-0.041446	-0.009258	-0.000090	0.013874
	-0.005957	0.063011	-0.064824	0.040304	-0.019076	0.033715
	0.012710					

x142	0.011406	0.009320	0.035674	-0.019156	0.012173	0.028564
	0.030630	0.015164	-0.011687	-0.000546	-0.005720	0.019392
	-0.018051	0.000643	0.007326	0.011991	-0.010742	-0.001595
	-0.006906	0.001742	0.000244	0.008616	0.008196	-0.004622
	-0.016998	0.038794	-0.038996	0.011836	0.004309	0.001673
	0.047020					
y142	0.028278	-0.011582	-0.001368	0.005737	-0.008228	0.035310
	-0.015506	0.012094	0.042836	-0.010270	-0.007429	0.027906
	-0.007611	-0.006756	0.003461	-0.014858	0.048977	-0.011793
	0.037128	0.050569	-0.020287	-0.033497	-0.017983	-0.009475
	-0.023799	0.020813	-0.058189	0.001038	-0.065482	-0.011424
	0.045283					
z142	-0.025418	0.000329	-0.003106	-0.015116	0.006395	0.014262
	0.021806	0.015015	-0.022784	-0.019735	-0.037298	0.021857
	0.016168	-0.043786	-0.007878	0.034626	0.034893	-0.065104
	0.052339	0.044776	-0.043693	-0.025396	0.000543	0.012103
	0.000987	0.058053	-0.043189	0.050018	-0.036654	0.060635
	0.002714					
x143	0.000682	0.013532	0.038929	-0.011825	0.016702	0.027979
	0.028224	0.019498	-0.018738	0.001389	-0.003196	0.016795
	-0.016860	0.004233	0.010849	0.010228	-0.020269	-0.000722
	-0.016958	0.003277	0.013128	0.006172	0.010830	-0.013084
	-0.010679	0.040907	-0.028232	0.009210	0.004604	0.001581
	0.026690					
y143	0.027194	-0.015996	0.007762	0.005830	-0.005068	0.037942
	-0.013724	0.003075	0.037858	-0.007993	-0.006906	0.033190
	-0.007320	-0.010942	0.016066	-0.013447	0.042015	-0.004271
	0.025854	0.049996	0.002032	-0.027467	-0.002933	-0.008047
	-0.016581	-0.000924	-0.015523	0.001821	-0.054336	-0.022959
	0.057716					
z143	-0.027657	0.004969	0.000908	-0.026329	0.006767	0.009466
	0.015308	0.016416	-0.019945	-0.008171	-0.035286	0.020766
	0.015659	-0.055697	0.002925	0.028001	0.032612	-0.051882
	0.044396	0.037662	-0.032358	-0.048046	0.005775	-0.005685
	-0.017050	0.033008	-0.041111	0.042841	-0.039825	0.068724
	-0.036840					
x144	-0.006477	0.017208	0.036201	-0.007944	0.017849	0.023627
	0.027143	0.022711	-0.022257	0.003902	-0.004534	0.008697
	-0.015513	0.014503	0.008051	0.009752	-0.026048	-0.001768
	-0.015878	0.007997	0.019436	0.004482	0.009777	-0.017016
	-0.008011	0.043160	-0.016854	0.005303	0.011334	0.003302
	0.014845					

y144	0.026745	-0.018356	0.014263	0.007671	-0.008064	0.042782
	-0.015420	0.005378	0.033775	-0.007279	-0.002627	0.033454
	-0.003525	-0.018251	0.026357	-0.016655	0.038959	0.006487
	0.011488	0.042863	0.006928	-0.021599	-0.005815	-0.013878
	-0.009285	-0.015305	-0.006567	-0.004715	-0.072523	-0.046827
	0.033087					
z144	-0.030180	0.009020	0.008606	-0.036894	0.010498	0.006687
	0.011429	0.019154	-0.014946	-0.003707	-0.032945	0.023324
	0.008591	-0.054312	0.006820	0.018986	0.031091	-0.022838
	0.050942	0.030165	-0.024220	-0.049201	0.028490	-0.013721
	-0.034853	0.029331	-0.024658	0.053324	-0.020420	0.070682
	-0.017248					
x145	-0.010593	0.018366	0.028942	-0.006559	0.019002	0.017580
	0.026455	0.023010	-0.025039	0.005368	-0.002441	-0.004283
	-0.015005	0.023165	0.006010	0.006144	-0.032393	-0.013831
	-0.018717	0.013349	0.020863	0.007250	-0.009057	-0.018581
	-0.003169	0.045918	-0.018124	-0.012833	0.010104	0.003429
	-0.020513					
y145	0.021481	-0.018964	0.019440	0.007437	-0.016184	0.042968
	-0.017751	0.010046	0.028988	-0.002891	-0.006368	0.035480
	-0.001371	-0.011340	0.034005	-0.004919	0.049247	0.016225
	0.016705	0.035783	0.006882	-0.018161	0.015511	-0.018913
	0.006521	-0.017828	0.007358	-0.001303	-0.073548	-0.057391
	0.063369					
z145	-0.024811	0.014461	0.012429	-0.036235	0.011250	0.000152
	0.009399	0.017586	-0.014825	-0.010779	-0.029867	0.025329
	0.010980	-0.057925	-0.006939	-0.022847	0.016190	0.009172
	0.051687	0.037573	-0.013403	-0.057075	0.034798	-0.007997
	-0.059536	0.016220	-0.025140	0.010919	-0.018173	0.075759
	-0.065077					
x146	-0.015706	0.021732	0.022863	-0.005680	0.017254	0.010313
	0.026619	0.023480	-0.030826	0.011962	-0.006974	-0.015211
	-0.009875	0.031079	0.001285	0.014443	-0.032359	-0.022274
	-0.020025	0.019868	0.016056	0.001355	-0.008246	-0.016818
	-0.009471	0.038806	-0.022397	-0.017674	0.013456	-0.003611
	-0.037581					
y146	0.019737	-0.020287	0.019151	0.010141	-0.018401	0.042164
	-0.020274	0.012156	0.032234	-0.005853	-0.006073	0.035897
	-0.007669	-0.009064	0.036129	-0.011785	0.046803	0.027268
	0.019565	0.027068	0.011090	-0.007268	0.007192	-0.025326
	0.028755	-0.008063	0.029599	0.000630	-0.066420	-0.044340
	0.064504					

z146	-0.031408	0.017215	0.017492	-0.045968	0.013776	0.003256
	0.005240	0.013874	-0.012688	-0.002375	-0.019095	0.024920
	0.005828	-0.058955	0.005129	-0.021695	0.017415	0.013671
	0.044056	0.022558	-0.007068	-0.052162	0.026823	-0.016345
	-0.062870	0.013816	-0.032463	0.006314	-0.009100	0.051824
	-0.068574					
x147	-0.019191	0.022120	0.016221	-0.003808	0.016062	0.003585
	0.026790	0.024787	-0.032586	0.010030	-0.010341	-0.021672
	-0.009830	0.037443	-0.003733	0.020364	-0.036391	-0.028730
	-0.014287	0.031324	0.014450	0.001309	-0.014970	-0.007598
	-0.011967	0.032184	-0.015111	-0.019361	0.015886	-0.004804
	-0.043159					
y147	0.016046	-0.018707	0.019343	0.011790	-0.021278	0.038444
	-0.021980	0.009503	0.029753	0.002807	-0.005864	0.027929
	-0.005073	-0.002261	0.034106	-0.014640	0.049026	0.034111
	0.016049	0.008716	0.005071	-0.005313	0.008640	-0.038125
	0.044230	0.006803	0.022076	-0.008902	-0.056305	-0.048810
	0.054584					
z147	-0.029712	0.019701	0.020524	-0.043631	0.013573	0.002144
	0.001109	0.017691	-0.011266	-0.014367	-0.015933	0.031798
	-0.002518	-0.060903	-0.005328	-0.039457	0.009282	0.021429
	0.049181	0.038750	0.001240	-0.047757	0.021805	-0.011192
	-0.064987	0.003000	-0.013632	-0.009458	-0.004242	0.043106
	-0.066812					
x148	-0.021742	0.022263	0.010349	-0.003744	0.013844	-0.003544
	0.026179	0.024169	-0.031302	0.012939	-0.012921	-0.028392
	-0.004743	0.045008	-0.005831	0.025912	-0.032167	-0.034717
	-0.009720	0.032472	0.009659	-0.003125	-0.014987	-0.007694
	-0.010380	0.046022	-0.020360	-0.030951	0.021333	-0.015176
	-0.048208					
y148	0.011387	-0.018396	0.018075	0.016046	-0.022417	0.032692
	-0.022182	0.010193	0.023128	0.001898	-0.006029	0.020275
	-0.008863	0.003393	0.027244	-0.013523	0.039804	0.036830
	0.016185	0.006644	0.001207	0.000914	-0.000292	-0.032357
	0.049224	-0.000797	0.027450	-0.004730	-0.047823	-0.039950
	0.046010					
z148	-0.032931	0.021467	0.022208	-0.043938	0.008474	0.004615
	-0.002110	0.014285	-0.008321	-0.007825	-0.009948	0.030210
	-0.011483	-0.058238	-0.004503	-0.048294	0.016732	0.024175
	0.053714	0.017063	-0.004809	-0.044075	0.016660	-0.025194
	-0.033453	0.022126	-0.000375	-0.005530	-0.014401	0.011849
	-0.042146					

x149	-0.025149	0.020661	0.005880	-0.005518	0.014148	-0.010383
	0.021913	0.023800	-0.029782	0.009787	-0.017545	-0.034986
	-0.000276	0.052113	-0.012538	0.020979	-0.038486	-0.025947
	0.004475	0.037799	0.009867	0.001191	-0.010416	-0.001476
	0.002137	0.046739	-0.020223	-0.033299	0.017195	-0.025822
	-0.046495					
y149	0.006561	-0.016727	0.015269	0.019608	-0.023974	0.025924
	-0.018592	0.010780	0.015955	0.004304	-0.004168	0.014080
	-0.012930	0.007226	0.025644	-0.002069	0.040869	0.019700
	0.006403	-0.000398	-0.008697	0.001662	-0.017337	-0.027246
	0.039395	0.000796	0.026379	-0.003991	-0.033263	-0.024745
	0.034932					
z149	-0.031412	0.024184	0.021064	-0.036477	0.001017	0.005336
	-0.004880	0.011445	-0.007337	-0.009599	-0.011829	0.031378
	-0.007248	-0.052028	-0.023061	-0.070582	0.005789	0.046576
	0.068613	0.019311	-0.002983	-0.057756	0.037781	-0.013916
	-0.003767	0.029396	0.019657	0.000752	-0.008320	-0.003922
	-0.015957					
x150	-0.026241	0.019109	0.006268	-0.006323	0.016686	-0.021803
	0.026844	0.029537	-0.026619	-0.001103	-0.018159	-0.022949
	-0.001122	0.065040	-0.024898	0.016450	-0.033421	-0.015811
	0.015656	0.046780	0.013007	0.002912	-0.023288	0.017859
	-0.009975	0.051519	-0.015287	-0.048443	-0.000615	-0.030370
	-0.039705					
y150	-0.001137	-0.010870	0.007847	0.020238	-0.024012	0.020368
	-0.022227	0.002278	0.001949	0.014763	-0.008147	0.001278
	-0.009209	-0.005004	0.035133	0.010262	0.036244	-0.007080
	-0.017352	-0.012137	-0.017913	0.001431	-0.017069	-0.023764
	0.034207	-0.009653	0.006573	0.006088	-0.007763	0.005038
	0.007908					
z150	-0.029216	0.021134	0.023784	-0.025736	-0.005621	0.004474
	-0.007289	0.023767	-0.003822	-0.025393	-0.009234	0.039670
	-0.013015	-0.031825	-0.049525	-0.075145	-0.002718	0.056691
	0.091316	0.040334	0.001505	-0.060974	0.042143	-0.000388
	0.029207	0.049766	0.067990	0.012894	-0.011646	-0.037143
	0.049930					
x151	-0.029241	0.017143	0.008583	-0.013824	0.016215	-0.030222
	0.025107	0.026745	-0.023017	-0.001956	-0.012551	-0.016774
	0.008604	0.068506	-0.023757	0.011392	-0.030456	-0.018151
	0.027050	0.033539	0.004364	0.024038	-0.047378	0.031870
	-0.001333	0.061861	-0.036547	-0.033997	-0.000252	-0.025167
	-0.019441					

y151	-0.005265	-0.009105	0.003771	0.022233	-0.022735	0.008756
	-0.019441	0.004002	0.000976	0.012267	-0.007659	-0.008859
	-0.012625	0.007457	0.029085	0.014721	0.032271	-0.006330
	-0.012335	-0.011925	-0.019709	0.000539	-0.020312	-0.017383
	0.032724	0.009819	0.012376	0.002798	0.000524	0.005043
	0.010058					
z151	-0.031242	0.021513	0.021366	-0.021688	-0.016586	0.008114
	-0.009679	0.018226	-0.008388	-0.014914	-0.004630	0.043163
	-0.006914	-0.021454	-0.052077	-0.072971	-0.003258	0.053965
	0.090950	0.022908	-0.003613	-0.060552	0.054538	0.006016
	0.088818	0.068901	0.083281	0.036599	-0.017037	-0.064076
	0.108406					
x152	-0.029208	0.018869	0.008651	-0.018761	0.017588	-0.033823
	0.023963	0.027769	-0.023149	0.002170	-0.010305	-0.012476
	0.006936	0.054577	-0.013869	0.023412	-0.013241	-0.032865
	0.006973	0.027323	0.000349	0.013106	-0.050231	0.050826
	0.008973	0.046342	-0.063081	-0.033171	0.002033	-0.002117
	-0.008555					
y152	-0.010209	-0.006911	0.000073	0.019232	-0.018586	-0.002466
	-0.017749	0.005084	0.001444	0.010837	-0.007851	-0.010016
	-0.012914	0.010300	0.032374	0.016861	0.029018	-0.013991
	-0.018003	-0.006071	-0.013033	0.001996	-0.021629	-0.012124
	0.027648	0.017627	0.010216	-0.001226	0.000869	0.007766
	0.007685					
z152	-0.027208	0.022255	0.011961	-0.018741	-0.014046	0.008685
	-0.013497	0.013813	-0.016795	-0.009930	-0.003599	0.038811
	0.007705	-0.024836	-0.038684	-0.068796	-0.012321	0.026511
	0.070944	0.030271	-0.015109	-0.062334	0.050001	0.007162
	0.083771	0.047258	0.049735	0.022376	-0.015706	-0.014787
	0.050638					
x153	-0.028182	0.023730	0.007812	-0.020971	0.015030	-0.032972
	0.018918	0.028751	-0.018608	0.010979	-0.009353	-0.002795
	0.005968	0.047953	-0.011474	0.026896	-0.004232	-0.029249
	-0.004720	0.039772	-0.000550	0.018207	-0.030560	0.031760
	-0.000700	0.053935	-0.067052	-0.041072	0.000726	0.021630
	-0.022337					
y153	-0.015539	-0.005010	-0.003375	0.013719	-0.013708	-0.012780
	-0.015390	0.006986	0.005128	0.011869	-0.005245	-0.009937
	-0.013878	0.013731	0.037878	0.021998	0.028917	-0.020903
	-0.023331	-0.001392	-0.007208	0.006613	-0.023245	-0.010320
	0.023718	0.026806	0.013232	-0.001035	-0.002560	0.015204
	0.008816					

z153	-0.023523	0.026852	0.007215	-0.013820	-0.011590	0.010814
	-0.017868	0.012200	-0.023782	-0.000772	-0.012241	0.044177
	0.017890	-0.036768	-0.033628	-0.064793	-0.020079	0.018985
	0.053475	0.042887	-0.020064	-0.065218	0.068457	-0.001313
	0.060591	0.027455	0.033712	0.033997	-0.005866	0.023333
	0.029523					
x154	-0.029804	0.024168	0.005632	-0.020241	0.011704	-0.028688
	0.015567	0.025072	-0.016391	0.011546	-0.009033	-0.005199
	0.013300	0.047256	-0.014667	0.035743	0.007932	-0.018713
	-0.003946	0.036034	0.007501	0.021640	-0.024195	0.033510
	-0.004108	0.051140	-0.052016	-0.042713	0.003364	0.027826
	-0.019511					
y154	-0.020209	-0.003322	-0.006699	0.009065	-0.008228	-0.023114
	-0.011722	0.008039	0.008022	0.010503	-0.003746	-0.010403
	-0.010115	0.017045	0.040731	0.021640	0.028741	-0.023404
	-0.024630	-0.000089	0.001522	0.012281	-0.025236	-0.001910
	0.019645	0.032675	0.017286	-0.012230	-0.005564	0.023379
	0.005110					
z154	-0.022966	0.029363	0.003472	-0.009810	-0.008873	0.012484
	-0.022508	0.010722	-0.027260	0.007648	-0.021396	0.046906
	0.025955	-0.049397	-0.027259	-0.049054	-0.023682	0.017174
	0.043481	0.047812	-0.023083	-0.075320	0.080399	-0.009217
	0.038437	0.008517	0.034623	0.071351	0.005520	0.045248
	0.037382					
x155	-0.030177	0.025523	0.002619	-0.019792	0.009526	-0.021421
	0.012000	0.024542	-0.010708	0.012029	-0.013280	-0.001931
	0.008335	0.035709	-0.012957	0.034456	0.010269	-0.019751
	-0.006872	0.027324	0.008958	0.019841	-0.023739	0.040192
	0.000106	0.048460	-0.033249	-0.022824	0.006047	0.024119
	-0.026916					
y155	-0.023672	-0.000883	-0.010441	0.004053	-0.002839	-0.031545
	-0.008547	0.008952	0.012181	0.008691	-0.004633	-0.007600
	-0.011479	0.014092	0.044749	0.015026	0.024003	-0.029614
	-0.027220	-0.002214	0.009677	0.016298	-0.029488	0.009017
	0.021670	0.037080	0.024561	-0.018455	-0.004809	0.031595
	-0.006060					
z155	-0.019402	0.027212	-0.000111	-0.006892	-0.003552	0.009907
	-0.025429	0.007847	-0.033326	0.008846	-0.023540	0.044081
	0.027862	-0.061317	-0.019699	-0.059545	-0.029376	-0.001848
	0.031672	0.046837	-0.029038	-0.056246	0.065840	-0.009252
	0.035510	0.001674	0.017825	0.043029	-0.012457	0.058466
	-0.007682					

x156	-0.027354	0.029571	-0.001815	-0.021369	0.004771	-0.016577
	0.006242	0.018152	-0.010951	0.015948	-0.015904	0.006497
	0.001261	0.031405	-0.013670	0.036325	0.010884	-0.014585
	-0.015114	0.027894	0.018028	0.020085	-0.023471	0.031746
	0.004997	0.049458	-0.035654	-0.010871	-0.004729	0.025641
	-0.023710					
y156	-0.026235	0.003547	-0.015096	-0.003421	0.000158	-0.039340
	-0.007152	0.006637	0.014911	0.012340	-0.005579	-0.001366
	-0.013544	0.015050	0.047488	0.015651	0.020209	-0.027392
	-0.032483	-0.000036	0.022608	0.017296	-0.027942	0.007696
	0.020598	0.042826	0.022667	-0.015755	-0.009549	0.042293
	0.000549					
z156	-0.015661	0.027153	-0.003272	-0.005860	0.000895	0.006923
	-0.029858	0.006457	-0.036609	0.015209	-0.025901	0.044930
	0.029933	-0.068243	-0.012892	-0.057919	-0.035123	-0.006275
	0.019517	0.049278	-0.030698	-0.050017	0.058516	-0.007665
	0.028610	0.001132	0.000481	0.037092	-0.025243	0.066531
	-0.022180					
x157	-0.026567	0.030307	-0.002269	-0.020653	0.002504	-0.003257
	-0.000779	0.015201	-0.010115	0.019392	-0.024373	0.011828
	-0.000383	0.020059	-0.018705	0.038969	0.011983	-0.012614
	-0.021378	0.015991	0.010979	0.023377	-0.023796	0.034979
	0.006541	0.042343	-0.035366	-0.011695	-0.016520	0.023196
	-0.031184					
y157	-0.029811	0.005158	-0.018079	-0.009476	0.003570	-0.040011
	-0.007891	0.007616	0.019059	0.015985	-0.011245	0.002040
	-0.011970	0.011546	0.045138	0.020141	0.013909	-0.027958
	-0.038056	-0.004909	0.023981	0.016164	-0.028504	0.016210
	0.013409	0.043894	0.022793	-0.022560	-0.013426	0.048514
	-0.000986					
z157	-0.013258	0.025847	-0.001472	-0.004759	0.007124	0.005953
	-0.034494	0.008183	-0.037125	0.022929	-0.032643	0.045584
	0.027614	-0.082705	-0.008808	-0.058446	-0.033770	-0.010712
	0.012272	0.042294	-0.036241	-0.032660	0.044782	-0.009568
	0.020614	-0.001380	-0.014230	0.024630	-0.043671	0.063208
	-0.032337					
x158	-0.022726	0.028796	-0.003286	-0.017289	-0.003341	0.006837
	-0.005431	0.012957	-0.009707	0.016037	-0.030195	0.014226
	0.002405	0.017175	-0.025595	0.036179	0.012195	-0.008784
	-0.018810	0.005996	0.007798	0.012626	-0.021321	0.028596
	0.008976	0.035005	-0.027138	-0.008736	-0.003842	0.014631
	-0.020400					

y158	-0.030814	0.004039	-0.022565	-0.013977	0.003447	-0.042903
	-0.005892	0.008341	0.023973	0.010897	-0.013826	0.001893
	-0.007087	0.018911	0.040724	0.019636	0.007720	-0.024658
	-0.028720	-0.011773	0.029331	0.004390	-0.025320	0.007280
	0.010873	0.047553	0.037215	-0.027203	0.005433	0.041540
	0.021626					
z158	-0.007726	0.024541	-0.000278	-0.001843	0.010394	0.000174
	-0.039363	0.009850	-0.037863	0.027130	-0.039229	0.043348
	0.028212	-0.095215	-0.008393	-0.057589	-0.032980	-0.005877
	0.008743	0.045260	-0.034277	-0.041820	0.038672	-0.007098
	0.006106	-0.005823	-0.018509	0.027310	-0.038353	0.063403
	-0.030913					
x159	-0.019538	0.029609	-0.002766	-0.013466	-0.006573	0.017855
	-0.010123	0.010475	-0.010457	0.017056	-0.036538	0.019831
	0.000557	0.012002	-0.024170	0.044704	0.011141	-0.007278
	-0.020327	0.007829	0.007594	0.007270	-0.014845	0.021856
	0.015016	0.024385	-0.021411	0.012032	-0.000895	0.008005
	-0.008447					
y159	-0.033621	0.005848	-0.026848	-0.018518	0.007955	-0.040968
	-0.004193	0.004935	0.025149	0.012865	-0.018774	0.005850
	-0.009221	0.023989	0.046380	0.037049	0.001646	-0.029568
	-0.029979	-0.000733	0.041311	0.006199	-0.018485	-0.005596
	0.010021	0.043494	0.044180	-0.009830	0.005094	0.036208
	0.027908					
z159	-0.005844	0.019309	0.001011	-0.004185	0.015937	-0.004014
	-0.045604	0.012663	-0.034854	0.035163	-0.038093	0.039259
	0.021249	-0.101797	0.001164	-0.039453	-0.027976	-0.005980
	0.008708	0.048312	-0.029216	-0.042403	0.023366	-0.010390
	0.006823	0.005166	-0.021455	0.043783	-0.044974	0.045337
	-0.010747					
x160	-0.014044	0.028301	0.000544	-0.009513	-0.009983	0.026018
	-0.014953	0.008214	-0.009446	0.018616	-0.035457	0.022121
	-0.001631	0.004484	-0.023360	0.044027	0.009620	-0.008076
	-0.020645	0.002732	0.004622	0.003754	-0.008813	0.024124
	0.021871	0.014901	-0.023157	0.016237	0.002310	0.001580
	-0.012838					
y160	-0.034239	0.001590	-0.028071	-0.022667	0.012666	-0.034117
	-0.004489	0.000130	0.028042	0.014372	-0.013142	0.005700
	-0.007856	0.030896	0.038830	0.041444	-0.004761	-0.034921
	-0.030375	-0.004073	0.051263	0.009336	-0.007788	-0.005228
	0.008373	0.039243	0.038953	-0.019079	0.007094	0.026041
	-0.002120					

z160	-0.001201	0.011141	0.004293	-0.004215	0.019740	-0.008647
	-0.050969	0.011893	-0.031931	0.040754	-0.033848	0.032133
	0.013904	-0.109624	0.001798	-0.036526	-0.021872	-0.006066
	0.006380	0.047508	-0.023898	-0.036193	0.002221	-0.006685
	0.004677	0.023109	-0.037051	0.025609	-0.046092	0.025293
	-0.021808					
x161	-0.009410	0.028246	0.003517	-0.006848	-0.014170	0.028264
	-0.020484	0.006678	-0.006074	0.019373	-0.031932	0.023522
	-0.003240	-0.000143	-0.018464	0.039885	0.006942	-0.004774
	-0.018927	-0.003836	-0.001063	0.002960	-0.005076	0.026232
	0.026088	0.008472	-0.019512	0.013326	0.004926	0.000458
	-0.007844					
y161	-0.033936	-0.001532	-0.027657	-0.027633	0.011045	-0.030353
	-0.008296	-0.005620	0.031118	0.019516	-0.017271	0.005548
	-0.006536	0.033277	0.031318	0.047476	-0.016418	-0.026047
	-0.035498	0.000926	0.064312	0.013117	-0.002503	-0.018105
	-0.001270	0.058760	0.031898	-0.026738	0.011296	0.012013
	-0.001052					
z161	-0.000610	0.011108	0.002345	-0.001052	0.021427	-0.011661
	-0.056275	0.007353	-0.023603	0.045339	-0.027976	0.025636
	0.004294	-0.096326	0.001537	-0.029867	-0.017762	0.004060
	0.012205	0.038231	-0.017730	-0.022869	0.002633	-0.010372
	-0.007653	0.005679	-0.026901	0.010868	-0.018787	0.013449
	-0.014193					
x162	-0.005169	0.029008	0.007378	-0.004160	-0.017056	0.027226
	-0.023833	0.009609	-0.003047	0.020246	-0.027540	0.025641
	-0.005196	-0.005390	-0.010759	0.033340	0.006467	-0.004751
	-0.016757	-0.012494	-0.012020	0.005929	-0.005621	0.030011
	0.027772	0.003428	-0.018535	0.009511	0.000509	-0.001054
	-0.004096					
y162	-0.035101	-0.002555	-0.027953	-0.028938	0.015890	-0.021724
	-0.004421	-0.003268	0.028581	0.017977	-0.019284	0.002562
	0.001108	0.029602	0.025363	0.058846	-0.019016	-0.029714
	-0.043755	0.006168	0.066287	0.027616	-0.002890	-0.021273
	-0.015643	0.054825	0.041644	-0.021190	0.023762	0.004853
	-0.023828					
z162	-0.002273	0.010628	0.001662	0.000055	0.028943	-0.012514
	-0.057717	0.009831	-0.021050	0.046324	-0.023164	0.017713
	-0.001404	-0.084694	0.006255	-0.022589	-0.006156	0.005904
	0.016703	0.026743	-0.023750	-0.006268	-0.000170	-0.010169
	-0.020871	-0.005303	-0.019638	0.003801	-0.012847	-0.006226
	-0.020516					

x163	-0.000468	0.029518	0.010740	-0.001079	-0.020778	0.024868
	-0.025438	0.012541	0.000335	0.020592	-0.023283	0.026683
	-0.006559	-0.009397	-0.003422	0.026730	0.004069	-0.004958
	-0.014562	-0.021866	-0.020820	0.005166	-0.006747	0.036368
	0.028403	0.000839	-0.012673	0.004350	-0.002145	-0.001705
	-0.000468					
y163	-0.031750	-0.004316	-0.027902	-0.026521	0.014438	-0.016306
	-0.003961	-0.001053	0.035455	0.015396	-0.024569	-0.001068
	0.007020	0.027019	0.017991	0.064582	-0.025270	-0.023529
	-0.037450	0.016759	0.069174	0.030628	-0.002615	-0.043603
	-0.011600	0.054106	0.025482	-0.012400	0.035416	0.015630
	-0.033580					
z163	-0.000842	0.009170	-0.000178	0.004119	0.032573	-0.012400
	-0.057362	0.009868	-0.013088	0.043750	-0.020678	0.010993
	-0.005955	-0.074837	0.008516	-0.018614	-0.005766	0.010193
	0.024460	0.021303	-0.019713	-0.001801	0.000267	-0.017147
	-0.023145	-0.008470	-0.018486	-0.000159	0.001051	-0.003661
	-0.017728					
x164	0.003693	0.029650	0.014478	0.003617	-0.023691	0.022329
	-0.025823	0.017922	0.003536	0.020562	-0.020025	0.028197
	-0.007444	-0.014302	0.003811	0.021217	0.001711	-0.004547
	-0.011227	-0.028958	-0.027923	0.010121	-0.010897	0.042818
	0.025804	0.001591	-0.010355	-0.001075	-0.005469	-0.000593
	-0.002966					
y164	-0.029071	-0.002313	-0.025868	-0.031902	0.017084	-0.015796
	-0.004323	-0.004733	0.035594	0.017173	-0.025221	-0.002715
	0.009189	0.026996	0.011430	0.056131	-0.021874	-0.023263
	-0.027858	0.010873	0.059304	0.029309	0.004423	-0.054613
	-0.005617	0.050723	0.024281	-0.000836	0.031309	-0.014443
	-0.012042					
z164	-0.001170	0.007680	0.000760	0.001670	0.040600	-0.013764
	-0.057904	0.009627	-0.010023	0.045487	-0.016064	0.005297
	-0.010765	-0.064988	0.011897	-0.020836	0.001486	0.008636
	0.029666	0.007275	-0.024027	0.006404	-0.003124	-0.022873
	-0.021475	-0.002078	-0.016371	0.001225	-0.002421	-0.034985
	-0.002842					
x165	0.007298	0.030690	0.016234	0.007535	-0.026322	0.017976
	-0.023911	0.021112	0.004647	0.023022	-0.016357	0.027039
	-0.009457	-0.016347	0.008430	0.011332	-0.004101	-0.002352
	-0.007547	-0.036729	-0.033067	0.015607	-0.013695	0.042095
	0.022324	0.007166	-0.007589	-0.008915	-0.002524	0.002203
	0.006671					

y165	-0.026165	-0.003460	-0.022593	-0.031690	0.017914	-0.012148
	-0.006256	-0.001611	0.043374	0.012130	-0.023723	0.003009
	0.016153	0.023415	0.007615	0.062630	-0.025370	-0.026941
	-0.025870	0.015955	0.056270	0.017928	-0.001645	-0.041872
	-0.003995	0.034670	0.012280	0.020979	0.031023	-0.033520
	-0.025565					
z165	-0.001211	0.001609	0.000723	0.001985	0.045715	-0.009975
	-0.055799	0.011360	0.000539	0.041161	-0.011252	0.004077
	-0.008363	-0.056739	0.016208	-0.014514	-0.002246	0.002366
	0.030245	0.002361	-0.020867	-0.004358	-0.003528	-0.010298
	-0.022314	0.001355	-0.019823	0.008148	0.007180	-0.049741
	-0.012511					
x166	0.009890	0.030539	0.019048	0.011962	-0.027818	0.015771
	-0.020917	0.027308	0.007533	0.020671	-0.013793	0.028073
	-0.009014	-0.018848	0.011036	0.006958	-0.011337	-0.006932
	-0.005857	-0.038775	-0.038117	0.017409	-0.021215	0.053577
	0.013344	0.009403	-0.006333	-0.012191	0.001770	-0.007353
	0.008967					
y166	-0.020396	-0.002813	-0.020495	-0.033681	0.016718	-0.014163
	-0.008955	-0.004630	0.040501	0.015534	-0.022028	0.000966
	0.015836	0.022436	0.004318	0.055421	-0.028952	-0.009492
	-0.019659	0.009074	0.054491	0.017755	0.002867	-0.053692
	0.005044	0.023078	0.001197	0.030691	0.020934	-0.029973
	-0.022935					
z166	0.000850	-0.002439	0.000640	0.000125	0.051830	-0.009858
	-0.053289	0.009447	-0.000870	0.045605	-0.007889	-0.001784
	-0.009235	-0.046599	0.013798	-0.015667	-0.005930	0.010996
	0.027355	-0.006900	-0.013882	-0.001866	-0.002831	-0.022893
	-0.019248	0.005065	-0.019109	0.002594	0.010336	-0.054374
	-0.010916					
x167	0.011755	0.030710	0.020656	0.014952	-0.028253	0.013631
	-0.017332	0.029201	0.010726	0.022918	-0.012591	0.030653
	-0.008089	-0.017181	0.015618	0.000946	-0.019461	-0.005648
	-0.005526	-0.045183	-0.041887	0.014704	-0.030207	0.057996
	0.003149	0.018408	-0.019959	-0.008792	0.010929	-0.019067
	0.001616					
y167	-0.015217	-0.001882	-0.016086	-0.031515	0.015051	-0.017469
	-0.009467	0.003009	0.038600	0.012948	-0.018993	0.002584
	0.018737	0.017675	-0.004026	0.051483	-0.025023	-0.004329
	-0.008478	0.009057	0.045135	0.029937	0.010157	-0.050571
	0.010366	0.004714	0.003501	0.026105	0.006195	-0.030270
	-0.012919					

z167	0.002915	-0.007648	0.003212	-0.000386	0.058833	-0.008876
	-0.049582	0.015237	-0.003361	0.047074	-0.006103	-0.003672
	-0.004852	-0.037367	0.005315	-0.012669	-0.004730	0.010161
	0.025893	-0.008992	-0.015206	0.008087	0.004307	-0.028891
	-0.018376	0.003854	-0.017486	-0.007996	0.005570	-0.064109
	-0.011587					
x168	0.013643	0.031444	0.021420	0.017275	-0.026376	0.011582
	-0.011890	0.031678	0.010688	0.025409	-0.010746	0.029684
	-0.005743	-0.017545	0.014104	-0.006908	-0.028056	-0.005416
	0.000580	-0.050216	-0.038012	0.019281	-0.034875	0.059777
	0.002648	0.010406	-0.010399	-0.013887	0.019661	-0.028767
	0.010896					
y168	-0.009906	-0.001561	-0.013563	-0.028056	0.009842	-0.019406
	-0.009585	0.006093	0.039256	0.010711	-0.017151	0.008334
	0.016381	0.016928	-0.008757	0.053755	-0.030706	-0.001781
	-0.007293	0.004214	0.031530	0.027518	0.003426	-0.044677
	0.011156	0.001660	-0.015843	0.034164	-0.002497	-0.028868
	-0.019425					
z168	0.007673	-0.015444	0.003499	-0.000954	0.058863	-0.004106
	-0.045035	0.015526	0.000124	0.045271	-0.005855	0.001002
	-0.006530	-0.022910	-0.004299	-0.000553	-0.023298	-0.002994
	0.015293	-0.008848	-0.009543	-0.009462	-0.002847	-0.033704
	-0.018870	0.018763	-0.030485	-0.002483	0.012372	-0.061813
	-0.021101					
x169	0.014800	0.029199	0.020592	0.020705	-0.027387	0.010649
	-0.004699	0.032020	0.011809	0.030087	-0.009182	0.038270
	-0.002317	-0.011067	0.014051	-0.011725	-0.040262	-0.008101
	0.004554	-0.058482	-0.036393	0.011548	-0.043924	0.057742
	-0.004839	-0.001946	-0.022521	-0.014905	0.029893	-0.013417
	0.010885					
y169	-0.005592	0.000800	-0.011940	-0.024482	0.005662	-0.019939
	-0.007146	0.009036	0.041953	0.014486	-0.015785	0.010769
	0.019058	0.011966	-0.013956	0.052571	-0.030773	-0.000358
	-0.005569	-0.005588	0.017606	0.035111	0.000112	-0.035714
	0.015160	-0.008302	-0.022371	0.031895	-0.004167	-0.020412
	-0.023596					
z169	0.009059	-0.020898	0.003350	-0.004399	0.061293	0.002093
	-0.037448	0.009058	0.004308	0.050634	-0.008037	0.001325
	0.002743	-0.012520	-0.016716	0.002980	-0.027912	-0.015945
	0.008673	-0.024490	-0.006469	-0.008896	0.000213	-0.033128
	-0.008107	0.013704	-0.036020	-0.011322	0.034307	-0.029313
	-0.026955					

x170	0.013415	0.025963	0.014568	0.024156	-0.030092	0.012380
	0.002756	0.033751	0.009603	0.036232	-0.000828	0.050416
	-0.002627	-0.006069	0.017711	-0.014861	-0.046740	-0.002293
	0.011479	-0.071716	-0.048555	0.017516	-0.059247	0.070216
	-0.013793	-0.006809	-0.040792	-0.010921	0.031909	0.002884
	-0.008462					
y170	-0.001493	0.003137	-0.010212	-0.017870	0.001046	-0.019799
	-0.005727	0.013359	0.045148	0.012970	-0.015381	0.011628
	0.017837	0.005650	-0.017484	0.046672	-0.027638	0.005824
	-0.000292	-0.007517	0.014690	0.036087	-0.003885	-0.031483
	0.010167	-0.007262	-0.017740	0.025161	-0.009214	-0.023624
	-0.027542					
z170	0.009556	-0.026846	0.002985	-0.006108	0.057460	0.007157
	-0.033488	0.009063	0.010030	0.040902	-0.009106	-0.000142
	0.005694	-0.001440	-0.018385	-0.006788	-0.018847	-0.023293
	0.000935	-0.021703	0.023588	-0.012403	0.004732	-0.046297
	-0.010930	0.023381	-0.031886	-0.036010	0.029608	-0.025147
	-0.038953					
x171	0.013352	0.023438	0.009595	0.022944	-0.029237	0.012222
	0.008441	0.035875	0.006049	0.033125	-0.002662	0.056412
	0.000123	-0.006578	0.018816	-0.019600	-0.042397	-0.005239
	0.012342	-0.061626	-0.034827	0.026447	-0.054259	0.068198
	-0.018085	0.000244	-0.049637	-0.003697	0.032280	0.002428
	0.001755					
y171	0.001969	0.004064	-0.007846	-0.010980	-0.005226	-0.018233
	-0.002519	0.018323	0.047611	0.011642	-0.013781	0.013403
	0.015844	0.003207	-0.022570	0.043650	-0.031791	0.017172
	0.015185	-0.009783	0.008770	0.039205	-0.018468	-0.035013
	0.007366	-0.014702	-0.012947	0.018746	-0.008122	-0.031352
	-0.031891					
z171	0.010842	-0.034079	0.003447	-0.006284	0.050056	0.016104
	-0.032168	0.009602	0.011524	0.036432	-0.002855	-0.000148
	0.003803	0.009427	-0.022449	-0.000981	-0.031039	-0.019557
	0.002709	-0.023061	0.030677	-0.007198	-0.018824	-0.062686
	-0.013796	0.010963	-0.016871	-0.045522	0.037971	-0.028433
	-0.046804					
x172	0.012336	0.021446	0.003223	0.023773	-0.030097	0.013738
	0.012580	0.037543	0.002067	0.027857	-0.004536	0.053727
	0.005692	-0.012599	0.019891	-0.016374	-0.041465	-0.004095
	0.019238	-0.054350	-0.029763	0.029701	-0.064561	0.051489
	-0.020144	-0.005155	-0.036307	0.001816	0.020712	-0.000159
	-0.017542					

y172	0.004924	0.004641	-0.005407	-0.005004	-0.011240	-0.017841
	0.001412	0.020277	0.049175	0.015237	-0.011546	0.016685
	0.013650	-0.002059	-0.025074	0.041082	-0.031421	0.024888
	0.027357	-0.011077	0.004710	0.037953	-0.030465	-0.035086
	0.004927	-0.022622	-0.014503	0.017464	-0.001560	-0.033989
	-0.033971					
z172	0.013585	-0.042694	0.004691	-0.010402	0.044963	0.019360
	-0.031840	0.005841	0.005156	0.036968	0.006869	0.008965
	-0.005883	0.025315	-0.021388	-0.000771	-0.026425	-0.023031
	-0.002652	-0.022625	0.024965	0.007817	-0.000223	-0.058246
	-0.010541	0.025260	-0.022054	-0.036563	0.073736	-0.009829
	-0.003573					
x173	0.010314	0.019335	-0.003098	0.020904	-0.026972	0.016457
	0.017727	0.036421	-0.003489	0.023046	-0.004846	0.053336
	0.002952	-0.015079	0.022879	-0.015592	-0.042318	-0.003306
	0.020917	-0.045508	-0.020646	0.037784	-0.067557	0.041877
	-0.025259	-0.004581	-0.019302	-0.000196	0.029138	-0.000021
	-0.017855					
y173	0.006773	0.005956	-0.005129	-0.000022	-0.015155	-0.016207
	0.005995	0.022563	0.048246	0.018340	-0.009062	0.023378
	0.013214	-0.005380	-0.026395	0.038254	-0.028518	0.034307
	0.034224	-0.009673	-0.002394	0.043714	-0.033720	-0.033909
	0.003509	-0.025475	-0.012115	0.018158	0.005506	-0.034434
	-0.026244					
z173	0.014717	-0.046960	0.003810	-0.011624	0.037797	0.022755
	-0.031850	0.003719	0.004866	0.032099	0.012291	0.016016
	0.008292	0.035822	-0.019334	0.005277	-0.021533	-0.019392
	0.001049	-0.016206	0.022992	0.008010	0.003901	-0.064018
	-0.010610	0.025011	-0.028048	-0.029658	0.080574	-0.005053
	0.005968					
x174	0.009446	0.016498	-0.008897	0.019592	-0.025500	0.015973
	0.016505	0.034889	-0.011532	0.023284	-0.008298	0.049736
	0.002753	-0.020853	0.027054	-0.015636	-0.035159	-0.002712
	0.019805	-0.039450	-0.020864	0.036096	-0.057593	0.037251
	-0.027523	-0.002421	-0.003480	0.009272	0.009780	0.007805
	-0.020749					
y174	0.008743	0.007552	-0.006226	0.005570	-0.019593	-0.015781
	0.010970	0.024417	0.045956	0.021346	-0.008203	0.027365
	0.011299	-0.011226	-0.027577	0.035828	-0.023555	0.043443
	0.042030	-0.006124	-0.011697	0.045736	-0.035424	-0.038587
	0.005541	-0.027040	-0.001913	0.020846	0.003550	-0.033184
	-0.020266					

z174	0.015158	-0.049281	-0.000228	-0.012707	0.028722	0.027104
	-0.027026	0.004147	0.004273	0.021255	0.015206	0.018532
	0.013222	0.042639	-0.016136	0.010016	-0.015685	-0.016448
	0.007259	-0.005756	0.021297	0.009573	-0.001453	-0.090157
	-0.003953	0.020361	-0.016192	-0.029831	0.076824	0.003597
	0.015485					
x175	0.008807	0.015121	-0.011566	0.014216	-0.021026	0.016897
	0.014918	0.029124	-0.016345	0.019572	-0.007996	0.046361
	0.005661	-0.025061	0.032885	-0.025763	-0.036514	-0.002058
	0.020385	-0.026622	-0.009682	0.031941	-0.059327	0.048877
	-0.024900	0.016390	-0.013714	0.017740	-0.017865	-0.012477
	-0.023529					
y175	0.009768	0.009879	-0.006899	0.010436	-0.021244	-0.013861
	0.016263	0.025338	0.044027	0.024530	-0.006057	0.032388
	0.009687	-0.016107	-0.027516	0.033094	-0.022797	0.048610
	0.046026	0.001776	-0.014049	0.047036	-0.038156	-0.040121
	0.003193	-0.022593	0.002070	0.019319	0.005371	-0.035856
	-0.015688					
z175	0.014312	-0.051161	-0.005541	-0.011018	0.020004	0.031234
	-0.025310	0.007593	-0.000575	0.018313	0.019657	0.022858
	0.011609	0.043468	-0.010654	0.023173	-0.009034	-0.019898
	-0.001091	0.001206	0.016929	0.015434	0.003755	-0.115515
	-0.010250	0.011408	0.011396	-0.030278	0.097456	0.023460
	0.028057					
x176	0.006855	0.013346	-0.013533	0.007414	-0.017094	0.019531
	0.010986	0.023547	-0.021551	0.013382	-0.008675	0.038808
	0.006066	-0.031840	0.035969	-0.035939	-0.040409	-0.004353
	0.019672	-0.014428	0.002779	0.026216	-0.057953	0.056331
	-0.020431	0.026198	-0.012152	0.022493	-0.037358	-0.020580
	-0.021783					
y176	0.010479	0.013179	-0.006346	0.014168	-0.021268	-0.011713
	0.020345	0.025626	0.042861	0.028053	-0.004418	0.032680
	0.003452	-0.028075	-0.028209	0.023819	-0.025380	0.051595
	0.049596	0.009546	-0.017505	0.047586	-0.043325	-0.032584
	0.006316	-0.018343	0.002481	0.025964	-0.006431	-0.050854
	-0.010542					
z176	0.015927	-0.052065	-0.012306	-0.008644	0.007195	0.030839
	-0.021836	0.007915	-0.004326	0.022090	0.025067	0.024870
	0.012729	0.045552	-0.001391	0.036885	-0.001509	-0.016826
	-0.014193	0.014060	0.011746	0.013109	0.014865	-0.131305
	-0.019197	0.022760	0.022508	-0.019285	0.093543	0.039263
	0.035361					

x177	0.002943	0.010786	-0.016990	0.001732	-0.014106	0.023465
	0.007968	0.016910	-0.028912	0.012276	-0.010804	0.029788
	0.007422	-0.032646	0.042425	-0.031851	-0.040353	-0.007731
	0.010460	-0.007992	0.017997	0.015081	-0.048766	0.048644
	-0.023269	0.036459	-0.001164	0.014639	-0.043109	-0.004978
	-0.016158					
y177	0.010093	0.015029	-0.005892	0.019949	-0.021274	-0.007460
	0.025412	0.026486	0.040612	0.034706	-0.004121	0.029824
	-0.004242	-0.038092	-0.025086	0.017995	-0.033745	0.056112
	0.047100	0.016996	-0.016012	0.035123	-0.038652	-0.023784
	0.001155	-0.013381	0.003480	0.030685	-0.004342	-0.057791
	-0.006977					
z177	0.016306	-0.052818	-0.016835	-0.013396	-0.004958	0.031080
	-0.023849	0.009471	-0.018159	0.017634	0.019069	0.021980
	0.011563	0.030847	0.006838	0.022774	0.002198	-0.019387
	-0.024392	0.028696	0.008572	0.012538	0.025025	-0.130244
	-0.025305	0.022101	0.026745	0.000298	0.075853	0.041533
	0.015510					
x178	0.000433	0.007956	-0.020200	-0.005474	-0.005877	0.025336
	-0.001024	0.013034	-0.031063	0.010755	-0.014531	0.015217
	0.000150	-0.029337	0.036370	-0.027387	-0.034254	-0.009984
	0.000234	-0.007856	0.023412	-0.002527	-0.039719	0.040679
	-0.013026	0.040875	-0.005080	-0.004543	-0.042208	0.000173
	-0.003055					
y178	0.011692	0.014923	-0.005918	0.031463	-0.021459	-0.003186
	0.031157	0.025311	0.036402	0.033873	-0.001324	0.022568
	-0.015314	-0.029726	-0.032558	0.022472	-0.038982	0.057904
	0.044989	0.017396	-0.016516	0.024602	-0.022960	-0.056129
	-0.006986	-0.002803	0.022177	-0.011980	0.017893	-0.035567
	-0.008915					
z178	0.014157	-0.055943	-0.022849	-0.018264	-0.020750	0.032639
	-0.025196	0.009803	-0.036604	0.022178	0.016054	0.032206
	0.017945	0.007534	0.018468	-0.002118	0.011155	-0.014408
	-0.021618	0.049733	0.002401	0.005663	0.031815	-0.087955
	-0.037301	0.021463	0.054695	0.053675	0.054899	0.013962
	-0.009782					
x179	-0.001335	0.009086	-0.025410	-0.005657	0.009142	0.027333
	-0.013605	0.020042	-0.023303	0.014874	-0.018955	-0.011844
	-0.020140	-0.010020	0.018785	-0.016396	-0.029177	-0.013788
	-0.024242	-0.019443	0.026484	-0.007210	-0.017752	0.023164
	0.000844	0.015687	-0.048818	-0.027328	-0.019041	0.013130
	0.020165					

y179	0.014944	0.019064	-0.001052	0.037483	-0.010354	-0.002760
	0.022543	0.025048	0.045361	0.028170	-0.009444	0.013154
	-0.028336	-0.010079	-0.056564	0.023956	-0.043223	0.041865
	0.048664	0.009742	-0.027198	0.014041	0.004170	-0.075822
	-0.004470	-0.007892	0.014304	-0.032018	0.034706	-0.047601
	-0.015627					
z179	0.013097	-0.061580	-0.028097	-0.028804	-0.026262	0.032100
	-0.022190	-0.007450	-0.040047	0.017216	0.010280	0.056599
	0.035911	-0.002781	0.026455	-0.006815	0.028064	-0.005907
	0.003736	0.053791	-0.000369	-0.010620	0.004300	-0.053969
	-0.038884	0.029464	0.091375	0.042636	0.028828	-0.010784
	-0.026890					
x180	-0.005205	0.005434	-0.032647	-0.018853	0.016397	0.026499
	-0.024927	0.013909	-0.027313	0.020142	-0.020933	-0.018876
	-0.021671	-0.010125	0.007877	-0.013908	-0.017301	-0.017621
	-0.038756	-0.023188	0.018462	-0.007106	-0.007114	0.021830
	0.003801	0.004645	-0.049028	-0.036662	-0.010298	0.008942
	0.028872					
y180	0.015552	0.020127	0.003141	0.040385	-0.012116	-0.000196
	0.018488	0.023137	0.038596	0.016099	-0.002208	0.007752
	-0.026496	-0.003307	-0.055519	0.025037	-0.058619	0.031417
	0.049125	0.004850	-0.034623	0.019518	0.029229	-0.097479
	-0.011774	-0.010277	0.015516	-0.024912	0.029858	-0.039443
	-0.015347					
z180	0.004416	-0.066407	-0.035999	-0.034629	-0.033826	0.036939
	-0.009034	-0.022049	-0.046668	0.002371	0.010763	0.064653
	0.047732	-0.013483	0.043183	-0.004414	0.046985	-0.007588
	0.019515	0.061035	0.009169	-0.031532	-0.022001	-0.014122
	-0.031706	0.076157	0.109488	0.020977	0.001018	-0.026375
	-0.041043					
x181	-0.008495	0.001572	-0.038216	-0.032136	0.019981	0.025757
	-0.032578	0.008681	-0.033073	0.025953	-0.021722	-0.016991
	-0.015719	-0.014498	0.004438	-0.013009	0.000477	-0.013663
	-0.049577	-0.023032	0.020033	-0.004141	-0.011431	0.021851
	0.008483	0.003329	-0.026721	-0.029027	-0.000455	0.015670
	-0.002606					
y181	0.011261	0.016624	0.003838	0.038580	-0.012833	0.008971
	0.022622	0.020489	0.030339	0.005185	0.001888	0.000587
	-0.025036	-0.008951	-0.042169	0.021967	-0.058331	0.036834
	0.044941	0.005950	-0.026256	0.017102	0.033397	-0.063441
	-0.010406	-0.001622	0.008318	-0.005167	0.018768	-0.026703
	-0.032667					

z181	0.000289	-0.068712	-0.043805	-0.035726	-0.035699	0.036649
	-0.000664	-0.028026	-0.049439	-0.002326	0.013178	0.065758
	0.040298	-0.014546	0.053860	0.005029	0.065365	-0.019353
	0.028071	0.052621	0.003916	-0.041007	-0.040323	-0.010651
	-0.021311	0.078012	0.085333	-0.018926	-0.024511	-0.041904
	0.015424					
x182	-0.012753	-0.000915	-0.043033	-0.042894	0.019355	0.022090
	-0.042381	0.003610	-0.039225	0.027984	-0.019495	-0.019140
	0.003799	-0.019770	0.000397	-0.014978	0.003436	-0.022267
	-0.053700	-0.017596	0.017845	-0.001522	-0.004661	0.028889
	-0.004612	0.004585	-0.005635	-0.014982	0.002112	0.006595
	-0.014696					
y182	0.010070	0.014238	-0.001026	0.037349	-0.008729	0.018074
	0.029910	0.013387	0.020574	0.001621	0.001214	-0.001016
	-0.037015	-0.017528	-0.033034	0.021152	-0.046322	0.047448
	0.036216	0.007371	-0.011552	0.001683	0.031578	-0.046774
	-0.000379	-0.004666	-0.005133	-0.009496	0.017188	-0.002854
	-0.039656					
z182	-0.002769	-0.066068	-0.041747	-0.038623	-0.030617	0.037232
	-0.002553	-0.025664	-0.048828	-0.005859	0.000797	0.063527
	0.037297	-0.009103	0.049028	0.007480	0.089021	-0.002736
	0.022779	0.038144	0.017490	-0.021753	-0.061026	0.006783
	-0.015796	0.073068	0.094515	-0.012432	-0.021405	-0.017081
	-0.041156					
x183	-0.015365	-0.003546	-0.048217	-0.049973	0.017346	0.023581
	-0.047929	-0.007178	-0.043939	0.029826	-0.020780	-0.017642
	0.013782	-0.032120	-0.000073	-0.019377	0.007845	-0.028670
	-0.058626	-0.014757	0.024303	0.007708	-0.004411	0.032036
	-0.004794	0.003772	0.024338	-0.004768	0.006690	0.000305
	-0.018205					
y183	0.006663	0.012210	-0.006285	0.029256	-0.001102	0.020616
	0.033755	0.012651	0.008684	0.004100	0.000089	-0.003819
	-0.041790	-0.026575	-0.030913	0.020315	-0.026745	0.050688
	0.027489	0.007855	-0.003782	-0.017829	0.029695	-0.027091
	-0.005309	-0.010142	-0.027992	-0.029244	0.005224	0.013645
	-0.022268					
z183	-0.003404	-0.064368	-0.044796	-0.035197	-0.031453	0.045691
	-0.004000	-0.034566	-0.042753	-0.017074	-0.003954	0.058102
	0.022566	-0.011341	0.044544	0.012259	0.080681	-0.008417
	0.023121	0.029714	0.015572	-0.031286	-0.055297	0.022765
	-0.011575	0.068470	0.075464	-0.017581	-0.025158	-0.023515
	-0.059556					

x184	-0.013724	-0.007580	-0.052693	-0.054637	0.015988	0.024463
	-0.051069	-0.012694	-0.049267	0.031422	-0.024522	-0.018553
	0.014788	-0.044386	0.005536	-0.017607	0.011907	-0.014886
	-0.064846	-0.009844	0.048363	-0.011583	-0.014376	0.043891
	0.006515	-0.008107	0.021993	0.003499	0.014619	0.013563
	-0.029190					
y184	0.003327	0.010409	-0.015387	0.022579	0.002429	0.017857
	0.033457	0.011406	0.003797	0.010555	0.004293	-0.006256
	-0.034986	-0.030410	-0.040238	0.012587	-0.009580	0.040488
	0.013660	0.020930	-0.012549	-0.034840	0.036196	0.001535
	-0.014185	-0.013644	-0.018223	-0.033605	0.015465	0.023122
	-0.010458					
z184	-0.005923	-0.056667	-0.041507	-0.037157	-0.019297	0.051023
	-0.009505	-0.045506	-0.041136	-0.029542	-0.010416	0.039682
	0.001446	-0.013695	0.041843	0.017916	0.061336	-0.006488
	0.026152	0.004743	0.023148	-0.026184	-0.061156	0.009210
	0.004605	0.044332	0.035903	-0.022986	-0.034536	-0.027995
	-0.018220					
x185	-0.013714	-0.009616	-0.050830	-0.062747	0.019173	0.022901
	-0.053900	-0.014500	-0.049235	0.035587	-0.029452	-0.015242
	0.009168	-0.052665	0.005191	-0.013301	0.017841	-0.012140
	-0.065824	-0.018117	0.053552	-0.017364	-0.020799	0.043827
	0.015487	-0.020416	0.017974	-0.011131	0.012198	0.009144
	-0.035374					
y185	0.000893	0.007063	-0.021452	0.011120	0.009030	0.021629
	0.023694	0.012940	0.001991	0.016621	0.001356	-0.009358
	-0.043803	-0.036749	-0.057290	0.008536	0.001447	0.042037
	-0.009368	0.026627	-0.024927	-0.040971	0.028134	-0.005012
	-0.009072	-0.022729	-0.032433	-0.057009	0.021261	0.020536
	-0.000216					
z185	-0.007047	-0.046467	-0.040881	-0.035904	-0.004533	0.043312
	-0.019303	-0.047997	-0.027917	-0.040921	-0.006706	0.012760
	-0.002887	-0.010496	0.030507	0.019149	0.042799	-0.011364
	0.042920	-0.008125	0.015584	-0.064067	-0.033184	0.013464
	-0.000325	0.014524	0.004073	-0.026822	-0.020399	-0.006090
	0.029816					
x186	-0.016232	-0.005379	-0.045168	-0.062666	0.012978	0.009691
	-0.059398	-0.009327	-0.043444	0.040841	-0.022267	-0.007004
	0.026978	-0.054854	0.012448	0.000508	0.020051	-0.032637
	-0.062648	-0.019017	0.035019	0.002669	-0.013333	0.030439
	0.019775	-0.002236	0.047281	-0.017648	0.024044	-0.029672
	-0.059625					

y186	-0.006507	0.007130	-0.026793	0.008994	0.014591	0.014713
	0.016260	0.015718	0.009251	0.019566	0.013546	-0.013399
	-0.022861	-0.037885	-0.062794	0.008727	0.012849	0.007088
	-0.017675	0.028065	-0.030503	-0.028289	0.013915	-0.013698
	0.014582	-0.007895	0.004824	-0.066567	0.011590	0.001866
	0.034763					
z186	-0.003374	-0.034992	-0.038936	-0.024561	-0.008157	0.022227
	-0.038797	-0.027292	-0.014062	-0.032385	0.010641	0.004655
	0.003815	-0.012760	0.011819	0.013901	0.042604	-0.011738
	0.040572	-0.018523	-0.007161	-0.057572	-0.020593	0.002707
	-0.007221	0.019702	-0.002771	-0.046445	0.001858	-0.009757
	0.001106					
x187	-0.012523	0.001403	-0.036367	-0.062294	0.007072	0.010011
	-0.062105	-0.009970	-0.031467	0.011960	-0.015819	-0.016247
	0.030316	-0.068472	0.003505	0.002617	0.023695	-0.017010
	-0.074628	-0.032912	0.023733	0.012062	-0.008820	-0.004982
	0.021512	-0.016326	0.011442	0.002525	0.053391	-0.009458
	-0.053047					
y187	-0.011830	0.005460	-0.028028	0.003567	0.023108	0.018802
	0.006849	0.012214	0.004435	0.009122	0.009211	-0.032693
	-0.006864	-0.029993	-0.062184	-0.000049	-0.021984	-0.005699
	-0.022075	0.037013	-0.050433	-0.009988	0.012792	-0.041956
	0.006048	-0.012110	0.004515	-0.045516	-0.012709	-0.002291
	0.037814					
z187	0.003720	-0.031015	-0.044329	-0.023191	-0.021420	0.011146
	-0.054154	-0.025481	-0.007794	-0.041060	0.028079	-0.001597
	0.008049	0.000336	-0.027823	0.016028	0.018549	-0.007552
	0.015869	-0.047897	0.016526	-0.004445	-0.035294	-0.018068
	0.008223	0.015903	-0.022078	-0.009042	0.054469	-0.013455
	-0.022482					
x188	-0.004785	0.000237	-0.042084	-0.061491	0.000481	0.007949
	-0.057263	-0.010660	-0.024348	0.016621	0.004249	-0.006485
	0.047979	-0.093312	-0.031494	0.014085	0.006106	-0.025612
	-0.047076	-0.072775	0.015575	0.011875	0.033669	0.010769
	0.010670	-0.001155	-0.019588	-0.008322	0.028562	-0.034433
	-0.028257					
y188	-0.007549	0.002862	-0.022735	-0.004985	0.032616	0.024146
	-0.001769	0.004166	0.004570	0.011218	0.010439	-0.022263
	-0.015938	-0.021999	-0.073647	-0.014000	-0.018548	0.009070
	-0.020643	0.049991	-0.073041	-0.029005	0.004692	-0.062177
	0.021648	0.001679	0.010150	-0.064875	-0.012308	0.003552
	0.020116					

z188	0.011768	-0.023565	-0.054005	-0.026476	-0.027286	-0.007581
	-0.049135	-0.023690	-0.026603	-0.037199	0.039500	-0.022242
	0.036942	-0.020817	-0.039070	0.030630	0.010091	-0.031869
	0.029525	-0.100420	0.011990	0.045489	-0.026048	0.015941
	-0.020720	0.019153	-0.009054	-0.033674	0.022647	-0.002338
	-0.004259					
x189	-0.000863	0.005200	-0.036188	-0.053350	-0.014102	0.007650
	-0.056792	-0.016082	-0.023664	0.013804	0.009332	-0.007147
	0.043130	-0.074652	-0.016395	-0.005068	0.008153	-0.005921
	-0.029158	-0.059999	0.027684	0.025819	0.032287	0.016253
	0.004709	0.001588	-0.019059	-0.006007	0.029844	-0.013983
	-0.016411					
y189	-0.003922	0.005598	-0.019991	-0.007318	0.029049	0.018748
	-0.006566	0.012888	0.008771	-0.000757	0.009929	-0.019057
	-0.033934	-0.026115	-0.090998	-0.017299	-0.022984	0.030978
	-0.047387	0.042774	-0.062544	-0.044708	0.021128	-0.060575
	0.038822	-0.014053	-0.028253	-0.079173	-0.002801	0.021671
	-0.014520					
z189	0.018717	-0.023673	-0.055740	-0.019941	-0.021913	-0.010064
	-0.043980	-0.036725	-0.045266	-0.023949	0.024132	-0.049520
	0.059850	0.008267	-0.011775	0.006709	0.011364	-0.050259
	0.031221	-0.063647	0.002424	0.076914	-0.083086	0.017152
	-0.029424	0.028651	0.037946	-0.030653	0.004201	-0.007009
	0.037339					
x190	0.004380	0.007048	-0.024792	-0.045710	-0.025900	0.000811
	-0.051523	-0.028001	-0.014034	0.009475	0.013975	0.007805
	0.019158	-0.051821	-0.019635	-0.021876	0.015277	0.003080
	-0.007504	-0.042449	0.037801	0.030205	0.039312	0.009637
	0.010038	0.014069	-0.035845	-0.008012	0.044857	-0.003331
	-0.013305					
y190	-0.003957	0.012698	-0.022242	-0.005626	0.019132	0.015972
	-0.005622	0.015668	-0.007213	0.002374	0.016242	-0.021266
	-0.035134	-0.031518	-0.085278	-0.014850	-0.029311	0.050602
	-0.056999	0.059641	-0.025892	-0.027232	0.037795	-0.038873
	0.027641	-0.031903	-0.038853	-0.075820	-0.048040	0.051978
	-0.025377					
z190	0.019759	-0.020671	-0.041628	-0.003651	-0.004744	-0.002575
	-0.026470	-0.031009	-0.040141	-0.023254	0.026676	-0.035707
	0.014213	0.026844	0.014795	0.001252	-0.005159	-0.031887
	0.028178	-0.012873	-0.015134	0.044951	-0.081976	0.005017
	-0.012600	0.012071	0.011161	-0.013383	0.014821	0.013818
	-0.004734					

x191	0.007247	0.009562	-0.017696	-0.048433	-0.027730	-0.003628
	-0.054448	-0.040810	-0.019232	0.027174	0.012400	0.013880
	0.020270	-0.041104	-0.024502	-0.030082	0.015192	0.014226
	0.009027	-0.018872	0.043358	0.036197	0.038331	0.006465
	-0.001596	0.012740	0.009476	0.007634	0.051410	-0.001610
	0.012997					
y191	-0.004202	0.014993	-0.019607	-0.005162	0.014226	0.008769
	-0.007801	0.015812	-0.012012	-0.003120	0.022833	-0.017674
	-0.032335	-0.031722	-0.089007	-0.001792	-0.038247	0.056206
	-0.055531	0.042820	-0.016676	-0.018479	0.048931	-0.030650
	0.017855	-0.023989	-0.044882	-0.057117	-0.060377	0.046652
	-0.021010					
z191	0.018824	-0.018092	-0.031944	0.004783	-0.003812	0.000202
	-0.014070	-0.026183	-0.029610	-0.042771	0.023038	-0.014889
	-0.007173	0.022824	0.025596	0.005834	-0.011510	-0.009270
	0.013828	-0.018461	-0.023867	0.017721	-0.055178	-0.003340
	-0.012062	0.015917	-0.027929	0.003530	0.035364	0.028050
	-0.002201					
x192	0.013075	0.010981	-0.011838	-0.033976	-0.028181	-0.000866
	-0.040338	-0.045008	-0.017621	0.025927	0.014965	0.028858
	0.003654	-0.032332	-0.019500	-0.034779	0.016324	0.022234
	0.017026	0.001649	0.044346	0.026375	0.041222	0.001675
	-0.010016	0.009921	0.003373	-0.003167	0.055432	0.028610
	0.003234					
y192	0.001760	0.018220	-0.007546	-0.012128	0.012044	-0.006489
	-0.016114	0.018985	-0.019633	-0.008181	0.020872	-0.013998
	-0.028319	-0.029710	-0.093634	0.005361	-0.034581	0.054689
	-0.033883	0.032887	-0.006156	-0.006095	0.061564	-0.014977
	0.002571	-0.021838	-0.038157	-0.032137	-0.051560	0.043392
	-0.008544					
z192	0.022362	-0.015953	-0.028345	0.012128	0.001587	-0.003258
	-0.005781	-0.014711	-0.033229	-0.054795	0.014668	0.009741
	-0.017971	0.017111	0.022883	-0.000374	-0.000086	0.005544
	0.000272	-0.007785	0.003537	0.008543	-0.035353	-0.012142
	-0.012885	0.008758	-0.043178	0.017358	0.057608	0.033528
	0.027115					
x193	0.018447	0.008518	-0.015091	-0.017353	-0.028476	0.006032
	-0.020229	-0.050424	-0.022980	0.034953	0.029384	0.034847
	-0.007214	-0.029685	-0.014675	-0.033429	0.011152	0.027092
	0.019522	0.005586	0.031380	0.013545	0.029560	0.017830
	-0.025156	0.021540	-0.000767	-0.026958	0.044713	0.038196
	-0.011120					

y193	0.011947	0.026415	0.008935	-0.017781	0.008159	-0.020733
	-0.022051	0.017384	-0.029170	-0.018719	0.007151	-0.006388
	-0.029365	-0.023561	-0.089370	0.007365	-0.031196	0.059555
	-0.012224	0.028131	-0.018180	-0.005689	0.073416	-0.001958
	-0.012258	-0.020830	-0.023345	-0.026752	-0.042426	0.045319
	-0.025013					
z193	0.027244	-0.015008	-0.027656	0.016571	0.000302	-0.005954
	0.004262	-0.006004	-0.036123	-0.062067	0.007421	0.021949
	-0.018957	0.015532	0.026899	-0.005128	0.006620	0.012963
	-0.010052	-0.000866	0.024160	0.008376	-0.027236	-0.012912
	-0.014573	0.012380	-0.040081	0.019507	0.059251	0.031474
	0.011714					
x194	0.022849	0.002571	-0.021798	0.003136	-0.022241	0.013677
	0.004515	-0.054049	-0.026244	0.045332	0.057067	0.034550
	-0.032902	-0.033039	-0.012981	-0.014829	0.009257	0.010759
	0.013482	0.010067	0.002589	-0.003584	0.005453	0.029166
	-0.030303	0.027673	-0.008660	-0.048083	0.014663	0.044842
	-0.010486					
y194	0.025021	0.038405	0.037569	-0.027613	0.002700	-0.040931
	-0.032409	0.013270	-0.041226	-0.039927	-0.012752	0.003380
	-0.043127	-0.011044	-0.072866	0.021074	-0.023570	0.056123
	0.014452	0.028812	-0.051543	-0.010515	0.073702	0.009037
	-0.027528	-0.025004	0.000401	-0.020489	-0.040631	0.073323
	-0.026906					
z194	0.030331	-0.011953	-0.028041	0.027192	0.001295	-0.007191
	0.022665	0.006107	-0.037602	-0.069740	-0.011551	0.029696
	-0.029087	0.009830	0.035882	-0.004796	0.027254	0.023570
	-0.017946	0.015430	0.028108	0.005254	-0.027332	-0.004739
	-0.021411	0.008702	-0.013986	0.020941	0.052999	0.048470
	-0.004571					
x195	0.025200	-0.003412	-0.031970	0.032230	-0.020452	0.019889
	0.034132	-0.066549	-0.028507	0.065742	0.097202	0.028843
	-0.073659	-0.031430	-0.012399	0.006712	-0.006737	-0.023862
	-0.010734	0.006403	-0.055769	-0.024409	-0.040103	0.019446
	-0.008429	0.020737	0.000567	-0.036848	-0.034701	0.027131
	-0.004995					
y195	0.035517	0.043427	0.074842	-0.054902	-0.010136	-0.068128
	-0.044503	0.001454	-0.058150	-0.053377	-0.012987	0.002097
	-0.051525	0.012100	-0.025116	0.064368	-0.007898	0.025461
	0.029465	0.010769	-0.048096	0.011892	0.036610	-0.001805
	-0.020556	-0.003474	-0.000127	0.015757	-0.026748	0.067299
	0.009991					

z195	0.031045	-0.011082	-0.042573	0.042156	0.003045	-0.007239
	0.054367	0.025428	-0.038102	-0.060601	-0.030275	0.034476
	-0.031512	-0.013394	0.038133	0.007723	0.054490	0.027292
	-0.033980	0.026668	0.039735	0.012400	-0.039713	-0.003516
	-0.019250	-0.006000	0.023863	0.038736	0.043160	0.027252
	-0.009217					
x196	0.025463	-0.005898	-0.035746	0.055308	-0.024837	0.013302
	0.050308	-0.082785	-0.038488	0.069881	0.125568	0.018211
	-0.093066	-0.021450	-0.005538	0.018621	-0.012190	-0.071147
	-0.027921	0.018047	-0.080928	-0.028452	-0.028408	0.018437
	-0.010766	0.018918	-0.007457	-0.008201	-0.068175	-0.018855
	-0.024217					
y196	0.032444	0.033908	0.084830	-0.071268	-0.019456	-0.071512
	-0.042139	-0.004829	-0.054743	-0.049986	-0.000511	-0.011169
	-0.058040	0.040558	0.037007	0.083921	0.014615	-0.002241
	0.005688	0.006114	-0.021436	0.026789	0.014375	-0.021146
	-0.018539	-0.001828	0.026704	0.036120	-0.030935	0.003894
	-0.011623					
z196	0.025618	-0.014537	-0.059659	0.057991	0.002876	-0.000735
	0.075980	0.036936	-0.026717	-0.038672	-0.034894	0.021520
	-0.030686	-0.027150	0.045818	0.034878	0.057979	0.003237
	-0.033682	0.036203	0.018347	0.017227	-0.036438	-0.010563
	-0.018107	-0.012433	0.045266	0.055177	0.023139	-0.014910
	-0.051313					
x197	0.077458	-0.036294	-0.111644	-0.165397	-0.035489	-0.040716
	0.067258	-0.159899	0.008469	0.024545	-0.060004	-0.021166
	0.072973	0.046493	-0.035991	-0.024768	-0.015343	0.009049
	-0.003062	0.053976	-0.045611	0.022929	-0.000800	0.001508
	0.032673	-0.051937	0.090067	-0.043200	-0.018529	0.026409
	0.004549					
y197	-0.114101	0.043267	-0.029785	0.062898	0.058382	0.028243
	-0.020619	-0.052664	0.057267	-0.023650	0.225471	0.052680
	0.113466	0.131431	0.004687	0.019563	-0.044739	-0.063504
	-0.018574	0.026345	-0.070240	0.079501	0.023717	-0.000632
	0.094269	-0.040311	0.023500	-0.023442	-0.008375	0.040203
	-0.010475					
z197	-0.126101	-0.039232	0.029425	0.049784	-0.035220	-0.022725
	0.102875	-0.096856	-0.122113	-0.016123	-0.076512	0.119738
	-0.008505	0.069705	-0.028529	0.088524	-0.096965	0.072131
	0.001402	-0.008372	-0.000992	-0.109356	-0.042663	0.034836
	-0.041889	-0.011527	-0.031306	-0.122050	-0.002369	-0.029180
	0.039826					

x198	0.077488	-0.036501	-0.104745	-0.168001	-0.041509	-0.040803
	0.069745	-0.156765	0.000521	0.034878	-0.069172	-0.019765
	0.069874	0.044277	-0.042875	-0.022368	-0.004381	0.005538
	0.002375	0.042478	-0.052709	0.037105	-0.023468	-0.002190
	0.026363	-0.045755	0.059758	-0.038667	-0.018250	0.021990
	0.018432					
y198	-0.106774	0.045946	-0.030702	0.063108	0.058551	0.030774
	-0.024177	-0.050619	0.051368	-0.029994	0.195246	0.059886
	0.105887	0.112295	0.005442	0.013063	-0.035820	-0.056076
	-0.016198	0.012852	-0.072819	0.063852	0.034587	-0.015392
	0.083042	-0.039448	0.019508	-0.013728	-0.012012	0.015266
	-0.023491					
z198	-0.117112	-0.036015	0.026672	0.057663	-0.027456	-0.024729
	0.085828	-0.085647	-0.109178	-0.021708	-0.075040	0.107525
	-0.006779	0.058920	-0.030930	0.075939	-0.092960	0.061971
	-0.008657	-0.015143	-0.010355	-0.106340	-0.029152	0.022661
	-0.025961	-0.014208	0.008685	-0.096524	0.000476	-0.028073
	0.039706					
x199	0.075604	-0.034250	-0.093238	-0.168852	-0.042063	-0.039492
	0.072544	-0.150024	-0.003544	0.042807	-0.072328	-0.019736
	0.064431	0.042367	-0.045981	-0.009226	0.007270	-0.002760
	0.009703	0.029884	-0.063172	0.040771	-0.036573	0.003687
	0.023255	-0.021147	0.039626	-0.031224	-0.015920	0.023513
	0.034160					
y199	-0.100239	0.047967	-0.029972	0.061600	0.057572	0.034273
	-0.028268	-0.045118	0.047748	-0.033810	0.159711	0.069893
	0.092966	0.091857	0.005973	0.003327	-0.025138	-0.048687
	-0.022462	-0.000009	-0.063461	0.048314	0.042811	-0.033225
	0.062541	-0.040722	0.021519	0.001141	-0.017345	-0.009153
	-0.020083					
z199	-0.107362	-0.032910	0.022624	0.065271	-0.016374	-0.027639
	0.067866	-0.077907	-0.095807	-0.032026	-0.070980	0.097187
	0.000086	0.050591	-0.033218	0.055696	-0.095641	0.049474
	-0.018204	-0.021324	-0.011934	-0.093644	-0.026387	0.016211
	-0.007841	-0.023601	0.033168	-0.090492	0.001986	-0.030922
	0.025126					
x200	0.075686	-0.030676	-0.079501	-0.160430	-0.040086	-0.037220
	0.069800	-0.132409	0.002605	0.045675	-0.067861	-0.021146
	0.058748	0.043158	-0.036337	-0.006899	0.007956	-0.013477
	0.017749	0.035999	-0.055221	0.032566	-0.023083	0.012057
	0.021357	-0.007608	0.026677	-0.030405	-0.016298	0.028872
	0.038331					

y200	-0.087861	0.045776	-0.028842	0.061689	0.055176	0.032841
	-0.034641	-0.040703	0.035680	-0.034595	0.121847	0.078088
	0.086265	0.071787	-0.002280	0.000883	-0.017448	-0.042164
	-0.028551	-0.012797	-0.063491	0.026279	0.032715	-0.019103
	0.046450	-0.027253	-0.000020	0.015900	-0.001467	0.002547
	-0.011709					
z200	-0.098813	-0.028420	0.019901	0.071375	-0.000184	-0.028665
	0.048303	-0.074231	-0.086328	-0.040321	-0.070259	0.092015
	0.005947	0.032054	-0.037872	0.043887	-0.090022	0.052697
	-0.028612	-0.049833	-0.028710	-0.064151	-0.032870	0.000055
	0.003228	-0.034751	0.047887	-0.064557	-0.004076	-0.030896
	0.043430					
x201	0.072566	-0.027707	-0.064137	-0.144492	-0.032350	-0.030638
	0.064371	-0.116586	0.008399	0.042686	-0.061013	-0.015274
	0.048928	0.038104	-0.030303	-0.007354	0.005892	-0.011516
	0.021062	0.032963	-0.055554	0.035816	-0.008367	0.016875
	0.017390	0.001021	0.016162	-0.028960	-0.021008	0.034722
	0.041170					
y201	-0.076743	0.040391	-0.023549	0.058126	0.054210	0.030572
	-0.036888	-0.036445	0.024065	-0.032958	0.083724	0.079569
	0.076420	0.050919	-0.005841	-0.006064	-0.003782	-0.037626
	-0.027121	-0.028868	-0.056045	0.022700	0.026768	-0.006979
	0.038300	-0.019287	-0.021752	0.015973	0.009862	0.011924
	0.004890					
z201	-0.085461	-0.026044	0.012684	0.073718	0.011199	-0.039114
	0.031746	-0.073015	-0.085267	-0.048692	-0.063987	0.075808
	0.018873	0.029182	-0.039114	0.022945	-0.080082	0.045596
	-0.050489	-0.056424	-0.028285	-0.051868	-0.026998	0.009620
	0.017680	-0.025243	0.045039	-0.049800	0.021056	-0.012200
	0.050035					
x202	0.066462	-0.023578	-0.044160	-0.124934	-0.020297	-0.019725
	0.059022	-0.096921	0.012057	0.034330	-0.052440	-0.011122
	0.040050	0.030958	-0.022659	-0.008571	0.005559	-0.002547
	0.011487	0.023095	-0.053811	0.039236	0.002887	0.023417
	0.012828	0.005220	0.009750	-0.026772	-0.012562	0.026872
	0.043676					
y202	-0.067593	0.031140	-0.018889	0.051836	0.052813	0.031030
	-0.037002	-0.034262	0.019549	-0.027340	0.054527	0.080474
	0.062614	0.028228	-0.002441	-0.011988	0.009318	-0.033814
	-0.019644	-0.031024	-0.043637	0.020853	0.023401	-0.003258
	0.028736	-0.012173	-0.041978	0.014733	0.019184	0.003940
	0.016553					

z202	-0.074467	-0.026527	0.002285	0.074738	0.024783	-0.049716
	0.013177	-0.072637	-0.067887	-0.050272	-0.061323	0.057885
	0.030240	0.016268	-0.038973	-0.007053	-0.056396	0.050326
	-0.051947	-0.062716	-0.023293	-0.021407	-0.001871	0.009814
	0.027659	-0.008891	0.051191	-0.038807	0.048544	-0.011094
	0.065196					
x203	0.055251	-0.019846	-0.022212	-0.102291	-0.005040	-0.005638
	0.050822	-0.077334	0.012389	0.026762	-0.038126	-0.005612
	0.027030	0.018595	-0.010297	-0.004333	0.007486	0.003482
	-0.002442	0.013657	-0.043335	0.041467	0.010240	0.021788
	0.014431	0.012270	-0.008784	-0.015665	-0.005242	0.010278
	0.036545					
y203	-0.056778	0.016241	-0.016477	0.041758	0.044838	0.029392
	-0.034559	-0.034305	0.021743	-0.022889	0.040187	0.076661
	0.044131	0.016513	0.007880	-0.009149	0.021733	-0.028272
	-0.012096	-0.017731	-0.032292	0.000900	0.015437	-0.000614
	0.014267	0.002507	-0.051316	0.016153	0.013853	-0.010428
	0.031635					
z203	-0.070116	-0.025865	-0.011607	0.069751	0.037682	-0.048796
	0.000765	-0.077687	-0.051356	-0.039537	-0.066270	0.043370
	0.033994	-0.002902	-0.024449	-0.009256	-0.013582	0.035858
	-0.056157	-0.079846	-0.033780	0.025516	-0.001002	0.003569
	0.021554	0.005921	0.012979	-0.029464	0.027513	-0.059055
	0.023647					
x204	0.038079	-0.017020	0.005373	-0.078869	0.012980	0.010173
	0.043936	-0.057825	0.008139	0.016354	-0.017805	0.006588
	0.009502	0.006245	0.000012	-0.003527	0.009750	0.010181
	-0.024027	-0.000258	-0.032214	0.043130	0.014564	0.012681
	0.011209	0.021276	-0.027236	0.002158	0.003113	-0.002503
	0.037710					
y204	-0.046614	-0.001433	-0.010606	0.030061	0.039433	0.027859
	-0.026880	-0.034680	0.026809	-0.010279	0.027492	0.069018
	0.016103	0.009641	0.021157	-0.007015	0.033160	-0.030314
	-0.003736	-0.007615	-0.020657	-0.006047	0.001153	0.000593
	0.002775	0.000939	-0.047260	0.014591	-0.007369	-0.034689
	0.045647					
z204	-0.065039	-0.025703	-0.023011	0.051824	0.044105	-0.048513
	0.002434	-0.080609	-0.035096	-0.032596	-0.068315	0.021233
	0.033519	-0.006071	-0.007986	-0.021159	0.011804	0.011804
	-0.056644	-0.079871	-0.050832	0.046177	-0.006917	-0.003571
	-0.020864	-0.006156	-0.016684	-0.020162	0.013318	-0.090242
	-0.005356					

x205	0.018055	-0.012748	0.037459	-0.052809	0.034858	0.022693
	0.036387	-0.037526	-0.000277	0.004828	0.005297	0.027065
	-0.013788	-0.009571	0.005330	-0.005046	0.005179	0.029636
	-0.049280	-0.014955	-0.013953	0.045115	0.022995	0.003767
	0.010160	0.040009	-0.039145	0.020655	0.005559	-0.010270
	0.038784					
y205	-0.035758	-0.020221	-0.005053	0.020271	0.036031	0.023485
	-0.018028	-0.035224	0.030765	0.011830	0.015778	0.056959
	-0.014535	0.003229	0.034382	-0.003234	0.040192	-0.034253
	0.003009	0.008592	0.002427	-0.009072	-0.010669	0.006041
	-0.011441	-0.023054	-0.037787	0.004366	-0.033720	-0.060708
	0.059907					
z205	-0.052707	-0.024369	-0.026884	0.032081	0.048135	-0.053053
	0.008293	-0.079004	-0.023441	-0.031178	-0.063636	0.010977
	0.028579	-0.013693	-0.005771	-0.044733	0.012831	0.013913
	-0.054851	-0.067134	-0.059358	0.067637	-0.011563	0.002228
	-0.029838	-0.012927	-0.013909	-0.017740	-0.010209	-0.081259
	-0.032408					
x206	-0.003881	-0.007049	0.068035	-0.027394	0.057061	0.027476
	0.028598	-0.017953	-0.012791	-0.002634	0.024881	0.052545
	-0.039904	-0.029321	0.011796	0.002335	-0.007493	0.060119
	-0.068654	-0.027905	0.013938	0.038478	0.044766	-0.004921
	0.011974	0.072042	-0.053978	0.027903	0.001947	-0.015003
	0.041679					
y206	-0.025339	-0.040831	-0.000618	0.010525	0.032477	0.020163
	-0.007567	-0.037131	0.036126	0.032925	0.010737	0.041489
	-0.043084	0.000132	0.050088	-0.005484	0.044931	-0.037559
	0.008245	0.031327	0.021068	-0.005341	-0.025741	0.017672
	-0.024405	-0.047221	-0.027278	-0.020337	-0.067037	-0.091639
	0.058170					
z206	-0.042750	-0.022093	-0.019661	0.015771	0.046669	-0.055522
	0.014989	-0.073678	-0.012361	-0.023130	-0.061777	0.012795
	0.007586	-0.020669	0.012664	-0.037164	-0.000706	0.022236
	-0.044628	-0.032694	-0.047414	0.069916	-0.003122	-0.011983
	-0.027733	-0.015306	-0.006465	-0.017421	-0.025230	-0.075953
	-0.024967					
x207	-0.003688	0.004305	0.067344	-0.017783	0.043351	0.038131
	0.027263	0.001510	-0.016180	-0.002437	0.018743	0.041242
	-0.023249	-0.010094	0.012820	-0.014764	-0.011253	0.048327
	-0.050816	-0.005679	0.011551	0.026614	0.035927	-0.012890
	0.029306	0.058664	-0.036155	0.023256	-0.002801	-0.017450
	0.025738					

y207	-0.023491	-0.046932	0.012181	0.001607	0.025501	0.016865
	-0.005246	-0.031673	0.042433	0.030459	0.012881	0.034225
	-0.044337	-0.007185	0.040538	0.023274	0.044431	-0.039807
	-0.014617	0.036432	0.026095	-0.005249	-0.028536	-0.012611
	-0.027025	-0.063680	0.000910	-0.024706	-0.067053	-0.068598
	0.036858					
z207	-0.044726	-0.020949	-0.002720	0.002092	0.052541	-0.052146
	0.018949	-0.063634	0.001910	-0.019323	-0.058530	0.013349
	-0.007664	-0.034796	0.022755	-0.033643	-0.019883	0.035421
	-0.027829	-0.022264	-0.036196	0.070169	-0.014123	-0.052340
	-0.012517	-0.019804	-0.007196	-0.023940	-0.052064	-0.061171
	0.020165					
x208	-0.000520	0.018289	0.058103	-0.010891	0.029822	0.039718
	0.024049	0.014534	-0.020267	-0.000270	0.010102	0.030324
	-0.011849	0.009675	0.012178	-0.024934	-0.008696	0.042293
	-0.032405	0.005799	0.006506	0.017547	0.027223	-0.016962
	0.038210	0.054901	-0.022472	0.013823	-0.009112	-0.024642
	0.014223					
y208	-0.021356	-0.050403	0.021327	-0.002139	0.019181	0.017134
	-0.005323	-0.026157	0.042016	0.027646	0.012326	0.030146
	-0.039365	-0.007514	0.037240	0.037813	0.041229	-0.038742
	-0.027850	0.036724	0.032394	0.001031	-0.021041	-0.026530
	-0.021196	-0.070609	0.015735	-0.031028	-0.063522	-0.052699
	0.027074					
z208	-0.048722	-0.016281	0.006484	-0.009788	0.052424	-0.046619
	0.019838	-0.050718	0.011650	-0.019165	-0.038882	0.012840
	-0.014888	-0.042968	0.025231	-0.027148	-0.032293	0.036052
	-0.006879	-0.018096	-0.032109	0.058962	-0.018201	-0.066368
	-0.019267	-0.010958	-0.005465	-0.017526	-0.042847	-0.048059
	0.004120					
x209	0.001436	0.030783	0.048308	-0.005782	0.018153	0.035422
	0.020067	0.021269	-0.022329	0.001465	-0.001119	0.022888
	-0.007312	0.027618	0.012027	-0.032588	-0.001061	0.041842
	-0.021546	0.015044	0.008518	0.013342	0.021617	-0.025046
	0.043301	0.053012	-0.013831	-0.001344	-0.015780	-0.037978
	0.014459					
y209	-0.018462	-0.051225	0.025681	-0.001436	0.013565	0.019791
	-0.006416	-0.018837	0.036723	0.025834	0.013821	0.024030
	-0.032282	-0.006223	0.033156	0.041591	0.032939	-0.040553
	-0.027963	0.032183	0.025047	0.003220	-0.015134	-0.027553
	-0.012788	-0.066102	0.025966	-0.029137	-0.054362	-0.034991
	0.010146					

z209	-0.050326	-0.012926	0.015814	-0.017016	0.043754	-0.035215
	0.015496	-0.046225	0.016323	-0.021076	-0.024312	0.010141
	-0.016724	-0.032348	0.037779	-0.036661	-0.026492	0.030982
	0.007409	-0.007119	-0.014114	0.042118	-0.014408	-0.086148
	-0.010321	-0.021783	-0.000231	-0.014175	-0.027884	-0.041262
	0.019004					
x210	0.002267	0.041627	0.039378	-0.004270	0.009437	0.026906
	0.019647	0.027793	-0.025830	0.005277	-0.010103	0.017432
	-0.009690	0.040355	0.005695	-0.037902	0.009551	0.036743
	-0.018568	0.019722	0.005323	0.010703	0.019879	-0.037581
	0.038527	0.049068	0.000875	-0.007628	-0.024187	-0.050217
	0.016724					
y210	-0.015390	-0.050347	0.027964	0.005283	0.007581	0.024122
	-0.011319	-0.014369	0.032434	0.023579	0.010514	0.016731
	-0.017249	-0.004472	0.035131	0.039540	0.022120	-0.034972
	-0.016450	0.029637	0.019601	-0.000334	-0.015097	-0.025268
	0.002893	-0.059730	0.021914	-0.031029	-0.038076	-0.020358
	-0.006554					
z210	-0.058197	-0.006764	0.018821	-0.029552	0.034863	-0.031806
	0.009665	-0.037765	0.014048	-0.020606	-0.008806	0.011629
	-0.023517	-0.026745	0.039293	-0.042806	-0.030877	0.011375
	0.014954	-0.007489	0.010358	0.041101	0.009915	-0.056829
	-0.019496	-0.044493	-0.009735	-0.023237	0.012520	-0.037476
	0.004529					
x211	0.001870	0.050251	0.031499	-0.003042	0.002355	0.018208
	0.016308	0.032006	-0.023601	0.010620	-0.017815	0.014526
	-0.014564	0.045764	0.003300	-0.032033	0.019962	0.036196
	-0.020327	0.020933	0.006212	0.010859	0.016909	-0.045710
	0.037288	0.042520	0.012414	-0.023075	-0.036986	-0.060625
	0.014694					
y211	-0.011928	-0.046517	0.027950	0.012161	-0.000021	0.024796
	-0.014036	-0.010346	0.021717	0.019095	0.006312	0.008508
	-0.006767	0.003785	0.032089	0.030901	0.013417	-0.031811
	-0.001187	0.026790	0.009327	-0.002102	-0.014838	-0.020975
	0.013210	-0.048911	0.014211	-0.024195	-0.012987	-0.008720
	-0.013370					
z211	-0.060212	-0.004483	0.020676	-0.030468	0.034573	-0.024433
	0.000476	-0.036304	0.017093	-0.017863	0.004180	0.004594
	-0.024832	-0.026171	0.043373	-0.036387	-0.031808	0.005209
	0.007091	-0.005495	0.017316	0.033656	0.007417	-0.049218
	-0.019393	-0.046706	-0.006715	-0.027886	0.000198	-0.037017
	-0.011028					

x212	-0.001376	0.057573	0.025072	-0.006153	-0.003280	0.006025
	0.018779	0.037062	-0.022047	0.017369	-0.023902	0.013296
	-0.021897	0.050111	-0.002519	-0.025541	0.031210	0.029478
	-0.021162	0.020524	0.004528	0.011115	0.009568	-0.056023
	0.022246	0.028853	0.021877	-0.028961	-0.041574	-0.069553
	0.010519					
y212	-0.007980	-0.041133	0.026121	0.022012	-0.007468	0.024561
	-0.019626	-0.008251	0.010640	0.014235	0.000929	-0.000313
	-0.000601	0.011088	0.029881	0.021882	0.005820	-0.025174
	0.007437	0.021975	0.000137	-0.003492	-0.014284	-0.013545
	0.025927	-0.035325	0.008220	-0.024338	0.003762	-0.000003
	-0.020784					
z212	-0.062413	0.000544	0.024139	-0.035311	0.032094	-0.021417
	0.001976	-0.030945	0.013165	-0.013623	0.005866	0.001420
	-0.027839	-0.018413	0.035974	-0.034936	-0.022692	-0.013861
	0.024335	-0.005310	0.014786	0.020828	0.000144	-0.052772
	-0.024330	-0.048626	-0.009305	0.001499	0.010475	-0.028583
	-0.009486					
x213	-0.005401	0.061778	0.018388	-0.007140	-0.006210	-0.003521
	0.018289	0.041435	-0.018313	0.025063	-0.027353	0.011796
	-0.025878	0.048700	0.001439	-0.017255	0.038644	0.022860
	-0.024215	0.017860	-0.001306	0.010607	0.009603	-0.060116
	0.014500	0.012899	0.031685	-0.037524	-0.049194	-0.071296
	-0.001245					
y213	-0.004771	-0.033569	0.024504	0.030058	-0.016573	0.021214
	-0.024052	-0.006278	-0.001856	0.012581	-0.007674	-0.007779
	-0.000079	0.017942	0.024258	0.015559	0.002341	-0.020367
	0.013552	0.015213	-0.003899	-0.003924	-0.016981	-0.012308
	0.031796	-0.023411	0.002414	-0.019204	0.018498	0.004719
	-0.021608					
z213	-0.061745	0.003463	0.022253	-0.027707	0.033801	-0.016608
	-0.005842	-0.032024	0.008762	-0.016376	0.000781	-0.010132
	-0.025303	-0.020991	0.040079	-0.035623	-0.014150	-0.015352
	0.036601	-0.004005	0.002862	0.011445	-0.002624	-0.036258
	-0.005440	-0.054646	-0.005042	0.001750	-0.008875	-0.004765
	-0.028605					
x214	-0.012670	0.062431	0.012590	-0.008680	-0.009426	-0.008808
	0.017923	0.046954	-0.013330	0.034101	-0.027511	0.010737
	-0.021920	0.045502	0.005329	-0.016579	0.035983	0.016414
	-0.021153	0.016676	-0.008081	0.002885	0.014514	-0.059176
	-0.003369	-0.011316	0.030568	-0.042560	-0.045410	-0.067384
	-0.025782					

y214	-0.001182	-0.024825	0.022033	0.038037	-0.024577	0.014678
	-0.028838	-0.004880	-0.013290	0.012773	-0.016322	-0.014870
	-0.005406	0.023937	0.022586	0.014877	0.003828	-0.015038
	0.013599	0.003759	-0.007347	0.001399	-0.015405	-0.012374
	0.041720	-0.008024	0.004278	-0.013037	0.028810	0.005440
	-0.019159					
z214	-0.065110	0.006611	0.024173	-0.024037	0.031413	-0.003648
	-0.009045	-0.034560	0.002400	-0.013686	-0.001415	-0.022495
	-0.016359	-0.017442	0.031024	-0.033632	-0.021366	-0.013101
	0.051126	0.016998	0.002708	-0.002897	-0.019259	-0.026159
	-0.018925	-0.071233	-0.012669	0.003356	-0.002140	0.026884
	-0.040007					
x215	-0.020770	0.061092	0.005623	-0.006242	-0.010989	-0.013537
	0.017928	0.057130	-0.005810	0.047748	-0.015529	0.017196
	-0.006775	0.044383	0.011376	-0.021809	0.026717	0.009825
	-0.017829	0.013323	-0.024670	-0.003515	0.030432	-0.042913
	-0.017083	-0.030889	0.027702	-0.046745	-0.027933	-0.065772
	-0.051132					
y215	0.000663	-0.017129	0.019545	0.045220	-0.032007	0.006538
	-0.034759	-0.003958	-0.018397	0.015070	-0.023752	-0.019013
	-0.016086	0.026479	0.021440	0.017488	-0.001237	-0.006145
	0.012217	-0.011859	-0.008070	0.002708	-0.015636	-0.019562
	0.053155	0.001672	0.011823	-0.009232	0.030930	0.005674
	-0.038600					
z215	-0.064495	0.012072	0.026842	-0.014793	0.025606	0.004856
	-0.009173	-0.028613	-0.000158	0.005142	0.007418	-0.026905
	-0.006016	-0.004762	0.022787	-0.007457	-0.015860	-0.013995
	0.048486	0.032854	-0.005231	-0.003218	-0.022919	-0.012242
	-0.011841	-0.068283	0.002258	0.003306	0.000622	0.036340
	-0.019903					
x216	-0.024634	0.064326	0.000762	-0.007629	-0.013872	-0.012713
	0.016367	0.049145	0.001285	0.046744	-0.016180	0.021284
	-0.000645	0.030852	0.005731	-0.025126	0.033724	-0.008176
	-0.023042	0.024265	-0.021071	-0.005148	0.030999	-0.029680
	-0.002620	-0.021372	0.013560	-0.029916	-0.035728	-0.073749
	-0.052251					
y216	-0.003611	-0.005008	0.014371	0.037221	-0.029302	-0.007519
	-0.032494	0.002068	-0.012178	0.018187	-0.027484	-0.007430
	-0.010528	0.011328	0.030892	0.004691	0.014787	-0.016669
	0.001542	-0.009143	-0.002170	0.002041	-0.007254	-0.019404
	0.054428	0.002114	0.016535	-0.009333	0.013242	-0.006698
	-0.021754					

z216	-0.061234	0.015411	0.026073	-0.006753	0.020293	0.008068
	-0.011006	-0.025401	-0.008480	0.010099	0.008490	-0.029272
	0.007697	-0.012706	0.023020	0.000295	-0.001861	-0.030420
	0.028219	0.040099	-0.009616	0.010640	-0.034222	0.031201
	-0.022685	-0.070617	-0.017071	-0.014304	-0.011467	0.045775
	-0.048196					
x217	-0.024516	0.064560	-0.001027	-0.008498	-0.014029	-0.012870
	0.013429	0.040394	0.007288	0.045833	-0.018349	0.030242
	0.001776	0.024437	0.004626	-0.027167	0.037598	-0.012145
	-0.020610	0.034103	-0.011734	-0.000106	0.030617	-0.021517
	0.002589	-0.012745	0.002983	-0.012517	-0.041868	-0.095042
	-0.033486					
y217	-0.007042	0.004532	0.009102	0.029877	-0.024575	-0.020944
	-0.031160	0.007903	-0.003601	0.020910	-0.027850	0.008734
	-0.006584	0.003810	0.041965	-0.008673	0.022877	-0.021996
	-0.005192	-0.006374	-0.001566	0.000891	-0.002627	-0.014988
	0.052591	0.001381	0.017534	-0.002385	-0.003022	-0.020634
	-0.011518					
z217	-0.053430	0.016166	0.026961	-0.000407	0.016965	0.009501
	-0.011531	-0.026513	-0.005770	0.014289	0.013382	-0.019866
	0.012582	-0.017219	0.014413	0.010204	0.003834	-0.023756
	0.029018	0.046869	-0.008950	0.021483	-0.039876	0.044740
	-0.035102	-0.064803	-0.024898	0.005334	-0.012532	0.004200
	-0.022588					
x218	-0.023498	0.063253	-0.001504	-0.007726	-0.011298	-0.012274
	0.011263	0.034619	0.008616	0.043442	-0.020356	0.032330
	0.000603	0.023124	-0.001195	-0.028680	0.039667	-0.015859
	-0.020272	0.038894	-0.008542	0.003011	0.025612	-0.012468
	0.001236	0.002169	-0.001305	-0.004765	-0.048042	-0.088043
	-0.024562					
y218	-0.010301	0.012860	0.004855	0.022142	-0.018446	-0.034325
	-0.025686	0.013929	0.002307	0.022142	-0.027456	0.021412
	-0.005682	0.002721	0.047510	-0.023087	0.029804	-0.029359
	-0.010954	-0.005081	-0.007612	-0.004257	-0.005310	-0.007249
	0.034667	0.001548	0.020389	0.006409	-0.010504	-0.018891
	-0.010286					
z218	-0.043549	0.015464	0.024735	0.009024	0.013597	0.011717
	-0.022127	-0.027130	-0.006018	0.019790	0.014467	-0.021121
	0.019698	-0.026523	0.004830	0.016158	0.002911	-0.019116
	0.027733	0.051033	0.004322	0.033753	-0.034129	0.038740
	-0.009197	-0.040400	-0.029434	-0.014717	-0.010567	0.009247
	0.002864					

x219	-0.022919	0.059464	-0.001533	-0.005096	-0.007760	-0.007065
	0.008423	0.029317	0.007427	0.040350	-0.021523	0.031391
	-0.003115	0.024835	-0.005711	-0.017673	0.038787	-0.012626
	-0.021344	0.036799	-0.007766	0.008974	0.019789	-0.002391
	0.000914	0.008870	-0.004373	0.006434	-0.046558	-0.072288
	-0.013448					
y219	-0.012212	0.018642	0.001559	0.017109	-0.010870	-0.044963
	-0.023328	0.017800	0.007608	0.023048	-0.028367	0.033822
	-0.005087	0.002168	0.055970	-0.032900	0.032721	-0.030838
	-0.016632	-0.006629	-0.009383	-0.006219	-0.004343	-0.000135
	0.023906	0.000664	0.022089	0.009611	-0.015285	-0.009005
	-0.003320					
z219	-0.037570	0.015146	0.021576	0.016104	0.007878	0.018398
	-0.026010	-0.030849	-0.007741	0.022212	0.017745	-0.026797
	0.016018	-0.036474	-0.005087	0.031340	-0.005695	-0.011855
	0.026950	0.039922	0.018961	0.048542	-0.031110	0.040363
	0.012668	-0.035678	-0.014944	-0.013564	0.001912	0.013712
	0.034612					
x220	-0.021290	0.054734	-0.000782	-0.003003	-0.004516	0.003633
	0.004913	0.022243	0.002815	0.037892	-0.028571	0.029399
	-0.006430	0.026917	-0.014428	-0.004713	0.037348	-0.007240
	-0.020831	0.037612	-0.006339	0.012101	0.018264	0.006152
	0.000760	0.011733	-0.002959	0.016703	-0.044558	-0.040861
	-0.012403					
y220	-0.013291	0.021449	-0.000374	0.014558	-0.002293	-0.054029
	-0.023755	0.019202	0.010716	0.025191	-0.032416	0.046479
	-0.002934	0.001664	0.060989	-0.036940	0.035643	-0.027815
	-0.020311	-0.007839	-0.010343	-0.006549	-0.000394	0.006982
	0.018886	0.005355	0.022287	0.008675	-0.018236	0.012333
	0.002120					
z220	-0.028014	0.017280	0.020960	0.015237	0.002144	0.024394
	-0.025850	-0.032377	-0.016953	0.028636	0.010705	-0.032627
	0.016697	-0.043380	-0.013445	0.041643	-0.010503	-0.007165
	0.035265	0.046127	0.018516	0.049520	-0.025376	0.038000
	0.013727	-0.027509	-0.006700	-0.002142	0.016033	0.047686
	0.030865					
x221	-0.020030	0.047356	-0.001772	-0.001855	-0.001098	0.014677
	0.002599	0.014981	-0.000105	0.036210	-0.035484	0.030936
	-0.008573	0.024058	-0.026056	0.010300	0.036675	-0.004503
	-0.024038	0.035182	0.000226	0.018399	0.009403	0.020402
	0.007139	0.011451	-0.001499	0.019052	-0.041286	-0.028545
	-0.010930					

y221	-0.014123	0.023024	-0.002903	0.010370	0.006674	-0.060500
	-0.020887	0.021443	0.012986	0.028668	-0.033088	0.059200
	-0.002510	0.002940	0.060720	-0.039195	0.036925	-0.027141
	-0.020949	-0.009601	-0.012695	-0.003516	-0.001898	0.018252
	0.011228	0.001594	0.021881	0.004262	-0.019320	0.022492
	0.003906					
z221	-0.017694	0.014535	0.017378	0.013529	0.003226	0.018811
	-0.032590	-0.033538	-0.015762	0.031602	0.002542	-0.031597
	0.016797	-0.064210	-0.008953	0.035689	-0.008739	0.001145
	0.019486	0.041488	0.031404	0.056523	-0.025753	0.046458
	0.015735	-0.012909	-0.001365	-0.012965	0.013112	0.031189
	0.038243					
x222	-0.020701	0.040329	-0.002263	0.001073	-0.001255	0.031074
	-0.000827	0.009246	-0.005380	0.032955	-0.039600	0.029663
	-0.010043	0.023708	-0.040413	0.027428	0.031656	-0.005183
	-0.029941	0.027896	0.003920	0.019441	0.004080	0.025158
	0.018550	0.009201	-0.001979	0.028198	-0.031987	-0.009660
	-0.011199					
y222	-0.016473	0.022260	-0.005694	0.008483	0.013917	-0.061036
	-0.020448	0.020615	0.016712	0.033311	-0.032370	0.067767
	-0.001422	0.002257	0.055521	-0.032620	0.036370	-0.025534
	-0.023290	-0.012737	-0.012775	0.002729	0.001996	0.022905
	0.014611	-0.001162	0.020247	0.000206	-0.020647	0.045031
	0.007473					
z222	-0.011012	0.012746	0.017566	0.012078	0.004733	0.013547
	-0.038916	-0.027624	-0.021039	0.037075	0.001866	-0.026688
	0.014804	-0.070122	-0.004737	0.025700	-0.007568	0.000429
	-0.002148	0.030157	0.023839	0.045988	-0.021091	0.039514
	0.024992	-0.010626	0.000187	-0.010211	0.017102	0.040915
	0.038645					
x223	-0.021775	0.034439	-0.003346	0.002575	-0.003092	0.048308
	-0.002590	0.001461	-0.011342	0.025822	-0.043923	0.027314
	-0.009340	0.027030	-0.051392	0.045177	0.023791	-0.008550
	-0.033125	0.021890	0.009056	0.018691	0.001638	0.023273
	0.029305	-0.001036	-0.000835	0.038555	-0.009024	-0.006864
	-0.011968					
y223	-0.019780	0.020791	-0.010989	0.004658	0.019885	-0.056311
	-0.017661	0.014986	0.020876	0.036602	-0.028843	0.075952
	0.000405	0.002014	0.045405	-0.018719	0.033761	-0.024689
	-0.019808	-0.016581	-0.012566	0.016971	0.003943	0.021206
	0.016230	-0.018475	0.015155	-0.002438	-0.009773	0.048799
	0.012345					

z223	-0.005788	0.013511	0.016907	0.000772	0.011197	-0.001763
	-0.034374	-0.022553	-0.027095	0.035671	0.006263	-0.027494
	0.012505	-0.057801	0.003539	0.010458	-0.010290	-0.007476
	-0.009251	0.020890	0.012229	0.040252	-0.027752	0.042471
	0.003790	-0.034425	0.014269	0.003694	0.039639	0.032176
	0.035091					
x224	-0.022203	0.028879	-0.003994	0.006804	-0.010077	0.065551
	-0.003726	-0.005659	-0.013685	0.014253	-0.050217	0.022452
	-0.007003	0.027281	-0.057129	0.063708	0.017446	-0.009653
	-0.033497	0.022948	0.016941	0.012756	0.006464	0.008843
	0.051609	0.005844	-0.001585	0.044281	0.012713	-0.010221
	-0.019633					
y224	-0.025787	0.019085	-0.018376	0.001280	0.022349	-0.043942
	-0.015485	0.006237	0.026322	0.041360	-0.026834	0.077613
	0.003088	-0.005525	0.027391	0.000224	0.029256	-0.025265
	-0.015670	-0.018990	-0.008523	0.025286	0.006376	0.011855
	0.031993	-0.025381	0.006746	-0.012137	-0.007713	0.062921
	0.005352					
z224	0.000840	0.008283	0.019571	-0.001747	0.015060	-0.016069
	-0.037531	-0.020528	-0.016721	0.033225	0.001500	-0.030264
	0.009476	-0.059128	0.019593	-0.005176	-0.009075	0.002157
	-0.014009	0.006399	0.007723	0.037427	-0.028657	0.039621
	0.011520	-0.018908	0.019497	-0.019636	0.027095	0.032398
	0.024377					
x225	-0.014653	0.024948	0.001829	0.002742	-0.013200	0.058226
	-0.009161	-0.003449	-0.004138	0.016784	-0.042775	0.011425
	-0.006020	0.022967	-0.037788	0.059420	0.010329	-0.008224
	-0.030007	0.006869	0.013486	0.009860	0.005617	0.018184
	0.053238	0.000586	0.013504	0.024040	0.017509	-0.013887
	-0.005734					
y225	-0.029898	0.014901	-0.019935	0.000224	0.020634	-0.033736
	-0.011332	-0.000534	0.028838	0.034485	-0.032211	0.073655
	0.003444	-0.004132	0.015462	0.006406	0.024767	-0.021070
	-0.022907	-0.009592	-0.008329	0.014235	0.002701	0.001768
	0.025819	-0.005605	0.007272	0.005626	-0.002750	0.065215
	-0.002459					
z225	0.000005	0.006628	0.018058	-0.003208	0.018759	-0.021896
	-0.041125	-0.017969	-0.009188	0.032407	0.002107	-0.025943
	0.006634	-0.063106	0.032032	-0.007395	-0.019434	0.010082
	0.005384	0.008559	-0.004239	0.033263	-0.016600	0.036742
	0.032424	0.002344	0.023851	-0.024046	0.030998	0.005776
	0.025893					

x226	-0.007399	0.022105	0.007561	-0.000129	-0.015493	0.052062
	-0.013333	0.002191	0.003294	0.018706	-0.034244	0.002191
	-0.007767	0.019226	-0.021576	0.052795	0.006406	-0.007147
	-0.023998	-0.006123	-0.000143	0.008375	0.002634	0.021707
	0.052405	0.006985	0.025656	0.010286	0.013472	-0.010942
	-0.010704					
y226	-0.032947	0.010983	-0.021095	-0.001595	0.020210	-0.026487
	-0.006536	-0.005453	0.031494	0.029255	-0.033555	0.065611
	0.002501	0.000271	0.005602	0.015114	0.023396	-0.019069
	-0.028481	-0.001891	-0.007480	0.006462	0.002521	-0.002600
	0.013156	0.013311	0.001450	0.013558	-0.001308	0.070369
	-0.020949					
z226	-0.003374	0.004756	0.015249	-0.006265	0.023360	-0.021082
	-0.038259	-0.012508	-0.008253	0.033241	0.009041	-0.030165
	-0.007615	-0.055845	0.021070	-0.009473	-0.009156	0.017372
	0.023127	-0.001129	-0.038107	0.025089	-0.018890	0.030078
	0.018935	0.025754	0.027888	-0.014502	0.016364	0.018428
	-0.017155					
x227	-0.001258	0.021147	0.013858	-0.003275	-0.018445	0.046935
	-0.016052	0.007435	0.010973	0.019673	-0.029839	-0.002978
	-0.006668	0.014862	-0.006074	0.045984	0.003274	-0.010272
	-0.020494	-0.013875	-0.001248	0.010576	-0.001731	0.019669
	0.055670	0.012511	0.036826	0.006175	0.014725	-0.011033
	-0.003300					
y227	-0.033301	0.006882	-0.021616	-0.003358	0.021946	-0.020703
	-0.001378	-0.012353	0.035550	0.024005	-0.034574	0.055362
	0.001666	0.001439	0.000711	0.024830	0.018971	-0.017309
	-0.026953	0.001740	-0.000981	0.004141	0.002751	-0.007998
	0.005590	0.018845	-0.001825	0.024809	0.001167	0.060597
	-0.021522					
z227	-0.007272	0.003948	0.014565	-0.007970	0.033165	-0.015079
	-0.036745	-0.016294	-0.000549	0.034522	0.006761	-0.031841
	-0.015309	-0.051768	0.012720	-0.006728	-0.000898	0.018156
	0.032340	-0.007485	-0.030295	0.025475	-0.023933	0.016867
	0.023092	0.042391	0.036830	0.011719	0.015532	0.015623
	-0.014491					
x228	0.004214	0.021136	0.019101	-0.005589	-0.022758	0.041660
	-0.017116	0.013538	0.016357	0.022846	-0.026142	-0.001719
	-0.002501	0.010992	0.009612	0.040347	0.000901	-0.014173
	-0.017958	-0.015856	0.001439	0.011280	-0.010104	0.018946
	0.054889	0.011324	0.045006	0.002349	0.017662	-0.010200
	0.009131					

y228	-0.031301	0.002838	-0.020341	-0.006897	0.024014	-0.017409
	0.002911	-0.016819	0.037076	0.019764	-0.035695	0.043008
	0.002893	0.003663	0.003557	0.033577	0.015501	-0.013808
	-0.021043	0.006327	0.003568	0.002212	0.004466	-0.015326
	-0.006684	0.019347	-0.008262	0.029433	-0.001513	0.044412
	-0.022353					
z228	-0.009544	0.003314	0.016380	-0.010863	0.040351	-0.012759
	-0.031666	-0.015660	0.001870	0.042680	0.000688	-0.024776
	-0.010380	-0.044943	0.020216	-0.004789	0.017664	0.014687
	0.041383	0.000353	-0.006864	0.021778	-0.029772	0.006132
	0.004176	0.035627	0.037604	0.014400	0.013369	0.001798
	0.016682					
x229	0.009365	0.021607	0.022288	-0.005483	-0.028413	0.035931
	-0.018894	0.020935	0.020659	0.025634	-0.023385	-0.000487
	0.000862	0.007614	0.022062	0.033698	-0.003990	-0.017344
	-0.019904	-0.018245	-0.002970	0.010431	-0.015154	0.010727
	0.056103	0.008435	0.053748	0.001613	0.020585	-0.000437
	0.013155					
y229	-0.028145	-0.000505	-0.018381	-0.010673	0.024816	-0.016894
	0.004695	-0.018579	0.038267	0.014188	-0.034356	0.031036
	0.005349	0.006239	0.011907	0.041476	0.009145	-0.009443
	-0.018091	0.009420	0.007711	0.001679	0.010505	-0.020170
	-0.014542	0.017807	-0.017481	0.023375	0.001714	0.025200
	-0.024484					
z229	-0.009895	0.000975	0.013944	-0.009200	0.044352	-0.011758
	-0.032636	-0.011317	0.003439	0.046901	-0.001932	-0.023856
	-0.009740	-0.040920	0.032770	0.004734	0.022567	0.014892
	0.036236	0.000636	-0.014766	0.007889	-0.020833	-0.008874
	-0.000895	0.022462	0.040679	0.004864	0.024912	0.006379
	0.013169					
x230	0.013558	0.022528	0.022766	-0.004853	-0.030636	0.033657
	-0.019984	0.027324	0.020979	0.028054	-0.016870	0.005485
	-0.000136	0.003346	0.027827	0.028735	-0.014609	-0.015672
	-0.017569	-0.021117	-0.013675	0.012219	-0.025959	0.011270
	0.054364	0.013708	0.057663	0.010165	0.031021	0.004291
	0.000778					
y230	-0.023298	-0.003164	-0.017203	-0.012860	0.026266	-0.018390
	0.005786	-0.020241	0.038503	0.010987	-0.031648	0.020592
	0.007899	0.005824	0.014191	0.050970	0.000073	-0.006850
	-0.012667	0.007962	-0.001976	0.000803	0.009521	-0.017149
	-0.019436	0.013882	-0.020458	0.021299	0.004954	0.005874
	-0.034498					

z230	-0.008367	-0.003487	0.008915	-0.007063	0.057403	-0.005830
	-0.030795	-0.010626	-0.001531	0.051006	-0.002535	-0.021881
	-0.016421	-0.039047	0.029243	0.019947	0.015725	0.017139
	0.044576	-0.002141	-0.033072	-0.002663	-0.019143	-0.008838
	-0.011019	0.028620	0.044937	0.005618	0.055671	-0.013677
	-0.033186					
x231	0.015951	0.024536	0.022671	-0.005224	-0.032612	0.029860
	-0.017475	0.034124	0.023159	0.028789	-0.010736	0.013748
	-0.000443	0.001461	0.033342	0.018774	-0.021663	-0.016092
	-0.017479	-0.020767	-0.005495	0.017650	-0.034095	0.012920
	0.052304	0.018270	0.052868	0.016307	0.030075	0.007684
	0.007757					
y231	-0.017836	-0.004151	-0.015867	-0.016872	0.022143	-0.021371
	0.004891	-0.018554	0.040376	0.007113	-0.029545	0.010469
	0.010786	0.003425	0.014366	0.053150	-0.003518	-0.003622
	-0.008853	0.006307	0.005231	0.005630	0.010231	-0.016734
	-0.020237	0.004919	-0.021682	0.013436	0.005331	-0.014453
	-0.034075					
z231	-0.006493	-0.006660	0.004166	-0.010641	0.063763	-0.003105
	-0.029171	-0.008982	0.001547	0.046391	-0.002926	-0.024955
	-0.022841	-0.035953	0.024646	0.022218	0.015807	0.015469
	0.046777	-0.005580	-0.006029	-0.004697	-0.012321	-0.000255
	-0.011673	0.024568	0.040119	-0.018372	0.061340	-0.036223
	-0.036555					
x232	0.017188	0.025578	0.020944	-0.003282	-0.035008	0.024863
	-0.014110	0.039450	0.021473	0.028846	-0.002652	0.022381
	-0.004741	0.001529	0.038266	0.008127	-0.025659	-0.017252
	-0.012877	-0.025064	0.000146	0.024843	-0.035820	0.013954
	0.048793	0.012622	0.045006	0.032001	0.015987	0.024071
	0.012263					
y232	-0.011528	-0.004174	-0.014264	-0.019072	0.013890	-0.022033
	0.003358	-0.013836	0.042379	0.007720	-0.024253	0.004725
	0.016463	0.001205	0.009309	0.051213	-0.004255	-0.004349
	-0.005884	0.003080	0.004852	0.012705	0.004293	-0.016359
	-0.015697	-0.003457	-0.022395	0.016680	0.005313	-0.019107
	-0.030488					
z232	-0.003087	-0.013531	0.001799	-0.010465	0.061406	0.000349
	-0.028210	-0.006815	-0.000096	0.044825	0.003625	-0.022398
	-0.028719	-0.030735	0.013088	0.019072	0.019865	0.005926
	0.048103	-0.015821	-0.003703	-0.006032	-0.005587	0.004428
	-0.004042	0.015403	0.031498	-0.009886	0.051304	-0.020260
	-0.037947					

x233	0.014166	0.028944	0.016502	-0.003583	-0.033153	0.024789
	-0.007853	0.037211	0.019313	0.031976	0.005504	0.029761
	-0.003703	0.004722	0.042049	0.002437	-0.026154	-0.022107
	-0.007159	-0.025363	0.006972	0.029189	-0.035841	0.017288
	0.040209	0.007678	0.028577	0.036862	0.007998	0.028446
	0.019763					
y233	-0.005130	-0.006062	-0.012411	-0.017881	0.006516	-0.020820
	0.000189	-0.008582	0.046451	0.004767	-0.020183	0.000326
	0.016227	0.001105	0.001256	0.047219	-0.008529	0.002260
	-0.005894	-0.001948	0.000110	0.017951	-0.012021	-0.017948
	-0.019424	-0.011869	-0.015198	0.025717	0.007175	-0.022124
	-0.025384					
z233	0.000874	-0.025063	0.001132	-0.008653	0.065146	0.010023
	-0.026997	-0.010347	0.005771	0.043609	0.004710	-0.018630
	-0.030372	-0.020867	-0.005459	0.016711	0.009464	0.001277
	0.031872	-0.022588	-0.004152	-0.016263	-0.023234	-0.005334
	-0.021149	0.011638	0.031651	0.006170	0.052736	-0.015925
	-0.030274					
x234	0.012161	0.028484	0.011891	-0.001201	-0.029946	0.023893
	-0.002531	0.040708	0.010741	0.037017	0.004295	0.028684
	-0.000941	0.006083	0.030946	-0.001713	-0.022718	-0.026565
	-0.003301	-0.024521	-0.001295	0.023062	-0.036723	0.011039
	0.031388	-0.004631	0.022484	0.022532	0.008067	0.022524
	0.021813					
y234	0.000858	-0.005421	-0.010994	-0.016380	-0.002325	-0.017304
	-0.001898	-0.001828	0.050016	0.007128	-0.016349	-0.004452
	0.013458	-0.000804	-0.002417	0.041557	-0.016572	0.008538
	0.001005	-0.001475	0.000409	0.025880	-0.014390	-0.022980
	-0.009364	-0.021940	-0.008593	0.023849	-0.003487	-0.019075
	-0.013654					
z234	0.007052	-0.036820	0.000045	-0.009015	0.065062	0.016151
	-0.025315	-0.010228	0.009450	0.044504	0.002159	-0.022183
	-0.028790	-0.009953	-0.015445	0.009639	-0.005471	-0.007063
	0.023760	-0.014700	0.001779	-0.016262	-0.009189	-0.022329
	-0.010581	0.004275	0.037521	0.001893	0.037303	0.000920
	-0.012611					
x235	0.011968	0.026112	0.007153	0.000381	-0.028059	0.023667
	0.000207	0.042320	0.004768	0.032666	0.002952	0.028350
	-0.003042	0.008558	0.030920	-0.008140	-0.022727	-0.030065
	-0.000620	-0.026165	-0.006204	0.021268	-0.035616	0.012798
	0.014915	-0.009574	0.010508	0.016200	0.012820	0.021421
	0.020759					

y235	0.004597	-0.003317	-0.010096	-0.013682	-0.010223	-0.015646
	-0.001532	0.002869	0.054157	0.008578	-0.014751	-0.004652
	0.009927	-0.001931	-0.004026	0.036225	-0.019333	0.010971
	0.006576	-0.002502	-0.004177	0.030102	-0.020928	-0.021758
	-0.009797	-0.022334	-0.008536	0.021311	-0.010601	-0.020319
	-0.003398					
z235	0.011340	-0.046493	-0.001076	-0.010839	0.059322	0.015816
	-0.020887	-0.009701	0.017111	0.037371	-0.000367	-0.018693
	-0.025535	0.005839	-0.017147	0.008348	-0.010803	-0.020414
	0.015619	-0.005975	0.003025	-0.006800	0.001005	-0.022319
	-0.015163	0.017285	0.029685	-0.004875	0.025738	0.008243
	-0.001752					
x236	0.010843	0.024015	0.002433	0.000926	-0.027631	0.024006
	0.002711	0.039189	-0.002557	0.030300	0.002998	0.031262
	-0.005672	0.008311	0.033921	-0.014410	-0.026181	-0.034103
	0.002620	-0.028616	-0.008020	0.016554	-0.031731	0.015889
	-0.000855	-0.013205	0.001862	0.011869	0.014376	0.017777
	0.019918					
y236	0.007438	-0.000866	-0.010112	-0.011137	-0.017572	-0.010751
	-0.002183	0.004514	0.054467	0.013256	-0.009927	-0.001265
	0.006755	-0.006295	-0.002981	0.028936	-0.022925	0.012553
	0.014813	-0.005970	-0.008403	0.030486	-0.022713	-0.014902
	-0.005641	-0.021669	-0.010620	0.018813	-0.013644	-0.022158
	0.005413					
z236	0.016370	-0.055607	-0.001735	-0.012725	0.050366	0.020625
	-0.020482	-0.010367	0.022516	0.031372	0.003405	-0.013952
	-0.018020	0.010590	-0.016901	0.007799	-0.018914	-0.026386
	0.019515	-0.001394	0.001989	0.006570	0.008092	-0.011800
	-0.008087	0.024689	0.026278	-0.005401	0.019456	0.010115
	0.000339					
x237	0.009433	0.023205	-0.001798	0.001345	-0.027546	0.024089
	0.004288	0.033719	-0.010393	0.029472	0.002495	0.033940
	-0.005281	0.005910	0.040576	-0.018352	-0.031289	-0.036651
	0.002536	-0.030269	-0.003032	0.011936	-0.024054	0.015306
	-0.009425	-0.015193	-0.005349	0.006305	0.018007	0.019564
	0.019953					
y237	0.008707	0.002601	-0.011519	-0.009943	-0.023680	-0.006798
	-0.000285	0.005775	0.052321	0.020537	-0.004499	0.003111
	0.004287	-0.009699	-0.002583	0.020775	-0.024225	0.011059
	0.018917	-0.011168	-0.009818	0.027657	-0.022139	-0.009959
	-0.003024	-0.021281	-0.014823	0.017330	-0.002673	-0.016591
	0.012597					

z237	0.019672	-0.062225	-0.001556	-0.017939	0.041109	0.021324
	-0.016258	-0.005417	0.024946	0.026128	0.008413	-0.014574
	-0.008733	0.016650	-0.022298	0.006659	-0.020584	-0.027709
	0.025539	0.000646	0.001237	0.018461	0.004938	-0.004232
	-0.006975	0.028014	0.025699	0.009603	0.039021	0.017142
	-0.005237					
x238	0.007922	0.023115	-0.004691	0.002376	-0.027268	0.024185
	0.005237	0.027570	-0.017271	0.027533	0.000450	0.033724
	-0.003676	0.002618	0.046631	-0.023091	-0.037979	-0.037715
	0.000276	-0.029463	0.004120	0.004812	-0.012733	0.013367
	-0.018525	-0.014195	-0.011417	0.001988	0.016759	0.024478
	0.017467					
y238	0.008072	0.006374	-0.014270	-0.007619	-0.027742	-0.003941
	0.000844	0.005162	0.048595	0.029525	0.000083	0.008465
	0.001123	-0.014139	0.000509	0.010726	-0.026401	0.008432
	0.014599	-0.015479	-0.011011	0.018245	-0.013554	-0.010793
	-0.007132	-0.020710	-0.020807	0.009555	0.003284	-0.012384
	0.017755					
z238	0.022124	-0.069083	-0.002574	-0.018156	0.032219	0.020055
	-0.019572	0.002721	0.025539	0.020710	0.019029	-0.013801
	-0.007920	0.021973	-0.020756	-0.002137	-0.023167	-0.025547
	0.018123	0.002043	0.002115	0.017699	0.006151	-0.012802
	-0.013124	0.038684	0.023956	0.012592	0.030127	0.005539
	-0.014721					
x239	0.007509	0.023170	-0.005972	0.003690	-0.025231	0.024071
	0.006470	0.022580	-0.021338	0.021791	-0.004441	0.032937
	-0.000815	0.000225	0.049403	-0.024804	-0.046432	-0.034169
	0.001334	-0.024924	0.015876	-0.000389	-0.002507	0.007382
	-0.021101	-0.008211	-0.010565	-0.003335	0.009251	0.021539
	0.014752					
y239	0.007771	0.011601	-0.017818	-0.007171	-0.030307	-0.000293
	0.005402	0.003178	0.044690	0.040215	0.001136	0.015128
	0.003089	-0.020620	0.000938	0.006215	-0.028635	0.003696
	0.013374	-0.016211	-0.012081	0.011793	-0.003960	-0.011064
	-0.003039	-0.021962	-0.024381	-0.000295	0.015791	-0.005906
	0.019814					
z239	0.024447	-0.073095	-0.004843	-0.021608	0.023549	0.021674
	-0.020524	0.014630	0.028710	0.015400	0.024611	-0.011415
	0.000052	0.024273	-0.022696	-0.007499	-0.023976	-0.022607
	0.020493	0.004304	-0.007008	0.025756	0.000572	-0.016668
	-0.010450	0.043124	0.029820	0.017705	0.031701	-0.006328
	-0.027567					

x240	0.007792	0.022301	-0.004762	0.006176	-0.021025	0.024582
	0.005164	0.020879	-0.023864	0.009925	-0.010879	0.027626
	0.001194	0.001791	0.049223	-0.025549	-0.056231	-0.027183
	0.004929	-0.014169	0.029399	-0.003661	0.007375	-0.000343
	-0.020153	0.005614	-0.006063	-0.004425	-0.002734	0.019030
	0.013983					
y240	0.007110	0.017337	-0.020255	-0.005855	-0.029446	0.002251
	0.010872	-0.001092	0.037528	0.049452	-0.000931	0.018863
	0.005435	-0.027761	0.002851	0.005046	-0.032764	-0.000331
	0.010122	-0.010248	-0.014701	0.008115	0.006262	-0.003711
	-0.000420	-0.016180	-0.034036	-0.001519	0.024799	0.005076
	0.016765					
z240	0.026639	-0.072722	-0.008140	-0.023589	0.016404	0.022434
	-0.025599	0.022895	0.021296	0.009451	0.026290	-0.004845
	0.005808	0.024979	-0.020179	-0.012775	-0.027715	-0.018842
	0.010425	0.009400	-0.024794	0.037791	-0.005168	-0.011189
	-0.009945	0.057062	0.021089	0.034239	0.024288	-0.002952
	-0.041214					
x241	0.008586	0.019353	-0.001785	0.010857	-0.016403	0.026356
	0.000568	0.023155	-0.023556	-0.006710	-0.018237	0.016433
	0.001598	0.007846	0.044659	-0.026878	-0.065046	-0.017417
	0.010951	0.000200	0.042946	-0.008854	0.018063	-0.018355
	-0.018062	0.020806	0.007249	-0.005467	-0.013698	0.015176
	0.012033					
y241	0.007092	0.021268	-0.019445	-0.003107	-0.028087	0.004515
	0.018784	-0.003812	0.032034	0.053230	-0.003813	0.016820
	0.006505	-0.030705	0.002094	0.003948	-0.034914	-0.001325
	0.013658	0.001086	-0.010560	0.002213	0.016149	-0.004105
	-0.006445	-0.009750	-0.029856	0.000609	0.033125	0.007009
	0.019284					
z241	0.026837	-0.069525	-0.010739	-0.024981	0.004681	0.025252
	-0.031480	0.027090	0.009674	0.006685	0.026191	-0.000422
	0.007235	0.024459	-0.010876	-0.022919	-0.026764	-0.016064
	-0.003700	0.009620	-0.037725	0.033814	-0.006815	-0.022449
	-0.025322	0.056474	0.022860	0.050653	0.014619	-0.004614
	-0.039369					
x242	0.008647	0.014621	0.001020	0.015557	-0.011231	0.029357
	-0.007857	0.027826	-0.021457	-0.022778	-0.025401	0.001619
	-0.001110	0.017647	0.036353	-0.026325	-0.070836	-0.008635
	0.011155	0.012557	0.052036	-0.015065	0.030637	-0.045089
	-0.010379	0.034174	0.021703	-0.005160	-0.020470	0.009573
	0.005150					

y242	0.007767	0.023307	-0.014708	0.000745	-0.024846	0.006704
	0.028900	-0.004988	0.029294	0.050007	-0.005312	0.011404
	0.005904	-0.029883	-0.006076	0.005273	-0.039762	-0.000098
	0.025647	0.016864	0.003128	0.001835	0.024371	-0.008153
	-0.011111	0.000364	-0.013472	-0.008656	0.046913	0.013182
	0.013903					
z242	0.025990	-0.063359	-0.012289	-0.026728	-0.007351	0.030614
	-0.037024	0.018861	-0.000342	0.005976	0.025699	0.005867
	0.009538	0.019157	0.000225	-0.035324	-0.035451	-0.009736
	-0.018404	0.004438	-0.036737	0.028495	-0.006124	-0.027866
	-0.035146	0.049435	0.027178	0.032117	0.020795	0.008641
	-0.047885					
x243	0.004253	0.010051	-0.004249	0.010837	-0.001940	0.025940
	-0.012400	0.026711	-0.022001	-0.021128	-0.021138	-0.005745
	-0.001880	0.017124	0.026250	-0.021695	-0.056827	-0.003775
	0.008034	0.002470	0.042166	-0.011487	0.019478	-0.028477
	-0.012607	0.027426	0.018179	-0.011960	-0.016798	0.005540
	0.011995					
y243	0.006687	0.022680	-0.009737	0.006891	-0.024403	0.013071
	0.029819	-0.006111	0.025777	0.043436	-0.009656	0.003579
	-0.003836	-0.031427	-0.000131	0.005111	-0.037682	-0.000408
	0.028353	0.020244	0.005856	0.002770	0.024634	-0.028040
	-0.007732	0.001521	-0.015078	-0.003641	0.041792	0.001026
	0.011578					
z243	0.020007	-0.057425	-0.014302	-0.029960	-0.012666	0.033878
	-0.037498	0.013541	-0.003295	0.005460	0.020154	0.015750
	0.011738	0.022912	-0.000787	-0.034040	-0.044417	-0.017034
	-0.017852	0.009543	-0.036029	0.025097	-0.006285	-0.018998
	-0.022438	0.055643	0.027676	0.033642	0.031532	-0.004340
	-0.072325					
x244	0.000245	0.005089	-0.009810	0.005572	0.004364	0.022804
	-0.016616	0.024198	-0.024023	-0.017252	-0.017636	-0.009116
	-0.000798	0.017233	0.015641	-0.017476	-0.042940	-0.002425
	0.001486	-0.007247	0.031042	-0.008358	0.011145	-0.017676
	-0.009010	0.018894	0.015934	-0.013961	-0.011381	0.001782
	0.005678					
y244	0.005041	0.021672	-0.005749	0.011022	-0.021191	0.017670
	0.029751	-0.004393	0.022271	0.035600	-0.011105	-0.006033
	-0.012879	-0.031686	0.005014	0.005671	-0.035032	0.001020
	0.029021	0.024593	0.008788	0.000012	0.027597	-0.040168
	-0.004169	0.000035	-0.019773	-0.003733	0.031303	-0.002609
	0.017481					

z244	0.014093	-0.051451	-0.015035	-0.034862	-0.016791	0.036008
	-0.040047	0.010090	-0.006304	0.001890	0.016385	0.022797
	0.012603	0.021606	0.002982	-0.030936	-0.053329	-0.019765
	-0.013894	0.012321	-0.027973	0.015833	0.004353	-0.006395
	-0.013782	0.060661	0.021380	0.027196	0.028252	-0.005862
	-0.064106					
x245	-0.003582	0.000418	-0.014549	-0.000346	0.007543	0.021382
	-0.020719	0.020940	-0.026989	-0.011875	-0.016090	-0.008941
	0.002037	0.014712	0.007164	-0.014755	-0.031862	-0.001938
	-0.004178	-0.016531	0.024741	-0.007278	0.006825	-0.009946
	-0.005066	0.015966	0.018798	-0.009173	-0.009789	0.002036
	-0.005137					
y245	0.003466	0.019911	-0.005166	0.015027	-0.016756	0.020985
	0.028404	-0.002651	0.018325	0.027172	-0.008712	-0.015234
	-0.017943	-0.029104	0.008309	0.008222	-0.029479	0.002962
	0.024136	0.027715	0.003521	-0.004545	0.025537	-0.045254
	-0.003915	-0.005658	-0.024170	-0.002769	0.028066	-0.003270
	0.021146					
z245	0.009556	-0.045663	-0.018738	-0.038404	-0.020520	0.035143
	-0.042036	0.004780	-0.010226	-0.007275	0.017086	0.027790
	0.014862	0.020918	0.005839	-0.030532	-0.054964	-0.022903
	-0.015013	0.013590	-0.026930	0.011497	0.005804	0.011225
	-0.007853	0.059954	0.008549	0.006239	0.031327	-0.008014
	-0.062289					
x246	-0.006607	-0.003290	-0.019321	-0.006189	0.007358	0.020116
	-0.025242	0.016398	-0.030975	-0.008070	-0.013875	-0.006022
	0.006406	0.010201	0.000033	-0.014115	-0.023864	-0.001683
	-0.009901	-0.020750	0.023063	-0.011594	0.004077	0.000138
	-0.002869	0.019082	0.021533	-0.003116	-0.005712	0.003262
	-0.014234					
y246	0.001210	0.017070	-0.005724	0.017792	-0.011943	0.024602
	0.028187	-0.001273	0.013821	0.020827	-0.007241	-0.022874
	-0.020529	-0.026234	0.012090	0.009914	-0.021090	0.003065
	0.018173	0.024631	-0.002613	-0.002506	0.023487	-0.049057
	-0.006016	-0.015562	-0.025306	-0.000272	0.021976	-0.003278
	0.019159					
z246	0.004306	-0.039818	-0.020967	-0.041600	-0.024737	0.034771
	-0.041410	0.001565	-0.010518	-0.013924	0.013788	0.030566
	0.013737	0.018539	0.013800	-0.024815	-0.052835	-0.023989
	-0.013199	0.007685	-0.023309	0.015421	0.013945	0.017281
	-0.006850	0.046610	0.002840	-0.003015	0.033718	-0.014317
	-0.056658					

x247	-0.009087	-0.005910	-0.023681	-0.011731	0.005238	0.019363
	-0.029642	0.011507	-0.034707	-0.005209	-0.013146	-0.002367
	0.010130	0.003925	-0.004606	-0.014543	-0.016708	-0.001885
	-0.015024	-0.024322	0.023866	-0.013667	0.005548	0.008264
	-0.002713	0.017912	0.023425	-0.000296	0.001073	0.000783
	-0.020623					
y247	-0.001484	0.013332	-0.008470	0.019198	-0.008150	0.027863
	0.027868	-0.000955	0.008567	0.015375	-0.004255	-0.027873
	-0.020969	-0.023290	0.013677	0.009561	-0.011795	0.002897
	0.010859	0.020193	-0.008423	-0.002268	0.019196	-0.045786
	-0.008907	-0.018150	-0.024212	0.002854	0.014564	0.000151
	0.014092					
z247	-0.000782	-0.033726	-0.022846	-0.042837	-0.028429	0.033103
	-0.041954	-0.000215	-0.009262	-0.019067	0.013080	0.033570
	0.010406	0.011968	0.022278	-0.018143	-0.051047	-0.022417
	-0.007207	0.002106	-0.018442	0.016797	0.021064	0.020148
	-0.004491	0.044536	0.000045	-0.008932	0.028150	-0.016177
	-0.042288					
x248	-0.011033	-0.007114	-0.026692	-0.015759	0.002548	0.019007
	-0.035174	0.008076	-0.037727	-0.001484	-0.012413	0.001677
	0.012843	-0.005875	-0.006027	-0.014735	-0.011552	-0.001833
	-0.017518	-0.027749	0.023105	-0.012956	0.008629	0.010954
	-0.007994	0.018010	0.024844	0.001540	0.005922	-0.003919
	-0.016800					
y248	-0.005057	0.009132	-0.013506	0.018359	-0.007056	0.031025
	0.027315	-0.002573	0.003233	0.010389	-0.002023	-0.030127
	-0.021233	-0.019682	0.012886	0.008279	-0.001566	0.000970
	-0.000046	0.015074	-0.010190	-0.004111	0.016859	-0.036299
	-0.012146	-0.020105	-0.022480	0.006598	0.012182	0.005240
	0.008881					
z248	-0.005997	-0.027806	-0.024735	-0.044465	-0.032265	0.031242
	-0.039207	-0.004234	-0.008482	-0.028688	0.012133	0.031476
	0.006436	0.009250	0.024366	-0.013996	-0.047872	-0.023991
	-0.007404	-0.002440	-0.009924	0.016278	0.027119	0.024386
	0.004992	0.035031	0.001575	-0.018091	0.020858	-0.020131
	-0.037768					
x249	-0.012735	-0.006632	-0.027602	-0.018846	0.000352	0.018439
	-0.039888	0.005025	-0.039495	-0.000866	-0.012527	0.003849
	0.013706	-0.017128	-0.006071	-0.017119	-0.007607	-0.003436
	-0.020843	-0.031594	0.021621	-0.005408	0.012742	0.008293
	-0.009437	0.018007	0.027861	-0.003270	0.000613	-0.012826
	-0.009887					

y249	-0.009441	0.004876	-0.021052	0.016319	-0.009056	0.033493
	0.024591	-0.004856	-0.002830	0.006981	0.001565	-0.029793
	-0.021225	-0.015206	0.011727	0.006720	0.008378	-0.003002
	-0.013018	0.010271	-0.011446	-0.008239	0.017550	-0.022008
	-0.020746	-0.019719	-0.024157	0.009970	0.017112	0.009660
	0.001363					
z249	-0.010428	-0.022633	-0.027639	-0.042710	-0.034829	0.030082
	-0.040306	-0.006334	-0.008765	-0.032668	0.015067	0.027905
	0.005042	0.005837	0.026175	-0.003512	-0.045269	-0.023675
	-0.005269	-0.006197	-0.011024	0.006775	0.032265	0.024837
	0.002387	0.026130	0.003099	-0.013548	0.028627	-0.014754
	-0.024104					
x250	-0.013720	-0.005793	-0.027235	-0.021359	0.000966	0.017584
	-0.046626	0.004185	-0.037814	-0.000749	-0.010081	0.002259
	0.014169	-0.027881	-0.006341	-0.017755	-0.006785	-0.003759
	-0.026347	-0.033189	0.012150	-0.000793	0.017515	-0.000148
	-0.008178	0.015409	0.032095	-0.010818	0.001083	-0.020386
	0.005516					
y250	-0.015054	0.001538	-0.029046	0.013821	-0.014285	0.036445
	0.020058	-0.007828	-0.009062	0.004353	0.005712	-0.026348
	-0.023152	-0.010598	0.010039	0.004157	0.016451	-0.008966
	-0.026306	0.001873	-0.013486	-0.008560	0.023427	-0.009923
	-0.033279	-0.016300	-0.025206	0.014966	0.024534	0.009528
	-0.002235					
z250	-0.013722	-0.018149	-0.030090	-0.037627	-0.039151	0.031085
	-0.038132	-0.011197	-0.013256	-0.038106	0.014324	0.024632
	0.005729	0.001659	0.029406	0.003272	-0.038395	-0.027025
	-0.004754	-0.007969	-0.004733	0.002125	0.034740	0.020035
	-0.003723	0.018303	0.005682	-0.007924	0.019122	-0.014472
	-0.015109					
x251	-0.013010	-0.004620	-0.026405	-0.020646	0.002612	0.016046
	-0.051116	0.003596	-0.038148	-0.001528	-0.007204	-0.002244
	0.020383	-0.038541	-0.000919	-0.015896	-0.006825	-0.003286
	-0.027334	-0.029406	0.006954	-0.001147	0.021187	-0.009896
	-0.013596	0.014661	0.045338	-0.005123	-0.009452	-0.025639
	0.022616					
y251	-0.021443	-0.001336	-0.035984	0.011291	-0.021623	0.039227
	0.013852	-0.011119	-0.015056	0.001734	0.011229	-0.021762
	-0.025016	-0.003874	0.008936	0.000346	0.017274	-0.016404
	-0.039032	-0.008964	-0.016377	-0.002885	0.031378	-0.003474
	-0.049901	-0.006968	-0.022845	0.024205	0.032564	0.002098
	-0.000965					

z251	-0.015906	-0.014467	-0.030249	-0.033770	-0.041093	0.033499
	-0.036930	-0.017121	-0.015226	-0.043863	0.013201	0.019196
	0.003725	-0.004929	0.025028	0.008952	-0.030991	-0.030686
	-0.014266	-0.015201	-0.008458	0.003763	0.030346	0.011402
	0.000999	0.005829	0.001046	-0.022424	0.015535	-0.017521
	-0.000840					
x252	-0.008292	-0.003378	-0.024053	-0.020185	0.003800	0.011664
	-0.053129	0.000041	-0.034504	0.003303	-0.003491	-0.001403
	0.019218	-0.042245	-0.000571	-0.014352	-0.009277	-0.001097
	-0.023909	-0.027724	0.014363	-0.011600	0.009957	-0.010804
	-0.014134	0.011916	0.042614	-0.001395	-0.009818	-0.045024
	0.025187					
y252	-0.021457	-0.002483	-0.036897	0.009018	-0.013563	0.034600
	0.012415	-0.005785	-0.013382	0.003077	0.010636	-0.023454
	-0.020155	-0.004535	0.001104	-0.005482	0.013216	-0.015553
	-0.032394	-0.003810	-0.019952	-0.002907	0.022622	-0.004371
	-0.042854	-0.002672	-0.015630	0.013181	0.020726	0.001160
	0.014140					
z252	-0.016582	-0.013012	-0.029340	-0.027244	-0.040885	0.035614
	-0.036617	-0.018999	-0.015871	-0.038942	0.012649	0.020287
	-0.002383	-0.010519	0.021797	0.018234	-0.024973	-0.030816
	-0.016170	-0.019203	-0.004467	-0.005362	0.031803	0.001921
	-0.005190	0.000940	0.000091	-0.011340	0.008974	-0.027527
	0.022333					
x253	-0.004849	-0.001847	-0.021153	-0.020392	0.004736	0.008951
	-0.054335	-0.001974	-0.032653	0.008173	-0.001047	0.000017
	0.014797	-0.043905	0.000167	-0.010038	-0.009624	0.002528
	-0.021996	-0.025918	0.018160	-0.017039	0.006801	-0.012743
	-0.018458	0.008239	0.036142	0.004572	-0.007518	-0.058682
	0.032137					
y253	-0.021851	-0.003654	-0.036332	0.006437	-0.005129	0.030476
	0.010318	-0.001094	-0.012116	0.005398	0.009431	-0.025423
	-0.017040	-0.003778	-0.004702	-0.008613	0.005518	-0.013582
	-0.026077	0.000007	-0.022815	-0.002051	0.015258	-0.009856
	-0.037068	0.003528	-0.005537	0.011105	0.008823	-0.001286
	0.031850					
z253	-0.016525	-0.012742	-0.029327	-0.021452	-0.041075	0.034515
	-0.034029	-0.023240	-0.016164	-0.039605	0.015826	0.016566
	-0.009080	-0.013882	0.018335	0.017393	-0.024374	-0.035323
	-0.015242	-0.020523	0.001733	-0.013081	0.022445	-0.004138
	-0.005140	-0.003542	0.005345	-0.007991	0.005873	-0.037801
	0.034156					

x254	-0.002365	-0.001077	-0.018191	-0.021014	0.004766	0.006073
	-0.053255	-0.005148	-0.030204	0.009455	0.001848	-0.000847
	0.009039	-0.045165	0.001908	-0.006972	-0.009101	0.004452
	-0.021613	-0.024862	0.021297	-0.018987	0.003362	-0.013582
	-0.020701	0.005108	0.027293	0.005449	-0.001865	-0.066508
	0.038044					
y254	-0.022600	-0.005252	-0.035187	0.003695	0.002534	0.026478
	0.008900	0.002314	-0.011514	0.007154	0.008552	-0.028707
	-0.015490	-0.000975	-0.008219	-0.011152	-0.003159	-0.012056
	-0.021594	0.002686	-0.024435	-0.000589	0.007717	-0.017089
	-0.033899	0.010882	0.003040	0.011629	-0.002731	-0.003750
	0.046746					
z254	-0.015488	-0.012800	-0.030560	-0.016698	-0.040832	0.033068
	-0.033622	-0.024970	-0.018342	-0.038067	0.017153	0.015061
	-0.016014	-0.015822	0.017060	0.019775	-0.023761	-0.037968
	-0.013479	-0.014820	0.004857	-0.017666	0.015056	-0.007561
	-0.003950	-0.007188	0.004797	-0.002858	0.002274	-0.042497
	0.033694					
x255	-0.000274	-0.000145	-0.015969	-0.021743	0.002846	0.004172
	-0.052357	-0.007581	-0.028463	0.008445	0.004783	-0.001689
	0.003921	-0.045418	0.003208	-0.005562	-0.008008	0.005203
	-0.020984	-0.021642	0.024307	-0.022931	0.000180	-0.012244
	-0.021061	0.000091	0.013621	0.003339	0.007106	-0.067334
	0.037517					
y255	-0.023689	-0.006993	-0.034683	0.000904	0.009125	0.023230
	0.007781	0.005370	-0.012077	0.008120	0.007508	-0.031738
	-0.015692	0.002990	-0.010515	-0.012518	-0.012526	-0.011062
	-0.018716	0.006305	-0.024180	-0.000353	-0.001731	-0.024024
	-0.030810	0.016754	0.008274	0.013071	-0.011971	-0.001823
	0.054399					
z255	-0.014285	-0.013760	-0.030343	-0.013800	-0.039030	0.029028
	-0.030396	-0.027163	-0.018305	-0.036502	0.018893	0.011674
	-0.022454	-0.013828	0.014829	0.022530	-0.021104	-0.039143
	-0.012835	-0.013407	0.002560	-0.012463	0.012460	-0.009882
	-0.005162	-0.003731	0.002529	0.003546	-0.001156	-0.051555
	0.043609					
x256	0.001581	0.000949	-0.013742	-0.022132	-0.000416	0.003170
	-0.052136	-0.009587	-0.026413	0.005802	0.007057	-0.001859
	-0.000025	-0.044822	0.005029	-0.005192	-0.005733	0.006187
	-0.020572	-0.019938	0.026749	-0.024503	0.002233	-0.006915
	-0.018237	-0.003086	-0.003494	0.000759	0.014977	-0.063681
	0.036286					

y256	-0.025281	-0.008862	-0.034565	-0.001820	0.014668	0.021014
	0.006853	0.008317	-0.013459	0.007700	0.005871	-0.034056
	-0.016469	0.008037	-0.011991	-0.013411	-0.021265	-0.010319
	-0.016907	0.008788	-0.022851	0.001555	-0.010039	-0.027913
	-0.026521	0.022605	0.008648	0.016426	-0.020928	0.003933
	0.059744					
z256	-0.012244	-0.015920	-0.030749	-0.011102	-0.036293	0.024410
	-0.027615	-0.030840	-0.017514	-0.035278	0.021523	0.006654
	-0.028306	-0.012779	0.015660	0.025287	-0.019906	-0.038637
	-0.015313	-0.012787	0.000112	-0.004862	0.010641	-0.017491
	-0.000539	-0.000097	-0.002148	-0.001276	-0.000209	-0.058083
	0.045762					
x257	0.004190	0.002397	-0.009569	-0.022048	-0.005362	0.001066
	-0.051055	-0.013460	-0.022988	0.003492	0.011430	-0.004020
	-0.004944	-0.045977	0.006800	-0.005027	-0.001815	0.007673
	-0.021608	-0.021176	0.027850	-0.026529	0.010682	0.000330
	-0.018087	-0.006969	-0.021891	0.000045	0.027874	-0.061212
	0.031644					
y257	-0.026544	-0.010465	-0.033399	-0.004866	0.018653	0.017768
	0.007073	0.009284	-0.014054	0.006585	0.005012	-0.038682
	-0.018348	0.011866	-0.012003	-0.013418	-0.028302	-0.009559
	-0.018897	0.008759	-0.019302	0.004310	-0.013102	-0.031060
	-0.023480	0.025953	0.003551	0.018611	-0.024856	0.011863
	0.059533					
z257	-0.010617	-0.018570	-0.033420	-0.009289	-0.032634	0.022487
	-0.025183	-0.032147	-0.018601	-0.036336	0.024142	0.004547
	-0.033698	-0.008010	0.011425	0.025317	-0.019670	-0.041580
	-0.013020	-0.012600	-0.008912	0.002027	0.000210	-0.019432
	0.008663	0.004838	-0.007031	-0.004694	-0.000834	-0.054480
	0.041933					
x258	0.007377	0.004585	-0.005629	-0.022285	-0.011570	-0.000827
	-0.050610	-0.015914	-0.017131	-0.000916	0.013546	-0.004482
	-0.006588	-0.045022	0.009309	-0.006244	0.003078	0.009662
	-0.023200	-0.024033	0.027460	-0.027127	0.020405	0.005959
	-0.013331	-0.009664	-0.040077	-0.003295	0.032393	-0.060609
	0.028013					
y258	-0.028771	-0.011861	-0.034524	-0.007244	0.022906	0.017811
	0.004392	0.013176	-0.016607	0.001510	0.003771	-0.036715
	-0.018727	0.017867	-0.015107	-0.014105	-0.033940	-0.008545
	-0.020157	0.004636	-0.019014	0.004650	-0.016500	-0.035466
	-0.014234	0.026484	-0.004324	0.020411	-0.032286	0.020139
	0.055333					

z258	-0.008085	-0.020183	-0.033815	-0.010066	-0.031042	0.016713
	-0.018134	-0.036944	-0.015722	-0.034372	0.024010	-0.006375
	-0.038096	-0.002587	0.012031	0.027592	-0.019670	-0.042131
	-0.015194	-0.012093	-0.013732	0.011521	-0.008019	-0.019513
	0.017220	0.014690	-0.013309	-0.003489	0.002688	-0.040748
	0.042692					
x259	0.011996	0.006949	0.001746	-0.021012	-0.019778	-0.003613
	-0.050256	-0.021344	-0.010093	-0.002441	0.018936	-0.007224
	-0.009222	-0.044396	0.012234	-0.010402	0.009707	0.011839
	-0.022956	-0.027537	0.029334	-0.029294	0.035067	0.011249
	-0.009217	-0.010338	-0.056332	-0.006826	0.039869	-0.062713
	0.022458					
y259	-0.029067	-0.013166	-0.033905	-0.011122	0.024173	0.014684
	0.002695	0.010899	-0.014751	-0.000016	0.004773	-0.043466
	-0.018277	0.020094	-0.015296	-0.011369	-0.039333	-0.005821
	-0.026260	-0.008240	-0.017535	0.005438	-0.017029	-0.036332
	-0.000459	0.029870	-0.017219	0.018865	-0.036547	0.031100
	0.041156					
z259	-0.008142	-0.023436	-0.035624	-0.009313	-0.027283	0.015226
	-0.014490	-0.036375	-0.019023	-0.036491	0.029054	-0.009039
	-0.045813	0.007921	0.010886	0.025437	-0.015587	-0.049573
	-0.013473	-0.010023	-0.017415	0.021290	-0.014159	-0.023801
	0.025520	0.019931	-0.020514	-0.000557	0.007008	-0.030600
	0.042189					
x260	0.017006	0.008154	0.011585	-0.021135	-0.029914	-0.007139
	-0.049755	-0.028717	-0.001106	-0.000367	0.030221	-0.011098
	-0.015952	-0.036829	0.019258	-0.010664	0.020188	0.007138
	-0.018565	-0.031192	0.031090	-0.029099	0.045273	0.010226
	0.002585	-0.001402	-0.066364	-0.002377	0.040650	-0.070523
	0.023550					
y260	-0.027128	-0.014167	-0.030062	-0.014470	0.022671	0.010459
	-0.000801	0.007485	-0.011664	-0.002445	0.007563	-0.051639
	-0.019106	0.020773	-0.019672	-0.007416	-0.039798	-0.002256
	-0.030507	-0.024855	-0.018423	0.005202	-0.011115	-0.031558
	0.013109	0.031943	-0.033612	0.017688	-0.031075	0.049116
	0.017108					
z260	-0.008524	-0.026043	-0.040296	-0.009450	-0.020764	0.013400
	-0.010970	-0.033181	-0.019377	-0.041995	0.033214	-0.011975
	-0.048620	0.022402	0.018814	0.020684	-0.016664	-0.050627
	-0.021313	-0.016918	-0.014648	0.036087	-0.023851	-0.037917
	0.052903	0.028283	-0.029823	-0.003842	-0.004024	-0.012838
	0.039426					

x261	0.019951	0.013674	0.011428	-0.022851	-0.032952	-0.012173
	-0.048581	-0.033778	-0.010623	-0.001940	0.029502	0.003230
	-0.017909	-0.029201	0.006489	-0.006961	0.017368	0.003923
	-0.015243	-0.021648	0.035932	-0.015849	0.043187	-0.008814
	0.002129	0.000693	-0.052210	-0.010531	0.034262	-0.043242
	0.023824					
y261	-0.021809	-0.010419	-0.021125	-0.017408	0.022775	0.003169
	-0.011867	0.014589	-0.012655	-0.017913	0.003033	-0.041937
	-0.023993	0.014628	-0.023473	-0.011786	-0.036005	0.004977
	-0.044401	-0.026821	-0.016106	-0.004929	0.001504	-0.018392
	0.009938	0.017611	-0.038103	0.014603	-0.030430	0.056771
	0.018800					
z261	-0.001931	-0.020591	-0.036203	-0.005239	-0.014115	0.008054
	-0.013798	-0.028192	-0.027165	-0.052215	0.028522	0.000209
	-0.051138	0.024176	0.004521	0.005814	-0.011658	-0.028278
	-0.029914	-0.014305	-0.015224	0.020601	-0.012121	-0.032048
	0.036668	0.015184	-0.034525	-0.012747	0.009932	0.006691
	0.023574					
x262	0.025260	0.020791	0.014912	-0.020228	-0.035193	-0.017509
	-0.044979	-0.040941	-0.018341	-0.003383	0.027232	0.016514
	-0.020782	-0.027225	-0.008614	-0.011442	0.011159	0.007740
	-0.007655	-0.007562	0.038647	-0.005586	0.050685	-0.014988
	-0.010274	0.000104	-0.039412	-0.017263	0.032688	-0.020487
	0.020601					
y262	-0.016205	-0.002528	-0.013176	-0.018524	0.021243	-0.004935
	-0.020273	0.020175	-0.016482	-0.028527	-0.007677	-0.029154
	-0.021596	0.008963	-0.032914	-0.015148	-0.032640	0.016655
	-0.057309	-0.025543	-0.014343	-0.013837	0.020136	-0.013859
	0.006381	-0.001676	-0.044776	0.005792	-0.030256	0.059393
	0.023487					
z262	0.006018	-0.017270	-0.031702	-0.003063	-0.008441	0.000899
	-0.012891	-0.024321	-0.025004	-0.059032	0.027987	0.002344
	-0.058174	0.020754	0.001738	0.002330	-0.006145	-0.013540
	-0.037379	-0.014989	-0.018854	0.011205	-0.010494	-0.020570
	0.022505	0.010179	-0.023917	-0.006112	0.026569	0.030387
	0.005276					
x263	0.029502	0.025403	0.019010	-0.018144	-0.037944	-0.022026
	-0.040467	-0.046150	-0.025777	-0.005775	0.026495	0.027634
	-0.024375	-0.020206	-0.015672	-0.014303	0.008202	0.006126
	0.003677	0.008907	0.043282	0.007446	0.055194	-0.021474
	-0.019551	0.005886	-0.024163	-0.016933	0.019214	-0.006286
	0.021448					

y263	-0.009862	0.004240	-0.003964	-0.022474	0.017649	-0.013888
	-0.028878	0.022825	-0.017954	-0.034637	-0.009167	-0.022771
	-0.025866	0.005950	-0.040424	-0.009919	-0.029410	0.023956
	-0.063905	-0.027996	-0.017074	-0.020599	0.030869	-0.014120
	0.010369	-0.015624	-0.043717	-0.001575	-0.021532	0.055209
	0.026784					
z263	0.013796	-0.013153	-0.027084	0.002190	-0.002943	-0.005878
	-0.009297	-0.015219	-0.025149	-0.068089	0.020747	0.010890
	-0.057588	0.017157	0.003587	-0.007816	0.004675	0.005242
	-0.041470	-0.009514	-0.009444	0.003871	-0.004055	-0.007152
	0.002589	0.004298	-0.012903	0.003498	0.028442	0.041176
	-0.001695					
x264	0.034135	0.029049	0.023832	-0.016420	-0.039223	-0.025746
	-0.036738	-0.051240	-0.028857	-0.006994	0.027315	0.034051
	-0.031961	-0.012235	-0.022438	-0.012447	0.006591	0.002587
	0.013545	0.023562	0.031897	0.015177	0.056561	-0.030094
	-0.016102	0.013571	-0.018541	-0.021610	0.012835	0.009853
	0.024016					
y264	-0.003194	0.009789	0.007224	-0.027292	0.013193	-0.023960
	-0.035973	0.025789	-0.019856	-0.039735	-0.005745	-0.018398
	-0.030782	0.005635	-0.041198	-0.002250	-0.026333	0.027974
	-0.060941	-0.028486	-0.016621	-0.020504	0.037986	-0.013179
	0.007830	-0.024087	-0.028157	-0.001764	-0.019386	0.040375
	0.026921					
z264	0.022351	-0.008527	-0.023975	0.008293	0.003265	-0.011374
	-0.006442	-0.005342	-0.024546	-0.074588	0.011767	0.021420
	-0.058148	0.011936	0.000307	-0.008266	0.016844	0.026084
	-0.043220	-0.003894	-0.019064	-0.005558	-0.005784	0.005853
	0.004358	-0.002439	-0.013844	0.004136	0.047335	0.054959
	-0.003365					
x265	0.037034	0.030126	0.026053	-0.012899	-0.040469	-0.026635
	-0.030593	-0.054769	-0.032097	-0.006323	0.033234	0.036003
	-0.041077	-0.006942	-0.021034	-0.010816	0.005214	-0.008186
	0.018048	0.030108	0.020246	0.017215	0.056811	-0.038257
	-0.018466	0.017195	-0.006588	-0.024409	-0.005855	0.018613
	0.011306					
y265	0.004665	0.018280	0.021694	-0.033411	0.008854	-0.036109
	-0.042077	0.026676	-0.022306	-0.046205	-0.008470	-0.012405
	-0.033885	0.007411	-0.042123	0.006166	-0.023134	0.033483
	-0.053807	-0.018600	-0.013779	-0.013902	0.047986	-0.013839
	0.003489	-0.030361	-0.016135	-0.004068	-0.012108	0.028872
	0.034694					

z265	0.027728	-0.006871	-0.023574	0.013763	0.003794	-0.015864
	0.003748	0.005862	-0.027301	-0.076997	0.000782	0.029983
	-0.056362	0.007248	0.008343	-0.014162	0.034227	0.035211
	-0.047005	0.002604	-0.004653	-0.010557	0.000664	0.005237
	-0.001119	-0.017463	0.002750	0.000784	0.043216	0.049746
	-0.015490					
x266	0.038533	0.028911	0.021979	-0.005024	-0.038057	-0.023907
	-0.020394	-0.061378	-0.032968	0.002829	0.047156	0.034544
	-0.048275	-0.008398	-0.013482	0.002406	-0.003627	-0.018910
	0.020303	0.020955	-0.010913	0.017572	0.044492	-0.033199
	-0.018780	0.026069	0.004649	-0.011298	-0.031312	0.026951
	-0.005395					
y266	0.013212	0.028345	0.037996	-0.039671	0.002763	-0.048950
	-0.049960	0.025916	-0.031170	-0.055805	-0.012876	-0.005021
	-0.037744	0.011579	-0.040289	0.015931	-0.021223	0.031697
	-0.038541	-0.007486	-0.017424	-0.002766	0.055194	-0.013197
	-0.004748	-0.031622	-0.000675	-0.000532	-0.015549	0.019472
	0.028626					
z266	0.029605	-0.006709	-0.024920	0.018307	0.002662	-0.019422
	0.019299	0.014443	-0.029861	-0.074036	-0.014865	0.035674
	-0.048867	0.000605	0.025212	-0.019072	0.053892	0.036167
	-0.051481	0.012070	0.029096	-0.005576	0.007542	-0.001230
	-0.002791	-0.030479	0.011591	0.008670	0.032769	0.036608
	-0.006677					
x267	0.037547	0.021401	0.008531	0.009320	-0.032936	-0.012749
	0.000151	-0.067455	-0.034988	0.024968	0.075059	0.035022
	-0.065196	-0.011090	-0.010658	0.015330	-0.012912	-0.029881
	0.020121	0.007889	-0.053663	0.004868	0.005626	-0.016110
	-0.019351	0.035947	0.007075	-0.004422	-0.044705	0.026561
	-0.020477					
y267	0.023962	0.040021	0.062258	-0.050939	-0.005799	-0.066847
	-0.059185	0.019085	-0.039436	-0.068484	-0.018937	-0.001824
	-0.042503	0.021674	-0.027171	0.035135	-0.019009	0.021953
	-0.011312	0.004001	-0.027469	0.014839	0.058405	-0.018817
	-0.011966	-0.023314	0.015926	0.018407	-0.025989	0.013460
	0.019180					
z267	0.030138	-0.007708	-0.032734	0.027118	0.002052	-0.017258
	0.040652	0.024556	-0.032578	-0.069670	-0.028650	0.041325
	-0.042864	-0.002079	0.038900	-0.016499	0.068439	0.036491
	-0.049734	0.021412	0.050700	0.003361	-0.009091	-0.006969
	-0.010567	-0.031700	0.021099	0.026320	0.030571	0.015774
	-0.006743					

x268	0.032516	0.004414	-0.018689	0.034311	-0.025546	0.006561
	0.030254	-0.074841	-0.038811	0.058305	0.108198	0.034916
	-0.086864	-0.022328	-0.009392	0.027382	-0.011471	-0.043335
	-0.004538	0.005610	-0.075973	-0.025207	-0.032033	0.010520
	-0.007609	0.029340	-0.002907	-0.016766	-0.055951	0.017636
	-0.016482					
y268	0.032562	0.042880	0.085777	-0.068571	-0.014714	-0.077911
	-0.058252	0.006935	-0.047259	-0.068925	-0.018930	-0.003631
	-0.048767	0.032904	0.005095	0.063629	0.001386	0.010783
	0.009654	0.009120	-0.021524	0.026538	0.046380	-0.027812
	-0.014087	-0.011329	0.020139	0.026932	-0.024516	0.024899
	0.009022					
z268	0.029129	-0.011213	-0.049920	0.044957	0.003168	-0.007696
	0.062323	0.032828	-0.033459	-0.057747	-0.036248	0.038631
	-0.036529	-0.014516	0.046102	0.008199	0.071768	0.027252
	-0.047699	0.029181	0.049732	0.009431	-0.031397	-0.010129
	-0.018622	-0.021834	0.037440	0.043914	0.028485	0.005081
	-0.027463					
x269	0.025463	-0.005898	-0.035746	0.055308	-0.024837	0.013302
	0.050308	-0.082785	-0.038488	0.069881	0.125568	0.018211
	-0.093066	-0.021450	-0.005538	0.018621	-0.012190	-0.071147
	-0.027921	0.018047	-0.080928	-0.028452	-0.028408	0.018437
	-0.010766	0.018918	-0.007457	-0.008201	-0.068175	-0.018855
	-0.024217					
y269	0.032444	0.033908	0.084830	-0.071268	-0.019456	-0.071512
	-0.042139	-0.004829	-0.054743	-0.049986	-0.000511	-0.011169
	-0.058040	0.040558	0.037007	0.083921	0.014615	-0.002241
	0.005688	0.006114	-0.021436	0.026789	0.014375	-0.021146
	-0.018539	-0.001828	0.026704	0.036120	-0.030935	0.003894
	-0.011623					
z269	0.025618	-0.014537	-0.059659	0.057991	0.002876	-0.000735
	0.075980	0.036936	-0.026717	-0.038672	-0.034894	0.021520
	-0.030686	-0.027150	0.045818	0.034878	0.057979	0.003237
	-0.033682	0.036203	0.018347	0.017227	-0.036438	-0.010563
	-0.018107	-0.012433	0.045266	0.055177	0.023139	-0.014910
	-0.051313					
x270	-0.024026	-0.014334	-0.014060	-0.019504	-0.009915	-0.047751
	0.031203	0.035398	-0.022685	0.004715	0.011620	-0.020886
	-0.011944	0.018848	-0.051215	0.007859	-0.030160	-0.045424
	-0.071601	-0.001447	0.002356	0.033792	0.008520	-0.006281
	-0.018623	-0.084681	-0.002033	0.038031	0.020818	0.023031
	-0.077999					

y270	0.013984	-0.011253	-0.004660	-0.027696	-0.002851	0.015569
	0.002617	0.033111	-0.040935	0.000335	0.031341	0.013871
	0.007196	-0.044608	-0.005350	-0.069339	0.054352	0.075247
	-0.013486	-0.035142	0.004564	-0.077723	-0.027748	-0.076956
	0.046312	-0.023362	0.033473	0.038599	-0.068659	-0.032788
	-0.005715					
z270	-0.053637	-0.013854	-0.004725	-0.081488	-0.030016	-0.005826
	-0.003693	0.070953	0.016355	-0.019199	-0.014946	-0.010236
	0.068907	0.010280	0.027396	-0.093210	0.042080	0.026585
	0.040486	0.036741	0.010703	-0.045506	-0.027162	0.079451
	0.012094	-0.036007	-0.113366	-0.016018	-0.042202	0.043555
	-0.082474					
x271	-0.020635	-0.012280	-0.013913	-0.023323	-0.017567	-0.053427
	0.025279	0.033482	-0.023968	-0.003137	0.019744	-0.016270
	-0.014741	0.009702	-0.042438	0.007263	-0.014820	-0.039410
	-0.072674	-0.011913	0.018494	0.015749	-0.002358	-0.016023
	-0.031203	-0.066690	-0.008001	0.035262	0.001400	0.022503
	-0.050320					
y271	0.016402	-0.013895	0.000035	-0.021072	-0.004218	0.015623
	0.006345	0.021505	-0.035399	-0.000449	0.028267	0.003027
	0.007810	-0.040038	-0.017663	-0.067479	0.044666	0.067757
	-0.016086	-0.025616	0.000203	-0.069247	-0.019756	-0.067106
	0.049573	-0.011680	0.038669	0.035987	-0.055538	-0.035882
	-0.019448					
z271	-0.047875	-0.009197	0.002846	-0.079698	-0.026656	-0.004640
	-0.000777	0.065063	0.018880	-0.016826	-0.006327	-0.009440
	0.048266	0.002970	0.027855	-0.076434	0.029786	0.025401
	0.027487	0.025373	0.016347	-0.033491	-0.026431	0.063074
	-0.005606	-0.032297	-0.093812	-0.022615	-0.037541	0.042175
	-0.058787					
x272	-0.019326	-0.010040	-0.021528	-0.027422	-0.023167	-0.052208
	0.018185	0.040079	-0.021195	-0.014223	0.017255	-0.006165
	-0.005616	0.013854	-0.039360	0.002810	-0.007667	-0.020842
	-0.064224	-0.017240	0.022011	-0.004174	-0.016831	-0.025998
	-0.037212	-0.043193	0.000041	0.029565	-0.018308	0.033409
	-0.024160					
y272	0.016811	-0.018314	0.005071	-0.015492	-0.006266	0.014899
	0.007643	0.010558	-0.029476	-0.001592	0.024995	-0.007903
	0.011653	-0.038000	-0.026077	-0.067755	0.039388	0.059248
	-0.016560	-0.018204	-0.001957	-0.061540	-0.012671	-0.056351
	0.050305	-0.003512	0.042936	0.026104	-0.042304	-0.028673
	-0.027354					

z272	-0.042941	-0.005001	0.006990	-0.075989	-0.023564	-0.002205
	0.001099	0.060604	0.020871	-0.016488	-0.004734	-0.004719
	0.033018	0.001130	0.024947	-0.063193	0.014200	0.025905
	0.014811	0.019718	0.019131	-0.025383	-0.029726	0.047600
	-0.010588	-0.025620	-0.074917	-0.024121	-0.042243	0.037010
	-0.040968					
x273	-0.020845	-0.011508	-0.023163	-0.030047	-0.024655	-0.047265
	0.013405	0.043372	-0.012849	-0.020033	0.012528	-0.006323
	-0.000706	0.010422	-0.042519	-0.005021	0.007395	-0.010114
	-0.048588	-0.027264	0.017155	-0.000639	-0.017160	-0.024872
	-0.045843	-0.031883	0.004897	0.018678	-0.018532	0.036467
	-0.001952					
y273	0.015933	-0.023152	0.011203	-0.011194	-0.009090	0.013141
	0.007232	0.000380	-0.023949	-0.001977	0.023783	-0.017393
	0.015320	-0.038563	-0.029355	-0.067683	0.035475	0.051098
	-0.015159	-0.014385	-0.001969	-0.053716	-0.007832	-0.046651
	0.049089	0.001838	0.046366	0.010860	-0.029968	-0.017757
	-0.028692					
z273	-0.038314	-0.002915	0.012480	-0.070648	-0.021763	-0.000684
	0.003107	0.053960	0.024585	-0.014505	-0.003810	-0.003010
	0.016969	-0.002834	0.020379	-0.052616	-0.000490	0.024118
	0.001698	0.018197	0.021889	-0.016816	-0.028423	0.038166
	-0.011600	-0.021271	-0.057973	-0.022090	-0.042102	0.026770
	-0.032792					
x274	-0.023451	-0.014368	-0.026070	-0.029650	-0.027464	-0.043334
	0.008417	0.044011	-0.007718	-0.018385	0.012029	-0.010449
	0.013214	0.006717	-0.043019	-0.006572	0.018832	0.003751
	-0.039662	-0.033309	-0.001792	-0.006582	-0.009440	-0.013262
	-0.055364	-0.011641	-0.004261	0.010184	-0.006810	0.020321
	-0.014305					
y274	0.014559	-0.027627	0.018404	-0.008383	-0.012716	0.010397
	0.005980	-0.007155	-0.019620	-0.004193	0.022709	-0.022950
	0.016622	-0.037357	-0.029039	-0.068263	0.034031	0.042183
	-0.012463	-0.010007	0.004308	-0.044422	-0.006668	-0.043620
	0.049197	0.004002	0.049629	-0.003959	-0.023531	-0.003769
	-0.017828					
z274	-0.034668	-0.002232	0.017655	-0.065238	-0.021042	0.001059
	0.004876	0.045870	0.030169	-0.012634	-0.005249	-0.003302
	0.002360	-0.007598	0.014521	-0.046959	-0.013631	0.021667
	-0.008178	0.017608	0.027318	-0.008455	-0.025577	0.031805
	-0.008311	-0.018281	-0.039412	-0.019390	-0.037891	0.015926
	-0.023964					

x275	-0.025499	-0.016419	-0.026369	-0.028714	-0.030492	-0.038675
	0.006727	0.040872	0.000919	-0.022487	0.010275	-0.010314
	0.016570	0.001685	-0.045986	-0.010225	0.021571	0.007902
	-0.034608	-0.033416	-0.003286	-0.005483	-0.004741	-0.008587
	-0.049483	-0.002451	0.004302	0.009059	0.001611	0.015469
	-0.003841					
y275	0.012714	-0.031088	0.024837	-0.005428	-0.016767	0.008090
	0.003070	-0.013756	-0.017893	-0.005998	0.024268	-0.026814
	0.017295	-0.036514	-0.027078	-0.065235	0.035071	0.034242
	-0.009158	-0.010150	0.006344	-0.035152	-0.010880	-0.042783
	0.047984	0.009097	0.049005	-0.018570	-0.017078	0.004534
	-0.009493					
z275	-0.030997	-0.002658	0.023114	-0.059063	-0.020547	0.001137
	0.004556	0.038347	0.034759	-0.010594	-0.005599	-0.002489
	-0.010827	-0.010617	0.008465	-0.040109	-0.024014	0.018332
	-0.018271	0.017985	0.027933	-0.002287	-0.025046	0.027005
	-0.007455	-0.014129	-0.025753	-0.016775	-0.037779	0.007382
	-0.019037					
x276	-0.026952	-0.018773	-0.025735	-0.026543	-0.032516	-0.035663
	0.003261	0.037867	0.007077	-0.026980	0.010907	-0.011092
	0.017047	-0.004919	-0.049118	-0.012522	0.023094	0.008294
	-0.030659	-0.034647	-0.003950	-0.001316	-0.009022	-0.002582
	-0.037113	0.003830	0.018171	0.007770	0.006558	0.012293
	0.000344					
y276	0.010513	-0.033873	0.030927	-0.002319	-0.021959	0.006650
	0.000261	-0.019350	-0.016216	-0.007395	0.025002	-0.029554
	0.017406	-0.035012	-0.024274	-0.062723	0.037929	0.027619
	-0.004748	-0.012459	0.007991	-0.025751	-0.013951	-0.044038
	0.044880	0.014199	0.046807	-0.031324	-0.011789	0.011396
	-0.000133					
z276	-0.027959	-0.003830	0.028618	-0.052964	-0.021094	0.000705
	0.003588	0.030760	0.039776	-0.009058	-0.006224	-0.001243
	-0.021987	-0.013060	0.002713	-0.034833	-0.032510	0.014754
	-0.027205	0.019936	0.027312	0.001910	-0.024187	0.022478
	-0.007556	-0.010110	-0.013255	-0.013322	-0.037149	0.001364
	-0.012059					
x277	-0.028878	-0.021147	-0.022438	-0.023823	-0.035106	-0.033656
	-0.000169	0.034471	0.012401	-0.028762	0.013931	-0.011716
	0.020058	-0.011620	-0.046562	-0.013324	0.027014	0.010739
	-0.025999	-0.039716	-0.007730	0.001204	-0.010251	-0.006845
	-0.031267	0.017283	0.023890	0.002011	0.004154	0.003760
	0.007842					

y277	0.008032	-0.035819	0.036132	0.000856	-0.026713	0.006418
	-0.002852	-0.023303	-0.015123	-0.008062	0.025025	-0.030940
	0.016861	-0.033302	-0.022470	-0.059718	0.041246	0.021502
	-0.000993	-0.015123	0.008457	-0.014523	-0.018051	-0.043504
	0.040539	0.018821	0.044938	-0.040198	-0.007349	0.017278
	0.006378					
z277	-0.025354	-0.005706	0.033502	-0.046744	-0.022045	-0.000120
	0.001751	0.023963	0.044375	-0.007784	-0.007069	-0.000120
	-0.031867	-0.014722	-0.004161	-0.030215	-0.039309	0.009962
	-0.035250	0.023873	0.025562	0.005341	-0.024765	0.021528
	-0.008156	-0.007918	-0.000179	-0.008214	-0.034517	-0.000763
	-0.007726					
x278	-0.031753	-0.023445	-0.019036	-0.021955	-0.037458	-0.030931
	-0.002786	0.033112	0.019922	-0.032337	0.015142	-0.008678
	0.020819	-0.015867	-0.047022	-0.011318	0.028974	0.010596
	-0.025763	-0.037793	-0.006570	0.004526	-0.008845	-0.004435
	-0.027212	0.030086	0.028203	0.004147	0.003871	-0.008201
	0.021490					
y278	0.005333	-0.036778	0.040896	0.004581	-0.030600	0.008343
	-0.004024	-0.027695	-0.013114	-0.006620	0.025295	-0.030742
	0.015060	-0.030099	-0.020773	-0.055177	0.043218	0.015067
	0.004858	-0.021172	0.006380	-0.002250	-0.019842	-0.043947
	0.034061	0.024337	0.040312	-0.048350	-0.000427	0.017365
	0.003832					
z278	-0.023137	-0.007915	0.038280	-0.039758	-0.023773	-0.000932
	-0.000416	0.016671	0.048352	-0.006123	-0.007529	0.000504
	-0.038729	-0.016301	-0.009207	-0.026806	-0.043532	0.004671
	-0.039359	0.025930	0.020246	0.007610	-0.026560	0.018819
	-0.009368	-0.006067	0.011259	-0.005597	-0.033032	-0.000586
	-0.009686					
x279	-0.036248	-0.024093	-0.016412	-0.020122	-0.039655	-0.029133
	-0.001843	0.033876	0.022721	-0.035127	0.018176	-0.005725
	0.023546	-0.019203	-0.052068	-0.008172	0.028463	0.006614
	-0.020339	-0.036495	-0.014213	0.010308	-0.020867	0.001080
	-0.024903	0.029120	0.037358	0.011691	-0.000219	0.000697
	0.021574					
y279	0.000121	-0.036406	0.041159	0.006190	-0.036710	0.008869
	-0.002666	-0.025909	-0.018567	-0.001556	0.024929	-0.035091
	0.025371	-0.025361	-0.014853	-0.052496	0.040140	0.014629
	0.014856	-0.015522	0.012562	0.002010	-0.036561	-0.041376
	0.019326	0.029875	0.036308	-0.038964	-0.004593	0.028319
	-0.005308					

z279	-0.021918	-0.010546	0.042052	-0.033825	-0.026540	-0.002074
	-0.003241	0.011485	0.051109	-0.002839	-0.008261	-0.001526
	-0.040897	-0.017149	-0.012364	-0.025574	-0.045026	0.003494
	-0.043841	0.033755	0.017370	0.006711	-0.028686	0.014882
	-0.011571	-0.001756	0.019520	-0.001418	-0.029134	0.001155
	-0.006276					
x280	-0.041900	-0.023117	-0.014159	-0.018638	-0.044211	-0.029747
	-0.001407	0.041742	0.019908	-0.037195	0.024026	-0.003458
	0.025428	-0.023596	-0.053268	0.002304	0.029287	0.000418
	-0.020327	-0.028783	-0.007614	0.007259	-0.028069	-0.007540
	-0.020428	0.022617	0.035745	0.015198	-0.008963	-0.006043
	0.024831					
y280	-0.005941	-0.035601	0.041303	0.006765	-0.043551	0.011168
	-0.003015	-0.021747	-0.025591	0.002664	0.022507	-0.042668
	0.036270	-0.022501	-0.014214	-0.051840	0.041182	0.013930
	0.017809	-0.008784	0.026317	0.008304	-0.043741	-0.052688
	0.005410	0.033841	0.035192	-0.023581	-0.005372	0.037867
	0.008495					
z280	-0.022190	-0.014089	0.047278	-0.027802	-0.030820	-0.000631
	-0.006657	0.006445	0.051797	-0.000421	-0.011373	-0.005293
	-0.037621	-0.018268	-0.018882	-0.030011	-0.041057	0.000114
	-0.046539	0.039472	0.018323	0.013473	-0.027553	0.003335
	-0.014582	0.004790	0.028176	0.007847	-0.025612	0.009618
	0.006494					
x281	-0.045783	-0.023898	-0.010499	-0.016335	-0.046093	-0.032082
	-0.003135	0.049304	0.020290	-0.038111	0.029198	0.000046
	0.026768	-0.025988	-0.056824	0.011702	0.031743	-0.005483
	-0.019679	-0.016699	-0.001188	0.005057	-0.028728	-0.010991
	-0.018683	0.020095	0.030512	0.018276	-0.011710	-0.013072
	0.022557					
y281	-0.010036	-0.034062	0.041450	0.008742	-0.047589	0.016395
	-0.003954	-0.020273	-0.025371	0.003255	0.017885	-0.042267
	0.039926	-0.018992	-0.017693	-0.049431	0.043467	0.012486
	0.017485	-0.011507	0.034447	0.016613	-0.042224	-0.061434
	0.003021	0.037176	0.031081	-0.018684	-0.004307	0.035835
	0.024139					
z281	-0.022903	-0.015804	0.051429	-0.021371	-0.035888	0.004269
	-0.009781	-0.000468	0.053896	0.000377	-0.015587	-0.004310
	-0.033343	-0.017546	-0.026680	-0.032024	-0.035523	-0.002899
	-0.046646	0.035934	0.018116	0.021321	-0.025593	-0.007795
	-0.010982	0.010911	0.033146	0.010421	-0.023706	0.011106
	0.019923					

x282 -0.049788 -0.023890 -0.007918 -0.013136 -0.047628 -0.031953
-0.002135 0.053826 0.023282 -0.039742 0.034539 0.003925
0.028397 -0.032971 -0.062141 0.016996 0.031524 -0.011037
-0.013233 -0.020168 0.002810 0.008450 -0.029104 -0.006652
-0.010942 0.013725 0.035821 0.019190 -0.012307 -0.012583
0.023014

y282 -0.012899 -0.031868 0.040332 0.010022 -0.050787 0.018980
-0.004510 -0.017054 -0.025773 0.004509 0.014189 -0.039649
0.042728 -0.013024 -0.019244 -0.043499 0.045684 0.010054
0.015005 -0.010075 0.039162 0.016261 -0.037514 -0.062384
-0.003289 0.038651 0.017095 -0.013405 -0.005792 0.032282
0.026071

z282 -0.023551 -0.016916 0.055024 -0.016100 -0.041479 0.007271
-0.013418 -0.003506 0.053405 0.001668 -0.020878 -0.001186
-0.025456 -0.010319 -0.030339 -0.028444 -0.025388 -0.007351
-0.047755 0.041893 0.018048 0.019912 -0.021496 -0.015960
-0.015327 0.016383 0.019677 0.014611 -0.024691 0.010684
0.020358

x283 -0.052469 -0.022800 -0.004883 -0.011729 -0.047788 -0.034283
-0.001204 0.060879 0.027174 -0.039107 0.036589 0.006554
0.029694 -0.034891 -0.064108 0.020043 0.034937 -0.016066
-0.005185 -0.021096 0.007702 0.011269 -0.024809 -0.001933
-0.010423 0.008870 0.032849 0.020031 -0.013326 -0.019603
0.016903

y283 -0.015080 -0.028764 0.037616 0.011541 -0.052836 0.020676
-0.004196 -0.015191 -0.025571 0.004780 0.012816 -0.036664
0.042973 -0.008940 -0.020830 -0.038096 0.043489 0.008217
0.012602 -0.010709 0.044611 0.015730 -0.035370 -0.058021
-0.003527 0.035035 0.008644 -0.010126 -0.006762 0.030557
0.024338

z283 -0.024948 -0.017044 0.057004 -0.010429 -0.047283 0.011685
-0.016113 -0.008242 0.051206 0.001356 -0.022956 0.002729
-0.016529 -0.005865 -0.034970 -0.023598 -0.019509 -0.010713
-0.047865 0.042979 0.019171 0.018882 -0.023147 -0.019740
-0.010451 0.015369 0.010402 0.019341 -0.025127 0.016070
0.020808

x284 -0.052895 -0.020029 -0.003919 -0.009526 -0.048475 -0.038282
0.000670 0.065329 0.027137 -0.036571 0.038996 0.008218
0.027601 -0.033750 -0.065724 0.027794 0.033606 -0.020753
0.005671 -0.022560 0.009429 0.006748 -0.020159 -0.000921
-0.007977 0.008143 0.026680 0.021674 -0.015493 -0.027021
0.009155

y284	-0.016582	-0.024984	0.033872	0.012938	-0.053796	0.020840
	-0.003260	-0.013586	-0.025007	0.004350	0.012699	-0.034151
	0.041294	-0.005497	-0.022392	-0.035396	0.041368	0.007031
	0.010260	-0.012422	0.047771	0.016994	-0.034096	-0.048591
	-0.002358	0.029453	0.001442	-0.007633	-0.008328	0.027558
	0.020876					
z284	-0.027462	-0.017070	0.059288	-0.006150	-0.052144	0.016159
	-0.018096	-0.010934	0.050330	-0.000299	-0.024655	0.005602
	-0.005991	-0.002705	-0.039178	-0.024356	-0.010820	-0.012962
	-0.047666	0.042243	0.021338	0.024115	-0.025697	-0.015757
	-0.004798	0.006923	0.001954	0.024105	-0.025090	0.019311
	0.020663					
x285	-0.052322	-0.017470	-0.004271	-0.007450	-0.046983	-0.042465
	0.002699	0.067360	0.026811	-0.031929	0.044829	0.009033
	0.027442	-0.033173	-0.064043	0.027552	0.025440	-0.021435
	0.014930	-0.023067	0.010043	-0.001944	-0.019072	0.002212
	-0.007044	-0.000042	0.028230	0.031086	-0.013466	-0.028271
	0.000438					
y285	-0.016926	-0.020716	0.029493	0.014530	-0.052729	0.019458
	-0.001747	-0.010980	-0.024861	0.004892	0.014597	-0.031942
	0.038348	-0.001214	-0.023256	-0.033928	0.037579	0.007418
	0.008747	-0.013491	0.048541	0.017034	-0.032879	-0.039221
	-0.002489	0.025189	-0.003468	-0.005644	-0.010248	0.023992
	0.013157					
z285	-0.029059	-0.015977	0.061519	-0.002889	-0.057095	0.018127
	-0.018912	-0.010874	0.048154	-0.002171	-0.026325	0.006649
	0.003083	0.004570	-0.044008	-0.021228	-0.001085	-0.013736
	-0.045003	0.043115	0.024089	0.028162	-0.027634	-0.011917
	0.000040	0.002678	-0.010485	0.026876	-0.025801	0.016953
	0.017464					
x286	-0.050130	-0.015209	-0.004127	-0.006978	-0.043305	-0.045865
	0.006849	0.068379	0.026867	-0.026963	0.049508	0.008233
	0.030006	-0.028923	-0.063720	0.024333	0.016674	-0.022593
	0.020039	-0.014401	0.013771	-0.014269	-0.017838	0.003745
	-0.002928	-0.007189	0.023329	0.033361	-0.006223	-0.033724
	-0.006709					
y286	-0.016435	-0.015751	0.024488	0.016156	-0.049931	0.017492
	0.000556	-0.008139	-0.025161	0.005570	0.017721	-0.029670
	0.034448	0.002833	-0.023351	-0.032725	0.032150	0.008348
	0.007243	-0.013860	0.048646	0.016394	-0.030287	-0.029060
	-0.002316	0.021014	-0.006189	-0.005045	-0.009663	0.020348
	0.006291					

z286	-0.029649	-0.013596	0.061387	0.000369	-0.061101	0.017615
	-0.018566	-0.010694	0.044451	-0.002981	-0.024324	0.006456
	0.007367	0.010857	-0.045223	-0.016584	0.001485	-0.012310
	-0.040096	0.042237	0.024141	0.027020	-0.029248	-0.004360
	0.001710	-0.004943	-0.015658	0.035889	-0.019627	0.017007
	0.014685					
x287	-0.045733	-0.011036	-0.005665	-0.005339	-0.040075	-0.050063
	0.008804	0.067336	0.025328	-0.020642	0.052023	0.005993
	0.026913	-0.019726	-0.061110	0.030547	0.008046	-0.026726
	0.028697	-0.013677	0.015448	-0.025260	-0.007359	0.010499
	-0.003347	-0.008782	0.013897	0.034751	0.006041	-0.031490
	-0.016927					
y287	-0.015213	-0.010054	0.019225	0.018358	-0.046455	0.015301
	0.002062	-0.005534	-0.026097	0.005281	0.021504	-0.026991
	0.029349	0.005670	-0.022622	-0.031320	0.027412	0.008646
	0.006159	-0.015563	0.046025	0.015744	-0.026945	-0.020084
	-0.002300	0.019077	-0.008543	-0.004823	-0.007498	0.018524
	-0.001205					
z287	-0.029656	-0.011179	0.060313	0.003181	-0.064871	0.015734
	-0.018841	-0.011530	0.041918	-0.006205	-0.021489	0.003527
	0.011137	0.015589	-0.043264	-0.017320	0.001752	-0.008601
	-0.037571	0.046311	0.025668	0.021130	-0.034332	0.002746
	0.001053	-0.016135	-0.018653	0.043415	-0.007527	0.014383
	0.010711					
x288	-0.041626	-0.005909	-0.007919	-0.003236	-0.032225	-0.049768
	0.006964	0.061466	0.024277	-0.011695	0.048337	0.001470
	0.021261	-0.018577	-0.056684	0.034353	0.010199	-0.030037
	0.028314	-0.028521	0.006900	-0.029211	0.004152	0.010143
	-0.002992	-0.016195	0.009392	0.027703	0.011482	-0.026281
	-0.018450					
y288	-0.013697	-0.004188	0.013699	0.021412	-0.041218	0.013157
	0.002874	-0.003021	-0.027968	0.004442	0.025772	-0.023712
	0.024069	0.006728	-0.020550	-0.029423	0.023668	0.009040
	0.005206	-0.019664	0.040592	0.015493	-0.022206	-0.013877
	0.000264	0.019377	-0.007900	-0.005708	-0.008720	0.015766
	-0.007698					
z288	-0.027490	-0.008223	0.058110	0.007607	-0.067703	0.010167
	-0.020042	-0.014492	0.036250	-0.006838	-0.015820	-0.001059
	0.012360	0.025033	-0.032469	-0.017720	-0.000114	-0.004363
	-0.032963	0.053349	0.030196	0.014112	-0.032114	0.005459
	0.001671	-0.013961	-0.018185	0.046079	0.001035	0.003136
	0.010722					

x289	-0.034665	-0.001966	-0.010831	-0.004164	-0.023776	-0.052274
	0.002305	0.056936	0.023295	-0.008929	0.044928	0.001807
	0.020844	-0.011596	-0.049630	0.033031	0.011547	-0.034571
	0.018380	-0.024425	0.015032	-0.034066	0.010336	0.013592
	-0.006773	-0.006983	-0.010179	0.006214	0.020540	-0.044876
	-0.020726					
y289	-0.011711	0.001961	0.008151	0.024308	-0.035346	0.010756
	0.002365	-0.000541	-0.029022	0.002359	0.030615	-0.017164
	0.020873	0.009985	-0.017521	-0.026922	0.018664	0.009544
	0.000211	-0.018981	0.041449	0.013178	-0.017563	-0.008329
	0.002915	0.023956	-0.013032	-0.009134	-0.006633	0.008015
	-0.011693					
z289	-0.023000	-0.005798	0.054196	0.014916	-0.065912	0.009382
	-0.024779	-0.018321	0.033490	-0.007129	-0.017693	-0.006175
	0.013767	0.031176	-0.026392	-0.018002	0.005081	0.000283
	-0.023645	0.049974	0.020179	0.006690	-0.030220	-0.002383
	0.005380	-0.005578	-0.003095	0.046837	0.008732	0.000133
	0.015224					
x290	-0.025096	-0.000124	-0.015388	-0.002384	-0.019203	-0.053556
	0.000083	0.051291	0.022835	-0.007480	0.036922	-0.004134
	0.021087	-0.004156	-0.048814	0.034415	0.006535	-0.041202
	0.018647	-0.017593	0.030282	-0.052542	0.018739	0.018787
	-0.006750	-0.002121	-0.013183	0.013685	0.037728	-0.039511
	-0.005139					
y290	-0.009772	0.007662	0.003232	0.027654	-0.031060	0.008751
	0.001935	0.002239	-0.031023	0.000220	0.033625	-0.010964
	0.018035	0.010906	-0.016423	-0.026833	0.014464	0.010178
	-0.003227	-0.019871	0.039271	0.010941	-0.011401	-0.003543
	0.008360	0.023913	-0.013891	-0.006268	-0.007264	0.008475
	-0.012279					
z290	-0.017762	-0.002790	0.052483	0.021531	-0.060326	0.007878
	-0.028147	-0.017095	0.031070	-0.006608	-0.021369	-0.009873
	0.015450	0.037137	-0.022435	-0.011812	0.011882	0.002011
	-0.019077	0.047705	0.005781	-0.002015	-0.025031	-0.011165
	-0.003117	0.008792	-0.004061	0.030468	0.016818	-0.011700
	0.004424					
x291	-0.016599	0.002335	-0.018099	0.001156	-0.014850	-0.056288
	-0.002835	0.047654	0.020283	0.001765	0.028833	-0.009335
	0.019679	-0.000800	-0.044032	0.034404	0.015843	-0.040755
	0.016918	-0.033529	0.016577	-0.060782	0.028820	0.015520
	-0.009027	0.001877	-0.023432	0.011161	0.022785	-0.029555
	-0.016671					

y291	-0.007595	0.012637	-0.001730	0.032171	-0.025858	0.005769
	0.000345	0.006230	-0.032634	-0.000297	0.037742	-0.004774
	0.016474	0.013313	-0.015107	-0.025695	0.010512	0.013548
	-0.006929	-0.021797	0.029557	0.006902	-0.005967	0.002050
	0.011592	0.025681	-0.017697	-0.004786	-0.010644	0.010156
	-0.021061					
z291	-0.013038	0.002134	0.049691	0.026831	-0.054841	0.008978
	-0.029765	-0.018439	0.029626	-0.007596	-0.026581	-0.012528
	0.013425	0.036318	-0.018665	-0.005128	0.014817	-0.006834
	-0.016976	0.047034	0.006545	-0.009137	-0.018614	-0.017947
	-0.006951	0.011211	0.002360	0.020094	0.033274	-0.016205
	0.007482					
x292	-0.007477	0.004497	-0.019295	0.002149	-0.009158	-0.055358
	-0.004246	0.043141	0.019571	0.007900	0.022544	-0.013187
	0.018400	0.001674	-0.037720	0.037131	0.021674	-0.041683
	0.013384	-0.041050	0.010635	-0.065359	0.038560	0.018714
	-0.011442	0.012955	-0.029978	0.006963	0.020486	-0.025668
	-0.026842					
y292	-0.004862	0.017219	-0.006044	0.035883	-0.019945	0.004547
	-0.001475	0.009751	-0.033153	-0.001667	0.040556	0.000388
	0.014034	0.016014	-0.014395	-0.024614	0.005987	0.015192
	-0.010035	-0.021869	0.024485	0.003670	0.000594	0.009617
	0.016354	0.027557	-0.017904	-0.004637	-0.010121	0.010347
	-0.027082					
z292	-0.008835	0.007710	0.046130	0.033500	-0.049962	0.008138
	-0.030665	-0.019321	0.027483	-0.006411	-0.030467	-0.015401
	0.013638	0.034485	-0.016071	0.001519	0.019493	-0.012528
	-0.014315	0.043422	0.002398	-0.018154	-0.012871	-0.026420
	-0.009894	0.011970	0.005375	0.011736	0.042721	-0.018503
	0.006629					
x293	0.002841	0.005781	-0.021500	0.002273	-0.006194	-0.052285
	-0.002544	0.036415	0.021277	0.007418	0.012740	-0.017270
	0.020143	0.004584	-0.034782	0.034757	0.021460	-0.041606
	0.016599	-0.042614	0.014999	-0.066611	0.040980	0.012205
	-0.003402	0.020078	-0.024046	0.003899	0.021864	-0.024179
	-0.012763					
y293	-0.001190	0.021067	-0.010194	0.039216	-0.014659	0.004065
	-0.002939	0.013298	-0.031509	-0.006516	0.039586	0.004554
	0.011718	0.022227	-0.016588	-0.026280	-0.000184	0.018320
	-0.009020	-0.019839	0.023985	0.002326	0.005253	0.012994
	0.024580	0.030622	-0.013432	-0.005549	-0.009845	0.007978
	-0.021195					

z293	-0.005230	0.014067	0.041798	0.040679	-0.043961	0.006710
	-0.031613	-0.020731	0.023934	-0.001207	-0.029694	-0.018352
	0.014762	0.027546	-0.011658	0.010712	0.023868	-0.019704
	-0.014825	0.037351	-0.006692	-0.027595	-0.003888	-0.028652
	-0.013346	0.007957	0.005253	0.006814	0.052053	-0.015844
	-0.002576					
x294	0.012827	0.006118	-0.022526	0.002392	-0.005934	-0.051246
	-0.000188	0.030906	0.021356	0.007563	0.006665	-0.024991
	0.019661	0.003475	-0.031957	0.030334	0.025554	-0.035828
	0.023426	-0.046581	0.013191	-0.068940	0.039341	0.002808
	0.006642	0.019949	-0.008038	-0.001490	0.013692	-0.020873
	-0.001541					
y294	0.002569	0.023590	-0.013257	0.042517	-0.009596	0.002645
	-0.004465	0.017807	-0.030056	-0.011376	0.038874	0.006438
	0.007665	0.029150	-0.020188	-0.028950	-0.005116	0.023638
	-0.004831	-0.018735	0.021742	-0.000845	0.007659	0.014567
	0.032407	0.032850	-0.004366	-0.007328	-0.011235	0.004097
	-0.014388					
z294	-0.001871	0.021342	0.036318	0.047434	-0.037819	0.006055
	-0.031956	-0.022322	0.021140	0.004688	-0.028366	-0.019973
	0.017553	0.019231	-0.007367	0.018962	0.027650	-0.026710
	-0.017726	0.032302	-0.014354	-0.033309	0.007052	-0.028075
	-0.014482	0.002598	0.002911	0.003658	0.058494	-0.010625
	-0.008528					
x295	0.021476	0.006387	-0.021892	-0.000726	-0.001496	-0.050136
	0.003220	0.027254	0.019856	0.012317	0.001489	-0.028847
	0.026326	0.004118	-0.023217	0.029746	0.030858	-0.034951
	0.018481	-0.037746	0.013102	-0.075739	0.039792	-0.007295
	-0.006718	0.023545	-0.009824	-0.003899	0.007029	-0.015630
	-0.003172					
y295	0.005497	0.026102	-0.016034	0.043041	-0.002535	0.000598
	-0.006930	0.023053	-0.031023	-0.012934	0.034223	0.009995
	0.005317	0.034178	-0.019289	-0.027756	-0.007582	0.021614
	-0.008868	-0.012972	0.019281	-0.006466	0.012604	0.017139
	0.025243	0.030093	-0.004550	-0.007233	-0.011281	0.006048
	-0.018096					
z295	0.002021	0.028448	0.030129	0.055428	-0.032017	0.005652
	-0.031868	-0.023506	0.019989	0.009582	-0.023118	-0.022193
	0.021092	0.011057	-0.005565	0.024864	0.029844	-0.027740
	-0.016977	0.026032	-0.023468	-0.037707	0.016597	-0.026965
	-0.007433	0.001116	0.003176	0.000449	0.059042	-0.007262
	-0.010372					

x296	0.028536	0.007656	-0.023167	-0.003206	-0.000528	-0.046526
	0.005244	0.017080	0.017371	0.016712	-0.001125	-0.029772
	0.027855	-0.007279	-0.017125	0.032605	0.030813	-0.041884
	0.012312	-0.034055	0.016268	-0.077706	0.041999	-0.005925
	-0.005438	0.005117	-0.008812	-0.001137	0.010193	0.008108
	-0.004852					
y296	0.008727	0.028285	-0.019346	0.044100	0.003578	0.000493
	-0.011589	0.024744	-0.033678	-0.014357	0.028853	0.014496
	0.000404	0.031203	-0.018557	-0.025762	-0.012230	0.012520
	-0.014297	-0.009935	0.018409	-0.012542	0.018404	0.022160
	0.022855	0.017397	-0.001784	-0.004466	-0.002411	0.018154
	-0.020320					
z296	0.005403	0.035157	0.024076	0.061992	-0.027176	0.003873
	-0.029551	-0.023007	0.020982	0.013777	-0.016476	-0.025122
	0.025655	0.008301	-0.007809	0.027562	0.031937	-0.021200
	-0.010981	0.021352	-0.029711	-0.038069	0.025294	-0.024104
	0.004504	0.002408	0.004898	-0.000400	0.050974	-0.007111
	-0.005638					
x297	0.036157	0.006482	-0.021232	-0.007153	0.002324	-0.042479
	0.007383	0.010579	0.017167	0.018257	0.001794	-0.027847
	0.030745	-0.011599	-0.011389	0.038812	0.029044	-0.033080
	0.015583	-0.034579	0.008793	-0.069359	0.041658	-0.002570
	-0.015646	0.007961	-0.009156	-0.015820	0.009665	0.012122
	-0.004265					
y297	0.012461	0.027870	-0.021132	0.045292	0.009258	-0.001710
	-0.016004	0.027582	-0.033806	-0.015063	0.024481	0.020397
	-0.004095	0.038487	-0.021338	-0.020280	-0.017093	0.013716
	-0.012584	-0.009943	0.007626	-0.013952	0.019608	0.035001
	0.019697	0.017705	0.000681	-0.005289	0.001861	0.014316
	-0.021299					
z297	0.008642	0.042596	0.016750	0.063796	-0.018801	0.004532
	-0.027680	-0.021545	0.020858	0.015392	-0.008197	-0.027963
	0.032312	-0.007845	-0.002957	0.030974	0.032435	-0.020095
	-0.010284	0.014078	-0.031791	-0.036826	0.038421	-0.032919
	0.009141	0.004359	0.004799	-0.011092	0.045254	-0.000162
	-0.006962					
x298	0.044842	0.003586	-0.017225	-0.012552	0.004888	-0.038274
	0.013193	0.002511	0.016633	0.019968	0.003126	-0.030805
	0.037414	-0.014568	-0.005379	0.035882	0.025789	-0.023470
	0.018460	-0.033717	0.008715	-0.061895	0.032941	-0.003206
	-0.015981	0.014149	-0.007827	-0.019279	0.002286	0.018189
	0.003812					

y298	0.017100	0.027757	-0.022642	0.043610	0.013292	-0.003970
	-0.016029	0.028702	-0.031281	-0.015587	0.018715	0.018869
	-0.006510	0.045418	-0.023413	-0.020382	-0.017092	0.017126
	-0.010789	-0.009530	0.004993	-0.013213	0.018824	0.036987
	0.019817	0.022422	0.002194	-0.000497	0.003431	0.006013
	-0.017163					
z298	0.014898	0.047913	0.007412	0.064406	-0.009267	0.001951
	-0.028709	-0.018320	0.016680	0.018745	-0.005747	-0.029405
	0.037765	-0.022572	0.003985	0.034296	0.025006	-0.018066
	-0.007990	0.008923	-0.034546	-0.036820	0.042103	-0.028406
	0.019909	0.008713	0.005455	-0.017899	0.031530	0.018342
	0.003144					
x299	0.050212	0.000671	-0.013018	-0.015477	0.002901	-0.033550
	0.015689	-0.007336	0.015811	0.019213	0.004063	-0.029864
	0.040008	-0.014259	-0.005460	0.033598	0.027246	-0.010477
	0.019768	-0.031138	0.003764	-0.057846	0.025136	0.003641
	-0.010116	0.016803	-0.011223	-0.009395	0.000205	0.015243
	0.010251					
y299	0.021877	0.027120	-0.026421	0.040349	0.014112	-0.004707
	-0.018841	0.025674	-0.031122	-0.016117	0.012202	0.019526
	-0.010506	0.048210	-0.027593	-0.018699	-0.015106	0.021790
	-0.007889	-0.010204	-0.003198	-0.018555	0.017694	0.037398
	0.019565	0.027218	0.004158	0.007858	0.012606	-0.002547
	-0.015757					
z299	0.021140	0.051612	-0.000438	0.063219	-0.003348	-0.003306
	-0.030216	-0.015222	0.012498	0.020952	-0.007616	-0.030388
	0.040055	-0.026988	0.006941	0.032606	0.025012	-0.013058
	-0.006323	0.011628	-0.031374	-0.035102	0.042060	-0.014619
	0.028761	0.010723	0.002576	-0.013654	0.012369	0.027520
	0.019644					
x300	0.052636	-0.002791	-0.004583	-0.017341	0.003581	-0.028731
	0.018680	-0.011815	0.015747	0.016657	0.005987	-0.026385
	0.043740	-0.013128	-0.005972	0.036285	0.025057	-0.001015
	0.018365	-0.023516	0.004448	-0.051556	0.019771	0.009914
	-0.006373	0.016675	-0.013053	-0.012945	-0.004301	0.013370
	0.010683					
y300	0.028234	0.025266	-0.028530	0.033229	0.015591	-0.008132
	-0.019900	0.026788	-0.029622	-0.017439	0.002462	0.019888
	-0.011257	0.051019	-0.030029	-0.009508	-0.019612	0.020634
	-0.005143	0.001705	-0.000399	-0.022197	0.018762	0.039991
	0.016942	0.028482	0.009901	-0.002603	0.015568	-0.005241
	-0.019257					

z300	0.026141	0.054469	-0.006659	0.060732	0.003719	-0.007993
	-0.031856	-0.011661	0.008578	0.024006	-0.007499	-0.029259
	0.040547	-0.028307	0.009390	0.031567	0.026829	-0.007164
	-0.003022	0.011393	-0.032934	-0.029671	0.039569	-0.000745
	0.032583	0.013663	-0.001742	-0.008616	-0.003364	0.029972
	0.030626					
x301	0.051766	-0.006523	0.007270	-0.015782	0.007111	-0.020366
	0.021297	-0.017132	0.017748	0.013129	0.009351	-0.022034
	0.048716	-0.017359	-0.004486	0.034880	0.026228	0.008299
	0.013515	-0.023587	0.002788	-0.039677	0.012640	0.009798
	-0.000931	0.013535	-0.010051	-0.011813	-0.008762	0.014061
	0.013608					
y301	0.033406	0.023760	-0.028138	0.026148	0.019251	-0.006785
	-0.020093	0.022950	-0.024069	-0.017077	-0.009451	0.018585
	-0.010457	0.043345	-0.026957	-0.006613	-0.016791	0.021001
	-0.003119	-0.003091	-0.002838	-0.006422	0.017166	0.036761
	0.020917	0.022824	0.020497	-0.008417	0.010907	-0.002788
	-0.014638					
z301	0.031094	0.056086	-0.011944	0.057061	0.009784	-0.013520
	-0.033231	-0.007633	0.004980	0.026025	-0.005153	-0.027898
	0.040862	-0.026839	0.011941	0.031538	0.027749	-0.002175
	-0.000992	0.014887	-0.032418	-0.030337	0.035890	0.010215
	0.032649	0.018826	-0.005835	-0.004040	-0.015041	0.029898
	0.038528					
x302	0.047047	-0.009970	0.021559	-0.012454	0.011043	-0.011865
	0.024260	-0.019264	0.015743	0.009679	0.015670	-0.015035
	0.051979	-0.022310	-0.002843	0.035886	0.028176	0.013334
	0.003034	-0.021414	0.002015	-0.029749	0.007714	0.009054
	-0.001112	0.008055	-0.010970	-0.010190	-0.010844	0.015605
	0.010022					
y302	0.036443	0.022774	-0.025825	0.017483	0.021507	-0.005365
	-0.019534	0.022795	-0.029640	-0.013286	-0.015891	0.022079
	-0.012945	0.035200	-0.022630	-0.000461	-0.011024	0.013716
	-0.005095	-0.000083	-0.002974	0.005473	0.019827	0.031161
	0.012480	0.012813	0.022204	-0.010761	0.008569	0.000544
	-0.017665					
z302	0.035165	0.057217	-0.016063	0.051588	0.016021	-0.018966
	-0.032682	-0.003048	0.004311	0.026021	-0.001878	-0.027487
	0.039907	-0.024230	0.014422	0.031946	0.027408	0.002781
	0.000827	0.017247	-0.034090	-0.028824	0.030244	0.021459
	0.030486	0.022431	-0.006735	-0.000683	-0.021218	0.028660
	0.042208					

x303	0.039808	-0.014181	0.037232	-0.006222	0.015144	-0.003371
	0.027166	-0.020172	0.015280	0.003774	0.023678	-0.007167
	0.054724	-0.023833	-0.003471	0.034681	0.030785	0.020111
	-0.008294	-0.018040	0.002929	-0.020413	0.002509	0.008302
	-0.002500	0.006195	-0.013194	-0.006720	-0.009864	0.017195
	0.006798					
y303	0.040727	0.020272	-0.024661	0.009562	0.021495	-0.006541
	-0.018128	0.021811	-0.030878	-0.013334	-0.019471	0.026300
	-0.010620	0.037547	-0.020294	0.002292	-0.008277	0.014893
	-0.005576	0.012930	0.001084	0.011546	0.024146	0.029788
	0.003281	0.013028	0.019199	-0.006151	0.011716	0.007675
	-0.014964					
z303	0.037227	0.057898	-0.018082	0.044393	0.022789	-0.022260
	-0.030936	0.001832	0.005550	0.025105	0.003137	-0.025961
	0.035626	-0.021191	0.016582	0.031782	0.027266	0.004702
	0.000902	0.015190	-0.035766	-0.021649	0.020815	0.029798
	0.026696	0.023746	-0.004565	0.000087	-0.023060	0.023916
	0.044604					
x304	0.031093	-0.019514	0.054546	0.002939	0.021744	0.006978
	0.030956	-0.020323	0.015574	-0.002124	0.032362	-0.001373
	0.059744	-0.026338	-0.006216	0.031144	0.033238	0.024462
	-0.021441	-0.014364	0.001263	-0.016950	-0.008904	0.000684
	-0.005809	0.008045	-0.013960	-0.001287	-0.010383	0.017083
	0.005966					
y304	0.045050	0.014698	-0.020093	0.004371	0.024218	-0.003367
	-0.015060	0.019258	-0.024995	-0.015030	-0.016640	0.024541
	-0.006624	0.040855	-0.023144	0.000503	-0.006654	0.016123
	-0.002897	0.020931	-0.005849	0.012198	0.012442	0.022087
	-0.000574	0.022146	0.022987	-0.000290	0.009640	0.010586
	0.002995					
z304	0.037757	0.058271	-0.017123	0.036221	0.029404	-0.024744
	-0.027695	0.007108	0.008972	0.022752	0.008188	-0.023528
	0.029630	-0.017890	0.017841	0.028495	0.027671	0.005346
	0.000221	0.011674	-0.032543	-0.012333	0.008763	0.032631
	0.019924	0.021432	0.000488	-0.000876	-0.019988	0.016372
	0.046171					
x305	0.020470	-0.024213	0.070886	0.013409	0.025921	0.015924
	0.033746	-0.018061	0.014163	-0.008978	0.040266	0.003641
	0.062926	-0.027376	-0.009188	0.029658	0.036596	0.029670
	-0.032223	-0.011135	0.003186	-0.014612	-0.018983	-0.002464
	-0.010486	0.009692	-0.019630	-0.000253	-0.011447	0.016355
	0.000373					

y305	0.045874	0.010589	-0.013710	-0.000983	0.025916	-0.001863
	-0.014018	0.019614	-0.024205	-0.015447	-0.011803	0.027183
	-0.002040	0.040604	-0.016749	0.006127	-0.005659	0.014996
	-0.002413	0.026316	-0.007493	0.012740	0.008534	0.018205
	-0.005778	0.022812	0.016978	-0.002102	0.006844	0.016537
	0.009341					
z305	0.036068	0.058188	-0.013649	0.026775	0.035726	-0.026143
	-0.023653	0.013620	0.014767	0.020621	0.014343	-0.019455
	0.020625	-0.015481	0.019743	0.025441	0.026484	0.004073
	-0.002566	0.003905	-0.028837	0.001918	-0.006633	0.033006
	0.012586	0.015743	0.007756	-0.005673	-0.012633	0.007124
	0.043580					
x306	0.010091	-0.027145	0.083123	0.023646	0.027130	0.023246
	0.035940	-0.014319	0.010784	-0.015049	0.046225	0.006533
	0.064898	-0.027172	-0.012433	0.026753	0.039127	0.034711
	-0.039000	-0.007261	0.007744	-0.013740	-0.026526	-0.003382
	-0.015281	0.010051	-0.031432	-0.001706	-0.012792	0.014522
	-0.009890					
y306	0.044483	0.005270	-0.005111	-0.005136	0.028171	0.000659
	-0.013485	0.021007	-0.025516	-0.016011	-0.006200	0.033350
	0.004221	0.037035	-0.008677	0.010444	0.001031	0.014181
	-0.008923	0.034218	-0.003434	0.015541	0.008607	0.015531
	-0.016080	0.011700	0.009872	-0.000710	0.006575	0.018830
	0.007720					
z306	0.032315	0.057292	-0.007775	0.016749	0.041915	-0.026585
	-0.018916	0.021091	0.021998	0.018923	0.021660	-0.014436
	0.009236	-0.012562	0.021823	0.023315	0.025698	0.000090
	-0.008055	-0.007071	-0.027341	0.018603	-0.025014	0.029497
	0.005577	0.009346	0.015797	-0.012095	-0.004291	-0.006475
	0.036333					
x307	0.002188	-0.022087	0.080412	0.025128	0.024663	0.025081
	0.032217	-0.018316	0.011513	-0.012315	0.043743	0.002583
	0.050164	-0.024772	-0.009595	0.010963	0.014582	0.033564
	-0.029244	-0.005923	0.009262	-0.008634	-0.016810	-0.010830
	0.002457	0.002357	-0.009443	0.005792	-0.001182	0.014792
	-0.024834					
y307	0.040242	-0.006697	0.014682	-0.007232	0.031718	0.013465
	-0.011900	0.019892	-0.020380	-0.018443	0.002690	0.035811
	0.012430	0.032949	-0.000153	0.011083	0.010611	0.006566
	-0.014399	0.038921	0.013641	0.013807	-0.004774	0.000454
	-0.007486	0.013082	0.028973	0.003718	-0.011249	0.024417
	0.020603					

z307	0.023163	0.055103	-0.001328	-0.003927	0.052407	-0.024438
	-0.011020	0.027143	0.037741	0.009572	0.028160	-0.004724
	0.001938	-0.011548	0.023161	0.005883	0.013390	0.005489
	-0.008808	0.000521	-0.018817	0.024744	-0.047391	-0.003232
	-0.009859	0.017315	0.014452	-0.009786	-0.011602	0.007269
	0.009830					
x308	-0.003061	-0.011695	0.065659	0.025952	0.015155	0.022458
	0.027913	-0.018763	0.008376	-0.009261	0.034754	-0.007438
	0.035425	-0.017318	-0.006297	-0.004635	0.002501	0.031192
	-0.013843	0.000628	0.010913	-0.006441	-0.009838	-0.015180
	0.012441	-0.004544	-0.007260	0.003266	-0.001220	0.009380
	-0.037349					
y308	0.036509	-0.012485	0.026456	-0.008067	0.031746	0.022794
	-0.011444	0.019954	-0.016763	-0.019800	0.005252	0.038638
	0.015187	0.031908	0.006668	0.012759	0.007154	0.006397
	-0.019436	0.040898	0.024047	0.018235	-0.008970	-0.007280
	-0.008072	0.017398	0.034511	0.000455	-0.017718	0.019994
	0.027203					
z308	0.013588	0.050467	0.005441	-0.020456	0.058498	-0.022477
	-0.005049	0.030235	0.048796	0.001603	0.032114	0.002934
	-0.005371	-0.009906	0.025208	-0.007489	0.002736	0.010226
	-0.008738	0.006454	-0.008698	0.023746	-0.058017	-0.030496
	-0.017943	0.023560	0.012494	-0.003862	-0.015303	0.016712
	-0.014274					
x309	-0.007214	-0.003135	0.050324	0.026390	0.004511	0.017690
	0.025081	-0.018072	0.007269	-0.007990	0.025558	-0.017761
	0.024361	-0.008676	-0.005740	-0.017098	-0.003948	0.027150
	-0.002431	0.008801	0.010229	-0.003649	-0.009680	-0.018159
	0.017483	-0.011141	-0.006471	-0.003509	-0.005051	0.004086
	-0.041515					
y309	0.032935	-0.013854	0.030697	-0.005061	0.028120	0.026639
	-0.013709	0.018829	-0.018364	-0.018139	0.002849	0.038077
	0.014693	0.030631	0.009934	0.014858	0.009337	0.007120
	-0.019199	0.038074	0.023968	0.015986	-0.006249	-0.007669
	-0.008755	0.017329	0.032292	-0.001608	-0.014096	0.007438
	0.018674					
z309	0.004661	0.046700	0.010835	-0.031942	0.060543	-0.019716
	-0.000615	0.030999	0.054862	-0.004325	0.033630	0.009105
	-0.011910	-0.007881	0.024919	-0.016385	-0.007188	0.013743
	-0.008440	0.009298	0.000787	0.019115	-0.063353	-0.046132
	-0.023074	0.026471	0.010378	0.000382	-0.014599	0.022618
	-0.029677					

x310	-0.011010	0.002598	0.036159	0.026702	-0.004783	0.008727
	0.023886	-0.015991	0.005786	-0.007059	0.017520	-0.026155
	0.015272	-0.002672	-0.006457	-0.024800	-0.003593	0.021687
	0.007763	0.016454	0.003240	0.000393	-0.010603	-0.015207
	0.015835	-0.011824	-0.010669	-0.007032	-0.014339	-0.003758
	-0.049291					
y310	0.029464	-0.014312	0.032613	-0.000350	0.021864	0.032328
	-0.017245	0.014914	-0.018280	-0.017896	-0.000799	0.032800
	0.014655	0.031296	0.008913	0.013266	0.007013	0.007868
	-0.018324	0.033075	0.024604	0.008959	-0.006653	-0.011869
	-0.003378	0.006847	0.035503	-0.009718	0.003587	0.000343
	0.019267					
z310	-0.003109	0.043976	0.014778	-0.038595	0.059459	-0.016595
	0.002234	0.029308	0.056338	-0.008947	0.033174	0.012463
	-0.016224	-0.005362	0.023282	-0.021028	-0.014624	0.015797
	-0.008425	0.009755	0.007037	0.010059	-0.064716	-0.056301
	-0.024634	0.026656	0.008163	0.003183	-0.012673	0.027222
	-0.034218					
x311	-0.015151	0.004261	0.024326	0.027934	-0.010575	0.001484
	0.023640	-0.015797	0.006289	-0.008379	0.012881	-0.032799
	0.009748	0.002413	-0.010495	-0.032259	-0.003927	0.013749
	0.016677	0.023988	-0.005169	0.003858	-0.013123	-0.015548
	0.007699	-0.009759	-0.012986	-0.012898	-0.010583	-0.010079
	-0.049585					
y311	0.026492	-0.012201	0.032172	0.003691	0.011711	0.031603
	-0.021064	0.012640	-0.019959	-0.017157	-0.007648	0.024810
	0.011698	0.030090	0.007518	0.012881	0.008042	0.011105
	-0.014895	0.026433	0.020901	0.003788	-0.008038	-0.004825
	0.007163	-0.005845	0.034776	-0.012523	0.008183	-0.004712
	0.010252					
z311	-0.010680	0.042493	0.017551	-0.042004	0.055604	-0.013346
	0.004174	0.026103	0.053969	-0.011826	0.032682	0.014932
	-0.019033	-0.001004	0.020306	-0.020944	-0.019488	0.016593
	-0.006551	0.009910	0.014338	-0.000381	-0.062417	-0.057268
	-0.024835	0.025035	0.003127	0.003595	-0.008024	0.027410
	-0.031999					
x312	-0.018031	0.002884	0.015307	0.027443	-0.015692	-0.007337
	0.026659	-0.016936	0.009911	-0.012382	0.008973	-0.040294
	0.008815	0.005930	-0.015550	-0.037441	-0.007371	0.005727
	0.027600	0.030439	-0.008038	0.011912	-0.015758	-0.013057
	0.002222	-0.007622	-0.005439	-0.019376	-0.005860	-0.010287
	-0.034416					

y312	0.022238	-0.008673	0.029797	0.009633	0.002520	0.029073
	-0.027900	0.010261	-0.023927	-0.015867	-0.013696	0.017812
	0.004916	0.027655	0.002076	0.008765	0.012243	0.013738
	-0.012865	0.021152	0.007641	-0.004616	-0.012719	0.005308
	0.011498	-0.016657	0.024699	-0.010713	0.016545	-0.012995
	-0.014067					
z312	-0.017458	0.041821	0.018796	-0.041534	0.048879	-0.009727
	0.005425	0.020460	0.049357	-0.013736	0.030517	0.014436
	-0.019446	0.004451	0.014942	-0.016289	-0.021536	0.016133
	-0.005005	0.008309	0.017592	-0.013849	-0.058564	-0.050464
	-0.022471	0.022003	-0.002470	0.004073	-0.000894	0.025664
	-0.023457					
x313	-0.022385	0.001146	0.008055	0.026492	-0.019094	-0.016347
	0.027582	-0.014269	0.006751	-0.012759	0.010341	-0.038001
	0.003950	0.004711	-0.015746	-0.038455	-0.009481	-0.003993
	0.032419	0.039611	-0.009596	0.018872	-0.012874	0.000635
	-0.009483	-0.006405	-0.003286	-0.024060	0.000791	-0.010893
	-0.041317					
y313	0.019105	-0.005747	0.027264	0.015058	-0.005933	0.022644
	-0.032736	0.004014	-0.021339	-0.018902	-0.022140	0.004911
	0.001116	0.026191	-0.011938	0.000428	0.013495	0.017287
	-0.005449	0.015495	-0.009918	-0.009559	-0.024110	0.011409
	0.018654	-0.024339	0.020443	-0.006445	0.026145	-0.017651
	-0.023517					
z313	-0.023970	0.041413	0.019636	-0.037024	0.039387	-0.005162
	0.005063	0.012546	0.040247	-0.013645	0.028005	0.012213
	-0.016721	0.011782	0.006970	-0.009963	-0.018699	0.013188
	-0.004319	0.005756	0.020211	-0.032843	-0.052191	-0.039262
	-0.022421	0.018028	-0.011698	0.004089	0.012185	0.019688
	-0.005774					
x314	-0.026413	-0.001400	0.002679	0.021097	-0.021550	-0.026364
	0.032718	-0.017433	0.012523	-0.012741	0.014417	-0.034889
	-0.002702	0.005133	-0.019189	-0.045750	-0.018996	-0.006893
	0.035708	0.042029	-0.003352	0.025194	-0.016141	0.015827
	-0.000477	-0.006536	0.006104	-0.024498	0.005121	0.004808
	-0.023382					
y314	0.015164	-0.003308	0.024819	0.020178	-0.012479	0.012079
	-0.038426	0.000594	-0.019453	-0.023149	-0.029456	-0.004252
	-0.003783	0.022429	-0.027353	-0.005570	0.013026	0.016096
	0.005040	0.016392	-0.031889	-0.009754	-0.032465	0.017029
	0.016004	-0.030245	0.013286	0.001161	0.037911	-0.022898
	-0.049097					

z314	-0.029714	0.041047	0.019395	-0.027834	0.026478	0.000199
	0.003882	0.001488	0.027930	-0.011267	0.024115	0.007150
	-0.012249	0.018522	-0.004236	-0.001534	-0.015970	0.010056
	-0.003135	-0.000647	0.021154	-0.056219	-0.044478	-0.016777
	-0.019574	0.009437	-0.016760	0.005187	0.031715	0.015948
	0.016618					
x315	-0.030968	-0.004915	-0.001962	0.016410	-0.021396	-0.034104
	0.034589	-0.014260	0.016115	-0.007665	0.026339	-0.024933
	-0.011417	0.007578	-0.012122	-0.053121	-0.019526	-0.001201
	0.038010	0.037115	-0.004743	0.030874	-0.005740	0.020373
	-0.009113	0.005375	0.006211	-0.022766	0.001316	0.006815
	-0.015692					
y315	0.009216	-0.001810	0.022046	0.020193	-0.017946	-0.003102
	-0.039059	-0.002882	-0.011750	-0.028094	-0.033591	-0.006084
	-0.008986	0.022332	-0.041104	-0.013995	0.002367	0.010125
	0.017743	0.020013	-0.044570	-0.002842	-0.033917	0.019752
	0.020829	-0.044453	-0.000381	0.012839	0.050789	-0.004599
	-0.073024					
z315	-0.035630	0.039346	0.018932	-0.014735	0.009925	0.005475
	0.001460	-0.010968	0.010923	-0.005011	0.019880	-0.000346
	-0.007144	0.025690	-0.018269	0.009351	-0.008880	0.006031
	-0.000376	-0.005575	0.019329	-0.081574	-0.029158	0.010523
	-0.015015	-0.003558	-0.020015	0.008615	0.052345	0.011973
	0.034257					
x316	-0.033969	-0.004833	-0.004584	0.011566	-0.017033	-0.037313
	0.037523	-0.009774	0.015773	-0.002536	0.031919	-0.014486
	-0.008422	0.008927	-0.002435	-0.046979	-0.012232	0.011766
	0.035089	0.035256	-0.001824	0.035659	0.001241	0.025285
	-0.019128	0.022691	0.004572	-0.029400	-0.004784	0.009754
	0.002622					
y316	0.000118	-0.004030	0.016849	0.018889	-0.020880	-0.013545
	-0.031587	-0.004515	-0.012177	-0.024200	-0.029388	-0.010470
	-0.012141	0.013956	-0.026171	-0.019092	0.006314	0.010157
	0.016920	0.012864	-0.039006	0.001931	-0.028242	0.028547
	0.024980	-0.014943	0.004773	0.006610	0.041070	-0.016583
	-0.046038					
z316	-0.031806	0.035398	0.018004	-0.000249	-0.004518	0.007640
	-0.003412	-0.009177	0.001438	-0.002159	0.007171	-0.005192
	0.013830	0.029984	-0.028293	0.030075	-0.010070	-0.005408
	0.001022	-0.018199	0.031899	-0.077079	-0.020934	0.011246
	-0.014657	-0.001419	-0.010191	0.020996	0.030084	0.016386
	0.043485					

x317	-0.035375	-0.002640	-0.007182	0.008874	-0.012275	-0.034238
	0.035756	-0.010792	0.017687	-0.001327	0.035328	-0.006353
	-0.011634	0.010006	-0.001674	-0.036754	-0.009881	0.020889
	0.036331	0.034106	0.003349	0.032226	-0.005651	0.026555
	-0.011610	0.030674	0.006286	-0.031174	0.003845	0.016694
	0.022831					
y317	-0.009431	-0.005856	0.011457	0.015269	-0.022306	-0.023657
	-0.021691	-0.004055	-0.008894	-0.020793	-0.021116	-0.009451
	-0.014758	0.006680	-0.009073	-0.025507	0.009435	0.009682
	0.016290	0.005437	-0.029954	0.007143	-0.021619	0.032035
	0.030106	0.007551	0.008106	0.002313	0.033272	-0.015709
	-0.021491					
z317	-0.027175	0.030987	0.017231	0.012535	-0.017270	0.008235
	-0.007844	-0.009184	-0.006374	0.001183	-0.002797	-0.012200
	0.027961	0.030879	-0.035757	0.045204	-0.011074	-0.015194
	0.002719	-0.028300	0.040830	-0.072590	-0.012610	0.011031
	-0.012525	-0.002013	0.002595	0.031486	0.010174	0.019465
	0.047606					
x318	-0.035453	0.000459	-0.008003	0.007340	-0.006117	-0.029987
	0.032272	-0.008377	0.017508	0.002746	0.030194	0.000375
	-0.014813	0.010789	-0.005348	-0.026580	-0.009595	0.026865
	0.030996	0.030372	-0.001794	0.033851	-0.004619	0.023032
	-0.010313	0.034699	0.001975	-0.033650	0.010928	0.020861
	0.031768					
y318	-0.017857	-0.006130	0.006515	0.010795	-0.021473	-0.034226
	-0.012172	-0.001358	-0.004865	-0.017238	-0.013862	-0.004485
	-0.017579	0.001785	0.005397	-0.031259	0.009554	0.008656
	0.014612	-0.000134	-0.024095	0.011744	-0.013568	0.031630
	0.032024	0.023174	0.006830	-0.001793	0.030490	-0.006856
	-0.003843					
z318	-0.021703	0.026614	0.016856	0.023207	-0.027280	0.005938
	-0.011680	-0.009250	-0.013084	0.005675	-0.010141	-0.019920
	0.037659	0.030022	-0.039337	0.054708	-0.010374	-0.020648
	0.001289	-0.034224	0.043477	-0.068263	-0.002592	0.011545
	-0.011540	-0.003860	0.013004	0.037381	-0.005507	0.020937
	0.047618					
x319	-0.034024	0.003405	-0.010584	0.008484	-0.002079	-0.022889
	0.028043	-0.009545	0.016012	0.003266	0.025504	0.007526
	-0.019576	0.015285	-0.012681	-0.013901	-0.008504	0.029489
	0.025588	0.030177	0.005780	0.035821	-0.011278	0.011244
	-0.010157	0.036806	0.005253	-0.043501	0.010622	0.023185
	0.027058					

y319	-0.025272	-0.005508	0.001021	0.006980	-0.020144	-0.043490
	-0.003668	0.000788	-0.000880	-0.015967	-0.006382	0.003312
	-0.020222	0.000234	0.016182	-0.033927	0.008447	0.007383
	0.013767	-0.002223	-0.013860	0.015460	-0.010053	0.026874
	0.032257	0.034706	0.007484	-0.010046	0.028360	0.004025
	0.006330					
z319	-0.015323	0.022592	0.016936	0.032098	-0.034741	0.001142
	-0.014739	-0.010182	-0.019084	0.010298	-0.013909	-0.027441
	0.042911	0.028161	-0.040114	0.058319	-0.010472	-0.023149
	-0.001142	-0.036944	0.042879	-0.061632	0.003728	0.011354
	-0.011147	-0.003995	0.019839	0.038713	-0.019677	0.019581
	0.043617					
x320	-0.031431	0.004641	-0.011969	0.010603	0.001034	-0.015253
	0.023978	-0.011715	0.016348	0.006751	0.015320	0.008903
	-0.020885	0.016209	-0.022382	-0.003117	-0.006329	0.033919
	0.024665	0.027837	0.010531	0.035767	-0.005957	0.009009
	-0.016181	0.030802	0.008253	-0.035274	0.005867	0.023088
	0.023895					
y320	-0.031698	-0.005196	-0.003258	0.003851	-0.018827	-0.052230
	0.004431	0.002347	0.004239	-0.013712	-0.001115	0.008856
	-0.021121	-0.001694	0.023540	-0.034522	0.006417	0.007832
	0.015690	-0.003771	-0.004916	0.017497	0.000194	0.026988
	0.027739	0.039985	0.006816	-0.011362	0.027017	0.013830
	0.016820					
z320	-0.008514	0.018966	0.017672	0.039182	-0.039673	-0.005893
	-0.017298	-0.010091	-0.023038	0.016174	-0.015512	-0.033927
	0.046556	0.025574	-0.038072	0.057698	-0.010646	-0.020045
	-0.005296	-0.036128	0.038899	-0.053733	0.010672	0.011878
	-0.014076	-0.001932	0.024982	0.042668	-0.029589	0.016545
	0.037449					
x321	-0.028290	0.004577	-0.014452	0.012193	0.008983	-0.003803
	0.016789	-0.014462	0.017526	0.010786	0.006836	0.010959
	-0.019720	0.016611	-0.030882	0.009706	-0.003491	0.036798
	0.021628	0.022431	0.008710	0.029890	-0.004612	0.011359
	-0.026146	0.021624	0.010989	-0.027975	0.001044	0.022807
	0.017165					
y321	-0.038119	-0.005202	-0.008414	0.000628	-0.013509	-0.056889
	0.009072	0.003753	0.010341	-0.012370	0.006090	0.015959
	-0.019739	-0.002351	0.030651	-0.030293	0.004099	0.007172
	0.016132	-0.006823	-0.001421	0.013946	0.005954	0.029905
	0.019230	0.041411	0.004262	-0.016727	0.025892	0.024797
	0.025266					

z321	-0.001382	0.015710	0.019062	0.043923	-0.041941	-0.015315
	-0.020101	-0.009826	-0.025811	0.022481	-0.014309	-0.039523
	0.046387	0.022370	-0.033149	0.051067	-0.011032	-0.014219
	-0.012153	-0.030459	0.032771	-0.044187	0.016466	0.010431
	-0.017296	0.002386	0.023979	0.042388	-0.035337	0.011541
	0.028185					
x322	-0.024878	0.002291	-0.016343	0.013080	0.017374	0.010157
	0.008862	-0.018180	0.020421	0.019494	-0.003050	0.009608
	-0.015657	0.015376	-0.041244	0.018889	0.004233	0.038085
	0.022404	0.014897	0.011883	0.024505	0.002903	0.011580
	-0.028696	0.011902	0.017421	-0.016867	-0.008657	0.018547
	0.013538					
y322	-0.045420	-0.007502	-0.012577	-0.002330	-0.008203	-0.056662
	0.012350	0.003411	0.019627	-0.006829	0.012971	0.018372
	-0.014962	-0.001844	0.033831	-0.026957	0.006700	0.005006
	0.022286	-0.011404	0.006988	0.011311	0.016735	0.031033
	0.018410	0.040617	0.001986	-0.020543	0.019845	0.032437
	0.037479					
z322	0.005901	0.012704	0.020439	0.046733	-0.040921	-0.026496
	-0.023416	-0.009583	-0.027618	0.028520	-0.008872	-0.045172
	0.042785	0.017943	-0.025706	0.038830	-0.012840	-0.004953
	-0.020055	-0.020666	0.022968	-0.034550	0.020180	0.008994
	-0.019906	0.008743	0.019019	0.039416	-0.035674	0.004216
	0.013871					
x323	-0.019297	-0.000418	-0.018629	0.012227	0.026183	0.024645
	-0.001421	-0.022120	0.023943	0.029268	-0.015498	0.009641
	-0.014236	0.009449	-0.049788	0.027794	0.008419	0.041121
	0.022696	0.004399	0.014632	0.014945	0.011817	0.010050
	-0.029744	0.000041	0.029568	0.006554	-0.018516	0.012329
	0.003160					
y323	-0.051226	-0.010436	-0.018339	-0.004858	-0.001892	-0.049231
	0.012091	0.000089	0.028751	-0.001069	0.013860	0.022858
	-0.012929	-0.003239	0.034514	-0.018311	0.003384	0.005369
	0.027112	-0.019655	0.013205	0.007986	0.024978	0.030630
	0.016974	0.026644	0.005125	-0.005661	0.016109	0.043068
	0.036398					
z323	0.013541	0.010418	0.021778	0.048082	-0.036778	-0.039370
	-0.026594	-0.009904	-0.028951	0.034236	-0.000031	-0.048839
	0.035582	0.012297	-0.015514	0.020799	-0.014862	0.008073
	-0.028973	-0.006449	0.009323	-0.022078	0.021206	0.007354
	-0.022660	0.013114	0.009894	0.030674	-0.030049	-0.009968
	0.003568					

x324	-0.011167	-0.002012	-0.017019	0.009498	0.031889	0.030592
	-0.012509	-0.023033	0.026391	0.034870	-0.025102	0.007276
	-0.015997	-0.000660	-0.052498	0.031949	0.011706	0.041858
	0.022927	-0.005173	0.016416	-0.006892	0.017171	0.007273
	-0.029213	0.003949	0.041242	0.021624	-0.033591	0.002746
	-0.008136					
y324	-0.052586	-0.013473	-0.021961	-0.005639	0.005212	-0.042370
	0.012817	-0.005161	0.033459	0.006965	0.015711	0.020519
	-0.012091	-0.000773	0.019264	-0.008113	0.009435	0.008642
	0.035206	-0.022009	0.012936	0.003686	0.029397	0.026362
	0.001176	0.021897	0.007641	-0.000677	0.005551	0.043510
	0.021063					
z324	0.022034	0.008171	0.022555	0.047529	-0.029399	-0.052960
	-0.030221	-0.010985	-0.031332	0.039558	0.011732	-0.051586
	0.023967	0.006627	-0.007430	-0.005535	-0.019387	0.025195
	-0.041316	0.012452	-0.006783	-0.002612	0.015023	0.008751
	-0.029412	0.017358	-0.008781	0.010491	-0.024796	-0.027041
	-0.017374					
x325	-0.002278	0.000759	-0.006223	0.008806	0.029488	0.033179
	-0.019906	-0.022228	0.021705	0.021619	-0.029870	0.000094
	-0.015430	0.010197	-0.036669	0.035163	0.014193	0.038758
	0.018594	-0.009150	0.008137	-0.004601	0.008627	0.002971
	-0.019850	-0.011312	0.035742	0.017474	-0.008096	-0.003069
	-0.008606					
y325	-0.050069	-0.017375	-0.024287	-0.006727	0.010243	-0.034601
	0.008666	-0.012245	0.039889	0.019175	0.012317	0.023965
	-0.007626	-0.007024	0.007969	0.003782	0.009933	0.017340
	0.038635	-0.032234	0.008189	0.003089	0.040242	0.019737
	-0.009218	0.018249	0.019910	0.018195	0.004849	0.026492
	0.016992					
z325	0.024504	0.006832	0.017411	0.042562	-0.010433	-0.063820
	-0.033543	-0.014799	-0.031227	0.038996	0.014421	-0.042868
	0.015536	0.018930	-0.002255	-0.010142	-0.019751	0.025631
	-0.029971	0.002702	-0.013008	-0.013385	-0.001594	-0.004096
	-0.029292	0.014842	-0.030211	0.011295	0.001874	-0.030174
	-0.016016					
x326	0.006809	0.004048	0.006282	0.007414	0.023084	0.028708
	-0.026066	-0.017875	0.017807	0.006366	-0.032933	-0.004044
	-0.014453	0.018863	-0.014739	0.031130	0.011984	0.031699
	0.013680	-0.012383	0.001853	-0.010337	-0.000089	-0.001431
	-0.009000	-0.017879	0.025553	0.007832	0.008545	-0.007640
	-0.005076					

y326	-0.046014	-0.018825	-0.021571	-0.010992	0.013888	-0.030940
	0.008088	-0.018994	0.039223	0.023568	0.010126	0.017941
	-0.005377	-0.001379	-0.004293	0.016927	0.011864	0.014184
	0.038784	-0.038289	0.005934	-0.003771	0.045610	0.013303
	-0.019212	0.025018	0.022923	0.032068	0.006653	0.030349
	0.003420					
z326	0.026592	0.004468	0.011748	0.037045	0.007469	-0.070805
	-0.034229	-0.018218	-0.030453	0.037833	0.016774	-0.033519
	0.008311	0.030976	-0.000117	-0.014544	-0.016896	0.024653
	-0.018237	-0.005513	-0.016668	-0.025433	-0.011078	-0.013074
	-0.031740	0.016558	-0.046163	0.015471	0.022073	-0.033309
	-0.015779					
x327	0.014001	0.007574	0.016867	0.003857	0.017639	0.022074
	-0.030630	-0.013106	0.015564	-0.006157	-0.035063	-0.005887
	-0.013680	0.025638	0.004189	0.023653	0.009774	0.024705
	0.012578	-0.018396	-0.004231	-0.015723	-0.008694	-0.003980
	0.001571	-0.018398	0.012117	0.001096	0.019242	-0.008642
	0.001229					
y327	-0.037874	-0.022207	-0.018957	-0.008391	0.014046	-0.027544
	0.002912	-0.024511	0.038273	0.023598	0.008302	0.013098
	0.000089	-0.000510	-0.004604	0.027110	0.008103	0.010428
	0.030964	-0.034940	0.005625	-0.010684	0.052744	0.004752
	-0.020227	0.026429	0.026884	0.031930	0.003108	0.026877
	-0.020780					
z327	0.028527	0.001180	0.005717	0.032944	0.023979	-0.073354
	-0.034286	-0.020610	-0.029996	0.038732	0.017093	-0.022827
	0.004042	0.039368	0.000812	-0.019259	-0.014199	0.024410
	-0.008150	-0.014885	-0.020790	-0.032438	-0.017406	-0.020008
	-0.032032	0.017837	-0.056514	0.017215	0.034035	-0.034956
	-0.017838					
x328	0.019865	0.011158	0.023876	0.001273	0.014009	0.015145
	-0.035370	-0.008622	0.012998	-0.014780	-0.037192	-0.004254
	-0.008438	0.029503	0.020090	0.014844	0.007863	0.019092
	0.011775	-0.025174	-0.011183	-0.022772	-0.015198	-0.004905
	0.010763	-0.016129	-0.000235	0.005967	0.027895	-0.008193
	0.005459					
y328	-0.030091	-0.026235	-0.013278	-0.011278	0.011260	-0.025569
	0.003463	-0.027857	0.038446	0.019973	0.005990	0.003660
	-0.002197	0.005336	-0.004880	0.031604	0.002787	0.006339
	0.028088	-0.029776	0.012075	-0.010719	0.051743	-0.004661
	-0.020818	0.020949	0.024340	0.015581	-0.002302	0.024912
	-0.031218					

z328	0.029675	-0.002789	0.000123	0.028472	0.038015	-0.073621
	-0.031851	-0.021140	-0.029455	0.041462	0.014955	-0.013549
	0.002914	0.046838	0.000004	-0.024958	-0.009531	0.021355
	0.000336	-0.021204	-0.018337	-0.037052	-0.020793	-0.027058
	-0.031642	0.018045	-0.061247	0.020126	0.040316	-0.034692
	-0.014052					
x329	0.023130	0.014418	0.025934	-0.002681	0.009845	0.007594
	-0.038873	-0.003292	0.010169	-0.019988	-0.038155	-0.003100
	-0.002243	0.032479	0.031496	0.004480	0.005988	0.012934
	0.013698	-0.033037	-0.012971	-0.027231	-0.022411	0.000522
	0.021344	-0.019677	-0.012545	0.012189	0.035473	-0.003037
	0.009323					
y329	-0.020690	-0.027601	-0.004523	-0.011681	0.010066	-0.024156
	0.002776	-0.031040	0.035191	0.015533	0.003250	0.003553
	-0.003936	0.010773	-0.004374	0.038353	-0.003206	0.007004
	0.023105	-0.026928	0.010860	-0.011441	0.047577	-0.018258
	-0.023346	0.027105	0.020402	0.012285	-0.010421	0.014957
	-0.030751					
z329	0.031284	-0.006371	-0.005294	0.026272	0.051205	-0.070433
	-0.028671	-0.019689	-0.029345	0.046435	0.011976	-0.002868
	0.002787	0.052625	-0.002675	-0.022827	-0.005724	0.020029
	0.006496	-0.027228	-0.019636	-0.045071	-0.020385	-0.034323
	-0.031996	0.020881	-0.057670	0.032374	0.044207	-0.032593
	-0.004410					
x330	0.025449	0.018907	0.024755	-0.004599	0.006893	0.002527
	-0.039168	0.003567	0.003173	-0.020228	-0.037426	0.003826
	0.008089	0.037775	0.036474	-0.002589	0.001818	0.007027
	0.012407	-0.039734	-0.015287	-0.027963	-0.031654	0.005035
	0.026712	-0.018306	-0.020668	0.024876	0.033910	-0.008925
	0.012170					
y330	-0.012734	-0.028654	0.004422	-0.015317	0.006209	-0.027763
	0.000135	-0.033715	0.032381	0.009944	-0.001908	0.001169
	-0.004472	0.010930	-0.001692	0.032974	-0.003664	0.005720
	0.022293	-0.027385	0.018697	-0.009070	0.045572	-0.023918
	-0.018564	0.019472	0.010472	0.010894	-0.009355	0.021357
	-0.022276					
z330	0.032299	-0.012727	-0.009426	0.023490	0.061717	-0.066329
	-0.022012	-0.017601	-0.026735	0.051350	0.005388	0.001554
	0.005202	0.056213	-0.003803	-0.028332	0.002459	0.014403
	0.012671	-0.029551	-0.009212	-0.048580	-0.011094	-0.040792
	-0.027331	0.016900	-0.051730	0.037495	0.044112	-0.023630
	0.003147					

x331	0.024808	0.022566	0.018104	-0.004696	0.000904	-0.000800
	-0.034601	0.008213	-0.006820	-0.017623	-0.040272	0.013354
	0.019723	0.045014	0.035837	-0.014035	-0.000018	-0.001563
	0.016374	-0.043523	-0.009308	-0.020660	-0.034643	-0.000558
	0.034536	-0.019119	-0.024079	0.042210	0.037421	0.003215
	0.028499					
y331	-0.004947	-0.026938	0.014174	-0.018935	0.003291	-0.032966
	-0.004276	-0.031177	0.027868	0.007820	-0.002542	0.000168
	-0.001757	0.009681	0.000388	0.029251	-0.002996	0.005341
	0.018433	-0.030246	0.021154	-0.004866	0.035041	-0.015783
	-0.016490	0.010782	-0.003892	0.008311	-0.011806	0.015060
	-0.024769					
z331	0.034347	-0.020999	-0.011543	0.021814	0.072432	-0.060762
	-0.013497	-0.014176	-0.022565	0.057915	0.002329	0.002643
	0.008937	0.059541	-0.005869	-0.028403	0.008451	0.009022
	0.015007	-0.031229	-0.004952	-0.049467	-0.002312	-0.040252
	-0.023983	0.017383	-0.048876	0.035984	0.041433	-0.013548
	-0.004047					
x332	0.021807	0.025847	0.007049	-0.003742	-0.001358	0.000973
	-0.027291	0.014204	-0.009898	-0.007179	-0.044411	0.024503
	0.028423	0.050214	0.030406	-0.026786	0.004525	-0.005833
	0.017768	-0.047308	-0.007806	-0.015465	-0.039392	-0.009632
	0.045533	-0.027387	-0.031503	0.055176	0.032916	0.019877
	0.038979					
y332	0.001179	-0.023200	0.021714	-0.021258	-0.003702	-0.036377
	-0.006418	-0.027111	0.019146	0.006143	-0.002761	-0.002350
	0.003194	0.011554	0.000528	0.023889	-0.002696	0.001888
	0.019089	-0.031414	0.027730	0.006390	0.022860	-0.007619
	-0.013196	0.002346	-0.011538	0.004706	-0.007060	0.007940
	-0.020431					
z332	0.036608	-0.030242	-0.010494	0.018647	0.080823	-0.055080
	-0.002176	-0.009992	-0.017011	0.062843	-0.000516	0.001097
	0.014876	0.065595	-0.009563	-0.028386	0.013032	0.003096
	0.014032	-0.032260	0.005897	-0.044735	0.008989	-0.040285
	-0.020921	0.020022	-0.045951	0.032750	0.031476	-0.009684
	-0.006514					
x333	0.019393	0.025328	-0.004640	-0.002031	-0.001138	0.004796
	-0.017506	0.022190	-0.005973	0.001105	-0.041229	0.030316
	0.032399	0.053016	0.022276	-0.035540	0.004858	-0.012619
	0.020234	-0.048524	-0.018156	-0.008922	-0.045420	-0.014632
	0.045574	-0.050211	-0.018549	0.050338	0.025907	0.030903
	0.021187					

y333 0.004973 -0.016810 0.023121 -0.020650 -0.010988 -0.032661
-0.007831 -0.020688 0.013298 0.009066 -0.009415 -0.003359
0.008537 0.015448 -0.000897 0.017677 -0.000047 0.002427
0.020689 -0.033361 0.031549 0.018113 0.008605 -0.007978
-0.001978 -0.007600 -0.017237 0.005639 -0.014498 0.006663
-0.003954

z333 0.039067 -0.040560 -0.008738 0.017182 0.087878 -0.048850
0.005761 0.000845 -0.007127 0.068228 -0.008345 0.002913
0.023089 0.070734 -0.017854 -0.028646 0.017356 -0.000885
0.013483 -0.028318 0.007925 -0.034334 0.022689 -0.048784
-0.011185 0.013760 -0.036472 0.040212 -0.015500 0.003844
0.002354

x334 0.018840 0.022664 -0.012080 0.003635 -0.001569 0.009467
-0.014573 0.028948 -0.003355 0.004603 -0.036553 0.036358
0.028371 0.039656 0.018232 -0.032873 -0.002302 -0.018341
0.023204 -0.039404 -0.025256 -0.008429 -0.044133 -0.013351
0.049556 -0.068499 0.005908 0.042649 0.018707 0.030366
0.019260

y334 0.008398 -0.009276 0.018790 -0.018859 -0.016463 -0.024926
-0.008183 -0.015287 0.012147 0.013926 -0.015271 -0.003810
0.011288 0.018351 -0.001955 0.008843 -0.000882 0.004985
0.022092 -0.036024 0.028136 0.023587 -0.008581 -0.009057
0.008169 -0.016373 -0.019227 0.003499 -0.019086 0.001300
0.011888

z334 0.041462 -0.045454 -0.007322 0.015217 0.084398 -0.047731
0.005219 0.006733 -0.003457 0.070331 -0.003744 0.012074
0.020566 0.071620 -0.022873 -0.029377 0.006572 -0.005797
0.010564 -0.020937 0.009547 -0.029000 0.025450 -0.051763
0.008865 0.014818 -0.029674 0.014201 -0.043477 0.001608
0.023508

x335 0.017829 0.020057 -0.018097 0.007693 -0.004844 0.010580
-0.008666 0.030078 -0.000526 0.008871 -0.028917 0.037385
0.022045 0.023866 0.022619 -0.038117 -0.007297 -0.025182
0.024195 -0.038345 -0.024391 -0.009328 -0.033939 -0.009243
0.047717 -0.071320 0.020986 0.049403 0.015187 0.027553
0.020340

y335 0.011988 -0.002996 0.012521 -0.017091 -0.020720 -0.017620
-0.006216 -0.011111 0.014208 0.019301 -0.018380 -0.003519
0.009258 0.015541 -0.002925 0.000194 -0.002128 0.008569
0.021765 -0.039205 0.022037 0.023411 -0.020236 -0.008024
0.015122 -0.023280 -0.017855 0.006016 -0.017356 -0.002782
0.020123

z335	0.043615	-0.050269	-0.002426	0.012974	0.073461	-0.047789
	0.007080	0.008482	-0.005558	0.069065	0.004236	0.012175
	0.012858	0.067156	-0.030215	-0.026618	0.003559	-0.011768
	0.007881	-0.017955	0.014920	-0.027717	0.028678	-0.044754
	0.023350	0.005073	-0.015028	-0.003628	-0.042409	0.001695
	0.036598					
x336	0.015769	0.017953	-0.023076	0.010035	-0.009010	0.013866
	-0.001647	0.026005	0.002485	0.009507	-0.024196	0.035003
	0.017096	0.013694	0.022262	-0.039192	-0.018142	-0.025823
	0.028233	-0.040159	-0.020245	-0.010882	-0.024985	-0.012006
	0.046466	-0.069913	0.029855	0.039537	0.015224	0.037988
	0.025828					
y336	0.014993	0.002310	0.005362	-0.015671	-0.024385	-0.010929
	-0.003113	-0.008154	0.018026	0.024582	-0.019879	-0.001739
	0.005045	0.009507	-0.005222	-0.006419	-0.004501	0.012320
	0.019848	-0.041679	0.012668	0.020153	-0.026101	-0.007842
	0.021496	-0.027436	-0.015537	0.005903	-0.011788	0.000850
	0.023616					
z336	0.045449	-0.054678	0.002150	0.010984	0.061828	-0.048229
	0.008551	0.010462	-0.006902	0.068344	0.012821	0.010389
	0.005722	0.060885	-0.033111	-0.023769	0.004813	-0.018170
	0.007920	-0.015850	0.018848	-0.025520	0.031832	-0.036365
	0.032415	-0.006953	-0.000905	-0.008339	-0.039321	0.001086
	0.038174					
x337	0.015673	0.017965	-0.024900	0.013517	-0.010251	0.013038
	0.003029	0.020878	0.002158	0.007762	-0.016676	0.033843
	0.014527	0.010364	0.021146	-0.036831	-0.028290	-0.026120
	0.030275	-0.036239	-0.018000	-0.013000	-0.020493	-0.021555
	0.036277	-0.054279	0.029963	0.025183	0.011707	0.038633
	0.027307					
y337	0.018023	0.008048	-0.001760	-0.013834	-0.027223	-0.005976
	0.000269	-0.006365	0.020870	0.028877	-0.019165	0.002984
	-0.000630	0.002387	-0.008876	-0.011309	-0.007954	0.013994
	0.015522	-0.040810	0.001487	0.014965	-0.030327	-0.011232
	0.026142	-0.024680	-0.013256	0.004444	-0.006288	0.006467
	0.024210					
z337	0.046445	-0.059386	0.005230	0.008006	0.050800	-0.045363
	0.010305	0.011767	-0.006068	0.066209	0.018869	0.007505
	0.000631	0.055057	-0.032516	-0.020965	0.004236	-0.022377
	0.008383	-0.014564	0.026723	-0.023047	0.031957	-0.023810
	0.043641	-0.021459	0.009137	-0.010192	-0.033522	0.003126
	0.036483					

x338	0.015180	0.017028	-0.022688	0.017159	-0.010616	0.012533
	0.005493	0.017363	-0.002348	0.005890	-0.011659	0.032301
	0.015600	0.009589	0.025415	-0.031337	-0.034419	-0.023161
	0.030847	-0.032793	-0.008962	-0.014139	-0.011583	-0.029492
	0.025060	-0.041896	0.025565	0.020683	0.006562	0.032171
	0.025733					
y338	0.019794	0.013109	-0.007696	-0.012157	-0.030907	-0.000673
	0.003884	-0.004682	0.020675	0.033605	-0.019034	0.009247
	-0.005522	-0.006278	-0.010204	-0.013069	-0.008682	0.015112
	0.008762	-0.038910	-0.005534	0.009693	-0.029664	-0.015750
	0.030701	-0.021340	-0.011437	0.009274	-0.001864	0.010776
	0.022160					
z338	0.047377	-0.062815	0.006987	0.004458	0.040709	-0.041643
	0.011611	0.013466	-0.003731	0.062239	0.024042	0.005101
	-0.002881	0.051527	-0.031837	-0.018468	0.002290	-0.023128
	0.012401	-0.012752	0.030959	-0.022130	0.029227	-0.012299
	0.049260	-0.033749	0.015787	-0.007002	-0.025984	0.003395
	0.033668					
x339	0.014816	0.015702	-0.019659	0.022432	-0.008095	0.013312
	0.007541	0.012027	-0.004064	-0.003219	-0.010789	0.027673
	0.019554	0.010389	0.029821	-0.027715	-0.043878	-0.020318
	0.037977	-0.028426	0.007634	-0.012830	-0.001703	-0.027297
	0.017823	-0.043010	0.031201	0.012890	0.006300	0.030324
	0.022054					
y339	0.020361	0.018001	-0.014903	-0.008695	-0.033603	0.006071
	0.009392	-0.006064	0.021568	0.034012	-0.022008	0.014656
	-0.007828	-0.017338	-0.011662	-0.015575	-0.010712	0.014367
	0.006613	-0.036073	-0.007811	0.005834	-0.026160	-0.013005
	0.038420	-0.028494	-0.002646	0.010510	0.006680	0.022307
	0.013357					
z339	0.048806	-0.065817	0.007786	-0.000146	0.030196	-0.037432
	0.011312	0.016374	-0.002151	0.058334	0.027863	0.003910
	-0.004804	0.050643	-0.028960	-0.016390	0.000380	-0.020843
	0.015824	-0.012929	0.032906	-0.020776	0.022067	-0.003641
	0.050838	-0.045618	0.013749	-0.001429	-0.021945	0.002350
	0.024509					
x340	0.014880	0.012951	-0.011438	0.027691	-0.002581	0.012425
	0.005593	0.010242	-0.007912	-0.009783	-0.007567	0.018944
	0.025315	0.018979	0.037096	-0.024978	-0.050511	-0.011399
	0.043178	-0.024459	0.019056	-0.007532	0.012175	-0.025004
	-0.001971	-0.031977	0.023398	0.005196	0.001592	0.023137
	0.015001					

y340	0.019559	0.021074	-0.018657	-0.005209	-0.035405	0.011579
	0.013543	-0.007199	0.018934	0.040097	-0.020582	0.017314
	-0.008845	-0.025262	-0.008502	-0.015511	-0.007886	0.016970
	0.002272	-0.035725	-0.017507	0.005546	-0.020624	-0.012321
	0.035279	-0.022282	-0.011283	0.012531	0.009524	0.030647
	-0.003411					
z340	0.050106	-0.067330	0.006155	-0.005293	0.019308	-0.031121
	0.011139	0.019172	-0.001214	0.052169	0.027218	0.005627
	-0.004073	0.049306	-0.025658	-0.014680	-0.000529	-0.017162
	0.019982	-0.013955	0.034617	-0.020754	0.013370	0.003747
	0.053163	-0.063965	0.009177	0.007296	-0.017777	0.000553
	0.006899					
x341	0.015827	0.010953	-0.002564	0.031553	0.005569	0.012887
	0.001084	0.008341	-0.007778	-0.024072	-0.005508	0.005129
	0.028732	0.028911	0.044421	-0.025485	-0.067526	-0.003261
	0.049212	-0.019843	0.044399	0.001295	0.026118	-0.016457
	-0.018162	-0.020530	0.026193	-0.003083	0.001393	0.019829
	0.023106					
y341	0.018282	0.025089	-0.022197	-0.002481	-0.035178	0.019161
	0.018663	-0.015766	0.018151	0.036735	-0.019958	0.017959
	-0.009876	-0.037191	0.000239	-0.013329	-0.022277	0.017804
	0.001892	-0.036101	-0.005277	0.008014	-0.016617	-0.006786
	0.046059	-0.020156	-0.009925	0.013585	0.012729	0.049153
	-0.004952					
z341	0.050222	-0.067379	0.002403	-0.012041	0.007837	-0.022466
	0.011808	0.022542	0.000033	0.045687	0.024750	0.009971
	-0.000081	0.049595	-0.022460	-0.010521	-0.000104	-0.011271
	0.023505	-0.016287	0.032732	-0.021230	0.000686	0.011583
	0.051885	-0.078360	-0.002763	0.017970	-0.012152	-0.000942
	-0.017509					
x342	0.015415	0.008597	0.004657	0.033456	0.014753	0.013995
	-0.006818	0.012820	-0.005635	-0.038527	-0.002732	-0.014597
	0.029358	0.041039	0.047254	-0.030627	-0.075353	0.001818
	0.049951	-0.010085	0.063416	0.010263	0.039343	-0.007301
	-0.044688	-0.000129	0.027251	-0.012341	0.007305	0.006044
	0.036460					
y342	0.017813	0.030696	-0.019404	0.001644	-0.028886	0.022759
	0.022798	-0.017038	0.014910	0.026823	-0.013989	0.014773
	-0.009352	-0.037497	0.009442	-0.012446	-0.032221	0.018070
	0.005953	-0.023726	0.002313	0.007589	-0.009528	-0.004953
	0.024141	-0.009696	-0.011062	0.011613	0.021880	0.051908
	-0.000075					

z342	0.047246	-0.065582	-0.002853	-0.019909	-0.002699	-0.011315
	0.012997	0.023656	0.000289	0.036432	0.019768	0.014571
	0.008509	0.050567	-0.019027	-0.001502	-0.000493	-0.003012
	0.022969	-0.019869	0.032349	-0.025641	-0.010102	0.024053
	0.051553	-0.082237	-0.025344	0.026345	-0.007396	0.005204
	-0.035381					
x343	0.008524	0.000174	0.003856	0.029467	0.016090	0.014190
	-0.008401	0.012228	-0.009439	-0.034624	-0.002293	-0.017440
	0.030714	0.036807	0.035181	-0.024512	-0.063936	-0.001945
	0.041734	-0.004556	0.046481	0.007544	0.033359	-0.004461
	-0.034266	-0.000258	0.023527	-0.009054	-0.003503	0.003954
	0.017089					
y343	0.019247	0.032663	-0.014909	0.009204	-0.025054	0.026602
	0.022077	-0.019485	0.007229	0.019468	-0.006517	0.012096
	-0.007735	-0.027235	0.015427	-0.015859	-0.046301	0.025595
	0.019167	-0.023861	0.016259	0.003234	0.005519	-0.011999
	0.015098	-0.015132	-0.004712	0.006235	0.021811	0.051999
	0.013022					
z343	0.042717	-0.062541	-0.003033	-0.026304	-0.004642	-0.004734
	0.013783	0.028185	0.001817	0.026594	0.023576	0.028142
	0.006272	0.045803	-0.015522	-0.005347	0.005854	0.005486
	0.020933	-0.022255	0.023661	-0.034611	-0.005813	0.034863
	0.060448	-0.071451	-0.013405	0.025059	0.002516	0.004552
	-0.029816					
x344	0.002218	-0.008483	-0.000090	0.024165	0.016227	0.013506
	-0.009965	0.012044	-0.011572	-0.029320	-0.003783	-0.018647
	0.028248	0.028815	0.021820	-0.018218	-0.044389	-0.007146
	0.028140	0.000821	0.030505	0.002786	0.025930	0.001416
	-0.021313	-0.001901	0.016000	-0.006387	-0.010360	-0.000127
	0.004066					
y344	0.018843	0.032007	-0.012486	0.013268	-0.021126	0.030282
	0.021203	-0.020841	-0.000981	0.014135	-0.001892	0.010502
	-0.004051	-0.020387	0.020660	-0.019014	-0.049357	0.029059
	0.023225	-0.019649	0.025112	0.002343	0.010332	-0.015439
	0.009813	-0.020372	-0.001704	0.003094	0.013806	0.052056
	0.009364					
z344	0.037341	-0.058287	-0.002903	-0.030740	-0.006883	0.000792
	0.013285	0.031017	0.003339	0.016095	0.026623	0.035333
	0.005016	0.040770	-0.012177	-0.007923	0.010616	0.013041
	0.018608	-0.023422	0.015919	-0.040510	-0.000584	0.043430
	0.066263	-0.055579	-0.003414	0.022395	0.011050	0.003892
	-0.023213					

x345	-0.002764	-0.015589	-0.004849	0.019734	0.016061	0.012883
	-0.013109	0.013400	-0.013278	-0.025953	-0.004595	-0.018166
	0.023833	0.021854	0.011518	-0.015324	-0.025112	-0.012470
	0.015773	0.006345	0.020940	-0.000682	0.021786	0.007669
	-0.011561	-0.004081	0.009721	-0.005421	-0.012203	-0.003720
	-0.003136					
y345	0.017625	0.029581	-0.012162	0.012741	-0.018393	0.031325
	0.022965	-0.023124	-0.003805	0.010925	-0.001702	0.003632
	-0.000640	-0.018533	0.022548	-0.015909	-0.044901	0.030728
	0.021425	-0.018022	0.023014	-0.000028	0.007992	-0.012161
	0.005045	-0.022271	-0.005985	0.000674	0.004268	0.043218
	0.008366					
z345	0.031951	-0.053072	-0.002570	-0.033297	-0.009716	0.004824
	0.011857	0.031725	0.006346	0.006040	0.028255	0.036028
	0.005918	0.037332	-0.009019	-0.008679	0.011287	0.015546
	0.017653	-0.024332	0.006754	-0.041230	0.006128	0.048141
	0.067286	-0.043579	0.001855	0.019362	0.011998	0.002611
	-0.014932					
x346	-0.005622	-0.020758	-0.009600	0.015681	0.015110	0.011050
	-0.016829	0.015024	-0.012060	-0.024485	-0.005322	-0.018067
	0.019293	0.015174	0.003845	-0.012347	-0.008176	-0.016457
	0.005608	0.009880	0.011117	-0.002380	0.016626	0.013314
	-0.004945	-0.006676	0.001616	-0.008709	-0.014593	-0.009528
	-0.005439					
y346	0.013526	0.024910	-0.012270	0.012241	-0.015538	0.034548
	0.022616	-0.023050	-0.010050	0.008690	-0.000602	-0.000272
	0.001410	-0.016811	0.022449	-0.018168	-0.036442	0.027173
	0.018080	-0.013004	0.023514	-0.004586	0.011897	-0.010392
	0.002050	-0.022271	-0.005180	0.005916	0.003428	0.037139
	0.008151					
z346	0.026270	-0.047653	-0.002295	-0.034371	-0.012918	0.008240
	0.008584	0.030713	0.009221	-0.002615	0.030014	0.033761
	0.007415	0.034623	-0.007912	-0.008978	0.011527	0.015912
	0.020158	-0.025830	-0.000370	-0.038779	0.014117	0.050486
	0.065064	-0.034608	0.004611	0.016356	0.009805	0.000079
	-0.014899					
x347	-0.007206	-0.025444	-0.012908	0.013477	0.015530	0.009959
	-0.022317	0.018427	-0.011294	-0.022914	-0.005300	-0.017150
	0.016570	0.007970	0.000807	-0.010708	0.005616	-0.021140
	-0.002242	0.015744	0.003570	-0.003052	0.013209	0.014040
	0.003109	-0.003304	-0.002974	-0.013475	-0.017686	-0.015297
	-0.001825					

y347	0.008191	0.020935	-0.014939	0.009014	-0.016343	0.035548
	0.023299	-0.023487	-0.012948	0.003697	-0.000344	-0.004949
	0.000241	-0.013536	0.017170	-0.020019	-0.024319	0.022375
	0.012877	-0.012861	0.018667	-0.007558	0.014523	-0.000888
	-0.006698	-0.029535	-0.008810	0.011802	0.005918	0.032302
	0.003481					
z347	0.021089	-0.042348	-0.002177	-0.034205	-0.016204	0.011082
	0.005730	0.028492	0.012320	-0.009562	0.030137	0.028824
	0.010440	0.031855	-0.006673	-0.008687	0.012346	0.015004
	0.023632	-0.026758	-0.007432	-0.033637	0.021658	0.049110
	0.059833	-0.027767	0.005390	0.015459	0.005764	-0.004627
	-0.020087					
x348	-0.008914	-0.029544	-0.014691	0.009844	0.017122	0.007571
	-0.026482	0.025851	-0.009112	-0.025133	-0.004159	-0.015247
	0.012674	0.003860	-0.006630	-0.010687	0.015096	-0.030402
	-0.008049	0.024689	-0.009215	-0.001440	0.003058	0.018310
	0.000078	-0.008076	-0.006708	-0.011989	-0.016340	-0.017663
	-0.002515					
y348	0.003199	0.017326	-0.019650	0.007597	-0.019471	0.037517
	0.020317	-0.027086	-0.015524	0.001508	-0.000642	-0.009554
	0.000140	-0.012400	0.018096	-0.021346	-0.009251	0.020116
	0.005082	-0.017378	0.017605	-0.010373	0.025812	0.002748
	-0.003570	-0.026221	-0.014831	0.009720	0.003345	0.025233
	0.007714					
z348	0.016233	-0.036742	-0.002288	-0.032392	-0.020260	0.012374
	0.002103	0.024346	0.015083	-0.015191	0.029541	0.021914
	0.013478	0.029965	-0.005008	-0.007470	0.014053	0.013820
	0.026956	-0.028788	-0.013682	-0.026979	0.030089	0.044924
	0.055342	-0.024624	0.002858	0.015657	-0.004286	-0.009133
	-0.028094					
x349	-0.010128	-0.031661	-0.016729	0.006739	0.016686	0.004684
	-0.029502	0.028404	-0.004810	-0.022266	-0.003668	-0.015878
	0.012316	-0.003060	-0.009621	-0.015020	0.014732	-0.031378
	-0.006753	0.021603	-0.013742	-0.000508	-0.000135	0.019272
	0.012276	-0.013712	-0.003951	-0.012055	-0.019226	-0.008978
	0.013140					
y349	-0.002476	0.012656	-0.025075	0.006220	-0.021823	0.039910
	0.015287	-0.024309	-0.018488	-0.006486	-0.000006	-0.011391
	-0.003080	-0.007956	0.017240	-0.019192	0.010730	0.006929
	-0.006866	-0.012589	0.008516	-0.008487	0.032827	0.007228
	-0.013245	-0.022627	-0.023946	0.008088	0.003879	0.014725
	0.003764					

z349	0.011400	-0.030748	-0.002809	-0.029255	-0.023895	0.013596
	-0.001320	0.018677	0.018291	-0.020978	0.028869	0.012551
	0.015514	0.028190	-0.002880	-0.005625	0.014809	0.012113
	0.031667	-0.029403	-0.016418	-0.016974	0.036458	0.040180
	0.048689	-0.024180	-0.001728	0.015891	-0.012068	-0.012107
	-0.037878					
x350	-0.009733	-0.031666	-0.016114	0.003298	0.018763	0.000844
	-0.035279	0.034226	-0.001737	-0.017770	-0.002438	-0.012695
	0.012795	-0.004434	-0.012394	-0.013041	0.019885	-0.024613
	-0.007192	0.022533	-0.029274	0.001452	-0.002551	0.012692
	0.013376	-0.010634	-0.018114	-0.015233	-0.016711	-0.004724
	0.007979					
y350	-0.009237	0.007900	-0.032853	0.004897	-0.026179	0.042554
	0.011301	-0.020920	-0.019248	-0.015200	0.000182	-0.015317
	-0.006049	-0.002165	0.018358	-0.020717	0.024472	-0.012410
	-0.016512	-0.011372	0.002898	-0.002627	0.038565	0.010085
	-0.016356	-0.022459	-0.025856	0.008901	0.001711	0.013497
	0.013944					
z350	0.006687	-0.024882	-0.003649	-0.024223	-0.027557	0.013988
	-0.005010	0.012516	0.021031	-0.025521	0.026503	-0.001706
	0.019081	0.025540	-0.000153	-0.002695	0.016930	0.008857
	0.037928	-0.027068	-0.022033	-0.004622	0.044168	0.032938
	0.037161	-0.024180	-0.012894	0.012365	-0.025668	-0.017757
	-0.046153					
x351	-0.004999	-0.029707	-0.013536	0.001304	0.024031	-0.001187
	-0.039578	0.034558	0.001953	-0.014236	0.000169	-0.012378
	0.012396	-0.010174	-0.013171	-0.011274	0.009111	-0.012802
	0.000159	0.017781	-0.028666	0.004855	-0.011871	0.007885
	0.028434	-0.006241	-0.007491	-0.013686	-0.014652	0.006026
	-0.007358					
y351	-0.016490	0.003592	-0.040371	0.002992	-0.028750	0.043589
	0.007833	-0.012308	-0.020244	-0.020200	-0.000493	-0.019219
	-0.006993	0.008369	0.020197	-0.022386	0.034098	-0.031051
	-0.023256	-0.009331	-0.006997	0.007133	0.041924	0.005767
	-0.020962	-0.017958	-0.030889	0.013566	-0.001747	0.018145
	0.026131					
z351	0.002173	-0.018598	-0.004663	-0.017658	-0.031240	0.015201
	-0.007867	0.005216	0.023513	-0.030900	0.023538	-0.018492
	0.023011	0.020204	0.001304	0.004098	0.014820	0.002542
	0.045246	-0.022839	-0.023847	0.010977	0.050534	0.023415
	0.024077	-0.027526	-0.022343	0.008221	-0.041091	-0.022967
	-0.047958					

x352	-0.000804	-0.027242	-0.010140	0.001040	0.028839	-0.004268
	-0.041578	0.035094	0.002857	-0.009820	0.003415	-0.012082
	0.012812	-0.013016	-0.017874	-0.003144	0.002011	0.002350
	0.005033	0.020419	-0.030687	-0.003476	-0.017136	0.003528
	0.027125	0.000807	-0.004567	-0.008329	-0.013405	0.005071
	-0.005338					
y352	-0.017244	-0.000315	-0.036440	0.001633	-0.019485	0.039682
	0.004140	-0.005308	-0.020850	-0.014914	0.002510	-0.020849
	-0.010137	0.007187	0.008868	-0.023244	0.028246	-0.029982
	-0.022402	-0.007887	-0.010222	0.004802	0.033598	0.001620
	-0.012023	-0.006219	-0.022215	0.016516	-0.003347	0.006578
	0.024647					
z352	0.003327	-0.017091	-0.003207	-0.014474	-0.034406	0.016953
	-0.005756	0.010256	0.026677	-0.032810	0.007866	-0.011081
	0.026754	0.011689	-0.000059	0.005257	0.016362	-0.000694
	0.031022	-0.019672	-0.037394	0.019997	0.047608	0.016873
	0.004049	-0.021030	-0.039145	-0.010177	-0.046406	-0.016731
	-0.058006					
x353	0.004138	-0.023666	-0.008521	0.002057	0.028673	-0.005386
	-0.041914	0.032347	0.004343	-0.009780	0.004743	-0.009599
	0.007960	-0.018016	-0.017928	0.003996	-0.006781	0.007260
	0.004037	0.026036	-0.025285	-0.003083	-0.021165	-0.001347
	0.029124	0.003251	-0.003111	-0.012746	-0.008414	0.005632
	-0.005554					
y353	-0.017096	-0.004120	-0.031509	0.000264	-0.009290	0.035243
	0.000954	0.003576	-0.020346	-0.007287	0.004468	-0.022564
	-0.012543	0.006063	-0.002809	-0.022741	0.019528	-0.026071
	-0.020089	-0.006109	-0.014495	0.004491	0.023343	-0.003010
	-0.002866	0.006666	-0.012508	0.020317	-0.005254	-0.001953
	0.018285					
z353	0.004470	-0.015237	-0.002600	-0.010929	-0.036703	0.018559
	-0.003175	0.014602	0.029397	-0.034201	-0.006208	-0.005032
	0.029044	0.002972	-0.001997	0.008761	0.015994	-0.002020
	0.019023	-0.017550	-0.046859	0.026757	0.045952	0.010816
	-0.012580	-0.015484	-0.049571	-0.019803	-0.047836	-0.011883
	-0.061087					
x354	0.007571	-0.020483	-0.006512	0.004005	0.027160	-0.004296
	-0.039735	0.028357	0.004589	-0.008452	0.004463	-0.005485
	0.003526	-0.020950	-0.018024	0.007081	-0.010128	0.010637
	0.001070	0.025785	-0.025274	0.000634	-0.021320	-0.006518
	0.028856	-0.001024	-0.008656	-0.013338	-0.001693	0.003874
	-0.009796					

y354	-0.016847	-0.007281	-0.027222	-0.000782	-0.000030	0.031580
	-0.000835	0.012742	-0.020125	0.000368	0.004847	-0.023886
	-0.013638	0.005983	-0.011876	-0.021786	0.011879	-0.022545
	-0.018726	-0.005168	-0.020100	0.007326	0.014182	-0.009517
	0.004908	0.017673	-0.005199	0.024250	-0.009190	-0.006104
	0.011327					
z354	0.005484	-0.013286	-0.002907	-0.007125	-0.037050	0.018961
	-0.000960	0.018080	0.030327	-0.035913	-0.017521	-0.001479
	0.030770	-0.004996	-0.004651	0.012872	0.015770	-0.002118
	0.009889	-0.015578	-0.052346	0.034648	0.044472	0.004736
	-0.024465	-0.011790	-0.054360	-0.024863	-0.048100	-0.008683
	-0.060081					
x355	0.010658	-0.017019	-0.007666	0.005201	0.024411	-0.003122
	-0.040085	0.024590	0.004491	-0.012071	0.004656	-0.003059
	-0.000945	-0.028075	-0.015170	0.011445	-0.013887	0.014843
	0.002898	0.026937	-0.025275	-0.003364	-0.022094	-0.006100
	0.022205	-0.007400	-0.003442	-0.012597	0.008112	0.009505
	-0.003782					
y355	-0.016716	-0.009736	-0.024856	-0.001988	0.007920	0.028811
	-0.003005	0.022137	-0.019753	0.005179	0.004565	-0.025823
	-0.013624	0.003576	-0.017652	-0.020107	0.004590	-0.018142
	-0.015050	-0.003786	-0.025531	0.009120	0.006621	-0.013472
	0.007106	0.025230	0.006419	0.026140	-0.012169	-0.002703
	0.009653					
z355	0.007013	-0.011144	-0.004790	-0.003094	-0.036326	0.018406
	0.001159	0.019660	0.029649	-0.038398	-0.025684	0.001199
	0.031773	-0.011289	-0.008206	0.016607	0.013838	-0.001766
	0.002926	-0.013680	-0.052016	0.039994	0.042587	-0.002332
	-0.031140	-0.006910	-0.053865	-0.026379	-0.048292	-0.007461
	-0.056626					
x356	0.013854	-0.013279	-0.009652	0.007003	0.020610	-0.002708
	-0.040202	0.016828	0.004278	-0.016180	0.007480	0.001008
	-0.005809	-0.033773	-0.009797	0.016978	-0.015572	0.019493
	0.003905	0.024935	-0.020990	-0.006754	-0.020634	-0.008836
	0.017931	-0.016889	-0.008832	-0.015176	0.013353	0.008968
	0.004347					
y356	-0.016522	-0.011679	-0.023678	-0.002352	0.014630	0.027035
	-0.004432	0.028919	-0.018489	0.009162	0.004775	-0.026066
	-0.012048	0.000740	-0.021112	-0.018061	-0.001589	-0.011711
	-0.011191	-0.004362	-0.029254	0.012594	0.002185	-0.018120
	0.007105	0.025406	0.012193	0.024562	-0.015641	0.000449
	0.010103					

z356	0.008789	-0.009106	-0.007359	0.001415	-0.034630	0.016838
	0.002945	0.020201	0.027324	-0.040731	-0.031209	0.002071
	0.031541	-0.016658	-0.012327	0.019713	0.011262	-0.000794
	-0.001983	-0.011842	-0.047513	0.044526	0.040859	-0.009167
	-0.035131	-0.003755	-0.047144	-0.022736	-0.047333	-0.007415
	-0.051075					
x357	0.016847	-0.009614	-0.011090	0.007386	0.015639	-0.003305
	-0.040719	0.009217	0.004146	-0.021447	0.011437	0.001455
	-0.010804	-0.038752	-0.001534	0.022736	-0.014015	0.021258
	0.006045	0.023326	-0.012666	-0.011372	-0.021969	-0.011570
	0.012525	-0.023383	-0.017082	-0.020260	0.012015	0.011060
	0.011089					
y357	-0.016467	-0.013239	-0.022528	-0.003279	0.020402	0.026195
	-0.006233	0.035683	-0.016016	0.011901	0.004783	-0.028525
	-0.008965	-0.003478	-0.022246	-0.017099	-0.005550	-0.004376
	-0.005222	-0.005272	-0.030084	0.015087	-0.003355	-0.021146
	0.000347	0.024540	0.018233	0.018189	-0.023316	0.012066
	0.011924					
z357	0.010812	-0.007231	-0.010484	0.006362	-0.032009	0.014413
	0.004811	0.019026	0.024036	-0.043342	-0.033895	0.001455
	0.030200	-0.021486	-0.016740	0.022344	0.008038	0.000384
	-0.004690	-0.011218	-0.039061	0.047434	0.041202	-0.014338
	-0.036953	-0.000981	-0.036596	-0.015771	-0.044770	-0.008358
	-0.044979					
x358	0.020134	-0.005337	-0.013294	0.007535	0.009845	-0.004902
	-0.040374	0.000092	0.001756	-0.029307	0.017997	0.000193
	-0.017713	-0.041104	0.008219	0.030970	-0.012179	0.019504
	0.004607	0.020754	-0.006430	-0.019228	-0.023532	-0.013440
	0.007123	-0.026561	-0.026459	-0.022449	0.015338	0.004977
	0.011927					
y358	-0.016409	-0.013927	-0.023086	-0.003095	0.025273	0.026695
	-0.006124	0.040149	-0.014315	0.012159	0.004627	-0.031563
	-0.005800	-0.006042	-0.023241	-0.015125	-0.010536	0.002151
	-0.002074	-0.006209	-0.034286	0.012966	-0.007999	-0.021417
	-0.008719	0.020581	0.025881	0.014398	-0.020797	0.015035
	0.006891					
z358	0.012986	-0.005896	-0.014473	0.011925	-0.029125	0.011340
	0.006302	0.016222	0.020171	-0.045095	-0.033871	-0.001441
	0.028446	-0.026232	-0.022303	0.023603	0.003228	0.002551
	-0.005567	-0.009874	-0.025631	0.049912	0.042048	-0.017125
	-0.036779	0.000213	-0.021552	-0.005412	-0.040414	-0.009566
	-0.038491					

x359	0.023078	-0.000999	-0.014391	0.007093	0.002351	-0.008459
	-0.042283	-0.009779	-0.002978	-0.037735	0.027103	-0.002145
	-0.027061	-0.042628	0.020563	0.043256	-0.008762	0.013511
	0.002201	0.017092	0.002008	-0.027703	-0.022107	-0.017776
	0.006539	-0.026047	-0.044366	-0.022449	0.015973	-0.000935
	0.014585					
y359	-0.017533	-0.014485	-0.023905	-0.001117	0.028982	0.028214
	-0.008267	0.042873	-0.013456	0.012869	0.005039	-0.034553
	-0.003931	-0.009969	-0.021730	-0.008500	-0.015598	0.007783
	0.000632	-0.008574	-0.037624	0.007591	-0.009198	-0.024272
	-0.015705	0.014527	0.023980	0.012016	-0.018860	0.020857
	0.006820					
z359	0.015129	-0.005154	-0.019481	0.017904	-0.025283	0.007592
	0.008129	0.012261	0.015130	-0.046755	-0.030551	-0.007092
	0.026238	-0.030102	-0.027524	0.024120	-0.002459	0.005457
	-0.003530	-0.008733	-0.007498	0.051576	0.042505	-0.017291
	-0.032741	0.000698	-0.001456	0.010334	-0.032956	-0.010957
	-0.030288					
x360	0.026895	0.004675	-0.012809	0.004201	-0.006248	-0.014967
	-0.044341	-0.020292	-0.008336	-0.047749	0.035817	-0.005011
	-0.035810	-0.041920	0.031615	0.053598	-0.003013	0.004666
	0.000814	0.013606	0.012533	-0.033905	-0.017748	-0.022741
	0.010672	-0.024351	-0.059317	-0.019028	0.014662	-0.008062
	0.017611					
y360	-0.018976	-0.013745	-0.027366	0.000561	0.031174	0.029449
	-0.008914	0.043039	-0.010887	0.008034	0.006563	-0.041526
	-0.004577	-0.016236	-0.015472	0.003689	-0.021360	0.008200
	0.002306	-0.010589	-0.039414	-0.007069	-0.015514	-0.026345
	-0.014939	0.000523	0.020756	0.014289	-0.012610	0.023862
	0.014810					
z360	0.017102	-0.005008	-0.025843	0.024050	-0.020376	0.003376
	0.009812	0.008439	0.008866	-0.047763	-0.024143	-0.014008
	0.022832	-0.032756	-0.030880	0.025402	-0.008503	0.009803
	0.000099	-0.008518	0.013856	0.053174	0.041774	-0.016371
	-0.025467	0.000758	0.020491	0.031434	-0.025959	-0.015677
	-0.018665					
x361	0.032701	0.010283	-0.001692	-0.002976	-0.010139	-0.021179
	-0.039954	-0.032256	-0.010885	-0.048485	0.029757	0.005455
	-0.036631	-0.029462	0.023826	0.037745	0.010286	0.010844
	0.008371	0.016665	0.031193	-0.019477	-0.001755	-0.030713
	0.010888	-0.019904	-0.054474	-0.011293	0.008957	-0.005459
	0.013399					

y361	-0.017963	-0.012900	-0.029354	0.001568	0.030717	0.028549
	-0.012098	0.042327	-0.011371	0.000530	0.008907	-0.044145
	-0.013172	-0.019642	-0.005706	0.019302	-0.024159	0.012075
	0.001395	-0.013457	-0.033033	-0.022518	-0.025395	-0.022794
	-0.022077	-0.007814	0.001901	-0.002769	-0.021489	0.022607
	0.016665					
z361	0.024549	-0.003725	-0.040684	0.028480	-0.013309	0.003984
	0.015932	0.009634	0.001746	-0.052877	-0.026518	-0.006940
	0.015707	-0.032162	-0.034164	0.016479	-0.007524	0.023613
	-0.002534	-0.012854	0.038464	0.037441	0.036883	-0.024628
	-0.007457	-0.019310	0.000489	0.027367	-0.021778	0.020947
	-0.017041					
x362	0.037424	0.015320	0.011473	-0.012056	-0.014261	-0.029529
	-0.035885	-0.041326	-0.012923	-0.046145	0.023593	0.018618
	-0.036425	-0.012798	0.016931	0.017344	0.024699	0.013342
	0.015773	0.019104	0.048149	-0.002864	0.009635	-0.035424
	0.015892	-0.012290	-0.045073	-0.001398	0.002639	0.003531
	0.014580					
y362	-0.014816	-0.012208	-0.026380	0.001020	0.027758	0.023483
	-0.016543	0.036539	-0.011231	-0.008000	0.008738	-0.043640
	-0.018497	-0.020089	0.003949	0.032877	-0.023480	0.016122
	-0.000737	-0.011002	-0.024358	-0.031452	-0.029619	-0.022056
	-0.011500	-0.013910	0.003452	-0.008301	-0.032252	0.020121
	0.024796					
z362	0.030948	-0.004706	-0.055392	0.034514	-0.006280	0.002663
	0.023381	0.011562	-0.004371	-0.054633	-0.026734	-0.001693
	0.009017	-0.032505	-0.031569	0.013082	-0.003420	0.036731
	-0.008058	-0.006470	0.058657	0.012366	0.031361	-0.027736
	0.001494	-0.033285	-0.016859	0.021394	-0.008621	0.044769
	-0.013874					
x363	0.042018	0.018452	0.020978	-0.018772	-0.020029	-0.038631
	-0.033879	-0.047776	-0.015075	-0.043502	0.022648	0.030240
	-0.035564	0.002843	0.014714	0.001992	0.032912	0.011913
	0.019535	0.025187	0.059982	0.002599	0.016993	-0.034138
	0.023948	-0.005325	-0.037559	0.008669	-0.002670	0.014898
	0.014802					
y363	-0.012552	-0.008633	-0.016847	-0.007056	0.024987	0.018031
	-0.016487	0.031230	-0.013875	-0.014239	0.003617	-0.041902
	-0.027187	-0.015554	0.006207	0.038595	-0.013273	0.015092
	0.002221	-0.016172	-0.018044	-0.019482	-0.038411	-0.029079
	-0.009262	-0.017165	0.007178	-0.004396	-0.037527	0.018869
	0.037092					

z363	0.032576	-0.006368	-0.063552	0.040007	0.002790	0.005596
	0.033793	0.014229	-0.008863	-0.051461	-0.029996	0.003729
	-0.003725	-0.041048	-0.026278	0.009475	0.010678	0.045265
	-0.011492	-0.014710	0.063940	0.012961	0.023990	-0.037613
	0.006262	-0.043668	-0.023106	0.012581	-0.007784	0.052823
	-0.001720					
x364	0.043866	0.021101	0.028656	-0.023478	-0.023213	-0.042473
	-0.030048	-0.051612	-0.017595	-0.039160	0.023321	0.040871
	-0.040144	0.010131	0.012196	-0.009629	0.040463	0.006948
	0.024849	0.018155	0.056577	0.020745	0.015594	-0.036970
	0.030221	0.000816	-0.029024	0.019759	-0.007997	0.022548
	0.009838					
y364	-0.008829	-0.004414	-0.005430	-0.015118	0.020656	0.009056
	-0.018965	0.025367	-0.016346	-0.020625	-0.001283	-0.039867
	-0.030696	-0.008627	0.011041	0.039881	-0.001803	0.013066
	0.003174	-0.017674	-0.007171	-0.008229	-0.037383	-0.028720
	-0.002224	-0.022575	0.011618	-0.008392	-0.051982	0.017830
	0.053018					
z364	0.031170	-0.008228	-0.067082	0.044435	0.011122	0.010254
	0.045800	0.017524	-0.013104	-0.046354	-0.033566	0.008294
	-0.016962	-0.052451	-0.021064	0.006847	0.028989	0.048645
	-0.013274	-0.031833	0.061008	0.028677	0.016687	-0.047923
	0.006451	-0.050955	-0.022449	0.006328	-0.015865	0.050360
	0.007875					
x365	0.045161	0.022442	0.032963	-0.025337	-0.028111	-0.045376
	-0.023764	-0.054310	-0.022416	-0.031110	0.027107	0.050000
	-0.045294	0.015388	0.011580	-0.017015	0.046313	-0.000341
	0.028041	0.012974	0.051068	0.033356	0.018769	-0.040158
	0.035067	0.006612	-0.023839	0.032916	-0.016079	0.026379
	0.009826					
y365	-0.001406	0.000915	0.007361	-0.024384	0.014580	-0.004964
	-0.024786	0.018034	-0.019089	-0.029811	-0.002634	-0.037771
	-0.030793	0.002980	0.015719	0.043609	0.002819	0.010954
	0.003202	-0.009660	0.003589	-0.007427	-0.039837	-0.016990
	0.001074	-0.024562	0.017551	-0.002792	-0.050904	0.026205
	0.052572					
z365	0.033083	-0.013373	-0.076871	0.051181	0.014233	0.009577
	0.056628	0.021295	-0.019399	-0.041300	-0.026296	0.009108
	-0.019102	-0.054366	-0.015763	0.016461	0.033027	0.051037
	-0.018751	-0.024445	0.058759	0.007771	0.006611	-0.039183
	-0.004635	-0.047639	-0.026895	0.023148	0.001869	0.056988
	-0.009248					

x366	0.046699	0.020736	0.027016	-0.018394	-0.031965	-0.043362
	-0.014626	-0.059779	-0.027859	-0.019939	0.041581	0.053595
	-0.053528	0.014130	0.007941	-0.011116	0.039294	-0.007336
	0.025918	0.008169	0.025516	0.028184	0.014434	-0.025636
	0.042711	0.009340	-0.010346	0.046107	-0.021598	0.034348
	-0.010713					
y366	0.007028	0.008755	0.025608	-0.035692	0.009066	-0.020525
	-0.033255	0.012680	-0.024170	-0.037262	-0.003966	-0.030909
	-0.030794	0.017454	0.021870	0.047619	0.008167	0.010322
	0.005872	-0.003398	0.004170	0.001100	-0.040992	-0.006593
	-0.001162	-0.021263	0.018831	0.005771	-0.045729	0.027592
	0.048120					
z366	0.031892	-0.016811	-0.082465	0.060165	0.019088	0.013783
	0.069395	0.024911	-0.024603	-0.032505	-0.018199	0.015120
	-0.025900	-0.061440	-0.010281	0.025416	0.040710	0.047455
	-0.020341	-0.032870	0.030081	0.012521	-0.015719	-0.026924
	-0.014720	-0.041937	-0.017134	0.034276	0.012454	0.054690
	-0.029108					
x367	0.045199	0.015682	0.012096	-0.000100	-0.030086	-0.030274
	0.001482	-0.064725	-0.034006	0.002281	0.068230	0.052533
	-0.066778	0.006043	0.002596	0.004372	0.024817	-0.020427
	0.017802	0.004819	-0.023638	0.011295	0.000773	0.001408
	0.036598	0.016586	0.003409	0.035268	-0.031131	0.036479
	-0.027815					
y367	0.018425	0.019675	0.049191	-0.051092	0.000470	-0.041763
	-0.043914	0.006216	-0.032689	-0.048315	-0.005581	-0.022414
	-0.035233	0.032136	0.027709	0.056516	0.012350	0.011304
	0.009143	0.000869	-0.003497	0.011168	-0.034429	0.002133
	-0.001659	-0.019393	0.022356	0.019667	-0.039294	0.027061
	0.039664					
z367	0.030793	-0.019943	-0.085824	0.068283	0.019372	0.014988
	0.081557	0.029036	-0.028596	-0.025058	-0.012311	0.022275
	-0.031774	-0.060770	0.002516	0.034647	0.050115	0.038930
	-0.026088	-0.023052	0.003987	0.006253	-0.039604	-0.004810
	-0.023257	-0.033971	-0.002266	0.043248	0.022386	0.040020
	-0.042787					
x368	0.036703	0.005017	-0.013633	0.031175	-0.025528	-0.004938
	0.031274	-0.073473	-0.037552	0.038166	0.102598	0.041851
	-0.087020	-0.011548	-0.002597	0.022666	0.008269	-0.046913
	-0.002796	0.005010	-0.075569	-0.011239	-0.017251	0.025441
	0.014184	0.021668	0.009053	0.011005	-0.045768	0.014592
	-0.047411					

y368	0.029705	0.031944	0.076745	-0.068841	-0.011607	-0.065744
	-0.051736	0.001287	-0.045758	-0.060470	-0.006993	-0.012849
	-0.043780	0.044283	0.030809	0.072381	0.015725	0.005052
	0.010680	0.002686	-0.013252	0.028125	-0.007215	-0.008514
	-0.004710	-0.011418	0.025961	0.037739	-0.035406	0.017732
	0.017905					
z368	0.028864	-0.019542	-0.077445	0.067859	0.011359	0.008793
	0.087167	0.032927	-0.029708	-0.025235	-0.019589	0.025586
	-0.036523	-0.046241	0.027794	0.036404	0.060623	0.021791
	-0.034133	0.014630	0.003013	-0.004903	-0.049347	0.011873
	-0.026179	-0.025900	0.018782	0.035669	0.021504	0.006776
	-0.024454					
x369	0.025811	-0.005712	-0.035303	0.054787	-0.024496	0.013208
	0.049720	-0.082411	-0.037873	0.069436	0.125231	0.019542
	-0.093796	-0.020687	-0.004501	0.018625	-0.010489	-0.067791
	-0.028983	0.017751	-0.078660	-0.026200	-0.030662	0.016851
	-0.008546	0.020605	-0.002422	-0.001813	-0.063856	-0.014500
	-0.040534					
y369	0.032406	0.033890	0.084784	-0.071215	-0.019486	-0.071499
	-0.042082	-0.004858	-0.054802	-0.049941	-0.000487	-0.011296
	-0.057963	0.040488	0.036905	0.083916	0.014452	-0.002556
	0.005785	0.006146	-0.021657	0.026586	0.014587	-0.021007
	-0.018748	-0.001995	0.026238	0.035511	-0.031355	0.003462
	-0.010051					
z369	0.024944	-0.014895	-0.060540	0.059038	0.002192	-0.000562
	0.077181	0.036189	-0.027973	-0.037789	-0.034218	0.018890
	-0.029237	-0.028688	0.043717	0.034888	0.054590	-0.003494
	-0.031523	0.036762	0.013730	0.012659	-0.031912	-0.007334
	-0.022585	-0.015838	0.035131	0.042278	0.014442	-0.023623
	-0.018396					
x370	-0.020644	-0.021355	-0.012207	-0.005112	-0.020523	-0.028804
	-0.004549	0.011250	0.040384	-0.019050	0.004878	-0.010669
	-0.025532	0.008303	0.014499	-0.012180	-0.003086	-0.002587
	-0.041973	-0.034783	-0.069647	-0.026796	0.012743	-0.002453
	-0.010631	-0.004391	0.060037	0.019627	-0.003890	0.008392
	0.004939					
y370	-0.009521	-0.015086	0.024157	-0.004666	-0.020476	0.002240
	0.030119	0.003849	-0.027538	0.006031	0.033499	-0.017095
	0.038744	-0.014659	0.012025	-0.040222	0.046128	-0.009179
	0.040979	-0.005743	0.016459	0.037975	-0.021119	-0.012279
	0.062867	-0.025391	0.049670	-0.115179	-0.007172	-0.030735
	-0.032012					

z370	-0.002214	-0.011662	0.036262	-0.049525	0.019461	0.016268
	0.023911	0.006991	0.025448	0.036489	-0.002208	0.012466
	-0.118747	-0.044998	-0.033673	-0.020033	-0.101100	0.036095
	-0.061710	0.018156	0.114628	0.017100	0.026566	0.071682
	0.033798	-0.009032	-0.003201	-0.040229	-0.012470	-0.124912
	-0.005338					
x371	-0.018494	-0.027391	-0.011615	-0.009007	-0.021605	-0.042111
	-0.007655	0.012870	0.058019	-0.039366	0.015911	0.001128
	-0.053176	-0.016483	-0.000177	-0.013947	-0.031953	0.000090
	-0.070374	-0.037414	-0.076174	-0.028101	0.017241	0.017421
	0.000393	-0.010454	0.087876	0.047658	0.000163	-0.031524
	0.031315					
y371	-0.015938	-0.022821	0.031451	0.002841	-0.037680	0.013373
	0.028093	0.003001	-0.038117	0.016445	0.017173	-0.018073
	0.050031	0.008631	0.005743	-0.044760	0.035144	-0.024684
	0.038188	0.000951	0.033857	0.046305	-0.011694	-0.031183
	0.031404	-0.022040	0.005005	-0.087514	0.012640	-0.003234
	-0.013917					
z371	-0.005155	-0.010841	0.037145	-0.038699	0.010297	0.016440
	0.013008	0.010984	0.029983	0.032403	-0.004156	0.004701
	-0.094176	-0.020715	-0.025789	-0.026523	-0.084274	0.011128
	-0.058569	0.013653	0.078501	0.003601	0.023008	0.052328
	0.023133	-0.022829	-0.004046	-0.033037	-0.004251	-0.084740
	0.010008					
x372	-0.025032	-0.027223	-0.010477	-0.007915	-0.026639	-0.048958
	-0.006600	0.012373	0.063208	-0.050820	0.020861	0.006780
	-0.057547	-0.032090	-0.015344	-0.003067	-0.032426	0.002153
	-0.063902	-0.027352	-0.069265	-0.022649	0.002454	0.026724
	0.008336	-0.000109	0.101803	0.019348	0.005023	-0.022327
	0.029228					
y372	-0.018837	-0.028262	0.040072	0.005848	-0.045480	0.015606
	0.019835	0.000978	-0.041061	0.016985	0.003847	-0.021430
	0.048121	0.018221	0.003534	-0.041395	0.031232	-0.020369
	0.039932	-0.010812	0.027998	0.039795	-0.010118	-0.035195
	0.018239	-0.013607	-0.032200	-0.058434	0.016378	-0.002241
	-0.008142					
z372	-0.010134	-0.013517	0.040379	-0.027166	0.001289	0.012597
	0.009447	0.008380	0.032642	0.029684	-0.009064	0.000961
	-0.071002	-0.003060	-0.020329	-0.027580	-0.070156	-0.007711
	-0.051698	0.013538	0.049688	-0.002018	0.017302	0.039348
	0.008627	-0.024621	-0.006106	-0.021327	0.002893	-0.055352
	0.007161					

x373	-0.030655	-0.023959	-0.012732	-0.006204	-0.029742	-0.052561
	-0.004319	0.014053	0.064966	-0.058847	0.027477	0.009711
	-0.053674	-0.047631	-0.022238	0.005697	-0.029797	0.002395
	-0.055575	-0.014027	-0.050092	-0.016808	-0.005681	0.023350
	0.007245	0.000645	0.111096	-0.012518	-0.003956	-0.009772
	0.015183					
y373	-0.021752	-0.028585	0.042272	0.004271	-0.048617	0.015604
	0.016152	-0.005987	-0.036349	0.013356	-0.000982	-0.024157
	0.037145	0.016336	0.004724	-0.038524	0.021584	-0.018113
	0.032008	-0.017098	0.038899	0.029065	-0.006147	-0.031272
	0.006321	-0.017228	-0.033410	-0.046214	0.027128	0.002071
	0.002011					
z373	-0.015568	-0.015868	0.043269	-0.020895	-0.006733	0.009434
	0.008775	0.002540	0.036787	0.026293	-0.013838	0.000271
	-0.057591	0.006400	-0.014298	-0.030181	-0.062640	-0.021359
	-0.052553	0.017575	0.037112	-0.006764	0.017735	0.033231
	-0.008640	-0.028643	-0.005990	-0.011188	0.010703	-0.038338
	0.003617					
x374	-0.033764	-0.020299	-0.014860	-0.002827	-0.032196	-0.053436
	-0.003581	0.017895	0.059533	-0.063417	0.030281	0.011665
	-0.043893	-0.057647	-0.027017	0.012978	-0.025086	-0.000390
	-0.044327	-0.004375	-0.034353	-0.006829	-0.011590	0.014798
	0.006739	0.004292	0.101555	-0.027676	-0.013000	0.004698
	0.003282					
y374	-0.022017	-0.027618	0.039566	0.005546	-0.052860	0.013716
	0.012453	-0.013080	-0.034477	0.008480	-0.002644	-0.027665
	0.028415	0.010188	0.000685	-0.036886	0.022727	-0.018168
	0.025520	-0.016458	0.038946	0.025126	-0.009323	-0.025383
	-0.000107	-0.009658	-0.021330	-0.035079	0.022559	0.021801
	0.004383					
z374	-0.019020	-0.018148	0.044881	-0.015028	-0.017332	0.007650
	0.007108	-0.003309	0.036515	0.022855	-0.018611	-0.001071
	-0.047789	0.010420	-0.013266	-0.031937	-0.050150	-0.033557
	-0.051931	0.022957	0.019752	-0.007910	0.016881	0.031656
	-0.017196	-0.026319	-0.001858	0.001333	0.010185	-0.011918
	-0.001782					
x375	-0.035480	-0.016422	-0.015345	0.000701	-0.033088	-0.052932
	-0.002736	0.022974	0.051736	-0.064126	0.029368	0.011270
	-0.031573	-0.062177	-0.028454	0.017838	-0.022642	-0.004105
	-0.029903	0.000772	-0.017353	0.001393	-0.012489	0.002658
	0.007893	0.004902	0.080114	-0.033557	-0.019863	0.011762
	-0.010075					

y375	-0.021189	-0.024708	0.036000	0.006740	-0.053300	0.011556
	0.011144	-0.017475	-0.030692	0.005313	-0.004521	-0.029764
	0.024784	0.006281	-0.001245	-0.032724	0.020185	-0.014135
	0.022140	-0.016606	0.039650	0.022236	-0.017235	-0.023029
	-0.003884	0.000801	-0.017214	-0.032252	0.022332	0.024242
	0.009157					
z375	-0.022281	-0.019223	0.047195	-0.010922	-0.025516	0.007935
	0.006730	-0.008153	0.037527	0.019445	-0.023717	-0.001710
	-0.035493	0.015799	-0.011077	-0.030339	-0.041825	-0.040481
	-0.049394	0.025381	0.006684	-0.007624	0.012070	0.031203
	-0.022286	-0.018543	-0.007430	0.006308	0.021139	-0.000661
	0.001323					
x376	-0.036004	-0.012614	-0.015012	0.003734	-0.032560	-0.051247
	-0.002246	0.028113	0.042512	-0.061667	0.027845	0.009340
	-0.020782	-0.062444	-0.028159	0.020847	-0.020975	-0.009005
	-0.015793	0.003201	-0.000022	0.007902	-0.007601	-0.009765
	0.005218	0.004015	0.055020	-0.031359	-0.026329	0.012714
	-0.024973					
y376	-0.019532	-0.020442	0.030714	0.007892	-0.051209	0.010455
	0.009607	-0.018735	-0.028923	0.004198	-0.003814	-0.030410
	0.019974	0.001769	-0.001944	-0.029588	0.017363	-0.010545
	0.016645	-0.018188	0.043777	0.022586	-0.020988	-0.021202
	-0.010558	0.008454	-0.019173	-0.030133	0.011757	0.024601
	0.001395					
z376	-0.025670	-0.019677	0.048842	-0.008430	-0.031917	0.011424
	0.005067	-0.010807	0.036542	0.017297	-0.025620	-0.002406
	-0.026993	0.017227	-0.007922	-0.029417	-0.033929	-0.046482
	-0.050424	0.023523	0.001178	-0.004770	0.012279	0.030495
	-0.032751	-0.014676	-0.018297	0.010656	0.021599	0.009916
	-0.006501					
x377	-0.034917	-0.008214	-0.014849	0.007144	-0.030541	-0.049944
	-0.001664	0.031350	0.033655	-0.056606	0.025601	0.006741
	-0.011267	-0.059451	-0.026285	0.022688	-0.017182	-0.016287
	-0.000964	0.004893	0.013203	0.014800	-0.001516	-0.022162
	0.000814	0.007176	0.029892	-0.022226	-0.028895	0.012369
	-0.037089					
y377	-0.016150	-0.015579	0.024866	0.010619	-0.048559	0.008489
	0.009388	-0.018710	-0.026824	0.003490	-0.004117	-0.030028
	0.015428	0.001716	-0.002888	-0.029724	0.016576	-0.005582
	0.015772	-0.020094	0.045978	0.027660	-0.024675	-0.024976
	-0.012983	0.016552	-0.019795	-0.026205	0.000632	0.024400
	0.001430					

z377	-0.027233	-0.020885	0.051205	-0.004556	-0.040068	0.014381
	0.004519	-0.013426	0.037130	0.014439	-0.030790	-0.004847
	-0.020347	0.023227	-0.005664	-0.033285	-0.024643	-0.047179
	-0.042603	0.018533	-0.005323	0.002861	0.013034	0.019807
	-0.037596	-0.009616	-0.020732	0.017872	0.020903	0.024820
	0.006325					
x378	-0.032163	-0.002313	-0.015534	0.012301	-0.026798	-0.049448
	-0.000636	0.033723	0.026101	-0.048017	0.027259	0.004716
	0.000382	-0.054415	-0.024859	0.028327	-0.011123	-0.026471
	0.013296	0.003881	0.022281	0.018174	0.002059	-0.025424
	-0.003447	0.013533	0.014997	-0.013206	-0.024162	0.007328
	-0.047370					
y378	-0.011731	-0.008761	0.017942	0.015160	-0.044075	0.005968
	0.009153	-0.016528	-0.026872	0.004205	-0.000458	-0.027001
	0.013457	0.002803	-0.005683	-0.026808	0.017128	-0.001661
	0.014137	-0.022284	0.046571	0.030818	-0.028758	-0.024720
	-0.012571	0.024528	-0.021700	-0.025149	-0.008648	0.022784
	-0.006896					
z378	-0.028548	-0.019979	0.053175	0.002442	-0.047631	0.016522
	0.002089	-0.014982	0.035212	0.015350	-0.029704	-0.004000
	-0.011037	0.027113	-0.007291	-0.031897	-0.010861	-0.050154
	-0.037828	0.009434	-0.010799	0.006085	0.014148	0.014082
	-0.039541	-0.000705	-0.023267	0.017298	0.023791	0.048285
	0.008838					
x379	-0.027963	0.004736	-0.017353	0.015253	-0.020151	-0.048983
	0.000185	0.034169	0.030562	-0.038851	0.030623	-0.000332
	0.003027	-0.052168	-0.027821	0.032219	-0.010299	-0.039993
	0.023508	0.000112	0.035680	0.009765	0.008977	-0.014881
	0.005315	-0.003323	0.014900	0.003528	-0.001137	0.004480
	-0.033635					
y379	-0.006174	-0.000436	0.009706	0.019626	-0.037308	0.002800
	0.008418	-0.012827	-0.025281	0.004172	0.004784	-0.022622
	0.010039	0.002934	-0.008460	-0.024635	0.015707	0.003412
	0.013739	-0.022887	0.048074	0.031188	-0.030702	-0.019602
	-0.004923	0.026138	-0.024820	-0.019958	-0.011985	0.012899
	-0.011914					
z379	-0.027729	-0.017584	0.052953	0.009109	-0.053478	0.017628
	-0.003709	-0.016242	0.031911	0.012718	-0.030716	-0.003438
	-0.004944	0.023780	-0.004203	-0.031273	0.005831	-0.045731
	-0.030441	0.000406	-0.012198	0.015698	0.015405	0.012675
	-0.040701	0.012576	-0.029783	0.015597	0.017410	0.040184
	0.002435					

x380	-0.024049	0.010332	-0.020516	0.015132	-0.017068	-0.049976
	-0.001303	0.030060	0.034864	-0.034099	0.028190	-0.002823
	0.000237	-0.051484	-0.027687	0.037225	-0.011344	-0.044619
	0.030292	-0.003911	0.038007	-0.003370	0.008902	-0.009553
	0.011605	-0.015964	0.008764	0.009940	0.011423	0.006931
	-0.024827					
y380	-0.002046	0.005795	0.003481	0.022933	-0.032243	-0.000006
	0.007780	-0.010723	-0.024341	0.003580	0.007408	-0.018767
	0.006777	0.002804	-0.009525	-0.022056	0.012306	0.006968
	0.014020	-0.021634	0.047361	0.028950	-0.032150	-0.016816
	0.001463	0.026713	-0.027083	-0.018717	-0.013017	0.008789
	-0.015660					
z380	-0.025439	-0.015456	0.050883	0.012135	-0.055045	0.017300
	-0.007097	-0.015875	0.035081	0.009576	-0.031870	-0.009188
	-0.001614	0.015668	-0.005742	-0.026778	0.010563	-0.047699
	-0.035505	0.001906	-0.006923	0.011595	0.023839	0.023439
	-0.034844	0.010188	-0.018327	0.006611	0.013651	0.045142
	0.000967					
x381	-0.019612	0.015202	-0.023391	0.016394	-0.015780	-0.048727
	0.001326	0.025063	0.028414	-0.027380	0.022536	-0.003462
	0.005681	-0.045725	-0.019704	0.042206	0.001078	-0.040251
	0.039346	-0.012872	0.033829	-0.001682	0.006689	-0.014921
	0.004186	-0.000959	-0.002185	0.000001	0.013101	-0.003052
	-0.033517					
y381	0.002341	0.011647	-0.002540	0.026585	-0.026964	-0.002326
	0.008252	-0.008084	-0.027327	0.002930	0.008540	-0.013886
	0.005045	0.004585	-0.008434	-0.017846	0.012135	0.011316
	0.014777	-0.019203	0.046023	0.028757	-0.034191	-0.016360
	0.002237	0.032112	-0.029858	-0.019776	-0.014621	0.001220
	-0.023012					
z381	-0.024734	-0.012180	0.049662	0.016437	-0.055363	0.019235
	-0.008341	-0.015836	0.039561	0.012559	-0.029446	-0.012857
	0.000579	0.010227	-0.009689	-0.021444	0.022884	-0.047773
	-0.036051	-0.001667	-0.002790	0.004335	0.039666	0.024658
	-0.039563	0.003978	0.003552	0.022686	0.025775	0.055536
	0.015310					
x382	-0.013216	0.018097	-0.026255	0.017482	-0.014134	-0.047437
	0.005429	0.018324	0.022327	-0.023523	0.017384	-0.006112
	0.008962	-0.040411	-0.012855	0.041394	0.011709	-0.038508
	0.047179	-0.020125	0.031683	0.005225	-0.000642	-0.017285
	0.000480	0.014559	-0.012981	-0.020553	0.013863	-0.019599
	-0.046198					

y382	0.006977	0.016794	-0.008499	0.029879	-0.021554	-0.004478
	0.009232	-0.005537	-0.030073	0.002285	0.008953	-0.010088
	0.002783	0.006348	-0.007711	-0.014418	0.012416	0.014545
	0.015315	-0.015274	0.044784	0.027229	-0.036353	-0.014823
	0.003781	0.039240	-0.030665	-0.023310	-0.013589	-0.005800
	-0.028980					
z382	-0.019381	-0.009120	0.046099	0.020466	-0.053248	0.016951
	-0.007626	-0.018507	0.044347	0.009912	-0.027084	-0.015358
	0.002518	0.009706	-0.011167	-0.022324	0.025744	-0.050158
	-0.035158	-0.005335	-0.000606	0.016115	0.035954	0.025970
	-0.033277	0.006379	0.004405	0.012604	0.021717	0.038093
	0.012019					
x383	-0.006435	0.019324	-0.027699	0.019015	-0.012180	-0.045063
	0.008863	0.011142	0.016897	-0.018947	0.013610	-0.009812
	0.012379	-0.035456	-0.008234	0.038156	0.021698	-0.036811
	0.053617	-0.026081	0.031115	0.007308	-0.001150	-0.016397
	-0.003858	0.026790	-0.019078	-0.038042	0.016172	-0.032380
	-0.056204					
y383	0.011605	0.020627	-0.013447	0.032886	-0.015012	-0.006116
	0.010090	-0.003193	-0.032093	0.001409	0.010148	-0.005557
	0.001782	0.009519	-0.007810	-0.013229	0.011104	0.017619
	0.016154	-0.009940	0.044146	0.026645	-0.036444	-0.009488
	0.002666	0.043901	-0.034114	-0.031516	-0.003613	-0.015818
	-0.036876					
z383	-0.014207	-0.004383	0.040080	0.027429	-0.051179	0.015964
	-0.010250	-0.020676	0.044679	0.010510	-0.025321	-0.019785
	0.003882	0.003494	-0.014520	-0.024053	0.031546	-0.051504
	-0.029744	-0.008501	-0.002121	0.013348	0.043645	0.025105
	-0.024973	0.010930	0.014343	0.009143	0.010571	0.029171
	0.007713					
x384	0.001078	0.018895	-0.026994	0.019692	-0.009033	-0.042218
	0.012246	0.003102	0.013224	-0.013850	0.012024	-0.014499
	0.014809	-0.029712	-0.006088	0.034680	0.028838	-0.036206
	0.056241	-0.029272	0.033580	0.004682	0.001144	-0.015638
	-0.008270	0.033510	-0.022928	-0.051338	0.024676	-0.039969
	-0.059775					
y384	0.016225	0.023520	-0.017543	0.035047	-0.008340	-0.007327
	0.010844	-0.001177	-0.033316	-0.000322	0.011792	-0.001857
	0.000415	0.012800	-0.009084	-0.012574	0.008813	0.019278
	0.017464	-0.002025	0.043894	0.027920	-0.034295	-0.002162
	-0.001102	0.049798	-0.038262	-0.042809	0.009893	-0.029413
	-0.045514					

z384	-0.008190	0.000639	0.034307	0.033666	-0.046929	0.013568
	-0.013227	-0.023820	0.043964	0.014918	-0.022506	-0.024973
	0.002755	-0.002697	-0.018363	-0.024880	0.038648	-0.054474
	-0.027874	-0.013874	-0.002034	0.001312	0.052358	0.018565
	-0.012911	0.008167	0.024288	0.011455	0.010967	0.029495
	0.008494					
x385	0.008595	0.017916	-0.023686	0.019624	-0.005187	-0.038363
	0.016492	-0.005122	0.013733	-0.009372	0.009292	-0.019170
	0.018275	-0.025519	-0.004099	0.035164	0.033256	-0.034847
	0.056312	-0.032718	0.034955	0.004654	0.004321	-0.014259
	-0.011801	0.035686	-0.023380	-0.057113	0.027894	-0.045142
	-0.053180					
y385	0.021980	0.025037	-0.021099	0.036361	-0.002486	-0.009152
	0.011223	0.000372	-0.033387	-0.002351	0.012119	-0.000890
	-0.001325	0.016025	-0.011800	-0.012667	0.004655	0.017904
	0.017402	0.007090	0.044054	0.026360	-0.029441	0.006357
	-0.005553	0.052598	-0.037764	-0.053536	0.021296	-0.035388
	-0.054795					
z385	-0.002956	0.008076	0.029531	0.037989	-0.040837	0.011469
	-0.012987	-0.026818	0.049880	0.019665	-0.026078	-0.029718
	0.003136	-0.011544	-0.017183	-0.014461	0.046075	-0.051513
	-0.027147	-0.023616	-0.010557	0.003490	0.057847	0.013000
	0.000660	0.008596	0.030470	0.022095	0.005084	0.018658
	0.026304					
x386	0.017006	0.015568	-0.020443	0.019196	-0.002072	-0.035983
	0.019935	-0.012889	0.013909	-0.005983	0.009367	-0.023631
	0.023997	-0.017308	-0.003387	0.035050	0.031082	-0.033489
	0.051909	-0.028405	0.040114	-0.004479	0.008593	-0.009891
	-0.017003	0.036998	-0.025924	-0.060857	0.028878	-0.040549
	-0.051029					
y386	0.027808	0.026158	-0.022403	0.036885	0.004328	-0.009798
	0.011567	0.001411	-0.031307	-0.004267	0.011110	0.000530
	-0.005013	0.018026	-0.016735	-0.011435	-0.000464	0.014680
	0.017229	0.014681	0.043958	0.027764	-0.022023	0.012715
	-0.011088	0.050861	-0.031397	-0.057720	0.029748	-0.039475
	-0.052297					
z386	0.004317	0.015334	0.019613	0.042383	-0.036846	0.004760
	-0.014333	-0.028813	0.048024	0.022364	-0.023939	-0.034552
	0.007371	-0.012555	-0.014589	-0.011621	0.049167	-0.049228
	-0.029314	-0.022260	-0.015408	-0.007137	0.060328	0.013495
	0.011995	0.016756	0.022645	0.017745	-0.003172	0.017140
	0.011499					

x387	0.024376	0.011882	-0.014089	0.019486	0.002714	-0.031441
	0.022885	-0.018042	0.015373	-0.002918	0.008190	-0.025591
	0.030169	-0.012074	-0.004699	0.038360	0.028482	-0.031805
	0.048050	-0.022370	0.043757	-0.013660	0.012361	-0.010199
	-0.023756	0.028591	-0.021714	-0.051610	0.025198	-0.028692
	-0.036785					
y387	0.034781	0.026675	-0.023193	0.036626	0.009494	-0.012042
	0.010948	0.001654	-0.027873	-0.003834	0.008144	-0.001822
	-0.007739	0.020989	-0.023510	-0.011871	-0.004223	0.009783
	0.013816	0.020828	0.042133	0.023564	-0.009014	0.021063
	-0.015704	0.046047	-0.023675	-0.058965	0.028158	-0.032299
	-0.051551					
z387	0.009725	0.022244	0.011428	0.046111	-0.028654	0.002515
	-0.015497	-0.027162	0.043258	0.022433	-0.021635	-0.030659
	0.008398	-0.017919	-0.011923	-0.003642	0.052194	-0.045432
	-0.024088	-0.016484	-0.018600	-0.010432	0.056929	0.008061
	0.015495	0.014795	0.023964	0.028341	-0.002969	0.014893
	0.017040					
x388	0.030026	0.007346	-0.004929	0.018409	0.004650	-0.025372
	0.025177	-0.022068	0.015854	-0.002392	0.005228	-0.028520
	0.034769	-0.018819	-0.004163	0.034684	0.033126	-0.025400
	0.037671	-0.021251	0.026071	-0.018671	0.016937	-0.019086
	-0.021469	0.011723	-0.009363	-0.037025	0.010645	-0.016668
	-0.012492					
y388	0.040048	0.028908	-0.021304	0.032456	0.012353	-0.010312
	0.010151	0.001990	-0.023031	-0.002192	-0.001922	-0.003176
	-0.011648	0.011746	-0.026047	-0.013454	0.006458	0.009076
	0.005272	0.016306	0.016750	0.031434	0.009478	0.017570
	-0.018392	0.032915	-0.003951	-0.040650	0.014130	-0.023017
	-0.023549					
z388	0.015728	0.026998	0.001968	0.047701	-0.019108	-0.001338
	-0.016263	-0.025288	0.036098	0.018642	-0.014874	-0.031414
	0.008937	-0.021889	-0.008516	-0.001084	0.045653	-0.043375
	-0.019161	-0.002889	-0.013743	-0.014970	0.044837	0.009710
	0.020682	0.012787	0.020396	0.018782	0.001077	0.009113
	0.004161					
x389	0.032949	-0.000451	0.011628	0.017283	0.007844	-0.015208
	0.027776	-0.025229	0.016439	-0.003469	0.009763	-0.020419
	0.046787	-0.023304	-0.004716	0.030578	0.033234	-0.014290
	0.026603	-0.020118	0.013138	-0.016869	0.012555	-0.021326
	-0.014052	0.013425	-0.002069	-0.021892	0.002281	-0.001574
	0.003153					

y389	0.045579	0.026155	-0.014658	0.024451	0.016756	-0.004312
	0.008347	0.001669	-0.021169	-0.003188	-0.000792	0.005401
	-0.010042	0.001758	-0.027691	-0.021570	0.013337	0.005054
	-0.001011	0.016467	-0.001409	0.049400	0.014767	0.025695
	-0.016874	0.043060	0.008073	-0.034721	-0.000859	-0.001364
	-0.018830					
z389	0.021205	0.032589	-0.005793	0.043652	-0.011325	-0.009693
	-0.014018	-0.022335	0.034751	0.017705	-0.009019	-0.028387
	0.014266	-0.020299	-0.004101	0.001375	0.040012	-0.035125
	-0.017254	0.010588	-0.011363	-0.015896	0.042526	0.011437
	0.024467	0.010105	0.020514	0.019795	0.007883	-0.001112
	0.009362					
x390	0.032127	-0.007736	0.028261	0.019113	0.012797	-0.006366
	0.030549	-0.025355	0.016713	-0.006381	0.016277	-0.012991
	0.055383	-0.024730	-0.006446	0.028396	0.027357	-0.004078
	0.016651	-0.018495	0.009198	-0.013247	0.003420	-0.020467
	-0.007354	0.012799	0.001808	-0.013066	-0.003080	0.010609
	0.010508					
y390	0.052514	0.022675	-0.011010	0.018051	0.022501	-0.004389
	0.006700	0.000744	-0.014781	-0.002740	0.005846	0.009323
	-0.005472	0.007954	-0.032170	-0.027553	0.009179	-0.000317
	-0.006582	0.032987	0.006742	0.054290	0.014945	0.042634
	-0.026146	0.055830	0.012294	-0.032195	-0.002057	0.013686
	-0.024149					
z390	0.023105	0.036868	-0.008484	0.035869	0.000349	-0.011107
	-0.007779	-0.016356	0.032313	0.013046	-0.004490	-0.024940
	0.014014	-0.021953	0.002128	0.003278	0.029468	-0.028961
	-0.016674	0.014913	-0.013894	0.001721	0.032698	0.017471
	0.024180	0.003385	0.019921	0.018273	0.011944	-0.005453
	0.019115					
x391	0.029071	-0.015512	0.044531	0.022913	0.016974	0.000487
	0.032282	-0.024823	0.017388	-0.007510	0.022596	-0.005158
	0.062173	-0.025179	-0.008916	0.028928	0.021167	0.003995
	0.010818	-0.017230	0.009424	-0.012985	-0.004254	-0.021637
	0.003597	0.011058	0.005927	-0.005641	-0.007051	0.017069
	0.011607					
y391	0.057631	0.019415	-0.007213	0.013661	0.027769	-0.005244
	0.005769	0.000148	-0.006008	-0.001561	0.012657	0.010868
	-0.002185	0.014241	-0.035663	-0.031103	0.003467	-0.006715
	-0.013808	0.051246	0.023716	0.049532	0.014429	0.054946
	-0.038027	0.061698	0.021211	-0.025661	-0.002158	0.020663
	-0.027909					

z391	0.023247	0.037450	-0.008515	0.025758	0.009771	-0.012945
	-0.002403	-0.009981	0.031579	0.010530	0.000045	-0.019733
	0.011377	-0.019655	0.009256	0.001733	0.022088	-0.026953
	-0.010168	0.020931	-0.013110	0.022926	0.027577	0.023016
	0.025659	-0.003231	0.018306	0.015727	0.011685	-0.015764
	0.013503					
x392	0.026108	-0.023008	0.058022	0.026954	0.020371	0.004233
	0.033932	-0.025293	0.019112	-0.007705	0.029016	0.000003
	0.067880	-0.025040	-0.015611	0.030701	0.016059	0.009746
	0.010090	-0.021275	0.008437	-0.017927	-0.017061	-0.023731
	0.014042	0.010337	0.012037	0.001999	-0.008529	0.025095
	0.013104					
y392	0.060682	0.015911	-0.001394	0.011155	0.032545	-0.005502
	0.006737	0.000338	0.002901	-0.000035	0.019307	0.012194
	0.000309	0.018131	-0.039361	-0.030472	-0.004418	-0.013872
	-0.019394	0.067663	0.044336	0.036687	0.013481	0.058785
	-0.047362	0.062764	0.036036	-0.015089	-0.000020	0.019313
	-0.028465					
z392	0.020994	0.036264	-0.006522	0.015555	0.019580	-0.014910
	0.004852	-0.002915	0.034869	0.004407	0.005468	-0.017853
	0.009902	-0.010736	0.009543	-0.003151	0.022823	-0.023368
	-0.005991	0.024358	-0.010838	0.031234	0.007479	0.026069
	0.009405	-0.004909	0.011514	0.018131	0.010117	-0.024485
	0.007750					
x393	0.018089	-0.023997	0.062095	0.029015	0.017831	0.009175
	0.030450	-0.022931	0.015000	-0.011637	0.028523	0.000739
	0.065342	-0.022234	-0.010249	0.027309	0.008321	0.017105
	0.010933	-0.024561	0.008787	-0.014948	-0.018173	-0.023663
	0.021634	0.004949	0.010512	0.004395	-0.007061	0.030393
	0.012940					
y393	0.060607	0.012423	0.004970	0.010300	0.035081	-0.003905
	0.005724	0.002447	0.010416	-0.000112	0.023367	0.013421
	-0.000030	0.021672	-0.039922	-0.024204	-0.009803	-0.017670
	-0.020699	0.075668	0.063109	0.022516	0.013722	0.057480
	-0.050627	0.061065	0.046410	-0.009900	0.001818	0.011641
	-0.027301					
z393	0.017497	0.032568	-0.002578	0.005894	0.024673	-0.015648
	0.008337	0.002522	0.039463	-0.001211	0.014196	-0.015075
	0.010156	-0.005559	0.015402	-0.002895	0.019366	-0.021662
	0.000263	0.027631	0.003284	0.029000	-0.008922	0.032482
	0.005941	-0.000725	0.007843	0.019677	0.016297	-0.023248
	0.009756					

x394	0.005967	-0.018807	0.057236	0.028976	0.012251	0.016701
	0.025604	-0.018044	0.004918	-0.017678	0.020034	-0.004076
	0.057065	-0.015757	0.002703	0.014606	-0.000862	0.023703
	0.009922	-0.022408	0.005112	-0.006746	-0.013557	-0.029436
	0.019655	-0.004350	0.001847	0.007521	-0.005126	0.029915
	0.011312					
y394	0.057238	0.010222	0.010248	0.010044	0.035623	0.002133
	0.004200	0.006744	0.012879	-0.003787	0.023708	0.014665
	0.000175	0.023129	-0.033037	-0.021467	-0.014287	-0.018831
	-0.022165	0.079358	0.072629	0.007230	0.014520	0.044066
	-0.052431	0.058418	0.053705	-0.005068	0.002601	-0.000344
	-0.021339					
z394	0.011901	0.028413	0.003782	-0.003484	0.032356	-0.012525
	0.012939	0.008042	0.046885	-0.004239	0.020356	-0.013311
	0.004952	-0.002533	0.016397	-0.006046	0.013556	-0.022195
	0.008712	0.020938	0.010940	0.023395	-0.028433	0.022355
	-0.003208	0.002789	0.013162	0.022618	0.017244	-0.029092
	0.011452					
x395	-0.003291	-0.013457	0.047159	0.027329	0.005679	0.018772
	0.021423	-0.015917	-0.002940	-0.018966	0.012115	-0.011169
	0.049768	-0.008780	0.010922	0.003014	-0.009613	0.027077
	0.011132	-0.018325	0.001915	0.001143	-0.011839	-0.030866
	0.019644	-0.011963	-0.004833	0.008041	-0.004534	0.032910
	0.004174					
y395	0.052222	0.008857	0.014637	0.009383	0.031753	0.010409
	0.000577	0.011556	0.011843	-0.009630	0.021238	0.015610
	-0.003729	0.021707	-0.023954	-0.017927	-0.014755	-0.016130
	-0.017653	0.072419	0.074539	-0.003149	0.017192	0.030094
	-0.048744	0.049779	0.054958	-0.007146	0.002250	-0.014096
	-0.014161					
z395	0.006695	0.025969	0.010642	-0.014789	0.034277	-0.008129
	0.016605	0.014652	0.054408	-0.006804	0.028235	-0.010393
	0.001405	0.000514	0.018862	-0.011313	0.010126	-0.016720
	0.014513	0.013697	0.010434	0.019146	-0.039428	0.009542
	-0.012798	0.008643	0.013789	0.021490	0.017545	-0.026189
	0.006436					
x396	-0.011045	-0.008792	0.033293	0.025880	-0.000707	0.014986
	0.018207	-0.017152	-0.009036	-0.016795	0.003727	-0.020469
	0.043782	-0.000523	0.011739	-0.006681	-0.015277	0.025943
	0.010014	-0.010367	0.001704	0.007194	-0.014312	-0.025038
	0.019410	-0.020804	-0.010285	0.010977	-0.002815	0.033484
	-0.006745					

y396	0.046492	0.006470	0.018870	0.008377	0.024673	0.019766
	-0.003513	0.015817	0.007559	-0.016185	0.018088	0.018245
	-0.008915	0.016586	-0.013215	-0.015263	-0.010503	-0.009996
	-0.010457	0.059434	0.066141	-0.011241	0.018626	0.010431
	-0.039704	0.040050	0.051089	-0.014789	0.000795	-0.023509
	-0.004458					
z396	-0.000722	0.026433	0.015613	-0.022091	0.032724	-0.004907
	0.018414	0.015624	0.061610	-0.013762	0.031735	-0.007608
	-0.011952	0.002422	0.017737	-0.027539	0.007137	-0.014135
	0.017599	0.007494	0.017625	0.012102	-0.045454	-0.009273
	-0.020763	0.012503	0.017627	0.029988	0.022201	-0.020861
	0.011264					
x397	-0.017478	-0.004569	0.016627	0.024182	-0.005826	0.002400
	0.015153	-0.022346	-0.013984	-0.011088	-0.006464	-0.033069
	0.039284	0.007667	0.003505	-0.017689	-0.014626	0.022106
	0.004816	0.004071	0.006259	0.011261	-0.025850	-0.010915
	0.017755	-0.033776	-0.015360	0.017025	-0.001186	0.022162
	-0.025194					
y397	0.040006	0.000732	0.023786	0.009255	0.015615	0.034342
	-0.005461	0.021168	0.002866	-0.024768	0.018681	0.024529
	-0.012688	0.010299	0.002159	-0.004741	-0.006269	-0.006280
	0.001659	0.035163	0.043822	-0.020716	0.027388	-0.006316
	-0.027287	0.035662	0.038103	-0.024762	0.001287	-0.012964
	0.005688					
z397	-0.008266	0.030314	0.018085	-0.027116	0.033128	-0.004788
	0.019155	0.012324	0.062288	-0.018052	0.035130	0.000829
	-0.020895	-0.001594	0.020921	-0.040577	0.000444	-0.013129
	0.023529	-0.001361	0.022337	0.001399	-0.038296	-0.030626
	-0.023668	0.009474	0.018914	0.024878	0.024453	-0.022759
	0.013523					
x398	-0.021950	-0.004684	0.002611	0.019671	-0.011666	-0.013872
	0.016980	-0.019039	-0.022509	-0.007285	-0.004524	-0.039780
	0.030476	0.007306	0.007829	-0.019453	-0.012203	0.015005
	0.002901	0.016834	0.010098	0.009041	-0.026344	-0.008190
	0.015929	-0.043981	-0.024717	0.023388	0.006784	0.026848
	-0.027662					
y398	0.029217	-0.000779	0.019766	0.011423	0.006468	0.042545
	-0.013385	0.013942	0.000784	-0.027513	0.003900	0.017182
	-0.009074	0.010186	-0.004771	-0.006895	0.000379	0.001040
	0.001941	0.020109	0.017100	-0.018570	0.016828	0.003105
	-0.014512	0.020443	0.034810	-0.029403	0.000702	-0.022733
	-0.001212					

z398	-0.014854	0.032835	0.017847	-0.026713	0.023313	-0.004818
	0.018077	0.003103	0.060636	-0.023613	0.030700	-0.000246
	-0.027570	-0.004921	0.021999	-0.041555	-0.004281	-0.016695
	0.021810	0.001017	0.028931	-0.019325	-0.031256	-0.033637
	-0.022674	-0.002694	0.023826	0.029788	0.026521	-0.027786
	0.018883					
x399	-0.025175	-0.005899	-0.007133	0.016138	-0.016406	-0.027918
	0.020017	-0.016029	-0.027090	-0.006891	0.000534	-0.041142
	0.023099	0.008180	0.009581	-0.015830	-0.011905	0.006623
	0.001821	0.024621	0.014892	0.005489	-0.017216	0.002262
	0.009356	-0.047594	-0.030589	0.025825	0.011573	0.035868
	-0.028913					
y399	0.020903	-0.001453	0.015798	0.012680	0.000591	0.041859
	-0.021689	0.005744	-0.002125	-0.029662	-0.006764	0.008577
	-0.005486	0.007809	-0.015064	-0.014581	0.011917	0.008273
	-0.004606	0.013999	-0.006336	-0.018461	-0.002936	0.014428
	-0.003632	0.005480	0.030408	-0.023750	0.002878	-0.039191
	-0.012932					
z399	-0.022003	0.034165	0.017963	-0.023135	0.013947	-0.000352
	0.019716	-0.008085	0.054781	-0.027292	0.027552	-0.001061
	-0.032549	-0.002422	0.015905	-0.028739	-0.007620	-0.022974
	0.020272	-0.001774	0.022077	-0.038162	-0.025674	-0.032296
	-0.025006	-0.010807	0.032833	0.032035	0.029115	-0.018611
	0.032770					
x400	-0.026741	-0.006464	-0.013624	0.012959	-0.017897	-0.038949
	0.023044	-0.015148	-0.029458	-0.007505	0.006555	-0.040765
	0.020129	0.008163	0.007626	-0.011707	-0.011723	-0.000287
	-0.001520	0.031525	0.019020	0.000277	-0.010133	0.013729
	-0.000088	-0.046709	-0.029107	0.028001	0.011389	0.037172
	-0.028012					
y400	0.013992	-0.001097	0.013772	0.012707	-0.003886	0.035177
	-0.029219	-0.001730	-0.004390	-0.032711	-0.014316	0.003336
	-0.003218	0.004816	-0.024835	-0.021830	0.023858	0.015722
	-0.013783	0.010723	-0.023590	-0.020447	-0.020694	0.026068
	0.005354	-0.007355	0.024450	-0.013046	0.005841	-0.052881
	-0.024581					
z400	-0.025079	0.034148	0.017462	-0.015796	0.005207	0.004140
	0.022247	-0.015225	0.049328	-0.028409	0.023477	-0.005457
	-0.032930	0.005671	0.008116	-0.024856	-0.012002	-0.022462
	0.029059	-0.004338	0.013797	-0.035124	-0.011720	-0.019293
	-0.012758	-0.003198	0.030506	0.015687	0.020432	-0.019035
	0.036749					

x401	-0.027800	-0.007176	-0.017073	0.010001	-0.016470	-0.044078
	0.024538	-0.016203	-0.028401	-0.007163	0.011990	-0.037604
	0.020410	0.006896	0.003828	-0.008030	-0.011132	-0.003650
	-0.005112	0.034960	0.021652	-0.003963	-0.004517	0.023686
	-0.010652	-0.040784	-0.020570	0.027985	0.006705	0.033470
	-0.021222					
y401	0.008604	0.000553	0.014197	0.011251	-0.004813	0.023861
	-0.034530	-0.008680	-0.008873	-0.036203	-0.019482	0.001225
	0.001195	-0.001542	-0.033086	-0.027967	0.035748	0.024071
	-0.029193	0.010230	-0.031106	-0.026237	-0.036976	0.040104
	0.013504	-0.018263	0.019435	-0.001832	0.003011	-0.072175
	-0.035686					
z401	-0.025637	0.032354	0.018563	-0.005871	-0.003862	0.007644
	0.022128	-0.022424	0.047524	-0.027246	0.021463	-0.008241
	-0.031105	0.020114	-0.005950	-0.018378	-0.019318	-0.015257
	0.041031	-0.007448	0.000255	-0.034158	-0.007234	-0.002505
	-0.005402	0.005968	0.030682	0.005263	0.015999	-0.029856
	0.039014					
x402	-0.029377	-0.008778	-0.020253	0.008273	-0.014659	-0.041969
	0.025392	-0.016014	-0.023075	-0.006105	0.017402	-0.034497
	0.021027	0.008297	0.001292	-0.003674	-0.011803	-0.005007
	0.000411	0.033873	0.021573	-0.003034	0.003277	0.025784
	-0.022578	-0.029748	-0.009517	0.023628	0.003618	0.037880
	-0.005536					
y402	0.001369	0.000432	0.009591	0.009596	-0.008640	0.013017
	-0.032241	-0.010805	-0.011803	-0.035441	-0.019844	-0.002740
	0.001798	-0.000732	-0.031340	-0.029624	0.037529	0.023092
	-0.029281	0.007368	-0.030326	-0.021738	-0.035944	0.044648
	0.016178	-0.018895	0.012112	0.006992	0.004463	-0.068412
	-0.034902					
z402	-0.026300	0.031792	0.016743	0.004346	-0.012352	0.010057
	0.020076	-0.024906	0.037829	-0.024129	0.015306	-0.010966
	-0.025835	0.024665	-0.007291	-0.005215	-0.018811	-0.019848
	0.034316	-0.008468	-0.009302	-0.030755	-0.001610	0.004599
	-0.006992	-0.004920	0.017201	0.002363	0.007328	-0.020353
	0.035835					
x403	-0.029482	-0.009410	-0.019921	0.006918	-0.009263	-0.035636
	0.022897	-0.016621	-0.016418	-0.004679	0.020468	-0.028566
	0.022910	0.005881	-0.003768	-0.001655	-0.009645	-0.001959
	0.005850	0.030117	0.017461	-0.006737	0.005785	0.026234
	-0.029976	-0.020459	0.002885	0.016969	0.003156	0.037350
	0.010031					

y403	-0.008279	-0.002015	-0.000041	0.007911	-0.014051	0.006206
	-0.022948	-0.009211	-0.013487	-0.029280	-0.015958	-0.008883
	0.000553	0.004545	-0.019576	-0.028223	0.031690	0.010771
	-0.016744	0.001275	-0.025547	-0.008910	-0.019994	0.037084
	0.012087	-0.009239	0.005364	0.014882	0.008722	-0.041454
	-0.021206					
z403	-0.024685	0.030702	0.015026	0.014958	-0.022859	0.010645
	0.017414	-0.026957	0.020767	-0.020393	0.007276	-0.013346
	-0.014446	0.021425	-0.007458	0.009539	-0.016397	-0.025564
	0.025133	-0.011739	-0.011630	-0.026640	0.006015	0.018885
	-0.001606	-0.016229	0.012293	-0.012813	0.004901	-0.007546
	0.031294					
x404	-0.027390	-0.008479	-0.016414	0.004981	-0.001671	-0.028543
	0.015487	-0.017948	-0.006575	-0.005424	0.021269	-0.020952
	0.020788	0.002805	-0.010928	0.001917	-0.004020	0.007531
	0.013525	0.027554	0.011372	-0.011629	0.002385	0.026530
	-0.026848	-0.014056	0.012180	0.011985	-0.000293	0.027938
	0.020837					
y404	-0.016707	-0.004430	-0.006478	0.004662	-0.014315	-0.000996
	-0.015358	-0.007684	-0.012393	-0.023704	-0.010841	-0.011562
	0.001748	0.004812	-0.009922	-0.028804	0.029247	0.004869
	-0.007564	-0.004171	-0.019938	-0.004712	-0.006901	0.033214
	0.011558	0.001329	0.004181	0.022211	0.006561	-0.027123
	-0.006473					
z404	-0.019844	0.027408	0.013487	0.023797	-0.033454	0.007292
	0.017178	-0.025567	0.008662	-0.019813	0.003077	-0.020093
	-0.006842	0.024578	-0.010398	0.018606	-0.012037	-0.025357
	0.027978	-0.004630	-0.012167	-0.016289	0.010765	0.012730
	0.005943	-0.019001	0.017975	-0.019739	-0.009470	-0.002941
	0.043444					
x405	-0.023191	-0.006422	-0.011696	0.003936	0.005473	-0.022450
	0.006851	-0.020086	0.004697	-0.006811	0.018989	-0.013345
	0.014705	0.000755	-0.018050	0.004479	0.000243	0.020240
	0.022774	0.023063	0.007410	-0.016311	-0.003514	0.024861
	-0.019990	-0.007916	0.013775	0.010490	0.000113	0.014899
	0.026932					
y405	-0.023965	-0.006291	-0.009943	-0.000642	-0.010311	-0.009544
	-0.009906	-0.005568	-0.007832	-0.019200	-0.005139	-0.010604
	0.003449	0.003528	-0.002694	-0.029754	0.028431	0.003668
	0.001286	-0.008349	-0.013440	-0.006918	0.003036	0.032330
	0.013516	0.010439	0.002179	0.030156	0.003277	-0.021210
	0.004167					

z405	-0.012434	0.023007	0.012145	0.034112	-0.042283	0.001119
	0.016091	-0.026233	0.000082	-0.020561	-0.000940	-0.026807
	0.000960	0.025816	-0.016038	0.021124	-0.015098	-0.021162
	0.024353	-0.005493	-0.013404	-0.004504	0.012302	0.009812
	0.009202	-0.021872	0.024738	-0.025966	-0.019250	-0.000269
	0.055441					
x406	-0.018090	-0.002924	-0.007974	0.001670	0.010452	-0.016943
	-0.000964	-0.023353	0.013231	-0.004026	0.015409	-0.006151
	0.003758	-0.000918	-0.018096	0.009775	0.003205	0.025958
	0.028762	0.015295	0.009178	-0.019299	-0.011348	0.019480
	-0.015621	-0.006240	0.005902	0.011950	0.002513	0.011978
	0.022392					
y406	-0.030528	-0.007293	-0.010847	-0.008375	-0.004040	-0.019625
	-0.006288	-0.002353	-0.001372	-0.014030	0.001808	-0.006290
	0.004004	0.002295	0.006015	-0.027541	0.026988	0.004384
	0.011084	-0.011795	-0.002682	-0.013230	0.011833	0.033857
	0.017248	0.017081	-0.003473	0.037714	0.001456	-0.016921
	0.007532					
z406	-0.004360	0.019114	0.010081	0.040074	-0.049020	-0.006229
	0.011437	-0.026861	-0.006531	-0.012618	-0.004443	-0.031090
	0.004924	0.022272	-0.016360	0.022251	-0.017292	-0.023906
	0.002075	-0.008067	-0.016041	0.007484	0.016604	0.014069
	-0.003943	-0.032935	0.026315	-0.028219	-0.023370	0.018164
	0.050190					
x407	-0.013292	-0.002036	-0.008800	0.003877	0.016328	-0.006737
	-0.005687	-0.025423	0.013711	0.000145	0.008297	-0.000597
	0.001265	-0.000968	-0.018730	0.024524	0.008174	0.035194
	0.030037	0.015947	0.011804	-0.015697	-0.005135	0.021516
	-0.014393	-0.015501	0.013697	0.019692	0.010398	0.001720
	0.019827					
y407	-0.035096	-0.008816	-0.012731	-0.011560	0.004208	-0.022543
	-0.003676	0.001680	-0.000914	-0.009261	0.004639	-0.000950
	0.009783	0.002378	0.010067	-0.013927	0.027723	0.011114
	0.018245	-0.005651	0.007034	-0.013999	0.031137	0.044139
	0.019312	0.007872	0.002948	0.045618	0.007961	-0.023261
	0.004528					
z407	0.003331	0.015611	0.009901	0.042894	-0.051741	-0.014390
	0.005077	-0.027372	-0.003901	-0.006367	-0.006181	-0.033400
	-0.000644	0.018321	-0.016878	0.011681	-0.013071	-0.018823
	-0.013767	-0.017129	-0.014471	0.012593	0.013586	0.011649
	-0.010326	-0.031186	0.019629	-0.022808	-0.040053	0.011315
	0.057922					

x408	-0.008896	-0.001734	-0.010606	0.005756	0.022685	0.002600
	-0.008305	-0.027923	0.016205	0.005988	0.000998	0.003437
	-0.001624	-0.002122	-0.021122	0.031345	0.009134	0.042083
	0.032520	0.016903	0.012226	-0.009338	0.000327	0.018135
	-0.017844	-0.020616	0.024964	0.019665	0.014508	-0.007084
	0.015419					
y408	-0.038910	-0.009770	-0.014277	-0.014159	0.012767	-0.025294
	-0.001910	0.004351	0.002666	-0.006229	0.008742	0.003990
	0.010637	0.000753	0.010307	-0.004864	0.027157	0.017818
	0.024752	0.002983	0.016142	-0.015394	0.051103	0.050640
	0.020092	0.002721	0.007707	0.045491	0.016298	-0.036576
	0.003744					
z408	0.010566	0.013584	0.009318	0.042746	-0.050984	-0.024463
	-0.000877	-0.025513	-0.002901	0.002787	-0.008048	-0.030925
	-0.005696	0.010726	-0.013595	-0.001361	-0.021265	-0.013912
	-0.026542	-0.014751	-0.011304	0.011578	0.013136	0.011007
	-0.017402	-0.024296	0.015988	-0.016486	-0.056251	0.015267
	0.044518					
x409	-0.004371	-0.002279	-0.011833	0.007745	0.028399	0.012024
	-0.008766	-0.031225	0.020297	0.011302	-0.005946	0.005231
	-0.001613	0.001061	-0.025920	0.032906	0.010147	0.049893
	0.038659	0.015126	0.009035	0.001113	0.000527	0.013288
	-0.027210	-0.021108	0.035876	0.015454	0.019809	-0.015286
	0.011388					
y409	-0.041624	-0.010270	-0.016128	-0.016102	0.020125	-0.027238
	-0.000990	0.004280	0.008006	-0.006295	0.013944	0.008047
	0.009232	0.001813	0.006769	0.000507	0.027156	0.024138
	0.030397	0.009099	0.027154	-0.016243	0.065945	0.056250
	0.019427	-0.001875	0.006597	0.037018	0.026843	-0.052649
	0.006674					
z409	0.018125	0.011568	0.010352	0.043398	-0.048098	-0.032102
	-0.004134	-0.024429	0.000413	0.007930	-0.006774	-0.029184
	-0.007347	0.011011	-0.014305	-0.012080	-0.021862	-0.003076
	-0.031467	-0.013731	-0.014892	0.014605	0.015672	0.005448
	-0.038687	-0.006768	0.007602	-0.012025	-0.047109	0.012792
	0.025444					
x410	-0.000584	-0.004208	-0.012285	0.009241	0.033538	0.022642
	-0.006973	-0.034310	0.024949	0.018215	-0.014440	0.004255
	0.000641	0.007933	-0.026963	0.033576	0.010181	0.057022
	0.047056	0.011115	0.001595	0.018127	-0.006552	0.003783
	-0.033031	-0.022366	0.054580	0.011175	0.019514	-0.016608
	0.011349					

y410	-0.044122	-0.012610	-0.018914	-0.018344	0.026082	-0.028142
	-0.000125	0.002199	0.014312	-0.005149	0.020315	0.008283
	0.005457	0.005382	0.003458	0.006912	0.028805	0.026138
	0.035573	0.011242	0.034630	-0.015281	0.071040	0.057988
	0.019888	-0.005264	0.003515	0.023615	0.029180	-0.068187
	0.010195					
z410	0.022462	0.007244	0.010731	0.040564	-0.043183	-0.038516
	-0.007351	-0.022714	0.005808	0.012735	-0.003139	-0.029906
	-0.012563	0.010520	-0.005024	-0.016783	-0.020723	-0.001075
	-0.033377	-0.014838	-0.017278	0.015936	0.011018	-0.005999
	-0.032335	0.009954	0.003851	-0.001398	-0.055695	0.011960
	0.020292					
x411	0.006431	-0.002122	-0.007424	0.007968	0.028851	0.022606
	-0.012844	-0.034035	0.024476	0.016322	-0.017614	0.004534
	-0.004231	0.008696	-0.019865	0.030676	0.008430	0.051700
	0.044829	0.004216	-0.001997	0.019537	-0.011605	-0.007889
	-0.033014	-0.018618	0.053601	0.010966	0.016837	-0.009962
	0.011506					
y411	-0.045408	-0.015177	-0.021366	-0.019186	0.031362	-0.025613
	0.001659	-0.003551	0.018540	-0.001605	0.024060	0.009082
	0.003857	0.010742	-0.003766	0.010728	0.029855	0.029046
	0.039906	0.009544	0.039761	-0.009773	0.068890	0.056577
	0.014686	-0.009156	0.002004	0.002420	0.032549	-0.075653
	0.012293					
z411	0.026027	0.004157	0.007629	0.037362	-0.032996	-0.044504
	-0.010148	-0.024138	0.008580	0.018908	0.002574	-0.024929
	-0.014465	0.008125	-0.000646	-0.023031	-0.018301	0.003305
	-0.027717	-0.015602	-0.021576	0.013188	0.006885	-0.013719
	-0.030509	0.017171	-0.000444	0.005432	-0.044468	0.012187
	0.008195					
x412	0.016871	0.004483	0.003549	0.003061	0.014599	0.011224
	-0.025602	-0.029029	0.020697	0.003667	-0.017098	0.004848
	-0.016139	0.005663	-0.004760	0.024336	0.006530	0.035754
	0.031393	-0.002970	0.000201	0.005253	-0.012725	-0.021513
	-0.026920	-0.010716	0.035328	0.017234	0.006480	-0.001704
	0.014237					
y412	-0.043297	-0.016593	-0.020489	-0.019680	0.030891	-0.023480
	0.000323	-0.010033	0.020330	0.000119	0.025484	0.008942
	0.000302	0.014625	-0.009455	0.015008	0.028870	0.028537
	0.036742	0.005271	0.041355	-0.006746	0.061559	0.050600
	0.008238	-0.011841	-0.003597	-0.018768	0.033877	-0.069964
	0.015053					

z412	0.027624	0.002219	0.002975	0.032207	-0.018427	-0.046226
	-0.009922	-0.025434	0.015537	0.022242	0.002633	-0.019343
	-0.015898	0.009915	0.000608	-0.026941	-0.009110	0.013888
	-0.018822	-0.010083	-0.023241	0.008012	0.001959	-0.020862
	-0.036318	0.010668	0.002490	0.018659	-0.023432	-0.000937
	0.001691					
x413	0.026536	0.011829	0.015773	-0.001586	0.003974	-0.000698
	-0.034593	-0.022921	0.016752	-0.009317	-0.016959	0.006980
	-0.024588	0.009945	0.009654	0.014292	0.002888	0.023765
	0.024272	-0.008448	-0.001969	-0.001497	-0.013866	-0.034683
	-0.020470	-0.006285	0.025091	0.026997	-0.002296	0.006182
	0.014220					
y413	-0.037569	-0.016937	-0.015252	-0.019868	0.026230	-0.022768
	-0.004356	-0.017655	0.018596	0.000146	0.023764	0.008107
	-0.003285	0.017641	-0.014825	0.018957	0.026017	0.024776
	0.029610	0.000629	0.039451	-0.004861	0.051005	0.041697
	0.003689	-0.015390	-0.013265	-0.034687	0.030854	-0.049516
	0.014444					
z413	0.029772	-0.000174	-0.002034	0.029359	0.000591	-0.047753
	-0.007092	-0.026587	0.019188	0.022080	0.007480	-0.009767
	-0.019087	0.015023	0.001144	-0.033010	-0.006981	0.021729
	-0.008442	-0.007557	-0.031786	0.004482	-0.007598	-0.023288
	-0.042233	0.002264	-0.005536	0.034326	-0.005756	-0.001404
	-0.014010					
x414	0.034638	0.019066	0.026793	-0.006356	-0.004200	-0.012470
	-0.039943	-0.014737	0.013750	-0.021936	-0.018372	0.010147
	-0.030381	0.019387	0.023766	0.001532	0.001758	0.014563
	0.021454	-0.012883	-0.006369	-0.003249	-0.013584	-0.047275
	-0.012951	-0.002811	0.024700	0.033716	-0.009956	0.005070
	0.010626					
y414	-0.028977	-0.016411	-0.007219	-0.020337	0.019687	-0.021377
	-0.010274	-0.025539	0.014610	0.001546	0.017193	0.006571
	-0.004681	0.022371	-0.018952	0.023967	0.023778	0.019401
	0.022042	-0.004638	0.032708	-0.002264	0.036038	0.031178
	0.003180	-0.020903	-0.017746	-0.038138	0.024083	-0.018060
	0.011250					
z414	0.032736	-0.004714	-0.008771	0.027751	0.015765	-0.046504
	-0.004490	-0.027201	0.020193	0.023004	0.007906	-0.001242
	-0.020292	0.023485	0.007524	-0.036198	0.008191	0.023453
	-0.002297	-0.010132	-0.033816	0.004273	-0.015538	-0.027872
	-0.040865	-0.005282	-0.015443	0.039390	0.007461	-0.003108
	-0.033323					

x415	0.038226	0.023799	0.029012	-0.006606	-0.007463	-0.018731
	-0.036533	-0.003684	0.013973	-0.030375	-0.020880	0.013252
	-0.039136	0.034117	0.027304	-0.008100	0.002737	0.013992
	0.021124	-0.018353	-0.018453	-0.004184	-0.018041	-0.056969
	-0.001858	-0.011283	0.023073	0.024873	-0.004715	-0.004279
	0.001884					
y415	-0.015841	-0.013157	0.008357	-0.025960	0.009514	-0.025621
	-0.018437	-0.030402	0.008090	0.000525	0.008571	0.004433
	0.000679	0.020462	-0.007886	0.027022	0.016743	0.003974
	0.014470	-0.008623	0.037043	-0.000003	0.027982	0.024389
	-0.001796	-0.014880	-0.004278	-0.003306	0.001736	0.016087
	0.015522					
z415	0.034918	-0.010852	-0.016760	0.027136	0.030499	-0.042056
	0.005593	-0.024788	0.021613	0.029227	0.002279	0.001570
	-0.019992	0.030524	0.004492	-0.033475	0.027654	0.020228
	-0.007473	-0.019202	-0.026466	-0.010319	-0.013145	-0.033018
	-0.051991	-0.019921	-0.021142	0.056297	0.016126	-0.001348
	-0.026770					
x416	0.041228	0.024165	0.024009	-0.000210	-0.008429	-0.021212
	-0.024746	0.008188	0.004121	-0.035609	-0.025638	0.022331
	-0.036804	0.037594	0.025600	-0.015081	-0.000605	0.003571
	0.015940	-0.008553	-0.022480	-0.008078	-0.019092	-0.052059
	0.011493	-0.010036	0.040775	0.033830	-0.000470	0.026526
	-0.012481					
y416	-0.001294	-0.003044	0.026078	-0.035242	-0.001303	-0.033244
	-0.027285	-0.029343	0.004778	-0.004292	0.003752	0.002490
	-0.005674	0.024770	0.001080	0.025837	0.011356	-0.001319
	0.013014	-0.019604	0.034960	0.010095	0.012819	0.014649
	-0.001722	-0.026399	-0.004698	0.008371	-0.015851	0.013556
	0.017027					
z416	0.039261	-0.021831	-0.025475	0.031798	0.044946	-0.033445
	0.020990	-0.018729	0.017703	0.035484	-0.000785	0.008977
	-0.014421	0.036418	-0.002859	-0.032198	0.033147	0.015063
	-0.014105	-0.027011	-0.007603	-0.016711	-0.004575	-0.024806
	-0.048111	-0.040148	-0.016920	0.047614	0.007682	0.011713
	-0.033553					
x417	0.042727	0.025274	0.016759	0.004161	-0.009878	-0.021287
	-0.015228	0.016889	-0.003248	-0.040227	-0.027690	0.029632
	-0.033456	0.029278	0.031049	-0.021818	-0.007614	-0.010219
	0.010335	-0.003046	-0.014017	-0.018361	-0.019341	-0.047062
	0.017062	-0.000117	0.056103	0.054812	-0.001993	0.034589
	-0.014928					

y417	0.008206	0.003166	0.033253	-0.037820	-0.007400	-0.033841
	-0.029379	-0.026359	0.003248	-0.006170	-0.004059	0.001597
	-0.010044	0.025823	0.003373	0.023531	0.008615	0.000925
	0.012581	-0.026594	0.029851	0.015510	-0.000357	0.008199
	0.002730	-0.038953	-0.004439	0.006798	-0.022229	0.010707
	0.011794					
z417	0.041237	-0.033422	-0.029983	0.035371	0.050861	-0.024438
	0.029399	-0.010155	0.011134	0.041263	-0.000510	0.012430
	-0.015296	0.035755	-0.010879	-0.021054	0.029437	0.010722
	-0.027680	-0.028605	-0.005139	-0.028150	0.004958	-0.031109
	-0.021529	-0.045987	0.000100	0.032263	-0.015252	0.019683
	-0.017110					
x418	0.041346	0.024742	0.004705	0.010458	-0.006816	-0.015152
	-0.005241	0.018539	-0.004383	-0.039273	-0.027554	0.035979
	-0.029691	0.017940	0.031562	-0.029933	-0.014710	-0.022645
	0.005944	0.000948	-0.004488	-0.027572	-0.025568	-0.038982
	0.014924	0.003017	0.052295	0.057019	0.002735	0.026383
	-0.018939					
y418	0.015688	0.008310	0.035069	-0.037854	-0.012280	-0.031872
	-0.027566	-0.022636	0.005706	-0.008674	-0.011419	0.000867
	-0.013041	0.023118	0.005245	0.016507	0.007927	0.005464
	0.013832	-0.028305	0.030999	0.020040	-0.011475	0.004301
	0.005465	-0.039882	-0.003790	0.005801	-0.023980	0.002395
	0.008980					
z418	0.042805	-0.042779	-0.026441	0.033417	0.050879	-0.021696
	0.030842	-0.006172	0.013620	0.039082	0.004798	0.013665
	-0.016097	0.037668	-0.018166	-0.019084	0.029570	0.008288
	-0.029417	-0.020791	-0.000709	-0.015468	0.002439	-0.030617
	-0.003865	-0.032223	0.000895	0.002920	-0.035300	0.015934
	-0.000774					
x419	0.037656	0.023406	-0.008572	0.018159	-0.002149	-0.005222
	0.003082	0.015257	-0.002589	-0.034349	-0.026219	0.039304
	-0.024382	0.006949	0.029854	-0.035065	-0.024783	-0.033541
	0.003897	-0.000694	0.003782	-0.035250	-0.030372	-0.029683
	0.010957	-0.003954	0.040046	0.051367	0.008464	0.011241
	-0.019678					
y419	0.021300	0.013377	0.032121	-0.035618	-0.016066	-0.027457
	-0.023045	-0.018762	0.009581	-0.009392	-0.017319	0.000736
	-0.015107	0.016743	0.006294	0.008648	0.005302	0.007649
	0.012822	-0.026519	0.033626	0.016045	-0.018706	0.003907
	0.004342	-0.037914	-0.004435	0.012472	-0.016580	-0.011006
	0.008517					

z419	0.042577	-0.048882	-0.017963	0.027866	0.043855	-0.023181
	0.028637	0.000031	0.014893	0.035663	0.009052	0.011190
	-0.014003	0.039318	-0.021496	-0.015557	0.028897	0.006518
	-0.022328	-0.007186	0.009982	-0.014052	0.011248	-0.024358
	0.003462	-0.022413	0.007816	0.012242	-0.033212	0.011374
	0.027730					
x420	0.032916	0.021202	-0.020249	0.026639	0.002257	0.006006
	0.009526	0.008170	-0.000237	-0.026899	-0.021574	0.039393
	-0.017775	-0.001551	0.028267	-0.037872	-0.037225	-0.042059
	0.004339	-0.008712	0.008284	-0.035417	-0.034205	-0.019516
	0.008384	-0.015811	0.026196	0.038085	0.008943	-0.001485
	-0.022677					
y420	0.026047	0.018734	0.024971	-0.030183	-0.017583	-0.020398
	-0.016563	-0.017213	0.014464	-0.007906	-0.021590	0.001489
	-0.017514	0.007531	0.005766	0.000664	-0.000650	0.006205
	0.007645	-0.028659	0.032832	0.011418	-0.026693	0.008541
	0.003526	-0.036858	-0.008371	0.012806	-0.008301	-0.026528
	0.002603					
z420	0.044079	-0.051523	-0.010551	0.025157	0.036874	-0.025497
	0.026293	0.005302	0.017640	0.032003	0.015292	0.009415
	-0.015223	0.037005	-0.028212	-0.015341	0.018810	0.005423
	-0.020101	-0.009503	0.002512	-0.002278	0.010820	-0.003919
	0.007834	-0.013669	0.014415	0.012033	-0.029334	0.015361
	0.029071					
x421	0.027769	0.017367	-0.027986	0.034111	0.005406	0.015308
	0.014357	0.000002	0.000305	-0.018495	-0.014774	0.034078
	-0.009939	-0.005198	0.029088	-0.037891	-0.047902	-0.045413
	0.010111	-0.016944	0.012164	-0.030966	-0.032243	-0.010876
	0.005052	-0.028973	0.016708	0.025395	0.002193	-0.007057
	-0.026058					
y421	0.029961	0.023785	0.015217	-0.022363	-0.018917	-0.010682
	-0.008253	-0.018024	0.019195	-0.003980	-0.024891	0.001388
	-0.019755	-0.004952	0.005742	-0.006252	-0.007284	0.001726
	0.001208	-0.031579	0.032985	0.005985	-0.031923	0.014973
	0.002415	-0.039987	-0.010784	0.012223	-0.000839	-0.039230
	-0.004185					
z421	0.045931	-0.052846	-0.005538	0.022129	0.026904	-0.027315
	0.025100	0.012396	0.021552	0.027543	0.017268	0.006784
	-0.018168	0.028478	-0.032548	-0.017248	0.014946	0.006642
	-0.021517	-0.008729	-0.001618	-0.001452	0.014656	0.009476
	0.007554	-0.012148	0.021648	0.011015	-0.023150	0.024371
	0.034706					

x422	0.022523	0.012148	-0.029206	0.039089	0.008754	0.019750
	0.016205	-0.006307	-0.002365	-0.011811	-0.006624	0.025501
	0.001556	-0.000971	0.032212	-0.033715	-0.058725	-0.042132
	0.020451	-0.019596	0.013370	-0.022384	-0.022622	-0.005201
	-0.001284	-0.035586	0.013085	0.016209	-0.010385	-0.006571
	-0.029851					
y422	0.033399	0.027780	0.005365	-0.012608	-0.020536	0.000848
	0.001868	-0.021250	0.023738	0.002825	-0.024643	-0.001553
	-0.021573	-0.016756	0.007158	-0.012221	-0.014898	-0.004511
	-0.004410	-0.033607	0.035401	0.003315	-0.033388	0.023750
	-0.000544	-0.041404	-0.009172	0.012331	0.007695	-0.046948
	-0.012843					
z422	0.046891	-0.053886	-0.000412	0.016347	0.017668	-0.027343
	0.022659	0.022507	0.025703	0.021679	0.021053	0.005886
	-0.022325	0.024544	-0.030066	-0.016252	0.010322	0.007111
	-0.022121	-0.005283	-0.003923	0.003122	0.014494	0.026176
	0.016745	0.004617	0.015624	0.003578	-0.019154	0.021732
	0.032052					
x423	0.016877	0.006263	-0.023256	0.039682	0.012584	0.017181
	0.012708	-0.007086	-0.010319	-0.010178	-0.001009	0.016142
	0.018907	0.011692	0.037009	-0.024220	-0.070879	-0.032184
	0.037517	-0.015466	0.007096	-0.010268	-0.005630	-0.002861
	-0.006836	-0.036673	0.018966	0.012352	-0.031526	0.003165
	-0.034870					
y423	0.037225	0.030733	-0.003379	-0.000668	-0.023241	0.012815
	0.013687	-0.026084	0.027084	0.011472	-0.020214	-0.007402
	-0.022980	-0.028046	0.009305	-0.017889	-0.023340	-0.011221
	-0.008910	-0.031036	0.039074	0.001328	-0.030224	0.033112
	-0.007830	-0.039355	-0.000083	0.017694	0.016076	-0.049000
	-0.020189					
z423	0.047609	-0.054443	0.002419	0.009732	0.009579	-0.024470
	0.021602	0.029128	0.026768	0.012205	0.029901	0.010541
	-0.027511	0.024398	-0.025263	-0.012756	0.002205	0.004634
	-0.014368	-0.003799	-0.006143	0.006498	0.000178	0.032239
	0.028476	0.009411	0.001976	-0.003305	-0.004803	0.018216
	0.028802					
x424	0.008705	-0.000474	-0.010392	0.033534	0.015690	0.006039
	0.002759	-0.000002	-0.024503	-0.013101	-0.002370	0.010147
	0.046402	0.029407	0.042045	-0.007116	-0.085386	-0.016736
	0.063500	-0.005876	-0.015493	0.006028	0.017842	-0.005225
	-0.000661	-0.034049	0.037840	0.016176	-0.063804	0.026393
	-0.048039					

y424	0.041690	0.033045	-0.009666	0.011924	-0.026230	0.023918
	0.024214	-0.030935	0.027727	0.020402	-0.013464	-0.018994
	-0.024354	-0.037245	0.014118	-0.020668	-0.037133	-0.018752
	-0.013140	-0.020830	0.044812	0.000292	-0.016175	0.039764
	-0.019254	-0.023739	0.022707	0.028872	0.024119	-0.045978
	-0.023226					
z424	0.045860	-0.051625	0.004752	0.001152	0.004967	-0.015090
	0.018385	0.030553	0.030393	0.003849	0.031847	0.010188
	-0.036850	0.020997	-0.020275	-0.014285	-0.010082	0.010799
	-0.010066	-0.008671	0.008379	0.000945	-0.010801	0.043058
	0.026370	0.012536	-0.014153	-0.008591	0.004010	-0.008830
	0.027804					
x425	0.005261	-0.005503	-0.013359	0.029660	0.018317	0.008033
	-0.003477	0.002732	-0.023489	-0.014998	0.001682	0.001715
	0.038674	0.027152	0.033512	-0.007289	-0.071016	-0.016669
	0.051118	0.000299	-0.013465	0.004564	0.019653	-0.007257
	-0.005863	-0.024362	0.020366	0.007727	-0.049451	0.015620
	-0.027196					
y425	0.037273	0.032202	-0.008967	0.012108	-0.024263	0.024788
	0.029636	-0.035699	0.021677	0.020643	-0.009604	-0.017983
	-0.018390	-0.031533	0.019398	-0.018645	-0.042237	-0.006820
	-0.007417	-0.020568	0.051436	0.001675	0.000415	0.040177
	-0.018699	-0.031912	0.026958	0.030711	0.016478	-0.031380
	-0.031311					
z425	0.044799	-0.048338	0.005083	-0.000776	0.000563	-0.011641
	0.018096	0.030171	0.028962	-0.003647	0.033726	0.015089
	-0.038108	0.021111	-0.014987	-0.016082	-0.011301	0.009902
	-0.004667	-0.012145	0.007523	0.005861	-0.016137	0.046407
	0.024671	0.004954	-0.014308	-0.017978	0.008413	-0.004845
	0.021727					
x426	0.000938	-0.010373	-0.016721	0.025031	0.019894	0.009101
	-0.009595	0.006671	-0.024378	-0.016922	0.002331	-0.003387
	0.034604	0.023371	0.024849	-0.005792	-0.056810	-0.018062
	0.041010	0.006936	-0.015331	0.003623	0.019984	-0.010726
	-0.005816	-0.016395	0.004787	0.001911	-0.039939	0.004340
	-0.011896					
y426	0.033415	0.030196	-0.008276	0.012518	-0.021958	0.026736
	0.032290	-0.039126	0.017820	0.020036	-0.004492	-0.018382
	-0.016708	-0.026998	0.023327	-0.017032	-0.042793	0.002273
	-0.002688	-0.020005	0.054980	0.002178	0.011675	0.042467
	-0.018137	-0.037575	0.030639	0.031902	0.009570	-0.015527
	-0.036409					

z426	0.041659	-0.044348	0.007367	-0.003099	-0.003407	-0.008024
	0.018711	0.031741	0.027228	-0.009098	0.032333	0.017331
	-0.038161	0.022287	-0.010534	-0.016354	-0.015491	0.008738
	0.000647	-0.010717	0.011292	0.008512	-0.010747	0.042487
	0.027910	0.004184	-0.015568	-0.026850	0.010306	-0.011164
	0.020381					
x427	-0.003351	-0.014684	-0.020350	0.020462	0.021290	0.009474
	-0.014923	0.010445	-0.026140	-0.018461	0.001981	-0.005635
	0.033620	0.019449	0.017233	-0.003823	-0.044621	-0.020095
	0.032569	0.012369	-0.019971	0.003540	0.018001	-0.013305
	-0.004572	-0.011493	-0.009173	-0.001041	-0.033708	-0.005784
	-0.000013					
y427	0.029632	0.026987	-0.007926	0.012248	-0.019977	0.029323
	0.032351	-0.040760	0.014410	0.018011	-0.000898	-0.018998
	-0.017387	-0.024566	0.025550	-0.016164	-0.037636	0.008394
	0.001503	-0.017311	0.056808	0.001920	0.020459	0.044088
	-0.014432	-0.038872	0.032236	0.028981	0.003992	-0.001851
	-0.037727					
z427	0.039063	-0.041152	0.007154	-0.002947	-0.005677	-0.003185
	0.016686	0.030357	0.029906	-0.012577	0.029910	0.015993
	-0.037604	0.020497	-0.005970	-0.010335	-0.021632	0.011169
	0.004046	-0.015133	0.007913	0.008244	-0.008328	0.048498
	0.028305	0.007274	-0.014123	-0.026043	0.004274	-0.001711
	0.020941					
x428	-0.007319	-0.018241	-0.023917	0.016156	0.022785	0.009496
	-0.019883	0.014030	-0.028218	-0.019130	0.000104	-0.006460
	0.034844	0.016513	0.010772	-0.001415	-0.035248	-0.021618
	0.025349	0.016983	-0.022891	0.004205	0.017306	-0.015713
	-0.001729	-0.007939	-0.020658	-0.002007	-0.030022	-0.015326
	0.011631					
y428	0.025535	0.022979	-0.008601	0.010916	-0.018208	0.031386
	0.031523	-0.040204	0.011099	0.015306	0.003338	-0.020216
	-0.020413	-0.022897	0.025309	-0.016068	-0.027934	0.010090
	0.004627	-0.015002	0.052226	-0.000457	0.023735	0.045028
	-0.012665	-0.037233	0.030130	0.024523	0.000846	0.007514
	-0.036902					
z428	0.036550	-0.037639	0.007664	-0.004892	-0.010489	-0.000725
	0.018072	0.029438	0.028055	-0.018276	0.030714	0.017473
	-0.033776	0.022758	-0.004361	-0.009975	-0.025728	0.014703
	0.009128	-0.016092	0.008885	0.006405	-0.009227	0.051601
	0.026715	0.004295	-0.012366	-0.024931	0.003131	0.000193
	0.018170					

x429	-0.010886	-0.020986	-0.027279	0.012150	0.024478	0.009237
	-0.023762	0.017291	-0.030147	-0.019449	-0.002034	-0.006023
	0.037486	0.014551	0.005316	0.000918	-0.029468	-0.021961
	0.020717	0.022032	-0.025070	0.006448	0.016965	-0.016924
	0.000511	-0.006656	-0.028756	-0.000676	-0.026591	-0.022391
	0.020492					
y429	0.021134	0.018336	-0.010137	0.009195	-0.016993	0.032914
	0.028546	-0.038624	0.007387	0.011538	0.007229	-0.021332
	-0.023426	-0.022748	0.024937	-0.015953	-0.015651	0.008275
	0.005750	-0.013326	0.046260	-0.003823	0.026432	0.044739
	-0.009598	-0.032188	0.023649	0.017955	-0.002941	0.009984
	-0.030665					
z429	0.034574	-0.034923	0.008831	-0.003599	-0.013811	0.002862
	0.015999	0.029118	0.027675	-0.020609	0.028625	0.020583
	-0.029685	0.025420	-0.002096	-0.004315	-0.027400	0.015431
	0.013435	-0.015312	0.008702	0.006843	-0.004776	0.049706
	0.029672	0.000740	-0.012337	-0.028970	0.002967	0.000619
	0.012194					
x430	-0.013647	-0.023367	-0.029974	0.008597	0.026540	0.009068
	-0.027674	0.019604	-0.030447	-0.018788	-0.005129	-0.004957
	0.040497	0.012311	0.002075	0.002433	-0.025518	-0.020414
	0.018272	0.027427	-0.023968	0.010679	0.017392	-0.016401
	0.003959	-0.007461	-0.032528	0.002381	-0.025470	-0.025273
	0.029284					
y430	0.016006	0.013991	-0.012649	0.006711	-0.015976	0.033740
	0.025273	-0.035670	0.002279	0.007378	0.011508	-0.022822
	-0.026091	-0.022906	0.022883	-0.015314	-0.003120	0.003823
	0.005432	-0.012205	0.038805	-0.007780	0.029968	0.041706
	-0.008015	-0.025457	0.012761	0.010480	-0.002804	0.006273
	-0.022841					
z430	0.031856	-0.032343	0.009758	-0.003392	-0.019107	0.002837
	0.016946	0.026541	0.025495	-0.020504	0.025381	0.019781
	-0.026913	0.026700	-0.000844	-0.000080	-0.030839	0.009999
	0.013064	-0.012734	0.011482	0.002147	0.002069	0.045756
	0.034082	-0.004759	-0.012955	-0.030751	-0.009824	0.008452
	0.012633					
x431	-0.015108	-0.025570	-0.031838	0.005520	0.029760	0.009340
	-0.031756	0.021641	-0.028391	-0.017318	-0.008469	-0.003509
	0.042658	0.008847	0.000019	0.003008	-0.022773	-0.015938
	0.019357	0.033900	-0.022963	0.015537	0.015671	-0.013075
	0.007313	-0.006961	-0.030489	0.007461	-0.023929	-0.023593
	0.032835					

y431	0.010030	0.009741	-0.015826	0.004273	-0.015095	0.033826
	0.022022	-0.032245	-0.004029	0.002759	0.016054	-0.024799
	-0.027463	-0.022185	0.019802	-0.013744	0.008042	-0.002916
	0.004353	-0.011400	0.032266	-0.011132	0.035613	0.035346
	-0.005374	-0.017306	-0.001203	0.003578	-0.002826	-0.003237
	-0.009823					
z431	0.030492	-0.029990	0.010334	-0.002026	-0.024811	0.003912
	0.016926	0.025727	0.025375	-0.021329	0.020338	0.019211
	-0.028481	0.025445	-0.000666	0.004940	-0.025818	0.011829
	0.017182	-0.012516	0.010904	0.002550	0.007017	0.042190
	0.041518	0.001246	-0.014043	-0.034194	-0.016327	0.010367
	0.006093					
x432	-0.015393	-0.027973	-0.032417	0.002542	0.034599	0.010640
	-0.037004	0.023149	-0.023102	-0.015562	-0.011552	-0.000430
	0.043597	0.004394	-0.000548	0.002733	-0.020694	-0.008885
	0.021763	0.040473	-0.021110	0.021219	0.010538	-0.004713
	0.011868	-0.006713	-0.025116	0.010600	-0.022420	-0.016527
	0.034972					
y432	0.003311	0.006055	-0.019156	0.001774	-0.013502	0.032637
	0.019290	-0.027829	-0.011456	-0.001560	0.020665	-0.027577
	-0.027845	-0.021348	0.015182	-0.011823	0.016053	-0.010551
	0.004395	-0.010208	0.026411	-0.013208	0.044242	0.024989
	-0.002982	-0.006877	-0.015369	-0.000100	-0.002169	-0.018118
	0.004939					
z432	0.028840	-0.026589	0.010006	-0.001078	-0.029012	0.004805
	0.018975	0.024709	0.024305	-0.023908	0.017679	0.019844
	-0.028836	0.026375	-0.004296	0.012309	-0.021119	0.007746
	0.017547	-0.013871	0.007957	-0.002018	0.001035	0.040174
	0.035198	0.000853	-0.022232	-0.033223	-0.009868	0.014166
	-0.001998					
x433	-0.013842	-0.030734	-0.031842	-0.000392	0.039922	0.012837
	-0.044486	0.024968	-0.014057	-0.014252	-0.014395	0.002651
	0.042232	-0.001394	-0.001589	0.000895	-0.015711	0.000837
	0.025762	0.046497	-0.020873	0.023555	0.000692	0.006570
	0.016696	-0.008096	-0.015571	0.010937	-0.020848	-0.004027
	0.038998					
y433	-0.003560	0.002649	-0.021794	-0.000377	-0.009036	0.030301
	0.016422	-0.022543	-0.019049	-0.004074	0.025454	-0.030667
	-0.026645	-0.021078	0.008480	-0.009349	0.017819	-0.016672
	0.006259	-0.008030	0.022906	-0.013684	0.053352	0.011159
	0.000928	0.006677	-0.028418	-0.000916	-0.001243	-0.039629
	0.018759					

z433	0.027403	-0.023153	0.011433	0.002143	-0.033918	0.007139
	0.015606	0.023381	0.023997	-0.026920	0.013486	0.019866
	-0.027293	0.019601	-0.006919	0.019219	-0.014120	0.007556
	0.017942	-0.020601	0.007251	-0.004787	0.006531	0.026424
	0.035944	0.010682	-0.022212	-0.040373	-0.014650	0.018177
	0.006869					
x434	-0.009285	-0.029184	-0.030363	-0.001210	0.041983	0.015761
	-0.044754	0.023790	-0.009566	-0.011994	-0.011130	0.006874
	0.038584	-0.004313	0.001462	0.003703	-0.020386	0.011143
	0.023906	0.047088	-0.013747	0.018762	-0.013639	0.010541
	0.012156	-0.024023	-0.006837	0.007764	-0.012318	0.004086
	0.039408					
y434	-0.006289	-0.000906	-0.024886	-0.003821	-0.002457	0.032531
	0.010957	-0.014946	-0.019326	0.000563	0.022364	-0.034673
	-0.021259	-0.013311	0.002686	-0.009833	0.013305	-0.018603
	-0.000369	-0.008310	0.017042	-0.008860	0.038270	0.012420
	-0.001365	0.004634	-0.021579	0.010810	-0.004402	-0.032613
	0.018000					
z434	0.026406	-0.021933	0.011023	0.004823	-0.035975	0.005443
	0.015136	0.024157	0.023380	-0.030087	0.008173	0.017511
	-0.019365	0.017725	-0.008610	0.022325	-0.007764	0.004406
	0.018870	-0.017809	0.009103	-0.003227	0.003039	0.018759
	0.029309	0.015742	-0.022020	-0.037312	-0.016886	0.020299
	0.005148					
x435	-0.005172	-0.027346	-0.029399	-0.000864	0.042961	0.016540
	-0.045464	0.021588	-0.005274	-0.010598	-0.008998	0.009694
	0.034867	-0.008609	0.003552	0.005480	-0.025607	0.019720
	0.023094	0.046335	-0.009574	0.014278	-0.022581	0.013759
	0.010222	-0.036779	0.000014	0.008433	-0.005698	0.007975
	0.038013					
y435	-0.008491	-0.004206	-0.027120	-0.006834	0.005355	0.034433
	0.006223	-0.007287	-0.019301	0.006337	0.019440	-0.038068
	-0.016062	-0.007670	-0.004655	-0.010176	0.006574	-0.018419
	-0.005751	-0.008639	0.013322	-0.005920	0.022510	0.013255
	-0.002026	0.003873	-0.014119	0.021651	-0.007130	-0.028334
	0.014615					
z435	0.024589	-0.020650	0.009957	0.008779	-0.036739	0.005669
	0.013570	0.026257	0.024302	-0.031259	0.000962	0.015683
	-0.015236	0.010391	-0.008191	0.025831	-0.002847	0.004088
	0.018657	-0.020504	0.006904	0.002675	0.003857	0.015671
	0.028076	0.021579	-0.023478	-0.032151	-0.018859	0.021885
	0.010094					

x436	-0.001615	-0.025087	-0.028871	0.000033	0.041973	0.015358
	-0.045437	0.017704	-0.002263	-0.010805	-0.006723	0.010070
	0.030675	-0.011891	0.004872	0.005701	-0.028553	0.024678
	0.022182	0.043106	-0.008618	0.011118	-0.027800	0.014585
	0.010871	-0.045405	0.005115	0.011427	-0.004087	0.008665
	0.033502					
y436	-0.010560	-0.007047	-0.028716	-0.009485	0.013274	0.035919
	0.002057	0.000044	-0.018981	0.012653	0.016377	-0.041091
	-0.011219	-0.004071	-0.012121	-0.010455	-0.001222	-0.016862
	-0.009626	-0.010231	0.010051	-0.003559	0.009439	0.014259
	-0.000573	0.003559	-0.005944	0.031650	-0.011400	-0.025365
	0.010049					
z436	0.023649	-0.020115	0.007274	0.012545	-0.037365	0.005154
	0.013368	0.026982	0.023609	-0.032586	-0.004527	0.013915
	-0.010965	0.006899	-0.007359	0.027707	0.004170	0.002487
	0.016094	-0.022285	0.005723	0.004964	0.003214	0.009019
	0.026384	0.022169	-0.026720	-0.025410	-0.020516	0.018935
	0.012465					
x437	0.001542	-0.022687	-0.028451	0.001475	0.039547	0.012779
	-0.045011	0.013166	-0.000345	-0.012891	-0.004197	0.009073
	0.025739	-0.014317	0.006150	0.006169	-0.030181	0.026900
	0.022254	0.040783	-0.007947	0.009710	-0.030000	0.012989
	0.012357	-0.050290	0.008702	0.012602	-0.001702	0.005605
	0.029325					
y437	-0.012504	-0.009519	-0.030291	-0.011786	0.020633	0.036967
	-0.001666	0.006479	-0.018329	0.018414	0.013549	-0.044519
	-0.006193	-0.001442	-0.019150	-0.010536	-0.008562	-0.015152
	-0.012559	-0.012401	0.007078	-0.001575	-0.001733	0.015210
	0.001494	0.004577	0.000888	0.041109	-0.015319	-0.025536
	0.004781					
z437	0.022316	-0.019624	0.004754	0.015742	-0.037304	0.004871
	0.012531	0.029709	0.022160	-0.034202	-0.009839	0.012205
	-0.007711	0.003361	-0.007627	0.029757	0.008489	0.000842
	0.015337	-0.021209	0.007439	0.008773	0.003621	0.003260
	0.024943	0.023258	-0.022891	-0.027118	-0.016492	0.016905
	0.016445					
x438	0.004437	-0.019979	-0.027935	0.002892	0.035577	0.009188
	-0.042939	0.008095	0.001072	-0.015903	-0.001733	0.007534
	0.019339	-0.016232	0.008836	0.006396	-0.029076	0.028388
	0.023367	0.040237	-0.004410	0.008748	-0.029480	0.009512
	0.014465	-0.050473	0.007048	0.011294	0.000044	0.001182
	0.024747					

y438	-0.014642	-0.011761	-0.031938	-0.013889	0.026908	0.037701
	-0.004617	0.012027	-0.017555	0.023295	0.011216	-0.049016
	-0.001826	0.000796	-0.024908	-0.010028	-0.014304	-0.013573
	-0.014780	-0.014590	0.005637	-0.000137	-0.010865	0.016106
	0.004683	0.007138	0.005161	0.048442	-0.018346	-0.026788
	0.000007					
z438	0.021528	-0.019401	0.002811	0.017264	-0.036653	0.003933
	0.014011	0.033846	0.021596	-0.033221	-0.015898	0.013540
	-0.003630	-0.000865	-0.006968	0.029440	0.011353	0.002576
	0.016232	-0.016675	0.014110	0.012606	0.006380	-0.005228
	0.021605	0.025001	-0.022637	-0.030040	-0.013501	0.011892
	0.014028					
x439	0.007621	-0.016933	-0.027871	0.005640	0.031375	0.005830
	-0.040391	0.002517	0.001833	-0.019595	0.003767	0.005463
	0.010623	-0.018439	0.013627	0.009378	-0.027803	0.030641
	0.026157	0.039531	-0.006462	0.007476	-0.031473	0.008048
	0.016157	-0.048215	0.003036	0.008495	0.003327	-0.001173
	0.019076					
y439	-0.016779	-0.013761	-0.033734	-0.015214	0.032076	0.038751
	-0.006805	0.017227	-0.016694	0.027180	0.010563	-0.054547
	0.001808	0.002250	-0.028143	-0.007583	-0.018277	-0.011198
	-0.016135	-0.016720	0.004063	-0.000163	-0.019601	0.017521
	0.008215	0.010004	0.004559	0.052864	-0.019665	-0.027050
	-0.004777					
z439	0.021647	-0.019228	-0.001095	0.020412	-0.032843	0.004202
	0.015169	0.037700	0.021363	-0.030209	-0.017040	0.013624
	-0.001607	-0.006301	-0.007340	0.029188	0.006356	0.005789
	0.016861	-0.015973	0.009027	0.015939	0.003995	-0.003329
	0.020265	0.024946	-0.015242	-0.027002	-0.006689	0.015347
	0.012551					
x440	0.011108	-0.013547	-0.026631	0.008252	0.025490	0.002075
	-0.037074	-0.004171	0.002115	-0.024932	0.009473	0.004253
	-0.000291	-0.019054	0.021925	0.012190	-0.022514	0.032635
	0.029428	0.042174	-0.003599	0.005833	-0.031829	0.002161
	0.016666	-0.044355	-0.004194	0.000919	0.003732	-0.004104
	0.018540					
y440	-0.018697	-0.015509	-0.035531	-0.015925	0.036030	0.040418
	-0.008465	0.021700	-0.015646	0.029216	0.011606	-0.060883
	0.004062	0.002877	-0.027711	-0.003139	-0.019917	-0.007258
	-0.016934	-0.017311	0.004269	-0.002660	-0.028410	0.018771
	0.010771	0.012418	-0.000842	0.053042	-0.019139	-0.024286
	-0.005530					

z440	0.021190	-0.019767	-0.004210	0.023473	-0.029710	0.003919
	0.015322	0.041480	0.021293	-0.026040	-0.020664	0.014517
	-0.000585	-0.008257	-0.006482	0.027236	0.005887	0.006847
	0.016288	-0.014108	0.009924	0.017842	0.004402	-0.005929
	0.016831	0.025158	-0.016185	-0.028692	-0.009382	0.014927
	0.009672					
x441	0.015182	-0.009701	-0.024191	0.010931	0.018987	-0.001921
	-0.033296	-0.012162	0.002056	-0.031607	0.015538	0.004107
	-0.012408	-0.018093	0.031174	0.015208	-0.014543	0.035029
	0.033206	0.046034	0.000639	0.005614	-0.032609	-0.006033
	0.015498	-0.038829	-0.010371	-0.007723	0.002098	-0.007941
	0.020502					
y441	-0.020260	-0.016953	-0.036958	-0.016170	0.039018	0.042827
	-0.009791	0.025762	-0.014187	0.028692	0.014608	-0.067333
	0.005063	0.002460	-0.023292	0.003431	-0.018224	-0.000328
	-0.016774	-0.017061	0.006086	-0.007895	-0.038013	0.019465
	0.009010	0.014824	-0.011387	0.046936	-0.017531	-0.017282
	-0.001264					
z441	0.020468	-0.020766	-0.009103	0.027895	-0.025658	0.004354
	0.015820	0.042702	0.022580	-0.024347	-0.024705	0.013958
	0.000870	-0.011250	-0.006542	0.024587	0.003449	0.006852
	0.014029	-0.014916	0.013168	0.022355	0.003881	-0.008905
	0.015001	0.020689	-0.014769	-0.022398	-0.010653	0.013593
	0.013286					
x442	0.019604	-0.004883	-0.018532	0.012024	0.011132	-0.006731
	-0.028869	-0.021166	0.001800	-0.036840	0.019385	0.006457
	-0.025018	-0.014324	0.039490	0.016386	-0.004166	0.035629
	0.038763	0.049982	0.005893	0.009338	-0.028071	-0.015582
	0.014186	-0.033589	-0.013760	-0.013623	-0.000934	-0.011750
	0.018778					
y442	-0.020233	-0.017505	-0.037526	-0.015334	0.040967	0.045014
	-0.010649	0.027728	-0.012648	0.025676	0.020208	-0.073386
	0.002915	0.002531	-0.013240	0.014749	-0.012791	0.009362
	-0.013923	-0.013342	0.012338	-0.017382	-0.055430	0.016768
	0.001918	0.016402	-0.026088	0.028309	-0.017348	-0.006831
	0.008586					
z442	0.019312	-0.022214	-0.015614	0.033299	-0.022218	0.004535
	0.019672	0.045436	0.023939	-0.023081	-0.027615	0.014284
	0.003430	-0.015593	-0.004419	0.021177	0.000249	0.009074
	0.011375	-0.011652	0.016798	0.028985	0.006790	-0.013266
	0.013381	0.014522	-0.014728	-0.011045	-0.008069	0.008556
	0.021285					

x443	0.021061	-0.000350	-0.006931	0.004413	0.002354	-0.008786
	-0.026628	-0.027658	0.000197	-0.034962	0.017483	0.013820
	-0.032047	-0.012437	0.034964	0.008650	0.003738	0.024032
	0.039863	0.034870	0.004066	0.020334	-0.004741	-0.023033
	0.018526	-0.029318	-0.009670	0.008883	-0.005761	-0.004228
	0.005630					
y443	-0.016190	-0.016557	-0.036008	-0.012870	0.033043	0.034857
	-0.008561	0.016734	-0.016365	0.022131	0.028711	-0.070463
	0.007394	0.001390	-0.001018	0.018904	-0.004874	0.003299
	-0.002267	-0.001904	0.026923	-0.030744	-0.088126	0.007991
	-0.010146	-0.001774	-0.039969	0.013638	-0.025653	-0.000515
	-0.001148					
z443	0.019356	-0.023867	-0.026264	0.040615	-0.014617	0.008617
	0.025792	0.047927	0.024059	-0.022921	-0.026882	0.014041
	0.001721	-0.014853	0.001835	0.018815	0.006050	0.013389
	0.005442	-0.006748	0.018895	0.029277	-0.007078	-0.008320
	0.010213	0.013041	-0.018807	-0.014832	0.005087	-0.001501
	0.018836					
x444	0.023886	0.003963	0.003637	-0.001643	-0.006071	-0.012870
	-0.022667	-0.034248	-0.001491	-0.032243	0.017526	0.021686
	-0.039985	-0.007895	0.029463	0.002144	0.010364	0.010321
	0.036925	0.024718	0.006701	0.031409	0.019870	-0.033197
	0.026916	-0.021690	-0.004162	0.033881	-0.012228	0.001687
	-0.009961					
y444	-0.009159	-0.013129	-0.029247	-0.014919	0.026497	0.022310
	-0.008244	0.008305	-0.022969	0.015209	0.034935	-0.066248
	0.000543	0.007209	0.007785	0.026541	0.003138	0.001701
	-0.000240	0.008440	0.028355	-0.037543	-0.107310	0.004668
	-0.018805	-0.012224	-0.043856	-0.004798	-0.032859	0.003159
	0.004724					
z444	0.019918	-0.025377	-0.036672	0.046925	-0.007239	0.011856
	0.032961	0.049441	0.025090	-0.022863	-0.029939	0.013668
	-0.002065	-0.016733	0.008801	0.018824	0.011144	0.016435
	-0.005031	0.000318	0.021960	0.025560	-0.008716	-0.006836
	0.016979	0.013154	-0.010184	-0.022232	0.011495	-0.005869
	0.032079					
x445	0.026618	0.006511	0.009689	-0.002938	-0.013193	-0.015826
	-0.016388	-0.041261	-0.004074	-0.027189	0.021151	0.028409
	-0.046103	-0.004290	0.025488	-0.001551	0.013476	-0.005048
	0.033847	0.018069	0.011824	0.040418	0.035118	-0.043287
	0.030613	-0.010345	-0.001370	0.056394	-0.020688	0.004570
	-0.032837					

y445	-0.001345	-0.008173	-0.017920	-0.020539	0.020227	0.007971
	-0.011628	0.003501	-0.030134	0.005584	0.036972	-0.061399
	-0.008338	0.017845	0.013253	0.034699	0.007585	0.000258
	-0.001218	0.014521	0.020994	-0.037077	-0.108107	0.007287
	-0.023510	-0.019179	-0.038773	-0.012390	-0.038331	0.004523
	0.015325					
z445	0.020331	-0.026164	-0.045668	0.055289	-0.003984	0.014753
	0.039305	0.048697	0.022923	-0.025121	-0.030571	0.017092
	-0.002495	-0.023749	0.016833	0.024502	0.011438	0.016380
	-0.012038	0.000586	0.024672	0.027499	-0.008559	-0.004130
	0.022710	0.010874	-0.009004	-0.028663	0.009493	-0.006275
	0.042475					
x446	0.028197	0.006513	0.009342	0.000144	-0.018515	-0.015739
	-0.004947	-0.047834	-0.008393	-0.018856	0.029006	0.032580
	-0.051761	-0.002343	0.022085	-0.002959	0.015947	-0.019362
	0.029437	0.014490	0.015322	0.041727	0.040934	-0.050919
	0.027038	-0.002180	-0.004245	0.068059	-0.026213	0.006694
	-0.053444					
y446	0.006064	-0.002111	-0.003738	-0.028333	0.013599	-0.007046
	-0.016800	0.001890	-0.036048	-0.005055	0.034076	-0.053781
	-0.015071	0.028890	0.018301	0.041212	0.012203	-0.000230
	-0.001397	0.018581	0.012860	-0.030914	-0.099713	0.012959
	-0.023204	-0.022783	-0.025832	-0.006874	-0.040878	0.004663
	0.017821					
z446	0.019548	-0.027232	-0.051918	0.059804	-0.000823	0.016758
	0.048666	0.046382	0.016599	-0.026979	-0.031208	0.017855
	-0.002158	-0.025303	0.017901	0.028172	0.016452	0.012909
	-0.013156	0.002272	0.029200	0.021569	-0.008669	-0.000113
	0.023396	0.010745	-0.010643	-0.028671	0.011650	-0.002626
	0.038777					
x447	0.029063	0.004604	0.004023	0.006605	-0.021360	-0.012315
	0.008093	-0.053313	-0.013660	-0.007010	0.040206	0.035043
	-0.058316	-0.002718	0.017974	-0.000796	0.015512	-0.030407
	0.021773	0.012310	0.007507	0.034033	0.039999	-0.050923
	0.018673	0.001957	-0.009272	0.063239	-0.031416	0.006674
	-0.061527					
y447	0.012196	0.003610	0.010080	-0.036175	0.007450	-0.020062
	-0.021429	0.001996	-0.039644	-0.014582	0.028024	-0.044851
	-0.019576	0.037824	0.023111	0.046381	0.017761	-0.000184
	0.000463	0.020411	0.005559	-0.022884	-0.088332	0.017645
	-0.021316	-0.024870	-0.012328	0.001570	-0.042127	0.003908
	0.016666					

z447	0.018912	-0.027570	-0.056062	0.062763	0.000373	0.017301
	0.056236	0.043705	0.010922	-0.028266	-0.030927	0.016757
	-0.003701	-0.025885	0.017160	0.030991	0.021529	0.010551
	-0.015791	0.007446	0.032121	0.015811	-0.009749	0.001978
	0.020666	0.007344	-0.011456	-0.025498	0.013992	-0.004652
	0.037179					
x448	0.029642	0.002278	-0.003246	0.014819	-0.022565	-0.008004
	0.020184	-0.057829	-0.018600	0.006567	0.054491	0.036700
	-0.065906	-0.004810	0.012422	0.002712	0.013267	-0.037691
	0.012185	0.010571	-0.009603	0.021960	0.034084	-0.044063
	0.009550	0.006242	-0.011799	0.052600	-0.036826	0.002388
	-0.064235					
y448	0.017941	0.008931	0.023633	-0.044000	0.001661	-0.031676
	-0.025897	0.002111	-0.042362	-0.024139	0.021617	-0.035942
	-0.023699	0.044860	0.027893	0.052724	0.021055	0.001181
	0.003942	0.020846	-0.004093	-0.017633	-0.075089	0.022592
	-0.020678	-0.026093	-0.001187	0.010675	-0.041490	0.003345
	0.013131					
z448	0.019055	-0.026176	-0.059095	0.064697	0.001523	0.015742
	0.062407	0.042116	0.006052	-0.028259	-0.029589	0.016087
	-0.006993	-0.028164	0.018245	0.031940	0.027831	0.005576
	-0.020017	0.009891	0.032307	0.015507	-0.011513	-0.000082
	0.014254	0.001101	-0.005769	-0.018254	0.014990	-0.007351
	0.034182					
x449	0.029772	-0.000311	-0.012850	0.024934	-0.023433	-0.001601
	0.033967	-0.062986	-0.024324	0.023443	0.073086	0.037300
	-0.076900	-0.008687	0.005499	0.007254	0.011845	-0.045005
	-0.001142	0.010632	-0.030990	0.006473	0.021933	-0.031399
	-0.002678	0.010772	-0.014406	0.041064	-0.042403	-0.004330
	-0.063015					
y449	0.023981	0.016070	0.041434	-0.053492	-0.004963	-0.044909
	-0.030272	0.001670	-0.044874	-0.034894	0.013949	-0.024946
	-0.030579	0.050057	0.032598	0.060943	0.026562	0.001666
	0.007769	0.017365	-0.017653	-0.007494	-0.057489	0.026042
	-0.022220	-0.026061	0.012806	0.022415	-0.037527	0.001123
	0.008307					
z449	0.020715	-0.024748	-0.062354	0.065432	0.002495	0.011158
	0.070498	0.040028	-0.003464	-0.026158	-0.029604	0.015402
	-0.012516	-0.032058	0.023816	0.029991	0.034394	0.002456
	-0.023637	0.016709	0.037943	0.012470	-0.011902	-0.004794
	0.008684	-0.004700	0.000149	-0.011163	0.010659	-0.009680
	0.029693					

x450	0.028665	-0.002771	-0.024910	0.039807	-0.022758	0.007641
	0.045780	-0.068549	-0.031614	0.047597	0.100361	0.034710
	-0.090087	-0.016426	-0.001273	0.016049	0.008335	-0.048994
	-0.015571	0.011145	-0.062873	-0.012349	0.001137	-0.004397
	-0.008985	0.019475	-0.012553	0.025871	-0.050340	-0.009265
	-0.058130					
y450	0.029881	0.025409	0.064589	-0.064876	-0.012327	-0.060411
	-0.036475	-0.000998	-0.050536	-0.045404	0.004927	-0.015879
	-0.042912	0.048619	0.034807	0.072506	0.026718	0.000112
	0.011856	0.009951	-0.027187	0.011089	-0.023857	0.014367
	-0.020412	-0.016141	0.024934	0.036204	-0.034344	0.004154
	0.002573					
z450	0.024712	-0.022109	-0.066627	0.066491	0.004999	0.008179
	0.079167	0.036214	-0.014219	-0.026330	-0.025256	0.018735
	-0.026580	-0.037347	0.031484	0.034929	0.042861	0.003100
	-0.032284	0.021078	0.016300	0.003097	-0.032831	0.003776
	-0.009147	-0.013347	0.018609	0.006074	0.014187	-0.017314
	-0.002760					
x451	0.025799	-0.005718	-0.035305	0.054787	-0.024497	0.013215
	0.049713	-0.082417	-0.037867	0.069445	0.125235	0.019516
	-0.093790	-0.020690	-0.004502	0.018617	-0.010514	-0.067809
	-0.028990	0.017769	-0.078643	-0.026191	-0.030651	0.016843
	-0.008546	0.020605	-0.002436	-0.001818	-0.063867	-0.014536
	-0.040515					
y451	0.032412	0.033890	0.084789	-0.071219	-0.019488	-0.071504
	-0.042082	-0.004864	-0.054802	-0.049945	-0.000480	-0.011293
	-0.057972	0.040486	0.036908	0.083921	0.014456	-0.002557
	0.005790	0.006140	-0.021652	0.026575	0.014587	-0.020995
	-0.018750	-0.001988	0.026228	0.035515	-0.031343	0.003485
	-0.010078					
z451	0.024939	-0.014900	-0.060548	0.059043	0.002189	-0.000560
	0.077181	0.036193	-0.027971	-0.037790	-0.034221	0.018883
	-0.029224	-0.028685	0.043724	0.034886	0.054592	-0.003505
	-0.031523	0.036766	0.013731	0.012659	-0.031907	-0.007344
	-0.022592	-0.015841	0.035123	0.042281	0.014436	-0.023635
	-0.018389					

Phylogenetic mapping

Dataset: PC scores, CovMatrix, BE_Cochlea, Procrustes coordinates

Data type: PC scores

Phylogeny: Phylogeny_BE_Cochlea

Method: weighted squared-change parsimony

The tree is rooted.

Tree length: 0.14002329

Permutation test against the null hypothesis of no phylogenetic signal

Number of randomization rounds: 10000

P-value: 0.0001

Canonical Variate Analysis: CVA ...BE_Cochlea

Dataset: BE_Cochlea

Classification criterion: environment

Groups		Observations
1.	coastal	15
2.	continental	8
3.	marine	7
4.	polar	2

Variation among groups, scaled by the inverse of the within-group variation

	Eigenvalues	% Variance	Cumulative %
1.	2.07340885	44.618	44.618
2.	1.85336713	39.883	84.500

3.	0.72028933	15.500	100.000

Mahalanobis distances among groups:

	coastal	continental	marine
continental	3.2256		
marine	3.3397	3.9255	
polar	3.5246	3.8803	4.3720

P-values from permutation tests (10000 permutation rounds) for Mahalanobis distances among groups:

	coastal	continental	marine
continental	<.0001		
marine	0.0001	0.0012	
polar	0.9696	0.8620	0.2792

Procrustes distances among groups:

	coastal	continental	marine
continental	0.0653		
marine	0.0811	0.1039	
polar	0.0870	0.0882	0.1086

P-values from permutation tests (10000 permutation rounds) for Procrustes distances among groups:

	coastal	continental	marine
[truncated: 410,821 more chars]
